# Deception Undermines the Stability of Cooperation in Games of Indirect Reciprocity

**DOI:** 10.1371/journal.pone.0147623

**Published:** 2016-01-29

**Authors:** Szabolcs Számadó, Ferenc Szalai, István Scheuring

**Affiliations:** 1 MTA-ELTE Theoretical Biology and Evolutionary Ecology Research Group, Pázmány Péter sétány, 1/c, H-1117, Budapest, Hungary; 2 MTA, Centre for Social Sciences, Országház u. 30. 1014, Budapest, Hungary; 3 Engame Academy, Bajcsy-Zsilinszky u. 50, 1054, Budapest, Hungary; Peking University, CHINA

## Abstract

Indirect reciprocity is often claimed as one of the key mechanisms of human cooperation. It works only if there is a reputational score keeping and each individual can inform with high probability which other individuals were good or bad in the previous round. Gossip is often proposed as a mechanism that can maintain such coherence of reputations in the face of errors of transmission. Random errors, however, are not the only source of uncertainty in such situations. The possibility of deceptive communication, where the signallers aim to misinform the receiver cannot be excluded. While there is plenty of evidence for deceptive communication in humans the possibility of deception is not yet incorporated into models of indirect reciprocity. Here we show that when deceptive strategies are allowed in the population it will cause the collapse of the coherence of reputations and thus in turn it results the collapse of cooperation. This collapse is independent of the norms and the cost and benefit values. It is due to the fact that there is no selection for honest communication in the framework of indirect reciprocity. It follows that indirect reciprocity can be only proposed plausibly as a mechanism of human cooperation if additional mechanisms are specified in the model that maintains honesty.

## Introduction

Indirect reciprocity (IR), i.e. repeated encounters between donors and beneficiaries where the donor receives a payback not from the beneficiary itself but later from another individual in the population, is identified to be one of the key mechanisms of human cooperation [1,2,3]. It is well known that for indirect reciprocity to work the reputation of the others have to be known with a certain accuracy [1,2]. Accordingly, there has to be a mechanism in the population that maintains the coherence of reputational scores. Gossip is claimed to be one such a mechanism [2, 3]. In a paper Ohtsuki et al. [2] have found that by allowing a sufficiently large number of communicational rounds gossip remains robust against erroneous signalling. Misinformation, however, can have three sources. The first one is incomplete observation, when the uncertainty of reputation is due to the uncertainty of observation [4]. The second one is the error of implementation, studied by Ohtsuki et al. [2] when the signaller sends a wrong signal by mistake. Finally, the last one is strategic misinformation: it is deception, when the signaller misleads the receiver to gain an advantage (this may or may not be intentional). Such strategic deception is well documented both in animal and in human communication systems [5,6,7,8,9,10,11,12] and well studied by game theoretical models [13,14,15,16]. However, the effect of such strategic deception on the stability of cooperation in games of indirect reciprocity has not yet been investigated, even though the problem has been recently acknowledged: “… when people provide information, why be truthful?” (pp. 418 [3]). Here we investigate this effect using one of the key models of indirect reciprocity [2]. We use the same setup as the original authors introducing the possibility of strategic deception into the system.

Indirect reciprocity can only work if it is possible to discriminate between individuals with different reputation [1,2]. In the simplest case (which is often used in models) there are two types of actions: either to be cooperative which has a cost *c* for the donor and a benefit *b* for the beneficiary (where *b* > *c*) or to be defective where there is no donation (no benefit and no cost). Further there are only two states of reputation: bad and good [2, 9]. It was shown by earlier models of IR [17] that indirect reciprocity work if the ability to distinguish between good and bad (*q*) is larger than a given threshold; namely *q*>*c*/*b* [10]. If this condition does not hold then individuals are unable to differentiate players of bad reputation from players with good reputation at a sufficient level to reward cooperative players effectively. In other words, indirect reciprocity assumes an indirect reward system for cooperators, and this reward system functions only if cooperators can be distinguished from non-cooperators with a reasonable accuracy. This ability to distinguish between good and bad individuals depends on the probability of assigning incorrect reputation (*μ*), where: *q* = 1–2 *μ* [2]. In turn, this probability can have two sources: (i) unintended mistakes of observation or implementation, (ii) and strategic misinformation: i.e. deceit. Ohtsuki et al. [2] have studied the effect of the former and have found that sufficiently large rounds of communication can keep the system stable (see Appendix 1. for an analytic estimation of what counts as sufficient). Here we study the effect of the second source of incorrect reputation, and thus in contrast to all previous studies we allow for the presence of dishonest signalling strategies. Note that the presence of cheating communication strategies differs from simple errors of implementation in a crucial way: while errors are random and infrequent by definition, dishonest signallers introduce errors into the system at every turn in a systematic way. This means that the effect of dishonest signallers over time will depend on their proportion in the population. Lies originated by dishonest signallers can be perpetuated by honest ones later on.

## Methods

To investigate the problem we use the model of indirect reciprocity described by Ohtsuki et al. [2]. The model considers a large population from which pairs of individuals are sampled to play a game. One player is the donor the other is the recipient. Following Ohtsuki et al. the donor can choose either to cooperate (C), to defect (D) or to punish (P). Cooperation has a cost *c* to the donor and increases the fitness of the recipient with a benefit *b*. Defection has no cost and gives no benefit. Punishment has a cost *α* to the punisher (donor) and it incurs a cost *β* to the receiver (where *α* < *β*) [2]. Individuals can have good (G) or bad (B) reputation, and it depends on their actions. The behaviour of the donor is regulated by a behavioural strategy, which specifies what action should be taken knowing the reputation of the receiver. For example, the behavioural strategy denoted by (C,D) means that the donor cooperates when the receiver has a good reputation and defects when it has a bad reputation. Reputational scores are updated using a social norm assumed to be followed by every individual in the population [2]. Ohtsuki et al. [2] investigated second order norms, that is, assessment depends on the action of the donor and the reputation of the recipient. Individuals have a personal accounting of reputations thus different individuals can assign different reputation to the same donor.

Ohtsuki et al. [2] show that errors in assigning reputation will undermine cooperation. To restore the coherence of reputational scores they introduce communicational rounds between randomly chosen pairs. One player in the pair is the receiver who asks about the reputation of a third randomly chosen individual (the subject) from the signaller and adopts the opinion of the signaller on the subject. In the original model it was assumed that such communication will be honest unless the signaller errs by mistake. Here we relax this assumption and introduce the possibility of cheating. A signalling strategy specifies the behaviour of the signaller. It may or may not depend on the reputation of the signaller and the subject -just like social norms. The simplest specification allows only two states: the individual is either honestly reporting all reputational scores or not. First order signalling strategies may take into account the perceived reputation (by the signaller) of the subject. Second order signalling strategies specify the behaviour of the signaller based on both the reputation of the signaller and that of the subject. Here we use first order signalling strategies. In this case the behaviour of the signaller depends on the perceived reputation of the subject. Accordingly, there can be four first order signalling strategies: S(G,G), S(G,B), S(B,B), S(B,G), where the signal in the first position specifies the communicated reputation if the subject’s reputation is good and the second specifies it when it is bad. In other words the first strategy reports a good reputation honestly but deceives the receiver when the subject’s reputation is bad. The second strategy is the honest one. The third strategy deceives the receiver when the subject’s reputation is good but it is honest in the other case, while the last strategy deceives the receiver in both cases.

We repeated the simulations with the same initial conditions as specified in Ohtsuki et al. [2] allowing signalling strategies to evolve. The initial population consisted of 80% of honest individuals (S(G,B)) while the rest was assigned randomly. Here we consider a private list of reputations in the same way as in the original model with communication ([2] SI). The initial frequencies of behavioural strategies were the same as described in the Ohtsuki et al. [2], namely 80% of the population started out with a cooperative strategy CD or CP (cooperate, defect and cooperate, punish respectively in case of good and bad reputation) depending on the norm used in the simulation and the rest was assigned randomly. We studied four second order norms identified as the norms most favourable to the evolution of cooperative behavioural rules [2]: ‘stern-judging’, ‘simple-standing’, ‘shunning’ and ‘scoring’ (see Table 1). These norms share the same feature that cooperating with recipients in good reputation results a good reputation, and that defecting or punishing recipients in good reputation results a bad reputation for the donor respectively. Regarding recipients in bad reputation these norms cover all the possible reactions (i.e. B,G; G,G; B,B; G,B; see Table 1) To investigate the effect of the parameters of the model on the outcome and compare them with previous results we mapped the range of parameters studied in Ohtsuki et al. [2], carrying out 100 independent runs with each parameter combination (*n* = 100, *c* = 1, *α* = 1, *β* = 4, the value of *b* changed from 1 to 5 with steps of 0.5). Note that neither of our simulations reproduce the original figures of the Ohtsuki et al. [2] article: because the ability to distinguish between good and bad (*q*) was a parameter controlled by the authors, whereas here *q* depends on the proportion of the cheaters which is allowed to change in our model and which depends on the success of various signalling strategies. To investigate the effect of the composition of the initial strategies we also repeated these runs starting with 90% cooperators and honest behavioural strategies respectively. Further, to investigate the effect of punishment we repeated the simulations with *β* = 6, for those norms that utilise punishment (i.e. scoring and shunning). All in all we had three main scenarios: (i) the original one, where the frequency of the cooperative behavioral strategy and the honest signalling strategy is 80% (MST80); (ii) the next one where these frequencies were set to 90% (MST90), and finally where these frequencies were 80% again but we set *β* = 6 (BETA6). Note, that both of these changes should favour cooperators: in the first case their initial frequency is much higher; in the second case punishment is more efficient. Collapse of cooperation was defined when the cooperative behavioural rule (CD or CP) that was the major strategy at the start in the population (i.e. at 80% or 90%) completely disappeared from the population and the average level of cooperativeness in the population fall below the threshold 0,05. When both conditions were satisfied the run was terminated and the frequencies of behavioural and signalling strategies were recorded alongside with the time of collapse.

**Table 1:** Social Norms. Definition of the second order social norms investigated in the model (after Ohtsuki et al. [2]). Second order social norms specify the donor’s reputation as a function of the donor’s action and the recipient’s reputation. Where C, D and P denote cooperate, defect and punish respectively; while G and B denote good and bad.

Social norm	Donor’s action	Recipient’s reputation	
		(G)	(B)
Scoring	C	G	G
	P	B	B
Simple-standing	C	G	G
	D	B	G
Stern-judging	C	G	B
	D	B	G
Shunning	C	G	B
	P	B	B

## Results

**Fig 1:**
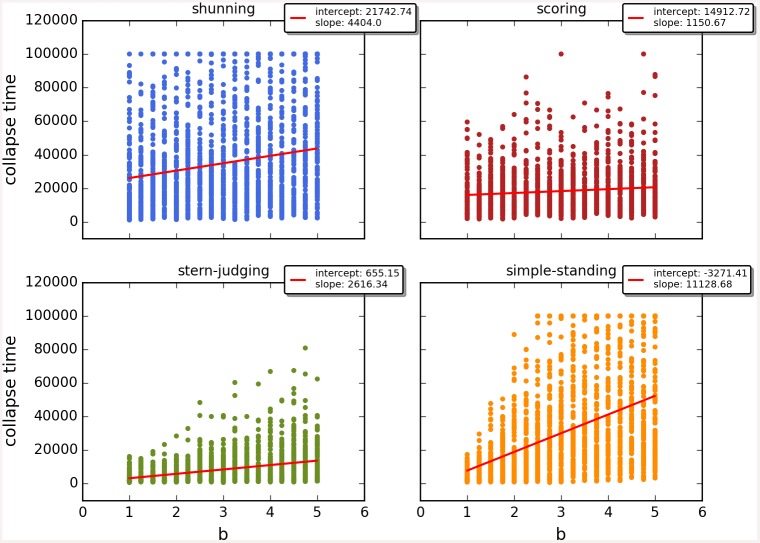
The time elapsed till the collapse of cooperation, MST80. The figure shows the average time until the collapse of cooperation as a function of the value of the benefit (*b*) for different social norms in the first scenario (MST80). Each symbol represents the collapse time of a single run; parameters are as follows: *n* = 100, *c* = 1, *α* = 1, *β* = 4, MST = 80%. Colour code: (a) shunning: blue; (b) scoring: red; (c) stern-judging: green; (d) simple-standing: orange. Regression lines were fitted to visualize the relationship between collapse times and *b*.

**Fig 2:**
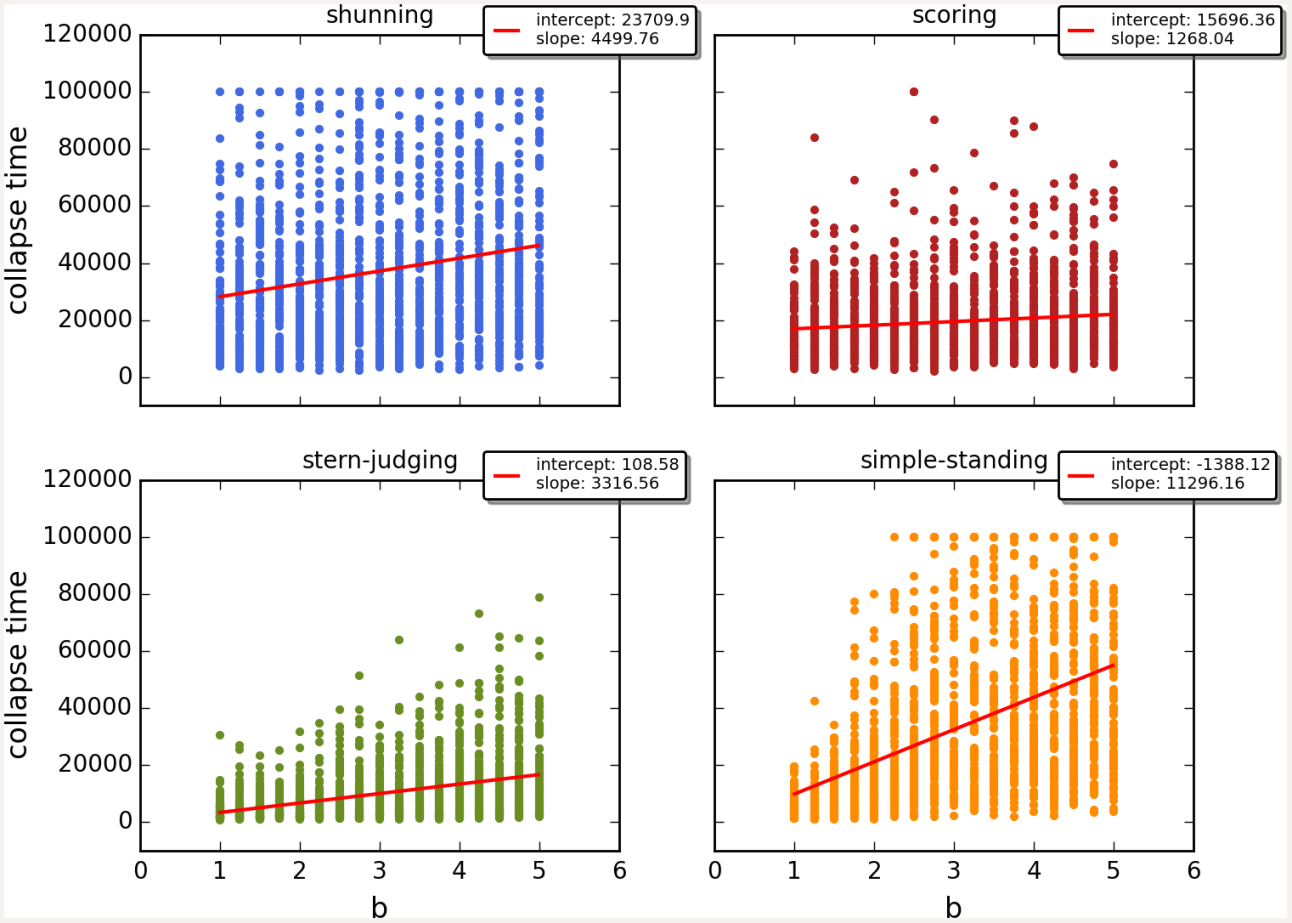
The time elapsed till the collapse of cooperation, MST90. The figure shows the average time until the collapse of cooperation as a function of the value of the benefit (*b*) for different social norms in the second scenario (MST90). Each symbol represents the collapse time of a single run; parameters are as follows: *n* = 100, *c* = 1, *α* = 1, *β* = 4, MST = 90%. Regression lines were fitted to visualize the relationship between collapse times and *b*.

**Fig 3:**
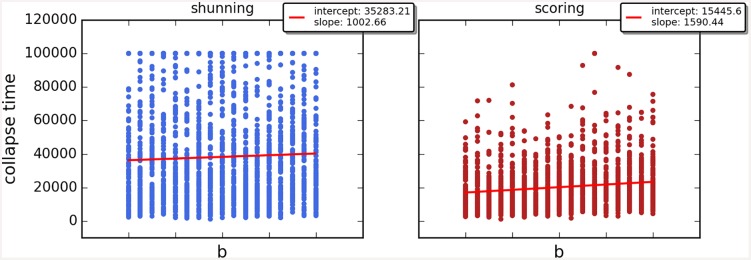
The time elapsed till the collapse of cooperation, BETA6. The figure shows the average time until the collapse of cooperation as a function of the value of the benefit (*b*) for different social norms in the third scenario (BETA6). Each symbol represents the collapse time of a single run; parameters are as follows: *n* = 100, *c* = 1, *α* = 1, *β* = 6, MST = 80%. Regression lines were fitted to visualize the relationship between collapse times and *b*.

**Fig 4:**
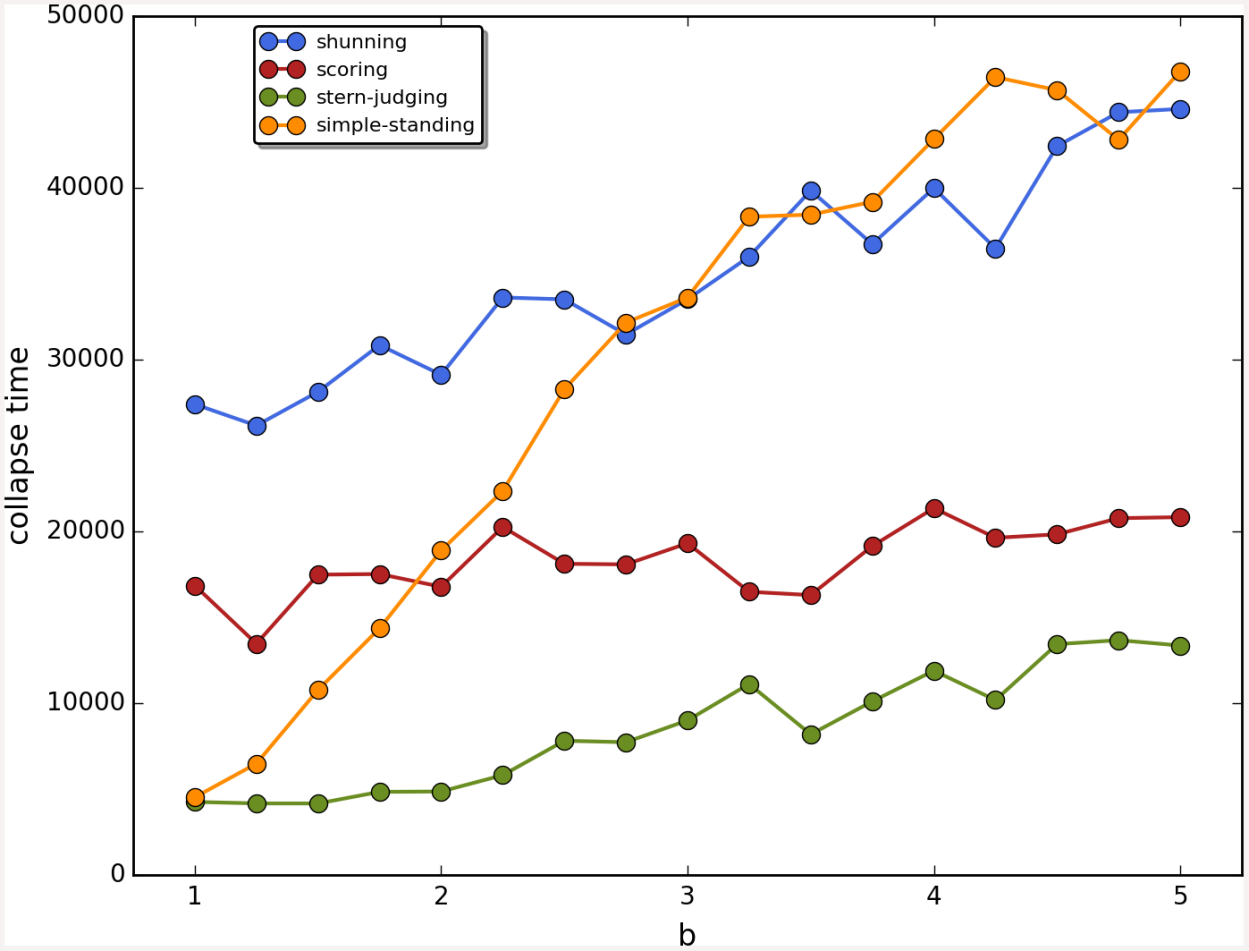
The average time elapsed till the collapse of cooperation, MST80. The figure shows the average time until the collapse of cooperation as a function of the value of the benefit (*b*) for different social norms in the first scenario (MST80). Each symbol represents the average of 100 runs, parameters are as follows: *n* = 100, *c* = 1, *α* = 1, *β* = 4, MST = 80%.

**Fig 5:**
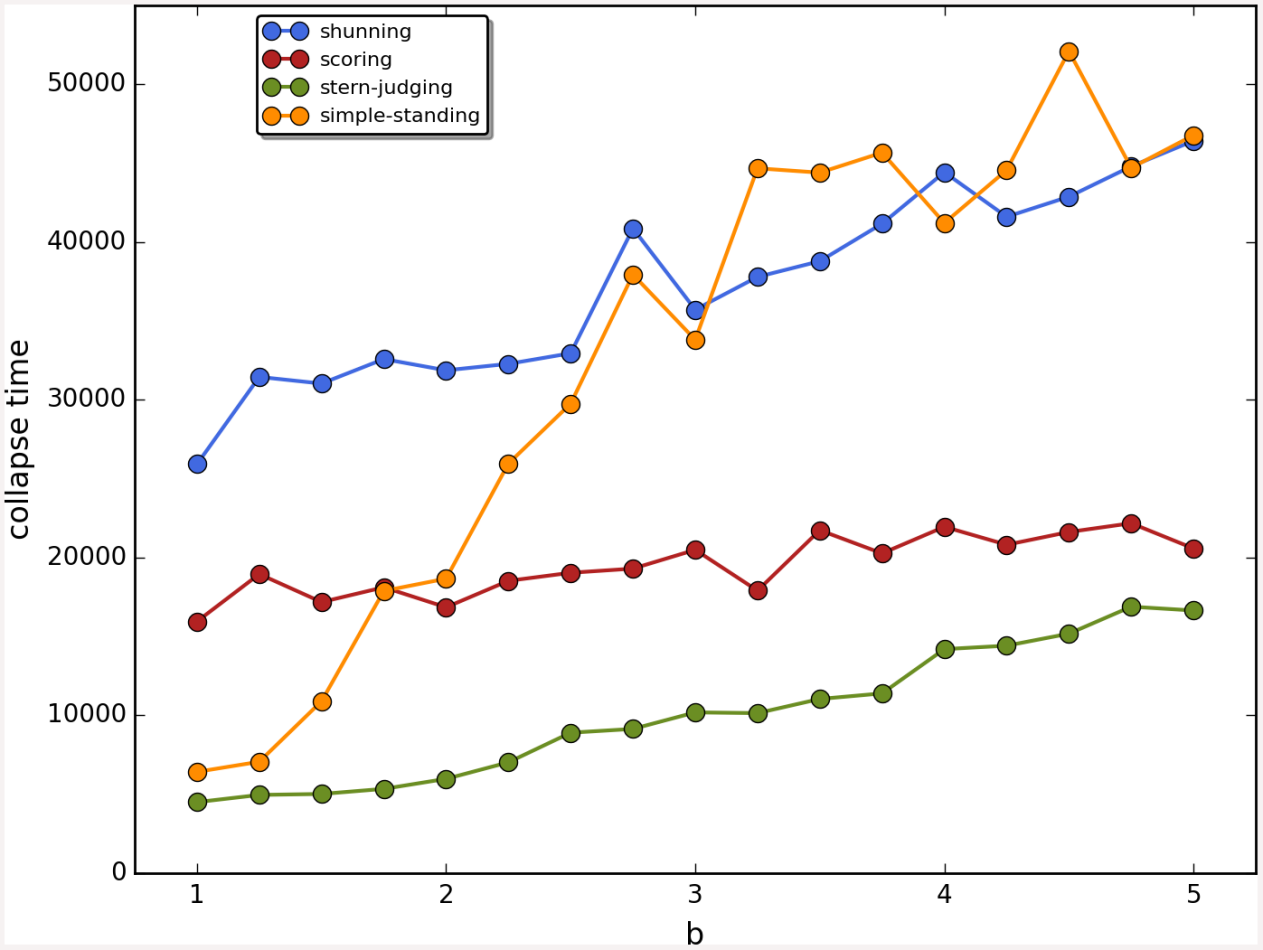
The average time elapsed till the collapse of cooperation, MST90. The figure shows the average time until the collapse of cooperation as a function of the value of the benefit (*b*) for different social norms in the second scenario (MST90). Each symbol represents the average of 100 runs, parameters are as follows: *n* = 100, *c* = 1, *α* = 1, *β* = 4, MST = 90%.

**Fig 6:**
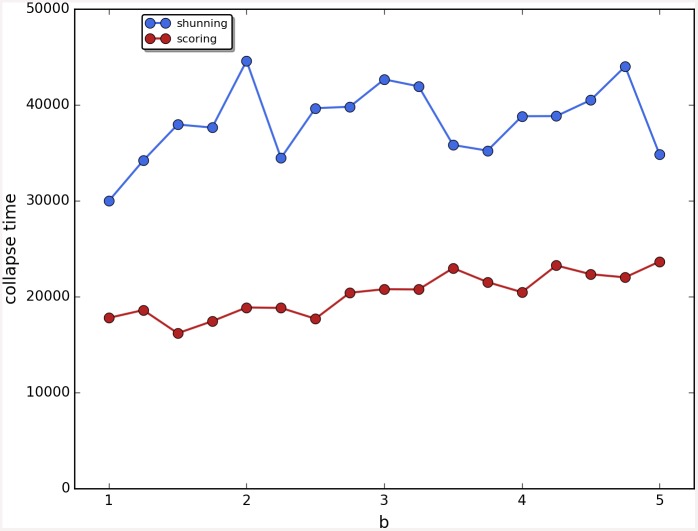
The average time elapsed till the collapse of cooperation, BETA6. The figure shows the average time until the collapse of cooperation as a function of the value of the benefit (*b*) for different social norms in the third scenario (BETA6). Each symbol represents the average of 100 runs, parameters are as follows: *n* = 100, *c* = 1, *α* = 1, *β* = 6, MST = 80%.

**Fig 7:**
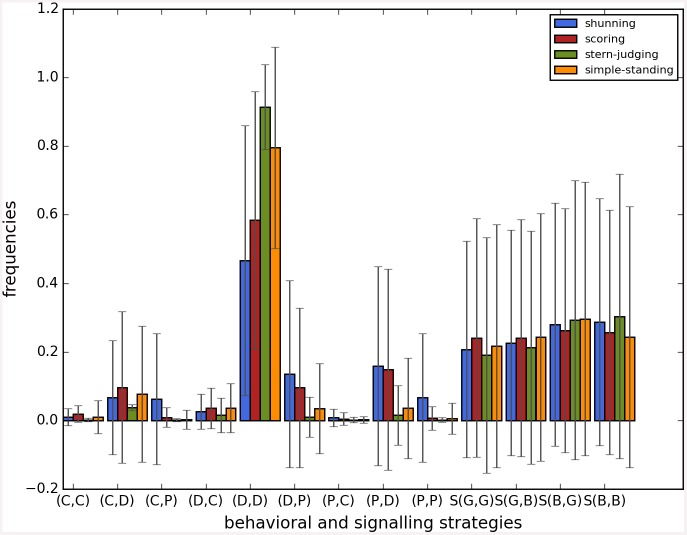
The frequencies of the behavioural and signalling strategies at the end of the simulations, MST80. The figure shows the frequencies of the behavioural and signalling strategies at the end of the simulations. Each bar represents the average of 100, error bars represent standard deviation, parameters are as follows: *n* = 100, *c* = 1, *α* = 1, *β* = 4, MST = 80%. The x axis shows the behavioral or signalling strategy, the y axis shows the average frequency of that strategy when runs were terminated.

**Fig 8:**
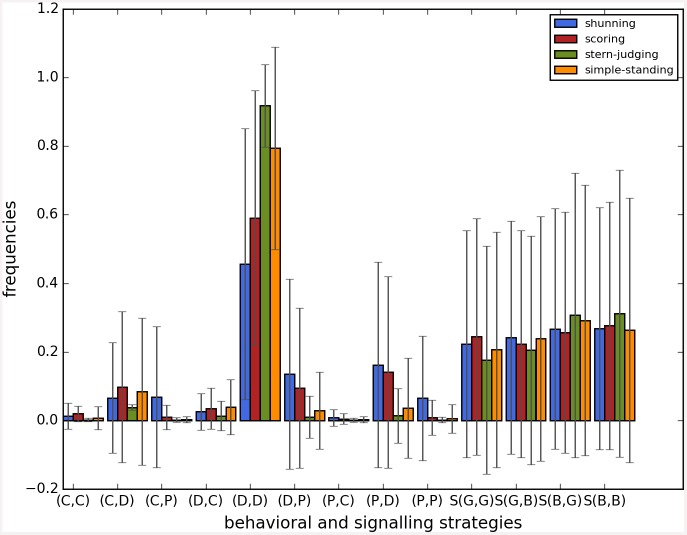
The frequencies of the behavioural and signalling strategies at the end of the simulations, MST90. The figure shows the frequencies of the behavioural and signalling strategies at the end of the simulations. Each bar represents the average of 100 runs, error bars represent standard deviation, parameters are as follows: *n* = 100, *c* = 1, *α* = 1, *β* = 4, MST = 90%. The x axis shows the behavioral or signalling strategy, the y axis shows the average frequency of that strategy when runs were terminated.

**Fig 9:**
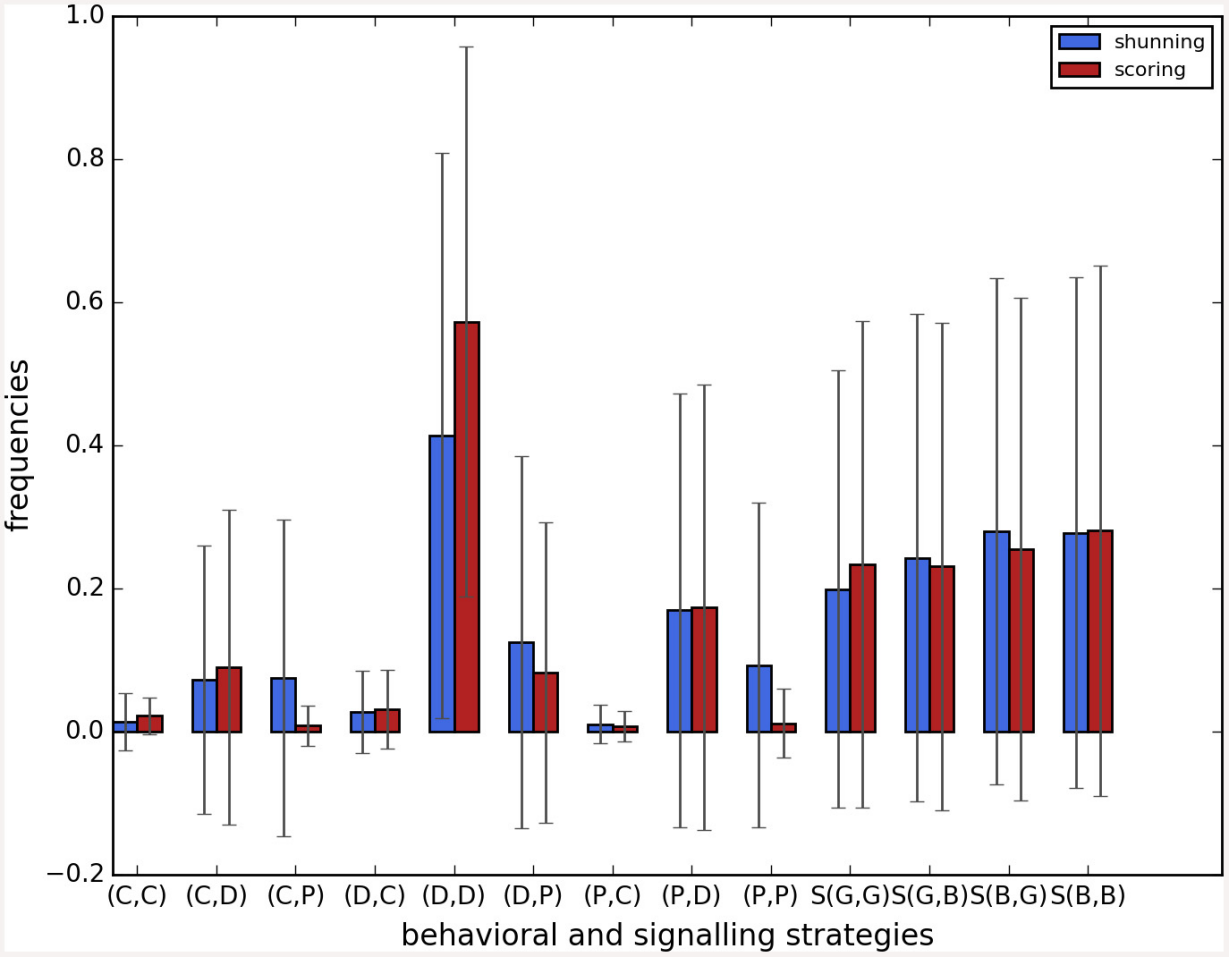
The frequencies of the behavioural and signalling strategies at the end of the simulations, BETA6. The figure shows the frequencies of the behavioural and signalling strategies at the end of the simulations. Each bar represents the average of 100 runs, error bars represent standard deviation, parameters are as follows: *n* = 100, *c* = 1, *α* = 1, *β* = 6, MST = 80%. The x axis shows the behavioral or signalling strategy, the y axis shows the average frequency of that strategy when runs were terminated.

Cooperation disappeared in almost all runs. Shunning and simple-standing seems to be more resistant to the informational uncertainty created by cheating than scoring and stern-judging. Figs 1–3 depict the collapse times as a function of *b* for the 3 main scenarios respectively (MST = 80%, MST = 90% and *β* = 6; see S1, S2 and S3 Tables for the data from which all summary figures were generated). While cooperation disappeared in all runs in case of stern-judging; cooperation survived above the 0.05 threshold after 10^4^ generations in some of the runs using the norms shunning and simple-standing and scoring (see Table 2). Figs 4–6 show the average collapse times as a function of *b* for the three main scenarios. As expected the average collapse times for scoring and stern-judging are consistently low, on the other hand shunning performs the best out of the investigated norms at low values of *b*. Simple-standing is the most sensitive to changes in *b*: at low values it performs as bad as stern-judging, however at high values of *b* it allows cooperation to survive as long as shunning on average or even longer. On the other end scoring is the least sensitive out of the investigated norms to changes in *b*, collapse times barely increased at high *b* values. Figs 7–9 depict the frequencies of behavioural and signalling strategies at the end of the runs for the three main scenarios. Not surprisingly the ‘selfish’ behavioural strategy (D,D) was the most frequent at the end of the runs. Other strategies were also found: (C,D), (D,P) and (P,D) mostly in the case of those runs that utilised norms with punishment (i.e. scoring and shunning, see Figs 7–9). The distribution of signalling strategies appears to be random, which is the result of no selection for or against honesty.

**Table 2:** The number of runs where cooperation survived in the three main scenarios. The table shows the number of runs where cooperation survived after 10^4^ steps above the 0.05 threshold in the three main scenarios as a function of the investigated social norms, out of 1700 runs that were made for each norm under each scenario (17x100, where 17 is the number of different *b* values investigated: from 1 to 5 with step 0.25).

	MST80	MST90	BETA6
Shunning	89	105	120
Scoring	2	2	2
Stern-judging	0	0	-
Simple-standing	96	107	-

**Fig 10:**
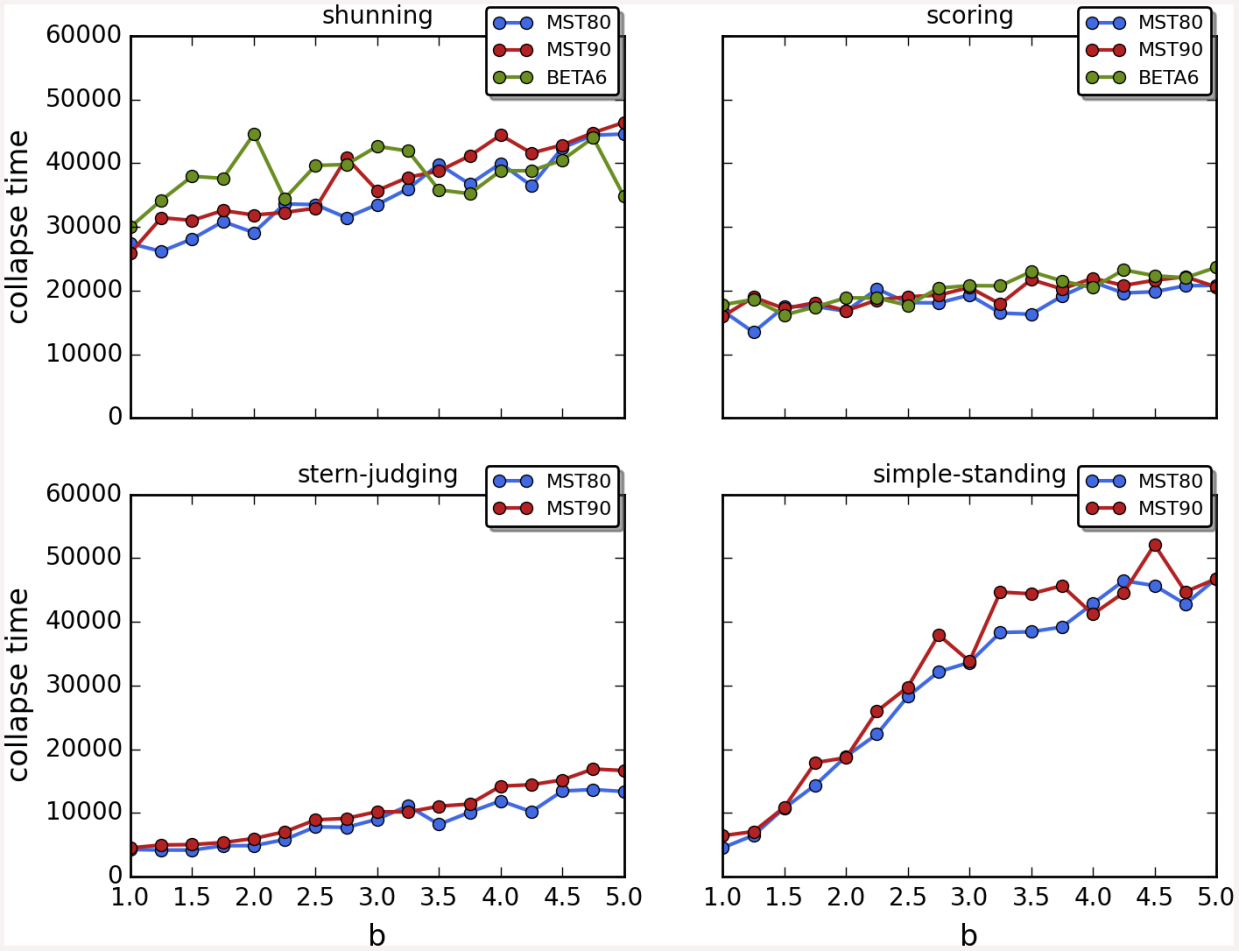
Comparison of the three main scenarios. The figure shows the average time until the collapse of cooperation as a function of the value of the benefit (*b*) for different social norms for the three main scenarios. Colour code: (a) MST80: blue, (b) MST90: red, (c) BETA6: green.

**Fig 11:**
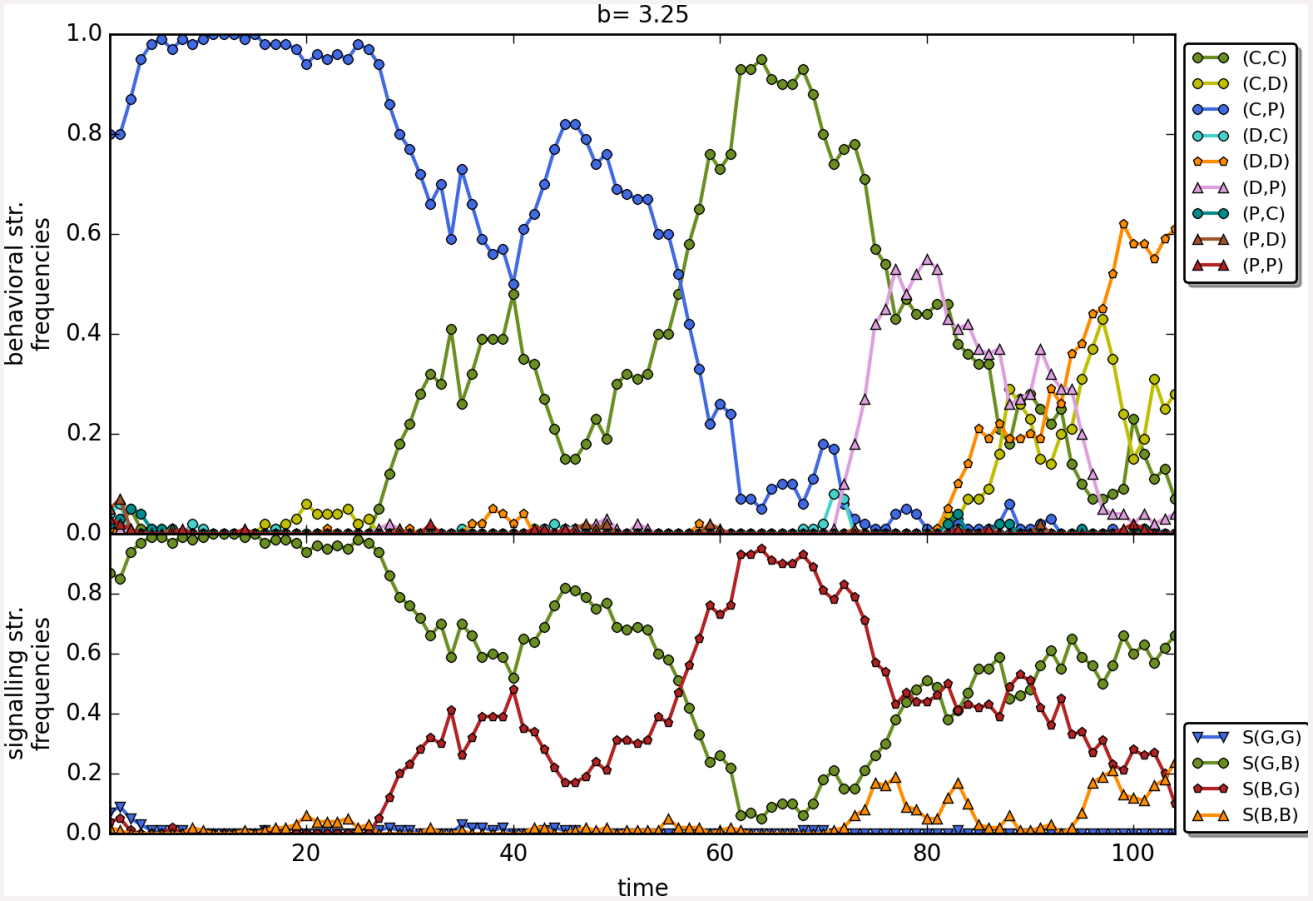
The timeline of an individual run using the social norm shunning. The figure shows the change in the frequencies of the behavioural and signalling strategies as a function of time for the social norms shunning. Numbers on the x axis denote 100 steps. Parameters: *n* = 100, *b* = 3.25, *c* = 1, *α* = 1, *β* = 4, MST = 80%. Colour code for behavioural strategies: (C,C).

**Fig 12:**
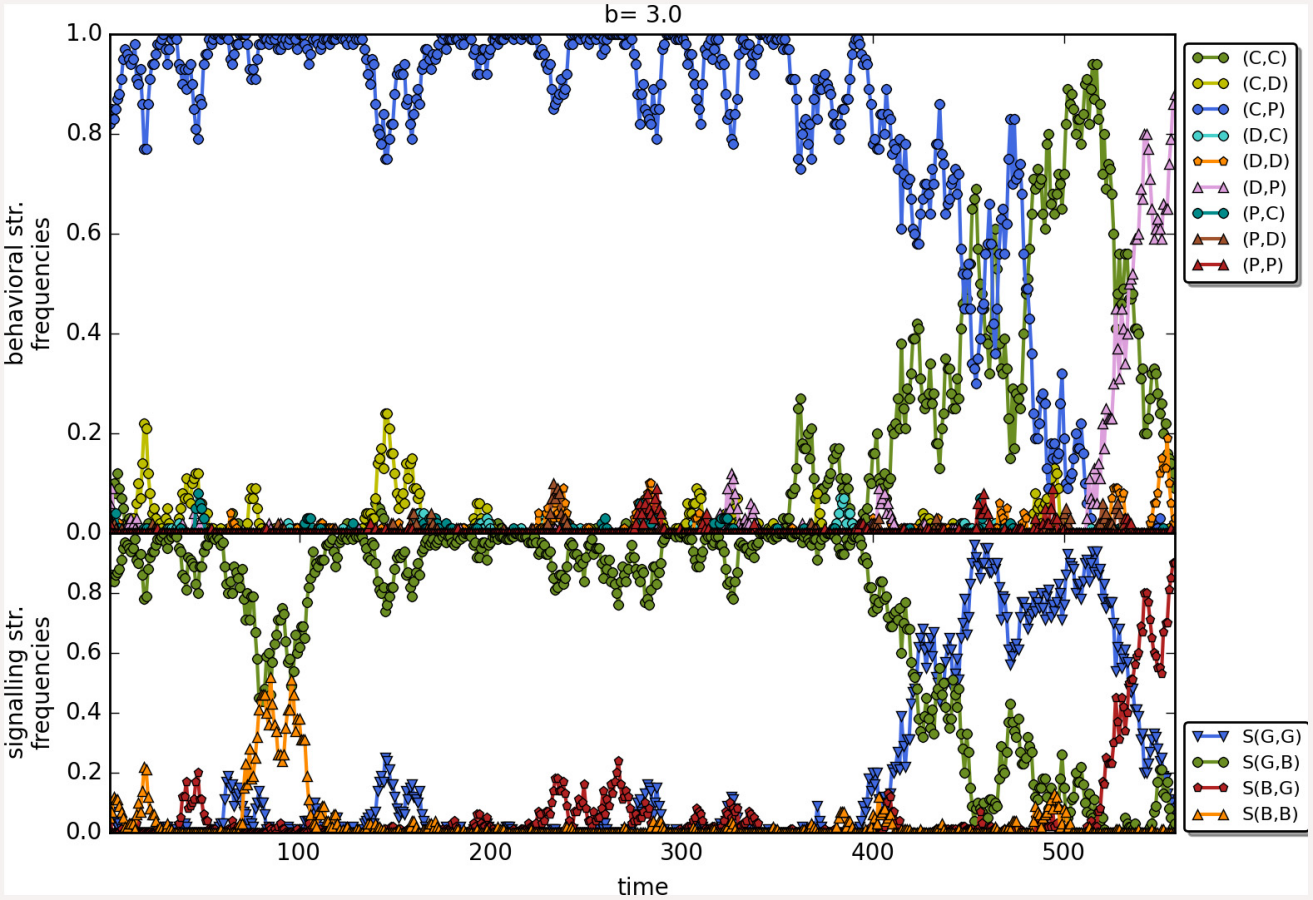
The timeline of an individual run using the social norm scoring. The figure shows the change in the frequencies of the behavioural and signalling strategies as a function of time for the social norms scoring. Numbers on the x axis denote 100 steps. Parameters: *n* = 100, *b* = 3.25, *c* = 1, *α* = 1, *β* = 4, MST = 80%.

**Fig 13:**
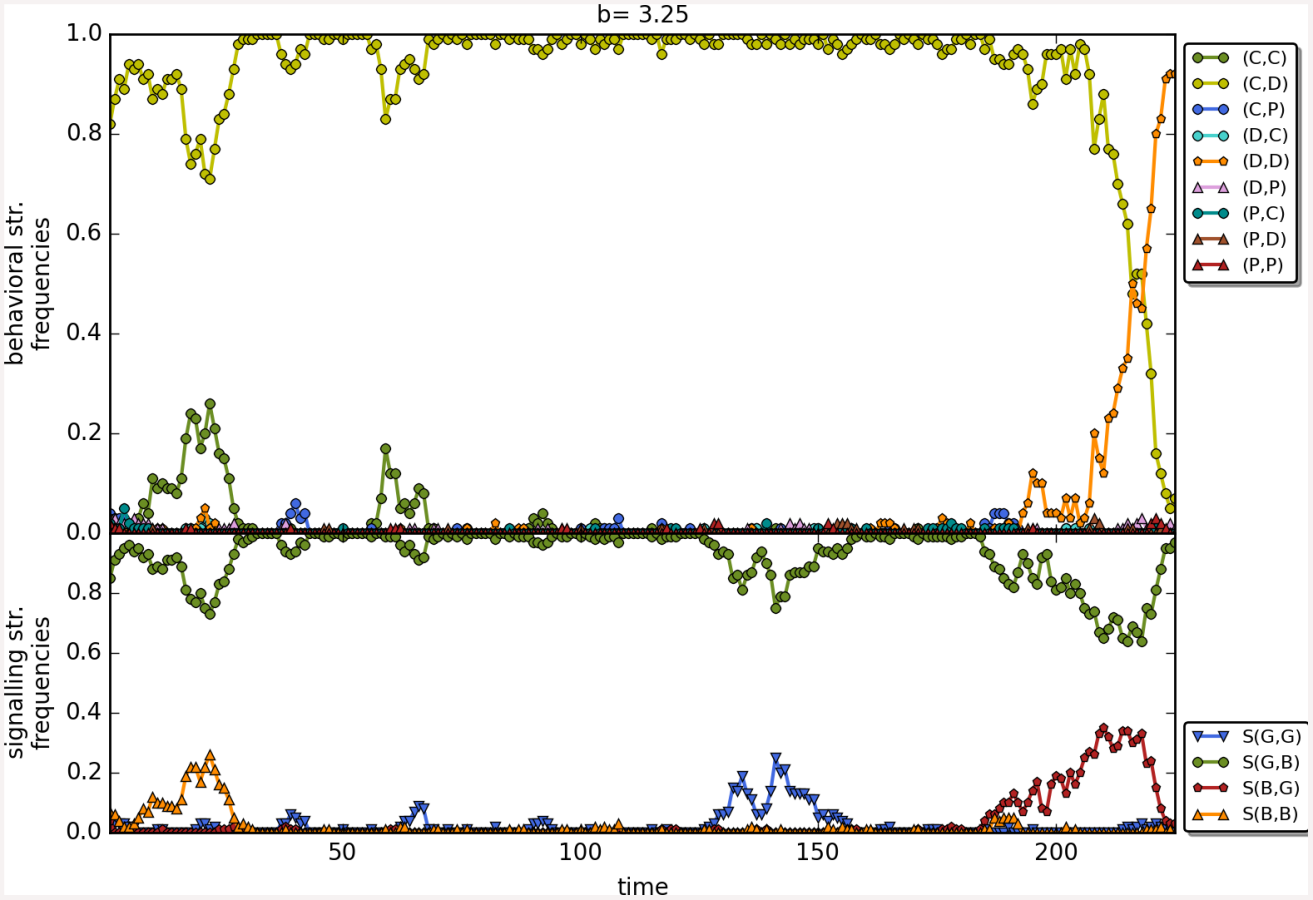
The timeline of an individual run using the social norm stern-judging. The figure shows the change in the frequencies of the behavioural and signalling strategies as a function of time for the social norms stern-judging. Numbers on the x axis denote 100 steps. Parameters: *n* = 100, *b* = 3.25, *c* = 1, *α* = 1, *β* = 4, MST = 80%.

**Fig 14:**
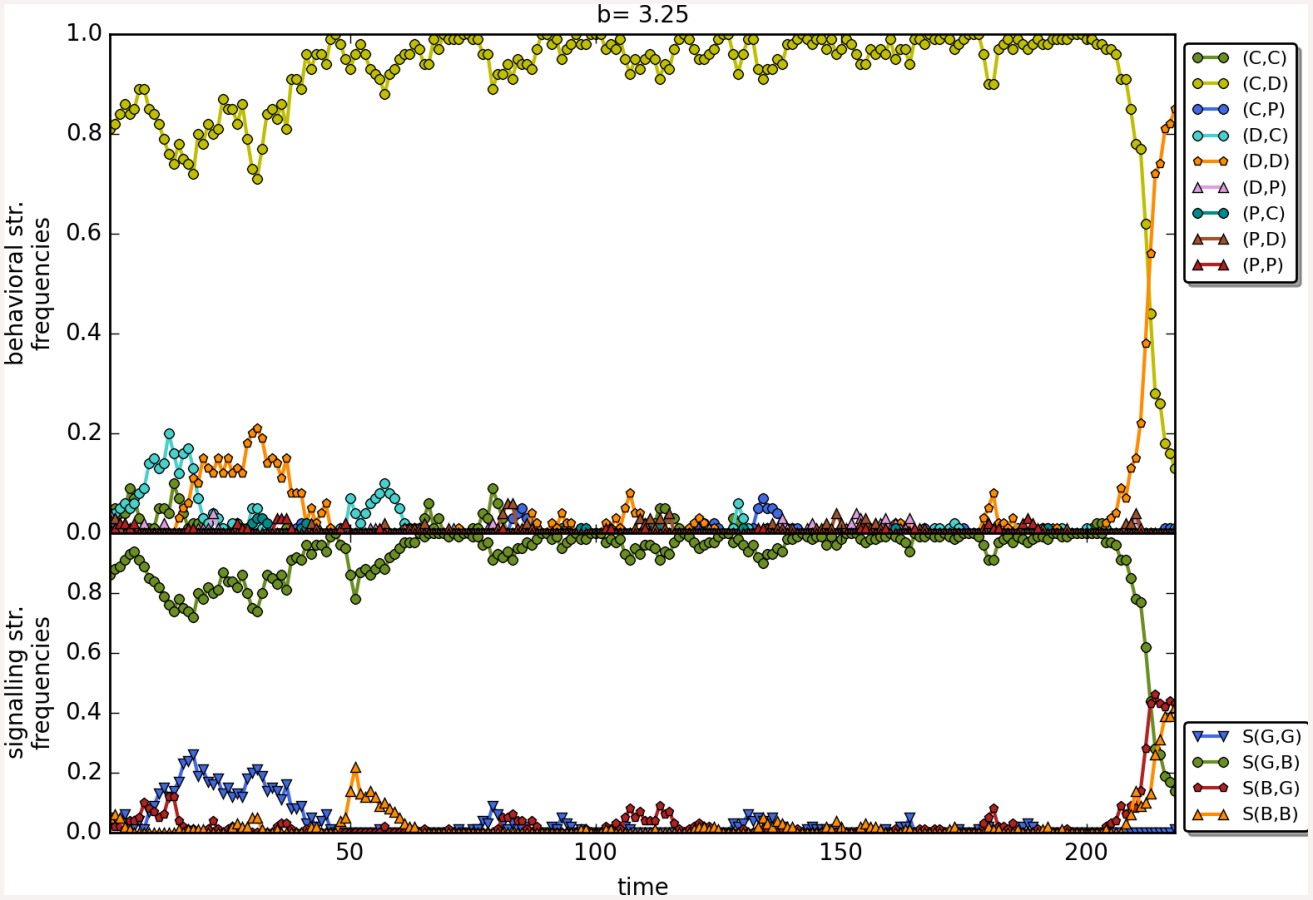
The timeline of an individual run using the social norm simple-standing. The figure shows the change in the frequencies of the behavioural and signalling strategies as a function of time for the social norms simple-standing. Numbers on the x axis denote 100 steps. Parameters: *n* = 100, *b* = 3.25, *c* = 1, *α* = 1, *β* = 4, MST = 80%.

The results are robust to the changes in the initial frequencies of strategies as well as in change of the value of *β*; see Fig 10 for a comparison of the results as a function of the three main scenarios. There are no apparent differences, perhaps shunning does slightly better at low values of *b* with *β* = 6. Last but not least, Figs 11–14 depict individual runs for each norm (see Figs A–E in S1 File for shunning, Figs A–E in S2 File for scoring, Figs A–E in S3 File for stern-judging and finally Figs A–E in S4 File for simple-standing). There is a marked difference between runs with and without punishment. When norms with punishment were used (i.e. scoring and shunning, Figs 11 and 12; and Figs A–E in S1 File, Figs A–E in S2 File respectively) then the original behavioural strategy (C,P) is very often replaced by some other cooperative strategies: (C,D), (D,C) or (C,C), and later these strategies were replaced by selfish types (mostly D,D). On the other hand, the original behavioural strategy (C,D) was mostly replaced directly by the selfish one (D,D) when using norms without punishment (simple-standing and stern-judging, Figs 13 and 14; and Figs A–E in S3 File, Figs A-E in S4 File respectively).

## Discussion

Indirect reciprocity crucially depends on the coherence of reputational scores in the population. We have shown that the introduction of deceptive strategies destroys this coherence, thus destroys cooperation itself. This effect is highly robust against the variation of the norms and parameters used in the simulation, though some norms and parameter combination can delay this effect more than some others. Norms treating donors that interact with receivers in bad reputation non-discriminately (i.e. assign good or bad reputation independently of the action taken by the donor; see shunning and simple-standing, Table 1) generally do better and they do better as function of *b*. On the other hand, norms (scoring, simple-standing) that assign reputation after an action to receivers in bad reputation based on the action of the donor itself collapse fast and they are not sensitive to the changes in *b* (i.e. collapse as fast regardless of high benefit). This is due to the fact that these later norms (scoring, simple-standing) are more sensitive to the information uncertainty created by the cheaters than the former ones. Also, there is a difference between norms in the sequence of events that leads to the disappearance of cooperation: in case of norms using punishment (shunning and scoring) the original behavioural strategy (C,P) is very often replaced by other cooperative strategies: (C,D), (D,C) or (C,C), and in turn these strategies are invaded by selfish ones. On the other hand, in case of norms without punishment (stern-judging, simple-standing) the original cooperative strategy (C,D) is usually directly replaced by selfish ones, mostly by (D,D). We think that this difference is due to the fact that punishment is costly for both donors and receivers; thus in the face of information uncertainty donors are better off not punishing at all. This selects for switching to other cooperative strategies, even to indiscriminate cooperation (C,C), especially if the frequency of cooperators is high (which it is at the beginning of the simulations). Of course, these cooperative strategies are also vulnerable to the information uncertainty, and indiscriminate cooperators are highly vulnerable to selfish behavioural strategies so cooperation disappears even after the switch.

What is the reason behind this collapse of cooperation? Assuming a finite group of cooperators playing an indirect reciprocity (IR) game, for cooperation to collapse two conditions have to be met: (i) the chance of assigning incorrect reputation has to be over the threshold, (ii) non-cooperative strategies have to be present. The first condition depends on the error of communication and on the frequency of dishonest signallers in the population. Ohtsuki et al. [2] have shown that allowing high rounds of communication can overcome the first problem (see Appendix 1). Thus, the question remains whether selection can eliminate dishonest signallers before the probability of assigning incorrect reputation reaches the threshold. Unfortunately, within the framework of IR the answer is no. Communication has no cost or benefit for the signaller, hence signalling strategies are expected to change according to random drift. The only chance for honesty to prevail is that dishonest strategies are lost due to the stochasticity of system while they are still rare. Thus, the first condition is guided by a random drift. In fact it can be shown that the frequency of an opinion which is actually in minority will increase by communication (see Appendix 2). Thus, this kind of opinion polymorphism remains present after any number of communication rounds with high probability. Accordingly, the time it takes cooperation to collapse depends on the social norm, and on the cost-benefit ratio (e.g. see Figs 4–6). Social norms that are not sensitive to the action of the donor towards receivers in bad reputation (i.e. shunning and simple-standing) and low cost-benefit ratio (*c*/*b*) favours cooperation, thus the collapse of cooperation can be delayed, but the system is not evolutionarily stable, it is at the mercy of random drift.

Few existing studies that investigate the role of deception or cost in the formation of reputational systems support our conclusions. Nakamaru and Kagawa [18] investigated a repeated prisoners’ dilemma game with reputations added and assumed that some individuals might lie about their own reputations. They found that if the number of interactions within a turn was high then TFT like cooperative strategies could be stable against (self-) liars; on the other hand if the number of interactions within a turn was low then the number of cheaters increased. They also found that conditional rumour users, who spread good rumour only if they had the corresponding personal experience could be also stable against cheaters. Note that both of these results rested on the fact that crosschecking is possible within the Nakamaru and Kagawa [18] model, assuming that the number of interactions within a turn is high. Once crosschecking is no longer efficient (as in the IR framework) Nakamaru and Kagawa [18] found that cheaters could increase. Note, that cheaters in the Nakamaru and Kagawa [18] model were self-liars; they lied only about their own good reputation. They were also defectors, thus Nakamaru and Kagawa [18] assumed linkage disequilibrium where deception was linked to non-cooperative behaviour. Here we investigated a more general case and found that cooperation was destroyed even without this restricting assumption.

Suzuki & Kimura [19] investigating a general IR game with costly reputation building found that this cost -however small- prevented the evolution of reputational systems in IR games, and thus resulted in the collapse of cooperation. Their result is very similar to ours and highlights a crucial problem with the assumptions of the IR models, namely that they all assume cost-free, honest reputational systems [2, 17, 20].

A related problem is that there is a growing number of models using reputational scores as a tool to generate and maintain cooperative behaviour in social dilemma games without even considering the possibility that the communication of these reputational scores need not be honest [21, 22, 23, 24]. While the current model investigates an IR game, it is reasonable to assume that the introduction of strategic deception results in the collapse of cooperation in other games as well for the exact same reasons as discussed above.

Recent experiments on the role of gossip might seem to contradict our conclusion as all of them show that gossip serves as a reliable tool to increase the cooperative effort in social dilemma games [25,26,27,28,29,30]. We think however, that there is no contradiction. Most of these experiments tests simple social dilemmas, like the dictator game [28,29,30] or the public goods game [25]. In many cases there is only a “threat of gossip” but no actual gossiping takes place [28,29,30]. Moreover this threat was only credible when the actors could be identified reliably [28,29] which is in line with our argument (i.e. the need of efficient crosschecking). More importantly, none of those experiments where gossiping took place provided incentive to be deceitful [26,27]: “Nonetheless, our design represents a benign world without any incentive for gossip authors to cheat” (pp. 2535 [27]). In sharp contrast to these results, empirical studies of natural situations, where conflicting interests abounds, show that gossip is very often used as “verbal aggression” [12]. Verbal aggression is deceit and misinformation, and on most occasions the very aim of it is to destroy the reputation of potential rivals. Such verbal aggression is a prominent feature of human communication and social life from college student networks, through economics to politics. In the light of such empirical data there is no a priori reason to assume that gossip must be honest.

Last but not least, one may argue that a more realistic implementation of communication could favour cooperative behavioural rules [3]. There are, however, theoretical reasons to think that the origin of honest communication of reputations cannot be explained in the IR framework. For honesty to be selected for either there should be shared interest between the signaller and the receiver [31], or signals have to have a marginal cost that can be related to its marginal benefits [32]; or receivers have to be able to crosscheck signals [32]. The first and second case clearly do not hold, as the signaller has no benefits (and thus no shared interest, as they never meet the receivers again) and has no costs (since signals are cost-free) under the strict IR assumption. The third case is highly unlikely to hold either since the IR framework assumes: (i) a large (infinite) population where ‘any two players are supposed to interact *at most once* with each other’ [1], (ii) large number of communication rounds [2] and (iii) random interactions [1,20]. In the light of these assumptions it is the most unlikely (explicitly impossible by definition *sensu* Nowak & Sigmund [1]) that any given individual would be able to follow all the interactions that is necessary to crosscheck the honesty of signals. Note that even if there was a chance for an individual to crosscheck an observer, the other individuals have to believe her report on the honesty of that observer. This however, would raise the problem of “second order honesty”, i.e. the problem that the reports of crosschecking observers might be deceptive as well. In other words, even potentially efficient crosschecking would not be a solution as it would only push the communication problem to another level (i.e. who will crosscheck the crosschecking observers’ reports). All in all, in order to keep the reputational system functional one would have to be able to come up with an alternative mechanism that can maintain the coherence of reputational scores. Please note, that we do not argue that IR cannot work at all, we just call attention to the fact that honesty cannot be selected for *within the IR framework*.

## Conclusions

We have shown that allowing cheating signalling strategies to evolve in games of IR undermines the coherence of reputational scores maintained by gossip, which in turn results in the collapse of cooperation. We have further shown that this is a direct consequence of the general assumption of the IR framework. Thus to maintain the coherence of the IR framework the honesty of communication requires special explanation. In turn, the current model predicts that cooperation maintained by indirect reciprocity should be rare or absent in those societies which lack those means that can maintain the coherence of reputational scores in the face of deceptive communicational strategies.

## Appendix 1

### Fixation time of the correct opinion in the Ohtsuki model

We have *i* number of individuals giving incorrect and *N-i* individuals transmitting correct information about the reputation of a third individual in the population. The information exchange algorithm defined by Ohtsuki et al. [2] (two individuals selected randomly, and one of them accepts the opinion of the other about the reputation of a randomly selected third individual) is a random drift process. The question is how many steps are needed on average that all opinion will be correct for a given individual? It is known from the literature that the average number of steps needed to fixation can be computed as (see e.g. [33,34]):

$$t_{i}=\frac{i}{3}(2N-i).$$ (1)

In our case *i* = *Nμ*/2 thus

$$t_{N\mu /2}=\frac{N\mu }{6}(2N-N\mu /2)=N^{2}\frac{\mu }{12}(4-\mu ).$$ (2)

If *μ*<<1 then previous equation

$$t_{N\mu /2}\approx \frac{1}{3}\mu N^{2}.$$ (3)

Since *N* = 100 and *μ* = 0.02 in [2], thus $t_{N\mu /2}\approx \frac{2}{3}N$ approximately. Since each player has on average *T* = *N* chances of asking in communication round within two cycle of IR games [2], it is highly probable that the correct information will be fixed or the number of individuals giving incorrect information will be at most very small.

## Appendix 2

### Polymorphic equilibrium due to dishonesty

The information exchange algorithm is the same as in Appendix 1, however if a dishonest individual accepts the opinion of another individual it will transmit just the opposite information later when it becomes an information donor. (There can be three different dishonest strategies, but for the sake of simplicity we consider the worst case scenario, that is, the completely dishonest strategy that communicates the opposite in every case.) We will show that in the above defined information system the opinion followed by the minority about any given individual increases—regardless whether it is correct or not-, thus fixation of an opinion is highly improbable (more precisely fixation needs a very long time [35]).

Let us denote the honest and dishonest signallers by *h* and $\bar{h}$ respectively and the frequencies of the signallers as $p_{G,h},p_{G,\bar{h}},p_{B,h},p_{B,\bar{h}}$, where the first index denotes the transporting signal and the second index refers to honesty. Assume that the population is in a state when G is more frequently transmitted than B, and these transmitted reputations are distributed roughly evenly between honest and dishonest individuals. Because of random mutations and numerous exchange of information this last one is a plausible assumption. By using the above defined notations these assumptions mean that $p_{G,h}\approx p_{G,\bar{h}},p_{B,h}\approx p_{B,\bar{h}}$ and $p_{G,*}>p_{B,*}$, where wildcard denotes either *h* or $\bar{h}$. In this system two kinds of change is possible: either if one receives G and signals B, or if one receives B and communicates G. The average probability that communicated reputation about a third person will change is as follows (where the first parenthesis gives the frequency of the signallers giving the given signal and the second one gives the frequency of the receivers who will signal the opposite on the next occasion, i.e. the frequency of dishonest signallers):

$$\Delta_{B,G}=\Delta_{G\to B}-\Delta_{B\to G}=\frac{1}{2}\left(p_{G,\bar{h}}+p_{G,h}\right)\left(p_{B,\bar{h}}+p_{G,\bar{h}}\right)-\frac{1}{2}\left(p_{B,\bar{h}}+p_{B,h}\right)\left(p_{G,\bar{h}}+p_{B,\bar{h}}\right),$$ (4)

where Δ_*G→B*_ is the probability that a G changes to a B (first part in Eq 4) and Δ_*B→G*_ is the probability that a B changes to a G (second part in Eq 4), thus Δ_*B*,*G*_ > 0 means that frequency of B increases. These values can be computed by producing the frequencies defined above and adding the different cases together. After rearranging the equation we have:

$$\Delta_{B,G}=\frac{1}{2}\left(\left[p_{G,h}-p_{B,h}\right]+\left[p_{G,\bar{h}}-p_{B,\bar{h}}\right]\right)\left(p_{G,\bar{h}}+p_{B,\bar{h}}\right).$$ (5)

Since $p_{G,*}>p_{B,*}$ (where asterisk denotes a wildcard) thus Δ_*B*,*G*_ > 0. Consequently the frequency of communicated B will increase if signalled B is rare compared to signalled G. The above argument is the same if we change the role of G and B, so G increases if it is rare in the population. This situation where the rare type enjoys an advantage over frequent type results in a polymorphism, i.e. both types co-exists in the population.

## Supporting Information

S1 File (PDF)

S2 File (PDF)

S3 File (PDF)

S4 File (PDF)

S1 Table (PDF)

S2 Table (PDF)

S3 Table (PDF)
