# Supplementary material for: Deception Undermines the Stability of Cooperation in Games of Indirect Reciprocity
Source: PLoS One. 2016 Jan 29;11(1):e0147623. doi: 10.1371/journal.pone.0147623 (PMC4732813; doi:10.1371/journal.pone.0147623)

S1 File. The timeline of individual runs using the social norm shunning.

The Figs A - E show the change in the frequencies of the behavioral and signalling strategies as a function of time for the social norms shunning. Numbers on the x axis denote 100 steps. Parameters: *b*= 1,2,3,4,5 for the figures respectively, *n*=100, *c*=1, *α*=1, *β*=4, MST=80%.

Figure A. Shunning.

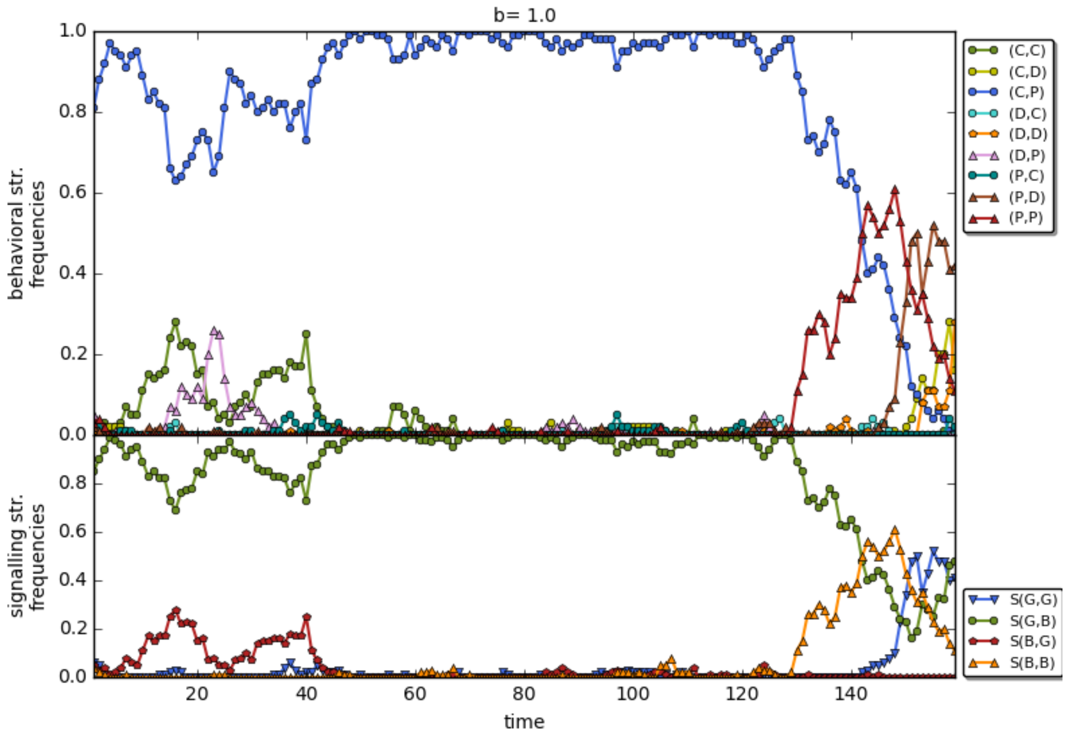

Figure B. Shunning.

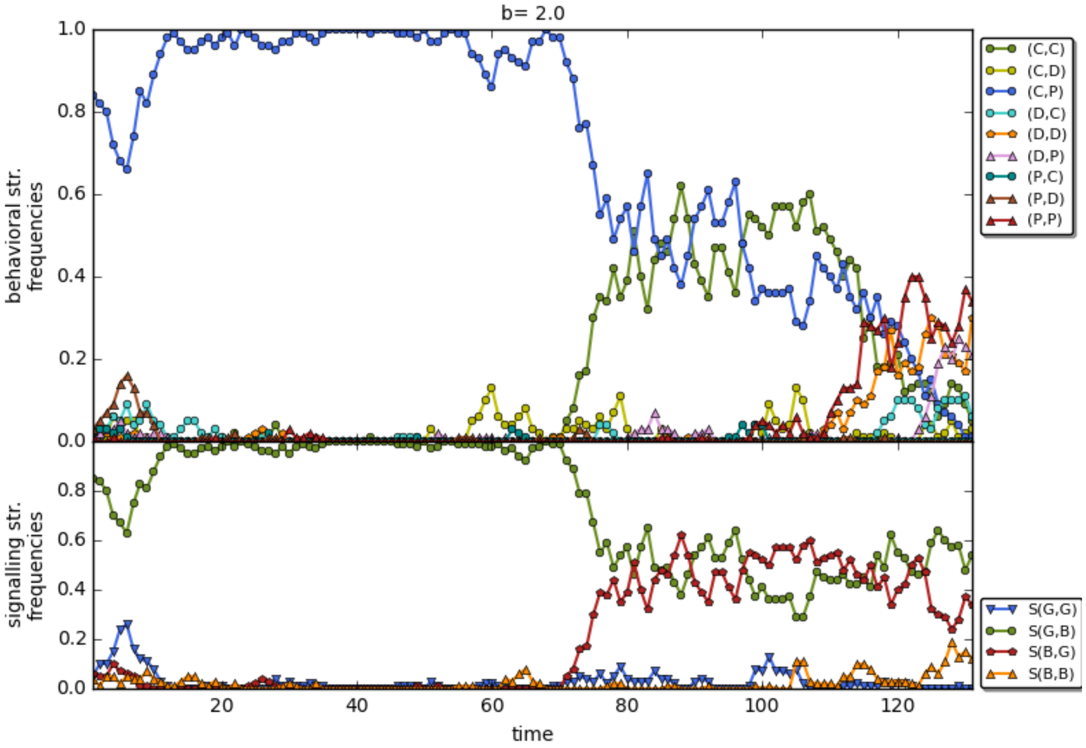

Figure C. Shunning.

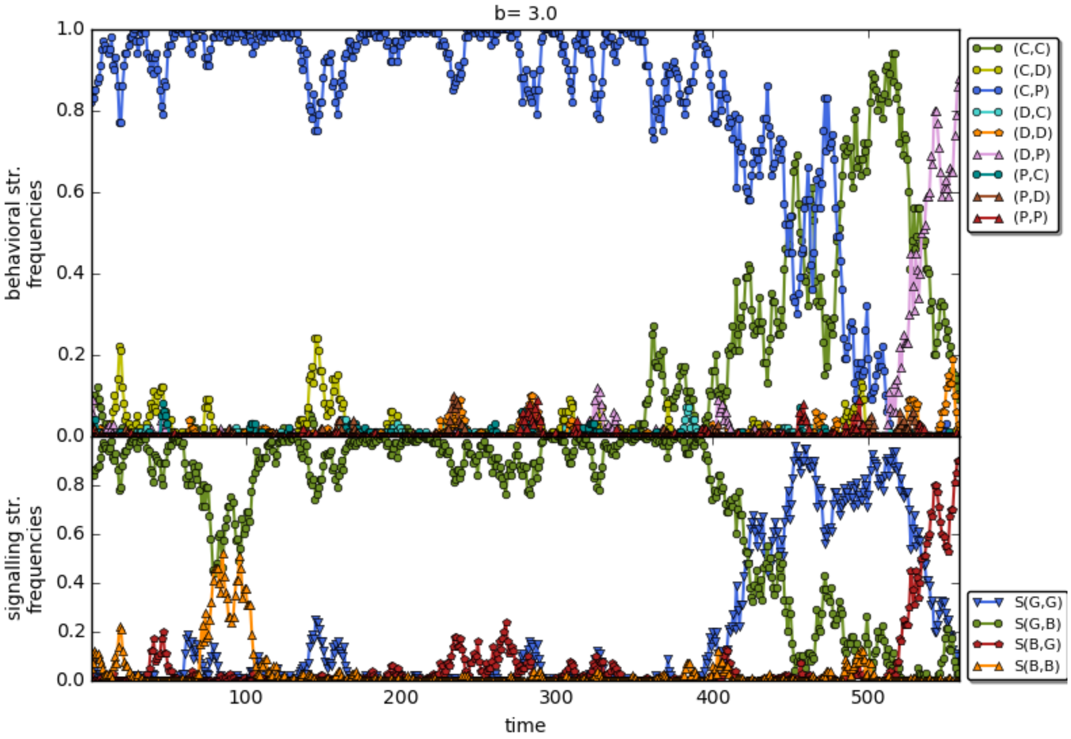

Figure D. Shunning.

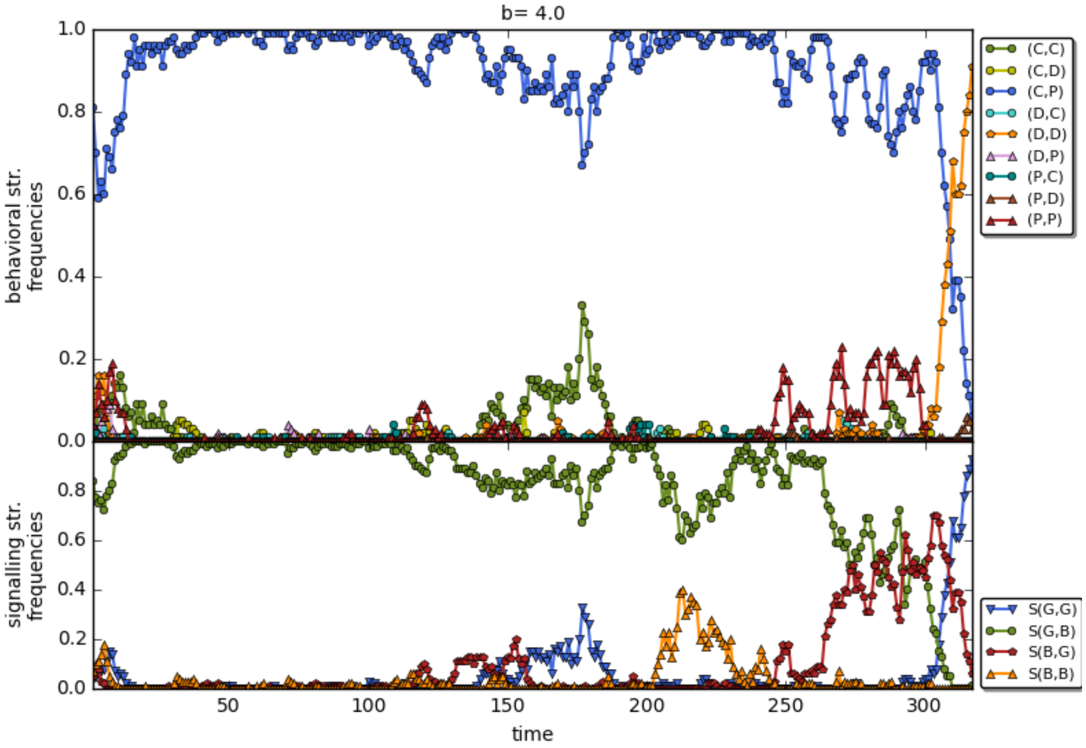

Figure E. Shunning.

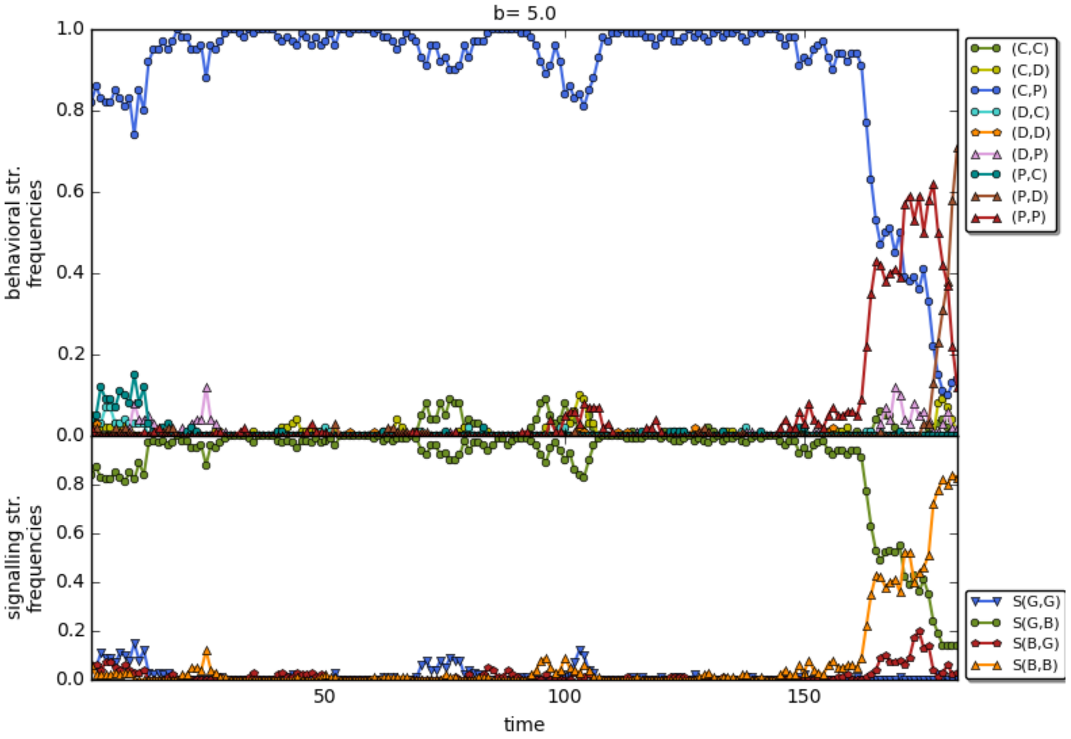

Supplement: S1 File — (PDF) [file pone.0147623.s001.pdf]
