# Supplementary material for: Deception Undermines the Stability of Cooperation in Games of Indirect Reciprocity
Source: PLoS One. 2016 Jan 29;11(1):e0147623. doi: 10.1371/journal.pone.0147623 (PMC4732813; doi:10.1371/journal.pone.0147623)

S2 File. The timeline of individual runs using the social norm scoring.

The Figs A-E show the change in the frequencies of the behavioral and signalling strategies as a function of time for the social norms scoring. Numbers on the x axis denote 100 steps. Parameters: *b*= 1,2,3,4,5 for the figures respectively, *n*=100, *c*=1, *α*=1, *β*=4, MST=80%.

Figure A. Scoring.

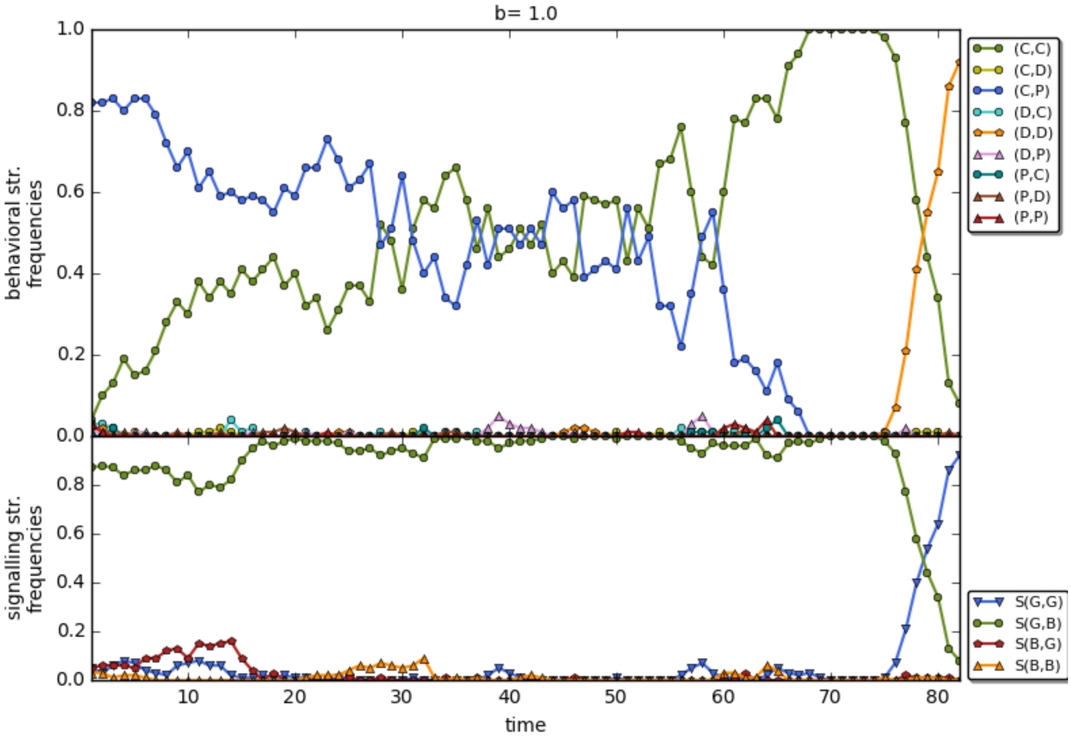

Figure B. Scoring.

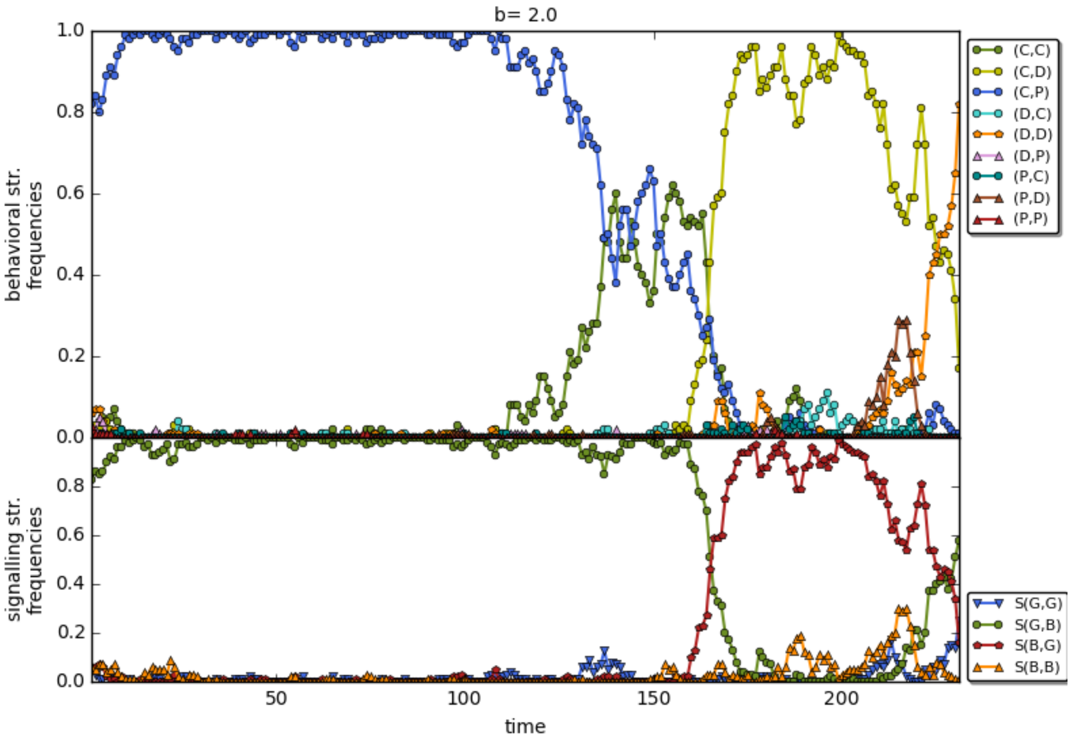

Figure C. Scoring.

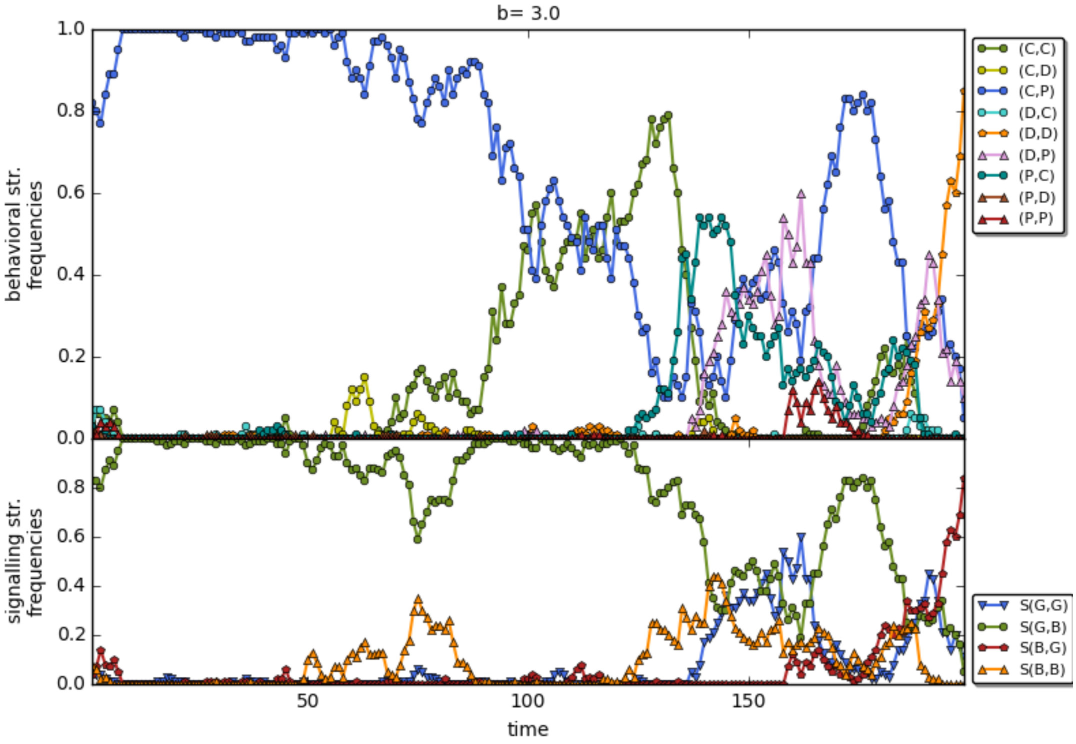

Figure D. Scoring.

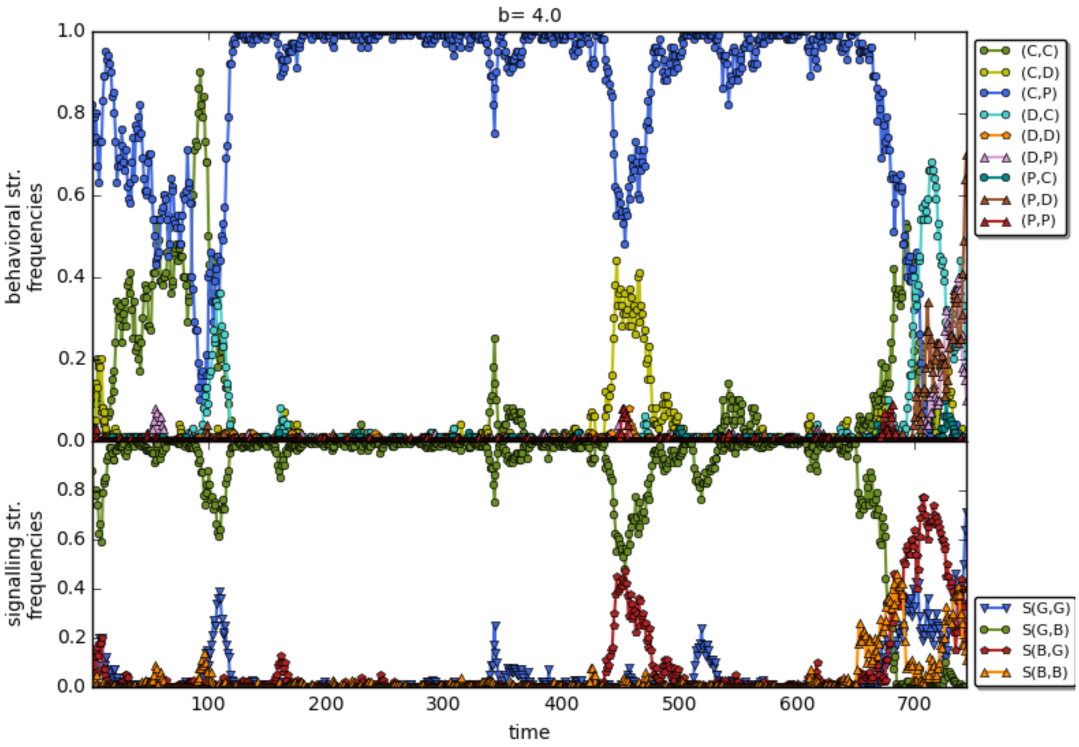

Figure E. Scoring.

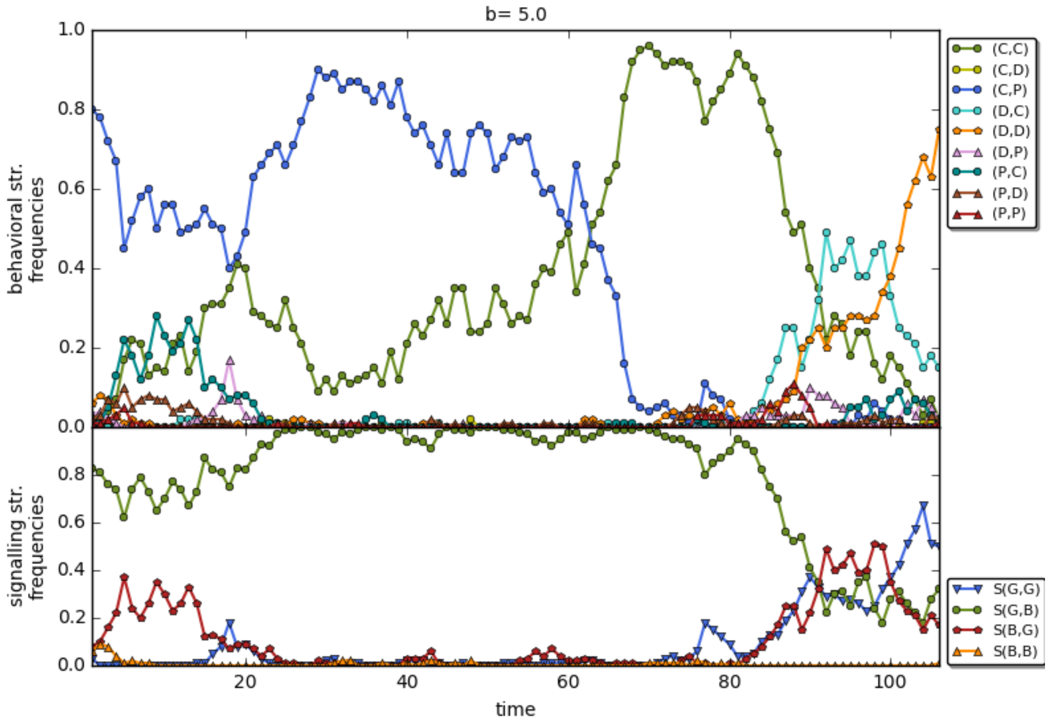

Supplement: S2 File — (PDF) [file pone.0147623.s002.pdf]
