# Supplementary material for: Deception Undermines the Stability of Cooperation in Games of Indirect Reciprocity
Source: PLoS One. 2016 Jan 29;11(1):e0147623. doi: 10.1371/journal.pone.0147623 (PMC4732813; doi:10.1371/journal.pone.0147623)

S3 File. The timeline of individual runs using the social norm stern-judging.

The Figs A - E show the change in the frequencies of the behavioral and signalling strategies as a function of time for the social norms stern-judging. Numbers on the x axis denote 100 steps. Parameters: b= 1,2,3,4,5 for the figures respectively, n=100, c=1, α=1, β=4, MST=80%.

Figure A. Stern-judging.

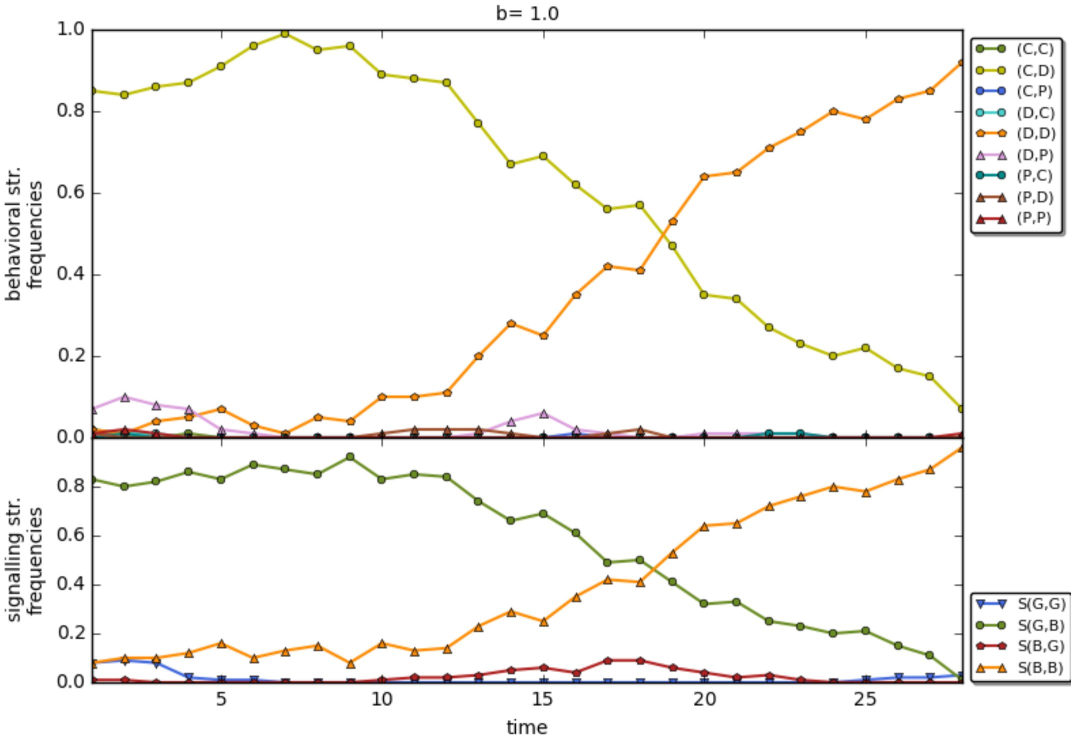

Figure B. Stern-judging.

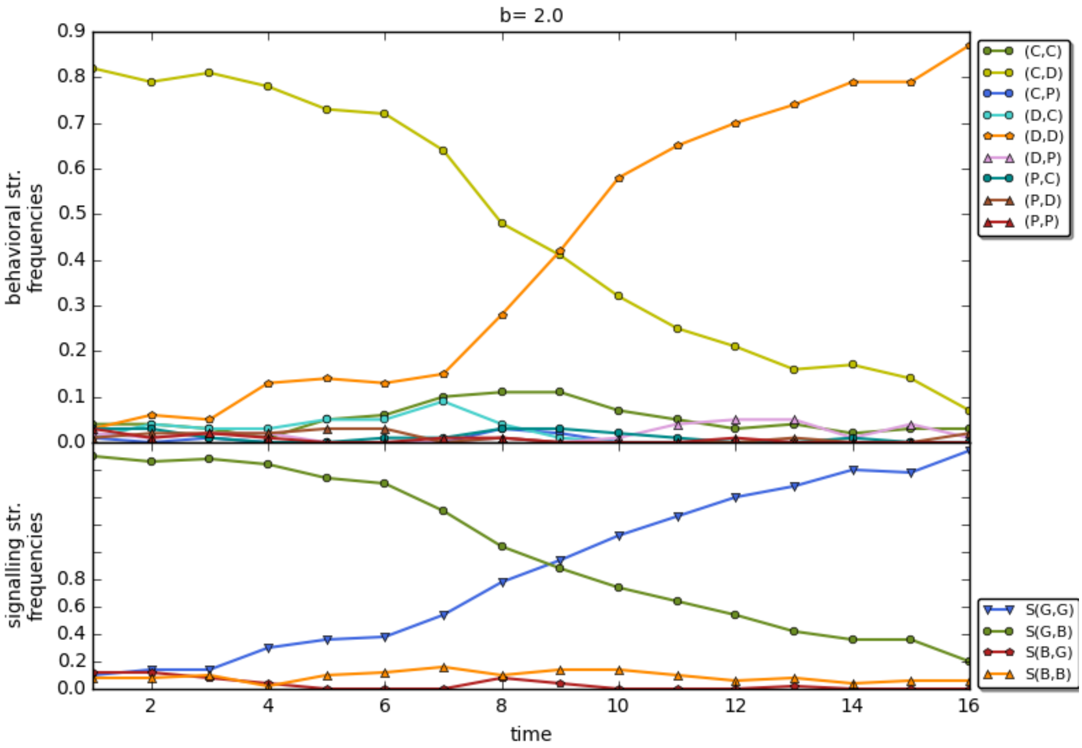

Figure C. Stern-judging.

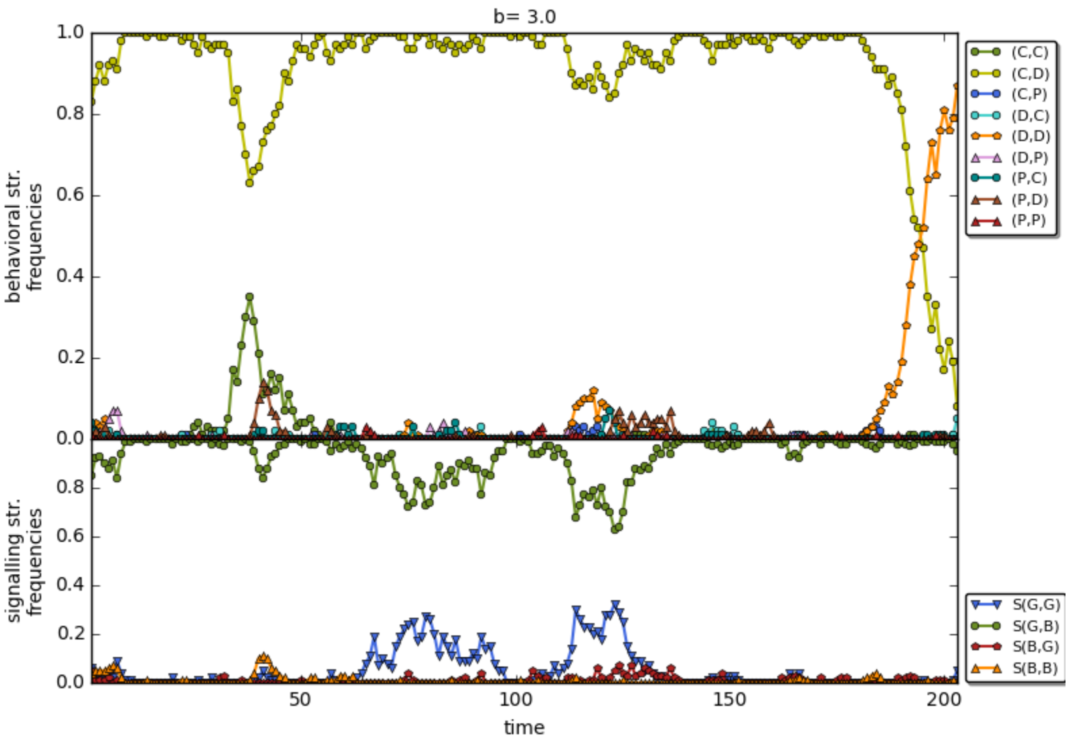

Figure D. Stern-judging.

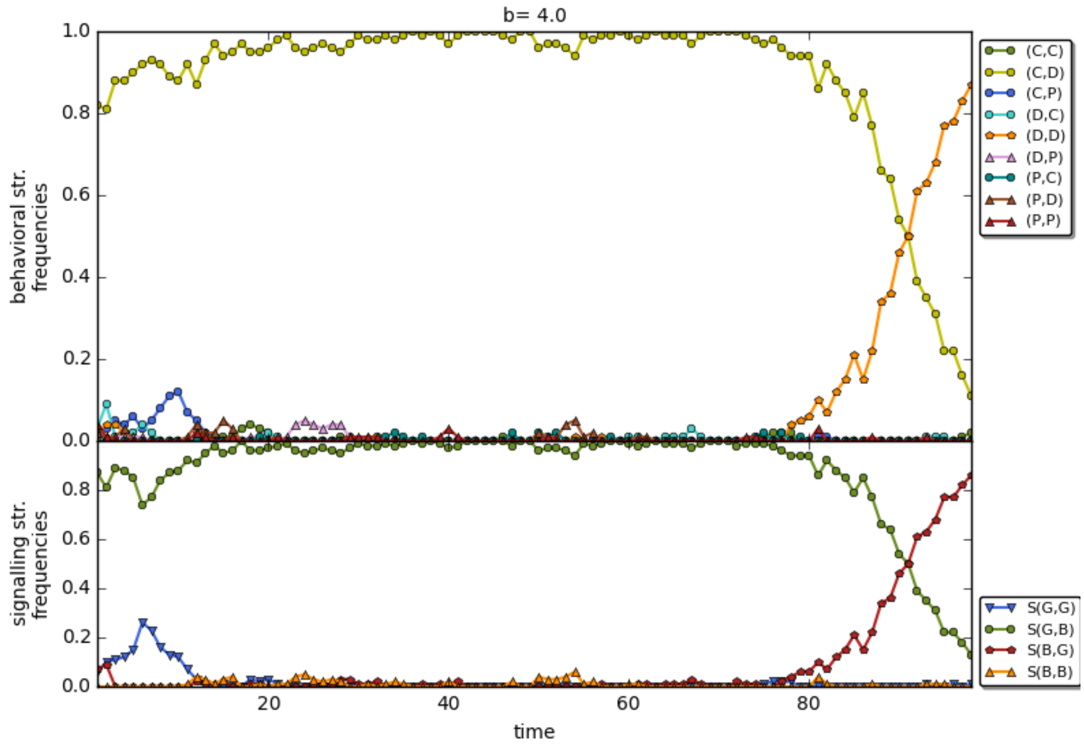

Figure E. Stern-judging.

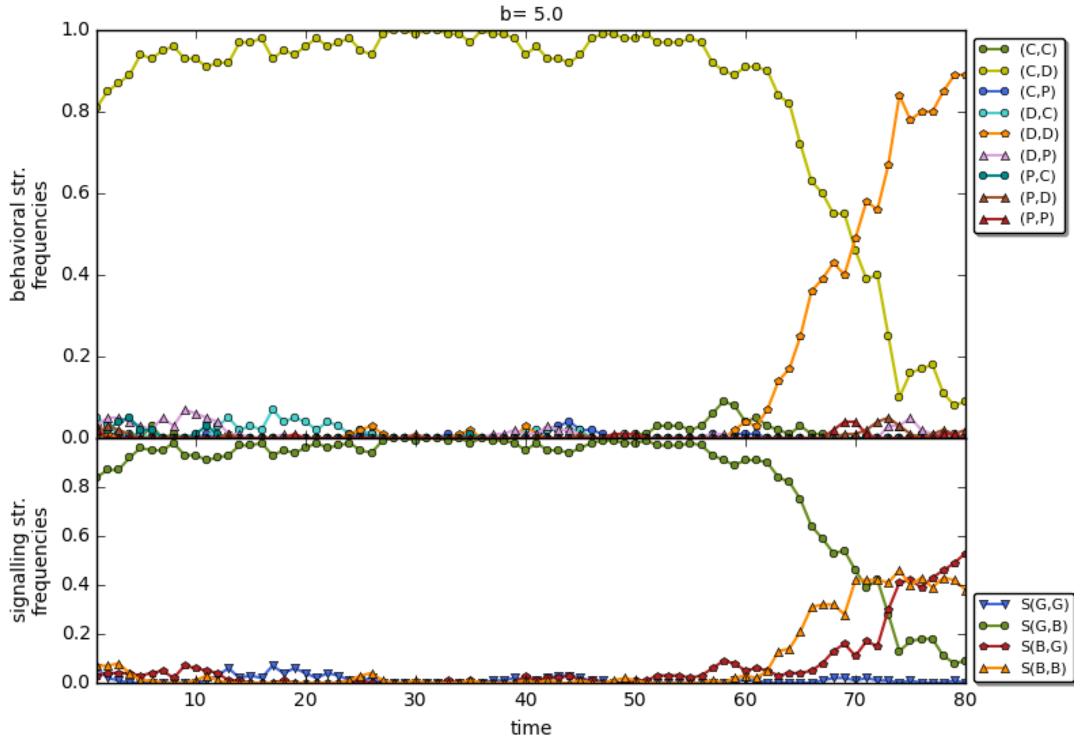

Supplement: S3 File — (PDF) [file pone.0147623.s003.pdf]
