# Supplementary material for: Deception Undermines the Stability of Cooperation in Games of Indirect Reciprocity
Source: PLoS One. 2016 Jan 29;11(1):e0147623. doi: 10.1371/journal.pone.0147623 (PMC4732813; doi:10.1371/journal.pone.0147623)
Supplement: S1 Table — (PDF) [file pone.0147623.s005.pdf]

S1 Table. Results of the MST=80% scenario.

shunning

b	time_to_cc	(C,C)	(C,D)	(C,P)	(D,C)	(D,D)	(D,P)	(P,C)	(P,D)	(P,P)	S(G,G)	S(G,B)	S(B,G)	S(B,B)	cooperatio
1	34398	0	0,01	0,04	0	0,5	0	0	0,39	0,06	0,01	0,66	0,07	0,26	0,002
1	15874	0	0,91	0	0	0	0	0	0,08	0,01	0	0	0,08	0,92	0,036
1	64700	0	0,01	0,04	0,01	0,94	0	0	0	0	0	0,04	0	0,96	0,01
1	31260	0	0	0,04	0	0,91	0,01	0	0,02	0,02	0,01	0,04	0,27	0,68	0,012
1	26994	0	0	0,04	0	0	0,12	0,06	0	0,78	0,05	0,13	0	0,82	0,046
1	7906	0	0	0	0,07	0	0,71	0,01	0,17	0,04	0,04	0,03	0,17	0,76	0,048
1	11759	0	0,09	0,03	0	0,58	0,29	0	0	0,01	0,28	0,11	0,6	0,01	0,042
1	8206	0	0	0,04	0	0,96	0	0	0	0	0,96	0,04	0	0	0,042
1	3089	0	0,01	0,04	0	0,95	0	0	0	0	0,12	0,02	0,02	0,84	0,006
1	30907	0	0	0	0,05	0	0,93	0,01	0,01	0	0	0,03	0,06	0,91	0,044
1	4296	0,02	0,05	0,04	0,01	0,58	0,01	0	0,28	0,01	0,01	0,9	0,09	0	0,046
1	4819	0,04	0	0,04	0	0,92	0	0	0	0	0	1	0	0	0,024
1	1344	0	0	0,04	0	0	0,9	0	0	0,06	0	0,04	0,9	0,06	0,028
1	11540	0	0	0	0,19	0	0	0	0,81	0	0,81	0	0	0,19	0,042
1	12664	0,01	0,1	0	0,02	0,86	0,01	0	0	0	0,1	0,06	0,83	0,01	0,048
1	1423	0	0,01	0,04	0	0,95	0	0	0	0	0,01	0,04	0,94	0,01	0,03
1	16977	0,05	0	0,02	0	0	0,1	0	0,76	0,07	0,07	0,02	0,11	0,8	0,048
1	86849	0	0,03	0,04	0,13	0	0,8	0	0	0	0,8	0,17	0,01	0,02	0,048
1	13234	0	0,05	0	0,05	0,89	0	0,01	0	0	0,01	0,06	0,92	0,01	0,046
1	32822	0	0	0,01	0,03	0,35	0,02	0,02	0,57	0	0	0,6	0	0,4	0,046
1	1111	0,05	0	0,04	0	0,04	0,46	0	0,41	0	0	0,13	0,46	0,41	0,038
1	55829	0,03	0,06	0	0	0	0,86	0	0	0,05	0	0,18	0,77	0,05	0,048
1	89091	0	0,06	0,04	0	0	0,66	0	0,24	0	0,06	0,04	0,9	0	0,046
1	2197	0	0	0,04	0,03	0,81	0,08	0	0,04	0	0	0,1	0,85	0,05	0,036
1	2086	0,03	0,02	0,01	0	0,87	0,05	0,02	0	0	0	0,03	0,89	0,08	0,04
1	50536	0	0,08	0	0	0,91	0	0,01	0	0	0,96	0	0,03	0,01	0,04
1	20178	0	0,25	0	0	0,74	0,01	0	0	0	0	0,38	0,25	0,37	0,044
1	4723	0	0,02	0,04	0	0,94	0	0	0	0	0,97	0,01	0,02	0	0,046
1	14725	0,04	0	0,04	0	0,78	0,09	0,01	0	0,04	0,04	0,81	0,11	0,04	0,046
1	49105	0	0,24	0	0	0,74	0	0	0,02	0	0	0,62	0,24	0,14	0,046

1	25252	0	0	0,04	0	0	0	0	0,96	0	0,03	0,01	0	0,96	0,002
1	11029	0	0,03	0,03	0,05	0	0,74	0	0,02	0,13	0,18	0,03	0,79	0	0,038
1	74084	0,04	0	0,04	0	0,91	0	0	0,01	0	0	0,06	0,9	0,04	0,044
1	34507	0,04	0	0,04	0	0,9	0	0	0	0,02	0,03	0,36	0,58	0,03	0,04
1	5018	0	0,08	0,02	0	0	0	0	0,9	0	0,89	0	0,01	0,1	0,048
1	86498	0	0,07	0,04	0	0	0,89	0	0	0	0	0,03	0,97	0	0,04
1	46997	0	0,9	0,01	0	0,09	0	0	0	0	0,08	0,01	0	0,91	0,048
1	88197	0	0	0,04	0	0,77	0	0	0,19	0	0	0,04	0	0,96	0
1	59965	0	0,07	0,01	0	0,89	0	0	0	0,03	0,89	0,08	0	0,03	0,042
1	25237	0	0	0	0,06	0,91	0	0	0,03	0	0,06	0,8	0	0,14	0,034
1	1880	0	0,05	0,03	0,05	0,84	0,03	0	0	0	0,84	0,08	0	0,08	0,048
1	19246	0	0	0,04	0	0,82	0,02	0	0,1	0,02	0,9	0,1	0	0	0,038
1	19529	0	0	0,04	0	0,8	0	0	0	0,16	0	0,04	0,78	0,18	0,024
1	8669	0	0,03	0	0,14	0,83	0	0	0	0	0,83	0,02	0,15	0	0,048
1	13461	0	0,94	0,04	0	0	0	0	0,01	0,01	0,01	0,03	0,01	0,95	0,038
1	42955	0,02	0,01	0,04	0	0,91	0,02	0	0	0	0,02	0,04	0,92	0,02	0,044
1	22055	0,01	0,02	0,03	0	0	0,88	0,04	0,02	0	0,94	0,01	0,05	0	0,036
1	7785	0	0,13	0,01	0,02	0	0	0	0,84	0	0	0,03	0,84	0,13	0,048
1	6422	0,04	0,01	0,04	0	0	0	0	0,91	0	0,05	0,01	0	0,94	0,04
1	15859	0	0,1	0,01	0	0,38	0	0,01	0,41	0,09	0,41	0,5	0	0,09	0,046
1	30865	0	0,03	0	0,19	0,05	0,72	0	0,01	0	0,96	0,03	0	0,01	0,038
1	39170	0	0	0,04	0,01	0,73	0	0,06	0	0,16	0,01	0,09	0,18	0,72	0,046
1	9699	0	0	0,04	0	0,29	0	0	0,67	0	0,29	0,04	0,67	0	0,034
1	83277	0	0,05	0	0,1	0,75	0,1	0	0	0	0,85	0	0,14	0,01	0,046
1	6326	0,01	0,02	0,04	0,01	0,85	0,01	0,02	0	0,04	0,04	0,9	0,01	0,05	0,048
1	21292	0	0,09	0	0,03	0,88	0	0	0	0	0	0,02	0,95	0,03	0,048
1	39756	0	0,11	0	0	0	0	0	0,89	0	0	0	1	0	0,048
1	46466	0	0	0	0,21	0,7	0,08	0	0,01	0	0,87	0,02	0	0,11	0,044
1	9334	0	0,09	0,03	0,03	0,81	0	0,01	0,03	0	0,12	0,32	0	0,56	0,048
1	33723	0,01	0	0,04	0	0,01	0,66	0	0	0,28	0,25	0,03	0,43	0,29	0,028
1	21408	0	0	0	0,22	0,76	0	0	0,02	0	0,76	0	0,24	0	0,04
1	25771	0	0	0,04	0	0,54	0	0,03	0,04	0,35	0,02	0,09	0,35	0,54	0,026
1	46891	0	0	0,04	0,08	0	0,17	0,05	0,66	0	0,74	0,22	0,03	0,01	0,044

1	35043	0	0,08	0	0	0,92	0	0	0	0	0,91	0	0,09	0	0,038
1	39869	0	0,13	0	0	0,86	0,01	0	0	0	0,14	0,01	0,85	0	0,046
1	16852	0	0	0,04	0	0	0,36	0,02	0,58	0	0,58	0,04	0,02	0,36	0,036
1	26164	0,02	0	0,04	0,01	0	0	0,01	0,05	0,87	0,17	0,05	0	0,78	0,042
1	38006	0	0,13	0	0,03	0,8	0,04	0	0	0	0,13	0,83	0,04	0	0,046
1	28308	0	0	0,03	0,09	0	0	0	0,86	0,02	0,1	0	0,9	0	0,048
1	4258	0,03	0	0,02	0	0,9	0,02	0,03	0	0	0,02	0,95	0,03	0	0,048
1	100000	0,12	0	0,82	0	0	0	0	0,06	0	0,21	0,79	0	0	0,814
1	50669	0	0,05	0	0,1	0,67	0,12	0,01	0,05	0	0,67	0,05	0,1	0,18	0,048
1	50794	0	0	0,04	0,03	0	0	0	0,76	0,17	0	0,07	0	0,93	0,022
1	6394	0	0	0,04	0,03	0,93	0	0	0	0	0	0,02	0,95	0,03	0,042
1	2218	0,02	0	0,04	0	0,93	0	0,01	0	0	0	0,8	0,01	0,19	0,032
1	10122	0	0,08	0	0	0,92	0	0	0	0	0,92	0	0,08	0	0,044
1	9539	0	0,94	0	0	0	0	0	0	0,06	0	0	0,06	0,94	0,044
1	19567	0	0,99	0	0	0	0,01	0	0	0	0,01	0,99	0	0	0,048
1	1837	0,01	0,27	0	0	0,72	0	0	0	0	0	0	0,28	0,72	0,042
1	19990	0,04	0	0,04	0	0,92	0	0	0	0	0,04	0,96	0	0	0,032
1	20214	0	0	0	0,08	0,92	0	0	0	0	0	0	0,39	0,61	0,048
1	7107	0	0,94	0,03	0,02	0	0,01	0	0	0	0	0,03	0,03	0,94	0,048
1	12117	0	0	0,04	0	0,01	0,95	0	0	0	0	0,05	0,05	0,9	0,004
1	17452	0,05	0	0	0	0	0,93	0,02	0	0	0,93	0,05	0	0,02	0,046
1	4194	0	0	0,04	0	0,3	0,12	0	0,44	0,1	0,28	0,17	0,33	0,22	0,032
1	9494	0	0,02	0,04	0,02	0,32	0	0,04	0,56	0	0,33	0,02	0,06	0,59	0,048
1	13576	0	0	0	0,09	0,67	0,23	0	0,01	0	0,38	0	0	0,62	0,048
1	5432	0	0	0,04	0	0,54	0,31	0,01	0,07	0,03	0,61	0,04	0,19	0,16	0,036
1	66960	0,05	0	0,02	0	0,92	0	0	0,01	0	0,07	0,06	0	0,87	0,044
1	93225	0	0,01	0,04	0	0,16	0,02	0	0,76	0,01	0,01	0	0,92	0,07	0,026
1	20179	0	0	0,04	0	0,8	0,16	0	0	0	0,8	0,04	0,16	0	0,032
1	40794	0	0,06	0,01	0	0,93	0	0	0	0	0,91	0,01	0	0,08	0,04
1	11098	0,02	0,01	0,04	0	0,55	0,22	0,02	0,12	0,02	0,37	0,08	0	0,55	0,042
1	72049	0	0	0,04	0	0,2	0,76	0	0	0	0,8	0,2	0	0	0,032
1	1501	0	0	0,04	0	0,09	0,83	0	0	0,04	0,12	0,05	0,83	0	0,018
1	61639	0	0,19	0	0,02	0,78	0	0	0	0,01	0	0,78	0,22	0	0,046

1	2272	0,01	0,15	0,04	0	0,14	0,17	0	0,48	0,01	0,16	0,69	0	0,15	0,046
1	38613	0	0	0,04	0,04	0,55	0,06	0	0,3	0,01	0,36	0,05	0	0,59	0,032
1	40361	0	0	0,04	0,1	0,86	0	0	0	0	0,99	0,01	0	0	0,038
1	3098	0	0	0,04	0	0	0	0	0,96	0	0	0,04	0	0,96	0,002
1,25	9722	0	0	0,04	0	0,1	0	0	0,86	0	0,02	0,14	0,84	0	0,018
1,25	5375	0	0,25	0	0	0,74	0,01	0	0	0	0,25	0	0,01	0,74	0,046
1,25	6992	0,01	0	0,01	0,12	0,85	0	0	0,01	0	0,88	0	0,01	0,11	0,048
1,25	34785	0,01	0	0,04	0	0,01	0,56	0,05	0	0,33	0,01	0,59	0,33	0,07	0,046
1,25	6713	0	0,09	0	0	0,91	0	0	0	0	0,57	0	0,43	0	0,038
1,25	8878	0	0	0	0,19	0,06	0,69	0	0	0,06	0,72	0,19	0,09	0	0,042
1,25	3220	0	0,11	0,01	0,01	0	0	0	0,87	0	0	0,11	0,87	0,02	0,046
1,25	14554	0	0	0	0,16	0,83	0,01	0	0	0	0,42	0,58	0	0	0,046
1,25	50134	0	0	0,04	0	0,06	0,66	0	0,23	0,01	0	0,09	0,9	0,01	0,024
1,25	43503	0	0,09	0	0	0,89	0,01	0	0	0,01	0,85	0,03	0,08	0,04	0,042
1,25	9710	0,03	0	0,04	0	0,91	0	0,02	0	0	0,07	0,02	0,91	0	0,048
1,25	6388	0,05	0	0	0	0,92	0,01	0	0	0,02	0	0,9	0,06	0,04	0,048
1,25	54372	0	0,12	0,04	0	0,84	0	0	0	0	0	0,04	0	0,96	0,002
1,25	63307	0	0	0	0	0,53	0,17	0,21	0,09	0	0,62	0,38	0	0	0,048
1,25	22340	0	0,14	0	0	0,86	0	0	0	0	0,02	0,01	0,97	0	0,046
1,25	11854	0,03	0,01	0,04	0	0	0	0	0,62	0,3	0,02	0,23	0,62	0,13	0,036
1,25	38386	0	0	0	0,08	0	0	0	0,92	0	0	0,78	0,15	0,07	0,046
1,25	7416	0	0,09	0	0,01	0,82	0	0	0,02	0,06	0,73	0,07	0,2	0	0,042
1,25	43036	0,01	0,95	0,02	0	0,01	0	0	0	0,01	0,04	0,05	0,01	0,9	0,044
1,25	14541	0	0,15	0,04	0	0,59	0,01	0,04	0	0,17	0	0,6	0,05	0,35	0,046
1,25	23931	0	0,02	0	0,07	0,91	0	0	0	0	0,09	0,89	0	0,02	0,038
1,25	8727	0	0,09	0,02	0	0,01	0,6	0	0,28	0	0,02	0,29	0,69	0	0,048
1,25	26604	0	0,01	0,04	0	0,85	0	0,09	0,01	0	0,85	0,04	0	0,11	0,046
1,25	33885	0	0,07	0,02	0	0,28	0,63	0	0	0	0,7	0,02	0,28	0	0,036
1,25	15498	0	0,09	0,04	0,03	0,22	0	0,01	0,61	0	0,03	0,31	0,01	0,65	0,044
1,25	49860	0	0,09	0,04	0	0,83	0,03	0	0,01	0	0	0,03	0,92	0,05	0,048
1,25	21649	0	0	0,04	0	0,96	0	0	0	0	0,03	0,91	0	0,06	0,008
1,25	22313	0	0,11	0	0	0,88	0	0,01	0	0	0	0,01	0,88	0,11	0,042
1,25	48695	0,03	0	0,03	0	0,5	0,44	0	0	0	0,02	0	0,97	0,01	0,034

1,25	16379	0	0	0,04	0	0,86	0	0,09	0	0,01	0	0,04	0,87	0,09	0,046
1,25	5588	0	0,11	0,02	0	0,73	0	0	0,14	0	0,25	0,02	0,73	0	0,038
1,25	41665	0	0,16	0,03	0	0	0,8	0,01	0	0	0,22	0,01	0,03	0,74	0,044
1,25	3405	0,01	0	0,04	0	0,64	0,22	0	0,01	0,08	0,86	0,12	0,02	0	0,048
1,25	66762	0,03	0	0	0,03	0,89	0,05	0	0	0	0,04	0,02	0	0,94	0,044
1,25	20832	0	0,04	0	0,08	0,88	0	0	0	0	0,06	0,07	0,87	0	0,048
1,25	21923	0	0,11	0	0,01	0,88	0	0	0	0	0,01	0	0,99	0	0,048
1,25	15219	0,01	0,05	0	0	0,92	0	0,02	0	0	0,92	0,02	0,05	0,01	0,044
1,25	57986	0	0,01	0,01	0,16	0,82	0	0	0	0	0,71	0	0,29	0	0,046
1,25	77260	0	0,1	0,04	0	0,01	0,85	0	0	0	0,09	0	0,91	0	0,042
1,25	1806	0	0	0,04	0	0,58	0,32	0	0	0,06	0,38	0,02	0,02	0,58	0,018
1,25	12411	0,03	0	0	0	0	0,83	0,05	0,09	0	0,07	0	0,84	0,09	0,048
1,25	9010	0	0,02	0,04	0,01	0,24	0	0	0	0,69	0	0,28	0,01	0,71	0,026
1,25	26563	0	0	0,04	0,15	0,81	0	0	0	0	0,81	0,19	0	0	0,048
1,25	82210	0,04	0,03	0,02	0	0,02	0	0,01	0,6	0,28	0,05	0,45	0,18	0,32	0,044
1,25	17493	0,05	0,04	0	0	0,91	0	0	0	0	0,05	0,9	0,01	0,04	0,048
1,25	11698	0	0	0,04	0	0	0,79	0,04	0,13	0	0	0,02	0,02	0,96	0,042
1,25	11936	0	0,24	0	0	0,76	0	0	0	0	0	0,76	0,24	0	0,048
1,25	28563	0	0,01	0,04	0	0,48	0	0	0,47	0	0	0,05	0,48	0,47	0,02
1,25	42538	0	0,08	0	0	0	0,91	0	0	0,01	0,99	0	0	0,01	0,044
1,25	39411	0,01	0,05	0	0,05	0,86	0	0	0,03	0	0,08	0,02	0,88	0,02	0,046
1,25	30688	0,01	0	0,04	0	0	0	0	0,21	0,74	0,2	0,04	0,17	0,59	0,016
1,25	1486	0	0,11	0,02	0	0,81	0,06	0	0	0	0	0,02	0,97	0,01	0,04
1,25	3317	0	0,01	0,04	0	0	0,51	0	0,44	0	0,01	0	0,95	0,04	0,034
1,25	35812	0,01	0	0,03	0	0,88	0,02	0,06	0	0	0	0,91	0,07	0,02	0,04
1,25	11044	0	0,08	0,04	0	0	0	0,03	0,85	0	0	0,04	0,11	0,85	0,044
1,25	8476	0	0	0,04	0	0,3	0,62	0,04	0	0	0,65	0	0,34	0,01	0,038
1,25	64522	0	0	0,04	0,01	0,81	0	0,01	0	0,13	0,01	0,85	0,14	0	0,032
1,25	42157	0	0,94	0,03	0	0,03	0	0	0	0	0,03	0	0,03	0,94	0,048
1,25	100000	0,09	0	0,9	0	0	0	0	0,01	0	0	0,63	0,09	0,28	0,436
1,25	42718	0	0	0,04	0	0	0,96	0	0	0	0,01	0,04	0,95	0	0,024
1,25	44384	0,03	0	0,04	0	0,88	0	0	0	0,05	0	0,04	0,96	0	0,036
1,25	3955	0	0,02	0,01	0,08	0,87	0	0	0,02	0	0,01	0,01	0,91	0,07	0,038

1,25	18623	0	0	0	0,09	0,8	0	0	0,1	0,01	0,02	0,01	0,35	0,62	0,048
1,25	19469	0	0,09	0	0	0,87	0	0,04	0	0	0,01	0	0,96	0,03	0,042
1,25	53354	0	0	0,04	0	0	0,92	0	0,04	0	0,95	0	0,01	0,04	0,022
1,25	20362	0	0	0,04	0	0,87	0,08	0,01	0	0	0	0,12	0,88	0	0,028
1,25	15531	0	0	0,04	0	0,89	0	0,04	0,03	0	0	0,04	0	0,96	0,046
1,25	18268	0	0,09	0,04	0	0,58	0,06	0	0,01	0,22	0,06	0,08	0,77	0,09	0,046
1,25	34116	0	0	0	0,06	0,94	0	0	0	0	0,05	0,95	0	0	0,046
1,25	20942	0	0,15	0,02	0,03	0,66	0,09	0,02	0,03	0	0,14	0,07	0,01	0,78	0,048
1,25	18230	0	0,49	0	0	0	0	0,03	0,48	0	0	0,96	0	0,04	0,048
1,25	76148	0	0	0,04	0,01	0,42	0,31	0	0,03	0,19	0,04	0,04	0,25	0,67	0,024
1,25	23820	0,03	0,01	0	0	0,94	0	0,02	0	0	0,05	0,94	0,01	0	0,042
1,25	15405	0	0,03	0	0,05	0,71	0	0,01	0,2	0	0,01	0	0,06	0,93	0,044
1,25	35785	0	0,29	0,01	0	0,7	0	0	0	0	0	0,01	0,29	0,7	0,048
1,25	42560	0	0,03	0,01	0,07	0,54	0	0	0,35	0	0,01	0,1	0,35	0,54	0,048
1,25	17752	0	0	0	0	0	0	0,11	0,89	0	0	0	0,89	0,11	0,048
1,25	18803	0	0	0	0,07	0,83	0	0	0,1	0	0,07	0	0,14	0,79	0,044
1,25	55497	0,04	0,7	0,02	0	0,02	0,03	0	0,19	0	0	0,03	0,02	0,95	0,048
1,25	8901	0	0,22	0,02	0,01	0,75	0	0	0	0	0,12	0,02	0,11	0,75	0,042
1,25	28261	0	0	0	0,08	0,78	0	0	0,14	0	0,07	0,78	0,15	0	0,046
1,25	28493	0,03	0,04	0	0	0,91	0,02	0	0	0	0,97	0	0,03	0	0,048
1,25	26563	0,03	0	0	0,06	0,66	0	0,07	0	0,18	0,66	0,26	0,06	0,02	0,046
1,25	7403	0	0	0,04	0	0	0	0,13	0,83	0	0,82	0,17	0	0,01	0,044
1,25	12300	0	0,06	0,04	0	0,89	0	0,01	0	0	0,09	0	0	0,91	0,014
1,25	7304	0,04	0	0,04	0	0,71	0	0	0,21	0	0,05	0,04	0	0,91	0,046
1,25	37618	0	0,02	0	0,12	0,78	0,05	0	0	0,03	0,85	0	0,1	0,05	0,046
1,25	13679	0	0	0,04	0	0	0,01	0,02	0,93	0	0,02	0,04	0,01	0,93	0,016
1,25	34341	0,03	0	0	0,04	0,93	0	0	0	0	0	0,03	0,97	0	0,048
1,25	16993	0	0	0,04	0,01	0,66	0	0	0,19	0,1	0,64	0,04	0,21	0,11	0,034
1,25	8114	0,01	0,06	0,04	0	0,89	0	0	0	0	0	0,06	0,92	0,02	0,044
1,25	35175	0,02	0,06	0,04	0	0,73	0	0	0,15	0	0,02	0,03	0,19	0,76	0,03
1,25	18288	0	0	0,01	0,05	0,67	0,02	0	0,25	0	0	0,28	0	0,72	0,044
1,25	11366	0	0,29	0	0,01	0,7	0	0	0	0	0	0	0,3	0,7	0,048
1,25	50443	0	0,28	0	0	0,71	0	0	0,01	0	0,03	0	0,25	0,72	0,048

1,25	36392	0	0,02	0,02	0,07	0,89	0	0	0	0	0,02	0,07	0,34	0,57	0,048
1,25	7375	0,01	0	0,04	0	0	0,86	0	0,02	0,07	0,02	0,04	0,87	0,07	0,038
1,25	2618	0	0	0,02	0,08	0,73	0	0	0,17	0	0,07	0,02	0,18	0,73	0,046
1,25	4810	0	0,09	0	0	0,8	0,11	0	0	0	0,62	0,12	0,16	0,1	0,036
1,25	9532	0	0,09	0,01	0,06	0	0,84	0	0	0	0,09	0,07	0,32	0,52	0,048
1,5	74767	0	0,07	0,04	0	0,87	0	0,02	0	0	0,09	0,6	0,31	0	0,042
1,5	7790	0	0	0,04	0	0,96	0	0	0	0	0	0,08	0,92	0	0,018
1,5	2436	0	0	0,04	0	0,79	0	0	0,09	0,08	0,09	0,04	0,08	0,79	0,014
1,5	15163	0	0	0	0,08	0,76	0,02	0,08	0,06	0	0,76	0,02	0,08	0,14	0,046
1,5	30082	0,03	0	0,04	0	0	0	0	0	0,93	0	0,04	0,02	0,94	0,044
1,5	16006	0,02	0,01	0,03	0,04	0,88	0	0,01	0,01	0	0,03	0,07	0,9	0	0,046
1,5	23978	0	0,07	0	0,04	0,89	0	0	0	0	0,89	0	0,07	0,04	0,04
1,5	12214	0	0	0	0,07	0,86	0	0	0,07	0	0	0,86	0,07	0,07	0,046
1,5	9299	0	0	0,04	0	0,76	0,2	0	0	0	0,41	0,04	0,35	0,2	0,02
1,5	77533	0	0,12	0	0	0,87	0	0	0,01	0	0	0	1	0	0,042
1,5	40853	0,05	0	0	0	0,93	0	0	0,02	0	0,05	0	0,93	0,02	0,044
1,5	49388	0,04	0	0	0	0	0,62	0,03	0,31	0	0,01	0,65	0	0,34	0,044
1,5	12204	0	0,14	0,04	0	0,82	0	0	0	0	0,18	0,82	0	0	0,046
1,5	35387	0,04	0	0,02	0	0	0	0	0,94	0	0,94	0	0,04	0,02	0,044
1,5	13074	0	0	0	0,09	0,77	0	0,01	0,13	0	0	0,14	0,78	0,08	0,044
1,5	3565	0	0	0,04	0	0	0	0	0,96	0	0	0,03	0,96	0,01	0,022
1,5	22364	0	0	0,04	0,03	0,07	0,38	0	0,46	0,02	0,36	0,16	0,38	0,1	0,032
1,5	41795	0	0,91	0,02	0	0,01	0	0,01	0	0,05	0,04	0	0,03	0,93	0,044
1,5	99910	0	0	0,01	0,12	0,87	0	0	0	0	0,11	0	0,88	0,01	0,048
1,5	35985	0	0	0,04	0,02	0,94	0	0	0	0	0	0	0,06	0,94	0,022
1,5	57236	0	0,12	0	0	0,88	0	0	0	0	0,39	0,61	0	0	0,046
1,5	36919	0	0	0,04	0	0,27	0	0	0,69	0	0	0,04	0,27	0,69	0,016
1,5	25752	0	0	0,04	0	0,62	0,32	0	0,01	0,01	0,01	0,64	0	0,35	0,01
1,5	5160	0	0,03	0,04	0	0,01	0	0	0,92	0	0	0,07	0	0,93	0
1,5	22805	0	0	0,04	0	0,87	0,01	0,05	0,03	0	0	0,94	0,06	0	0,042
1,5	4522	0,01	0	0	0,08	0	0,33	0	0,57	0,01	0	0,01	0,66	0,33	0,04
1,5	12331	0	0,39	0	0	0,23	0,29	0	0	0,09	0	0,38	0,1	0,52	0,048
1,5	13178	0	0,04	0	0,15	0,8	0	0	0	0,01	0,81	0,15	0,04	0	0,048

1,5	15938	0,02	0	0,04	0	0,94	0	0	0	0	0,01	0,01	0,02	0,96	0,024
1,5	31728	0	0	0,04	0,05	0,2	0,52	0	0,02	0,17	0,01	0,05	0,42	0,52	0,042
1,5	18921	0	0	0	0,06	0,83	0,06	0	0,05	0	0,05	0,83	0	0,12	0,046
1,5	13906	0	0	0	0,11	0,7	0	0	0,19	0	0,19	0,11	0,7	0	0,048
1,5	68548	0	0,13	0	0	0,87	0	0	0	0	0,02	0,11	0,87	0	0,048
1,5	9660	0,01	0,07	0,04	0,01	0,86	0	0	0,01	0	0,08	0,04	0	0,88	0,022
1,5	27626	0,04	0	0,02	0,01	0	0	0	0,93	0	0	0,06	0	0,94	0,044
1,5	39694	0	0	0,04	0,14	0,8	0	0	0,02	0	0,78	0	0,18	0,04	0,046
1,5	3015	0	0	0,04	0	0,36	0,05	0,02	0,53	0	0,53	0,08	0,37	0,02	0,04
1,5	24596	0,03	0	0,04	0	0	0	0	0,92	0,01	0	0,22	0,03	0,75	0,036
1,5	20102	0	0,05	0,04	0	0	0,02	0	0,3	0,59	0,32	0,14	0,01	0,53	0,046
1,5	36367	0,02	0	0,04	0	0,92	0	0	0,02	0	0	0,04	0,94	0,02	0,042
1,5	80893	0	0,1	0	0,01	0	0,86	0,01	0,02	0	0,05	0,01	0,92	0,02	0,046
1,5	30100	0	0,23	0,04	0	0	0,73	0	0	0	0,01	0,26	0	0,73	0,04
1,5	1940	0	0	0,04	0	0,09	0,81	0	0,03	0,03	0,82	0,04	0,03	0,11	0,036
1,5	9769	0	0,18	0,01	0,02	0,78	0	0	0	0,01	0	0,83	0,12	0,05	0,042
1,5	22803	0	0,99	0	0	0	0	0	0,01	0	0,01	0,99	0	0	0,032
1,5	33517	0	0	0,04	0,03	0,93	0	0	0	0	0,97	0	0,03	0	0,044
1,5	1749	0	0	0,04	0	0,77	0,19	0	0	0	0,96	0,04	0	0	0,042
1,5	9595	0	0	0,04	0	0	0	0	0,96	0	0	0	0,96	0,04	0,018
1,5	15074	0	0	0,04	0,03	0	0,62	0	0	0,31	0,62	0,04	0,03	0,31	0,036
1,5	13037	0	0,1	0	0,06	0,84	0	0	0	0	0,06	0	0	0,94	0,044
1,5	6641	0,05	0	0	0	0,38	0	0	0,42	0,15	0,2	0,38	0,42	0	0,046
1,5	3816	0	0,03	0,04	0	0,84	0,09	0	0	0	0,96	0	0,04	0	0,044
1,5	34455	0	0	0	0,23	0,7	0	0,01	0,06	0	0,86	0,08	0	0,06	0,046
1,5	60418	0	0	0	0,28	0,72	0	0	0	0	1	0	0	0	0,042
1,5	100000	0	0,19	0,38	0	0,13	0	0	0,03	0,27	0	0,38	0,3	0,32	0,2
1,5	17894	0,03	0,93	0,03	0	0	0	0	0,01	0	0,03	0,03	0	0,94	0,048
1,5	15365	0	0	0,04	0	0,01	0,95	0	0	0	0	0,04	0	0,96	0,004
1,5	2458	0,04	0	0	0	0,64	0	0,03	0	0,29	0	0,04	0,8	0,16	0,04
1,5	5100	0	0	0,04	0	0	0	0	0,96	0	0,96	0,04	0	0	0,038
1,5	17063	0	0	0	0,11	0,76	0	0	0,13	0	0,05	0	0,95	0	0,046
1,5	21051	0	0	0	0,09	0,9	0,01	0	0	0	0,09	0	0,31	0,6	0,046

1,5	24912	0	0,06	0	0,05	0,89	0	0	0	0	0,85	0,15	0	0	0,046
1,5	2598	0	0,01	0,04	0	0,78	0	0	0,17	0	0,16	0,83	0,01	0	0,024
1,5	18489	0,01	0,07	0,01	0	0,13	0,17	0	0,53	0,08	0,73	0,02	0,2	0,05	0,048
1,5	7167	0,02	0	0	0	0,94	0	0,04	0	0	0,03	0,01	0,02	0,94	0,046
1,5	9188	0	0,04	0,03	0	0,52	0,29	0,04	0,08	0	0,48	0,08	0,31	0,13	0,046
1,5	31849	0	0,26	0	0	0	0,07	0	0,52	0,15	0,15	0,26	0	0,59	0,048
1,5	6795	0,06	0	0	0	0	0,52	0,01	0,36	0,05	0,3	0,1	0,6	0	0,048
1,5	46170	0	0,01	0	0,19	0,64	0,14	0	0,01	0,01	0,66	0,19	0,14	0,01	0,046
1,5	3619	0	0,02	0,04	0,03	0,27	0	0	0,64	0	0,06	0,01	0,02	0,91	0,03
1,5	18282	0	0,07	0,04	0,03	0,85	0	0	0,01	0	0,1	0,88	0,01	0,01	0,044
1,5	56394	0	0	0	0,15	0,48	0,34	0	0	0,03	0,34	0	0,66	0	0,044
1,5	66872	0	0	0,04	0	0,02	0,94	0	0	0	0,06	0,06	0,88	0	0,022
1,5	69533	0	0	0	0,09	0,37	0,03	0	0,5	0,01	0,17	0	0,34	0,49	0,048
1,5	5823	0	0,09	0	0	0,82	0,09	0	0	0	0,99	0	0	0,01	0,046
1,5	4532	0	0	0,04	0,04	0,78	0	0	0,14	0	0	1	0	0	0,038
1,5	27034	0	0,13	0	0,03	0,83	0,01	0	0	0	0,13	0,83	0,04	0	0,046
1,5	23772	0,01	0	0	0,22	0,77	0	0	0	0	0,9	0,01	0,08	0,01	0,048
1,5	71447	0,01	0	0	0,09	0,88	0	0	0,01	0,01	0,09	0	0,89	0,02	0,048
1,5	14776	0	0	0,04	0,1	0,79	0	0	0,06	0,01	0,69	0,26	0,04	0,01	0,042
1,5	41697	0	0	0,04	0	0	0	0	0,94	0,02	0	0,04	0,02	0,94	0,004
1,5	22129	0	0,05	0	0,06	0	0	0,01	0,88	0	0,01	0,88	0,05	0,06	0,04
1,5	13287	0,05	0	0	0	0,94	0	0,01	0	0	0	0	1	0	0,036
1,5	51866	0,01	0,01	0,04	0	0,75	0	0	0,19	0	0,05	0,62	0,01	0,32	0,016
1,5	76045	0	0	0,04	0	0	0	0	0,96	0	0	0,04	0,96	0	0,024
1,5	2693	0	0	0,04	0	0,95	0	0	0	0,01	0	0,04	0,95	0,01	0,026
1,5	16375	0	0,13	0	0,01	0	0,46	0	0,4	0	0	0,83	0,13	0,04	0,048
1,5	10521	0	0	0,04	0	0,48	0,02	0	0,32	0,14	0	0,53	0,47	0	0,022
1,5	13735	0	0	0,04	0	0,57	0,19	0	0	0,2	0	0,01	0,76	0,23	0,016
1,5	100000	0	0	0,96	0	0,04	0	0	0	0	0	0,96	0,04	0	0,53
1,5	80368	0	0,09	0	0,04	0,83	0	0,02	0	0,02	0,06	0	0	0,94	0,046
1,5	10019	0	0	0	0,04	0,96	0	0	0	0	0	0	0,04	0,96	0,038
1,5	58706	0,01	0	0	0,08	0,87	0,02	0	0,01	0,01	0,09	0,82	0,08	0,01	0,046
1,5	26672	0	0,03	0,04	0	0,03	0,89	0	0	0,01	0,93	0,03	0	0,04	0,048

1,5	21652	0,06	0	0	0	0,01	0,9	0	0,01	0,02	0,06	0	0,23	0,71	0,048
1,5	70681	0,02	0,14	0	0	0,83	0	0	0,01	0	0,14	0,86	0	0	0,036
1,5	37930	0	0	0,04	0	0,93	0	0,01	0,02	0	0,02	0,05	0,93	0	0,036
1,5	10711	0,02	0	0,03	0,05	0	0,41	0	0	0,49	0,03	0,51	0	0,46	0,044
1,5	19901	0	0	0,03	0,06	0,91	0	0	0	0	0	0	0,06	0,94	0,046
1,5	3792	0	0	0,04	0	0,07	0,89	0	0	0	0,08	0,04	0,85	0,03	0,03
1,75	24713	0	0	0	0,21	0,79	0	0	0	0	0,75	0,14	0,11	0	0,048
1,75	6338	0,02	0,08	0	0	0,05	0	0	0,85	0	0,05	0,1	0,85	0	0,048
1,75	7682	0,02	0	0,04	0	0,94	0	0	0	0	0,91	0,09	0	0	0,046
1,75	21277	0,02	0	0,04	0	0,91	0,01	0	0	0,02	0,91	0,04	0,01	0,04	0,046
1,75	7267	0	0	0,04	0	0,01	0	0,01	0,94	0	0,03	0,32	0,62	0,03	0,03
1,75	51639	0,02	0	0,04	0	0,93	0,01	0	0	0	0,95	0,05	0	0	0,044
1,75	22307	0	0,89	0	0,03	0,07	0	0	0	0,01	0,01	0,01	0,02	0,96	0,042
1,75	100000	0	0	0,99	0	0	0,01	0	0	0	0	0,98	0,02	0	0,604
1,75	3401	0,05	0,03	0,04	0	0,8	0,08	0	0	0	0,13	0,83	0	0,04	0,048
1,75	12227	0	0,01	0,04	0	0,9	0	0	0,05	0	0	0,94	0,05	0,01	0,008
1,75	7400	0	0,18	0	0	0	0	0	0,82	0	0,18	0	0,47	0,35	0,044
1,75	4367	0,01	0	0,01	0,05	0,92	0	0	0,01	0	0,07	0,01	0	0,92	0,044
1,75	3523	0,02	0	0,04	0,01	0	0,92	0	0,01	0	0	0,06	0,93	0,01	0,044
1,75	13027	0	0	0,04	0,01	0	0,95	0	0	0	0,01	0	0,04	0,95	0,008
1,75	100000	0,02	0	0,78	0	0	0,2	0	0	0	0,2	0,68	0,02	0,1	0,408
1,75	81868	0,06	0	0	0	0	0,93	0	0	0,01	0,01	0	0,99	0	0,048
1,75	54248	0,03	0	0	0,04	0,48	0,05	0	0,39	0,01	0,48	0,4	0,03	0,09	0,044
1,75	23651	0,01	0,1	0	0,02	0,44	0	0,01	0,42	0	0,1	0,44	0,44	0,02	0,032
1,75	61496	0	0,11	0	0	0,89	0	0	0	0	0	0,08	0,92	0	0,044
1,75	12271	0	0	0	0,02	0	0	0,05	0,93	0	0,03	0,92	0,02	0,03	0,046
1,75	11571	0,01	0	0	0,04	0,94	0	0	0,01	0	0,06	0,89	0,05	0	0,04
1,75	6485	0	0,3	0	0	0,66	0,04	0	0	0	0	0,04	0,3	0,66	0,046
1,75	13179	0	0	0,04	0	0,02	0,93	0	0	0,01	0,93	0,05	0	0,02	0,04
1,75	26783	0,05	0	0	0	0	0,89	0	0,06	0	0,05	0,06	0,89	0	0,042
1,75	17712	0,01	0,03	0,01	0,06	0,87	0,02	0	0	0	0	0,1	0,85	0,05	0,042
1,75	47858	0	0	0	0,24	0,62	0,14	0	0	0	0,85	0	0,14	0,01	0,046
1,75	14606	0	0,07	0	0	0,86	0	0,01	0,06	0	0,93	0	0,07	0	0,048

1,75	17163	0,01	0,28	0	0	0,7	0	0	0	0,01	0,01	0	0,29	0,7	0,048
1,75	16000	0	0	0,04	0	0,95	0	0	0	0,01	0	0,04	0,95	0,01	0,018
1,75	28497	0	0	0	0,01	0,89	0	0,08	0	0,02	0	0,12	0,78	0,1	0,046
1,75	35556	0	0	0	0,07	0,92	0	0	0,01	0	0	0,93	0,07	0	0,044
1,75	12273	0	0,02	0,04	0	0	0,32	0	0,33	0,29	0,29	0,06	0,65	0	0,042
1,75	20160	0	0,01	0,04	0	0,74	0	0	0,21	0	0,74	0,03	0,23	0	0,048
1,75	64485	0,01	0	0,04	0,02	0,9	0	0,01	0	0,02	0,91	0,05	0,03	0,01	0,036
1,75	52700	0,05	0	0,01	0	0,85	0,09	0	0	0	0,14	0,01	0	0,85	0,046
1,75	7906	0	0,21	0	0	0,5	0,2	0	0,09	0	0,21	0,25	0,04	0,5	0,048
1,75	33075	0	0,02	0	0,18	0,8	0	0	0	0	0,8	0,04	0,16	0	0,048
1,75	79757	0,01	0,08	0	0,02	0,76	0,01	0	0,12	0	0,68	0,3	0	0,02	0,048
1,75	51829	0	0	0	0,11	0,39	0,46	0,04	0	0	0,41	0,35	0,04	0,2	0,044
1,75	48277	0,02	0,83	0,01	0	0,14	0	0	0	0	0	0,01	0,02	0,97	0,044
1,75	16179	0	0	0	0,11	0,84	0,04	0,01	0	0	0,11	0	0,85	0,04	0,04
1,75	21343	0,01	0,08	0	0	0,11	0,23	0	0,54	0,03	0,29	0,12	0,36	0,23	0,044
1,75	31938	0	0,29	0	0,01	0,69	0	0	0	0,01	0	0,01	0,29	0,7	0,046
1,75	27267	0	0	0,04	0,01	0,01	0	0	0,94	0	0,02	0,03	0,01	0,94	0,02
1,75	2665	0	0,1	0,04	0	0,03	0,71	0	0	0,12	0,01	0,15	0,71	0,13	0,04
1,75	24132	0	0,12	0	0	0,79	0,07	0,02	0	0	0,02	0,19	0,79	0	0,048
1,75	34802	0	0,01	0,04	0,01	0	0,87	0	0,07	0	0	0,11	0,88	0,01	0,028
1,75	27933	0	0	0	0,16	0,79	0	0	0,05	0	0,81	0,03	0	0,16	0,036
1,75	33582	0	0,01	0	0,06	0,85	0	0	0,08	0	0	0,08	0,07	0,85	0,04
1,75	38773	0	0	0,01	0,05	0,88	0	0	0	0,06	0	0,02	0,07	0,91	0,044
1,75	13984	0	0	0,04	0	0,76	0	0,01	0,19	0	0,2	0,77	0,03	0	0,032
1,75	13844	0	0	0,04	0	0,91	0	0	0	0,05	0	0,09	0	0,91	0,002
1,75	27617	0	0,01	0,04	0	0,51	0	0	0,44	0	0	0,51	0,22	0,27	0,01
1,75	27340	0	0,18	0	0,01	0,72	0	0	0,08	0,01	0,09	0,72	0,13	0,06	0,046
1,75	26294	0	0,12	0	0	0	0,82	0	0	0,06	0,12	0,06	0,82	0	0,044
1,75	25496	0,01	0	0,04	0	0,95	0	0	0	0	0,59	0,04	0	0,37	0,028
1,75	23503	0	0	0,04	0	0,96	0	0	0	0	0,04	0	0,82	0,14	0,02
1,75	95256	0	0	0	0,23	0,76	0	0	0	0,01	0,76	0	0,24	0	0,042
1,75	19992	0	0,06	0	0,06	0,68	0,13	0	0,07	0	0,19	0,01	0,18	0,62	0,048
1,75	48443	0,01	0	0,04	0	0,26	0	0,02	0,66	0,01	0,01	0,05	0,92	0,02	0,042

1,75	40481	0,01	0,09	0	0,03	0,86	0	0	0	0,01	0,17	0	0,78	0,05	0,044
1,75	52660	0	0,16	0	0	0,79	0,03	0,02	0	0	0	0,82	0,18	0	0,048
1,75	15627	0	0,15	0	0	0	0	0	0,85	0	0,01	0	0,7	0,29	0,046
1,75	100000	0	0	1	0	0	0	0	0	0	0	1	0	0	0,62
1,75	50990	0	0,86	0,04	0	0	0	0	0	0,1	0,03	0,08	0,01	0,88	0,044
1,75	11612	0	0	0,04	0	0	0,94	0,02	0	0	0,05	0,04	0,91	0	0,046
1,75	74552	0,03	0	0,03	0,01	0	0	0,01	0,92	0	0,01	0,03	0,03	0,93	0,042
1,75	21789	0,05	0	0,03	0	0	0	0	0,9	0,02	0	0,07	0,91	0,02	0,046
1,75	5868	0	0,06	0	0,03	0,89	0,02	0	0	0	0,89	0	0,11	0	0,048
1,75	20537	0,04	0,01	0,04	0	0,91	0	0	0	0	0,05	0,71	0,01	0,23	0,044
1,75	47565	0	0,04	0,01	0,13	0,72	0,1	0	0	0	0,86	0,01	0,13	0	0,046
1,75	15176	0	0,03	0,04	0,01	0	0,01	0	0,91	0	0,01	0	0,95	0,04	0,048
1,75	14494	0,03	0,17	0,01	0	0	0,02	0	0,77	0	0,03	0,01	0,17	0,79	0,042
1,75	24903	0	0	0	0,07	0	0	0,04	0,89	0	0,04	0	0,89	0,07	0,04
1,75	3297	0	0	0,04	0,03	0,7	0,22	0	0,01	0	0,23	0,04	0,73	0	0,034
1,75	7653	0	0,01	0,04	0	0,94	0	0,01	0	0	0,93	0,05	0,02	0	0,048
1,75	51724	0	0	0,04	0,06	0,69	0,01	0	0,02	0,18	0	0,1	0,69	0,21	0,044
1,75	11963	0	0	0	0,07	0,78	0	0	0	0,15	0,01	0	0,21	0,78	0,048
1,75	22957	0	0,12	0	0,01	0,81	0,04	0	0	0,02	0,16	0	0,83	0,01	0,046
1,75	26385	0	0	0,04	0	0	0	0	0,96	0	0	0,04	0,96	0	0,02
1,75	21888	0	0,02	0	0,13	0,59	0,24	0	0,02	0	0,74	0,02	0,24	0	0,048
1,75	30404	0,03	0	0,04	0	0,09	0,07	0,01	0,75	0,01	0,02	0,48	0,37	0,13	0,048
1,75	17723	0	0,12	0	0,01	0,87	0	0	0	0	0	0,01	0,99	0	0,048
1,75	17562	0,03	0	0,04	0	0	0,2	0,01	0,25	0,47	0	0,18	0,11	0,71	0,042
1,75	28242	0	0,51	0,04	0	0,44	0	0,01	0	0	0	0,03	0,02	0,95	0,032
1,75	17512	0	0,01	0,01	0,15	0,81	0,02	0	0	0	0,83	0,01	0,01	0,15	0,048
1,75	18510	0,02	0,15	0	0	0,83	0	0	0	0	0,19	0,79	0	0,02	0,048
1,75	21525	0	0	0,04	0,01	0	0	0	0,06	0,89	0,18	0,07	0,04	0,71	0,024
1,75	10297	0	0,24	0,04	0	0,72	0	0	0	0	0,18	0,04	0,06	0,72	0,042
1,75	36978	0	0	0	0,11	0,22	0	0	0,67	0	0	0	0,78	0,22	0,046
1,75	73590	0	0,03	0,02	0,1	0,85	0	0	0	0	0,82	0	0,18	0	0,048
1,75	22465	0	0,26	0	0,01	0	0,02	0	0,7	0,01	0,02	0,01	0,24	0,73	0,048
1,75	10842	0,03	0,01	0,04	0	0,91	0	0	0	0,01	0,02	0,04	0,94	0	0,046

1,75	39271	0	0,9	0	0,01	0	0	0,02	0	0,07	0	0,02	0,01	0,97	0,048
1,75	10986	0,01	0	0,04	0,03	0,92	0	0	0	0	0	0,07	0,93	0	0,04
1,75	14100	0	0	0,04	0	0	0,09	0	0,02	0,85	0,1	0,05	0	0,85	0,028
1,75	29294	0	0,08	0	0,04	0,87	0	0	0	0,01	0,05	0	0,87	0,08	0,042
1,75	68340	0	0	0,01	0,09	0,88	0	0,02	0	0	0	0,01	0,9	0,09	0,048
1,75	83499	0	0	0	0,22	0,78	0	0	0	0	0,78	0,22	0	0	0,044
1,75	86477	0	0	0,04	0,02	0	0,94	0	0	0	0,02	0,04	0,94	0	0,034
2	17835	0	0	0,04	0	0,01	0	0	0,95	0	0,28	0,67	0	0,05	0,034
2	35911	0	0	0	0,16	0,84	0	0	0	0	0,74	0,1	0,16	0	0,044
2	79820	0,06	0,01	0	0	0,85	0,08	0	0	0	0,14	0,11	0	0,75	0,04
2	48417	0,01	0	0	0,16	0,63	0,2	0	0	0	0,75	0,01	0,2	0,04	0,042
2	7248	0	0,06	0,04	0	0,02	0,02	0	0,72	0,14	0,1	0	0,74	0,16	0,048
2	4163	0	0,02	0	0,16	0,79	0	0	0,03	0	0,72	0,21	0,01	0,06	0,044
2	59290	0,03	0	0,04	0	0,1	0	0	0,49	0,34	0,62	0,04	0	0,34	0,046
2	33904	0	0	0,04	0	0	0	0	0	0,96	0,01	0,04	0	0,95	0,022
2	6688	0	0	0,04	0	0	0,96	0	0	0	0	0,04	0,96	0	0,022
2	2310	0	0,04	0,04	0	0,5	0,28	0	0,12	0,02	0,54	0,06	0,4	0	0,046
2	12051	0	0,01	0,04	0,01	0	0,94	0	0	0	0,02	0	0	0,98	0,016
2	26980	0	0	0	0,14	0,77	0,08	0	0,01	0	0,16	0	0,77	0,07	0,046
2	91367	0	0,22	0	0	0,65	0	0	0	0,13	0,22	0,01	0,12	0,65	0,034
2	4330	0	0	0,01	0,13	0,86	0	0	0	0	0,12	0,02	0,86	0	0,048
2	5923	0,02	0	0,04	0	0,02	0,91	0	0	0,01	0,04	0,94	0,02	0	0,034
2	13287	0	0	0,04	0	0	0,37	0	0,59	0	0,37	0,04	0,5	0,09	0,028
2	3316	0	0,01	0,04	0	0	0,04	0	0,15	0,76	0,79	0,08	0	0,13	0,042
2	34918	0,04	0	0,03	0	0,78	0,14	0	0	0,01	0,16	0,02	0,82	0	0,048
2	6319	0	0,03	0,04	0	0	0,58	0	0,02	0,33	0,04	0,05	0,58	0,33	0,036
2	18767	0,05	0,02	0	0	0,78	0	0	0,15	0	0,21	0	0,78	0,01	0,048
2	53925	0	0	0,04	0	0,52	0,33	0	0,11	0	0,52	0,13	0,02	0,33	0,018
2	12376	0,01	0,08	0	0,02	0,02	0	0,02	0,85	0	0,02	0,7	0,18	0,1	0,04
2	10357	0	0,12	0	0	0,64	0	0	0,23	0,01	0,24	0,02	0,62	0,12	0,048
2	1499	0	0	0,04	0	0,43	0,13	0	0,4	0	0,17	0,6	0	0,23	0,018
2	27215	0	0,17	0,03	0,01	0,79	0	0	0	0	0	0,82	0,17	0,01	0,04
2	2974	0	0	0	0,19	0,73	0,04	0,01	0,03	0	0,73	0,04	0,22	0,01	0,048

2	11622	0	0,02	0,04	0	0,64	0	0	0,3	0	0,04	0,02	0,94	0	0,044
2	9535	0	0	0,04	0	0,94	0,01	0	0,01	0	0	0,03	0,96	0,01	0,026
2	28953	0	0	0,04	0,02	0,02	0	0,01	0,91	0	0,03	0,04	0,02	0,91	0,02
2	20889	0	0	0	0,13	0,87	0	0	0	0	0,13	0	0,87	0	0,048
2	26400	0	0	0,04	0	0,94	0,01	0	0	0,01	0	0,02	0,94	0,04	0,03
2	21367	0	0	0,04	0	0	0	0,03	0,02	0,91	0,03	0,05	0	0,92	0,022
2	33872	0	0	0,01	0	0,92	0	0,06	0	0,01	0	0	0,02	0,98	0,046
2	62201	0,05	0	0	0	0	0,82	0	0,1	0,03	0,04	0,02	0,92	0,02	0,044
2	5281	0	0,3	0	0	0	0	0	0,7	0	0	0,03	0,27	0,7	0,048
2	23330	0,01	0	0,04	0	0,44	0	0,03	0,48	0	0,01	0,52	0,41	0,06	0,018
2	45220	0	0	0	0,19	0,81	0	0	0	0	0,79	0	0,19	0,02	0,04
2	30361	0	0,17	0	0,04	0,73	0,06	0	0	0	0,16	0,05	0,06	0,73	0,04
2	22984	0	0	0,04	0	0,64	0	0	0	0,32	0	0,36	0,21	0,43	0,008
2	100000	0	0	0,95	0	0	0	0,05	0	0	0	1	0	0	0,66
2	10099	0,01	0,03	0	0,06	0	0	0	0,9	0	0,04	0,93	0	0,03	0,042
2	15014	0,05	0,01	0,03	0	0	0	0	0,91	0	0,03	0	0,01	0,96	0,048
2	57619	0,01	0	0,02	0	0,85	0	0,12	0	0	0,85	0,14	0,01	0	0,042
2	35901	0,03	0	0,04	0	0,77	0	0	0	0,16	0,76	0,08	0,16	0	0,044
2	57090	0	0	0,04	0	0,96	0	0	0	0	0,96	0,04	0	0	0,036
2	1347	0,01	0,02	0,04	0	0,9	0	0,02	0,01	0	0,92	0,05	0,02	0,01	0,046
2	90572	0,02	0,01	0	0,04	0,68	0,05	0	0,2	0	0,07	0	0,25	0,68	0,046
2	73964	0	0,17	0,01	0	0	0,23	0	0,59	0	0,01	0,8	0,19	0	0,046
2	44682	0	0	0	0,11	0,89	0	0	0	0	0,39	0,03	0,11	0,47	0,048
2	13096	0,04	0,01	0,02	0,01	0,4	0,22	0	0	0,3	0	0,64	0,3	0,06	0,048
2	17672	0	0,15	0,01	0,03	0	0	0	0,81	0	0,15	0,01	0	0,84	0,044
2	36279	0	0,26	0	0	0,74	0	0	0	0	0,26	0	0	0,74	0,048
2	38706	0	0,05	0,04	0	0,64	0	0,02	0	0,25	0,23	0,04	0	0,73	0,03
2	71484	0,01	0	0,04	0,02	0	0,16	0	0,77	0	0	0,04	0,93	0,03	0,04
2	9395	0	0	0,01	0,05	0,92	0	0	0,01	0,01	0	0,98	0	0,02	0,04
2	14272	0,02	0	0,01	0,03	0,93	0	0	0	0,01	0,01	0,02	0,04	0,93	0,048
2	22925	0	0	0,02	0,08	0,9	0	0	0	0	0,51	0,07	0	0,42	0,038
2	9772	0	0,2	0,01	0	0,76	0	0	0,03	0	0,01	0,79	0,2	0	0,046
2	11831	0	0,12	0	0	0	0	0	0,88	0	0	0,01	0,99	0	0,048

2	46375	0	0,14	0	0	0	0	0	0,86	0	0	0	0,86	0,14	0,044
2	5290	0,04	0	0,02	0,05	0	0,44	0	0,44	0,01	0,45	0,02	0,49	0,04	0,046
2	35246	0,03	0,01	0,04	0,01	0,7	0	0	0,21	0	0,24	0,05	0	0,71	0,044
2	53553	0,06	0	0,01	0	0,92	0	0	0,01	0	0	0,01	0,98	0,01	0,044
2	21938	0	0,01	0	0,14	0,4	0	0	0,44	0,01	0,55	0,04	0,37	0,04	0,048
2	61749	0,01	0,06	0	0	0,93	0	0	0	0	0,93	0	0,06	0,01	0,042
2	10441	0	0,02	0,04	0	0	0,72	0	0	0,22	0	0,04	0,74	0,22	0,024
2	100000	0	0,01	0,98	0	0	0	0	0	0,01	0,04	0,95	0,01	0	0,628
2	15581	0	0,31	0	0	0,68	0,01	0	0	0	0,01	0	0,31	0,68	0,048
2	26831	0	0,01	0,03	0,06	0,82	0,08	0	0	0	0,09	0,83	0,08	0	0,042
2	27239	0	0	0	0,05	0,93	0	0,01	0,01	0	0,01	0,98	0	0,01	0,048
2	7970	0	0	0	0,17	0,01	0,07	0,01	0,74	0	0,74	0	0,01	0,25	0,036
2	8018	0	0,11	0,01	0,02	0,19	0	0	0,67	0	0,19	0,01	0,68	0,12	0,044
2	38442	0	0,15	0	0	0,85	0	0	0	0	0,15	0	0,55	0,3	0,048
2	7808	0,01	0	0,04	0	0,91	0	0,04	0	0	0,03	0,06	0,91	0	0,038
2	12662	0	0,02	0,04	0	0,86	0,07	0,01	0	0	0,01	0,04	0,81	0,14	0,034
2	49238	0	0,03	0,03	0	0	0,94	0	0	0	0,97	0,03	0	0	0,042
2	43763	0	0,19	0	0	0,16	0	0	0,63	0,02	0	0,63	0,19	0,18	0,038
2	5802	0	0,11	0	0	0,14	0,71	0	0	0,04	0,15	0	0,79	0,06	0,038
2	10475	0,01	0	0	0,05	0,94	0	0	0	0	0,01	0,03	0	0,96	0,044
2	17234	0	0	0,04	0	0,96	0	0	0	0	0,96	0	0,04	0	0,042
2	2425	0	0,02	0,04	0	0,58	0,01	0	0,33	0,02	0,04	0,05	0,35	0,56	0,014
2	20917	0,01	0,1	0	0	0,88	0	0	0,01	0	0,27	0	0,72	0,01	0,044
2	44060	0,05	0,06	0	0	0,89	0	0	0	0	0,05	0,01	0,05	0,89	0,038
2	52630	0,03	0,15	0	0	0,82	0	0	0	0	0	0,84	0,12	0,04	0,044
2	42631	0	0,12	0	0	0,81	0,07	0	0	0	0,13	0	0,83	0,04	0,046
2	87944	0	0,22	0	0	0,07	0	0	0,71	0	0,19	0,03	0,07	0,71	0,042
2	19870	0	0,07	0	0	0	0,85	0	0	0,08	0,87	0,08	0,05	0	0,046
2	27555	0,06	0	0	0	0,08	0,18	0	0,67	0,01	0,66	0,15	0,19	0	0,048
2	5453	0	0	0,04	0	0,93	0	0	0	0,03	0	0,04	0,96	0	0,026
2	18462	0	0,1	0	0	0,65	0	0	0,24	0,01	0,66	0,24	0	0,1	0,048
2	6139	0	0,07	0	0,06	0,83	0,04	0	0	0	0	0,06	0,07	0,87	0,048
2	2622	0	0	0,04	0,02	0,91	0,01	0,02	0	0	0,01	0,03	0,94	0,02	0,032

2	33018	0	0,07	0,01	0,03	0,04	0,77	0	0	0,08	0,05	0	0,87	0,08	0,038
2	64888	0	0	0,04	0	0,59	0	0	0,37	0	0	0,41	0,59	0	0,018
2	72043	0	0	0,02	0,09	0,85	0	0	0	0,04	0	0,02	0,86	0,12	0,044
2	10818	0	0	0,04	0,06	0	0	0	0,9	0	0	0,11	0	0,89	0,048
2	3308	0,01	0,01	0,04	0	0,23	0,11	0	0,6	0	0,6	0,05	0,11	0,24	0,03
2	64895	0	0,05	0,04	0	0,76	0	0	0,03	0,12	0,03	0,01	0,93	0,03	0,044
2	25725	0,04	0,02	0	0,01	0,93	0	0	0	0	0,97	0,02	0,01	0	0,048
2	6107	0,01	0	0,04	0	0	0	0	0,94	0,01	0	0,05	0,01	0,94	0,014
2,25	78547	0,04	0,03	0	0	0,89	0	0,02	0,01	0,01	0,07	0,02	0	0,91	0,046
2,25	9681	0	0	0,04	0	0,91	0	0	0	0,05	0	0,04	0,9	0,06	0,022
2,25	17407	0,01	0	0,04	0,02	0,05	0,86	0,02	0	0	0	0,11	0,89	0	0,046
2,25	40333	0	0,1	0,04	0	0,84	0,02	0	0	0	0,04	0	0,86	0,1	0,046
2,25	6783	0	0,01	0,04	0	0,94	0	0	0,01	0	0,28	0,04	0,01	0,67	0,016
2,25	19340	0	0	0,02	0	0,01	0,28	0,13	0,56	0	0,56	0,07	0,24	0,13	0,048
2,25	33417	0	0	0,04	0	0	0	0	0,96	0	0	1	0	0	0,006
2,25	31322	0	0,17	0	0	0,83	0	0	0	0	0,16	0,83	0,01	0	0,048
2,25	46312	0	0,09	0	0	0,6	0,31	0	0	0	0,4	0	0,6	0	0,04
2,25	43307	0	0	0,04	0	0	0	0,01	0	0,95	0	0,05	0,01	0,94	0,026
2,25	47605	0	0	0,04	0	0	0,61	0,01	0,07	0,27	0,31	0	0,07	0,62	0,01
2,25	52293	0	0	0,03	0,09	0,82	0,01	0	0	0,05	0	0,09	0,85	0,06	0,048
2,25	53924	0	0	0	0,06	0,94	0	0	0	0	0	0,94	0,06	0	0,048
2,25	4873	0,06	0	0,01	0,01	0,91	0,01	0	0	0	0,16	0,83	0,01	0	0,046
2,25	84290	0	0	0	0,07	0,93	0	0	0	0	0,07	0,42	0	0,51	0,048
2,25	35826	0	0,77	0	0	0,02	0	0	0,2	0,01	0,09	0,19	0	0,72	0,048
2,25	19815	0	0	0	0,06	0,94	0	0	0	0	0,01	0	0	0,99	0,042
2,25	17430	0	0,02	0,04	0	0,66	0	0,01	0,25	0,02	0,25	0,42	0,06	0,27	0,036
2,25	3351	0,02	0,03	0,04	0	0	0,43	0	0	0,48	0,04	0,37	0,51	0,08	0,044
2,25	77384	0	0	0,04	0	0,94	0	0	0,02	0	0,94	0,03	0,03	0	0,046
2,25	10350	0	0	0,04	0	0	0,96	0	0	0	0,96	0,04	0	0	0,044
2,25	29326	0	0	0	0,12	0,12	0	0	0,76	0	0	0	0,75	0,25	0,046
2,25	57875	0	0	0,04	0	0	0,56	0	0,03	0,37	0	0,6	0,37	0,03	0,03
2,25	9493	0	0	0,04	0	0	0,02	0,03	0,91	0	0,02	0,39	0,53	0,06	0,042
2,25	42391	0,03	0	0,04	0	0,01	0,63	0	0,28	0,01	0,17	0,34	0,07	0,42	0,04

2,25	19456	0,01	0	0,04	0,02	0,51	0	0	0,42	0	0	0,39	0,45	0,16	0,03
2,25	14850	0	0,15	0,01	0	0,05	0	0	0,79	0	0,04	0	0,79	0,17	0,048
2,25	50673	0	0,02	0,04	0	0	0,76	0	0	0,18	0	0,2	0,76	0,04	0,034
2,25	31298	0	0	0,01	0,07	0,91	0	0,01	0	0	0,07	0	0,92	0,01	0,048
2,25	22533	0	0,09	0	0	0,7	0,17	0,04	0	0	0,04	0,17	0,79	0	0,04
2,25	17470	0	0	0	0,11	0,88	0	0	0,01	0	0,11	0	0,88	0,01	0,042
2,25	2395	0	0	0,04	0	0	0,96	0	0	0	0	0,09	0,91	0	0,014
2,25	14513	0	0,07	0	0	0,87	0	0,06	0	0	0,87	0,02	0,11	0	0,048
2,25	69241	0	0	0,02	0,07	0,9	0	0	0	0,01	0,01	0,01	0,9	0,08	0,044
2,25	81460	0	0,08	0,02	0,01	0	0,01	0	0,87	0,01	0	0,12	0,87	0,01	0,046
2,25	57592	0,05	0	0,01	0	0,42	0,51	0	0,01	0	0	0,44	0,51	0,05	0,048
2,25	5090	0	0	0,03	0	0	0,86	0,11	0	0	0,06	0,03	0,86	0,05	0,046
2,25	2547	0	0	0,04	0	0	0,52	0	0,22	0,22	0	0,48	0,52	0	0,028
2,25	31566	0	0,01	0,04	0	0,87	0	0	0,08	0	0	0,88	0	0,12	0
2,25	100000	0	0	0,98	0	0	0	0	0,01	0,01	0,01	0,85	0,14	0	0,516
2,25	15616	0	0	0,04	0	0,24	0,57	0,08	0,07	0	0,36	0,56	0,08	0	0,042
2,25	12929	0	0,11	0,04	0	0,84	0	0	0,01	0	0,01	0,15	0,8	0,04	0,048
2,25	37508	0	0	0,04	0	0,93	0	0,01	0,01	0,01	0	0,04	0,94	0,02	0,028
2,25	9946	0	0,22	0,03	0,01	0,65	0	0	0,09	0	0,09	0,04	0,22	0,65	0,042
2,25	6701	0,05	0	0,03	0,02	0,9	0	0	0	0	0,07	0	0,93	0	0,048
2,25	88911	0,01	0,11	0	0,01	0,87	0	0	0	0	0,35	0,64	0	0,01	0,034
2,25	19636	0	0	0,04	0,02	0,72	0,01	0,03	0,1	0,08	0,1	0,78	0,09	0,03	0,046
2,25	39406	0	0,53	0	0,01	0,45	0	0	0	0,01	0	0,54	0,1	0,36	0,048
2,25	100000	0	0	0,98	0	0	0	0,01	0,01	0	0	0,94	0,05	0,01	0,524
2,25	8340	0	0,15	0	0	0,85	0	0	0	0	0,15	0	0,85	0	0,046
2,25	70383	0	0,05	0	0,09	0,85	0,01	0	0	0	0,9	0,01	0,09	0	0,046
2,25	54408	0,01	0,31	0,01	0	0,64	0	0	0,03	0	0,14	0,02	0,07	0,77	0,042
2,25	28639	0	0	0,04	0	0,96	0	0	0	0	0	0,02	0,98	0	0,03
2,25	7454	0	0	0,04	0	0,48	0,26	0,01	0	0,21	0,09	0,67	0,12	0,12	0,03
2,25	16740	0	0,17	0	0	0	0,18	0	0,65	0	0	0,17	0,67	0,16	0,042
2,25	24639	0	0,04	0,02	0	0,94	0	0	0	0	0,98	0,01	0,01	0	0,04
2,25	57821	0	0,04	0,04	0	0,07	0	0	0,72	0,13	0,14	0,09	0,03	0,74	0,018
2,25	37500	0	0	0	0,06	0	0,02	0	0,92	0	0,01	0,06	0,01	0,92	0,042

2,25	85714	0,05	0	0,04	0	0,89	0	0	0	0,02	0,03	0,92	0,05	0	0,048
2,25	21507	0	0,11	0	0	0,24	0,64	0,01	0	0	0,02	0,66	0,32	0	0,044
2,25	15861	0	0	0,04	0,01	0,81	0	0	0	0,14	0,82	0,03	0,14	0,01	0,026
2,25	48704	0,01	0	0	0,11	0,85	0	0,02	0,01	0	0,07	0,05	0,88	0	0,042
2,25	36233	0	0,07	0	0	0	0,03	0	0,9	0	0,97	0,01	0,02	0	0,042
2,25	24085	0,02	0	0,04	0,01	0,92	0	0	0,01	0	0,01	0,15	0,83	0,01	0,046
2,25	26166	0	0	0	0,11	0	0	0	0,88	0,01	0,3	0,55	0,11	0,04	0,042
2,25	28030	0,03	0	0,04	0	0,93	0	0	0	0	0,02	0,04	0,94	0	0,048
2,25	18575	0,06	0	0	0	0	0,91	0	0,03	0	0,06	0,93	0	0,01	0,048
2,25	74430	0	0,07	0,01	0	0	0,91	0,01	0	0	0,92	0,08	0	0	0,046
2,25	14430	0	0,14	0	0	0,86	0	0	0	0	0	0,14	0,86	0	0,04
2,25	41930	0	0	0,04	0,02	0,93	0	0,01	0	0	0,03	0	0	0,97	0,028
2,25	7239	0	0	0	0,3	0,69	0,01	0	0	0	0,99	0	0	0,01	0,044
2,25	38999	0	0,2	0	0	0,76	0	0	0	0,04	0,02	0,78	0,18	0,02	0,046
2,25	23350	0	0	0,04	0,01	0,95	0	0	0	0	0	0	0,05	0,95	0,01
2,25	39909	0,04	0	0,04	0	0,86	0	0	0,06	0	0,06	0,04	0,04	0,86	0,048
2,25	45294	0	0	0,04	0	0	0,96	0	0	0	0	0,85	0	0,15	0,028
2,25	23950	0,03	0	0,03	0,02	0,91	0	0,01	0	0	0,94	0	0,01	0,05	0,048
2,25	4071	0	0,27	0	0	0,12	0	0,01	0,6	0	0	0,12	0,28	0,6	0,046
2,25	5118	0,04	0	0,02	0	0,91	0	0,02	0	0,01	0	0,93	0,07	0	0,044
2,25	78124	0,03	0	0,03	0	0,73	0,06	0,01	0,14	0	0	0,08	0,79	0,13	0,04
2,25	18051	0	0,25	0	0	0,75	0	0	0	0	0	0,75	0,25	0	0,046
2,25	35150	0,02	0	0,03	0,03	0,92	0	0	0	0	0,02	0,04	0,92	0,02	0,042
2,25	14143	0,03	0	0,02	0	0,73	0,14	0	0,08	0	0,03	0,16	0,73	0,08	0,042
2,25	56804	0	0	0,04	0,02	0,65	0	0,03	0,26	0	0,26	0,04	0,02	0,68	0,038
2,25	34686	0	0,3	0	0	0,7	0	0	0	0	0,08	0	0,27	0,65	0,048
2,25	51237	0	0	0,01	0,06	0,93	0	0	0	0	0	0,01	0	0,99	0,048
2,25	27928	0,05	0	0,01	0	0,16	0,03	0	0,75	0	0	0,08	0,81	0,11	0,048
2,25	100000	0	0,01	0,99	0	0	0	0	0	0	0	0,99	0	0,01	0,538
2,25	25382	0	0,08	0	0,05	0,73	0	0	0	0,14	0,19	0,61	0,06	0,14	0,048
2,25	43520	0	0	0	0,04	0,93	0	0,02	0	0,01	0,02	0,03	0	0,95	0,042
2,25	12272	0,01	0,02	0,01	0,13	0,83	0	0	0	0	0,83	0,03	0	0,14	0,044
2,25	22731	0,01	0	0	0,19	0,15	0,61	0,01	0,03	0	0,96	0	0,03	0,01	0,048

2,25	12226	0	0	0,04	0	0,01	0,01	0	0,94	0	0,02	0,04	0	0,94	0
2,25	3686	0	0,11	0	0,01	0	0	0	0,88	0	0,09	0	0,85	0,06	0,042
2,25	36223	0	0	0,04	0,02	0,94	0	0	0	0	0,04	0,02	0	0,94	0,032
2,25	29338	0,04	0	0	0,01	0	0	0	0,95	0	0	0	0,12	0,88	0,036
2,25	15064	0,01	0,07	0	0	0,41	0,5	0	0	0,01	0,92	0,01	0,07	0	0,042
2,25	31121	0	0	0	0,07	0,88	0,01	0	0,04	0	0,01	0,88	0,05	0,06	0,048
2,25	41139	0	0,12	0	0	0,87	0	0,01	0	0	0,01	0	0,99	0	0,048
2,25	15167	0	0,05	0	0,05	0,89	0	0,01	0	0	0,05	0,95	0	0	0,048
2,25	4434	0,02	0,12	0,04	0,03	0,23	0	0	0,56	0	0,07	0,11	0,08	0,74	0,048
2,5	23821	0	0,06	0	0,05	0,87	0,01	0	0	0,01	0,48	0,05	0,46	0,01	0,042
2,5	2352	0	0	0,04	0	0,05	0,59	0	0	0,32	0	0,04	0,05	0,91	0,01
2,5	28249	0	0,02	0,03	0,01	0,69	0	0,05	0,2	0	0	0,17	0,09	0,74	0,046
2,5	62984	0	0,11	0,01	0	0,87	0,01	0	0	0	0,11	0,25	0,63	0,01	0,042
2,5	23942	0	0,23	0	0,01	0,76	0	0	0	0	0,23	0	0	0,77	0,036
2,5	14087	0	0	0,04	0	0,86	0,1	0	0	0	0	0,04	0,1	0,86	0,002
2,5	95878	0	0,21	0,02	0,01	0,01	0,01	0	0,65	0,09	0,04	0	0,3	0,66	0,046
2,5	15326	0	0	0,03	0,16	0,21	0,02	0	0,57	0,01	0,8	0,18	0,01	0,01	0,048
2,5	42826	0,06	0	0	0	0	0,55	0,01	0,38	0	0,33	0,02	0,59	0,06	0,042
2,5	20356	0,02	0,03	0,02	0,01	0,2	0	0,01	0,25	0,46	0,68	0,27	0,02	0,03	0,04
2,5	43905	0	0,03	0,02	0,06	0,82	0	0,06	0,01	0	0,82	0,09	0,09	0	0,046
2,5	25675	0	0	0,04	0	0,96	0	0	0	0	0	0	0,97	0,03	0,016
2,5	40858	0	0,12	0	0	0,87	0	0	0	0,01	0	0	0,99	0,01	0,04
2,5	8058	0,03	0,97	0	0	0	0	0	0	0	0	0	0,03	0,97	0,046
2,5	4641	0,04	0	0,04	0,02	0,72	0,18	0	0	0	0,19	0,72	0,09	0	0,044
2,5	100000	0	0	0,98	0	0	0,02	0	0	0	0,02	0,98	0	0	0,68
2,5	13061	0	0,94	0,04	0	0	0	0	0,01	0,01	0	0,01	0	0,99	0,018
2,5	36650	0	0,23	0,02	0	0	0	0	0,75	0	0,03	0	0,25	0,72	0,046
2,5	37407	0,04	0,02	0	0,01	0,82	0	0	0,11	0	0,04	0,12	0,02	0,82	0,038
2,5	7977	0	0	0,04	0,01	0,87	0,01	0	0,04	0,03	0,05	0,19	0,07	0,69	0,022
2,5	29697	0,03	0	0	0,02	0,92	0,01	0,01	0,01	0	0,04	0,02	0,93	0,01	0,048
2,5	5813	0,04	0	0,01	0	0,83	0	0	0,12	0	0,86	0,13	0,01	0	0,04
2,5	34628	0	0	0,04	0	0,96	0	0	0	0	0,96	0,04	0	0	0,038
2,5	33026	0	0	0,04	0,01	0,95	0	0	0	0	0,01	0,02	0,95	0,02	0,024

2,5	32917	0	0	0,04	0	0,87	0	0	0,09	0	0,49	0,02	0,45	0,04	0,018
2,5	100000	0	0	0,96	0	0,03	0,01	0	0	0	0	0,96	0	0,04	0,508
2,5	3446	0	0,01	0,04	0,02	0,76	0	0	0	0,17	0,03	0,19	0,03	0,75	0,028
2,5	63993	0	0	0,04	0	0,52	0,03	0	0	0,41	0	0,04	0	0,96	0,004
2,5	64052	0	0,12	0	0	0,88	0	0	0	0	0	0	1	0	0,042
2,5	19310	0	0	0	0,09	0,74	0	0	0,17	0	0,04	0	0,09	0,87	0,048
2,5	75043	0	0,26	0	0,01	0,73	0	0	0	0	0,25	0,01	0	0,74	0,048
2,5	46965	0,03	0,01	0,02	0,02	0,84	0,08	0	0	0	0,72	0,1	0	0,18	0,044
2,5	19262	0	0,07	0	0	0,32	0,61	0	0	0	1	0	0	0	0,036
2,5	67920	0,05	0,03	0,02	0	0,89	0	0	0	0,01	0,09	0,91	0	0	0,046
2,5	24439	0	0	0,02	0,05	0,93	0	0	0	0	0	0,02	0,02	0,96	0,044
2,5	52597	0	0	0,04	0,06	0	0,03	0	0,85	0,02	0	0,06	0,88	0,06	0,046
2,5	5342	0	0	0,04	0,05	0,8	0,11	0	0	0	0,61	0,35	0,04	0	0,044
2,5	37475	0	0,19	0,02	0	0,74	0	0	0	0,05	0,19	0,81	0	0	0,048
2,5	7522	0	0	0,04	0	0	0,01	0	0	0,95	0	0,04	0,01	0,95	0,008
2,5	18263	0	0	0,04	0	0,95	0	0,01	0	0	0,04	0,22	0,01	0,73	0,012
2,5	31674	0,02	0	0,04	0	0,64	0	0	0,28	0,02	0,3	0,04	0,02	0,64	0,038
2,5	8037	0	0	0,04	0	0,81	0	0	0,15	0	0	0	0,04	0,96	0,002
2,5	4148	0	0	0,04	0,04	0,56	0,23	0	0	0,13	0,52	0,08	0,17	0,23	0,04
2,5	18005	0	0,11	0,01	0	0,84	0	0,01	0,03	0	0,03	0,01	0,95	0,01	0,044
2,5	28663	0	0	0,03	0,06	0,64	0	0,02	0	0,25	0,42	0,4	0,06	0,12	0,04
2,5	22942	0,04	0	0,04	0	0,89	0	0,01	0	0,02	0,01	0,04	0,94	0,01	0,044
2,5	62870	0	0	0,04	0	0,03	0	0	0,93	0	0,03	0	0,93	0,04	0,018
2,5	10400	0,05	0	0,03	0	0	0	0	0,91	0,01	0,02	0,93	0	0,05	0,044
2,5	100000	0,06	0,03	0,91	0	0	0	0	0	0	0,09	0,88	0	0,03	0,636
2,5	26105	0,05	0	0	0	0	0	0	0,95	0	0,95	0,05	0	0	0,048
2,5	68509	0	0	0,04	0	0	0,96	0	0	0	0,01	0,03	0	0,96	0,01
2,5	32305	0	0,9	0	0,01	0,09	0	0	0	0	0	0	0,09	0,91	0,048
2,5	19416	0	0,28	0	0	0,72	0	0	0	0	0,28	0	0	0,72	0,044
2,5	17999	0,04	0	0,03	0	0,93	0	0	0	0	0,99	0,01	0	0	0,048
2,5	63914	0	0	0,04	0,01	0,3	0,61	0	0	0,04	0,05	0,34	0,61	0	0,038
2,5	5033	0,04	0	0	0,01	0	0,95	0	0	0	0,05	0	0	0,95	0,044
2,5	10294	0	0	0	0,06	0,89	0	0	0,05	0	0,06	0,89	0,05	0	0,04

2,5	19381	0,01	0,11	0,04	0	0,76	0,07	0,01	0	0	0,02	0,63	0	0,35	0,02
2,5	22472	0,01	0,04	0	0,07	0,84	0,03	0,01	0	0	0,84	0	0,11	0,05	0,038
2,5	18903	0	0,02	0,04	0,04	0	0,9	0	0	0	0,04	0,04	0,02	0,9	0,042
2,5	12993	0	0,21	0,04	0	0	0	0	0,05	0,7	0,05	0,25	0	0,7	0,048
2,5	32516	0	0,01	0,04	0,01	0,02	0,02	0	0,9	0	0	0,06	0,93	0,01	0,026
2,5	22748	0	0	0	0,04	0,95	0	0,01	0	0	0	0,01	0,03	0,96	0,048
2,5	15909	0	0,06	0,04	0	0,09	0,81	0	0	0	0	0,04	0,81	0,15	0,038
2,5	29419	0	0	0,04	0,03	0	0,83	0	0	0,1	0	0,1	0,86	0,04	0,036
2,5	24334	0	0	0,04	0	0,03	0,25	0,06	0	0,62	0	0,37	0,62	0,01	0,048
2,5	34713	0	0,11	0	0,02	0,5	0,37	0	0	0	0,24	0,02	0,74	0	0,044
2,5	25188	0	0	0,04	0	0	0,02	0	0,84	0,1	0,85	0,03	0	0,12	0,036
2,5	34522	0,01	0	0,02	0,1	0,86	0	0,01	0	0	0,85	0,14	0,01	0	0,04
2,5	83385	0	0	0,04	0	0,94	0,01	0,01	0	0	0	0,03	0,94	0,03	0,022
2,5	25760	0	0	0,02	0,06	0	0,88	0,04	0	0	0,06	0,86	0,06	0,02	0,048
2,5	38856	0,04	0	0	0	0,88	0,07	0,01	0	0	0,89	0,11	0	0	0,038
2,5	22548	0	0,04	0,04	0	0,64	0,23	0,05	0	0	0,07	0,05	0,23	0,65	0,048
2,5	5427	0	0	0,04	0	0,23	0,73	0	0	0	0	0,04	0,96	0	0,022
2,5	18877	0	0,08	0	0	0,91	0,01	0	0	0	0,92	0	0,08	0	0,048
2,5	100000	0	0	1	0	0	0	0	0	0	0	0,99	0	0,01	0,658
2,5	64553	0,02	0,93	0,02	0	0,01	0,01	0	0,01	0	0,03	0	0,01	0,96	0,046
2,5	23558	0,05	0	0,01	0	0,94	0	0	0	0	0,94	0	0,05	0,01	0,048
2,5	14475	0	0	0,04	0	0,89	0	0	0,04	0,03	0,03	0,89	0,05	0,03	0,01
2,5	17183	0	0,09	0	0,06	0,71	0	0	0,14	0	0,23	0,06	0	0,71	0,042
2,5	13837	0,01	0,01	0,04	0	0	0,34	0,03	0,53	0,04	0,01	0,38	0,6	0,01	0,046
2,5	74192	0	0	0,04	0	0,86	0	0	0,1	0	0	0,04	0,1	0,86	0,004
2,5	26867	0	0	0	0,19	0,26	0,55	0	0	0	0,43	0,57	0	0	0,046
2,5	82486	0	0,03	0,04	0	0,68	0	0	0	0,25	0,09	0,72	0	0,19	0,028
2,5	44660	0	0	0,04	0	0,92	0	0,04	0	0	0,92	0	0,04	0,04	0,048
2,5	16641	0	0	0	0,1	0	0,38	0	0,52	0	0	0,01	0,52	0,47	0,048
2,5	27585	0	0,62	0	0	0,06	0,04	0	0,28	0	0,12	0,06	0	0,82	0,046
2,5	55548	0	0,08	0	0	0,86	0,06	0	0	0	0,88	0	0,06	0,06	0,046
2,5	49179	0	0	0,04	0,04	0	0,92	0	0	0	0	0	0,08	0,92	0,04
2,5	17072	0,03	0,02	0,01	0,02	0,92	0	0	0	0	0,02	0,01	0,93	0,04	0,042

2,5	5968	0,05	0	0	0,01	0,93	0	0	0	0,01	0,05	0,01	0	0,94	0,048
2,5	4154	0	0	0,04	0	0	0,35	0	0,61	0	0	0,31	0,35	0,34	0,014
2,5	100000	0	0,23	0	0	0,47	0,3	0	0	0	0,28	0,02	0,47	0,23	0,092
2,5	13567	0	0,08	0,04	0	0	0	0	0,59	0,29	0	0,04	0,59	0,37	0,036
2,5	6941	0	0,59	0,03	0,02	0,2	0	0	0,13	0,03	0,03	0,16	0,02	0,79	0,046
2,5	19341	0	0,14	0,02	0	0,35	0,47	0	0	0,02	0	0,61	0,37	0,02	0,048
2,5	65199	0,04	0	0,03	0	0,71	0	0	0,07	0,15	0,75	0,1	0	0,15	0,044
2,5	4648	0	0,02	0	0	0	0,89	0,09	0	0	0,21	0	0,02	0,77	0,046
2,5	17103	0,04	0	0,03	0	0,93	0	0	0	0	0,61	0,03	0,36	0	0,046
2,5	78225	0	0	0,04	0	0	0	0,01	0,92	0,03	0	0,95	0,02	0,03	0,018
2,75	19407	0	0,03	0	0,16	0,81	0	0	0	0	0,81	0	0,19	0	0,038
2,75	4349	0,01	0	0,04	0	0,02	0	0	0,93	0	0	0,95	0,04	0,01	0,022
2,75	35465	0,03	0	0	0	0,92	0,01	0,04	0	0	0,07	0,87	0,06	0	0,046
2,75	16322	0,03	0	0,04	0,01	0	0,82	0,02	0	0,08	0,04	0,83	0,13	0	0,048
2,75	27656	0	0	0,04	0	0,79	0,05	0,06	0	0,06	0,09	0,8	0,11	0	0,046
2,75	32270	0	0	0,04	0,01	0,74	0	0	0	0,21	0,25	0,2	0,48	0,07	0,042
2,75	36032	0	0	0	0,29	0	0,7	0	0	0,01	0,87	0,09	0,04	0	0,04
2,75	90049	0	0	0,04	0,01	0,72	0,23	0	0	0	0,24	0,73	0,01	0,02	0,034
2,75	30992	0	0	0,04	0	0,91	0,01	0,04	0	0	0	0,05	0,04	0,91	0,03
2,75	15731	0	0	0,04	0,02	0,3	0	0	0	0,64	0,12	0,04	0	0,84	0,02
2,75	26534	0,01	0	0	0,02	0	0,9	0,07	0	0	0,02	0	0,91	0,07	0,048
2,75	41817	0,01	0	0,04	0	0,59	0	0	0	0,36	0,01	0,04	0,95	0	0,028
2,75	36186	0	0,09	0,03	0	0,12	0,37	0	0,33	0,06	0,36	0	0,46	0,18	0,042
2,75	69046	0	0	0,04	0	0,27	0	0	0,69	0	0	0,18	0,36	0,46	0,02
2,75	6970	0	0,14	0	0	0,85	0	0	0,01	0	0	0	1	0	0,048
2,75	12986	0	0,02	0	0,1	0,88	0	0	0	0	0	0	1	0	0,048
2,75	17194	0,01	0,07	0,02	0	0,28	0,38	0,03	0,09	0,12	0,03	0,43	0,54	0	0,044
2,75	58986	0,01	0	0,02	0,13	0,77	0,03	0,04	0	0	0,78	0,04	0,14	0,04	0,034
2,75	33728	0	0,05	0,04	0,03	0	0,55	0	0	0,33	0,6	0,02	0,36	0,02	0,046
2,75	6280	0	0,07	0	0	0	0,92	0	0	0,01	0,92	0	0,07	0,01	0,044
2,75	19045	0,05	0	0	0	0	0	0	0	0,95	0	0	0,05	0,95	0,044
2,75	40595	0,01	0,09	0,02	0	0,58	0,13	0	0,17	0	0,1	0,6	0,3	0	0,046
2,75	42243	0,06	0	0	0	0	0,94	0	0	0	0,06	0	0	0,94	0,036

2,75	37547	0,02	0	0	0,05	0,71	0	0	0,22	0	0,24	0	0	0,76	0,04
2,75	17071	0	0,08	0	0	0,92	0	0	0	0	0,92	0	0	0,08	0,044
2,75	11062	0	0	0	0,12	0,88	0	0	0	0	0,12	0	0,88	0	0,044
2,75	18561	0	0	0,04	0,06	0,02	0,73	0	0	0,15	0,87	0,04	0,02	0,07	0,044
2,75	7711	0,04	0	0	0	0,75	0,2	0,01	0	0	0,04	0,2	0	0,76	0,046
2,75	34907	0	0,01	0,04	0,02	0	0,79	0	0	0,14	0,04	0,18	0	0,78	0,032
2,75	4378	0	0	0,04	0	0,6	0,35	0	0	0,01	0	0,05	0,95	0	0,022
2,75	24473	0	0	0	0,06	0,53	0	0	0,4	0,01	0,04	0,58	0	0,38	0,042
2,75	23205	0	0,31	0,01	0	0,68	0	0	0	0	0,01	0	0,31	0,68	0,032
2,75	24169	0	0,86	0,01	0	0,06	0,01	0	0,06	0	0,07	0,01	0	0,92	0,044
2,75	27503	0,03	0	0	0	0,9	0	0,05	0,02	0	0,09	0,9	0,01	0	0,046
2,75	25341	0	0,01	0,04	0	0	0	0	0,94	0,01	0	0,04	0,02	0,94	0,002
2,75	8694	0	0	0,04	0	0,34	0,48	0,06	0	0,08	0	0,54	0,08	0,38	0,042
2,75	81262	0	0	0,04	0	0,46	0,35	0,03	0,12	0	0	0,16	0,38	0,46	0,034
2,75	100000	0	0,05	0,41	0,02	0	0,26	0	0,26	0	0,28	0,45	0,02	0,25	0,23
2,75	11295	0,05	0	0,02	0	0,11	0	0	0,82	0	0	0,84	0,11	0,05	0,046
2,75	12860	0	0,14	0	0,02	0	0	0	0,84	0	0,09	0,77	0,14	0	0,046
2,75	31172	0	0	0,03	0	0,01	0	0,02	0,94	0	0	0,04	0,94	0,02	0,022
2,75	9110	0	0	0,04	0,01	0	0	0	0,46	0,49	0,46	0,04	0	0,5	0,026
2,75	29425	0	0,02	0,04	0	0	0,14	0	0,28	0,52	0,02	0,01	0,42	0,55	0,026
2,75	100000	0	0	0,99	0	0	0	0	0	0,01	0,01	0,99	0	0	0,61
2,75	41570	0	0	0	0,14	0	0	0	0,86	0	0,09	0	0,9	0,01	0,042
2,75	40732	0,06	0	0,01	0	0,28	0,09	0,01	0,33	0,22	0,47	0,06	0,28	0,19	0,044
2,75	6469	0	0,14	0,04	0,02	0,75	0,01	0	0,04	0	0	0,93	0,03	0,04	0,032
2,75	21873	0	0,01	0	0,17	0,81	0	0	0	0,01	0,83	0	0,12	0,05	0,044
2,75	12321	0	0,9	0	0,03	0	0,05	0	0,02	0	0,02	0,03	0	0,95	0,036
2,75	31890	0	0,15	0	0	0,85	0	0	0	0	0,08	0,4	0,52	0	0,048
2,75	19714	0,02	0	0,04	0,02	0,31	0,01	0	0,6	0	0,31	0,04	0,02	0,63	0,036
2,75	45392	0	0,01	0,04	0	0,94	0	0	0,01	0	0,01	0,04	0,94	0,01	0,032
2,75	9464	0	0,29	0	0	0,7	0	0	0,01	0	0	0	0,29	0,71	0,03
2,75	41767	0,04	0	0,02	0,01	0,93	0	0	0	0	0,86	0,02	0,04	0,08	0,042
2,75	24014	0,02	0	0,04	0	0,71	0	0,01	0,09	0,13	0	0,14	0,36	0,5	0,042
2,75	23150	0,01	0,93	0	0,01	0,01	0,02	0,01	0	0,01	0,01	0	0,02	0,97	0,048

2,75	85552	0,01	0	0	0,17	0,8	0,01	0	0	0,01	0,76	0,23	0	0,01	0,048
2,75	22891	0	0,17	0	0	0,81	0	0	0	0,02	0,01	0,01	0,98	0	0,048
2,75	100000	0	0	0,98	0	0	0,01	0	0,01	0	0	0,98	0,02	0	0,556
2,75	4321	0	0	0,04	0	0,09	0,4	0,01	0,46	0	0,05	0,46	0,4	0,09	0,028
2,75	10022	0,05	0	0	0,01	0,94	0	0	0	0	0,01	0,04	0,95	0	0,04
2,75	24207	0	0	0,04	0	0	0	0,01	0,55	0,4	0	0,53	0	0,47	0,006
2,75	17465	0,04	0	0	0	0	0	0,03	0,93	0	0,42	0	0,02	0,56	0,046
2,75	3317	0	0	0,04	0	0,95	0	0	0	0,01	0,01	0,04	0,95	0	0,026
2,75	87426	0	0,1	0	0	0,9	0	0	0	0	0,57	0,33	0,1	0	0,044
2,75	29619	0,02	0	0,02	0,1	0,86	0	0	0	0	0,87	0,12	0,01	0	0,048
2,75	64059	0,03	0	0,01	0,08	0,27	0	0,01	0,6	0	0,88	0,1	0,01	0,01	0,044
2,75	27169	0,01	0	0	0,05	0,94	0	0	0	0	0,08	0	0	0,92	0,046
2,75	51721	0	0,21	0	0	0,77	0	0	0,02	0	0	0,79	0,21	0	0,046
2,75	24844	0,01	0	0,04	0	0	0	0,01	0	0,94	0,01	0,05	0,94	0	0,032
2,75	14139	0	0	0	0,05	0	0,91	0,04	0	0	0	0	0,18	0,82	0,036
2,75	70987	0,01	0,01	0	0,1	0	0,88	0	0	0	0	0	0,99	0,01	0,046
2,75	11764	0	0	0,04	0	0,88	0	0,06	0	0,02	0,06	0,92	0,02	0	0,046
2,75	99863	0	0	0	0,16	0	0,81	0	0,03	0	0,73	0	0,19	0,08	0,048
2,75	4839	0,03	0	0,03	0	0,74	0,2	0	0	0	0,95	0,03	0	0,02	0,048
2,75	77123	0	0	0	0,19	0,8	0	0,01	0	0	0,8	0,01	0,19	0	0,048
2,75	6051	0	0,92	0	0	0	0,08	0	0	0	0,08	0	0	0,92	0,042
2,75	5559	0,02	0	0,04	0,03	0,9	0	0	0,01	0	0,04	0,9	0,05	0,01	0,046
2,75	79030	0	0,15	0,02	0	0,74	0	0	0	0,09	0,06	0,51	0,43	0	0,048
2,75	20419	0	0	0	0,1	0,86	0	0,02	0,02	0	0,02	0	0,86	0,12	0,044
2,75	19638	0	0	0,04	0,02	0,01	0,06	0	0	0,87	0,06	0,05	0	0,89	0,032
2,75	18367	0	0,96	0	0,02	0	0	0,01	0	0,01	0,03	0	0	0,97	0,038
2,75	39976	0,04	0,02	0	0	0	0,93	0,01	0	0	0,05	0	0,02	0,93	0,048
2,75	2556	0	0,46	0,01	0	0,48	0,02	0,01	0	0,02	0,02	0,32	0,09	0,57	0,048
2,75	7960	0	0,17	0,02	0,01	0,71	0	0	0,09	0	0,19	0,02	0,31	0,48	0,048
2,75	21216	0	0,01	0,04	0,01	0,46	0	0	0,45	0,03	0,03	0,04	0,45	0,48	0,026
2,75	4974	0	0	0	0,08	0,36	0	0	0,56	0	0	0,26	0,38	0,36	0,046
2,75	5359	0	0,12	0,01	0	0,86	0,01	0	0	0	0,12	0,07	0,8	0,01	0,046
2,75	20757	0,01	0,02	0,04	0,01	0,92	0	0	0	0	0,01	0	0,93	0,06	0,042

2,75	30569	0	0	0,04	0	0,21	0,01	0	0	0,74	0,21	0,04	0,01	0,74	0,01
2,75	47955	0	0	0	0,06	0,87	0	0,01	0,06	0	0,01	0,93	0,06	0	0,046
2,75	29122	0	0	0,04	0	0,96	0	0	0	0	0	0,04	0,96	0	0,026
2,75	17271	0	0	0,03	0,11	0	0	0	0,86	0	0	0,03	0,87	0,1	0,048
2,75	5745	0,03	0	0,04	0,01	0	0,59	0	0	0,33	0,59	0,05	0,03	0,33	0,046
2,75	38348	0,01	0,31	0	0	0,64	0,02	0	0,01	0,01	0,02	0,95	0,03	0	0,046
2,75	94719	0	0,01	0	0,06	0,34	0	0	0,59	0	0	0,6	0,03	0,37	0,04
2,75	61254	0	0,05	0,02	0	0	0,93	0	0	0	0,97	0	0,03	0	0,048
2,75	16798	0	0	0,04	0,03	0,09	0	0,01	0	0,83	0	0,13	0,08	0,79	0,044
2,75	25406	0	0,11	0	0,01	0,88	0	0	0	0	0	0,01	0,99	0	0,046
2,75	18177	0	0	0	0,06	0,89	0	0	0	0,05	0,01	0	0,06	0,93	0,038
3	57336	0	0,14	0	0	0,78	0	0	0,08	0	0	0	0,95	0,05	0,048
3	40586	0	0	0,04	0,02	0,15	0,03	0	0,57	0,19	0,01	0,21	0,14	0,64	0,016
3	61339	0	0,09	0	0,07	0,64	0	0	0,2	0	0,01	0,07	0,19	0,73	0,048
3	17388	0,05	0	0,02	0	0,02	0	0	0,87	0,04	0,05	0	0,93	0,02	0,048
3	12857	0,06	0	0	0	0	0	0	0,94	0	0	0,94	0,06	0	0,046
3	1970	0,01	0	0,04	0	0,77	0	0	0,18	0	0,77	0,04	0,18	0,01	0,044
3	19339	0	0	0,01	0,07	0,92	0	0	0	0	0,07	0,19	0	0,74	0,048
3	20039	0,02	0	0,04	0	0	0	0	0,94	0	0	0,26	0,68	0,06	0,042
3	16881	0	0	0,04	0	0,17	0,35	0,02	0,42	0	0,17	0,06	0,42	0,35	0,034
3	54441	0	0	0	0,05	0,9	0,05	0	0	0	0,09	0,01	0	0,9	0,048
3	12245	0	0	0,01	0,06	0	0	0	0,93	0	0	0,05	0	0,95	0,042
3	5434	0	0	0,03	0	0,66	0	0,05	0,2	0,06	0,19	0,09	0,06	0,66	0,048
3	9189	0,04	0,04	0	0	0	0	0	0,91	0,01	0,02	0,04	0,89	0,05	0,042
3	19515	0	0,08	0,04	0	0,87	0	0	0	0,01	0,02	0,07	0,04	0,87	0,008
3	48555	0	0	0,01	0,24	0,75	0	0	0	0	0,99	0	0,01	0	0,044
3	27636	0,04	0	0	0	0,91	0,04	0	0	0,01	0,08	0,01	0,91	0	0,048
3	34606	0	0	0,04	0,03	0	0,06	0	0	0,87	0	0,04	0,09	0,87	0,028
3	51632	0,06	0	0,01	0	0,91	0	0	0,01	0,01	0,06	0,01	0,02	0,91	0,046
3	66056	0	0	0,04	0	0,95	0,01	0	0	0	0,6	0	0,01	0,39	0,026
3	11862	0	0	0,04	0	0	0,01	0	0	0,95	0	0,05	0	0,95	0,016
3	54793	0	0,16	0,01	0,01	0,82	0	0	0	0	0,09	0,73	0,17	0,01	0,044
3	19480	0	0,17	0	0	0,81	0	0,02	0	0	0,19	0,81	0	0	0,042

3	28189	0	0,1	0	0,06	0,84	0	0	0	0	0,1	0	0,06	0,84	0,048
3	12001	0	0	0,04	0	0,91	0,05	0	0	0	0	0,04	0,95	0,01	0,03
3	100000	0	0,02	0,95	0	0,02	0,01	0	0	0	0	0,99	0	0,01	0,55
3	100000	0	0	0,99	0	0	0	0	0	0,01	0	0,99	0,01	0	0,602
3	55015	0	0,09	0,01	0	0	0	0,01	0,89	0	0,1	0	0,9	0	0,042
3	44324	0,04	0	0	0,06	0,62	0,27	0	0	0,01	0,93	0	0	0,07	0,044
3	81980	0	0,01	0,04	0,02	0,89	0,01	0	0,03	0	0,9	0	0,03	0,07	0,038
3	48835	0	0	0	0,25	0,7	0,05	0	0	0	0,94	0,05	0,01	0	0,048
3	75396	0	0	0,04	0,01	0,87	0	0	0	0,08	0	0,92	0,08	0	0,014
3	37094	0	0,72	0,04	0	0	0,02	0	0,03	0,19	0,01	0,04	0,03	0,92	0,044
3	8410	0,05	0	0,01	0	0	0,01	0	0,93	0	0,05	0,01	0,93	0,01	0,044
3	51467	0	0,15	0,04	0	0	0	0	0,02	0,79	0,17	0,04	0	0,79	0,044
3	8893	0	0,06	0,02	0	0	0	0	0,92	0	0,98	0,02	0	0	0,048
3	38843	0,02	0,06	0,03	0	0,53	0,15	0,01	0,2	0	0	0,03	0,82	0,15	0,048
3	55489	0	0	0	0,06	0,94	0	0	0	0	0,06	0,94	0	0	0,044
3	96590	0,01	0,13	0,03	0	0	0	0,02	0,73	0,08	0,01	0,85	0,02	0,12	0,048
3	92952	0	0	0	0	0,95	0	0,05	0	0	0	0,93	0	0,07	0,046
3	8267	0	0	0,04	0,04	0,7	0,08	0	0,06	0,08	0,02	0,2	0,78	0	0,036
3	26171	0	0	0	0,06	0,92	0	0	0	0,02	0	0,92	0,08	0	0,046
3	13970	0,01	0	0,01	0,06	0,91	0,01	0	0	0	0,02	0,02	0,96	0	0,046
3	100000	0,06	0,02	0,89	0,02	0	0	0	0	0,01	0	0,91	0	0,09	0,498
3	71344	0	0,83	0	0	0,13	0	0,01	0,03	0	0,01	0,72	0,03	0,24	0,046
3	25276	0	0,28	0	0	0,7	0	0	0	0,02	0,28	0	0,02	0,7	0,046
3	55408	0	0	0,04	0	0,94	0	0,02	0	0	0,98	0	0,02	0	0,044
3	4170	0	0	0,04	0	0,87	0	0	0	0,09	0,87	0	0,13	0	0,03
3	7195	0	0,07	0,03	0	0,87	0	0,02	0	0,01	0,11	0	0,86	0,03	0,038
3	9224	0	0	0,04	0	0,93	0,02	0	0,01	0	0	0,04	0,02	0,94	0
3	77048	0	0,01	0	0,05	0,94	0	0	0	0	0,05	0	0,01	0,94	0,034
3	21564	0	0,04	0,04	0	0,9	0,02	0	0	0	0,44	0,06	0,07	0,43	0,032
3	20229	0	0,12	0	0	0,77	0,07	0	0,04	0	0	0,08	0,85	0,07	0,048
3	8961	0	0	0,04	0	0,53	0	0	0	0,43	0	0,51	0,06	0,43	0,006
3	100000	0,37	0	0,63	0	0	0	0	0	0	0	0,63	0,37	0	0,694
3	11965	0	0,08	0,01	0,05	0,86	0	0	0	0	0,01	0	0,94	0,05	0,048

3	26587	0	0,02	0,04	0	0,78	0	0	0,16	0	0,22	0,04	0	0,74	0,016
3	24043	0	0	0	0	0	0,14	0,11	0,43	0,32	0	0,46	0,43	0,11	0,046
3	4704	0,04	0	0,02	0	0,91	0	0	0	0,03	0,91	0,05	0,04	0	0,046
3	43810	0,01	0,2	0	0	0	0	0	0,79	0	0	0,03	0,36	0,61	0,044
3	10176	0	0,08	0	0,05	0,71	0	0	0,16	0	0	0,05	0,71	0,24	0,048
3	23562	0	0,01	0,04	0	0,91	0	0	0,04	0	0,04	0,06	0,89	0,01	0,028
3	29198	0	0,09	0,03	0	0,83	0	0,05	0	0	0,18	0,07	0,09	0,66	0,042
3	36693	0	0	0	0,26	0,61	0,11	0	0	0,02	0,86	0,02	0,12	0	0,046
3	42122	0	0,22	0,04	0	0,04	0	0	0,7	0	0	0,04	0,26	0,7	0,044
3	9150	0,01	0,07	0	0	0,92	0	0	0	0	0,93	0	0,07	0	0,044
3	3329	0	0	0	0,03	0,9	0	0,06	0	0,01	0	0,03	0,9	0,07	0,046
3	16372	0	0	0,04	0	0,96	0	0	0	0	0,04	0,62	0,34	0	0,02
3	30579	0	0,09	0	0,05	0,84	0,01	0	0	0,01	0,05	0,1	0	0,85	0,048
3	64570	0,02	0	0,04	0	0	0,92	0,01	0,01	0	0,02	0,02	0	0,96	0,034
3	48918	0	0	0,02	0,05	0,81	0	0,07	0	0,05	0,39	0,06	0,33	0,22	0,046
3	7838	0,05	0	0,04	0	0,01	0,01	0	0,25	0,64	0,25	0,05	0,01	0,69	0,048
3	37023	0,03	0,03	0,02	0	0,74	0,15	0	0	0,03	0,02	0,18	0,77	0,03	0,048
3	22024	0	0,01	0,04	0	0,69	0	0,01	0	0,25	0,02	0,05	0,12	0,81	0,014
3	65142	0	0	0	0,11	0,89	0	0	0	0	0	0	1	0	0,04
3	2560	0	0	0,04	0	0,91	0,01	0	0	0,04	0	0,05	0,04	0,91	0,002
3	16273	0,01	0	0,04	0	0,57	0	0	0,38	0	0	0,42	0,58	0	0,02
3	3158	0,01	0	0,01	0	0,02	0	0,09	0,87	0	0,09	0,01	0,88	0,02	0,042
3	5620	0	0	0,04	0,01	0,03	0,03	0	0	0,89	0,07	0,04	0,03	0,86	0,02
3	48104	0	0	0,04	0,01	0,83	0,02	0	0,1	0	0	0,11	0,09	0,8	0,01
3	9959	0	0,02	0,01	0,04	0	0,89	0,04	0	0	0,03	0,88	0,05	0,04	0,046
3	10250	0,03	0	0,04	0	0,93	0	0	0	0	0	0,58	0,42	0	0,042
3	19120	0	0	0,04	0	0,01	0,14	0,01	0,8	0	0	0,05	0,8	0,15	0,03
3	17698	0	0	0	0,22	0,78	0	0	0	0	0,78	0,22	0	0	0,046
3	2332	0	0	0,04	0	0,96	0	0	0	0	0,96	0,04	0	0	0,038
3	5030	0	0,02	0,04	0,01	0,63	0	0	0	0,3	0	0,04	0,65	0,31	0,024
3	3515	0	0,01	0,04	0,05	0,01	0,87	0,01	0	0,01	0,06	0,07	0,01	0,86	0,044
3	8299	0	0,01	0,01	0	0,15	0	0,06	0,77	0	0	0,98	0	0,02	0,048
3	16003	0,02	0	0	0,04	0,94	0	0	0	0	0	0,98	0,02	0	0,046

3	7750	0,04	0	0,02	0	0,74	0	0,01	0	0,19	0,74	0,06	0,13	0,07	0,044
3	64757	0	0	0,04	0	0,03	0,12	0,01	0,57	0,23	0,6	0,02	0,03	0,35	0,03
3	74911	0,01	0	0,04	0,04	0	0	0	0,91	0	0	0,04	0,03	0,93	0,048
3	16772	0,02	0	0,04	0	0,03	0,03	0	0	0,88	0	0	0,03	0,97	0,018
3	32877	0,04	0	0,04	0	0	0	0	0	0,92	0	0,04	0,1	0,86	0,038
3	33778	0,04	0	0	0,03	0,9	0,03	0	0	0	0,04	0	0,96	0	0,046
3	52230	0,04	0	0,04	0,01	0,91	0	0	0	0	0,13	0,01	0,82	0,04	0,048
3	100000	0	0	0,53	0	0	0	0	0	0,47	0	0,53	0	0,47	0,152
3	11272	0	0	0,04	0,03	0,9	0,02	0	0	0,01	0,01	0,02	0,93	0,04	0,034
3	17187	0	0,01	0	0,07	0,92	0	0	0	0	0	0,01	0,05	0,94	0,048
3	11128	0	0,15	0,03	0	0	0,72	0,04	0,06	0	0	0,72	0,04	0,24	0,046
3	55736	0,06	0	0	0	0,07	0,87	0	0	0	0,04	0,07	0,89	0	0,046
3,25	11715	0	0,05	0,04	0	0	0,54	0	0	0,37	0,51	0,12	0	0,37	0,046
3,25	15867	0	0,01	0,04	0,04	0,23	0,03	0	0,62	0,03	0,91	0	0	0,09	0,038
3,25	72591	0	0,08	0,03	0	0,46	0,37	0	0,02	0,04	0,5	0,02	0,4	0,08	0,048
3,25	20334	0	0,12	0	0	0,01	0	0	0,87	0	0	0,07	0,92	0,01	0,042
3,25	15264	0,04	0,01	0	0,01	0,37	0	0	0,57	0	0,06	0,57	0	0,37	0,044
3,25	74863	0	0,33	0	0	0,67	0	0	0	0	0,03	0	0,32	0,65	0,042
3,25	30151	0	0,12	0,03	0,02	0,81	0	0,02	0	0	0,14	0,02	0,03	0,81	0,04
3,25	4912	0	0	0,04	0	0	0,04	0,1	0,79	0,03	0	0,7	0,29	0,01	0,048
3,25	13238	0	0	0	0,15	0,82	0	0,01	0,02	0	0,82	0,08	0,09	0,01	0,04
3,25	3143	0,03	0,04	0,04	0	0	0,89	0	0	0	0	0,04	0,77	0,19	0,048
3,25	14831	0	0,11	0	0	0,89	0	0	0	0	0,09	0,02	0,89	0	0,032
3,25	7728	0	0	0	0,05	0	0	0	0,94	0,01	0	0	0	1	0,044
3,25	4744	0	0	0,04	0	0	0,18	0	0,42	0,36	0,42	0,04	0	0,54	0,022
3,25	22056	0	0,28	0,04	0,01	0	0	0,01	0,66	0	0,01	0,32	0	0,67	0,044
3,25	31351	0	0,95	0	0,05	0	0	0	0	0	0,01	0,03	0,01	0,95	0,038
3,25	16934	0,06	0	0	0	0,29	0,65	0	0	0	0,98	0	0	0,02	0,046
3,25	15223	0	0,01	0,04	0	0,91	0	0,04	0	0	0,44	0,03	0,53	0	0,048
3,25	9405	0	0	0	0,06	0	0	0	0,94	0	0	0	0	1	0,042
3,25	42907	0	0,1	0,04	0	0,85	0,01	0	0	0	0	0,1	0,05	0,85	0,01
3,25	12123	0,07	0	0	0	0	0	0	0,93	0	0,09	0	0	0,91	0,048
3,25	16876	0	0	0	0,06	0,94	0	0	0	0	0	0,06	0	0,94	0,044

3,25	73931	0,06	0	0	0	0,93	0	0,01	0	0	0,11	0	0,89	0	0,048
3,25	56609	0,01	0	0,04	0,01	0	0,9	0	0,04	0	0,9	0,01	0,04	0,05	0,048
3,25	49774	0	0,51	0,01	0	0	0,14	0	0,34	0	0	0	0,14	0,86	0,048
3,25	15991	0	0	0	0,08	0,92	0	0	0	0	0,08	0,92	0	0	0,048
3,25	4798	0	0	0,04	0,02	0	0,01	0	0,17	0,76	0,04	0,04	0,18	0,74	0,036
3,25	14731	0	0,03	0,03	0,06	0,69	0	0	0,19	0	0	0,06	0,88	0,06	0,04
3,25	62066	0	0,05	0	0,02	0	0,02	0,04	0,87	0	0,04	0,87	0,02	0,07	0,042
3,25	44230	0,01	0	0,04	0	0,39	0,38	0,03	0	0,15	0	0,31	0,3	0,39	0,042
3,25	89567	0	0	0,04	0	0	0	0	0,96	0	0,7	0,15	0,12	0,03	0,038
3,25	69236	0,02	0	0,02	0,06	0,8	0	0	0,1	0	0,16	0,02	0,74	0,08	0,042
3,25	2387	0	0	0,04	0	0	0,49	0	0,47	0	0	0,04	0	0,96	0,004
3,25	25490	0,01	0,11	0	0	0	0,88	0	0	0	0,02	0	0,97	0,01	0,048
3,25	35132	0	0	0,01	0,03	0	0,86	0,05	0,01	0,04	0,07	0,01	0,86	0,06	0,046
3,25	39947	0	0	0	0,17	0	0,7	0,01	0	0,12	0,82	0	0,17	0,01	0,042
3,25	13667	0	0	0	0,12	0	0,82	0	0,06	0	0	0,44	0,48	0,08	0,044
3,25	6121	0	0,03	0,04	0	0,83	0	0	0,03	0,07	0,04	0,82	0	0,14	0,008
3,25	51931	0	0	0,04	0	0	0	0	0,95	0,01	0,81	0,19	0	0	0,03
3,25	16388	0	0,06	0,01	0	0,93	0	0	0	0	0,93	0	0,06	0,01	0,046
3,25	68593	0	0,19	0	0	0	0,78	0	0	0,03	0,19	0	0,3	0,51	0,048
3,25	100000	0	0	0,89	0,01	0	0	0	0	0,1	0	0,66	0	0,34	0,366
3,25	79114	0,02	0	0,04	0	0,94	0	0	0	0	0	0,02	0,02	0,96	0,022
3,25	100000	0	0,04	0,93	0	0,01	0	0,02	0	0	0,05	0,95	0	0	0,73
3,25	91584	0	0,14	0	0	0	0	0	0,86	0	0	0,01	0,86	0,13	0,048
3,25	1437	0,03	0,03	0,04	0,01	0,02	0	0	0,87	0	0,4	0,03	0,03	0,54	0,044
3,25	35067	0,04	0,27	0,04	0	0,61	0	0	0,04	0	0,04	0,04	0	0,92	0,044
3,25	56783	0	0,85	0	0	0,09	0	0	0,06	0	0	0,02	0,09	0,89	0,034
3,25	49498	0	0	0,04	0	0,02	0,37	0	0	0,57	0,37	0,06	0	0,57	0,016
3,25	33291	0,05	0	0,01	0	0,93	0	0,01	0	0	0,98	0	0	0,02	0,048
3,25	84970	0,05	0	0	0,03	0,69	0,2	0	0	0,03	0,79	0,15	0	0,06	0,048
3,25	20056	0	0,33	0	0	0,67	0	0	0	0	0,03	0,04	0,3	0,63	0,046
3,25	5291	0	0,01	0,04	0	0,91	0	0	0	0,04	0	0,04	0,93	0,03	0,02
3,25	4095	0	0	0,04	0,02	0	0,01	0	0	0,93	0	0,07	0	0,93	0,024
3,25	56042	0	0,19	0	0	0,39	0,22	0,01	0,19	0	0	0,19	0,42	0,39	0,044

3,25	11667	0	0,01	0,04	0	0	0,94	0	0	0,01	0	0,95	0	0,05	0,034
3,25	17568	0	0,02	0	0,06	0,91	0,01	0	0	0	0,07	0,89	0,04	0	0,044
3,25	34242	0	0,01	0,04	0,07	0,88	0	0	0	0	0,03	0,02	0,95	0	0,048
3,25	54089	0	0,01	0	0,26	0,72	0	0	0	0,01	0,91	0	0,08	0,01	0,046
3,25	7949	0,07	0	0	0	0	0	0	0,92	0,01	0,14	0	0,86	0	0,044
3,25	28098	0	0	0,04	0	0,94	0,02	0	0	0	0	0,04	0,95	0,01	0,022
3,25	40523	0,03	0,04	0,04	0	0,89	0	0	0	0	0,04	0,04	0,92	0	0,048
3,25	11719	0	0	0,04	0	0	0,58	0	0	0,38	0,26	0	0,16	0,58	0,016
3,25	74431	0	0,03	0	0,06	0,73	0	0	0,18	0	0,03	0	0,24	0,73	0,046
3,25	41679	0	0	0,04	0	0,96	0	0	0	0	0	0,04	0,96	0	0,02
3,25	32371	0	0,13	0,02	0	0,44	0,33	0	0,08	0	0,14	0,02	0,84	0	0,048
3,25	95488	0	0	0,04	0	0,87	0	0	0,09	0	0	0,01	0,13	0,86	0,004
3,25	26189	0	0	0,04	0	0,75	0	0	0,04	0,17	0	0,05	0,75	0,2	0,028
3,25	65091	0	0,15	0	0	0,68	0,01	0	0,16	0	0,34	0,04	0,28	0,34	0,044
3,25	16921	0	0,01	0,04	0	0,93	0	0	0	0,02	0,06	0	0,01	0,93	0,002
3,25	19022	0	0,09	0	0,06	0,85	0	0	0	0	0,06	0	0,09	0,85	0,048
3,25	20774	0,01	0,02	0,04	0,01	0	0	0	0,92	0	0,02	0,03	0	0,95	0,02
3,25	2211	0	0,07	0,01	0,01	0	0,8	0,04	0	0,07	0,18	0,01	0,8	0,01	0,048
3,25	100000	0	0	0,99	0	0	0	0,01	0	0	0	1	0	0	0,704
3,25	70810	0	0	0	0,05	0,95	0	0	0	0	0,08	0	0	0,92	0,036
3,25	20932	0	0	0,04	0	0,01	0	0	0,91	0,04	0,01	0,08	0	0,91	0,002
3,25	7324	0	0	0	0,07	0,9	0	0	0	0,03	0,02	0,03	0,04	0,91	0,048
3,25	45465	0,05	0	0,02	0	0	0,69	0	0	0,24	0	0,69	0,02	0,29	0,048
3,25	60310	0	0	0,04	0	0,03	0,64	0	0,26	0,03	0	0,11	0,21	0,68	0,014
3,25	23154	0,02	0	0,03	0	0	0,91	0,04	0	0	0,04	0,03	0,93	0	0,046
3,25	31485	0,05	0	0	0	0,5	0,12	0	0,18	0,15	0,12	0	0,88	0	0,042
3,25	87352	0,03	0,04	0	0,02	0,81	0	0,01	0,09	0	0,04	0	0,82	0,14	0,048
3,25	28066	0,06	0	0	0	0,93	0,01	0	0	0	0,06	0,19	0	0,75	0,042
3,25	31590	0	0,05	0,04	0	0,91	0	0	0	0	0,07	0	0,93	0	0,038
3,25	95314	0	0	0	0,13	0,87	0	0	0	0	0,16	0	0,57	0,27	0,046
3,25	53805	0	0,01	0,04	0,01	0,92	0	0	0	0,02	0,03	0,92	0,05	0	0,026
3,25	3108	0,01	0	0,02	0,09	0,75	0	0	0,13	0	0,01	0,15	0,84	0	0,048
3,25	100000	0	0	1	0	0	0	0	0	0	0	1	0	0	0,64

3,25	29467	0	0	0	0,1	0	0,56	0,04	0,3	0	0,57	0,32	0	0,11	0,046
3,25	100000	0,02	0	0,98	0	0	0	0	0	0	0	0,97	0,02	0,01	0,526
3,25	3944	0	0,88	0	0	0	0	0	0,1	0,02	0	0	0,1	0,9	0,048
3,25	16953	0	0,14	0	0	0,84	0	0	0,02	0	0	0,14	0,86	0	0,044
3,25	13765	0	0,07	0,04	0	0	0,89	0	0	0	0,11	0,85	0,04	0	0,036
3,25	39727	0	0	0,04	0	0,37	0,53	0,06	0	0	0,39	0,04	0,04	0,53	0,044
3,25	27819	0	0,12	0	0	0,72	0	0	0,16	0	0,04	0	0,96	0	0,042
3,25	4994	0	0	0,04	0	0,51	0,02	0	0,21	0,22	0,74	0,22	0	0,04	0,036
3,25	11026	0,01	0	0,04	0	0	0,01	0	0,94	0	0,08	0,05	0,87	0	0,038
3,25	33256	0,05	0	0	0,02	0,92	0	0	0,01	0	0,97	0,01	0,02	0	0,048
3,25	22347	0	0	0,04	0	0	0,91	0,05	0	0	0	0,02	0,97	0,01	0,034
3,25	5116	0,02	0,04	0,03	0	0,73	0,04	0	0,13	0,01	0,44	0,41	0,06	0,09	0,046
3,25	18099	0	0	0,03	0,09	0,03	0	0	0,85	0	0	0,06	0,85	0,09	0,046
3,5	100000	0	0	0,97	0	0	0	0	0,03	0	0	0,97	0,03	0	0,498
3,5	5560	0,06	0	0	0	0	0,01	0	0,93	0	0	0	0,06	0,94	0,048
3,5	71367	0	0	0	0,06	0,94	0	0	0	0	0	0	0,17	0,83	0,042
3,5	3398	0,03	0	0,04	0	0,85	0,08	0	0	0	0,85	0,12	0,02	0,01	0,038
3,5	27670	0	0,06	0,01	0,03	0,89	0	0	0,01	0	0,9	0,04	0	0,06	0,044
3,5	13760	0,03	0	0,02	0,04	0,04	0	0,01	0,86	0	0,89	0,06	0,01	0,04	0,048
3,5	58962	0,01	0	0	0,21	0,76	0	0,01	0,01	0	0,79	0,19	0,02	0	0,044
3,5	22399	0,01	0,12	0	0,01	0,85	0	0	0	0,01	0,01	0	0,99	0	0,048
3,5	23466	0	0	0,04	0	0	0,95	0	0,01	0	0,04	0	0,01	0,95	0,008
3,5	6042	0	0,04	0,04	0	0	0,9	0,02	0	0	0	0,05	0,9	0,05	0,048
3,5	15174	0	0,01	0,04	0,06	0,8	0,04	0	0	0,05	0,06	0,07	0,85	0,02	0,046
3,5	37450	0,01	0	0,04	0	0	0	0,03	0,78	0,14	0,05	0,81	0	0,14	0,044
3,5	12234	0	0	0,02	0,07	0,52	0	0,01	0,16	0,22	0,07	0,01	0,18	0,74	0,048
3,5	7371	0	0	0,02	0,09	0,65	0,02	0	0,22	0	0,02	0	0,87	0,11	0,048
3,5	8441	0	0,07	0	0,07	0,79	0	0	0,07	0	0,1	0	0,11	0,79	0,046
3,5	1706	0	0,01	0	0,01	0,89	0,01	0,06	0,01	0,01	0,02	0,92	0,03	0,03	0,046
3,5	21414	0,05	0	0,01	0	0,39	0	0,01	0,54	0	0	0,07	0,54	0,39	0,04
3,5	35789	0	0,01	0,04	0	0	0,92	0	0,01	0,02	0,71	0,01	0,26	0,02	0,048
3,5	85694	0	0,08	0,02	0	0,64	0,26	0	0	0	0,73	0,01	0,26	0	0,046
3,5	22796	0	0,24	0,02	0	0	0,47	0	0,26	0,01	0,1	0,27	0,05	0,58	0,046

3,5	17200	0	0	0,04	0,11	0	0	0	0	0,85	0,06	0,06	0,88	0	0,044
3,5	51745	0,04	0,04	0	0	0,9	0,01	0	0,01	0	0,05	0,91	0,04	0	0,038
3,5	13224	0	0	0,01	0	0	0,03	0,06	0,88	0,02	0,03	0,06	0,06	0,85	0,046
3,5	61548	0	0,25	0,02	0	0,6	0,03	0	0,1	0	0,27	0	0,03	0,7	0,042
3,5	58703	0	0	0	0,2	0,79	0,01	0	0	0	0,79	0,01	0,2	0	0,042
3,5	30612	0	0,77	0	0	0	0,18	0	0,01	0,04	0	0,21	0,04	0,75	0,046
3,5	38014	0	0	0,04	0	0,86	0,1	0	0	0	0,04	0,86	0,1	0	0,026
3,5	92255	0	0	0,04	0	0,93	0,03	0	0	0	0,02	0,93	0,01	0,04	0,008
3,5	58468	0	0,01	0,04	0	0,93	0,02	0	0	0	0,04	0,83	0,02	0,11	0,014
3,5	100000	0	0	1	0	0	0	0	0	0	0	1	0	0	0,598
3,5	72877	0	0	0,04	0	0,37	0,58	0	0	0,01	0,63	0	0	0,37	0,02
3,5	35896	0	0,06	0,04	0,01	0	0,88	0	0,01	0	0,08	0,88	0	0,04	0,04
3,5	7237	0,02	0,44	0	0,01	0,21	0	0	0,32	0	0,02	0,55	0,01	0,42	0,048
3,5	3346	0,01	0	0,02	0	0,45	0	0,06	0,46	0	0	0,94	0,06	0	0,048
3,5	67509	0	0,13	0	0	0,69	0,18	0	0	0	0,28	0,7	0	0,02	0,036
3,5	6170	0	0,01	0,04	0	0,01	0	0	0,48	0,46	0	0,54	0,46	0	0,026
3,5	36013	0	0,1	0	0,02	0,88	0	0	0	0	0	0	1	0	0,038
3,5	69006	0	0,01	0,04	0	0,88	0	0,06	0	0,01	0	0,92	0,08	0	0,048
3,5	67824	0,05	0	0,01	0	0	0,94	0	0	0	0,97	0	0,01	0,02	0,038
3,5	13984	0	0,12	0	0	0,88	0	0	0	0	0	0	1	0	0,048
3,5	9551	0,04	0	0	0,01	0	0	0	0,04	0,91	0,01	0,04	0	0,95	0,044
3,5	42241	0	0,94	0	0,01	0	0	0,02	0	0,03	0	0	0,02	0,98	0,048
3,5	95786	0	0	0,04	0	0,1	0	0	0,86	0	0,01	0	0,86	0,13	0,016
3,5	54171	0,06	0	0	0	0,81	0,13	0	0	0	0	0,81	0,08	0,11	0,048
3,5	25151	0	0,2	0,01	0	0,03	0	0,02	0,71	0,03	0,2	0,03	0,02	0,75	0,042
3,5	36627	0,02	0,06	0	0	0,85	0	0	0,05	0,02	0,92	0,07	0,01	0	0,048
3,5	15792	0	0,95	0	0,05	0	0	0	0	0	0	0,95	0	0,05	0,046
3,5	22376	0	0	0,04	0	0	0	0,04	0	0,92	0	0,02	0,93	0,05	0,042
3,5	19986	0	0	0	0,05	0,93	0	0,01	0,01	0	0,01	0,99	0	0	0,048
3,5	34930	0,03	0,44	0,04	0	0,49	0	0	0	0	0,05	0,02	0	0,93	0,042
3,5	100000	0	0	0,99	0	0,01	0	0	0	0	0	0,99	0	0,01	0,616
3,5	23496	0	0,13	0	0,02	0,16	0,69	0	0	0	0,13	0,72	0,15	0	0,048
3,5	57710	0	0,08	0	0	0,73	0	0,02	0	0,17	0,67	0,06	0,1	0,17	0,042

3,5	10205	0	0,01	0,04	0,01	0,04	0,19	0	0	0,71	0,01	0,25	0	0,74	0,022
3,5	51679	0,03	0,04	0	0,02	0,42	0,01	0	0,48	0	0,48	0,01	0,03	0,48	0,048
3,5	16165	0,05	0,15	0	0	0,79	0	0,01	0	0	0	0,15	0,05	0,8	0,048
3,5	80447	0	0	0,04	0,04	0	0,7	0	0,09	0,13	0,1	0,04	0,03	0,83	0,04
3,5	100000	0	0	0,99	0	0	0	0	0,01	0	0,01	0,99	0	0	0,698
3,5	48075	0	0	0,04	0	0	0,95	0	0	0,01	0	0,95	0	0,05	0,016
3,5	11769	0,04	0	0,01	0	0,95	0	0	0	0	0,04	0	0,95	0,01	0,048
3,5	46895	0	0	0,04	0	0,94	0	0,01	0,01	0	0	0,98	0,01	0,01	0,016
3,5	88746	0	0	0,03	0	0,48	0	0,16	0,23	0,1	0,82	0,03	0,15	0	0,048
3,5	10605	0	0	0	0	0	0	0,11	0,03	0,86	0	0,72	0,14	0,14	0,046
3,5	78986	0,01	0	0,02	0	0,63	0	0,09	0,25	0	0	0,02	0,98	0	0,04
3,5	47498	0	0	0	0,13	0	0,87	0	0	0	0	0,87	0,13	0	0,042
3,5	5584	0,05	0	0,03	0	0,02	0,18	0	0,72	0	0,1	0,01	0,82	0,07	0,046
3,5	78781	0,01	0,12	0	0,01	0,46	0	0	0,4	0	0,01	0,52	0,41	0,06	0,048
3,5	25902	0,06	0	0	0	0,78	0,15	0	0,01	0	0,15	0,06	0,78	0,01	0,046
3,5	42617	0	0	0,04	0	0,89	0	0,07	0	0	0,89	0,04	0,07	0	0,042
3,5	41601	0,05	0	0	0,01	0,03	0,83	0	0,08	0	0,15	0,85	0	0	0,046
3,5	28303	0,05	0	0	0,01	0,86	0	0,01	0,07	0	0,05	0,09	0,3	0,56	0,046
3,5	78819	0	0	0,04	0	0	0,96	0	0	0	0	0,17	0	0,83	0,008
3,5	9505	0	0	0	0	0,9	0,01	0,08	0,01	0	0	0,8	0,09	0,11	0,044
3,5	26646	0	0	0,04	0	0	0	0	0,07	0,89	0,07	0,06	0	0,87	0,008
3,5	14125	0	0	0,04	0	0,73	0	0	0,23	0	0,03	0,23	0	0,74	0,004
3,5	49375	0,01	0,01	0,04	0,02	0,26	0,66	0	0	0	0,85	0,1	0,05	0	0,04
3,5	30303	0	0	0,04	0,01	0,78	0	0	0,17	0	0,15	0	0,04	0,81	0,01
3,5	73405	0	0	0,04	0,02	0,04	0,48	0	0,36	0,06	0,36	0,06	0,54	0,04	0,026
3,5	48157	0	0	0,04	0	0,69	0	0	0,27	0	0,27	0,07	0	0,66	0,006
3,5	9368	0	0,1	0,04	0	0	0,27	0,01	0	0,58	0,01	0,31	0,09	0,59	0,04
3,5	32483	0,06	0,01	0	0	0,86	0	0	0,06	0,01	0,93	0,06	0	0,01	0,048
3,5	17864	0,05	0	0,04	0	0,91	0	0	0	0	0,09	0,8	0	0,11	0,044
3,5	100000	0	0	0,99	0	0	0	0	0	0,01	0	1	0	0	0,688
3,5	100000	0	0	0,32	0	0,08	0,57	0	0,01	0,02	0	0,46	0,07	0,47	0,094
3,5	19748	0,05	0	0,03	0	0,05	0	0	0,87	0	0	0,95	0,05	0	0,048
3,5	23204	0	0	0,04	0	0,9	0	0	0	0,06	0,42	0,07	0,03	0,48	0,014

3,5	78319	0	0	0,01	0,06	0,93	0	0	0	0	0,02	0,98	0	0	0,044
3,5	28720	0	0,27	0	0	0,66	0	0	0,07	0	0	0,08	0,26	0,66	0,036
3,5	52052	0	0	0,04	0	0,94	0	0	0	0,02	0,94	0,04	0,02	0	0,046
3,5	100000	0	0	0,99	0,01	0	0	0	0	0	0	0,99	0	0,01	0,6
3,5	23670	0	0,03	0	0,1	0,82	0	0	0	0,05	0,82	0	0,03	0,15	0,042
3,5	10503	0	0,05	0	0,04	0,88	0,01	0,01	0,01	0	0,06	0	0,01	0,93	0,044
3,5	26904	0	0	0	0,12	0,88	0	0	0	0	0	0	0,99	0,01	0,042
3,5	3104	0	0	0,04	0	0,96	0	0	0	0	0,96	0,04	0	0	0,036
3,5	28724	0	0,96	0	0,04	0	0	0	0	0	0	0,04	0,01	0,95	0,046
3,5	26961	0	0,12	0	0	0	0	0	0,88	0	0,26	0,09	0,61	0,04	0,048
3,5	31868	0	0,01	0,04	0	0,14	0,1	0	0,45	0,26	0	0,04	0	0,96	0
3,5	30247	0,02	0	0,04	0	0,94	0	0	0	0	0	0,04	0,96	0	0,044
3,5	19289	0	0	0,04	0	0,94	0	0	0,02	0	0,04	0	0,92	0,04	0,024
3,5	57134	0	0,95	0	0,01	0	0	0	0	0,04	0	0	0,05	0,95	0,048
3,75	18369	0	0,17	0	0	0,31	0,28	0,01	0,23	0	0,02	0,33	0,5	0,15	0,046
3,75	16280	0,03	0	0	0,18	0	0,79	0	0	0	1	0	0	0	0,048
3,75	15329	0	0	0,03	0,04	0	0,07	0,05	0,72	0,09	0	0,04	0,8	0,16	0,042
3,75	22513	0,03	0,97	0	0	0	0	0	0	0	0,01	0	0,01	0,98	0,044
3,75	65960	0	0,05	0,01	0	0,89	0	0,05	0	0	0,1	0,01	0	0,89	0,048
3,75	67957	0	0	0	0,24	0	0,76	0	0	0	0,68	0,24	0,08	0	0,048
3,75	41676	0,01	0,12	0	0	0,85	0	0	0,02	0	0,02	0,01	0,81	0,16	0,042
3,75	14644	0,02	0,17	0	0	0,79	0	0,02	0	0	0,02	0,96	0,02	0	0,038
3,75	18636	0,01	0,02	0	0	0	0,01	0,19	0,02	0,75	0,97	0	0,03	0	0,048
3,75	98258	0	0,03	0	0,06	0,07	0	0	0,82	0,02	0,07	0	0,11	0,82	0,038
3,75	6787	0	0,14	0	0,02	0	0,82	0	0	0,02	0,02	0	0,96	0,02	0,046
3,75	17905	0,05	0,03	0	0	0,88	0,01	0	0,03	0	0,77	0,01	0	0,22	0,046
3,75	6539	0	0,06	0	0,05	0	0,88	0,01	0	0	0	0,02	0,09	0,89	0,044
3,75	16594	0	0	0	0,06	0,93	0	0	0,01	0	0	0	0,06	0,94	0,048
3,75	30563	0,02	0	0,04	0	0,91	0	0	0,03	0	0,62	0,04	0,04	0,3	0,042
3,75	48293	0	0,1	0	0	0,9	0	0	0	0	0,02	0,09	0,89	0	0,042
3,75	52612	0	0,06	0,04	0	0,9	0	0	0	0	0,01	0,1	0,89	0	0,038
3,75	21122	0,01	0	0,04	0,01	0	0	0	0,93	0,01	0,01	0,57	0,37	0,05	0,024
3,75	39158	0	0	0	0,18	0,82	0	0	0	0	0,85	0	0,15	0	0,048

3,75	47240	0	0,14	0,04	0	0	0,04	0	0	0,78	0,18	0,04	0	0,78	0,042
3,75	16927	0	0,05	0	0,11	0,84	0	0	0	0	0,84	0	0,11	0,05	0,048
3,75	28917	0	0,01	0	0,06	0,68	0	0	0,25	0	0,06	0,94	0	0	0,046
3,75	100000	0	0	0,49	0	0	0	0,47	0	0,04	0	0,73	0,27	0	0,484
3,75	44566	0	0	0	0,06	0,94	0	0	0	0	0,05	0,02	0,01	0,92	0,042
3,75	12095	0,02	0	0	0,03	0,9	0,03	0	0,02	0	0,04	0,9	0,02	0,04	0,044
3,75	13421	0	0	0	0,26	0,74	0	0	0	0	1	0	0	0	0,046
3,75	100000	0	0	0,95	0,02	0	0	0	0,01	0,02	0	0,97	0,03	0	0,52
3,75	19415	0	0	0,04	0	0,94	0	0	0	0,02	0	0,04	0,94	0,02	0,02
3,75	36618	0	0,65	0,04	0	0	0,29	0	0,02	0	0	0	0,06	0,94	0,042
3,75	38604	0	0,08	0,04	0	0	0	0,01	0,87	0	0,01	0,08	0,04	0,87	0,018
3,75	66492	0,03	0	0,02	0,01	0,78	0	0	0,16	0	0,81	0,18	0	0,01	0,048
3,75	55105	0,03	0	0,04	0	0,92	0	0	0	0,01	0,04	0	0,96	0	0,04
3,75	19681	0	0,02	0,04	0,03	0	0	0,02	0	0,89	0	0,06	0,03	0,91	0,042
3,75	45951	0	0	0	0,08	0,92	0	0	0	0	0,14	0,86	0	0	0,044
3,75	23497	0	0,03	0	0,11	0,39	0,46	0	0,01	0	0	0,12	0,88	0	0,048
3,75	7096	0,01	0	0	0,17	0,81	0	0	0	0,01	0,81	0	0,17	0,02	0,042
3,75	24486	0,04	0	0,04	0	0,92	0	0	0	0	0,04	0,04	0	0,92	0,048
3,75	93194	0	0	0,04	0	0,7	0	0,01	0,03	0,22	0,73	0	0	0,27	0,038
3,75	16616	0	0	0,04	0	0,83	0,13	0	0	0	0,13	0,04	0,83	0	0,03
3,75	17542	0	0,08	0	0	0,74	0,1	0,06	0,02	0	0,12	0,74	0,12	0,02	0,048
3,75	50461	0	0	0,04	0	0,91	0	0,01	0,03	0,01	0,04	0	0,95	0,01	0,034
3,75	28664	0	0	0	0,05	0,94	0	0	0,01	0	0,06	0	0	0,94	0,044
3,75	62198	0	0,01	0,04	0	0,94	0	0	0	0,01	0,94	0,01	0,04	0,01	0,048
3,75	7604	0	0,22	0,01	0	0,77	0	0	0	0	0	0,77	0,23	0	0,04
3,75	34864	0,05	0,02	0	0	0,93	0	0	0	0	0,05	0,95	0	0	0,044
3,75	9631	0	0	0,04	0,01	0,92	0,03	0	0	0	0,03	0	0,04	0,93	0,012
3,75	56325	0	0,03	0,01	0,09	0,87	0	0	0	0	0,9	0	0,01	0,09	0,042
3,75	43424	0	0	0,04	0,04	0,82	0	0	0,04	0,06	0,82	0,07	0,05	0,06	0,038
3,75	4416	0	0	0,04	0,02	0,21	0,72	0	0,01	0	0	0,22	0,06	0,72	0,014
3,75	64371	0,01	0	0,01	0,05	0,87	0	0	0,06	0	0	0,95	0,05	0	0,044
3,75	34471	0	0	0	0,13	0,84	0,03	0	0	0	0,03	0	0,97	0	0,046
3,75	100000	0	0,01	0,99	0	0	0	0	0	0	0	0,99	0,01	0	0,648

3,75	16173	0,05	0	0	0	0	0,95	0	0	0	0,02	0,03	0	0,95	0,038
3,75	16984	0	0	0	0,18	0,63	0,13	0,01	0	0,05	0,63	0,17	0,19	0,01	0,044
3,75	22731	0	0	0,04	0	0	0,29	0	0,67	0	0,69	0	0,02	0,29	0,03
3,75	35246	0	0,13	0,04	0	0	0	0,01	0	0,82	0,07	0	0,05	0,88	0,046
3,75	54479	0	0,23	0,04	0	0	0	0	0,73	0	0	0,27	0	0,73	0,014
3,75	20640	0	0	0,04	0	0	0,07	0	0,15	0,74	0	0,11	0,74	0,15	0,018
3,75	42102	0	0	0,04	0	0,74	0	0	0	0,22	0,01	0,77	0	0,22	0,01
3,75	84543	0,03	0	0	0,06	0,87	0	0	0	0,04	0,07	0	0,93	0	0,046
3,75	14590	0	0	0,04	0	0,04	0	0	0	0,92	0	0,04	0,92	0,04	0,028
3,75	42955	0	0,13	0,01	0,02	0,75	0,01	0	0,08	0	0,16	0,81	0,01	0,02	0,044
3,75	49351	0	0,11	0	0,01	0,23	0,01	0	0,64	0	0,36	0,01	0,63	0	0,048
3,75	11629	0,01	0,23	0,04	0	0,71	0	0,01	0	0	0,02	0,04	0	0,94	0,036
3,75	13013	0	0	0	0,06	0,93	0	0	0	0,01	0,06	0	0	0,94	0,04
3,75	4601	0	0	0,04	0	0,67	0,2	0,02	0,07	0	0,22	0,04	0,54	0,2	0,024
3,75	46033	0,03	0	0	0	0,94	0	0,03	0	0	0	0	0,97	0,03	0,046
3,75	97130	0,02	0	0	0,01	0,46	0,01	0,03	0,47	0	0,02	0,47	0,02	0,49	0,044
3,75	97807	0	0,96	0	0,04	0	0	0	0	0	0	0,1	0	0,9	0,038
3,75	8891	0	0,04	0,01	0,07	0,88	0	0	0	0	0	0,12	0,88	0	0,044
3,75	38992	0	0,04	0	0,06	0,2	0	0	0,7	0	0,01	0,85	0,04	0,1	0,048
3,75	12649	0	0	0,02	0,07	0,49	0,31	0,01	0,05	0,05	0,05	0,4	0,22	0,33	0,044
3,75	21349	0	0,16	0	0,02	0,67	0	0,01	0,14	0	0,02	0,68	0,16	0,14	0,046
3,75	8788	0	0	0,01	0,07	0,01	0	0	0,91	0	0,02	0,96	0,01	0,01	0,048
3,75	56061	0,02	0	0,04	0,01	0	0,59	0	0	0,34	0,02	0	0,01	0,97	0,04
3,75	14969	0	0,61	0,04	0	0	0,05	0,01	0,03	0,26	0,02	0,06	0	0,92	0,038
3,75	29171	0	0,17	0	0	0	0	0,01	0,82	0	0	0,01	0,62	0,37	0,044
3,75	60797	0,02	0	0,03	0,04	0,89	0	0,01	0	0,01	0,04	0,01	0,91	0,04	0,046
3,75	34144	0	0	0,03	0	0,88	0	0,09	0	0	0,03	0,1	0,87	0	0,048
3,75	94704	0	0	0	0,19	0,75	0	0	0,06	0	0,75	0,06	0	0,19	0,048
3,75	26981	0,01	0,14	0	0	0,85	0	0	0	0	0	0	0,67	0,33	0,048
3,75	28406	0,02	0	0,04	0	0,94	0	0	0	0	0,02	0	0,98	0	0,036
3,75	35567	0	0	0	0,07	0	0	0	0,93	0	0	0,93	0,02	0,05	0,048
3,75	83785	0,03	0	0,04	0	0,44	0	0	0,49	0	0	0	0	1	0,024
3,75	100000	0,01	0	0,97	0	0	0	0,02	0	0	0,02	0,97	0,01	0	0,586

3,75	6257	0,06	0	0	0	0,86	0,01	0	0,05	0,02	0,86	0	0,13	0,01	0,046
3,75	45458	0	0,12	0,01	0	0,86	0,01	0	0	0	0	0,02	0,98	0	0,044
3,75	9743	0	0	0	0,24	0,76	0	0	0	0	0,76	0,23	0,01	0	0,044
3,75	36794	0	0,02	0,01	0,07	0	0,59	0	0,31	0	0,01	0,02	0,96	0,01	0,038
3,75	3953	0	0,15	0	0	0,85	0	0	0	0	0,48	0	0,3	0,22	0,048
3,75	53757	0	0	0	0,1	0,06	0,83	0	0,01	0	0,05	0,83	0,07	0,05	0,042
3,75	12922	0	0	0,04	0	0	0,37	0	0,59	0	0,41	0	0	0,59	0,014
3,75	4079	0,03	0	0,04	0	0	0	0	0	0,93	0	0,07	0	0,93	0,036
3,75	100000	0	0	1	0	0	0	0	0	0	0	1	0	0	0,728
3,75	17029	0	0	0,04	0	0,23	0	0	0,73	0	0,01	0,47	0,03	0,49	0,002
3,75	23498	0	0,05	0	0,09	0,84	0	0	0,02	0	0,84	0,02	0,14	0	0,046
3,75	30621	0,03	0	0,04	0	0,92	0,01	0	0	0	0,03	0,03	0,44	0,5	0,044
3,75	19556	0	0	0,04	0	0	0,02	0	0,26	0,68	0	0,97	0,03	0	0,03
3,75	26663	0	0	0,04	0	0	0	0	0	0,96	0	0,01	0,03	0,96	0,008
3,75	14032	0,01	0	0,04	0	0,53	0,03	0	0,39	0	0,03	0,53	0,44	0	0,03
4	8820	0,01	0,05	0,04	0	0	0,33	0	0,57	0	0,01	0	0,44	0,55	0,046
4	15221	0	0	0,04	0	0	0,83	0	0,13	0	0	0,17	0,47	0,36	0,022
4	34729	0	0,02	0,04	0	0,63	0,03	0	0,28	0	0,01	0,72	0,27	0	0,032
4	59405	0,01	0,11	0	0	0,08	0,76	0	0,04	0	0,02	0,1	0,87	0,01	0,048
4	100000	0,01	0,35	0	0,64	0	0	0	0	0	0,39	0	0	0,61	0,556
4	24404	0,01	0,06	0,02	0	0,9	0	0,01	0	0	0,07	0,02	0,9	0,01	0,04
4	100000	0,01	0,72	0	0	0	0,03	0,22	0	0,02	0,02	0,8	0,17	0,01	0,39
4	61993	0,01	0	0,04	0	0	0	0	0,95	0	0	0,05	0,95	0	0,044
4	79679	0	0	0	0,06	0,93	0	0,01	0	0	0	0,93	0,07	0	0,048
4	78431	0,04	0	0,04	0	0,91	0	0	0,01	0	0,04	0,04	0,91	0,01	0,044
4	97480	0	0	0,04	0	0	0	0,01	0,95	0	0	0,04	0,09	0,87	0,008
4	4310	0	0,21	0	0,04	0,73	0	0,02	0	0	0	0	0,06	0,94	0,048
4	57008	0,05	0,01	0	0	0	0	0	0,94	0	0	0,95	0,05	0	0,046
4	67501	0,03	0,02	0,03	0	0,79	0,06	0	0	0,07	0,06	0,13	0,81	0	0,04
4	13554	0	0,02	0,04	0,05	0,81	0	0	0,08	0	0,85	0,01	0,09	0,05	0,048
4	29237	0,05	0	0,03	0	0,43	0,38	0	0,11	0	0,01	0,04	0,48	0,47	0,048
4	5882	0	0	0,04	0	0,02	0	0	0	0,94	0	0,04	0,13	0,83	0,014
4	21056	0,06	0	0	0	0	0,45	0	0,45	0,04	0,65	0,25	0,1	0	0,048

4	100000	0	0,06	0	0,32	0,57	0,01	0	0,04	0	0,32	0,11	0,57	0	0,182
4	13967	0	0	0,04	0	0	0	0,01	0,93	0,02	0,01	0,99	0	0	0,024
4	38012	0,01	0,05	0,03	0	0,86	0	0	0	0,05	0,34	0,54	0,09	0,03	0,038
4	9433	0	0	0,04	0,07	0,89	0	0	0	0	0,89	0,08	0	0,03	0,046
4	73731	0	0,16	0	0,02	0,47	0	0	0,34	0,01	0	0,19	0,34	0,47	0,046
4	97615	0	0	0	0,08	0,62	0,3	0	0	0	0,31	0,07	0	0,62	0,042
4	39772	0	0	0,04	0	0	0	0	0	0,96	0	0,04	0	0,96	0,018
4	31553	0	0	0	0,06	0,18	0	0	0,76	0	0,02	0,94	0	0,04	0,044
4	23297	0	0,97	0	0	0	0,01	0,01	0,01	0	0	0,96	0,01	0,03	0,046
4	16422	0	0,01	0,04	0	0	0,01	0	0,92	0,02	0,92	0	0,06	0,02	0,042
4	24400	0	0	0,04	0	0,16	0	0	0	0,8	0	0,04	0,01	0,95	0,008
4	55939	0	0,05	0,04	0,01	0,9	0	0	0	0	0,01	0,04	0,05	0,9	0,004
4	30338	0	0	0	0,25	0,69	0	0	0,03	0,03	0,86	0,04	0,08	0,02	0,04
4	100000	0,4	0	0,58	0	0	0	0	0,01	0,01	0,4	0,58	0	0,02	0,924
4	6303	0	0,04	0,04	0	0,48	0,03	0	0,41	0	0,04	0	0,36	0,6	0,03
4	29713	0	0	0,04	0	0,8	0	0	0,16	0	0	0,01	0,78	0,21	0,018
4	45586	0	0,04	0,03	0	0,84	0	0,06	0,03	0	0	0,05	0,92	0,03	0,036
4	33681	0	0	0,02	0,11	0,87	0	0	0	0	0,59	0,3	0,11	0	0,044
4	58435	0	0,11	0,02	0,04	0,78	0	0	0	0,05	0,13	0,09	0,11	0,67	0,048
4	40007	0	0	0,04	0	0	0	0	0,89	0,07	0,45	0,04	0	0,51	0,024
4	69210	0,06	0,02	0,01	0	0,91	0	0	0	0	0,06	0,01	0,93	0	0,048
4	100000	0	0	0,98	0,02	0	0	0	0	0	0	0,98	0,02	0	0,598
4	37426	0	0	0,04	0	0,65	0,23	0	0,08	0	0,04	0,31	0	0,65	0,006
4	55440	0	0,02	0	0,06	0	0	0	0,92	0	0	0,06	0	0,94	0,044
4	15863	0	0,21	0,03	0	0	0	0	0	0,76	0	0	0,21	0,79	0,044
4	30197	0,04	0	0	0	0	0,82	0,02	0,12	0	0,11	0,07	0	0,82	0,046
4	29110	0	0	0	0,01	0,78	0,01	0,08	0,12	0	0,05	0,79	0,12	0,04	0,042
4	32945	0	0	0,04	0	0	0	0,04	0,91	0,01	0	0,96	0	0,04	0,03
4	3729	0	0	0,04	0	0	0,36	0	0	0,6	0,17	0,4	0	0,43	0,014
4	100000	0,09	0	0,9	0	0	0	0	0	0,01	0,08	0,9	0,01	0,01	0,662
4	51174	0	0	0,04	0,07	0	0	0	0	0,89	0	0,11	0	0,89	0,048
4	31651	0	0	0,04	0	0,91	0	0	0,04	0,01	0,95	0	0,05	0	0,038
4	19166	0	0	0,03	0,12	0	0,07	0,01	0,77	0	0,38	0,62	0	0	0,044

4	16264	0	0	0,04	0,02	0,61	0	0,06	0	0,27	0,03	0,05	0,65	0,27	0,048
4	11411	0	0,24	0	0	0,76	0	0	0	0	0,19	0,06	0,24	0,51	0,042
4	22009	0	0	0	0,05	0,15	0,69	0,05	0,03	0,03	0,2	0,65	0,03	0,12	0,048
4	39436	0,06	0	0	0	0,12	0	0	0	0,82	0,41	0,06	0,53	0	0,046
4	11849	0	0,91	0,02	0,01	0	0,01	0	0,04	0,01	0,04	0,01	0,02	0,93	0,048
4	100000	0,01	0	0,59	0	0,06	0,34	0	0	0	0,35	0,26	0,35	0,04	0,352
4	49788	0	0,01	0,04	0	0,9	0,05	0	0	0	0,07	0,02	0,01	0,9	0,002
4	10397	0	0	0,04	0	0,13	0	0,01	0,22	0,6	0,58	0,05	0,22	0,15	0,022
4	9172	0	0	0,04	0	0,76	0,2	0	0	0	0,8	0	0,17	0,03	0,034
4	17849	0,06	0	0	0	0,07	0	0	0,87	0	0	0,92	0,07	0,01	0,046
4	41548	0	0,32	0	0	0	0,02	0	0,66	0	0,2	0,14	0	0,66	0,048
4	10549	0,02	0,04	0,01	0	0,91	0	0,02	0	0	0	0,03	0,97	0	0,048
4	20444	0	0	0	0,11	0	0	0	0,89	0	0	0	0,87	0,13	0,038
4	13153	0	0,29	0	0	0,71	0	0	0	0	0	0	0,29	0,71	0,044
4	14776	0,04	0,03	0	0,01	0,6	0,21	0	0	0,11	0,09	0,15	0,07	0,69	0,044
4	34416	0	0	0,04	0	0,93	0	0	0	0,03	0	0,04	0,17	0,79	0,008
4	17604	0	0	0,04	0	0,77	0	0,01	0	0,18	0,77	0,11	0	0,12	0,042
4	3125	0	0	0,04	0	0,87	0	0	0	0,09	0,12	0,18	0,01	0,69	0,014
4	24073	0	0	0,04	0,01	0	0,56	0	0,01	0,38	0	0,04	0,57	0,39	0,03
4	10355	0,02	0	0,02	0,01	0,92	0	0,03	0	0	0,02	0	0,02	0,96	0,046
4	22325	0,01	0,02	0	0,04	0,92	0	0,01	0	0	0	0,93	0,07	0	0,046
4	43135	0	0,05	0,04	0	0,62	0	0	0	0,29	0	0,04	0	0,96	0,008
4	15735	0	0	0	0,11	0,76	0,12	0,01	0	0	0	0,11	0,89	0	0,048
4	24650	0,03	0,02	0	0,03	0,9	0	0	0,01	0,01	0,07	0,9	0,03	0	0,046
4	39094	0,01	0	0,04	0	0	0,89	0	0	0,06	0,9	0	0	0,1	0,042
4	76849	0,06	0	0,01	0	0,93	0	0	0	0	0,07	0,93	0	0	0,048
4	49616	0	0	0	0,19	0,8	0	0	0,01	0	0,8	0,2	0	0	0,042
4	100000	0	0	1	0	0	0	0	0	0	0	1	0	0	0,618
4	26821	0	0	0,04	0,01	0,03	0,92	0	0	0	0,95	0,04	0,01	0	0,038
4	86783	0,07	0	0	0	0,92	0	0	0	0,01	0,03	0,01	0,96	0	0,048
4	15872	0	0	0,04	0,01	0	0,12	0	0	0,83	0	0,15	0,01	0,84	0,016
4	10103	0	0	0,04	0,01	0,7	0	0,06	0,05	0,14	0,01	0,79	0	0,2	0,048
4	21892	0,02	0,08	0	0,04	0	0	0	0,86	0	0,02	0,12	0	0,86	0,044

4	50063	0,06	0	0,01	0	0,15	0	0	0,75	0,03	0	0,22	0,03	0,75	0,046
4	83731	0,03	0,02	0,03	0	0,09	0,02	0,01	0,8	0	0,04	0,01	0,1	0,85	0,04
4	90972	0	0,08	0	0	0,91	0	0	0,01	0	0,91	0	0,09	0	0,042
4	50299	0,01	0,26	0	0	0,73	0	0	0	0	0,26	0,02	0	0,72	0,042
4	15211	0	0	0,04	0,05	0,05	0,86	0	0	0	0,86	0	0,1	0,04	0,046
4	4016	0	0,68	0	0	0	0,04	0,02	0,23	0,03	0,04	0,17	0,02	0,77	0,046
4	33287	0	0	0,02	0,07	0,89	0	0	0	0,02	0	0,9	0,07	0,03	0,046
4	15669	0	0,56	0	0	0,43	0	0,01	0	0	0,01	0	0,14	0,85	0,048
4	24638	0,03	0,05	0,01	0	0	0,91	0	0	0	0,03	0,95	0,01	0,01	0,042
4	33866	0	0,03	0	0,05	0,11	0	0	0,74	0,07	0	0,27	0,12	0,61	0,044
4	78324	0,03	0,13	0,01	0	0,78	0	0	0,05	0	0,12	0,79	0,09	0	0,048
4	88909	0	0,13	0	0	0,08	0,75	0,01	0	0,03	0	0,21	0,76	0,03	0,046
4	49842	0,03	0	0	0,01	0,01	0,91	0,01	0,03	0	0,02	0	0,03	0,95	0,038
4	3645	0,01	0	0,04	0	0	0	0	0,9	0,05	0	0,09	0,01	0,9	0,01
4	9137	0	0	0,04	0	0	0	0	0	0,96	0	0,04	0	0,96	0,008
4	53105	0	0,11	0,04	0	0	0,09	0	0,74	0,02	0	0,88	0,02	0,1	0,03
4,25	6965	0	0,35	0,04	0	0	0	0	0,61	0	0	0	0,04	0,96	0,028
4,25	49888	0,02	0	0,03	0	0	0	0,04	0,91	0	0	0,74	0,25	0,01	0,046
4,25	33009	0	0,04	0,04	0	0	0,89	0	0	0,03	0,03	0,04	0,05	0,88	0,028
4,25	10494	0	0	0,04	0,05	0,85	0,02	0,01	0,02	0,01	0,02	0,1	0,88	0	0,048
4,25	29198	0	0,01	0,04	0	0,94	0,01	0	0	0	0,94	0	0,04	0,02	0,044
4,25	28366	0,02	0	0	0,18	0,79	0	0,01	0	0	0,79	0,01	0,2	0	0,048
4,25	18547	0	0,25	0	0,05	0,7	0	0	0	0	0,02	0,72	0,01	0,25	0,046
4,25	57005	0	0,11	0	0,05	0,02	0,82	0	0	0	0,19	0	0	0,81	0,046
4,25	5657	0	0,01	0,04	0	0,76	0,19	0	0	0	0,76	0,23	0	0,01	0,034
4,25	47071	0	0	0,04	0	0	0,68	0	0,28	0	0	0,3	0,66	0,04	0,038
4,25	56816	0	0,13	0	0	0,87	0	0	0	0	0	0	1	0	0,04
4,25	12464	0	0	0	0,14	0,2	0	0	0,66	0	0,04	0	0,96	0	0,048
4,25	31009	0	0	0,01	0,01	0,89	0	0,07	0,02	0	0,09	0,91	0	0	0,048
4,25	7239	0	0	0,03	0	0,89	0	0,07	0,01	0	0	0,92	0,08	0	0,046
4,25	16523	0	0,06	0,04	0	0,88	0,01	0,01	0	0	0,07	0	0,88	0,05	0,048
4,25	5900	0,02	0,01	0,01	0,05	0,66	0,04	0	0,18	0,03	0	0,02	0,81	0,17	0,042
4,25	29004	0,02	0,08	0	0	0,88	0	0	0	0,02	0,03	0	0,89	0,08	0,04

4,25	3881	0	0	0,04	0,01	0,03	0,91	0,01	0	0	0	0,92	0,08	0	0,042
4,25	24942	0	0,01	0,04	0	0,64	0,01	0	0,06	0,24	0	0,64	0,06	0,3	0,012
4,25	68195	0	0	0,04	0	0,86	0	0	0,1	0	0,07	0,1	0,83	0	0,036
4,25	17084	0,01	0,02	0,04	0,04	0,01	0,74	0	0,14	0	0,06	0,06	0,01	0,87	0,044
4,25	73548	0,05	0	0	0	0	0	0	0,94	0,01	0,01	0	0,99	0	0,048
4,25	93555	0	0,03	0	0,19	0	0,11	0	0,67	0	0,78	0,02	0,17	0,03	0,048
4,25	12541	0	0,17	0,01	0	0,54	0	0,02	0	0,26	0,04	0,55	0,15	0,26	0,044
4,25	35803	0,02	0	0,03	0	0	0,9	0,02	0,03	0	0,43	0,02	0,54	0,01	0,04
4,25	19765	0	0,16	0	0	0,84	0	0	0	0	0,05	0	0,91	0,04	0,034
4,25	5255	0	0,01	0,04	0	0,94	0,01	0	0	0	0,47	0,05	0	0,48	0,026
4,25	35850	0	0	0,04	0,02	0	0	0	0,94	0	0,46	0	0,02	0,52	0,036
4,25	16073	0,01	0	0,04	0	0	0,88	0,06	0	0,01	0,01	0,06	0,88	0,05	0,044
4,25	14838	0,06	0	0	0	0	0,73	0	0,21	0	0,64	0,09	0,21	0,06	0,048
4,25	26457	0	0	0,04	0	0,32	0,11	0	0,01	0,52	0	0,01	0,12	0,87	0,008
4,25	17827	0	0,14	0	0	0,82	0	0	0,04	0	0,14	0,4	0,43	0,03	0,048
4,25	10714	0,06	0	0	0	0,85	0,03	0,01	0,04	0,01	0,79	0,08	0,03	0,1	0,046
4,25	75079	0,02	0,06	0	0,04	0	0	0	0,88	0	0,02	0,06	0,04	0,88	0,044
4,25	20486	0	0,11	0	0,01	0	0,86	0,02	0	0	0,05	0,07	0,87	0,01	0,048
4,25	8458	0,06	0	0	0	0,45	0,49	0	0	0	0,07	0,45	0	0,48	0,04
4,25	84675	0	0	0,02	0,07	0	0,7	0,01	0,2	0	0,01	0,93	0	0,06	0,048
4,25	37796	0,01	0	0,04	0	0	0,09	0	0,08	0,78	0,2	0,04	0,01	0,75	0,036
4,25	20006	0,04	0	0	0,04	0,89	0	0,03	0	0	0,93	0,02	0,05	0	0,046
4,25	26368	0	0,26	0,03	0	0,7	0	0,01	0	0	0	0,03	0,27	0,7	0,038
4,25	64593	0	0,07	0,01	0	0,86	0	0	0,06	0	0,87	0,06	0,07	0	0,044
4,25	14952	0,03	0	0	0	0	0,81	0,07	0,09	0	0,84	0,04	0,03	0,09	0,048
4,25	30065	0	0,12	0	0	0	0,88	0	0	0	0,12	0	0,88	0	0,048
4,25	8459	0	0,04	0,04	0	0	0,01	0	0	0,91	0,05	0,37	0	0,58	0,028
4,25	21291	0	0,29	0	0	0,7	0	0	0	0,01	0,29	0	0,01	0,7	0,046
4,25	100000	0	0,1	0,51	0	0	0	0,37	0	0,02	0,02	0,54	0,44	0	0,484
4,25	11838	0,05	0	0	0	0,39	0	0,02	0,54	0	0,07	0,54	0,39	0	0,048
4,25	21137	0,03	0	0	0,07	0	0,9	0	0	0	0,25	0,07	0,65	0,03	0,044
4,25	57456	0	0,36	0,04	0	0,6	0	0	0	0	0,04	0,36	0	0,6	0,036
4,25	9112	0	0,13	0	0,02	0,85	0	0	0	0	0	0,01	0,86	0,13	0,044

4,25	45195	0,01	0	0,04	0	0,83	0	0,02	0,09	0,01	0,15	0,02	0,83	0	0,048
4,25	15226	0	0	0,04	0,02	0,93	0	0,01	0	0	0,17	0,02	0	0,81	0,022
4,25	83211	0,04	0	0,04	0	0,04	0,47	0	0	0,41	0,06	0,02	0,8	0,12	0,038
4,25	40729	0	0	0,01	0,11	0,88	0	0	0	0	0,01	0,11	0,88	0	0,046
4,25	57670	0	0,02	0,02	0,07	0,7	0,13	0	0	0,06	0	0,17	0,83	0	0,044
4,25	45536	0,02	0	0	0,07	0	0,89	0	0,02	0	0	0,96	0	0,04	0,046
4,25	21168	0	0,63	0,02	0	0,01	0	0	0	0,34	0,02	0,04	0,01	0,93	0,044
4,25	100000	0	0	0,93	0	0	0,04	0,03	0	0	0	0,91	0,06	0,03	0,458
4,25	20024	0	0	0	0,07	0,93	0	0	0	0	0	0,24	0,07	0,69	0,048
4,25	26036	0	0,01	0,04	0	0,71	0,01	0	0,23	0	0	0,01	0,72	0,27	0,026
4,25	54163	0,01	0,02	0,04	0	0,03	0	0	0,78	0,12	0,02	0,05	0,12	0,81	0,026
4,25	100000	0	0	0,95	0	0	0,01	0,02	0	0,02	0,02	0,98	0	0	0,618
4,25	10826	0	0	0	0,1	0,09	0,81	0	0	0	0,11	0	0,1	0,79	0,048
4,25	30441	0	0	0,04	0	0,74	0,22	0	0	0	0	0	0,3	0,7	0,008
4,25	21747	0	0,01	0	0,01	0	0,05	0,06	0,85	0,02	0,26	0,08	0	0,66	0,048
4,25	21118	0	0,05	0	0,06	0,89	0	0	0	0	0	0,95	0,05	0	0,044
4,25	62119	0,02	0	0,04	0,02	0,66	0	0	0	0,26	0	0,04	0,28	0,68	0,048
4,25	32593	0	0,34	0,02	0,01	0,63	0	0	0	0	0,05	0,85	0,04	0,06	0,048
4,25	18571	0	0	0	0,18	0,82	0	0	0	0	0,82	0,18	0	0	0,044
4,25	12058	0,01	0	0,04	0	0	0	0	0,95	0	0,03	0,95	0,01	0,01	0,016
4,25	54665	0	0	0,04	0	0,66	0	0,01	0,29	0	0,29	0,04	0,66	0,01	0,028
4,25	10826	0	0	0,04	0	0,96	0	0	0	0	0	0,04	0	0,96	0,002
4,25	24325	0	0,02	0,04	0,01	0,01	0,88	0	0	0,04	0,02	0,89	0,04	0,05	0,048
4,25	100000	0	0	0,99	0	0	0	0	0	0,01	0,01	0,99	0	0	0,718
4,25	9099	0,05	0	0	0	0,43	0	0,01	0,51	0	0	0,45	0,52	0,03	0,048
4,25	72919	0	0,13	0	0,04	0	0	0	0,83	0	0,01	0	0,28	0,71	0,048
4,25	57317	0	0	0,04	0	0,85	0,07	0	0,04	0	0,01	0,11	0,04	0,84	0,004
4,25	10234	0	0	0,04	0	0,67	0	0	0,29	0	0,67	0,04	0,27	0,02	0,028
4,25	65782	0,03	0,03	0	0,01	0,93	0	0	0	0	0,07	0	0,93	0	0,046
4,25	84067	0,01	0,05	0	0	0,79	0	0,05	0	0,1	0,87	0	0,11	0,02	0,046
4,25	13260	0,01	0	0	0	0,8	0	0,1	0,09	0	0,07	0,1	0,81	0,02	0,04
4,25	15058	0	0	0,02	0,07	0,85	0,03	0	0	0,03	0,06	0	0,05	0,89	0,048
4,25	62545	0	0,12	0	0	0,01	0,7	0	0,17	0	0,2	0,18	0,62	0	0,048

4,25	20423	0	0,07	0,02	0,06	0,54	0	0	0,31	0	0	0,59	0,1	0,31	0,048
4,25	39695	0	0	0	0	0,81	0	0,09	0,05	0,05	0	0,81	0,14	0,05	0,048
4,25	53940	0,05	0	0	0	0	0,93	0	0	0,02	0	0,11	0	0,89	0,046
4,25	6719	0,02	0	0	0,12	0,86	0	0	0	0	0,85	0	0	0,15	0,046
4,25	89519	0,01	0,17	0	0,02	0,8	0	0	0	0	0,01	0,8	0,19	0	0,048
4,25	3268	0	0	0,03	0,03	0,02	0	0,04	0,88	0	0,03	0,92	0,05	0	0,048
4,25	18739	0	0,16	0	0	0,51	0,06	0	0,27	0	0,17	0,1	0,57	0,16	0,04
4,25	100000	0	0	0,97	0,03	0	0	0	0	0	0,23	0,74	0	0,03	0,69
4,25	73797	0,05	0	0,01	0	0	0	0	0	0,94	0,99	0,01	0	0	0,046
4,25	2222	0,04	0	0,04	0	0,92	0	0	0	0	0,04	0,93	0	0,03	0,036
4,25	4577	0,02	0	0	0,12	0,15	0,64	0,02	0	0,05	0,87	0	0,13	0	0,042
4,25	3301	0	0	0,04	0	0	0	0	0,95	0,01	0	0	0,04	0,96	0,004
4,25	100000	0,02	0	0,92	0	0	0,05	0,01	0	0	0,01	0,94	0,04	0,01	0,502
4,25	78174	0	0	0,04	0	0,38	0	0	0,01	0,57	0,32	0,1	0	0,58	0,02
4,25	40261	0	0	0,04	0,06	0,9	0	0	0	0	0,9	0,06	0,04	0	0,046
4,25	47709	0	0	0,04	0	0,01	0	0	0	0,95	0	0,04	0,16	0,8	0,008
4,25	33713	0	0,91	0	0	0	0	0	0	0,09	0	0	0,06	0,94	0,046
4,5	9933	0	0	0,04	0	0,72	0	0	0,24	0	0	0,01	0,15	0,84	0,004
4,5	39885	0	0	0,04	0,01	0	0,95	0	0	0	0	0,04	0,64	0,32	0,026
4,5	4377	0	0,02	0,02	0,03	0,87	0,04	0,01	0,01	0	0,03	0,88	0,07	0,02	0,048
4,5	18850	0	0,07	0,04	0	0	0	0	0,14	0,75	0,07	0,18	0	0,75	0,036
4,5	25613	0	0,07	0	0	0,01	0,86	0,03	0,02	0,01	0,11	0,05	0,84	0	0,04
4,5	26297	0	0,31	0	0	0,65	0,03	0	0	0,01	0,31	0,03	0,01	0,65	0,046
4,5	8112	0	0	0,04	0,01	0	0	0	0	0,95	0	0,04	0	0,96	0,018
4,5	10310	0	0,12	0,04	0	0	0	0	0	0,84	0,01	0,15	0	0,84	0,032
4,5	100000	0	0	0,97	0	0	0,03	0	0	0	0	0,99	0	0,01	0,632
4,5	23931	0	0,08	0,04	0	0,88	0	0	0	0	0,04	0,05	0,91	0	0,044
4,5	57681	0	0	0	0,19	0,76	0,05	0	0	0	0,76	0	0,15	0,09	0,04
4,5	26586	0	0	0,04	0	0	0	0	0,96	0	0	0	0,04	0,96	0,004
4,5	83561	0	0	0,01	0	0,73	0,1	0,16	0	0	0,74	0	0,09	0,17	0,046
4,5	4970	0	0	0	0,04	0,83	0	0,02	0,11	0	0,04	0,13	0	0,83	0,034
4,5	30660	0,06	0	0	0	0	0	0	0,92	0,02	0	0,02	0,92	0,06	0,048
4,5	53934	0,04	0,03	0,01	0	0,92	0	0	0	0	0,35	0,03	0	0,62	0,046

4,5	23474	0	0,96	0	0,04	0	0	0	0	0	0	0,96	0	0,04	0,046
4,5	67738	0,06	0	0	0	0,63	0,02	0	0,29	0	0	0,68	0,29	0,03	0,044
4,5	34785	0,07	0	0	0	0,71	0	0	0,22	0	0,07	0	0,29	0,64	0,044
4,5	30598	0	0	0,04	0	0,96	0	0	0	0	0	0	0,99	0,01	0,03
4,5	100000	0	0,02	0,94	0	0	0	0,04	0	0	0	0,96	0,04	0	0,542
4,5	61187	0	0,11	0,03	0	0	0,86	0	0	0	0,11	0,86	0,03	0	0,048
4,5	88537	0,07	0	0	0	0,27	0,05	0	0	0,61	0,05	0	0,27	0,68	0,048
4,5	34303	0	0,02	0	0,09	0	0,04	0,01	0,84	0	0,11	0	0,89	0	0,048
4,5	39209	0,05	0,14	0	0	0,81	0	0	0	0	0,04	0,95	0	0,01	0,044
4,5	42105	0	0	0,04	0	0	0	0	0,03	0,93	0	0,11	0,03	0,86	0,01
4,5	100000	0	0	0,7	0	0	0,15	0	0,01	0,14	0,15	0,7	0,15	0	0,37
4,5	38274	0	0	0,04	0	0,96	0	0	0	0	0	0,04	0	0,96	0
4,5	30381	0	0	0	0,07	0,89	0	0	0,04	0	0,37	0	0	0,63	0,048
4,5	100000	0	0,02	0,98	0	0	0	0	0	0	0	1	0	0	0,63
4,5	84521	0,03	0,01	0,02	0,03	0,72	0,07	0	0,12	0	0	0,1	0,15	0,75	0,046
4,5	7146	0	0	0	0,06	0,94	0	0	0	0	0,06	0,93	0	0,01	0,048
4,5	12426	0	0	0,04	0,02	0,92	0	0	0	0,02	0	0,05	0,93	0,02	0,03
4,5	16921	0,04	0,13	0	0	0,2	0,12	0	0,51	0	0,06	0,04	0,13	0,77	0,048
4,5	40104	0	0	0,04	0,01	0	0,18	0	0,77	0	0,15	0,04	0,77	0,04	0,028
4,5	100000	0	0	0,98	0	0	0	0	0,02	0	0,02	0,98	0	0	0,684
4,5	100000	0,01	0,16	0,83	0	0	0	0	0	0	0	0,52	0,48	0	0,492
4,5	42400	0	0,2	0	0,03	0,76	0	0	0,01	0	0,01	0,03	0,2	0,76	0,044
4,5	78053	0,01	0,01	0,04	0	0,92	0,01	0	0,01	0	0,22	0	0,71	0,07	0,048
4,5	31822	0,03	0	0,04	0	0,02	0,6	0	0	0,31	0,06	0,68	0,22	0,04	0,048
4,5	10881	0,02	0	0,04	0,01	0	0,01	0	0,8	0,12	0	0,04	0,88	0,08	0,044
4,5	100000	0	0	0,99	0	0	0	0,01	0	0	0	0,98	0,02	0	0,556
4,5	10962	0,01	0	0,04	0,01	0,32	0,47	0	0,1	0,05	0,71	0,14	0	0,15	0,034
4,5	22918	0,06	0	0	0	0,7	0	0	0,11	0,13	0,01	0,69	0,13	0,17	0,046
4,5	10863	0,03	0	0,04	0	0,77	0,15	0	0,01	0	0,02	0,04	0,11	0,83	0,028
4,5	100000	0	0,04	0,9	0	0	0	0	0,05	0,01	0	0,98	0,01	0,01	0,508
4,5	100000	0	0	1	0	0	0	0	0	0	0	0,93	0	0,07	0,456
4,5	22262	0,04	0	0,01	0,03	0,9	0	0	0,02	0	0,06	0,03	0,91	0	0,048
4,5	60711	0	0	0	0,06	0,88	0,05	0,01	0	0	0,06	0	0,01	0,93	0,048

4,5	13127	0,03	0	0,04	0	0	0,01	0,01	0,91	0	0,03	0	0,95	0,02	0,038
4,5	16300	0	0	0	0,11	0,6	0,16	0	0	0,13	0,16	0	0,73	0,11	0,04
4,5	100000	0,03	0	0,74	0	0	0,06	0,03	0	0,14	0,13	0,77	0,06	0,04	0,446
4,5	62918	0,01	0,28	0,03	0	0,66	0	0	0	0,02	0	0,05	0,29	0,66	0,048
4,5	36234	0,01	0	0,04	0,01	0,8	0	0	0,13	0,01	0,02	0,18	0,01	0,79	0,022
4,5	66320	0,06	0	0	0,01	0,05	0,87	0	0,01	0	0,94	0,06	0	0	0,046
4,5	79666	0	0,96	0,04	0	0	0	0	0	0	0	0,04	0	0,96	0,022
4,5	36891	0	0	0,04	0	0,78	0,03	0,01	0,1	0,04	0,67	0,1	0	0,23	0,04
4,5	9408	0	0	0	0,16	0,77	0	0	0,07	0	0,77	0	0,09	0,14	0,048
4,5	62787	0,05	0,01	0	0	0,05	0,89	0	0	0	0,43	0,4	0,05	0,12	0,044
4,5	9479	0	0,08	0,04	0,03	0,69	0,01	0	0,14	0,01	0,11	0,88	0,01	0	0,044
4,5	8037	0	0	0	0,11	0,89	0	0	0	0	0,11	0	0,89	0	0,048
4,5	48058	0	0	0	0,07	0,92	0	0	0,01	0	0,08	0,92	0	0	0,048
4,5	89454	0,06	0	0	0	0	0,94	0	0	0	0,99	0	0,01	0	0,046
4,5	61251	0	0	0	0,09	0	0,91	0	0	0	0,09	0,91	0	0	0,046
4,5	42722	0	0,05	0,03	0	0,9	0	0,02	0	0	0,9	0,02	0,06	0,02	0,048
4,5	72029	0	0	0,04	0,02	0,03	0	0	0,23	0,68	0,12	0,04	0,68	0,16	0,024
4,5	48706	0,03	0	0,04	0	0,21	0	0	0,72	0	0	0,03	0,25	0,72	0,022
4,5	17469	0	0	0,04	0	0,96	0	0	0	0	0	0	1	0	0,016
4,5	17881	0	0	0,04	0	0,9	0	0	0	0,06	0,83	0,07	0,06	0,04	0,024
4,5	66328	0	0	0,04	0,01	0,59	0	0	0	0,36	0	0,63	0,01	0,36	0,022
4,5	11303	0	0,04	0	0,11	0,85	0	0	0	0	0,89	0	0,11	0	0,044
4,5	31111	0	0,03	0,04	0	0	0	0,02	0,49	0,42	0	0,34	0	0,66	0,02
4,5	3991	0,01	0	0,04	0	0,84	0	0	0,11	0	0	0,57	0,43	0	0,02
4,5	30507	0,05	0	0	0	0,92	0	0	0,03	0	0	0	1	0	0,046
4,5	7481	0,01	0	0,04	0	0,59	0,06	0	0	0,3	0,01	0,7	0	0,29	0,016
4,5	40368	0	0	0,02	0	0,92	0	0,05	0	0,01	0	0,04	0,03	0,93	0,038
4,5	18487	0	0	0,01	0,19	0,8	0	0	0	0	0,8	0,19	0	0,01	0,048
4,5	19329	0,03	0,74	0	0	0,21	0,01	0	0	0,01	0,03	0,22	0,01	0,74	0,044
4,5	23413	0	0,1	0,04	0	0	0,82	0,01	0	0,03	0	0,82	0,1	0,08	0,04
4,5	11102	0	0	0,04	0,01	0	0	0	0,95	0	0,01	0,91	0,01	0,07	0,004
4,5	100000	0	0,05	0,91	0,04	0	0	0	0	0	0,25	0,53	0,18	0,04	0,592
4,5	7624	0	0	0,04	0,01	0	0,4	0	0	0,55	0	0,04	0,41	0,55	0,03

4,5	39985	0	0	0,01	0,27	0,02	0,7	0	0	0	0,97	0,01	0,01	0,01	0,048
4,5	62738	0	0,34	0,04	0	0,07	0	0	0,49	0,06	0,06	0,05	0,07	0,82	0,024
4,5	55093	0,01	0	0,04	0	0	0,03	0	0,9	0,02	0,02	0,05	0,01	0,92	0,012
4,5	100000	0	0	0,99	0	0	0	0	0,01	0	0	1	0	0	0,586
4,5	49184	0,01	0	0	0,15	0,84	0	0	0	0	0,8	0,01	0,06	0,13	0,044
4,5	46921	0	0,08	0	0,06	0,24	0,62	0	0	0	0,06	0	0,3	0,64	0,044
4,5	77596	0,01	0	0,04	0	0	0	0,02	0,91	0,02	0,91	0,02	0,04	0,03	0,024
4,5	23064	0	0,02	0	0,09	0,84	0	0	0,03	0,02	0,02	0,52	0,46	0	0,048
4,5	19310	0	0,88	0	0,03	0	0	0	0,07	0,02	0	0,05	0,03	0,92	0,046
4,5	14536	0,04	0,4	0	0	0,56	0	0	0	0	0	0,4	0,04	0,56	0,048
4,5	17692	0	0	0,03	0,06	0,7	0	0	0,04	0,17	0,17	0,09	0,17	0,57	0,044
4,5	24423	0,06	0	0	0	0,93	0,01	0	0	0	0	0,94	0	0,06	0,048
4,5	39703	0	0,01	0,04	0,02	0	0	0	0,92	0,01	0	0,04	0,01	0,95	0,022
4,5	9315	0	0,02	0,03	0	0,95	0	0	0	0	0,97	0	0	0,03	0,046
4,5	6128	0	0	0,04	0	0,68	0,24	0,03	0,01	0	0,01	0,85	0,14	0	0,04
4,5	10779	0,01	0,04	0,01	0,04	0	0,46	0	0,41	0,03	0,43	0,1	0,47	0	0,048
4,5	9604	0	0	0	0,1	0,69	0,21	0	0	0	0	0,21	0,79	0	0,042
4,5	100000	0,16	0	0,84	0	0	0	0	0	0	0,09	0,77	0,14	0	0,654
4,75	15409	0	0,13	0	0	0,87	0	0	0	0	0,43	0,44	0,13	0	0,044
4,75	77666	0,05	0,04	0	0	0	0,91	0	0	0	0,04	0	0,05	0,91	0,048
4,75	98525	0	0,12	0	0	0,88	0	0	0	0	0,12	0	0,88	0	0,042
4,75	31357	0	0	0,04	0	0,96	0	0	0	0	0,04	0	0	0,96	0
4,75	15508	0,04	0	0,03	0	0,02	0,17	0	0,74	0	0,18	0,08	0,04	0,7	0,044
4,75	75722	0	0,3	0	0	0,63	0	0	0,07	0	0,1	0,09	0,22	0,59	0,044
4,75	15194	0,06	0	0	0,01	0,93	0	0	0	0	0,93	0,01	0,06	0	0,048
4,75	21753	0,03	0	0,01	0,07	0,89	0	0	0	0	0,84	0,09	0,07	0	0,048
4,75	94475	0	0	0	0,13	0,87	0	0	0	0	0,01	0	0,99	0	0,046
4,75	100000	0,02	0	0,97	0	0	0	0,01	0	0	0	0,97	0,02	0,01	0,576
4,75	24717	0	0,01	0,02	0,09	0,72	0	0	0,16	0	0	0	0,88	0,12	0,048
4,75	14218	0,06	0	0	0	0,03	0,08	0	0,79	0,04	0,09	0,08	0,81	0,02	0,046
4,75	21361	0	0	0,01	0,08	0,77	0	0	0,14	0	0,06	0,58	0,08	0,28	0,048
4,75	19153	0,01	0,1	0	0	0,83	0,02	0,03	0,01	0	0,32	0,1	0,52	0,06	0,048
4,75	42175	0	0,11	0,01	0	0,83	0	0	0,05	0	0,01	0,11	0,88	0	0,046

4,75	8110	0	0,28	0	0	0,68	0	0	0,02	0,02	0	0	0,3	0,7	0,048
4,75	100000	0	0,01	0,12	0,05	0	0,5	0	0,32	0	0	0,13	0,5	0,37	0,08
4,75	2644	0,05	0	0,02	0	0	0	0,01	0,91	0,01	0,01	0,02	0,06	0,91	0,048
4,75	64023	0	0	0,01	0,04	0,9	0,01	0,03	0,01	0	0,01	0,01	0,05	0,93	0,046
4,75	9352	0,04	0,1	0	0	0	0	0	0,82	0,04	0,04	0	0,1	0,86	0,036
4,75	24237	0	0	0,03	0,08	0	0	0	0,54	0,35	0,08	0,55	0,01	0,36	0,048
4,75	25403	0	0	0	0,06	0,94	0	0	0	0	0,06	0	0,06	0,88	0,046
4,75	12944	0,05	0	0,03	0	0,4	0,52	0	0	0	0,45	0	0,01	0,54	0,048
4,75	12369	0	0	0,04	0,09	0,12	0,73	0,02	0	0	0,71	0,04	0,03	0,22	0,048
4,75	31088	0	0,04	0,04	0	0	0,32	0	0,21	0,39	0,39	0,36	0,22	0,03	0,04
4,75	85049	0,05	0	0	0	0,71	0,15	0	0,09	0	0,18	0	0,82	0	0,048
4,75	4753	0,01	0,01	0,04	0	0	0	0	0	0,94	0	0,05	0,94	0,01	0,034
4,75	24156	0,01	0	0	0,09	0,06	0,76	0	0,01	0,07	0,04	0,1	0,76	0,1	0,048
4,75	39398	0	0	0	0,06	0,94	0	0	0	0	0	0	0,06	0,94	0,048
4,75	83583	0	0	0,03	0,16	0,81	0	0	0	0	0,86	0,03	0	0,11	0,046
4,75	50631	0	0	0	0,08	0,06	0	0	0	0,86	0	0	0	1	0,048
4,75	14201	0	0	0,04	0,04	0,84	0	0	0,08	0	0,84	0,1	0,02	0,04	0,036
4,75	36490	0	0	0	0,06	0,92	0	0	0,02	0	0,06	0,88	0,01	0,05	0,042
4,75	100000	0	0	0,15	0,02	0,09	0,61	0	0,13	0	0,18	0,14	0,09	0,59	0,048
4,75	24981	0,03	0	0,02	0	0,94	0	0	0,01	0	0,92	0,06	0	0,02	0,046
4,75	68027	0	0	0	0,12	0,42	0	0	0,46	0	0,46	0	0,37	0,17	0,036
4,75	44464	0	0,68	0	0,02	0	0,05	0,01	0,24	0	0,01	0,69	0	0,3	0,04
4,75	100000	0	0	1	0	0	0	0	0	0	0	1	0	0	0,684
4,75	52487	0,05	0	0	0	0,94	0	0,01	0	0	0,06	0,12	0	0,82	0,046
4,75	37759	0	0,11	0,04	0	0,83	0,02	0	0	0	0,1	0,04	0,84	0,02	0,044
4,75	40481	0	0	0,04	0	0	0,96	0	0	0	0	0	0,97	0,03	0,03
4,75	29854	0	0,07	0,04	0	0,89	0	0	0	0	0,04	0	0,96	0	0,048
4,75	8193	0,01	0	0	0,13	0	0,56	0	0,3	0	0,56	0,16	0,08	0,2	0,048
4,75	65963	0	0	0	0,24	0,69	0	0	0,07	0	0,69	0,24	0,07	0	0,046
4,75	3473	0	0,01	0,04	0	0	0,02	0	0,9	0,03	0	0,04	0,93	0,03	0,03
4,75	39758	0	0	0	0,11	0,89	0	0	0	0	0	0	1	0	0,046
4,75	8898	0,01	0	0,04	0,01	0,4	0	0	0	0,54	0	0,39	0,6	0,01	0,04
4,75	3170	0	0	0,02	0	0	0,78	0,2	0	0	0,72	0,08	0,2	0	0,046

4,75	28994	0	0,14	0,01	0	0	0,84	0,01	0	0	0	0,16	0,83	0,01	0,046
4,75	34285	0,05	0	0,01	0	0	0	0	0,94	0	0	0,99	0,01	0	0,044
4,75	29012	0,03	0	0,01	0	0,93	0	0,03	0	0	0	0,04	0,96	0	0,04
4,75	90438	0	0,07	0	0,03	0,85	0,04	0	0,01	0	0,66	0,11	0,04	0,19	0,042
4,75	42409	0	0	0,04	0	0,68	0	0	0,25	0,03	0	0,04	0,96	0	0,03
4,75	34630	0,06	0	0	0	0	0,93	0	0	0,01	0,33	0	0	0,67	0,048
4,75	32995	0,06	0	0,01	0	0,92	0	0,01	0	0	0,97	0,01	0	0,02	0,046
4,75	30245	0	0	0,04	0	0,78	0,01	0	0,17	0	0,92	0	0,07	0,01	0,032
4,75	2709	0	0	0,04	0,02	0,93	0	0,01	0	0	0,94	0,04	0	0,02	0,04
4,75	10358	0	0,13	0	0	0,86	0	0	0	0,01	0,01	0	0,99	0	0,044
4,75	55949	0	0	0,03	0	0,54	0,04	0,07	0,32	0	0	0,35	0,11	0,54	0,048
4,75	47525	0	0	0,03	0,08	0	0,86	0	0,03	0	0	0,11	0,89	0	0,048
4,75	100000	0,26	0,02	0,72	0	0	0	0	0	0	0	1	0	0	0,742
4,75	39869	0	0,24	0	0	0,61	0,15	0	0	0	0,24	0	0,15	0,61	0,046
4,75	100000	0,37	0,01	0,35	0	0	0,01	0	0,26	0	0	0,27	0,35	0,38	0,502
4,75	29157	0	0	0,03	0,06	0	0	0	0	0,91	0	0,03	0	0,97	0,046
4,75	8567	0,06	0,01	0	0	0,92	0	0	0,01	0	0	0	0,99	0,01	0,04
4,75	25735	0,01	0	0,02	0	0	0	0,13	0,84	0	0,98	0,02	0	0	0,04
4,75	26530	0	0,29	0	0	0,58	0,13	0	0	0	0,01	0	0,29	0,7	0,048
4,75	14654	0	0,02	0,04	0	0	0	0	0,94	0	0,94	0,02	0	0,04	0,042
4,75	78782	0	0	0,04	0	0	0	0	0,38	0,58	0	0,04	0,38	0,58	0,014
4,75	41400	0	0,18	0,04	0	0,72	0	0,01	0,01	0,04	0,03	0,02	0,04	0,91	0,02
4,75	9789	0	0,14	0	0	0	0,02	0	0,83	0,01	0,01	0	0,83	0,16	0,044
4,75	16269	0	0,02	0,04	0	0,92	0	0,02	0	0	0,02	0,04	0,01	0,93	0,024
4,75	5971	0,05	0	0	0	0,92	0,02	0,01	0	0	0,98	0,01	0	0,01	0,046
4,75	42532	0,03	0	0,03	0	0,3	0,51	0	0	0,13	0,04	0,42	0,52	0,02	0,038
4,75	100000	0	0	0,93	0	0,01	0	0,06	0	0	0	0,94	0	0,06	0,504
4,75	45791	0	0,18	0,04	0	0	0	0	0	0,78	0	0,17	0,01	0,82	0,032
4,75	100000	0,02	0	0,98	0	0	0	0	0	0	0	0,98	0,02	0	0,594
4,75	34168	0	0	0	0,06	0,91	0	0	0,03	0	0,08	0	0,03	0,89	0,048
4,75	37164	0	0	0,01	0	0,84	0	0,12	0	0,03	0,12	0,04	0,84	0	0,048
4,75	41371	0	0,95	0	0,03	0	0,02	0	0	0	0	0,03	0,02	0,95	0,046
4,75	37619	0,02	0,62	0	0	0,36	0	0	0	0	0,05	0,63	0	0,32	0,048

4,75	34723	0	0	0,04	0	0,02	0,66	0	0	0,28	0	0,04	0,81	0,15	0,018
4,75	7541	0	0	0	0,09	0,71	0	0	0,11	0,09	0	0	0,29	0,71	0,048
4,75	44773	0,01	0	0	0,09	0,9	0	0	0	0	0	0	1	0	0,048
4,75	38659	0	0	0	0,12	0,86	0	0	0,02	0	0,12	0	0,88	0	0,048
4,75	54168	0	0	0,04	0	0,02	0,94	0	0	0	0	0	0,06	0,94	0,006
4,75	92072	0	0,22	0	0	0,78	0	0	0	0	0	0,79	0,21	0	0,046
4,75	96553	0	0,08	0,04	0	0,88	0	0	0	0	0,04	0	0,08	0,88	0,018
4,75	67689	0	0,33	0	0	0,67	0	0	0	0	0,04	0	0,29	0,67	0,044
4,75	10905	0	0	0,04	0,04	0,09	0,33	0	0,5	0	0,55	0,09	0,05	0,31	0,046
4,75	26328	0	0,17	0,04	0	0,79	0	0	0	0	0,16	0,74	0,04	0,06	0,048
4,75	23185	0,01	0,08	0,04	0,01	0	0,86	0	0	0	0,05	0,75	0,07	0,13	0,048
4,75	76915	0,05	0	0,04	0	0,68	0,19	0	0,04	0	0,02	0,11	0,68	0,19	0,048
4,75	100000	0	0	1	0	0	0	0	0	0	0,01	0,94	0	0,05	0,492
4,75	100000	0,09	0	0,91	0	0	0	0	0	0	0,82	0,18	0	0	0,964
4,75	100000	0,14	0,12	0,62	0	0	0	0,02	0,08	0,02	0	0,7	0,28	0,02	0,47
4,75	47310	0,04	0,01	0,03	0	0,63	0,26	0	0,03	0	0,26	0,07	0,63	0,04	0,048
4,75	65011	0,05	0	0	0	0,95	0	0	0	0	0,95	0	0,05	0	0,048
4,75	57511	0,03	0,02	0,04	0	0,83	0	0	0,06	0,02	0,03	0,04	0,93	0	0,046
4,75	98883	0,01	0	0,03	0,03	0,01	0,05	0,01	0	0,86	0	0,09	0,01	0,9	0,044
5	18101	0	0,06	0	0,04	0	0	0	0,9	0	0,78	0,04	0	0,18	0,048
5	37351	0,05	0	0,01	0	0,94	0	0	0	0	0,06	0	0,94	0	0,042
5	62617	0	0	0,04	0,01	0,86	0	0,09	0	0	0,88	0,04	0,07	0,01	0,046
5	38795	0	0	0,04	0	0,94	0,01	0,01	0	0	0,22	0	0,73	0,05	0,028
5	80105	0,02	0	0	0	0,37	0,43	0,1	0,08	0	0,76	0,1	0,04	0,1	0,042
5	17086	0	0	0	0,3	0,69	0	0	0,01	0	0,98	0,01	0,01	0	0,048
5	61633	0,02	0,14	0	0,01	0,8	0	0	0,03	0	0,03	0,81	0,14	0,02	0,048
5	8586	0,03	0,03	0,02	0	0,92	0	0	0	0	0,03	0,02	0,95	0	0,046
5	16237	0,06	0	0	0,01	0	0	0	0,77	0,16	0,7	0,04	0,06	0,2	0,048
5	72558	0,02	0	0,03	0	0	0	0,08	0,87	0	0,86	0,03	0,02	0,09	0,044
5	54687	0	0,1	0,04	0	0,82	0	0,04	0	0	0,02	0,02	0,04	0,92	0,048
5	17081	0	0,11	0	0	0,89	0	0	0	0	0,12	0	0,88	0	0,038
5	44908	0	0	0,04	0,01	0,7	0	0,01	0,18	0,06	0,01	0	0,05	0,94	0,018
5	24535	0,04	0	0,04	0,01	0	0	0	0	0,91	0	0,03	0,02	0,95	0,042

5	74983	0	0	0,04	0	0	0,9	0,05	0	0,01	0	0,05	0,95	0	0,034
5	14783	0	0	0,04	0	0,21	0,75	0	0	0	0,04	0	0,92	0,04	0,018
5	32291	0	0	0,04	0,02	0,09	0,8	0	0,05	0	0,01	0,12	0,82	0,05	0,038
5	58995	0	0	0	0	0	0,88	0,12	0	0	0,12	0	0,78	0,1	0,048
5	100000	0	0,01	0,96	0	0	0	0	0	0,03	0	0,97	0	0,03	0,562
5	32277	0	0,01	0,04	0	0	0,27	0,05	0	0,63	0,24	0,04	0,53	0,19	0,046
5	100000	0,13	0	0,8	0	0	0	0	0	0,07	0	0,8	0	0,2	0,478
5	56657	0	0,11	0,04	0	0	0	0	0	0,85	0	0,1	0,13	0,77	0,026
5	100000	0	0	1	0	0	0	0	0	0	0	1	0	0	0,706
5	5737	0,03	0	0,04	0	0,92	0	0,01	0	0	0,99	0	0,01	0	0,048
5	95276	0	0	0,04	0,05	0,65	0,04	0,06	0,16	0	0,74	0,05	0,17	0,04	0,048
5	8587	0,05	0	0,02	0	0	0	0,01	0,9	0,02	0,05	0,94	0	0,01	0,048
5	20816	0	0	0	0,11	0,44	0,42	0,02	0	0,01	0,03	0	0,97	0	0,048
5	38186	0	0,06	0,04	0	0,13	0,34	0	0	0,43	0	0,1	0,13	0,77	0,028
5	22064	0	0,27	0	0	0,73	0	0	0	0	0,27	0	0	0,73	0,046
5	99787	0,02	0	0	0	0,09	0,8	0,08	0	0,01	0,29	0,56	0,15	0	0,044
5	97401	0	0,15	0	0	0,84	0	0,01	0	0	0,15	0	0,84	0,01	0,044
5	19803	0,01	0,1	0	0	0,79	0,02	0	0,08	0	0	0,02	0,98	0	0,048
5	15924	0	0	0,04	0	0	0,92	0	0	0,04	0,06	0	0,02	0,92	0,012
5	32809	0,03	0	0,04	0,03	0,03	0	0	0,87	0	0,01	0,03	0,03	0,93	0,048
5	19879	0,06	0	0	0	0,14	0	0	0,73	0,07	0,21	0	0,33	0,46	0,048
5	24028	0,01	0	0,04	0	0	0,14	0,04	0,33	0,44	0,09	0,19	0,7	0,02	0,044
5	52204	0	0,1	0	0	0,66	0	0,05	0,19	0	0	0,66	0,24	0,1	0,048
5	100000	0,07	0,01	0,91	0	0	0,01	0	0	0	0	0,99	0	0,01	0,62
5	2547	0	0	0	0,07	0,89	0	0	0,04	0	0,04	0	0,07	0,89	0,048
5	42927	0,01	0,06	0	0	0	0,85	0	0	0,08	0,94	0	0,06	0	0,044
5	52956	0	0	0,04	0	0,95	0,01	0	0	0	0,99	0	0	0,01	0,044
5	28649	0	0	0,04	0	0,03	0,3	0	0,6	0,03	0,6	0,06	0,27	0,07	0,026
5	69215	0	0	0,04	0	0	0,14	0	0	0,82	0,03	0,17	0	0,8	0,008
5	32000	0,02	0	0,04	0	0,91	0	0	0,03	0	0,14	0,02	0	0,84	0,02
5	28740	0	0	0,04	0	0,27	0,62	0,07	0	0	0	0,93	0,07	0	0,044
5	50976	0,05	0,18	0	0	0	0	0,01	0,76	0	0	0,13	0	0,87	0,048
5	41183	0	0,01	0,04	0	0,52	0	0	0,43	0	0,43	0,04	0,43	0,1	0,038

5	13443	0,03	0	0,03	0,01	0	0,48	0,01	0,44	0	0,01	0,04	0,35	0,6	0,048
5	25342	0	0	0	0,19	0,79	0,01	0	0	0,01	0,76	0,23	0,01	0	0,046
5	18186	0	0	0,04	0	0	0,02	0	0,86	0,08	0	0,04	0,02	0,94	0
5	4550	0	0	0,04	0	0,55	0	0,02	0,13	0,26	0	0,02	0,73	0,25	0,04
5	30795	0	0	0,04	0	0,83	0,03	0,01	0,06	0,03	0,83	0,04	0,09	0,04	0,03
5	17607	0	0	0,04	0	0	0,34	0	0	0,62	0	0,38	0	0,62	0,022
5	85311	0	0,48	0	0,06	0,46	0	0	0	0	0	0,15	0	0,85	0,048
5	35668	0,01	0	0,01	0,1	0,88	0	0	0	0	0,02	0	0,88	0,1	0,036
5	37011	0	0	0,04	0	0,73	0	0,02	0,21	0	0,21	0	0,04	0,75	0,032
5	68835	0	0,22	0,04	0	0	0	0	0,74	0	0,04	0	0,22	0,74	0,042
5	56817	0	0,14	0	0,02	0,2	0,61	0	0,03	0	0,36	0,02	0,06	0,56	0,048
5	28303	0	0,01	0	0,12	0,77	0	0	0,08	0,02	0,08	0,13	0,77	0,02	0,046
5	100000	0	0	0,99	0	0	0	0,01	0	0	0,01	0,99	0	0	0,646
5	100000	0	0	0,99	0	0	0	0	0,01	0	0	0,97	0	0,03	0,514
5	77156	0	0	0	0,37	0,62	0	0,01	0	0	1	0	0	0	0,048
5	72006	0	0,18	0	0	0,6	0,13	0	0,09	0	0,18	0,38	0,35	0,09	0,048
5	40084	0	0	0,04	0	0,6	0	0,02	0	0,34	0	0,56	0,01	0,43	0,03
5	100000	0,03	0	0,97	0	0	0	0	0	0	0	0,97	0,03	0	0,542
5	25177	0,04	0	0	0,03	0	0,93	0	0	0	0,93	0,03	0,03	0,01	0,044
5	49431	0	0,01	0	0,06	0,89	0,03	0	0	0,01	0,04	0	0,06	0,9	0,04
5	66959	0,01	0	0	0,06	0,92	0	0	0	0,01	0	0,55	0,45	0	0,048
5	10384	0	0	0	0,11	0,89	0	0	0	0	0,11	0	0,89	0	0,048
5	100000	0	0	0,99	0,01	0	0	0	0	0	0	1	0	0	0,682
5	2203	0	0	0,04	0,02	0,87	0	0	0,06	0,01	0,16	0,09	0,03	0,72	0,02
5	99871	0,06	0	0	0	0,89	0	0	0,05	0	0	0,91	0,03	0,06	0,048
5	12961	0	0,04	0,01	0,04	0,89	0	0,02	0	0	0,07	0	0,04	0,89	0,048
5	30665	0	0	0,04	0	0	0,01	0	0	0,95	0	0,04	0,01	0,95	0,014
5	63742	0,06	0	0	0	0,14	0	0	0,8	0	0,06	0,14	0	0,8	0,044
5	100000	0,16	0	0,84	0	0	0	0	0	0	0,15	0,82	0	0,03	0,718
5	36224	0	0,08	0,04	0	0,14	0	0	0	0,74	0,08	0,1	0	0,82	0,018
5	41147	0	0,18	0,04	0	0,15	0	0	0	0,63	0	0,04	0	0,96	0,032
5	2495	0	0	0,04	0	0,59	0,15	0,12	0	0,1	0,71	0,05	0,24	0	0,046
5	46787	0,03	0	0,01	0	0,84	0,01	0,09	0,02	0	0,91	0,01	0	0,08	0,042

5	8505	0	0,12	0	0,01	0,35	0,38	0	0	0,14	0,14	0,14	0,66	0,06	0,046
5	10662	0	0,03	0	0,07	0,88	0	0	0,02	0	0,03	0,05	0,49	0,43	0,036
5	71879	0,01	0	0	0,03	0,84	0,11	0,01	0	0	0,04	0,12	0,01	0,83	0,044
5	30819	0	0	0,04	0	0,91	0,01	0	0,02	0,02	0,02	0,02	0,03	0,93	0
5	64907	0	0	0,04	0	0,11	0	0,02	0,33	0,5	0,44	0,04	0	0,52	0,022
5	41396	0	0	0,04	0	0,69	0	0	0	0,27	0,05	0,67	0	0,28	0,01
5	18881	0	0	0	0,06	0,94	0	0	0	0	0	0,95	0,05	0	0,046
5	15222	0,04	0,01	0,04	0,01	0,9	0	0	0	0	0	0,04	0,06	0,9	0,048
5	47946	0,03	0	0,01	0	0,92	0,02	0,02	0	0	0,03	0,05	0	0,92	0,046
5	22338	0	0,01	0	0,07	0,92	0	0	0	0	0,04	0,87	0,09	0	0,034
5	84368	0	0	0,04	0	0,02	0	0	0,07	0,87	0	0,04	0,07	0,89	0,01
5	28854	0	0	0,02	0,07	0,89	0	0	0	0,02	0	0,92	0,08	0	0,046
5	8017	0	0	0	0,13	0,76	0,1	0	0,01	0	0	0,01	0,99	0	0,048
5	72143	0	0	0,04	0	0	0,94	0	0	0,02	0	0,04	0,96	0	0,026
5	23233	0	0,5	0	0	0,48	0,02	0	0	0	0,09	0,47	0,02	0,42	0,048
5	25004	0	0	0,04	0	0,6	0,21	0,01	0	0,14	0,83	0,05	0	0,12	0,024
5	41192	0	0,05	0,04	0	0,01	0,34	0	0,56	0	0,37	0,01	0,62	0	0,048
5	58362	0	0	0	0,06	0,92	0,02	0	0	0	0,05	0	0,01	0,94	0,046
5	23318	0	0,09	0	0,04	0,05	0	0	0,82	0	0,02	0,91	0	0,07	0,048
5	37476	0	0	0,01	0	0,9	0,02	0,07	0	0	0	0,88	0,08	0,04	0,038
	34954,75	0,0103	0,06689	0,06231	0,0256	0,46652	0,13534	0,00805	0,1586	0,0664	0,20712	0,22641	0,27954	0,28692	0,065451

scoring

b	time_to_cc	(C,C)	(C,D)	(C,P)	(D,C)	(D,D)	(D,P)	(P,C)	(P,D)	(P,P)	S(G,G)	S(G,B)	S(B,G)	S(B,B)	
1	14024	0	0	0,01	0,16	0,81	0,01	0	0,01	0	0,81	0,01	0,18	0	0,048
1	2527	0	0,09	0,04	0	0,87	0	0	0	0	0,09	0,04	0,87	0	0,044
1	14769	0,02	0,07	0	0,05	0,86	0	0	0	0	0,01	0,04	0,95	0	0,048
1	55014	0,05	0	0,04	0	0,9	0	0	0,01	0	0	0,05	0,05	0,9	0,042
1	3408	0,07	0	0,01	0	0,74	0,18	0	0	0	0,18	0,01	0,07	0,74	0,048
1	18419	0	0,07	0,01	0	0,81	0,09	0,02	0	0	0,83	0,01	0,06	0,1	0,044
1	14112	0,06	0	0	0	0	0,01	0	0,93	0	0	0,06	0,37	0,57	0,04
1	9875	0,03	0	0,03	0	0,94	0	0	0	0	0,92	0,06	0,02	0	0,046
1	4960	0	0,13	0	0	0,82	0	0,01	0,03	0,01	0	0,01	0,98	0,01	0,044
1	49771	0	0	0,04	0,01	0,9	0,01	0	0,04	0	0,45	0,05	0,5	0	0,036

1	14819	0,06	0,01	0,02	0	0,02	0	0	0,88	0,01	0,01	0,01	0,04	0,94	0,042
1	4520	0,01	0	0,04	0	0	0	0	0,95	0	0,21	0,04	0	0,75	0,024
1	13784	0	0	0	0,2	0,8	0	0	0	0	0,8	0,2	0	0	0,046
1	16104	0,05	0,01	0	0	0,9	0	0	0,04	0	0	0,95	0,04	0,01	0,046
1	7371	0,05	0	0,01	0	0,94	0	0	0	0	0	0,05	0,93	0,02	0,046
1	13444	0,05	0	0	0,01	0,16	0,72	0	0	0,06	0,06	0,05	0,73	0,16	0,048
1	6132	0,02	0,01	0,02	0	0	0,89	0,06	0	0	0,03	0,85	0,1	0,02	0,048
1	23231	0,04	0,96	0	0	0	0	0	0	0	0	0,04	0	0,96	0,046
1	14890	0	0,98	0,01	0,01	0	0	0	0	0	0,04	0,01	0,01	0,94	0,044
1	2943	0,02	0,04	0	0	0,87	0,06	0	0	0,01	0,94	0	0	0,06	0,036
1	16483	0,02	0,35	0	0,01	0,55	0,06	0	0	0,01	0	0,41	0,03	0,56	0,044
1	5575	0	0,16	0	0	0,02	0	0	0,82	0	0	0,82	0,16	0,02	0,042
1	4436	0	0,01	0,01	0,27	0	0,7	0	0,01	0	0,98	0	0,01	0,01	0,048
1	2136	0,02	0,92	0,02	0	0,04	0	0	0	0	0	0,04	0,04	0,92	0,046
1	3207	0	0,98	0,02	0	0	0	0	0	0	0	0	0,06	0,94	0,032
1	59460	0	0,01	0	0,12	0,8	0,07	0	0	0	0,08	0,12	0,8	0	0,046
1	26078	0,05	0	0,01	0	0,7	0	0	0,24	0	0,24	0,71	0	0,05	0,046
1	36282	0	0,07	0,01	0	0,92	0	0	0	0	0,92	0,01	0,07	0	0,048
1	8535	0	0	0,04	0	0,96	0	0	0	0	0,96	0	0	0,04	0,042
1	15340	0,02	0,18	0	0	0,76	0	0	0,04	0	0,06	0,76	0,12	0,06	0,036
1	10030	0	0	0	0,21	0,69	0	0	0,1	0	0,7	0,27	0,03	0	0,048
1	7557	0,03	0	0	0,04	0,92	0	0,01	0	0	0,04	0,01	0,92	0,03	0,046
1	13069	0,02	0,01	0,03	0	0,94	0	0	0	0	0,94	0,04	0,02	0	0,044
1	14923	0,06	0	0,02	0	0,74	0	0	0,16	0,02	0,02	0,1	0,14	0,74	0,044
1	22021	0	0,02	0	0,17	0,77	0,04	0	0	0	0,77	0,02	0,17	0,04	0,044
1	4524	0,03	0	0,04	0	0,93	0	0	0	0	0,93	0	0,06	0,01	0,048
1	20401	0,01	0,96	0	0,01	0	0,01	0,01	0	0	0	0	0,02	0,98	0,038
1	26785	0	0,02	0	0,07	0,81	0,09	0	0	0,01	0,09	0,02	0,07	0,82	0,044
1	12010	0,05	0	0	0	0,78	0,16	0	0	0,01	0,01	0,05	0,94	0	0,046
1	16623	0	0	0	0,22	0	0,42	0	0,36	0	0,59	0,36	0	0,05	0,046
1	5796	0	0	0,01	0,11	0,55	0	0	0,33	0	0,34	0,55	0	0,11	0,046
1	12750	0,03	0,02	0	0	0,94	0	0,01	0	0	0,86	0,03	0,09	0,02	0,046
1	7403	0,05	0,01	0	0	0,03	0,75	0	0,11	0,05	0,02	0,22	0,73	0,03	0,042

1	10838	0,01	0	0,04	0	0,89	0,02	0,04	0	0	0,03	0,73	0,24	0	0,046
1	15923	0,05	0	0	0	0,91	0,04	0	0	0	0,05	0	0,84	0,11	0,048
1	12359	0	0,07	0	0	0,76	0	0	0,16	0,01	0,97	0	0,03	0	0,048
1	16852	0	0	0	0,31	0,69	0	0	0	0	0,99	0,01	0	0	0,048
1	5086	0	0	0,01	0,07	0,92	0	0	0	0	0	0,01	0,06	0,93	0,046
1	36940	0,05	0,02	0	0	0,9	0	0	0,03	0	0	0,03	0,96	0,01	0,048
1	8142	0,05	0	0	0	0,95	0	0	0	0	0,95	0,05	0	0	0,04
1	18365	0,03	0,03	0	0,06	0,85	0,02	0	0	0,01	0,85	0,03	0,06	0,06	0,048
1	32839	0	0,01	0,04	0,03	0,89	0	0	0,03	0	0,76	0,17	0,01	0,06	0,042
1	5471	0,03	0,35	0,02	0	0	0,01	0	0,59	0	0	0,04	0,03	0,93	0,048
1	8391	0	0	0	0,12	0,88	0	0	0	0	0	0	1	0	0,046
1	34353	0	0	0	0,09	0,91	0	0	0	0	0	0	1	0	0,044
1	4802	0	0,11	0	0,05	0,78	0	0	0,06	0	0,08	0,08	0	0,84	0,046
1	12261	0,05	0	0	0	0,85	0,04	0,01	0,05	0	0	0,91	0,05	0,04	0,046
1	40761	0,03	0,02	0	0	0,03	0	0,01	0,91	0	0,94	0,03	0,03	0	0,048
1	14634	0	0,09	0	0,04	0,86	0	0,01	0	0	0,07	0,04	0,89	0	0,046
1	4741	0,01	0,64	0	0	0,35	0	0	0	0	0	1	0	0	0,038
1	7938	0	0,25	0	0	0	0,75	0	0	0	0,25	0	0	0,75	0,046
1	39689	0	0	0	0,1	0,9	0	0	0	0	0,1	0	0,9	0	0,044
1	31185	0	0	0	0,08	0,85	0	0	0,07	0	0,19	0	0,08	0,73	0,048
1	50081	0	0,05	0,01	0	0,81	0,13	0	0	0	0,91	0,07	0,02	0	0,042
1	11625	0	0	0	0,08	0,68	0	0	0,23	0,01	0	0,23	0,45	0,32	0,044
1	41419	0	0,11	0	0,03	0,85	0,01	0	0	0	0	0,11	0,86	0,03	0,048
1	4606	0	0,18	0	0,03	0,79	0	0	0	0	0,18	0,12	0	0,7	0,046
1	54986	0	0	0,04	0	0,27	0	0	0,68	0,01	0,83	0	0,13	0,04	0,038
1	3994	0,01	0,11	0	0	0,88	0	0	0	0	0,11	0,01	0,88	0	0,048
1	18064	0,01	0	0	0,06	0,92	0	0,01	0	0	0,06	0,76	0	0,18	0,042
1	17303	0	0,18	0	0	0,82	0	0	0	0	0,18	0,82	0	0	0,044
1	15104	0	0	0	0,11	0,89	0	0	0	0	0,08	0	0,92	0	0,046
1	37599	0	0	0	0,19	0,71	0,07	0	0	0,03	0,82	0	0,17	0,01	0,042
1	21586	0	0,18	0	0,02	0,78	0	0	0,02	0	0	0,89	0,09	0,02	0,048
1	6235	0,02	0,21	0,04	0	0,08	0,65	0	0	0	0	0,23	0,03	0,74	0,048
1	16028	0	0,13	0	0	0,86	0	0	0	0,01	0	0	1	0	0,044

1	17283	0,01	0	0	0,1	0,48	0	0	0,41	0	0,1	0,41	0,49	0	0,048
1	40056	0,01	0	0	0,06	0,91	0	0	0,02	0	0,02	0,91	0,07	0	0,046
1	12516	0	0,97	0	0	0	0	0	0,03	0	0	1	0	0	0,034
1	6926	0	0,06	0	0	0,9	0,04	0	0	0	0,96	0	0	0,04	0,038
1	11176	0	0,13	0	0	0,74	0,06	0	0,06	0,01	0,23	0,74	0,03	0	0,044
1	41375	0	0	0	0,08	0,91	0	0	0	0,01	0,08	0,01	0,37	0,54	0,048
1	4168	0	0,09	0,04	0	0,69	0	0	0,18	0	0	0,72	0,09	0,19	0,03
1	7787	0	0,93	0	0	0,06	0	0,01	0	0	0,06	0,01	0	0,93	0,046
1	2121	0	0	0,03	0,06	0,89	0	0	0	0,02	0	0,03	0	0,97	0,048
1	35181	0	0	0,04	0	0,73	0,07	0	0,16	0	0	0,2	0,69	0,11	0,026
1	17707	0	0,28	0,01	0	0,71	0	0	0	0	0	0,01	0,28	0,71	0,048
1	2244	0	0,02	0,02	0,11	0,85	0	0	0	0	0,9	0,02	0,08	0	0,044
1	12496	0,04	0	0	0,01	0	0,33	0,04	0,58	0	0,58	0,33	0,04	0,05	0,048
1	4426	0,05	0	0,01	0	0,12	0	0,01	0,81	0	0	0,93	0,01	0,06	0,044
1	17681	0	0	0	0,29	0,71	0	0	0	0	1	0	0	0	0,044
1	16198	0	0	0	0,11	0,74	0,14	0,01	0	0	0,25	0,01	0,74	0	0,046
1	7093	0,01	0,12	0	0	0,73	0,13	0	0,01	0	0	0,01	0,87	0,12	0,048
1	11646	0	0,26	0	0	0,39	0	0	0,35	0	0	0,39	0,26	0,35	0,048
1	12266	0,01	0,05	0	0	0,03	0,9	0	0	0,01	0,9	0,04	0,06	0	0,04
1	11775	0,02	0,02	0,02	0,03	0,9	0,01	0	0	0	0	0,04	0,93	0,03	0,044
1	28595	0,06	0	0	0	0	0,94	0	0	0	0,94	0,06	0	0	0,036
1	25526	0,06	0	0	0	0,04	0	0	0,9	0	0	0,8	0,2	0	0,046
1	17447	0,06	0,01	0	0	0,29	0,64	0	0	0	0,64	0,28	0,06	0,02	0,048
1	5324	0,04	0	0	0,02	0	0	0	0,94	0	0,02	0,94	0	0,04	0,036
1,25	22616	0	0,05	0	0,08	0,84	0,03	0	0	0	0,03	0,84	0,13	0	0,048
1,25	13677	0	0,97	0	0	0	0,03	0	0	0	0,03	0,07	0	0,9	0,04
1,25	8312	0,03	0	0,03	0	0	0,01	0,03	0,9	0	0,69	0,25	0,03	0,03	0,046
1,25	12160	0	0,19	0,02	0	0,57	0	0	0,21	0,01	0,2	0,02	0,19	0,59	0,048
1,25	18448	0	0	0	0,07	0,82	0,03	0	0,08	0	0,08	0,07	0,03	0,82	0,048
1,25	7202	0	0	0,04	0	0	0	0,04	0,91	0,01	0	0,95	0,02	0,03	0,04
1,25	10546	0,04	0	0	0	0	0,94	0,02	0	0	0,04	0,02	0	0,94	0,042
1,25	5953	0,04	0	0	0,03	0,93	0	0	0	0	0,04	0,93	0,03	0	0,048
1,25	6956	0	0,24	0	0	0,76	0	0	0	0	0	0,76	0,24	0	0,048

1,25	3987	0,01	0,27	0	0	0,72	0	0	0	0	0	0,02	0,26	0,72	0,038
1,25	2351	0	0,03	0	0,12	0,84	0	0	0	0,01	0,84	0,02	0,12	0,02	0,048
1,25	4911	0,06	0	0,02	0	0,69	0,05	0	0,17	0,01	0	0,71	0,12	0,17	0,048
1,25	8538	0	0,04	0	0,09	0,78	0,09	0	0	0	0,04	0,09	0,78	0,09	0,048
1,25	9061	0	0,03	0	0,13	0,84	0	0	0	0	0,87	0	0	0,13	0,044
1,25	24340	0	0	0,03	0,06	0,71	0	0	0,2	0	0,25	0,54	0,02	0,19	0,038
1,25	10414	0	0,07	0	0,01	0,92	0	0	0	0	0,8	0	0,19	0,01	0,046
1,25	52083	0,01	0,09	0	0	0	0,9	0	0	0	0,01	0,11	0,88	0	0,046
1,25	7104	0	0,21	0	0	0,69	0,02	0,02	0,06	0	0	0,01	0,29	0,7	0,044
1,25	10244	0,04	0	0,01	0	0,95	0	0	0	0	0	0,03	0,95	0,02	0,046
1,25	25381	0,06	0	0	0	0,64	0,29	0,01	0	0	0,64	0	0	0,36	0,042
1,25	3286	0,01	0	0,04	0	0,93	0,01	0	0,01	0	0,01	0,05	0,01	0,93	0,014
1,25	15393	0	0,14	0	0	0	0	0,01	0,85	0	0,14	0,86	0	0	0,046
1,25	3912	0,04	0	0,01	0	0,88	0,07	0	0	0	0,57	0,32	0,07	0,04	0,046
1,25	18353	0,01	0	0,04	0,01	0	0	0,03	0,91	0	0,02	0,02	0	0,96	0,044
1,25	19582	0	0,56	0	0,01	0,42	0	0	0,01	0	0	0,98	0,01	0,01	0,046
1,25	2836	0,06	0	0,01	0	0,93	0	0	0	0	0,94	0,06	0	0	0,046
1,25	29129	0	0	0	0,13	0,77	0,04	0	0,05	0,01	0,1	0,04	0,86	0	0,046
1,25	21466	0,01	0,06	0	0	0,91	0	0,02	0	0	0,92	0,02	0,06	0	0,042
1,25	17970	0,05	0	0	0,01	0,04	0,87	0,03	0	0	0,9	0,05	0,01	0,04	0,038
1,25	11117	0	0,01	0	0,06	0,85	0	0	0,08	0	0,08	0,86	0,01	0,05	0,046
1,25	13329	0,04	0,03	0	0	0,34	0,05	0	0,5	0,04	0,04	0,07	0,86	0,03	0,042
1,25	11965	0,07	0	0	0	0,87	0,04	0	0,01	0,01	0,06	0,87	0,07	0	0,048
1,25	5270	0,01	0,06	0	0	0,92	0,01	0	0	0	0,98	0,01	0	0,01	0,048
1,25	8706	0	0,03	0	0,16	0	0,74	0	0	0,07	0,74	0,16	0,1	0	0,042
1,25	15366	0	0,08	0	0,04	0,87	0,01	0	0	0	0,01	0	0,97	0,02	0,048
1,25	3455	0,03	0	0	0	0	0	0,02	0,95	0	0	0	0,03	0,97	0,04
1,25	7438	0,01	0	0	0,12	0,87	0	0	0	0	0	0,13	0,87	0	0,048
1,25	12283	0,06	0,02	0,02	0	0,57	0	0	0,33	0	0,35	0,02	0	0,63	0,046
1,25	8685	0	0	0	0,17	0,82	0	0,01	0	0	0,73	0	0,25	0,02	0,046
1,25	9240	0,05	0,01	0	0	0,93	0	0,01	0	0	0	0,01	0	0,99	0,04
1,25	14904	0,04	0	0,01	0,03	0,92	0	0	0	0	0,92	0,07	0	0,01	0,03
1,25	3144	0,05	0	0	0	0,91	0,04	0	0	0	0,01	0,91	0,04	0,04	0,048

1,25	18264	0	0	0	0,1	0,73	0	0	0,17	0	0,17	0,73	0	0,1	0,046
1,25	19728	0,01	0	0,02	0,13	0	0,78	0	0,03	0,03	0,83	0,01	0,02	0,14	0,046
1,25	13587	0	0,1	0	0,01	0,88	0,01	0	0	0	0,51	0	0,48	0,01	0,048
1,25	13089	0	0	0	0,11	0	0,18	0	0,71	0	0,05	0,36	0,59	0	0,038
1,25	5681	0,05	0	0	0	0,95	0	0	0	0	0,95	0	0,05	0	0,046
1,25	9341	0,05	0,01	0	0	0,93	0	0	0,01	0	0	0,02	0,93	0,05	0,038
1,25	15449	0,04	0	0,01	0	0,53	0,42	0	0	0	0,53	0,01	0,44	0,02	0,046
1,25	19117	0	0,95	0	0,02	0	0,02	0	0,01	0	0,04	0	0	0,96	0,048
1,25	18130	0,06	0	0	0	0,94	0	0	0	0	0	0,06	0	0,94	0,048
1,25	8038	0	0,22	0	0,01	0,76	0	0	0,01	0	0,01	0,76	0,22	0,01	0,046
1,25	28634	0,04	0,08	0	0	0,87	0	0,01	0	0	0,04	0,33	0	0,63	0,044
1,25	9346	0,05	0,02	0	0	0,79	0,14	0	0	0	0,69	0,26	0	0,05	0,048
1,25	13893	0	0,22	0	0	0,76	0,01	0,01	0	0	0,29	0	0,12	0,59	0,034
1,25	6215	0,05	0	0,01	0	0,94	0	0	0	0	0	0,01	0,99	0	0,048
1,25	11962	0	0,07	0	0	0,92	0	0	0,01	0	0,92	0	0,08	0	0,046
1,25	13400	0	0	0	0,12	0,88	0	0	0	0	0	0	1	0	0,046
1,25	6463	0,04	0,61	0,01	0	0	0,01	0	0,32	0,01	0,01	0,05	0,01	0,93	0,048
1,25	12587	0	0,22	0	0,02	0,76	0	0	0	0	0,22	0	0,01	0,77	0,042
1,25	1650	0	0	0,04	0,02	0	0	0,05	0,89	0	0,05	0,04	0,32	0,59	0,048
1,25	15482	0	0,94	0	0	0	0	0	0,04	0,02	0,06	0,02	0	0,92	0,04
1,25	8324	0,04	0,05	0,01	0	0,74	0,16	0	0	0	0,58	0,01	0,04	0,37	0,048
1,25	18567	0	0,66	0	0	0,34	0	0	0	0	0	0,61	0,05	0,34	0,048
1,25	14944	0	0,19	0	0,02	0,79	0	0	0	0	0,03	0,79	0,14	0,04	0,042
1,25	13355	0	0,09	0	0,01	0,87	0	0,01	0	0,02	0,11	0	0,88	0,01	0,038
1,25	6669	0,03	0	0	0,04	0,92	0,01	0	0	0	0,01	0,91	0,05	0,03	0,046
1,25	3299	0,02	0,04	0,01	0,01	0,91	0	0	0,01	0	0,91	0,01	0,02	0,06	0,038
1,25	6225	0,01	0	0,04	0	0,93	0,01	0	0	0,01	0,02	0,97	0,01	0	0,016
1,25	9873	0	0	0	0,06	0,94	0	0	0	0	0	0,94	0,06	0	0,046
1,25	11484	0,06	0	0	0	0,02	0,83	0	0,09	0	0,65	0,02	0,09	0,24	0,044
1,25	38039	0	0	0	0,07	0,89	0,04	0	0	0	0,07	0,03	0,04	0,86	0,048
1,25	10441	0	0	0	0,22	0,76	0	0	0,02	0	0,76	0,22	0,02	0	0,032
1,25	20361	0,04	0	0	0	0,92	0	0	0	0,04	0	0	0,04	0,96	0,048
1,25	11498	0,01	0,15	0,01	0	0,83	0	0	0	0	0,05	0,1	0,85	0	0,046

1,25	21029	0	0,11	0,01	0	0	0,88	0	0	0	0	0,98	0,01	0,01	0,048
1,25	7794	0	0,03	0,04	0	0,93	0	0	0	0	0,04	0,02	0,93	0,01	0,044
1,25	32384	0,04	0	0	0	0	0,93	0,02	0,01	0	0,03	0,02	0,93	0,02	0,046
1,25	27093	0	0,21	0	0	0,79	0	0	0	0	0	0,82	0,18	0	0,048
1,25	7988	0,01	0	0,04	0	0,93	0	0,01	0,01	0	0,93	0,06	0,01	0	0,036
1,25	18606	0	0,12	0	0	0,83	0,03	0,01	0,01	0	0,04	0,13	0,82	0,01	0,044
1,25	45965	0	0,17	0,01	0,01	0,73	0,01	0	0,07	0	0	0,08	0,9	0,02	0,046
1,25	10146	0	0,02	0	0,07	0,9	0	0	0,01	0	0,1	0,9	0	0	0,044
1,25	7369	0	0,14	0	0	0,78	0,01	0	0,07	0	0,02	0,02	0,85	0,11	0,046
1,25	7462	0	0,03	0	0,08	0,88	0	0	0,01	0	0,03	0,03	0,93	0,01	0,042
1,25	6257	0,03	0	0	0,03	0	0	0,02	0,92	0	0,53	0	0,07	0,4	0,048
1,25	39232	0,02	0,02	0,01	0,08	0,07	0,34	0	0,46	0	0,46	0,44	0,02	0,08	0,046
1,25	7949	0	0,29	0,01	0	0,6	0,05	0	0,05	0	0,04	0,05	0,3	0,61	0,048
1,25	3191	0,05	0	0	0	0,9	0,01	0,04	0	0	0,94	0,01	0	0,05	0,048
1,25	19739	0,05	0	0,01	0	0,03	0,23	0	0,68	0	0,01	0	0,91	0,08	0,048
1,25	17188	0,02	0	0	0,13	0,58	0,03	0	0,22	0,02	0,53	0,22	0,24	0,01	0,046
1,25	18667	0,01	0,46	0,02	0,02	0,49	0	0	0	0	0	0,47	0,03	0,5	0,048
1,25	21977	0,01	0,17	0	0,02	0,78	0	0	0,02	0	0	0,8	0,19	0,01	0,048
1,25	3409	0,03	0,11	0	0	0,77	0	0	0,07	0,02	0,05	0,81	0,11	0,03	0,046
1,25	18078	0	0,08	0	0	0,91	0	0	0	0,01	0,93	0	0,07	0	0,042
1,25	5350	0,06	0	0	0	0,37	0	0,01	0,52	0,04	0,29	0,66	0,01	0,04	0,048
1,25	22200	0	0,03	0,04	0	0,04	0,79	0	0,04	0,06	0,6	0,12	0	0,28	0,04
1,25	5574	0	0	0,04	0	0,04	0,83	0	0,09	0	0,08	0,05	0,83	0,04	0,02
1,25	3333	0	0,06	0	0	0,87	0	0,07	0	0	0,1	0,03	0,87	0	0,044
1,25	14109	0	0,09	0,04	0	0,01	0,05	0	0,81	0	0,12	0,87	0	0,01	0,044
1,5	19407	0,01	0	0,04	0	0,03	0	0	0,92	0	0,03	0,02	0,95	0	0,04
1,5	9889	0,01	0	0	0,05	0,93	0,01	0	0	0	0	0,98	0,01	0,01	0,048
1,5	7338	0	0	0	0,1	0,85	0	0	0,05	0	0	0,1	0,9	0	0,048
1,5	10021	0,06	0	0	0	0,36	0,56	0	0	0,02	0,36	0,06	0,56	0,02	0,044
1,5	20408	0,03	0,03	0,04	0	0,88	0	0	0	0,02	0,05	0,01	0,92	0,02	0,046
1,5	9841	0	0,08	0	0,05	0,87	0	0	0	0	0,07	0,9	0,02	0,01	0,048
1,5	46697	0,03	0,01	0,01	0	0	0,87	0,01	0,01	0,06	0,9	0,01	0,06	0,03	0,046
1,5	21269	0,03	0	0	0,03	0,94	0	0	0	0	0,13	0,81	0,03	0,03	0,048

1,5	15490	0	0,22	0	0,01	0,62	0	0	0,13	0,02	0,22	0,08	0,04	0,66	0,046
1,5	14663	0,06	0	0	0	0,94	0	0	0	0	0,04	0,02	0	0,94	0,048
1,5	48390	0	0,05	0,04	0	0,87	0	0	0,04	0	0,09	0,22	0	0,69	0,01
1,5	8749	0	0	0	0,22	0,78	0	0	0	0	0,78	0,22	0	0	0,04
1,5	33798	0,05	0	0	0	0	0,95	0	0	0	0,95	0,05	0	0	0,04
1,5	17619	0	0	0	0,1	0	0,02	0	0,88	0	0,02	0,1	0,88	0	0,044
1,5	4856	0,01	0	0	0,11	0	0	0	0,88	0	0,03	0,08	0,83	0,06	0,048
1,5	2271	0,04	0	0	0	0,96	0	0	0	0	0	0,96	0,04	0	0,042
1,5	22167	0	1	0	0	0	0	0	0	0	0	1	0	0	0,016
1,5	15123	0	0	0	0,36	0,64	0	0	0	0	1	0	0	0	0,044
1,5	26142	0,04	0	0	0,01	0,66	0,25	0	0,04	0	0,29	0,66	0	0,05	0,042
1,5	3643	0,03	0	0,02	0	0,95	0	0	0	0	0,95	0	0,03	0,02	0,046
1,5	15114	0	0,83	0	0	0,17	0	0	0	0	0	1	0	0	0,042
1,5	13972	0,01	0	0	0,07	0,91	0,01	0	0	0	0,02	0,98	0	0	0,046
1,5	7404	0,01	0,09	0,01	0	0,89	0	0	0	0	0,09	0,01	0,9	0	0,048
1,5	26764	0,03	0	0	0,09	0	0,88	0	0	0	0,91	0	0,09	0	0,036
1,5	2183	0,05	0	0,02	0	0,92	0,01	0	0	0	0,01	0,02	0,02	0,95	0,046
1,5	21752	0,05	0	0	0	0,44	0,12	0	0,39	0	0	0,49	0,01	0,5	0,044
1,5	28209	0,04	0	0,02	0	0,88	0,06	0	0	0	0,88	0,01	0,09	0,02	0,046
1,5	33325	0,03	0	0	0,07	0	0,9	0	0	0	0,67	0,03	0,3	0	0,04
1,5	16789	0	0,97	0	0,03	0	0	0	0	0	0	0	0,03	0,97	0,044
1,5	10737	0,06	0	0	0	0,94	0	0	0	0	0	1	0	0	0,04
1,5	9877	0,05	0	0	0	0,89	0,01	0	0,05	0	0,01	0	0,93	0,06	0,046
1,5	2634	0,06	0	0	0	0,31	0	0	0,63	0	0	0	0	1	0,044
1,5	30170	0,04	0,03	0,03	0	0,6	0	0	0,3	0	0,03	0,61	0,3	0,06	0,046
1,5	17144	0	0	0	0,08	0,87	0,04	0	0	0,01	0,01	0,85	0,08	0,06	0,048
1,5	18536	0	0	0,01	0,11	0,88	0	0	0	0	0	0,11	0,88	0,01	0,044
1,5	10660	0	0,97	0,01	0	0	0,02	0	0	0	0	0,69	0,02	0,29	0,04
1,5	44546	0,05	0	0	0	0,03	0	0	0,92	0	0	0,08	0	0,92	0,042
1,5	21017	0	0	0	0,19	0,79	0	0	0,02	0	0,75	0,19	0	0,06	0,048
1,5	11310	0,04	0	0,04	0	0,01	0,87	0,01	0,01	0,02	0,04	0,07	0,89	0	0,048
1,5	9256	0,05	0	0,01	0	0	0,94	0	0	0	1	0	0	0	0,048
1,5	13895	0,02	0,01	0	0,03	0,93	0	0	0	0,01	0,04	0	0,01	0,95	0,048

1,5	11557	0,01	0,9	0,03	0	0,03	0	0,01	0,02	0	0,03	0,01	0,01	0,95	0,038
1,5	11285	0,03	0,02	0	0	0,92	0	0,02	0,01	0	0,03	0,92	0,04	0,01	0,048
1,5	26454	0	0,01	0	0,18	0,79	0,01	0,01	0	0	0,79	0	0,19	0,02	0,042
1,5	16118	0,01	0	0,04	0	0,79	0,12	0,04	0	0	0,79	0,13	0,04	0,04	0,036
1,5	23787	0,04	0	0	0,03	0,93	0	0	0	0	0,03	0,96	0	0,01	0,042
1,5	10935	0	0	0	0,1	0,88	0	0	0,01	0,01	0,11	0	0,88	0,01	0,046
1,5	6360	0	0	0	0,04	0,96	0	0	0	0	0	0,02	0,02	0,96	0,044
1,5	35582	0	0,08	0	0	0,92	0	0	0	0	0,92	0	0,08	0	0,048
1,5	7404	0	0	0	0,12	0,85	0,03	0	0	0	0,01	0,12	0,87	0	0,044
1,5	25462	0	0,03	0	0,08	0	0	0	0,89	0	0	0,89	0,11	0	0,044
1,5	12064	0,03	0,18	0	0	0,79	0	0	0	0	0,18	0,12	0	0,7	0,048
1,5	9626	0	0	0	0,06	0,94	0	0	0	0	0,01	0,99	0	0	0,044
1,5	22744	0	0	0	0,08	0,92	0	0	0	0	0,08	0,92	0	0	0,042
1,5	12369	0	0,91	0	0	0	0,01	0	0,08	0	0,08	0,01	0	0,91	0,048
1,5	7224	0	0,16	0	0,01	0,83	0	0	0	0	0,01	0,82	0,16	0,01	0,036
1,5	23556	0	0,08	0	0	0,91	0,01	0	0	0	0,77	0	0,23	0	0,046
1,5	4082	0,01	0,07	0	0	0,9	0	0	0,02	0	0,94	0,02	0	0,04	0,046
1,5	6684	0	0,78	0	0	0	0,21	0	0,01	0	0,03	0,09	0	0,88	0,04
1,5	18803	0,02	0	0,04	0	0,93	0	0,01	0	0	0	0,94	0,06	0	0,046
1,5	5933	0	0	0,02	0,08	0,87	0,01	0	0	0,02	0,01	0,9	0,08	0,01	0,048
1,5	23338	0	0	0,01	0,11	0,64	0,04	0	0,2	0	0,64	0	0,04	0,32	0,044
1,5	5090	0,01	0,27	0	0	0,67	0,02	0	0	0,03	0,05	0,67	0,03	0,25	0,028
1,5	5875	0,04	0,01	0,04	0	0,69	0	0	0,22	0	0,02	0,05	0,22	0,71	0,038
1,5	42691	0	0,01	0	0,11	0,85	0,02	0	0	0,01	0,03	0,11	0,85	0,01	0,048
1,5	7180	0	0,08	0	0	0,91	0,01	0	0	0	0,91	0	0,08	0,01	0,046
1,5	21125	0,02	0,82	0,04	0,01	0	0	0	0,11	0	0	0,16	0	0,84	0,046
1,5	8195	0	0	0,04	0	0	0	0	0,96	0	0	0,04	0,96	0	0,022
1,5	31554	0,02	0,04	0,04	0	0,87	0,01	0,01	0	0,01	0,02	0,9	0,02	0,06	0,04
1,5	7157	0,06	0	0	0	0,94	0	0	0	0	0,93	0,02	0	0,05	0,046
1,5	19085	0	0,13	0	0	0	0	0	0,87	0	0	0,13	0,87	0	0,048
1,5	26178	0	1	0	0	0	0	0	0	0	0	1	0	0	0,038
1,5	41021	0	1	0	0	0	0	0	0	0	0	1	0	0	0,038
1,5	9468	0	0	0,04	0	0	0,23	0	0,67	0,06	0,59	0	0,04	0,37	0,02

1,5	49064	0,04	0	0	0	0,61	0,3	0,02	0,03	0	0,34	0,05	0,61	0	0,038
1,5	4887	0,05	0	0,01	0	0,94	0	0	0	0	0	0,94	0,01	0,05	0,046
1,5	20922	0	0	0	0,06	0,94	0	0	0	0	0,06	0	0	0,94	0,048
1,5	36346	0,02	0	0	0,01	0	0,22	0,04	0,71	0	0,06	0,04	0	0,9	0,046
1,5	4350	0,02	0	0	0,1	0,77	0	0	0	0,11	0,79	0,11	0	0,1	0,042
1,5	4390	0,05	0,06	0,03	0	0,86	0	0	0	0	0,05	0	0,03	0,92	0,048
1,5	6340	0	0,13	0	0	0,86	0,01	0	0	0	0,01	0,13	0,86	0	0,042
1,5	11360	0,04	0	0	0,02	0,92	0	0	0,02	0	0,04	0,02	0,02	0,92	0,048
1,5	6897	0,03	0	0,04	0	0	0	0,03	0,89	0,01	0,01	0,05	0	0,94	0,048
1,5	7434	0,03	0,88	0	0	0,01	0,08	0	0	0	0	0,01	0	0,99	0,034
1,5	21247	0	0,97	0	0,02	0	0	0	0,01	0	0	0	0,03	0,97	0,042
1,5	10824	0	0,05	0,04	0	0,88	0,03	0	0	0	0,93	0,04	0,03	0	0,042
1,5	27968	0,05	0	0	0	0	0	0	0,95	0	0,05	0	0	0,95	0,048
1,5	14014	0,03	0,04	0	0,01	0,91	0	0,01	0	0	0	0,04	0	0,96	0,042
1,5	37707	0	0,01	0	0,18	0,7	0,1	0	0	0,01	0,81	0,01	0	0,18	0,042
1,5	5275	0	0,95	0,04	0	0	0	0,01	0	0	0	0,04	0	0,96	0,022
1,5	24121	0	0	0,04	0	0,95	0	0	0	0,01	0,93	0	0,07	0	0,022
1,5	15905	0	0,03	0	0,13	0,84	0	0	0	0	0,86	0	0,02	0,12	0,044
1,5	11939	0	0	0	0,22	0,78	0	0	0	0	0,78	0	0,22	0	0,044
1,5	13135	0	0	0,03	0,02	0,92	0	0,03	0	0	0,02	0,03	0,03	0,92	0,042
1,5	27551	0,04	0	0,04	0,02	0,9	0	0	0	0	0	0,94	0,06	0	0,044
1,5	10225	0	0	0,04	0	0,9	0	0	0,06	0	0	0,03	0,97	0	0,028
1,5	18837	0	0,03	0	0,15	0,73	0,09	0	0	0	0,82	0	0,03	0,15	0,046
1,5	37276	0,01	0	0	0,16	0,83	0	0	0	0	0,73	0,17	0,01	0,09	0,046
1,5	19409	0,06	0	0	0	0,94	0	0	0	0	0,01	0,02	0,06	0,91	0,046
1,5	31804	0,05	0	0	0,01	0,91	0	0	0	0,03	0	0,93	0,06	0,01	0,034
1,75	17323	0	0,05	0,04	0	0,89	0	0,01	0,01	0	0,05	0,04	0,9	0,01	0,042
1,75	16899	0	0,18	0,03	0	0,74	0,05	0	0	0	0,16	0,78	0,02	0,04	0,048
1,75	8860	0	0	0,01	0,07	0,92	0	0	0	0	0,08	0,92	0	0	0,048
1,75	8783	0,02	0	0	0	0,94	0	0,04	0	0	0	0,95	0,03	0,02	0,046
1,75	26720	0	0,02	0	0,07	0,91	0	0	0	0	0	0,91	0,07	0,02	0,044
1,75	9553	0,04	0,12	0	0	0,84	0	0	0	0	0,12	0,87	0,01	0	0,048
1,75	16175	0	0	0	0,05	0,95	0	0	0	0	0,05	0,95	0	0	0,046

1,75	14024	0,01	0,23	0	0	0,75	0	0,01	0	0	0,01	0,75	0,13	0,11	0,038
1,75	14093	0,02	0,01	0,01	0	0,92	0	0,04	0	0	0,03	0,02	0,93	0,02	0,04
1,75	36729	0,02	0,96	0	0	0,02	0	0	0	0	0,02	0,02	0	0,96	0,036
1,75	17991	0,01	0	0	0,14	0,85	0	0	0	0	0,78	0,07	0,01	0,14	0,046
1,75	9765	0,06	0	0	0	0,94	0	0	0	0	0	0,06	0,94	0	0,046
1,75	11055	0	0,01	0	0,11	0,71	0,06	0,01	0,1	0	0,2	0	0,79	0,01	0,046
1,75	26528	0	0,14	0	0	0,84	0	0,01	0	0,01	0	0	0,98	0,02	0,038
1,75	24723	0	0,28	0	0	0	0	0	0,72	0	0	0	0,28	0,72	0,048
1,75	24571	0	0,33	0,01	0,01	0,62	0	0	0	0,03	0,02	0,24	0,1	0,64	0,042
1,75	7844	0,03	0	0	0	0,45	0,47	0,05	0	0	0,52	0,03	0	0,45	0,046
1,75	6805	0	0,13	0	0	0,03	0	0	0,7	0,14	0,01	0,03	0,83	0,13	0,044
1,75	28611	0,03	0	0,04	0	0,8	0	0,01	0,12	0	0	0,84	0,13	0,03	0,048
1,75	30515	0,04	0	0,02	0	0,92	0	0	0	0,02	0	0,02	0,96	0,02	0,046
1,75	22670	0	0	0	0,07	0,93	0	0	0	0	0	0	0,12	0,88	0,044
1,75	15138	0	0	0	0,21	0,76	0	0	0,02	0,01	0,78	0	0,18	0,04	0,044
1,75	4194	0,06	0	0,03	0	0	0,07	0	0,84	0	0	0,1	0	0,9	0,048
1,75	8815	0,06	0	0	0	0,94	0	0	0	0	0,94	0,06	0	0	0,048
1,75	12725	0	0	0,04	0	0,95	0	0	0,01	0	0,91	0,04	0,05	0	0,03
1,75	37757	0,04	0	0,01	0,01	0,87	0,07	0	0	0	0,04	0,86	0	0,1	0,046
1,75	10428	0	0	0	0,11	0,09	0,01	0	0,79	0	0	0,01	0,83	0,16	0,048
1,75	10317	0	1	0	0	0	0	0	0	0	0	1	0	0	0,028
1,75	19012	0	0,21	0	0	0,79	0	0	0	0	0	0,79	0,21	0	0,042
1,75	8809	0	0	0	0,09	0,41	0,21	0	0,28	0,01	0	0,5	0,22	0,28	0,048
1,75	6443	0,05	0	0,01	0,01	0,89	0,02	0	0,02	0	0	0	0,91	0,09	0,046
1,75	7014	0	0,29	0,04	0	0,14	0,01	0	0,52	0	0	0,01	0,33	0,66	0,046
1,75	56818	0	0	0	0,07	0,92	0,01	0	0	0	0,07	0	0,01	0,92	0,046
1,75	18939	0,02	0,15	0	0	0,81	0,02	0	0	0	0	0,81	0,17	0,02	0,042
1,75	13379	0	0	0	0,05	0,95	0	0	0	0	0,05	0	0	0,95	0,042
1,75	24971	0,01	0,06	0,01	0	0,03	0,88	0	0,01	0	0,92	0	0,07	0,01	0,046
1,75	4803	0	0,22	0,01	0	0,77	0	0	0	0	0	0,78	0,22	0	0,044
1,75	32712	0	0,25	0	0	0	0,12	0	0,63	0	0	0,63	0,25	0,12	0,048
1,75	8675	0,04	0	0,01	0,02	0,08	0,19	0	0,66	0	0	0,7	0,11	0,19	0,042
1,75	19338	0	0	0	0,07	0,84	0,08	0,01	0	0	0	0,85	0,05	0,1	0,044

1,75	18784	0,03	0,03	0,02	0	0,59	0,01	0	0,32	0	0,64	0	0,02	0,34	0,038
1,75	26957	0,03	0,06	0	0	0,91	0	0	0	0	0,31	0,69	0	0	0,048
1,75	9518	0,06	0	0	0	0,03	0,11	0	0,8	0	0,05	0,8	0	0,15	0,046
1,75	22459	0	0,08	0,01	0	0,17	0,03	0,01	0,65	0,05	0,73	0,09	0	0,18	0,048
1,75	16792	0,04	0	0,04	0	0,92	0	0	0	0	0	0,08	0,92	0	0,046
1,75	16422	0,06	0	0	0	0,92	0	0	0,01	0,01	0,92	0,06	0,02	0	0,048
1,75	35229	0	0	0	0,11	0,89	0	0	0	0	0,11	0	0,89	0	0,048
1,75	14010	0	0,6	0	0	0,4	0	0	0	0	0,08	0,4	0,01	0,51	0,048
1,75	23621	0,05	0	0	0	0,91	0,04	0	0	0	0,96	0	0,04	0	0,042
1,75	4857	0,02	0	0,03	0,09	0,78	0,02	0,01	0,05	0	0,89	0,03	0	0,08	0,048
1,75	27833	0	0	0	0,11	0,84	0,05	0	0	0	0,02	0	0,84	0,14	0,044
1,75	14384	0,07	0	0	0	0,87	0	0	0,06	0	0,02	0,84	0,01	0,13	0,048
1,75	15297	0,02	0,05	0,01	0	0,86	0	0,01	0,05	0	0,07	0,03	0,84	0,06	0,048
1,75	18674	0,06	0	0	0	0,93	0	0	0	0,01	0,93	0	0,07	0	0,044
1,75	4427	0,02	0,05	0	0	0,93	0	0	0	0	1	0	0	0	0,042
1,75	32831	0,02	0	0	0	0,11	0,8	0,07	0	0	0	0,11	0,82	0,07	0,046
1,75	25935	0,04	0,21	0,01	0	0,74	0	0	0	0	0,04	0,01	0	0,95	0,048
1,75	11458	0,04	0	0,01	0,02	0,91	0	0	0,01	0,01	0,01	0,06	0,92	0,01	0,038
1,75	10788	0	0	0	0,11	0,73	0,14	0	0	0,02	0,11	0,02	0,73	0,14	0,046
1,75	10778	0,04	0	0,02	0	0,77	0,1	0	0,05	0,02	0,8	0,02	0,07	0,11	0,048
1,75	28459	0,03	0	0,04	0	0,82	0,11	0	0	0	0	0,14	0,76	0,1	0,048
1,75	20555	0	0,01	0,01	0,21	0,77	0	0	0	0	0,77	0	0,22	0,01	0,048
1,75	32088	0,02	0,02	0	0,02	0	0	0,03	0,88	0,03	0,01	0,97	0,02	0	0,048
1,75	8600	0	0	0	0,07	0	0,11	0	0,82	0	0	0,24	0,11	0,65	0,048
1,75	28470	0	0	0	0,09	0,88	0	0	0,03	0	0,03	0	0,97	0	0,042
1,75	14170	0,04	0,02	0	0	0,92	0	0	0	0,02	0	0,04	0,96	0	0,048
1,75	15653	0,06	0	0	0	0	0	0	0,94	0	0,94	0	0,04	0,02	0,042
1,75	55766	0	0	0	0,14	0,85	0	0	0	0,01	0,14	0	0,84	0,02	0,04
1,75	9521	0,03	0	0,01	0	0,06	0,84	0,05	0,01	0	0,09	0	0,85	0,06	0,044
1,75	6696	0	0,22	0,03	0	0,69	0,02	0	0,04	0	0,05	0,9	0,05	0	0,046
1,75	21602	0	0,01	0,04	0,07	0,12	0,76	0	0	0	0,18	0,14	0,68	0	0,038
1,75	8387	0	0	0,04	0,01	0	0,26	0	0,69	0	0	0,28	0,72	0	0,026
1,75	6806	0,05	0	0	0	0,94	0	0	0	0,01	0	0,04	0,02	0,94	0,042

1,75	4952	0	1	0	0	0	0	0	0	0	0	1	0	0	0,028
1,75	1833	0,01	0	0,02	0,06	0,9	0	0	0	0,01	0,01	0,96	0,02	0,01	0,038
1,75	5116	0,01	0,07	0,03	0	0,89	0	0	0	0	0	0,03	0,97	0	0,046
1,75	32605	0,02	0	0	0,05	0	0	0	0,9	0,03	0,02	0,93	0	0,05	0,046
1,75	6791	0,04	0,05	0,04	0	0	0	0	0,87	0	0,05	0,1	0	0,85	0,034
1,75	8373	0	0	0	0,19	0,79	0	0,01	0	0,01	0,79	0,15	0,05	0,01	0,046
1,75	44513	0,04	0	0,03	0	0,75	0	0,01	0,16	0,01	0,17	0,78	0,01	0,04	0,046
1,75	4020	0	0	0,01	0,06	0	0,01	0	0,92	0	0	0,98	0,01	0,01	0,048
1,75	2613	0	0,08	0	0	0,92	0	0	0	0	0,92	0	0,08	0	0,048
1,75	7522	0,05	0	0,02	0	0	0,9	0	0,03	0	0	0,02	0,93	0,05	0,048
1,75	23150	0	0	0	0,06	0,92	0	0,01	0	0,01	0,06	0,93	0	0,01	0,048
1,75	12596	0,03	0,02	0,03	0	0,86	0,02	0,03	0	0,01	0,89	0,04	0,06	0,01	0,048
1,75	30321	0,01	0	0	0,05	0,8	0,14	0	0	0	0	0,94	0,05	0,01	0,046
1,75	25783	0,04	0,01	0	0	0,56	0,38	0,01	0	0	0,22	0,01	0,76	0,01	0,042
1,75	15031	0	0,11	0	0	0,89	0	0	0	0	0,04	0,06	0,89	0,01	0,04
1,75	32490	0	0,02	0	0,21	0,77	0	0	0	0	0,98	0	0	0,02	0,048
1,75	10640	0,05	0	0	0	0,95	0	0	0	0	0	0,93	0,07	0	0,046
1,75	11585	0	0,11	0	0	0	0	0,01	0,88	0	0,11	0	0,89	0	0,038
1,75	10640	0,06	0	0	0	0	0	0	0,9	0,04	0,05	0,94	0,01	0	0,048
1,75	15533	0,02	0,02	0,04	0	0,92	0	0	0	0	0,05	0,92	0,01	0,02	0,038
1,75	9805	0,05	0	0,04	0	0,9	0	0	0,01	0	0,06	0,92	0,02	0	0,048
1,75	20817	0	0,34	0	0	0,59	0,03	0	0,04	0	0,13	0,59	0,04	0,24	0,046
1,75	17882	0	0	0	0,13	0,01	0	0	0,86	0	0	0,01	0,99	0	0,042
1,75	9386	0,06	0	0	0	0,94	0	0	0	0	0	0,73	0,02	0,25	0,048
1,75	30421	0,05	0	0	0	0,38	0,3	0	0,26	0,01	0	0,69	0	0,31	0,046
1,75	7681	0	0,24	0	0,02	0,74	0	0	0	0	0,11	0,74	0	0,15	0,048
1,75	31492	0,07	0	0	0	0,89	0	0	0,04	0	0,93	0	0,06	0,01	0,044
2	10600	0	0,02	0	0,08	0,87	0,02	0	0,01	0	0,06	0,8	0,12	0,02	0,048
2	13463	0	0,06	0	0,04	0,89	0	0	0,01	0	0,01	0	0,97	0,02	0,046
2	24557	0	0,07	0	0,06	0,87	0	0	0	0	0,07	0,51	0	0,42	0,048
2	10587	0	0	0	0,08	0,91	0,01	0	0	0	0	0,91	0,08	0,01	0,046
2	4915	0,03	0	0,04	0	0,92	0	0,01	0	0	0,93	0,04	0,03	0	0,046
2	17609	0	0	0,04	0,05	0,91	0	0	0	0	0,05	0,04	0,91	0	0,046

2	29824	0	0,52	0	0	0,23	0,24	0	0	0,01	0,01	0,52	0	0,47	0,044
2	4805	0,03	0	0,01	0,03	0,93	0	0	0	0	0,76	0,2	0	0,04	0,048
2	11704	0,01	0,59	0,03	0	0,37	0	0	0	0	0	0,37	0,06	0,57	0,044
2	9377	0	1	0	0	0	0	0	0	0	0	1	0	0	0,018
2	28805	0	0	0	0,06	0,94	0	0	0	0	0,06	0	0	0,94	0,048
2	11829	0	0,03	0,04	0	0	0	0	0,93	0	0	0,51	0,04	0,45	0,006
2	23363	0,01	0	0	0,14	0,8	0,04	0	0,01	0	0,78	0,07	0	0,15	0,048
2	18890	0	0	0	0,09	0,89	0	0	0	0,02	0,04	0,89	0,06	0,01	0,044
2	30323	0	0,19	0	0,01	0,79	0	0	0	0,01	0,02	0,79	0,19	0	0,048
2	4323	0,02	0	0,04	0,01	0,93	0	0	0	0	0,93	0,03	0,04	0	0,042
2	10118	0	0	0	0,14	0,84	0	0,02	0	0	0	0	1	0	0,048
2	6200	0,04	0,01	0	0	0,93	0	0,02	0	0	0,96	0,01	0,01	0,02	0,048
2	7254	0,05	0	0,02	0	0,89	0	0	0,04	0	0,95	0,05	0	0	0,046
2	4599	0	0,01	0,04	0	0,9	0	0,05	0	0	0,01	0,04	0,05	0,9	0,042
2	23394	0,01	0	0,04	0,03	0,91	0	0	0,01	0	0,05	0	0,03	0,92	0,044
2	11863	0	0,07	0	0,05	0,88	0	0	0	0	0	0,07	0,88	0,05	0,044
2	4076	0	0	0,04	0	0	0,58	0	0,38	0	0,01	0,12	0,87	0	0,022
2	28930	0,04	0	0,03	0	0,88	0	0	0,05	0	0	0,07	0,88	0,05	0,04
2	63885	0,05	0	0,02	0	0	0	0,01	0,92	0	0,03	0	0,92	0,05	0,048
2	9817	0,07	0	0	0	0,71	0	0	0,21	0,01	0,01	0,19	0,61	0,19	0,046
2	32026	0	0	0	0,09	0,85	0,06	0	0	0	0,06	0,86	0	0,08	0,048
2	49396	0	0,3	0,01	0	0,68	0,01	0	0	0	0,01	0	0,3	0,69	0,048
2	30655	0	0,01	0	0,07	0,92	0	0	0	0	0,07	0	0	0,93	0,046
2	46197	0	0	0,01	0,1	0,88	0	0,01	0	0	0,1	0	0,9	0	0,044
2	2431	0	0	0,04	0	0,62	0,31	0	0	0,03	0,28	0,04	0,37	0,31	0,028
2	3063	0	0,24	0	0	0,42	0,02	0	0,32	0	0,01	0	0,42	0,57	0,048
2	31857	0,01	0	0,01	0,18	0,8	0	0	0	0	0,83	0,01	0,15	0,01	0,048
2	17813	0	0,05	0	0,06	0,72	0,04	0	0,13	0	0,03	0,02	0,89	0,06	0,048
2	24155	0	0	0	0,2	0,79	0,01	0	0	0	0,79	0	0,21	0	0,046
2	17405	0	0	0,04	0,05	0,91	0	0	0	0	0	0,09	0	0,91	0,044
2	19548	0,04	0	0,01	0	0	0,75	0	0,2	0	0,95	0	0,01	0,04	0,04
2	4354	0	0,08	0	0	0,92	0	0	0	0	0,92	0	0	0,08	0,048
2	17266	0	0	0	0,32	0,68	0	0	0	0	1	0	0	0	0,046

2	6116	0,01	0,2	0	0	0,77	0	0,02	0	0	0,22	0	0,01	0,77	0,042
2	4672	0,04	0	0,04	0	0,91	0	0,01	0	0	0,01	0,95	0,04	0	0,042
2	29565	0	0,19	0	0	0,79	0	0	0,02	0	0,19	0,79	0	0,02	0,046
2	8736	0	0	0	0,07	0	0	0	0,92	0,01	0,01	0,91	0,01	0,07	0,046
2	8665	0	0,15	0,03	0	0,31	0	0	0,51	0	0	0,02	0,67	0,31	0,046
2	7762	0,05	0,01	0	0	0,46	0,14	0	0,34	0	0,8	0,15	0,01	0,04	0,046
2	9648	0,05	0,17	0	0	0	0	0	0,78	0	0	0	0,17	0,83	0,048
2	27022	0	0,06	0	0	0,93	0	0	0,01	0	0,99	0	0	0,01	0,046
2	15853	0,01	0,29	0	0	0,7	0	0	0	0	0	0	0,3	0,7	0,048
2	12465	0	0	0,04	0	0,96	0	0	0	0	0,96	0	0,04	0	0,028
2	12866	0	0,85	0,04	0,01	0	0,1	0	0	0	0	0,05	0	0,95	0,04
2	11027	0	0	0	0,1	0,48	0,42	0	0	0	0,39	0,03	0,1	0,48	0,044
2	7624	0,05	0	0,02	0	0,93	0	0	0	0	0,93	0	0,05	0,02	0,046
2	42758	0,05	0	0	0	0	0	0	0,94	0,01	0,88	0,06	0,06	0	0,048
2	14421	0	0	0,04	0	0	0,04	0	0,92	0	0,05	0,04	0	0,91	0
2	11554	0,04	0,18	0	0	0	0	0	0,78	0	0	0,78	0	0,22	0,036
2	14927	0	0,14	0	0	0,86	0	0	0	0	0,14	0,01	0,85	0	0,046
2	20456	0,04	0,02	0,02	0	0,9	0	0	0	0,02	0,03	0,02	0,9	0,05	0,046
2	22058	0	0,01	0	0,07	0,91	0	0	0	0,01	0,01	0	0,07	0,92	0,048
2	10322	0,03	0,08	0,03	0	0,54	0	0	0,32	0	0,04	0,34	0,05	0,57	0,04
2	26757	0	0,1	0	0,02	0,83	0,01	0,03	0,01	0	0,39	0	0,37	0,24	0,048
2	25932	0	0	0,04	0,09	0,35	0,5	0	0,02	0	0,35	0,01	0,02	0,62	0,046
2	4775	0	0,04	0,03	0	0,93	0	0	0	0	0,93	0,07	0	0	0,046
2	23327	0,01	0,23	0	0	0,68	0,08	0	0	0	0,05	0,04	0,23	0,68	0,036
2	10639	0	0,05	0	0,05	0,86	0,04	0	0	0	0	0	0,09	0,91	0,03
2	12467	0,01	0,05	0	0,02	0,92	0	0	0	0	0,92	0	0,08	0	0,048
2	22805	0,02	0	0,04	0	0,82	0,12	0	0	0	0,82	0,06	0	0,12	0,048
2	10018	0	0	0	0,06	0,94	0	0	0	0	0,06	0,94	0	0	0,036
2	7049	0	0,22	0	0	0,78	0	0	0	0	0,22	0,45	0	0,33	0,046
2	22710	0,02	0,07	0,02	0	0,6	0,29	0	0	0	0,29	0,05	0,66	0	0,044
2	5862	0,06	0	0	0	0,02	0,9	0	0,02	0	0,87	0	0,06	0,07	0,048
2	6817	0	0,14	0	0	0,85	0	0	0	0,01	0,12	0,02	0,86	0	0,044
2	8432	0	0	0	0,29	0,7	0	0	0,01	0	0,97	0,01	0,02	0	0,048

2	8653	0	0,96	0,04	0	0	0	0	0	0	0	0,04	0	0,96	0,016
2	5100	0	0,01	0	0,18	0,81	0	0	0	0	0,82	0,18	0	0	0,038
2	18736	0	0,72	0,04	0	0,24	0	0	0	0	0,01	0,04	0	0,95	0,024
2	11341	0,01	0	0,04	0	0,1	0,03	0	0,81	0,01	0,01	0,54	0,01	0,44	0,016
2	15496	0,01	0	0,04	0	0,95	0	0	0	0	0	0,04	0,96	0	0,036
2	5812	0	0,02	0,01	0,05	0,92	0	0	0	0	0	0,28	0,02	0,7	0,04
2	5445	0,01	0,06	0	0,04	0,86	0	0	0	0,03	0,01	0,08	0,86	0,05	0,048
2	7647	0,06	0,01	0,01	0	0,89	0,03	0	0	0	0,04	0,91	0	0,05	0,048
2	7343	0	0,09	0	0,02	0,17	0	0	0,72	0	0,02	0,17	0,81	0	0,046
2	10117	0	0,16	0	0	0,82	0,02	0	0	0	0	0	0,71	0,29	0,048
2	5181	0,01	0,1	0,01	0	0,88	0	0	0	0	0	0,11	0,89	0	0,048
2	9772	0,01	0,96	0,02	0,01	0	0	0	0	0	0	0,02	0,02	0,96	0,048
2	39768	0	0,04	0,01	0,07	0,85	0	0,01	0,02	0	0	0,11	0,87	0,02	0,04
2	29302	0,01	0,01	0,02	0,06	0,78	0	0	0,12	0	0,01	0,01	0,88	0,1	0,048
2	9147	0,05	0	0,02	0	0,9	0,02	0,01	0	0	0	0,95	0,02	0,03	0,048
2	27385	0	0,17	0,04	0,01	0	0,01	0	0,75	0,02	0,01	0,04	0,04	0,91	0,022
2	40423	0	0	0	0,13	0,81	0,06	0	0	0	0,13	0	0,87	0	0,046
2	19525	0	0,49	0	0	0,51	0	0	0	0	0	0	0,15	0,85	0,046
2	5399	0	0	0,04	0	0,92	0,03	0,01	0	0	0,01	0,46	0,03	0,5	0,008
2	14992	0	0	0	0,1	0,89	0,01	0	0	0	0,01	0,1	0,89	0	0,042
2	40684	0	0,12	0	0,01	0,87	0	0	0	0	0,01	0,12	0,87	0	0,044
2	7896	0,03	0,01	0	0,03	0,91	0	0	0,01	0,01	0,91	0,01	0,04	0,04	0,044
2	23417	0	0	0,01	0,11	0	0,88	0	0	0	0	0,88	0,01	0,11	0,048
2	44923	0	0	0	0,28	0	0,72	0	0	0	0,84	0,16	0	0	0,048
2	12561	0,01	0	0	0,18	0,8	0	0,01	0	0	0,91	0	0,02	0,07	0,048
2	10335	0,04	0,01	0	0	0,94	0	0	0	0,01	0,95	0,04	0	0,01	0,042
2	10921	0,03	0	0,04	0	0,93	0	0	0	0	0,03	0,01	0,95	0,01	0,046
2	23008	0	0,13	0	0	0,87	0	0	0	0	0,25	0,62	0,13	0	0,044
2,25	5497	0,02	0,05	0,01	0,02	0,9	0	0	0	0	0,9	0,02	0,02	0,06	0,04
2,25	11491	0,04	0	0	0,02	0,75	0	0	0,19	0	0,16	0,8	0	0,04	0,046
2,25	14933	0,01	0	0,03	0,09	0,86	0	0,01	0	0	0,82	0	0,15	0,03	0,044
2,25	4636	0,01	0	0	0,07	0,91	0	0	0,01	0	0,01	0,91	0,07	0,01	0,048
2,25	34809	0	0	0	0,06	0,84	0	0	0,07	0,03	0,1	0	0	0,9	0,042

2,25	36486	0,01	0,23	0,01	0	0,74	0	0	0,01	0	0,23	0,02	0,02	0,73	0,04
2,25	29318	0	0	0	0,32	0,67	0	0	0,01	0	1	0	0	0	0,042
2,25	8224	0	0,41	0	0	0,57	0,01	0,01	0	0	0	0,28	0,14	0,58	0,048
2,25	8865	0,05	0,02	0	0	0,48	0,43	0,02	0	0	0,05	0,02	0,5	0,43	0,042
2,25	52859	0	0,01	0,04	0,09	0,21	0	0	0,63	0,02	0,03	0,1	0,66	0,21	0,048
2,25	21542	0,03	0,07	0	0	0,86	0	0	0,04	0	0,07	0,86	0,04	0,03	0,048
2,25	19809	0	0	0,01	0,06	0,89	0	0	0,04	0	0,04	0	0,07	0,89	0,042
2,25	12446	0	0,07	0	0,01	0,91	0	0,01	0	0	0,91	0,02	0,04	0,03	0,044
2,25	14866	0	0,59	0	0	0,37	0,04	0	0	0	0,02	0,37	0,03	0,58	0,034
2,25	19393	0	0	0,03	0,09	0,88	0	0	0	0	0,87	0,1	0	0,03	0,048
2,25	9361	0	0,68	0	0	0,32	0	0	0	0	0,11	0	0	0,89	0,048
2,25	4790	0,02	0	0	0,04	0,93	0	0,01	0	0	0,04	0	0,25	0,71	0,046
2,25	6576	0,05	0	0	0	0,85	0,1	0	0	0	0,1	0,05	0	0,85	0,042
2,25	6631	0,03	0	0,01	0,01	0,88	0	0,01	0,06	0	0,04	0,06	0	0,9	0,042
2,25	20634	0	0,1	0	0,02	0,49	0	0	0,39	0	0,49	0,49	0,02	0	0,048
2,25	21630	0,02	0,34	0,02	0	0	0	0	0,62	0	0,01	0,63	0,02	0,34	0,048
2,25	35937	0,01	0	0	0,06	0,93	0	0	0	0	0	0,94	0,05	0,01	0,048
2,25	31280	0,03	0,04	0,01	0,03	0,86	0	0	0,03	0	0,04	0,92	0,03	0,01	0,048
2,25	30624	0	0,02	0	0,1	0	0,01	0	0,87	0	0,04	0,02	0,85	0,09	0,046
2,25	86382	0	0,15	0,01	0	0,84	0	0	0	0	0,02	0,41	0,57	0	0,046
2,25	10378	0,04	0,12	0	0,02	0,82	0	0	0	0	0,02	0,73	0,04	0,21	0,048
2,25	21257	0,04	0,13	0	0	0,77	0	0	0,06	0	0,04	0,13	0	0,83	0,048
2,25	8387	0,01	0	0,04	0	0,8	0	0	0,15	0	0,04	0,8	0,15	0,01	0,026
2,25	6696	0	0	0	0,06	0,93	0	0	0,01	0	0,02	0,06	0	0,92	0,046
2,25	35622	0	0	0,01	0,08	0	0	0,01	0,9	0	0,09	0,81	0	0,1	0,048
2,25	23840	0	0	0	0,11	0,82	0,07	0	0	0	0,08	0,09	0,83	0	0,044
2,25	17215	0	0,01	0,02	0,07	0,83	0	0	0,04	0,03	0,07	0,48	0,19	0,26	0,048
2,25	28115	0	0,99	0	0	0	0	0	0,01	0	0	0,99	0	0,01	0,026
2,25	12400	0	0	0,04	0,01	0	0	0	0,95	0	0,02	0,95	0,01	0,02	0,016
2,25	20542	0	1	0	0	0	0	0	0	0	0	0,98	0	0,02	0,04
2,25	23805	0,05	0	0	0	0,47	0	0,01	0,47	0	0,46	0,01	0,48	0,05	0,044
2,25	42534	0,06	0	0	0	0,94	0	0	0	0	0	0,94	0	0,06	0,042
2,25	15815	0,02	0,93	0	0	0,02	0	0	0,03	0	0,02	0,05	0	0,93	0,044

2,25	60998	0	0,13	0	0	0,87	0	0	0	0	0	0	1	0	0,048
2,25	19363	0,05	0	0	0,04	0	0,91	0	0	0	0,95	0	0,04	0,01	0,046
2,25	34328	0	0,12	0	0,01	0	0,75	0	0,12	0	0,12	0	0,88	0	0,046
2,25	13647	0,01	0,29	0	0	0,7	0	0	0	0	0	0	0,29	0,71	0,044
2,25	7661	0,01	0,02	0,04	0	0,91	0	0	0	0,02	0,6	0,03	0,34	0,03	0,044
2,25	20806	0	0	0	0,26	0,74	0	0	0	0	0,74	0	0,26	0	0,04
2,25	13458	0	0,19	0,03	0,01	0,77	0	0	0	0	0	0,82	0,18	0	0,048
2,25	23100	0,03	0,03	0	0,03	0,89	0	0	0,02	0	0	0,03	0,92	0,05	0,046
2,25	29955	0	0	0	0,25	0,75	0	0	0	0	0,81	0,19	0	0	0,048
2,25	5229	0,06	0	0	0	0	0,81	0	0,08	0,05	0,06	0	0,13	0,81	0,048
2,25	7330	0,06	0,01	0,03	0	0,9	0	0	0	0	0,01	0,03	0,06	0,9	0,048
2,25	14932	0,01	0	0	0,13	0,78	0,08	0	0	0	0,78	0	0	0,22	0,042
2,25	18189	0	0,05	0	0,1	0,83	0	0	0	0,02	0,88	0	0,12	0	0,046
2,25	70701	0,03	0	0,01	0,03	0,92	0	0	0	0,01	0,01	0	0,96	0,03	0,046
2,25	5812	0	0,1	0	0,01	0,89	0	0	0	0	0,12	0	0,87	0,01	0,036
2,25	12047	0	0,99	0	0	0,01	0	0	0	0	0	1	0	0	0,028
2,25	22845	0	0,2	0	0	0,79	0	0	0	0,01	0	0,79	0,21	0	0,042
2,25	17238	0	0,94	0	0	0,06	0	0	0	0	0	0,94	0	0,06	0,04
2,25	7243	0,05	0	0	0,03	0,92	0	0	0	0	0	0,04	0,93	0,03	0,038
2,25	5982	0,06	0	0	0	0,94	0	0	0	0	0	0,06	0	0,94	0,044
2,25	17163	0	0,11	0	0	0,88	0	0	0,01	0	0,66	0,23	0	0,11	0,038
2,25	15142	0	0	0	0,07	0	0,93	0	0	0	0,07	0	0	0,93	0,048
2,25	30771	0,04	0	0,01	0	0,95	0	0	0	0	0,34	0,64	0,01	0,01	0,038
2,25	8390	0,03	0	0,04	0	0,8	0	0	0,13	0	0,13	0,8	0,04	0,03	0,042
2,25	5844	0	0,23	0	0	0,68	0,01	0	0,08	0	0,01	0,3	0,24	0,45	0,038
2,25	12233	0	0,94	0,03	0,02	0	0	0	0,01	0	0	0,01	0,02	0,97	0,048
2,25	6544	0,07	0	0	0	0,24	0,69	0	0	0	0,3	0	0,01	0,69	0,048
2,25	31385	0	0	0	0,11	0	0,05	0	0,84	0	0,02	0,74	0,19	0,05	0,044
2,25	6868	0	0,13	0	0,04	0,62	0	0	0,21	0	0	0	0,32	0,68	0,042
2,25	14043	0,03	0,03	0	0	0,92	0,02	0	0	0	0,95	0,03	0	0,02	0,048
2,25	42159	0,03	0	0	0,04	0,86	0,07	0	0	0	0,86	0	0,03	0,11	0,046
2,25	26349	0	0,06	0,02	0	0	0,89	0	0	0,03	0,93	0,01	0,06	0	0,046
2,25	18921	0,01	0	0	0,15	0,84	0	0	0	0	0,84	0	0,15	0,01	0,046

2,25	13723	0,07	0	0,01	0	0,92	0	0	0	0	0	0,01	0,07	0,92	0,044
2,25	7961	0,04	0,02	0	0	0,84	0,1	0	0	0	0,88	0,02	0	0,1	0,04
2,25	29630	0	0,16	0	0,01	0,82	0	0,01	0	0	0,01	0	0,82	0,17	0,046
2,25	59540	0	0,01	0	0,06	0,93	0	0	0	0	0	0,99	0	0,01	0,038
2,25	10534	0,02	0,96	0	0	0	0,02	0	0	0	0	0,02	0,02	0,96	0,046
2,25	29198	0	0	0	0,3	0,7	0	0	0	0	1	0	0	0	0,044
2,25	15523	0	0,02	0,04	0	0	0,01	0	0,91	0,02	0,04	0,33	0,13	0,5	0,014
2,25	10620	0,04	0	0,04	0	0	0,2	0,01	0,7	0,01	0,04	0,17	0,1	0,69	0,048
2,25	5457	0,06	0	0	0	0	0,94	0	0	0	0	0	0,98	0,02	0,048
2,25	9181	0	0,22	0	0	0,75	0	0	0,03	0	0	0	0,49	0,51	0,048
2,25	16455	0	0	0,03	0,09	0,85	0	0	0,03	0	0,04	0	0,96	0	0,046
2,25	10662	0,05	0	0	0	0,42	0,51	0,01	0	0,01	0,05	0,01	0,82	0,12	0,038
2,25	34630	0	0,07	0	0	0,92	0	0	0	0,01	0,93	0	0,07	0	0,046
2,25	10177	0	0,08	0	0	0,54	0,25	0	0,13	0	0,54	0,38	0,08	0	0,048
2,25	23716	0,06	0	0	0	0,36	0,56	0	0,02	0	0,38	0,06	0,13	0,43	0,048
2,25	15055	0,01	0	0	0,15	0,72	0,05	0,02	0,05	0	0,86	0,03	0,04	0,07	0,042
2,25	20266	0	0	0,01	0,15	0	0	0	0,62	0,22	0,62	0	0,22	0,16	0,042
2,25	8976	0	0	0,01	0,11	0,84	0	0	0,04	0	0	0,01	0,95	0,04	0,046
2,25	29063	0,01	0,06	0,01	0	0,68	0,14	0,02	0,08	0	0,07	0,68	0,14	0,11	0,036
2,25	15882	0	0,13	0	0	0,85	0	0	0,02	0	0,02	0	0,85	0,13	0,04
2,25	9622	0,05	0,06	0,03	0	0,03	0	0	0,82	0,01	0,01	0,09	0,05	0,85	0,048
2,25	4338	0	0	0	0,11	0,89	0	0	0	0	0	0	0,89	0,11	0,044
2,25	22263	0,01	0,04	0	0,09	0,58	0,28	0	0	0	0,9	0	0,1	0	0,044
2,25	11615	0,05	0	0,02	0	0,72	0,19	0	0,02	0	0,21	0,28	0,51	0	0,044
2,25	13707	0	0,01	0,03	0,1	0,7	0,04	0,01	0,11	0	0,71	0,02	0,25	0,02	0,044
2,25	23163	0,03	0	0	0	0	0,09	0,04	0,84	0	0	0,88	0	0,12	0,048
2,25	11443	0,03	0	0,02	0,04	0,88	0	0	0,03	0	0,04	0,89	0	0,07	0,046
2,25	14672	0,06	0	0	0	0,94	0	0	0	0	0	0,06	0	0,94	0,048
2,25	76768	0	0,08	0,04	0	0	0,69	0	0,19	0	0,3	0	0,12	0,58	0,046
2,5	5271	0	0	0	0,03	0,76	0,03	0,05	0,04	0,09	0,05	0,88	0,06	0,01	0,048
2,5	46379	0	0	0	0	0	0	0,3	0,7	0	0,92	0	0	0,08	0,042
2,5	14356	0,05	0,06	0	0	0,8	0,09	0	0	0	0,04	0,88	0	0,08	0,046
2,5	20421	0	0,96	0,01	0,02	0	0	0	0,01	0	0	0,01	0,03	0,96	0,038

2,5	37582	0,03	0	0	0	0,9	0	0,06	0,01	0	0	0,09	0,9	0,01	0,038
2,5	18345	0,05	0	0,02	0	0	0,93	0	0	0	0,07	0	0,21	0,72	0,044
2,5	11437	0	0	0,01	0,12	0,63	0	0	0,24	0	0,42	0,34	0,24	0	0,046
2,5	15319	0,05	0	0,02	0,01	0,79	0,09	0	0,04	0	0,04	0,15	0,8	0,01	0,048
2,5	7316	0,01	0,05	0	0	0	0,78	0	0,12	0,04	0,76	0,15	0,05	0,04	0,044
2,5	18663	0,01	0,13	0	0	0,78	0,05	0,03	0	0	0,14	0,86	0	0	0,044
2,5	6190	0	0,84	0	0	0,05	0	0	0,11	0	0	0	0,11	0,89	0,048
2,5	12845	0	0	0,01	0,17	0,45	0,37	0	0	0	0,54	0	0,45	0,01	0,044
2,5	4074	0,05	0,02	0,02	0	0,91	0	0	0	0	0,31	0,02	0,05	0,62	0,044
2,5	11330	0	0	0	0,06	0,64	0,29	0	0	0,01	0,07	0,12	0	0,81	0,036
2,5	7366	0,03	0,02	0,04	0	0,89	0	0	0	0,02	0,01	0,05	0,92	0,02	0,044
2,5	59497	0,04	0	0	0,02	0,94	0	0	0	0	0,02	0	0,04	0,94	0,036
2,5	23179	0,05	0	0,04	0	0,88	0,01	0	0,01	0,01	0	0,81	0,13	0,06	0,046
2,5	14373	0,05	0	0	0,03	0,88	0	0	0,04	0	0,04	0	0,88	0,08	0,048
2,5	4930	0,02	0,01	0,01	0,04	0,92	0	0	0	0	0,03	0,92	0	0,05	0,048
2,5	7404	0,04	0,13	0	0	0,81	0	0,02	0	0	0	0,06	0,1	0,84	0,044
2,5	7655	0	0,12	0	0	0,88	0	0	0	0	0	0	1	0	0,046
2,5	17321	0,05	0	0	0	0	0	0,01	0,94	0	0,01	0,94	0	0,05	0,046
2,5	70660	0	0	0	0,18	0,79	0,02	0	0,01	0	0,78	0,18	0,02	0,02	0,04
2,5	27277	0	0,21	0	0	0,79	0	0	0	0	0	0,79	0,21	0	0,046
2,5	4014	0	0	0,04	0,01	0,95	0	0	0	0	0	0,04	0,96	0	0,028
2,5	5719	0	0,97	0	0,02	0	0,01	0	0	0	0,02	0	0	0,98	0,042
2,5	13246	0,04	0	0,04	0	0,65	0,27	0	0	0	0	0,69	0,04	0,27	0,048
2,5	10090	0	0,45	0,03	0	0	0	0,03	0,49	0	0,04	0,34	0	0,62	0,048
2,5	10738	0	0,03	0	0,07	0,85	0	0	0,05	0	0,03	0,9	0,07	0	0,048
2,5	22041	0	0,06	0	0,06	0,27	0,61	0	0	0	0,12	0	0,87	0,01	0,042
2,5	26109	0,01	0,16	0	0	0,81	0	0	0	0,02	0,01	0,81	0,18	0	0,044
2,5	31767	0,05	0	0	0	0,91	0,04	0	0	0	0,04	0,01	0,25	0,7	0,042
2,5	17097	0	0,11	0,01	0,03	0,84	0,01	0	0	0	0,12	0,18	0,7	0	0,048
2,5	67046	0,06	0	0	0	0,43	0,05	0	0,46	0	0,06	0,46	0,05	0,43	0,048
2,5	21496	0	0,02	0	0,07	0,84	0,07	0	0	0	0,03	0,07	0	0,9	0,044
2,5	16880	0,04	0,02	0	0,01	0,89	0	0,04	0	0	0,9	0,04	0,02	0,04	0,048
2,5	10453	0	0,01	0,02	0,07	0,88	0	0	0,02	0	0	0,03	0,02	0,95	0,038

2,5	18550	0	0,59	0	0,01	0,4	0	0	0	0	0	0,99	0,01	0	0,048
2,5	9110	0,02	0,23	0	0	0,74	0	0,01	0	0	0	0	0,25	0,75	0,046
2,5	17446	0	0,03	0	0,09	0,87	0	0,01	0	0	0,65	0,27	0,02	0,06	0,042
2,5	22387	0,05	0,67	0	0	0	0	0	0,28	0	0,01	0	0	0,99	0,048
2,5	5307	0,05	0	0	0	0,92	0	0	0,01	0,02	0	0,02	0,06	0,92	0,048
2,5	15247	0,01	0,25	0	0	0,73	0	0	0,01	0	0,05	0,09	0,17	0,69	0,036
2,5	5610	0	0,96	0,02	0,02	0	0	0	0	0	0,02	0	0,02	0,96	0,046
2,5	15938	0	0,34	0	0,01	0,64	0	0,01	0	0	0	0,98	0,01	0,01	0,038
2,5	55735	0,02	0	0,01	0	0,58	0	0,08	0,31	0	0,39	0,03	0,58	0	0,048
2,5	7521	0	0	0	0,09	0,33	0	0	0,58	0	0	0	0,58	0,42	0,046
2,5	31126	0	0,76	0	0,02	0	0,02	0	0,2	0	0,02	0,2	0	0,78	0,04
2,5	15848	0	0	0,04	0	0	0,94	0	0,02	0	0,08	0,01	0,91	0	0,032
2,5	17194	0	0	0	0,06	0,93	0	0	0	0,01	0	0,01	0,06	0,93	0,044
2,5	10717	0	0	0	0,1	0,01	0,51	0,01	0,37	0	0,38	0,1	0,01	0,51	0,044
2,5	10762	0,01	0	0	0,04	0	0	0	0,95	0	0,01	0	0	0,99	0,044
2,5	30115	0,02	0	0	0,07	0,22	0,52	0,01	0,16	0	0,17	0,6	0,23	0	0,044
2,5	11239	0	0,27	0	0	0,59	0	0	0,14	0	0	0	0,27	0,73	0,044
2,5	22942	0,05	0	0	0	0,93	0	0	0	0,02	0	0,07	0,92	0,01	0,048
2,5	37094	0	0	0	0,06	0	0	0	0,94	0	0	0,06	0	0,94	0,044
2,5	14283	0	0	0	0,12	0,37	0,51	0	0	0	0,37	0,03	0,51	0,09	0,044
2,5	8241	0,04	0	0,03	0,02	0,91	0	0	0	0	0,02	0,03	0,95	0	0,048
2,5	44257	0,01	0	0	0,23	0,75	0,01	0	0	0	0,75	0,24	0	0,01	0,04
2,5	17146	0,06	0	0	0	0,93	0,01	0	0	0	0	0,07	0,93	0	0,048
2,5	16443	0	0,09	0,02	0	0,19	0,23	0	0,47	0	0,14	0,23	0,63	0	0,048
2,5	44395	0	0,18	0	0,01	0,76	0	0	0,01	0,04	0,01	0,8	0,18	0,01	0,036
2,5	28551	0,01	0,07	0,01	0,05	0,85	0	0	0,01	0	0,05	0,08	0,85	0,02	0,044
2,5	4753	0,03	0,77	0	0	0,2	0	0	0	0	0,03	0,03	0	0,94	0,044
2,5	16585	0	0	0	0,07	0	0,03	0	0,87	0,03	0	0,03	0,03	0,94	0,046
2,5	13624	0	0,23	0	0	0,76	0,01	0	0	0	0,02	0,76	0,21	0,01	0,042
2,5	33269	0,05	0	0,01	0	0,86	0,07	0	0	0,01	0,07	0,05	0,88	0	0,046
2,5	17164	0,01	0,17	0	0	0	0	0	0,82	0	0,18	0	0	0,82	0,04
2,5	25533	0	0,09	0	0,02	0,89	0	0	0	0	0	0	0,98	0,02	0,044
2,5	10043	0	0,08	0	0	0,6	0,32	0	0	0	0,77	0	0,23	0	0,048

2,5	6158	0,01	0,01	0	0,06	0,92	0	0	0	0	0,05	0,92	0	0,03	0,046
2,5	15806	0,06	0	0	0	0	0,91	0,02	0,01	0	0,42	0,01	0	0,57	0,04
2,5	14402	0,05	0	0,02	0	0	0,21	0	0,72	0	0	0,02	0,27	0,71	0,042
2,5	9708	0	0	0	0,09	0,23	0,08	0	0,6	0	0	0	0,7	0,3	0,042
2,5	31494	0	0,1	0	0	0,54	0,36	0	0	0	0,57	0,35	0	0,08	0,038
2,5	16747	0	0,2	0	0	0,8	0	0	0	0	0	0,81	0,19	0	0,046
2,5	15657	0,03	0	0,01	0,02	0,94	0	0	0	0	0	0,95	0,03	0,02	0,034
2,5	21971	0	0	0,04	0,04	0	0,9	0,02	0	0	0,01	0,04	0,95	0	0,044
2,5	6420	0,01	0,02	0	0,15	0,82	0	0	0	0	0,76	0,23	0,01	0	0,046
2,5	20718	0,07	0	0	0	0,02	0	0	0,91	0	0	0,02	0,91	0,07	0,048
2,5	11240	0,01	0	0	0,02	0	0,72	0,25	0	0	0,98	0,01	0,01	0	0,044
2,5	7011	0	0	0,04	0,07	0	0	0,01	0,88	0	0,77	0,03	0	0,2	0,044
2,5	17338	0	0,1	0	0,05	0,73	0	0	0,12	0	0,11	0,1	0,06	0,73	0,04
2,5	10138	0,01	0,09	0	0	0,89	0	0	0,01	0	0	0,1	0,89	0,01	0,04
2,5	24522	0,06	0	0	0	0,13	0	0	0,81	0	0	0	0,94	0,06	0,046
2,5	5229	0	0,21	0	0	0,79	0	0	0	0	0	0,79	0,21	0	0,044
2,5	18472	0	0	0,04	0	0,95	0,01	0	0	0	0	0,04	0	0,96	0
2,5	5803	0,02	0,1	0	0	0,88	0	0	0	0	0	0	0,88	0,12	0,048
2,5	8922	0,06	0	0	0	0	0	0	0,89	0,05	0,89	0,06	0,05	0	0,046
2,5	25973	0,01	0	0	0,19	0,8	0	0	0	0	0,8	0	0,2	0	0,042
2,5	8810	0	0	0,02	0,06	0,91	0	0	0,01	0	0	0,99	0,01	0	0,048
2,5	6694	0,03	0	0,01	0,03	0,25	0	0	0,67	0,01	0	0,69	0,26	0,05	0,046
2,5	25609	0,05	0	0,01	0	0,91	0	0	0,03	0	0,83	0,09	0,03	0,05	0,044
2,5	10347	0,06	0	0	0	0	0	0	0,93	0,01	0,01	0,92	0	0,07	0,046
2,5	7865	0,01	0	0	0,11	0	0	0,01	0,87	0	0,01	0,07	0,87	0,05	0,044
2,5	4564	0,01	0	0,04	0	0	0	0,02	0,93	0	0,02	0,04	0,93	0,01	0,046
2,5	12507	0,04	0	0,02	0,01	0,93	0	0	0	0	0	0,02	0,97	0,01	0,046
2,5	18286	0,02	0,06	0,04	0	0,4	0,28	0,01	0,19	0	0,42	0	0,29	0,29	0,044
2,5	28271	0,05	0,01	0	0	0,94	0	0	0	0	0,99	0,01	0	0	0,044
2,5	14898	0	0	0	0,07	0,07	0	0	0,86	0	0	1	0	0	0,048
2,75	23121	0,02	0,09	0	0	0,87	0,01	0	0,01	0	0,02	0	0,98	0	0,044
2,75	8562	0	0	0	0,1	0,04	0	0	0,84	0,02	0	0,01	0,84	0,15	0,046
2,75	22439	0,04	0	0	0,05	0,83	0	0	0,08	0	0	0,05	0,89	0,06	0,044

2,75	29493	0,01	0	0	0,09	0,89	0	0	0,01	0	0,04	0,01	0,95	0	0,046
2,75	22451	0,07	0	0	0	0,92	0	0	0,01	0	0,52	0,39	0	0,09	0,04
2,75	15114	0	0,89	0	0	0	0	0,01	0,09	0,01	0,05	0,03	0	0,92	0,044
2,75	15023	0	0,22	0	0,03	0,64	0	0	0,11	0	0,15	0	0,09	0,76	0,048
2,75	9681	0,05	0	0	0,04	0,74	0,17	0	0	0	0,64	0,17	0	0,19	0,044
2,75	15512	0	0	0,02	0,07	0,89	0	0	0,02	0	0,02	0,91	0,07	0	0,048
2,75	4785	0,05	0	0	0,01	0,94	0	0	0	0	0	0	1	0	0,046
2,75	8776	0,02	0	0,04	0,02	0,03	0,01	0	0,88	0	0,03	0,05	0,92	0	0,042
2,75	10115	0	0	0,04	0,04	0,82	0,01	0	0,09	0	0	0,08	0,82	0,1	0,026
2,75	7799	0,01	0,96	0	0	0	0	0,03	0	0	0	0,01	0	0,99	0,048
2,75	6653	0	0	0,04	0	0	0	0,01	0,95	0	0,11	0,04	0,85	0	0,026
2,75	10942	0,02	0	0	0	0,84	0,11	0,03	0	0	0	0,89	0	0,11	0,046
2,75	12309	0,06	0	0	0	0	0,93	0	0,01	0	0,93	0	0,06	0,01	0,048
2,75	4607	0	0,13	0	0,07	0,8	0	0	0	0	0,07	0,92	0,01	0	0,048
2,75	41118	0,02	0,18	0	0	0,77	0	0	0,02	0,01	0,02	0,77	0,16	0,05	0,048
2,75	9371	0,01	0,1	0	0	0,87	0,02	0	0	0	0	0,01	0,97	0,02	0,042
2,75	11587	0,04	0	0,02	0	0	0,94	0	0	0	0,02	0,12	0,86	0	0,046
2,75	21777	0,04	0	0,01	0	0	0	0	0,94	0,01	0,94	0	0,01	0,05	0,046
2,75	10256	0,03	0	0	0,02	0,94	0,01	0	0	0	0	0,98	0,02	0	0,046
2,75	62731	0	0	0,02	0,05	0,89	0,02	0,02	0	0	0,05	0,92	0,01	0,02	0,044
2,75	37363	0,06	0	0	0	0,94	0	0	0	0	0,94	0	0	0,06	0,04
2,75	20026	0	0	0	0,08	0,92	0	0	0	0	0	0,98	0,02	0	0,046
2,75	15487	0,02	0,96	0,02	0	0	0	0	0	0	0	0,02	0,02	0,96	0,044
2,75	22713	0	0,01	0	0,29	0,69	0	0	0	0,01	0,98	0,01	0	0,01	0,046
2,75	23266	0,07	0	0	0	0,91	0,01	0	0	0,01	0,03	0,95	0,01	0,01	0,046
2,75	23361	0	0,06	0	0,08	0	0,86	0	0	0	0,85	0	0,06	0,09	0,046
2,75	55697	0,03	0	0	0,06	0	0,59	0	0,32	0	0,26	0,66	0	0,08	0,042
2,75	7031	0	0,04	0	0,07	0,07	0,82	0	0	0	0,04	0,01	0,06	0,89	0,038
2,75	18490	0	0,21	0,01	0	0,78	0	0	0	0	0	0,78	0,22	0	0,042
2,75	20564	0	0	0,01	0,09	0,9	0	0	0	0	0,01	0	0,62	0,37	0,044
2,75	21330	0	0	0	0,08	0,9	0,02	0	0	0	0,1	0,9	0	0	0,046
2,75	18516	0	0	0	0,11	0,88	0	0	0	0,01	0,11	0	0,89	0	0,04
2,75	9749	0,01	0	0,04	0	0,94	0	0	0	0,01	0,04	0,29	0,02	0,65	0,012

2,75	10963	0,05	0,01	0	0	0,94	0	0	0	0	0	0	1	0	0,046
2,75	4698	0	0,01	0	0,07	0,89	0,01	0	0	0,02	0	0,07	0,02	0,91	0,048
2,75	14063	0	0,12	0	0,01	0,86	0	0	0,01	0	0,01	0	0,98	0,01	0,046
2,75	10880	0,05	0,01	0	0	0	0	0	0,94	0	0,01	0,94	0,05	0	0,046
2,75	13684	0,01	0	0	0	0,88	0,01	0,1	0	0	0	0,11	0,89	0	0,046
2,75	21099	0	0,13	0,01	0,03	0	0,83	0	0	0	0	0,13	0,87	0	0,048
2,75	16655	0,01	0,16	0	0	0,18	0,01	0	0,64	0	0,17	0,64	0	0,19	0,048
2,75	9505	0,03	0,54	0	0,01	0,41	0	0	0,01	0	0	0	0,04	0,96	0,044
2,75	22472	0,06	0	0	0	0	0,94	0	0	0	0,96	0,04	0	0	0,046
2,75	3608	0	0	0,04	0,12	0,8	0,02	0	0	0,02	0,72	0,24	0,04	0	0,048
2,75	18444	0	0,01	0	0,06	0,93	0	0	0	0	0,03	0,01	0,05	0,91	0,048
2,75	17982	0,01	0	0,01	0,2	0,78	0	0	0	0	0,98	0,02	0	0	0,048
2,75	5569	0	0	0,03	0,08	0,86	0	0	0,03	0	0	0,9	0,07	0,03	0,048
2,75	10082	0	0	0	0,28	0	0	0	0,72	0	0,72	0,28	0	0	0,046
2,75	16044	0,03	0	0,04	0	0,5	0,01	0	0,42	0	0,01	0,46	0,49	0,04	0,04
2,75	29254	0,04	0,12	0	0	0,83	0	0,01	0	0	0	0,04	0	0,96	0,036
2,75	10833	0,02	0	0,03	0	0,95	0	0	0	0	0,95	0,03	0,02	0	0,048
2,75	6142	0,06	0,02	0,03	0	0	0	0	0,89	0	0,06	0,03	0,02	0,89	0,048
2,75	21237	0,05	0	0,02	0	0,81	0,08	0,01	0,03	0	0,01	0,02	0,92	0,05	0,046
2,75	3865	0,05	0	0,04	0	0,9	0	0	0	0,01	0,35	0,6	0,05	0	0,046
2,75	13166	0	0	0	0,4	0,6	0	0	0	0	1	0	0	0	0,044
2,75	26421	0	0,04	0,03	0,05	0,8	0	0	0,08	0	0	0,83	0,17	0	0,048
2,75	12484	0,05	0	0	0	0,92	0	0	0,01	0,02	0	0,92	0,02	0,06	0,04
2,75	24629	0,05	0	0,04	0	0,25	0	0	0,66	0	0,13	0,02	0,28	0,57	0,046
2,75	57240	0	0,05	0	0,09	0,85	0	0	0,01	0	0,86	0,05	0,04	0,05	0,048
2,75	7701	0	0,22	0	0	0,78	0	0	0	0	0,23	0	0,14	0,63	0,046
2,75	15062	0,01	0	0	0,16	0,54	0,24	0	0,05	0	0,71	0	0,05	0,24	0,046
2,75	9451	0,03	0,05	0	0	0,91	0	0	0	0,01	0,95	0	0,04	0,01	0,048
2,75	34526	0	0	0	0,09	0,89	0,02	0	0	0	0,02	0,9	0,08	0	0,044
2,75	8144	0	0	0,03	0,05	0,91	0	0,01	0	0	0,05	0,03	0,18	0,74	0,044
2,75	6241	0,02	0,05	0	0,03	0,9	0	0	0	0	0	0,92	0,08	0	0,048
2,75	20797	0	0,01	0	0,21	0,78	0	0	0	0	0,78	0,01	0,21	0	0,048
2,75	35490	0,04	0	0	0,15	0,76	0	0	0,05	0	0,96	0,04	0	0	0,046

2,75	4557	0,02	0,02	0	0,04	0,92	0	0	0	0	0,02	0,02	0	0,96	0,046
2,75	22673	0	0,07	0,01	0	0,92	0	0	0	0	0,93	0,07	0	0	0,044
2,75	7770	0	0,32	0	0	0,67	0	0	0	0,01	0,12	0	0,21	0,67	0,042
2,75	29262	0	0,08	0	0	0,92	0	0	0	0	0,92	0	0	0,08	0,044
2,75	10093	0	0	0,02	0,06	0,86	0	0	0,06	0	0,02	0,92	0	0,06	0,038
2,75	4761	0,03	0	0	0,12	0,85	0	0	0	0	0,8	0,13	0,07	0	0,046
2,75	13413	0,04	0	0	0,01	0,86	0	0	0,09	0	0,02	0,86	0	0,12	0,044
2,75	12769	0,05	0	0,01	0	0,94	0	0	0	0	0	0	0,94	0,06	0,048
2,75	7062	0,02	0,01	0	0,04	0,01	0	0	0,92	0	0,01	0	0,03	0,96	0,04
2,75	12337	0	0	0	0,14	0,86	0	0	0	0	0,34	0,14	0,52	0	0,048
2,75	9813	0	0	0,02	0,06	0,5	0,37	0,02	0,03	0	0,07	0,02	0,52	0,39	0,04
2,75	14657	0,03	0	0	0,05	0,92	0	0	0	0	0,01	0,81	0,15	0,03	0,046
2,75	66630	0	0	0	0,07	0,89	0	0	0,04	0	0	0,59	0,07	0,34	0,046
2,75	10841	0	0	0,04	0,1	0,58	0,26	0,02	0	0	0,83	0,06	0,06	0,05	0,048
2,75	14052	0,06	0	0	0	0,91	0	0	0,03	0	0,97	0	0,02	0,01	0,046
2,75	16522	0,04	0	0,04	0	0	0	0	0,91	0,01	0,39	0,56	0	0,05	0,046
2,75	8854	0,03	0,61	0	0	0,36	0	0	0	0	0	0,59	0	0,41	0,046
2,75	13058	0	0,19	0	0	0,81	0	0	0	0	0	0,75	0,25	0	0,048
2,75	7999	0,05	0	0,02	0	0,07	0,01	0,01	0,84	0	0,02	0	0,09	0,89	0,048
2,75	42690	0,03	0,02	0,04	0	0,9	0	0	0,01	0	0	0,25	0,74	0,01	0,046
2,75	4387	0,02	0	0,04	0,03	0,87	0	0,03	0,01	0	0,43	0,52	0,03	0,02	0,048
2,75	23308	0	0	0	0,06	0,91	0,03	0	0	0	0	0	0,03	0,97	0,046
2,75	15007	0	0	0,04	0	0	0,93	0,03	0	0	0	0	0,96	0,04	0,046
2,75	19981	0,03	0,2	0,02	0,02	0	0	0	0,73	0	0,03	0	0	0,97	0,048
2,75	15716	0,04	0,15	0	0	0,8	0,01	0	0	0	0	0,21	0	0,79	0,048
2,75	21488	0	0	0	0,12	0,88	0	0	0	0	0,01	0,46	0,53	0	0,046
2,75	62110	0,05	0,04	0	0	0,77	0,14	0	0	0	0,05	0,36	0,59	0	0,046
2,75	16806	0	0,04	0,01	0,09	0,85	0	0	0,01	0	0,04	0,08	0,88	0	0,048
2,75	31950	0,02	0	0,01	0,04	0,92	0,01	0	0	0	0,03	0,05	0,1	0,82	0,046
2,75	32709	0	0,15	0	0	0,84	0	0,01	0	0	0,16	0,69	0,13	0,02	0,048
2,75	18441	0,04	0	0	0,03	0,8	0	0	0,13	0	0	0,8	0,13	0,07	0,048
3	6091	0,01	0	0	0,02	0,95	0	0,02	0	0	0	0,03	0	0,97	0,048
3	8558	0	0	0	0,05	0,94	0	0,01	0	0	0	0,06	0,01	0,93	0,048

3	9723	0,06	0,05	0	0	0,89	0	0	0	0	0,05	0,01	0,05	0,89	0,048
3	18162	0,01	0,1	0	0	0	0,89	0	0	0	0	0	0,99	0,01	0,044
3	12554	0,05	0	0,03	0	0,88	0,02	0,02	0	0	0,05	0,02	0,93	0	0,044
3	12694	0	0	0	0,06	0	0	0	0,94	0	0	0,94	0	0,06	0,048
3	6496	0	0,95	0	0	0	0	0	0,05	0	0,01	0,06	0,05	0,88	0,036
3	13736	0,05	0	0	0	0,95	0	0	0	0	0	0,05	0	0,95	0,042
3	18794	0	0	0	0,09	0,91	0	0	0	0	0	0,07	0,93	0	0,048
3	16610	0	0,13	0	0	0,83	0	0	0,04	0	0,01	0,03	0,96	0	0,04
3	21549	0	0	0	0,21	0,71	0,07	0	0	0,01	0,78	0	0,21	0,01	0,044
3	5886	0,05	0	0,01	0	0,94	0	0	0	0	0	0	0,33	0,67	0,048
3	20787	0	0	0	0,28	0,72	0	0	0	0	1	0	0	0	0,048
3	10639	0,01	0,27	0,01	0	0,71	0	0	0	0	0,27	0,01	0	0,72	0,046
3	15712	0	0,03	0	0,08	0,89	0	0	0	0	0,03	0	0,86	0,11	0,046
3	9493	0,04	0	0,02	0	0,84	0,1	0	0	0	0,21	0,65	0,14	0	0,04
3	6728	0	0,01	0,04	0,01	0,92	0,01	0,01	0	0	0,01	0,02	0,97	0	0,04
3	22619	0	0,22	0	0	0,73	0	0	0,05	0	0	0,73	0,22	0,05	0,048
3	25854	0,03	0,07	0	0	0,89	0	0	0,01	0	0	0,3	0,66	0,04	0,046
3	27189	0,03	0,01	0	0,07	0	0,89	0	0	0	0,9	0,03	0	0,07	0,048
3	21988	0	0,16	0	0	0	0,01	0	0,83	0	0,16	0,83	0,01	0	0,046
3	22058	0	0	0,02	0,05	0,91	0	0	0,02	0	0,07	0	0,02	0,91	0,048
3	23945	0	0	0	0,13	0,83	0	0	0,02	0,02	0,16	0,01	0,83	0	0,044
3	8861	0,05	0	0	0,03	0,01	0,85	0	0,06	0	0,01	0,06	0,85	0,08	0,048
3	25553	0	0,96	0	0	0	0	0,04	0	0	0	0,04	0	0,96	0,04
3	48897	0	0,1	0	0,01	0,56	0,3	0	0	0,03	0,67	0,16	0	0,17	0,046
3	6831	0,04	0,05	0	0,01	0,13	0,77	0	0	0	0,07	0,15	0	0,78	0,048
3	28616	0	0	0,04	0,01	0,95	0	0	0	0	0	1	0	0	0,012
3	10625	0	0,28	0	0	0,72	0	0	0	0	0,27	0	0	0,73	0,048
3	35854	0	0,01	0	0,28	0,7	0	0	0	0,01	0,99	0	0,01	0	0,042
3	18892	0	0	0,04	0	0,91	0	0,04	0	0,01	0,01	0,7	0,04	0,25	0,036
3	34530	0	0,01	0,01	0,09	0,85	0	0	0	0,04	0,39	0,03	0,05	0,53	0,048
3	4261	0	0	0	0,14	0	0,86	0	0	0	0,5	0	0,14	0,36	0,046
3	8629	0	0,18	0	0	0,82	0	0	0	0	0,18	0,82	0	0	0,044
3	27635	0	0	0	0,07	0,93	0	0	0	0	0,07	0,93	0	0	0,048

3	17258	0	0	0	0,11	0,83	0,04	0	0,01	0,01	0,1	0,02	0,78	0,1	0,042
3	10092	0,02	0,08	0,01	0	0	0,04	0	0,85	0	0	0,02	0,89	0,09	0,046
3	53203	0	0	0	0,14	0,85	0	0	0,01	0	0,14	0	0,86	0	0,046
3	36667	0,04	0	0,01	0	0	0,87	0,04	0,04	0	0,04	0,04	0,91	0,01	0,048
3	7266	0,05	0	0,01	0	0,41	0	0	0,53	0	0	0,95	0,01	0,04	0,048
3	8596	0	0,03	0	0,15	0	0,75	0,04	0	0,03	0,75	0,25	0	0	0,048
3	18624	0	0,07	0,04	0	0,58	0,06	0,02	0,23	0	0,23	0,01	0,73	0,03	0,04
3	36679	0	0,01	0	0	0,89	0	0,1	0	0	0	0,06	0,84	0,1	0,048
3	14087	0	0,96	0,04	0	0	0	0	0	0	0	0	0,04	0,96	0,044
3	19720	0,05	0	0,01	0	0,94	0	0	0	0	0	0,06	0,94	0	0,044
3	14631	0	0,28	0,04	0	0	0,01	0	0,67	0	0	0,04	0,28	0,68	0,04
3	5745	0,03	0,03	0	0	0	0,03	0	0,91	0	0,94	0,01	0	0,05	0,048
3	10391	0	0	0	0,22	0,71	0	0,01	0,06	0	0,91	0	0	0,09	0,042
3	16927	0,03	0	0,03	0	0,89	0,05	0	0	0	0,89	0,03	0,03	0,05	0,04
3	35540	0	0,14	0	0	0,86	0	0	0	0	0	0,02	0,98	0	0,044
3	15545	0,01	0,12	0	0	0,87	0	0	0	0	0,12	0,01	0,87	0	0,042
3	30531	0	0,06	0,04	0,04	0	0	0,01	0,85	0	0,02	0,06	0,04	0,88	0,034
3	15714	0,07	0	0	0	0,93	0	0	0	0	0,93	0,07	0	0	0,03
3	10832	0,04	0	0	0	0,91	0	0,05	0	0	0,89	0,07	0,02	0,02	0,042
3	1896	0,02	0	0,04	0	0,89	0	0	0	0,05	0	0,04	0,96	0	0,04
3	10417	0,01	0	0,01	0,17	0,8	0	0	0,01	0	0,98	0,01	0	0,01	0,046
3	4200	0	0,11	0,04	0	0,84	0	0	0,01	0	0	0,05	0,83	0,12	0,044
3	12227	0,02	0	0	0,08	0,82	0	0	0,07	0,01	0	0,16	0,83	0,01	0,048
3	50872	0,01	0	0,01	0,05	0,9	0	0,03	0	0	0,07	0,01	0,9	0,02	0,048
3	12547	0,01	0,11	0	0	0,67	0	0,01	0,2	0	0,2	0,79	0,01	0	0,046
3	7430	0,06	0,01	0	0	0,14	0,15	0	0,64	0	0,15	0,01	0,63	0,21	0,048
3	16864	0,03	0	0,01	0,04	0,89	0	0	0,03	0	0,06	0,89	0,01	0,04	0,048
3	24675	0,03	0	0	0,1	0,17	0,67	0	0	0,03	0,25	0,07	0,63	0,05	0,04
3	23231	0,04	0	0,01	0	0,95	0	0	0	0	0,95	0,01	0	0,04	0,042
3	16357	0	0,95	0	0	0	0	0,03	0	0,02	0	0,07	0	0,93	0,048
3	21973	0,04	0	0,02	0	0,7	0	0	0,24	0	0,02	0	0,94	0,04	0,046
3	15163	0,03	0,06	0	0	0,57	0,2	0	0,11	0,03	0,07	0,7	0,2	0,03	0,048
3	27484	0	0,1	0	0,03	0,83	0	0	0,04	0	0,07	0,11	0,75	0,07	0,048

3	9995	0	0,13	0,01	0	0	0,86	0	0	0	0	0,01	0,99	0	0,046
3	44896	0	0	0	0,22	0,75	0,03	0	0	0	0,85	0,03	0,12	0	0,048
3	6113	0	0,08	0,03	0,01	0,56	0,02	0	0,27	0,03	0,36	0,62	0	0,02	0,046
3	33887	0	0	0	0,19	0	0	0	0,8	0,01	0,79	0,01	0	0,2	0,048
3	18050	0,04	0,01	0	0,01	0,94	0	0	0	0	0,02	0,95	0	0,03	0,048
3	9424	0,05	0	0	0	0,57	0	0	0,38	0	0,01	0	0	0,99	0,048
3	12891	0,04	0	0	0	0,95	0,01	0	0	0	0,82	0,17	0	0,01	0,044
3	100000	0,29	0,05	0,66	0	0	0	0	0	0	0,05	0,95	0	0	0,988
3	10372	0,02	0,03	0	0	0	0,01	0	0,94	0	0,97	0	0	0,03	0,04
3	7760	0	0	0	0,08	0,75	0	0	0,17	0	0	0	0,25	0,75	0,048
3	10281	0	0	0	0,21	0,79	0	0	0	0	0,79	0	0,21	0	0,044
3	11444	0,01	0,01	0,03	0,09	0,71	0,15	0	0	0	0,64	0,19	0	0,17	0,048
3	33775	0,03	0	0,01	0	0,92	0	0,04	0	0	0,08	0,85	0,04	0,03	0,048
3	7873	0,05	0	0,02	0	0,91	0	0	0,02	0	0,91	0,02	0,07	0	0,046
3	33611	0	0,08	0	0,05	0,86	0,01	0	0	0	0,7	0,01	0,29	0	0,048
3	5609	0	0,12	0	0	0	0,79	0	0,09	0	0,12	0	0,87	0,01	0,046
3	22235	0	0,02	0,01	0,15	0,81	0,01	0	0	0	0,79	0,16	0,05	0	0,046
3	28082	0,02	0,01	0	0	0,66	0,15	0,06	0,07	0,03	0	0,05	0,35	0,6	0,048
3	29571	0	0,17	0	0	0,7	0	0	0,13	0	0,13	0,48	0,21	0,18	0,048
3	12268	0	0	0	0,26	0	0,74	0	0	0	0,85	0,11	0,04	0	0,046
3	18296	0	0	0,01	0,06	0,91	0	0	0	0,02	0	0,98	0	0,02	0,048
3	20916	0,05	0	0	0	0,95	0	0	0	0	0,05	0	0,95	0	0,046
3	10010	0,04	0,01	0	0	0,93	0,01	0,01	0	0	0,01	0,97	0	0,02	0,042
3	42765	0	0	0	0,09	0,64	0,26	0	0	0,01	0,09	0,9	0	0,01	0,042
3	4817	0,06	0	0	0	0,94	0	0	0	0	0,06	0,88	0,06	0	0,042
3	17492	0,04	0	0	0	0,78	0	0,02	0,16	0	0,17	0,03	0	0,8	0,048
3	26616	0,01	0	0	0,07	0,25	0,59	0	0,08	0	0	0,29	0,14	0,57	0,042
3	11378	0,02	0,97	0	0,01	0	0	0	0	0	0	0	0,03	0,97	0,038
3	3624	0,02	0	0,01	0,04	0,01	0,01	0	0,91	0	0,01	0	0,23	0,76	0,046
3	30237	0	0,05	0	0,07	0	0,79	0	0	0,09	0	0,37	0,42	0,21	0,044
3	36537	0	0,01	0,01	0,16	0,82	0	0	0	0	0,83	0	0,16	0,01	0,04
3	19893	0	0	0,04	0	0,82	0,14	0	0	0	0,13	0,04	0,83	0	0,022
3,25	23203	0	0,06	0	0,08	0,86	0	0	0	0	0,02	0,3	0,68	0	0,048

3,25	6065	0,01	0,17	0	0	0,38	0,42	0	0	0,02	0,01	0,21	0,44	0,34	0,048
3,25	13075	0	0,21	0	0	0,78	0,01	0	0	0	0,01	0,78	0,21	0	0,042
3,25	7448	0	0,16	0	0	0,84	0	0	0	0	0	0,42	0,58	0	0,042
3,25	16808	0	0,1	0,01	0	0,79	0	0	0,1	0	0,54	0,25	0,21	0	0,046
3,25	39644	0	0,03	0	0,07	0,89	0	0,01	0	0	0,17	0	0,77	0,06	0,046
3,25	28262	0,01	0,14	0	0	0,79	0	0	0,05	0,01	0,14	0,79	0,01	0,06	0,046
3,25	28699	0,04	0,44	0,01	0	0,51	0	0	0	0	0	0,03	0,02	0,95	0,048
3,25	27006	0,01	0	0	0,06	0,86	0	0	0	0,07	0,06	0,01	0	0,93	0,046
3,25	8518	0,05	0	0,04	0	0	0	0	0,91	0	0	0,03	0,06	0,91	0,046
3,25	23267	0,01	0	0	0,05	0,94	0	0	0	0	0,02	0,75	0	0,23	0,048
3,25	45009	0	0	0	0,09	0	0,43	0	0,42	0,06	0,09	0,47	0	0,44	0,046
3,25	9929	0	0,94	0	0	0	0,03	0,03	0	0	0	0,06	0	0,94	0,042
3,25	7064	0,04	0,09	0	0	0,6	0	0	0,26	0,01	0,01	0,61	0,3	0,08	0,046
3,25	3832	0,01	0	0,04	0,01	0,94	0	0	0	0	0	0,65	0,35	0	0,028
3,25	14411	0,03	0,02	0,01	0,03	0,9	0	0	0,01	0	0,05	0,91	0,03	0,01	0,044
3,25	7209	0,04	0	0	0	0,95	0	0,01	0	0	0,04	0	0,96	0	0,046
3,25	9132	0,01	0	0	0,09	0,42	0,48	0	0	0	0,49	0,09	0	0,42	0,044
3,25	26789	0	0,12	0	0	0,04	0	0	0,84	0	0,03	0,09	0,88	0	0,048
3,25	22429	0,04	0,07	0,01	0	0	0	0	0,88	0	0,01	0,35	0,23	0,41	0,048
3,25	17469	0	0	0	0,08	0	0	0	0,91	0,01	0	0,99	0	0,01	0,048
3,25	10281	0	0,12	0	0,01	0,08	0,78	0	0,01	0	0	0,13	0,87	0	0,042
3,25	28697	0	0	0,02	0	0,2	0,64	0,13	0	0,01	0,15	0,84	0	0,01	0,042
3,25	8881	0,06	0,01	0	0	0,89	0,01	0	0,02	0,01	0	0	0,97	0,03	0,042
3,25	17339	0,03	0	0	0	0,01	0	0,03	0,93	0	0,01	0,99	0	0	0,046
3,25	10567	0,03	0	0,03	0	0,92	0,01	0	0,01	0	0,96	0,03	0	0,01	0,038
3,25	17985	0	0	0,03	0,1	0,86	0	0	0,01	0	0,12	0,1	0,77	0,01	0,048
3,25	9362	0	0	0	0,09	0,83	0,02	0	0,06	0	0,12	0,82	0	0,06	0,048
3,25	6167	0,03	0,55	0	0	0,42	0	0	0	0	0	0,39	0,02	0,59	0,04
3,25	13558	0,06	0	0	0	0,94	0	0	0	0	0	0,06	0	0,94	0,048
3,25	7039	0	0,04	0,01	0,06	0,87	0,02	0	0	0	0,06	0,01	0,46	0,47	0,048
3,25	4145	0,04	0	0	0,03	0	0,92	0	0,01	0	0,01	0,03	0,94	0,02	0,048
3,25	16360	0,02	0,08	0	0	0,75	0,03	0	0,12	0	0,11	0	0,84	0,05	0,048
3,25	17056	0,05	0	0	0,01	0,9	0,04	0	0	0	0,04	0,9	0,05	0,01	0,048

3,25	2864	0,05	0	0,01	0	0,94	0	0	0	0	0,01	0,06	0,93	0	0,04
3,25	15827	0,05	0	0,02	0	0,93	0	0	0	0	0,93	0,02	0,05	0	0,046
3,25	40052	0,03	0	0	0,03	0,94	0	0	0	0	0	0,03	0	0,97	0,046
3,25	62489	0,03	0,01	0	0,01	0	0	0,09	0,86	0	0,86	0,01	0,09	0,04	0,048
3,25	20142	0	0	0	0,22	0,52	0,26	0	0	0	0,74	0,26	0	0	0,042
3,25	21552	0,01	0	0,04	0	0	0	0	0,95	0	0	0,04	0,96	0	0,028
3,25	8696	0,06	0	0	0	0,09	0,01	0,01	0,83	0	0	0,7	0,06	0,24	0,044
3,25	16766	0,01	0	0	0,06	0,93	0	0	0	0	0,03	0,01	0,06	0,9	0,046
3,25	7249	0,01	0	0,01	0,03	0,01	0,26	0,04	0,64	0	0,03	0,03	0,68	0,26	0,04
3,25	6568	0	0,02	0	0	0,83	0,04	0,11	0	0	0,02	0,04	0,94	0	0,04
3,25	9844	0,07	0	0	0	0,79	0,05	0	0,09	0	0	0,09	0,12	0,79	0,048
3,25	9224	0,06	0	0	0	0,91	0	0	0,02	0,01	0,65	0	0,27	0,08	0,048
3,25	13000	0,05	0	0,04	0	0,9	0,01	0	0	0	0,06	0,04	0	0,9	0,038
3,25	40587	0,01	0,01	0,01	0,07	0	0	0	0,9	0	0,02	0,89	0,07	0,02	0,044
3,25	3393	0,02	0	0,04	0	0,25	0	0	0,69	0	0,25	0,04	0	0,71	0,032
3,25	14266	0,06	0	0	0,02	0,71	0	0	0,16	0,05	0,02	0,18	0,75	0,05	0,048
3,25	11936	0,06	0	0	0	0,92	0,01	0	0	0,01	0	0	0,93	0,07	0,048
3,25	11843	0,01	0,18	0,04	0	0,75	0	0,02	0	0	0	0,09	0,03	0,88	0,048
3,25	27931	0	0	0	0,06	0,89	0	0	0,05	0	0	0,06	0,05	0,89	0,046
3,25	31210	0,06	0	0	0	0	0	0	0,94	0	0	0,03	0,88	0,09	0,036
3,25	11859	0	0,01	0	0,17	0,79	0,01	0	0,02	0	0,79	0,02	0,17	0,02	0,048
3,25	10480	0,04	0,03	0	0	0,78	0	0	0,15	0	0,2	0,04	0,76	0	0,046
3,25	11287	0	0	0	0,07	0,93	0	0	0	0	0,12	0	0	0,88	0,042
3,25	7604	0,03	0,06	0	0	0,9	0,01	0	0	0	0,06	0	0,94	0	0,044
3,25	34319	0	1	0	0	0	0	0	0	0	0	0	0,06	0,94	0,038
3,25	19136	0	0,12	0	0	0,03	0,03	0	0,82	0	0,15	0,64	0,21	0	0,032
3,25	16096	0,02	0	0	0,12	0,86	0	0	0	0	0,86	0,03	0,02	0,09	0,048
3,25	30002	0,01	0,13	0	0	0	0,85	0	0,01	0	0,13	0,01	0,85	0,01	0,044
3,25	36514	0,01	0,12	0	0	0,07	0,03	0	0,77	0	0	0	0,89	0,11	0,042
3,25	11078	0	0,03	0,01	0,07	0	0	0	0,89	0	0,01	0	0,46	0,53	0,048
3,25	6288	0	0	0,03	0,1	0	0,78	0	0,09	0	0,09	0,81	0	0,1	0,048
3,25	25315	0	0,92	0,02	0	0	0,05	0	0	0,01	0	0,04	0,06	0,9	0,046
3,25	10632	0	0,6	0	0	0,1	0	0	0,24	0,06	0,07	0,11	0	0,82	0,04

3,25	11710	0	0,97	0	0,03	0	0	0	0	0	0	0	0,01	0,99	0,048
3,25	16638	0	0	0,03	0	0,91	0	0,06	0	0	0	0,94	0,06	0	0,044
3,25	17403	0	0	0	0,1	0,83	0,04	0	0	0,03	0,04	0,83	0,13	0	0,046
3,25	25948	0,05	0,01	0	0	0,94	0	0	0	0	0,01	0	0,05	0,94	0,048
3,25	7775	0,04	0	0,01	0,03	0,92	0	0	0	0	0,96	0	0	0,04	0,042
3,25	25901	0,03	0,02	0,04	0,03	0,04	0	0	0	0,84	0	0,49	0,47	0,04	0,048
3,25	21689	0	0,01	0	0,06	0,93	0	0	0	0	0	0,99	0,01	0	0,048
3,25	27956	0	0,2	0,01	0,04	0,75	0	0	0	0	0	0,2	0,06	0,74	0,04
3,25	16729	0	0,83	0,02	0	0,06	0,02	0	0,01	0,06	0,03	0	0,05	0,92	0,044
3,25	15728	0,01	0	0	0,05	0,83	0,04	0	0,02	0,05	0	0,04	0,13	0,83	0,048
3,25	32544	0	0,01	0,01	0,1	0,72	0,01	0	0,15	0	0,15	0,01	0,73	0,11	0,046
3,25	9679	0,01	0,97	0	0,02	0	0	0	0	0	0	0,02	0,01	0,97	0,046
3,25	4205	0,06	0	0	0	0,94	0	0	0	0	0	0,94	0	0,06	0,048
3,25	9470	0	0	0,04	0,07	0	0,44	0	0,45	0	0,51	0,01	0,04	0,44	0,046
3,25	11917	0	0,06	0	0	0,9	0,02	0,02	0	0	0,94	0	0,06	0	0,046
3,25	2267	0,07	0	0,02	0	0,35	0	0	0,56	0	0,07	0,02	0,56	0,35	0,046
3,25	18660	0,04	0,03	0	0	0,72	0,19	0	0,02	0	0,02	0,21	0,77	0	0,046
3,25	24254	0	0	0	0,07	0,92	0,01	0	0	0	0,01	0,01	0,06	0,92	0,048
3,25	7019	0	0,14	0	0	0,84	0,02	0	0	0	0,14	0	0,86	0	0,044
3,25	12924	0,06	0,01	0	0	0,75	0,12	0,01	0,03	0,02	0,79	0,12	0	0,09	0,046
3,25	7336	0,06	0,06	0	0	0,82	0	0	0,06	0	0,07	0,82	0	0,11	0,048
3,25	4113	0,05	0	0,01	0	0,8	0	0	0,14	0	0	0,01	0	0,99	0,044
3,25	10827	0,07	0	0,02	0	0,9	0,01	0	0	0	0,02	0,9	0	0,08	0,042
3,25	9570	0,02	0,01	0	0,12	0,83	0,02	0	0	0	0,86	0,12	0	0,02	0,046
3,25	23752	0	0	0	0,25	0,73	0	0,01	0	0,01	0,73	0,01	0,25	0,01	0,048
3,25	10359	0	0,62	0	0	0,28	0	0	0	0,1	0,1	0	0	0,9	0,046
3,25	5177	0,07	0	0	0	0,03	0,31	0	0,59	0	0,45	0	0,55	0	0,048
3,25	13617	0,04	0	0	0,02	0,02	0	0	0,92	0	0	0,92	0,02	0,06	0,044
3,25	13136	0	0,45	0	0,03	0	0	0	0,51	0,01	0	0,44	0,03	0,53	0,046
3,25	3838	0	0,03	0	0,08	0,87	0	0	0,01	0,01	0,04	0	0,87	0,09	0,046
3,25	23126	0,02	0	0,04	0,01	0,89	0	0,01	0,03	0	0,91	0,04	0,05	0	0,046
3,25	34250	0,02	0,2	0	0,02	0,76	0	0	0	0	0	0,02	0,2	0,78	0,042
3,25	10396	0,02	0,23	0	0	0,72	0,03	0	0	0	0	0,75	0,06	0,19	0,046

3,5	10019	0	0	0	0,04	0	0	0,03	0,93	0	0	0,03	0,11	0,86	0,048
3,5	11284	0	0	0,01	0,08	0,64	0	0	0,27	0	0,29	0,06	0,01	0,64	0,044
3,5	5742	0	0,01	0	0,07	0,92	0	0	0	0	0,01	0	0,07	0,92	0,048
3,5	5625	0	0,23	0,04	0	0,66	0,07	0	0	0	0	0,04	0,07	0,89	0,026
3,5	11324	0,05	0,11	0,01	0	0,78	0,05	0	0	0	0,02	0,01	0,15	0,82	0,04
3,5	14139	0,01	0	0	0,01	0,93	0	0,05	0	0	0,05	0,95	0	0	0,046
3,5	6070	0,06	0	0	0	0,9	0,03	0	0	0,01	0,06	0,03	0,91	0	0,048
3,5	7353	0,06	0	0	0	0,04	0	0	0,9	0	0,31	0,63	0	0,06	0,038
3,5	7821	0,02	0,98	0	0	0	0	0	0	0	0,02	0,01	0	0,97	0,046
3,5	6301	0	0,97	0,03	0	0	0	0	0	0	0,03	0	0	0,97	0,042
3,5	13789	0	0	0	0,05	0,93	0	0,01	0	0,01	0,01	0,93	0,01	0,05	0,046
3,5	2704	0,05	0	0,01	0	0,3	0	0	0,64	0	0,23	0,65	0,07	0,05	0,046
3,5	10588	0,03	0,02	0,03	0	0,81	0,09	0	0,01	0,01	0,86	0	0	0,14	0,04
3,5	24173	0,03	0,02	0	0,03	0,55	0,03	0	0,34	0	0,06	0,03	0,57	0,34	0,046
3,5	12353	0,04	0	0	0,02	0,12	0	0	0,76	0,06	0,93	0	0,03	0,04	0,048
3,5	8523	0,04	0	0	0	0,77	0,01	0,01	0,15	0,02	0,01	0,76	0,17	0,06	0,046
3,5	10877	0,04	0,01	0,02	0,02	0,91	0	0	0	0	0,92	0,01	0,05	0,02	0,042
3,5	25119	0	0	0,02	0,12	0,85	0,01	0	0	0	0,07	0,01	0,9	0,02	0,048
3,5	18136	0	0,23	0	0	0,08	0,03	0,01	0,65	0	0	0,05	0,31	0,64	0,046
3,5	23971	0	0,04	0	0,1	0,85	0	0,01	0	0	0,89	0	0	0,11	0,038
3,5	4232	0,02	0	0,02	0	0	0	0,04	0,92	0	0	0,96	0,04	0	0,048
3,5	18290	0,01	0,11	0	0,01	0,84	0,02	0,01	0	0	0,02	0,84	0,13	0,01	0,038
3,5	13286	0	0,42	0	0	0,26	0,31	0,01	0	0	0	0,26	0,06	0,68	0,042
3,5	25494	0	0	0	0,06	0,94	0	0	0	0	0	0,99	0	0,01	0,042
3,5	7796	0,01	0,15	0	0	0,83	0,01	0	0	0	0,02	0,84	0,14	0	0,044
3,5	6544	0	0,03	0,04	0,04	0,89	0	0	0	0	0,03	0,8	0,04	0,13	0,04
3,5	10283	0	0,93	0	0,01	0,01	0	0	0,05	0	0,05	0,01	0,01	0,93	0,042
3,5	11119	0,01	0	0	0,06	0,93	0	0	0	0	0	0,93	0,06	0,01	0,044
3,5	8183	0	0,13	0	0	0	0	0,01	0,86	0	0,13	0,87	0	0	0,048
3,5	7368	0	0	0	0,06	0,94	0	0	0	0	0	1	0	0	0,044
3,5	15894	0,03	0	0	0,03	0,93	0	0,01	0	0	0	0	0,05	0,95	0,048
3,5	4634	0	0	0,04	0	0,3	0	0,07	0,58	0,01	0,3	0,58	0,07	0,05	0,048
3,5	17916	0,06	0,01	0	0	0,9	0,03	0	0	0	0	0,95	0,04	0,01	0,048

3,5	10357	0,01	0	0	0,08	0,86	0,05	0	0	0	0,05	0,86	0,08	0,01	0,048
3,5	16841	0,01	0,05	0	0,04	0,82	0	0	0,08	0	0,01	0	0,13	0,86	0,046
3,5	14976	0	0,01	0	0,1	0,89	0	0	0	0	0,08	0,11	0,81	0	0,046
3,5	23553	0	0,11	0,03	0,07	0,79	0	0	0	0	0	0,03	0,11	0,86	0,048
3,5	21372	0,06	0	0	0	0,89	0,03	0	0,01	0,01	0,02	0,06	0,92	0	0,046
3,5	29457	0,03	0	0,03	0,01	0,93	0	0	0	0	0	0,03	0,85	0,12	0,034
3,5	20200	0	0,02	0	0,1	0,88	0	0	0	0	0	0,1	0,84	0,06	0,044
3,5	17435	0	0	0	0,06	0,94	0	0	0	0	0,06	0,08	0,09	0,77	0,048
3,5	6229	0,04	0,03	0	0,03	0,9	0	0	0	0	0,03	0,1	0,87	0	0,048
3,5	12912	0,03	0	0,03	0	0,59	0	0,01	0,34	0	0,37	0,03	0,36	0,24	0,04
3,5	40714	0	0,08	0	0,06	0	0	0	0,86	0	0	1	0	0	0,042
3,5	32113	0	0	0	0	0,5	0,3	0,2	0	0	0,56	0,29	0,15	0	0,04
3,5	25275	0	0	0,04	0	0,77	0,17	0	0,01	0,01	0	0,17	0,05	0,78	0,004
3,5	10084	0	0,07	0,04	0	0,02	0	0,01	0,84	0,02	0,31	0,08	0,6	0,01	0,048
3,5	13986	0,01	0	0,04	0,03	0,91	0	0,01	0	0	0	0,03	0	0,97	0,048
3,5	10704	0,05	0,19	0,03	0	0,7	0,03	0	0	0	0	0,06	0,01	0,93	0,048
3,5	25575	0,01	0,02	0	0,12	0,85	0	0	0	0	0,85	0	0,04	0,11	0,042
3,5	7013	0,06	0	0	0	0,93	0,01	0	0	0	0,61	0	0,37	0,02	0,046
3,5	28670	0,01	0,97	0	0	0,02	0	0	0	0	0,06	0	0	0,94	0,046
3,5	9503	0,07	0	0	0	0,56	0,19	0	0,16	0,02	0,16	0,02	0,82	0	0,048
3,5	18470	0	0	0,04	0,02	0,71	0,22	0,01	0	0	0,24	0,04	0,71	0,01	0,03
3,5	28196	0,01	0,04	0,04	0,05	0,86	0	0	0	0	0	0,04	0,02	0,94	0,048
3,5	37058	0,06	0	0	0	0,86	0,05	0	0,03	0	0,05	0,03	0,06	0,86	0,044
3,5	9503	0,05	0	0	0,01	0,94	0	0	0	0	0	0,02	0,05	0,93	0,048
3,5	8004	0	0,79	0	0,02	0,19	0	0	0	0	0	0,04	0,02	0,94	0,046
3,5	15477	0	0,96	0	0,04	0	0	0	0	0	0	0	0	1	0,042
3,5	54420	0,03	0,72	0	0	0,2	0	0,01	0,04	0	0	0,12	0	0,88	0,042
3,5	14584	0,04	0,1	0,01	0	0,73	0	0	0	0,12	0	0,74	0,17	0,09	0,048
3,5	7151	0,03	0	0,03	0,02	0,88	0,02	0	0	0,02	0,91	0,03	0,06	0	0,046
3,5	14642	0,01	0,26	0,01	0	0,07	0,46	0	0,08	0,11	0,01	0,24	0,08	0,67	0,046
3,5	14299	0,02	0,01	0,02	0,07	0,88	0	0	0	0	0,9	0,02	0,07	0,01	0,038
3,5	38751	0	0	0,01	0,11	0,88	0	0	0	0	0,01	0	0,99	0	0,048
3,5	34371	0	0,09	0	0	0,77	0,07	0	0,07	0	0,83	0,01	0,01	0,15	0,046

3,5	34795	0,01	0,23	0,04	0	0,69	0,01	0,02	0	0	0	0,04	0,24	0,72	0,046
3,5	11668	0	0,16	0	0,04	0,79	0,01	0	0	0	0	0,86	0,04	0,1	0,044
3,5	5824	0,06	0	0	0	0,94	0	0	0	0	0,94	0,02	0,01	0,03	0,048
3,5	5700	0	0	0	0,18	0,82	0	0	0	0	0,56	0,01	0,43	0	0,046
3,5	9964	0	0,1	0	0,01	0,01	0,88	0	0	0	0,1	0	0,9	0	0,046
3,5	17481	0,04	0	0,02	0	0,91	0,02	0,01	0	0	0,02	0,44	0	0,54	0,048
3,5	14037	0,05	0	0,01	0	0,92	0,02	0	0	0	0	0,96	0,04	0	0,036
3,5	11055	0	0,02	0	0,18	0,77	0	0	0,02	0,01	0,8	0,02	0,17	0,01	0,044
3,5	12105	0,05	0	0	0	0,95	0	0	0	0	0	0,18	0,77	0,05	0,036
3,5	16270	0	0,12	0,04	0,02	0	0	0	0,82	0	0,06	0,63	0	0,31	0,048
3,5	56316	0,04	0	0,02	0	0,62	0	0	0,32	0	0,62	0,02	0,36	0	0,042
3,5	7357	0	0,07	0	0,06	0,85	0	0,01	0	0,01	0	0,85	0,02	0,13	0,048
3,5	9766	0	0,01	0	0,07	0,92	0	0	0	0	0	0,92	0	0,08	0,046
3,5	10228	0,03	0,03	0	0,01	0,93	0	0	0	0	0,94	0,03	0,03	0	0,04
3,5	13858	0,05	0	0	0	0,94	0	0	0	0,01	0	0,01	0	0,99	0,042
3,5	15315	0	0,03	0,04	0	0,9	0	0	0,03	0	0	0	0,97	0,03	0,042
3,5	5365	0	0,06	0,01	0,04	0,07	0	0	0,82	0	0	0,11	0,01	0,88	0,038
3,5	70798	0	0,02	0	0,06	0,79	0,13	0	0	0	0,08	0	0	0,92	0,044
3,5	3719	0,02	0,01	0,04	0	0,9	0	0,03	0	0	0,04	0,04	0,03	0,89	0,046
3,5	38458	0	0	0	0,06	0	0,89	0,01	0	0,04	0,07	0,01	0,04	0,88	0,048
3,5	17658	0,01	0	0	0,05	0	0,94	0	0	0	0,07	0	0	0,93	0,048
3,5	9189	0,04	0,11	0	0,01	0,83	0,01	0	0	0	0,11	0,37	0	0,52	0,048
3,5	13912	0,06	0,01	0,02	0,01	0	0,78	0	0,12	0	0,01	0,12	0,78	0,09	0,044
3,5	23326	0	0,17	0	0,01	0,37	0	0	0,36	0,09	0,01	0	0,44	0,55	0,04
3,5	11998	0,05	0	0,03	0	0,91	0	0,01	0	0	0,05	0	0,01	0,94	0,044
3,5	3484	0,02	0,97	0,01	0	0	0	0	0	0	0	0,01	0,02	0,97	0,048
3,5	4775	0,01	0	0,01	0	0	0,82	0,15	0,01	0	0,82	0,01	0	0,17	0,046
3,5	25558	0,01	0,01	0	0,07	0,88	0	0	0,03	0	0,02	0,03	0,88	0,07	0,042
3,5	18080	0	0,15	0,01	0	0,83	0	0	0	0,01	0,17	0,66	0,16	0,01	0,046
3,5	24198	0	0	0	0,1	0,86	0,03	0,01	0	0	0	0,04	0,96	0	0,048
3,5	8225	0,04	0	0,01	0,01	0,94	0	0	0	0	0,98	0,01	0	0,01	0,046
3,5	13150	0,01	0	0	0,18	0,81	0	0	0	0	0,81	0	0,19	0	0,034
3,5	11070	0,02	0	0,03	0,01	0,55	0	0	0,32	0,07	0,72	0,01	0,22	0,05	0,03

3,5	15742	0,02	0,95	0	0	0	0	0,03	0	0	0	0,07	0	0,93	0,038
3,75	19968	0	0,21	0,01	0	0,78	0	0	0	0	0,01	0,79	0,19	0,01	0,044
3,75	7446	0,01	0,19	0	0,01	0,63	0,01	0	0,15	0	0,22	0,12	0,02	0,64	0,048
3,75	33949	0	0,28	0	0	0,72	0	0	0	0	0,28	0	0	0,72	0,046
3,75	7755	0	0	0,04	0	0,96	0	0	0	0	0	0,41	0,57	0,02	0,018
3,75	30725	0,07	0	0	0	0,88	0	0,02	0,03	0	0,88	0,07	0,02	0,03	0,044
3,75	16803	0	0,07	0	0,04	0,85	0	0,02	0,02	0	0	0,02	0,08	0,9	0,044
3,75	16365	0	0	0	0,09	0,63	0,03	0	0,25	0	0,25	0,29	0,05	0,41	0,048
3,75	17475	0	0,15	0,01	0	0,43	0,26	0	0,15	0	0,34	0,43	0	0,23	0,046
3,75	28436	0	0,15	0	0	0,85	0	0	0	0	0	0	0,78	0,22	0,048
3,75	35338	0	0	0	0,09	0,9	0	0	0,01	0	0	0,09	0,89	0,02	0,042
3,75	35682	0	0	0,04	0	0	0,08	0	0,88	0	0	0	0,68	0,32	0,016
3,75	18294	0	0,59	0	0,03	0	0,04	0	0,34	0	0	0,53	0	0,47	0,048
3,75	10100	0	0,12	0	0,01	0,85	0,01	0	0,01	0	0,12	0,02	0,86	0	0,042
3,75	17986	0,04	0	0	0	0,94	0	0,01	0	0,01	0	0,04	0,01	0,95	0,048
3,75	23045	0,02	0	0,04	0	0	0	0	0,94	0	0	0,87	0,11	0,02	0,042
3,75	7815	0	0	0,04	0	0,73	0	0,02	0,21	0	0,68	0,03	0,29	0	0,044
3,75	13279	0	0,12	0	0	0,88	0	0	0	0	0,12	0	0,88	0	0,048
3,75	27609	0	0,21	0	0	0,76	0,01	0	0,02	0	0	0,03	0,58	0,39	0,04
3,75	41183	0	0	0	0,36	0,64	0	0	0	0	1	0	0	0	0,046
3,75	26744	0	0,05	0,01	0	0,94	0	0	0	0	0,94	0,01	0,05	0	0,042
3,75	9454	0	0	0	0,07	0,93	0	0	0	0	0,07	0,93	0	0	0,036
3,75	46328	0	0,01	0	0,19	0,24	0,56	0	0	0	0,56	0,43	0	0,01	0,048
3,75	12400	0	0,06	0	0,04	0,82	0	0	0,08	0	0	0,1	0,9	0	0,036
3,75	12389	0	0	0	0,01	0,93	0	0,06	0	0	0,01	0,98	0	0,01	0,044
3,75	23620	0	0,27	0	0	0,73	0	0	0	0	0,27	0	0	0,73	0,044
3,75	20188	0,06	0	0	0	0,89	0,02	0	0,03	0	0,09	0,01	0,03	0,87	0,044
3,75	13184	0	0,02	0	0,11	0,87	0	0	0	0	0	0	0,87	0,13	0,046
3,75	36865	0,05	0	0	0	0	0,92	0	0,02	0,01	0	0	0,94	0,06	0,044
3,75	13912	0,01	0	0,01	0,15	0	0	0	0,83	0	0,83	0,01	0	0,16	0,04
3,75	21047	0,04	0,19	0,02	0	0,75	0	0	0	0	0	0,75	0,06	0,19	0,044
3,75	20304	0	0,08	0	0	0,92	0	0	0	0	0,92	0	0	0,08	0,044
3,75	27031	0	0	0	0,09	0,91	0	0	0	0	0	0,91	0,09	0	0,048

3,75	7629	0,06	0	0	0	0,92	0	0,01	0,01	0	0,01	0	0,99	0	0,048
3,75	27437	0	0	0,04	0	0	0	0	0,96	0	0	0,36	0,02	0,62	0,002
3,75	8272	0,02	0	0,04	0	0,9	0	0,04	0	0	0,01	0,93	0,02	0,04	0,048
3,75	15945	0,04	0,96	0	0	0	0	0	0	0	0	0	0	1	0,046
3,75	44921	0,05	0,02	0,01	0	0,02	0	0	0,9	0	0,02	0,91	0	0,07	0,048
3,75	57058	0,02	0,04	0	0	0,93	0	0,01	0	0	0,93	0,04	0,01	0,02	0,036
3,75	24630	0,06	0	0,01	0	0,93	0	0	0	0	0,06	0,01	0	0,93	0,048
3,75	11673	0	0	0,04	0	0	0,09	0	0,87	0	0	0,04	0,96	0	0,026
3,75	10060	0	0,09	0,01	0	0,62	0,18	0	0,1	0	0,62	0	0,27	0,11	0,048
3,75	13608	0,02	0,03	0,02	0,02	0,76	0,14	0	0	0,01	0,73	0,14	0,09	0,04	0,036
3,75	15493	0	0,1	0	0	0,04	0,86	0	0	0	0,1	0,04	0,86	0	0,042
3,75	17889	0	0,13	0	0	0,86	0	0	0	0,01	0,13	0,01	0,86	0	0,04
3,75	9806	0	0	0,02	0	0	0,85	0,13	0	0	0,52	0,35	0	0,13	0,044
3,75	43371	0	0,22	0	0,03	0,01	0,09	0	0,59	0,06	0,15	0	0	0,85	0,042
3,75	29725	0	0	0	0,12	0	0	0	0,85	0,03	0,03	0,12	0,85	0	0,04
3,75	9886	0,07	0	0	0	0,68	0	0	0,25	0	0,25	0	0	0,75	0,046
3,75	8755	0,05	0,05	0	0	0,17	0,04	0	0,69	0	0	0,22	0,05	0,73	0,044
3,75	20980	0	0	0	0,07	0,89	0	0	0,04	0	0,03	0,93	0,04	0	0,044
3,75	8674	0,06	0	0,01	0	0,43	0,45	0	0	0,05	0,44	0,05	0,14	0,37	0,048
3,75	16941	0,06	0	0,02	0	0,42	0,12	0	0,38	0	0,09	0,39	0,08	0,44	0,048
3,75	12670	0,06	0	0	0	0,02	0,02	0	0,9	0	0,16	0	0,82	0,02	0,044
3,75	19853	0	0,95	0,03	0,02	0	0	0	0	0	0,02	0	0	0,98	0,042
3,75	14256	0	0,07	0,01	0	0,87	0,03	0	0	0,02	0,87	0,06	0,01	0,06	0,048
3,75	15593	0	1	0	0	0	0	0	0	0	0	1	0	0	0,048
3,75	16695	0	0	0	0,13	0,84	0,03	0	0	0	0,35	0,62	0,03	0	0,046
3,75	10316	0,01	0,06	0	0,02	0,9	0,01	0	0	0	0,9	0,01	0,02	0,07	0,042
3,75	23036	0	0,05	0,04	0,03	0,88	0	0	0	0	0,11	0,03	0,21	0,65	0,04
3,75	5464	0,02	0,06	0	0,03	0,74	0,09	0	0,06	0	0,56	0	0,14	0,3	0,046
3,75	15689	0,04	0	0	0	0,89	0	0,05	0,02	0	0,89	0	0,04	0,07	0,048
3,75	20473	0,03	0,58	0	0	0,38	0	0	0	0,01	0,01	0,27	0,02	0,7	0,044
3,75	8429	0	0,07	0	0	0,89	0,03	0,01	0	0	0,89	0,01	0,1	0	0,048
3,75	10988	0,04	0	0,01	0	0,94	0	0,01	0	0	0,03	0,01	0,02	0,94	0,048
3,75	30420	0	0,09	0	0,04	0,87	0	0	0	0	0,75	0	0	0,25	0,048

3,75	10647	0	0,11	0	0,01	0,77	0,11	0	0	0	0,21	0,01	0,78	0	0,044
3,75	4869	0,05	0	0,01	0	0	0,21	0	0,73	0	0,73	0,21	0,05	0,01	0,048
3,75	29805	0	0,07	0,01	0	0,92	0	0	0	0	0,92	0	0,07	0,01	0,048
3,75	6972	0,05	0	0	0	0,81	0,13	0	0	0,01	0	0,06	0,83	0,11	0,044
3,75	31967	0	0,14	0	0	0,85	0	0,01	0	0	0,15	0,77	0,08	0	0,044
3,75	26602	0	0,02	0	0,09	0,67	0,01	0	0,21	0	0,01	0,21	0,76	0,02	0,048
3,75	66560	0	0	0	0,13	0,82	0,05	0	0	0	0,15	0	0,85	0	0,048
3,75	7770	0,07	0	0	0	0,92	0,01	0	0	0	0,02	0	0	0,98	0,046
3,75	8625	0	0	0	0,13	0,87	0	0	0	0	0,13	0,12	0,75	0	0,048
3,75	7035	0,03	0,1	0	0	0,55	0	0	0,32	0	0,33	0,11	0,01	0,55	0,046
3,75	21097	0	0,34	0	0	0,64	0,01	0	0	0,01	0,01	0,01	0,21	0,77	0,034
3,75	27487	0,04	0	0	0	0,18	0,09	0,03	0,66	0	0	0,04	0,69	0,27	0,048
3,75	8900	0	0,95	0	0,02	0	0	0	0,03	0	0,03	0	0	0,97	0,044
3,75	11064	0,05	0	0,01	0	0,91	0,02	0	0	0,01	0	0,03	0,05	0,92	0,042
3,75	14634	0,06	0	0	0	0,89	0	0	0,04	0,01	0,87	0,05	0,02	0,06	0,044
3,75	8462	0	0,19	0,01	0,05	0,74	0,01	0	0	0	0	0	0,01	0,99	0,042
3,75	13208	0,02	0	0	0,05	0,77	0,16	0	0	0	0,02	0,77	0,16	0,05	0,046
3,75	12647	0	0	0	0,18	0,81	0	0,01	0	0	0,81	0	0	0,19	0,046
3,75	31370	0,01	0	0,04	0,01	0,05	0	0	0,89	0	0,17	0,06	0	0,77	0,02
3,75	20688	0	0,16	0	0	0,76	0,03	0	0,05	0	0,05	0,03	0,92	0	0,048
3,75	14978	0,01	0	0	0,2	0,78	0	0	0,01	0	0,83	0,16	0	0,01	0,04
3,75	8928	0,05	0	0,02	0	0,16	0	0	0,77	0	0	0,02	0	0,98	0,034
3,75	9821	0	0	0	0,06	0	0	0	0,94	0	0	0,06	0	0,94	0,048
3,75	7675	0,01	0	0	0	0	0,92	0,07	0	0	0	0	0,16	0,84	0,048
3,75	21474	0,01	0	0	0,15	0,16	0	0	0,68	0	0,68	0	0,01	0,31	0,046
3,75	47448	0	0	0,04	0	0	0,89	0,01	0,06	0	0,07	0,12	0,81	0	0,026
3,75	20522	0,07	0	0	0,01	0,92	0	0	0	0	0,94	0,04	0	0,02	0,048
3,75	10590	0,03	0	0	0,02	0,95	0	0	0	0	0,08	0,02	0	0,9	0,042
3,75	10724	0	0,91	0	0	0,03	0	0	0	0,06	0,05	0	0	0,95	0,044
3,75	34836	0	0,09	0	0,05	0,85	0	0	0	0,01	0	0,1	0,9	0	0,048
3,75	6928	0,04	0,01	0	0	0	0,18	0	0	0,77	0,19	0,77	0,04	0	0,044
3,75	23971	0,04	0	0	0	0,02	0,02	0	0,91	0,01	0	0,04	0,04	0,92	0,042
3,75	12579	0	0,01	0	0,05	0,93	0	0	0,01	0	0,04	0,93	0,02	0,01	0,034

3,75	8406	0,03	0	0,03	0	0,93	0	0	0,01	0	0,03	0,02	0,95	0	0,046
3,75	6094	0	0	0,03	0,15	0,72	0	0	0,1	0	0,72	0,1	0,18	0	0,046
4	29621	0,04	0,01	0	0	0,62	0,32	0	0	0,01	0,67	0	0,33	0	0,048
4	13491	0	0	0	0,17	0,66	0,17	0	0	0	0,72	0,11	0,01	0,16	0,044
4	7766	0,05	0	0	0	0,82	0,09	0	0,02	0,02	0,84	0,06	0,06	0,04	0,038
4	36383	0,04	0	0,03	0,01	0,91	0	0	0	0,01	0,01	0,94	0,04	0,01	0,044
4	15163	0,03	0	0	0,06	0,01	0	0	0,9	0	0,28	0	0,69	0,03	0,042
4	28704	0,02	0	0	0,07	0	0,83	0	0	0,08	0,02	0	0,9	0,08	0,048
4	14569	0,05	0	0	0,01	0,87	0	0	0,07	0	0,05	0,02	0,93	0	0,044
4	16706	0	0,17	0,02	0,05	0,52	0,17	0	0,07	0	0,08	0,22	0	0,7	0,048
4	8101	0,01	0,96	0	0,03	0	0	0	0	0	0,01	0,02	0	0,97	0,036
4	15784	0,02	0,1	0	0	0,64	0	0	0,21	0,03	0,31	0,01	0,04	0,64	0,048
4	24333	0,06	0	0	0	0	0	0	0,94	0	0,92	0	0,01	0,07	0,048
4	21061	0,02	0,15	0	0	0,83	0	0	0	0	0,01	0,03	0,38	0,58	0,048
4	49638	0,02	0,05	0	0	0,93	0	0	0	0	0,98	0	0,01	0,01	0,046
4	14395	0	0	0	0,07	0,12	0	0	0,81	0	0	0,91	0,08	0,01	0,042
4	7440	0,07	0	0	0	0,91	0,01	0	0	0,01	0,01	0,92	0,07	0	0,046
4	14081	0,04	0	0,03	0	0,9	0,03	0	0	0	0,64	0,02	0,3	0,04	0,048
4	21797	0,05	0,01	0	0,01	0,91	0,01	0	0	0,01	0,03	0,61	0,36	0	0,046
4	35705	0,01	0,05	0	0,03	0,79	0,11	0	0	0,01	0,79	0,12	0,08	0,01	0,038
4	15387	0,04	0	0,02	0	0,94	0	0	0	0	0,82	0,16	0	0,02	0,048
4	10798	0,01	0	0	0,21	0,01	0,77	0	0	0	0,98	0,01	0,01	0	0,04
4	6528	0,02	0	0,04	0	0,85	0	0	0	0,09	0,02	0,06	0,83	0,09	0,046
4	7032	0,01	0,03	0	0,07	0,89	0	0	0	0	0	0,04	0,96	0	0,036
4	36862	0	0	0	0,07	0,92	0,01	0	0	0	0,07	0,8	0,01	0,12	0,048
4	31796	0,05	0,01	0,01	0	0,73	0,11	0	0	0,09	0,01	0,33	0,55	0,11	0,04
4	17613	0	0,01	0	0,15	0,74	0,09	0	0	0,01	0,54	0,09	0,36	0,01	0,048
4	40867	0	0	0,03	0,06	0,53	0,38	0	0	0	0,03	0,03	0	0,94	0,046
4	24940	0,04	0	0,03	0	0,86	0,06	0,01	0	0	0,02	0,06	0,02	0,9	0,04
4	16045	0,04	0	0,01	0,01	0,93	0	0	0,01	0	0,04	0,01	0	0,95	0,048
4	32524	0	0,05	0,03	0,04	0	0	0	0,88	0	0	0,09	0,91	0	0,046
4	14215	0,01	0,2	0	0,01	0,66	0	0,01	0,11	0	0,12	0,22	0,01	0,65	0,042
4	36868	0	0,02	0	0,16	0,82	0	0	0	0	0,8	0,02	0,18	0	0,044

4	14258	0	0	0	0,16	0	0,79	0	0,05	0	0,6	0	0,16	0,24	0,038
4	6794	0,04	0	0,04	0	0,7	0	0	0,21	0,01	0	0,04	0,92	0,04	0,048
4	66173	0	0,28	0,01	0	0	0	0	0,71	0	0,27	0	0,02	0,71	0,046
4	30963	0	0,01	0	0,13	0,85	0	0	0,01	0	0,17	0,12	0,71	0	0,048
4	15771	0	0	0	0,09	0,47	0,19	0	0,18	0,07	0,22	0,47	0,31	0	0,038
4	18922	0,05	0,02	0	0	0,93	0	0	0	0	0,08	0,02	0,88	0,02	0,044
4	8662	0,06	0	0	0	0,89	0,04	0	0	0,01	0	0,01	0,98	0,01	0,042
4	11567	0	0	0	0,23	0,72	0	0	0,04	0,01	0,73	0	0,27	0	0,042
4	24273	0,06	0	0	0	0,92	0	0	0,02	0	0,06	0,02	0,92	0	0,042
4	35611	0,04	0,03	0	0	0,93	0	0	0	0	0	0,07	0,66	0,27	0,044
4	13388	0	0,09	0	0	0,87	0	0	0	0,04	0,98	0	0,02	0	0,046
4	8314	0	0,01	0	0,15	0	0,51	0	0,3	0,03	0,3	0,03	0,54	0,13	0,046
4	24003	0	0,01	0	0,1	0,89	0	0	0	0	0,1	0,74	0,16	0	0,048
4	9665	0,03	0,67	0	0	0,28	0,01	0,01	0	0	0	0,28	0,01	0,71	0,046
4	27349	0	0,04	0	0,09	0	0,09	0	0,75	0,03	0,11	0,03	0,73	0,13	0,046
4	4248	0,05	0,01	0	0	0,93	0	0	0,01	0	0,93	0	0,01	0,06	0,048
4	14292	0,03	0	0,03	0	0,77	0,17	0	0	0	0,8	0,19	0	0,01	0,04
4	15832	0,05	0	0	0,01	0,94	0	0	0	0	0	0	0,95	0,05	0,048
4	11219	0,06	0	0	0	0,83	0,1	0	0,01	0	0,83	0,1	0,06	0,01	0,046
4	18703	0	0,72	0	0	0,23	0,05	0	0	0	0,05	0,22	0,01	0,72	0,046
4	13544	0	0	0	0,22	0,78	0	0	0	0	0,78	0,22	0	0	0,042
4	12794	0,04	0	0,02	0	0,94	0	0	0	0	0,94	0,02	0,02	0,02	0,048
4	6307	0,04	0,03	0,03	0	0,64	0,02	0	0,24	0	0	0,02	0,33	0,65	0,046
4	21200	0	0	0,04	0	0,87	0,09	0	0	0	0	0,05	0,95	0	0,026
4	17631	0,02	0,01	0,03	0	0	0,9	0,04	0	0	0,93	0	0,03	0,04	0,042
4	12823	0,01	0	0,04	0,06	0	0,58	0	0	0,31	0,31	0,03	0,57	0,09	0,046
4	23658	0,07	0	0,01	0	0,89	0,01	0	0	0,02	0,01	0,89	0,02	0,08	0,048
4	46069	0,05	0,02	0	0	0,93	0	0	0	0	1	0	0	0	0,048
4	6505	0,06	0	0	0	0	0	0	0,92	0,02	0	0,87	0,07	0,06	0,046
4	62874	0	0,05	0	0,07	0,14	0	0	0,74	0	0,52	0,07	0,14	0,27	0,048
4	15908	0	0	0	0,07	0	0	0	0,92	0,01	0,01	0,14	0	0,85	0,042
4	53267	0	0,06	0	0	0	0,94	0	0	0	0,94	0	0,06	0	0,046
4	10936	0,01	0,9	0	0	0,04	0,01	0	0,03	0,01	0,04	0	0,02	0,94	0,04

4	3678	0,02	0	0	0,03	0,94	0	0,01	0	0	0,03	0,95	0,02	0	0,048
4	14724	0,04	0	0	0,01	0	0,94	0	0	0,01	0,04	0,01	0,35	0,6	0,048
4	10086	0,03	0,04	0	0	0,18	0	0	0,75	0	0	0	0,9	0,1	0,036
4	4573	0,06	0	0	0	0,89	0	0	0,05	0	0	0	0,93	0,07	0,048
4	14001	0,08	0	0	0	0	0,92	0	0	0	0,08	0	0	0,92	0,048
4	19838	0,05	0	0	0	0	0	0	0,95	0	0	0	0,21	0,79	0,048
4	28829	0	0	0	0,16	0,83	0,01	0	0	0	0,84	0	0	0,16	0,038
4	10943	0,04	0	0,04	0	0,92	0	0	0	0	0	0,88	0,11	0,01	0,044
4	42590	0	0	0	0,08	0,9	0,02	0	0	0	0,08	0,9	0,02	0	0,044
4	8051	0,02	0	0	0,04	0,79	0,15	0	0	0	0	0	0,02	0,98	0,046
4	11138	0	0,01	0	0,17	0,82	0	0	0	0	0,82	0,16	0,02	0	0,04
4	23365	0	0,14	0	0	0,86	0	0	0	0	0	0,14	0,86	0	0,044
4	28357	0	0,08	0	0,03	0,89	0	0	0	0	0,98	0	0,02	0	0,042
4	9719	0,01	0,03	0,01	0	0,84	0	0,1	0	0,01	0,95	0,01	0,03	0,01	0,048
4	25420	0,06	0	0	0	0	0,94	0	0	0	0,94	0	0,06	0	0,044
4	76498	0,05	0	0	0	0,01	0,91	0,03	0	0	0,96	0,02	0,01	0,01	0,046
4	19400	0,03	0,95	0,01	0	0,01	0	0	0	0	0	0,04	0,01	0,95	0,046
4	9042	0	0,03	0	0,1	0,87	0	0	0	0	0	0,13	0,87	0	0,046
4	13907	0	0,01	0	0,09	0	0	0	0,9	0	0	0,91	0,09	0	0,046
4	12484	0	0,11	0,01	0	0,82	0,01	0,01	0	0,04	0,57	0,08	0,02	0,33	0,048
4	38347	0,02	0,06	0	0	0	0,83	0	0,07	0,02	0,19	0	0,77	0,04	0,046
4	54435	0	0,32	0	0	0,68	0	0	0	0	0	0,05	0,27	0,68	0,04
4	46133	0,05	0	0	0	0,73	0,22	0	0	0	0,89	0,11	0	0	0,048
4	15724	0,05	0	0	0	0	0	0	0,95	0	0	0,95	0	0,05	0,048
4	7301	0	0	0	0,06	0,94	0	0	0	0	0	0,94	0	0,06	0,046
4	14617	0	0	0	0,07	0,93	0	0	0	0	0	0	0,07	0,93	0,044
4	4280	0	0	0	0,07	0,86	0,01	0,06	0	0	0,06	0,01	0,93	0	0,048
4	5784	0,05	0	0,01	0	0,94	0	0	0	0	0,01	0	0,98	0,01	0,042
4	14686	0	0	0	0,26	0	0,74	0	0	0	0,72	0,23	0,05	0	0,042
4	49707	0,06	0	0	0	0	0,07	0	0,72	0,15	0	0,16	0,76	0,08	0,034
4	7132	0,01	0	0	0,17	0,11	0,68	0	0,03	0	0,66	0,21	0,1	0,03	0,042
4	15383	0	0,22	0	0	0,15	0	0	0,63	0	0,12	0	0,25	0,63	0,048
4	8776	0,04	0,01	0	0	0,02	0	0	0,93	0	0,93	0,02	0,01	0,04	0,036

4	18697	0,05	0	0	0	0	0	0	0,95	0	0,05	0	0,95	0	0,036
4	23905	0,04	0	0,01	0,02	0,93	0	0	0	0	0,03	0	0,04	0,93	0,046
4	74310	0	0	0,01	0,17	0	0,11	0	0,71	0	0,72	0	0,17	0,11	0,046
4,25	25874	0,01	0,32	0,01	0,01	0,65	0	0	0	0	0,19	0	0	0,81	0,046
4,25	18971	0	0,14	0	0	0,86	0	0	0	0	0	0	0,86	0,14	0,046
4,25	18845	0,06	0	0	0	0,32	0,12	0	0,5	0	0,18	0	0,82	0	0,048
4,25	12733	0	0,74	0	0,02	0,03	0	0	0,21	0	0	0	0,05	0,95	0,048
4,25	32233	0	0,02	0	0	0	0	0,19	0,79	0	0,79	0,21	0	0	0,048
4,25	31938	0	0	0,04	0,06	0,87	0	0	0,03	0	0,1	0	0,03	0,87	0,046
4,25	10225	0,04	0	0,02	0	0,92	0	0,02	0	0	0,93	0,02	0,01	0,04	0,048
4,25	26248	0	0,34	0	0,03	0,61	0	0	0	0,02	0	0	0,03	0,97	0,046
4,25	5753	0	0	0,04	0	0,95	0	0	0	0,01	0	0,05	0,95	0	0,024
4,25	20284	0,01	0,1	0	0	0,89	0	0	0	0	0,02	0	0,98	0	0,048
4,25	5923	0,06	0	0	0	0	0,27	0	0,67	0	0,61	0,02	0,25	0,12	0,044
4,25	40823	0,01	0	0,04	0	0	0	0,01	0,94	0	0,01	0,01	0,29	0,69	0,022
4,25	58318	0	0	0,01	0,15	0,83	0	0	0	0,01	0,83	0,02	0	0,15	0,044
4,25	9472	0	0	0	0,11	0,85	0,04	0	0	0	0	0,09	0,85	0,06	0,046
4,25	12574	0	0,12	0,03	0,03	0,82	0	0	0	0	0	0,82	0,15	0,03	0,048
4,25	17126	0,03	0,93	0,04	0	0	0	0	0	0	0	0	0	1	0,036
4,25	10352	0	0,11	0	0	0,05	0,81	0	0,03	0	0	0,03	0,92	0,05	0,036
4,25	5718	0,06	0	0	0	0	0,57	0	0,37	0	0,94	0	0,01	0,05	0,048
4,25	5003	0,01	0	0	0,05	0,94	0	0	0	0	0	0	0,05	0,95	0,048
4,25	18906	0,06	0,08	0,03	0	0,81	0	0	0,02	0	0	0,05	0	0,95	0,048
4,25	18171	0,05	0,01	0	0	0	0	0	0,94	0	0	0,14	0,81	0,05	0,044
4,25	30274	0,02	0,07	0,01	0,02	0,85	0,03	0	0	0	0,04	0,01	0,88	0,07	0,048
4,25	29399	0,05	0	0,01	0	0,6	0,02	0	0,32	0	0,45	0,01	0,54	0	0,048
4,25	24197	0,05	0	0	0	0,45	0,46	0	0,04	0	0	0,05	0,12	0,83	0,038
4,25	4397	0,04	0	0,01	0	0,22	0,04	0,03	0,66	0	0,42	0	0,07	0,51	0,048
4,25	17257	0	0	0,04	0	0,81	0,09	0,05	0	0,01	0,01	0,81	0,09	0,09	0,044
4,25	3181	0,06	0	0	0	0,24	0	0	0,69	0,01	0	0	0,03	0,97	0,048
4,25	23280	0,05	0	0	0	0,95	0	0	0	0	0	1	0	0	0,048
4,25	16967	0,02	0,01	0,04	0	0,93	0	0	0	0	0	0,05	0,11	0,84	0,024
4,25	7190	0,05	0	0	0	0	0,85	0	0,05	0,05	0,01	0	0,9	0,09	0,046

4,25	9667	0,04	0,27	0,02	0	0	0	0	0,67	0	0	0,69	0	0,31	0,046
4,25	9784	0,05	0	0	0	0,93	0,02	0	0	0	0	0,07	0,93	0	0,04
4,25	19850	0	0	0,02	0,11	0,71	0,15	0	0,01	0	0	0,15	0,74	0,11	0,048
4,25	5100	0,05	0	0,01	0	0,93	0	0,01	0	0	0	0,01	0,06	0,93	0,048
4,25	8576	0	0	0,04	0	0,05	0,78	0,13	0	0	0,78	0,22	0	0	0,048
4,25	3611	0,06	0	0	0	0,67	0,27	0	0	0	0	0,27	0,73	0	0,048
4,25	26467	0,01	0	0	0,09	0,89	0,01	0	0	0	0,1	0	0,89	0,01	0,044
4,25	12248	0	0,14	0	0	0,86	0	0	0	0	0	0	1	0	0,044
4,25	17056	0	0	0	0,06	0,01	0	0	0,93	0	0,01	0,99	0	0	0,044
4,25	3487	0,06	0,01	0	0	0,45	0	0	0,48	0	0,01	0	0,06	0,93	0,04
4,25	17100	0	0	0	0,07	0,79	0	0	0,14	0	0	0,93	0,07	0	0,044
4,25	5692	0,05	0	0	0	0	0,01	0	0,91	0,03	0	0,96	0,01	0,03	0,038
4,25	18681	0	0,09	0	0,01	0,89	0	0	0	0,01	0,63	0	0,37	0	0,048
4,25	13155	0,04	0,04	0,03	0	0,89	0	0	0	0	0,04	0,04	0,89	0,03	0,046
4,25	12758	0	0,14	0,01	0	0,55	0,04	0,03	0,11	0,12	0,31	0,12	0	0,57	0,046
4,25	10328	0,05	0	0	0	0	0	0	0,89	0,06	0	0,89	0,05	0,06	0,048
4,25	9003	0	0	0	0,24	0,76	0	0	0	0	0,85	0	0,11	0,04	0,048
4,25	8404	0	0	0	0,07	0,91	0,01	0,01	0	0	0,09	0,77	0	0,14	0,048
4,25	5401	0,06	0	0	0	0,05	0,02	0	0,79	0,08	0,81	0,06	0,13	0	0,034
4,25	28752	0	0	0,01	0,1	0,88	0,01	0	0	0	0,01	0,1	0,88	0,01	0,044
4,25	6058	0,05	0	0,01	0	0,94	0	0	0	0	0,71	0,01	0	0,28	0,044
4,25	22671	0	0	0	0,02	0,86	0,09	0,03	0	0	0,03	0	0	0,97	0,03
4,25	13665	0,06	0,01	0	0	0,05	0	0	0,8	0,08	0,01	0,06	0,8	0,13	0,048
4,25	8843	0,02	0,25	0	0	0,49	0,22	0	0,02	0	0	0,12	0,15	0,73	0,048
4,25	28975	0,02	0	0,04	0	0	0,82	0,06	0	0,06	0,04	0	0,9	0,06	0,048
4,25	3994	0	1	0	0	0	0	0	0	0	0	1	0	0	0,048
4,25	29605	0	0	0	0,12	0,06	0,82	0	0	0	0,02	0,12	0,86	0	0,036
4,25	36025	0,05	0	0	0,05	0,9	0	0	0	0	0,95	0	0,05	0	0,038
4,25	25741	0,04	0,05	0	0	0,87	0,03	0,01	0	0	0,03	0,47	0,07	0,43	0,046
4,25	7747	0	0,09	0,02	0,03	0,82	0,03	0	0	0,01	0,08	0,02	0,9	0	0,046
4,25	58734	0	0,98	0	0	0	0	0	0,01	0,01	0	0,99	0	0,01	0,048
4,25	13041	0,02	0,98	0	0	0	0	0	0	0	0	0	0,02	0,98	0,038
4,25	17096	0	0,53	0	0,04	0,15	0	0,01	0,27	0	0	0,36	0	0,64	0,046

4,25	18087	0,05	0	0	0	0,93	0	0,02	0	0	0,09	0,84	0,07	0	0,044
4,25	3168	0,03	0	0,02	0	0,92	0	0,03	0	0	0	0,05	0,03	0,92	0,046
4,25	30078	0	0,14	0	0	0,69	0	0	0,17	0	0,17	0,69	0,14	0	0,042
4,25	27907	0	0,07	0	0	0,93	0	0	0	0	1	0	0	0	0,048
4,25	20238	0,05	0,01	0	0,01	0	0,93	0	0	0	0,98	0,02	0	0	0,046
4,25	33170	0	0,06	0	0,05	0	0	0,02	0,87	0	0,07	0,87	0,06	0	0,048
4,25	30097	0	0	0,02	0,06	0,92	0	0	0	0	0,04	0	0	0,96	0,046
4,25	12765	0	0,04	0	0	0	0,61	0,1	0,23	0,02	0,29	0,03	0,58	0,1	0,048
4,25	47619	0,06	0	0,03	0	0,9	0	0	0	0,01	0,04	0,03	0,02	0,91	0,048
4,25	21108	0,04	0	0	0	0,85	0	0	0,11	0	0	0,04	0	0,96	0,03
4,25	21127	0,03	0	0	0,02	0,93	0	0,02	0	0	0,02	0,9	0,03	0,05	0,042
4,25	6956	0,05	0	0	0	0,83	0,06	0	0	0,06	0,06	0,91	0,03	0	0,048
4,25	20290	0	0	0	0,19	0,65	0,03	0	0,13	0	0,65	0,22	0,13	0	0,042
4,25	7070	0,01	0,04	0,03	0	0,77	0,03	0,06	0,06	0	0,04	0,07	0,12	0,77	0,046
4,25	11127	0,03	0,06	0	0	0	0,26	0,01	0,64	0	0,32	0	0,67	0,01	0,048
4,25	12978	0	0,02	0	0,17	0,79	0,02	0	0	0	0,86	0,06	0,02	0,06	0,048
4,25	5290	0,03	0	0	0,04	0,93	0	0	0	0	0,93	0	0,04	0,03	0,048
4,25	8275	0	0,12	0	0,01	0,87	0	0	0	0	0	0	1	0	0,048
4,25	7156	0,01	0,06	0,02	0,04	0,87	0	0	0	0	0,02	0,01	0,91	0,06	0,046
4,25	10184	0,01	0,06	0	0	0,85	0,07	0	0	0,01	0,91	0	0,07	0,02	0,048
4,25	21797	0,06	0	0	0	0	0	0	0,94	0	0	0	0,87	0,13	0,048
4,25	47438	0	0,24	0,04	0	0	0	0,01	0,71	0	0	0,24	0,01	0,75	0,024
4,25	21620	0,03	0	0	0	0,32	0	0,03	0,62	0	0	0,03	0	0,97	0,048
4,25	25094	0,05	0	0,01	0,01	0	0,01	0,01	0,86	0,05	0,01	0	0,92	0,07	0,044
4,25	31440	0	0	0	0,29	0	0,7	0	0,01	0	0,96	0,03	0,01	0	0,044
4,25	48465	0,04	0	0,02	0	0,26	0,6	0	0,08	0	0,86	0	0,02	0,12	0,048
4,25	27186	0,01	0,2	0	0,01	0,75	0	0	0,03	0	0	0,83	0,14	0,03	0,048
4,25	67339	0,06	0	0	0	0,88	0,06	0	0	0	0,88	0,06	0,06	0	0,048
4,25	53720	0,04	0,96	0	0	0	0	0	0	0	0	0	0	1	0,046
4,25	8976	0	0,19	0	0,01	0,8	0	0	0	0	0	0,77	0,23	0	0,046
4,25	30037	0	0	0,02	0,06	0,91	0	0	0	0,01	0,01	0,2	0,06	0,73	0,046
4,25	46530	0,06	0	0	0	0,07	0,84	0	0,02	0,01	0,06	0,07	0	0,87	0,044
4,25	8123	0,02	0,04	0	0	0,93	0	0	0,01	0	0,86	0,06	0,02	0,06	0,048

4,25	14142	0,01	0	0,04	0	0,95	0	0	0	0	0,95	0,04	0,01	0	0,048
4,25	27752	0	0	0	0,06	0,93	0,01	0	0	0	0	0,06	0	0,94	0,048
4,25	16709	0	0,07	0	0	0,61	0,32	0	0	0	0,92	0,08	0	0	0,048
4,25	32686	0	0,03	0	0,11	0,86	0	0	0	0	0,09	0	0,91	0	0,046
4,5	24561	0	0,14	0	0	0,85	0	0	0	0,01	0,05	0	0,81	0,14	0,048
4,5	17691	0,02	0,06	0	0,01	0,73	0,17	0	0	0,01	0,24	0,42	0,32	0,02	0,048
4,5	11284	0	0,93	0	0,03	0	0,04	0	0	0	0	0,03	0	0,97	0,046
4,5	37949	0	0,08	0	0,03	0	0	0	0,89	0	0	0,06	0,92	0,02	0,048
4,5	27703	0,05	0	0	0	0,88	0	0,06	0	0,01	0,94	0	0,06	0	0,048
4,5	7389	0,05	0	0,01	0	0	0	0,01	0,93	0	0	0,93	0,07	0	0,044
4,5	17098	0,01	0,16	0	0	0	0,8	0	0	0,03	0	0,8	0,05	0,15	0,044
4,5	46605	0,03	0,07	0	0	0,9	0	0	0	0	0,01	0,11	0,87	0,01	0,044
4,5	15741	0,01	0	0	0,07	0	0	0	0,92	0	0,01	0,07	0,41	0,51	0,048
4,5	19420	0,05	0	0	0	0,8	0,13	0,01	0,01	0	0,06	0,91	0	0,03	0,048
4,5	26741	0,06	0	0	0,01	0	0,93	0	0	0	0,93	0	0,01	0,06	0,046
4,5	12814	0	0,73	0	0,04	0,23	0	0	0	0	0	0	0,02	0,98	0,048
4,5	5689	0,03	0	0	0,01	0,03	0	0	0,93	0	0,04	0	0	0,96	0,048
4,5	23438	0	0,17	0	0	0,21	0,47	0	0,15	0	0	0,02	0,64	0,34	0,046
4,5	16754	0	0,01	0	0,15	0,82	0	0	0,02	0	0,51	0,32	0,17	0	0,048
4,5	20166	0	0,01	0,01	0,07	0,91	0	0	0	0	0	0,99	0,01	0	0,048
4,5	24164	0	0,07	0	0,06	0,87	0	0	0	0	0,07	0,87	0,06	0	0,044
4,5	20335	0,05	0	0	0	0,04	0	0	0,81	0,1	0,1	0,04	0	0,86	0,046
4,5	19078	0,05	0	0,02	0	0,29	0	0	0,59	0,05	0	0,65	0,02	0,33	0,044
4,5	41965	0	0	0	0,08	0,9	0	0,01	0	0,01	0,08	0,91	0,01	0	0,044
4,5	37420	0,04	0	0	0,07	0,88	0	0,01	0	0	0,82	0,03	0,14	0,01	0,042
4,5	9547	0,02	0	0,04	0	0,74	0	0	0,2	0	0	0,23	0,03	0,74	0,026
4,5	38647	0	0	0	0,23	0,38	0,37	0,01	0,01	0	0,83	0,02	0	0,15	0,042
4,5	13940	0	0	0	0,07	0,9	0,03	0	0	0	0,07	0,03	0	0,9	0,046
4,5	26081	0	0,03	0	0,05	0,89	0,01	0	0,01	0,01	0,03	0,04	0,01	0,92	0,048
4,5	5657	0	0,12	0	0	0,87	0	0,01	0	0	0,13	0	0,87	0	0,048
4,5	9189	0,02	0,07	0	0	0,91	0	0	0	0	0,42	0,56	0,02	0	0,048
4,5	29030	0,05	0	0,01	0	0,94	0	0	0	0	0,8	0,01	0,19	0	0,044
4,5	17510	0	0,95	0	0,01	0,04	0	0	0	0	0	0,01	0,04	0,95	0,034

4,5	6658	0,01	0,27	0,02	0	0,38	0	0	0,31	0,01	0	0	0,22	0,78	0,048
4,5	5868	0,06	0	0	0	0,93	0	0	0,01	0	0,93	0,01	0	0,06	0,046
4,5	14119	0	0	0	0,08	0,91	0	0	0,01	0	0	0,95	0,05	0	0,046
4,5	6996	0	0,08	0	0	0,92	0	0	0	0	0,92	0,06	0	0,02	0,048
4,5	13570	0,02	0,01	0,02	0,09	0,84	0,02	0	0	0	0,89	0,01	0,06	0,04	0,044
4,5	22974	0,02	0,02	0,01	0,03	0	0,1	0	0,82	0	0,01	0,12	0,85	0,02	0,048
4,5	51473	0,05	0,03	0,01	0	0,61	0,21	0	0,05	0,04	0,05	0,09	0,58	0,28	0,048
4,5	8721	0	0,06	0	0	0,94	0	0	0	0	1	0	0	0	0,038
4,5	47200	0	0	0,04	0	0,9	0	0	0,06	0	0	0,94	0,06	0	0,006
4,5	45697	0	0,1	0	0,04	0,86	0	0	0	0	0,31	0,64	0	0,05	0,046
4,5	21072	0,03	0,02	0	0	0	0,92	0	0	0,03	0,94	0,02	0,04	0	0,038
4,5	30773	0,07	0	0	0	0,9	0	0	0	0,03	0,03	0,9	0	0,07	0,048
4,5	12618	0,01	0	0	0,16	0,82	0	0	0	0,01	0,82	0,02	0	0,16	0,048
4,5	27150	0	0,08	0	0,02	0,9	0	0	0	0	0,9	0,08	0	0,02	0,048
4,5	22107	0	0	0,04	0,02	0,93	0	0	0,01	0	0,04	0,01	0,02	0,93	0,024
4,5	17680	0,05	0,01	0,01	0	0,93	0	0	0	0	0,01	0,93	0,01	0,05	0,038
4,5	18867	0	0,13	0	0	0,83	0	0	0,01	0,03	0,01	0	0,99	0	0,034
4,5	6800	0,07	0	0	0	0,92	0,01	0	0	0	0	0,01	0,09	0,9	0,048
4,5	10107	0,05	0,01	0,01	0	0	0,93	0	0	0	0,06	0,01	0,81	0,12	0,048
4,5	9583	0,01	0	0,04	0,03	0,9	0,02	0	0	0	0,86	0,11	0,02	0,01	0,044
4,5	57737	0	0,27	0	0	0,71	0,01	0	0	0,01	0,01	0	0,28	0,71	0,048
4,5	11745	0	0	0	0,11	0,88	0	0	0,01	0	0	0	0,88	0,12	0,048
4,5	5686	0	0	0,04	0	0	0,03	0	0,93	0	0	0,93	0,04	0,03	0,018
4,5	29722	0	0	0	0,11	0,88	0,01	0	0	0	0	0	0,99	0,01	0,046
4,5	25417	0	0,12	0	0,04	0,83	0	0,01	0	0	0,06	0,01	0,1	0,83	0,048
4,5	30562	0	0,19	0	0	0,26	0	0	0,55	0	0	0	0,57	0,43	0,048
4,5	14025	0,03	0	0,01	0,04	0,91	0,01	0	0	0	0,2	0,05	0,72	0,03	0,044
4,5	20901	0,04	0	0	0,02	0,46	0	0	0,48	0	0,85	0	0	0,15	0,038
4,5	25584	0,03	0	0,03	0	0,94	0	0	0	0	0,79	0,03	0,03	0,15	0,048
4,5	3906	0,02	0	0,02	0,08	0	0,03	0,02	0,83	0	0,74	0,1	0	0,16	0,046
4,5	10663	0,06	0	0	0,01	0,4	0	0	0,53	0	0,15	0	0,79	0,06	0,048
4,5	11355	0	0	0	0,1	0,66	0	0	0,24	0	0,37	0	0,1	0,53	0,048
4,5	5245	0,05	0,03	0	0	0,92	0	0	0	0	0,92	0,03	0	0,05	0,044

4,5	14414	0,05	0	0	0	0,95	0	0	0	0	0	0,99	0,01	0	0,044
4,5	2295	0	0	0,04	0	0,96	0	0	0	0	0,96	0,04	0	0	0,04
4,5	3811	0,04	0,01	0,03	0	0	0	0	0,92	0	0,01	0,95	0,04	0	0,036
4,5	58375	0,01	0	0	0,15	0,84	0	0	0	0	0,84	0	0,01	0,15	0,044
4,5	7946	0	0	0	0,32	0,65	0,03	0	0	0	1	0	0	0	0,044
4,5	7771	0	0	0	0,06	0,94	0	0	0	0	0	0,94	0	0,06	0,04
4,5	16798	0	0	0	0,11	0,88	0,01	0	0	0	0	0,11	0,88	0,01	0,046
4,5	37736	0	0	0,02	0,07	0,9	0,01	0	0	0	0,03	0,04	0,02	0,91	0,04
4,5	14440	0	0,33	0,01	0	0,3	0,36	0	0	0	0,03	0,6	0	0,37	0,042
4,5	37946	0	0	0	0,24	0,74	0	0	0,02	0	0,74	0,01	0,25	0	0,048
4,5	35263	0	0	0	0,2	0,79	0,01	0	0	0	0,81	0,02	0,17	0	0,048
4,5	13567	0,06	0	0	0	0,59	0	0,01	0,24	0,1	0,25	0,65	0	0,1	0,048
4,5	32099	0,06	0	0	0	0	0,94	0	0	0	0,94	0	0,06	0	0,046
4,5	26075	0,03	0,04	0	0,04	0,88	0	0,01	0	0	0,88	0,03	0,01	0,08	0,04
4,5	13855	0	0	0	0,2	0	0	0	0,8	0	0,8	0	0	0,2	0,046
4,5	18177	0,05	0	0	0	0,91	0,03	0,01	0	0	0,92	0	0	0,08	0,048
4,5	17219	0,02	0,21	0	0	0,74	0,03	0	0	0	0,21	0,05	0	0,74	0,04
4,5	6726	0	0,03	0	0,07	0	0	0	0,9	0	0	0,07	0	0,93	0,042
4,5	9354	0	0,01	0,01	0,1	0,88	0	0	0	0	0,11	0	0,89	0	0,048
4,5	13989	0,05	0	0	0	0,93	0	0	0,02	0	0	0,95	0	0,05	0,048
4,5	52798	0,06	0	0	0	0,4	0,51	0	0,03	0	0,46	0	0,03	0,51	0,048
4,5	7895	0,02	0	0	0,03	0,9	0	0	0,05	0	0	0	0,1	0,9	0,046
4,5	48146	0,04	0,02	0	0	0,91	0,03	0	0	0	0,02	0,07	0,91	0	0,044
4,5	12303	0	0,06	0	0	0,94	0	0	0	0	0,99	0,01	0	0	0,038
4,5	8734	0,05	0,15	0	0	0,66	0	0	0,14	0	0	0,66	0	0,34	0,048
4,5	12211	0	0	0	0,13	0	0,87	0	0	0	0,13	0,62	0	0,25	0,044
4,5	9663	0	0	0	0,1	0,9	0	0	0	0	0	0,71	0,29	0	0,046
4,5	18019	0,05	0,01	0	0	0	0,92	0,02	0	0	0,93	0,02	0	0,05	0,044
4,5	9154	0,07	0	0,01	0	0	0	0	0,92	0	0,02	0,01	0,91	0,06	0,046
4,5	12045	0,01	0,04	0,01	0,02	0,39	0,03	0,03	0,47	0	0,06	0,05	0,84	0,05	0,038
4,5	13204	0,06	0	0,02	0	0,83	0,06	0,02	0	0,01	0,02	0,02	0,87	0,09	0,048
4,5	14554	0,06	0	0	0	0,94	0	0	0	0	0	0,94	0	0,06	0,042
4,5	16766	0	0	0	0,24	0,73	0	0	0,03	0	0,93	0	0,07	0	0,046

4,5	18236	0	0,05	0	0,07	0,88	0	0	0	0	0	0,12	0,88	0	0,046
4,5	22234	0	0,1	0	0,04	0,24	0	0	0,62	0	0,24	0	0,41	0,35	0,046
4,5	19285	0	0,02	0	0,1	0,8	0	0	0,08	0	0,1	0	0,83	0,07	0,044
4,5	8863	0,01	0,07	0	0	0,79	0,13	0	0	0	0,79	0,08	0	0,13	0,044
4,5	14897	0,04	0,08	0,03	0	0,83	0	0	0,02	0	0,02	0,85	0	0,13	0,04
4,75	19450	0,02	0	0,04	0,01	0,26	0,64	0,03	0	0	0,02	0	0,71	0,27	0,048
4,75	4754	0,05	0	0	0	0,93	0	0	0,02	0	0	0	0,01	0,99	0,046
4,75	12263	0,03	0,02	0,04	0	0,91	0	0	0	0	0	0,04	0,03	0,93	0,022
4,75	8848	0	0	0	0,08	0,29	0,02	0	0,61	0	0,17	0,04	0,13	0,66	0,048
4,75	24201	0	0,02	0,03	0,05	0	0	0	0,9	0	0	0,9	0,07	0,03	0,044
4,75	14184	0	0	0	0,11	0,86	0,03	0	0	0	0,11	0,43	0,43	0,03	0,038
4,75	14859	0	0	0	0,05	0	0	0,01	0,9	0,04	0,04	0,03	0,01	0,92	0,044
4,75	13126	0	0	0	0,11	0,01	0,88	0	0	0	0	0	0,89	0,11	0,046
4,75	30792	0	0	0	0,06	0,94	0	0	0	0	0	0,06	0	0,94	0,048
4,75	8736	0,03	0	0,04	0,01	0,91	0	0	0,01	0	0	0,05	0,91	0,04	0,048
4,75	30842	0	0	0	0,07	0,93	0	0	0	0	0,05	0	0,02	0,93	0,048
4,75	25263	0	0	0,04	0,13	0,83	0	0	0	0	0,99	0,01	0	0	0,046
4,75	12611	0,06	0	0	0	0,93	0	0	0,01	0	0,01	0,95	0,04	0	0,04
4,75	5004	0,05	0	0	0	0,94	0	0,01	0	0	0,01	0	0,93	0,06	0,046
4,75	19719	0	0,05	0,01	0,06	0,87	0,01	0	0	0	0,01	0,88	0,11	0	0,048
4,75	15895	0,05	0,01	0	0	0	0,94	0	0	0	0,94	0	0,06	0	0,044
4,75	15566	0,01	0	0	0,15	0,84	0	0	0	0	0,85	0	0,01	0,14	0,044
4,75	41800	0	0,21	0,01	0	0,49	0,05	0,05	0,19	0	0,01	0	0,07	0,92	0,046
4,75	10873	0,04	0	0	0	0,96	0	0	0	0	0,96	0	0	0,04	0,04
4,75	53558	0,02	0	0,04	0	0,3	0,14	0	0,48	0,02	0,24	0,05	0,56	0,15	0,04
4,75	16919	0,04	0,09	0	0	0,87	0	0	0	0	0,01	0,98	0,01	0	0,046
4,75	15246	0	0	0	0,07	0,93	0	0	0	0	0,07	0,93	0	0	0,048
4,75	28648	0,02	0,05	0	0	0,93	0	0	0	0	0,98	0,02	0	0	0,048
4,75	25369	0	0	0	0,13	0,87	0	0	0	0	0,02	0	0,98	0	0,044
4,75	7493	0,04	0,02	0,04	0	0,02	0	0	0,88	0	0	0,94	0,02	0,04	0,048
4,75	6192	0	1	0	0	0	0	0	0	0	0	1	0	0	0,036
4,75	30876	0	0,23	0	0	0,65	0,11	0	0,01	0	0	0,65	0,22	0,13	0,034
4,75	25447	0	0,11	0	0,04	0	0	0	0,85	0	0,01	0	0,14	0,85	0,044

4,75	4717	0,04	0	0,04	0	0,19	0	0	0,73	0	0,04	0,77	0	0,19	0,048
4,75	26393	0,06	0	0	0	0,94	0	0	0	0	0,05	0,89	0,06	0	0,044
4,75	100000	0,18	0	0,82	0	0	0	0	0	0	0,88	0,02	0	0,1	0,958
4,75	18042	0,06	0	0	0	0,94	0	0	0	0	0	0	0	1	0,048
4,75	11056	0,06	0	0	0	0,9	0,04	0	0	0	0	0	0,96	0,04	0,042
4,75	12127	0	0	0	0,04	0	0,01	0,08	0,87	0	0	0,04	0,95	0,01	0,046
4,75	13731	0,01	0	0	0,09	0	0	0	0,9	0	0,09	0	0,91	0	0,042
4,75	10133	0,03	0,08	0	0	0,86	0	0,02	0,01	0	0,11	0,15	0	0,74	0,042
4,75	12871	0,04	0,1	0	0	0,05	0,75	0	0,06	0	0,06	0	0,16	0,78	0,048
4,75	34714	0,05	0	0	0	0,33	0,62	0	0	0	0,05	0,37	0,57	0,01	0,044
4,75	20863	0	0	0	0,2	0,78	0	0	0	0,02	0,71	0	0,22	0,07	0,048
4,75	11034	0	0,01	0,02	0,14	0,83	0	0	0	0	0,84	0,07	0	0,09	0,042
4,75	27583	0,05	0	0,01	0	0,79	0,12	0	0	0,03	0,79	0,07	0	0,14	0,042
4,75	23344	0	0	0	0,07	0,9	0	0	0,01	0,02	0,07	0,01	0	0,92	0,048
4,75	8356	0	0,07	0	0,02	0,85	0	0,02	0,03	0,01	0,92	0,01	0,07	0	0,046
4,75	7468	0,05	0	0	0	0,94	0	0,01	0	0	0	0,06	0	0,94	0,048
4,75	23794	0,01	0	0	0,09	0,28	0	0	0,62	0	0,34	0,38	0	0,28	0,044
4,75	6881	0,04	0,05	0	0	0,86	0,04	0,01	0	0	0,05	0,9	0,01	0,04	0,042
4,75	14060	0,05	0	0,02	0	0,9	0,02	0	0,01	0	0,03	0,92	0,05	0	0,048
4,75	19967	0,05	0	0,03	0	0,8	0	0	0,11	0,01	0	0,03	0	0,97	0,046
4,75	10681	0,01	0	0	0	0,04	0	0,06	0,88	0,01	0	0,88	0,01	0,11	0,042
4,75	4543	0,06	0	0,01	0	0,01	0	0,01	0,91	0	0,04	0,02	0,06	0,88	0,046
4,75	40248	0,05	0	0	0	0,94	0	0,01	0	0	0,75	0,01	0,24	0	0,036
4,75	16085	0,01	0,04	0,01	0,06	0,86	0	0	0,02	0	0,02	0,1	0,88	0	0,048
4,75	8678	0,05	0	0,02	0	0,04	0	0,01	0,88	0	0	0,94	0,06	0	0,046
4,75	24109	0,05	0	0	0	0	0,1	0	0,85	0	0,15	0,85	0	0	0,038
4,75	10910	0	0	0,01	0,07	0,77	0,14	0,01	0	0	0,21	0	0,06	0,73	0,048
4,75	30710	0,02	0	0	0,07	0,71	0,19	0	0,01	0	0,71	0,19	0,01	0,09	0,046
4,75	12994	0,06	0	0	0	0	0,91	0	0	0,03	0,98	0	0,02	0	0,044
4,75	11745	0	0,24	0,03	0	0	0,26	0,02	0	0,45	0,01	0,04	0	0,95	0,048
4,75	10056	0,05	0,01	0	0	0,93	0	0	0,01	0	0,01	0	0,94	0,05	0,046
4,75	13135	0,06	0	0,01	0	0,01	0,04	0,01	0,87	0	0,05	0,93	0,01	0,01	0,048
4,75	20732	0,03	0	0,04	0,01	0	0,9	0,02	0	0	0,04	0,02	0,91	0,03	0,044

4,75	19244	0	0	0	0,06	0,93	0,01	0	0	0	0,03	0	0,01	0,96	0,048
4,75	7131	0	0	0,04	0	0,14	0	0	0,82	0	0	0,82	0,04	0,14	0,008
4,75	12984	0,04	0	0	0,06	0,01	0,89	0	0	0	0,57	0,01	0,38	0,04	0,048
4,75	5594	0,06	0	0	0	0	0	0	0,94	0	0	0	0	1	0,048
4,75	26124	0,06	0	0	0	0,92	0	0	0	0,02	0,01	0	0,92	0,07	0,048
4,75	5472	0,06	0,03	0	0	0	0	0	0,9	0,01	0	0,01	0,03	0,96	0,048
4,75	17695	0	0	0	0,21	0,69	0,06	0	0,04	0	0,89	0,01	0	0,1	0,044
4,75	36737	0	0,14	0	0	0,83	0	0	0,03	0	0	0,03	0,83	0,14	0,048
4,75	6784	0	0,07	0	0,02	0,79	0,11	0	0,01	0	0,91	0,04	0	0,05	0,048
4,75	37321	0,05	0,01	0	0	0,94	0	0	0	0	0,01	0	0,1	0,89	0,046
4,75	7557	0	0	0	0,06	0,94	0	0	0	0	0,04	0	0,06	0,9	0,046
4,75	6387	0	0	0,01	0,18	0	0,11	0,01	0,69	0	0,51	0,29	0,2	0	0,04
4,75	23002	0	0	0,02	0,11	0,82	0,04	0,01	0	0	0,52	0,04	0,41	0,03	0,04
4,75	38345	0	0	0	0,05	0,92	0	0,02	0	0,01	0,06	0,01	0	0,93	0,046
4,75	5151	0,05	0	0	0	0	0	0	0,95	0	0	0	0,92	0,08	0,036
4,75	30314	0,06	0	0	0	0,81	0,11	0,01	0	0,01	0,01	0,06	0,85	0,08	0,044
4,75	12757	0,04	0	0,01	0	0,95	0	0	0	0	0,04	0,96	0	0	0,046
4,75	53138	0,03	0	0	0,03	0,9	0,03	0	0,01	0	0,02	0,93	0	0,05	0,048
4,75	31872	0	0,14	0	0	0,81	0,05	0	0	0	0	0,02	0,98	0	0,044
4,75	11539	0,05	0	0	0,01	0,84	0	0	0,08	0,02	0,02	0,08	0,9	0	0,048
4,75	19327	0	0,09	0	0	0	0,91	0	0	0	0,68	0	0,09	0,23	0,042
4,75	39624	0	0,07	0	0	0,92	0	0	0	0,01	0,92	0,03	0,04	0,01	0,048
4,75	7406	0,06	0	0	0	0,92	0,01	0	0	0,01	0	0,02	0,93	0,05	0,048
4,75	6707	0,05	0	0	0	0,76	0	0	0,19	0	0,04	0	0,16	0,8	0,048
4,75	42008	0	0	0	0,07	0,93	0	0	0	0	0,07	0,93	0	0	0,046
4,75	20428	0,05	0	0	0	0,9	0,03	0	0	0,02	0,05	0	0,93	0,02	0,048
4,75	9209	0,01	0,91	0	0,01	0	0	0,01	0,06	0	0,01	0	0,01	0,98	0,046
4,75	61404	0	0,17	0	0	0,72	0,11	0	0	0	0,11	0,72	0,17	0	0,042
4,75	9337	0	0	0	0,11	0	0	0	0,89	0	0,11	0,89	0	0	0,048
4,75	33588	0,05	0	0	0,06	0,89	0	0	0	0	0,95	0	0,05	0	0,046
4,75	22856	0,05	0	0,04	0	0,88	0,03	0	0	0	0	0,01	0,06	0,93	0,044
4,75	7071	0,05	0	0	0	0,94	0,01	0	0	0	0	0	0,95	0,05	0,04
4,75	34398	0,01	0	0	0,16	0,83	0	0	0	0	0,79	0	0,09	0,12	0,044

4,75	47116	0	0,03	0	0,07	0,83	0	0	0,07	0	0,07	0,83	0	0,1	0,046
4,75	20501	0	0	0,01	0,06	0,91	0	0,02	0	0	0	0,06	0,92	0,02	0,048
4,75	33505	0,07	0	0	0	0,93	0	0	0	0	0,93	0	0,02	0,05	0,048
4,75	76956	0	0	0	0,06	0,94	0	0	0	0	0	0,06	0	0,94	0,048
4,75	9721	0,03	0	0,02	0,03	0,89	0,02	0,01	0	0	0,03	0,92	0	0,05	0,04
4,75	20420	0,05	0	0	0	0,95	0	0	0	0	0,01	0,04	0	0,95	0,042
5	41030	0,03	0,03	0,04	0	0,25	0,65	0	0	0	0,04	0,05	0,89	0,02	0,048
5	13495	0,04	0,03	0,01	0	0,92	0	0	0	0	0,04	0,34	0,62	0	0,048
5	14932	0	0,67	0	0	0	0	0	0,3	0,03	0	0,23	0,07	0,7	0,04
5	8844	0	0,11	0	0,02	0,85	0	0,02	0	0	0,11	0,85	0,04	0	0,044
5	27074	0	0,14	0	0,02	0	0,04	0	0,8	0	0	0,87	0,13	0	0,046
5	6383	0,02	0	0	0,05	0,57	0,36	0	0	0	0,01	0,45	0,49	0,05	0,042
5	11851	0,06	0	0,01	0	0,93	0	0	0	0	0	0	0,99	0,01	0,048
5	3210	0,03	0,34	0,02	0	0,59	0	0,01	0,01	0	0	0,02	0,03	0,95	0,048
5	20941	0,08	0	0	0	0	0,9	0	0	0,02	0	0,51	0,47	0,02	0,048
5	8560	0	0	0,04	0	0	0	0	0,95	0,01	0,45	0,04	0,01	0,5	0,024
5	58397	0	0,09	0	0	0,79	0,11	0	0	0,01	0,88	0,09	0,02	0,01	0,04
5	7172	0,03	0	0	0,15	0,81	0,01	0	0	0	0,95	0	0,01	0,04	0,038
5	21573	0,05	0	0	0,01	0,94	0	0	0	0	0	0,05	0	0,95	0,048
5	14608	0	0	0,02	0,11	0	0	0	0,87	0	0	0,02	0,87	0,11	0,044
5	29946	0	0,14	0	0	0,8	0,05	0	0	0,01	0,06	0	0,93	0,01	0,048
5	14690	0	0	0,01	0,07	0,7	0	0	0,22	0	0,22	0,07	0,02	0,69	0,046
5	35827	0,04	0,07	0	0	0,88	0	0	0	0,01	0	0	0,95	0,05	0,048
5	31353	0	0	0	0,35	0,65	0	0	0	0	1	0	0	0	0,048
5	35867	0	0,07	0	0,07	0,85	0,01	0	0	0	0,07	0	0,85	0,08	0,048
5	17720	0,03	0	0,04	0	0,4	0,01	0	0,52	0	0,39	0,5	0,07	0,04	0,044
5	7955	0,05	0,02	0,01	0	0	0,35	0	0,57	0	0	0,13	0,36	0,51	0,04
5	23483	0	0,01	0	0,12	0,87	0	0	0	0	0,01	0,12	0,87	0	0,044
5	17178	0	0	0,01	0,07	0,91	0	0	0,01	0	0,08	0	0,17	0,75	0,04
5	17166	0	1	0	0	0	0	0	0	0	0	1	0	0	0,02
5	75251	0,06	0	0,01	0	0,91	0	0	0,01	0,01	0,01	0,75	0,18	0,06	0,038
5	15185	0,03	0	0,03	0,02	0	0	0,02	0,74	0,16	0,25	0,03	0,43	0,29	0,046
5	16985	0	0	0,02	0,08	0,53	0	0	0,31	0,06	0,02	0,15	0,31	0,52	0,048

5	24470	0,05	0	0,01	0	0,94	0	0	0	0	0,94	0,01	0,05	0	0,048
5	87889	0,04	0,01	0,02	0,02	0,91	0	0	0	0	0	0,94	0,02	0,04	0,046
5	53238	0	0	0	0,05	0,94	0	0,01	0	0	0,06	0	0	0,94	0,048
5	12908	0,02	0	0	0,04	0,15	0,06	0	0,73	0	0,02	0	0,19	0,79	0,042
5	22881	0,01	0,04	0	0,06	0,11	0,04	0	0,74	0	0,06	0,9	0	0,04	0,042
5	6942	0,04	0	0,02	0,01	0,92	0	0	0	0,01	0	0,03	0,96	0,01	0,048
5	30115	0,05	0	0	0	0,64	0	0	0,31	0	0,01	0,12	0	0,87	0,046
5	48498	0,05	0	0	0	0,18	0,13	0	0,61	0,03	0,01	0	0,78	0,21	0,044
5	16866	0,07	0	0	0	0,92	0	0	0	0,01	0	0,06	0,93	0,01	0,044
5	9236	0	0	0	0,13	0	0,86	0	0	0,01	0,13	0,87	0	0	0,048
5	26786	0	0,17	0	0,01	0,81	0	0	0,01	0	0,01	0	0,68	0,31	0,048
5	19200	0	0,11	0	0	0,01	0,84	0,02	0	0,02	0,36	0,04	0,57	0,03	0,048
5	19434	0	0	0,02	0,07	0,91	0	0	0	0	0	0,98	0,02	0	0,048
5	41217	0	0,19	0	0	0,81	0	0	0	0	0,19	0,81	0	0	0,046
5	5652	0,06	0	0	0	0	0,68	0	0,21	0,05	0,04	0,69	0,27	0	0,044
5	11602	0	0	0	0,21	0,77	0,01	0	0	0,01	0,77	0,21	0	0,02	0,048
5	6961	0	0,25	0	0	0,72	0	0	0,03	0	0,25	0	0,03	0,72	0,042
5	35884	0	0,04	0	0,06	0	0	0,01	0,89	0	0,04	0,95	0	0,01	0,048
5	14283	0	0,17	0	0	0,81	0,01	0,01	0	0	0,01	0,8	0,18	0,01	0,042
5	20051	0,03	0,81	0	0	0,16	0	0	0	0	0	0,19	0	0,81	0,044
5	12260	0,02	0	0,04	0	0	0	0,01	0,93	0	0,02	0,93	0,02	0,03	0,038
5	6819	0	0	0,04	0,01	0,95	0	0	0	0	0,72	0,04	0	0,24	0,028
5	14768	0	0	0	0,1	0,89	0	0,01	0	0	0	0,01	0,99	0	0,048
5	9981	0,03	0	0,01	0	0,87	0,03	0,03	0,02	0,01	0,83	0,04	0,03	0,1	0,04
5	18446	0	0,08	0	0	0	0,9	0,02	0	0	0,8	0	0,17	0,03	0,048
5	4794	0	0,26	0,02	0	0	0,7	0,01	0	0,01	0	0,27	0	0,73	0,046
5	21859	0	0,31	0	0	0,69	0	0	0	0	0	0	0,31	0,69	0,048
5	17231	0	0	0	0,13	0	0	0,01	0,86	0	0	0,13	0,87	0	0,048
5	10595	0	0	0	0,07	0,93	0	0	0	0	0	0,07	0	0,93	0,048
5	16810	0,05	0,09	0	0	0,81	0	0	0,05	0	0	0,05	0,09	0,86	0,046
5	27479	0	0	0,03	0,12	0,33	0,04	0	0,48	0	0,34	0	0,64	0,02	0,038
5	14208	0,02	0	0,03	0,01	0	0,94	0	0	0	0,92	0	0,04	0,04	0,044
5	21635	0,01	0,05	0	0,02	0,78	0,14	0	0	0	0,84	0,16	0	0	0,044

5	2850	0,06	0	0	0	0,12	0	0	0,57	0,25	0,2	0	0,75	0,05	0,044
5	10882	0,07	0	0,01	0	0,9	0,01	0,01	0	0	0,01	0,01	0,91	0,07	0,046
5	32054	0	0,04	0,04	0,03	0	0	0	0,89	0	0,03	0,04	0,93	0	0,04
5	8270	0,06	0,08	0	0	0,86	0	0	0	0	0,06	0	0,08	0,86	0,048
5	17394	0	0,01	0	0,01	0,92	0,01	0,05	0	0	0,01	0,93	0,01	0,05	0,048
5	11457	0,06	0	0	0	0	0	0,01	0,9	0,03	0	0	0,93	0,07	0,048
5	13943	0	0	0	0,07	0,9	0	0	0,03	0	0	0,9	0,1	0	0,044
5	19537	0	0,02	0	0,12	0,81	0	0	0,05	0	0,12	0	0,83	0,05	0,044
5	15446	0,02	0	0	0,06	0,92	0	0	0	0	0,02	0,92	0,01	0,05	0,044
5	48459	0,05	0	0	0,01	0,49	0,15	0,01	0,29	0	0,05	0,05	0,3	0,6	0,046
5	18171	0	0,1	0	0	0,76	0	0,03	0,1	0,01	0,01	0,01	0,88	0,1	0,046
5	9586	0,05	0	0,01	0	0,68	0,26	0	0	0	0	0,06	0,94	0	0,042
5	19643	0,07	0	0,01	0	0,92	0	0	0	0	0,07	0,09	0	0,84	0,048
5	6441	0,05	0	0	0	0,01	0	0	0,94	0	0	0	0,95	0,05	0,044
5	86540	0,06	0	0	0,01	0,49	0,01	0	0,43	0	0,01	0,49	0,44	0,06	0,044
5	4801	0,01	0	0,04	0	0,94	0	0	0	0,01	0	0,05	0,13	0,82	0,024
5	11386	0,01	0,24	0	0,01	0,72	0	0	0,02	0	0	0,53	0,22	0,25	0,048
5	48865	0,02	0,1	0	0	0,69	0,19	0	0	0	0,02	0	0,98	0	0,046
5	9684	0	0	0,04	0	0	0,43	0	0,53	0	0,39	0	0,53	0,08	0,024
5	12201	0,05	0	0	0	0,92	0	0,01	0	0,02	0	0,01	0,97	0,02	0,042
5	7051	0,06	0	0	0	0,19	0,75	0	0	0	0,25	0	0,75	0	0,042
5	20547	0,07	0	0	0	0	0,93	0	0	0	0	0,07	0,93	0	0,048
5	34426	0,03	0,04	0	0	0,91	0	0,01	0,01	0	0,92	0,02	0,03	0,03	0,048
5	27420	0,05	0,01	0	0,01	0,75	0,18	0	0	0	0,03	0,23	0,73	0,01	0,042
5	18436	0,01	0	0,02	0,08	0,05	0,83	0	0	0,01	0,02	0,11	0,82	0,05	0,046
5	10783	0,06	0	0	0,01	0,93	0	0	0	0	0	0,52	0,15	0,33	0,046
5	29985	0	0,11	0	0,02	0,78	0,06	0,01	0	0,02	0,03	0,02	0,95	0	0,048
5	14945	0,01	0,03	0	0,06	0,89	0	0	0	0,01	0	0	0,98	0,02	0,044
5	17779	0,01	0,17	0	0	0,78	0	0,04	0	0	0,01	0,17	0	0,82	0,048
5	15937	0	0	0,01	0,08	0,39	0,52	0	0	0	0	0,53	0,07	0,4	0,04
5	40866	0	0,9	0,01	0	0,01	0	0	0,08	0	0,08	0,01	0	0,91	0,042
5	32536	0	0,96	0	0,03	0	0	0	0,01	0	0,02	0	0,02	0,96	0,048
5	8289	0,05	0	0	0,01	0,93	0,01	0	0	0	0	0,94	0	0,06	0,042

5	11898	0,01	0,2	0	0	0,79	0	0	0	0	0,02	0,79	0,18	0,01	0,036
5	4666	0,02	0,01	0,01	0	0	0,86	0,1	0	0	0,97	0,02	0	0,01	0,048
5	21048	0,03	0,53	0	0	0,43	0	0	0,01	0	0,03	0,01	0	0,96	0,04
5	6569	0	0	0,02	0,07	0,84	0	0	0,07	0	0	0,93	0	0,07	0,042
5	25370	0	0	0	0,1	0,88	0,01	0,01	0	0	0,01	0,02	0,85	0,12	0,04
5	8658	0	0,02	0,04	0	0,56	0,01	0	0,37	0	0,78	0	0	0,22	0,038
5	10559	0,01	0	0,01	0,09	0,85	0,01	0,02	0,01	0	0,62	0,28	0,1	0	0,038
	18364,73	0,0194	0,09627	0,00905	0,03573	0,58416	0,09576	0,00483	0,14848	0,00636	0,24073	0,23997	0,26264	0,25666	0,044848

stern-judging

b	time_to_cc	(C,C)	(C,D)	(C,P)	(D,C)	(D,D)	(D,P)	(P,C)	(P,D)	(P,P)	S(G,G)	S(G,B)	S(B,G)	S(B,B)	
1	2326	0,01	0,04	0	0,01	0,93	0,01	0	0	0	0,94	0,04	0,01	0,01	0,04
1	1422	0	0,04	0	0,05	0,9	0	0	0	0,01	0,91	0,04	0,05	0	0,03
1	5644	0	0,04	0	0	0,84	0	0	0,12	0	0,12	0,04	0	0,84	0,02
1	8020	0	0,04	0	0	0,96	0	0	0	0	0,94	0,04	0	0,02	0,046
1	7324	0,01	0,04	0	0	0,93	0	0	0,02	0	0,02	0	0,15	0,83	0,034
1	7351	0,01	0,02	0	0,07	0,9	0	0	0	0	0,09	0	0,91	0	0,046
1	2477	0,01	0,04	0,01	0	0,92	0,01	0	0,01	0	0,01	0,04	0,92	0,03	0,04
1	1839	0	0,04	0	0	0,96	0	0	0	0	0,95	0	0	0,05	0,034
1	2105	0	0,04	0	0,02	0,92	0,02	0	0	0	0	0,43	0,55	0,02	0,036
1	1471	0	0,04	0	0	0,94	0	0	0,02	0	0,03	0,04	0,93	0	0,026
1	1723	0	0,04	0	0,08	0,73	0,09	0	0,05	0,01	0,7	0,17	0,13	0	0,044
1	15140	0	0	0	0,07	0,92	0	0	0	0,01	0,03	0,16	0	0,81	0,04
1	1483	0	0,04	0	0,01	0,94	0	0	0	0,01	0	0,99	0,01	0	0,032
1	3776	0	0,04	0	0	0,96	0	0	0	0	0	0,04	0,96	0	0,02
1	4171	0	0,04	0	0	0,94	0	0	0,02	0	0	0,06	0,94	0	0,024
1	3487	0	0,04	0	0	0,96	0	0	0	0	0,94	0,04	0,02	0	0,034
1	6505	0,02	0,04	0	0	0,94	0	0	0	0	0,94	0,04	0,02	0	0,044
1	843	0	0,04	0	0	0,96	0	0	0	0	0,26	0,05	0,69	0	0,03
1	2080	0	0,04	0	0	0,96	0	0	0	0	0,47	0,04	0	0,49	0,018
1	1775	0	0,04	0	0	0,95	0	0	0	0,01	0,01	0,99	0	0	0,03
1	4035	0	0,04	0	0	0,95	0,01	0	0	0	0,02	0,02	0,96	0	0,024
1	3518	0	0,04	0	0	0,95	0	0	0,01	0	0	0,04	0,01	0,95	0,01
1	1950	0	0,04	0	0	0,96	0	0	0	0	0,96	0,04	0	0	0,028

1	1640	0	0,04	0	0	0,96	0	0	0	0	0	1	0	0	0,018
1	11753	0	0,04	0	0,1	0,84	0,02	0	0	0	0,84	0,06	0,1	0	0,044
1	4946	0	0,04	0	0	0,96	0	0	0	0	0,38	0,07	0	0,55	0,024
1	2429	0	0,04	0	0	0,77	0	0,01	0,18	0	0,77	0,04	0,19	0	0,036
1	3173	0	0,04	0	0	0,95	0	0,01	0	0	0,95	0,04	0,01	0	0,036
1	2163	0,01	0,04	0	0	0,95	0	0	0	0	0	0,99	0	0,01	0,022
1	2756	0	0,03	0	0,08	0,89	0	0	0	0	0	1	0	0	0,04
1	1663	0	0,04	0	0	0,96	0	0	0	0	0	0,04	0,96	0	0,022
1	1247	0	0,04	0	0	0,96	0	0	0	0	0,22	0,06	0	0,72	0,022
1	2396	0,01	0,04	0	0	0,94	0,01	0	0	0	0	0,05	0,94	0,01	0,02
1	2108	0	0,04	0	0	0,96	0	0	0	0	0	0,04	0	0,96	0,012
1	4106	0	0,04	0	0	0,94	0	0,01	0,01	0	0,02	0,02	0,02	0,94	0,006
1	8637	0	0	0	0,41	0,59	0	0	0	0	1	0	0	0	0,048
1	7030	0	0,04	0	0	0,96	0	0	0	0	0	1	0	0	0,024
1	1175	0	0,04	0	0	0,91	0	0	0,04	0,01	0	0,98	0,01	0,01	0,026
1	4677	0	0,04	0,01	0	0,94	0	0	0,01	0	0	0,99	0,01	0	0,03
1	1595	0	0,04	0	0	0,96	0	0	0	0	0,42	0,04	0	0,54	0,012
1	4220	0	0,04	0,01	0,09	0,86	0	0	0	0	0,85	0,04	0,05	0,06	0,04
1	6093	0	0,04	0	0	0,83	0	0	0,13	0	0,08	0,85	0	0,07	0,016
1	2446	0	0,04	0	0	0,96	0	0	0	0	0,96	0,04	0	0	0,032
1	7431	0	0,04	0	0	0,95	0	0	0,01	0	0	0,05	0,95	0	0,022
1	3762	0	0	0	0,41	0,59	0	0	0	0	1	0	0	0	0,048
1	7799	0	0,04	0	0	0,96	0	0	0	0	0	0,04	0	0,96	0,006
1	12717	0	0,04	0	0	0,95	0	0	0,01	0	0	0,04	0,01	0,95	0,01
1	1251	0	0,04	0	0,04	0,92	0	0	0	0	0,08	0,07	0,85	0	0,044
1	5059	0,01	0,04	0	0	0,95	0	0	0	0	0	0,04	0	0,96	0,016
1	2772	0,01	0,04	0	0	0,95	0	0	0	0	0,03	0,02	0	0,95	0,016
1	990	0	0,04	0	0,01	0,93	0,02	0	0	0	0,93	0,06	0	0,01	0,034
1	7416	0	0,04	0,01	0	0,9	0	0	0,05	0	0	0,06	0,55	0,39	0,018
1	8714	0	0,04	0	0	0,96	0	0	0	0	0	0,04	0,95	0,01	0,028
1	2158	0	0,04	0	0	0,96	0	0	0	0	0	1	0	0	0,028
1	4374	0	0,04	0,02	0	0,92	0	0,01	0	0,01	0,07	0,05	0,87	0,01	0,036
1	1739	0	0,04	0	0	0,96	0	0	0	0	0	0,04	0,96	0	0,038

1	8690	0	0,04	0	0	0,94	0,01	0	0,01	0	0,94	0,05	0	0,01	0,04
1	1835	0	0,03	0	0,15	0,78	0	0	0	0,04	0,82	0,03	0	0,15	0,042
1	1694	0,01	0,04	0	0	0,95	0	0	0	0	0	0,05	0,95	0	0,034
1	3315	0	0,04	0	0	0,91	0,04	0	0,01	0	0	1	0	0	0,028
1	4209	0	0	0	0,18	0,82	0	0	0	0	0,18	0,82	0	0	0,038
1	1998	0	0,04	0	0	0,95	0	0,01	0	0	0,97	0,03	0	0	0,04
1	2944	0	0,02	0	0,01	0	0	0,07	0,9	0	0,02	0,02	0,02	0,94	0,048
1	1919	0	0,04	0	0	0,94	0	0	0,02	0	0	0,06	0	0,94	0,012
1	1439	0	0,04	0	0,04	0,82	0,1	0	0	0	0,89	0,07	0	0,04	0,04
1	4393	0	0,04	0	0	0,96	0	0	0	0	0	0,17	0,83	0	0,034
1	11077	0,01	0,04	0	0,01	0,94	0	0	0	0	0	0	0,99	0,01	0,04
1	2067	0	0,04	0	0	0,96	0	0	0	0	0	0,04	0	0,96	0,016
1	9378	0	0,04	0	0	0,92	0	0	0,04	0	0	0,96	0	0,04	0,016
1	1311	0,02	0,04	0	0	0,91	0,02	0,01	0	0	0,91	0,04	0,02	0,03	0,046
1	8999	0	0,04	0	0	0,96	0	0	0	0	0	1	0	0	0,02
1	2390	0,01	0,04	0,01	0,01	0,92	0	0,01	0	0	0	0,96	0,03	0,01	0,046
1	5414	0,05	0,04	0	0	0,22	0	0	0,68	0,01	0	0,03	0	0,97	0,048
1	3794	0	0,04	0	0	0,96	0	0	0	0	0	1	0	0	0,024
1	1254	0	0,04	0	0,01	0,94	0	0	0	0,01	0,01	0,98	0,01	0	0,044
1	1461	0	0,04	0	0	0,94	0,02	0	0	0	0,02	0,04	0,94	0	0,026
1	1846	0	0,04	0	0,01	0,82	0,03	0	0,1	0	0,04	0,04	0	0,92	0,022
1	1110	0,03	0,02	0	0	0,92	0	0,01	0,02	0	0,7	0,05	0,25	0	0,038
1	1587	0,01	0,04	0	0	0,95	0	0	0	0	0,96	0,04	0	0	0,046
1	5256	0	0,04	0	0	0,9	0,05	0	0,01	0	0	0,95	0	0,05	0,022
1	6278	0	0,04	0	0	0,96	0	0	0	0	0,96	0,04	0	0	0,036
1	1256	0,01	0,04	0	0	0,94	0	0	0	0,01	0	0,05	0,94	0,01	0,042
1	4554	0	0,04	0	0	0,95	0	0	0	0,01	0	0,04	0,01	0,95	0,008
1	1854	0	0,04	0,01	0	0,95	0	0	0	0	0,52	0,48	0	0	0,044
1	2450	0	0,04	0	0	0,96	0	0	0	0	0	0,04	0	0,96	0,012
1	1686	0	0,04	0	0	0,96	0	0	0	0	0,01	0,99	0	0	0,038
1	1884	0	0,04	0	0	0,96	0	0	0	0	0	0,04	0,95	0,01	0,016
1	8727	0	0,04	0	0	0,95	0	0	0,01	0	0,03	0,95	0,02	0	0,03
1	1156	0	0,04	0	0	0,93	0	0,01	0	0,02	0,95	0,05	0	0	0,036

1	1984	0	0,04	0	0	0,96	0	0	0	0	0	0,94	0	0,06	0,014
1	16285	0	0	0	0,34	0,62	0,03	0	0,01	0	0,96	0,03	0,01	0	0,042
1	12237	0,01	0,04	0	0	0,94	0	0,01	0	0	0,01	0,78	0,16	0,05	0,042
1	4993	0	0,04	0	0	0,95	0,01	0	0	0	0	0,04	0,07	0,89	0,01
1	9068	0	0,04	0	0	0,96	0	0	0	0	0	1	0	0	0,028
1	5307	0,02	0,04	0	0	0,88	0,05	0,01	0	0	0,02	0,06	0,88	0,04	0,044
1	3297	0,03	0,04	0	0	0,93	0	0	0	0	0	0,06	0,01	0,93	0,042
1	5344	0	0,04	0	0	0,96	0	0	0	0	0	0,04	0	0,96	0,008
1	2397	0	0,04	0,01	0	0,95	0	0	0	0	0	0,99	0,01	0	0,028
1	2533	0	0,04	0	0	0,89	0	0	0,07	0	0,07	0,04	0	0,89	0,012
1	9703	0	0,04	0	0	0,95	0	0	0,01	0	0	0,03	0,02	0,95	0,004
1,25	2848	0	0,04	0	0,01	0,95	0	0	0	0	0,93	0,05	0	0,02	0,038
1,25	12171	0	0,04	0	0,03	0,92	0	0	0,01	0	0,01	0,54	0,44	0,01	0,04
1,25	4743	0	0,04	0	0	0,95	0	0	0,01	0	0,01	0,32	0	0,67	0,01
1,25	2253	0	0,04	0	0	0,96	0	0	0	0	0	0,04	0	0,96	0,016
1,25	2402	0	0,04	0,01	0	0,95	0	0	0	0	0	1	0	0	0,028
1,25	11172	0	0	0	0,08	0,91	0,01	0	0	0	0,08	0	0,01	0,91	0,044
1,25	3452	0	0,04	0	0,01	0,86	0,07	0	0,02	0	0	0,98	0	0,02	0,032
1,25	3753	0	0,04	0	0	0,96	0	0	0	0	0,29	0,04	0,67	0	0,026
1,25	3494	0,01	0,04	0	0	0,93	0	0,01	0,01	0	0,02	0,06	0	0,92	0,012
1,25	1863	0	0,04	0	0,01	0,95	0	0	0	0	0,01	0,04	0	0,95	0,018
1,25	3480	0	0	0	0,14	0,86	0	0	0	0	0,05	0,95	0	0	0,048
1,25	6676	0	0,04	0	0	0,95	0	0	0	0,01	0	0,05	0	0,95	0,004
1,25	2286	0	0,04	0	0,01	0,95	0	0	0	0	0,01	0,04	0	0,95	0,018
1,25	8394	0	0	0	0,08	0,88	0,02	0,01	0,01	0	0,09	0	0,01	0,9	0,046
1,25	4543	0,01	0,04	0	0	0,95	0	0	0	0	0	0,98	0,02	0	0,038
1,25	8978	0	0,04	0	0	0,95	0,01	0	0	0	0,18	0,04	0,78	0	0,026
1,25	3649	0	0,04	0	0	0,73	0,22	0	0,01	0	0,92	0,04	0,04	0	0,028
1,25	14678	0	0,04	0	0	0,92	0	0,01	0,03	0	0,01	0,04	0,83	0,12	0,034
1,25	5119	0	0,04	0,01	0	0,94	0,01	0	0	0	0,02	0,04	0	0,94	0,012
1,25	4996	0	0,04	0	0	0,96	0	0	0	0	0,9	0,04	0,06	0	0,028
1,25	1698	0	0,04	0	0	0,96	0	0	0	0	0	0,04	0	0,96	0,008
1,25	1544	0,01	0,04	0	0	0,93	0	0	0,02	0	0,01	0,04	0,93	0,02	0,036

1,25	3098	0	0,04	0	0,01	0,95	0	0	0	0	0,95	0,04	0,01	0	0,034
1,25	1274	0	0,04	0	0	0,96	0	0	0	0	0	0,97	0	0,03	0,02
1,25	4862	0	0,04	0	0	0,96	0	0	0	0	0	0,04	0	0,96	0,008
1,25	4788	0	0,03	0	0,07	0,84	0,03	0,03	0	0	0,06	0,89	0,02	0,03	0,048
1,25	1846	0	0,04	0	0	0,95	0	0,01	0	0	0	0,99	0	0,01	0,038
1,25	9028	0,01	0,04	0	0	0,95	0	0	0	0	0	0,03	0,95	0,02	0,034
1,25	1824	0	0,04	0,01	0	0,91	0	0	0,04	0	0,23	0,73	0,04	0	0,032
1,25	2145	0	0,04	0	0	0,94	0	0,01	0	0,01	0,01	0,05	0,92	0,02	0,03
1,25	1323	0	0,04	0	0	0,95	0	0	0	0,01	0,95	0,04	0,01	0	0,048
1,25	1373	0	0,04	0	0	0,96	0	0	0	0	0	0,04	0,96	0	0,022
1,25	1680	0	0,04	0	0,04	0,92	0	0	0	0	0	1	0	0	0,038
1,25	1517	0	0,04	0	0,01	0,95	0	0	0	0	0	1	0	0	0,024
1,25	2179	0	0,04	0	0,01	0,94	0	0,01	0	0	0	0,18	0,81	0,01	0,04
1,25	12271	0	0,04	0	0	0,96	0	0	0	0	0,02	0,02	0,96	0	0,028
1,25	1344	0	0,04	0	0	0,94	0,01	0	0,01	0	0,94	0,04	0,01	0,01	0,046
1,25	1587	0,01	0,04	0	0	0,95	0	0	0	0	0	0,04	0,95	0,01	0,044
1,25	4760	0	0,04	0	0	0,93	0	0	0	0,03	0	0,99	0	0,01	0,026
1,25	1799	0	0,04	0,01	0	0,95	0	0	0	0	0,95	0,05	0	0	0,044
1,25	10217	0	0,04	0	0	0,94	0	0,02	0	0	0,95	0,04	0,01	0	0,046
1,25	1629	0	0,04	0	0	0,96	0	0	0	0	0	0,04	0	0,96	0,016
1,25	3263	0	0,04	0	0	0,95	0,01	0	0	0	0,03	0,04	0,93	0	0,026
1,25	3441	0	0,04	0	0	0,96	0	0	0	0	0,24	0,76	0	0	0,026
1,25	2093	0	0,04	0	0	0,96	0	0	0	0	0	0,04	0,96	0	0,014
1,25	15100	0	0,04	0	0,02	0,94	0	0	0	0	0,02	0,04	0	0,94	0,03
1,25	1407	0	0,04	0	0	0,95	0	0,01	0	0	0,01	0,04	0	0,95	0,016
1,25	1241	0	0,04	0,01	0,01	0,94	0	0	0	0	0,94	0,05	0	0,01	0,046
1,25	1651	0	0,04	0	0	0,9	0	0	0,06	0	0	0,04	0,9	0,06	0,026
1,25	1756	0	0,04	0	0	0,94	0,02	0	0	0	0	0,98	0,01	0,01	0,034
1,25	1902	0	0,04	0,02	0	0,94	0	0	0	0	0	0,06	0	0,94	0,008
1,25	13286	0	0	0	0,22	0,62	0,15	0	0	0,01	0,62	0,23	0,15	0	0,046
1,25	9679	0	0,04	0	0	0,93	0	0	0,03	0	0,03	0,04	0,93	0	0,02
1,25	4854	0	0,04	0	0	0,96	0	0	0	0	0	0,04	0,96	0	0,02
1,25	3888	0	0,04	0	0	0,96	0	0	0	0	0	0,04	0,46	0,5	0,018

1,25	10506	0	0	0,03	0,14	0,83	0	0	0	0	0,14	0,86	0	0	0,048
1,25	3404	0	0,04	0	0	0,96	0	0	0	0	0	0,04	0	0,96	0,022
1,25	1474	0	0,04	0	0	0,96	0	0	0	0	0,96	0,04	0	0	0,042
1,25	2777	0	0,04	0,01	0	0,84	0	0	0,11	0	0,12	0,04	0,81	0,03	0,04
1,25	1605	0	0,04	0	0,01	0,94	0,01	0	0	0	0,02	0,9	0	0,08	0,03
1,25	1681	0	0,04	0	0	0,85	0,09	0	0,02	0	0,84	0,05	0,02	0,09	0,036
1,25	14257	0	0,04	0	0	0,96	0	0	0	0	0	1	0	0	0,022
1,25	2320	0,01	0,04	0	0	0,95	0	0	0	0	0	0,05	0	0,95	0,018
1,25	12257	0	0,04	0,01	0	0,95	0	0	0	0	0,88	0,1	0	0,02	0,042
1,25	1710	0,02	0,04	0	0	0,94	0	0	0	0	0,94	0,04	0,02	0	0,04
1,25	1882	0	0,04	0	0	0,96	0	0	0	0	0,96	0,04	0	0	0,038
1,25	3202	0	0,04	0	0	0,95	0	0	0	0,01	0	0,05	0	0,95	0,018
1,25	2895	0	0,04	0	0	0,95	0	0	0,01	0	0,95	0,04	0	0,01	0,038
1,25	2080	0	0,04	0	0	0,92	0,04	0	0	0	0	1	0	0	0,036
1,25	984	0	0,04	0	0	0,96	0	0	0	0	0,96	0,04	0	0	0,034
1,25	2463	0	0,04	0	0,05	0,83	0,08	0	0	0	0,04	0,05	0,91	0	0,038
1,25	2937	0	0,04	0	0	0,96	0	0	0	0	0,96	0,04	0	0	0,036
1,25	2150	0	0,04	0	0	0,9	0	0	0	0,06	0,13	0,87	0	0	0,026
1,25	2911	0	0,02	0	0,23	0,74	0	0	0	0,01	0,88	0,12	0	0	0,038
1,25	3084	0	0,04	0	0	0,95	0	0,01	0	0	0,01	0,04	0,95	0	0,04
1,25	1293	0	0,04	0	0	0,95	0,01	0	0	0	0,01	0,04	0,95	0	0,024
1,25	3030	0	0,04	0	0	0,96	0	0	0	0	0	0,04	0	0,96	0,006
1,25	2976	0	0,04	0	0,01	0,94	0,01	0	0	0	0	0,06	0,94	0	0,03
1,25	2146	0	0,04	0	0,01	0,95	0	0	0	0	0,32	0,67	0,01	0	0,04
1,25	1382	0	0,04	0	0	0,92	0	0	0,04	0	0,81	0,19	0	0	0,03
1,25	1213	0	0,04	0	0,12	0,83	0	0	0,01	0	0,83	0,17	0	0	0,044
1,25	3847	0	0,04	0	0	0,96	0	0	0	0	0	1	0	0	0,02
1,25	2711	0	0,04	0	0	0,95	0	0,01	0	0	0,01	0	0	0,99	0,022
1,25	2223	0	0,04	0	0,01	0,95	0	0	0	0	0	0,5	0,01	0,49	0,016
1,25	1625	0	0,04	0	0	0,95	0,01	0	0	0	0,01	0,06	0,93	0	0,016
1,25	7103	0	0,04	0,01	0	0	0	0,01	0,94	0	0,9	0,03	0,05	0,02	0,044
1,25	1720	0	0,04	0	0	0,95	0,01	0	0	0	0	0,99	0,01	0	0,016
1,25	3816	0	0,04	0	0	0,96	0	0	0	0	0	0,53	0	0,47	0,014

1,25	1852	0	0,04	0	0	0,94	0	0	0,02	0	0,88	0,04	0,02	0,06	0,038
1,25	5960	0	0	0	0,29	0,7	0,01	0	0	0	0,69	0,29	0	0,02	0,046
1,25	1300	0	0,04	0	0,01	0,95	0	0	0	0	0,95	0,03	0	0,02	0,036
1,25	6355	0	0	0	0,12	0,84	0,04	0	0	0	0,18	0,63	0,01	0,18	0,04
1,25	3178	0	0,04	0,01	0,12	0,55	0,28	0	0	0	0,97	0,03	0	0	0,048
1,25	9741	0,01	0	0	0,11	0,87	0	0,01	0	0	0,11	0,13	0,76	0	0,042
1,25	2562	0	0,04	0	0	0,95	0	0	0	0,01	0,01	0,98	0,01	0	0,032
1,25	3214	0	0,04	0	0	0,96	0	0	0	0	0,8	0,04	0,16	0	0,03
1,25	9463	0	0,04	0	0	0,91	0	0	0	0,05	0,92	0,04	0	0,04	0,032
1,25	2712	0	0,04	0,01	0,05	0,9	0	0	0	0	0,84	0,11	0,05	0	0,042
1,25	3677	0	0,04	0	0,08	0,88	0	0	0	0	0,82	0,04	0,06	0,08	0,036
1,25	6614	0	0,04	0	0	0,92	0	0,01	0,01	0,02	0	0,04	0,95	0,01	0,026
1,5	8024	0	0,02	0	0,18	0,76	0	0	0,04	0	0,78	0,18	0,02	0,02	0,048
1,5	6160	0	0,04	0	0	0,91	0,04	0,01	0	0	0,01	0,95	0	0,04	0,032
1,5	7150	0	0,04	0	0,01	0,94	0	0,01	0	0	0,77	0,05	0	0,18	0,024
1,5	3371	0	0,04	0	0	0,96	0	0	0	0	0	0,04	0	0,96	0,006
1,5	1444	0	0,04	0	0	0,71	0,24	0,01	0	0	0,72	0,04	0,24	0	0,036
1,5	3011	0	0,04	0,01	0	0	0,03	0,03	0,89	0	0,07	0,05	0,05	0,83	0,022
1,5	1557	0	0,04	0	0	0,95	0	0	0	0,01	0	0,02	0,98	0	0,028
1,5	1678	0	0,04	0,01	0	0,95	0	0	0	0	0,08	0,04	0	0,88	0,022
1,5	1341	0,01	0,04	0	0	0,95	0	0	0	0	0	0,04	0,95	0,01	0,032
1,5	1042	0	0,04	0	0	0,94	0,01	0	0,01	0	0,02	0,04	0,94	0	0,024
1,5	4324	0	0,04	0	0	0,96	0	0	0	0	0,96	0,04	0	0	0,046
1,5	1871	0	0,04	0	0,01	0,91	0	0	0,04	0	0	0,04	0,04	0,92	0,018
1,5	4513	0	0	0	0,2	0,77	0	0,02	0	0,01	0,84	0	0,13	0,03	0,028
1,5	2130	0	0,04	0	0	0,94	0,02	0	0	0	0,8	0,2	0	0	0,032
1,5	9900	0	0,04	0	0	0,96	0	0	0	0	0,01	0,03	0,96	0	0,018
1,5	3002	0	0,04	0	0	0,95	0,01	0	0	0	0,02	0,04	0,94	0	0,028
1,5	1131	0	0,04	0	0	0,93	0	0	0,03	0	0	0,04	0,94	0,02	0,022
1,5	2068	0,01	0,04	0,01	0	0,94	0	0	0	0	0,93	0,06	0,01	0	0,04
1,5	1899	0	0,04	0	0,04	0,87	0	0	0,05	0	0	0,13	0,87	0	0,046
1,5	1246	0	0,04	0	0	0,96	0	0	0	0	0,96	0,04	0	0	0,034
1,5	1676	0	0,04	0	0	0,96	0	0	0	0	0,03	0,97	0	0	0,022

1,5	11271	0	0	0	0,15	0,84	0	0,01	0	0	0,15	0,02	0,83	0	0,048
1,5	3498	0	0,04	0	0	0,96	0	0	0	0	0	0,04	0	0,96	0,014
1,5	3289	0	0,04	0	0	0,96	0	0	0	0	0	0,96	0	0,04	0,02
1,5	1399	0	0,04	0	0	0,91	0	0	0,05	0	0,96	0,04	0	0	0,036
1,5	2228	0	0,04	0	0	0,91	0	0	0,05	0	0,02	0,97	0	0,01	0,03
1,5	2706	0	0,04	0	0	0,96	0	0	0	0	0	0,45	0,03	0,52	0,024
1,5	12782	0	0,04	0	0	0,96	0	0	0	0	0	0,07	0,93	0	0,016
1,5	3428	0	0,04	0	0	0,95	0,01	0	0	0	0,96	0,04	0	0	0,038
1,5	2177	0	0,04	0	0,01	0,95	0	0	0	0	0	0,22	0,01	0,77	0,024
1,5	2700	0	0,04	0	0	0,91	0,05	0	0	0	0,68	0,04	0,23	0,05	0,044
1,5	6462	0	0,04	0	0	0,93	0,01	0,01	0,01	0	0	0,04	0,94	0,02	0,028
1,5	1381	0	0,04	0	0	0,96	0	0	0	0	0,01	0,03	0,96	0	0,032
1,5	1698	0	0,04	0	0	0,87	0,07	0,02	0	0	0,02	0,05	0,93	0	0,044
1,5	6322	0	0,04	0	0	0,96	0	0	0	0	0	0,04	0,96	0	0,034
1,5	3756	0	0,04	0	0,08	0,88	0	0	0	0	0	0,99	0	0,01	0,048
1,5	19124	0	0,04	0	0	0,95	0	0	0	0,01	0	0,04	0,01	0,95	0,006
1,5	1468	0	0,04	0	0	0,9	0	0	0,06	0	0,9	0,04	0,06	0	0,046
1,5	5611	0	0,04	0	0	0,96	0	0	0	0	0	1	0	0	0,03
1,5	10571	0	0,04	0	0	0,96	0	0	0	0	0	0,04	0	0,96	0,01
1,5	3450	0	0,04	0	0,02	0,92	0	0,01	0	0,01	0,02	0,03	0,02	0,93	0,042
1,5	2896	0	0,04	0	0	0,91	0	0	0,03	0,02	0,01	0,04	0,94	0,01	0,032
1,5	2793	0	0,04	0	0	0,96	0	0	0	0	0	0,04	0,36	0,6	0,014
1,5	2758	0	0,04	0	0,01	0,95	0	0	0	0	0	0,04	0,01	0,95	0,018
1,5	17817	0	0,04	0,01	0	0,95	0	0	0	0	0,01	0,98	0	0,01	0,022
1,5	3207	0	0,04	0	0,01	0,94	0	0	0,01	0	0	0,97	0,03	0	0,034
1,5	1652	0	0,04	0	0	0,94	0,02	0	0	0	0,02	0,17	0,67	0,14	0,01
1,5	3288	0	0,04	0	0	0,94	0	0	0,02	0	0,02	0,03	0,06	0,89	0,01
1,5	6744	0,01	0	0	0,28	0,68	0,01	0,02	0	0	0,97	0,01	0,01	0,01	0,04
1,5	6942	0	0,04	0	0	0,95	0	0	0	0,01	0,01	0,89	0,1	0	0,024
1,5	3422	0,01	0,01	0,05	0	0,93	0	0	0	0	0,93	0,03	0,04	0	0,042
1,5	1980	0	0,04	0	0	0,95	0	0	0,01	0	0,01	0,04	0,95	0	0,03
1,5	2992	0	0,04	0	0	0,96	0	0	0	0	0	0,03	0,96	0,01	0,02
1,5	5328	0	0,04	0	0	0,96	0	0	0	0	0,01	0,03	0,96	0	0,02

1,5	7084	0	0,04	0	0	0,96	0	0	0	0	0	1	0	0	0,032
1,5	1861	0	0,04	0	0	0,93	0,03	0	0	0	0,93	0,04	0	0,03	0,042
1,5	2029	0	0,04	0,01	0	0,95	0	0	0	0	0	0,05	0	0,95	0,006
1,5	4040	0	0,04	0	0	0,96	0	0	0	0	0,96	0,04	0	0	0,036
1,5	13038	0	0,04	0	0	0,96	0	0	0	0	0	0,04	0	0,96	0,002
1,5	1058	0	0,04	0	0	0,96	0	0	0	0	0,62	0,38	0	0	0,032
1,5	5386	0	0,04	0	0	0,96	0	0	0	0	0,01	0,99	0	0	0,03
1,5	5835	0,02	0	0	0,16	0,82	0	0	0	0	0,84	0,01	0,13	0,02	0,048
1,5	1457	0	0,04	0	0	0,95	0	0	0,01	0	0	0,05	0,95	0	0,024
1,5	1903	0	0,04	0	0	0,96	0	0	0	0	0	0,03	0,96	0,01	0,026
1,5	2074	0	0,04	0	0,01	0,95	0	0	0	0	0	0,35	0	0,65	0,018
1,5	1571	0	0,04	0	0	0,95	0,01	0	0	0	0,95	0,04	0,01	0	0,028
1,5	1774	0	0,04	0	0,01	0,94	0,01	0	0	0	0	0,05	0,01	0,94	0,02
1,5	4500	0	0,04	0	0	0,96	0	0	0	0	0,01	0,03	0,95	0,01	0,02
1,5	5283	0	0,04	0	0	0,94	0	0	0,02	0	0	0,04	0,94	0,02	0,018
1,5	2351	0	0,04	0	0	0,96	0	0	0	0	0	0,98	0	0,02	0,026
1,5	6591	0	0,04	0	0	0,96	0	0	0	0	0	0,04	0	0,96	0,008
1,5	2663	0	0,04	0	0	0,96	0	0	0	0	0	0,04	0,96	0	0,024
1,5	10872	0	0,04	0	0	0,96	0	0	0	0	0	0,92	0,07	0,01	0,024
1,5	1882	0	0,04	0	0	0,96	0	0	0	0	0	0,03	0,97	0	0,018
1,5	2502	0	0,04	0	0	0,96	0	0	0	0	0	0,04	0	0,96	0,016
1,5	7317	0	0,04	0	0,17	0,77	0,01	0	0,01	0	0,91	0,08	0	0,01	0,048
1,5	10063	0	0,04	0	0	0,93	0,01	0	0,02	0	0,09	0,91	0	0	0,034
1,5	1823	0	0,04	0	0	0,95	0,01	0	0	0	0	0,05	0	0,95	0,01
1,5	1788	0	0,04	0	0	0,94	0	0	0	0,02	0	0,99	0	0,01	0,028
1,5	2311	0	0,04	0	0	0,96	0	0	0	0	0	0,01	0	0,99	0,01
1,5	8124	0	0,04	0	0	0,96	0	0	0	0	0	0,58	0	0,42	0,004
1,5	1798	0	0,04	0,01	0	0,95	0	0	0	0	0,95	0,04	0	0,01	0,046
1,5	3180	0	0,04	0	0	0,94	0	0,02	0	0	0,96	0,02	0,02	0	0,04
1,5	2040	0	0,04	0	0	0,96	0	0	0	0	0,04	0,03	0,92	0,01	0,01
1,5	5101	0	0,04	0	0,01	0,95	0	0	0	0	0	0,69	0,3	0,01	0,026
1,5	1421	0,01	0,04	0	0	0,95	0	0	0	0	0,01	0,89	0,1	0	0,032
1,5	1690	0,01	0,04	0	0,07	0,76	0,1	0	0	0,02	0,87	0,04	0,07	0,02	0,042

1,5	1636	0	0,04	0	0	0,95	0	0	0	0,01	0,21	0,05	0	0,74	0,026
1,5	3962	0	0,04	0	0	0,96	0	0	0	0	0	0,04	0	0,96	0,004
1,5	5737	0	0,04	0	0	0,94	0,02	0	0	0	0,02	0,04	0,94	0	0,028
1,5	2033	0	0,04	0	0	0,96	0	0	0	0	0	0,04	0	0,96	0,018
1,5	4116	0	0,04	0	0	0,95	0	0,01	0	0	0	0,91	0	0,09	0,034
1,5	2171	0	0,04	0	0	0,94	0,01	0	0,01	0	0,01	0,98	0,01	0	0,03
1,5	1878	0,01	0,04	0	0	0,95	0	0	0	0	0,01	0,04	0,95	0	0,028
1,5	2142	0	0,04	0	0	0,93	0	0	0,03	0	0	0,04	0,03	0,93	0,012
1,5	10840	0	0,04	0	0	0,96	0	0	0	0	0,02	0,4	0,58	0	0,024
1,5	3240	0	0,04	0	0	0,96	0	0	0	0	0,01	0,79	0,2	0	0,024
1,5	2537	0	0,04	0	0	0,96	0	0	0	0	0	0,04	0	0,96	0,006
1,5	1434	0	0,04	0	0	0,96	0	0	0	0	0,01	0,03	0,96	0	0,02
1,5	6405	0	0,04	0,02	0,01	0,92	0	0	0,01	0	0,02	0,01	0,95	0,02	0,038
1,75	7931	0	0,04	0	0	0	0	0,02	0,94	0	0	1	0	0	0,026
1,75	2434	0	0,04	0	0	0,95	0	0	0	0,01	0	0,38	0,59	0,03	0,022
1,75	4918	0	0,04	0	0	0,94	0	0	0,01	0,01	0,01	0,98	0,01	0	0,024
1,75	1966	0	0,04	0	0	0,96	0	0	0	0	0	0,04	0	0,96	0,006
1,75	2663	0	0,04	0	0,02	0,94	0	0	0	0	0	0,94	0,04	0,02	0,024
1,75	1353	0,01	0,04	0	0	0,93	0	0	0,02	0	0,03	0,03	0,94	0	0,038
1,75	11008	0	0,04	0	0	0,76	0,2	0	0	0	0,76	0,04	0	0,2	0,032
1,75	4286	0	0,04	0	0	0,95	0	0	0,01	0	0,73	0,04	0,01	0,22	0,026
1,75	2744	0	0,04	0	0	0,95	0,01	0	0	0	0	0,04	0	0,96	0,01
1,75	23264	0	0,04	0	0	0,78	0,18	0	0	0	0,82	0	0	0,18	0,038
1,75	6594	0	0,04	0	0	0,81	0,01	0	0,14	0	0,14	0,04	0,01	0,81	0,014
1,75	1505	0	0,03	0,01	0,14	0,8	0,02	0	0	0	0,8	0,17	0	0,03	0,048
1,75	5578	0	0,04	0	0	0,93	0	0	0,03	0	0	0,04	0	0,96	0,016
1,75	1958	0	0,04	0,01	0,05	0,9	0	0	0	0	0,01	0,02	0,95	0,02	0,046
1,75	1746	0	0,04	0	0,01	0,95	0	0	0	0	0	0,04	0,01	0,95	0,018
1,75	1669	0	0,04	0	0,09	0,86	0	0	0,01	0	0,86	0,04	0,1	0	0,048
1,75	7024	0	0,04	0,01	0	0,93	0	0	0,02	0	0,81	0,04	0,15	0	0,044
1,75	3419	0	0,04	0	0	0,95	0	0	0,01	0	0,58	0,01	0	0,41	0,026
1,75	4777	0	0,04	0	0	0,95	0	0,01	0	0	0,94	0,06	0	0	0,044
1,75	3651	0	0,04	0	0	0,95	0	0	0	0,01	0,95	0,04	0,01	0	0,034

1,75	1694	0	0,04	0	0	0,96	0	0	0	0	0	1	0	0	0,022
1,75	1463	0	0,04	0	0,06	0,9	0	0	0	0	0,92	0,02	0	0,06	0,04
1,75	3150	0	0,04	0	0	0,96	0	0	0	0	0	1	0	0	0,016
1,75	5693	0	0,04	0	0	0,96	0	0	0	0	0,96	0,04	0	0	0,046
1,75	4983	0	0,04	0	0,02	0,93	0	0	0	0,01	0,02	0,07	0,9	0,01	0,028
1,75	7173	0	0,04	0	0,01	0,92	0	0,01	0,01	0,01	0,92	0,06	0,01	0,01	0,044
1,75	2092	0	0,04	0	0	0,96	0	0	0	0	0	0,04	0	0,96	0,012
1,75	7952	0	0,02	0	0,11	0,86	0	0,01	0	0	0,11	0,88	0	0,01	0,048
1,75	1492	0	0,04	0	0	0,95	0	0	0	0,01	0,01	0,04	0,95	0	0,026
1,75	13705	0	0,04	0	0	0,93	0,01	0,02	0	0	0,02	0,13	0,85	0	0,044
1,75	2029	0	0,04	0	0	0,96	0	0	0	0	0,96	0,04	0	0	0,036
1,75	7428	0,01	0,04	0	0	0	0	0	0,95	0	0	0,05	0,95	0	0,048
1,75	6719	0	0,04	0	0	0,93	0,03	0	0	0	0	0,04	0,96	0	0,016
1,75	2777	0	0,04	0	0	0,96	0	0	0	0	0	0,04	0	0,96	0,01
1,75	10075	0	0,04	0	0	0,88	0	0	0,08	0	0	0,12	0	0,88	0,01
1,75	17671	0	0,04	0	0,01	0,95	0	0	0	0	0,85	0,04	0,1	0,01	0,04
1,75	1108	0	0,04	0	0,01	0,92	0	0	0	0,03	0,01	0,19	0,77	0,03	0,028
1,75	9827	0	0,04	0	0,01	0,95	0	0	0	0	0,01	0,05	0	0,94	0,016
1,75	9794	0	0,04	0	0	0,95	0,01	0	0	0	0,01	0,03	0,95	0,01	0,03
1,75	5597	0	0,04	0	0	0,95	0	0	0	0,01	0	0,99	0	0,01	0,016
1,75	9566	0,03	0,02	0	0,06	0,17	0	0	0,67	0,05	0,72	0,19	0,06	0,03	0,042
1,75	8084	0,01	0	0	0,07	0,81	0,1	0,01	0	0	0,01	0,08	0	0,91	0,044
1,75	1531	0	0,04	0	0	0,94	0,01	0	0,01	0	0,03	0,95	0,01	0,01	0,036
1,75	1472	0	0,04	0	0	0,96	0	0	0	0	0,96	0,03	0	0,01	0,044
1,75	1493	0	0,04	0	0	0,96	0	0	0	0	0,45	0,54	0	0,01	0,028
1,75	5029	0	0,04	0	0,02	0,9	0,03	0,01	0	0	0,01	0,94	0,02	0,03	0,026
1,75	5860	0	0,04	0	0	0,94	0	0	0	0,02	0	0,05	0	0,95	0,008
1,75	2250	0	0,04	0,01	0	0,95	0	0	0	0	0,6	0,39	0,01	0	0,028
1,75	2818	0	0	0	0,07	0,85	0,08	0	0	0	0,05	0,02	0,04	0,89	0,046
1,75	3141	0,01	0,04	0	0	0,94	0,01	0	0	0	0,02	0,04	0,49	0,45	0,026
1,75	2719	0	0,04	0	0	0,95	0	0,01	0	0	0	0,98	0	0,02	0,036
1,75	10851	0	0,04	0	0	0,93	0,01	0,02	0	0	0	0,06	0,93	0,01	0,026
1,75	8075	0	0	0,04	0,06	0,28	0,42	0	0,19	0,01	0,01	0	0,25	0,74	0,042

1,75	12583	0	0	0	0,42	0	0,58	0	0	0	1	0	0	0	0,046
1,75	1665	0	0,04	0	0	0,96	0	0	0	0	0,45	0,06	0,22	0,27	0,024
1,75	3361	0	0	0	0,11	0,88	0	0	0	0,01	0,12	0,88	0	0	0,048
1,75	3072	0	0,04	0,01	0	0,94	0	0	0	0,01	0,01	0,97	0	0,02	0,028
1,75	1757	0	0,04	0	0,04	0,79	0,02	0	0,11	0	0,11	0,87	0,02	0	0,042
1,75	2276	0	0,04	0	0	0,96	0	0	0	0	0,06	0,03	0,91	0	0,026
1,75	9948	0	0	0	0,41	0,59	0	0	0	0	1	0	0	0	0,048
1,75	1374	0	0,04	0,02	0	0,94	0	0	0	0	0,94	0,06	0	0	0,042
1,75	1091	0	0,03	0	0,11	0,84	0	0,01	0,01	0	0,04	0,11	0,85	0	0,046
1,75	1500	0	0,04	0	0	0,94	0	0	0,02	0	0	0,32	0,68	0	0,018
1,75	5388	0	0,04	0,02	0	0,94	0	0	0	0	0	0,03	0,94	0,03	0,038
1,75	2111	0	0,04	0	0	0,96	0	0	0	0	0,01	0,03	0	0,96	0,01
1,75	10172	0	0	0	0,2	0,62	0,16	0	0,02	0	0,61	0,02	0,37	0	0,042
1,75	3445	0,03	0,04	0	0	0,93	0	0	0	0	0,02	0,04	0,01	0,93	0,036
1,75	2129	0	0,04	0	0	0,95	0	0	0,01	0	0	0,03	0,79	0,18	0,028
1,75	9927	0	0,04	0	0	0,96	0	0	0	0	0	0,04	0,77	0,19	0,032
1,75	4534	0	0,04	0	0	0,96	0	0	0	0	0	0,04	0	0,96	0,01
1,75	4894	0	0,04	0	0	0,93	0,01	0	0	0,02	0,95	0,05	0	0	0,032
1,75	3399	0	0,04	0	0	0,96	0	0	0	0	0	0,04	0	0,96	0,01
1,75	2940	0	0	0	0,31	0,69	0	0	0	0	0,99	0,01	0	0	0,042
1,75	5200	0	0,04	0	0,01	0,93	0	0	0,02	0	0,89	0,05	0,06	0	0,032
1,75	7740	0	0,04	0	0	0,95	0	0	0	0,01	0,95	0,04	0,01	0	0,038
1,75	2287	0	0,04	0,01	0	0,94	0	0	0	0,01	0,14	0,86	0	0	0,026
1,75	1534	0	0,04	0	0,01	0,95	0	0	0	0	0	0,05	0,95	0	0,026
1,75	1999	0	0,04	0,03	0	0,93	0	0	0	0	0,94	0,04	0	0,02	0,044
1,75	11952	0	0,04	0	0	0,96	0	0	0	0	0,01	0,94	0	0,05	0,018
1,75	3419	0	0,04	0	0	0,96	0	0	0	0	0	0,04	0	0,96	0,02
1,75	2209	0	0,03	0	0,11	0,82	0	0,04	0	0	0,21	0,07	0,72	0	0,048
1,75	9441	0	0,04	0	0	0,94	0	0,02	0	0	0	0,04	0,96	0	0,03
1,75	5721	0	0,04	0	0	0,95	0,01	0	0	0	0	0,05	0	0,95	0,014
1,75	1508	0	0,04	0	0	0,94	0,02	0	0	0	0,01	0,03	0,92	0,04	0,024
1,75	3519	0	0,04	0	0	0,95	0	0	0,01	0	0,01	0,04	0	0,95	0,006
1,75	1567	0	0,04	0	0	0,94	0	0,02	0	0	0	0,04	0,94	0,02	0,044

1,75	1734	0	0,04	0	0	0,96	0	0	0	0	0,96	0,04	0	0	0,028
1,75	4124	0	0,04	0	0	0,9	0,06	0	0	0	0,9	0,07	0	0,03	0,042
1,75	11941	0	0	0	0,22	0,76	0	0	0	0,02	0,75	0	0,22	0,03	0,038
1,75	2104	0	0,04	0	0,01	0,95	0	0	0	0	0	1	0	0	0,036
1,75	1742	0	0,04	0	0	0,95	0	0,01	0	0	0	0,04	0,96	0	0,032
1,75	957	0	0,04	0	0	0,93	0	0	0,03	0	0,03	0,74	0,23	0	0,024
1,75	1673	0,01	0,04	0	0	0,95	0	0	0	0	0	0,05	0,94	0,01	0,048
1,75	2684	0	0,04	0	0,01	0,95	0	0	0	0	0	0,04	0	0,96	0,016
1,75	2308	0,01	0,04	0	0	0,87	0,07	0	0	0,01	0,66	0,04	0	0,3	0,036
1,75	7639	0	0,04	0	0	0,91	0	0	0,05	0	0	0,04	0,05	0,91	0,01
1,75	1456	0	0,04	0	0	0,96	0	0	0	0	0	0,04	0,96	0	0,022
1,75	5815	0,01	0,04	0	0,03	0,84	0,06	0	0,02	0	0,87	0,02	0,04	0,07	0,046
1,75	6623	0	0,04	0	0,02	0,94	0	0	0	0	0,94	0,05	0	0,01	0,044
1,75	1510	0	0,04	0	0	0,96	0	0	0	0	0,02	0,02	0,64	0,32	0,016
2	6484	0	0,04	0	0	0,92	0	0,04	0	0	0,02	0,04	0,92	0,02	0,044
2	4055	0	0,04	0,01	0,02	0,91	0	0	0,02	0	0,94	0,04	0,02	0	0,042
2	1059	0	0,04	0	0,14	0,82	0	0	0	0	0,82	0,18	0	0	0,042
2	2705	0	0,04	0	0	0,79	0,01	0	0,16	0	0,1	0,05	0	0,85	0,008
2	4260	0	0,04	0	0	0,96	0	0	0	0	0	0,04	0	0,96	0,006
2	16424	0	0,01	0	0,14	0,85	0	0	0	0	0,15	0,85	0	0	0,048
2	9082	0,01	0,04	0	0	0,93	0	0	0	0,02	0	0,05	0,71	0,24	0,04
2	3582	0	0,04	0	0,03	0,93	0	0	0	0	0,92	0,05	0	0,03	0,032
2	2623	0	0,04	0	0	0,96	0	0	0	0	0	0,04	0,14	0,82	0,012
2	3405	0	0,04	0	0	0,92	0	0	0	0,04	0,92	0,08	0	0	0,034
2	3735	0	0,04	0	0	0,96	0	0	0	0	0,01	0,98	0,01	0	0,026
2	1321	0,01	0,04	0	0	0,93	0,02	0	0	0	0,02	0,04	0,94	0	0,032
2	10807	0	0,04	0	0	0,96	0	0	0	0	0,96	0,04	0	0	0,04
2	28480	0	0,04	0	0	0,96	0	0	0	0	0	0,03	0	0,97	0,012
2	10070	0	0,04	0,01	0	0,95	0	0	0	0	0	0,98	0,02	0	0,02
2	4136	0	0,04	0	0	0,96	0	0	0	0	0	0,04	0,91	0,05	0,024
2	1218	0	0,04	0	0	0,94	0,02	0	0	0	0,79	0,04	0,15	0,02	0,034
2	7243	0,02	0,04	0	0	0,93	0,01	0	0	0	0	0,06	0,93	0,01	0,032
2	13946	0	0	0	0,08	0,85	0,07	0	0	0	0,07	0	0,08	0,85	0,048

2	2392	0	0,03	0	0,05	0,92	0	0	0	0	0,92	0,03	0	0,05	0,04
2	4040	0	0,04	0	0	0,93	0	0	0,02	0,01	0,02	0,02	0,01	0,95	0,01
2	1702	0	0,04	0	0,05	0,91	0	0	0	0	0,91	0,04	0	0,05	0,046
2	2502	0	0,04	0	0	0,95	0	0	0,01	0	0,95	0,04	0	0,01	0,034
2	4419	0	0,04	0	0,01	0,95	0	0	0	0	0	0,05	0,95	0	0,016
2	2372	0	0,04	0	0	0,95	0	0	0,01	0	0	0,99	0,01	0	0,036
2	4629	0	0,04	0,01	0	0,95	0	0	0	0	0,01	0,03	0	0,96	0,008
2	2743	0	0,04	0	0	0,96	0	0	0	0	0	0,04	0,96	0	0,016
2	2348	0	0,04	0	0	0,96	0	0	0	0	0,01	0,03	0,96	0	0,012
2	2928	0	0,04	0	0,01	0,95	0	0	0	0	0,01	0,04	0,11	0,84	0,022
2	5897	0	0,04	0	0	0,96	0	0	0	0	0	0	0,04	0,96	0,008
2	1529	0,01	0,04	0	0,01	0,92	0	0	0	0,02	0,04	0,04	0,92	0	0,046
2	5200	0	0,04	0	0	0,86	0	0	0,1	0	0	0,14	0,86	0	0,016
2	3447	0	0,04	0,01	0	0,94	0	0	0,01	0	0,38	0,03	0,01	0,58	0,032
2	1345	0,03	0,02	0	0	0,83	0,01	0,02	0,09	0	0,71	0,2	0,07	0,02	0,046
2	8423	0	0,04	0	0	0,96	0	0	0	0	0	0,04	0	0,96	0,01
2	5443	0	0,04	0	0,02	0,94	0	0	0	0	0,94	0,05	0	0,01	0,04
2	1464	0	0,04	0	0	0,95	0	0	0,01	0	0,01	0,47	0	0,52	0,006
2	2175	0	0,04	0	0	0,96	0	0	0	0	0	0,04	0	0,96	0,01
2	5920	0	0,04	0	0	0,89	0,05	0	0,02	0	0,89	0,09	0,02	0	0,042
2	1175	0	0,04	0	0	0,87	0,01	0,02	0,06	0	0,88	0,05	0,03	0,04	0,044
2	2913	0	0,04	0	0	0,96	0	0	0	0	0	1	0	0	0,024
2	5252	0	0,04	0	0	0,96	0	0	0	0	0	0,85	0,15	0	0,022
2	1766	0	0,04	0	0	0,96	0	0	0	0	0,03	0,01	0,26	0,7	0,014
2	3287	0	0,04	0	0,02	0,93	0	0	0	0,01	0	0,04	0	0,96	0,022
2	2829	0,01	0,04	0	0,01	0,91	0,03	0	0	0	0	0,12	0,88	0	0,038
2	14944	0	0,04	0,01	0	0,95	0	0	0	0	0	0,97	0	0,03	0,026
2	9144	0	0,04	0	0	0,96	0	0	0	0	0	0,04	0	0,96	0,006
2	6169	0	0,04	0	0,06	0,89	0	0	0,01	0	0,89	0,02	0,03	0,06	0,04
2	13572	0	0,04	0	0	0,95	0	0,01	0	0	0	0,04	0,01	0,95	0,018
2	1534	0,02	0,04	0	0	0,93	0	0	0,01	0	0,93	0,05	0	0,02	0,046
2	2215	0	0,04	0	0,01	0,95	0	0	0	0	0	0,05	0,95	0	0,032
2	11301	0	0,04	0	0	0,94	0	0	0,01	0,01	0,01	0,04	0	0,95	0,014

2	2063	0	0,04	0	0	0,96	0	0	0	0	0	1	0	0	0,04
2	14681	0	0,04	0	0	0,95	0,01	0	0	0	0,04	0,01	0	0,95	0,012
2	3682	0	0,04	0	0	0,94	0	0	0,01	0,01	0,01	0,04	0	0,95	0,008
2	4217	0	0,04	0	0	0,96	0	0	0	0	0	0	1	0	0,03
2	2204	0	0,04	0	0	0,96	0	0	0	0	0,96	0,04	0	0	0,036
2	6874	0	0,04	0	0	0,96	0	0	0	0	0	0,64	0,36	0	0,016
2	1910	0	0,04	0	0	0,95	0	0,01	0	0	0,01	0,04	0,95	0	0,032
2	1506	0	0,04	0	0	0,96	0	0	0	0	0,96	0,03	0	0,01	0,04
2	1756	0	0,04	0	0	0,96	0	0	0	0	0	0,14	0,86	0	0,032
2	2664	0	0,04	0,01	0	0,95	0	0	0	0	0,01	0,72	0,27	0	0,042
2	1708	0	0,04	0	0	0,91	0,05	0	0	0	0,02	0,04	0,9	0,04	0,03
2	1392	0	0,04	0	0	0,96	0	0	0	0	0	0,04	0	0,96	0,01
2	1507	0	0,03	0	0	0,96	0	0	0,01	0	0,97	0,03	0	0	0,036
2	1685	0	0,04	0	0	0,92	0,01	0,01	0,02	0	0,01	0,97	0	0,02	0,026
2	3880	0	0,04	0	0	0,96	0	0	0	0	0	1	0	0	0,03
2	4555	0	0,04	0	0	0,96	0	0	0	0	0	0,04	0,96	0	0,018
2	12507	0	0,04	0	0	0,96	0	0	0	0	0	0,02	0	0,98	0,002
2	1838	0	0,04	0	0,01	0,95	0	0	0	0	0	0,05	0,31	0,64	0,012
2	3353	0	0,04	0	0,01	0,94	0	0	0,01	0	0	0,04	0,95	0,01	0,038
2	4582	0	0,04	0	0	0,96	0	0	0	0	0,02	0,04	0,94	0	0,028
2	2004	0	0,04	0	0	0,96	0	0	0	0	0,97	0,01	0	0,02	0,03
2	4706	0	0,04	0	0	0,91	0,02	0	0,01	0,02	0,02	0,94	0,01	0,03	0,016
2	9113	0	0,04	0	0	0,96	0	0	0	0	0	0,08	0	0,92	0,018
2	1749	0	0,04	0	0	0,94	0	0	0	0,02	0	0,8	0,02	0,18	0,014
2	1442	0	0,04	0	0	0,9	0,06	0	0	0	0,5	0,04	0,46	0	0,036
2	3400	0	0,04	0	0	0,96	0	0	0	0	0	1	0	0	0,03
2	2796	0	0,04	0	0	0,96	0	0	0	0	0	0,04	0	0,96	0,002
2	13551	0	0,04	0	0,03	0,93	0	0	0	0	0,01	0,06	0,93	0	0,034
2	2113	0	0,04	0	0	0,96	0	0	0	0	0	1	0	0	0,026
2	5564	0	0,04	0	0	0,95	0	0,01	0	0	0	0,02	0,98	0	0,026
2	2000	0	0,04	0	0	0,95	0	0	0,01	0	0	0,04	0,01	0,95	0,01
2	1131	0	0,04	0	0	0,9	0	0,01	0,05	0	0,06	0,67	0,27	0	0,018
2	1039	0	0,04	0	0	0,96	0	0	0	0	0	0,19	0,81	0	0,024

2	8132	0	0,04	0	0	0,93	0	0	0,02	0,01	0	0,06	0,87	0,07	0,018
2	16870	0	0	0	0,05	0,9	0	0,02	0	0,03	0,06	0,01	0	0,93	0,044
2	1105	0	0,04	0	0	0,94	0	0	0,02	0	0	0,06	0	0,94	0,008
2	5838	0	0,04	0	0	0,96	0	0	0	0	0	0,04	0,96	0	0,034
2	2280	0	0,04	0	0	0,95	0,01	0	0	0	0,01	0,04	0	0,95	0,016
2	7174	0	0,04	0	0	0,96	0	0	0	0	0,92	0,04	0,04	0	0,026
2	3660	0	0,04	0	0	0,93	0,02	0	0,01	0	0,93	0,04	0,03	0	0,042
2	3025	0	0,04	0	0	0,96	0	0	0	0	0,96	0,04	0	0	0,044
2	1460	0	0,04	0	0,02	0,94	0	0	0	0	0	0,04	0,85	0,11	0,022
2	12866	0	0,04	0	0,01	0,93	0,02	0	0	0	0,03	0,04	0,93	0	0,028
2	5126	0	0,03	0	0,16	0,79	0	0,01	0,01	0	0,86	0,04	0,1	0	0,036
2	2999	0	0,04	0	0	0,96	0	0	0	0	0	0,08	0,04	0,88	0,01
2	1522	0	0,04	0	0	0,94	0	0,02	0	0	0,24	0,04	0,66	0,06	0,044
2	1807	0	0,04	0	0	0,96	0	0	0	0	0	0,04	0,96	0	0,026
2	1281	0	0,04	0	0	0,96	0	0	0	0	0	0,05	0	0,95	0,012
2,25	7850	0	0,04	0	0	0,95	0	0	0	0,01	0,01	0,04	0,95	0	0,022
2,25	2654	0	0,04	0	0	0,96	0	0	0	0	0,32	0,18	0,5	0	0,026
2,25	10622	0	0,04	0	0,01	0,95	0	0	0	0	0,01	0,04	0,95	0	0,02
2,25	5949	0	0,04	0	0	0,96	0	0	0	0	0	0,04	0	0,96	0,004
2,25	33034	0	0,04	0	0,03	0,9	0	0	0,02	0,01	0,01	0,95	0,02	0,02	0,046
2,25	2750	0	0,04	0,01	0	0,92	0	0	0	0,03	0,77	0,06	0,03	0,14	0,042
2,25	2035	0	0,04	0	0,08	0,88	0	0	0	0	0,88	0,05	0,07	0	0,048
2,25	5267	0	0,04	0	0	0,91	0,05	0	0	0	0,91	0,09	0	0	0,034
2,25	14084	0,02	0,04	0	0	0,94	0	0	0	0	0,02	0,98	0	0	0,042
2,25	4340	0	0,04	0,02	0,01	0,93	0	0	0	0	0	0,05	0,95	0	0,03
2,25	1889	0	0,04	0	0	0,95	0	0	0	0,01	0	0,05	0,95	0	0,022
2,25	8760	0	0,04	0	0	0,94	0	0,01	0	0,01	0,01	0,04	0,01	0,94	0,012
2,25	10467	0	0,04	0	0	0,95	0	0	0	0,01	0,01	0	0,31	0,68	0,01
2,25	16067	0	0,04	0,01	0	0,95	0	0	0	0	0	0,04	0,01	0,95	0,022
2,25	2222	0	0,04	0,01	0,04	0,88	0	0	0,03	0	0,01	0,07	0,92	0	0,032
2,25	11040	0	0,04	0	0	0,95	0	0,01	0	0	0	0,92	0,08	0	0,03
2,25	14357	0	0	0	0,32	0,66	0	0	0,02	0	0,66	0,32	0,02	0	0,046
2,25	4258	0	0,04	0	0	0,96	0	0	0	0	0,01	0	0,04	0,95	0,008

2,25	5936	0	0,04	0	0	0,94	0	0	0,02	0	0	0,04	0,94	0,02	0,026
2,25	1759	0	0,04	0	0	0,96	0	0	0	0	0,01	0,04	0,95	0	0,028
2,25	3557	0,01	0,04	0	0	0,95	0	0	0	0	0	0,99	0	0,01	0,036
2,25	5362	0	0,04	0	0	0,96	0	0	0	0	0	0,95	0,05	0	0,022
2,25	7171	0	0,04	0	0	0,87	0	0	0,06	0,03	0,43	0,05	0,06	0,46	0,034
2,25	1468	0	0,04	0	0	0,95	0	0,01	0	0	0	0,04	0,96	0	0,026
2,25	12142	0	0,04	0	0,04	0,91	0,01	0	0	0	0	0,94	0,06	0	0,04
2,25	1533	0,01	0,04	0	0	0,94	0	0	0,01	0	0	0,98	0,01	0,01	0,042
2,25	2376	0	0,04	0	0	0,96	0	0	0	0	0	0,06	0	0,94	0,008
2,25	4060	0	0,04	0,01	0,01	0,93	0	0,01	0	0	0	0,05	0,02	0,93	0,032
2,25	4160	0	0,04	0,01	0	0,92	0	0	0,02	0,01	0,03	0,04	0	0,93	0,006
2,25	2315	0	0,04	0	0	0,96	0	0	0	0	0	0,04	0,07	0,89	0,008
2,25	9717	0	0,04	0	0	0,95	0	0	0,01	0	0	0,05	0,01	0,94	0,008
2,25	1473	0	0,04	0	0	0,96	0	0	0	0	0	0,04	0,44	0,52	0,018
2,25	3745	0	0,04	0	0,16	0,8	0	0	0	0	0,8	0,04	0,16	0	0,048
2,25	1362	0	0,04	0	0	0,96	0	0	0	0	0	0,04	0,96	0	0,022
2,25	2501	0	0,04	0	0	0,93	0,03	0	0	0	0,01	0,04	0,78	0,17	0,024
2,25	7184	0,01	0,04	0	0,02	0,93	0	0	0	0	0,05	0,06	0,89	0	0,03
2,25	5873	0	0,04	0	0,01	0,92	0,03	0	0	0	0,03	0,04	0,92	0,01	0,034
2,25	16554	0	0	0	0,11	0,89	0	0	0	0	0,11	0	0,75	0,14	0,042
2,25	7200	0	0,04	0	0	0,96	0	0	0	0	0	1	0	0	0,024
2,25	6314	0	0,04	0	0	0,95	0	0	0,01	0	0	1	0	0	0,024
2,25	2398	0	0,04	0	0	0,94	0	0,02	0	0	0,01	0,98	0,01	0	0,036
2,25	8120	0	0	0	0,13	0,83	0	0,01	0,02	0,01	0,1	0,87	0,03	0	0,048
2,25	2250	0	0,04	0	0	0,96	0	0	0	0	0,03	0,01	0	0,96	0,018
2,25	9761	0,02	0,04	0,01	0	0,93	0	0	0	0	0,64	0,02	0,31	0,03	0,034
2,25	2788	0	0,04	0	0	0,96	0	0	0	0	0	0,17	0,83	0	0,028
2,25	7585	0	0,04	0	0,01	0,94	0,01	0	0	0	0,04	0,96	0	0	0,042
2,25	2452	0	0,04	0	0	0,93	0	0	0,02	0,01	0	0,04	0,02	0,94	0,006
2,25	5700	0	0,04	0	0	0,96	0	0	0	0	0	0,04	0	0,96	0,01
2,25	6105	0	0,04	0	0	0,96	0	0	0	0	0	1	0	0	0,024
2,25	16173	0	0,04	0	0,01	0,95	0	0	0	0	0	0,89	0,1	0,01	0,026
2,25	1951	0	0,04	0	0,01	0,95	0	0	0	0	0,96	0,04	0	0	0,046

2,25	3057	0,05	0,03	0	0	0,91	0	0	0	0,01	0,02	0,94	0	0,04	0,048
2,25	3074	0	0,03	0	0,08	0,89	0	0	0	0	0	0,92	0,08	0	0,042
2,25	4512	0	0,04	0	0,09	0,87	0	0	0	0	0,87	0,04	0,09	0	0,04
2,25	2439	0	0,04	0	0	0,96	0	0	0	0	0,01	0,04	0	0,95	0,006
2,25	7469	0	0,04	0	0	0,96	0	0	0	0	0	0,04	0	0,96	0,014
2,25	2511	0	0,04	0	0	0,94	0,01	0	0	0,01	0,01	0,05	0,94	0	0,024
2,25	1235	0	0,04	0	0	0,92	0	0	0,04	0	0,96	0,04	0	0	0,044
2,25	8702	0	0,04	0	0	0,94	0	0	0,02	0	0	0	1	0	0,026
2,25	3083	0	0,04	0	0	0,95	0	0	0	0,01	0,01	0,02	0,95	0,02	0,038
2,25	4154	0	0,04	0	0,01	0,95	0	0	0	0	0,13	0,04	0,83	0	0,032
2,25	13539	0	0,04	0	0	0,96	0	0	0	0	0	0,03	0	0,97	0,004
2,25	1413	0	0,04	0	0	0,95	0,01	0	0	0	0,01	0,04	0	0,95	0,018
2,25	6182	0	0,04	0	0	0,95	0,01	0	0	0	0	0,04	0,01	0,95	0,006
2,25	1509	0	0,04	0	0,02	0,94	0	0	0	0	0,02	0,04	0,92	0,02	0,042
2,25	1848	0	0,04	0	0	0,88	0,08	0	0	0	0	0,04	0,96	0	0,014
2,25	1493	0	0,03	0	0,1	0,84	0	0	0,03	0	0,1	0,06	0,84	0	0,046
2,25	9327	0	0,04	0	0	0,96	0	0	0	0	0	0,74	0	0,26	0,02
2,25	2404	0	0,04	0	0,08	0,88	0	0	0	0	0,75	0,04	0,21	0	0,044
2,25	2133	0	0,04	0,01	0	0,95	0	0	0	0	0	0,04	0,01	0,95	0,016
2,25	5300	0	0,04	0	0,04	0,92	0	0	0	0	0,92	0,04	0,04	0	0,048
2,25	3085	0	0,04	0	0,01	0,93	0	0	0	0,02	0,01	0,04	0,95	0	0,04
2,25	26080	0	0,04	0	0,01	0,89	0,05	0	0,01	0	0	0,09	0,91	0	0,034
2,25	3518	0	0,04	0,01	0	0,92	0,03	0	0	0	0,03	0,96	0	0,01	0,03
2,25	6417	0	0,04	0	0	0,96	0	0	0	0	0	0,04	0	0,96	0,012
2,25	7684	0	0,04	0	0	0,96	0	0	0	0	0	0,04	0	0,96	0,01
2,25	3793	0	0,04	0	0	0,95	0,01	0	0	0	0,01	0,03	0,96	0	0,038
2,25	2162	0	0,04	0	0	0,94	0,02	0	0	0	0	0,7	0,3	0	0,034
2,25	4149	0	0,04	0	0	0,65	0,29	0	0,02	0	0,8	0,04	0,14	0,02	0,032
2,25	7261	0	0,04	0	0,01	0,95	0	0	0	0	0,15	0,04	0,8	0,01	0,016
2,25	5404	0,01	0,04	0	0	0,95	0	0	0	0	0,01	0,04	0	0,95	0,016
2,25	12331	0	0,04	0	0	0,94	0,02	0	0	0	0,12	0,83	0,03	0,02	0,014
2,25	2478	0	0,04	0	0	0,96	0	0	0	0	0,96	0,04	0	0	0,048
2,25	2541	0,01	0,04	0	0,01	0,94	0	0	0	0	0,01	0,04	0	0,95	0,034

2,25	2118	0	0,04	0	0	0,95	0	0	0	0,01	0,01	0,04	0	0,95	0,004
2,25	1234	0	0,04	0	0	0,93	0	0,02	0	0,01	0,89	0,05	0,02	0,04	0,04
2,25	8080	0	0,04	0	0	0,94	0	0	0,02	0	0	0,98	0,02	0	0,02
2,25	8046	0	0	0	0,14	0,86	0	0	0	0	0	0	1	0	0,044
2,25	7639	0	0,04	0	0,01	0,93	0	0	0,02	0	0	0,11	0	0,89	0,016
2,25	10992	0	0,04	0	0	0,96	0	0	0	0	0	1	0	0	0,02
2,25	5226	0	0,04	0	0	0,96	0	0	0	0	0,02	0,04	0	0,94	0,014
2,25	3964	0	0,04	0	0	0,95	0	0	0	0,01	0	0,74	0	0,26	0,024
2,25	1826	0	0,04	0,01	0	0,94	0,01	0	0	0	0	0,04	0,25	0,71	0,012
2,25	1677	0	0,04	0	0,01	0,78	0,16	0	0	0,01	0,95	0,05	0	0	0,038
2,25	5112	0	0,04	0	0,01	0,94	0	0	0,01	0	0,54	0,04	0,02	0,4	0,038
2,25	3683	0	0,04	0	0	0,93	0	0	0,03	0	0,95	0,02	0,03	0	0,042
2,25	2151	0	0,04	0	0	0,96	0	0	0	0	0	0,04	0	0,96	0,016
2,25	2523	0	0,04	0	0,09	0,87	0	0	0	0	0,65	0,05	0,22	0,08	0,046
2,25	5663	0,01	0,04	0	0	0,94	0	0	0	0,01	0	0,99	0	0,01	0,036
2,25	2040	0	0,04	0	0	0,95	0	0	0,01	0	0,02	0,98	0	0	0,026
2,5	9631	0	0,04	0	0	0,96	0	0	0	0	0	0,04	0	0,96	0,01
2,5	16701	0	0,04	0	0	0,96	0	0	0	0	0,01	0,04	0	0,95	0,016
2,5	6023	0	0,04	0	0	0,94	0	0	0,02	0	0	0,04	0	0,96	0,006
2,5	3078	0	0,04	0	0	0,94	0,01	0,01	0	0	0,01	0,98	0	0,01	0,018
2,5	2225	0	0,04	0	0,01	0,95	0	0	0	0	0	0,81	0,18	0,01	0,042
2,5	10900	0	0,04	0	0	0,96	0	0	0	0	0,02	0,04	0,94	0	0,028
2,5	8360	0	0,04	0	0	0,92	0,03	0	0	0,01	0,92	0,05	0,03	0	0,038
2,5	2482	0	0	0	0,28	0,63	0	0	0,09	0	0,89	0,11	0	0	0,036
2,5	11624	0	0,04	0	0	0,94	0,02	0	0	0	0,94	0,06	0	0	0,036
2,5	2967	0	0,04	0	0	0,96	0	0	0	0	0	0,04	0	0,96	0,004
2,5	4236	0	0,04	0	0	0,96	0	0	0	0	0	0,04	0	0,96	0,01
2,5	26396	0	0,04	0	0,02	0,9	0,02	0,02	0	0	0,06	0,02	0,02	0,9	0,032
2,5	4091	0	0,04	0	0,01	0,95	0	0	0	0	0,01	0,04	0,22	0,73	0,022
2,5	6300	0	0,04	0	0	0,96	0	0	0	0	0	0,04	0,96	0	0,026
2,5	2727	0	0,04	0,01	0	0,94	0,01	0	0	0	0,22	0,05	0	0,73	0,014
2,5	2708	0	0,04	0	0,01	0,95	0	0	0	0	0,01	0,99	0	0	0,024
2,5	8896	0	0,04	0,01	0	0,95	0	0	0	0	0	0,04	0	0,96	0,008

2,5	48425	0	0,04	0	0	0,96	0	0	0	0	0,95	0,04	0	0,01	0,036
2,5	2279	0	0,04	0	0	0,96	0	0	0	0	0	0,04	0	0,96	0,02
2,5	2063	0	0,04	0,01	0,01	0,93	0,01	0	0	0	0,01	0,04	0,01	0,94	0,012
2,5	20505	0	0,04	0	0,03	0,93	0	0	0	0	0,04	0,91	0	0,05	0,032
2,5	4782	0,01	0,04	0	0,18	0,69	0,08	0	0	0	0,95	0,04	0	0,01	0,048
2,5	8794	0	0,04	0	0,03	0,77	0,15	0	0,01	0	0,04	0,03	0,92	0,01	0,044
2,5	7966	0	0,04	0	0	0,96	0	0	0	0	0	0,04	0	0,96	0,01
2,5	8634	0	0,04	0	0	0,96	0	0	0	0	0	0,04	0	0,96	0,018
2,5	10353	0,01	0,04	0	0	0,94	0	0	0,01	0	0,01	0,04	0	0,95	0,02
2,5	9981	0	0,04	0	0	0,96	0	0	0	0	0	0,04	0,96	0	0,024
2,5	1794	0	0,04	0,01	0,02	0,92	0	0,01	0	0	0,01	0,96	0,02	0,01	0,024
2,5	16320	0	0,04	0	0	0,96	0	0	0	0	0,96	0	0	0,04	0,04
2,5	10610	0	0,04	0	0	0,96	0	0	0	0	0	0,04	0,95	0,01	0,018
2,5	25039	0	0,01	0	0,15	0,03	0,6	0	0,21	0	0,6	0,01	0,18	0,21	0,048
2,5	3166	0,01	0,04	0	0	0,95	0	0	0	0	0	0,05	0	0,95	0,02
2,5	23108	0	0,04	0,03	0	0,89	0,04	0	0	0	0,92	0,08	0	0	0,048
2,5	2217	0	0,04	0	0	0,94	0	0	0	0,02	0	0,04	0	0,96	0,012
2,5	15102	0	0,04	0	0	0,96	0	0	0	0	0	0,8	0,2	0	0,018
2,5	1850	0	0,04	0	0	0,95	0	0	0,01	0	0	0,97	0	0,03	0,022
2,5	18195	0	0,04	0	0	0,92	0,01	0	0,03	0	0,03	0,04	0,03	0,9	0,012
2,5	2224	0	0,04	0	0,01	0,95	0	0	0	0	0	0,04	0,01	0,95	0,014
2,5	7216	0	0,04	0	0	0,96	0	0	0	0	0	0,04	0,07	0,89	0,008
2,5	5868	0	0,01	0	0,13	0,86	0	0	0	0	0,07	0,02	0,91	0	0,048
2,5	1700	0,01	0,04	0	0	0,95	0	0	0	0	0	0,99	0	0,01	0,044
2,5	2616	0	0,04	0	0,01	0,92	0	0	0,03	0	0	0,04	0,96	0	0,034
2,5	4681	0	0	0	0,19	0,8	0	0	0	0,01	0,81	0	0,19	0	0,044
2,5	14330	0	0,04	0	0	0,96	0	0	0	0	0	0,04	0	0,96	0,024
2,5	5610	0	0,04	0	0	0,95	0	0	0,01	0	0	0,07	0,92	0,01	0,02
2,5	8258	0	0,04	0	0,04	0,88	0,04	0	0	0	0,92	0,03	0,03	0,02	0,04
2,5	2043	0	0,04	0	0	0,94	0	0	0	0,02	0	0,03	0	0,97	0,014
2,5	1963	0	0,04	0	0	0,95	0,01	0	0	0	0	0,9	0,01	0,09	0,032
2,5	13796	0	0,04	0	0	0,93	0	0	0,03	0	0,01	0,05	0	0,94	0,004
2,5	7741	0	0,04	0	0	0,96	0	0	0	0	0	0,04	0	0,96	0,004

2,5	5404	0	0,04	0	0,02	0,93	0	0	0,01	0	0,95	0	0	0,05	0,044
2,5	8894	0	0,04	0	0	0,96	0	0	0	0	0	1	0	0	0,024
2,5	19481	0	0,04	0	0	0,86	0,04	0	0,06	0	0	0,41	0,59	0	0,022
2,5	1906	0	0,04	0	0	0,96	0	0	0	0	0	0,17	0,83	0	0,022
2,5	40701	0,01	0,04	0	0	0,91	0,04	0	0	0	0	0,94	0,04	0,02	0,04
2,5	2375	0	0,04	0	0	0,96	0	0	0	0	0,38	0,04	0	0,58	0,032
2,5	5121	0	0,04	0	0	0,96	0	0	0	0	0	1	0	0	0,024
2,5	17802	0	0,04	0,02	0	0,91	0,03	0	0	0	0,8	0,05	0,13	0,02	0,048
2,5	1916	0	0,04	0	0	0,96	0	0	0	0	0	1	0	0	0,03
2,5	3331	0	0,04	0	0	0,95	0	0	0,01	0	0,01	0,99	0	0	0,03
2,5	6581	0	0,04	0	0	0,94	0	0	0,02	0	0,22	0,04	0,74	0	0,034
2,5	2366	0	0,04	0	0	0,95	0	0,01	0	0	0,34	0,03	0,62	0,01	0,032
2,5	5193	0,01	0,04	0,01	0	0,94	0	0	0	0	0	0,48	0,02	0,5	0,028
2,5	1485	0	0,04	0	0	0,96	0	0	0	0	0	0,04	0	0,96	0
2,5	6078	0	0,01	0	0,2	0,66	0,1	0	0	0,03	0,76	0	0,21	0,03	0,048
2,5	12170	0	0,04	0	0,02	0,93	0	0	0,01	0	0,02	0,04	0,91	0,03	0,038
2,5	1871	0	0,01	0	0,34	0,65	0	0	0	0	0,98	0,01	0	0,01	0,048
2,5	2002	0	0,04	0	0	0,96	0	0	0	0	0,76	0,04	0	0,2	0,03
2,5	4225	0	0,04	0	0	0,95	0	0	0,01	0	0,01	0,98	0,01	0	0,028
2,5	1827	0	0,04	0	0	0,9	0,05	0	0	0,01	0	0,14	0,85	0,01	0,024
2,5	2020	0	0,04	0	0	0,94	0,01	0,01	0	0	0,91	0,07	0,02	0	0,038
2,5	3016	0	0,04	0	0,03	0,93	0	0	0	0	0,02	0,98	0	0	0,034
2,5	1909	0	0,04	0	0	0,96	0	0	0	0	0,73	0,12	0,14	0,01	0,028
2,5	4399	0	0,04	0	0	0,94	0	0,02	0	0	0,02	0	0,98	0	0,036
2,5	1340	0	0,04	0	0	0,96	0	0	0	0	0,3	0,02	0,02	0,66	0,016
2,5	2464	0	0,04	0	0	0,96	0	0	0	0	0	0,04	0	0,96	0,014
2,5	9962	0	0,04	0	0	0,95	0	0	0,01	0	0	0,04	0,96	0	0,024
2,5	3714	0	0,04	0,01	0	0,93	0	0,01	0,01	0	0,94	0,02	0	0,04	0,044
2,5	2155	0	0,04	0	0	0,92	0,03	0	0	0,01	0,01	0,04	0,95	0	0,03
2,5	3066	0	0,04	0	0,01	0,91	0	0,02	0	0,02	0	0,05	0,91	0,04	0,036
2,5	2458	0	0,04	0	0	0,95	0	0	0,01	0	0,01	0,99	0	0	0,024
2,5	7165	0	0,04	0,01	0	0,95	0	0	0	0	0	0,9	0,1	0	0,038
2,5	16336	0	0	0	0,16	0,83	0	0	0,01	0	0,01	0	0,99	0	0,048

2,5	6114	0	0,04	0	0	0,96	0	0	0	0	0	0,05	0,95	0	0,03
2,5	13424	0	0,04	0	0	0,94	0,02	0	0	0	0,95	0,04	0	0,01	0,042
2,5	2311	0	0,04	0	0	0,95	0	0,01	0	0	0	0,05	0	0,95	0,026
2,5	3242	0	0,04	0	0,02	0,94	0	0	0	0	0	0,05	0,01	0,94	0,026
2,5	13098	0	0,04	0	0,07	0,89	0	0	0	0	0,89	0,04	0	0,07	0,048
2,5	14671	0	0,04	0	0	0,94	0	0	0,02	0	0	0,98	0,02	0	0,016
2,5	5899	0	0,04	0	0	0,93	0	0,03	0	0	0,87	0,13	0	0	0,042
2,5	7739	0	0,04	0	0	0,95	0	0	0	0,01	0	0,05	0	0,95	0,006
2,5	3086	0	0,04	0	0	0,96	0	0	0	0	0	1	0	0	0,026
2,5	4413	0	0,04	0	0	0,95	0	0	0	0,01	0,91	0,01	0	0,08	0,038
2,5	3179	0	0,04	0	0	0,96	0	0	0	0	0	0,07	0	0,93	0,008
2,5	1356	0	0,04	0	0,01	0,95	0	0	0	0	0	0,05	0	0,95	0,014
2,5	19299	0,01	0	0	0,3	0	0,68	0	0	0,01	0,99	0	0,01	0	0,04
2,5	5348	0	0,04	0	0	0,95	0	0	0	0,01	0	0,04	0	0,96	0,018
2,5	3153	0	0,04	0	0	0,95	0	0	0,01	0	0,01	0,44	0	0,55	0,01
2,5	4388	0	0,04	0	0	0,95	0	0	0,01	0	0	0,78	0,02	0,2	0,02
2,5	11273	0	0,04	0	0,04	0,92	0	0	0	0	0	0,04	0,96	0	0,048
2,75	10669	0	0,04	0	0,03	0,93	0	0	0	0	0	0,04	0,96	0	0,044
2,75	3323	0	0,04	0	0	0,96	0	0	0	0	0	0,74	0	0,26	0,018
2,75	1839	0	0,04	0,03	0	0,93	0	0	0	0	0,01	0,99	0	0	0,044
2,75	9576	0	0,04	0	0	0,93	0,03	0	0	0	0,93	0,04	0,03	0	0,044
2,75	6099	0	0,04	0,01	0	0,95	0	0	0	0	0,01	0,04	0	0,95	0,014
2,75	12097	0	0,04	0	0	0,96	0	0	0	0	0	0,04	0,96	0	0,024
2,75	8986	0	0	0	0,08	0,89	0	0	0	0,03	0,17	0	0,06	0,77	0,044
2,75	1857	0	0,04	0	0	0,91	0,04	0,01	0	0	0	1	0	0	0,028
2,75	11304	0,01	0,04	0	0,01	0,92	0	0	0	0,02	0,01	0,09	0,9	0	0,038
2,75	16706	0	0,04	0,01	0	0,94	0	0	0	0,01	0	0,04	0,01	0,95	0,004
2,75	3083	0	0,04	0	0	0,96	0	0	0	0	0	0,04	0	0,96	0,01
2,75	4181	0	0,04	0,01	0	0,93	0	0,02	0	0	0,67	0,06	0	0,27	0,038
2,75	9984	0,02	0,04	0	0	0,94	0	0	0	0	0	0,04	0,02	0,94	0,032
2,75	21381	0	0,04	0	0	0,96	0	0	0	0	0	0,96	0	0,04	0,022
2,75	4165	0	0,04	0	0	0,95	0	0	0	0,01	0,01	0,98	0,01	0	0,018
2,75	16206	0	0,04	0	0	0,96	0	0	0	0	0,05	0,04	0	0,91	0,014

2,75	8284	0,01	0,04	0	0	0,95	0	0	0	0	0	0,05	0	0,95	0,02
2,75	9977	0	0,04	0	0	0,96	0	0	0	0	0,86	0,04	0,1	0	0,036
2,75	10708	0	0,04	0	0	0,93	0	0,03	0	0	0,79	0,04	0,14	0,03	0,038
2,75	3082	0	0,04	0	0,07	0,87	0	0	0	0,02	0,25	0,68	0,07	0	0,042
2,75	1468	0	0,04	0	0	0,95	0,01	0	0	0	0,55	0,44	0,01	0	0,034
2,75	1945	0	0,04	0,01	0	0,95	0	0	0	0	0,01	0,99	0	0	0,032
2,75	10007	0,01	0,04	0	0,01	0,94	0	0	0	0	0,03	0,03	0	0,94	0,032
2,75	1727	0	0,04	0	0	0,94	0,02	0	0	0	0,74	0,06	0	0,2	0,026
2,75	1529	0	0,04	0	0	0,96	0	0	0	0	0	1	0	0	0,024
2,75	4593	0	0,04	0,01	0	0,95	0	0	0	0	0	0,95	0	0,05	0,03
2,75	1763	0	0,04	0	0	0,94	0	0	0	0,02	0	0,03	0,53	0,44	0,024
2,75	1792	0	0,04	0,01	0	0,95	0	0	0	0	0,01	0,04	0	0,95	0,024
2,75	8107	0	0,04	0	0	0,95	0	0	0	0,01	0	0,04	0,01	0,95	0,012
2,75	24674	0	0,04	0	0	0,96	0	0	0	0	0,94	0,06	0	0	0,03
2,75	5135	0	0,04	0	0	0,96	0	0	0	0	0,88	0,02	0	0,1	0,024
2,75	5868	0	0,04	0	0	0,96	0	0	0	0	0	0,75	0,25	0	0,022
2,75	10487	0	0,04	0,01	0	0,86	0,03	0	0,06	0	0,05	0,11	0,83	0,01	0,032
2,75	14831	0	0,04	0	0	0,96	0	0	0	0	0	0,05	0,94	0,01	0,024
2,75	5020	0	0,04	0	0	0,95	0,01	0	0	0	0,38	0,04	0,01	0,57	0,018
2,75	5364	0	0,04	0	0	0,96	0	0	0	0	0,86	0,04	0	0,1	0,036
2,75	6346	0	0,03	0,02	0	0,95	0	0	0	0	0,13	0,05	0,82	0	0,046
2,75	10179	0	0,04	0	0	0,96	0	0	0	0	0	0,04	0,03	0,93	0,012
2,75	2772	0	0,04	0	0	0,79	0	0	0,17	0	0,79	0,04	0	0,17	0,038
2,75	1882	0	0,04	0	0	0,96	0	0	0	0	0	0,04	0	0,96	0,01
2,75	4175	0	0,04	0	0	0,9	0,04	0,02	0	0	0,31	0,04	0,65	0	0,038
2,75	40157	0	0,04	0	0	0,94	0	0	0	0,02	0	0,01	0,97	0,02	0,024
2,75	9895	0	0,04	0,01	0,02	0,93	0	0	0	0	0	0,04	0,02	0,94	0,032
2,75	2324	0	0,04	0,01	0	0,95	0	0	0	0	0	0,04	0,96	0	0,038
2,75	1763	0	0,04	0	0	0,96	0	0	0	0	0	1	0	0	0,02
2,75	20436	0	0,04	0	0,07	0,88	0	0,01	0	0	0,6	0,08	0,32	0	0,034
2,75	12636	0	0,04	0	0	0,96	0	0	0	0	0	0,04	0,87	0,09	0,018
2,75	15494	0	0	0	0,43	0,57	0	0	0	0	1	0	0	0	0,046
2,75	9773	0	0,04	0	0	0,95	0	0	0,01	0	0	0,04	0	0,96	0,008

2,75	24236	0	0,03	0	0,11	0,77	0,08	0,01	0	0	0,78	0,11	0,11	0	0,046
2,75	7393	0	0,04	0	0,01	0,95	0	0	0	0	0	0,04	0	0,96	0,018
2,75	2871	0	0,04	0	0,01	0,95	0	0	0	0	0	0,04	0,28	0,68	0,018
2,75	8217	0	0,02	0	0,07	0,83	0	0	0,08	0	0,09	0	0,08	0,83	0,038
2,75	4617	0	0,04	0	0	0,94	0	0	0,02	0	0,02	0,04	0,94	0	0,022
2,75	16218	0	0	0	0,13	0,86	0,01	0	0	0	0,14	0,03	0,83	0	0,046
2,75	1722	0	0,04	0	0	0,96	0	0	0	0	0,06	0,92	0,02	0	0,026
2,75	1786	0	0,04	0	0,15	0,75	0,01	0	0,05	0	0,76	0,19	0,01	0,04	0,048
2,75	2737	0,01	0,04	0	0	0,95	0	0	0	0	0	0,04	0	0,96	0,026
2,75	9555	0	0	0	0,19	0,81	0	0	0	0	0,4	0,6	0	0	0,032
2,75	14642	0,01	0	0	0,1	0,89	0	0	0	0	0,01	0,89	0,1	0	0,042
2,75	1500	0	0,04	0	0,01	0,93	0,02	0	0	0	0	0,44	0,53	0,03	0,026
2,75	2900	0	0,04	0	0	0,88	0,08	0	0	0	0,96	0,04	0	0	0,042
2,75	6785	0	0,04	0	0	0,96	0	0	0	0	0	1	0	0	0,034
2,75	13282	0	0,04	0,01	0	0,92	0,02	0	0,01	0	0,87	0,05	0,06	0,02	0,036
2,75	18349	0	0,04	0	0	0,96	0	0	0	0	0,96	0,03	0	0,01	0,032
2,75	2404	0,02	0,03	0	0	0,91	0	0,04	0	0	0,75	0,07	0,02	0,16	0,046
2,75	1890	0	0,04	0	0	0,95	0	0,01	0	0	0	0,99	0	0,01	0,028
2,75	4859	0	0,04	0	0	0,93	0,02	0	0,01	0	0,34	0,29	0,34	0,03	0,028
2,75	8385	0	0,04	0	0	0,94	0,01	0	0,01	0	0	0,05	0,94	0,01	0,042
2,75	1109	0,01	0,04	0	0	0,94	0,01	0	0	0	0,23	0,05	0	0,72	0,032
2,75	23670	0	0,04	0	0	0,95	0	0,01	0	0	0	0,04	0,01	0,95	0,006
2,75	2063	0	0,04	0	0	0,94	0,01	0	0	0,01	0	0,06	0,7	0,24	0,014
2,75	1856	0	0,04	0,01	0	0,95	0	0	0	0	0,4	0,04	0,55	0,01	0,024
2,75	13900	0	0,04	0	0	0,96	0	0	0	0	0	0,04	0,96	0	0,024
2,75	2830	0,01	0,04	0	0	0,94	0	0	0	0,01	0	0,03	0,03	0,94	0,02
2,75	4006	0	0,04	0	0	0,94	0,02	0	0	0	0,06	0,04	0,9	0	0,02
2,75	6183	0	0,04	0	0	0,92	0	0,04	0	0	0	0,99	0	0,01	0,04
2,75	20578	0	0,04	0	0	0,96	0	0	0	0	0,96	0,04	0	0	0,032
2,75	2442	0	0	0	0,34	0,66	0	0	0	0	1	0	0	0	0,046
2,75	6906	0	0,04	0	0	0,96	0	0	0	0	0	0,04	0	0,96	0,014
2,75	2387	0	0,04	0	0	0,95	0,01	0	0	0	0,01	0,99	0	0	0,016
2,75	14754	0,01	0,04	0	0	0,95	0	0	0	0	0	0,05	0,95	0	0,034

2,75	20406	0	0,04	0	0	0,96	0	0	0	0	0,96	0,04	0	0	0,044
2,75	4715	0	0,04	0	0	0,94	0	0	0	0,02	0	0,04	0,95	0,01	0,026
2,75	1755	0	0,04	0	0	0,94	0,02	0	0	0	0	0,98	0	0,02	0,014
2,75	6454	0	0,04	0	0	0,95	0	0	0,01	0	0,95	0,04	0,01	0	0,038
2,75	2550	0	0,04	0	0	0,96	0	0	0	0	0	0,28	0	0,72	0,004
2,75	11611	0,01	0,04	0	0	0,94	0	0	0,01	0	0,94	0,04	0,01	0,01	0,034
2,75	4748	0	0,04	0,01	0	0,95	0	0	0	0	0,1	0,48	0,02	0,4	0,026
2,75	1806	0,01	0,04	0	0,01	0,94	0	0	0	0	0	0,01	0,98	0,01	0,044
2,75	14595	0	0,04	0	0	0,96	0	0	0	0	0	0,04	0	0,96	0,008
2,75	3236	0	0,04	0	0,01	0,95	0	0	0	0	0,03	0,02	0	0,95	0,016
2,75	3318	0	0,04	0	0,02	0,94	0	0	0	0	0,94	0,04	0,02	0	0,044
2,75	2984	0	0,04	0	0	0,96	0	0	0	0	0	0,05	0	0,95	0,008
2,75	3275	0	0,04	0	0	0	0	0	0,96	0	0,94	0,04	0	0,02	0,048
2,75	2257	0	0,04	0	0	0,89	0	0	0,06	0,01	0,01	0,49	0	0,5	0,008
2,75	2095	0	0,04	0	0	0,95	0	0	0	0,01	0	0,94	0,06	0	0,028
2,75	2280	0	0,04	0	0	0,95	0	0,01	0	0	0	0,05	0,95	0	0,032
2,75	7206	0	0,04	0,01	0	0,89	0	0	0,05	0,01	0,08	0,03	0	0,89	0,016
2,75	4859	0	0,04	0	0,02	0,93	0	0,01	0	0	0,02	0,04	0,93	0,01	0,04
3	8520	0	0,04	0	0	0,96	0	0	0	0	0	0,02	0,98	0	0,016
3	3384	0,01	0,04	0	0	0,95	0	0	0	0	0	0,05	0	0,95	0,018
3	40550	0	0,04	0	0	0,96	0	0	0	0	0	1	0	0	0,03
3	4901	0	0,04	0	0	0,96	0	0	0	0	0	0,04	0,78	0,18	0,036
3	1540	0	0,04	0,01	0,03	0,9	0	0	0	0,02	0,04	0,06	0,9	0	0,046
3	3453	0	0,04	0	0	0,96	0	0	0	0	0	0,04	0,05	0,91	0,01
3	10741	0	0,04	0,01	0	0,93	0,02	0	0	0	0,02	0,97	0,01	0	0,042
3	39815	0	0,04	0	0,01	0,95	0	0	0	0	0,01	0,94	0,01	0,04	0,03
3	1967	0,01	0,04	0	0	0,95	0	0	0	0	0	0,04	0,96	0	0,038
3	17005	0	0,04	0	0	0,96	0	0	0	0	0,96	0,04	0	0	0,034
3	1735	0	0,04	0	0	0,96	0	0	0	0	0	0,04	0,96	0	0,028
3	11638	0	0,04	0	0,01	0,95	0	0	0	0	0,95	0,04	0,01	0	0,048
3	20679	0	0,04	0	0	0,96	0	0	0	0	0	0,04	0,96	0	0,014
3	20052	0	0,04	0	0	0,94	0	0	0	0,02	0	0,06	0,93	0,01	0,016
3	2857	0	0,04	0	0	0,96	0	0	0	0	0	0,09	0	0,91	0,004

3	8822	0	0,04	0	0	0,95	0	0	0	0,01	0	0,98	0	0,02	0,022
3	19441	0	0,04	0	0	0,96	0	0	0	0	0	0,04	0,96	0	0,024
3	26371	0,01	0,04	0	0	0,95	0	0	0	0	0	0,04	0,95	0,01	0,034
3	10436	0	0,04	0	0	0,96	0	0	0	0	0	0,04	0	0,96	0,01
3	1687	0	0,04	0	0	0,95	0	0	0	0,01	0	0,42	0	0,58	0,018
3	7596	0	0,04	0	0	0,96	0	0	0	0	0	0,02	0	0,98	0,012
3	1290	0	0,04	0	0	0,96	0	0	0	0	0	1	0	0	0,018
3	23667	0	0,04	0	0	0,95	0,01	0	0	0	0,01	0,04	0,95	0	0,03
3	16425	0	0	0,01	0,11	0,87	0,01	0	0	0	0	0,87	0,13	0	0,048
3	2411	0	0,04	0	0,03	0,92	0	0	0	0,01	0,93	0,04	0,03	0	0,04
3	2393	0	0,04	0	0	0,96	0	0	0	0	0,01	0,03	0	0,96	0,01
3	3616	0	0,04	0	0	0,95	0,01	0	0	0	0	0,04	0,01	0,95	0,014
3	13080	0	0	0	0,06	0,93	0	0,01	0	0	0,06	0	0	0,94	0,036
3	2239	0	0,04	0	0	0,96	0	0	0	0	0	0,04	0,96	0	0,024
3	3108	0,01	0,04	0	0	0,9	0,05	0	0	0	0,01	0,94	0	0,05	0,04
3	7250	0	0,04	0	0	0,96	0	0	0	0	0	0,04	0,96	0	0,026
3	6939	0	0,04	0	0	0,96	0	0	0	0	0	1	0	0	0,018
3	14943	0	0,04	0	0	0,86	0,02	0	0,08	0	0,08	0,69	0,02	0,21	0,024
3	26306	0	0,04	0	0	0,96	0	0	0	0	0,09	0,04	0,87	0	0,026
3	3991	0	0,04	0	0	0,96	0	0	0	0	0,01	0,99	0	0	0,028
3	11920	0	0,04	0	0	0,96	0	0	0	0	0	0,04	0,01	0,95	0,01
3	3736	0	0,04	0	0,03	0,9	0,01	0,02	0	0	0,9	0,04	0,03	0,03	0,044
3	14736	0	0,04	0	0	0,94	0,01	0	0	0,01	0	0,04	0,01	0,95	0,004
3	20375	0	0,04	0	0	0,96	0	0	0	0	0,03	0,04	0,93	0	0,024
3	5436	0	0,04	0	0	0,86	0,01	0	0,09	0	0,01	0,95	0	0,04	0,01
3	14170	0	0,04	0	0	0,95	0	0	0,01	0	0,38	0,6	0	0,02	0,04
3	1284	0	0,04	0	0	0,93	0,03	0	0	0	0	0,04	0,91	0,05	0,03
3	8454	0	0,04	0	0	0,95	0	0	0	0,01	0	0,99	0,01	0	0,022
3	2314	0	0,04	0	0	0,92	0,02	0	0,02	0	0,02	0,06	0,92	0	0,026
3	3325	0	0,04	0	0	0,96	0	0	0	0	0	0	0,99	0,01	0,02
3	2186	0	0,04	0	0,01	0,95	0	0	0	0	0	0,04	0,01	0,95	0,016
3	16445	0	0,04	0	0	0,96	0	0	0	0	0	0,04	0	0,96	0,01
3	7448	0	0,04	0	0	0,96	0	0	0	0	0	0,04	0	0,96	0,01

3	17213	0	0,04	0	0	0,93	0,03	0	0	0	0,93	0,01	0,03	0,03	0,042
3	20296	0	0,04	0	0,03	0,91	0	0,02	0	0	0,05	0,95	0	0	0,044
3	2374	0	0,04	0	0	0,95	0	0	0,01	0	0	0,99	0	0,01	0,022
3	13169	0	0,04	0	0	0,96	0	0	0	0	0,96	0,04	0	0	0,04
3	1645	0	0,04	0	0	0,79	0,01	0	0,15	0,01	0	0,82	0,16	0,02	0,018
3	3035	0	0,04	0	0	0,96	0	0	0	0	0	0,04	0	0,96	0,01
3	13458	0	0,04	0	0	0,95	0	0,01	0	0	0	0,04	0,96	0	0,026
3	9199	0	0,04	0	0	0,95	0	0	0,01	0	0	0,04	0,95	0,01	0,034
3	2518	0	0,04	0	0	0,96	0	0	0	0	0	0,04	0	0,96	0,004
3	2744	0	0,04	0	0,01	0	0,03	0,02	0,9	0	0,9	0,07	0,03	0	0,048
3	9638	0	0,04	0	0	0,95	0	0	0	0,01	0,86	0,05	0,09	0	0,028
3	7653	0	0,04	0	0	0,96	0	0	0	0	0	0,98	0	0,02	0,02
3	11066	0	0,04	0	0	0,95	0	0	0,01	0	0,06	0,04	0,9	0	0,028
3	7219	0	0,04	0	0	0,96	0	0	0	0	0	0,04	0	0,96	0,006
3	4294	0,02	0,04	0	0	0,93	0,01	0	0	0	0,92	0,05	0	0,03	0,042
3	8247	0	0,04	0	0	0,96	0	0	0	0	0	0,04	0,96	0	0,022
3	4988	0	0,04	0	0	0,96	0	0	0	0	0	0,95	0	0,05	0,022
3	9862	0,01	0,04	0	0	0,94	0	0,01	0	0	0,01	0,94	0,03	0,02	0,044
3	4382	0	0,04	0	0,01	0,95	0	0	0	0	0	0,04	0	0,96	0,012
3	11707	0	0,04	0	0	0,92	0	0	0,04	0	0	0,07	0,93	0	0,026
3	13663	0,01	0,04	0	0	0,95	0	0	0	0	0	0,36	0,64	0	0,036
3	8178	0	0,04	0	0	0,95	0,01	0	0	0	0,01	0,04	0,95	0	0,036
3	14987	0	0,04	0	0	0,95	0	0	0,01	0	0,01	0,04	0,95	0	0,032
3	1478	0	0,04	0	0,07	0,88	0	0	0,01	0	0,89	0,04	0,07	0	0,038
3	4298	0	0,04	0	0	0,94	0	0	0,02	0	0,01	0,04	0,94	0,01	0,024
3	14473	0	0,04	0	0	0,92	0	0	0,04	0	0,02	0,06	0	0,92	0,02
3	862	0,02	0,04	0	0	0,9	0	0	0,04	0	0	0,08	0,91	0,01	0,042
3	1921	0	0,04	0	0	0,95	0	0,01	0	0	0	0,04	0	0,96	0,016
3	10463	0	0,03	0	0,11	0,86	0	0	0	0	0,11	0,77	0,03	0,09	0,044
3	2449	0	0,04	0	0	0,95	0,01	0	0	0	0,01	0,99	0	0	0,024
3	4127	0,01	0,04	0	0,01	0,94	0	0	0	0	0	1	0	0	0,044
3	5554	0	0,04	0	0	0,95	0	0	0,01	0	0,01	0,04	0	0,95	0,016
3	7226	0	0,04	0,01	0	0,94	0	0,01	0	0	0,02	0,03	0	0,95	0,03

3	6979	0	0,04	0	0	0,96	0	0	0	0	0,18	0,04	0,78	0	0,022
3	14546	0	0,04	0	0	0,95	0	0	0,01	0	0	0,7	0,3	0	0,024
3	1717	0	0,04	0	0	0,95	0,01	0	0	0	0,01	0,99	0	0	0,03
3	2660	0	0,04	0	0	0,96	0	0	0	0	0	0,04	0	0,96	0,01
3	7146	0	0,04	0	0	0,92	0	0	0,04	0	0	0,04	0,03	0,93	0,006
3	15831	0	0,04	0	0	0,96	0	0	0	0	0	0,04	0	0,96	0,01
3	8604	0	0,04	0	0	0,96	0	0	0	0	0	0,04	0,96	0	0,03
3	7100	0	0,04	0	0	0,96	0	0	0	0	0	0,04	0,73	0,23	0,028
3	6665	0	0,04	0	0	0,8	0	0	0,13	0,03	0	0,32	0,68	0	0,03
3	2151	0	0,04	0	0	0,95	0	0,01	0	0	0,01	0,04	0,95	0	0,02
3	9333	0	0,02	0	0,13	0,81	0,03	0,01	0	0	0,5	0,49	0,01	0	0,048
3	18788	0	0,04	0,01	0	0,88	0,05	0	0,02	0	0,93	0,04	0,01	0,02	0,048
3	3500	0	0,04	0	0	0,91	0,05	0	0	0	0,05	0,05	0,9	0	0,018
3	12934	0	0,04	0	0	0,94	0	0	0,02	0	0,02	0,98	0	0	0,02
3	2193	0	0,04	0	0	0,94	0,02	0	0	0	0,01	0,05	0,94	0	0,018
3	7334	0	0,04	0	0	0,95	0	0	0	0,01	0,05	0,84	0	0,11	0,028
3	2012	0	0,04	0	0,17	0,62	0	0	0,17	0	0,62	0,34	0,03	0,01	0,048
3	10919	0	0,04	0,01	0	0,93	0	0	0,02	0	0	0,07	0,93	0	0,04
3	2421	0,01	0,04	0	0	0,79	0,07	0,09	0	0	0,07	0,11	0,81	0,01	0,04
3,25	2040	0,01	0,04	0	0	0,94	0	0,01	0	0	0,07	0,13	0,01	0,79	0,03
3,25	6985	0	0,04	0	0	0,95	0	0,01	0	0	0	0,05	0,95	0	0,034
3,25	20957	0	0,04	0	0	0,96	0	0	0	0	0,01	0,03	0	0,96	0,01
3,25	12360	0	0,04	0	0	0,96	0	0	0	0	0	0,04	0	0,96	0,008
3,25	17551	0	0,04	0	0,01	0,95	0	0	0	0	0	0,06	0	0,94	0,018
3,25	6615	0	0,04	0	0	0,96	0	0	0	0	0	0	0,04	0,96	0,01
3,25	18496	0	0,04	0	0	0,95	0	0	0	0,01	0,14	0,05	0,01	0,8	0,014
3,25	5628	0	0,04	0,02	0	0,87	0,03	0,04	0	0	0	0,08	0,91	0,01	0,048
3,25	4846	0	0,04	0,04	0	0,87	0	0,01	0,04	0	0,03	0,09	0,87	0,01	0,04
3,25	6310	0	0,04	0	0,02	0,92	0,01	0	0,01	0	0,01	0,06	0,93	0	0,042
3,25	33194	0	0,04	0	0	0,96	0	0	0	0	0	1	0	0	0,02
3,25	5679	0	0,03	0	0,07	0,85	0,01	0,01	0,03	0	0,08	0,07	0,85	0	0,032
3,25	7481	0	0,04	0,01	0	0,95	0	0	0	0	0,02	0,09	0,88	0,01	0,026
3,25	8020	0	0,04	0	0,02	0,94	0	0	0	0	0	0,04	0,96	0	0,024

3,25	13070	0	0,04	0	0	0,96	0	0	0	0	0	0,99	0	0,01	0,024
3,25	13231	0	0,04	0	0	0,96	0	0	0	0	0	0,04	0,96	0	0,018
3,25	3549	0	0,04	0	0	0,94	0	0	0,02	0	0,02	0,04	0	0,94	0,008
3,25	6221	0,01	0,04	0	0	0,9	0,01	0	0,04	0	0,01	0,05	0,9	0,04	0,036
3,25	5343	0	0,04	0	0	0,96	0	0	0	0	0,96	0,04	0	0	0,038
3,25	16428	0,02	0,02	0	0,09	0,86	0	0	0,01	0	0,85	0,01	0,02	0,12	0,044
3,25	21672	0	0,04	0,01	0	0,95	0	0	0	0	0	0,96	0,04	0	0,03
3,25	25942	0	0,04	0	0	0,88	0,07	0	0,01	0	0,94	0,05	0,01	0	0,04
3,25	2963	0	0,04	0	0	0,8	0,16	0	0	0	0,16	0,07	0,77	0	0,022
3,25	16049	0,02	0,04	0	0	0,92	0	0	0	0,02	0,02	0,86	0,12	0	0,042
3,25	3259	0	0	0,01	0,3	0,48	0,2	0	0,01	0	0,79	0,2	0	0,01	0,048
3,25	1699	0,02	0,04	0	0,01	0,92	0	0	0,01	0	0,02	0,96	0,01	0,01	0,048
3,25	5685	0	0,04	0	0	0,95	0	0	0,01	0	0,57	0,02	0,39	0,02	0,044
3,25	5327	0	0,04	0	0	0,94	0,01	0	0,01	0	0,01	0,04	0,95	0	0,026
3,25	13176	0	0,04	0	0	0,94	0	0	0,02	0	0,12	0,04	0,02	0,82	0,01
3,25	8904	0	0,04	0	0	0,84	0,11	0	0	0,01	0,02	0,02	0,84	0,12	0,032
3,25	23915	0,01	0,04	0	0,01	0,93	0	0	0,01	0	0,01	0,33	0,01	0,65	0,032
3,25	10633	0	0	0	0,22	0,06	0	0	0,72	0	0,71	0,23	0,06	0	0,048
3,25	38821	0	0,04	0	0	0,84	0,06	0	0,06	0	0,86	0,04	0,1	0	0,04
3,25	5604	0	0,04	0	0	0,94	0,01	0	0	0,01	0,01	0,97	0,02	0	0,04
3,25	8832	0	0,04	0	0	0,94	0,02	0	0	0	0	0,06	0,94	0	0,028
3,25	5750	0	0,04	0	0,01	0,95	0	0	0	0	0,01	0,04	0,95	0	0,024
3,25	1383	0,01	0,04	0	0	0,95	0	0	0	0	0,73	0,05	0	0,22	0,04
3,25	10357	0,04	0,03	0	0	0,9	0,01	0	0	0,02	0,58	0,04	0,33	0,05	0,048
3,25	9260	0	0,04	0	0	0,96	0	0	0	0	0	0,04	0	0,96	0,016
3,25	2277	0	0,04	0	0	0,95	0	0	0	0,01	0,75	0,04	0,21	0	0,042
3,25	6502	0	0,04	0	0	0,96	0	0	0	0	0	0,04	0,96	0	0,014
3,25	8572	0	0,04	0,01	0	0	0,07	0	0,88	0	0	0,88	0,07	0,05	0,02
3,25	25882	0	0,04	0	0,01	0,94	0	0	0	0,01	0,01	0,98	0,01	0	0,036
3,25	52434	0	0,04	0,01	0	0,95	0	0	0	0	0	0,04	0,04	0,92	0,012
3,25	2795	0	0,04	0	0	0,92	0	0	0,02	0,02	0,92	0,06	0,02	0	0,032
3,25	13285	0	0,01	0	0,09	0,9	0	0	0	0	0,1	0	0,01	0,89	0,044
3,25	5416	0	0,04	0	0	0,96	0	0	0	0	0	0,04	0	0,96	0,008

3,25	5508	0	0	0	0,38	0	0,61	0	0,01	0	0,96	0,04	0	0	0,046
3,25	4750	0	0	0,01	0,11	0,88	0	0	0	0	0,12	0,88	0	0	0,048
3,25	1543	0	0,04	0	0	0,96	0	0	0	0	0	1	0	0	0,022
3,25	5683	0	0,04	0	0	0,96	0	0	0	0	0,01	0,03	0	0,96	0,01
3,25	6418	0	0,04	0	0,01	0,95	0	0	0	0	0	0,04	0,96	0	0,036
3,25	8631	0	0,04	0	0	0,96	0	0	0	0	0	0,04	0	0,96	0,008
3,25	8776	0	0,04	0	0	0,96	0	0	0	0	0	0,04	0,96	0	0,024
3,25	2735	0	0,04	0	0	0,96	0	0	0	0	0	0,04	0	0,96	0,01
3,25	11847	0,01	0,04	0	0,01	0,94	0	0	0	0	0,94	0,05	0,01	0	0,042
3,25	8608	0	0,04	0	0	0,96	0	0	0	0	0	1	0	0	0,026
3,25	19853	0	0,04	0	0	0,96	0	0	0	0	0	0,04	0	0,96	0,014
3,25	15748	0,01	0,04	0	0	0,93	0	0	0,01	0,01	0,01	0,03	0	0,96	0,024
3,25	13981	0	0,04	0	0	0,91	0,05	0	0	0	0,92	0,02	0,06	0	0,036
3,25	20560	0	0,04	0	0	0,96	0	0	0	0	0,12	0,88	0	0	0,016
3,25	3251	0,01	0,04	0	0	0,95	0	0	0	0	0	0,04	0,95	0,01	0,036
3,25	60306	0	0,04	0	0	0,96	0	0	0	0	0	0,16	0	0,84	0,01
3,25	2711	0,01	0,04	0,01	0	0,93	0	0	0,01	0	0	0,04	0,94	0,02	0,04
3,25	11011	0	0,04	0	0	0,96	0	0	0	0	0	0,04	0	0,96	0,012
3,25	5498	0	0,04	0	0	0,95	0,01	0	0	0	0,1	0,87	0	0,03	0,032
3,25	21380	0,01	0,04	0,01	0	0,94	0	0	0	0	0,02	0,04	0,94	0	0,032
3,25	13442	0	0,04	0,02	0	0,93	0	0	0	0,01	0,19	0,04	0,02	0,75	0,018
3,25	2911	0	0,04	0	0	0,96	0	0	0	0	0	0,04	0	0,96	0,01
3,25	1650	0	0,04	0	0,01	0,93	0	0	0,02	0	0,03	0,04	0	0,93	0,006
3,25	2882	0	0,04	0	0	0,96	0	0	0	0	0	1	0	0	0,028
3,25	1711	0	0,04	0	0	0,96	0	0	0	0	0,01	0,03	0	0,96	0,018
3,25	6999	0,01	0,01	0	0,17	0,81	0	0	0	0	0,81	0,01	0,08	0,1	0,046
3,25	4551	0	0,04	0	0	0,96	0	0	0	0	0	0,45	0,02	0,53	0,024
3,25	3471	0	0,04	0	0,01	0,95	0	0	0	0	0	0,04	0,01	0,95	0,022
3,25	1890	0	0,04	0	0	0,95	0	0	0	0,01	0,95	0,04	0,01	0	0,036
3,25	2587	0	0,04	0	0	0,96	0	0	0	0	0	0,03	0,97	0	0,03
3,25	7009	0	0,04	0	0	0,92	0	0,01	0,03	0	0,03	0,04	0,79	0,14	0,03
3,25	17836	0	0,04	0	0	0,96	0	0	0	0	0	0,04	0,96	0	0,024
3,25	2309	0	0,04	0	0	0,96	0	0	0	0	0	0,04	0	0,96	0,01

3,25	2644	0	0,04	0	0	0,96	0	0	0	0	0	0,97	0	0,03	0,02
3,25	21207	0	0,04	0	0,01	0,94	0,01	0	0	0	0	0,05	0	0,95	0,01
3,25	4422	0,01	0,03	0	0,1	0	0	0,01	0,8	0,05	0,71	0,17	0,12	0	0,046
3,25	2799	0	0,04	0	0	0,95	0	0	0,01	0	0,21	0,04	0,01	0,74	0,024
3,25	2050	0	0,04	0	0	0,95	0	0	0	0,01	0,02	0,04	0,62	0,32	0,014
3,25	31442	0	0,04	0	0	0,95	0	0,01	0	0	0,93	0,05	0,02	0	0,046
3,25	3510	0	0,04	0	0	0,96	0	0	0	0	0	0,04	0	0,96	0,006
3,25	14206	0	0,04	0	0	0,96	0	0	0	0	0	0,01	0,03	0,96	0,012
3,25	6529	0	0	0,01	0,07	0	0,07	0,04	0,81	0	0	0,13	0,85	0,02	0,042
3,25	16173	0	0,04	0	0	0,95	0,01	0	0	0	0	0,03	0,01	0,96	0,016
3,25	3037	0	0,04	0	0,01	0,95	0	0	0	0	0	0,05	0	0,95	0,008
3,25	5340	0	0,04	0	0	0,96	0	0	0	0	0	0,01	0,03	0,96	0,004
3,25	26183	0	0,04	0	0	0,92	0,04	0	0	0	0	0,01	0,08	0,91	0,012
3,25	5539	0,02	0,04	0	0	0,92	0	0,02	0	0	0,89	0,06	0,03	0,02	0,048
3,25	11386	0	0,04	0	0	0,96	0	0	0	0	0	0,04	0,96	0	0,028
3,25	17198	0	0,04	0	0	0,96	0	0	0	0	0	0,04	0,18	0,78	0,016
3,25	3345	0	0,04	0	0	0,96	0	0	0	0	0,07	0,04	0,02	0,87	0,012
3,25	31978	0	0,04	0	0	0,96	0	0	0	0	0	0,04	0	0,96	0,006
3,25	22528	0	0,04	0,02	0	0,93	0	0	0,01	0	0,01	0,06	0	0,93	0,016
3,25	21773	0	0,04	0	0,01	0,93	0,01	0	0,01	0	0,02	0,05	0,42	0,51	0,022
3,5	6968	0	0,04	0	0	0,96	0	0	0	0	0,13	0,04	0	0,83	0,012
3,5	2351	0	0,04	0	0,09	0,87	0	0	0	0	0	0,13	0,87	0	0,044
3,5	9175	0,01	0,04	0	0	0,95	0	0	0	0	0	0,05	0,95	0	0,026
3,5	1908	0	0,04	0	0,02	0,92	0	0,01	0	0,01	0,01	0,06	0,92	0,01	0,032
3,5	7776	0	0,04	0	0	0,96	0	0	0	0	0	1	0	0	0,032
3,5	1993	0	0,04	0	0	0,96	0	0	0	0	0	0,02	0,96	0,02	0,03
3,5	3165	0	0,04	0	0	0,87	0,08	0,01	0	0	0,95	0,04	0,01	0	0,044
3,5	2180	0	0,04	0	0	0,94	0	0,02	0	0	0	0,06	0,94	0	0,024
3,5	6447	0	0,04	0	0	0,96	0	0	0	0	0	0,04	0	0,96	0,012
3,5	8546	0	0,04	0	0	0,95	0	0,01	0	0	0,95	0,04	0	0,01	0,04
3,5	2272	0	0,04	0	0	0,95	0	0,01	0	0	0	0,08	0	0,92	0,022
3,5	20181	0	0,04	0	0	0,96	0	0	0	0	0	0,97	0,03	0	0,01
3,5	8119	0	0,04	0	0	0,95	0	0	0	0,01	0	0,05	0	0,95	0,016

3,5	26250	0	0,04	0	0	0,96	0	0	0	0	0	0,04	0,96	0	0,02
3,5	3731	0	0,04	0	0	0,96	0	0	0	0	0	0,04	0,61	0,35	0,022
3,5	11435	0,01	0,04	0	0,01	0,93	0	0,01	0	0	0,63	0,06	0,3	0,01	0,02
3,5	3310	0	0,04	0	0	0,96	0	0	0	0	0	0,04	0,96	0	0,022
3,5	9952	0	0,04	0	0	0,96	0	0	0	0	0	0,04	0,96	0	0,026
3,5	2284	0	0,04	0	0	0,91	0	0,05	0	0	0,03	0,03	0,01	0,93	0,024
3,5	1568	0	0,04	0	0	0,96	0	0	0	0	0	0,04	0,63	0,33	0,032
3,5	1615	0	0,04	0	0	0,96	0	0	0	0	0,5	0,05	0,43	0,02	0,034
3,5	18600	0	0,04	0	0	0,93	0,03	0	0	0	0,93	0,04	0	0,03	0,044
3,5	2887	0	0,04	0	0	0,89	0,07	0	0	0	0	0,04	0,85	0,11	0,016
3,5	16218	0	0,04	0	0	0,96	0	0	0	0	0	0,04	0,96	0	0,032
3,5	1758	0	0,04	0	0,01	0,93	0	0	0,02	0	0,02	0,98	0	0	0,036
3,5	11511	0	0,04	0	0	0,95	0	0,01	0	0	0,95	0,05	0	0	0,042
3,5	10309	0	0,04	0	0	0,96	0	0	0	0	0,04	0,04	0,92	0	0,028
3,5	16231	0,01	0,04	0	0	0,86	0,03	0	0,06	0	0,07	0,84	0,03	0,06	0,038
3,5	16435	0	0	0,01	0,08	0,78	0,12	0	0	0,01	0,08	0,01	0,13	0,78	0,046
3,5	20873	0	0,04	0	0,01	0,95	0	0	0	0	0,01	0,04	0	0,95	0,016
3,5	9502	0	0,04	0	0	0,95	0	0	0	0,01	0	0,37	0	0,63	0,01
3,5	2636	0	0,04	0	0	0,95	0	0,01	0	0	0	0,04	0,95	0,01	0,024
3,5	40110	0	0,04	0	0,09	0,87	0	0	0	0	0,87	0,04	0	0,09	0,044
3,5	1504	0	0,04	0	0	0,96	0	0	0	0	0,24	0,04	0	0,72	0,02
3,5	25803	0,01	0,04	0	0	0,94	0,01	0	0	0	0,94	0,05	0,01	0	0,032
3,5	21821	0	0,04	0	0	0,95	0	0	0	0,01	0,02	0,03	0	0,95	0,004
3,5	23650	0	0,04	0	0	0,96	0	0	0	0	0	0,04	0	0,96	0,006
3,5	21386	0	0,04	0	0	0,95	0	0	0,01	0	0,01	0,04	0	0,95	0,002
3,5	1387	0	0,04	0,01	0,01	0,91	0,02	0,01	0	0	0,16	0,1	0,72	0,02	0,036
3,5	3224	0	0,04	0,02	0	0,94	0	0	0	0	0,02	0,39	0,59	0	0,044
3,5	27127	0	0,04	0	0	0,92	0,03	0,01	0	0	0	0,04	0,94	0,02	0,028
3,5	3885	0	0,04	0	0	0,96	0	0	0	0	0	0,05	0,86	0,09	0,018
3,5	3141	0	0,04	0	0	0,96	0	0	0	0	0	0,04	0,96	0	0,028
3,5	9432	0,01	0,04	0	0	0,95	0	0	0	0	0,04	0	0,96	0	0,04
3,5	6112	0	0,04	0	0	0,95	0,01	0	0	0	0,13	0,05	0,82	0	0,028
3,5	9369	0,01	0,04	0	0,01	0,94	0	0	0	0	0	0,05	0,94	0,01	0,048

3,5	3175	0	0,04	0	0	0,96	0	0	0	0	0	0,04	0	0,96	0,014
3,5	2491	0	0,04	0	0	0,96	0	0	0	0	0	0,04	0	0,96	0,012
3,5	2062	0	0,04	0	0,01	0,94	0	0	0,01	0	0	0,05	0,95	0	0,032
3,5	10614	0	0,04	0	0	0,96	0	0	0	0	0,04	0,04	0	0,92	0,016
3,5	9017	0,01	0,04	0	0	0,95	0	0	0	0	0,79	0,15	0,05	0,01	0,046
3,5	4602	0	0,04	0	0	0,96	0	0	0	0	0	0,04	0	0,96	0,006
3,5	3336	0	0,04	0	0,04	0,9	0	0	0,02	0	0	0,04	0,94	0,02	0,038
3,5	2713	0	0,04	0	0	0,91	0	0	0,04	0,01	0	0,04	0,05	0,91	0,016
3,5	2094	0	0,04	0	0	0,95	0,01	0	0	0	0,85	0,05	0	0,1	0,036
3,5	16124	0	0,04	0	0,03	0,81	0,02	0	0,09	0,01	0,05	0,13	0,73	0,09	0,034
3,5	3085	0,03	0,01	0,01	0,01	0,94	0	0	0	0	0,83	0,14	0,03	0	0,042
3,5	5870	0	0,04	0	0	0,96	0	0	0	0	0	0,04	0,96	0	0,02
3,5	1816	0	0,04	0	0,04	0,92	0	0	0	0	0	0,99	0,01	0	0,04
3,5	8710	0	0,04	0	0,02	0,93	0	0	0	0,01	0	0,04	0,96	0	0,038
3,5	2353	0	0,04	0	0	0,95	0,01	0	0	0	0,95	0	0,01	0,04	0,042
3,5	3394	0	0,04	0,01	0	0,94	0	0	0,01	0	0,09	0,03	0,01	0,87	0,02
3,5	6491	0	0,04	0	0	0,94	0	0	0,02	0	0,01	0,04	0,94	0,01	0,032
3,5	1442	0	0,04	0	0	0,94	0	0	0	0,02	0	0,06	0,94	0	0,024
3,5	7193	0	0	0	0,25	0,74	0	0	0,01	0	0,69	0,25	0,01	0,05	0,048
3,5	2758	0	0,04	0,01	0	0,95	0	0	0	0	0	0,04	0,01	0,95	0,014
3,5	2234	0	0,04	0	0,01	0,95	0	0	0	0	0	0,04	0,95	0,01	0,028
3,5	15888	0	0,04	0	0	0,96	0	0	0	0	0	0,82	0	0,18	0,02
3,5	11146	0	0,04	0,01	0	0,95	0	0	0	0	0,94	0,04	0,02	0	0,048
3,5	2701	0	0,04	0	0	0,96	0	0	0	0	0	0,04	0	0,96	0,004
3,5	15097	0	0,04	0	0,01	0,94	0,01	0	0	0	0,03	0,01	0,14	0,82	0,026
3,5	11902	0	0,04	0	0,01	0,91	0	0	0,04	0	0	0	0,95	0,05	0,022
3,5	15067	0	0,04	0	0	0,94	0,02	0	0	0	0,95	0,03	0	0,02	0,04
3,5	4777	0,01	0,04	0	0	0,94	0,01	0	0	0	0,02	0,04	0	0,94	0,024
3,5	1823	0	0,04	0	0	0,96	0	0	0	0	0	0,05	0,95	0	0,018
3,5	5094	0	0	0	0,18	0,82	0	0	0	0	0,64	0	0,36	0	0,048
3,5	6567	0	0,04	0	0	0,94	0	0	0,01	0,01	0,02	0,05	0	0,93	0,012
3,5	4896	0	0,04	0,01	0	0,95	0	0	0	0	0,36	0,63	0,01	0	0,048
3,5	20993	0	0,04	0	0	0,78	0,18	0	0	0	0,81	0	0	0,19	0,04

3,5	13634	0	0,04	0	0	0,96	0	0	0	0	0	0,04	0,01	0,95	0,008
3,5	3438	0,01	0,04	0	0	0,93	0,01	0	0,01	0	0,01	0,02	0,02	0,95	0,014
3,5	1625	0	0,04	0	0	0,96	0	0	0	0	0	0,04	0,75	0,21	0,024
3,5	4536	0	0,04	0	0	0,96	0	0	0	0	0	0,04	0,96	0	0,032
3,5	17857	0	0,04	0	0	0,9	0	0	0,05	0,01	0,03	0,15	0,01	0,81	0,018
3,5	4704	0	0,04	0	0,01	0,95	0	0	0	0	0	0,04	0,95	0,01	0,034
3,5	2078	0	0,04	0	0	0,96	0	0	0	0	0	0,05	0,95	0	0,02
3,5	1982	0	0,04	0	0	0,96	0	0	0	0	0	0,22	0	0,78	0,008
3,5	6594	0	0,04	0	0,01	0,95	0	0	0	0	0,01	0,99	0	0	0,024
3,5	14544	0	0,04	0	0	0,96	0	0	0	0	0	0,04	0,96	0	0,018
3,5	17548	0	0	0	0,09	0,91	0	0	0	0	0,09	0	0	0,91	0,046
3,5	3349	0	0,04	0	0	0,96	0	0	0	0	0	0,04	0,96	0	0,02
3,5	8906	0	0,04	0	0	0,96	0	0	0	0	0,44	0,01	0,55	0	0,034
3,5	9956	0,02	0,04	0	0	0,93	0	0	0,01	0	0,01	0,99	0	0	0,046
3,5	4987	0	0,04	0	0,01	0,93	0	0	0,02	0	0,03	0,97	0	0	0,02
3,5	1623	0	0,04	0	0	0,96	0	0	0	0	0	0,04	0	0,96	0,004
3,5	1345	0	0,04	0	0	0,95	0,01	0	0	0	0,03	0,02	0,19	0,76	0,022
3,5	2131	0	0,04	0,01	0	0,94	0	0	0	0,01	0	0,05	0,03	0,92	0,018
3,5	2239	0	0,04	0	0,07	0,83	0	0	0,01	0,05	0,82	0,05	0,05	0,08	0,042
3,5	5632	0	0,04	0	0	0,95	0	0	0,01	0	0	0,01	0	0,99	0,01
3,5	6142	0	0,04	0	0	0,94	0	0	0,02	0	0	0,04	0,94	0,02	0,02
3,75	7842	0	0,04	0	0	0,96	0	0	0	0	0	1	0	0	0,014
3,75	5846	0	0,04	0	0	0,96	0	0	0	0	0	0,04	0	0,96	0,004
3,75	37299	0	0,04	0	0	0,95	0	0,01	0	0	0	0,05	0,74	0,21	0,022
3,75	22733	0	0,04	0	0	0,96	0	0	0	0	0,92	0,04	0,04	0	0,026
3,75	8684	0	0,04	0	0	0,95	0,01	0	0	0	0,95	0,04	0,01	0	0,04
3,75	32406	0	0,04	0	0	0,96	0	0	0	0	0	0,04	0,96	0	0,03
3,75	2658	0	0,04	0	0	0,96	0	0	0	0	0	0,08	0,92	0	0,022
3,75	14836	0,01	0,04	0	0	0,95	0	0	0	0	0,01	0,84	0	0,15	0,032
3,75	1624	0	0,04	0	0	0,92	0	0	0,04	0	0,04	0,04	0,92	0	0,022
3,75	6485	0	0,04	0	0,03	0,92	0	0,01	0	0	0,82	0,01	0,04	0,13	0,034
3,75	15454	0,01	0,04	0,02	0	0,93	0	0	0	0	0,02	0,01	0,93	0,04	0,04
3,75	3917	0	0,04	0	0	0,96	0	0	0	0	0	0,81	0	0,19	0,024

3,75	4906	0	0,04	0,01	0,04	0,9	0,01	0	0	0	0,94	0,05	0	0,01	0,046
3,75	11711	0	0,04	0	0	0,96	0	0	0	0	0,01	0,04	0	0,95	0,006
3,75	1323	0	0,04	0	0	0,94	0	0,01	0,01	0	0	0,04	0	0,96	0,016
3,75	2724	0	0,04	0	0	0,95	0	0	0	0,01	0	0,04	0,94	0,02	0,024
3,75	4548	0	0,04	0	0	0,96	0	0	0	0	0,04	0,04	0,92	0	0,03
3,75	14717	0	0,04	0	0	0,92	0	0	0,04	0	0	0,92	0,04	0,04	0,026
3,75	15539	0	0,04	0	0	0,96	0	0	0	0	0	1	0	0	0,024
3,75	5967	0	0,04	0	0	0,92	0,03	0,01	0	0	0,02	0,96	0,02	0	0,038
3,75	11827	0	0,04	0	0	0,96	0	0	0	0	0,02	0,04	0	0,94	0,008
3,75	20146	0	0,04	0	0	0,96	0	0	0	0	0,11	0,03	0,86	0	0,022
3,75	1981	0	0,04	0	0	0,96	0	0	0	0	0	0,04	0	0,96	0,014
3,75	2552	0	0,04	0	0	0,95	0,01	0	0	0	0,02	0,04	0,93	0,01	0,026
3,75	2866	0	0,04	0	0	0,95	0	0	0,01	0	0	0,04	0,95	0,01	0,028
3,75	1933	0	0,04	0,01	0	0,95	0	0	0	0	0,87	0,04	0,08	0,01	0,036
3,75	2178	0	0,04	0	0	0,96	0	0	0	0	0	0,28	0,72	0	0,03
3,75	3701	0	0,04	0	0	0,96	0	0	0	0	0,96	0	0	0,04	0,044
3,75	2811	0	0,04	0	0	0,95	0	0	0,01	0	0,26	0,07	0,01	0,66	0,018
3,75	11042	0	0,04	0	0,01	0,95	0	0	0	0	0	0,04	0,69	0,27	0,036
3,75	11462	0	0,04	0	0	0,92	0	0	0,04	0	0	0,08	0	0,92	0,004
3,75	1232	0	0,04	0	0,01	0,95	0	0	0	0	0,48	0,05	0,05	0,42	0,026
3,75	12056	0	0	0	0,1	0,85	0	0	0,02	0,03	0,09	0,41	0,02	0,48	0,048
3,75	1777	0	0,04	0	0	0,95	0	0	0	0,01	0,01	0,04	0	0,95	0,01
3,75	25502	0	0,04	0	0	0,96	0	0	0	0	0,1	0,04	0	0,86	0,016
3,75	4314	0	0,04	0	0	0,94	0,01	0	0	0,01	0,01	0,03	0,96	0	0,03
3,75	1687	0	0,04	0	0	0,96	0	0	0	0	0	0,02	0	0,98	0,01
3,75	1645	0	0,04	0	0,02	0,94	0	0	0	0	0,02	0,04	0,94	0	0,044
3,75	16471	0	0,04	0	0	0,96	0	0	0	0	0,29	0,04	0,67	0	0,032
3,75	19478	0	0,04	0	0,01	0,95	0	0	0	0	0,07	0,04	0	0,89	0,022
3,75	7768	0,01	0,04	0,01	0	0,93	0	0	0	0,01	0,03	0,02	0,02	0,93	0,022
3,75	5737	0	0,04	0	0	0,96	0	0	0	0	0,01	0,99	0	0	0,02
3,75	11856	0	0,04	0	0	0,95	0,01	0	0	0	0,04	0,01	0,95	0	0,028
3,75	16570	0	0,04	0,01	0	0,94	0,01	0	0	0	0	0,04	0,01	0,95	0,006
3,75	20587	0	0,04	0	0	0,96	0	0	0	0	0	0,04	0,96	0	0,024

3,75	1785	0	0,04	0	0	0,96	0	0	0	0	0	0,04	0,86	0,1	0,028
3,75	8768	0	0,04	0	0	0,94	0	0	0	0,02	0,95	0,03	0	0,02	0,038
3,75	11574	0	0,04	0	0	0,96	0	0	0	0	0,9	0,1	0	0	0,04
3,75	21400	0,01	0,04	0	0,01	0,92	0,02	0	0	0	0	0,07	0,93	0	0,032
3,75	22905	0	0	0	0,13	0,87	0	0	0	0	0,12	0,01	0,87	0	0,044
3,75	7460	0	0,04	0,01	0,01	0,82	0	0,04	0	0,08	0,02	0,16	0,01	0,81	0,044
3,75	7310	0	0,04	0	0,04	0,9	0,01	0	0	0,01	0	0,03	0,91	0,06	0,04
3,75	18415	0,01	0,04	0	0	0,95	0	0	0	0	0	0,04	0,01	0,95	0,026
3,75	2008	0	0,04	0	0,01	0,95	0	0	0	0	0	0,04	0,95	0,01	0,04
3,75	2535	0	0,04	0	0	0,95	0	0,01	0	0	0,96	0,04	0	0	0,044
3,75	15366	0	0,04	0	0	0,95	0	0	0,01	0	0	0,05	0	0,95	0,008
3,75	27498	0	0,04	0	0	0,96	0	0	0	0	0	0,04	0	0,96	0,014
3,75	1298	0	0,04	0	0	0,94	0	0	0,02	0	0	0,97	0	0,03	0,028
3,75	6132	0	0,04	0	0,02	0,89	0,02	0	0,03	0	0,91	0,05	0	0,04	0,036
3,75	2077	0	0,04	0	0,01	0,95	0	0	0	0	0	0,07	0,93	0	0,026
3,75	5600	0,01	0,04	0	0,03	0,92	0	0	0	0	0	0,07	0,93	0	0,036
3,75	4994	0	0,03	0,02	0,11	0,82	0,01	0	0	0,01	0,82	0,03	0,12	0,03	0,048
3,75	5554	0	0,04	0	0	0,96	0	0	0	0	0	0,04	0	0,96	0,006
3,75	8663	0	0,04	0	0,01	0,95	0	0	0	0	0,01	0,03	0	0,96	0,02
3,75	5927	0	0,04	0	0	0,95	0	0	0,01	0	0	0,05	0	0,95	0,014
3,75	8907	0	0,04	0	0	0,96	0	0	0	0	0	0,04	0	0,96	0,012
3,75	4034	0	0,04	0	0	0,95	0	0	0,01	0	0	0,99	0	0,01	0,022
3,75	3386	0	0,04	0	0	0,96	0	0	0	0	0	1	0	0	0,018
3,75	6480	0,02	0,04	0	0	0,91	0,03	0	0	0	0	0,1	0,04	0,86	0,026
3,75	6383	0	0,04	0	0	0,95	0	0	0,01	0	0,01	0,04	0	0,95	0,008
3,75	1782	0,01	0,04	0	0	0,95	0	0	0	0	0	0,04	0,95	0,01	0,03
3,75	2619	0	0,04	0	0,03	0,93	0	0	0	0	0	0,01	0,96	0,03	0,032
3,75	1609	0	0,04	0	0,02	0,93	0	0,01	0	0	0,01	0,97	0	0,02	0,042
3,75	3326	0	0,04	0	0,01	0,95	0	0	0	0	0	0,05	0,95	0	0,038
3,75	12140	0	0,04	0	0,01	0,95	0	0	0	0	0	0,04	0,95	0,01	0,024
3,75	6708	0	0,04	0	0	0,96	0	0	0	0	0	1	0	0	0,024
3,75	7088	0	0,04	0	0	0,95	0	0	0	0,01	0,95	0,04	0,01	0	0,036
3,75	28517	0	0,04	0	0	0,95	0,01	0	0	0	0,17	0,04	0,18	0,61	0,018

3,75	37845	0	0,04	0	0,03	0,92	0	0,01	0	0	0	0,07	0,92	0,01	0,038
3,75	2360	0,02	0,04	0	0	0,94	0	0	0	0	0,02	0,04	0	0,94	0,038
3,75	9561	0	0,04	0	0	0,96	0	0	0	0	0,01	0,04	0,17	0,78	0,014
3,75	18597	0	0,04	0	0,03	0,93	0	0	0	0	0,93	0,04	0,01	0,02	0,032
3,75	21546	0	0,04	0	0,02	0,86	0,01	0	0,07	0	0	0,94	0,04	0,02	0,036
3,75	3126	0	0,04	0,01	0	0,95	0	0	0	0	0	0,99	0	0,01	0,024
3,75	4256	0	0,03	0	0,07	0,85	0	0,03	0	0,02	0	0,88	0,09	0,03	0,048
3,75	25558	0,01	0,04	0,02	0	0,93	0	0	0	0	0,03	0,04	0,93	0	0,032
3,75	21939	0	0,04	0,01	0	0,91	0	0	0,04	0	0	0,08	0,01	0,91	0,004
3,75	4888	0	0,04	0	0	0,96	0	0	0	0	0,59	0,04	0	0,37	0,03
3,75	1659	0	0,04	0	0	0,95	0	0	0	0,01	0	0,05	0	0,95	0,004
3,75	11626	0	0	0	0,16	0,81	0	0	0,03	0	0,13	0,81	0,06	0	0,04
3,75	1692	0	0,04	0	0	0,94	0	0,01	0	0,01	0,01	0,05	0,94	0	0,032
3,75	2613	0	0,04	0	0	0,95	0	0	0	0,01	0	0	1	0	0,022
3,75	9338	0	0,04	0,03	0,01	0,92	0	0	0	0	0,03	0	0,04	0,93	0,018
3,75	3205	0	0,04	0	0	0,96	0	0	0	0	0	0,04	0	0,96	0,008
3,75	14839	0	0,04	0	0,05	0,69	0	0	0,22	0	0,04	0,27	0	0,69	0,046
3,75	1675	0	0,04	0	0	0,95	0,01	0	0	0	0	0,04	0,01	0,95	0,006
3,75	6329	0	0,04	0	0	0,96	0	0	0	0	0,29	0,02	0,68	0,01	0,03
3,75	25066	0	0,04	0	0	0,95	0	0,01	0	0	0	0,05	0	0,95	0,006
3,75	2555	0	0,04	0	0,03	0,91	0,02	0	0	0	0,91	0,04	0,02	0,03	0,044
3,75	59489	0	0,04	0	0	0,96	0	0	0	0	0	0,03	0,01	0,96	0,008
4	5231	0	0,04	0	0,04	0,89	0,01	0	0,02	0	0,88	0,1	0	0,02	0,026
4	1596	0	0,04	0	0	0,89	0,07	0	0	0	0,05	0,06	0,89	0	0,016
4	2778	0	0,04	0	0	0,95	0	0	0,01	0	0	0,05	0	0,95	0,006
4	11928	0	0,04	0	0,01	0,94	0	0,01	0	0	0,01	0,04	0,15	0,8	0,022
4	3270	0	0,04	0	0	0,95	0	0	0,01	0	0,01	0,04	0,95	0	0,03
4	8889	0	0,04	0,03	0,05	0,88	0	0	0	0	0,02	0,1	0,88	0	0,042
4	17714	0	0,04	0	0	0,95	0	0	0,01	0	0	0,05	0,95	0	0,028
4	2504	0	0,04	0,01	0	0,95	0	0	0	0	0	0,04	0,01	0,95	0,008
4	5500	0	0,04	0	0,03	0,92	0	0	0,01	0	0,01	0,07	0,92	0	0,03
4	23769	0	0,04	0	0	0,95	0,01	0	0	0	0,02	0,05	0,93	0	0,02
4	957	0	0,04	0,03	0	0,93	0	0	0	0	0,96	0,04	0	0	0,048

4	5913	0,01	0,04	0,01	0	0,94	0	0	0	0	0	0,04	0,96	0	0,044
4	34736	0	0,04	0	0	0,96	0	0	0	0	0	0,04	0	0,96	0,004
4	12282	0	0,04	0	0	0,76	0,2	0	0	0	0,03	0	0,77	0,2	0,03
4	1842	0	0,04	0	0	0,96	0	0	0	0	0	0,04	0	0,96	0,008
4	20534	0	0,04	0	0	0,94	0	0,02	0	0	0,94	0,05	0	0,01	0,036
4	1928	0	0,04	0	0,01	0,94	0,01	0	0	0	0,43	0,57	0	0	0,036
4	14057	0	0,04	0	0	0,96	0	0	0	0	0	0,04	0,96	0	0,03
4	9200	0	0,04	0	0	0,96	0	0	0	0	0	0,04	0,96	0	0,018
4	2355	0	0,04	0	0	0,96	0	0	0	0	0,95	0,04	0	0,01	0,034
4	6862	0	0,04	0	0	0,95	0	0	0	0,01	0	0,99	0	0,01	0,024
4	1716	0	0,04	0	0	0,93	0	0,03	0	0	0	0,04	0,93	0,03	0,038
4	66719	0	0,04	0	0	0,96	0	0	0	0	0	0	0	1	0,002
4	1526	0	0,04	0	0	0,96	0	0	0	0	0	0,05	0,95	0	0,028
4	14559	0	0,04	0	0	0,96	0	0	0	0	0	0,04	0,96	0	0,026
4	24369	0	0,04	0	0	0,94	0	0	0	0,02	0	0,05	0,9	0,05	0,022
4	42912	0,02	0	0	0,14	0,23	0,54	0	0,07	0	0,54	0,44	0	0,02	0,048
4	4471	0,01	0,04	0	0	0,95	0	0	0	0	0	0,04	0,02	0,94	0,022
4	8606	0	0,04	0	0	0,92	0,03	0,01	0	0	0,02	0,81	0,16	0,01	0,026
4	9919	0	0,04	0	0	0,92	0,04	0	0	0	0	0,04	0,96	0	0,014
4	6673	0	0,04	0	0,01	0,95	0	0	0	0	0	0,02	0,95	0,03	0,026
4	1510	0	0,04	0	0	0,96	0	0	0	0	0	0,04	0,96	0	0,026
4	4551	0,01	0,04	0	0	0,95	0	0	0	0	0,04	0,01	0,95	0	0,036
4	4785	0	0,04	0	0	0,96	0	0	0	0	0	0,04	0,96	0	0,026
4	13064	0	0,04	0	0,01	0,95	0	0	0	0	0	0,05	0	0,95	0,008
4	5486	0	0,04	0	0	0,89	0,07	0	0	0	0,87	0,09	0	0,04	0,048
4	2841	0	0,04	0	0	0,96	0	0	0	0	0	0,04	0,96	0	0,03
4	23304	0	0,04	0	0	0,79	0	0	0,17	0	0,09	0,04	0	0,87	0,004
4	30870	0	0,04	0	0	0,94	0,01	0	0,01	0	0	0,04	0,02	0,94	0,008
4	2453	0	0,04	0	0	0,95	0,01	0	0	0	0	0,04	0,01	0,95	0,012
4	8852	0	0,01	0	0,1	0,89	0	0	0	0	0	0,83	0,1	0,07	0,046
4	22843	0	0,04	0	0	0,96	0	0	0	0	0	0,03	0	0,97	0,012
4	6462	0	0,04	0,02	0	0,91	0	0	0,03	0	0	0,96	0,02	0,02	0,03
4	2682	0	0,04	0	0	0,94	0	0	0,02	0	0,02	0,04	0	0,94	0,006

4	7867	0	0,04	0	0	0,95	0,01	0	0	0	0,01	0,04	0,95	0	0,02
4	2657	0	0,04	0	0	0,95	0	0,01	0	0	0	0,04	0,09	0,87	0,012
4	27751	0,01	0,04	0,02	0	0,93	0	0	0	0	0	0,03	0,02	0,95	0,02
4	2906	0,01	0,04	0	0	0,9	0,05	0	0	0	0,9	0,09	0,01	0	0,046
4	5823	0,01	0,04	0	0	0,93	0	0,02	0	0	0,04	0,01	0,07	0,88	0,036
4	5822	0	0,04	0	0	0,96	0	0	0	0	0	0,04	0	0,96	0,008
4	15340	0	0,04	0	0	0	0,96	0	0	0	0,04	0	0,96	0	0,018
4	12310	0	0,04	0,01	0	0,94	0	0,01	0	0	0,01	0,04	0,95	0	0,036
4	30721	0	0,04	0	0,07	0,88	0	0,01	0	0	0,71	0,01	0	0,28	0,042
4	10068	0	0,04	0,01	0	0,93	0	0	0,02	0	0,01	0,98	0,01	0	0,022
4	15133	0	0,04	0	0	0,96	0	0	0	0	0	0,95	0,02	0,03	0,014
4	5039	0	0,04	0	0	0,84	0	0	0,12	0	0	0,03	0,01	0,96	0,016
4	4394	0	0,01	0	0,22	0,77	0	0	0	0	0,78	0,02	0,2	0	0,048
4	34616	0	0,04	0	0	0,96	0	0	0	0	0	0,96	0	0,04	0,03
4	2129	0,01	0,04	0	0	0,9	0,05	0	0	0	0,01	0,04	0,9	0,05	0,038
4	44451	0	0,04	0,02	0	0,93	0	0	0,01	0	0,01	0,03	0,58	0,38	0,022
4	8696	0	0,04	0	0	0,96	0	0	0	0	0	0,03	0,96	0,01	0,036
4	5207	0,01	0,04	0,01	0	0,82	0,12	0	0	0	0,04	0,95	0,01	0	0,038
4	36898	0	0	0	0,13	0,83	0,01	0,03	0	0	0	0,04	0,96	0	0,046
4	1662	0	0,04	0	0,01	0,95	0	0	0	0	0,4	0,6	0	0	0,042
4	1772	0	0,04	0	0	0,96	0	0	0	0	0	0,04	0	0,96	0,014
4	2157	0	0,04	0	0	0,95	0	0	0	0,01	0	0,04	0,95	0,01	0,02
4	5729	0	0,04	0	0,02	0,94	0	0	0	0	0,02	0,04	0	0,94	0,022
4	11140	0	0,04	0	0	0,95	0	0,01	0	0	0	0,04	0,01	0,95	0,026
4	32253	0	0	0,03	0,06	0,88	0,03	0	0	0	0,03	0,03	0,06	0,88	0,038
4	1555	0	0,04	0	0	0,95	0	0	0	0,01	0	0,04	0,95	0,01	0,034
4	14493	0,02	0,04	0	0	0,93	0,01	0	0	0	0,03	0,04	0	0,93	0,034
4	2782	0	0,04	0	0	0,96	0	0	0	0	0,23	0,04	0	0,73	0,012
4	11386	0	0,04	0	0	0,96	0	0	0	0	0	0,04	0	0,96	0,014
4	7387	0	0,04	0	0	0,93	0	0	0,03	0	0,03	0,97	0	0	0,034
4	3469	0	0,04	0	0	0,95	0,01	0	0	0	0	0,05	0,95	0	0,026
4	22871	0,01	0,04	0	0	0,94	0,01	0	0	0	0,01	0,04	0,33	0,62	0,028
4	13589	0	0,04	0	0	0,96	0	0	0	0	0	0,69	0	0,31	0,022

4	16194	0	0,04	0	0	0,96	0	0	0	0	0	1	0	0	0,026
4	22324	0	0,04	0	0	0,96	0	0	0	0	0	0,04	0	0,96	0,014
4	7945	0	0,04	0	0	0,96	0	0	0	0	0	0,03	0,96	0,01	0,012
4	8959	0	0,04	0	0,03	0,93	0	0	0	0	0,11	0,03	0,01	0,85	0,04
4	1805	0	0,04	0	0	0,95	0	0	0	0,01	0	0,04	0,96	0	0,014
4	5353	0	0,04	0	0,01	0,95	0	0	0	0	0,95	0,05	0	0	0,044
4	3394	0	0,04	0	0	0,96	0	0	0	0	0,77	0,04	0,18	0,01	0,04
4	2913	0,04	0,03	0	0	0,93	0	0	0	0	0,47	0,03	0,46	0,04	0,04
4	15078	0	0,04	0	0	0,92	0	0,01	0,02	0,01	0,02	0,05	0	0,93	0,024
4	2670	0,01	0,04	0	0	0,95	0	0	0	0	0	0,08	0	0,92	0,024
4	2862	0	0,04	0,01	0	0,95	0	0	0	0	0	0,04	0,95	0,01	0,046
4	38536	0	0,04	0	0	0,94	0	0	0	0,02	0,04	0,02	0,01	0,93	0,008
4	1981	0	0,04	0	0	0,96	0	0	0	0	0	0,03	0,08	0,89	0,014
4	27598	0	0,04	0	0	0,95	0,01	0	0	0	0,05	0	0	0,95	0,014
4	1675	0,01	0,04	0,01	0	0,94	0	0	0	0	0,59	0,38	0,03	0	0,04
4	26275	0	0,04	0	0,01	0,88	0	0	0,07	0	0,95	0	0,01	0,04	0,042
4	5860	0	0,01	0	0,24	0,75	0	0	0	0	0,84	0,04	0,12	0	0,048
4	32267	0	0,04	0	0	0,89	0	0	0,07	0	0	0,08	0	0,92	0,01
4	8334	0	0,04	0	0	0,94	0,02	0	0	0	0	0,06	0,94	0	0,026
4	2073	0	0,04	0	0	0,96	0	0	0	0	0	0,04	0	0,96	0,014
4	2180	0	0,04	0,01	0	0,95	0	0	0	0	0	0,06	0	0,94	0,014
4	28107	0	0	0	0,12	0,76	0,08	0	0,03	0,01	0,13	0,75	0,1	0,02	0,046
4	9777	0,01	0,04	0	0	0,94	0	0	0	0,01	0,01	0,05	0,93	0,01	0,024
4,25	3316	0	0,04	0	0	0,96	0	0	0	0	0	0,99	0	0,01	0,014
4,25	3475	0,01	0,04	0	0	0,95	0	0	0	0	0	0,04	0,96	0	0,028
4,25	9828	0	0,04	0	0	0,96	0	0	0	0	0	0	0,04	0,96	0,016
4,25	4964	0	0,04	0	0	0,96	0	0	0	0	0	0,04	0,96	0	0,03
4,25	3085	0	0,04	0	0	0,96	0	0	0	0	0	0,04	0,96	0	0,022
4,25	12070	0	0	0	0,25	0,75	0	0	0	0	0,75	0	0,25	0	0,046
4,25	12741	0,02	0,04	0	0	0,94	0	0	0	0	0	0,93	0,03	0,04	0,042
4,25	23850	0,04	0	0	0,02	0,81	0,07	0	0	0,06	0,09	0	0,05	0,86	0,046
4,25	2677	0,01	0,04	0,01	0	0,94	0	0	0	0	0	0,98	0,01	0,01	0,042
4,25	2476	0	0,04	0	0	0,95	0,01	0	0	0	0,01	0,04	0,95	0	0,014

4,25	2859	0	0,04	0	0	0,95	0	0,01	0	0	0	0,99	0	0,01	0,038
4,25	7399	0	0,04	0	0	0,96	0	0	0	0	0	0	0,01	0,99	0,006
4,25	22214	0	0,04	0	0	0,94	0	0,02	0	0	0	0,25	0,73	0,02	0,034
4,25	8619	0,01	0,04	0	0	0,95	0	0	0	0	0	0,07	0,93	0	0,018
4,25	12309	0	0,04	0	0	0,96	0	0	0	0	0	1	0	0	0,026
4,25	5924	0	0,04	0	0	0,96	0	0	0	0	0	0,04	0	0,96	0,014
4,25	11920	0,01	0,04	0	0	0,95	0	0	0	0	0	0,02	0,98	0	0,034
4,25	14235	0,01	0,04	0	0	0,95	0	0	0	0	0	0,05	0,95	0	0,032
4,25	2278	0	0,04	0	0	0,94	0	0	0,02	0	0,02	0,04	0,94	0	0,018
4,25	2199	0	0,04	0,02	0,01	0,93	0	0	0	0	0,93	0,07	0	0	0,046
4,25	25215	0	0,04	0	0,01	0,95	0	0	0	0	0,95	0,03	0,01	0,01	0,036
4,25	12357	0	0,04	0	0	0,96	0	0	0	0	0	0,88	0,12	0	0,026
4,25	1953	0,02	0,04	0	0	0,93	0	0	0	0,01	0,95	0,05	0	0	0,046
4,25	6887	0	0,04	0,01	0,04	0,91	0	0	0	0	0,91	0,04	0,05	0	0,048
4,25	6555	0	0,04	0	0	0,95	0	0	0,01	0	0	0,05	0	0,95	0,018
4,25	3523	0	0,04	0	0	0,96	0	0	0	0	0	0,04	0	0,96	0,01
4,25	4106	0	0,04	0	0	0,96	0	0	0	0	0	1	0	0	0,016
4,25	15096	0	0,04	0	0	0,96	0	0	0	0	0	1	0	0	0,034
4,25	6850	0	0,04	0	0,01	0,94	0,01	0	0	0	0	0,03	0,02	0,95	0,026
4,25	30118	0,03	0,04	0	0	0,92	0	0,01	0	0	0	0,07	0	0,93	0,042
4,25	5562	0,01	0,04	0	0	0,91	0,04	0	0	0	0,04	0,04	0,92	0	0,042
4,25	3972	0,01	0,04	0,01	0	0,94	0	0	0	0	0	0,99	0	0,01	0,04
4,25	11286	0	0,04	0	0,04	0,89	0,02	0	0,01	0	0	0,04	0,89	0,07	0,042
4,25	23345	0,01	0,04	0,01	0	0,92	0,01	0,01	0	0	0,93	0,06	0	0,01	0,042
4,25	1920	0	0,04	0	0	0,96	0	0	0	0	0	0,04	0	0,96	0,008
4,25	49860	0	0,04	0	0,01	0,84	0	0,01	0,1	0	0,04	0,05	0,18	0,73	0,042
4,25	12328	0	0,04	0	0	0,96	0	0	0	0	0	0,04	0,96	0	0,02
4,25	24044	0	0,04	0	0,02	0,94	0	0	0	0	0,01	0,96	0,03	0	0,03
4,25	1873	0,02	0,04	0	0,01	0,91	0,02	0	0	0	0,01	0,04	0,02	0,93	0,026
4,25	16845	0	0,04	0	0	0,94	0	0,01	0	0,01	0,01	0,98	0,01	0	0,03
4,25	2568	0	0,04	0	0	0,96	0	0	0	0	0	0,04	0	0,96	0,01
4,25	10531	0,02	0,03	0	0	0,94	0	0,01	0	0	0,95	0,04	0	0,01	0,036
4,25	26681	0,01	0,04	0	0,01	0,94	0	0	0	0	0,06	0,04	0,81	0,09	0,034

4,25	1922	0	0,04	0,02	0	0	0	0	0,94	0	0	0,04	0,01	0,95	0,016
4,25	22244	0	0,04	0	0	0,94	0,01	0	0,01	0	0	0,05	0,94	0,01	0,024
4,25	15327	0	0,04	0	0,01	0,95	0	0	0	0	0	0,04	0	0,96	0,014
4,25	2023	0	0,04	0	0,01	0,94	0,01	0	0	0	0,03	0,17	0,01	0,79	0,022
4,25	2803	0	0,04	0	0	0,96	0	0	0	0	0	0,04	0,96	0	0,018
4,25	3381	0	0,04	0	0	0,96	0	0	0	0	0	0,01	0	0,99	0,012
4,25	2481	0	0,04	0	0	0,95	0	0	0	0,01	0	0,07	0	0,93	0,014
4,25	6668	0	0,04	0	0	0,96	0	0	0	0	0,87	0,1	0	0,03	0,042
4,25	5064	0	0,04	0	0	0,95	0	0,01	0	0	0,11	0,04	0,85	0	0,028
4,25	16223	0	0,04	0,01	0,03	0,92	0	0	0	0	0	0,01	0,07	0,92	0,032
4,25	3663	0,01	0,04	0	0	0,95	0	0	0	0	0	0,05	0	0,95	0,016
4,25	3309	0	0,04	0	0,01	0,94	0	0	0,01	0	0	0,02	0,05	0,93	0,01
4,25	4600	0	0,04	0	0	0,94	0,01	0,01	0	0	0,02	0,04	0	0,94	0,012
4,25	11780	0	0,04	0	0	0,95	0	0,01	0	0	0,01	0,04	0,12	0,83	0,024
4,25	6570	0	0,04	0	0	0,94	0	0	0,02	0	0	0,04	0	0,96	0,008
4,25	3169	0	0,04	0	0	0,96	0	0	0	0	0	0,04	0,01	0,95	0,014
4,25	15548	0	0,04	0	0	0,95	0	0	0,01	0	0,04	0,04	0,92	0	0,032
4,25	7602	0,03	0,04	0	0	0,93	0	0	0	0	0	0,02	0,95	0,03	0,046
4,25	1342	0	0,04	0	0	0,94	0	0	0,02	0	0	0,07	0,02	0,91	0,016
4,25	3841	0	0,04	0,01	0	0,95	0	0	0	0	0,01	0,04	0	0,95	0,014
4,25	1785	0	0,04	0	0,01	0,95	0	0	0	0	0,01	0,37	0,62	0	0,026
4,25	4727	0	0,04	0	0	0,96	0	0	0	0	0	0,03	0,97	0	0,024
4,25	10517	0	0,04	0,01	0	0,94	0	0	0	0,01	0	0,05	0,01	0,94	0,006
4,25	37383	0	0,04	0	0	0,95	0,01	0	0	0	0	0,05	0,95	0	0,03
4,25	20089	0	0,04	0	0	0,96	0	0	0	0	0	0,04	0,96	0	0,01
4,25	19466	0	0,04	0	0	0,95	0	0	0	0,01	0,14	0,04	0,82	0	0,022
4,25	10934	0	0,04	0	0,01	0,93	0,02	0	0	0	0	0,97	0,02	0,01	0,03
4,25	3011	0,01	0,04	0,03	0	0,92	0	0	0	0	0,92	0,05	0,02	0,01	0,048
4,25	4834	0	0,04	0	0	0,96	0	0	0	0	0	0,06	0,44	0,5	0,024
4,25	1274	0,02	0,04	0	0,02	0,92	0	0	0	0	0,02	0,04	0,94	0	0,042
4,25	9144	0	0,04	0	0	0,96	0	0	0	0	0	0,04	0	0,96	0,008
4,25	4083	0	0,04	0	0,04	0,83	0,04	0,01	0,04	0	0,1	0,79	0,11	0	0,046
4,25	1915	0	0,04	0	0,09	0,86	0	0	0	0,01	0,86	0,05	0,04	0,05	0,046

4,25	5797	0	0,04	0,01	0	0,95	0	0	0	0	0	0,99	0	0,01	0,022
4,25	17103	0	0,04	0	0	0,95	0,01	0	0	0	0,01	0,04	0,95	0	0,034
4,25	8295	0	0,04	0	0	0,96	0	0	0	0	0	0,04	0,09	0,87	0,022
4,25	6138	0,01	0,04	0	0	0,93	0,02	0	0	0	0,93	0,04	0,03	0	0,04
4,25	18314	0	0	0	0,13	0,85	0,01	0	0	0,01	0,1	0,88	0,02	0	0,04
4,25	2896	0	0,04	0	0	0,96	0	0	0	0	0,1	0,02	0	0,88	0,01
4,25	49350	0	0,04	0	0	0,9	0,01	0	0,05	0	0,06	0,04	0	0,9	0,014
4,25	3303	0	0,04	0	0,07	0,89	0	0	0	0	0,96	0,04	0	0	0,04
4,25	10873	0	0,04	0	0	0,96	0	0	0	0	0,04	0	0	0,96	0,006
4,25	19669	0	0,04	0	0,01	0,95	0	0	0	0	0	0,04	0,96	0	0,03
4,25	5892	0	0,04	0	0	0,96	0	0	0	0	0	0,06	0	0,94	0,016
4,25	34636	0	0,04	0,01	0	0,95	0	0	0	0	0	0,04	0,96	0	0,026
4,25	3662	0	0,04	0	0	0,96	0	0	0	0	0	0,04	0	0,96	0,014
4,25	9740	0	0	0	0,42	0	0,58	0	0	0	0,99	0,01	0	0	0,046
4,25	3784	0	0,04	0	0	0,95	0	0,01	0	0	0,01	0,04	0,95	0	0,024
4,25	4266	0	0,04	0	0	0,94	0,01	0,01	0	0	0,96	0,04	0	0	0,048
4,25	17343	0	0,04	0	0	0,95	0	0	0,01	0	0	0,05	0	0,95	0,004
4,25	2303	0	0,04	0	0,02	0,83	0	0	0,09	0,02	0	0,04	0,01	0,95	0,028
4,25	4776	0	0,04	0	0	0,96	0	0	0	0	0,01	0,04	0	0,95	0,004
4,25	3466	0	0,04	0	0	0,95	0	0	0,01	0	0,08	0,04	0	0,88	0,014
4,25	13490	0	0,04	0	0	0,96	0	0	0	0	0	0,04	0	0,96	0,01
4,25	20161	0	0,04	0	0	0,96	0	0	0	0	0,96	0	0,02	0,02	0,04
4,25	5792	0	0,04	0	0,05	0,91	0	0	0	0	0,11	0,87	0	0,02	0,048
4,25	6588	0	0,01	0	0,12	0,84	0	0,02	0	0,01	0	0	0,99	0,01	0,046
4,5	9199	0	0,04	0	0	0,96	0	0	0	0	0,35	0,05	0	0,6	0,028
4,5	1889	0	0,04	0	0	0,95	0,01	0	0	0	0	0,58	0,42	0	0,03
4,5	5053	0	0,04	0	0	0,96	0	0	0	0	0	0,05	0,95	0	0,03
4,5	3456	0	0,04	0	0	0,96	0	0	0	0	0	0,53	0,47	0	0,018
4,5	4187	0	0,04	0	0,01	0,91	0	0	0,04	0	0,01	0,08	0	0,91	0,022
4,5	1164	0	0,04	0	0	0,95	0	0	0,01	0	0,01	0,03	0,96	0	0,032
4,5	4058	0	0,04	0	0	0,91	0	0	0,05	0	0	0,13	0	0,87	0,008
4,5	14837	0,01	0,04	0	0	0,95	0	0	0	0	0,01	0,04	0	0,95	0,014
4,5	16218	0,01	0,04	0	0	0,95	0	0	0	0	0	0,04	0,01	0,95	0,02

4,5	16670	0	0,04	0	0	0,96	0	0	0	0	0	0,55	0,45	0	0,034
4,5	17989	0	0	0	0,1	0,88	0	0	0	0,02	0,08	0	0,54	0,38	0,036
4,5	23022	0	0,04	0	0	0,96	0	0	0	0	0	0,04	0	0,96	0,01
4,5	7514	0	0,04	0	0	0,96	0	0	0	0	0	0,04	0,96	0	0,024
4,5	2258	0,01	0,04	0	0	0,93	0	0,01	0,01	0	0,02	0,03	0,04	0,91	0,022
4,5	1982	0	0,04	0	0	0,95	0	0	0	0,01	0	0,04	0	0,96	0,016
4,5	2149	0	0,04	0	0	0,95	0	0,01	0	0	0,01	0,04	0,95	0	0,028
4,5	9064	0	0,04	0	0	0,96	0	0	0	0	0	0,04	0	0,96	0,016
4,5	2231	0	0,03	0,02	0	0,86	0,09	0	0	0	0,83	0,12	0,05	0	0,046
4,5	7842	0	0,04	0	0	0,95	0	0	0,01	0	0,01	0,03	0	0,96	0,006
4,5	38072	0	0,04	0	0	0,96	0	0	0	0	0,01	0,04	0	0,95	0,006
4,5	1948	0	0,04	0	0,01	0,94	0	0,01	0	0	0	0,05	0,01	0,94	0,02
4,5	18724	0	0,03	0	0,15	0,82	0	0	0	0	0,96	0	0	0,04	0,044
4,5	7613	0,01	0,04	0	0	0,95	0	0	0	0	0,03	0,02	0,95	0	0,032
4,5	32954	0,01	0,04	0	0	0,95	0	0	0	0	0	0,02	0,16	0,82	0,024
4,5	18109	0	0,04	0	0	0,84	0	0	0,12	0	0,12	0,66	0	0,22	0,024
4,5	29740	0	0,04	0	0	0,9	0	0,01	0,05	0	0,05	0,04	0,01	0,9	0,018
4,5	11860	0	0,04	0	0	0,94	0,01	0,01	0	0	0,01	0,05	0,94	0	0,01
4,5	4921	0	0,04	0	0	0,95	0	0,01	0	0	0	0,64	0,04	0,32	0,016
4,5	19010	0	0,04	0	0	0,96	0	0	0	0	0,01	0,04	0,95	0	0,04
4,5	6247	0	0,04	0	0	0,86	0,07	0	0	0,03	0,07	0,03	0,89	0,01	0,024
4,5	4954	0	0,04	0,03	0	0,93	0	0	0	0	0,73	0,06	0,21	0	0,042
4,5	13904	0	0,04	0	0	0,9	0	0	0,06	0	0,9	0,06	0,04	0	0,028
4,5	12890	0	0,04	0,01	0	0,94	0	0	0	0,01	0,01	0,05	0,94	0	0,032
4,5	23043	0	0,04	0	0	0,94	0,01	0	0,01	0	0	0,04	0,02	0,94	0,006
4,5	2601	0	0,04	0,01	0,01	0,93	0,01	0	0	0	0	0,95	0,03	0,02	0,044
4,5	11754	0	0,04	0	0,01	0,95	0	0	0	0	0,01	0,04	0	0,95	0,016
4,5	49426	0,02	0,03	0	0,02	0,86	0	0	0,07	0	0,06	0,03	0,88	0,03	0,03
4,5	12025	0	0,04	0	0	0,96	0	0	0	0	0	0,04	0	0,96	0,002
4,5	2846	0	0,04	0	0,01	0,95	0	0	0	0	0,04	0,01	0,01	0,94	0,01
4,5	53780	0	0,04	0	0	0,92	0,04	0	0	0	0,9	0,1	0	0	0,042
4,5	2163	0	0	0	0,34	0,63	0	0,02	0,01	0	0,96	0,02	0,01	0,01	0,048
4,5	26755	0	0,04	0	0	0,96	0	0	0	0	0	0,04	0,96	0	0,024

4,5	2224	0	0,04	0,01	0	0,93	0,02	0	0	0	0,02	0,01	0,93	0,04	0,03
4,5	5201	0	0,04	0	0	0,94	0	0,01	0,01	0	0,95	0,04	0	0,01	0,044
4,5	16164	0	0,04	0	0	0,95	0	0	0,01	0	0,02	0,04	0	0,94	0,002
4,5	10466	0	0,04	0	0	0,96	0	0	0	0	0	0,04	0,96	0	0,032
4,5	16948	0	0,04	0	0,01	0,94	0	0	0,01	0	0,94	0,05	0	0,01	0,032
4,5	2351	0	0,04	0	0,01	0,95	0	0	0	0	0,01	0,04	0,95	0	0,032
4,5	4478	0	0,04	0	0	0,96	0	0	0	0	0	0,04	0	0,96	0,016
4,5	67384	0	0,04	0	0	0,94	0	0	0	0,02	0	0,9	0,06	0,04	0,026
4,5	1325	0,01	0,04	0	0	0,95	0	0	0	0	0	0,71	0,01	0,28	0,034
4,5	7930	0,05	0	0	0	0,95	0	0	0	0	0,05	0	0,95	0	0,042
4,5	8184	0	0,04	0	0,01	0,95	0	0	0	0	0	0,03	0,97	0	0,028
4,5	3631	0,01	0,04	0	0	0,93	0,01	0,01	0	0	0,29	0,05	0,66	0	0,034
4,5	12020	0	0,04	0	0	0,95	0,01	0	0	0	0	0,05	0	0,95	0,012
4,5	56849	0	0,04	0,01	0	0,95	0	0	0	0	0	0,01	0,04	0,95	0,018
4,5	2248	0	0,04	0	0	0,94	0,02	0	0	0	0,94	0,04	0,02	0	0,046
4,5	12729	0	0,04	0	0,01	0,95	0	0	0	0	0,38	0,04	0	0,58	0,032
4,5	34967	0	0,04	0	0,01	0,95	0	0	0	0	0,04	0,02	0,94	0	0,04
4,5	2815	0	0,04	0	0	0,96	0	0	0	0	0	0,04	0,01	0,95	0,022
4,5	2735	0	0,03	0,03	0	0,94	0	0	0	0	0,99	0	0,01	0	0,042
4,5	48011	0,01	0,04	0	0,03	0,91	0	0	0	0,01	0,01	0,04	0,94	0,01	0,034
4,5	19205	0	0,04	0	0	0,96	0	0	0	0	0,03	0,04	0,93	0	0,012
4,5	2055	0	0,04	0	0	0,92	0	0	0,04	0	0	0,22	0,74	0,04	0,032
4,5	9948	0	0,04	0	0	0,96	0	0	0	0	0,02	0,02	0,96	0	0,024
4,5	19792	0	0,04	0	0	0,96	0	0	0	0	0	0,04	0	0,96	0,01
4,5	28397	0,02	0,04	0,01	0	0,81	0,01	0	0,06	0,05	0,11	0	0,83	0,06	0,048
4,5	21176	0	0,04	0	0	0,96	0	0	0	0	0	0,04	0	0,96	0,004
4,5	10768	0	0,04	0,01	0	0,94	0	0	0,01	0	0,95	0,02	0,01	0,02	0,04
4,5	1569	0	0,04	0	0	0,96	0	0	0	0	0	0,09	0,91	0	0,038
4,5	8242	0	0,04	0	0	0,95	0,01	0	0	0	0	0,07	0	0,93	0,016
4,5	15549	0	0,04	0	0,08	0,88	0	0	0	0	0,54	0,38	0,06	0,02	0,028
4,5	25814	0,02	0,04	0	0	0,94	0	0	0	0	0	0,04	0,94	0,02	0,042
4,5	38658	0,01	0,04	0	0	0,95	0	0	0	0	0	0,04	0,95	0,01	0,034
4,5	4233	0	0,04	0	0	0,96	0	0	0	0	0	0,04	0,96	0	0,036

4,5	7089	0	0,04	0	0,01	0,95	0	0	0	0	0	0,04	0,95	0,01	0,022
4,5	33713	0	0,04	0	0	0,93	0	0	0,03	0	0	0,04	0,03	0,93	0,012
4,5	6317	0	0,04	0,01	0	0,95	0	0	0	0	0,01	0,06	0,93	0	0,02
4,5	2390	0	0,04	0	0	0,75	0	0	0,21	0	0,19	0,06	0	0,75	0,01
4,5	24018	0	0,04	0	0,01	0,95	0	0	0	0	0	0,02	0,74	0,24	0,024
4,5	23321	0,01	0,04	0	0	0,95	0	0	0	0	0	0,04	0,07	0,89	0,02
4,5	7855	0,01	0	0,01	0,04	0	0,93	0	0,01	0	0,01	0,98	0	0,01	0,046
4,5	2406	0,01	0,04	0	0	0,85	0,1	0	0	0	0,09	0,04	0,86	0,01	0,038
4,5	1598	0	0,04	0	0	0,96	0	0	0	0	0,29	0,04	0,36	0,31	0,024
4,5	31315	0	0,04	0	0	0,9	0,01	0	0,05	0	0,33	0,09	0,01	0,57	0,026
4,5	11484	0	0,04	0	0	0,96	0	0	0	0	0	0,04	0	0,96	0,008
4,5	18104	0	0,04	0	0	0,96	0	0	0	0	0	0,04	0	0,96	0,014
4,5	9395	0	0,04	0	0	0,94	0	0	0,01	0,01	0,01	0,05	0	0,94	0,01
4,5	3316	0	0,04	0	0	0,92	0	0	0,04	0	0,03	0,03	0,02	0,92	0,01
4,5	6931	0	0,04	0,01	0	0,93	0	0	0,01	0,01	0,02	0,2	0	0,78	0,018
4,5	3227	0	0	0,02	0,14	0,84	0	0	0	0	0,14	0,08	0,77	0,01	0,048
4,5	2920	0	0,04	0	0	0,95	0	0	0,01	0	0,01	0,02	0,97	0	0,02
4,5	8445	0,03	0,04	0	0	0,92	0	0	0,01	0	0	0,04	0,93	0,03	0,042
4,5	6929	0	0,04	0	0	0,95	0	0	0,01	0	0,01	0,04	0	0,95	0,012
4,5	21294	0	0	0	0,43	0,57	0	0	0	0	0,99	0	0,01	0	0,046
4,5	2066	0	0,04	0	0	0,94	0	0	0,02	0	0,11	0,04	0	0,85	0,016
4,5	1300	0,01	0,04	0	0	0,95	0	0	0	0	0,01	0,04	0,95	0	0,028
4,5	2797	0	0,04	0	0	0,95	0	0	0,01	0	0	0,03	0,96	0,01	0,026
4,5	5621	0	0,04	0,01	0,02	0,92	0,01	0	0	0	0,02	0,31	0,66	0,01	0,044
4,5	19488	0	0,04	0	0	0,96	0	0	0	0	0	0,04	0	0,96	0,014
4,75	11476	0	0,04	0	0,02	0,94	0	0	0	0	0	0,98	0	0,02	0,036
4,75	14483	0	0,04	0	0	0,91	0	0	0,04	0,01	0,05	0,04	0,89	0,02	0,014
4,75	15937	0	0,04	0	0,01	0,95	0	0	0	0	0	0,05	0	0,95	0,014
4,75	17391	0,01	0,04	0	0	0,93	0	0,02	0	0	0	0,04	0,12	0,84	0,036
4,75	5905	0	0,04	0	0	0,91	0,05	0	0	0	0	0,95	0	0,05	0,03
4,75	3786	0	0,04	0,01	0	0,93	0	0	0,02	0	0,93	0,02	0,04	0,01	0,04
4,75	4481	0	0,04	0	0	0,96	0	0	0	0	0	0,27	0	0,73	0,014
4,75	13968	0	0,04	0	0	0,96	0	0	0	0	0,02	0,88	0	0,1	0,02

4,75	30317	0	0,04	0	0	0,95	0	0,01	0	0	0	0,04	0,01	0,95	0,022
4,75	25377	0	0,04	0	0	0,96	0	0	0	0	0	0,04	0	0,96	0,004
4,75	6841	0,01	0,04	0	0	0,95	0	0	0	0	0,02	0,03	0,95	0	0,042
4,75	9578	0	0	0,01	0	0	0	0,24	0,66	0,09	0,99	0	0,01	0	0,048
4,75	7911	0	0,04	0	0	0,94	0,01	0	0,01	0	0	0,99	0	0,01	0,024
4,75	9704	0	0,04	0	0	0,96	0	0	0	0	0	0,04	0	0,96	0,006
4,75	2387	0	0,04	0	0,02	0,91	0,02	0	0,01	0	0	0,98	0,01	0,01	0,028
4,75	8079	0	0,04	0	0	0,96	0	0	0	0	0,04	0,04	0	0,92	0,014
4,75	18728	0	0,04	0	0	0,95	0	0,01	0	0	0,96	0,03	0,01	0	0,044
4,75	9698	0	0,04	0,01	0	0,95	0	0	0	0	0,02	0,04	0,94	0	0,026
4,75	3041	0,01	0,04	0	0,01	0,94	0	0	0	0	0	0,04	0,02	0,94	0,026
4,75	28358	0	0,04	0	0,01	0,95	0	0	0	0	0	0,34	0,01	0,65	0,018
4,75	9842	0	0,04	0	0	0,96	0	0	0	0	0	0,09	0	0,91	0,01
4,75	3826	0,01	0,04	0	0	0,92	0,01	0	0,02	0	0	0,03	0,95	0,02	0,038
4,75	1980	0	0,04	0	0	0,96	0	0	0	0	0	1	0	0	0,026
4,75	16418	0	0,04	0	0	0,95	0,01	0	0	0	0	0,99	0,01	0	0,022
4,75	2141	0	0,04	0	0	0,96	0	0	0	0	0	0,04	0	0,96	0,004
4,75	4589	0	0,04	0	0	0,96	0	0	0	0	0	0,04	0	0,96	0,016
4,75	6270	0,01	0,04	0	0	0,9	0	0,01	0	0,04	0,05	0,28	0,65	0,02	0,02
4,75	4031	0	0,04	0	0	0,92	0	0	0,04	0	0	0,94	0,06	0	0,02
4,75	3005	0	0,04	0	0	0,93	0,02	0	0	0,01	0,05	0,04	0,91	0	0,028
4,75	1695	0	0,04	0	0	0,96	0	0	0	0	0	0,06	0,94	0	0,03
4,75	13310	0,01	0,04	0,01	0	0,92	0,01	0	0	0,01	0,01	0,13	0,02	0,84	0,032
4,75	6204	0,02	0,03	0	0	0,95	0	0	0	0	0	0,06	0,92	0,02	0,044
4,75	16511	0	0,04	0	0	0,96	0	0	0	0	0	0,04	0	0,96	0,004
4,75	6191	0	0,04	0	0	0,96	0	0	0	0	0,57	0,04	0	0,39	0,036
4,75	81013	0	0,04	0	0	0,83	0	0	0,13	0	0	0,08	0	0,92	0,004
4,75	2468	0	0,04	0	0,03	0,88	0,04	0	0	0,01	0,88	0,04	0,05	0,03	0,028
4,75	12166	0	0,04	0	0	0,92	0	0,04	0	0	0,03	0,04	0,92	0,01	0,044
4,75	16633	0	0,04	0,02	0	0,88	0,04	0	0,02	0	0	0,07	0,9	0,03	0,042
4,75	2645	0	0,04	0	0	0,96	0	0	0	0	0	0,06	0	0,94	0,012
4,75	6273	0	0,04	0,01	0	0,95	0	0	0	0	0	0,04	0,95	0,01	0,028
4,75	1732	0	0,04	0	0,06	0,88	0,01	0,01	0	0	0,02	0,15	0,52	0,31	0,048

4,75	20702	0	0,04	0	0,01	0,95	0	0	0	0	0	0,04	0,95	0,01	0,03
4,75	4150	0	0,03	0	0,12	0,85	0	0	0	0	0,85	0,03	0,12	0	0,048
4,75	4975	0	0,04	0	0	0,96	0	0	0	0	0	0,05	0,07	0,88	0,012
4,75	9265	0	0,04	0	0,01	0,67	0,01	0	0,26	0,01	0,29	0,02	0,67	0,02	0,03
4,75	6247	0,03	0,03	0,01	0	0,93	0	0	0	0	0	0,04	0,94	0,02	0,046
4,75	18232	0,02	0,04	0	0	0	0,89	0	0	0,05	0,04	0	0,91	0,05	0,04
4,75	45302	0,05	0	0	0	0,74	0	0,03	0,18	0	0,02	0,21	0,77	0	0,04
4,75	37119	0	0,04	0	0	0,95	0	0	0,01	0	0	0,04	0,96	0	0,03
4,75	2470	0	0,04	0	0	0,95	0	0,01	0	0	0,02	0,02	0,01	0,95	0,024
4,75	11614	0	0,04	0	0	0,96	0	0	0	0	0	0,02	0,98	0	0,036
4,75	9067	0	0,04	0	0	0,96	0	0	0	0	0	0,89	0,11	0	0,014
4,75	13476	0	0,04	0	0	0,96	0	0	0	0	0,01	0,99	0	0	0,038
4,75	2459	0	0,04	0	0	0,96	0	0	0	0	0	0,04	0,96	0	0,02
4,75	25408	0	0,04	0	0,02	0,93	0,01	0	0	0	0	0,04	0,95	0,01	0,034
4,75	40543	0	0,04	0	0	0	0	0	0,96	0	0	0	0	1	0,008
4,75	65368	0	0,04	0	0	0,95	0	0,01	0	0	0,01	0,04	0	0,95	0,02
4,75	31195	0	0,04	0	0	0,84	0	0	0,12	0	0	0,56	0,4	0,04	0,018
4,75	3261	0	0,04	0	0	0,96	0	0	0	0	0	1	0	0	0,022
4,75	30115	0	0	0	0,08	0,5	0,35	0,04	0,03	0	0	0,48	0,47	0,05	0,048
4,75	2115	0,01	0,04	0	0	0,95	0	0	0	0	0	0,04	0,88	0,08	0,022
4,75	5019	0	0,04	0	0	0,93	0	0,01	0,02	0	0,02	0,04	0,01	0,93	0,026
4,75	15495	0	0,04	0	0	0,96	0	0	0	0	0	0,04	0	0,96	0,008
4,75	2551	0	0,04	0	0	0,96	0	0	0	0	0,05	0,04	0	0,91	0,012
4,75	5175	0	0,04	0	0	0,96	0	0	0	0	0,45	0,02	0,53	0	0,03
4,75	6028	0,01	0,04	0	0	0,95	0	0	0	0	0	0,09	0	0,91	0,022
4,75	17862	0,04	0,04	0	0	0,92	0	0	0	0	0	0,04	0	0,96	0,046
4,75	2216	0	0,04	0	0	0,96	0	0	0	0	0,01	0,04	0	0,95	0,01
4,75	16598	0,01	0,04	0	0	0,95	0	0	0	0	0,03	0,96	0	0,01	0,032
4,75	9366	0	0,04	0	0	0,96	0	0	0	0	0	0,04	0,96	0	0,02
4,75	3886	0	0,04	0	0,01	0,94	0	0	0,01	0	0	0,96	0	0,04	0,026
4,75	38959	0	0,04	0	0	0,28	0	0	0,68	0	0,31	0	0	0,69	0,02
4,75	6379	0	0,04	0	0	0,96	0	0	0	0	0	0,04	0,8	0,16	0,034
4,75	9498	0	0,04	0	0	0,94	0	0	0,01	0,01	0,01	0,98	0,01	0	0,016

4,75	2035	0	0,04	0,01	0,05	0,9	0	0	0	0	0,9	0,04	0	0,06	0,036
4,75	13939	0	0,04	0	0	0,96	0	0	0	0	0	0,04	0	0,96	0,012
4,75	49600	0	0,04	0	0	0,96	0	0	0	0	0	0,04	0	0,96	0,014
4,75	9047	0	0,04	0,01	0	0,95	0	0	0	0	0	0,04	0,96	0	0,028
4,75	13194	0	0,04	0	0	0,95	0	0,01	0	0	0	0,04	0,95	0,01	0,032
4,75	1415	0,03	0,04	0	0	0,92	0,01	0	0	0	0,01	0,07	0,92	0	0,04
4,75	12019	0	0,04	0	0	0,96	0	0	0	0	0	0,04	0,96	0	0,026
4,75	1560	0	0,04	0	0	0,96	0	0	0	0	0	0,04	0	0,96	0,008
4,75	10465	0,01	0,04	0,01	0	0,94	0	0	0	0	0	0,04	0,94	0,02	0,038
4,75	19459	0	0,04	0	0	0,96	0	0	0	0	0	0,54	0	0,46	0,02
4,75	15410	0	0,04	0	0	0,96	0	0	0	0	0	1	0	0	0,024
4,75	8993	0	0,04	0	0	0,95	0	0	0	0,01	0,55	0,03	0	0,42	0,02
4,75	3251	0	0,04	0	0	0,96	0	0	0	0	0,42	0,58	0	0	0,038
4,75	13006	0	0,04	0,01	0	0,93	0	0	0,02	0	0	0,04	0,95	0,01	0,028
4,75	10528	0	0,04	0	0	0,9	0	0	0,06	0	0	0,1	0,02	0,88	0,01
4,75	6775	0	0,04	0	0	0,96	0	0	0	0	0	0,89	0	0,11	0,03
4,75	10850	0	0,04	0	0	0,7	0	0	0,26	0	0	0,3	0	0,7	0,01
4,75	24700	0	0,03	0	0,18	0,79	0	0	0	0	0,79	0,03	0,18	0	0,046
4,75	22243	0	0,04	0,01	0,02	0,92	0	0	0	0,01	0,02	0,06	0,92	0	0,034
4,75	26691	0	0	0,01	0,27	0,63	0,05	0	0,04	0	0,86	0,04	0,07	0,03	0,048
4,75	3214	0	0,04	0	0	0,95	0,01	0	0	0	0,02	0,47	0,02	0,49	0,012
4,75	10145	0	0,04	0	0	0,96	0	0	0	0	0,96	0,04	0	0	0,04
4,75	22720	0	0,04	0	0	0,95	0	0,01	0	0	0	0,98	0	0,02	0,028
4,75	20093	0,01	0,04	0	0	0,95	0	0	0	0	0,01	0,04	0,94	0,01	0,032
4,75	25639	0	0,04	0	0	0,96	0	0	0	0	0	1	0	0	0,024
4,75	21415	0	0,04	0	0	0,95	0	0	0,01	0	0	0,04	0,94	0,02	0,032
5	32890	0,01	0,04	0	0	0,93	0	0	0,02	0	0,02	0,01	0,25	0,72	0,014
5	4296	0	0,04	0,01	0	0,94	0	0	0,01	0	0,94	0,02	0,01	0,03	0,04
5	1963	0	0,04	0	0	0,96	0	0	0	0	0	0,04	0,96	0	0,014
5	14490	0	0,04	0	0	0,96	0	0	0	0	0	0,04	0,96	0	0,02
5	5652	0	0,04	0	0	0,96	0	0	0	0	0	0,03	0,01	0,96	0,01
5	13630	0	0,04	0,01	0	0,95	0	0	0	0	0,01	0,04	0,95	0	0,026
5	27060	0	0,04	0	0	0,96	0	0	0	0	0	0,04	0	0,96	0,01

5	32079	0	0,04	0	0	0,96	0	0	0	0	0	0,05	0,95	0	0,028
5	23239	0	0,04	0	0	0,95	0,01	0	0	0	0,01	0,04	0	0,95	0,004
5	1810	0	0,04	0	0	0,94	0	0,02	0	0	0	0,04	0,94	0,02	0,034
5	4905	0	0,04	0	0	0,96	0	0	0	0	0,96	0,04	0	0	0,046
5	20511	0	0,04	0	0	0,96	0	0	0	0	0	0,04	0,96	0	0,034
5	62427	0	0,04	0	0	0,94	0	0,02	0	0	0,01	0,04	0,94	0,01	0,034
5	19461	0	0,04	0	0,02	0,92	0	0	0,02	0	0	0,04	0,89	0,07	0,036
5	4704	0	0,04	0,01	0	0,94	0	0	0	0,01	0,01	0,04	0,94	0,01	0,034
5	24174	0	0,04	0	0	0,91	0,05	0	0	0	0	0,04	0,91	0,05	0,012
5	27850	0	0,04	0	0	0,96	0	0	0	0	0	0,04	0,96	0	0,022
5	20137	0	0,04	0	0,03	0,86	0,02	0,01	0,04	0	0	0,08	0,89	0,03	0,042
5	8057	0	0,04	0	0	0,96	0	0	0	0	0	0,04	0	0,96	0,01
5	1615	0	0,04	0,01	0	0,95	0	0	0	0	0	0,05	0,69	0,26	0,032
5	3295	0	0,04	0	0	0,96	0	0	0	0	0	0,04	0,96	0	0,022
5	16769	0	0,04	0	0	0,96	0	0	0	0	0	0,03	0,97	0	0,032
5	11474	0	0,04	0	0	0,96	0	0	0	0	0,45	0,36	0,18	0,01	0,024
5	12834	0	0,04	0	0,01	0,94	0	0	0	0,01	0,01	0,02	0,97	0	0,034
5	4008	0	0,04	0	0,01	0,94	0,01	0	0	0	0	0,07	0,01	0,92	0,024
5	2350	0	0,04	0	0,01	0,95	0	0	0	0	0	0,04	0,96	0	0,022
5	11505	0	0,04	0	0	0,95	0	0	0	0,01	0,3	0,04	0,65	0,01	0,026
5	14266	0	0,04	0,01	0	0,95	0	0	0	0	0	0,04	0	0,96	0,01
5	40198	0	0,04	0	0	0,96	0	0	0	0	0	0,05	0	0,95	0,014
5	13293	0	0,04	0	0	0,96	0	0	0	0	0,06	0,9	0	0,04	0,022
5	1854	0	0,04	0	0,02	0,9	0,02	0	0,02	0	0,56	0,04	0,03	0,37	0,038
5	9301	0	0,04	0	0,03	0,92	0	0,01	0	0	0,66	0,28	0	0,06	0,046
5	20862	0	0,04	0	0	0,95	0	0,01	0	0	0,01	0,03	0,01	0,95	0,01
5	22575	0	0,04	0	0	0,95	0,01	0	0	0	0	0,03	0,96	0,01	0,02
5	4774	0	0,04	0	0	0,95	0,01	0	0	0	0	0,62	0,08	0,3	0,024
5	3306	0	0,04	0	0	0,93	0,01	0	0,02	0	0,02	0,05	0	0,93	0,004
5	32471	0	0,04	0	0	0,91	0,05	0	0	0	0,24	0,35	0,28	0,13	0,034
5	14135	0	0,04	0	0	0,96	0	0	0	0	0,03	0,42	0	0,55	0,014
5	9653	0	0,04	0	0	0,93	0	0	0,03	0	0	0,04	0,96	0	0,032
5	18107	0	0,04	0	0	0,94	0	0	0,02	0	0	0,04	0,81	0,15	0,03

5	3036	0	0,04	0	0	0,96	0	0	0	0	0	0,04	0	0,96	0,006
5	2841	0	0,04	0,01	0,01	0,94	0	0	0	0	0,94	0,05	0,01	0	0,038
5	25988	0	0,04	0	0,08	0,86	0	0	0,02	0	0,08	0	0,86	0,06	0,046
5	3634	0	0,04	0	0,01	0,95	0	0	0	0	0	0,04	0	0,96	0,016
5	4756	0	0,04	0,01	0	0,84	0	0	0,11	0	0,02	0,03	0,95	0	0,022
5	39809	0	0,04	0	0	0,96	0	0	0	0	0,04	0	0	0,96	0,01
5	16272	0	0,04	0	0	0,95	0	0	0	0,01	0,01	0,04	0	0,95	0,006
5	19200	0	0,04	0	0	0,91	0,04	0	0	0,01	0,01	0,04	0,95	0	0,022
5	1586	0	0,04	0,01	0	0,95	0	0	0	0	0,01	0,04	0,95	0	0,032
5	7979	0	0,04	0	0	0,9	0	0	0,06	0	0	0,04	0,45	0,51	0,022
5	19341	0	0,04	0	0	0,93	0	0	0,03	0	0	0,05	0,95	0	0,026
5	26273	0	0,04	0	0	0,95	0	0	0	0,01	0	0,04	0,08	0,88	0,012
5	5074	0	0,04	0	0,05	0,89	0,01	0,01	0	0	0,01	0,93	0,01	0,05	0,042
5	22414	0	0,04	0	0	0,96	0	0	0	0	0	0,04	0	0,96	0,01
5	3468	0,01	0,04	0	0,01	0,92	0	0	0,01	0,01	0,03	0,95	0,02	0	0,046
5	2429	0	0,04	0	0,01	0,94	0	0	0	0,01	0,02	0,98	0	0	0,03
5	1495	0,01	0,04	0	0	0,95	0	0	0	0	0,01	0,04	0,5	0,45	0,03
5	17718	0	0,04	0	0	0,96	0	0	0	0	0	0,04	0,96	0	0,026
5	28124	0	0,04	0	0	0,96	0	0	0	0	0	0,04	0,96	0	0,036
5	2592	0	0,04	0	0	0,94	0,01	0	0,01	0	0	0,04	0	0,96	0,014
5	8531	0	0,04	0,01	0	0,93	0,02	0	0	0	0,81	0,05	0,14	0	0,042
5	2907	0	0,04	0	0	0,95	0	0	0,01	0	0,47	0,04	0	0,49	0,02
5	18759	0	0,04	0	0	0,96	0	0	0	0	0	0,04	0,96	0	0,038
5	4170	0,01	0,04	0	0	0,95	0	0	0	0	0,95	0,03	0	0,02	0,048
5	11850	0	0,04	0	0	0,94	0,01	0,01	0	0	0	0,05	0,01	0,94	0,022
5	34347	0	0,04	0	0	0,87	0,07	0	0,01	0,01	0,69	0,04	0,19	0,08	0,042
5	12537	0	0,04	0	0,02	0,94	0	0	0	0	0,01	0,05	0,94	0	0,036
5	11677	0	0,04	0	0	0,96	0	0	0	0	0	0,04	0	0,96	0,014
5	6347	0	0,04	0	0	0,94	0,01	0	0,01	0	0,02	0,28	0	0,7	0,01
5	10837	0	0,04	0	0	0,95	0,01	0	0	0	0,01	0,03	0,95	0,01	0,024
5	6319	0	0,04	0	0	0,96	0	0	0	0	0	0,04	0,96	0	0,024
5	40641	0	0,04	0	0	0,95	0	0	0,01	0	0	0,95	0,02	0,03	0,024
5	9137	0	0,04	0,01	0	0,95	0	0	0	0	0,95	0,04	0,01	0	0,046

5	23537	0,02	0,03	0	0,01	0,93	0	0	0,01	0	0	0,98	0,01	0,01	0,048
5	7107	0	0,04	0	0	0,95	0	0,01	0	0	0	0,04	0,01	0,95	0,024
5	7247	0	0,04	0,03	0	0,88	0	0,04	0	0,01	0,04	0,04	0,75	0,17	0,044
5	27695	0	0,04	0	0,01	0,94	0	0,01	0	0	0	0,05	0	0,95	0,02
5	17913	0	0,04	0	0	0,96	0	0	0	0	0,04	0	0	0,96	0,012
5	2238	0	0,04	0	0	0,96	0	0	0	0	0	0,01	0,03	0,96	0,014
5	7900	0	0,04	0	0	0,95	0	0	0	0,01	0	0,05	0,95	0	0,03
5	9019	0	0,04	0	0	0,94	0,01	0	0,01	0	0	0,06	0	0,94	0,004
5	1690	0	0,04	0	0	0,96	0	0	0	0	0	0,04	0	0,96	0,008
5	10504	0	0,04	0	0	0,95	0,01	0	0	0	0	0,04	0,95	0,01	0,028
5	25242	0,01	0,04	0	0	0,95	0	0	0	0	0,01	0,09	0,9	0	0,04
5	6393	0	0,04	0	0,02	0,93	0	0	0,01	0	0,94	0,06	0	0	0,048
5	4243	0	0,04	0	0,05	0,91	0	0	0	0	0	0,04	0,96	0	0,042
5	11134	0	0,04	0,01	0,02	0,92	0	0	0,01	0	0	0,04	0,04	0,92	0,032
5	16342	0	0,04	0	0	0,94	0	0,01	0,01	0	0	0,04	0,01	0,95	0,016
5	3807	0	0,04	0	0,01	0,95	0	0	0	0	0	0,13	0,01	0,86	0,018
5	11810	0	0,04	0	0	0,94	0	0	0,02	0	0	0,04	0,02	0,94	0,012
5	23825	0	0,04	0	0	0,96	0	0	0	0	0	0,04	0,96	0	0,01
5	23682	0	0,04	0	0	0,92	0	0	0	0,04	0	0,45	0,01	0,54	0,016
5	2663	0	0,04	0	0	0,94	0,01	0	0	0,01	0,01	0,05	0,01	0,93	0,008
5	4192	0	0,04	0	0	0,95	0,01	0	0	0	0	0,03	0,02	0,95	0,01
5	24681	0	0,04	0	0,01	0,92	0,01	0	0	0,02	0,02	0,93	0	0,05	0,028
5	2531	0	0,04	0	0,01	0,95	0	0	0	0	0	0,99	0,01	0	0,03
5	2823	0,01	0,04	0	0	0,95	0	0	0	0	0,01	0,17	0,82	0	0,04
5	2538	0	0,04	0	0	0,96	0	0	0	0	0,96	0,04	0	0	0,038
5	3175	0	0,04	0	0	0,96	0	0	0	0	0	0,04	0	0,96	0,006
5	1684	0	0,04	0	0	0,95	0,01	0	0	0	0	0,05	0	0,95	0,006
	8504,174	0,0017	0,03771	0,00141	0,01528	0,91428	0,01059	0,00185	0,01526	0,00192	0,19069	0,21275	0,29294	0,30362	0,027188

simple-standing

b	time_to_cc	(C,C)	(C,D)	(C,P)	(D,C)	(D,D)	(D,P)	(P,C)	(P,D)	(P,P)	S(G,G)	S(G,B)	S(B,G)	S(B,B)	
1	2712	0	0,04	0	0	0,96	0	0	0	0	0,01	0,03	0,96	0	0,024
1	13819	0	0,04	0	0	0,96	0	0	0	0	0	0,65	0	0,35	0,012
1	5300	0	0,04	0	0	0,96	0	0	0	0	0	0,04	0,01	0,95	0,008

1	2132	0,01	0,04	0	0	0,94	0,01	0	0	0	0,94	0,05	0,01	0	0,048
1	3515	0	0,04	0	0	0,95	0	0	0,01	0	0	0,04	0	0,96	0,008
1	11473	0	0	0	0,11	0,86	0,02	0,01	0	0	0	0,88	0,12	0	0,048
1	4262	0	0,04	0	0,08	0,73	0,15	0	0	0	0	0,15	0,85	0	0,046
1	6904	0	0,04	0	0	0,95	0,01	0	0	0	0	0,99	0	0,01	0,036
1	11047	0	0,04	0	0	0,64	0,31	0	0,01	0	0,04	0	0,94	0,02	0,02
1	5436	0	0,04	0,01	0	0,95	0	0	0	0	0,04	0,93	0,03	0	0,026
1	1996	0	0,04	0	0,14	0,82	0	0	0	0	0,8	0,18	0	0,02	0,048
1	2407	0	0,04	0	0	0,93	0,03	0	0	0	0,03	0	0	0,97	0,008
1	7386	0	0	0	0,1	0,52	0,25	0	0,13	0	0,01	0,93	0,03	0,03	0,046
1	1577	0	0,04	0	0	0,96	0	0	0	0	0,01	0,99	0	0	0,024
1	2386	0	0	0	0,15	0,85	0	0	0	0	0,17	0,83	0	0	0,044
1	1572	0	0,04	0,01	0	0,94	0	0	0	0,01	0,64	0,32	0,03	0,01	0,046
1	3357	0,01	0,04	0	0,09	0,85	0	0	0,01	0	0,02	0,93	0,05	0	0,036
1	1349	0,01	0,04	0	0	0,94	0	0	0	0,01	0	0,04	0,95	0,01	0,022
1	6078	0	0,03	0	0,09	0,88	0	0	0	0	0	0,91	0,09	0	0,046
1	2760	0	0,04	0	0	0,94	0,02	0	0	0	0,01	0,98	0,01	0	0,012
1	4638	0	0,04	0	0,03	0,93	0	0	0	0	0,92	0	0,03	0,05	0,04
1	13580	0,01	0,04	0	0	0,91	0,02	0	0,02	0	0,84	0,06	0	0,1	0,036
1	2216	0	0,04	0	0	0,84	0	0	0,12	0	0,11	0,01	0	0,88	0,024
1	9888	0	0	0	0,08	0,89	0,03	0	0	0	0,03	0,08	0	0,89	0,048
1	1627	0	0,04	0	0	0,94	0,02	0	0	0	0,01	0,74	0,05	0,2	0,018
1	6018	0,01	0	0	0,37	0,62	0	0	0	0	0,94	0	0,06	0	0,048
1	5956	0	0,04	0	0,02	0,94	0	0	0	0	0,71	0,03	0,02	0,24	0,03
1	8486	0	0,04	0	0	0,84	0,11	0	0,01	0	0,02	0,88	0,09	0,01	0,026
1	2799	0	0,04	0,01	0	0,94	0	0	0,01	0	0	0,47	0	0,53	0,016
1	6870	0	0	0	0,08	0,34	0	0	0,57	0,01	0	0,43	0	0,57	0,048
1	1644	0	0,04	0	0	0,95	0	0	0	0,01	0	0,05	0,95	0	0,028
1	6997	0	0	0	0,08	0,92	0	0	0	0	0	0,08	0	0,92	0,048
1	1290	0,01	0,04	0	0	0,94	0,01	0	0	0	0,07	0,9	0,01	0,02	0,028
1	4719	0	0,04	0	0	0,96	0	0	0	0	0	0,04	0,96	0	0,016
1	2043	0	0,04	0	0,04	0,92	0	0	0	0	0	0,97	0,02	0,01	0,038
1	2894	0	0,04	0	0	0,96	0	0	0	0	0,01	0,99	0	0	0,028

1	4437	0	0,04	0,02	0	0,94	0	0	0	0	0	0,99	0,01	0	0,032
1	8502	0	0	0,01	0,07	0,9	0	0,02	0	0	0,09	0,01	0,17	0,73	0,042
1	3911	0,01	0,04	0	0	0,93	0	0	0	0,02	0,02	0,91	0	0,07	0,036
1	3190	0	0,04	0	0,06	0,9	0	0	0	0	0	1	0	0	0,048
1	2140	0	0,04	0	0	0,95	0	0	0	0,01	0	0,04	0,95	0,01	0,024
1	4545	0	0	0,01	0	0,54	0	0,1	0,25	0,1	0,11	0,22	0,49	0,18	0,048
1	4338	0	0,04	0	0,01	0,94	0,01	0	0	0	0	0,04	0	0,96	0,018
1	2948	0	0,04	0	0	0,94	0,01	0	0	0,01	0,03	0,02	0,01	0,94	0,012
1	1801	0	0,04	0	0	0,94	0,02	0	0	0	0,02	0,06	0,92	0	0,02
1	1067	0,01	0,04	0	0	0,9	0,03	0	0,02	0	0	0,07	0,91	0,02	0,034
1	7842	0	0	0	0,09	0	0	0	0,91	0	0	0,93	0	0,07	0,048
1	2881	0	0,01	0,01	0,2	0,78	0	0	0	0	0,79	0,01	0	0,2	0,048
1	1808	0	0,04	0	0,02	0,89	0,05	0	0	0	0,65	0,04	0,29	0,02	0,038
1	2146	0	0,04	0	0	0,96	0	0	0	0	0	1	0	0	0,03
1	17304	0	0,04	0	0	0,95	0	0,01	0	0	0	0,05	0,95	0	0,028
1	2721	0	0,04	0	0	0,96	0	0	0	0	0	0,04	0	0,96	0,004
1	1685	0	0,04	0	0	0,93	0	0,01	0,02	0	0	0,04	0,95	0,01	0,03
1	11895	0	0	0	0,11	0,84	0,03	0,02	0	0	0,04	0	0,93	0,03	0,044
1	3016	0	0,04	0	0,05	0,91	0	0	0	0	0	0,04	0,96	0	0,046
1	3748	0,01	0,04	0	0,02	0,93	0	0	0	0	0,36	0,63	0,01	0	0,042
1	2371	0	0,04	0	0	0,95	0	0	0,01	0	0	0,04	0,96	0	0,02
1	2318	0	0,04	0,01	0	0,95	0	0	0	0	0,01	0,04	0	0,95	0,018
1	9635	0	0	0	0,11	0,84	0,05	0	0	0	0,01	0,11	0,84	0,04	0,048
1	2877	0	0,04	0	0,01	0,94	0	0	0,01	0	0	0,98	0,01	0,01	0,048
1	2568	0	0,04	0	0	0,92	0	0,03	0,01	0	0,04	0,04	0,92	0	0,036
1	5491	0	0,04	0,01	0	0,94	0,01	0	0	0	0	0,04	0,02	0,94	0,014
1	2067	0	0,04	0	0,01	0,95	0	0	0	0	0	0,03	0,96	0,01	0,022
1	1856	0	0,04	0	0	0,9	0,06	0	0	0	0	0,06	0,94	0	0,022
1	3714	0	0,04	0	0	0,94	0	0,01	0,01	0	0,01	0,16	0,01	0,82	0,008
1	1452	0	0,04	0	0,01	0,94	0	0,01	0	0	0,04	0,01	0	0,95	0,03
1	1599	0	0,04	0	0	0,96	0	0	0	0	0	0,03	0,96	0,01	0,026
1	1604	0	0,04	0	0,03	0,93	0	0	0	0	0	0,26	0,03	0,71	0,038
1	5567	0	0,04	0	0	0,93	0	0	0,03	0	0,18	0,04	0	0,78	0,014

1	1273	0	0,04	0	0	0,96	0	0	0	0	0	0,04	0,96	0	0,02
1	2160	0	0,04	0	0	0,96	0	0	0	0	0	0,14	0	0,86	0,01
1	2265	0	0,04	0	0	0,96	0	0	0	0	0	0,05	0,95	0	0,03
1	2534	0	0,04	0	0	0,94	0,02	0	0	0	0,93	0,05	0	0,02	0,048
1	12690	0	0,04	0	0	0,92	0,02	0	0	0,02	0,93	0,05	0	0,02	0,046
1	2025	0	0,04	0	0	0,96	0	0	0	0	0	1	0	0	0,032
1	1517	0	0,04	0	0,04	0,71	0,21	0	0	0	0,92	0,08	0	0	0,044
1	3363	0	0,04	0	0	0,91	0,03	0	0	0,02	0,91	0,09	0	0	0,034
1	12101	0	0,04	0	0	0,84	0,12	0	0	0	0,84	0,04	0,12	0	0,04
1	3688	0	0,04	0	0,01	0,93	0,02	0	0	0	0,01	0,04	0,01	0,94	0,024
1	2048	0	0,04	0	0,04	0,92	0	0	0	0	0,04	0,04	0,92	0	0,046
1	5364	0	0,04	0	0	0,94	0	0	0	0,02	0	0,05	0,94	0,01	0,026
1	5565	0	0,04	0,01	0,06	0,37	0,23	0,01	0,28	0	0,33	0,21	0,46	0	0,044
1	2734	0,01	0,04	0	0	0,93	0	0,02	0	0	0	0,05	0,95	0	0,048
1	15304	0	0,04	0	0	0,95	0,01	0	0	0	0	0,05	0,95	0	0,036
1	6190	0	0,04	0	0	0,95	0,01	0	0	0	0,95	0,05	0	0	0,046
1	3271	0	0,04	0	0,01	0,94	0	0,01	0	0	0,79	0,04	0,02	0,15	0,036
1	8446	0	0,02	0	0,11	0,83	0,04	0	0	0	0,13	0,04	0,83	0	0,046
1	2290	0	0,04	0	0,03	0,82	0,11	0	0	0	0,62	0,28	0,1	0	0,048
1	1406	0	0,04	0	0	0,95	0	0	0	0,01	0	0,99	0	0,01	0,026
1	2686	0	0,04	0	0	0,96	0	0	0	0	0	0,04	0,96	0	0,024
1	4445	0	0,04	0	0	0,96	0	0	0	0	0,01	0,04	0,55	0,4	0,01
1	1313	0	0,04	0	0	0,96	0	0	0	0	0	0,04	0,96	0	0,028
1	903	0	0,04	0	0	0,95	0	0	0,01	0	0	0,05	0,95	0	0,024
1	8061	0	0,04	0	0,02	0,9	0	0,01	0,03	0	0,01	0,86	0,03	0,1	0,03
1	2502	0,01	0,03	0	0,07	0,88	0	0	0,01	0	0,01	0,62	0,37	0	0,042
1	2685	0	0,04	0	0	0,95	0	0,01	0	0	0,03	0,04	0,01	0,92	0,024
1	8643	0	0	0	0,39	0,61	0	0	0	0	1	0	0	0	0,044
1	4917	0	0,04	0	0	0,94	0,01	0	0	0,01	0,02	0,04	0,94	0	0,028
1	2212	0	0,04	0	0	0,95	0	0,01	0	0	0	0,04	0,31	0,65	0,012
1	3501	0,01	0,04	0	0	0,93	0,02	0	0	0	0	0,03	0,08	0,89	0,018
1,25	8343	0	0,04	0	0,03	0,82	0,1	0	0,01	0	0,65	0,04	0,13	0,18	0,04
1,25	21598	0	0	0	0,12	0,88	0	0	0	0	0	0,88	0,12	0	0,048

1,25	8517	0,01	0	0	0,27	0,62	0,1	0	0	0	0,99	0	0	0,01	0,048
1,25	5452	0	0,04	0	0,02	0,94	0	0	0	0	0	0,04	0,94	0,02	0,026
1,25	16133	0	0	0	0,14	0,71	0,15	0	0	0	0,15	0,7	0,15	0	0,046
1,25	5998	0	0,04	0	0,03	0,93	0	0	0	0	0,03	0,96	0	0,01	0,038
1,25	2186	0,01	0,04	0	0	0,94	0	0,01	0	0	0	0,04	0,95	0,01	0,042
1,25	1467	0,02	0,04	0	0,01	0,81	0,12	0	0	0	0,66	0,16	0	0,18	0,048
1,25	5420	0	0,04	0,01	0,01	0,93	0	0	0,01	0	0,02	0,98	0	0	0,038
1,25	1697	0	0,04	0	0,01	0,95	0	0	0	0	0	0,04	0,01	0,95	0,016
1,25	9032	0	0,04	0	0,01	0,94	0,01	0	0	0	0	0,98	0,02	0	0,044
1,25	12888	0	0,04	0	0	0,96	0	0	0	0	0,96	0,04	0	0	0,036
1,25	1405	0	0,04	0	0	0,96	0	0	0	0	0,05	0,95	0	0	0,03
1,25	8065	0	0,04	0	0	0,92	0	0,01	0,03	0	0	0,05	0,95	0	0,034
1,25	2835	0	0,04	0,01	0	0,95	0	0	0	0	0,77	0,04	0,18	0,01	0,044
1,25	29525	0	0,01	0	0,25	0,74	0	0	0	0	0,88	0,11	0	0,01	0,04
1,25	4327	0	0,04	0	0	0,96	0	0	0	0	0	0,04	0,96	0	0,026
1,25	12343	0	0	0,1	0,07	0,01	0,05	0	0,72	0,05	0,01	0,02	0,85	0,12	0,046
1,25	12295	0	0,04	0	0,02	0,84	0	0	0,1	0	0	0,14	0,86	0	0,03
1,25	3587	0	0,04	0	0	0,96	0	0	0	0	0,05	0,04	0	0,91	0,006
1,25	1082	0	0,04	0	0	0,85	0,1	0	0	0,01	0,42	0,57	0,01	0	0,034
1,25	25893	0	0,04	0	0	0,92	0	0	0,04	0	0,04	0,04	0	0,92	0,008
1,25	3103	0	0,04	0	0,04	0,9	0,01	0	0,01	0	0,94	0,04	0,01	0,01	0,03
1,25	13701	0	0	0	0,22	0,78	0	0	0	0	0,78	0	0,18	0,04	0,048
1,25	1667	0	0,04	0	0,01	0,93	0,02	0	0	0	0,01	0,19	0,02	0,78	0,022
1,25	12921	0	0,04	0	0	0,95	0	0	0	0,01	0,22	0,05	0	0,73	0,022
1,25	5038	0	0,04	0	0	0	0,01	0	0,81	0,14	0,12	0,69	0	0,19	0,014
1,25	15281	0	0,04	0	0	0,96	0	0	0	0	0	0,14	0	0,86	0,016
1,25	3083	0	0	0,01	0,27	0,72	0	0	0	0	0,73	0,26	0,01	0	0,04
1,25	3044	0	0,04	0	0	0,91	0	0	0,05	0	0	0,95	0,05	0	0,044
1,25	3857	0	0,02	0	0,09	0,86	0,03	0	0	0	0,11	0,58	0	0,31	0,034
1,25	3058	0	0,04	0	0	0,96	0	0	0	0	0	0,88	0,08	0,04	0,02
1,25	1813	0	0,04	0	0	0,96	0	0	0	0	0	0,04	0,96	0	0,034
1,25	5604	0	0,04	0	0	0,95	0	0,01	0	0	0,02	0,97	0	0,01	0,02
1,25	10885	0	0,04	0	0	0,96	0	0	0	0	0	0,04	0	0,96	0,012

1,25	5320	0	0,04	0	0	0,96	0	0	0	0	0,03	0,01	0,96	0	0,022
1,25	4247	0	0,04	0	0	0,96	0	0	0	0	0	0,04	0	0,96	0,016
1,25	1680	0	0,04	0	0	0,96	0	0	0	0	0	0,96	0	0,04	0,014
1,25	1153	0	0,04	0	0,04	0,91	0	0	0	0,01	0,53	0,04	0,01	0,42	0,042
1,25	18677	0	0,02	0	0,09	0,89	0	0	0	0	0,02	0	0,98	0	0,04
1,25	1632	0	0,04	0	0,01	0,91	0,02	0	0,02	0	0,04	0,05	0,91	0	0,02
1,25	18468	0,01	0,04	0	0	0,93	0,01	0,01	0	0	0,04	0,01	0,02	0,93	0,02
1,25	5448	0	0,04	0	0	0,91	0,05	0	0	0	1	0	0	0	0,036
1,25	8559	0	0,04	0	0	0	0	0,01	0,95	0	0	0,05	0	0,95	0,024
1,25	5985	0	0,04	0	0	0,96	0	0	0	0	0	0,53	0,47	0	0,026
1,25	1853	0	0,04	0	0	0,96	0	0	0	0	0	1	0	0	0,016
1,25	2370	0	0,04	0	0,11	0,83	0,02	0	0	0	0,82	0,06	0,01	0,11	0,042
1,25	5471	0	0,04	0	0	0,94	0	0,02	0	0	0,94	0,04	0,02	0	0,038
1,25	2681	0	0,04	0	0,01	0,95	0	0	0	0	0	0,04	0	0,96	0,006
1,25	2087	0	0,04	0	0	0,96	0	0	0	0	0	0,04	0	0,96	0,008
1,25	3705	0	0,01	0	0,14	0,8	0,02	0,02	0	0,01	0,8	0,03	0,16	0,01	0,048
1,25	2175	0	0,02	0	0,13	0,65	0	0	0,2	0	0	1	0	0	0,048
1,25	14819	0	0,04	0	0	0,94	0	0	0,01	0,01	0,01	0,05	0,94	0	0,014
1,25	21205	0	0,04	0	0,03	0,93	0	0	0	0	0,93	0,03	0,01	0,03	0,048
1,25	6968	0	0,04	0,01	0	0,91	0,04	0	0	0	0,88	0,08	0,04	0	0,036
1,25	11958	0	0	0	0,15	0,84	0	0	0	0,01	0,16	0	0,84	0	0,048
1,25	15250	0	0,04	0,01	0	0,95	0	0	0	0	0	0,04	0	0,96	0,016
1,25	1732	0	0,04	0	0	0,93	0,01	0,01	0,01	0	0,01	0,98	0	0,01	0,034
1,25	7743	0	0,03	0,01	0,12	0,78	0,01	0	0,05	0	0,79	0,07	0	0,14	0,044
1,25	5321	0	0,04	0	0,04	0,92	0	0	0	0	0,92	0,04	0	0,04	0,042
1,25	9264	0	0	0	0,14	0,85	0	0	0	0,01	0,14	0,85	0,01	0	0,046
1,25	4265	0	0	0	0,22	0,78	0	0	0	0	0,78	0,19	0	0,03	0,046
1,25	6648	0	0,04	0	0	0,96	0	0	0	0	0	0,04	0	0,96	0,014
1,25	2740	0	0,04	0	0,01	0,91	0	0,01	0,03	0	0,92	0,04	0,04	0	0,046
1,25	2979	0	0,04	0,01	0,01	0,88	0,05	0	0,01	0	0	0,96	0,01	0,03	0,04
1,25	2106	0	0,04	0	0,01	0,93	0	0	0,02	0	0	0,04	0,94	0,02	0,026
1,25	3069	0	0,04	0	0	0,96	0	0	0	0	0,88	0,11	0,01	0	0,038
1,25	3992	0	0,04	0,01	0	0,95	0	0	0	0	0,01	0,04	0	0,95	0,022

1,25	16944	0	0	0	0,08	0,92	0	0	0	0	0	0,08	0	0,92	0,048
1,25	3567	0	0,04	0	0,01	0,9	0	0	0,05	0	0,06	0,92	0	0,02	0,018
1,25	1150	0	0,04	0,01	0	0,95	0	0	0	0	0,95	0,05	0	0	0,044
1,25	3206	0	0,04	0	0	0,93	0,03	0	0	0	0,95	0,05	0	0	0,04
1,25	2187	0	0,04	0	0	0,96	0	0	0	0	0,96	0,02	0,02	0	0,032
1,25	12425	0	0,04	0	0,01	0,94	0,01	0	0	0	0,9	0,05	0	0,05	0,044
1,25	5979	0	0,04	0	0	0,95	0	0,01	0	0	0,96	0,04	0	0	0,038
1,25	2648	0,01	0,04	0	0	0,93	0	0,01	0,01	0	0,01	0,04	0,95	0	0,044
1,25	2652	0	0,04	0	0,02	0,85	0,09	0	0	0	0,86	0,08	0,04	0,02	0,04
1,25	8519	0	0,04	0	0	0,95	0	0	0,01	0	0	0,99	0,01	0	0,028
1,25	1652	0	0,04	0	0	0,94	0,01	0,01	0	0	0,94	0,05	0,01	0	0,04
1,25	4546	0	0	0	0,41	0,58	0,01	0	0	0	0,98	0,02	0	0	0,048
1,25	2228	0	0,04	0	0	0,95	0	0	0,01	0	0	0,04	0,95	0,01	0,028
1,25	6743	0	0,04	0,01	0	0,94	0,01	0	0	0	0,94	0,05	0	0,01	0,048
1,25	9160	0	0,04	0	0	0,96	0	0	0	0	0	0,04	0	0,96	0,008
1,25	2547	0	0,04	0	0,01	0,93	0	0	0,02	0	0	0,07	0,93	0	0,022
1,25	7478	0	0	0	0,13	0,87	0	0	0	0	0,06	0	0,9	0,04	0,042
1,25	1624	0	0,04	0	0	0,93	0	0	0,02	0,01	0,01	0,24	0,73	0,02	0,018
1,25	1373	0	0,04	0	0	0,95	0	0	0,01	0	0	0,04	0,96	0	0,034
1,25	10364	0	0	0,01	0,08	0,91	0	0	0	0	0,06	0,32	0	0,62	0,046
1,25	1200	0	0,04	0	0,01	0,95	0	0	0	0	0,94	0,05	0	0,01	0,038
1,25	3752	0,01	0,04	0	0	0,95	0	0	0	0	0	0,99	0	0,01	0,032
1,25	8018	0	0,04	0	0	0,95	0	0	0,01	0	0,01	0,05	0,94	0	0,028
1,25	1561	0	0,04	0	0	0,96	0	0	0	0	0,39	0,02	0	0,59	0,016
1,25	3497	0	0,04	0	0	0,95	0	0	0	0,01	0,07	0,04	0	0,89	0,018
1,25	4223	0	0	0	0,12	0,82	0,05	0	0	0,01	0,12	0	0,82	0,06	0,042
1,25	1700	0	0,04	0	0,01	0,95	0	0	0	0	0	0,04	0,96	0	0,036
1,25	5285	0	0	0	0,1	0,89	0,01	0	0	0	0	0,89	0	0,11	0,048
1,25	2296	0	0,04	0	0,03	0,93	0	0	0	0	0	0,97	0	0,03	0,042
1,25	2364	0	0	0	0,11	0,88	0	0,01	0	0	0,11	0,01	0,6	0,28	0,048
1,25	11584	0	0	0	0,07	0,91	0,01	0	0	0,01	0,02	0,07	0	0,91	0,042
1,25	1170	0	0,04	0	0	0,95	0	0,01	0	0	0,77	0,21	0	0,02	0,03
1,5	11324	0	0,04	0	0,06	0,9	0	0	0	0	0	0,04	0,96	0	0,042

1,5	8199	0	0,04	0	0	0,96	0	0	0	0	0	0,04	0	0,96	0,02
1,5	9657	0	0,04	0	0	0,96	0	0	0	0	0	0,04	0,01	0,95	0,018
1,5	10243	0	0	0	0,11	0,83	0,06	0	0	0	0	0,83	0,11	0,06	0,044
1,5	3954	0,01	0,03	0	0,09	0,87	0	0	0	0	0,87	0,13	0	0	0,04
1,5	37972	0	0,04	0	0	0,95	0	0,01	0	0	0	0	0,04	0,96	0,01
1,5	5389	0,01	0,04	0	0,03	0,92	0	0	0	0	0,01	0,04	0,95	0	0,046
1,5	2463	0	0,04	0	0	0,91	0	0	0,05	0	0	0,06	0,91	0,03	0,028
1,5	4269	0	0,04	0	0	0,93	0,03	0	0	0	0	1	0	0	0,022
1,5	2270	0	0,04	0	0	0,95	0	0	0	0,01	0,85	0,04	0	0,11	0,038
1,5	5929	0	0,04	0,01	0	0,95	0	0	0	0	0,01	0,04	0,29	0,66	0,022
1,5	2987	0	0,04	0	0	0,96	0	0	0	0	0	0,07	0,91	0,02	0,02
1,5	2779	0	0,04	0	0,02	0,94	0	0	0	0	0,01	0,04	0,01	0,94	0,024
1,5	16314	0	0,04	0,05	0	0,86	0,01	0,01	0,03	0	0,05	0,08	0,87	0	0,046
1,5	10450	0	0,01	0	0,13	0,86	0	0	0	0	0,14	0	0,84	0,02	0,04
1,5	2785	0	0,04	0	0,01	0,92	0,01	0	0,02	0	0	0,05	0,92	0,03	0,038
1,5	12505	0,01	0,03	0	0,05	0,88	0	0,01	0,01	0,01	0,01	0,04	0,07	0,88	0,036
1,5	18037	0	0,04	0	0,02	0,91	0	0	0,03	0	0,02	0,07	0	0,91	0,024
1,5	5732	0	0,04	0	0,13	0,81	0	0	0,02	0	0,81	0,04	0,15	0	0,046
1,5	2305	0,01	0,04	0	0,02	0,92	0,01	0	0	0	0,02	0,95	0,03	0	0,04
1,5	11347	0	0,04	0	0	0,95	0,01	0	0	0	0,95	0,04	0,01	0	0,048
1,5	8293	0	0,04	0	0	0,95	0,01	0	0	0	0	0,05	0	0,95	0,004
1,5	5663	0,03	0	0,02	0	0,87	0,03	0,05	0	0	0	0,92	0,05	0,03	0,048
1,5	21903	0	0	0	0,24	0,56	0,17	0	0	0,03	0,59	0,17	0,24	0	0,048
1,5	2757	0	0,04	0	0	0,95	0	0	0	0,01	0	0,05	0	0,95	0,014
1,5	1367	0	0,03	0,02	0,11	0,84	0	0	0	0	0,01	0,98	0	0,01	0,04
1,5	9961	0	0	0	0,07	0	0	0	0,93	0	0	0,06	0	0,94	0,044
1,5	5704	0	0,04	0	0	0,94	0	0	0,01	0,01	0,01	0,04	0,94	0,01	0,012
1,5	13396	0	0,04	0	0	0,96	0	0	0	0	0,95	0,05	0	0	0,04
1,5	11299	0	0	0	0,1	0,89	0	0,01	0	0	0	0,1	0,89	0,01	0,048
1,5	15435	0	0	0	0,13	0,85	0,02	0	0	0	0,14	0	0,86	0	0,046
1,5	7936	0	0,04	0	0	0,95	0	0	0	0,01	0,01	0	0	0,99	0,01
1,5	34732	0,01	0,04	0	0	0,94	0	0,01	0	0	0	0,05	0,94	0,01	0,044
1,5	2772	0	0,04	0	0	0,96	0	0	0	0	0	1	0	0	0,022

1,5	2748	0	0,04	0	0	0,96	0	0	0	0	0	0,87	0,13	0	0,018
1,5	8923	0	0,03	0,01	0,09	0,87	0	0	0	0	0,05	0,86	0,09	0	0,048
1,5	2203	0	0,04	0,01	0	0,93	0	0	0,01	0,01	0	0,05	0,01	0,94	0,008
1,5	13719	0	0	0	0,11	0,59	0,29	0	0,01	0	0,02	0,04	0,65	0,29	0,046
1,5	2003	0,02	0,01	0	0,09	0,82	0	0	0,06	0	0,06	0,89	0,03	0,02	0,044
1,5	16515	0	0,04	0	0	0,96	0	0	0	0	0,21	0,04	0,75	0	0,022
1,5	1750	0	0,04	0,01	0	0,95	0	0	0	0	0	0,04	0,01	0,95	0,014
1,5	1674	0	0,04	0	0	0,87	0,08	0	0	0,01	0,01	0,04	0,87	0,08	0,022
1,5	15652	0	0	0,01	0,07	0,92	0	0	0	0	0	0,07	0	0,93	0,046
1,5	2175	0,03	0,04	0	0	0,92	0	0	0	0,01	0	1	0	0	0,04
1,5	32238	0	0,04	0	0	0,95	0	0,01	0	0	0	0,04	0,96	0	0,024
1,5	5576	0	0,04	0	0	0,95	0	0	0,01	0	0,56	0,07	0,37	0	0,04
1,5	6004	0	0,04	0	0	0,92	0,01	0,03	0	0	0,12	0,02	0,86	0	0,048
1,5	38599	0	0,04	0	0,02	0,94	0	0	0	0	0	0,04	0,96	0	0,048
1,5	2022	0,02	0,04	0	0	0,91	0	0	0,01	0,02	0,91	0,06	0,01	0,02	0,048
1,5	4339	0	0,04	0	0	0,96	0	0	0	0	0	0,02	0,98	0	0,024
1,5	2989	0	0,04	0	0,01	0,95	0	0	0	0	0,01	0,16	0,82	0,01	0,032
1,5	11281	0	0	0	0,32	0,47	0,2	0,01	0	0	0,79	0,2	0,01	0	0,044
1,5	23870	0	0	0,01	0,18	0,77	0	0,01	0,03	0	0,78	0,01	0,2	0,01	0,038
1,5	14039	0,03	0,04	0	0,02	0,9	0,01	0	0	0	0	0,94	0,06	0	0,048
1,5	5723	0	0,04	0	0	0,95	0	0	0,01	0	0	0,04	0	0,96	0,008
1,5	16104	0	0	0	0,12	0,61	0,26	0	0	0,01	0,09	0,18	0,47	0,26	0,048
1,5	24117	0	0	0	0,07	0,92	0	0,01	0	0	0,01	0	0,07	0,92	0,046
1,5	18681	0	0,04	0	0	0,96	0	0	0	0	0,25	0,04	0,71	0	0,024
1,5	1990	0,01	0,04	0	0,09	0,82	0	0	0,04	0	0,86	0,05	0	0,09	0,048
1,5	6489	0	0	0	0,14	0,86	0	0	0	0	0,14	0	0,86	0	0,046
1,5	1487	0	0,04	0	0	0,94	0,02	0	0	0	0	0,04	0,63	0,33	0,026
1,5	4728	0	0,04	0	0	0,94	0	0	0,02	0	0	0,06	0,92	0,02	0,028
1,5	2020	0	0,04	0	0,08	0,86	0,01	0	0	0,01	0	0,99	0,01	0	0,04
1,5	18425	0	0,04	0	0	0	0,02	0	0,94	0	0,02	0	0	0,98	0,01
1,5	20703	0,01	0,04	0	0	0,94	0,01	0	0	0	0	0,06	0,94	0	0,034
1,5	44413	0	0,01	0	0,14	0,84	0	0	0,01	0	0,14	0,84	0,01	0,01	0,048
1,5	3087	0	0,04	0	0	0,94	0,01	0	0,01	0	0	0,05	0,94	0,01	0,022

1,5	1501	0	0,04	0	0	0,91	0,05	0	0	0	0,95	0,04	0	0,01	0,038
1,5	16434	0	0	0	0,12	0,74	0,14	0	0	0	0	0,12	0,84	0,04	0,046
1,5	5262	0	0,04	0	0	0,96	0	0	0	0	0,01	0,19	0	0,8	0,01
1,5	13079	0	0,04	0	0	0,96	0	0	0	0	0	0,04	0	0,96	0,012
1,5	9690	0,01	0,04	0,03	0	0,88	0,04	0	0	0	0,17	0,68	0,15	0	0,048
1,5	17093	0	0,01	0	0,11	0,87	0	0,01	0	0	0	0,07	0,91	0,02	0,048
1,5	1340	0	0,04	0	0	0,96	0	0	0	0	0	1	0	0	0,018
1,5	2437	0	0,04	0	0	0,96	0	0	0	0	0,02	0,02	0	0,96	0,016
1,5	8647	0	0	0	0,06	0,91	0	0,01	0	0,02	0,09	0	0	0,91	0,044
1,5	10148	0	0,04	0	0	0,96	0	0	0	0	0	1	0	0	0,032
1,5	14093	0	0,04	0	0,02	0,91	0	0	0,03	0	0	0,95	0,02	0,03	0,036
1,5	7268	0	0,04	0	0	0,96	0	0	0	0	0,54	0,45	0	0,01	0,044
1,5	9594	0	0	0,01	0,12	0,85	0,02	0	0	0	0,16	0,71	0,13	0	0,048
1,5	12152	0	0,04	0	0	0,96	0	0	0	0	0	0,04	0,04	0,92	0,014
1,5	7554	0	0,04	0	0	0,96	0	0	0	0	0,03	0,9	0	0,07	0,028
1,5	8453	0	0,04	0,01	0	0,76	0,19	0	0	0	0,79	0,19	0	0,02	0,042
1,5	1677	0	0,04	0	0	0,96	0	0	0	0	0,96	0,04	0	0	0,034
1,5	2533	0	0,04	0	0	0,95	0	0	0,01	0	0,95	0,04	0,01	0	0,042
1,5	7133	0	0,04	0	0	0,74	0,19	0	0,02	0,01	0,03	0,76	0,19	0,02	0,022
1,5	18089	0	0	0	0,09	0,4	0,32	0	0,19	0	0,21	0,32	0,1	0,37	0,048
1,5	988	0	0,04	0	0	0,96	0	0	0	0	0	0,04	0,96	0	0,02
1,5	22604	0	0,04	0	0,1	0,84	0,02	0	0	0	0,84	0,02	0,14	0	0,048
1,5	3238	0	0,04	0	0	0,96	0	0	0	0	0	0,04	0	0,96	0,006
1,5	8525	0,01	0,04	0	0	0,93	0	0	0,02	0	0,01	0,04	0,02	0,93	0,022
1,5	5496	0	0,04	0	0,05	0,91	0	0	0	0	0,04	0,96	0	0	0,038
1,5	41791	0	0,04	0	0	0,95	0,01	0	0	0	0,01	0,08	0,91	0	0,026
1,5	24460	0	0,03	0,04	0	0,5	0,05	0	0,38	0	0,13	0,08	0,79	0	0,048
1,5	1771	0	0,04	0	0	0,94	0,01	0,01	0	0	0,01	0,04	0,94	0,01	0,026
1,5	47740	0	0	0	0,11	0,75	0,12	0	0	0,02	0,24	0,01	0	0,75	0,048
1,5	12532	0	0,04	0	0	0,96	0	0	0	0	0,01	0,03	0	0,96	0,01
1,5	4022	0	0	0,02	0,27	0,1	0,6	0,01	0	0	0,87	0,11	0	0,02	0,048
1,5	20989	0,02	0,04	0	0,01	0,92	0	0,01	0	0	0,94	0,05	0,01	0	0,04
1,5	4394	0	0,01	0	0,09	0,79	0,01	0	0,08	0,02	0,09	0,02	0,1	0,79	0,048

1,75	25181	0	0	0	0,25	0	0,74	0,01	0	0	0,86	0,14	0	0	0,048
1,75	19988	0	0	0	0,13	0,87	0	0	0	0	0,15	0,72	0,13	0	0,048
1,75	27144	0	0,04	0	0	0,95	0	0	0,01	0	0	0,04	0,95	0,01	0,018
1,75	24233	0	0,04	0	0	0,96	0	0	0	0	0	0,02	0,98	0	0,016
1,75	3917	0	0,04	0	0	0,85	0	0,02	0,08	0,01	0,02	0,04	0,09	0,85	0,036
1,75	25821	0	0	0	0,15	0,85	0	0	0	0	0,15	0,85	0	0	0,048
1,75	7949	0	0	0	0,23	0,77	0	0	0	0	0,77	0	0,23	0	0,036
1,75	26954	0	0,04	0	0	0,96	0	0	0	0	0,3	0,04	0	0,66	0,016
1,75	1604	0	0,04	0	0,02	0,9	0	0	0,03	0,01	0	0,04	0,94	0,02	0,03
1,75	27198	0	0,04	0	0,06	0,01	0	0	0,86	0,03	0,03	0,06	0,85	0,06	0,046
1,75	35062	0	0,04	0	0,01	0,83	0,12	0	0	0	0	0,04	0,83	0,13	0,028
1,75	2292	0	0,04	0	0	0,96	0	0	0	0	0,21	0,04	0	0,75	0,02
1,75	25923	0	0,04	0	0	0,96	0	0	0	0	0	0,04	0,96	0	0,044
1,75	2084	0	0,04	0	0,01	0,95	0	0	0	0	0,12	0,03	0,01	0,84	0,024
1,75	13384	0	0	0	0,11	0,86	0,02	0,01	0	0	0	0	0,96	0,04	0,046
1,75	5216	0	0,02	0	0,11	0,78	0,09	0	0	0	0,03	0,8	0,11	0,06	0,046
1,75	31997	0	0,04	0,01	0	0,95	0	0	0	0	0,03	0,04	0,01	0,92	0,022
1,75	1764	0	0,04	0	0	0,96	0	0	0	0	0,27	0,37	0,36	0	0,02
1,75	15841	0	0,04	0	0	0,96	0	0	0	0	0	0,04	0,25	0,71	0,022
1,75	14319	0	0,04	0	0	0,96	0	0	0	0	0	0,04	0,96	0	0,032
1,75	10105	0	0,04	0	0	0,96	0	0	0	0	0	0,04	0,95	0,01	0,028
1,75	30036	0	0,04	0	0	0,94	0	0	0	0,02	0,94	0,06	0	0	0,032
1,75	8759	0	0,04	0	0	0,95	0,01	0	0	0	0	0,99	0	0,01	0,022
1,75	9425	0	0,04	0	0,03	0,92	0	0	0	0,01	0,01	0,96	0	0,03	0,036
1,75	14172	0	0	0	0,14	0,84	0,02	0	0	0	0,12	0,16	0,72	0	0,04
1,75	19453	0	0,01	0	0,12	0,81	0,06	0	0	0	0,12	0	0,82	0,06	0,04
1,75	1083	0	0,04	0	0,02	0,94	0	0	0	0	0,02	0,21	0,75	0,02	0,028
1,75	6799	0	0,04	0	0	0,96	0	0	0	0	0	0,03	0	0,97	0,014
1,75	14499	0	0,03	0,02	0,09	0,85	0,01	0	0	0	0	0,88	0,11	0,01	0,044
1,75	19326	0	0	0	0,09	0,9	0	0	0	0,01	0,09	0	0,01	0,9	0,048
1,75	1749	0	0,04	0,01	0	0,86	0,09	0	0	0	0,95	0,04	0,01	0	0,042
1,75	7052	0	0,04	0	0	0,96	0	0	0	0	0	0,04	0	0,96	0,006
1,75	17001	0	0,04	0	0,01	0,95	0	0	0	0	0	0,05	0,95	0	0,032

1,75	10749	0	0,01	0	0,25	0	0,74	0	0	0	0,76	0,24	0	0	0,042
1,75	24046	0	0,04	0	0	0,95	0,01	0	0	0	0,01	0	0	0,99	0,01
1,75	32090	0	0,04	0	0,08	0,87	0	0	0	0,01	0,09	0,02	0,89	0	0,042
1,75	4689	0	0,04	0	0	0,95	0	0	0	0,01	0,95	0,04	0	0,01	0,034
1,75	11217	0	0,04	0	0	0,96	0	0	0	0	0,96	0,03	0	0,01	0,034
1,75	8791	0	0,04	0	0	0,95	0	0	0	0,01	0,02	0,96	0,02	0	0,032
1,75	6536	0	0	0	0,27	0,73	0	0	0	0	0,77	0	0,23	0	0,046
1,75	39048	0	0,04	0	0	0,95	0	0	0,01	0	0,55	0,03	0,42	0	0,042
1,75	2365	0	0,04	0	0	0,96	0	0	0	0	0,01	0,04	0,95	0	0,022
1,75	13087	0	0,04	0	0	0,96	0	0	0	0	0	0,27	0,69	0,04	0,036
1,75	12247	0	0,01	0	0,06	0,91	0	0	0,02	0	0,05	0,01	0,03	0,91	0,038
1,75	45518	0	0,04	0	0	0,95	0,01	0	0	0	0	0,22	0,77	0,01	0,026
1,75	29153	0	0,04	0	0,07	0,88	0	0	0	0,01	0,02	0,92	0,06	0	0,046
1,75	19195	0	0,04	0	0	0,96	0	0	0	0	0	0,04	0,96	0	0,02
1,75	2645	0	0,04	0	0	0,95	0	0	0,01	0	0	0,05	0,95	0	0,03
1,75	8079	0,01	0	0	0,09	0,9	0	0	0	0	0	0,99	0	0,01	0,048
1,75	16021	0	0,04	0	0	0,96	0	0	0	0	0	0,04	0,96	0	0,024
1,75	27215	0	0	0	0,06	0	0,9	0	0,04	0	0,06	0	0,04	0,9	0,044
1,75	30043	0,01	0	0	0,15	0,83	0	0,01	0	0	0,15	0,83	0,01	0,01	0,048
1,75	13085	0	0,04	0	0,06	0,9	0	0	0	0	0	0,94	0,06	0	0,038
1,75	9115	0	0	0	0,08	0,92	0	0	0	0	0,08	0	0	0,92	0,044
1,75	3753	0	0,04	0	0,02	0,92	0,02	0	0	0	0	0,08	0,92	0	0,032
1,75	10832	0	0,04	0	0	0,93	0,02	0	0	0,01	0	0,04	0,95	0,01	0,022
1,75	4937	0	0,04	0	0	0,94	0	0	0,01	0,01	0,92	0,04	0	0,04	0,048
1,75	2278	0	0,04	0	0	0,95	0	0,01	0	0	0,01	0,04	0,91	0,04	0,034
1,75	11110	0	0,04	0	0	0,93	0	0	0,01	0,02	0,93	0,07	0	0	0,034
1,75	19982	0	0,04	0	0	0,96	0	0	0	0	0	0,07	0,77	0,16	0,016
1,75	28020	0,01	0,04	0,01	0	0,89	0	0	0,04	0,01	0,03	0,06	0,89	0,02	0,04
1,75	16136	0	0,03	0	0,05	0,9	0	0	0	0,02	0	0,02	0,08	0,9	0,048
1,75	4711	0	0,04	0	0,03	0,33	0	0	0,6	0	0,33	0,07	0	0,6	0,042
1,75	2213	0,01	0,04	0	0	0,9	0	0	0,05	0	0	0,04	0,06	0,9	0,02
1,75	3301	0	0,04	0	0,01	0,94	0	0,01	0	0	0,01	0,04	0,01	0,94	0,014
1,75	22374	0	0,04	0	0	0,95	0	0	0	0,01	0,95	0,05	0	0	0,04

1,75	1543	0	0,04	0	0	0,96	0	0	0	0	0	0,04	0	0,96	0,008
1,75	2355	0	0,04	0	0,04	0,92	0	0	0	0	0,92	0,01	0,03	0,04	0,046
1,75	7416	0	0,04	0	0	0,96	0	0	0	0	0,04	0	0,96	0	0,022
1,75	13295	0,01	0,04	0	0	0,94	0	0	0	0,01	0	0,01	0	0,99	0,02
1,75	9912	0	0,04	0	0	0,94	0,01	0,01	0	0	0	0,05	0	0,95	0,014
1,75	10139	0	0,04	0	0	0,96	0	0	0	0	0	0,04	0,96	0	0,028
1,75	14667	0	0,04	0	0	0,96	0	0	0	0	0	0,96	0	0,04	0,02
1,75	26140	0	0	0	0,3	0,7	0	0	0	0	0,96	0	0	0,04	0,048
1,75	11976	0	0,04	0	0,01	0,94	0	0	0	0,01	0,01	0,04	0,94	0,01	0,03
1,75	37692	0	0,04	0	0	0	0,96	0	0	0	0,99	0	0,01	0	0,036
1,75	2499	0	0	0	0,24	0,74	0,02	0	0	0	0,81	0	0,19	0	0,046
1,75	22933	0	0	0	0,27	0,72	0	0	0,01	0	0,73	0,27	0	0	0,048
1,75	22034	0	0,04	0	0	0,95	0	0	0	0,01	0	0,96	0,03	0,01	0,03
1,75	1370	0	0,03	0,04	0	0,85	0	0,01	0,07	0	0,88	0,07	0,01	0,04	0,036
1,75	7736	0	0,04	0	0	0,95	0	0	0	0,01	0	0,05	0	0,95	0,01
1,75	5854	0	0,04	0	0,06	0,86	0	0	0,04	0	0,03	0,04	0	0,93	0,048
1,75	9904	0	0,04	0,01	0	0,94	0	0	0	0,01	0,01	0,04	0,94	0,01	0,024
1,75	7470	0	0,04	0	0,02	0,93	0	0,01	0	0	0,03	0,02	0,02	0,93	0,026
1,75	16594	0	0,04	0	0	0,94	0	0	0	0,02	0	0,04	0,94	0,02	0,03
1,75	1592	0	0,04	0	0,02	0,92	0	0	0,02	0	0	0,04	0,08	0,88	0,032
1,75	2975	0	0,04	0	0,02	0,87	0,05	0,01	0,01	0	0,94	0,05	0	0,01	0,034
1,75	21602	0	0,04	0,01	0	0,95	0	0	0	0	0	0,89	0,06	0,05	0,038
1,75	40005	0	0,04	0	0	0,94	0	0	0,02	0	0	0,05	0,94	0,01	0,018
1,75	7989	0	0,04	0	0	0,95	0	0,01	0	0	0,01	0,04	0,06	0,89	0,022
1,75	2392	0	0,04	0	0	0,95	0	0	0	0,01	0	0,04	0,01	0,95	0,016
1,75	8849	0	0,04	0	0,05	0,91	0	0	0	0	0,91	0,04	0	0,05	0,04
1,75	11508	0	0	0	0,12	0,88	0	0	0	0	0	0,01	0,99	0	0,048
1,75	17115	0	0,04	0	0	0,96	0	0	0	0	0	0,04	0	0,96	0,008
1,75	4625	0	0,04	0	0	0,91	0,05	0	0	0	0,91	0,01	0,08	0	0,036
1,75	25162	0	0,04	0	0	0,96	0	0	0	0	0	0,12	0,88	0	0,024
1,75	50452	0	0,04	0	0	0,94	0	0	0,01	0,01	0,01	0,04	0,04	0,91	0,01
1,75	11828	0	0,04	0	0,01	0,92	0,01	0,02	0	0	0,01	0,97	0	0,02	0,038
1,75	2468	0	0,04	0	0	0,96	0	0	0	0	0	0,95	0	0,05	0,02

1,75	3776	0	0,02	0	0,1	0,85	0,01	0,02	0	0	0,18	0,7	0,11	0,01	0,046
2	2514	0	0,04	0	0	0,94	0	0	0,02	0	0,06	0	0	0,94	0,02
2	42561	0,01	0	0	0,14	0,73	0,12	0	0	0	0,21	0	0,78	0,01	0,046
2	1849	0	0,04	0	0,02	0,9	0,03	0,01	0	0	0,95	0,05	0	0	0,03
2	20900	0	0,01	0	0,1	0,84	0,05	0	0	0	0,02	0,94	0,01	0,03	0,042
2	32704	0	0,04	0	0	0,78	0	0	0,18	0	0	0	0	1	0,004
2	32255	0	0,04	0	0	0,95	0	0	0	0,01	0,01	0,03	0,95	0,01	0,028
2	36018	0	0,04	0	0	0,95	0	0,01	0	0	0,01	0,04	0,95	0	0,028
2	6619	0,01	0,04	0	0,01	0,94	0	0	0	0	0,01	0,04	0,01	0,94	0,022
2	19318	0	0	0,02	0,11	0,76	0	0	0	0,11	0,03	0,84	0,11	0,02	0,044
2	34375	0,01	0,04	0	0	0,94	0	0,01	0	0	0,94	0,04	0,01	0,01	0,044
2	3888	0	0,04	0,01	0,03	0,87	0,02	0	0	0,03	0	0,05	0,93	0,02	0,032
2	9797	0	0	0	0,36	0,64	0	0	0	0	1	0	0	0	0,048
2	5592	0,01	0,04	0	0	0,95	0	0	0	0	0	0,05	0	0,95	0,034
2	1756	0	0,04	0	0	0,96	0	0	0	0	0,96	0,04	0	0	0,048
2	2587	0	0,04	0	0	0,92	0	0	0,04	0	0	0,04	0	0,96	0,012
2	26150	0	0,04	0,01	0,01	0,94	0	0	0	0	0	0,05	0,95	0	0,032
2	15694	0	0,04	0	0	0,96	0	0	0	0	0,95	0,05	0	0	0,034
2	2756	0	0,04	0	0,02	0,94	0	0	0	0	0,02	0,04	0,94	0	0,038
2	2860	0	0,04	0	0,05	0,85	0,06	0	0	0	0	0,04	0,05	0,91	0,044
2	11880	0,02	0,04	0	0	0,94	0	0	0	0	0	0,04	0	0,96	0,024
2	15313	0	0,04	0	0	0,96	0	0	0	0	0	0,04	0	0,96	0,016
2	19078	0	0,04	0	0	0,95	0,01	0	0	0	0,95	0,04	0,01	0	0,036
2	69025	0	0,04	0	0	0,96	0	0	0	0	0	0,04	0,96	0	0,034
2	15398	0	0,04	0	0	0,95	0,01	0	0	0	0,95	0,05	0	0	0,03
2	15735	0	0	0	0,09	0,91	0	0	0	0	0,09	0	0,31	0,6	0,04
2	51944	0	0,04	0	0	0,96	0	0	0	0	0	0,02	0,96	0,02	0,028
2	3882	0	0	0	0,1	0,88	0	0,02	0	0	0,02	0,1	0,88	0	0,044
2	21328	0	0,04	0	0	0,96	0	0	0	0	0	0,04	0	0,96	0,01
2	5621	0	0	0	0,11	0,86	0	0,01	0,02	0	0	0,86	0,12	0,02	0,048
2	30506	0,01	0,04	0	0	0,94	0,01	0	0	0	0,95	0,02	0,01	0,02	0,03
2	7487	0	0,04	0,02	0,04	0,9	0	0	0	0	0	0,04	0,96	0	0,03
2	14989	0	0,04	0	0	0,96	0	0	0	0	0	0,03	0,09	0,88	0,016

2	13066	0	0,04	0	0	0,95	0	0,01	0	0	0,01	0,04	0	0,95	0,014
2	46966	0	0,04	0	0	0,38	0,58	0	0	0	0,04	0	0,96	0	0,026
2	37094	0	0	0,04	0,08	0,01	0	0,01	0,86	0	0	0,42	0,57	0,01	0,048
2	61388	0	0	0	0,47	0,53	0	0	0	0	1	0	0	0	0,046
2	10366	0	0,04	0	0	0,96	0	0	0	0	0	0,96	0	0,04	0,028
2	15100	0	0	0	0,14	0,86	0	0	0	0	0,14	0,86	0	0	0,044
2	50247	0,01	0,04	0	0	0,95	0	0	0	0	0,95	0	0,02	0,03	0,034
2	4234	0	0,04	0	0	0,96	0	0	0	0	0	0,04	0,96	0	0,03
2	33437	0,01	0,04	0	0	0,95	0	0	0	0	0	0,06	0,93	0,01	0,032
2	1017	0	0,04	0	0,02	0,94	0	0	0	0	0,42	0,01	0,54	0,03	0,04
2	11578	0	0,04	0	0,03	0,88	0,05	0	0	0	0	0,04	0,91	0,05	0,038
2	3814	0,01	0	0	0,1	0,85	0,01	0	0	0,03	0,04	0,95	0	0,01	0,048
2	28693	0,02	0	0	0,11	0,59	0,24	0	0	0,04	0,41	0	0,59	0	0,042
2	1592	0	0,04	0,01	0	0,95	0	0	0	0	0	0,04	0,95	0,01	0,034
2	34416	0	0,04	0	0	0,94	0	0,02	0	0	0	0,06	0,94	0	0,024
2	4049	0	0,04	0	0	0,96	0	0	0	0	0	0,02	0,97	0,01	0,026
2	13792	0	0	0	0,07	0,87	0,03	0,01	0,02	0	0,03	0,02	0,01	0,94	0,044
2	1514	0	0,04	0	0,1	0,86	0	0	0	0	0,86	0,04	0,1	0	0,04
2	38406	0	0,04	0	0	0,96	0	0	0	0	0,96	0,03	0	0,01	0,038
2	2144	0	0,03	0	0,19	0,63	0,14	0	0	0,01	0,78	0,18	0,04	0	0,042
2	7421	0	0,04	0	0,01	0,95	0	0	0	0	0,95	0,02	0,02	0,01	0,038
2	5630	0	0	0	0,16	0	0	0,06	0,78	0	0,78	0,06	0	0,16	0,046
2	4223	0	0	0	0,13	0,87	0	0	0	0	0	0,67	0,33	0	0,048
2	43787	0	0,04	0	0,01	0,94	0	0	0	0,01	0	0,04	0,94	0,02	0,032
2	5088	0	0,04	0	0	0,86	0,1	0	0	0	0,84	0,04	0,01	0,11	0,04
2	62285	0	0	0	0,09	0,64	0,25	0	0,01	0,01	0,03	0,7	0,02	0,25	0,046
2	1444	0	0,03	0	0,14	0,83	0	0	0	0	0,97	0,03	0	0	0,044
2	14704	0	0,04	0	0	0,95	0	0	0,01	0	0,95	0,02	0,03	0	0,038
2	11815	0	0,04	0,01	0	0,94	0	0,01	0	0	0	0,95	0,03	0,02	0,04
2	1542	0	0,04	0	0	0,96	0	0	0	0	0	0,04	0	0,96	0,006
2	8450	0	0,04	0	0	0,96	0	0	0	0	0	0,04	0,96	0	0,022
2	18530	0	0,04	0	0	0,96	0	0	0	0	0	0,02	0,96	0,02	0,03
2	6151	0	0,04	0	0	0,94	0,02	0	0	0	0	0,06	0,94	0	0,018

2	40273	0	0,04	0	0	0,95	0	0,01	0	0	0,02	0,02	0,2	0,76	0,03
2	46830	0	0	0	0,08	0	0,41	0,02	0,49	0	0,26	0,41	0,08	0,25	0,032
2	21485	0,01	0,04	0	0	0,95	0	0	0	0	0	1	0	0	0,048
2	12999	0	0,02	0	0,19	0,79	0	0	0	0	0,79	0,21	0	0	0,048
2	17911	0	0,04	0	0,02	0,93	0	0	0	0,01	0	0,94	0,05	0,01	0,026
2	3811	0	0,04	0	0	0,95	0	0	0	0,01	0,95	0,03	0,02	0	0,042
2	1843	0	0,04	0	0,01	0,94	0	0,01	0	0	0,01	0,06	0	0,93	0,036
2	28545	0	0,04	0	0	0,96	0	0	0	0	0	0,04	0,96	0	0,018
2	1610	0	0,04	0	0	0,92	0,04	0	0	0	0,06	0,6	0,04	0,3	0,032
2	18407	0	0,04	0	0	0,95	0,01	0	0	0	0	0,04	0,96	0	0,03
2	17541	0	0,04	0	0,01	0,95	0	0	0	0	0	0,09	0,67	0,24	0,04
2	4338	0	0,04	0	0,01	0,95	0	0	0	0	0,29	0,04	0,51	0,16	0,026
2	35900	0	0	0	0,08	0,88	0,01	0	0,03	0	0,08	0	0,22	0,7	0,042
2	44289	0,01	0,04	0	0,01	0,94	0	0	0	0	0,94	0,01	0	0,05	0,048
2	8520	0	0,04	0	0	0,96	0	0	0	0	0	0,04	0,96	0	0,02
2	3278	0,02	0,04	0	0,01	0,91	0	0	0,02	0	0	0,05	0,92	0,03	0,042
2	6171	0	0	0	0,26	0,73	0	0,01	0	0	0,72	0,09	0,19	0	0,048
2	5787	0	0,04	0	0	0,95	0	0	0	0,01	0,95	0,01	0,04	0	0,034
2	16714	0	0,04	0	0	0,95	0	0	0,01	0	0,19	0,04	0,77	0	0,022
2	12410	0	0	0,01	0,12	0,75	0,12	0	0	0	0,13	0	0,86	0,01	0,044
2	8855	0	0,04	0	0,07	0,84	0,05	0	0	0	0,05	0,04	0,91	0	0,038
2	4875	0	0,04	0	0	0,96	0	0	0	0	0	1	0	0	0,016
2	16274	0	0,04	0,02	0,02	0,9	0	0	0,02	0	0	0,91	0,02	0,07	0,034
2	5644	0	0,04	0	0	0,95	0	0	0,01	0	0	0,97	0,02	0,01	0,022
2	43799	0	0,01	0,01	0,13	0,85	0	0	0	0	0,15	0,85	0	0	0,048
2	20238	0	0,04	0	0	0,95	0,01	0	0	0	0,02	0,97	0,01	0	0,028
2	65739	0	0,04	0	0	0,95	0	0	0	0,01	0,75	0,05	0,2	0	0,04
2	1889	0	0,02	0	0,25	0,72	0	0	0,01	0	0,96	0,01	0	0,03	0,042
2	19600	0	0,04	0	0	0,95	0	0	0	0,01	0,01	0,04	0,95	0	0,014
2	2383	0	0,04	0,01	0	0,95	0	0	0	0	0	0,04	0,01	0,95	0,006
2	8915	0	0,04	0	0	0,96	0	0	0	0	0	0,03	0,94	0,03	0,028
2	43068	0	0,04	0	0	0,96	0	0	0	0	0,96	0,02	0	0,02	0,04
2	16240	0	0,04	0	0	0,96	0	0	0	0	0,49	0,47	0,04	0	0,048

2	88990	0	0,02	0	0,1	0,88	0	0	0	0	0,02	0,9	0,08	0	0,044
2	7117	0	0,04	0	0	0,96	0	0	0	0	0	0,04	0	0,96	0,01
2,25	26373	0	0,04	0	0	0,96	0	0	0	0	0	0,96	0,04	0	0,018
2,25	3040	0	0	0	0,08	0,91	0	0	0	0,01	0,07	0,02	0	0,91	0,046
2,25	7880	0	0,04	0,01	0	0,94	0	0	0	0,01	0,01	0,04	0	0,95	0,016
2,25	2269	0,01	0,04	0	0	0,93	0	0,01	0,01	0	0,02	0,04	0,01	0,93	0,022
2,25	4690	0	0,04	0	0	0,96	0	0	0	0	0	0,04	0	0,96	0,016
2,25	8934	0	0,04	0,02	0,01	0,92	0	0	0,01	0	0,03	0,05	0	0,92	0,022
2,25	2481	0,02	0,04	0,02	0	0,9	0	0	0,02	0	0,03	0,06	0,9	0,01	0,048
2,25	4111	0	0,04	0	0	0,88	0,08	0	0	0	0,08	0	0,91	0,01	0,022
2,25	16090	0	0,04	0	0	0,96	0	0	0	0	0	0	0	1	0,014
2,25	13965	0	0,04	0	0,01	0,95	0	0	0	0	0,04	0,88	0	0,08	0,03
2,25	2055	0,01	0,04	0	0	0,95	0	0	0	0	0	0,04	0	0,96	0,02
2,25	53635	0	0	0	0,06	0,93	0	0,01	0	0	0,06	0	0,01	0,93	0,044
2,25	19001	0	0,04	0	0	0,96	0	0	0	0	0	0,04	0	0,96	0,016
2,25	16116	0	0,04	0	0	0,95	0	0	0	0,01	0	0,04	0,01	0,95	0,004
2,25	33181	0	0,04	0	0	0,96	0	0	0	0	0	0,04	0,96	0	0,028
2,25	73719	0	0,04	0	0	0,95	0	0,01	0	0	0	0,04	0	0,96	0,018
2,25	8147	0	0,04	0	0,02	0,94	0	0	0	0	0	0,06	0,94	0	0,028
2,25	29919	0	0,04	0	0	0,96	0	0	0	0	0	0,04	0,96	0	0,032
2,25	3499	0	0,04	0	0	0,95	0	0	0	0,01	0,96	0,04	0	0	0,046
2,25	16739	0,01	0,04	0	0,03	0,92	0	0	0	0	0,01	0,96	0,03	0	0,022
2,25	5012	0	0,04	0,01	0	0,94	0,01	0	0	0	0,02	0,04	0,94	0	0,034
2,25	25694	0	0,04	0	0	0,92	0	0	0,04	0	0	0,98	0	0,02	0,022
2,25	42958	0	0,04	0	0	0,95	0	0,01	0	0	0,95	0,05	0	0	0,044
2,25	12127	0	0	0,01	0,13	0,82	0,02	0	0	0,02	0,14	0,02	0,84	0	0,048
2,25	5770	0	0,04	0	0	0,96	0	0	0	0	0,04	0	0	0,96	0,014
2,25	2204	0	0,01	0,01	0,34	0,64	0	0	0	0	0,99	0,01	0	0	0,046
2,25	7818	0	0,03	0,01	0,11	0,67	0,18	0	0	0	0,74	0,15	0,11	0	0,048
2,25	48282	0,01	0,04	0	0	0,94	0	0	0,01	0	0	0,82	0,14	0,04	0,044
2,25	5200	0,01	0,04	0	0	0,76	0	0	0,19	0	0	0,23	0	0,77	0,02
2,25	35332	0	0	0	0,37	0,61	0	0,01	0	0,01	0,99	0	0	0,01	0,046
2,25	53795	0	0,03	0,01	0,22	0,69	0,05	0	0	0	0,91	0	0,06	0,03	0,046

2,25	69628	0	0,04	0	0,01	0,89	0,06	0	0	0	0	0,04	0,9	0,06	0,028
2,25	2204	0,01	0,04	0	0	0,94	0,01	0	0	0	0	0,04	0,95	0,01	0,028
2,25	8408	0	0,04	0	0,08	0,88	0	0	0	0	0,83	0,04	0,05	0,08	0,034
2,25	1690	0	0,04	0	0	0,91	0,02	0	0,03	0	0,05	0,03	0,02	0,9	0,004
2,25	32685	0,03	0,04	0	0	0,91	0,01	0	0,01	0	0,27	0,05	0,55	0,13	0,032
2,25	17651	0	0,04	0	0	0,95	0	0	0	0,01	0,01	0,04	0,95	0	0,03
2,25	10456	0,01	0,04	0	0	0,94	0	0	0,01	0	0,79	0,04	0,16	0,01	0,048
2,25	25961	0	0,04	0	0,05	0,89	0,02	0	0	0	0,88	0,05	0,07	0	0,048
2,25	79962	0	0	0	0,26	0,74	0	0	0	0	0,73	0,21	0,05	0,01	0,048
2,25	10517	0	0,04	0	0	0,96	0	0	0	0	0	0,03	0,97	0	0,038
2,25	5527	0,01	0,04	0	0	0,95	0	0	0	0	0	0,04	0	0,96	0,012
2,25	42096	0	0,04	0	0	0,96	0	0	0	0	0	0,04	0	0,96	0,018
2,25	14187	0	0,04	0	0	0,96	0	0	0	0	0	0,04	0,96	0	0,024
2,25	53332	0	0,04	0	0	0,96	0	0	0	0	0	0,04	0,96	0	0,028
2,25	17434	0	0,04	0	0	0,95	0	0	0,01	0	0	0,02	0,03	0,95	0,016
2,25	8877	0	0,04	0	0	0,96	0	0	0	0	0,96	0,04	0	0	0,044
2,25	30117	0,03	0,02	0	0	0,92	0	0,02	0	0,01	0,93	0,05	0	0,02	0,042
2,25	1883	0,02	0,04	0,01	0,03	0,89	0,01	0	0	0	0,03	0,93	0,03	0,01	0,048
2,25	2203	0,01	0,04	0	0	0,94	0,01	0	0	0	0,01	0,98	0	0,01	0,038
2,25	24946	0	0,04	0	0,03	0,93	0	0	0	0	0,32	0,59	0	0,09	0,034
2,25	3939	0	0,04	0	0	0,96	0	0	0	0	0,04	0	0,96	0	0,02
2,25	9397	0	0,04	0	0	0	0	0	0,96	0	0,45	0,47	0,08	0	0,034
2,25	49623	0	0	0	0,08	0,9	0,01	0	0	0,01	0,09	0	0	0,91	0,048
2,25	7095	0	0,04	0	0	0,95	0,01	0	0	0	0	0,01	0,96	0,03	0,028
2,25	6641	0	0,04	0	0	0,96	0	0	0	0	0	0,01	0,99	0	0,02
2,25	1351	0	0,04	0	0,12	0,79	0	0	0,05	0	0,79	0,14	0,04	0,03	0,048
2,25	37534	0	0,04	0	0,01	0,95	0	0	0	0	0	0,72	0	0,28	0,038
2,25	53285	0	0	0	0,16	0	0,06	0	0,78	0	0,3	0,16	0,48	0,06	0,038
2,25	35963	0,01	0,04	0	0	0,95	0	0	0	0	0,07	0,05	0	0,88	0,028
2,25	8630	0,01	0,04	0,01	0	0,94	0	0	0	0	0,94	0,04	0,02	0	0,042
2,25	9942	0	0	0	0,12	0,88	0	0	0	0	0	0	1	0	0,038
2,25	26154	0,01	0	0	0,19	0,8	0	0	0	0	0,65	0,35	0	0	0,044
2,25	22662	0	0,04	0	0	0,94	0	0	0	0,02	0,02	0,04	0,94	0	0,024

2,25	21604	0	0,04	0,03	0	0,83	0,1	0	0	0	0,83	0,03	0,1	0,04	0,04
2,25	1114	0	0,04	0	0	0,9	0	0,06	0	0	0,93	0,06	0	0,01	0,042
2,25	17680	0	0,04	0	0	0,95	0	0,01	0	0	0	0,05	0,95	0	0,028
2,25	61350	0	0	0	0,2	0,78	0	0	0,02	0	0,78	0	0,02	0,2	0,048
2,25	7671	0,01	0,02	0	0,07	0,85	0,03	0	0	0,02	0	0,4	0,6	0	0,044
2,25	31209	0	0,02	0	0,19	0,77	0	0	0	0,02	0,75	0,02	0,21	0,02	0,048
2,25	70564	0	0,04	0	0,03	0,93	0	0	0	0	0,88	0,04	0,05	0,03	0,034
2,25	38476	0	0,04	0	0	0,96	0	0	0	0	0,93	0,07	0	0	0,038
2,25	10801	0	0	0	0,1	0,73	0,1	0	0	0,07	0,16	0,08	0	0,76	0,048
2,25	50245	0	0,04	0	0	0,95	0,01	0	0	0	0,03	0	0,92	0,05	0,018
2,25	7459	0	0,04	0	0	0,96	0	0	0	0	0	1	0	0	0,032
2,25	18099	0	0,04	0	0	0,96	0	0	0	0	0	0,11	0,89	0	0,02
2,25	19528	0	0,04	0	0	0,94	0,01	0	0	0,01	0,06	0	0,94	0	0,022
2,25	4959	0	0,04	0	0	0,96	0	0	0	0	0	0,05	0	0,95	0,006
2,25	79885	0	0,04	0	0,01	0,95	0	0	0	0	0,95	0,01	0	0,04	0,036
2,25	13719	0	0,04	0	0	0,96	0	0	0	0	0,03	0,06	0,91	0	0,032
2,25	14922	0	0,04	0	0	0,96	0	0	0	0	0	0,04	0	0,96	0,006
2,25	16916	0	0,04	0	0,13	0,73	0,01	0	0,08	0,01	0,68	0,1	0,09	0,13	0,046
2,25	12922	0	0,04	0	0	0,95	0	0	0	0,01	0,01	0,04	0	0,95	0,008
2,25	1514	0	0,04	0	0,02	0,93	0,01	0	0	0	0	0,04	0,94	0,02	0,042
2,25	43295	0	0,04	0	0,12	0,84	0	0	0	0	0,84	0,12	0	0,04	0,046
2,25	15254	0,02	0,04	0	0	0,93	0	0	0,01	0	0,93	0,05	0,02	0	0,044
2,25	35511	0	0,04	0	0	0,96	0	0	0	0	0,96	0,04	0	0	0,038
2,25	7590	0	0,04	0	0	0,96	0	0	0	0	0,87	0,1	0,03	0	0,042
2,25	18097	0	0,04	0	0,06	0,87	0	0	0,03	0	0	0,04	0,93	0,03	0,048
2,25	12589	0	0	0	0,26	0,74	0	0	0	0	0,74	0,19	0,02	0,05	0,042
2,25	34827	0	0,04	0	0	0,96	0	0	0	0	0	0,04	0	0,96	0,012
2,25	52712	0	0,04	0	0,02	0,86	0	0	0,06	0,02	0,92	0,06	0,02	0	0,042
2,25	22718	0	0,04	0	0	0,96	0	0	0	0	0,9	0,04	0,06	0	0,026
2,25	18103	0,01	0,03	0,01	0,13	0,79	0,02	0	0	0,01	0,81	0,04	0,14	0,01	0,046
2,25	22573	0	0,04	0	0,01	0,95	0	0	0	0	0	0,99	0	0,01	0,03
2,25	2004	0	0,04	0	0	0,96	0	0	0	0	0	0,1	0,9	0	0,032
2,25	11225	0	0,04	0	0,01	0,95	0	0	0	0	0,01	0,04	0,95	0	0,026

2,25	46847	0	0	0	0,36	0,64	0	0	0	0	0,99	0	0	0,01	0,04
2,25	26426	0	0,04	0	0	0,95	0	0	0,01	0	0,01	0	0,99	0	0,026
2,25	43019	0	0,03	0	0,18	0,76	0,03	0	0	0	0,78	0	0,22	0	0,048
2,5	100000	0,63	0,29	0	0	0,08	0	0	0	0	0,08	0,91	0,01	0	0,884
2,5	6051	0,01	0,04	0	0	0,95	0	0	0	0	0	0,95	0,01	0,04	0,03
2,5	20080	0	0,04	0	0	0,95	0,01	0	0	0	0,97	0,03	0	0	0,044
2,5	9731	0	0,04	0	0	0,96	0	0	0	0	0	1	0	0	0,032
2,5	10779	0,01	0,04	0	0,01	0,92	0	0	0,02	0	0,92	0,02	0,06	0	0,044
2,5	3695	0	0,04	0	0,04	0,92	0	0	0	0	0	0,06	0,94	0	0,026
2,5	69910	0	0,02	0,02	0,08	0,86	0,02	0	0	0	0,08	0,89	0	0,03	0,046
2,5	31120	0	0,04	0	0,07	0,89	0	0	0	0	0,89	0,04	0	0,07	0,042
2,5	35716	0,05	0	0	0,01	0,94	0	0	0	0	0,04	0,59	0,35	0,02	0,048
2,5	1729	0	0,04	0	0	0,96	0	0	0	0	0	0,51	0,49	0	0,034
2,5	42197	0	0,04	0	0	0,94	0	0,01	0	0,01	0	0,02	0,42	0,56	0,016
2,5	100000	0	1	0	0	0	0	0	0	0	0	1	0	0	0,942
2,5	8270	0	0,04	0	0,01	0,95	0	0	0	0	0	0,04	0,91	0,05	0,036
2,5	30298	0	0,04	0,01	0	0,95	0	0	0	0	0,04	0	0,96	0	0,012
2,5	3480	0	0,04	0	0,01	0,94	0	0	0,01	0	0,01	0,04	0,95	0	0,024
2,5	3502	0	0,04	0,02	0	0,9	0	0,01	0,03	0	0,06	0,02	0,01	0,91	0,014
2,5	22019	0	0,04	0	0	0,96	0	0	0	0	0	0,04	0	0,96	0,01
2,5	48004	0,01	0,04	0	0	0,95	0	0	0	0	0	0,05	0	0,95	0,018
2,5	11610	0	0,04	0	0	0,93	0	0,01	0,02	0	0,01	0,98	0,01	0	0,024
2,5	18160	0	0,04	0	0	0	0,07	0	0,89	0	0	0,93	0	0,07	0,018
2,5	87260	0	0,04	0	0	0,96	0	0	0	0	0	0,94	0,06	0	0,034
2,5	10458	0	0	0	0,3	0,61	0,09	0	0	0	0,92	0,08	0	0	0,046
2,5	38303	0,01	0,04	0	0	0,95	0	0	0	0	0	0,04	0,83	0,13	0,036
2,5	3924	0	0,04	0	0	0,96	0	0	0	0	0	0,04	0,96	0	0,022
2,5	16066	0	0,04	0	0,02	0,93	0,01	0	0	0	0,03	0,04	0	0,93	0,016
2,5	67146	0	0	0	0,13	0	0,02	0	0,85	0	0	0,14	0,85	0,01	0,048
2,5	4459	0	0,04	0	0	0,94	0	0	0,02	0	0,89	0,04	0,02	0,05	0,034
2,5	11229	0	0,04	0	0	0,96	0	0	0	0	0,18	0,4	0,41	0,01	0,042
2,5	15766	0	0,04	0	0	0,95	0	0	0	0,01	0,95	0,01	0,03	0,01	0,026
2,5	38337	0	0	0,01	0,09	0,89	0	0	0,01	0	0,09	0,01	0,37	0,53	0,036

2,5	38425	0	0,04	0	0,02	0,94	0	0	0	0	0,94	0,02	0,03	0,01	0,048
2,5	15749	0	0,02	0	0,15	0,06	0,76	0	0	0,01	0,78	0	0,14	0,08	0,048
2,5	24946	0	0,04	0	0	0,96	0	0	0	0	0,04	0	0,96	0	0,018
2,5	32721	0	0,04	0	0	0,96	0	0	0	0	0	0,04	0,96	0	0,01
2,5	34345	0	0,04	0	0,02	0,94	0	0	0	0	0,03	0,03	0,94	0	0,026
2,5	20127	0	0,03	0	0,02	0,95	0	0	0	0	0,97	0	0	0,03	0,038
2,5	34767	0	0,04	0,02	0,01	0	0,93	0	0	0	0,05	0	0,95	0	0,034
2,5	4637	0,01	0	0	0,2	0,61	0,17	0	0	0,01	0,75	0,04	0,01	0,2	0,046
2,5	42669	0	0	0	0,1	0,9	0	0	0	0	0	0	0,9	0,1	0,046
2,5	2850	0	0,04	0	0,02	0,94	0	0	0	0	0,94	0,01	0,02	0,03	0,04
2,5	29719	0	0	0,01	0,09	0,9	0	0	0	0	0,1	0	0	0,9	0,046
2,5	52181	0	0,04	0	0	0,95	0	0	0	0,01	0,04	0,01	0,9	0,05	0,03
2,5	26918	0,01	0,04	0	0	0,94	0	0	0,01	0	0,01	0,04	0,95	0	0,038
2,5	16473	0	0,04	0	0	0,96	0	0	0	0	0	0,04	0	0,96	0,022
2,5	17623	0,01	0,04	0,02	0	0,9	0	0	0,03	0	0,05	0,03	0,01	0,91	0,04
2,5	18075	0,01	0	0	0,15	0,84	0	0	0	0	0	0	0,99	0,01	0,048
2,5	5599	0	0,04	0	0	0,96	0	0	0	0	0	0	0	1	0,01
2,5	3027	0	0,04	0	0	0,96	0	0	0	0	0	0,04	0,96	0	0,024
2,5	1946	0	0,04	0	0,05	0,91	0	0	0	0	0,91	0,04	0	0,05	0,046
2,5	9378	0	0	0	0,07	0,85	0	0,01	0,06	0,01	0,09	0,01	0	0,9	0,046
2,5	32737	0	0,04	0	0	0,95	0	0,01	0	0	0	0,04	0	0,96	0,024
2,5	46495	0	0,04	0	0	0,95	0	0	0,01	0	0	0,04	0,01	0,95	0,006
2,5	45384	0	0,04	0	0	0,93	0,02	0,01	0	0	0,95	0,04	0	0,01	0,038
2,5	47181	0,02	0,04	0	0,01	0,93	0	0	0	0	0,01	0,06	0,93	0	0,042
2,5	2178	0	0	0	0,14	0,86	0	0	0	0	0,3	0	0,68	0,02	0,048
2,5	14620	0	0	0,01	0,07	0,92	0	0	0	0	0,08	0	0	0,92	0,042
2,5	100000	0,12	0,88	0	0	0	0	0	0	0	0,1	0,27	0,02	0,61	0,42
2,5	4760	0,05	0	0	0	0,95	0	0	0	0	0	0,05	0,95	0	0,048
2,5	8455	0	0,04	0	0,01	0,94	0,01	0	0	0	0	0,02	0,96	0,02	0,034
2,5	40921	0	0,04	0	0,04	0,92	0	0	0	0	0	0,04	0,04	0,92	0,026
2,5	35858	0	0,04	0	0	0,95	0	0	0,01	0	0,95	0,04	0,01	0	0,034
2,5	30529	0	0,03	0	0,07	0,14	0	0	0,53	0,23	0	0,08	0,23	0,69	0,048
2,5	5590	0	0,04	0	0	0,96	0	0	0	0	0	0,04	0,96	0	0,022

2,5	14098	0	0,04	0	0	0,96	0	0	0	0	0	0	1	0	0,032
2,5	2476	0	0,04	0	0	0,96	0	0	0	0	0,96	0,04	0	0	0,044
2,5	62091	0,06	0	0	0	0,92	0,02	0	0	0	0,02	0,06	0	0,92	0,048
2,5	27020	0	0,04	0,01	0,06	0,88	0	0	0,01	0	0,03	0,04	0,89	0,04	0,044
2,5	100000	0	0,88	0,1	0	0,02	0	0	0	0	0,1	0,9	0	0	0,938
2,5	10896	0	0,04	0	0	0,96	0	0	0	0	0	0,04	0,96	0	0,024
2,5	33483	0	0,04	0	0	0,77	0,01	0	0,17	0,01	0,01	0,01	0,77	0,21	0,034
2,5	20436	0	0,04	0	0	0,96	0	0	0	0	0,02	0,02	0	0,96	0,006
2,5	5784	0	0,04	0	0	0,95	0,01	0	0	0	0	0	0,05	0,95	0,006
2,5	95807	0,01	0,04	0	0,01	0,94	0	0	0	0	0	0,05	0,88	0,07	0,044
2,5	14225	0	0,02	0	0,15	0,83	0	0	0	0	0	0,17	0,83	0	0,048
2,5	7037	0	0,04	0	0	0,94	0	0	0,02	0	0	0,04	0,39	0,57	0,018
2,5	16772	0	0	0	0,13	0,86	0,01	0	0	0	0	1	0	0	0,04
2,5	15556	0	0,04	0,02	0	0,92	0,01	0,01	0	0	0	0,05	0,85	0,1	0,044
2,5	21027	0	0,04	0,01	0	0,95	0	0	0	0	0,03	0,93	0,03	0,01	0,026
2,5	73152	0	0,04	0	0	0,96	0	0	0	0	0,96	0,03	0	0,01	0,046
2,5	11962	0	0,04	0	0	0,96	0	0	0	0	0,01	0,04	0	0,95	0,008
2,5	16162	0,01	0,04	0	0,03	0,92	0	0	0	0	0,01	0,95	0	0,04	0,034
2,5	2932	0	0,04	0,01	0	0,95	0	0	0	0	0	0,04	0,44	0,52	0,028
2,5	29077	0	0,04	0	0	0,96	0	0	0	0	0,03	0	0,97	0	0,034
2,5	14853	0	0,04	0	0	0,96	0	0	0	0	0	0,04	0	0,96	0,006
2,5	39490	0,01	0,04	0	0	0	0,9	0	0,05	0	0,9	0,01	0,09	0	0,04
2,5	1771	0	0,04	0	0	0,9	0,04	0	0	0,02	0,94	0,04	0	0,02	0,038
2,5	23090	0	0,04	0	0,02	0,92	0	0	0	0,02	0,89	0,03	0,02	0,06	0,034
2,5	9257	0	0,04	0	0	0,96	0	0	0	0	0	0,04	0,96	0	0,028
2,5	2256	0	0,04	0	0,11	0,84	0,01	0	0	0	0,78	0,04	0,13	0,05	0,046
2,5	1212	0,01	0,04	0	0	0,94	0	0	0,01	0	0,01	0,05	0,94	0	0,026
2,5	5593	0	0,04	0	0	0,96	0	0	0	0	0	0,04	0,11	0,85	0,014
2,5	46412	0,01	0,04	0	0	0,95	0	0	0	0	0	0,04	0,69	0,27	0,036
2,5	19346	0	0,04	0	0	0,96	0	0	0	0	1	0	0	0	0,042
2,5	100000	0	0,97	0	0	0	0	0,03	0	0	0,02	0,26	0,01	0,71	0,278
2,5	59086	0	0,04	0	0	0,96	0	0	0	0	0	0,04	0,96	0	0,03
2,5	42871	0,04	0,02	0	0,01	0,93	0	0	0	0	0,94	0,04	0,01	0,01	0,046

2,5	10674	0	0,04	0	0	0,95	0	0	0	0,01	0	0,04	0,02	0,94	0,006
2,5	13365	0	0,04	0	0	0,96	0	0	0	0	0	0,04	0,96	0	0,022
2,5	42233	0,01	0,04	0	0	0,95	0	0	0	0	0	0,05	0	0,95	0,022
2,5	100000	0	1	0	0	0	0	0	0	0	0	0,98	0	0,02	0,898
2,75	57162	0	0,02	0	0,24	0,7	0,04	0	0	0	0,72	0,24	0,04	0	0,046
2,75	2304	0	0,04	0	0	0,96	0	0	0	0	0,01	0,98	0,01	0	0,03
2,75	100000	0	1	0	0	0	0	0	0	0	0	1	0	0	0,974
2,75	68049	0	0,04	0	0	0,96	0	0	0	0	0,01	0,04	0,95	0	0,008
2,75	2129	0,01	0,04	0	0	0,92	0,01	0	0,02	0	0	0,08	0,92	0	0,028
2,75	64016	0	0,04	0,01	0	0,92	0,03	0	0	0	0	0,08	0,92	0	0,022
2,75	25059	0	0,04	0	0	0,96	0	0	0	0	0,01	0,03	0	0,96	0,002
2,75	16014	0	0,04	0,01	0,03	0,92	0	0	0	0	0,01	0,96	0,02	0,01	0,048
2,75	15647	0	0,04	0	0	0,96	0	0	0	0	0	0,03	0,96	0,01	0,032
2,75	36732	0	0,04	0,02	0	0,93	0,01	0	0	0	0,93	0,04	0,02	0,01	0,044
2,75	31430	0	0,04	0	0	0,94	0,01	0	0,01	0	0,51	0	0,48	0,01	0,02
2,75	2652	0	0,04	0	0	0,95	0	0,01	0	0	0,01	0,04	0,95	0	0,036
2,75	2679	0	0,04	0	0	0,96	0	0	0	0	0,02	0,02	0,22	0,74	0,008
2,75	17539	0	0,04	0	0	0,96	0	0	0	0	0,5	0,04	0,46	0	0,02
2,75	42918	0	0,02	0	0,2	0,75	0	0	0,03	0	0,7	0,27	0,03	0	0,042
2,75	32139	0	0,04	0	0,06	0,9	0	0	0	0	0	0,98	0	0,02	0,048
2,75	29028	0	0	0	0,13	0,87	0	0	0	0	0	0	0,95	0,05	0,048
2,75	2459	0	0,04	0	0	0,94	0	0,02	0	0	0	0,06	0	0,94	0,02
2,75	69875	0,01	0,03	0	0,08	0,66	0,21	0,01	0	0	0,56	0,12	0,32	0	0,042
2,75	29007	0	0,04	0	0,01	0,95	0	0	0	0	0	0,04	0,96	0	0,02
2,75	4095	0	0,03	0	0,08	0,87	0,01	0,01	0	0	0	0,03	0,88	0,09	0,048
2,75	45624	0	0,04	0	0,14	0,01	0,81	0	0	0	0,85	0,01	0,14	0	0,038
2,75	100000	0,02	0	0,98	0	0	0	0	0	0	0,98	0	0	0,02	0,982
2,75	17304	0,03	0,04	0	0	0,9	0,01	0,02	0	0	0,01	0,09	0	0,9	0,042
2,75	65388	0,01	0	0	0,09	0,89	0,01	0	0	0	0	0,1	0,9	0	0,03
2,75	39413	0	0,04	0,03	0,01	0,91	0	0,01	0	0	0,02	0,04	0,02	0,92	0,028
2,75	3924	0	0,04	0	0	0,96	0	0	0	0	0	0,04	0,96	0	0,024
2,75	63442	0	0,02	0,03	0,06	0,86	0	0,01	0	0,02	0	0,05	0,92	0,03	0,04
2,75	18568	0	0,04	0,01	0	0,94	0	0	0,01	0	0,01	0,04	0,02	0,93	0,01

2,75	72171	0	0	0	0,12	0,87	0	0	0,01	0	0,01	0,87	0,12	0	0,044
2,75	3868	0	0,04	0	0	0,96	0	0	0	0	0	0,04	0	0,96	0,008
2,75	854	0	0,04	0	0	0,96	0	0	0	0	0,01	0,17	0,56	0,26	0,028
2,75	28221	0,01	0,04	0	0,01	0,94	0	0	0	0	0,94	0,05	0	0,01	0,044
2,75	21559	0	0,04	0	0	0,92	0	0,02	0,02	0	0	0	1	0	0,038
2,75	8250	0	0,04	0	0	0,96	0	0	0	0	0	0,04	0,12	0,84	0,012
2,75	52894	0	0,04	0	0	0,95	0,01	0	0	0	0	0,95	0,01	0,04	0,022
2,75	15728	0	0,04	0	0	0,95	0	0	0,01	0	0,97	0,02	0	0,01	0,04
2,75	4053	0	0,04	0	0	0,93	0,01	0,01	0	0,01	0	0	0,5	0,5	0,02
2,75	55830	0	0,04	0	0,01	0,95	0	0	0	0	0,02	0	0,05	0,93	0,016
2,75	34445	0	0	0	0,28	0,65	0,06	0	0,01	0	0,76	0,23	0,01	0	0,048
2,75	12116	0	0	0	0,08	0,91	0	0,01	0	0	0,01	0	0,99	0	0,042
2,75	40835	0	0,04	0	0	0,96	0	0	0	0	0,01	0	0,19	0,8	0,016
2,75	8129	0	0,01	0	0,07	0,92	0	0	0	0	0,07	0	0,01	0,92	0,04
2,75	37885	0	0,04	0	0	0,96	0	0	0	0	0	0,04	0,96	0	0,038
2,75	29835	0,01	0,04	0	0,02	0,93	0	0	0	0	0,03	0,93	0,02	0,02	0,042
2,75	9607	0	0,04	0	0,01	0,85	0,01	0	0,09	0	0	0,01	0,09	0,9	0,012
2,75	13856	0	0,04	0	0	0,96	0	0	0	0	0	0,96	0,04	0	0,02
2,75	100000	0	1	0	0	0	0	0	0	0	0,04	0,96	0	0	0,956
2,75	22892	0	0,04	0	0	0,94	0	0,01	0,01	0	0,01	0,02	0,95	0,02	0,034
2,75	10520	0	0,04	0	0,03	0,88	0,05	0	0	0	0,04	0,9	0,05	0,01	0,038
2,75	43275	0	0,04	0	0	0,96	0	0	0	0	0	0,04	0,03	0,93	0,018
2,75	26755	0	0	0	0,45	0	0,54	0	0,01	0	1	0	0	0	0,046
2,75	9678	0,01	0,04	0	0	0	0	0,03	0,92	0	0,04	0	0,96	0	0,048
2,75	3279	0	0,04	0,01	0	0,95	0	0	0	0	0,01	0,97	0,02	0	0,024
2,75	28144	0	0,04	0	0	0,96	0	0	0	0	0	0,04	0	0,96	0,006
2,75	4244	0	0	0	0,13	0,82	0	0,01	0,04	0	0,13	0,01	0,86	0	0,038
2,75	51199	0	0,04	0	0,05	0,88	0,01	0	0	0,02	0,07	0,05	0,88	0	0,044
2,75	24241	0	0,04	0	0	0,92	0	0	0,04	0	0	0,07	0	0,93	0,012
2,75	44553	0	0,04	0	0	0,96	0	0	0	0	0	0,04	0	0,96	0,008
2,75	72993	0	0,04	0	0,01	0,95	0	0	0	0	0,69	0,04	0	0,27	0,046
2,75	16654	0	0,04	0	0	0,94	0,01	0	0	0,01	0	0,06	0,94	0	0,028
2,75	22915	0	0,04	0	0	0,91	0	0	0,05	0	0	0,04	0,91	0,05	0,032

2,75	27039	0	0,04	0	0	0,96	0	0	0	0	0	0,28	0,04	0,68	0,012
2,75	31149	0,01	0,04	0,01	0	0,91	0,01	0,02	0	0	0,01	0	0,92	0,07	0,04
2,75	18805	0,01	0,04	0	0	0,93	0,02	0	0	0	0,97	0,02	0,01	0	0,038
2,75	58319	0	0,04	0,01	0,07	0,87	0,01	0	0	0	0	0,05	0,07	0,88	0,046
2,75	10659	0	0,04	0	0,03	0,92	0	0	0,01	0	0,04	0,07	0,62	0,27	0,036
2,75	19692	0	0,02	0	0,08	0,86	0,04	0	0	0	0	0,12	0,02	0,86	0,04
2,75	12043	0	0,04	0	0	0,95	0	0	0	0,01	0	0,96	0,04	0	0,026
2,75	88857	0,01	0,01	0	0,05	0,84	0,04	0,04	0	0,01	0,05	0,01	0,93	0,01	0,046
2,75	40120	0	0	0	0,11	0,89	0	0	0	0	0	1	0	0	0,038
2,75	35767	0	0	0	0,08	0	0	0	0,92	0	0,04	0	0,04	0,92	0,046
2,75	88553	0	0	0	0,43	0,57	0	0	0	0	1	0	0	0	0,046
2,75	100000	0,01	0,98	0	0,01	0	0	0	0	0	0	0,97	0,02	0,01	0,868
2,75	28561	0	0	0	0,16	0,82	0,02	0	0	0	0,16	0,02	0,82	0	0,046
2,75	11290	0	0,01	0	0,1	0	0	0	0,89	0	0,01	0,94	0	0,05	0,046
2,75	16633	0	0,04	0	0	0,95	0,01	0	0	0	0	0,05	0	0,95	0,002
2,75	41244	0,01	0,04	0	0,05	0,89	0	0	0	0,01	0,01	0,05	0,05	0,89	0,044
2,75	5712	0	0,03	0,04	0	0,93	0	0	0	0	0,8	0,2	0	0	0,048
2,75	12241	0	0,04	0	0	0,94	0	0	0,02	0	0	0,05	0,95	0	0,028
2,75	4373	0,03	0,03	0	0	0,92	0	0,01	0	0,01	0,89	0,06	0	0,05	0,048
2,75	41014	0	0	0	0,11	0,8	0,09	0	0	0	0	0,11	0,89	0	0,038
2,75	4460	0	0,04	0	0	0,96	0	0	0	0	0,02	0,98	0	0	0,032
2,75	44214	0	0,04	0,01	0,02	0,06	0	0	0,87	0	0,07	0,84	0,07	0,02	0,036
2,75	4320	0	0,04	0	0	0,96	0	0	0	0	0	0,04	0	0,96	0,01
2,75	11385	0,06	0	0	0	0,94	0	0	0	0	0	1	0	0	0,048
2,75	14984	0	0	0	0,16	0,84	0	0	0	0	0,16	0,84	0	0	0,048
2,75	26471	0	0,04	0	0	0,96	0	0	0	0	0	0,04	0,07	0,89	0,008
2,75	52711	0	0,04	0	0	0,96	0	0	0	0	0,96	0,04	0	0	0,042
2,75	2797	0	0,04	0,02	0	0,93	0	0,01	0	0	0,01	0,03	0,01	0,95	0,03
2,75	95652	0	0,04	0	0	0,95	0,01	0	0	0	0,05	0,47	0,48	0	0,026
2,75	19429	0	0,04	0	0	0,96	0	0	0	0	0,1	0,04	0	0,86	0,014
2,75	23689	0	0,03	0	0,16	0,81	0	0	0	0	0,82	0,02	0,16	0	0,048
2,75	84229	0	0,04	0	0,01	0,95	0	0	0	0	0,01	0,04	0	0,95	0,024
2,75	100000	0,34	0,66	0	0	0	0	0	0	0	0	0,74	0,26	0	0,728

2,75	50869	0	0,04	0,01	0	0,95	0	0	0	0	0	0,01	0,96	0,03	0,034
2,75	19815	0	0,04	0	0	0,96	0	0	0	0	0,01	0,87	0,11	0,01	0,02
2,75	5391	0	0,04	0	0	0,95	0	0	0,01	0	0,05	0,5	0,44	0,01	0,022
2,75	11507	0	0	0	0,08	0,85	0,07	0	0	0	0,08	0,07	0	0,85	0,046
2,75	15763	0,01	0,04	0	0	0,94	0	0,01	0	0	0,02	0,04	0,94	0	0,034
3	100000	0	0,99	0	0	0	0	0	0	0,01	0,01	0,99	0	0	0,956
3	45449	0	0	0	0,3	0,67	0,02	0,01	0	0	0,91	0,03	0,04	0,02	0,048
3	15376	0	0,04	0	0	0,89	0	0	0,06	0,01	0	0,04	0	0,96	0,008
3	28414	0,02	0,04	0,01	0	0,91	0	0	0,01	0,01	0,01	0,06	0,93	0	0,03
3	43987	0	0,04	0	0	0,96	0	0	0	0	0	0,04	0	0,96	0,006
3	27216	0	0,04	0	0	0,94	0,01	0,01	0	0	0,01	0,97	0,01	0,01	0,036
3	3895	0	0	0,01	0,36	0,63	0	0	0	0	0,96	0,01	0	0,03	0,048
3	99071	0	0,04	0	0	0,85	0,1	0,01	0	0	0,04	0,85	0,11	0	0,022
3	51638	0	0,01	0	0,18	0,78	0	0,02	0,01	0	0,78	0,02	0,2	0	0,046
3	56920	0	0	0,01	0,18	0,76	0	0	0,05	0	0,71	0,06	0,23	0	0,036
3	20338	0	0	0	0,1	0,67	0,23	0	0	0	0,1	0	0,57	0,33	0,048
3	2511	0,03	0,01	0	0,12	0,82	0	0	0,02	0	0,83	0,03	0,02	0,12	0,048
3	39530	0	0,04	0	0	0,96	0	0	0	0	0	0,02	0,96	0,02	0,024
3	40031	0	0,03	0	0,07	0,76	0	0	0,14	0	0	0,76	0,1	0,14	0,042
3	11229	0,01	0,04	0	0	0,95	0	0	0	0	0	0,03	0,04	0,93	0,02
3	22448	0	0,04	0,01	0	0,95	0	0	0	0	0	0,98	0	0,02	0,01
3	27420	0	0,04	0	0	0,95	0,01	0	0	0	0,01	0,04	0,95	0	0,034
3	5954	0	0,01	0	0,11	0,88	0	0	0	0	0	0,8	0,2	0	0,048
3	29454	0	0,04	0	0	0,95	0	0,01	0	0	0	0,04	0,07	0,89	0,016
3	36173	0	0,04	0,01	0	0,94	0,01	0	0	0	0,04	0,94	0,02	0	0,028
3	24291	0,01	0,04	0	0	0,94	0	0	0	0,01	0,01	0	0,04	0,95	0,018
3	24202	0	0,04	0	0	0,8	0	0	0,16	0	0	0,04	0	0,96	0,006
3	67139	0	0,04	0	0	0,95	0,01	0	0	0	0,01	0,03	0,96	0	0,028
3	6362	0	0,04	0	0	0,96	0	0	0	0	0	0,04	0	0,96	0,004
3	2670	0	0,04	0	0	0,96	0	0	0	0	0	0,05	0,95	0	0,03
3	23293	0	0,04	0	0	0,95	0	0,01	0	0	0	0,04	0,96	0	0,034
3	32707	0	0,04	0	0	0,96	0	0	0	0	0	0,04	0	0,96	0,01
3	7020	0	0,04	0	0	0,96	0	0	0	0	0	0,04	0,96	0	0,032

3	8506	0,06	0	0	0	0,82	0,1	0	0	0,02	0,08	0,9	0,02	0	0,046
3	26297	0	0	0	0,12	0,86	0	0,01	0	0,01	0,13	0	0,87	0	0,046
3	26767	0	0,04	0,01	0	0,95	0	0	0	0	0,02	0,04	0	0,94	0,02
3	33015	0	0,04	0	0	0,96	0	0	0	0	0	0,99	0,01	0	0,02
3	12176	0	0,04	0,01	0	0,95	0	0	0	0	0,05	0	0,95	0	0,036
3	75003	0	0,04	0	0	0,96	0	0	0	0	0	0,04	0	0,96	0,014
3	30934	0,01	0,04	0,01	0	0,94	0	0	0	0	0,01	0,04	0,22	0,73	0,022
3	75482	0	0,04	0	0,03	0,89	0	0,01	0,03	0	0	0,05	0,03	0,92	0,042
3	25979	0	0,04	0	0	0,94	0	0	0,02	0	0	0,94	0,02	0,04	0,012
3	21400	0	0,04	0,01	0	0,95	0	0	0	0	0	0,01	0	0,99	0,01
3	33766	0	0,04	0	0	0,96	0	0	0	0	0	0,02	0,98	0	0,032
3	60399	0	0,04	0	0	0,96	0	0	0	0	0	0,05	0	0,95	0,012
3	53282	0	0	0	0,32	0,68	0	0	0	0	0,99	0	0	0,01	0,046
3	55159	0	0,04	0	0	0,96	0	0	0	0	0	0,04	0	0,96	0,008
3	5330	0,05	0,01	0	0	0,94	0	0	0	0	0,98	0,01	0,01	0	0,046
3	56556	0	0,04	0	0	0,95	0	0	0,01	0	0	0	0,96	0,04	0,018
3	25714	0	0,01	0	0,11	0,88	0	0	0	0	0,11	0	0,88	0,01	0,04
3	42152	0	0,04	0	0	0,91	0	0	0,05	0	0	0,04	0,01	0,95	0,006
3	2068	0	0,04	0	0	0,96	0	0	0	0	0,02	0,04	0	0,94	0,006
3	38992	0	0,04	0	0	0,96	0	0	0	0	0,09	0,04	0	0,87	0,01
3	39731	0,01	0,04	0	0	0,95	0	0	0	0	0,83	0,08	0,01	0,08	0,048
3	76863	0	0	0	0,08	0	0,89	0,03	0	0	0,02	0,45	0,49	0,04	0,046
3	41899	0	0,04	0	0,06	0,9	0	0	0	0	0,04	0,14	0,76	0,06	0,04
3	14465	0	0	0	0,12	0,88	0	0	0	0	0	0,88	0	0,12	0,048
3	25121	0,01	0,04	0,01	0	0,85	0	0,02	0,07	0	0	0,89	0,11	0	0,032
3	65447	0	0,04	0,03	0,01	0,92	0	0	0	0	0,01	0,04	0	0,95	0,022
3	43083	0	0,04	0	0	0,68	0,28	0	0	0	0,18	0	0,82	0	0,032
3	32381	0	0,04	0	0	0,96	0	0	0	0	0	0,04	0	0,96	0,018
3	41536	0	0,04	0,02	0	0,92	0,01	0	0,01	0	0,03	0,02	0,13	0,82	0,014
3	87498	0	0	0	0,26	0,73	0,01	0	0	0	0,75	0	0,25	0	0,048
3	26348	0	0,04	0	0,05	0,9	0	0	0	0,01	0	0,91	0,05	0,04	0,04
3	12751	0	0,04	0	0	0,96	0	0	0	0	0	0	0	1	0,018
3	10968	0	0,04	0	0,04	0,92	0	0	0	0	0	0,04	0,92	0,04	0,046

3	32997	0	0,04	0	0,08	0,82	0,06	0	0	0	0,09	0,91	0	0	0,042
3	17251	0	0,04	0	0,06	0,8	0,1	0	0	0	0,8	0,04	0,06	0,1	0,044
3	34173	0	0,03	0	0,11	0,85	0	0	0	0,01	0,03	0,86	0,11	0	0,044
3	17537	0	0,04	0	0	0,96	0	0	0	0	0	0,02	0,98	0	0,034
3	100000	0	0,99	0	0,01	0	0	0	0	0	0	0,99	0,01	0	0,93
3	75812	0,03	0,03	0	0	0,92	0,01	0	0,01	0	0,92	0,03	0,03	0,02	0,048
3	100000	0	0	0	0,55	0,45	0	0	0	0	0,54	0,13	0,01	0,32	0,2
3	6184	0	0,04	0,01	0,01	0,93	0,01	0	0	0	0,02	0,04	0,93	0,01	0,034
3	16359	0,04	0,02	0	0	0,93	0	0	0,01	0	0,94	0,06	0	0	0,048
3	23469	0	0,04	0	0	0,96	0	0	0	0	0	0,04	0,96	0	0,028
3	30166	0	0,04	0	0	0,93	0	0	0,02	0,01	0,02	0,05	0,93	0	0,022
3	44036	0	0,04	0	0	0,91	0	0	0,05	0	0	0,09	0,91	0	0,036
3	49168	0	0,04	0	0	0,96	0	0	0	0	0	1	0	0	0,02
3	3184	0	0,04	0	0	0,94	0	0	0	0,02	0,02	0,04	0,94	0	0,024
3	73822	0	0,04	0	0	0,94	0	0	0,02	0	0	0	0,06	0,94	0,014
3	46752	0	0,04	0	0	0,94	0,01	0,01	0	0	0	0,06	0,94	0	0,032
3	1058	0	0,04	0	0,03	0,92	0	0	0	0,01	0,91	0,03	0,02	0,04	0,03
3	33425	0	0,04	0	0	0,96	0	0	0	0	0	0	0	1	0,002
3	24120	0	0,04	0	0	0,92	0	0	0,04	0	0,02	0,08	0	0,9	0,006
3	23987	0	0,04	0,01	0	0,94	0	0	0,01	0	0,01	0,04	0,95	0	0,032
3	1917	0	0,04	0	0	0,95	0	0	0,01	0	0,13	0,04	0,12	0,71	0,018
3	31425	0	0	0,03	0,23	0,02	0,71	0,01	0	0	0,94	0,05	0,01	0	0,048
3	15345	0	0,02	0	0,12	0,84	0,01	0	0,01	0	0,14	0,76	0	0,1	0,04
3	30521	0	0,04	0	0,02	0,93	0	0,01	0	0	0,01	0,04	0,93	0,02	0,048
3	98575	0	0,04	0	0	0,95	0	0	0	0,01	0	0,05	0	0,95	0,002
3	12078	0	0,04	0	0	0,96	0	0	0	0	0	0,96	0,04	0	0,016
3	14644	0	0,03	0	0,18	0,71	0,08	0	0	0	0,8	0,02	0,18	0	0,046
3	17632	0	0,04	0	0	0,76	0,2	0	0	0	0,76	0,2	0	0,04	0,03
3	20636	0,02	0,04	0	0,02	0,91	0	0,01	0	0	0,01	0,01	0,94	0,04	0,048
3	1998	0,01	0,02	0	0,12	0,81	0,04	0	0	0	0,58	0,29	0,01	0,12	0,048
3	1977	0	0,04	0	0	0,96	0	0	0	0	0	0,04	0,89	0,07	0,032
3	3471	0	0,04	0	0,02	0,94	0	0	0	0	0,94	0,03	0	0,03	0,048
3	39480	0,01	0,04	0	0	0,95	0	0	0	0	0,95	0,01	0,03	0,01	0,042

3	67104	0	0,04	0	0,07	0,88	0	0	0	0,01	0,94	0,04	0,01	0,01	0,04
3	47061	0,03	0	0	0	0,8	0,06	0,05	0,06	0	0	0,89	0,11	0	0,04
3	17996	0,01	0,04	0	0	0,95	0	0	0	0	0	0,04	0,95	0,01	0,034
3	28068	0	0,04	0	0	0,96	0	0	0	0	0	0,02	0,03	0,95	0,016
3	30727	0	0,04	0	0	0,93	0	0,02	0,01	0	0	0,3	0	0,7	0,034
3	5845	0,01	0	0	0,09	0,88	0	0	0	0,02	0	0,91	0,06	0,03	0,044
3,25	67932	0	0,04	0	0	0,96	0	0	0	0	0	0,09	0	0,91	0,004
3,25	63896	0	0,04	0	0,04	0	0	0	0,92	0	0	0,04	0	0,96	0,042
3,25	31602	0	0,04	0	0	0,94	0	0	0	0,02	0,02	0,04	0	0,94	0,014
3,25	79661	0	0	0,02	0,1	0,72	0,03	0	0,13	0	0,05	0,17	0,76	0,02	0,034
3,25	11099	0,01	0,04	0	0,05	0,9	0	0	0	0	0,05	0,01	0,78	0,16	0,044
3,25	10696	0	0,04	0,01	0	0,67	0,27	0	0	0,01	0,98	0,01	0	0,01	0,048
3,25	89999	0,01	0,04	0	0	0,92	0	0	0,03	0	0	0,04	0,95	0,01	0,032
3,25	57298	0,01	0,04	0	0	0,95	0	0	0	0	0,04	0	0	0,96	0,016
3,25	9694	0	0,04	0	0	0,88	0	0	0,08	0	0	0,08	0,04	0,88	0,008
3,25	1249	0	0,04	0	0,12	0,84	0	0	0	0	0,84	0,16	0	0	0,038
3,25	16992	0,04	0,03	0	0	0,93	0	0	0	0	0,23	0,03	0,74	0	0,048
3,25	29773	0,05	0,02	0,01	0	0,61	0,19	0	0,12	0	0,17	0,21	0,61	0,01	0,048
3,25	89072	0	0,04	0	0	0,95	0	0,01	0	0	0,03	0,04	0,01	0,92	0,014
3,25	26068	0,01	0,04	0	0,02	0,91	0	0	0	0,02	0	0,34	0	0,66	0,034
3,25	31118	0,02	0	0	0,11	0,53	0,33	0	0,01	0	0,33	0,55	0,02	0,1	0,046
3,25	14324	0	0,04	0	0,03	0,93	0	0	0	0	0,04	0	0,93	0,03	0,034
3,25	16905	0	0,04	0	0	0,96	0	0	0	0	0	0,04	0,96	0	0,026
3,25	56614	0	0,04	0	0,05	0,91	0	0	0	0	0	0,02	0,9	0,08	0,046
3,25	54988	0	0,04	0	0,02	0,94	0	0	0	0	0	0,04	0,02	0,94	0,03
3,25	39606	0	0,04	0,01	0	0,95	0	0	0	0	0	0,05	0,87	0,08	0,026
3,25	13100	0,01	0,04	0	0	0,94	0,01	0	0	0	0	0,97	0,01	0,02	0,036
3,25	88383	0	0,04	0	0	0,96	0	0	0	0	0,07	0,04	0,89	0	0,02
3,25	100000	0,01	0,97	0,02	0	0	0	0	0	0	0	0,97	0,01	0,02	0,834
3,25	73870	0	0	0,01	0,16	0	0,16	0	0,67	0	0,83	0	0,01	0,16	0,048
3,25	14233	0	0	0	0,3	0,69	0,01	0	0	0	0,69	0,31	0	0	0,048
3,25	19044	0	0,04	0	0,05	0,88	0,01	0	0,02	0	0,03	0	0,92	0,05	0,044
3,25	54457	0	0	0	0,12	0,87	0,01	0	0	0	0,13	0,01	0,84	0,02	0,046

3,25	49802	0,04	0,04	0	0	0,72	0,19	0	0	0,01	0,08	0	0,73	0,19	0,042
3,25	94410	0	0,04	0	0	0,96	0	0	0	0	0	0,02	0,98	0	0,026
3,25	52532	0	0,04	0	0	0,96	0	0	0	0	0,23	0	0,73	0,04	0,028
3,25	69912	0	0,04	0	0	0,95	0,01	0	0	0	0,01	0,04	0,17	0,78	0,012
3,25	34626	0	0,04	0	0	0,96	0	0	0	0	0,04	0	0	0,96	0,008
3,25	11150	0	0,04	0	0	0,96	0	0	0	0	0,96	0,01	0,03	0	0,028
3,25	100000	0	0,98	0	0	0,02	0	0	0	0	0	1	0	0	0,892
3,25	85694	0	0,04	0	0,01	0,95	0	0	0	0	0	0,96	0	0,04	0,028
3,25	39334	0	0,04	0	0	0,96	0	0	0	0	0	0,03	0,01	0,96	0,006
3,25	23488	0	0,03	0	0,09	0,84	0,04	0	0	0	0,09	0,01	0,9	0	0,048
3,25	29787	0	0,04	0	0	0,79	0	0	0,17	0	0,16	0,05	0	0,79	0,02
3,25	6633	0	0,04	0	0	0,96	0	0	0	0	0,95	0	0,01	0,04	0,042
3,25	100000	0	0,99	0	0	0	0	0,01	0	0	0	0,99	0,01	0	0,89
3,25	30928	0	0	0	0,12	0	0,29	0	0,59	0	0	0,7	0,3	0	0,036
3,25	18414	0	0	0,02	0,17	0,81	0	0	0	0	0,49	0,32	0,18	0,01	0,046
3,25	67258	0	0,04	0,03	0,03	0,9	0	0	0	0	0,04	0,91	0,03	0,02	0,034
3,25	11267	0	0	0	0,25	0,73	0	0	0	0,02	0,74	0,25	0,01	0	0,048
3,25	5724	0	0	0,01	0	0,86	0	0,12	0,01	0	0	0,87	0,12	0,01	0,046
3,25	25828	0	0,04	0	0	0,96	0	0	0	0	0	0,03	0	0,97	0,008
3,25	52834	0,02	0	0	0,17	0,81	0	0	0	0	0,81	0,01	0,16	0,02	0,046
3,25	28898	0	0,04	0	0	0	0,96	0	0	0	0,96	0,04	0	0	0,036
3,25	16422	0	0,04	0	0	0,96	0	0	0	0	0,25	0,04	0,71	0	0,032
3,25	22454	0	0,04	0	0	0,95	0	0,01	0	0	0	0,97	0,02	0,01	0,04
3,25	9584	0	0,04	0	0	0,96	0	0	0	0	0	0,03	0,01	0,96	0,008
3,25	7881	0	0,04	0	0	0,96	0	0	0	0	0	0,04	0	0,96	0,012
3,25	8650	0	0,04	0	0,06	0,9	0	0	0	0	0,06	0,05	0	0,89	0,04
3,25	4888	0	0	0	0,14	0,82	0,02	0,01	0,01	0	0,01	0,96	0,03	0	0,048
3,25	34423	0	0,04	0	0	0,93	0	0	0	0,03	0,8	0,04	0	0,16	0,028
3,25	13973	0	0,04	0	0	0,96	0	0	0	0	0	0,02	0,96	0,02	0,018
3,25	100000	0	0,99	0	0	0	0	0	0,01	0	0	0,99	0,01	0	0,924
3,25	42332	0	0,04	0	0	0,95	0	0,01	0	0	0	0,95	0,01	0,04	0,028
3,25	20406	0	0,04	0	0	0,96	0	0	0	0	0,01	0,65	0,31	0,03	0,018
3,25	26754	0	0,04	0	0	0,93	0,03	0	0	0	0,01	0,01	0,98	0	0,02

3,25	82377	0	0,04	0	0	0,96	0	0	0	0	0	0	0,04	0,96	0,01
3,25	24525	0	0,04	0	0	0,08	0,19	0	0,69	0	0,05	0,55	0,4	0	0,034
3,25	27894	0	0,04	0	0,02	0,89	0	0	0,05	0	0	0,93	0,05	0,02	0,034
3,25	11334	0	0,04	0	0,01	0,95	0	0	0	0	0,03	0,02	0,95	0	0,034
3,25	100000	0,06	0,83	0	0,11	0	0	0	0	0	0,11	0,83	0	0,06	0,744
3,25	61352	0	0	0	0,13	0,85	0,01	0,01	0	0	0,14	0,86	0	0	0,034
3,25	28285	0	0,04	0	0	0,95	0	0	0,01	0	0	0,28	0,71	0,01	0,024
3,25	20707	0,01	0,04	0	0	0,94	0	0	0	0,01	0	0,99	0	0,01	0,028
3,25	58119	0	0,04	0,01	0	0,95	0	0	0	0	0	0,05	0	0,95	0,008
3,25	20576	0	0,04	0,01	0	0,95	0	0	0	0	0,01	0,03	0,95	0,01	0,034
3,25	26355	0	0,04	0	0,02	0,94	0	0	0	0	0,86	0,03	0,08	0,03	0,04
3,25	74358	0	0,04	0	0	0,96	0	0	0	0	0,04	0	0,96	0	0,026
3,25	78441	0	0,04	0	0	0,94	0,02	0	0	0	0,16	0,03	0,02	0,79	0,014
3,25	6962	0	0,04	0	0	0,95	0,01	0	0	0	0	0,04	0,95	0,01	0,02
3,25	9814	0	0,04	0,02	0,03	0,78	0	0,03	0,1	0	0,05	0,04	0,81	0,1	0,046
3,25	19213	0	0,04	0	0	0,96	0	0	0	0	0	0	1	0	0,018
3,25	76614	0	0,04	0	0	0,95	0	0,01	0	0	0	0,04	0	0,96	0,03
3,25	17908	0	0,04	0	0,06	0,9	0	0	0	0	0,89	0,04	0,01	0,06	0,042
3,25	20690	0	0,04	0	0	0,96	0	0	0	0	0	0,04	0	0,96	0,014
3,25	46364	0	0,04	0	0	0	0,01	0	0,95	0	0	0,04	0,01	0,95	0,006
3,25	17022	0	0	0	0,39	0	0,61	0	0	0	1	0	0	0	0,044
3,25	33932	0	0,04	0,01	0,01	0,94	0	0	0	0	0,04	0,01	0,01	0,94	0,022
3,25	100000	0	0,94	0,01	0,04	0	0	0,01	0	0	0,01	0,94	0,05	0	0,744
3,25	3930	0	0,04	0	0	0,96	0	0	0	0	0,96	0,04	0	0	0,048
3,25	27458	0	0,04	0	0	0,93	0,03	0	0	0	0	0,97	0	0,03	0,032
3,25	12505	0,01	0	0	0,11	0,88	0	0	0	0	0,01	0,1	0,89	0	0,046
3,25	40589	0	0	0,01	0,2	0,79	0	0	0	0	0,8	0	0,2	0	0,046
3,25	29558	0,01	0,04	0,01	0	0,94	0	0	0	0	0,01	0,89	0	0,1	0,044
3,25	10919	0	0,04	0	0	0,94	0,01	0	0	0,01	0,01	0,04	0,95	0	0,026
3,25	21718	0	0,04	0	0,01	0	0,95	0	0	0	0,95	0,04	0	0,01	0,048
3,25	52097	0	0,04	0	0	0,82	0,01	0	0,13	0	0,01	0,04	0,93	0,02	0,014
3,25	100000	0	1	0	0	0	0	0	0	0	0	1	0	0	0,93
3,25	6377	0	0,04	0,01	0	0,95	0	0	0	0	0	0,06	0,08	0,86	0,008

3,25	12247	0	0,01	0	0,3	0,69	0	0	0	0	1	0	0	0	0,046
3,25	12704	0	0,04	0	0	0,94	0	0	0	0,02	0	0,02	0	0,98	0,004
3,25	6590	0	0,04	0	0	0,95	0,01	0	0	0	0,01	0,04	0,95	0	0,028
3,25	100000	0,01	0,95	0,03	0	0	0	0,01	0	0	0,57	0,41	0,01	0,01	0,924
3,25	7194	0	0	0	0,15	0,7	0,14	0	0	0,01	0,29	0,01	0,7	0	0,048
3,25	4212	0	0,04	0	0,01	0,95	0	0	0	0	0	0,03	0,01	0,96	0,016
3,25	13003	0,01	0,04	0	0	0,92	0,03	0	0	0	0,89	0,02	0,09	0	0,036
3,5	100000	0,03	0,96	0	0	0	0	0	0	0,01	0,01	0,96	0	0,03	0,87
3,5	39063	0,01	0	0	0,09	0,48	0,4	0	0	0,02	0,01	0,11	0,87	0,01	0,048
3,5	27866	0,03	0,04	0	0	0,93	0	0	0	0	0	0,05	0,95	0	0,048
3,5	42393	0,01	0,04	0	0	0,94	0	0	0	0,01	0,01	0,05	0	0,94	0,022
3,5	9595	0	0,01	0	0,18	0,81	0	0	0	0	0,81	0,01	0,18	0	0,046
3,5	35157	0	0	0	0,15	0,85	0	0	0	0	0,15	0	0,85	0	0,048
3,5	18308	0	0,04	0	0,01	0,95	0	0	0	0	0,02	0,97	0	0,01	0,032
3,5	34181	0,01	0,04	0	0	0,95	0	0	0	0	0,04	0,01	0,93	0,02	0,044
3,5	100000	0	1	0	0	0	0	0	0	0	0	1	0	0	0,958
3,5	61082	0	0,04	0	0	0,96	0	0	0	0	0	0,03	0,97	0	0,02
3,5	37678	0	0,04	0	0,01	0,95	0	0	0	0	0,01	0,04	0	0,95	0,02
3,5	5055	0	0,04	0	0	0,96	0	0	0	0	0,96	0	0,01	0,03	0,044
3,5	14709	0	0,04	0	0	0,94	0	0,01	0,01	0	0	0,4	0,01	0,59	0,02
3,5	97627	0	0,04	0,01	0	0,94	0,01	0	0	0	0	0,95	0,04	0,01	0,03
3,5	53953	0	0,04	0	0,05	0,9	0	0	0	0,01	0,05	0,95	0	0	0,036
3,5	49998	0	0,04	0	0	0,96	0	0	0	0	0	0,03	0,94	0,03	0,014
3,5	8718	0	0,04	0	0,01	0,94	0,01	0	0	0	0,34	0	0	0,66	0,028
3,5	15881	0	0,04	0	0	0,96	0	0	0	0	0,96	0	0	0,04	0,046
3,5	100000	0,04	0,96	0	0	0	0	0	0	0	0	0,95	0,01	0,04	0,82
3,5	26018	0,01	0	0	0,06	0,77	0	0,01	0,15	0	0	0,07	0	0,93	0,048
3,5	20183	0	0,04	0	0	0,96	0	0	0	0	0,04	0	0,96	0	0,024
3,5	47502	0,01	0,04	0	0	0,95	0	0	0	0	0,03	0,04	0,92	0,01	0,028
3,5	100000	0	1	0	0	0	0	0	0	0	0	1	0	0	0,99
3,5	39468	0	0,01	0	0,08	0,9	0	0	0,01	0	0,08	0	0	0,92	0,048
3,5	4956	0,03	0,02	0	0	0,93	0	0,01	0,01	0	0,05	0,93	0,01	0,01	0,03
3,5	27047	0	0	0,01	0,03	0,52	0	0,1	0,34	0	0,53	0,18	0,19	0,1	0,046

3,5	32440	0	0,04	0	0	0,96	0	0	0	0	0,01	0,04	0,95	0	0,032
3,5	24769	0	0,04	0	0	0,9	0	0	0	0,06	0,05	0,01	0,93	0,01	0,016
3,5	8993	0	0,04	0	0	0,96	0	0	0	0	0,01	0,03	0,96	0	0,022
3,5	30640	0	0,04	0	0	0,95	0	0	0,01	0	0	1	0	0	0,018
3,5	81401	0,05	0,01	0	0	0,93	0	0,01	0	0	0,04	0,01	0,94	0,01	0,046
3,5	16759	0	0,04	0	0	0,94	0	0	0	0,02	0,57	0,04	0	0,39	0,024
3,5	58197	0,05	0,02	0	0	0,93	0	0	0	0	0	0,08	0	0,92	0,042
3,5	41682	0	0,04	0	0	0,96	0	0	0	0	0	0,96	0	0,04	0,018
3,5	61392	0	0,04	0,01	0,06	0,76	0,07	0	0,06	0	0,07	0,87	0	0,06	0,046
3,5	87922	0	0	0	0,14	0,48	0,36	0	0,02	0	0,36	0,14	0,49	0,01	0,048
3,5	81646	0	0,04	0	0	0,96	0	0	0	0	0	0,01	0,99	0	0,03
3,5	37330	0,01	0,04	0	0	0,94	0,01	0	0	0	0,01	0,98	0,01	0	0,03
3,5	59197	0	0,04	0	0	0,95	0	0,01	0	0	0	0,01	0	0,99	0,012
3,5	4913	0	0,04	0	0	0,96	0	0	0	0	0	0,04	0,96	0	0,022
3,5	87797	0	0,04	0	0,06	0,9	0	0	0	0	0	0,9	0,04	0,06	0,046
3,5	22352	0	0	0	0,1	0,9	0	0	0	0	0,01	0	0,75	0,24	0,048
3,5	26025	0	0,04	0	0	0,92	0	0	0,01	0,03	0,08	0,01	0,91	0	0,026
3,5	27164	0,01	0,04	0	0	0,89	0	0	0,06	0	0,03	0,26	0,01	0,7	0,026
3,5	12744	0,01	0,04	0	0	0,95	0	0	0	0	0	0,01	0,04	0,95	0,02
3,5	15505	0,01	0,04	0	0,04	0,9	0	0	0	0,01	0,44	0,01	0,5	0,05	0,04
3,5	59813	0	0,04	0	0,06	0,75	0,15	0	0	0	0,94	0	0,06	0	0,028
3,5	33624	0,05	0	0	0	0,48	0	0	0,43	0,04	0	0,09	0,48	0,43	0,042
3,5	8759	0	0,04	0	0	0,96	0	0	0	0	0	0,04	0	0,96	0,008
3,5	10204	0	0,04	0	0,06	0,89	0	0	0,01	0	0,04	0,02	0,05	0,89	0,048
3,5	13918	0,05	0	0	0,03	0,92	0	0	0	0	0	0,05	0,93	0,02	0,044
3,5	40897	0	0,04	0	0,02	0,94	0	0	0	0	0	0,94	0,04	0,02	0,036
3,5	12821	0,01	0,04	0	0	0,95	0	0	0	0	0,78	0,04	0,18	0	0,048
3,5	100000	0,56	0,23	0	0,04	0,17	0	0	0	0	0	0,96	0	0,04	0,768
3,5	17582	0	0,04	0	0	0,95	0,01	0	0	0	0,99	0,01	0	0	0,042
3,5	63962	0	0,04	0	0,01	0,94	0	0	0	0,01	0,06	0	0	0,94	0,024
3,5	69609	0,03	0,03	0	0,04	0,88	0	0	0,02	0	0,5	0,01	0,46	0,03	0,048
3,5	55341	0	0,01	0,01	0,08	0,76	0,01	0,01	0,12	0	0,01	0	0,98	0,01	0,046
3,5	16249	0	0	0	0,36	0,56	0,07	0	0	0,01	0,92	0	0,08	0	0,048

3,5	57317	0	0,01	0	0,11	0	0,01	0	0,61	0,26	0,01	0,25	0,74	0	0,04
3,5	32939	0,02	0,03	0	0,08	0	0,87	0	0	0	0,89	0,08	0	0,03	0,048
3,5	32431	0	0	0	0,13	0,82	0,04	0	0,01	0	0,14	0,01	0,85	0	0,048
3,5	47551	0	0,01	0,01	0,07	0,85	0	0	0,06	0	0,06	0,05	0,01	0,88	0,044
3,5	35778	0	0,02	0	0,11	0,85	0	0	0	0,02	0,04	0,05	0,88	0,03	0,048
3,5	100000	0	0,96	0,03	0,01	0	0	0	0	0	0,01	0,96	0	0,03	0,832
3,5	13996	0	0,04	0	0,02	0,94	0	0	0	0	0,88	0,06	0	0,06	0,04
3,5	66449	0	0,04	0	0,01	0,94	0,01	0	0	0	0	0,05	0,01	0,94	0,02
3,5	22089	0,02	0	0,07	0	0	0,9	0	0	0,01	0	0,02	0,95	0,03	0,042
3,5	16087	0	0,04	0	0,08	0,83	0	0	0,05	0	0,06	0,05	0,88	0,01	0,03
3,5	20000	0	0,03	0	0,1	0,87	0	0	0	0	0,1	0,87	0,03	0	0,044
3,5	81068	0	0,04	0,01	0	0,93	0	0,01	0	0,01	0,01	0,05	0,01	0,93	0,026
3,5	5289	0,02	0,04	0	0	0,93	0,01	0	0	0	0	0,98	0,02	0	0,028
3,5	34545	0	0,04	0	0,02	0,91	0	0	0,03	0	0,02	0,07	0,91	0	0,03
3,5	10007	0	0,04	0	0	0,96	0	0	0	0	0	0,04	0,96	0	0,03
3,5	47517	0,02	0,04	0,01	0	0,9	0,01	0,02	0	0	0,92	0,04	0,02	0,02	0,046
3,5	31839	0	0,04	0	0	0,94	0,02	0	0	0	0	0,51	0	0,49	0,008
3,5	22813	0	0,04	0	0	0,95	0	0	0	0,01	0	0,03	0,01	0,96	0,006
3,5	23590	0	0	0	0,08	0,75	0,17	0	0	0	0,08	0	0	0,92	0,048
3,5	3863	0	0,04	0	0,02	0,93	0,01	0	0	0	0,01	0,07	0	0,92	0,022
3,5	2021	0	0,04	0	0	0,96	0	0	0	0	0	0,99	0	0,01	0,034
3,5	44709	0	0,04	0	0	0,96	0	0	0	0	0,96	0	0	0,04	0,042
3,5	29813	0,01	0,04	0,03	0	0,9	0,02	0	0	0	0,01	0,88	0,06	0,05	0,036
3,5	45515	0,01	0,04	0	0,06	0,16	0,72	0	0,01	0	0,66	0,08	0,1	0,16	0,048
3,5	100000	0	0,99	0	0,01	0	0	0	0	0	0	0,99	0	0,01	0,932
3,5	22215	0	0	0	0,12	0,15	0,01	0	0,72	0	0,07	0,91	0,02	0	0,046
3,5	19366	0	0	0	0,07	0,67	0	0	0,26	0	0,02	0,07	0,01	0,9	0,044
3,5	4255	0	0,01	0	0,38	0,6	0	0,01	0	0	0,97	0	0,02	0,01	0,048
3,5	26016	0	0,04	0	0,01	0,4	0	0	0,54	0,01	0,41	0	0,58	0,01	0,03
3,5	38046	0	0,04	0	0,01	0,95	0	0	0	0	0	0,04	0,01	0,95	0,026
3,5	44819	0	0	0,01	0,07	0,92	0	0	0	0	0,09	0	0	0,91	0,034
3,5	36215	0	0,04	0	0	0,96	0	0	0	0	0	0,03	0,97	0	0,032
3,5	19658	0	0,04	0	0	0,96	0	0	0	0	0	0,04	0,01	0,95	0,01

3,5	6224	0	0,04	0	0,03	0,93	0	0	0	0	0	0,04	0	0,96	0,048
3,5	24461	0	0,04	0	0,1	0,86	0	0	0	0	0,86	0,04	0,1	0	0,04
3,5	12241	0,01	0,04	0	0	0,95	0	0	0	0	0	0,04	0,95	0,01	0,042
3,5	2661	0	0,04	0	0	0,96	0	0	0	0	0	0,04	0,18	0,78	0,01
3,5	78698	0,01	0,04	0	0,03	0,92	0	0	0	0	0	0,03	0,93	0,04	0,038
3,5	100000	0	0,99	0	0	0,01	0	0	0	0	0	1	0	0	0,928
3,5	19479	0	0	0	0,08	0,92	0	0	0	0	0,08	0,29	0	0,63	0,046
3,5	24458	0	0,04	0,01	0	0,95	0	0	0	0	0,94	0,05	0,01	0	0,038
3,75	21189	0,05	0,01	0	0	0,93	0	0,01	0	0	0,99	0	0,01	0	0,046
3,75	35441	0	0,04	0	0,07	0,81	0,06	0,01	0	0,01	0,81	0,01	0,14	0,04	0,044
3,75	22220	0,02	0,03	0	0	0,95	0	0	0	0	0,95	0,05	0	0	0,034
3,75	74471	0	0,04	0	0	0,96	0	0	0	0	0	0,04	0,96	0	0,022
3,75	33272	0	0	0	0,16	0,84	0	0	0	0	0	0	1	0	0,042
3,75	31064	0,04	0	0,01	0,01	0,91	0	0,03	0	0	0,03	0	0,91	0,06	0,046
3,75	34969	0	0,04	0,01	0,01	0,94	0	0	0	0	0	0,06	0,94	0	0,036
3,75	17332	0	0	0	0,12	0,85	0	0,03	0	0	0	0,12	0,85	0,03	0,048
3,75	33729	0	0,04	0	0,02	0,92	0,01	0,01	0	0	0,01	0,01	0,92	0,06	0,038
3,75	99996	0,05	0	0	0	0	0,94	0,01	0	0	0,95	0	0,04	0,01	0,048
3,75	39459	0	0,04	0	0,02	0,93	0	0,01	0	0	0,36	0,6	0,01	0,03	0,042
3,75	31446	0	0,04	0	0	0,96	0	0	0	0	0	0	0	1	0,008
3,75	15028	0	0,04	0	0	0,95	0	0	0	0,01	0	0,05	0	0,95	0,01
3,75	100000	0	1	0	0	0	0	0	0	0	0	1	0	0	0,954
3,75	36692	0	0,04	0	0	0,93	0	0	0,02	0,01	0	0,05	0	0,95	0,008
3,75	81027	0	0,03	0	0,1	0,87	0	0	0	0	0,08	0,8	0,12	0	0,046
3,75	27183	0,02	0,03	0,01	0,01	0,93	0	0	0	0	0,95	0,03	0,02	0	0,048
3,75	16201	0	0,04	0	0	0,95	0	0	0,01	0	0,02	0,04	0,94	0	0,028
3,75	100000	0	0,97	0	0	0,03	0	0	0	0	0,03	0,97	0	0	0,904
3,75	21392	0,05	0	0	0	0,92	0	0,03	0	0	0,9	0,07	0,03	0	0,048
3,75	19907	0	0,04	0,01	0,03	0,91	0	0	0,01	0	0,46	0,52	0,01	0,01	0,042
3,75	9390	0	0	0	0,2	0,56	0,22	0	0	0,02	0,75	0,01	0	0,24	0,048
3,75	16673	0,01	0,04	0	0	0,95	0	0	0	0	0	0,04	0,01	0,95	0,014
3,75	38015	0	0,04	0	0	0,96	0	0	0	0	0,04	0,02	0,02	0,92	0,01
3,75	18418	0	0	0	0,24	0,75	0	0	0	0,01	0,76	0	0,24	0	0,046

3,75	12906	0	0,04	0	0	0,96	0	0	0	0	0,96	0,04	0	0	0,046
3,75	15639	0	0,04	0	0	0,96	0	0	0	0	0,04	0	0,96	0	0,024
3,75	100000	0	1	0	0	0	0	0	0	0	0	1	0	0	0,946
3,75	16328	0,05	0	0,02	0	0,84	0,02	0	0,07	0	0,02	0,09	0,89	0	0,044
3,75	17466	0	0	0	0,11	0,8	0,08	0	0	0,01	0,13	0	0	0,87	0,044
3,75	10974	0	0,04	0,01	0	0,95	0	0	0	0	0	0,01	0,95	0,04	0,034
3,75	21880	0,02	0,04	0,01	0	0,14	0	0,01	0,77	0,01	0,01	0,19	0,8	0	0,046
3,75	29534	0	0,04	0	0	0,96	0	0	0	0	0	0	0	1	0,012
3,75	51642	0	0,04	0	0	0,94	0,01	0,01	0	0	0	0,04	0,96	0	0,026
3,75	100000	0,01	0,91	0	0,01	0,06	0	0	0	0,01	0,01	0,92	0,06	0,01	0,686
3,75	11136	0	0,04	0,01	0,06	0,88	0,01	0	0	0	0	0,04	0,9	0,06	0,046
3,75	90388	0	0,04	0	0	0,94	0	0,02	0	0	0,02	0,04	0,94	0	0,044
3,75	79815	0	0,04	0	0	0,96	0	0	0	0	0,6	0	0,36	0,04	0,042
3,75	81410	0	0	0	0,1	0,7	0,11	0	0,09	0	0,07	0,81	0,01	0,11	0,048
3,75	10683	0,01	0	0	0,08	0,9	0,01	0	0	0	0,01	0,9	0	0,09	0,048
3,75	4813	0	0,04	0	0	0,96	0	0	0	0	0	0,04	0,96	0	0,028
3,75	16115	0,06	0	0	0	0,92	0	0,02	0	0	0,92	0	0,02	0,06	0,044
3,75	55708	0	0	0	0,24	0,65	0,11	0	0	0	0,61	0,05	0,24	0,1	0,048
3,75	29896	0	0,04	0	0,07	0,87	0	0	0	0,02	0,87	0	0,09	0,04	0,036
3,75	4254	0	0,04	0	0	0,96	0	0	0	0	0	0,04	0,96	0	0,034
3,75	79889	0	0,04	0	0	0,95	0	0	0	0,01	0,03	0,01	0,96	0	0,026
3,75	13537	0,06	0	0,02	0	0,91	0,01	0	0	0	0,92	0	0,06	0,02	0,048
3,75	9954	0	0,04	0	0	0,96	0	0	0	0	0	0,04	0	0,96	0,006
3,75	5264	0	0	0	0,34	0,65	0	0,01	0	0	0,98	0	0,02	0	0,042
3,75	16197	0,05	0,02	0	0	0,93	0	0	0	0	0	0,07	0,93	0	0,048
3,75	7770	0,05	0	0	0,01	0,92	0	0	0,02	0	0,97	0,02	0	0,01	0,048
3,75	46909	0,04	0,02	0	0	0,93	0	0	0	0,01	1	0	0	0	0,04
3,75	2386	0,03	0,04	0	0	0,93	0	0	0	0	0	0,06	0,92	0,02	0,048
3,75	46686	0	0,04	0	0,02	0,94	0	0	0	0	0	0,06	0,94	0	0,03
3,75	14709	0	0,04	0	0	0,95	0,01	0	0	0	0,04	0,63	0	0,33	0,026
3,75	22508	0	0,04	0	0	0,94	0	0	0,02	0	0	0,05	0,95	0	0,02
3,75	18060	0	0,04	0	0	0,96	0	0	0	0	0	1	0	0	0,018
3,75	26849	0	0	0	0,17	0,45	0	0	0,38	0	0,45	0	0,55	0	0,048

3,75	5959	0	0	0,01	0,12	0,87	0	0	0	0	0	0,12	0,87	0,01	0,048
3,75	75625	0	0	0,03	0,06	0,55	0,31	0	0,05	0	0	0,37	0,08	0,55	0,044
3,75	35150	0,03	0,03	0	0	0,91	0	0	0,02	0,01	0	0,89	0,07	0,04	0,044
3,75	52709	0,05	0	0	0	0,86	0	0	0,08	0,01	0,06	0,07	0,87	0	0,044
3,75	22358	0	0,04	0	0,08	0,88	0	0	0	0	0,89	0,1	0	0,01	0,048
3,75	1629	0,01	0,04	0	0	0,95	0	0	0	0	0	0,04	0,95	0,01	0,024
3,75	69399	0	0,04	0	0	0	0	0	0,96	0	0,96	0	0,04	0	0,036
3,75	26187	0	0	0	0,06	0	0	0	0	0,94	0	0,9	0	0,1	0,046
3,75	5858	0	0,04	0	0	0,96	0	0	0	0	0	0	0,96	0,04	0,028
3,75	6836	0	0	0	0,13	0,87	0	0	0	0	0	0	1	0	0,046
3,75	51452	0	0	0	0,12	0	0	0	0,88	0	0,01	0,99	0	0	0,04
3,75	53439	0	0	0	0,07	0,89	0,04	0	0	0	0,11	0,01	0	0,88	0,042
3,75	69961	0	0,04	0	0	0,96	0	0	0	0	0	0	0,04	0,96	0,006
3,75	92375	0	0,04	0	0,02	0,92	0	0,02	0	0	0	0,64	0,36	0	0,046
3,75	81374	0	0,04	0	0,01	0,9	0	0	0,02	0,03	0	0	0,93	0,07	0,03
3,75	47784	0	0	0	0,1	0,8	0	0	0,1	0	0,1	0	0,8	0,1	0,046
3,75	5329	0	0,04	0	0	0,96	0	0	0	0	0,04	0,01	0,38	0,57	0,006
3,75	46607	0	0,04	0	0	0,96	0	0	0	0	0,04	0,94	0	0,02	0,024
3,75	47892	0	0,04	0	0,01	0,95	0	0	0	0	0	0,99	0,01	0	0,024
3,75	20708	0	0	0	0,43	0,55	0,01	0	0,01	0	0,97	0,01	0,01	0,01	0,044
3,75	98618	0	0,04	0	0,01	0,95	0	0	0	0	0	0	0,96	0,04	0,028
3,75	40260	0	0	0	0,15	0,84	0	0	0	0,01	0,15	0,84	0,01	0	0,044
3,75	75814	0	0,04	0	0,14	0,8	0,02	0	0	0	0,8	0,06	0	0,14	0,046
3,75	16122	0,02	0,04	0	0	0,93	0	0,01	0	0	0,03	0,04	0,93	0	0,042
3,75	40934	0	0,04	0	0	0,94	0,02	0	0	0	0,14	0,85	0,01	0	0,034
3,75	40975	0	0,04	0	0	0,92	0,04	0	0	0	0,04	0,04	0,92	0	0,02
3,75	10285	0,01	0,04	0	0	0,95	0	0	0	0	0	0,01	0	0,99	0,014
3,75	1739	0,02	0,04	0	0,02	0,92	0	0	0	0	0,92	0,04	0	0,04	0,046
3,75	51681	0	0,04	0,01	0	0,93	0,01	0	0,01	0	0	0,96	0,04	0	0,034
3,75	91156	0	0,04	0	0	0,96	0	0	0	0	0	0,04	0	0,96	0,014
3,75	18432	0	0,04	0,01	0	0,94	0	0	0	0,01	0,01	0,03	0,07	0,89	0,014
3,75	25568	0,05	0	0	0	0,87	0,01	0	0,07	0	0,02	0,05	0,87	0,06	0,032
3,75	83502	0	0,03	0	0,12	0,8	0,03	0	0,01	0,01	0,15	0,76	0,05	0,04	0,044

3,75	44140	0,01	0,04	0	0	0,81	0,14	0	0	0	0,96	0,04	0	0	0,036
3,75	58480	0	0	0,01	0,11	0,85	0,03	0	0	0	0,03	0,01	0,96	0	0,046
3,75	28120	0	0,04	0	0	0,94	0	0,02	0	0	0,94	0,02	0	0,04	0,046
3,75	58005	0	0,04	0	0	0,7	0,25	0	0,01	0	0,73	0,26	0	0,01	0,036
3,75	25903	0	0,04	0	0	0,95	0,01	0	0	0	0,2	0,76	0,04	0	0,024
3,75	100000	0,02	0,96	0	0	0	0,02	0	0	0	0	0,96	0	0,04	0,794
3,75	11816	0	0,04	0	0,01	0,95	0	0	0	0	0,39	0,01	0,59	0,01	0,044
3,75	69505	0	0,04	0	0,03	0,92	0	0,01	0	0	0,93	0	0,04	0,03	0,044
3,75	63427	0	0,01	0	0,07	0,92	0	0	0	0	0,06	0	0	0,94	0,046
4	22923	0	0,01	0	0,1	0,07	0,35	0	0,47	0	0	0,91	0,09	0	0,042
4	12436	0	0,04	0	0	0,96	0	0	0	0	0	0,03	0	0,97	0,012
4	100000	0,35	0,64	0	0	0,01	0	0	0	0	0,01	0,99	0	0	0,97
4	100000	0,16	0,79	0	0	0,05	0	0	0	0	0,05	0,79	0,16	0	0,71
4	14864	0	0,01	0	0,24	0,73	0,02	0	0	0	0,59	0,39	0,02	0	0,044
4	39894	0	0,03	0	0,09	0,87	0	0	0,01	0	0,86	0	0,04	0,1	0,044
4	13234	0	0,04	0	0	0,95	0	0	0	0,01	0,83	0,13	0,04	0	0,04
4	50028	0	0	0	0,13	0,86	0	0,01	0	0	0,1	0,03	0,87	0	0,048
4	55660	0	0,04	0	0	0,96	0	0	0	0	0	0,04	0,01	0,95	0,014
4	55878	0,02	0,04	0	0	0,92	0	0,01	0	0,01	0,93	0	0,05	0,02	0,046
4	100000	0	0,98	0,02	0	0	0	0	0	0	0,06	0,92	0,02	0	0,934
4	17417	0	0,04	0	0,03	0,92	0	0	0	0,01	0,02	0,96	0,02	0	0,032
4	22720	0	0,04	0	0	0,96	0	0	0	0	0	0,04	0,96	0	0,022
4	11744	0	0,04	0	0	0,96	0	0	0	0	0	0,04	0	0,96	0,004
4	58096	0	0,04	0	0	0,96	0	0	0	0	0	0,96	0	0,04	0,02
4	100000	0	1	0	0	0	0	0	0	0	0	1	0	0	0,948
4	40089	0,06	0	0	0	0,45	0,49	0	0	0	0,51	0,36	0	0,13	0,048
4	7718	0	0,04	0	0	0,96	0	0	0	0	0	0,96	0,04	0	0,026
4	12601	0	0,04	0	0	0,96	0	0	0	0	0	0,31	0	0,69	0,01
4	25779	0	0	0	0,11	0,88	0	0,01	0	0	0,03	0,97	0	0	0,046
4	100000	0,09	0,9	0	0	0	0	0	0,01	0	0,01	0,89	0,01	0,09	0,724
4	37118	0	0	0	0,19	0,03	0,78	0	0	0	0,79	0	0,03	0,18	0,046
4	47665	0	0	0	0,15	0,85	0	0	0	0	0,15	0,85	0	0	0,048
4	31581	0,01	0,04	0	0	0,95	0	0	0	0	0,01	0,01	0,91	0,07	0,036

4	5694	0	0,04	0	0	0,81	0,15	0	0	0	0,84	0,1	0	0,06	0,036
4	100000	0,27	0,7	0	0	0	0,03	0	0	0	0	1	0	0	0,914
4	45420	0,05	0	0	0	0,94	0	0	0	0,01	0	0,06	0	0,94	0,048
4	56553	0,05	0	0	0	0,83	0,02	0	0,1	0	0,1	0,05	0	0,85	0,04
4	81812	0	0,04	0	0	0,95	0	0,01	0	0	0,01	0	0,97	0,02	0,018
4	61021	0	0	0	0,08	0,46	0,41	0	0,05	0	0,26	0	0,23	0,51	0,046
4	28100	0	0,04	0	0	0,95	0	0	0,01	0	0,04	0	0	0,96	0,008
4	25802	0	0,04	0	0	0,96	0	0	0	0	0,96	0	0	0,04	0,042
4	77914	0	0,04	0	0,06	0,86	0,03	0	0,01	0	0,93	0,01	0,06	0	0,038
4	26495	0	0,04	0	0	0,95	0,01	0	0	0	0,82	0,04	0,14	0	0,024
4	93755	0,01	0,04	0	0,01	0,94	0	0	0	0	0,08	0,02	0	0,9	0,036
4	47200	0	0,04	0	0	0,95	0,01	0	0	0	0	0,04	0,95	0,01	0,02
4	100000	0	0,99	0	0	0	0	0	0	0,01	0	1	0	0	0,92
4	3144	0	0,04	0,01	0	0,95	0	0	0	0	0	0,05	0	0,95	0,014
4	27006	0	0,02	0,01	0,11	0	0,86	0	0	0	0,88	0,07	0,05	0	0,046
4	100000	0	0,54	0,46	0	0	0	0	0	0	0	1	0	0	0,954
4	19702	0	0,04	0	0	0	0	0	0,96	0	0	0,52	0	0,48	0,012
4	47159	0	0,04	0	0	0,88	0,06	0	0,02	0	0,02	0	0,06	0,92	0,006
4	28158	0	0,04	0	0	0,96	0	0	0	0	0	0,91	0,05	0,04	0,022
4	29787	0	0,04	0	0	0,96	0	0	0	0	0,94	0	0,01	0,05	0,042
4	100000	0,16	0,82	0	0	0	0	0	0,01	0,01	0,01	0,83	0	0,16	0,684
4	23342	0	0,04	0	0	0,95	0	0,01	0	0	0	0,05	0,95	0	0,022
4	59869	0	0,04	0,05	0	0,77	0,12	0	0,01	0,01	0,13	0,59	0,28	0	0,044
4	91624	0	0,04	0	0	0,94	0,02	0	0	0	0	0,01	0	0,99	0,018
4	23048	0	0,03	0,01	0	0,01	0,95	0	0	0	0,97	0	0,03	0	0,036
4	73991	0	0,04	0	0	0,92	0	0,03	0,01	0	0	0,41	0,57	0,02	0,044
4	62774	0,01	0,04	0	0	0,94	0	0	0,01	0	0	0,01	0,05	0,94	0,018
4	57395	0	0,04	0	0,01	0,95	0	0	0	0	0,04	0	0	0,96	0,018
4	36813	0	0,04	0	0,03	0,93	0	0	0	0	0,93	0,03	0,04	0	0,044
4	29527	0	0	0	0,13	0,61	0,07	0,09	0	0,1	0,69	0,15	0,16	0	0,036
4	20134	0	0	0	0,33	0,38	0,29	0	0	0	0,7	0	0,29	0,01	0,046
4	2703	0	0,04	0,02	0,01	0,92	0	0	0	0,01	0	0,46	0,52	0,02	0,038
4	12835	0	0,04	0,01	0	0,92	0	0	0,03	0	0,04	0,04	0	0,92	0,02

4	37569	0	0,04	0	0,01	0,89	0	0,03	0,02	0,01	0,06	0,07	0,86	0,01	0,046
4	48728	0	0,04	0	0	0,96	0	0	0	0	0	0,04	0,62	0,34	0,032
4	29785	0,05	0,04	0	0	0,89	0,02	0	0	0	0	0,07	0,76	0,17	0,048
4	28161	0	0,04	0	0,1	0,86	0	0	0	0	0,86	0,04	0	0,1	0,046
4	7061	0	0	0	0,09	0	0,15	0	0,27	0,49	0	0,85	0	0,15	0,046
4	89194	0,01	0,04	0	0	0,95	0	0	0	0	0	0	0,01	0,99	0,026
4	13230	0	0,04	0	0,07	0,85	0	0	0,04	0	0	0,04	0,92	0,04	0,038
4	11961	0	0,04	0	0	0,94	0,01	0	0	0,01	0	0,05	0,94	0,01	0,028
4	32150	0	0,04	0	0	0,92	0,02	0,02	0	0	0,06	0,92	0	0,02	0,026
4	80163	0,06	0	0,01	0	0,71	0,22	0	0	0	0,06	0	0,94	0	0,04
4	77764	0,05	0	0	0,01	0,93	0	0,01	0	0	0,94	0,05	0	0,01	0,048
4	18957	0	0,04	0	0	0,96	0	0	0	0	0	0,04	0	0,96	0,008
4	9206	0	0,04	0	0	0,95	0	0	0,01	0	0,97	0	0,01	0,02	0,03
4	17605	0	0,04	0	0	0,96	0	0	0	0	0	0,03	0,07	0,9	0,012
4	28328	0	0,04	0	0	0,31	0	0	0,6	0,05	0,05	0,09	0,05	0,81	0,014
4	100000	0	0,92	0,05	0	0	0	0	0,03	0	0	0,97	0,03	0	0,832
4	38740	0	0,04	0	0,01	0,95	0	0	0	0	0	0	0,95	0,05	0,026
4	16695	0	0,04	0	0,02	0,94	0	0	0	0	0	0,05	0,01	0,94	0,028
4	11597	0	0,04	0,06	0	0,8	0,1	0	0	0	0,9	0,03	0,01	0,06	0,046
4	100000	0,14	0,85	0	0	0	0	0,01	0	0	0,14	0,85	0	0,01	0,946
4	2103	0	0,04	0	0,03	0,88	0,05	0	0	0	0,88	0,07	0	0,05	0,048
4	68758	0	0,04	0	0	0,96	0	0	0	0	0	0	1	0	0,026
4	9510	0,05	0,04	0	0	0	0	0	0,91	0	0	0,96	0	0,04	0,046
4	20715	0	0	0	0,07	0,93	0	0	0	0	0	0	0,07	0,93	0,046
4	86716	0,06	0	0	0	0,92	0	0	0,02	0	0,98	0	0,02	0	0,048
4	29741	0	0,04	0	0	0,78	0	0,01	0,17	0	0,2	0,01	0,04	0,75	0,022
4	37757	0	0,04	0	0	0,93	0,03	0	0	0	0,03	0,93	0,03	0,01	0,032
4	25797	0	0,04	0	0	0,95	0	0	0	0,01	0,01	0,04	0	0,95	0,006
4	49722	0	0,04	0	0	0,96	0	0	0	0	0	0,04	0,2	0,76	0,012
4	51603	0	0,04	0	0	0,94	0	0	0,01	0,01	0	0,94	0,04	0,02	0,02
4	100000	0,2	0,62	0	0,01	0,12	0,04	0,01	0	0	0	0,67	0,21	0,12	0,528
4	27801	0	0,04	0	0,01	0,94	0	0,01	0	0	0,02	0	0	0,98	0,022
4	10629	0,04	0,02	0,01	0	0,93	0	0	0	0	0,7	0,26	0,04	0	0,048

4	77648	0	0,04	0	0,16	0,79	0,01	0	0	0	0,79	0,2	0,01	0	0,04
4	6211	0,04	0	0,01	0	0,94	0	0,01	0	0	0,01	0,05	0,93	0,01	0,048
4	35311	0	0,04	0	0	0,96	0	0	0	0	0	0	0,99	0,01	0,032
4	13599	0,01	0,04	0	0	0,95	0	0	0	0	0	0,99	0,01	0	0,026
4	12711	0,06	0	0	0,01	0,93	0	0	0	0	0,8	0,05	0,15	0	0,048
4	11651	0,01	0,04	0	0,07	0,87	0	0	0,01	0	0,08	0,03	0,88	0,01	0,046
4	71318	0	0,04	0	0	0,96	0	0	0	0	0,01	0	0,03	0,96	0,008
4	19542	0	0,04	0	0,02	0,94	0	0	0	0	0	0,94	0,02	0,04	0,03
4	21846	0,02	0,03	0	0,05	0,03	0,87	0	0	0	0,9	0,05	0,03	0,02	0,048
4	20413	0,01	0,01	0,01	0,07	0,88	0	0	0,02	0	0,06	0	0,06	0,88	0,048
4,25	61701	0	0,04	0	0	0,95	0,01	0	0	0	0	0,02	0,98	0	0,022
4,25	23865	0	0,04	0	0	0,96	0	0	0	0	0,96	0,04	0	0	0,04
4,25	17935	0	0,04	0	0	0,89	0,06	0	0,01	0	0,07	0	0,89	0,04	0,03
4,25	28836	0	0,04	0,01	0	0,92	0,01	0,02	0	0	0,01	0	0,2	0,79	0,034
4,25	1793	0	0,04	0	0	0,96	0	0	0	0	0	0,19	0,81	0	0,016
4,25	63322	0	0,04	0	0	0,95	0	0	0	0,01	0,01	0,93	0,04	0,02	0,016
4,25	22524	0	0,04	0	0	0,84	0	0,06	0,06	0	0,1	0,9	0	0	0,044
4,25	13650	0,03	0,02	0	0	0,95	0	0	0	0	0,92	0,05	0	0,03	0,046
4,25	12067	0,05	0,02	0	0	0,88	0,05	0	0	0	0,72	0,05	0,17	0,06	0,048
4,25	47731	0,02	0,01	0	0	0,04	0	0,06	0,5	0,37	0,02	0,55	0,37	0,06	0,038
4,25	100000	0,73	0,18	0,09	0	0	0	0	0	0	0,07	0,81	0,03	0,09	0,93
4,25	13190	0	0,04	0	0	0,96	0	0	0	0	0,96	0,01	0,03	0	0,036
4,25	41484	0,01	0,04	0	0	0,93	0,01	0	0,01	0	0,01	0,95	0	0,04	0,032
4,25	48175	0,01	0,04	0	0	0,86	0	0	0	0,09	0,62	0,04	0,25	0,09	0,038
4,25	69495	0,05	0,02	0	0,01	0,92	0	0	0	0	0,01	0,05	0,02	0,92	0,048
4,25	100000	0	0,98	0	0	0	0,02	0	0	0	0,01	0,98	0	0,01	0,874
4,25	18578	0	0,04	0	0	0,95	0	0,01	0	0	0,02	0,95	0	0,03	0,032
4,25	15134	0	0,04	0	0	0,95	0	0,01	0	0	0	0	0,09	0,91	0,02
4,25	17885	0	0,04	0	0,01	0,95	0	0	0	0	0,96	0,04	0	0	0,038
4,25	31350	0,02	0,04	0	0	0,94	0	0	0	0	0,94	0,06	0	0	0,036
4,25	39325	0	0,04	0	0	0,93	0	0	0,02	0,01	0	0,06	0	0,94	0,01
4,25	18643	0,04	0,02	0	0	0	0,07	0	0,87	0	0,02	0,05	0,88	0,05	0,042
4,25	12721	0	0	0,02	0,07	0,8	0	0,01	0,1	0	0,03	0	0,08	0,89	0,04

4,25	100000	0	1	0	0	0	0	0	0	0	0	1	0	0	0,98
4,25	59926	0,03	0,04	0	0	0,89	0	0,02	0	0,02	0,06	0,02	0,03	0,89	0,046
4,25	82268	0,01	0,04	0	0	0,85	0,1	0	0	0	0,05	0,08	0,85	0,02	0,032
4,25	100000	0,03	0,95	0	0	0	0	0,01	0	0,01	0,04	0,95	0,01	0	0,884
4,25	30588	0	0,04	0	0	0,96	0	0	0	0	0	0,04	0,96	0	0,028
4,25	20772	0,03	0	0	0,05	0,91	0	0	0,01	0	0	0,03	0,92	0,05	0,04
4,25	5982	0,01	0,03	0	0,16	0	0,77	0,01	0	0,02	0,96	0,02	0,01	0,01	0,048
4,25	94556	0	0,04	0	0	0,94	0	0,02	0	0	0	0,04	0,94	0,02	0,036
4,25	58066	0,01	0,04	0,02	0	0,93	0	0	0	0	0	0,05	0,02	0,93	0,022
4,25	11409	0,05	0	0,01	0	0,22	0,38	0	0,34	0	0,39	0,34	0	0,27	0,044
4,25	24889	0	0,04	0	0	0,96	0	0	0	0	0,03	0	0	0,97	0,01
4,25	42386	0	0,04	0	0	0,96	0	0	0	0	0,99	0	0,01	0	0,038
4,25	59795	0	0,04	0	0	0,96	0	0	0	0	0	0,04	0,96	0	0,026
4,25	21760	0	0,04	0,02	0	0,94	0	0	0	0	0	0	0,96	0,04	0,034
4,25	45373	0	0,04	0	0	0,96	0	0	0	0	0	0	0,04	0,96	0,018
4,25	100000	0,02	0,9	0,06	0	0	0	0	0,02	0	0,09	0,91	0	0	0,93
4,25	100000	0	0,98	0	0	0	0,01	0	0	0,01	0,01	0,89	0,09	0,01	0,714
4,25	31018	0	0,04	0	0	0,93	0,01	0	0,02	0	0	0,06	0,94	0	0,022
4,25	43994	0	0,04	0	0	0,94	0	0	0,02	0	0,05	0,04	0,02	0,89	0,016
4,25	2018	0	0,04	0	0	0,94	0,02	0	0	0	0	0,04	0,94	0,02	0,022
4,25	25238	0	0	0	0,1	0	0,66	0	0,23	0,01	0	0,25	0,09	0,66	0,046
4,25	76035	0	0,04	0	0	0,96	0	0	0	0	0	0	1	0	0,022
4,25	100000	0,46	0,11	0	0,15	0,03	0,07	0	0	0,18	0	0,65	0,35	0	0,582
4,25	17582	0	0,04	0	0	0,96	0	0	0	0	0,04	0	0,84	0,12	0,026
4,25	62824	0	0	0	0,09	0,5	0,23	0,05	0,13	0	0,09	0,5	0,36	0,05	0,046
4,25	27720	0	0,04	0	0	0,96	0	0	0	0	0	0	1	0	0,022
4,25	71823	0	0,04	0	0,02	0,92	0	0	0,02	0	0,96	0,02	0,02	0	0,048
4,25	55185	0,01	0,04	0	0	0,93	0	0,01	0	0,01	0,93	0	0,02	0,05	0,036
4,25	5280	0	0,04	0	0	0,96	0	0	0	0	0,01	0,04	0,95	0	0,014
4,25	33639	0	0,04	0	0	0,96	0	0	0	0	0	0,04	0	0,96	0,006
4,25	61762	0	0,04	0,01	0	0,92	0	0,03	0	0	0,92	0	0	0,08	0,038
4,25	86092	0	0,04	0	0	0,93	0	0	0,03	0	0	0,03	0,96	0,01	0,022
4,25	84286	0	0	0	0,15	0,13	0,24	0	0,48	0	0,12	0,47	0,4	0,01	0,048

4,25	5166	0	0	0	0,1	0,9	0	0	0	0	0	0	0,53	0,47	0,046
4,25	9570	0	0,04	0	0,07	0,89	0	0	0	0	0,01	0,06	0,89	0,04	0,046
4,25	7719	0	0,04	0	0,01	0,93	0,02	0	0	0	0,01	0,05	0,92	0,02	0,022
4,25	29583	0,01	0,04	0	0	0,95	0	0	0	0	0	0,02	0,98	0	0,034
4,25	77982	0	0,04	0	0	0,96	0	0	0	0	0	0,96	0	0,04	0,02
4,25	19188	0	0	0	0,15	0,83	0,01	0,01	0	0	0	0	1	0	0,044
4,25	85249	0,05	0	0	0,02	0	0,77	0	0,14	0,02	0,93	0	0,07	0	0,044
4,25	6398	0,03	0,04	0	0	0,93	0	0	0	0	0	0,06	0	0,94	0,04
4,25	81344	0	0	0	0,15	0,85	0	0	0	0	0,15	0,85	0	0	0,044
4,25	100000	0,16	0,84	0	0	0	0	0	0	0	0	0,84	0	0,16	0,716
4,25	36625	0,04	0	0,01	0	0,95	0	0	0	0	0,95	0,05	0	0	0,046
4,25	22123	0	0	0	0,3	0,62	0	0	0,08	0	0,74	0,07	0,19	0	0,046
4,25	42596	0,01	0,04	0	0	0,9	0,01	0,03	0,01	0	0,05	0,93	0,02	0	0,048
4,25	100000	0	0,95	0,05	0	0	0	0	0	0	0	0,97	0,03	0	0,868
4,25	8739	0,01	0,04	0	0	0,95	0	0	0	0	0,04	0	0	0,96	0,018
4,25	12116	0,01	0,04	0	0	0	0	0	0,95	0	0,01	0,95	0,04	0	0,04
4,25	46144	0,04	0,04	0	0	0,85	0,05	0	0,02	0	0,94	0,04	0,02	0	0,04
4,25	85272	0	0	0	0,12	0,81	0	0	0,07	0	0,04	0,8	0,12	0,04	0,044
4,25	38386	0	0,04	0	0	0,94	0,01	0,01	0	0	0,02	0,01	0,95	0,02	0,034
4,25	85863	0	0	0,02	0,09	0,47	0,4	0	0	0,02	0	0,47	0,11	0,42	0,044
4,25	80952	0,01	0,04	0,02	0	0,93	0	0	0	0	0	0,02	0,04	0,94	0,03
4,25	100000	0,02	0,98	0	0	0	0	0	0	0	0,02	0,98	0	0	0,972
4,25	33475	0	0,04	0,01	0	0,9	0,05	0	0	0	0,1	0,04	0,81	0,05	0,028
4,25	33502	0	0,04	0	0	0,96	0	0	0	0	0,01	0,04	0	0,95	0,014
4,25	25951	0	0	0	0,16	0	0	0	0,83	0,01	0,11	0,16	0,73	0	0,048
4,25	100000	0	1	0	0	0	0	0	0	0	0	1	0	0	0,96
4,25	9660	0	0	0	0,15	0,8	0,04	0	0,01	0	0,55	0,01	0,44	0	0,042
4,25	93516	0	0,04	0,01	0	0,88	0,07	0	0	0	0,95	0	0,05	0	0,038
4,25	53482	0	0	0	0,19	0,8	0	0	0	0,01	0,23	0,77	0	0	0,048
4,25	100000	0	1	0	0	0	0	0	0	0	0	1	0	0	0,968
4,25	55206	0	0,04	0,01	0	0	0	0	0,95	0	0	0	0	1	0,014
4,25	54502	0	0,04	0	0	0,96	0	0	0	0	0,02	0,94	0	0,04	0,022
4,25	3034	0	0,04	0,01	0,02	0,93	0	0	0	0	0,01	0,98	0	0,01	0,044

4,25	38822	0,01	0	0	0,15	0,8	0	0,03	0	0,01	0,8	0,01	0,04	0,15	0,04
4,25	49223	0,01	0,04	0,01	0	0,93	0	0	0	0,01	0,01	0	0,97	0,02	0,042
4,25	48901	0	0,04	0,01	0	0,94	0	0,01	0	0	0,05	0,01	0,94	0	0,038
4,25	30748	0	0,01	0	0,08	0,75	0,08	0	0,07	0,01	0,01	0,15	0,75	0,09	0,03
4,25	100000	0,13	0,85	0	0,02	0	0	0	0	0	0,12	0,86	0,02	0	0,898
4,25	15099	0	0,04	0,01	0	0,94	0	0	0	0,01	0,95	0	0	0,05	0,044
4,25	18024	0	0,04	0	0	0,95	0	0,01	0	0	0,54	0,05	0,38	0,03	0,032
4,25	46289	0,01	0,04	0	0	0,85	0,02	0	0,07	0,01	0	0,12	0,85	0,03	0,038
4,25	15107	0	0,04	0	0	0,96	0	0	0	0	0	0,04	0	0,96	0,008
4,25	8478	0	0,04	0	0	0,96	0	0	0	0	0	0,02	0	0,98	0,014
4,25	69249	0,06	0	0	0	0,83	0,1	0	0	0,01	0,06	0,01	0,93	0	0,038
4,5	57119	0	0,04	0	0	0,96	0	0	0	0	0,93	0,03	0,04	0	0,03
4,5	11656	0	0,04	0	0,06	0,9	0	0	0	0	0	0,95	0	0,05	0,048
4,5	12901	0	0,04	0	0,04	0,61	0,31	0	0	0	0,92	0,03	0,04	0,01	0,032
4,5	60244	0,01	0,04	0	0,01	0,94	0	0	0	0	0,01	0,04	0,94	0,01	0,032
4,5	47429	0	0,04	0	0,01	0,84	0	0,01	0,09	0,01	0,04	0,09	0	0,87	0,026
4,5	26807	0	0	0	0,1	0,04	0,04	0	0,64	0,18	0,18	0,73	0,05	0,04	0,048
4,5	100000	0,08	0,82	0,08	0,01	0	0	0,01	0	0	0,03	0,9	0,07	0	0,76
4,5	64121	0	0,04	0	0	0,96	0	0	0	0	0	0,04	0	0,96	0,01
4,5	49638	0	0,04	0	0,02	0,94	0	0	0	0	0,3	0,66	0,04	0	0,034
4,5	10380	0	0,04	0	0	0,94	0,02	0	0	0	0,94	0,02	0	0,04	0,042
4,5	51230	0	0	0	0,21	0,78	0	0	0	0,01	0,46	0,21	0,33	0	0,048
4,5	100000	0	1	0	0	0	0	0	0	0	0	1	0	0	0,962
4,5	73696	0	0,04	0	0	0,96	0	0	0	0	0	0	0	1	0,01
4,5	57027	0,01	0,04	0	0	0,95	0	0	0	0	0	0	0,96	0,04	0,036
4,5	37968	0	0,04	0	0,02	0,92	0,01	0,01	0	0	0	0,6	0,01	0,39	0,048
4,5	35862	0	0,01	0	0	0	0	0,17	0,04	0,78	0,78	0,15	0,02	0,05	0,048
4,5	38555	0	0,04	0	0	0,95	0,01	0	0	0	0,01	0,04	0,95	0	0,028
4,5	14208	0	0	0	0,09	0,38	0,53	0	0	0	0,22	0	0,27	0,51	0,044
4,5	68933	0,02	0,04	0	0,03	0,91	0	0	0	0	0,07	0	0,91	0,02	0,048
4,5	19848	0	0,04	0	0	0,96	0	0	0	0	0	0,04	0	0,96	0,01
4,5	94784	0	0,04	0	0,02	0,94	0	0	0	0	0,04	0	0,02	0,94	0,036
4,5	56674	0,01	0,04	0	0	0,95	0	0	0	0	0,04	0	0,96	0	0,048

4,5	96518	0,03	0,04	0	0,01	0,88	0	0	0,02	0,02	0,05	0,73	0,22	0	0,046
4,5	24510	0	0,04	0,01	0	0	0	0	0,95	0	0	0	1	0	0,022
4,5	21504	0,01	0,04	0	0	0,94	0	0	0	0,01	0,94	0,02	0	0,04	0,028
4,5	60639	0	0,04	0	0	0,92	0	0	0,04	0	0	0,03	0,92	0,05	0,036
4,5	22295	0,01	0,04	0	0	0,92	0,03	0	0	0	0,42	0,04	0,54	0	0,024
4,5	8975	0,05	0,02	0	0,01	0,91	0	0	0	0,01	0,92	0,02	0,01	0,05	0,048
4,5	24978	0	0,04	0	0,04	0	0,91	0	0,01	0	0,95	0,01	0,04	0	0,04
4,5	60456	0	0	0	0	0	0,26	0,09	0,64	0,01	0,01	0,64	0	0,35	0,048
4,5	6463	0	0,04	0	0,01	0,95	0	0	0	0	0,01	0,05	0	0,94	0,016
4,5	19948	0	0,01	0	0,08	0,85	0,04	0,02	0	0	0	0,95	0,05	0	0,046
4,5	38837	0	0,04	0	0	0,96	0	0	0	0	0	0	0,04	0,96	0,014
4,5	39331	0	0,02	0,02	0,1	0,85	0	0	0,01	0	0,12	0,02	0,85	0,01	0,048
4,5	25755	0	0,04	0,02	0	0,92	0	0,01	0,01	0	0	0,04	0,94	0,02	0,03
4,5	72515	0	0,04	0	0,01	0	0,95	0	0	0	0,06	0,11	0,83	0	0,026
4,5	17922	0	0,04	0,02	0	0,93	0	0	0,01	0	0,02	0,04	0,01	0,93	0,01
4,5	50369	0	0,04	0	0	0,95	0	0	0	0,01	0	0,1	0	0,9	0,012
4,5	18119	0	0,04	0	0,01	0,94	0	0	0,01	0	0	0,04	0,01	0,95	0,014
4,5	54141	0	0,04	0	0	0,94	0	0	0,01	0,01	0,01	0,01	0,01	0,97	0
4,5	8626	0	0,04	0	0	0,84	0,1	0,02	0	0	0,14	0,86	0	0	0,044
4,5	43421	0	0,04	0	0	0,95	0	0	0	0,01	0	0,04	0,95	0,01	0,034
4,5	100000	0	1	0	0	0	0	0	0	0	0	1	0	0	0,938
4,5	56335	0	0,04	0	0,04	0,91	0	0	0,01	0	0,92	0,01	0,04	0,03	0,034
4,5	36284	0	0,02	0	0,1	0,88	0	0	0	0	0,09	0	0,88	0,03	0,046
4,5	7804	0,05	0,04	0	0	0,91	0	0	0	0	0	0,99	0,01	0	0,046
4,5	69457	0	0,04	0	0	0,9	0	0	0,06	0	0,06	0,01	0,89	0,04	0,038
4,5	22994	0	0,04	0	0	0,71	0,2	0	0	0,05	0,71	0,28	0	0,01	0,036
4,5	9162	0	0,04	0,01	0	0,94	0,01	0	0	0	0,01	0	0,06	0,93	0,018
4,5	74603	0	0,04	0	0	0,95	0	0	0,01	0	0	0,02	0,95	0,03	0,022
4,5	8720	0,03	0,03	0,01	0	0,93	0	0	0	0	0,52	0,06	0,41	0,01	0,046
4,5	69710	0	0	0	0,06	0,94	0	0	0	0	0	0	0	1	0,04
4,5	27216	0	0	0	0,14	0,86	0	0	0	0	0,1	0,86	0	0,04	0,046
4,5	60278	0	0,04	0	0	0,96	0	0	0	0	0	0,96	0,04	0	0,024
4,5	100000	0,45	0,49	0	0,04	0	0,01	0	0	0,01	0,41	0,44	0,1	0,05	0,872

4,5	33341	0	0,04	0,01	0,06	0,85	0	0,04	0	0	0,05	0,85	0,06	0,04	0,046
4,5	23306	0	0,02	0	0,18	0,79	0,01	0	0	0	0,77	0,18	0	0,05	0,042
4,5	4320	0	0,04	0	0	0,96	0	0	0	0	0	0,04	0	0,96	0,014
4,5	13918	0	0	0	0,39	0,61	0	0	0	0	0,99	0	0	0,01	0,044
4,5	100000	0	0,98	0	0,01	0	0	0	0	0,01	0,01	0,98	0	0,01	0,896
4,5	74379	0,05	0	0	0,02	0,9	0	0,02	0,01	0	0,01	0,94	0,02	0,03	0,048
4,5	31190	0	0,04	0	0,02	0,94	0	0	0	0	0,59	0,04	0,37	0	0,032
4,5	45272	0	0	0	0,36	0,64	0	0	0	0	0,96	0,04	0	0	0,042
4,5	14093	0	0,04	0,01	0,01	0,93	0	0	0,01	0	0,01	0,05	0	0,94	0,022
4,5	20565	0	0,04	0	0,01	0	0,95	0	0	0	0,95	0	0,05	0	0,042
4,5	31335	0	0,04	0	0,01	0,94	0	0,01	0	0	0,05	0,81	0,14	0	0,038
4,5	44075	0,01	0	0,08	0,01	0,49	0	0	0,41	0	0,13	0,37	0,5	0	0,038
4,5	39857	0	0,04	0	0	0,96	0	0	0	0	0	0	0,73	0,27	0,024
4,5	100000	0	0,94	0	0	0,05	0	0,01	0	0	0	1	0	0	0,816
4,5	66822	0	0,04	0	0	0,95	0	0	0,01	0	0	0,19	0,8	0,01	0,024
4,5	100000	0	1	0	0	0	0	0	0	0	0	0,99	0	0,01	0,944
4,5	12135	0	0,04	0	0	0,95	0	0	0,01	0	0	0,05	0,95	0	0,028
4,5	90996	0,01	0,04	0	0,03	0,92	0	0	0	0	0	0,95	0,03	0,02	0,046
4,5	22812	0	0,03	0	0,06	0,89	0,02	0	0	0	0,01	0	0,05	0,94	0,046
4,5	96518	0	0,04	0	0	0,96	0	0	0	0	0	0,96	0,04	0	0,034
4,5	45734	0	0,01	0	0,1	0,88	0	0	0,01	0	0	0,14	0,86	0	0,046
4,5	19742	0,06	0,01	0	0	0,93	0	0	0	0	0,05	0,94	0	0,01	0,044
4,5	41195	0	0,04	0	0	0,96	0	0	0	0	0	0	0	1	0,008
4,5	9603	0	0,04	0	0	0,96	0	0	0	0	0	0,03	0	0,97	0,008
4,5	50925	0,01	0,04	0	0,03	0,92	0	0	0	0	0,01	0,04	0,8	0,15	0,048
4,5	21452	0	0,04	0	0	0,96	0	0	0	0	0	0,96	0	0,04	0,022
4,5	100000	0	0,98	0	0,01	0	0	0,01	0	0	0,35	0,64	0	0,01	0,912
4,5	3644	0	0,04	0	0,04	0,92	0	0	0	0	0	1	0	0	0,04
4,5	100000	0,06	0,94	0	0	0	0	0	0	0	0	0,88	0	0,12	0,678
4,5	100000	0,27	0,72	0	0	0	0,01	0	0	0	0,27	0,73	0	0	0,972
4,5	31585	0	0,04	0	0,06	0,9	0	0	0	0	0,06	0,94	0	0	0,046
4,5	12451	0	0,04	0	0	0,96	0	0	0	0	0	0,04	0	0,96	0,014
4,5	7944	0	0,04	0	0	0,96	0	0	0	0	0	0,04	0,91	0,05	0,024

4,5	58179	0	0,01	0	0,06	0,91	0	0,02	0	0	0,06	0,12	0	0,82	0,044
4,5	35768	0	0	0	0,08	0	0,55	0	0,29	0,08	0	1	0	0	0,048
4,5	20047	0	0,01	0	0,14	0,85	0	0	0	0	0,64	0,01	0	0,35	0,042
4,5	24303	0	0,04	0,01	0	0,95	0	0	0	0	0,7	0	0,13	0,17	0,038
4,5	47071	0	0,04	0	0	0,85	0,02	0	0,09	0	0,87	0	0,04	0,09	0,044
4,5	47390	0	0,04	0	0,01	0,94	0	0,01	0	0	0	0,92	0,02	0,06	0,024
4,5	84768	0	0,04	0	0,01	0,95	0	0	0	0	0	0,18	0	0,82	0,012
4,5	22716	0	0,04	0	0	0,96	0	0	0	0	0	0	1	0	0,02
4,5	82066	0	0,04	0	0	0,95	0	0	0,01	0	0	0,04	0	0,96	0,018
4,5	56210	0	0,04	0	0,01	0,93	0,01	0	0,01	0	0,01	0,04	0,94	0,01	0,026
4,5	34234	0	0,04	0	0	0,96	0	0	0	0	0	0,96	0	0,04	0,022
4,5	100000	0,76	0,18	0	0,06	0	0	0	0	0	0,76	0,18	0,06	0	0,93
4,75	92880	0,04	0	0,01	0	0,95	0	0	0	0	0,05	0	0	0,95	0,04
4,75	13748	0	0	0	0,07	0,92	0	0	0,01	0	0,08	0	0	0,92	0,042
4,75	30542	0	0,04	0	0	0,94	0,01	0,01	0	0	0,02	0,05	0,92	0,01	0,03
4,75	13093	0	0,03	0,01	0,05	0	0,33	0	0,58	0	0,03	0,63	0,33	0,01	0,038
4,75	96158	0,04	0,04	0	0	0,92	0	0	0	0	0	0,94	0,04	0,02	0,048
4,75	67152	0,04	0,03	0	0	0	0,43	0	0,5	0	0,5	0,03	0,42	0,05	0,044
4,75	12670	0	0,04	0	0	0,94	0	0	0,01	0,01	0,02	0,04	0,94	0	0,032
4,75	100000	0,81	0,19	0	0	0	0	0	0	0	0,81	0,19	0	0	1
4,75	14664	0	0,04	0	0	0,93	0	0,01	0,02	0	0,04	0	0,95	0,01	0,032
4,75	13297	0,02	0,04	0	0	0,92	0	0	0	0,02	0,02	0,05	0	0,93	0,038
4,75	52089	0	0,03	0,01	0,05	0,91	0	0	0	0	0,84	0,09	0,03	0,04	0,048
4,75	100000	0,02	0,96	0	0	0	0	0	0,01	0,01	0,16	0,8	0,02	0,02	0,846
4,75	14626	0	0,04	0	0,11	0,85	0	0	0	0	0,96	0,03	0,01	0	0,036
4,75	4867	0	0	0	0,28	0,63	0,09	0	0	0	0,87	0	0,13	0	0,046
4,75	87842	0	0	0	0,11	0,78	0	0	0,11	0	0,18	0	0,04	0,78	0,048
4,75	54912	0	0	0	0,14	0,86	0	0	0	0	0	0	1	0	0,044
4,75	67980	0,07	0	0	0	0,8	0,08	0	0,05	0	0,11	0,09	0	0,8	0,046
4,75	82891	0	0,04	0	0	0,95	0	0,01	0	0	0	0,03	0,88	0,09	0,026
4,75	21390	0	0,04	0	0	0,95	0	0	0,01	0	0	0,34	0,01	0,65	0,014
4,75	57610	0	0,02	0	0,1	0,85	0,03	0	0	0	0,01	0,88	0,1	0,01	0,03
4,75	22587	0	0,04	0	0,08	0,88	0	0	0	0	0,06	0,94	0	0	0,032

4,75	41885	0	0	0	0,08	0,84	0	0	0,08	0	0,07	0	0,1	0,83	0,036
4,75	100000	0,21	0,78	0	0	0	0	0,01	0	0	0	0,92	0,08	0	0,788
4,75	34506	0,05	0	0	0,01	0,92	0,01	0	0,01	0	0,01	0,06	0,92	0,01	0,048
4,75	15023	0	0,04	0	0	0,77	0,19	0	0	0	0,77	0	0,07	0,16	0,04
4,75	36870	0	0,04	0	0	0,94	0,02	0	0	0	0,02	0,03	0,95	0	0,02
4,75	10948	0	0,04	0	0	0,59	0,37	0	0	0	0,04	0	0,96	0	0,024
4,75	55453	0,06	0	0	0,01	0,92	0	0	0	0,01	0,01	0,06	0,92	0,01	0,04
4,75	21918	0,04	0	0	0,02	0,93	0	0	0,01	0	0	0,05	0,04	0,91	0,042
4,75	17014	0,02	0	0,06	0,01	0,1	0,81	0	0	0	0,89	0,1	0	0,01	0,042
4,75	28906	0,01	0,04	0	0	0,95	0	0	0	0	0,01	0	0	0,99	0,026
4,75	54048	0,07	0	0	0	0,93	0	0	0	0	0,98	0	0,02	0	0,046
4,75	100000	0	1	0	0	0	0	0	0	0	0,04	0,96	0	0	0,986
4,75	57928	0	0,04	0	0	0,96	0	0	0	0	0	0,96	0,04	0	0,026
4,75	43203	0	0	0	0,15	0,84	0,01	0	0	0	0,15	0,84	0,01	0	0,04
4,75	34888	0	0	0,02	0,07	0,91	0	0	0	0	0,02	0	0	0,98	0,042
4,75	100000	0,41	0,34	0	0	0	0,01	0	0	0,24	0	0,34	0,25	0,41	0,558
4,75	27554	0	0	0	0,4	0	0,59	0	0	0,01	0,99	0	0	0,01	0,04
4,75	100000	0,01	0,99	0	0	0	0	0	0	0	0	1	0	0	0,976
4,75	15807	0	0	0	0,08	0,85	0	0	0,07	0	0,12	0,03	0	0,85	0,042
4,75	48439	0	0,04	0	0	0,95	0	0,01	0	0	0,96	0,04	0	0	0,034
4,75	100000	0	1	0	0	0	0	0	0	0	0	1	0	0	0,96
4,75	2440	0,01	0,04	0	0	0,95	0	0	0	0	0,01	0,03	0,93	0,03	0,028
4,75	70126	0	0	0	0,08	0,92	0	0	0	0	0,08	0	0	0,92	0,048
4,75	35421	0,05	0	0	0	0,95	0	0	0	0	0	0,05	0,95	0	0,034
4,75	73438	0,03	0,03	0	0	0,94	0	0	0	0	0,83	0,03	0,14	0	0,044
4,75	47041	0	0,04	0	0	0,92	0,04	0	0	0	0,92	0	0,04	0,04	0,04
4,75	8356	0	0	0	0,07	0,92	0	0,01	0	0	0,07	0,01	0	0,92	0,046
4,75	23945	0	0,04	0	0	0,94	0,02	0	0	0	0,94	0	0,04	0,02	0,042
4,75	27714	0,03	0	0	0,06	0,01	0,9	0	0	0	0,62	0,02	0,28	0,08	0,038
4,75	53923	0	0	0	0,23	0,27	0,5	0	0	0	0,63	0,01	0,34	0,02	0,046
4,75	23587	0	0	0	0,39	0,61	0	0	0	0	1	0	0	0	0,048
4,75	100000	0,03	0,97	0	0	0	0	0	0	0	0,03	0,97	0	0	0,986
4,75	23825	0,05	0	0	0,02	0,88	0,04	0	0	0,01	0	0,96	0,04	0	0,046

4,75	39042	0	0,04	0	0	0,93	0,01	0	0,02	0	0	0,01	0,03	0,96	0,008
4,75	39816	0	0,04	0	0,01	0,41	0,54	0	0	0	0,98	0	0,02	0	0,036
4,75	83084	0	0	0	0,13	0,85	0	0,02	0	0	0,13	0,85	0	0,02	0,048
4,75	14238	0	0,04	0,01	0,01	0,93	0	0	0,01	0	0,11	0,02	0,04	0,83	0,02
4,75	30173	0	0,04	0	0,03	0,93	0	0	0	0	0,03	0	0,93	0,04	0,044
4,75	70436	0	0	0	0,07	0,74	0	0,01	0,16	0,02	0	0,01	0,25	0,74	0,044
4,75	3222	0	0	0	0,09	0,91	0	0	0	0	0,07	0,27	0	0,66	0,048
4,75	10852	0	0,03	0	0,15	0,82	0	0	0	0	0,82	0,07	0	0,11	0,042
4,75	28543	0	0	0	0,07	0,93	0	0	0	0	0	0,07	0	0,93	0,048
4,75	15531	0,01	0	0	0,29	0,03	0,67	0	0	0	0,96	0	0,01	0,03	0,048
4,75	18544	0,03	0	0	0,02	0	0	0,02	0,82	0,11	0,08	0,79	0	0,13	0,048
4,75	14249	0	0	0,01	0,09	0	0	0	0,9	0	0	0,9	0,1	0	0,044
4,75	28319	0	0,04	0	0	0,91	0,04	0	0	0,01	0	0,05	0,91	0,04	0,016
4,75	100000	0,28	0,68	0,04	0	0	0	0	0	0	0	0,68	0,04	0,28	0,616
4,75	20186	0	0	0	0,05	0	0	0	0,04	0,91	0	0,05	0	0,95	0,046
4,75	32504	0	0,03	0	0,07	0,9	0	0	0	0	0,03	0	0	0,97	0,048
4,75	9141	0,01	0,04	0	0	0,95	0	0	0	0	0,01	0,04	0	0,95	0,022
4,75	4114	0,02	0,03	0	0,04	0,86	0	0	0,05	0	0,05	0,08	0,86	0,01	0,042
4,75	95461	0	0,04	0	0	0,96	0	0	0	0	0,96	0,04	0	0	0,048
4,75	40112	0	0	0	0,36	0,63	0,01	0	0	0	0,98	0	0,01	0,01	0,038
4,75	13474	0	0,04	0	0	0,96	0	0	0	0	0	0	1	0	0,02
4,75	26197	0	0	0	0,1	0	0,01	0	0,75	0,14	0	0,75	0,23	0,02	0,044
4,75	72244	0	0,01	0	0,1	0,18	0,1	0,01	0,54	0,06	0,06	0,13	0,71	0,1	0,044
4,75	31579	0	0,04	0	0	0,96	0	0	0	0	0	0,04	0	0,96	0,02
4,75	15533	0	0,04	0	0	0,95	0	0	0,01	0	0	0,04	0,01	0,95	0,008
4,75	73346	0	0,04	0	0	0,96	0	0	0	0	0,02	0	0,98	0	0,026
4,75	27030	0	0,04	0,01	0,03	0,92	0	0	0	0	0	0,01	0,96	0,03	0,042
4,75	100000	0,06	0,91	0	0,03	0	0	0	0	0	0,01	0,93	0	0,06	0,746
4,75	11683	0	0,04	0	0,01	0,93	0,02	0	0	0	0,94	0	0,02	0,04	0,036
4,75	12039	0	0	0	0,11	0,72	0	0	0,01	0,16	0,01	0,11	0,88	0	0,046
4,75	64221	0	0,04	0	0	0,09	0	0	0,87	0	0	0,46	0,5	0,04	0,02
4,75	47918	0	0,04	0	0,01	0,95	0	0	0	0	0,04	0,01	0,95	0	0,018
4,75	12489	0,01	0,04	0	0	0,95	0	0	0	0	0	0,01	0	0,99	0,022

4,75	3952	0	0,04	0,01	0,01	0,94	0	0	0	0	0,01	0,05	0,94	0	0,036
4,75	40851	0	0,04	0	0	0,95	0	0	0,01	0	0,12	0,04	0,84	0	0,016
4,75	79489	0	0,04	0	0	0,96	0	0	0	0	0	0,14	0,86	0	0,024
4,75	32758	0,04	0,04	0	0	0,79	0,06	0	0,07	0	0,05	0,15	0,8	0	0,048
4,75	35385	0,02	0,01	0	0,04	0,93	0	0	0	0	0,03	0,94	0,01	0,02	0,042
4,75	18663	0,01	0,04	0	0	0,95	0	0	0	0	0,9	0	0,04	0,06	0,036
4,75	28725	0	0,04	0	0,01	0,94	0	0	0,01	0	0,02	0,76	0,04	0,18	0,022
4,75	12830	0	0,04	0	0	0,96	0	0	0	0	0	0,04	0,96	0	0,024
4,75	33116	0,02	0,04	0	0,01	0,81	0,12	0	0	0	0,12	0,05	0,83	0	0,04
4,75	91097	0,06	0,03	0	0	0	0	0	0,91	0	0	0,48	0	0,52	0,046
4,75	46640	0	0,04	0	0,07	0,89	0	0	0	0	0,89	0,04	0,07	0	0,048
4,75	88878	0,01	0,04	0	0	0,94	0	0	0,01	0	0,94	0,04	0,02	0	0,038
4,75	6783	0	0,04	0	0,01	0,93	0,01	0	0,01	0	0,02	0,04	0,93	0,01	0,034
5	49924	0,01	0,04	0	0,03	0,8	0	0	0,12	0	0,01	0,07	0,82	0,1	0,044
5	29114	0,05	0	0,01	0	0,91	0,01	0,02	0	0	0,94	0	0,04	0,02	0,048
5	18857	0	0,04	0	0	0,96	0	0	0	0	0	0	0	1	0,012
5	63719	0	0,04	0	0,01	0,87	0	0	0,08	0	0,08	0	0,23	0,69	0,02
5	16099	0	0,04	0	0	0,94	0	0,01	0	0,01	0	0,02	0,02	0,96	0,01
5	12695	0,03	0,03	0	0	0	0,18	0,02	0,74	0	0	0,78	0,02	0,2	0,042
5	55771	0	0	0	0,09	0,91	0	0	0	0	0,09	0	0	0,91	0,046
5	25666	0	0,04	0	0	0,02	0	0	0,93	0,01	0,01	0,04	0,1	0,85	0,002
5	3499	0	0,04	0,03	0	0,7	0,21	0,02	0	0	0,21	0,13	0	0,66	0,036
5	23478	0	0,04	0	0	0,95	0	0	0,01	0	0	0,04	0,96	0	0,028
5	24690	0,06	0	0	0	0,87	0,07	0	0	0	0,81	0,13	0,01	0,05	0,044
5	39044	0,05	0,03	0	0	0,71	0	0	0,2	0,01	0,26	0,18	0,03	0,53	0,046
5	29823	0	0,04	0	0	0,96	0	0	0	0	0	0,04	0	0,96	0,008
5	23844	0,06	0	0	0	0,94	0	0	0	0	0,03	0,03	0,94	0	0,048
5	100000	0	0,97	0,01	0,01	0	0	0	0	0,01	0,02	0,98	0	0	0,898
5	92520	0,03	0,03	0	0	0,69	0,23	0,02	0	0	0	0,03	0,72	0,25	0,042
5	31639	0	0,04	0	0	0,92	0	0	0,04	0	0,04	0	0,04	0,92	0,016
5	25354	0	0,04	0	0,02	0,92	0,02	0	0	0	0,02	0,04	0,03	0,91	0,03
5	100000	0	0,83	0,16	0	0	0	0,01	0	0	0,01	0,83	0,01	0,15	0,6
5	24789	0	0,01	0	0,11	0,52	0,2	0,01	0,15	0	0,18	0,68	0,12	0,02	0,044

5	49924	0	0,04	0	0,05	0,91	0	0	0	0	0	0,24	0,75	0,01	0,038
5	48745	0	0	0	0,09	0,43	0	0	0,48	0	0	0	0,52	0,48	0,048
5	68148	0,01	0	0	0,17	0	0,8	0	0,02	0	0,8	0	0,19	0,01	0,046
5	27104	0	0,04	0	0,01	0,95	0	0	0	0	0	0	0,95	0,05	0,038
5	100000	0,11	0,88	0	0	0	0	0	0,01	0	0	0,88	0	0,12	0,718
5	36127	0	0,04	0	0	0,91	0	0	0,05	0	0	0,04	0,08	0,88	0,012
5	81562	0	0	0	0,11	0,87	0	0,01	0	0,01	0	0,99	0,01	0	0,048
5	29321	0,07	0	0	0	0,93	0	0	0	0	0	0,21	0,79	0	0,044
5	37851	0,04	0,03	0	0	0,9	0	0	0,03	0	0,03	0,95	0	0,02	0,048
5	71725	0	0,04	0,01	0	0,16	0,79	0	0	0	0,83	0,01	0,16	0	0,046
5	30800	0	0,04	0	0	0,96	0	0	0	0	0	0,04	0	0,96	0,018
5	11891	0	0,04	0	0	0,96	0	0	0	0	0	0,01	0,96	0,03	0,02
5	19093	0	0,04	0	0	0,93	0	0	0,01	0,02	0	0,06	0,94	0	0,018
5	20689	0	0,01	0	0,3	0,69	0	0	0	0	0,99	0,01	0	0	0,048
5	100000	0	0,99	0	0	0,01	0	0	0	0	0	1	0	0	0,946
5	100000	0	0,97	0,01	0,02	0	0	0	0	0	0,01	0	0	0,99	0,2
5	31765	0	0,04	0	0	0,96	0	0	0	0	0	0,04	0	0,96	0,01
5	100000	0,04	0,96	0	0	0	0	0	0	0	0,04	0,96	0	0	0,958
5	6927	0	0	0	0,1	0,89	0	0	0	0,01	0,01	0,02	0,11	0,86	0,048
5	18617	0	0	0	0,01	0	0,94	0,05	0	0	0,01	0,94	0	0,05	0,048
5	61239	0,01	0,04	0	0,02	0,93	0	0	0	0	0,07	0	0	0,93	0,042
5	100000	0	0,98	0	0	0	0	0,01	0,01	0	0	1	0	0	0,9
5	90876	0	0,04	0	0	0,93	0,01	0,02	0	0	0	0,04	0,94	0,02	0,03
5	89702	0	0,04	0	0	0,96	0	0	0	0	0,04	0,11	0,85	0	0,02
5	17826	0	0	0	0,1	0,87	0,02	0,01	0	0	0,05	0,83	0,02	0,1	0,048
5	18690	0,06	0	0,02	0	0,92	0	0	0	0	0,06	0,15	0	0,79	0,048
5	4828	0	0	0	0,09	0,4	0	0	0,47	0,04	0,01	0	0,55	0,44	0,048
5	31857	0,05	0	0	0	0,95	0	0	0	0	0	0,05	0,95	0	0,044
5	23110	0	0,04	0	0	0,96	0	0	0	0	0	0	0,04	0,96	0,01
5	100000	0	0,96	0	0	0	0	0	0,04	0	0,31	0,65	0,04	0	0,858
5	99390	0	0,04	0	0,04	0,92	0	0	0	0	0,04	0,92	0	0,04	0,046
5	21599	0,01	0,04	0,01	0	0,94	0	0	0	0	0	0,25	0,71	0,04	0,038
5	10049	0	0,04	0	0,01	0,95	0	0	0	0	0,06	0	0	0,94	0,016

5	13620	0,06	0	0	0	0,92	0,02	0	0	0	0	0,06	0,92	0,02	0,048
5	19614	0,01	0	0,01	0,12	0,86	0	0	0	0	0,13	0,01	0,86	0	0,044
5	96771	0	0,04	0	0	0,95	0,01	0	0	0	0	0	0,96	0,04	0,028
5	28708	0	0,04	0	0	0,96	0	0	0	0	0,04	0,09	0	0,87	0,024
5	57321	0	0	0	0	0,26	0	0,09	0	0,65	0	0	0,26	0,74	0,046
5	15517	0,03	0,03	0	0,02	0,92	0	0	0	0	0,02	0,92	0,06	0	0,044
5	51212	0	0,04	0	0	0,73	0,23	0	0	0	0	0,03	0,97	0	0,024
5	58371	0	0,04	0	0	0,96	0	0	0	0	0	0,01	0,96	0,03	0,024
5	31186	0,01	0,04	0,01	0	0,94	0	0	0	0	0,01	0,01	0,94	0,04	0,04
5	56109	0	0,02	0	0,1	0,88	0	0	0	0	0	0,9	0,1	0	0,038
5	15969	0	0,04	0,01	0	0,95	0	0	0	0	0,06	0	0,87	0,07	0,038
5	100000	0,11	0,66	0,16	0	0,01	0	0	0,06	0	0,72	0,27	0,01	0	0,878
5	43743	0,05	0	0	0,01	0,54	0,4	0	0	0	0,28	0	0,54	0,18	0,038
5	8067	0	0,03	0	0,11	0,67	0,19	0	0	0	0,19	0,67	0,14	0	0,046
5	10811	0,01	0,04	0	0	0,93	0	0	0,01	0,01	0,01	0,05	0,94	0	0,026
5	100000	0,13	0,87	0	0	0	0	0	0	0	0,13	0,87	0	0	0,976
5	36039	0	0,04	0	0,03	0,92	0,01	0	0	0	0,01	0,03	0,04	0,92	0,034
5	55599	0	0,04	0	0	0,95	0	0	0	0,01	0,95	0,03	0,01	0,01	0,048
5	20744	0,05	0	0	0	0,93	0,02	0	0	0	0,02	0,95	0,03	0	0,046
5	14549	0	0,04	0	0	0,95	0	0	0	0,01	0,01	0,25	0,74	0	0,024
5	100000	0,03	0,96	0	0	0	0	0,01	0	0	0	0,98	0,02	0	0,876
5	52231	0	0,04	0	0	0,96	0	0	0	0	0,85	0	0	0,15	0,044
5	73374	0,05	0,02	0,01	0	0,91	0	0,01	0	0	0,01	0,04	0,04	0,91	0,048
5	87719	0	0,04	0,04	0,02	0,89	0	0	0	0,01	0	0,02	0,93	0,05	0,046
5	8851	0	0,04	0	0	0,95	0,01	0	0	0	0	0,96	0,01	0,03	0,026
5	100000	0	0,98	0	0	0	0	0,02	0	0	0	0,98	0,02	0	0,86
5	41658	0	0,01	0	0,15	0,62	0	0	0,22	0	0,22	0,62	0,12	0,04	0,042
5	42721	0,02	0,04	0	0	0,94	0	0	0	0	0,02	0,96	0,02	0	0,048
5	20242	0	0,04	0	0	0,94	0	0,01	0	0,01	0	0,04	0,94	0,02	0,026
5	100000	0	0,99	0	0	0,01	0	0	0	0	0	0,99	0	0,01	0,914
5	9606	0	0,04	0	0	0,96	0	0	0	0	0	0,93	0,07	0	0,036
5	18276	0	0,04	0,01	0,01	0,94	0	0	0	0	0	0,04	0,95	0,01	0,04
5	52805	0	0	0	0,12	0,81	0	0	0	0,07	0,02	0,81	0,15	0,02	0,048

5	24090	0,01	0,04	0	0	0,95	0	0	0	0	0,98	0	0	0,02	0,044
5	100000	0	1	0	0	0	0	0	0	0	0	1	0	0	0,988
5	39232	0	0	0	0,24	0	0	0	0,76	0	0,76	0	0,01	0,23	0,048
5	20074	0	0,04	0	0	0,95	0	0	0,01	0	0,01	0,04	0	0,95	0,006
5	100000	0,13	0,85	0	0	0,01	0,01	0	0	0	0,01	0,59	0,27	0,13	0,592
5	90512	0,04	0,03	0,01	0	0,92	0	0	0	0	0,05	0,03	0,92	0	0,048
5	38271	0	0,04	0,01	0	0,93	0,01	0,01	0	0	0,01	0,5	0,49	0	0,038
5	50534	0,06	0	0	0	0	0	0	0,9	0,04	0	0,06	0,04	0,9	0,044
5	66427	0	0,04	0	0	0,95	0	0,01	0	0	0,01	0,04	0,04	0,91	0,03
5	16731	0	0,04	0,02	0	0,91	0	0,01	0,02	0	0,03	0,04	0,91	0,02	0,042
5	67544	0	0,04	0	0	0,96	0	0	0	0	0,96	0	0	0,04	0,026
5	50639	0,01	0,04	0	0	0,89	0	0	0	0,06	0,01	0,05	0,94	0	0,034
5	14517	0	0	0	0,04	0,59	0,23	0,04	0,1	0	0,01	0	0,06	0,93	0,048
5	9155	0	0,04	0	0	0,94	0	0	0,01	0,01	0	0,05	0,01	0,94	0,014
	30114,62	0,0093	0,07679	0,00325	0,03638	0,79579	0,03475	0,00249	0,03558	0,00571	0,21757	0,24284	0,29635	0,24324	0,078691
