# Supplementary material for: Deception Undermines the Stability of Cooperation in Games of Indirect Reciprocity
Source: PLoS One. 2016 Jan 29;11(1):e0147623. doi: 10.1371/journal.pone.0147623 (PMC4732813; doi:10.1371/journal.pone.0147623)
Supplement: S2 Table — (PDF) [file pone.0147623.s006.pdf]

S2 Table. Results of the MST=90% scenario.

shunning

b	time_to_c	(C,C)	(C,D)	(C,P)	(D,C)	(D,D)	(D,P)	(P,C)	(P,D)	(P,P)	S(G,G)	S(G,B)	S(B,G)	S(B,B)	cooperatio
1	30011	0	0	0	0,11	0,87	0,01	0,01	0	0	0,14	0	0,85	0,01	0,048
1	38307	0	0	0,04	0,1	0,84	0,02	0	0	0	0,82	0,07	0	0,11	0,04
1	15177	0	0,3	0,01	0	0,68	0	0	0,01	0	0	0,02	0,3	0,68	0,044
1	32023	0	0,08	0	0	0,9	0	0	0,02	0	0,9	0,02	0,08	0	0,04
1	11034	0	0	0,04	0	0,11	0	0	0,44	0,41	0	0,04	0,45	0,51	0,012
1	40105	0	0,38	0,01	0	0,57	0,02	0,01	0	0,01	0	0,91	0,07	0,02	0,048
1	14506	0	0	0	0,05	0,94	0,01	0	0	0	0,01	0	0	0,99	0,044
1	3780	0	0,01	0,04	0,05	0,89	0	0	0,01	0	0,01	0	0,98	0,01	0,036
1	9637	0,01	0	0,01	0,19	0,77	0,02	0	0	0	0,92	0	0,04	0,04	0,046
1	15847	0,07	0	0	0,01	0,01	0,91	0	0	0	0,91	0,01	0,07	0,01	0,048
1	20374	0,04	0	0,03	0	0	0	0	0,93	0	0,93	0,03	0	0,04	0,04
1	43833	0	0	0	0,05	0	0	0	0,95	0	0	0	0,08	0,92	0,044
1	17813	0	0	0,01	0,27	0,72	0	0	0	0	0,99	0	0	0,01	0,042
1	22693	0,05	0	0	0	0,95	0	0	0	0	0,05	0,01	0	0,94	0,046
1	6271	0	0,17	0	0	0,83	0	0	0	0	0	0,05	0,77	0,18	0,048
1	8038	0	0,16	0	0	0,84	0	0	0	0	0	0,49	0,51	0	0,046
1	46756	0	0	0,04	0	0,96	0	0	0	0	0,55	0,04	0	0,41	0,028
1	6999	0	0,04	0,04	0	0,21	0,65	0,02	0,04	0	0,03	0,1	0,86	0,01	0,044
1	28565	0	0	0,04	0	0	0	0	0,76	0,2	0,01	0,03	0	0,96	0,002
1	33667	0	0,34	0	0	0,34	0	0	0,32	0	0,01	0,01	0,31	0,67	0,044
1	63443	0	0,03	0,01	0	0,58	0,3	0,07	0	0,01	0,89	0,01	0,02	0,08	0,046
1	38085	0	0,14	0	0,05	0,78	0,03	0	0	0	0,13	0,79	0,03	0,05	0,044
1	23887	0,01	0	0,04	0	0,51	0	0	0,44	0	0,45	0,04	0	0,51	0,022
1	20777	0,02	0	0	0,04	0,93	0,01	0	0	0	0,05	0,93	0,02	0	0,048
1	15761	0,04	0	0,02	0	0,15	0	0,01	0,78	0	0	0,02	0,83	0,15	0,048
1	14489	0	0,96	0,04	0	0	0	0	0	0	0	0,04	0,03	0,93	0,046
1	17946	0	0	0,04	0,01	0,94	0,01	0	0	0	0,01	0,04	0	0,95	0,01
1	12446	0	0,01	0,04	0	0,92	0	0	0	0,03	0,01	0,04	0,94	0,01	0,03
1	74698	0	0,02	0,01	0,1	0,87	0	0	0	0	0,02	0,1	0,87	0,01	0,046
1	8792	0	0	0,04	0,01	0,84	0,1	0,01	0	0	0	0,04	0,44	0,52	0,034

1	12353	0	0	0,01	0,18	0,81	0	0	0	0	0,81	0,01	0,17	0,01	0,04
1	5672	0,03	0	0,03	0,03	0	0,01	0	0,9	0	0,03	0	0,94	0,03	0,048
1	14344	0,04	0	0,02	0,01	0,93	0	0	0	0	0,05	0,07	0,88	0	0,044
1	53759	0	0	0	0,23	0,72	0,05	0	0	0	0,72	0,02	0,26	0	0,048
1	27033	0,06	0	0	0	0,91	0,03	0	0	0	0,5	0,07	0,43	0	0,042
1	23419	0	0,04	0,04	0,02	0	0,67	0	0	0,23	0	0,1	0	0,9	0,026
1	23637	0	0,62	0,02	0	0	0	0,01	0	0,35	0	0,03	0,01	0,96	0,042
1	7361	0,02	0	0,04	0	0,1	0	0	0,84	0	0,86	0,04	0,1	0	0,048
1	40513	0,06	0	0,03	0	0	0,91	0	0	0	0,05	0,04	0	0,91	0,048
1	26528	0	0,01	0,04	0	0,22	0	0	0,73	0	0	0,04	0,73	0,23	0,016
1	19520	0	0,17	0	0,01	0,81	0,01	0	0	0	0	0,55	0,44	0,01	0,042
1	50691	0,02	0	0,04	0,03	0,59	0	0	0,31	0,01	0,01	0,84	0,15	0	0,046
1	32305	0,01	0,54	0	0	0,32	0	0	0,13	0	0	0,01	0,13	0,86	0,048
1	10932	0	0	0	0,15	0,03	0,82	0	0	0	0,15	0,82	0	0,03	0,048
1	29312	0	0,05	0	0,06	0,85	0,02	0	0,02	0	0,8	0,04	0,11	0,05	0,048
1	68677	0	0	0,04	0	0	0	0	0	0,96	0	0,04	0	0,96	0,008
1	68823	0,02	0,22	0	0	0	0	0	0,76	0	0	0,02	0,22	0,76	0,042
1	29427	0	0	0,04	0	0,06	0,9	0	0	0	0	0	0	1	0,002
1	6945	0,02	0	0,03	0,04	0,91	0	0	0	0	0,91	0,07	0,02	0	0,048
1	12974	0,02	0	0,04	0	0,84	0,1	0	0	0	0,74	0,25	0	0,01	0,048
1	53478	0	0	0,01	0,2	0,79	0	0	0	0	0,79	0,01	0,2	0	0,046
1	56882	0	0,24	0	0	0,76	0	0	0	0	0	0,76	0,24	0	0,046
1	31679	0	0	0,04	0	0	0	0,01	0,94	0,01	0,01	0,05	0	0,94	0,012
1	37925	0,04	0,01	0	0	0,92	0	0,03	0	0	0,92	0,01	0,07	0	0,046
1	17220	0	0,24	0	0	0,75	0,01	0	0	0	0,03	0,72	0,24	0,01	0,044
1	22779	0,02	0	0,02	0	0,96	0	0	0	0	0,98	0,01	0,01	0	0,038
1	26138	0	0	0	0,09	0,43	0	0	0,48	0	0	0	0,57	0,43	0,044
1	13310	0	0	0,04	0	0,13	0	0,04	0,54	0,25	0,15	0,19	0	0,66	0,042
1	72680	0	0	0	0,09	0,27	0	0	0,64	0	0,34	0	0	0,66	0,048
1	19464	0	0	0,03	0,13	0	0	0	0,83	0,01	0,19	0,06	0,67	0,08	0,048
1	40799	0	0,01	0,01	0	0,06	0	0,07	0,85	0	0	0,01	0,14	0,85	0,044
1	10134	0	0,01	0,01	0,09	0,52	0	0	0,37	0	0	0,02	0,37	0,61	0,048
1	8452	0	0	0,04	0,02	0,68	0	0	0,25	0,01	0,01	0,01	0,28	0,7	0,038

1	20165	0	0	0,04	0	0,42	0,5	0	0,02	0,02	0,06	0	0,52	0,42	0,01
1	5417	0	0	0	0,11	0,49	0	0	0,01	0,39	0,02	0,49	0,39	0,1	0,048
1	83776	0	0	0	0,07	0,9	0,01	0	0,02	0	0,09	0,91	0	0	0,04
1	16186	0	0,13	0	0	0,1	0,62	0	0,15	0	0,15	0,1	0,62	0,13	0,042
1	69685	0,07	0,01	0	0	0,92	0	0	0	0	0,07	0,91	0,01	0,01	0,048
1	4699	0	0	0,02	0,09	0,33	0	0	0,54	0,02	0,54	0,04	0,02	0,4	0,046
1	8681	0,04	0	0,02	0,06	0,17	0,7	0	0	0,01	0,04	0,05	0,72	0,19	0,048
1	40215	0	0	0,04	0,01	0	0,62	0,07	0,18	0,08	0,54	0,26	0,01	0,19	0,048
1	16577	0	0,02	0	0,08	0,89	0	0	0,01	0	0,08	0	0,02	0,9	0,046
1	22951	0	0	0,04	0	0	0	0,01	0	0,95	0	0,05	0,09	0,86	0,022
1	15001	0	0,04	0,04	0	0,21	0,27	0,06	0	0,38	0,27	0,02	0,17	0,54	0,042
1	11904	0	0,08	0	0,06	0,84	0	0	0,02	0	0,07	0,08	0	0,85	0,048
1	100000	0	0	0	0,18	0,76	0,05	0	0	0,01	0,19	0,76	0	0,05	0,116
1	7146	0	0	0	0	0,9	0	0,1	0	0	0,1	0	0,9	0	0,048
1	24206	0	0,01	0,04	0	0,12	0	0	0,01	0,82	0	0,04	0	0,96	0,018
1	21492	0	0	0,01	0,11	0,18	0,13	0,03	0,39	0,15	0,57	0,17	0,01	0,25	0,042
1	13018	0,02	0,05	0,03	0,03	0,83	0,02	0	0	0,02	0	0,03	0,88	0,09	0,046
1	14289	0,06	0,86	0	0,01	0	0	0	0,07	0	0	0,06	0	0,94	0,048
1	28661	0	0,05	0	0,1	0,85	0	0	0	0	0,95	0	0,05	0	0,046
1	68519	0	0,01	0	0,12	0,87	0	0	0	0	0,03	0,01	0,96	0	0,038
1	23297	0	0,08	0	0	0,91	0	0	0,01	0	0,87	0	0,09	0,04	0,048
1	34004	0	0	0	0,13	0,87	0	0	0	0	0	0,13	0,87	0	0,048
1	6499	0,01	0	0	0,05	0,94	0	0	0	0	0,01	0,94	0,05	0	0,048
1	27006	0	0,03	0	0,05	0	0,92	0	0	0	0	0,08	0,01	0,91	0,038
1	11857	0	0,03	0,01	0,15	0,8	0	0	0,01	0	0,98	0,01	0,01	0	0,046
1	11805	0	0,01	0,04	0,03	0,01	0,89	0,02	0	0	0	0,89	0,03	0,08	0,046
1	46030	0,03	0	0,01	0,03	0	0	0	0,93	0	0,17	0,03	0	0,8	0,038
1	28333	0,01	0,11	0	0	0,66	0,22	0	0	0	0,12	0	0,66	0,22	0,046
1	9903	0,02	0,03	0	0,01	0,48	0,46	0	0	0	0,48	0,45	0,04	0,03	0,042
1	21178	0	0,06	0,03	0	0,35	0,56	0	0	0	0	0,02	0,95	0,03	0,042
1	11196	0,04	0	0	0	0,85	0	0	0,08	0,03	0,08	0,05	0	0,87	0,032
1	4305	0,06	0	0,02	0,01	0,91	0	0	0	0	0,07	0,14	0,04	0,75	0,048
1	10522	0	0,21	0,01	0	0,78	0	0	0	0	0	0,79	0,21	0	0,048

1	19985	0,06	0,02	0	0	0,92	0	0	0	0	0,09	0,91	0	0	0,046
1	4024	0,02	0	0,04	0	0,9	0	0	0	0,04	0,9	0	0,1	0	0,042
1	8030	0,04	0	0,01	0,02	0	0,92	0	0	0,01	0,92	0,01	0,04	0,03	0,048
1	11002	0,03	0	0,03	0	0,42	0	0,04	0,46	0,02	0,46	0,08	0,04	0,42	0,048
1,25	29953	0	0,3	0	0	0,65	0,03	0	0,02	0	0,03	0	0,31	0,66	0,046
1,25	55635	0	0,15	0	0	0,72	0	0	0,08	0,05	0,15	0	0,72	0,13	0,046
1,25	19006	0,03	0	0	0,04	0,76	0	0	0,17	0	0,17	0,03	0,8	0	0,042
1,25	6322	0,02	0,04	0	0,01	0	0,05	0,02	0,86	0	0,02	0,86	0,05	0,07	0,048
1,25	94248	0	0	0	0,12	0,66	0,2	0	0	0,02	0	0,2	0,8	0	0,046
1,25	35744	0,03	0,07	0	0	0,63	0	0,01	0,02	0,24	0,01	0,07	0,58	0,34	0,048
1,25	11993	0	0	0,04	0	0,94	0	0,02	0	0	0,95	0,04	0,01	0	0,032
1,25	71399	0,04	0	0,01	0,06	0,89	0	0	0	0	0,97	0	0,03	0	0,048
1,25	3156	0	0	0,04	0	0,85	0	0	0,11	0	0	0,88	0,11	0,01	0,012
1,25	20223	0	0	0,04	0	0,96	0	0	0	0	0	0,04	0,96	0	0,028
1,25	3730	0,05	0	0,01	0	0,94	0	0	0	0	0,05	0,01	0	0,94	0,042
1,25	36813	0,02	0	0,04	0	0,07	0	0,02	0,79	0,06	0,47	0,35	0,09	0,09	0,048
1,25	22989	0	0	0	0,12	0	0	0	0,88	0	0	0,11	0,89	0	0,048
1,25	90785	0	0,12	0	0	0	0,74	0	0,14	0	0,09	0,63	0,28	0	0,04
1,25	9387	0	0,83	0,04	0	0,02	0	0	0,11	0	0	0,62	0	0,38	0,036
1,25	27536	0,01	0,14	0,01	0,02	0,82	0	0	0	0	0,07	0,03	0,09	0,81	0,044
1,25	40478	0	0	0	0,06	0,92	0,01	0	0	0,01	0,03	0	0	0,97	0,048
1,25	100000	0,04	0	0,43	0,15	0,21	0,01	0,16	0	0	0,25	0,58	0	0,17	0,394
1,25	62040	0	0,04	0,04	0	0,91	0	0	0,01	0	0,01	0,89	0,04	0,06	0,01
1,25	16863	0	0,98	0	0	0	0,02	0	0	0	0	0,98	0	0,02	0,042
1,25	4916	0	0,14	0,01	0	0,84	0	0	0,01	0	0	0,01	0,9	0,09	0,046
1,25	18860	0	0,02	0,04	0,05	0,79	0	0	0,1	0	0,77	0,06	0,1	0,07	0,04
1,25	3102	0	0	0,04	0	0,51	0	0	0,45	0	0	0,55	0,45	0	0,018
1,25	24027	0,03	0,03	0,04	0	0,02	0,79	0,02	0	0,07	0,03	0,01	0,15	0,81	0,048
1,25	11390	0	0	0,04	0	0,01	0,13	0,01	0,81	0	0,02	0,03	0,01	0,94	0,016
1,25	18374	0,01	0,08	0,03	0	0	0,88	0	0	0	0,01	0,03	0,96	0	0,048
1,25	17146	0,02	0,07	0	0	0,65	0,23	0,03	0	0	0,3	0,01	0,69	0	0,048
1,25	24204	0	0	0	0	0	0	0,13	0	0,87	0	0	0,87	0,13	0,048
1,25	25598	0,02	0,09	0,01	0	0,58	0	0	0,3	0	0,1	0,01	0,3	0,59	0,044

1,25	28672	0,01	0,99	0	0	0	0	0	0	0	0	0,99	0,01	0	0,028
1,25	5047	0	0,57	0,02	0	0,02	0	0	0	0,39	0	0,04	0,01	0,95	0,046
1,25	2792	0,05	0,01	0	0	0,62	0,05	0,01	0,25	0,01	0,01	0,56	0,3	0,13	0,044
1,25	57099	0	0	0,04	0,01	0,94	0	0,01	0	0	0,05	0,01	0	0,94	0,024
1,25	57773	0	0	0,01	0,07	0,89	0,01	0	0,02	0	0,08	0,02	0,03	0,87	0,042
1,25	43265	0	0	0,04	0	0	0,09	0	0,09	0,78	0,01	0,12	0	0,87	0,004
1,25	11405	0	0	0,04	0	0,95	0	0	0	0,01	0,01	0,03	0,96	0	0,028
1,25	34050	0	0,12	0	0	0,85	0	0	0,03	0	0,03	0	0,97	0	0,048
1,25	27710	0	0	0	0,07	0,92	0	0	0,01	0	0	0,93	0,07	0	0,048
1,25	10329	0,01	0,86	0	0	0	0,12	0,01	0	0	0	0	0,01	0,99	0,048
1,25	29217	0	0,3	0	0	0,7	0	0	0	0	0	0	0,34	0,66	0,042
1,25	22009	0	0	0	0,11	0,89	0	0	0	0	0	0	0,99	0,01	0,048
1,25	18910	0,01	0,2	0	0,01	0,02	0	0	0,76	0	0,01	0,01	0,22	0,76	0,04
1,25	12023	0,02	0	0,04	0	0,87	0	0,05	0,02	0	0,87	0,1	0,03	0	0,042
1,25	23653	0,01	0	0	0	0,1	0	0,07	0	0,82	0	0	0,18	0,82	0,046
1,25	31005	0	0,03	0	0,09	0,87	0	0,01	0	0	0,1	0,09	0,8	0,01	0,048
1,25	59440	0	0,02	0,04	0	0,93	0	0	0	0,01	0,03	0,04	0	0,93	0,01
1,25	18844	0	0,3	0	0	0,44	0,26	0	0	0	0,3	0	0,02	0,68	0,046
1,25	44131	0	0,03	0,04	0	0,54	0,39	0	0	0	0,01	0,03	0,57	0,39	0,026
1,25	9951	0	0,01	0,04	0	0	0	0	0,94	0,01	0	0,05	0,95	0	0,028
1,25	31394	0	0,96	0,03	0,01	0	0	0	0	0	0	0,77	0,01	0,22	0,046
1,25	45544	0	0,09	0,01	0	0,84	0,06	0	0	0	0,5	0	0,45	0,05	0,036
1,25	13639	0	0	0	0,06	0,01	0	0	0,88	0,05	0,06	0	0	0,94	0,044
1,25	23526	0,04	0	0,01	0,01	0,94	0	0	0	0	0,04	0,96	0	0	0,048
1,25	11908	0	0,01	0,04	0	0,75	0,03	0	0,17	0	0,01	0,81	0,01	0,17	0,002
1,25	8175	0	0	0,04	0	0,96	0	0	0	0	0,96	0,04	0	0	0,036
1,25	9245	0	0	0,04	0,01	0	0,95	0	0	0	0,86	0	0,1	0,04	0,038
1,25	33972	0,01	0,05	0	0	0	0,94	0	0	0	0,95	0,01	0,04	0	0,044
1,25	34302	0,03	0	0	0	0,95	0	0,02	0	0	0,05	0,95	0	0	0,038
1,25	45141	0,03	0	0,04	0,03	0	0,19	0	0,71	0	0,23	0,12	0,65	0	0,048
1,25	60943	0	0	0	0,24	0,76	0	0	0	0	0,76	0,24	0	0	0,044
1,25	32604	0	0	0	0,08	0,31	0,06	0,04	0,5	0,01	0,09	0,25	0,48	0,18	0,046
1,25	100000	0,01	0	0,96	0	0	0,01	0,02	0	0	0,02	0,96	0,01	0,01	0,612

1,25	100000	0,56	0	0,44	0	0	0	0	0	0	0,79	0,21	0	0	0,998
1,25	12211	0	0	0,04	0	0,57	0	0	0,39	0	0	0,61	0,39	0	0,022
1,25	8513	0,03	0,18	0	0	0,17	0,1	0	0,52	0	0,09	0,2	0,05	0,66	0,046
1,25	28027	0	0,22	0	0	0,75	0,01	0,01	0,01	0	0,01	0,55	0,22	0,22	0,038
1,25	53862	0,03	0	0,04	0	0,8	0	0,02	0,01	0,1	0	0,07	0,02	0,91	0,04
1,25	38474	0,03	0,01	0,04	0,02	0,06	0,84	0	0	0	0,03	0,42	0,22	0,33	0,048
1,25	92750	0	0	0,04	0	0,08	0,84	0,04	0	0	0,04	0,04	0,88	0,04	0,04
1,25	12465	0	0	0,04	0	0,06	0,82	0	0,01	0,07	0	0,05	0,88	0,07	0,02
1,25	9294	0	0	0,04	0	0,1	0	0	0,85	0,01	0	0,05	0	0,95	0,002
1,25	44078	0	0,09	0,01	0,01	0,85	0,01	0	0,03	0	0,01	0,02	0,88	0,09	0,04
1,25	17646	0	0	0,04	0	0,93	0	0,03	0	0	0,49	0,04	0,03	0,44	0,022
1,25	24965	0	0	0,01	0,26	0,01	0,71	0,01	0	0	0,93	0,06	0,01	0	0,044
1,25	22384	0,01	0	0,04	0	0,02	0	0	0,93	0	0,04	0	0,03	0,93	0,016
1,25	9063	0	0,23	0	0	0,51	0,11	0	0,15	0	0,16	0,59	0	0,25	0,046
1,25	29649	0,01	0,05	0,04	0	0	0,02	0	0,88	0	0,02	0,89	0,06	0,03	0,03
1,25	43417	0,02	0	0,04	0	0,94	0	0	0	0	0,02	0,04	0,94	0	0,046
1,25	34198	0	0,03	0,04	0	0,9	0,01	0	0,02	0	0,05	0,02	0,91	0,02	0,048
1,25	7208	0	0	0	0,13	0,86	0	0	0,01	0	0,68	0,01	0,04	0,27	0,046
1,25	7991	0,01	0,12	0	0,01	0,02	0,84	0	0	0	0	0,93	0,03	0,04	0,042
1,25	22013	0	0,22	0	0	0,77	0,01	0	0	0	0,33	0,31	0	0,36	0,042
1,25	47787	0	0	0,04	0	0	0,94	0,02	0	0	0	0,03	0	0,97	0,028
1,25	9495	0	0	0,04	0	0,86	0	0,02	0,08	0	0,94	0,04	0	0,02	0,036
1,25	10519	0,03	0	0,02	0	0,9	0,04	0,01	0	0	0,75	0,17	0,08	0	0,046
1,25	7293	0	0	0,04	0	0	0,07	0	0	0,89	0	0,11	0	0,89	0,018
1,25	14539	0,01	0	0,04	0	0	0,05	0	0,9	0	0	0,09	0,01	0,9	0,016
1,25	17602	0	0,08	0,04	0,01	0,02	0	0,01	0,84	0	0,02	0,03	0,11	0,84	0,038
1,25	13655	0	0	0,04	0	0,93	0,02	0	0,01	0	0	0,06	0,89	0,05	0,03
1,25	100000	0	0	0,99	0,01	0	0	0	0	0	0,01	0,99	0	0	0,69
1,25	17032	0	0,08	0	0	0,92	0	0	0	0	0,92	0	0,08	0	0,042
1,25	59479	0	0,09	0	0,01	0,64	0,25	0	0,01	0	0,85	0,01	0,13	0,01	0,046
1,25	30353	0,04	0,06	0,03	0,01	0,75	0	0	0	0,11	0,12	0,06	0,07	0,75	0,04
1,25	73882	0	0	0,04	0	0	0,74	0	0,01	0,21	0,06	0,05	0,15	0,74	0,012
1,25	47990	0	0,17	0	0,01	0,82	0	0	0	0	0,17	0,83	0	0	0,042

1,25	30074	0,01	0,58	0,03	0	0	0	0	0,38	0	0,01	0,62	0	0,37	0,046
1,25	38920	0	0,13	0	0	0,8	0,07	0	0	0	0,13	0,07	0,8	0	0,048
1,25	37339	0,01	0	0,04	0	0,02	0,91	0	0	0,02	0,03	0,04	0,02	0,91	0,022
1,25	34980	0	0	0	0,09	0	0,05	0	0,86	0	0,05	0,86	0,09	0	0,048
1,25	32563	0	0,02	0,04	0	0,02	0,01	0	0	0,91	0,05	0,04	0	0,91	0,016
1,5	42834	0,01	0,17	0	0,02	0,79	0,01	0	0	0	0,24	0	0,02	0,74	0,048
1,5	3076	0	0	0,01	0,06	0,87	0,01	0	0,05	0	0	0,07	0,05	0,88	0,038
1,5	39326	0	0,04	0,01	0,08	0,87	0	0	0	0	0,04	0,05	0,88	0,03	0,044
1,5	17591	0,06	0,01	0,03	0	0,9	0	0	0	0	0	0,01	0,81	0,18	0,048
1,5	10973	0,01	0,07	0,04	0,01	0	0,87	0	0	0	0,01	0,05	0,93	0,01	0,042
1,5	58437	0	0,08	0,04	0,01	0,37	0,05	0,01	0,44	0	0,08	0,49	0,38	0,05	0,044
1,5	49311	0	0,12	0	0	0,88	0	0	0	0	0,01	0	0,99	0	0,048
1,5	10451	0,02	0	0,04	0	0,47	0	0,02	0,45	0	0,03	0,49	0,01	0,47	0,034
1,5	37980	0	0,19	0,04	0	0	0,74	0,02	0	0,01	0,03	0,04	0,08	0,85	0,044
1,5	18908	0,02	0	0,04	0	0,01	0	0	0,93	0	0,92	0,05	0,01	0,02	0,042
1,5	58481	0	0	0,04	0	0,62	0,08	0	0,17	0,09	0,08	0,66	0,26	0	0,022
1,5	60466	0	0	0,04	0	0,51	0	0	0,11	0,34	0,51	0,15	0,03	0,31	0,022
1,5	74883	0	0,29	0	0	0,7	0	0	0	0,01	0,19	0	0,09	0,72	0,048
1,5	14488	0	0	0,04	0	0,96	0	0	0	0	0	0,04	0,96	0	0,024
1,5	52921	0	0	0,04	0	0,63	0	0	0,04	0,29	0	0,08	0	0,92	0,006
1,5	8535	0	0	0,04	0,01	0	0,95	0	0	0	0,01	0	0,99	0	0,034
1,5	13073	0	0	0,01	0,07	0,91	0	0,01	0	0	0	0,02	0,91	0,07	0,048
1,5	57792	0	0	0,04	0	0,48	0	0	0,48	0	0	0,03	0,01	0,96	0,002
1,5	19039	0	0	0	0,12	0,88	0	0	0	0	0,12	0	0,88	0	0,048
1,5	51827	0	0	0,02	0,09	0	0,3	0	0,59	0	0,12	0,56	0,3	0,02	0,046
1,5	27361	0	0	0	0,13	0,85	0,01	0	0,01	0	0,14	0	0,85	0,01	0,048
1,5	13400	0	0	0,02	0,13	0,84	0,01	0	0	0	0,03	0,13	0,84	0	0,046
1,5	36231	0	0,09	0	0	0,82	0	0	0,09	0	0,82	0	0,12	0,06	0,048
1,5	48418	0,01	0	0,04	0,04	0,03	0	0,01	0,75	0,12	0,12	0,84	0,03	0,01	0,048
1,5	3009	0	0,01	0,04	0,02	0,62	0	0,01	0	0,3	0	0,66	0,02	0,32	0,028
1,5	46603	0	0	0,04	0	0,96	0	0	0	0	0,95	0,02	0,03	0	0,024
1,5	62250	0,02	0	0,04	0	0,94	0	0	0	0	0	0,04	0,96	0	0,036
1,5	13474	0	0	0,04	0	0,14	0,82	0	0	0	0,01	0,18	0	0,81	0,008

1,5	12430	0	0	0,04	0	0,95	0,01	0	0	0	0,02	0,04	0,01	0,93	0
1,5	29583	0	0	0	0,17	0,8	0	0	0,01	0,02	0,73	0,01	0,14	0,12	0,038
1,5	3949	0,02	0,95	0,01	0	0	0	0,01	0,01	0	0,02	0,01	0,01	0,96	0,046
1,5	42499	0	0,09	0	0	0,86	0,04	0,01	0	0	0,22	0,04	0,73	0,01	0,042
1,5	17324	0	0	0	0,07	0,92	0	0	0,01	0	0,07	0,83	0,01	0,09	0,046
1,5	13624	0	0	0	0,06	0,92	0,02	0	0	0	0,02	0	0,06	0,92	0,044
1,5	12128	0	0	0,04	0	0,46	0,06	0	0,14	0,3	0,48	0,08	0,14	0,3	0,028
1,5	24916	0	0	0,04	0,03	0	0	0,01	0,92	0	0,01	0,07	0,92	0	0,044
1,5	46385	0	0	0	0,01	0,84	0,02	0,12	0	0,01	0,15	0	0,85	0	0,046
1,5	17842	0	0	0	0,16	0,81	0,03	0	0	0	0,03	0,01	0,96	0	0,04
1,5	81392	0,01	0,13	0,01	0,01	0,81	0	0,03	0	0	0,01	0,01	0	0,98	0,046
1,5	47148	0,04	0	0,01	0	0	0,93	0,02	0	0	1	0	0	0	0,044
1,5	67069	0	0,09	0,03	0	0,21	0	0	0,67	0	0,17	0,04	0,79	0	0,044
1,5	9534	0	0	0,01	0,1	0,26	0,02	0	0,6	0,01	0	0,36	0,53	0,11	0,048
1,5	15307	0	0,34	0	0	0,66	0	0	0	0	0	0	0,27	0,73	0,042
1,5	38697	0	0	0,04	0	0,03	0,02	0,04	0,81	0,06	0,05	0,93	0	0,02	0,042
1,5	50719	0	0,14	0	0,02	0,83	0,01	0	0	0	0,16	0,83	0,01	0	0,042
1,5	48123	0	0	0,04	0	0	0,12	0	0,76	0,08	0,01	0,04	0,12	0,83	0,01
1,5	4740	0	0	0,04	0	0,04	0	0	0,92	0	0,02	0,03	0	0,95	0
1,5	23704	0	0,2	0	0	0,79	0	0	0,01	0	0,2	0,79	0	0,01	0,046
1,5	7319	0	0,6	0	0	0,08	0,3	0	0	0,02	0,03	0	0,05	0,92	0,048
1,5	32967	0	0	0,04	0,03	0,93	0	0	0	0	0,05	0,33	0	0,62	0,04
1,5	21563	0,05	0	0,01	0	0,35	0	0	0,59	0	0,94	0,06	0	0	0,044
1,5	26733	0	0,91	0	0	0,03	0,04	0	0,02	0	0,03	0	0,04	0,93	0,042
1,5	24707	0	0,29	0,01	0	0,7	0	0	0	0	0	0,05	0,29	0,66	0,046
1,5	39430	0	0	0,04	0	0,84	0	0	0	0,12	0	0,16	0,84	0	0,022
1,5	5742	0	0,17	0,01	0,02	0,6	0	0	0,2	0	0,22	0,58	0	0,2	0,044
1,5	23927	0	0,01	0,04	0,05	0	0	0	0,89	0,01	0,01	0,9	0,05	0,04	0,048
1,5	32972	0	0,12	0	0	0,87	0	0	0	0,01	0,68	0	0,12	0,2	0,044
1,5	27707	0	0,04	0,04	0,02	0,9	0	0	0	0	0,04	0,08	0,88	0	0,042
1,5	50960	0	0	0,04	0	0,73	0	0	0,03	0,2	0,03	0,04	0,62	0,31	0,014
1,5	4369	0,04	0	0,04	0	0,08	0,84	0	0	0	0,03	0,08	0,86	0,03	0,044
1,5	100000	0	0	1	0	0	0	0	0	0	0	1	0	0	0,616

1,5	6786	0	0	0,04	0,04	0	0	0	0,92	0	0	0,04	0,04	0,92	0,042
1,5	9115	0,05	0	0	0	0,69	0,15	0,01	0,1	0	0,2	0,02	0,68	0,1	0,048
1,5	18331	0	0,14	0	0	0,84	0	0	0	0,02	0	0,14	0,85	0,01	0,04
1,5	30947	0,04	0,04	0	0	0,05	0	0	0,87	0	0	0,02	0,66	0,32	0,046
1,5	17926	0,06	0	0	0	0,93	0	0,01	0	0	0,93	0	0,06	0,01	0,048
1,5	30936	0	0,29	0	0	0,45	0,26	0	0	0	0	0	0,29	0,71	0,046
1,5	24545	0,06	0	0,01	0	0,93	0	0	0	0	0,1	0,01	0,89	0	0,042
1,5	38935	0,01	0	0,04	0	0,21	0,37	0,01	0,09	0,27	0,09	0,09	0,5	0,32	0,03
1,5	19671	0	0	0,02	0,11	0	0,35	0	0	0,52	0	0,48	0,52	0	0,048
1,5	38220	0,01	0	0	0,07	0,91	0,01	0	0	0	0	0,93	0,07	0	0,046
1,5	92495	0	0,1	0,02	0	0,77	0	0,01	0	0,1	0	0,14	0,86	0	0,048
1,5	5618	0	0,28	0	0	0,72	0	0	0	0	0,27	0,01	0	0,72	0,042
1,5	23458	0	0,15	0	0	0,38	0	0	0,47	0	0	0,38	0,48	0,14	0,044
1,5	59508	0	0,06	0,04	0	0	0,01	0	0,89	0	0	0,1	0	0,9	0
1,5	30895	0	0	0,03	0	0,8	0	0,06	0	0,11	0	0,87	0	0,13	0,046
1,5	17273	0	0,12	0	0,03	0,83	0	0,01	0,01	0	0,12	0,83	0,02	0,03	0,042
1,5	12475	0	0	0	0,08	0,86	0	0	0,05	0,01	0,07	0,87	0	0,06	0,048
1,5	17221	0,02	0	0	0,04	0	0	0,02	0,82	0,1	0	0	0,94	0,06	0,048
1,5	68882	0	0,25	0,02	0	0,73	0	0	0	0	0,04	0	0,29	0,67	0,048
1,5	29605	0	0,25	0	0,01	0,73	0,01	0	0	0	0,25	0	0	0,75	0,04
1,5	54020	0,03	0,01	0,04	0,01	0,91	0	0	0	0	0,03	0,04	0,91	0,02	0,042
1,5	19150	0	0,07	0,03	0	0,9	0	0	0	0	0,07	0,04	0,89	0	0,034
1,5	37597	0,02	0	0	0,06	0,03	0	0	0,89	0	0,02	0,03	0,9	0,05	0,038
1,5	25520	0	0,12	0	0	0,86	0	0	0,02	0	0,02	0	0,93	0,05	0,046
1,5	26089	0,05	0	0,01	0	0	0,92	0,01	0	0,01	0,92	0,01	0,07	0	0,048
1,5	47020	0	0,01	0,04	0	0,21	0,71	0	0,03	0	0	0,01	0,95	0,04	0,024
1,5	16186	0	0,14	0	0	0,82	0	0	0,04	0	0,04	0,07	0,75	0,14	0,044
1,5	25552	0,01	0	0,04	0	0	0,94	0	0	0,01	0,15	0,12	0	0,73	0,022
1,5	16739	0	0,01	0,01	0,18	0,79	0,01	0	0	0	0,96	0	0,02	0,02	0,048
1,5	84887	0	0	0,04	0,01	0,46	0,06	0	0,43	0	0,42	0,55	0,01	0,02	0,022
1,5	24512	0,01	0	0,04	0	0	0	0	0,94	0,01	0,01	0	0,99	0	0,028
1,5	10137	0	0,08	0	0	0,89	0,02	0	0,01	0	0,89	0	0,1	0,01	0,044
1,5	4983	0	0	0,04	0	0,96	0	0	0	0	0,43	0,03	0,54	0	0,03

1,5	27361	0	0,07	0,01	0,01	0,2	0,7	0,01	0	0	0,81	0,02	0,17	0	0,046
1,5	51156	0	0,02	0,04	0	0,29	0,57	0	0	0,08	0,7	0,17	0,13	0	0,038
1,5	7013	0	0,06	0	0,07	0,87	0	0	0	0	0	0	1	0	0,044
1,5	11534	0	0	0	0,12	0	0	0	0,88	0	0	0,13	0,87	0	0,046
1,5	54930	0	0	0	0,11	0	0,66	0	0,23	0	0	0,34	0,66	0	0,048
1,5	9462	0	0	0,04	0	0	0	0,04	0	0,92	0,04	0,04	0,01	0,91	0,048
1,75	38688	0	0,39	0,04	0	0,01	0,56	0	0	0	0	0,05	0,05	0,9	0,048
1,75	9145	0,05	0	0	0	0,05	0,57	0	0,33	0	0,1	0,33	0,02	0,55	0,048
1,75	28698	0	0,12	0	0	0,87	0	0	0,01	0	0	0	0,99	0,01	0,042
1,75	32234	0,01	0,01	0,03	0,03	0,85	0	0	0,07	0	0,01	0,04	0,95	0	0,04
1,75	22308	0	0	0	0,08	0,72	0	0	0,2	0	0,01	0,92	0,07	0	0,048
1,75	30781	0	0,18	0	0	0,71	0,09	0,01	0,01	0	0	0,69	0,31	0	0,048
1,75	7962	0	0	0,04	0,04	0	0,92	0	0	0	0,21	0,17	0	0,62	0,03
1,75	9248	0	0,01	0,04	0	0,21	0,03	0	0,71	0	0,22	0	0,42	0,36	0,026
1,75	39188	0	0,61	0,04	0	0,25	0	0	0,1	0	0	0,29	0,1	0,61	0,042
1,75	43294	0	0,12	0	0	0,01	0	0	0,8	0,07	0	0,07	0,92	0,01	0,044
1,75	23345	0	0	0,04	0	0	0,93	0,02	0	0,01	0,93	0,04	0,01	0,02	0,048
1,75	55644	0	0,23	0	0	0,75	0	0	0,01	0,01	0	0,76	0,23	0,01	0,034
1,75	27282	0,05	0	0	0	0	0,01	0	0,94	0	0,01	0,04	0,01	0,94	0,04
1,75	21556	0,01	0,01	0,04	0,05	0,87	0	0	0,02	0	0,01	0,05	0,88	0,06	0,044
1,75	68403	0,01	0,1	0,02	0,02	0,66	0,17	0	0,01	0,01	0,02	0,02	0,31	0,65	0,04
1,75	22411	0	0,03	0,04	0	0	0	0	0,93	0	0,94	0,04	0,02	0	0,048
1,75	19211	0,01	0,08	0,04	0	0,1	0,21	0	0	0,56	0,01	0,09	0,08	0,82	0,034
1,75	20998	0,01	0,06	0	0	0,86	0	0	0,07	0	0,97	0	0,03	0	0,048
1,75	100000	0,02	0	0,96	0	0	0	0,02	0	0	0,02	0,95	0,02	0,01	0,616
1,75	100000	0	0	1	0	0	0	0	0	0	0	0,99	0,01	0	0,614
1,75	20665	0,05	0,03	0,01	0	0	0	0	0,91	0	0,05	0,18	0,01	0,76	0,046
1,75	12940	0	0	0,04	0	0,96	0	0	0	0	0,04	0,96	0	0	0,014
1,75	6333	0,05	0	0,03	0	0	0,03	0	0,89	0	0,01	0,36	0,55	0,08	0,048
1,75	42478	0,01	0,98	0	0	0	0	0	0	0,01	0,01	0,99	0	0	0,042
1,75	27082	0,01	0	0,04	0	0	0,95	0	0	0	0,96	0,02	0,02	0	0,048
1,75	26238	0	0,93	0	0,04	0	0	0	0,02	0,01	0,02	0,04	0,01	0,93	0,044
1,75	71138	0	0	0	0,11	0,21	0,65	0	0,01	0,02	0,11	0,86	0,01	0,02	0,048

1,75	56267	0,04	0	0,04	0	0	0	0	0,92	0	0	0,06	0,02	0,92	0,028
1,75	67216	0	0,15	0	0	0,84	0	0	0	0,01	0	0,01	0,98	0,01	0,048
1,75	12240	0	0	0,04	0	0	0	0	0	0,96	0	0,04	0,96	0	0,02
1,75	43557	0,01	0,03	0	0,06	0,1	0,79	0,01	0	0	0,06	0,02	0,26	0,66	0,048
1,75	25972	0,01	0,17	0	0	0,79	0	0	0,03	0	0,04	0,79	0,17	0	0,048
1,75	13554	0	0,95	0	0	0	0	0,02	0	0,03	0,04	0,04	0	0,92	0,044
1,75	20504	0,06	0	0	0,01	0	0,93	0	0	0	0,06	0,94	0	0	0,048
1,75	11129	0	0,01	0	0,07	0,92	0	0	0	0	0,02	0,98	0	0	0,044
1,75	51271	0	0	0,04	0	0	0,13	0	0,42	0,41	0,11	0,48	0	0,41	0,014
1,75	54389	0	0,73	0,04	0	0	0	0,02	0,05	0,16	0	0,09	0	0,91	0,046
1,75	58045	0	0,04	0,04	0	0	0,42	0	0	0,5	0	0,03	0,43	0,54	0,024
1,75	51811	0	0,07	0	0	0,92	0	0	0,01	0	0,94	0	0,06	0	0,046
1,75	45959	0	0	0,04	0	0,73	0	0,1	0,13	0	0,71	0,04	0	0,25	0,044
1,75	35964	0	0	0,04	0	0	0	0	0,96	0	0,96	0,04	0	0	0,036
1,75	40320	0	0	0,04	0,01	0,61	0	0	0	0,34	0,44	0,22	0	0,34	0,026
1,75	19766	0,01	0	0,04	0	0	0,94	0	0,01	0	0,02	0,54	0	0,44	0,026
1,75	21437	0	0,03	0,04	0	0,91	0	0,01	0	0,01	0,01	0,94	0,04	0,01	0,02
1,75	6292	0	0,06	0	0,06	0,82	0,04	0	0	0,02	0,88	0,04	0,08	0	0,048
1,75	34238	0,06	0	0	0	0	0,92	0	0,02	0	0,98	0	0	0,02	0,046
1,75	12608	0,02	0,02	0,04	0	0,16	0,74	0	0,02	0	0,81	0,02	0,05	0,12	0,036
1,75	14260	0	0,16	0	0	0,82	0,02	0	0	0	0,04	0,01	0,95	0	0,048
1,75	10602	0,04	0	0	0	0,04	0	0,01	0,9	0,01	0	0,06	0,9	0,04	0,048
1,75	68763	0	0,06	0,04	0	0,82	0,07	0,01	0	0	0	0,17	0,83	0	0,048
1,75	47196	0	0,16	0	0	0,84	0	0	0	0	0	0	1	0	0,046
1,75	26236	0	0,97	0	0	0	0	0	0,03	0	0	0,97	0,01	0,02	0,048
1,75	13041	0,02	0	0,04	0	0,86	0	0,03	0	0,05	0	0,04	0,1	0,86	0,044
1,75	25678	0	0	0	0,1	0,89	0	0,01	0	0	0,01	0	0,99	0	0,042
1,75	33463	0	0	0,04	0	0	0,02	0	0,94	0	0	0,04	0	0,96	0,002
1,75	6334	0,06	0	0	0	0,23	0,7	0	0,01	0	0	0	0,98	0,02	0,048
1,75	80713	0	0,01	0,04	0,04	0	0,9	0	0	0,01	0,05	0,01	0,28	0,66	0,046
1,75	4267	0	0	0,04	0,04	0,76	0,16	0	0	0	0,73	0,04	0,03	0,2	0,022
1,75	32623	0	0	0,04	0,02	0	0,05	0	0	0,89	0	0,86	0,07	0,07	0,036
1,75	27863	0	0,15	0	0	0,84	0	0,01	0	0	0,01	0	0,99	0	0,046

1,75	8493	0	0	0	0,27	0,73	0	0	0	0	1	0	0	0	0,048
1,75	45927	0	0	0,04	0	0,47	0,36	0	0,03	0,1	0,09	0,05	0	0,86	0,002
1,75	60518	0	0	0,04	0	0,21	0,01	0	0,74	0	0	0,74	0,01	0,25	0,002
1,75	23678	0	0,13	0	0	0,78	0	0	0,09	0	0	0,13	0,74	0,13	0,04
1,75	53965	0	0	0	0,06	0,94	0	0	0	0	0	0	0,06	0,94	0,04
1,75	3014	0	0	0,03	0	0,88	0	0,09	0	0	0,09	0,02	0,89	0	0,036
1,75	37257	0	0	0,04	0	0	0	0	0,96	0	0	0,04	0,96	0	0,022
1,75	100000	0,07	0	0,79	0	0	0	0,13	0,01	0	0,14	0,79	0	0,07	0,578
1,75	29521	0	0,06	0	0,06	0,88	0	0	0	0	0,88	0	0,12	0	0,048
1,75	36117	0,04	0,01	0	0	0	0,66	0,05	0,24	0	0,25	0	0,68	0,07	0,044
1,75	100000	0	0	0,95	0,02	0	0	0,02	0,01	0	0	0,97	0,01	0,02	0,526
1,75	5300	0	0,09	0	0,03	0,88	0	0	0	0	0,08	0,12	0,8	0	0,044
1,75	33055	0	0	0,04	0	0,23	0	0	0,3	0,43	0,25	0,04	0,05	0,66	0,03
1,75	7702	0,02	0	0	0,12	0	0,78	0	0	0,08	0,81	0,05	0,09	0,05	0,042
1,75	14010	0	0,99	0	0	0	0	0	0	0,01	0,09	0,01	0	0,9	0,044
1,75	19895	0,04	0	0,03	0	0	0,91	0	0	0,02	0,05	0,02	0,93	0	0,042
1,75	10581	0,05	0	0	0	0,92	0,03	0	0	0	0,05	0,95	0	0	0,04
1,75	3881	0	0,04	0,04	0	0,66	0	0	0,15	0,11	0,66	0,04	0,14	0,16	0,042
1,75	19927	0,03	0	0,03	0	0,94	0	0	0	0	0,97	0,03	0	0	0,048
1,75	5765	0,05	0	0	0	0	0,01	0	0,94	0	0,05	0,01	0	0,94	0,042
1,75	6248	0,01	0,22	0	0	0	0	0	0,77	0	0,22	0,01	0	0,77	0,034
1,75	43715	0	0,08	0	0	0,91	0,01	0	0	0	0,92	0,08	0	0	0,048
1,75	20312	0	0	0	0,01	0,89	0	0,1	0	0	0,01	0	0,98	0,01	0,046
1,75	33465	0,01	0,1	0,02	0	0	0,87	0	0	0	0,1	0,03	0,87	0	0,048
1,75	26972	0,01	0,95	0,04	0	0	0	0	0	0	0,02	0,04	0	0,94	0,032
1,75	17801	0,01	0	0,04	0,01	0	0,09	0	0,81	0,04	0,87	0,04	0,04	0,05	0,046
1,75	26886	0	0,05	0	0,06	0,89	0	0	0	0	0	0,89	0,11	0	0,04
1,75	7794	0	0,23	0	0	0,75	0	0,02	0	0	0,23	0,13	0	0,64	0,044
1,75	25300	0,01	0	0,04	0	0,95	0	0	0	0	0,01	0,04	0,95	0	0,03
1,75	38364	0,01	0	0	0,15	0,83	0,01	0	0	0	0,73	0,01	0,25	0,01	0,042
1,75	30531	0,06	0,01	0	0	0	0,92	0	0,01	0	0,07	0,93	0	0	0,044
1,75	74740	0	0,13	0,01	0	0,8	0,02	0	0,01	0,03	0,13	0,01	0,81	0,05	0,048
1,75	5710	0	0,06	0,01	0,01	0,91	0	0	0	0,01	0,92	0,07	0,01	0	0,048

1,75	8869	0	0,97	0	0,03	0	0	0	0	0	0,01	0	0,03	0,96	0,048
1,75	6486	0	0,13	0,04	0	0	0	0,03	0,8	0	0	0,04	0,13	0,83	0,04
1,75	100000	0,21	0	0,79	0	0	0	0	0	0	0,21	0,79	0	0	0,91
1,75	45482	0	0,77	0	0	0	0	0	0,23	0	0	1	0	0	0,046
1,75	35011	0,01	0	0,01	0	0,3	0,1	0,11	0,47	0	0	0,13	0,87	0	0,046
1,75	54686	0	0	0,04	0	0,59	0,3	0,07	0	0	0,1	0,04	0,27	0,59	0,046
1,75	30619	0	0,21	0,04	0	0,7	0,01	0	0,03	0,01	0,19	0,08	0,03	0,7	0,04
2	42729	0,02	0,31	0	0	0	0,67	0	0	0	0,01	0,31	0	0,68	0,046
2	63963	0	0	0	0,07	0,8	0	0	0,12	0,01	0,19	0,01	0	0,8	0,048
2	15067	0	0	0	0,13	0,87	0	0	0	0	0,12	0	0,88	0	0,048
2	7833	0,04	0	0	0,06	0	0,9	0	0	0	0,04	0	0,96	0	0,046
2	19626	0,05	0	0	0	0	0,95	0	0	0	0	0,85	0,05	0,1	0,04
2	100000	0	0,03	0,91	0	0	0	0	0,05	0,01	0	0,91	0	0,09	0,432
2	46257	0	0	0,04	0	0	0	0	0,89	0,07	0,44	0,04	0,52	0	0,04
2	20472	0	0	0,04	0	0	0,92	0	0,04	0	0,79	0	0	0,21	0,02
2	31037	0	0	0,04	0	0	0	0	0,96	0	0	0	1	0	0,024
2	23923	0	0,1	0,04	0,05	0,8	0,01	0	0	0	0,12	0,07	0,01	0,8	0,046
2	66682	0,01	0,06	0	0	0,92	0	0,01	0	0	0,92	0,01	0,07	0	0,048
2	37734	0	0,03	0,04	0	0,35	0,01	0	0	0,57	0,35	0,04	0,04	0,57	0,024
2	57520	0,06	0	0	0	0,7	0	0	0,24	0	0,29	0	0,23	0,48	0,044
2	11934	0,01	0	0,04	0	0	0,15	0	0,8	0	0,78	0,2	0	0,02	0,032
2	4256	0	0,54	0,04	0,03	0	0	0	0,38	0,01	0	0,42	0,04	0,54	0,046
2	26103	0	0	0,04	0	0	0	0	0,05	0,91	0,05	0,06	0	0,89	0,012
2	18666	0,05	0,02	0	0	0,93	0	0	0	0	0,95	0,05	0	0	0,048
2	8175	0,01	0	0,04	0,01	0	0	0,02	0,92	0	0	0,04	0,07	0,89	0,04
2	40928	0	0	0,04	0	0,13	0,83	0	0	0	0,13	0,04	0	0,83	0,01
2	3120	0,01	0,03	0,04	0,03	0	0,89	0	0	0	0,01	0,05	0,04	0,9	0,038
2	6554	0	0	0,04	0	0,52	0,06	0	0,16	0,22	0,58	0,42	0	0	0,034
2	7003	0,05	0,06	0	0	0	0,81	0	0,08	0	0,18	0	0,81	0,01	0,048
2	33739	0,01	0,08	0,01	0	0,9	0	0	0	0	0,01	0	0,98	0,01	0,046
2	15965	0,01	0,01	0,04	0	0,94	0	0	0	0	0,01	0	0,04	0,95	0,016
2	27086	0	0	0,04	0	0,96	0	0	0	0	0	0,03	0,96	0,01	0,022
2	28108	0	0	0,04	0	0,82	0	0	0,14	0	0	0,18	0	0,82	0,002

2	100000	0	0	0,97	0	0	0,02	0,01	0	0	0	0,96	0,04	0	0,55
2	26016	0	0	0,04	0,01	0,77	0	0	0,18	0	0,01	0,18	0,16	0,65	0,02
2	24050	0	0	0,04	0	0,39	0	0	0,57	0	0,27	0,2	0	0,53	0,012
2	9178	0	0,02	0,04	0	0,93	0	0	0	0,01	0,02	0,04	0	0,94	0,004
2	44727	0	0,1	0,01	0,06	0,83	0	0	0	0	0	0,01	0,1	0,89	0,048
2	20078	0	0	0	0,1	0,87	0	0,02	0,01	0	0,19	0,02	0,79	0	0,048
2	22839	0	0,01	0,04	0	0,95	0	0	0	0	0,07	0,04	0,2	0,69	0,01
2	36681	0	0	0,04	0	0,93	0	0,03	0	0	0,01	0,97	0	0,02	0,028
2	71529	0	0	0	0,07	0,93	0	0	0	0	0,05	0,93	0,02	0	0,046
2	18180	0	0	0,03	0,12	0,85	0	0	0	0	0,85	0,03	0,12	0	0,036
2	11575	0,02	0	0,03	0,04	0,9	0	0,01	0	0	0,03	0,02	0,84	0,11	0,048
2	30126	0,01	0	0	0,1	0,83	0,05	0	0,01	0	0,1	0,11	0,79	0	0,048
2	43160	0	0	0,04	0	0	0,86	0	0,06	0,04	0,01	0	0,91	0,08	0,018
2	51104	0,05	0	0,02	0	0,91	0	0,01	0,01	0	0,05	0,93	0,02	0	0,048
2	14337	0	0,11	0,03	0	0,23	0,21	0,03	0	0,39	0,21	0,04	0,34	0,41	0,046
2	18851	0	0,24	0	0	0,76	0	0	0	0	0,14	0,79	0	0,07	0,044
2	12297	0,05	0	0,03	0	0,84	0	0	0,02	0,06	0	0,9	0,07	0,03	0,04
2	27531	0	0	0,04	0	0,96	0	0	0	0	0,96	0	0,04	0	0,032
2	44452	0	0	0	0,06	0,87	0,04	0,01	0,02	0	0	0,87	0,11	0,02	0,048
2	9942	0,06	0,03	0,01	0	0,82	0	0	0,08	0	0,03	0,07	0,07	0,83	0,046
2	57905	0,05	0	0,01	0	0,07	0	0	0,87	0	0,07	0,06	0	0,87	0,044
2	9625	0	0,53	0,04	0	0	0	0	0,02	0,41	0	0,05	0,01	0,94	0,04
2	37915	0	0	0	0,2	0,75	0	0	0	0,05	0,69	0,03	0,2	0,08	0,048
2	33390	0	0,04	0,02	0,05	0,87	0	0,01	0	0,01	0,05	0,06	0,88	0,01	0,042
2	3658	0,06	0	0	0	0,62	0,31	0,01	0	0	0,05	0,62	0,02	0,31	0,048
2	4549	0	0	0,04	0	0,92	0	0	0,02	0,02	0,01	0,03	0,02	0,94	0,002
2	4007	0,06	0	0	0	0,94	0	0	0	0	0,06	0	0,94	0	0,044
2	100000	0	0,3	0	0	0	0	0	0,7	0	0	0,3	0,7	0	0,16
2	24075	0	0,24	0,04	0	0,7	0,02	0	0	0	0,01	0,02	0,25	0,72	0,044
2	85847	0	0	0	0,06	0,9	0,02	0	0,02	0	0,07	0,03	0	0,9	0,044
2	7991	0	0	0	0,19	0,8	0	0,01	0	0	0,8	0	0,19	0,01	0,046
2	17261	0	0	0,04	0	0,96	0	0	0	0	0,98	0	0	0,02	0,036
2	13113	0,01	0,16	0,04	0	0,79	0	0	0	0	0,02	0,81	0,16	0,01	0,044

2	13525	0	0	0,04	0	0	0	0	0,96	0	0	0,03	0	0,97	0
2	16284	0	0	0,04	0,02	0,89	0,03	0,01	0,01	0	0,03	0,07	0,86	0,04	0,04
2	25663	0	0,07	0	0,06	0,76	0	0	0,1	0,01	0,01	0,82	0,1	0,07	0,046
2	9463	0,06	0	0,02	0	0,91	0	0	0	0,01	0,06	0,03	0,91	0	0,042
2	16635	0	0,01	0	0,11	0,83	0,03	0	0	0,02	0,06	0,01	0,83	0,1	0,048
2	9776	0,06	0	0	0	0,92	0,02	0	0	0	0,06	0,29	0	0,65	0,048
2	10527	0	0,17	0,04	0	0,76	0	0	0,03	0	0,04	0,01	0,18	0,77	0,03
2	75137	0	0,13	0,03	0	0,37	0,45	0	0,02	0	0,01	0,02	0,6	0,37	0,044
2	26636	0,03	0,01	0,01	0	0	0,94	0	0,01	0	0,96	0,01	0	0,03	0,036
2	12540	0,01	0,17	0	0	0,71	0,11	0	0	0	0,31	0,48	0,03	0,18	0,044
2	40541	0,04	0,06	0,04	0	0	0	0	0,84	0,02	0,06	0	0,04	0,9	0,046
2	25612	0	0,97	0	0,03	0	0	0	0	0	0	0,99	0	0,01	0,032
2	20750	0,05	0	0,01	0,06	0,88	0	0	0	0	0,87	0,11	0,01	0,01	0,046
2	32816	0	0	0	0,06	0,02	0	0	0,92	0	0,02	0,94	0	0,04	0,038
2	11726	0,02	0	0,01	0,11	0,01	0,83	0	0	0,02	0,84	0,02	0,14	0	0,042
2	28327	0,04	0	0,04	0	0,24	0,43	0	0,06	0,19	0,16	0,1	0,5	0,24	0,048
2	23150	0	0,02	0,01	0,16	0,81	0	0	0	0	0,82	0,16	0,01	0,01	0,048
2	98485	0	0	0,04	0	0,34	0,62	0	0	0	0,03	0,04	0,59	0,34	0,018
2	20354	0	0	0	0,2	0,1	0,66	0	0,04	0	0,76	0,04	0,2	0	0,044
2	53545	0	0,01	0,04	0	0,92	0	0,02	0,01	0	0,02	0,92	0,06	0	0,03
2	46414	0	0,18	0	0	0,76	0,05	0	0,01	0	0,06	0	0,76	0,18	0,046
2	100000	0	0	0,9	0,09	0	0,01	0	0	0	0	0,9	0,1	0	0,452
2	14179	0	0	0,04	0	0,93	0	0,03	0	0	0,93	0,04	0	0,03	0,046
2	11510	0	0	0,04	0	0,02	0,41	0,01	0,48	0,04	0	0,06	0,87	0,07	0,02
2	100000	0	0	1	0	0	0	0	0	0	0	1	0	0	0,756
2	13420	0	0	0,04	0,02	0,25	0	0	0,69	0	0,03	0,95	0,02	0	0,014
2	22519	0	0	0	0,06	0,34	0	0	0,6	0	0,02	0,92	0	0,06	0,048
2	8838	0	0,44	0,04	0	0	0,02	0	0,15	0,35	0	0,19	0,02	0,79	0,048
2	42970	0	0,14	0,02	0,01	0,53	0	0	0,25	0,05	0,39	0,02	0,06	0,53	0,048
2	3989	0	0,94	0,01	0,05	0	0	0	0	0	0	0,01	0	0,99	0,046
2	94827	0,05	0,95	0	0	0	0	0	0	0	0	0,95	0	0,05	0,048
2	13792	0	0	0,04	0,06	0	0	0	0,88	0,02	0,88	0,02	0,04	0,06	0,038
2	32311	0	0,02	0,04	0	0,82	0,12	0	0	0	0,02	0,22	0,76	0	0,036

2	92956	0,03	0	0	0,01	0	0,94	0	0,01	0,01	0,03	0,01	0,96	0	0,044
2	26951	0	0,01	0,04	0,03	0,02	0	0	0,9	0	0,62	0,05	0,33	0	0,046
2	77300	0	0,03	0	0,06	0,91	0	0	0	0	0,06	0,91	0,03	0	0,044
2	9457	0,04	0,04	0	0	0,89	0,03	0	0	0	0,05	0,61	0,32	0,02	0,048
2	22043	0	0	0,04	0,03	0	0,11	0	0,8	0,02	0,11	0,06	0,8	0,03	0,038
2	12109	0,04	0	0,02	0	0,34	0,43	0	0,17	0	0,03	0	0,46	0,51	0,042
2	24643	0,05	0	0	0	0,51	0	0	0,22	0,22	0	0,52	0,19	0,29	0,046
2	45053	0,01	0	0,04	0	0	0,95	0	0	0	0	0,04	0,02	0,94	0,016
2,25	43098	0	0	0,04	0	0,96	0	0	0	0	0	0	0,11	0,89	0,006
2,25	32776	0,04	0	0,01	0	0,36	0,58	0,01	0	0	0	0,01	0,98	0,01	0,044
2,25	6499	0	0,1	0,03	0	0,01	0,86	0	0	0	0,01	0,25	0,74	0	0,048
2,25	50895	0	0	0,04	0	0,96	0	0	0	0	0,01	0,95	0,03	0,01	0,006
2,25	19165	0,05	0	0,04	0,01	0,8	0,1	0	0	0	0,1	0,05	0,04	0,81	0,032
2,25	19250	0	0	0,04	0	0	0,96	0	0	0	0	0,96	0	0,04	0,022
2,25	4168	0	0,95	0	0	0,02	0	0	0	0,03	0,05	0	0	0,95	0,034
2,25	10793	0	0,09	0,04	0	0	0	0	0,87	0	0,09	0,81	0,09	0,01	0,048
2,25	2443	0	0	0	0,13	0,01	0,02	0	0,83	0,01	0,15	0	0,83	0,02	0,048
2,25	35454	0	0	0,01	0,09	0	0,77	0,01	0	0,12	0	0,79	0,12	0,09	0,048
2,25	6881	0	0,07	0	0	0,93	0	0	0	0	0,93	0	0,07	0	0,048
2,25	31503	0,02	0,01	0,04	0	0,8	0	0	0,06	0,07	0,02	0,04	0,06	0,88	0,02
2,25	33489	0,01	0	0,04	0	0	0,7	0,02	0	0,23	0,04	0,71	0,23	0,02	0,024
2,25	60119	0	0	0	0,07	0,92	0	0	0,01	0	0,05	0,95	0	0	0,048
2,25	11723	0	0,14	0	0	0	0,39	0	0,47	0	0,47	0	0,39	0,14	0,048
2,25	33024	0	0,85	0	0	0,13	0	0	0	0,02	0,02	0,78	0	0,2	0,032
2,25	15338	0	0,12	0	0	0	0	0,04	0,81	0,03	0	0,85	0	0,15	0,048
2,25	5971	0	0,01	0,04	0	0,12	0	0	0,83	0	0	0,87	0	0,13	0,004
2,25	35173	0,01	0,04	0,02	0	0,87	0	0,06	0	0	0,88	0,04	0,08	0	0,044
2,25	29009	0	0,03	0,04	0,01	0,88	0	0	0	0,04	0,05	0,04	0	0,91	0,012
2,25	72676	0	0	0,04	0	0,04	0,76	0	0,16	0	0,81	0,15	0,04	0	0,044
2,25	42706	0,01	0	0,04	0,01	0,17	0,77	0	0	0	0,95	0,04	0	0,01	0,04
2,25	25382	0,01	0,4	0,04	0	0,54	0	0,01	0	0	0,01	0,58	0	0,41	0,04
2,25	25570	0	0	0	0,19	0,62	0	0	0,19	0	0,78	0,19	0	0,03	0,048
2,25	45177	0	0,09	0	0	0,01	0	0	0,9	0	0,73	0,01	0,09	0,17	0,048

2,25	13064	0,01	0	0,04	0,01	0,92	0,02	0	0	0	0,01	0,04	0,44	0,51	0,022
2,25	8700	0	0	0,04	0	0	0	0,01	0,94	0,01	0,01	0,05	0	0,94	0,008
2,25	9800	0	0,07	0,02	0,07	0,82	0	0	0,02	0	0,11	0	0,09	0,8	0,046
2,25	38524	0	0,01	0,04	0,02	0,92	0	0	0,01	0	0,12	0	0,82	0,06	0,038
2,25	44114	0	0	0,03	0	0	0,83	0,14	0	0	0,83	0,03	0,14	0	0,046
2,25	28510	0	0	0	0,12	0	0	0	0,87	0,01	0,01	0,08	0,91	0	0,044
2,25	15076	0,04	0	0	0,01	0	0,8	0	0,15	0	0,05	0	0,81	0,14	0,048
2,25	25057	0	0,16	0	0	0,79	0	0	0,01	0,04	0	0,2	0,79	0,01	0,044
2,25	20058	0	0,12	0	0	0,36	0	0,02	0,49	0,01	0	0,49	0,51	0	0,048
2,25	64319	0,06	0	0,03	0	0,09	0	0	0,82	0	0	0,12	0,06	0,82	0,048
2,25	52536	0,05	0,01	0	0	0,94	0	0	0	0	0	0,01	0,99	0	0,042
2,25	28228	0	0,02	0,01	0,06	0,89	0,02	0	0	0	0,02	0,02	0,08	0,88	0,048
2,25	21195	0,06	0	0	0,01	0,92	0	0	0	0,01	0,94	0,01	0,05	0	0,042
2,25	12432	0	0	0,04	0,04	0	0	0	0	0,92	0,04	0,03	0,01	0,92	0,038
2,25	36252	0	0,03	0	0	0	0,82	0,15	0	0	0,54	0	0,46	0	0,04
2,25	39673	0	0,01	0	0,07	0,87	0,02	0	0,03	0	0,1	0,03	0	0,87	0,048
2,25	39448	0	0,01	0,03	0	0,16	0,6	0,08	0	0,12	0,16	0,13	0,6	0,11	0,046
2,25	7659	0	0	0,04	0	0,96	0	0	0	0	0	0,04	0,25	0,71	0,01
2,25	16879	0,06	0	0	0	0,89	0,04	0	0,01	0	0,07	0,43	0,5	0	0,044
2,25	86927	0	0,04	0,03	0	0,87	0,05	0,01	0	0	0,87	0,08	0,01	0,04	0,03
2,25	53673	0,05	0,02	0	0	0	0	0	0,93	0	0,98	0,01	0	0,01	0,042
2,25	12946	0	0	0,01	0	0,06	0,09	0,11	0,73	0	0,03	0,2	0,71	0,06	0,046
2,25	16778	0,01	0,03	0	0,07	0,61	0,28	0	0	0	0,07	0,08	0,61	0,24	0,046
2,25	18854	0	0	0,04	0,04	0,15	0	0,04	0,73	0	0,01	0,04	0,91	0,04	0,044
2,25	25964	0,01	0,18	0	0	0,55	0,26	0	0	0	0,26	0	0,18	0,56	0,046
2,25	30699	0	0,03	0,04	0,05	0,75	0,01	0	0,11	0,01	0,03	0,06	0,88	0,03	0,04
2,25	31293	0	0,14	0,01	0	0,85	0	0	0	0	0,01	0,25	0,74	0	0,046
2,25	100000	0	0	0,98	0,02	0	0	0	0	0	0	0,97	0,03	0	0,534
2,25	34666	0,05	0	0,01	0,01	0,91	0	0	0,02	0	0,01	0,01	0,05	0,93	0,046
2,25	53210	0	0,1	0	0	0,9	0	0	0	0	0,76	0,08	0	0,16	0,046
2,25	8491	0	0,15	0,04	0	0,76	0	0	0	0,05	0,03	0,79	0,03	0,15	0,042
2,25	42245	0	0	0,04	0,01	0,5	0,06	0	0,39	0	0	0,55	0,45	0	0,028
2,25	67932	0	0	0,04	0	0,12	0,83	0	0,01	0	0,17	0,79	0	0,04	0,022

2,25	93944	0	0,01	0	0,05	0,93	0	0,01	0	0	0,05	0,01	0,01	0,93	0,046
2,25	15209	0	0,06	0,04	0	0,03	0,5	0	0,3	0,07	0,12	0,04	0,54	0,3	0,042
2,25	33652	0	0	0,04	0	0	0	0	0,96	0	0	0,02	0	0,98	0
2,25	60458	0,03	0,31	0	0	0,66	0	0	0	0	0,03	0,31	0	0,66	0,028
2,25	44133	0	0	0,04	0,09	0,85	0	0	0,02	0	0,01	0	0,96	0,03	0,046
2,25	95636	0	0	0,04	0,03	0,62	0,04	0,02	0,25	0	0	0,04	0,28	0,68	0,044
2,25	16520	0	0	0	0,22	0,72	0,06	0	0	0	0,72	0,06	0,22	0	0,036
2,25	14544	0	0,01	0,02	0,07	0,22	0	0	0,68	0	0,22	0	0,02	0,76	0,04
2,25	13250	0	0,01	0,04	0	0,63	0	0	0,32	0	0,32	0,01	0	0,67	0,016
2,25	37610	0,02	0,07	0,04	0	0,87	0	0	0	0	0,02	0,11	0,87	0	0,044
2,25	45460	0	0	0	0,3	0,68	0,01	0	0,01	0	0,98	0,01	0,01	0	0,048
2,25	37072	0,04	0	0,04	0	0,77	0,14	0	0,01	0	0,37	0	0,14	0,49	0,042
2,25	22405	0,02	0	0	0,02	0,94	0,01	0,01	0	0	0,04	0,94	0	0,02	0,048
2,25	36370	0,04	0,02	0,02	0	0	0	0	0,92	0	0,46	0,02	0,02	0,5	0,048
2,25	22705	0	0	0,04	0	0,37	0	0	0,59	0	0	0,41	0	0,59	0
2,25	35370	0	0,2	0	0,01	0,78	0,01	0	0	0	0	0,79	0,21	0	0,038
2,25	20514	0,02	0,07	0	0	0,61	0,3	0	0	0	0,24	0,13	0,61	0,02	0,042
2,25	80260	0	0	0,04	0	0,78	0	0	0,05	0,13	0,01	0,03	0,82	0,14	0,018
2,25	28411	0	0	0,04	0	0,56	0,05	0,01	0,03	0,31	0	0,12	0	0,88	0,012
2,25	46410	0	0	0,04	0	0,35	0	0	0,57	0,04	0,27	0,04	0,61	0,08	0,036
2,25	25002	0,01	0,02	0,03	0,04	0,82	0,08	0	0	0	0,17	0	0,82	0,01	0,04
2,25	10805	0,01	0,06	0	0	0,91	0,02	0	0	0	0,91	0	0,06	0,03	0,046
2,25	6798	0	0	0	0,05	0,95	0	0	0	0	0	0	0,05	0,95	0,046
2,25	47772	0,02	0	0,04	0,01	0,64	0	0	0,11	0,18	0,18	0,29	0	0,53	0,032
2,25	20295	0	0	0	0,08	0,92	0	0	0	0	0,08	0,92	0	0	0,048
2,25	8760	0	0	0,04	0	0	0	0	0,93	0,03	0	0,04	0,03	0,93	0,002
2,25	38765	0	0	0,04	0	0,27	0,69	0	0	0	0	0,34	0	0,66	0,006
2,25	7497	0	0,05	0,03	0,01	0	0,88	0,03	0	0	0	0,06	0,88	0,06	0,04
2,25	76549	0,01	0	0,04	0	0,94	0	0	0	0,01	0	0,05	0,01	0,94	0,008
2,25	13847	0	0	0	0,27	0,65	0,08	0	0	0	0,78	0,22	0	0	0,042
2,25	18021	0	0	0	0,18	0,81	0	0	0,01	0	0,81	0	0,18	0,01	0,034
2,25	19671	0	0	0	0,12	0,01	0	0	0,87	0	0	0,01	0,87	0,12	0,046
2,25	58799	0	0,03	0	0,11	0,86	0	0	0	0	0,86	0,11	0,03	0	0,048

2,25	35696	0,05	0	0	0	0	0,67	0	0,28	0	0,06	0	0,67	0,27	0,044
2,25	42421	0	0,11	0	0,02	0,85	0	0	0	0,02	0,11	0,02	0,87	0	0,048
2,25	15584	0,05	0,01	0	0	0,93	0,01	0	0	0	0,06	0	0,01	0,93	0,046
2,25	16924	0	0,2	0	0,03	0,75	0	0	0,02	0	0,03	0,02	0,2	0,75	0,046
2,25	38907	0	0,01	0,04	0	0,65	0,2	0	0,1	0	0	0	1	0	0,02
2,25	68472	0	0,15	0	0	0,02	0,83	0	0	0	0,3	0,21	0,09	0,4	0,046
2,25	17876	0	0,08	0,04	0	0,39	0	0	0,49	0	0,08	0,43	0,49	0	0,04
2,25	28723	0	0	0,04	0	0	0	0	0,89	0,07	0	0,11	0	0,89	0
2,25	7587	0	0,16	0,04	0	0,51	0	0	0,29	0	0	0,71	0,29	0	0,046
2,5	20659	0	0	0,04	0	0,02	0,93	0	0,01	0	0,01	0,61	0	0,38	0,024
2,5	35585	0	0,02	0,01	0,04	0,72	0	0,01	0,2	0	0,01	0,22	0,11	0,66	0,042
2,5	43125	0	0,26	0	0,01	0,57	0,05	0	0,11	0	0,19	0	0,05	0,76	0,046
2,5	59469	0	0	0	0,32	0,53	0,15	0	0	0	0,98	0,01	0	0,01	0,048
2,5	85378	0	0,02	0	0,05	0	0,88	0,05	0	0	0,04	0,79	0,12	0,05	0,04
2,5	32440	0	0,12	0	0,01	0	0,22	0	0,65	0	0,5	0	0	0,5	0,046
2,5	39084	0	0,06	0	0,02	0,9	0	0,02	0	0	0,41	0	0,59	0	0,048
2,5	18471	0	0,1	0,04	0	0	0	0	0,86	0	0	0,86	0,05	0,09	0,028
2,5	39328	0	0	0	0,1	0,9	0	0	0	0	0	0	0,9	0,1	0,046
2,5	32198	0,01	0,01	0,04	0	0,94	0	0	0	0	0,01	0,03	0,02	0,94	0,01
2,5	92230	0,01	0	0,01	0,08	0	0	0	0,9	0	0,11	0,88	0,01	0	0,038
2,5	27071	0	0,12	0	0,01	0,74	0	0,01	0,12	0	0,25	0,12	0,49	0,14	0,044
2,5	40615	0	0	0,04	0	0	0,96	0	0	0	0	0,04	0	0,96	0,006
2,5	23571	0	0	0,04	0	0	0,73	0	0	0,23	0	0,24	0,03	0,73	0,006
2,5	49209	0	0,01	0,04	0	0	0,88	0	0	0,07	0,01	0,07	0,88	0,04	0,02
2,5	34548	0	0,19	0	0	0,25	0,52	0	0,02	0,02	0,19	0,56	0	0,25	0,046
2,5	100000	0	0,08	0,92	0	0	0	0	0	0	0	0,7	0,03	0,27	0,344
2,5	9868	0,02	0	0,01	0,01	0	0	0,03	0,93	0	0,04	0,94	0	0,02	0,044
2,5	72479	0,07	0	0	0	0,76	0,17	0	0	0	0,81	0,17	0,02	0	0,046
2,5	33716	0	0,24	0,01	0	0,71	0,04	0	0	0	0,01	0,02	0,35	0,62	0,038
2,5	41608	0,05	0	0,01	0	0,19	0	0	0,75	0	0,75	0,06	0	0,19	0,046
2,5	29814	0	0	0	0,07	0	0	0	0,93	0	0	0,93	0	0,07	0,04
2,5	45934	0,04	0	0	0,04	0,91	0	0	0	0,01	0,05	0,04	0,91	0	0,048
2,5	20353	0,03	0,94	0,01	0	0	0	0	0,01	0,01	0,04	0,05	0,01	0,9	0,048

2,5	15170	0	0,02	0	0,1	0,85	0,02	0	0,01	0	0,11	0,02	0,86	0,01	0,04
2,5	44597	0	0	0	0,08	0,88	0	0	0,04	0	0,29	0,04	0,08	0,59	0,046
2,5	61419	0,01	0,03	0,04	0	0,81	0	0,01	0	0,1	0	0,07	0,92	0,01	0,048
2,5	36670	0	0,19	0,02	0,01	0	0,1	0	0	0,68	0,2	0,02	0,1	0,68	0,044
2,5	26018	0	0,06	0,04	0	0	0	0,02	0,88	0	0	0,06	0	0,94	0,022
2,5	8368	0	0	0	0,1	0,01	0,74	0	0	0,15	0	0,3	0,68	0,02	0,048
2,5	28818	0,05	0	0	0	0,92	0	0	0,03	0	0,08	0	0,92	0	0,044
2,5	8804	0	0	0,04	0,01	0	0,2	0,03	0	0,72	0	0,07	0	0,93	0,042
2,5	5913	0	0,26	0,01	0	0,73	0	0	0	0	0,27	0,09	0	0,64	0,046
2,5	57987	0	0	0,04	0	0,67	0,29	0	0	0	0,96	0	0,04	0	0,026
2,5	46080	0	0,08	0,02	0,01	0,89	0	0	0	0	0,08	0,03	0,89	0	0,032
2,5	24409	0	0	0	0,08	0,67	0	0	0,25	0	0,26	0	0,08	0,66	0,048
2,5	44188	0	0,01	0,04	0	0,17	0	0	0,78	0	0	0,04	0,18	0,78	0,012
2,5	44468	0,02	0	0,04	0	0,94	0	0	0	0	0	0,01	0,02	0,97	0,03
2,5	25632	0,01	0,05	0,01	0,02	0	0	0	0,91	0	0,94	0	0,06	0	0,048
2,5	100000	0	0,03	0,97	0	0	0	0	0	0	0	1	0	0	0,786
2,5	18178	0,02	0	0,04	0	0,03	0,03	0	0	0,88	0,08	0,03	0	0,89	0,034
2,5	13617	0,01	0,01	0,01	0,08	0,89	0	0	0	0	0,01	0,03	0,96	0	0,048
2,5	14075	0	0	0,04	0	0	0,02	0,01	0,93	0	0,01	0,04	0	0,95	0,008
2,5	25454	0	0	0,04	0	0	0,22	0	0,37	0,37	0	0,04	0,37	0,59	0,014
2,5	30036	0	0	0,04	0,01	0,94	0	0	0	0,01	0,01	0,91	0,04	0,04	0,01
2,5	35614	0	0	0,04	0	0,91	0	0	0	0,05	0,87	0,09	0	0,04	0,04
2,5	25511	0	0,19	0	0	0,56	0,21	0	0,01	0,03	0,21	0,26	0,13	0,4	0,048
2,5	11776	0	0	0,04	0	0,96	0	0	0	0	0,04	0,96	0	0	0,008
2,5	2854	0,01	0	0,04	0	0,17	0,62	0	0,04	0,12	0,02	0,06	0,66	0,26	0,038
2,5	24573	0	0,13	0	0	0,45	0,4	0	0	0,02	0,13	0,4	0,47	0	0,046
2,5	9191	0	0,16	0	0	0,65	0	0,01	0,18	0	0,39	0,15	0	0,46	0,04
2,5	29013	0,02	0	0,04	0	0,93	0	0	0,01	0	0	0,04	0,96	0	0,036
2,5	11945	0	0,02	0,04	0,03	0,09	0,53	0,04	0,25	0	0,04	0,38	0	0,58	0,046
2,5	15052	0,04	0	0,04	0	0,54	0	0	0	0,38	0,53	0,05	0	0,42	0,048
2,5	8197	0	0	0,04	0	0,86	0	0,1	0	0	0,86	0,04	0,1	0	0,04
2,5	26317	0	0,24	0	0	0,75	0	0	0,01	0	0	0,75	0,25	0	0,044
2,5	7054	0,04	0	0,03	0	0,91	0	0,02	0	0	0,04	0,91	0,02	0,03	0,048

2,5	57905	0	0	0	0,05	0,95	0	0	0	0	0,04	0,01	0	0,95	0,046
2,5	47639	0	0	0,04	0	0	0,01	0	0,91	0,04	0,02	0,04	0,93	0,01	0,018
2,5	38511	0	0,01	0,04	0,03	0,82	0,1	0	0	0	0,1	0,03	0,03	0,84	0,038
2,5	30203	0	0,05	0	0,06	0,62	0,04	0	0,23	0	0,09	0,02	0,19	0,7	0,04
2,5	17470	0	0,11	0	0	0,87	0,01	0	0	0,01	0,01	0	0,99	0	0,048
2,5	10392	0	0,06	0	0,05	0,83	0,01	0	0,05	0	0,06	0,1	0,01	0,83	0,046
2,5	27122	0	0,14	0,01	0,01	0,84	0	0	0	0	0	0,14	0,85	0,01	0,042
2,5	11477	0	0	0	0,07	0,93	0	0	0	0	0,07	0,93	0	0	0,046
2,5	67996	0,03	0	0,04	0	0,65	0,01	0	0,12	0,15	0,78	0,04	0,01	0,17	0,048
2,5	33814	0	0,05	0	0,03	0,9	0	0,02	0	0	0,87	0	0,13	0	0,038
2,5	8494	0	0	0,04	0	0,76	0	0,01	0	0,19	0	0,04	0,96	0	0,022
2,5	59109	0,06	0	0	0	0,82	0,12	0	0	0	0,06	0,82	0,12	0	0,048
2,5	13421	0,01	0,21	0,03	0	0,75	0	0	0	0	0,23	0,01	0,05	0,71	0,048
2,5	40643	0	0	0	0,1	0,69	0,01	0	0,2	0	0,2	0,69	0,11	0	0,048
2,5	33190	0,06	0	0,02	0	0,82	0	0	0,1	0	0,02	0,82	0,16	0	0,044
2,5	17758	0	0,09	0,04	0	0,42	0	0	0,08	0,37	0,03	0,14	0,43	0,4	0,04
2,5	66299	0	0,25	0	0	0,63	0,06	0	0,06	0	0,06	0,64	0,12	0,18	0,048
2,5	28655	0,03	0	0,04	0	0,62	0,31	0	0	0	0,95	0,04	0,01	0	0,044
2,5	68336	0	0	0	0,07	0,83	0	0	0,1	0	0,01	0,92	0,07	0	0,048
2,5	26612	0,01	0,06	0,04	0	0	0,65	0	0,24	0	0,02	0,04	0,87	0,07	0,038
2,5	46242	0	0,08	0	0,04	0,71	0,17	0	0	0	0,06	0,09	0,69	0,16	0,044
2,5	19553	0	0	0	0,22	0,43	0,35	0	0	0	0,65	0,35	0	0	0,048
2,5	13720	0	0	0,04	0	0	0	0	0,96	0	0,96	0	0	0,04	0,024
2,5	21488	0	0	0,04	0,02	0,94	0	0	0	0	0,81	0,12	0,03	0,04	0,042
2,5	15569	0	0	0,04	0	0,95	0	0	0	0,01	0,03	0,95	0	0,02	0,004
2,5	6361	0,01	0	0,03	0	0,82	0,06	0,08	0	0	0,78	0,08	0,14	0	0,046
2,5	79887	0,08	0	0	0	0,92	0	0	0	0	1	0	0	0	0,048
2,5	16484	0	0,22	0	0	0,1	0,68	0	0	0	0,21	0,1	0,01	0,68	0,046
2,5	55148	0,06	0	0	0	0	0,88	0	0,06	0	0,06	0	0,94	0	0,046
2,5	18100	0	0	0,04	0,05	0,33	0	0,01	0	0,57	0,32	0,57	0	0,11	0,044
2,5	8610	0	0	0,04	0	0,93	0	0	0,01	0,02	0,13	0,84	0,02	0,01	0,018
2,5	44534	0,05	0	0	0	0,95	0	0	0	0	0	0	1	0	0,046
2,5	33207	0,02	0,11	0	0	0	0,61	0	0,26	0	0,26	0	0,13	0,61	0,048

2,5	25976	0,02	0	0,04	0	0	0	0,01	0,93	0	0	0,16	0,05	0,79	0,034
2,5	50585	0	0	0	0,06	0,92	0	0	0	0,02	0	0,92	0,08	0	0,046
2,5	41178	0,01	0,41	0,04	0,01	0	0	0	0,53	0	0	0,03	0,03	0,94	0,036
2,5	22190	0,02	0,02	0,04	0	0	0,9	0	0,02	0	0,04	0,36	0,6	0	0,048
2,5	4601	0	0	0,04	0	0,89	0	0,07	0	0	0	0,03	0,07	0,9	0,046
2,5	27546	0	0	0	0,07	0,03	0,66	0,16	0	0,08	0,77	0	0,16	0,07	0,04
2,5	27918	0,06	0	0	0	0	0	0	0,94	0	0	0	1	0	0,048
2,5	27361	0,02	0	0,04	0	0,92	0	0,02	0	0	0,94	0,04	0,02	0	0,046
2,5	43100	0	0	0,04	0	0	0	0	0,96	0	0	0,09	0,91	0	0,024
2,5	27041	0	0,02	0,03	0	0,02	0	0,06	0,87	0	0	0,06	0,92	0,02	0,046
2,75	20287	0,05	0	0,03	0	0,9	0	0	0	0,02	0,07	0,84	0,03	0,06	0,044
2,75	15392	0,04	0	0,02	0	0,94	0	0	0	0	0,98	0,01	0	0,01	0,044
2,75	44064	0	0	0	0,09	0,67	0,21	0,01	0,01	0,01	0,23	0,7	0,06	0,01	0,048
2,75	8436	0	0,07	0,04	0	0	0,7	0	0	0,19	0,19	0,75	0	0,06	0,038
2,75	45033	0,01	0,01	0,03	0,03	0,89	0	0	0,03	0	0,9	0,08	0	0,02	0,044
2,75	100000	0	0	1	0	0	0	0	0	0	0	1	0	0	0,702
2,75	18084	0,01	0,11	0,04	0	0	0,81	0,02	0,01	0	0	0,86	0,01	0,13	0,048
2,75	33069	0	0,01	0	0,1	0,89	0	0	0	0	0,05	0,04	0,88	0,03	0,046
2,75	15118	0,01	0	0,04	0	0	0	0,02	0,55	0,38	0	0,04	0,02	0,94	0,036
2,75	12269	0	0,03	0,03	0	0	0	0,07	0,87	0	0	0,06	0,04	0,9	0,048
2,75	22791	0,01	0	0,04	0,01	0	0	0	0,93	0,01	0,01	0,05	0,01	0,93	0,024
2,75	21173	0,01	0	0	0,06	0,93	0	0	0	0	0	0,92	0,08	0	0,048
2,75	20069	0	0	0,02	0,08	0,73	0	0,03	0	0,14	0	0,05	0,87	0,08	0,048
2,75	19778	0	0,93	0	0	0	0	0,02	0,05	0	0	0,93	0,02	0,05	0,048
2,75	75959	0,03	0	0	0,03	0,78	0,09	0	0,07	0	0,17	0	0	0,83	0,038
2,75	22463	0	0	0,04	0	0,72	0,24	0	0	0	0	0,04	0,74	0,22	0,024
2,75	13548	0	0	0,04	0,07	0,89	0	0	0	0	0,02	0	0,87	0,11	0,032
2,75	96114	0,05	0	0	0	0,92	0,02	0	0,01	0	0,04	0,01	0,1	0,85	0,044
2,75	97098	0	0	0,04	0	0	0	0,04	0,84	0,08	0	0,08	0,92	0	0,044
2,75	32356	0	0,15	0	0	0,79	0,04	0	0	0,02	0,02	0,04	0,94	0	0,048
2,75	100000	0	0,01	0,96	0,01	0	0,01	0	0	0,01	0,02	0,96	0,01	0,01	0,608
2,75	28278	0	0	0,04	0	0	0	0	0,4	0,56	0,4	0,04	0	0,56	0,022
2,75	22855	0	0	0,04	0	0,96	0	0	0	0	0	0,96	0,03	0,01	0

2,75	29058	0,05	0	0,01	0	0	0,02	0	0,92	0	0	0,03	0,05	0,92	0,042
2,75	20259	0	0,07	0	0	0,92	0	0	0	0,01	0,88	0,05	0,04	0,03	0,04
2,75	4949	0	0,38	0,04	0	0	0	0	0	0,58	0	0,03	0,01	0,96	0,044
2,75	18817	0	0	0	0,14	0,6	0	0	0,01	0,25	0,71	0	0,05	0,24	0,044
2,75	15043	0	0,06	0,04	0	0,44	0	0,03	0,25	0,18	0,24	0,34	0,39	0,03	0,048
2,75	43535	0,03	0	0,04	0	0	0	0	0,79	0,14	0	0,8	0,11	0,09	0,042
2,75	58611	0	0,03	0	0	0,59	0,23	0,09	0,06	0	0,23	0	0,68	0,09	0,046
2,75	35159	0	0	0,04	0	0,52	0,28	0,09	0	0,07	0,2	0,11	0,09	0,6	0,048
2,75	11717	0,01	0	0,02	0,08	0,88	0,01	0	0	0	0,1	0,02	0,88	0	0,044
2,75	18889	0	0	0,04	0	0	0,01	0	0,93	0,02	0,06	0,91	0	0,03	0,014
2,75	17948	0	0	0,04	0	0,85	0,11	0	0	0	0,52	0,37	0	0,11	0,034
2,75	11901	0	0	0,04	0	0,09	0	0	0,87	0	0,01	0,09	0	0,9	0,002
2,75	94585	0	0	0,04	0	0,86	0,1	0	0	0	0	0,04	0,1	0,86	0,012
2,75	67656	0,01	0,05	0	0,07	0,13	0,01	0	0,73	0	0,79	0,13	0,08	0	0,048
2,75	100000	0,01	0	0,99	0	0	0	0	0	0	0,14	0,85	0	0,01	0,756
2,75	36909	0	0,27	0	0,01	0,65	0,07	0	0	0	0	0,07	0,28	0,65	0,048
2,75	14499	0,05	0,09	0	0	0	0,8	0	0	0,06	0,14	0,06	0	0,8	0,048
2,75	25276	0,01	0,03	0	0,09	0,78	0,07	0	0,02	0	0,78	0,01	0,18	0,03	0,042
2,75	29017	0	0	0,04	0,05	0,86	0,03	0,02	0	0	0,42	0,09	0,49	0	0,044
2,75	41252	0,01	0,13	0,04	0,03	0,09	0	0	0,02	0,68	0	0,06	0,06	0,88	0,044
2,75	76223	0,04	0	0,01	0	0,91	0,04	0	0	0	0,95	0	0	0,05	0,04
2,75	80473	0,01	0	0	0,22	0	0,76	0,01	0	0	0,9	0	0,09	0,01	0,046
2,75	66517	0,02	0	0,02	0	0,31	0	0,05	0,6	0	0,05	0,05	0,59	0,31	0,046
2,75	7770	0	0	0,04	0,02	0,11	0	0	0,81	0,02	0,01	0,86	0,02	0,11	0,018
2,75	30914	0	0,02	0,04	0	0,88	0	0	0,06	0	0	0,07	0,91	0,02	0,042
2,75	81677	0	0,14	0	0,01	0	0,85	0	0	0	0	0,99	0,01	0	0,048
2,75	7179	0	0	0,04	0	0,93	0	0,02	0	0,01	0,01	0,97	0,02	0	0,014
2,75	17547	0	0	0,04	0	0,87	0	0	0,09	0	0	0,04	0	0,96	0
2,75	46855	0,04	0,05	0	0	0	0,82	0	0,01	0,08	0,04	0,82	0,08	0,06	0,044
2,75	2409	0	0	0,04	0	0,88	0	0,01	0,07	0	0	0,04	0,88	0,08	0,032
2,75	35486	0	0	0,04	0,02	0,93	0	0	0	0,01	0,93	0	0,01	0,06	0,048
2,75	38536	0	0,42	0	0	0,57	0	0	0,01	0	0,12	0,57	0	0,31	0,04
2,75	54365	0	0	0	0,07	0,92	0	0	0	0,01	0,06	0,92	0,01	0,01	0,046

2,75	24890	0	0	0,03	0	0,86	0,01	0,08	0,02	0	0,1	0	0,89	0,01	0,046
2,75	28480	0,02	0,09	0,04	0	0,56	0	0	0,27	0,02	0,09	0,07	0,26	0,58	0,048
2,75	28999	0,02	0	0,04	0,06	0,01	0	0	0,87	0	0,67	0,04	0,24	0,05	0,048
2,75	24655	0	0	0,01	0,09	0,45	0,44	0	0,01	0	0	0,01	0,51	0,48	0,048
2,75	61720	0	0	0,04	0	0,93	0	0,02	0	0,01	0	0,04	0,94	0,02	0,038
2,75	25308	0,03	0	0,02	0	0,2	0,01	0,01	0,73	0	0,01	0,22	0,73	0,04	0,04
2,75	62439	0	0	0,04	0	0,91	0	0,05	0	0	0	0,95	0,05	0	0,046
2,75	59580	0,03	0	0,04	0	0,91	0	0	0	0,02	0,03	0,95	0	0,02	0,038
2,75	12103	0	0,01	0,04	0	0,11	0	0	0,82	0,02	0,01	0,35	0,62	0,02	0,036
2,75	47017	0	0	0	0,13	0,6	0	0	0,27	0	0,27	0	0,73	0	0,046
2,75	17812	0	0,1	0	0,06	0	0	0	0,84	0	0	0,16	0,39	0,45	0,044
2,75	12053	0	0,19	0	0,03	0,55	0	0	0,22	0,01	0,01	0,07	0,19	0,73	0,044
2,75	69534	0	0,01	0,04	0	0	0	0,01	0,23	0,71	0	0,95	0,01	0,04	0,024
2,75	87465	0	0,26	0	0	0,65	0,01	0	0,06	0,02	0,26	0,16	0,06	0,52	0,046
2,75	24813	0,04	0,04	0,01	0,01	0,02	0,85	0	0,03	0	0,03	0,07	0,01	0,89	0,044
2,75	33654	0,01	0	0,04	0	0,54	0	0	0	0,41	0,01	0,03	0,3	0,66	0,012
2,75	16790	0	0,26	0,03	0	0,01	0	0	0,7	0	0,2	0,03	0,06	0,71	0,038
2,75	24490	0,01	0	0	0,1	0	0,89	0	0	0	0	0,1	0,9	0	0,044
2,75	16009	0,06	0,02	0,01	0	0,91	0	0	0	0	0,06	0,02	0	0,92	0,048
2,75	28197	0,01	0	0	0,12	0,87	0	0	0	0	0,07	0	0,93	0	0,048
2,75	55970	0,02	0	0,04	0	0,92	0	0,01	0	0,01	0,94	0,05	0,01	0	0,046
2,75	32211	0	0,08	0,03	0	0,49	0,37	0	0	0,03	0,49	0,11	0,37	0,03	0,048
2,75	56069	0	0,17	0	0	0	0,82	0	0,01	0	0	0,98	0,01	0,01	0,046
2,75	3723	0	0	0,04	0	0	0	0	0,96	0	0	0,07	0,93	0	0,014
2,75	76422	0,05	0	0,01	0	0,88	0,01	0	0,05	0	0,93	0,05	0,02	0	0,048
2,75	48086	0,05	0	0	0,01	0,59	0	0	0,35	0	0,35	0	0,06	0,59	0,042
2,75	76061	0	0,04	0	0,08	0,87	0	0	0,01	0	0,08	0,03	0,89	0	0,048
2,75	57588	0	0,08	0	0,01	0,83	0,05	0	0	0,03	0,97	0,01	0,02	0	0,048
2,75	77926	0	0	0,04	0	0	0,96	0	0	0	0	0,04	0,96	0	0,036
2,75	5073	0	0	0	0,07	0,83	0	0	0,1	0	0,1	0	0,07	0,83	0,046
2,75	16347	0,04	0	0,01	0	0	0,94	0	0,01	0	0,96	0	0	0,04	0,046
2,75	34948	0	0,24	0	0	0,53	0	0	0,23	0	0,24	0,53	0	0,23	0,048
2,75	70699	0,02	0	0,04	0	0,93	0,01	0	0	0	0,01	0,06	0,93	0	0,026

2,75	27930	0,03	0,04	0,01	0	0,92	0	0	0	0	0,03	0,01	0,92	0,04	0,044
2,75	22301	0	0,02	0,02	0,1	0,85	0	0	0,01	0	0,99	0,01	0	0	0,046
2,75	19841	0,04	0,03	0,01	0	0,9	0	0	0,01	0,01	0,03	0,02	0,91	0,04	0,046
2,75	69069	0,02	0,1	0,04	0	0,06	0	0	0,75	0,03	0,07	0,2	0,03	0,7	0,038
2,75	86640	0,02	0	0,04	0	0	0	0	0,94	0	0	0,38	0	0,62	0,016
2,75	8131	0	0,11	0	0	0,45	0,3	0	0,1	0,04	0,53	0,32	0,01	0,14	0,048
2,75	89178	0,02	0	0,04	0	0,94	0	0	0	0	0	0,02	0,94	0,04	0,044
2,75	100000	0	0	0,99	0	0,01	0	0	0	0	0	0,99	0	0,01	0,6
2,75	36274	0,06	0	0	0	0,94	0	0	0	0	0,06	0,94	0	0	0,048
2,75	100000	0,07	0	0,92	0	0	0	0	0,01	0	0,01	0,92	0,07	0	0,498
2,75	84980	0	0	0	0,31	0,68	0,01	0	0	0	0,99	0	0	0,01	0,048
3	15363	0,01	0	0	0,06	0,84	0	0	0,09	0	0	0,84	0,08	0,08	0,044
3	20287	0	0	0,03	0,13	0,38	0,28	0	0,17	0,01	0,68	0,01	0,31	0	0,042
3	38653	0	0	0,04	0,05	0,89	0	0,02	0	0	0,94	0	0,02	0,04	0,038
3	28230	0,05	0	0	0	0	0	0	0,75	0,2	0,05	0,2	0,71	0,04	0,04
3	4918	0	0	0,04	0	0	0,49	0	0,04	0,43	0,02	0,04	0,47	0,47	0,034
3	31438	0,01	0,02	0,04	0,01	0,86	0	0	0	0,06	0,88	0,04	0	0,08	0,046
3	52714	0	0	0,04	0	0,05	0,91	0	0	0	0,08	0,03	0,88	0,01	0,024
3	32934	0	0,1	0,02	0	0,85	0,02	0	0,01	0	0,01	0,12	0,87	0	0,046
3	11353	0	0,06	0,04	0	0,87	0	0	0	0,03	0	0,04	0,96	0	0,036
3	4873	0,01	0	0,04	0	0,56	0	0,04	0	0,35	0,05	0,04	0,75	0,16	0,042
3	60486	0	0,12	0	0	0	0,85	0,03	0	0	0,12	0,03	0,85	0	0,048
3	10519	0,01	0	0,04	0,04	0,83	0,04	0,03	0	0,01	0,91	0,04	0,04	0,01	0,044
3	17033	0	0	0,04	0	0	0,62	0	0,33	0,01	0,01	0,01	0,94	0,04	0,024
3	95418	0	0	0	0,12	0,86	0	0	0	0,02	0,02	0,12	0,86	0	0,048
3	19940	0	0	0	0,14	0	0,79	0	0	0,07	0,01	0	0,92	0,07	0,048
3	39361	0,02	0,05	0	0,05	0,85	0,02	0	0,01	0	0,05	0,02	0,88	0,05	0,048
3	43436	0	0	0	0,21	0,76	0	0	0,03	0	0,79	0	0,21	0	0,042
3	100000	0	0	0,99	0	0	0	0	0	0,01	0	1	0	0	0,602
3	39761	0,06	0	0	0	0,76	0,18	0	0	0	0,06	0,76	0,17	0,01	0,042
3	53500	0	0	0	0,35	0,65	0	0	0	0	1	0	0	0	0,048
3	58161	0	0,17	0,01	0	0,76	0,05	0	0	0,01	0,2	0,74	0	0,06	0,036
3	30882	0,01	0,13	0	0	0,83	0	0,02	0	0,01	0,14	0,85	0	0,01	0,048

3	8889	0,02	0,32	0,02	0	0,26	0,29	0	0	0,09	0,03	0,33	0	0,64	0,044
3	15859	0	0,08	0	0	0,89	0	0	0,03	0	0,91	0,03	0,06	0	0,044
3	11695	0	0,13	0	0	0,01	0	0	0,83	0,03	0	0,01	0,96	0,03	0,046
3	45249	0,06	0	0	0	0,16	0,76	0,02	0	0	0,78	0,06	0,16	0	0,046
3	24294	0	0	0	0,31	0	0,67	0,01	0	0,01	1	0	0	0	0,044
3	10385	0,01	0	0,04	0,03	0	0,84	0	0	0,08	0,03	0,04	0,85	0,08	0,038
3	9349	0	0,08	0	0	0,91	0	0,01	0	0	0,9	0,01	0,09	0	0,042
3	37943	0	0	0,04	0	0,01	0,01	0	0,08	0,86	0,02	0,04	0,08	0,86	0,012
3	30586	0	0	0,04	0,01	0	0,05	0,03	0	0,87	0	0,06	0,09	0,85	0,04
3	72542	0	0	0,04	0	0,96	0	0	0	0	0	0	0	1	0
3	100000	0	0	1	0	0	0	0	0	0	0	1	0	0	0,686
3	20463	0	0	0	0,08	0,92	0	0	0	0	0	0	0,52	0,48	0,046
3	85315	0	0	0,04	0	0,92	0,03	0,01	0	0	0,92	0,03	0,01	0,04	0,028
3	96449	0	0	0	0,06	0,71	0,01	0,06	0	0,16	0,32	0	0,52	0,16	0,046
3	20751	0	0	0,04	0	0,96	0	0	0	0	1	0	0	0	0,036
3	20132	0	0,01	0,03	0,06	0,9	0	0	0	0	0	0,03	0,97	0	0,042
3	24643	0	0,24	0,04	0	0,26	0	0	0,01	0,45	0	0,55	0	0,45	0,042
3	47971	0,01	0	0,04	0	0,92	0	0	0,03	0	0,01	0,07	0,92	0	0,028
3	47641	0,06	0	0	0,01	0	0,84	0	0	0,09	0,06	0,84	0,01	0,09	0,042
3	19396	0	0	0	0,33	0,67	0	0	0	0	1	0	0	0	0,044
3	99852	0	0	0,04	0	0,66	0,25	0,01	0,04	0	0,02	0,66	0,28	0,04	0,03
3	100000	0	0,6	0	0,02	0	0,3	0	0,06	0,02	0,48	0,2	0,32	0	0,388
3	7999	0,01	0	0,04	0,06	0	0,89	0	0	0	0,01	0,89	0	0,1	0,046
3	33661	0	0	0	0,15	0,81	0	0	0,04	0	0,14	0,01	0,84	0,01	0,044
3	9298	0,04	0	0,04	0	0,2	0	0,01	0	0,71	0,2	0,04	0,05	0,71	0,046
3	8330	0	0,03	0,04	0	0,9	0,02	0,01	0	0	0,93	0,02	0,05	0	0,042
3	5779	0,05	0	0	0	0,01	0,93	0,01	0	0	0,93	0,02	0,05	0	0,048
3	16930	0,01	0	0,04	0	0,05	0	0	0,17	0,73	0,24	0,05	0,05	0,66	0,028
3	7181	0	0	0,04	0	0,09	0	0,08	0,48	0,31	0	0,12	0,48	0,4	0,046
3	26114	0	0,13	0	0	0,87	0	0	0	0	0	0	1	0	0,046
3	18449	0,01	0,06	0,04	0	0,19	0,69	0,01	0	0	0,2	0,1	0,69	0,01	0,044
3	42037	0	0	0,04	0	0,33	0	0	0,57	0,06	0	0,61	0,39	0	0,016
3	10309	0	0	0,04	0	0	0,78	0	0,18	0	0,01	0,03	0,18	0,78	0,012

3	9990	0,02	0,09	0	0,02	0,63	0	0	0,24	0	0,09	0,63	0,18	0,1	0,048
3	69162	0	0,16	0,01	0	0,67	0	0,01	0,15	0	0,27	0,16	0,05	0,52	0,046
3	5860	0,03	0,03	0	0,01	0,89	0	0	0,02	0,02	0,94	0,04	0,01	0,01	0,044
3	70572	0	0,08	0	0	0,84	0,03	0	0,03	0,02	0,89	0,08	0	0,03	0,044
3	12780	0	0,06	0,02	0	0,8	0	0	0,1	0,02	0,84	0	0,1	0,06	0,048
3	25348	0	0,21	0	0	0,77	0,02	0	0	0	0	0,62	0,36	0,02	0,044
3	32904	0,01	0	0,04	0	0,28	0	0	0	0,67	0	0,33	0	0,67	0,022
3	4968	0	0,08	0	0,04	0,41	0	0	0,47	0	0,47	0,48	0	0,05	0,048
3	33469	0	0,02	0,04	0	0	0,04	0,01	0,89	0	0	0,09	0,89	0,02	0,036
3	51628	0	0	0,04	0	0	0,02	0	0,9	0,04	0	0,08	0,92	0	0,024
3	5652	0	0	0	0,11	0,84	0	0,04	0,01	0	0,78	0,01	0	0,21	0,042
3	36435	0	0	0	0,04	0,66	0	0,02	0,28	0	0	0,94	0,04	0,02	0,048
3	47329	0,02	0	0,04	0	0,73	0	0	0,02	0,19	0,75	0,23	0,02	0	0,044
3	100000	0	0	1	0	0	0	0	0	0	0	1	0	0	0,632
3	85723	0,06	0	0	0	0,94	0	0	0	0	0	0	0,06	0,94	0,046
3	27506	0	0,04	0,04	0	0,81	0	0	0	0,11	0,68	0,17	0,04	0,11	0,044
3	45661	0	0,08	0	0	0,82	0,1	0	0	0	0,88	0,02	0	0,1	0,042
3	4515	0	0	0	0,08	0	0,82	0,1	0	0	0,82	0	0,1	0,08	0,038
3	10582	0	0,01	0,04	0	0,64	0,01	0	0,05	0,25	0,03	0,29	0,31	0,37	0,016
3	77085	0	0,31	0	0	0,68	0	0,01	0	0	0	0	0,31	0,69	0,042
3	3429	0	0	0,04	0	0,9	0	0	0	0,06	0,78	0,16	0,06	0	0,03
3	81795	0	0	0,04	0	0,96	0	0	0	0	0	0,04	0,96	0	0,02
3	100000	0	0	0,41	0	0	0,29	0	0	0,3	0	0,41	0,59	0	0,24
3	26416	0	0,05	0,04	0	0,87	0	0	0	0,04	0,06	0,01	0,02	0,91	0,018
3	23042	0,01	0	0,01	0,02	0,64	0,22	0,1	0	0	0,64	0,04	0,29	0,03	0,046
3	26298	0	0	0,04	0	0	0,27	0	0,18	0,51	0,27	0	0,51	0,22	0,026
3	22343	0	0,2	0,01	0	0,03	0	0	0	0,76	0,23	0,01	0	0,76	0,046
3	72340	0	0,01	0,04	0	0,91	0,04	0	0	0	0,01	0,04	0,92	0,03	0,038
3	84483	0,01	0,01	0,02	0	0,85	0,03	0,08	0	0	0,86	0,02	0,08	0,04	0,046
3	19043	0	0	0,04	0,06	0,9	0	0	0	0	0,01	0,06	0,93	0	0,046
3	14349	0,01	0,17	0	0	0,76	0	0	0,06	0	0,01	0,76	0,23	0	0,048
3	15468	0,05	0	0	0	0	0,95	0	0	0	0	0,01	0,99	0	0,048
3	59881	0	0,45	0,01	0,04	0,49	0	0	0	0,01	0,03	0	0,03	0,94	0,048

3	29124	0	0,01	0,04	0,01	0,93	0,01	0	0	0	0,93	0,04	0,01	0,02	0,042
3	7933	0,01	0	0	0,09	0,68	0	0	0,22	0	0	0,24	0,75	0,01	0,044
3	36532	0	0,05	0,04	0	0	0	0	0,77	0,14	0,17	0,06	0,77	0	0,032
3	34132	0,01	0	0,04	0	0,61	0,09	0,05	0,04	0,16	0	0,57	0,1	0,33	0,044
3	15483	0	0	0,04	0,04	0	0	0	0,06	0,86	0,88	0,09	0	0,03	0,04
3	23990	0	0,03	0	0,09	0,85	0	0	0,03	0	0,03	0	0,88	0,09	0,044
3	25030	0,05	0	0	0,02	0,93	0	0	0	0	0,06	0,94	0	0	0,04
3	10899	0,01	0	0	0,06	0,8	0,05	0	0	0,08	0,08	0,18	0,67	0,07	0,034
3	29548	0	0	0,04	0	0,43	0,45	0,01	0	0,07	0,01	0,45	0,43	0,11	0,016
3	63017	0,04	0	0,04	0,02	0,88	0	0	0	0,02	0,06	0,04	0	0,9	0,046
3	25416	0,04	0	0,03	0	0,93	0	0	0	0	0,93	0,03	0,04	0	0,048
3	19658	0	0,62	0,01	0	0	0	0	0,32	0,05	0	0,45	0,05	0,5	0,038
3,25	12734	0	0	0	0,13	0,87	0	0	0	0	0,06	0	0,87	0,07	0,048
3,25	16220	0	0	0,02	0,1	0,48	0,4	0	0	0	0,1	0,02	0,48	0,4	0,048
3,25	22453	0	0,2	0	0	0,07	0,73	0	0	0	0,18	0	0,07	0,75	0,048
3,25	80058	0	0	0,02	0	0	0,01	0,09	0,84	0,04	0	0,84	0,11	0,05	0,048
3,25	14192	0,01	0,04	0,04	0	0,9	0	0,01	0	0	0,01	0,95	0,03	0,01	0,02
3,25	62196	0	0	0,04	0	0,82	0,07	0	0,07	0	0,11	0,05	0,02	0,82	0,008
3,25	100000	0	0,01	0,92	0	0	0	0	0	0,07	0	0,65	0,35	0	0,422
3,25	41876	0	0	0,04	0	0	0,04	0,04	0,09	0,79	0,04	0,17	0,79	0	0,048
3,25	62003	0	0	0,04	0	0	0,65	0	0,31	0	0,3	0,11	0	0,59	0,022
3,25	76083	0	0	0	0,1	0	0,1	0	0,75	0,05	0	0,1	0,75	0,15	0,048
3,25	33118	0,01	0,1	0	0	0,51	0	0	0,38	0	0,5	0,02	0,1	0,38	0,046
3,25	11650	0,01	0	0,04	0	0	0	0,02	0,78	0,15	0	0,08	0,81	0,11	0,046
3,25	7470	0	0,18	0	0	0	0,82	0	0	0	0,17	0	0,29	0,54	0,046
3,25	100000	0,01	0	0,98	0,01	0	0	0	0	0	0	1	0	0	0,72
3,25	68213	0	0,09	0	0	0,77	0,04	0	0,1	0	0,82	0	0,14	0,04	0,046
3,25	18813	0	0	0,04	0,02	0,17	0,44	0	0	0,33	0,61	0,04	0,03	0,32	0,032
3,25	26722	0	0	0,02	0,16	0,79	0	0,02	0	0,01	0,9	0,08	0	0,02	0,048
3,25	24847	0	0	0,03	0	0	0	0,06	0	0,91	0,01	0,08	0	0,91	0,04
3,25	9255	0,07	0	0	0	0,91	0	0	0,02	0	0	0,93	0,07	0	0,048
3,25	8025	0	0	0,04	0	0,01	0,73	0	0	0,22	0,73	0,04	0,01	0,22	0,03
3,25	9778	0,05	0,04	0	0	0,9	0	0	0	0,01	0,01	0,08	0,85	0,06	0,046

3,25	32254	0	0,11	0	0	0,85	0	0,03	0	0,01	0,11	0,01	0,88	0	0,046
3,25	54175	0	0,3	0	0	0,7	0	0	0	0	0,27	0,03	0	0,7	0,034
3,25	28531	0,02	0	0	0,17	0,74	0,07	0	0	0	0,83	0	0,17	0	0,046
3,25	28728	0	0	0	0,32	0,68	0	0	0	0	0,99	0	0	0,01	0,048
3,25	100000	0	0	0,98	0	0,01	0	0,01	0	0	0,01	0,98	0	0,01	0,58
3,25	12349	0,02	0,27	0	0	0,06	0	0	0	0,65	0	0,35	0	0,65	0,044
3,25	22116	0	0	0,01	0,05	0,92	0,02	0	0	0	0	0,05	0,01	0,94	0,046
3,25	13450	0	0	0,04	0	0,7	0	0	0,02	0,24	0,02	0,69	0,01	0,28	0,004
3,25	29866	0	0,07	0,03	0	0,76	0,12	0	0,01	0,01	0,74	0	0,09	0,17	0,046
3,25	69963	0,03	0,04	0,03	0	0	0	0	0	0,9	0	0,07	0	0,93	0,038
3,25	20045	0,05	0	0,04	0	0,52	0,06	0	0	0,33	0	0,04	0,06	0,9	0,048
3,25	41900	0	0	0,01	0,09	0,07	0,83	0	0	0	0,09	0,83	0	0,08	0,046
3,25	29143	0,01	0,02	0	0,06	0,91	0	0	0	0	0,02	0,01	0,06	0,91	0,046
3,25	15515	0	0	0,04	0,03	0,01	0,01	0	0,91	0	0,02	0,01	0,04	0,93	0,032
3,25	4621	0	0	0,04	0	0,01	0,89	0,02	0,04	0	0,02	0,04	0,89	0,05	0,038
3,25	100000	0,01	0	0,99	0	0	0	0	0	0	0,12	0,87	0	0,01	0,784
3,25	52582	0	0	0,04	0	0,79	0,01	0,01	0	0,15	0,32	0,05	0,62	0,01	0,03
3,25	9579	0,01	0	0,03	0,06	0,56	0,03	0	0,02	0,29	0,02	0,2	0,77	0,01	0,04
3,25	26412	0,06	0	0	0	0	0,93	0,01	0	0	0,06	0,26	0,68	0	0,048
3,25	34121	0	0,01	0	0,22	0,76	0	0	0,01	0	0,76	0,23	0,01	0	0,048
3,25	9155	0	0	0,04	0	0	0	0	0,96	0	0	0,04	0,03	0,93	0,004
3,25	32499	0,02	0	0,04	0	0,94	0	0	0	0	0,94	0,04	0,02	0	0,048
3,25	14673	0	0,75	0,04	0	0,19	0	0	0,02	0	0,02	0,36	0	0,62	0,048
3,25	91745	0	0	0,03	0,05	0,03	0	0,08	0,81	0	0,76	0,11	0,08	0,05	0,044
3,25	15494	0	0	0	0,17	0,77	0	0	0,06	0	0,8	0	0,03	0,17	0,042
3,25	30954	0	0,01	0,01	0,09	0	0,02	0,01	0,84	0,02	0,08	0,01	0,88	0,03	0,048
3,25	37453	0,01	0,3	0,04	0	0,57	0	0	0,08	0	0	0,62	0,08	0,3	0,044
3,25	6160	0	0,07	0	0	0,85	0,02	0	0,05	0,01	0,96	0,02	0,01	0,01	0,044
3,25	35588	0,03	0,02	0,04	0	0,79	0,02	0	0,08	0,02	0,01	0,06	0,84	0,09	0,048
3,25	10035	0	0,02	0	0,07	0,68	0	0	0,23	0	0,25	0	0	0,75	0,048
3,25	19691	0,05	0	0,01	0	0,93	0,01	0	0	0	0,01	0,01	0,98	0	0,046
3,25	100000	0	0	0,81	0	0	0	0,19	0	0	0	0,81	0	0,19	0,428
3,25	24401	0	0,02	0,04	0	0	0,67	0	0,26	0,01	0,01	0,03	0,69	0,27	0,026

3,25	100000	0	0	0,93	0	0,07	0	0	0	0	0	0,91	0,07	0,02	0,456
3,25	84711	0	0,1	0,04	0,03	0	0,08	0	0,75	0	0,03	0,04	0,15	0,78	0,048
3,25	76843	0	0	0,04	0	0	0,15	0	0	0,81	0	0,04	0,96	0	0,024
3,25	6182	0	0,01	0,04	0	0	0	0	0,04	0,91	0,04	0,09	0	0,87	0,012
3,25	70771	0,05	0	0	0	0,69	0	0	0,26	0	0,05	0,95	0	0	0,036
3,25	37894	0,05	0	0	0,01	0,93	0	0	0,01	0	0,05	0	0,94	0,01	0,048
3,25	63723	0	0,27	0	0	0,02	0,71	0	0	0	0,05	0	0,2	0,75	0,048
3,25	16265	0	0,07	0,02	0,04	0,13	0,05	0	0,06	0,63	0,23	0,41	0,32	0,04	0,048
3,25	10627	0	0,03	0	0,17	0,8	0	0	0	0	0,8	0	0,2	0	0,048
3,25	13786	0	0,89	0,04	0	0,04	0	0	0,02	0,01	0	0,08	0,08	0,84	0,044
3,25	38288	0	0,2	0	0,02	0,78	0	0	0	0	0,02	0,78	0,2	0	0,04
3,25	8402	0	0,01	0	0,08	0,91	0	0	0	0	0,08	0	0,47	0,45	0,046
3,25	5701	0,06	0,03	0	0	0,89	0	0	0,01	0,01	0,03	0,04	0,9	0,03	0,048
3,25	30488	0,01	0	0,04	0	0,83	0	0,03	0,09	0	0,1	0,04	0,74	0,12	0,042
3,25	99679	0	0	0	0,13	0,87	0	0	0	0	0	0,12	0,88	0	0,046
3,25	11573	0,05	0	0	0	0	0,76	0	0,18	0,01	0,05	0,01	0	0,94	0,04
3,25	5913	0,02	0,01	0,04	0,03	0,66	0,2	0	0,04	0	0	0,7	0,22	0,08	0,048
3,25	45543	0	0	0,01	0	0	0,04	0,21	0,74	0	0,74	0,02	0,2	0,04	0,04
3,25	82020	0	0,28	0	0	0,69	0	0	0,03	0	0,03	0	0,28	0,69	0,038
3,25	45588	0	0	0	0,05	0,92	0	0,01	0,02	0	0	0	0,02	0,98	0,048
3,25	41059	0,06	0	0	0,01	0,9	0,03	0	0	0	0,09	0,01	0,81	0,09	0,048
3,25	35809	0,04	0	0,01	0	0,91	0	0	0,02	0,02	0,93	0,03	0,04	0	0,048
3,25	47978	0	0,05	0,02	0	0,92	0	0,01	0	0	0,92	0,06	0,02	0	0,048
3,25	100000	0	0	1	0	0	0	0	0	0	0	0,81	0	0,19	0,382
3,25	13681	0	0	0,01	0	0,92	0	0,07	0	0	0	0,94	0,06	0	0,048
3,25	10465	0,04	0	0,02	0	0	0,93	0,01	0	0	0,97	0,03	0	0	0,042
3,25	76089	0	0	0	0,01	0,31	0	0,15	0	0,53	0,84	0	0	0,16	0,042
3,25	100000	0	0	0,95	0	0	0	0	0	0,05	0,05	0,56	0	0,39	0,308
3,25	13557	0	0,16	0	0	0,33	0,03	0	0,48	0	0	0,33	0,63	0,04	0,046
3,25	2859	0,03	0	0,04	0	0,24	0	0	0,66	0,03	0,24	0,07	0,03	0,66	0,04
3,25	78407	0,04	0	0	0,02	0,93	0,01	0	0	0	0	0,05	0,02	0,93	0,046
3,25	3091	0	0	0,04	0	0,95	0	0,01	0	0	0,96	0,03	0	0,01	0,036
3,25	70403	0,06	0	0,01	0	0,01	0,53	0	0,27	0,12	0,13	0,81	0,06	0	0,046

3,25	9006	0,04	0	0,04	0	0,87	0,02	0,02	0	0,01	0,04	0,93	0,03	0	0,044
3,25	10256	0	0,27	0,02	0	0,67	0,04	0	0	0	0,02	0	0,31	0,67	0,032
3,25	25119	0	0	0,04	0	0,45	0,48	0,03	0	0	0,93	0,03	0,04	0	0,048
3,25	39792	0,05	0	0,02	0	0,84	0,09	0	0	0	0,05	0,86	0,09	0	0,044
3,25	43558	0,01	0,21	0	0,02	0,72	0,02	0,01	0,01	0	0,01	0,21	0,03	0,75	0,044
3,25	23391	0	0,1	0	0	0	0,29	0	0,6	0,01	0,46	0,1	0,44	0	0,046
3,25	46631	0,02	0	0,03	0	0,86	0,04	0,05	0	0	0,86	0,09	0,05	0	0,044
3,25	11464	0	0,2	0,01	0	0,79	0	0	0	0	0	0,8	0,2	0	0,04
3,25	4534	0	0	0,04	0	0,03	0	0	0,63	0,3	0,63	0,04	0,03	0,3	0,022
3,25	50058	0,03	0	0,03	0,01	0,42	0,5	0,01	0	0	0,54	0,45	0,01	0	0,048
3,25	49154	0,02	0,94	0	0,01	0,03	0	0	0	0	0,02	0,01	0	0,97	0,042
3,25	41725	0	0	0,04	0	0,96	0	0	0	0	0,96	0,04	0	0	0,042
3,25	18309	0,05	0	0,03	0	0,9	0	0	0,02	0	0,07	0,93	0	0	0,046
3,5	14339	0,06	0	0	0	0,84	0,09	0	0	0,01	0,05	0,02	0,09	0,84	0,046
3,5	13726	0,06	0	0	0	0	0,94	0	0	0	0,06	0	0	0,94	0,046
3,5	44665	0	0,03	0	0,19	0,76	0	0	0,01	0,01	0,76	0,02	0,22	0	0,048
3,5	27442	0	0,04	0	0	0,3	0,14	0,09	0	0,43	0,3	0,26	0,34	0,1	0,044
3,5	24464	0	0,14	0	0	0,05	0	0	0,81	0	0,05	0	0,95	0	0,044
3,5	98700	0	0	0,04	0	0	0,09	0,01	0	0,86	0	0,04	0,01	0,95	0,01
3,5	18408	0,02	0,29	0	0	0	0,64	0,01	0,04	0	0	0	0,05	0,95	0,048
3,5	90497	0	0	0,04	0	0,45	0,36	0,01	0	0,14	0,45	0	0,37	0,18	0,032
3,5	9770	0	0,17	0	0,03	0,71	0	0	0	0,09	0,29	0	0	0,71	0,044
3,5	26546	0,01	0	0	0,16	0,01	0,81	0	0,01	0	0,44	0,4	0,16	0	0,048
3,5	34047	0,05	0	0	0	0,92	0,03	0	0	0	0,12	0	0,88	0	0,036
3,5	100000	0,08	0,02	0,89	0,01	0	0	0	0	0	0	0,9	0	0,1	0,506
3,5	64651	0,02	0,01	0,04	0	0,67	0	0	0,26	0	0,27	0,05	0	0,68	0,032
3,5	12639	0,04	0,05	0	0	0,9	0	0	0	0,01	0,1	0	0	0,9	0,046
3,5	19454	0	0	0,01	0,18	0,73	0	0	0,08	0	0,87	0	0	0,13	0,04
3,5	33082	0,05	0	0,03	0	0,89	0,02	0	0,01	0	0,89	0,03	0,03	0,05	0,044
3,5	41451	0	0,37	0	0	0,63	0	0	0	0	0	0,91	0,09	0	0,046
3,5	28844	0	0	0	0,1	0,9	0	0	0	0	0	0	0,9	0,1	0,044
3,5	13008	0,05	0	0	0	0	0,95	0	0	0	0,99	0,01	0	0	0,038
3,5	26806	0,01	0	0	0	0	0	0,1	0,89	0	0	0,01	0,99	0	0,046

3,5	87351	0,06	0,02	0	0	0	0,02	0	0,9	0	0,23	0,06	0,71	0	0,046
3,5	100000	0,01	0	0,99	0	0	0	0	0	0	0	0,99	0	0,01	0,594
3,5	89141	0	0,04	0,04	0,02	0	0	0	0,86	0,04	0,86	0,06	0,04	0,04	0,048
3,5	9166	0	0,01	0,04	0	0,35	0	0	0,6	0	0	0,44	0,55	0,01	0,02
3,5	100000	0	0	0,99	0	0,01	0	0	0	0	0	1	0	0	0,634
3,5	100000	0,01	0	0,38	0	0	0	0	0	0,61	0	0,37	0,62	0,01	0,174
3,5	33817	0	0,07	0	0	0,89	0,01	0,01	0	0,02	0,89	0	0,09	0,02	0,042
3,5	100000	0	0	0,97	0,01	0	0,02	0	0	0	0,02	0,97	0	0,01	0,608
3,5	38845	0	0,13	0,04	0	0,71	0,12	0	0	0	0,08	0,79	0,04	0,09	0,026
3,5	23133	0,06	0	0	0	0,01	0,55	0	0,38	0	0,44	0	0,02	0,54	0,036
3,5	45285	0,05	0	0,01	0	0	0,93	0	0,01	0	0	0,01	0,06	0,93	0,046
3,5	42960	0,01	0,01	0,02	0,04	0,81	0	0	0,11	0	0	0,06	0,01	0,93	0,044
3,5	19329	0	0	0,04	0	0,94	0	0	0,02	0	0,94	0,04	0	0,02	0,034
3,5	8154	0	0	0,04	0,01	0	0,91	0	0,04	0	0,04	0,01	0,95	0	0,038
3,5	10150	0,05	0	0,04	0	0,83	0	0,01	0,04	0,03	0,04	0,05	0,06	0,85	0,04
3,5	24676	0,04	0	0	0,03	0,93	0	0	0	0	0,04	0	0,83	0,13	0,046
3,5	31262	0,03	0	0	0,02	0,91	0,03	0	0,01	0	0,05	0,92	0	0,03	0,046
3,5	16031	0	0,01	0,04	0	0	0,44	0	0,43	0,08	0	0,5	0,43	0,07	0,03
3,5	15691	0	0,19	0	0	0,05	0	0	0,45	0,31	0,09	0,19	0,31	0,41	0,044
3,5	21836	0	0,23	0	0	0,77	0	0	0	0	0	0,77	0,22	0,01	0,048
3,5	53761	0	0	0,04	0	0,81	0	0	0,15	0	0,15	0,04	0	0,81	0,01
3,5	70394	0	0	0,04	0,02	0,9	0	0	0,04	0	0,01	0,92	0,06	0,01	0,024
3,5	4949	0,05	0	0	0,02	0,01	0,92	0	0	0	0,92	0	0,07	0,01	0,044
3,5	63559	0	0	0	0,34	0,66	0	0	0	0	1	0	0	0	0,036
3,5	9862	0	0	0,04	0	0	0	0	0,96	0	0	0,02	0	0,98	0
3,5	9556	0,02	0	0,04	0	0	0	0	0,94	0	0,96	0,01	0,03	0	0,048
3,5	74442	0,01	0,02	0	0,08	0,88	0,01	0	0	0	0,1	0	0,9	0	0,046
3,5	100000	0,21	0	0,79	0	0	0	0	0	0	0,21	0,73	0	0,06	0,742
3,5	11646	0	0	0,04	0	0	0,01	0	0	0,95	0	0,04	0	0,96	0,01
3,5	39571	0	0,08	0	0,03	0,84	0	0,03	0,01	0,01	0,09	0,88	0,03	0	0,046
3,5	54288	0,04	0	0,03	0	0	0,3	0,01	0,62	0	0,31	0,07	0	0,62	0,046
3,5	20422	0	0,29	0	0	0,7	0	0,01	0	0	0,01	0	0,29	0,7	0,048
3,5	10104	0	0	0	0,16	0,76	0,08	0	0	0	0,14	0,02	0,84	0	0,044

3,5	56638	0	0	0,04	0	0,38	0,01	0	0,16	0,41	0	0,58	0,01	0,41	0,006
3,5	29212	0	0,58	0,02	0	0	0	0	0,31	0,09	0,02	0,58	0	0,4	0,046
3,5	18781	0	0,03	0,04	0	0,88	0,04	0	0,01	0	0,91	0,04	0,05	0	0,046
3,5	63264	0,01	0	0,04	0	0	0,66	0	0	0,29	0,67	0,03	0,04	0,26	0,044
3,5	8987	0	0,78	0	0	0,05	0	0	0,13	0,04	0,05	0,04	0,03	0,88	0,046
3,5	100000	0	0	0,98	0	0	0	0	0,02	0	0,02	0,92	0,05	0,01	0,492
3,5	6816	0	0,07	0	0,01	0,89	0	0,03	0	0	0,16	0,07	0,77	0	0,042
3,5	57938	0	0,01	0	0,25	0,74	0	0	0	0	1	0	0	0	0,048
3,5	75083	0,04	0	0,04	0	0,92	0	0	0	0	0,02	0	0,98	0	0,042
3,5	13185	0	0	0,04	0	0,09	0,15	0	0,52	0,2	0,52	0,18	0,01	0,29	0,034
3,5	9993	0	0	0	0,08	0,91	0,01	0	0	0	0,08	0,91	0	0,01	0,048
3,5	100000	0,16	0	0,83	0	0	0,01	0	0	0	0,03	0,83	0,01	0,13	0,55
3,5	19309	0	0,12	0,04	0	0,84	0	0	0	0	0,1	0,44	0,46	0	0,046
3,5	41180	0	0	0,04	0	0	0	0	0,92	0,04	0,04	0,05	0,47	0,44	0,01
3,5	11700	0	0,1	0,04	0	0,27	0	0	0,04	0,55	0	0,33	0,65	0,02	0,038
3,5	20772	0,01	0,09	0	0	0	0	0	0,87	0,03	0,87	0	0,1	0,03	0,044
3,5	20775	0	0,24	0	0	0,02	0,04	0	0,7	0	0	0,72	0,14	0,14	0,042
3,5	53688	0	0,07	0	0,05	0,84	0,04	0	0	0	0,04	0,77	0,19	0	0,034
3,5	45950	0	0,08	0	0	0,92	0	0	0	0	0,92	0	0,08	0	0,046
3,5	13214	0	0,2	0	0,02	0,76	0	0,01	0,01	0	0,02	0,96	0,01	0,01	0,04
3,5	30881	0	0,11	0,04	0	0	0	0	0,83	0,02	0,08	0,92	0	0	0,046
3,5	26129	0,02	0	0,04	0,01	0	0,15	0	0	0,78	0	0,04	0,03	0,93	0,042
3,5	7490	0	0	0	0,23	0,73	0,02	0	0,02	0	0,82	0,02	0,01	0,15	0,046
3,5	6329	0	0,23	0	0	0,7	0,07	0	0	0	0	0,7	0,23	0,07	0,038
3,5	46616	0	0,31	0,04	0	0	0	0	0,02	0,63	0	0,03	0	0,97	0,036
3,5	9583	0	0,21	0	0	0,72	0,05	0	0,02	0	0,01	0,72	0,23	0,04	0,044
3,5	24099	0,02	0,02	0,01	0,05	0,88	0	0	0	0,02	0,07	0,04	0,87	0,02	0,044
3,5	15139	0	0	0,04	0	0	0	0	0,96	0	0	0	0	1	0,002
3,5	13402	0	0	0	0,07	0,93	0	0	0	0	0,07	0,93	0	0	0,048
3,5	30362	0,01	0	0,04	0	0	0,01	0	0,94	0	0,25	0,04	0,69	0,02	0,028
3,5	52141	0,03	0	0,01	0,03	0,86	0,07	0	0	0	0,01	0,07	0,07	0,85	0,042
3,5	19901	0	0,17	0	0	0,56	0,26	0,01	0	0	0,22	0,65	0	0,13	0,048
3,5	21952	0,02	0,04	0,01	0	0	0,91	0,02	0	0	0,08	0	0,9	0,02	0,048

3,5	6926	0,03	0,01	0	0	0,94	0,01	0	0,01	0	0,98	0,01	0	0,01	0,04
3,5	52532	0	0,02	0	0,06	0,9	0	0,02	0	0	0,07	0,21	0,28	0,44	0,04
3,5	67493	0	0	0,04	0	0,48	0,48	0	0	0	0,52	0	0	0,48	0,026
3,5	45479	0,05	0	0,01	0	0,03	0,87	0	0,04	0	0,08	0	0,88	0,04	0,048
3,5	100000	0	0	0,99	0	0	0,01	0	0	0	0	0,99	0,01	0	0,534
3,5	26107	0,04	0,04	0	0	0,92	0	0	0	0	0,08	0,92	0	0	0,044
3,5	23619	0	0,24	0,04	0	0,01	0,45	0	0	0,26	0,05	0,23	0	0,72	0,038
3,5	4402	0	0	0,04	0,01	0,94	0,01	0	0	0	0,95	0,01	0,04	0	0,036
3,5	18871	0	0	0,04	0	0,93	0	0	0	0,03	0,93	0,04	0	0,03	0,038
3,5	38201	0,03	0	0,04	0	0,93	0	0	0	0	0,73	0,04	0	0,23	0,04
3,5	39990	0	0,32	0	0	0,68	0	0	0	0	0	0	0,32	0,68	0,044
3,5	45507	0,04	0,01	0	0	0,53	0,4	0,01	0,01	0	0,92	0	0,07	0,01	0,048
3,5	76393	0,05	0	0	0	0,01	0	0	0,94	0	0	0,95	0,05	0	0,048
3,5	80931	0	0,19	0	0,05	0,76	0	0	0	0	0,07	0,17	0	0,76	0,046
3,75	84566	0	0	0	0,12	0	0	0	0,88	0	0	0,55	0,41	0,04	0,046
3,75	8880	0	0,02	0,03	0	0,89	0	0,05	0	0,01	0	0,89	0,11	0	0,048
3,75	11756	0	0	0,04	0	0	0,11	0	0,7	0,15	0	0,13	0,55	0,32	0,016
3,75	37247	0,01	0	0,04	0	0,91	0	0	0,02	0,02	0,93	0,05	0	0,02	0,042
3,75	45837	0,06	0	0	0,01	0,91	0	0	0	0,02	0,93	0,01	0	0,06	0,048
3,75	19209	0	0,01	0,04	0	0	0,68	0	0,01	0,26	0,01	0,01	0,26	0,72	0,02
3,75	12705	0	0,01	0,04	0	0,95	0	0	0	0	0	0,01	0,96	0,03	0,028
3,75	100000	0,14	0	0,78	0,04	0,03	0	0	0	0,01	0,44	0,39	0,15	0,02	0,696
3,75	16127	0,02	0	0	0,04	0,94	0	0	0	0	0,02	0,94	0,02	0,02	0,044
3,75	32427	0	0	0,04	0	0,7	0	0,01	0,24	0,01	0,01	0,28	0,71	0	0,036
3,75	78248	0,01	0	0,04	0,01	0,89	0	0,01	0,02	0,02	0,02	0,95	0,03	0	0,048
3,75	26136	0,01	0	0,04	0	0,93	0	0	0,02	0	0,05	0,01	0,81	0,13	0,042
3,75	17850	0,01	0	0,04	0	0	0,38	0	0,56	0,01	0,18	0,01	0,81	0	0,038
3,75	43335	0	0	0,04	0,01	0	0,74	0,04	0	0,17	0,01	0,24	0,75	0	0,042
3,75	8834	0	0	0,04	0	0,47	0,02	0	0,01	0,46	0,43	0,08	0,02	0,47	0,018
3,75	62397	0	0	0,04	0	0	0,88	0	0,02	0,06	0,02	0,33	0,61	0,04	0,026
3,75	10794	0	0,03	0	0,12	0,56	0,26	0,01	0,01	0,01	0,79	0,01	0,06	0,14	0,04
3,75	11123	0,05	0	0	0,04	0	0,14	0	0,77	0	0,77	0	0,08	0,15	0,044
3,75	44324	0,03	0	0,04	0	0	0	0	0,93	0	0	0,04	0,93	0,03	0,044

3,75	34910	0	0	0,04	0,1	0,04	0,81	0	0	0,01	0,02	0,18	0,8	0	0,046
3,75	27240	0	0	0,04	0	0,05	0,91	0	0	0	0	0,09	0,35	0,56	0,022
3,75	35881	0,01	0,1	0	0,04	0,81	0	0,01	0,03	0	0,09	0,81	0,03	0,07	0,042
3,75	23014	0,03	0	0,04	0	0,3	0,03	0	0,52	0,08	0,01	0,59	0,32	0,08	0,048
3,75	2986	0	0	0,04	0	0,93	0	0	0	0,03	0,93	0,04	0,03	0	0,036
3,75	100000	0,03	0	0,97	0	0	0	0	0	0	0,03	0,97	0	0	0,646
3,75	45966	0,01	0	0,04	0	0	0	0,04	0,91	0	0,07	0	0	0,93	0,048
3,75	38745	0	0	0,04	0	0	0,95	0	0	0,01	0	0,04	0	0,96	0,01
3,75	23916	0	0	0	0	0,95	0	0,05	0	0	0	0,05	0	0,95	0,04
3,75	35150	0	0,03	0	0,06	0	0	0	0,91	0	0	0,06	0,03	0,91	0,046
3,75	20485	0	0,19	0,02	0	0,7	0	0,01	0	0,08	0	0,73	0,17	0,1	0,046
3,75	60824	0	0	0,03	0,06	0,91	0	0	0	0	0,04	0,82	0	0,14	0,044
3,75	22574	0,04	0	0	0,06	0,9	0	0	0	0	0,9	0	0,04	0,06	0,044
3,75	8389	0,01	0	0,04	0	0	0	0,01	0,94	0	0	0,03	0,01	0,96	0,022
3,75	100000	0	0	0,98	0,02	0	0	0	0	0	0	1	0	0	0,736
3,75	61043	0	0	0,01	0,03	0,5	0	0,06	0	0,4	0	0,51	0,27	0,22	0,048
3,75	45060	0	0,26	0,01	0	0,7	0,02	0	0	0,01	0,25	0	0,05	0,7	0,042
3,75	47364	0	0,01	0,04	0	0	0	0	0,37	0,58	0,03	0,01	0,32	0,64	0,024
3,75	17280	0	0	0	0,29	0,71	0	0	0	0	1	0	0	0	0,048
3,75	19696	0,01	0,02	0	0	0,66	0,01	0,09	0,21	0	0,03	0,09	0,87	0,01	0,048
3,75	53923	0	0,12	0,01	0,02	0,8	0	0	0	0,05	0,03	0,06	0,68	0,23	0,046
3,75	8979	0	0,03	0,02	0	0,92	0	0,03	0	0	0,92	0,01	0,03	0,04	0,03
3,75	44102	0,02	0	0,04	0	0	0	0	0,94	0	0,02	0	0,98	0	0,048
3,75	22515	0,04	0,04	0	0	0,26	0	0,01	0,63	0,02	0,55	0,12	0,12	0,21	0,044
3,75	53577	0	0,68	0,04	0,01	0,26	0,01	0	0	0	0,05	0,09	0,01	0,85	0,036
3,75	13867	0	0,97	0	0,03	0	0	0	0	0	0,01	0	0,02	0,97	0,046
3,75	34903	0	0,26	0	0	0,72	0,01	0	0	0,01	0,02	0	0,35	0,63	0,038
3,75	100000	0,02	0	0,63	0	0	0,01	0,29	0,05	0	0,05	0,63	0,31	0,01	0,454
3,75	17797	0	0,42	0,04	0	0,45	0	0	0	0,09	0	0,42	0,09	0,49	0,046
3,75	23872	0	0,82	0	0	0,12	0,02	0	0	0,04	0	0,12	0,06	0,82	0,048
3,75	24751	0	0,08	0,04	0	0,88	0	0	0	0	0,08	0,04	0,88	0	0,042
3,75	26210	0	0	0,01	0,13	0,84	0	0,01	0,01	0	0,62	0,01	0,01	0,36	0,048
3,75	27925	0,06	0	0,02	0,01	0,88	0,03	0	0	0	0,9	0,02	0,05	0,03	0,046

3,75	26512	0,05	0	0	0	0,95	0	0	0	0	1	0	0	0	0,048
3,75	17854	0,01	0	0	0,08	0,9	0	0,01	0	0	0,01	0	0,91	0,08	0,048
3,75	23554	0	0	0,03	0,03	0	0,02	0,02	0,9	0	0	0,93	0,03	0,04	0,048
3,75	24047	0,04	0,01	0	0	0,95	0	0	0	0	0,99	0,01	0	0	0,046
3,75	100000	0	0	1	0	0	0	0	0	0	0	1	0	0	0,608
3,75	58964	0	0	0,04	0,03	0,6	0,07	0	0,26	0	0,07	0,63	0,04	0,26	0,034
3,75	10174	0	0	0,04	0	0,96	0	0	0	0	0,63	0,04	0,33	0	0,034
3,75	15813	0,01	0	0	0,01	0	0,69	0,07	0,22	0	0	0	0,91	0,09	0,044
3,75	9198	0,02	0	0	0,18	0	0,79	0,01	0	0	0,87	0,12	0	0,01	0,048
3,75	89652	0,03	0	0,04	0	0,33	0,1	0	0,45	0,05	0,03	0,1	0,37	0,5	0,046
3,75	61663	0	0,01	0,04	0	0,86	0	0,08	0,01	0	0,87	0,04	0,08	0,01	0,048
3,75	100000	0,03	0	0,97	0	0	0	0	0	0	0	1	0	0	0,702
3,75	66008	0	0,07	0,04	0,01	0	0,88	0	0	0	0,15	0	0,85	0	0,048
3,75	22789	0	0,39	0	0	0	0	0	0	0,61	0	0,39	0	0,61	0,046
3,75	14461	0,03	0	0	0	0,04	0,19	0,05	0,67	0,02	0,67	0,01	0,04	0,28	0,048
3,75	10638	0	0	0,04	0	0,76	0,19	0	0,01	0	0,02	0,04	0,76	0,18	0,016
3,75	33804	0	0	0,04	0	0	0	0,05	0,91	0	0	0	0,96	0,04	0,042
3,75	71607	0	0,14	0	0	0	0,01	0	0,85	0	0,01	0	0,85	0,14	0,044
3,75	38455	0,06	0	0	0	0,91	0	0	0	0,03	0,33	0	0,61	0,06	0,046
3,75	79262	0,01	0,11	0	0	0,25	0,63	0	0	0	0,14	0	0,86	0	0,048
3,75	74293	0,02	0	0,04	0	0,87	0,07	0	0	0	0	0,9	0,07	0,03	0,034
3,75	16064	0	0	0,04	0	0,69	0,04	0	0,23	0	0,18	0,09	0,04	0,69	0,008
3,75	37853	0,06	0	0,02	0	0,92	0	0	0	0	0,06	0,02	0,89	0,03	0,046
3,75	46545	0	0,09	0	0,03	0,87	0	0	0	0,01	0,61	0	0,39	0	0,038
3,75	24431	0	0,08	0,01	0,05	0	0	0	0,86	0	0	0	0,92	0,08	0,048
3,75	12761	0	0	0,01	0	0,84	0,04	0,11	0	0	0	0,01	0,95	0,04	0,048
3,75	95131	0	0,02	0	0,02	0,01	0	0,08	0,87	0	0,03	0,01	0,87	0,09	0,038
3,75	100000	0	0	0,73	0,07	0,05	0,15	0	0	0	0,05	0,73	0,22	0	0,396
3,75	30981	0	0,02	0,02	0	0,27	0,01	0,11	0,57	0	0,29	0,02	0,58	0,11	0,048
3,75	68343	0	0,02	0	0,06	0,85	0,07	0	0	0	0,01	0,02	0,05	0,92	0,04
3,75	65678	0	0	0	0,09	0,91	0	0	0	0	0	0	1	0	0,036
3,75	27334	0	0	0	0,06	0,91	0	0	0,02	0,01	0,07	0,02	0,02	0,89	0,038
3,75	17259	0	0,05	0	0,09	0	0,1	0	0,76	0	0,05	0	0,86	0,09	0,048

3,75	38670	0	0,06	0	0	0	0,93	0,01	0	0	1	0	0	0	0,048
3,75	29319	0	0,23	0,01	0,01	0,71	0	0	0	0,04	0,23	0,01	0,03	0,73	0,036
3,75	50948	0	0	0,04	0	0,51	0,04	0	0,41	0	0,04	0	0,51	0,45	0,004
3,75	44753	0	0,01	0	0,13	0,86	0	0	0	0	0	0,13	0,87	0	0,046
3,75	4450	0,06	0	0	0	0,25	0,37	0	0,03	0,29	0,31	0	0,26	0,43	0,048
3,75	24019	0	0,03	0	0,06	0,9	0	0	0,01	0	0	0,93	0,01	0,06	0,048
3,75	82496	0	0	0	0,11	0,58	0,04	0	0,27	0	0,05	0,09	0,86	0	0,048
3,75	53159	0	0,26	0	0	0	0	0	0,74	0	0,26	0	0	0,74	0,048
3,75	46079	0,01	0,01	0,04	0	0,94	0	0	0	0	0,94	0,01	0,03	0,02	0,042
3,75	100000	0,02	0,45	0,48	0,02	0	0,03	0	0	0	0,5	0,5	0	0	0,888
3,75	35175	0,05	0	0	0	0,88	0	0	0	0,07	0,03	0	0,97	0	0,048
3,75	100000	0,21	0	0,64	0	0,08	0	0,01	0,06	0	0	0,66	0,07	0,27	0,41
3,75	41388	0	0	0,04	0	0	0,01	0	0,82	0,13	0	0,04	0,01	0,95	0,004
3,75	40323	0	0	0	0,08	0,23	0,12	0	0,01	0,56	0	0,56	0,14	0,3	0,038
3,75	39929	0,05	0	0	0,03	0,89	0,03	0	0	0	0,87	0,05	0,07	0,01	0,046
4	14865	0,01	0,21	0,04	0	0,13	0,01	0	0,04	0,56	0,08	0,04	0,08	0,8	0,046
4	15034	0	0,12	0	0	0,88	0	0	0	0	0	0,06	0,93	0,01	0,046
4	100000	0	0,01	0,98	0	0	0	0,01	0	0	0,01	0,97	0,01	0,01	0,498
4	32035	0	0,08	0	0	0,84	0	0	0,08	0	0,92	0	0,08	0	0,038
4	3291	0	0,06	0,04	0,04	0,75	0,11	0	0	0	0,08	0,89	0,03	0	0,044
4	7995	0	0	0,04	0	0	0,72	0,05	0	0,19	0,19	0,1	0	0,71	0,042
4	14206	0	0	0,04	0	0,94	0	0,02	0	0	0,06	0	0	0,94	0,02
4	6805	0	0	0,04	0,02	0	0,91	0	0	0,03	0,03	0,04	0,92	0,01	0,02
4	38039	0	0,01	0	0,06	0,9	0,02	0	0,01	0	0,03	0	0,06	0,91	0,048
4	42347	0,02	0,25	0	0	0,3	0	0	0	0,43	0,03	0,25	0,02	0,7	0,038
4	24143	0	0	0,04	0	0,96	0	0	0	0	0	0,04	0,96	0	0,024
4	29345	0,04	0,01	0,02	0	0,76	0,15	0	0	0,02	0,91	0,05	0,04	0	0,048
4	78697	0,06	0	0,01	0	0,93	0	0	0	0	0	0,02	0	0,98	0,042
4	26796	0,04	0	0	0,03	0,93	0	0	0	0	0,04	0	0,93	0,03	0,046
4	34326	0,03	0	0	0,02	0	0,01	0	0,94	0	0,01	0,06	0	0,93	0,046
4	84930	0,01	0,02	0,04	0	0,91	0	0	0	0,02	0	0,08	0	0,92	0,01
4	23180	0	0,03	0,04	0	0,51	0,26	0	0,1	0,06	0	0,55	0,36	0,09	0,016
4	18504	0,03	0	0	0,04	0,93	0	0	0	0	0	0,02	0,94	0,04	0,04

4	57822	0	0,14	0	0	0	0	0	0,86	0	0	0,05	0,95	0	0,048
4	34257	0,04	0	0,02	0	0,89	0	0	0,05	0	0,93	0,02	0,04	0,01	0,048
4	100000	0	0	0	0,3	0,68	0,02	0	0	0	0,13	0	0,27	0,6	0,244
4	64024	0,03	0	0,04	0	0,92	0	0	0,01	0	0,03	0	0,92	0,05	0,032
4	54736	0	0,12	0,04	0	0	0,26	0	0,58	0	0	0,01	0,7	0,29	0,042
4	96899	0,02	0	0,01	0,05	0,88	0	0	0,02	0,02	0,83	0,01	0,09	0,07	0,04
4	10773	0,04	0,94	0	0,02	0	0	0	0	0	0	0	0	1	0,046
4	8434	0	0,95	0,04	0	0	0	0	0,01	0	0,05	0	0	0,95	0,046
4	51477	0,03	0	0,03	0,05	0,77	0,11	0	0	0,01	0,82	0,15	0,03	0	0,048
4	79347	0	0	0	0,13	0,87	0	0	0	0	0,07	0,06	0,87	0	0,048
4	65544	0	0,04	0,04	0,02	0,9	0	0	0	0	0,96	0	0,04	0	0,048
4	30934	0	0	0,04	0	0,7	0,25	0	0,01	0	0,74	0,21	0,05	0	0,046
4	20397	0	0	0	0	0,01	0	0,06	0,93	0	0	0,97	0,01	0,02	0,048
4	87190	0	0,09	0,02	0	0,74	0,07	0,03	0	0,05	0,15	0,74	0,11	0	0,04
4	27089	0	0	0,04	0	0	0,94	0,02	0	0	0,21	0,74	0,04	0,01	0,032
4	22955	0	0,01	0,04	0	0,95	0	0	0	0	0,03	0	0,96	0,01	0,024
4	22710	0,06	0	0	0	0,93	0,01	0	0	0	0,05	0,75	0,2	0	0,048
4	9448	0	0	0,04	0	0	0	0	0	0,96	0	0,04	0	0,96	0,006
4	35308	0	0,13	0,02	0	0,54	0,02	0	0	0,29	0,29	0,15	0,02	0,54	0,04
4	88865	0	0	0	0,07	0,86	0	0	0,06	0,01	0,2	0,64	0	0,16	0,044
4	20820	0	0	0	0,06	0,9	0	0	0,04	0	0	0	0,06	0,94	0,048
4	97678	0	0,02	0,02	0,07	0,89	0	0	0	0	0,09	0,91	0	0	0,048
4	57276	0,04	0,06	0,02	0	0,8	0	0	0,08	0	0	0,82	0,1	0,08	0,048
4	9758	0	0	0,02	0	0	0,65	0,13	0	0,2	0,16	0,02	0,82	0	0,044
4	39342	0	0	0,01	0,03	0,02	0	0,03	0,91	0	0	0,05	0	0,95	0,048
4	4745	0,01	0,01	0,04	0	0,88	0	0	0	0,06	0,7	0,05	0,25	0	0,042
4	23532	0,02	0,05	0	0	0	0,92	0	0	0,01	0,24	0	0,76	0	0,034
4	66642	0,07	0	0	0	0,93	0	0	0	0	0,06	0	0	0,94	0,048
4	26806	0	0,01	0	0,07	0,92	0	0	0	0	0	0,93	0,01	0,06	0,046
4	34697	0	0,21	0	0	0	0	0	0	0,79	0,08	0	0,13	0,79	0,046
4	38866	0	0	0	0,05	0	0,22	0,05	0,68	0	0,05	0,67	0,28	0	0,048
4	17591	0,02	0	0,01	0	0,51	0	0,04	0,42	0	0	0,01	0,06	0,93	0,048
4	100000	0	0	1	0	0	0	0	0	0	0	0,95	0,05	0	0,534

4	20542	0	0,1	0,01	0	0,64	0,24	0	0	0,01	0,1	0,02	0,88	0	0,04
4	78188	0	0	0	0	0,85	0	0,1	0	0,05	0,15	0,85	0	0	0,046
4	20463	0	0,03	0,04	0	0,9	0	0,03	0	0	0	0,02	0,08	0,9	0,034
4	79860	0	0	0,04	0	0,01	0,02	0	0,93	0	0	0,02	0,95	0,03	0,024
4	71932	0	0	0,02	0,05	0	0	0	0	0,93	0	0,02	0,01	0,97	0,042
4	74490	0	0	0,04	0	0,77	0	0	0,19	0	0,12	0,1	0,77	0,01	0,028
4	50383	0	0	0,04	0,04	0,24	0	0,01	0,66	0,01	0,01	0,02	0,3	0,67	0,038
4	100000	0	0	1	0	0	0	0	0	0	0,65	0,35	0	0	0,984
4	46644	0	0	0,04	0,01	0,93	0	0	0,02	0	0,01	0,04	0,02	0,93	0,006
4	64751	0	0,05	0	0,06	0,03	0,86	0	0	0	0	0	1	0	0,046
4	100000	0	0	0,98	0,01	0,01	0	0	0	0	0,01	0,99	0	0	0,802
4	28737	0,06	0	0	0	0	0,89	0,02	0	0,03	0,91	0,04	0	0,05	0,048
4	8245	0,01	0	0,04	0	0	0	0,01	0,86	0,08	0,87	0,04	0,01	0,08	0,044
4	20533	0	0,04	0,04	0	0,53	0,01	0	0,38	0	0	0,04	0,58	0,38	0,032
4	62336	0,05	0	0	0	0,95	0	0	0	0	0,95	0,04	0,01	0	0,04
4	9931	0	0	0,04	0	0	0	0	0,55	0,41	0	0,05	0,55	0,4	0,028
4	60890	0	0,42	0	0	0,48	0,1	0	0	0	0,06	0,41	0,11	0,42	0,046
4	23427	0	0	0,04	0	0	0,06	0	0,9	0	0	0,04	0,9	0,06	0,024
4	22151	0	0,04	0,03	0	0,93	0	0	0	0	0,94	0,04	0	0,02	0,046
4	30828	0	0	0	0,06	0,01	0	0	0,93	0	0	0	0,05	0,95	0,048
4	100000	0,63	0,09	0,24	0	0,04	0	0	0	0	0	0,92	0,03	0,05	0,794
4	100000	0,03	0	0,91	0,04	0	0,01	0,01	0	0	0	0,9	0,04	0,06	0,53
4	21598	0,06	0,02	0	0	0,01	0,55	0	0,33	0,03	0,04	0,56	0,37	0,03	0,038
4	100000	0	0	1	0	0	0	0	0	0	0	1	0	0	0,578
4	32623	0	0,91	0	0,01	0,01	0	0	0,07	0	0,07	0,02	0	0,91	0,04
4	17994	0,04	0,01	0,04	0	0	0,02	0,01	0,88	0	0	0,06	0,94	0	0,04
4	100000	0,08	0	0,9	0	0,02	0	0	0	0	0	0,9	0,02	0,08	0,508
4	70899	0	0	0,04	0,03	0,79	0,02	0	0,12	0	0	0,11	0,05	0,84	0,03
4	36526	0	0	0,04	0	0,37	0,06	0	0,04	0,49	0,04	0,41	0,06	0,49	0,01
4	39793	0,01	0	0,04	0	0,53	0,18	0	0	0,24	0,03	0,04	0	0,93	0,016
4	28834	0,01	0,03	0,02	0,04	0	0	0	0,9	0	0,08	0,52	0,01	0,39	0,048
4	58963	0,06	0	0	0	0,93	0	0	0,01	0	0,07	0	0,93	0	0,046
4	100000	0	0	0,98	0	0,02	0	0	0	0	0	0,98	0	0,02	0,606

4	22586	0,06	0	0	0	0	0,89	0	0	0,05	0	0,03	0,94	0,03	0,048
4	9553	0,03	0	0	0,06	0,89	0	0	0,02	0	0,03	0,02	0,74	0,21	0,048
4	14131	0	0,05	0,01	0	0,05	0,89	0	0	0	0,91	0,05	0,03	0,01	0,048
4	45029	0,05	0	0	0	0	0,95	0	0	0	0,7	0	0,11	0,19	0,048
4	55633	0	0	0	0,09	0,91	0	0	0	0	0,09	0	0,91	0	0,04
4	85346	0	0	0,04	0	0,89	0,03	0	0	0,04	0,89	0,04	0,07	0	0,04
4	34844	0	0,05	0,04	0	0,86	0	0,02	0,02	0,01	0,06	0,01	0,88	0,05	0,046
4	14413	0	0	0,04	0,01	0	0	0	0,95	0	0	0	0,01	0,99	0,012
4	23141	0,05	0,13	0,03	0	0,79	0	0	0	0	0,05	0,14	0	0,81	0,046
4	10959	0	0,04	0	0	0,76	0,02	0,06	0,12	0	0,16	0,8	0,04	0	0,036
4	62473	0	0,03	0	0,1	0,85	0,02	0	0	0	0,61	0,02	0,01	0,36	0,048
4	100000	0	0	0,98	0	0	0,02	0	0	0	0	0,98	0,02	0	0,522
4	39470	0,04	0	0	0,08	0,24	0,32	0	0,32	0	0,32	0,32	0,24	0,12	0,04
4	16493	0	0	0,04	0	0,95	0	0,01	0	0	0,09	0,9	0,01	0	0,004
4	2597	0	0	0,04	0	0,85	0,1	0	0	0,01	0,76	0,05	0,1	0,09	0,032
4	24114	0	0	0,04	0	0,95	0	0	0,01	0	0,35	0	0,04	0,61	0,018
4,25	34236	0,05	0	0,02	0,05	0,84	0	0	0,01	0,03	0,88	0,06	0	0,06	0,046
4,25	16668	0	0,08	0	0	0	0,92	0	0	0	0,96	0	0,04	0	0,046
4,25	35514	0	0	0,04	0	0	0,05	0	0,9	0,01	0	0,04	0,95	0,01	0,034
4,25	10726	0	0	0,04	0	0,02	0	0	0,93	0,01	0,02	0,95	0,01	0,02	0,01
4,25	12699	0	0	0,04	0	0,64	0,19	0	0	0,13	0	0,05	0,82	0,13	0,022
4,25	100000	0,05	0	0,92	0	0	0,03	0	0	0	0,05	0,74	0	0,21	0,424
4,25	6261	0	0	0,04	0	0	0,96	0	0	0	0	0,04	0,96	0	0,018
4,25	36097	0,02	0	0,03	0	0,92	0	0,03	0	0	0,92	0,03	0,05	0	0,042
4,25	74408	0,01	0,05	0,04	0	0	0	0,01	0	0,89	0	0,11	0	0,89	0,032
4,25	46981	0,07	0	0	0	0,01	0,86	0	0,06	0	0,08	0	0,81	0,11	0,048
4,25	52717	0	0	0,04	0	0	0,96	0	0	0	0,02	0,01	0	0,97	0,006
4,25	79495	0	0	0	0,07	0,88	0	0	0,05	0	0,01	0,88	0,11	0	0,048
4,25	30107	0,02	0	0	0	0,85	0	0,13	0	0	0,85	0	0,15	0	0,04
4,25	18887	0	0,02	0,04	0	0,94	0	0	0	0	0,94	0	0,04	0,02	0,046
4,25	67902	0	0,07	0	0	0,88	0	0,05	0	0	0,95	0,02	0,03	0	0,048
4,25	29206	0,05	0,01	0	0	0,21	0	0	0,73	0	0,95	0,05	0	0	0,048
4,25	25650	0,03	0	0,04	0	0,93	0	0	0	0	0	0	1	0	0,046

4,25	50201	0,04	0	0,03	0	0	0,93	0	0	0	0,93	0	0,03	0,04	0,042
4,25	16891	0,05	0,01	0,04	0	0	0,74	0	0	0,16	0,05	0,18	0	0,77	0,042
4,25	39547	0	0	0,04	0,01	0,89	0,06	0	0	0	0,89	0,08	0,03	0	0,046
4,25	17714	0,05	0	0,03	0	0	0,03	0	0,89	0	0	0,09	0,02	0,89	0,048
4,25	69118	0	0	0	0,11	0	0,88	0	0	0,01	0,01	0,86	0,11	0,02	0,048
4,25	7538	0,04	0,09	0	0	0,78	0	0,01	0,08	0	0,03	0,09	0,09	0,79	0,042
4,25	71676	0	0,34	0	0	0,49	0	0	0,17	0	0	0,17	0,34	0,49	0,048
4,25	100000	0,22	0	0,77	0	0,01	0	0	0	0	0,77	0	0,23	0	0,814
4,25	77576	0,01	0,01	0,04	0	0,91	0,02	0,01	0	0	0	0,02	0,91	0,07	0,044
4,25	40480	0	0,06	0,04	0	0,2	0	0	0,39	0,31	0	0,39	0,31	0,3	0,044
4,25	34703	0	0,3	0,01	0,01	0,09	0	0	0,43	0,16	0,14	0,33	0	0,53	0,042
4,25	10108	0	0,02	0	0,05	0,88	0	0,01	0,04	0	0,01	0,59	0,07	0,33	0,042
4,25	37583	0	0,3	0,04	0	0,66	0	0	0	0	0	0,3	0	0,7	0,006
4,25	17423	0,03	0,1	0	0	0,7	0,16	0	0,01	0	0,2	0	0,1	0,7	0,046
4,25	13237	0	0,11	0,03	0	0	0	0	0,86	0	0	0	0,89	0,11	0,048
4,25	60420	0	0,1	0	0	0,13	0,18	0	0,58	0,01	0,68	0	0,32	0	0,044
4,25	16736	0	0,04	0,04	0	0,92	0	0	0	0	0,1	0,04	0	0,86	0,004
4,25	17992	0	0,02	0,04	0	0,33	0,54	0	0	0,07	0,35	0,07	0,55	0,03	0,042
4,25	73608	0	0,01	0	0,27	0,72	0	0	0	0	0,93	0,06	0	0,01	0,044
4,25	30112	0	0,13	0	0,05	0,82	0	0	0	0	0,05	0,03	0	0,92	0,044
4,25	35271	0	0	0,04	0	0,37	0,55	0,04	0	0	0,41	0,04	0	0,55	0,038
4,25	33165	0	0	0,04	0	0	0,02	0,01	0	0,93	0,01	0,04	0	0,95	0,018
4,25	31603	0	0	0	0,19	0,81	0	0	0	0	0,81	0	0,19	0	0,042
4,25	8862	0	0,04	0,04	0	0,91	0,01	0	0	0	0	0	0,92	0,08	0,044
4,25	54126	0	0	0,01	0,06	0,86	0	0	0,07	0	0,14	0	0	0,86	0,044
4,25	33353	0	0,01	0,01	0	0	0	0,1	0,06	0,82	0,17	0,01	0,01	0,81	0,046
4,25	42514	0	0,24	0,01	0,01	0,68	0,06	0	0	0	0,24	0,01	0,01	0,74	0,042
4,25	88779	0	0,02	0,04	0,01	0,05	0	0	0	0,88	0,11	0,05	0,77	0,07	0,042
4,25	76072	0	0	0	0,25	0,75	0	0	0	0	0,94	0	0,05	0,01	0,036
4,25	9775	0,03	0,02	0,03	0	0,03	0,88	0	0	0,01	0,09	0,89	0,02	0	0,048
4,25	31763	0	0,02	0	0,07	0,86	0	0,01	0,04	0	0,05	0,91	0,04	0	0,048
4,25	26065	0,03	0,05	0,02	0	0,16	0,71	0	0	0,03	0,2	0,03	0,77	0	0,046
4,25	23907	0,05	0	0	0	0,95	0	0	0	0	1	0	0	0	0,048

4,25	19718	0,05	0,03	0	0	0,92	0	0	0	0	0,08	0	0,92	0	0,048
4,25	23299	0	0,13	0,04	0	0	0,75	0	0	0,08	0,14	0,04	0	0,82	0,036
4,25	30040	0,03	0	0	0,03	0,92	0	0	0	0,02	0,01	0,94	0,03	0,02	0,038
4,25	59696	0	0	0,02	0,09	0	0,88	0,01	0	0	0	0,75	0,23	0,02	0,038
4,25	48745	0	0	0,04	0	0,01	0	0,14	0,81	0	0,8	0,17	0,03	0	0,048
4,25	100000	0	0,02	0,92	0,02	0,04	0	0	0	0	0,02	0,92	0,06	0	0,588
4,25	91754	0	0	0	0,08	0,91	0	0	0,01	0	0,08	0,91	0,01	0	0,048
4,25	22127	0	0	0,04	0	0,58	0	0	0	0,38	0	0,62	0,38	0	0,02
4,25	49318	0	0,51	0,03	0,01	0,01	0	0	0	0,44	0	0,04	0	0,96	0,046
4,25	24982	0,04	0	0	0,03	0,84	0	0	0,06	0,03	0	0,09	0,91	0	0,048
4,25	3786	0	0	0,04	0	0,85	0	0,1	0,01	0	0,85	0,04	0,1	0,01	0,046
4,25	29328	0,05	0	0	0	0,95	0	0	0	0	0,05	0	0	0,95	0,036
4,25	99265	0,04	0	0	0	0	0	0,02	0,94	0	0,04	0	0	0,96	0,048
4,25	64834	0	0	0	0,08	0,91	0	0	0	0,01	0,08	0,01	0,26	0,65	0,046
4,25	100000	0,02	0	0,76	0	0	0	0,02	0,05	0,15	0	0,78	0,2	0,02	0,448
4,25	82047	0	0	0,04	0	0	0,92	0	0,04	0	0	0,49	0,48	0,03	0,03
4,25	53663	0	0,12	0	0,01	0	0,83	0	0	0,04	0,01	0,04	0,95	0	0,032
4,25	17751	0,03	0,96	0	0,01	0	0	0	0	0	0,01	0,03	0	0,96	0,042
4,25	31108	0,01	0	0	0	0,86	0,01	0,07	0,05	0	0,06	0,86	0,08	0	0,048
4,25	20927	0	0,09	0	0,03	0	0,33	0	0,55	0	0,63	0	0,33	0,04	0,048
4,25	26169	0	0	0,04	0	0,88	0	0	0,07	0,01	0,05	0	0,07	0,88	0
4,25	20141	0,06	0	0	0	0	0,89	0	0,05	0	0,2	0	0	0,8	0,042
4,25	48937	0,05	0	0	0	0,93	0,02	0	0	0	0,07	0	0,93	0	0,048
4,25	50854	0	0	0,04	0	0,31	0,5	0,01	0	0,14	0,55	0,31	0	0,14	0,026
4,25	32920	0	0,08	0	0	0	0,79	0,06	0	0,07	0,13	0,02	0,85	0	0,048
4,25	23918	0	0,24	0	0,05	0	0	0	0,71	0	0	0,71	0	0,29	0,046
4,25	52902	0	0	0,04	0	0,88	0	0,05	0	0,03	0,06	0,92	0,02	0	0,044
4,25	44621	0	0	0	0,21	0,65	0,14	0	0	0	0,86	0	0	0,14	0,04
4,25	17851	0,02	0	0,04	0,01	0	0,61	0,04	0	0,28	0,01	0,08	0,61	0,3	0,048
4,25	78520	0	0	0,04	0	0	0	0,01	0,95	0	0	0,04	0,92	0,04	0,032
4,25	25442	0	0,24	0	0	0,69	0	0	0,07	0	0,07	0,69	0,13	0,11	0,048
4,25	33843	0,04	0	0,02	0	0,82	0,1	0,01	0	0,01	0	0,02	0,88	0,1	0,04
4,25	17605	0	0	0,02	0,06	0,92	0	0	0	0	0	0,93	0,05	0,02	0,048

4,25	75527	0	0,17	0,02	0	0,53	0	0,02	0	0,26	0,19	0,28	0	0,53	0,044
4,25	50597	0	0	0,04	0	0,12	0,1	0	0,74	0	0	0	0,84	0,16	0,018
4,25	38055	0	0	0,03	0	0,9	0	0,05	0	0,02	0,04	0,03	0,02	0,91	0,04
4,25	44508	0,04	0,05	0	0	0,82	0	0	0,08	0,01	0,08	0,87	0,05	0	0,044
4,25	55949	0,03	0,02	0,03	0	0	0,51	0	0	0,41	0	0,03	0,53	0,44	0,042
4,25	89864	0,01	0	0	0,04	0,9	0	0	0,04	0,01	0,01	0,94	0,05	0	0,042
4,25	27872	0	0,06	0	0,08	0,67	0	0	0,19	0	0,86	0,08	0,06	0	0,034
4,25	17318	0	0	0	0,11	0,89	0	0	0	0	0	0	1	0	0,04
4,25	33531	0	0	0,03	0,06	0,73	0	0,01	0,17	0	0,06	0,03	0,74	0,17	0,04
4,25	30764	0	0,11	0,01	0	0	0,34	0,03	0,51	0	0,51	0,11	0,01	0,37	0,048
4,25	24297	0,01	0,02	0,01	0,06	0,83	0	0	0	0,07	0,02	0,02	0,87	0,09	0,046
4,25	30815	0	0	0	0,19	0,81	0	0	0	0	0,81	0	0,19	0	0,048
4,25	100000	0	0,9	0,09	0	0	0	0,01	0	0	0,02	0	0,2	0,78	0,168
4,25	35871	0,04	0	0	0,01	0,9	0,05	0	0	0	0,04	0,91	0,05	0	0,038
4,25	38901	0	0	0	0,12	0,8	0	0	0,08	0	0	0,12	0,88	0	0,048
4,25	37347	0	0	0,04	0	0,93	0,01	0	0,01	0,01	0,95	0,04	0,01	0	0,04
4,25	7557	0	0	0	0,12	0	0,05	0	0,79	0,04	0,12	0	0,83	0,05	0,048
4,5	68882	0	0,01	0,04	0,01	0	0	0	0,92	0,02	0	0,05	0	0,95	0,006
4,5	100000	0	0,04	0,94	0	0	0	0,01	0,01	0	0	0,99	0,01	0	0,592
4,5	33477	0	0	0	0,16	0	0,83	0	0,01	0	0,54	0,04	0,42	0	0,044
4,5	98480	0	0,16	0	0	0,78	0	0	0	0,06	0	0,16	0,78	0,06	0,048
4,5	7617	0	0,22	0,04	0	0,02	0,65	0	0	0,07	0,11	0,14	0	0,75	0,048
4,5	33579	0	0	0	0,09	0,9	0	0,01	0	0	0,22	0,78	0	0	0,044
4,5	11634	0	0,19	0,04	0	0	0	0	0,01	0,76	0,01	0,19	0,05	0,75	0,044
4,5	42184	0,05	0	0	0	0	0,93	0,01	0,01	0	0,98	0	0,02	0	0,046
4,5	44803	0	0	0,04	0	0,94	0	0	0,02	0	0,07	0	0	0,93	0
4,5	100000	0,05	0	0,91	0,03	0,01	0	0	0	0	0,09	0,87	0,03	0,01	0,646
4,5	10815	0	0	0	0,38	0,62	0	0	0	0	1	0	0	0	0,04
4,5	55767	0,02	0	0,02	0,05	0,91	0	0	0	0	0,03	0,91	0,01	0,05	0,048
4,5	14036	0	0,08	0,01	0,01	0,1	0	0,01	0,79	0	0,01	0,08	0,9	0,01	0,048
4,5	59221	0	0	0,04	0	0	0	0	0,93	0,03	0,93	0,03	0,03	0,01	0,036
4,5	39955	0,01	0	0,04	0	0,95	0	0	0	0	0,65	0,03	0,32	0	0,038
4,5	57032	0,07	0	0	0	0	0,91	0,02	0	0	0,89	0,07	0	0,04	0,046

4,5	25058	0	0	0	0,28	0,71	0	0	0,01	0	0,96	0	0,01	0,03	0,048
4,5	21500	0	0,28	0	0	0	0	0	0,72	0	0	0	0,28	0,72	0,044
4,5	70741	0	0	0,04	0	0	0	0,04	0,51	0,41	0,49	0,03	0,03	0,45	0,046
4,5	11306	0,01	0	0,04	0	0	0	0	0,58	0,37	0	0,26	0,37	0,37	0,03
4,5	100000	0,09	0	0,86	0	0	0,01	0	0	0,04	0	0,97	0	0,03	0,568
4,5	22106	0	0,02	0,01	0,09	0,17	0,62	0	0,09	0	0,62	0,27	0,11	0	0,04
4,5	71666	0	0	0,04	0	0	0,9	0,06	0	0	0	0,96	0,04	0	0,048
4,5	3158	0	0	0,04	0	0,55	0	0	0,22	0,19	0	0,58	0,22	0,2	0,014
4,5	10764	0,04	0	0	0,08	0,88	0	0	0	0	0,91	0,09	0	0	0,042
4,5	40863	0	0,13	0	0	0,86	0,01	0	0	0	0	0,01	0,86	0,13	0,038
4,5	19026	0,02	0,01	0,04	0	0	0	0	0,93	0	0,03	0,04	0,93	0	0,042
4,5	22454	0	0,17	0,02	0,03	0	0	0	0	0,78	0	0,15	0,07	0,78	0,048
4,5	74259	0,02	0	0	0,09	0,52	0,14	0	0,23	0	0,66	0,01	0,33	0	0,048
4,5	26888	0	0,22	0	0,03	0,68	0,01	0	0,06	0	0,03	0	0,29	0,68	0,046
4,5	87578	0	0	0,04	0	0,31	0	0	0,07	0,58	0,19	0,09	0,15	0,57	0,024
4,5	4796	0	0	0,01	0	0,8	0,07	0,12	0	0	0,59	0,01	0,12	0,28	0,048
4,5	36678	0	0,12	0	0	0	0,88	0	0	0	0	1	0	0	0,04
4,5	34969	0,01	0	0,04	0	0	0	0	0,95	0	0	0,05	0,95	0	0,022
4,5	9040	0	0,03	0	0,06	0,53	0,03	0,05	0,28	0,02	0,34	0,03	0,58	0,05	0,048
4,5	65114	0	0,14	0,03	0,02	0	0	0	0,03	0,78	0,17	0,03	0	0,8	0,048
4,5	31431	0,01	0,02	0	0	0,71	0	0,09	0,16	0,01	0	0	1	0	0,046
4,5	35182	0	0	0	0,16	0	0,81	0	0	0,03	0,16	0	0,84	0	0,044
4,5	17550	0,05	0	0,01	0	0	0,93	0,01	0	0	0,05	0	0,01	0,94	0,048
4,5	96702	0,04	0,34	0	0	0,62	0	0	0	0	0,04	0	0	0,96	0,04
4,5	7328	0	0	0,03	0,05	0,21	0,11	0	0	0,6	0,11	0,03	0	0,86	0,032
4,5	48218	0	0	0,04	0	0,93	0	0	0,03	0	0	0,97	0,03	0	0,006
4,5	58061	0	0,01	0,04	0	0,94	0	0	0	0,01	0,05	0	0,94	0,01	0,016
4,5	18947	0	0,11	0,04	0	0	0	0	0	0,85	0,01	0,15	0	0,84	0,034
4,5	14377	0	0,12	0,02	0,02	0,78	0	0	0	0,06	0,05	0,02	0,67	0,26	0,046
4,5	100000	0,17	0,03	0,8	0	0	0	0	0	0	0,16	0,8	0,04	0	0,77
4,5	64849	0	0,01	0	0,15	0,82	0,01	0	0,01	0	0,82	0,01	0,05	0,12	0,046
4,5	17314	0	0,01	0	0,07	0	0	0	0,9	0,02	0,09	0	0,41	0,5	0,044
4,5	60375	0	0	0,04	0	0,35	0	0	0,61	0	0	0,39	0,61	0	0,016

4,5	43090	0	0	0	0	0,9	0,04	0,06	0	0	0,04	0	0,06	0,9	0,046
4,5	13971	0	0	0	0,09	0,7	0,2	0,01	0	0	0,09	0	0,7	0,21	0,046
4,5	66901	0	0,02	0	0,1	0,88	0	0	0	0	0	0	0,77	0,23	0,048
4,5	15892	0	0	0	0,06	0,94	0	0	0	0	0	1	0	0	0,042
4,5	40029	0	0	0	0,12	0,86	0	0	0,02	0	0,14	0	0,86	0	0,046
4,5	5942	0	0	0,04	0	0,26	0,01	0,02	0,57	0,1	0,6	0,12	0,28	0	0,04
4,5	35444	0	0,07	0	0,01	0	0	0,01	0,91	0	0,79	0,2	0,01	0	0,046
4,5	96748	0	0	0,04	0	0,96	0	0	0	0	0	0,05	0	0,95	0,002
4,5	68824	0	0,04	0,02	0	0,8	0	0,06	0,05	0,03	0,04	0,85	0,11	0	0,048
4,5	26191	0,03	0,05	0	0,02	0,03	0,29	0	0,51	0,07	0,07	0,36	0,5	0,07	0,046
4,5	22678	0,01	0	0,04	0	0	0	0,01	0,79	0,15	0,8	0,15	0,01	0,04	0,042
4,5	45096	0	0,07	0	0	0,01	0,92	0	0	0	0,92	0,07	0,01	0	0,04
4,5	11934	0	0,12	0,04	0	0,82	0	0	0	0,02	0,03	0,02	0,83	0,12	0,042
4,5	20098	0	0	0	0,25	0,73	0	0	0	0,02	0,88	0,12	0	0	0,046
4,5	11911	0	0,01	0,04	0	0	0	0	0,22	0,73	0,2	0,04	0	0,76	0,022
4,5	29938	0,01	0	0,04	0	0	0,03	0,01	0,02	0,89	0,04	0,04	0,01	0,91	0,034
4,5	11381	0,01	0	0,02	0	0,83	0	0,12	0,02	0	0,83	0,14	0,03	0	0,04
4,5	24527	0	0	0,03	0	0,77	0	0,1	0,1	0	0,45	0,51	0,03	0,01	0,042
4,5	40224	0,04	0	0,03	0	0,92	0	0,01	0	0	0,01	0,94	0,05	0	0,04
4,5	18517	0	0,03	0	0,08	0,88	0,01	0	0	0	0,01	0	0,96	0,03	0,046
4,5	12001	0,01	0	0,04	0	0,11	0,8	0	0	0,04	0,02	0,02	0,06	0,9	0,026
4,5	37796	0,06	0,89	0	0	0,05	0	0	0	0	0	0,18	0	0,82	0,046
4,5	56946	0	0	0,04	0	0,3	0	0	0	0,66	0	0,34	0	0,66	0,008
4,5	19272	0	0,36	0,04	0	0,04	0	0	0,56	0	0,04	0,02	0	0,94	0,032
4,5	61261	0	0,13	0	0	0,86	0	0	0	0,01	0,01	0	0,99	0	0,048
4,5	55518	0,05	0,04	0	0	0,91	0	0	0	0	0,05	0,04	0	0,91	0,048
4,5	100000	0,02	0	0,97	0	0,01	0	0	0	0	0	0,77	0	0,23	0,412
4,5	57609	0,05	0	0,02	0	0,93	0	0	0	0	0,88	0,02	0,1	0	0,048
4,5	43531	0,06	0	0	0	0,79	0,15	0	0	0	0,07	0,93	0	0	0,048
4,5	56477	0	0,29	0,04	0	0	0	0	0,5	0,17	0,24	0,04	0	0,72	0,044
4,5	59194	0	0,96	0	0,03	0	0	0,01	0	0	0	0	0,03	0,97	0,044
4,5	31029	0,03	0	0,02	0	0,15	0,12	0,01	0,67	0	0,12	0,06	0,64	0,18	0,04
4,5	100000	0	0,04	0,86	0	0	0	0	0	0,1	0,02	0,78	0,2	0	0,444

4,5	47705	0,04	0	0,04	0	0,78	0	0	0,14	0	0,3	0,52	0,18	0	0,046
4,5	40436	0	0,22	0,02	0	0,15	0	0	0,61	0	0,17	0	0,22	0,61	0,048
4,5	72705	0	0,02	0,04	0	0	0	0	0,04	0,9	0,02	0,04	0	0,94	0,02
4,5	36336	0	0,01	0,04	0,02	0,92	0	0,01	0	0	0	0	0,96	0,04	0,044
4,5	53127	0	0,16	0	0	0,63	0	0	0,21	0	0,26	0,48	0,16	0,1	0,048
4,5	24513	0	0	0	0,18	0,82	0	0	0	0	0,71	0	0,16	0,13	0,046
4,5	48710	0	0	0,04	0	0	0,05	0	0,91	0	0	0,75	0,25	0	0,028
4,5	45887	0,05	0	0	0	0,95	0	0	0	0	0,05	0	0	0,95	0,048
4,5	21978	0	0	0,04	0	0	0	0	0,15	0,81	0	0,03	0,18	0,79	0,006
4,5	28687	0	0	0	0,03	0	0,16	0,03	0,72	0,06	0,17	0,02	0,06	0,75	0,038
4,5	35514	0	0,02	0	0,16	0	0,8	0	0,01	0,01	0,82	0,01	0,16	0,01	0,04
4,5	15765	0,04	0	0	0	0	0,49	0,05	0,09	0,33	0,09	0,33	0,49	0,09	0,046
4,5	68130	0,06	0	0	0	0,9	0	0	0,02	0,02	0,96	0,03	0	0,01	0,04
4,5	39319	0	0	0,04	0	0,64	0	0	0,02	0,3	0,64	0,04	0,02	0,3	0,03
4,5	100000	0,35	0,02	0,55	0	0	0,04	0,03	0,01	0	0,37	0,21	0	0,42	0,652
4,5	20455	0	0	0,04	0,03	0,02	0	0	0	0,91	0	0,05	0,95	0	0,04
4,5	100000	0,03	0	0,96	0	0	0	0,01	0	0	0	0,98	0	0,02	0,604
4,5	42738	0,01	0	0,04	0	0	0	0,01	0,94	0	0,02	0	0,04	0,94	0,026
4,75	33852	0	0	0	0	0	0,88	0,12	0	0	0,12	0	0,65	0,23	0,044
4,75	20985	0,05	0	0	0	0,36	0,1	0	0,49	0	0,15	0,47	0,12	0,26	0,048
4,75	31645	0	0,07	0	0,05	0,87	0	0	0,01	0	0,94	0,01	0	0,05	0,048
4,75	32294	0	0	0,02	0	0,57	0,01	0,08	0,02	0,3	0,09	0,58	0,32	0,01	0,04
4,75	89877	0,06	0	0	0	0,81	0	0	0,13	0	0,05	0	0	0,95	0,04
4,75	25564	0	0	0	0,1	0,53	0	0	0,37	0	0,38	0,09	0	0,53	0,048
4,75	100000	0	0	1	0	0	0	0	0	0	0	1	0	0	0,63
4,75	20645	0	0	0,04	0	0,09	0	0	0,87	0	0,16	0,8	0,04	0	0,024
4,75	74920	0	0,02	0	0,24	0	0,73	0	0,01	0	0,96	0	0,03	0,01	0,046
4,75	43425	0	0	0,01	0,07	0,92	0	0	0	0	0,08	0,92	0	0	0,042
4,75	3370	0	0,02	0,04	0	0,91	0	0	0,03	0	0	0,05	0,04	0,91	0,002
4,75	29182	0	0	0,03	0,02	0,88	0	0,06	0	0,01	0,09	0	0,03	0,88	0,044
4,75	100000	0	0,01	0,98	0	0	0	0	0,01	0	0,01	0,99	0	0	0,704
4,75	27179	0	0	0,04	0,01	0,05	0,01	0	0,89	0	0,05	0,06	0,89	0	0,028
4,75	10908	0	0,38	0,02	0	0,58	0	0	0	0,02	0,11	0,49	0,02	0,38	0,04

4,75	10705	0	0,01	0,04	0	0,16	0,72	0,02	0	0,05	0,79	0,12	0,05	0,04	0,042
4,75	49803	0,01	0,04	0	0,06	0,82	0,07	0	0	0	0,01	0	0,9	0,09	0,046
4,75	7473	0	0	0	0,31	0,69	0	0	0	0	1	0	0	0	0,046
4,75	16254	0	0	0,01	0,12	0,74	0,08	0	0,04	0,01	0,11	0,08	0,75	0,06	0,048
4,75	11326	0	0	0,04	0	0,93	0	0	0	0,03	0	0,03	0,01	0,96	0
4,75	61920	0	0	0	0	0,91	0	0,07	0	0,02	0	0,03	0,06	0,91	0,046
4,75	25439	0	0,2	0	0,01	0,79	0	0	0	0	0,01	0,79	0,2	0	0,046
4,75	91324	0,03	0,02	0,03	0	0,8	0,01	0	0	0,11	0,13	0,81	0,01	0,05	0,04
4,75	15431	0	0	0,04	0	0,04	0	0	0,63	0,29	0,02	0,06	0,92	0	0,03
4,75	39850	0	0	0,04	0	0	0,03	0	0,5	0,43	0,47	0,04	0,03	0,46	0,018
4,75	35389	0,01	0,16	0,04	0,03	0,69	0,04	0	0,02	0,01	0,05	0,04	0,03	0,88	0,046
4,75	14194	0,03	0	0	0	0,92	0	0,05	0	0	0	0	0,95	0,05	0,044
4,75	25821	0	0	0,04	0,1	0,05	0,27	0	0,54	0	0,81	0,05	0,14	0	0,048
4,75	100000	0	0	0,96	0	0	0	0,03	0	0,01	0	0,96	0	0,04	0,492
4,75	69083	0	0	0	0,31	0,69	0	0	0	0	0,93	0,02	0,01	0,04	0,048
4,75	100000	0	0	0,99	0	0	0	0,01	0	0	0	0,99	0	0,01	0,714
4,75	10984	0	0	0	0,07	0,84	0	0	0,09	0	0,07	0	0,09	0,84	0,046
4,75	66118	0	0,67	0	0	0,31	0	0	0	0,02	0,02	0,98	0	0	0,044
4,75	23100	0	0,18	0,02	0,04	0,75	0,01	0	0	0	0,2	0	0,05	0,75	0,044
4,75	39795	0	0,06	0,04	0	0,02	0,04	0	0,84	0	0,04	0,04	0,92	0	0,038
4,75	78349	0,05	0	0,01	0,06	0,87	0	0	0	0,01	0,93	0,02	0,05	0	0,042
4,75	19722	0	0,02	0,04	0	0,94	0	0	0	0	0,04	0,02	0,92	0,02	0,036
4,75	36930	0	0,03	0,04	0	0,39	0	0	0,12	0,42	0	0,07	0,51	0,42	0,028
4,75	69989	0	0	0,04	0	0	0	0	0,96	0	0	0,04	0,96	0	0,02
4,75	21394	0,04	0	0,02	0	0	0	0	0,92	0,02	0,91	0,01	0,02	0,06	0,048
4,75	61714	0	0,1	0	0,02	0,55	0	0	0,33	0	0,08	0	0,92	0	0,046
4,75	48034	0	0,17	0	0	0	0,83	0	0	0	0	0,83	0,17	0	0,04
4,75	100000	0,01	0	0,99	0	0	0	0	0	0	0,21	0,78	0,01	0	0,868
4,75	30861	0,06	0	0	0	0,85	0,08	0	0,01	0	0,07	0,85	0,08	0	0,044
4,75	13550	0	0	0,04	0	0	0	0	0,96	0	0	0,03	0	0,97	0
4,75	31206	0	0	0	0,3	0,66	0,03	0	0	0,01	0,96	0	0,04	0	0,048
4,75	8951	0	0,01	0	0,06	0,9	0,01	0	0,02	0	0,09	0,82	0,01	0,08	0,048
4,75	30218	0,05	0	0	0	0,78	0,01	0	0,15	0,01	0,16	0	0,78	0,06	0,042

4,75	43017	0	0	0	0,2	0,8	0	0	0	0	0,83	0	0,17	0	0,044
4,75	46453	0,05	0	0,01	0	0,92	0,02	0	0	0	0,53	0,47	0	0	0,036
4,75	100000	0	0	0,82	0	0	0,02	0,05	0	0,11	0	0,93	0,02	0,05	0,458
4,75	55198	0	0,02	0,02	0,08	0	0	0	0,88	0	0,08	0,02	0,83	0,07	0,044
4,75	54262	0,01	0	0	0,08	0,16	0,75	0	0	0	0,01	0,83	0	0,16	0,048
4,75	96197	0	0	0,01	0,17	0,8	0,02	0	0	0	0,8	0,2	0	0	0,042
4,75	70056	0	0	0	0,08	0,37	0	0	0,55	0	0	0,18	0,55	0,27	0,048
4,75	69892	0	0	0,04	0	0,19	0,03	0,04	0	0,7	0,04	0,06	0	0,9	0,042
4,75	52746	0,06	0	0	0	0	0	0	0,94	0	0	0	0,06	0,94	0,048
4,75	23113	0,03	0	0,03	0	0	0	0	0,94	0	0,96	0,03	0,01	0	0,042
4,75	38878	0	0	0,04	0,02	0	0,93	0	0,01	0	0	0,94	0	0,06	0,024
4,75	100000	0	0	1	0	0	0	0	0	0	0	1	0	0	0,646
4,75	25188	0	0	0	0,34	0,66	0	0	0	0	1	0	0	0	0,04
4,75	65718	0,05	0	0	0,01	0,94	0	0	0	0	0,05	0,01	0	0,94	0,042
4,75	38349	0,03	0	0,04	0	0,03	0,82	0	0,07	0,01	0,11	0,02	0,04	0,83	0,046
4,75	23013	0	0,15	0	0,04	0,78	0	0	0,02	0,01	0,19	0,02	0	0,79	0,046
4,75	41591	0,06	0	0	0	0,9	0	0,01	0,01	0,02	0,95	0,02	0,01	0,02	0,048
4,75	53180	0	0	0,04	0,04	0,79	0	0	0,05	0,08	0	0,13	0,08	0,79	0,048
4,75	35567	0,06	0	0	0,01	0	0,93	0	0	0	0,06	0,01	0	0,93	0,044
4,75	43528	0,05	0,01	0	0	0,94	0	0	0	0	0,05	0	0,95	0	0,044
4,75	69210	0,03	0	0	0	0,89	0,02	0,06	0	0	0,06	0,03	0,89	0,02	0,038
4,75	100000	0,91	0	0,05	0,01	0,01	0	0	0,02	0	0,01	0,96	0,01	0,02	0,926
4,75	100000	0,04	0,35	0	0,04	0,01	0,17	0	0,29	0,1	0,35	0,29	0,1	0,26	0,218
4,75	32852	0,03	0	0,04	0	0,84	0	0,02	0	0,07	0,05	0,03	0,82	0,1	0,046
4,75	13702	0	0	0,04	0	0,83	0,01	0,08	0	0,04	0,14	0,86	0	0	0,048
4,75	26511	0	0,13	0	0	0	0,01	0	0,86	0	0,03	0	0,84	0,13	0,048
4,75	7771	0,04	0	0	0	0,91	0	0,02	0	0,03	0	0,94	0,06	0	0,042
4,75	28502	0	0	0,04	0	0	0,01	0	0	0,95	0	0,05	0,95	0	0,02
4,75	35966	0	0,13	0	0,02	0,48	0	0,01	0,23	0,13	0,15	0,48	0,24	0,13	0,044
4,75	52603	0	0,15	0	0	0,77	0,07	0,01	0	0	0,18	0,72	0,06	0,04	0,044
4,75	19339	0,01	0,17	0	0,03	0	0,01	0,02	0,76	0	0,05	0,2	0	0,75	0,048
4,75	40129	0,06	0	0	0	0,87	0,07	0	0	0	0,93	0	0,07	0	0,042
4,75	3602	0	0	0,04	0,05	0,04	0	0	0,11	0,76	0,14	0,04	0,05	0,77	0,042

4,75	20884	0,02	0	0,03	0,07	0,86	0	0	0,02	0	0,88	0	0,06	0,06	0,046
4,75	82682	0	0	0,04	0	0,92	0	0,04	0	0	0	0,05	0,92	0,03	0,034
4,75	16153	0,02	0,02	0,01	0	0,16	0,79	0	0	0	0,95	0,02	0,03	0	0,044
4,75	18966	0	0,88	0	0	0,05	0	0	0,07	0	0,07	0,24	0	0,69	0,044
4,75	17821	0	0,29	0,01	0	0,7	0	0	0	0	0	0,01	0,29	0,7	0,048
4,75	7943	0	0	0,04	0	0	0,82	0	0,14	0	0,7	0	0,26	0,04	0,028
4,75	100000	0	0,05	0,94	0	0	0	0,01	0	0	0	0,99	0	0,01	0,648
4,75	16647	0	0,11	0,04	0	0	0	0	0,85	0	0,11	0,89	0	0	0,036
4,75	74906	0	0	0,04	0	0,24	0	0	0	0,72	0	0,76	0,24	0	0,028
4,75	8546	0,04	0	0,04	0,01	0,91	0	0	0	0	0,05	0,02	0	0,93	0,042
4,75	61148	0	0,08	0	0	0	0	0	0,92	0	0,92	0	0	0,08	0,046
4,75	35459	0	0	0,04	0,03	0	0	0,02	0,91	0	0	0,02	0,96	0,02	0,038
4,75	47188	0,05	0,02	0,04	0	0,89	0	0	0	0	0,06	0,9	0,04	0	0,048
4,75	43847	0,06	0	0	0	0,9	0,04	0	0	0	0,1	0,77	0,13	0	0,046
4,75	37384	0,05	0	0	0	0,95	0	0	0	0	0,04	0,96	0	0	0,042
4,75	18626	0,04	0	0	0,08	0	0	0	0,84	0,04	0,84	0,12	0,02	0,02	0,044
4,75	100000	0	0,28	0,35	0	0,01	0,19	0,17	0	0	0,29	0,41	0,3	0	0,456
4,75	56502	0	0,15	0	0	0	0,77	0	0	0,08	0	0,84	0,08	0,08	0,044
4,75	100000	0,01	0	0,96	0,02	0	0	0	0	0,01	0	0,98	0,01	0,01	0,518
5	45592	0	0	0	0,07	0,83	0	0	0,09	0,01	0,01	0,46	0,16	0,37	0,048
5	76548	0	0,07	0,04	0	0,89	0	0	0	0	0,01	0,03	0,96	0	0,034
5	100000	0,07	0	0,9	0	0	0,03	0	0	0	0,07	0,9	0,03	0	0,66
5	7355	0	0	0,04	0	0	0,05	0	0	0,91	0	0,04	0,96	0	0,016
5	20423	0	0,04	0	0,08	0,87	0	0,01	0	0	0,18	0,09	0,72	0,01	0,048
5	15573	0,02	0,25	0	0	0,02	0,71	0	0	0	0,02	0,03	0,07	0,88	0,042
5	26044	0	0,14	0	0	0,71	0	0	0,15	0	0	0,18	0,71	0,11	0,048
5	32285	0	0	0	0,1	0,89	0	0,01	0	0	0,04	0,06	0,89	0,01	0,044
5	65109	0	0	0	0,11	0,75	0	0	0,12	0,02	0,12	0,12	0,75	0,01	0,048
5	42751	0,04	0,01	0,01	0,02	0,8	0,01	0,05	0	0,06	0,85	0,06	0,07	0,02	0,046
5	39447	0	0	0	0,15	0,72	0,13	0	0	0	0,68	0	0,32	0	0,048
5	14044	0	0	0	0,07	0,91	0	0	0,02	0	0	0,77	0,07	0,16	0,046
5	100000	0	0	1	0	0	0	0	0	0	0	1	0	0	0,67
5	21307	0,05	0	0	0	0,02	0,55	0	0,31	0,07	0,15	0,45	0,38	0,02	0,048

5	45988	0	0,65	0,02	0	0	0	0	0	0,33	0	0,24	0	0,76	0,046
5	23218	0	0	0	0	0	0	0,19	0,81	0	0,76	0,05	0	0,19	0,048
5	24834	0	0,08	0	0	0,86	0	0	0,03	0,03	0,86	0	0,11	0,03	0,048
5	32829	0	0,88	0	0	0	0	0,04	0,08	0	0	1	0	0	0,048
5	14466	0	0,08	0,04	0	0	0	0	0	0,88	0	0,02	0	0,98	0,026
5	47924	0	0,02	0,04	0	0,91	0,02	0,01	0	0	0,55	0,41	0,02	0,02	0,032
5	100000	0,01	0	0,43	0,05	0	0,04	0	0,04	0,43	0	0,48	0,04	0,48	0,13
5	29085	0	0,01	0,04	0	0	0	0,04	0,91	0	0	0,05	0,91	0,04	0,042
5	52608	0	0	0,04	0	0,86	0	0	0,03	0,07	0	0,9	0,03	0,07	0,006
5	100000	0	0	0,73	0	0,01	0	0,06	0,01	0,19	0	0,92	0,01	0,07	0,362
5	100000	0	0	1	0	0	0	0	0	0	0	1	0	0	0,576
5	63598	0	0	0	0,08	0,55	0,37	0	0	0	0,37	0,08	0	0,55	0,032
5	97517	0,02	0,02	0,04	0	0	0,08	0	0	0,84	0,02	0,06	0	0,92	0,028
5	18116	0	0,02	0,04	0	0,93	0,01	0	0	0	0,01	0,03	0,01	0,95	0,004
5	47742	0,04	0	0	0	0,01	0,09	0,04	0,82	0	0	0,13	0,86	0,01	0,044
5	23049	0	0,01	0,01	0,03	0,88	0	0,07	0	0	0,75	0,04	0,06	0,15	0,036
5	100000	0	0	0,94	0,02	0	0	0,04	0	0	0,02	0,94	0,02	0,02	0,54
5	22380	0	0,09	0,04	0	0	0,55	0	0,31	0,01	0,32	0,09	0	0,59	0,048
5	100000	0,1	0,43	0,42	0,05	0	0	0	0	0	0,69	0,1	0,16	0,05	0,796
5	48816	0,06	0	0	0	0,94	0	0	0	0	0	0	0,08	0,92	0,046
5	9212	0,03	0	0	0	0	0	0,03	0,94	0	0,02	0,03	0,03	0,92	0,046
5	9781	0	0	0,03	0	0,92	0,01	0,04	0	0	0,91	0	0,03	0,06	0,034
5	73739	0	0	0	0,07	0,93	0	0	0	0	0	0,93	0,07	0	0,048
5	83210	0,03	0,05	0	0	0,01	0,91	0	0	0	0,01	0,4	0,54	0,05	0,042
5	9380	0	0	0,02	0,12	0,01	0,03	0	0,81	0,01	0,01	0,2	0,73	0,06	0,042
5	34798	0	0	0,04	0,06	0	0,02	0,01	0,84	0,03	0,01	0,1	0,88	0,01	0,046
5	15778	0	0	0,01	0,04	0	0	0,04	0	0,91	0	0,01	0,13	0,86	0,046
5	23265	0,06	0,02	0	0	0,87	0,05	0	0	0	0,11	0,89	0	0	0,044
5	100000	0	0,01	0,98	0	0	0,01	0	0	0	0	0,98	0,01	0,01	0,6
5	16725	0,07	0	0	0	0,93	0	0	0	0	0,07	0,76	0,17	0	0,048
5	3981	0	0	0	0,06	0	0	0	0,91	0,03	0,06	0	0,03	0,91	0,044
5	56609	0,04	0,02	0	0	0,01	0,91	0,02	0	0	0	0,05	0,71	0,24	0,048
5	63009	0	0,13	0	0	0,48	0,39	0	0	0	0,57	0,25	0	0,18	0,048

5	38653	0,05	0	0,04	0	0,67	0	0	0,24	0	0,05	0,52	0,43	0	0,046
5	85235	0	0,01	0,04	0	0,81	0	0	0	0,14	0,95	0	0,01	0,04	0,036
5	14485	0	0	0	0,22	0,75	0	0	0,03	0	0,75	0,04	0,21	0	0,044
5	93582	0	0,35	0,01	0	0,01	0,58	0	0,05	0	0	0,01	0,12	0,87	0,04
5	27626	0,04	0,03	0	0	0,01	0,91	0	0,01	0	0,05	0,91	0	0,04	0,04
5	31924	0	0	0	0,04	0,94	0	0,02	0	0	0,05	0,95	0	0	0,048
5	47153	0	0,2	0	0	0,74	0,06	0	0	0	0	0,77	0,23	0	0,048
5	100000	0	0	0,97	0	0,01	0	0,02	0	0	0,99	0,01	0	0	0,954
5	31601	0	0,12	0,01	0	0,8	0	0	0,07	0	0,02	0,03	0,92	0,03	0,046
5	86394	0	0	0,04	0	0,02	0	0	0	0,94	0	0,95	0,02	0,03	0,01
5	20664	0,01	0	0	0,1	0,53	0,36	0	0	0	0,19	0,1	0,7	0,01	0,048
5	17370	0	0,09	0,04	0	0	0	0	0	0,87	0	0,13	0	0,87	0,026
5	100000	0,01	0	0,99	0	0	0	0	0	0	0,6	0,4	0	0	0,98
5	14705	0	0	0	0,06	0,94	0	0	0	0	0,06	0	0	0,94	0,048
5	8672	0	0	0,04	0	0	0	0	0	0,96	0,02	0	0,96	0,02	0,018
5	47581	0	0	0,04	0,03	0	0,39	0	0	0,54	0	0	0,61	0,39	0,036
5	61120	0,01	0	0,04	0	0,93	0	0	0,01	0,01	0,01	0,05	0,02	0,92	0,014
5	37676	0	0	0,04	0	0,03	0,16	0	0,17	0,6	0,24	0,05	0,68	0,03	0,024
5	76801	0	0,04	0,04	0	0,24	0,08	0	0,43	0,17	0,11	0,28	0,03	0,58	0,008
5	100000	0,15	0	0,84	0,01	0	0	0	0	0	0	0,99	0	0,01	0,664
5	85100	0	0	0,04	0,01	0	0	0	0,9	0,05	0	0,04	0,01	0,95	0,01
5	31894	0	0	0	0,15	0,84	0	0	0,01	0	0,45	0,54	0	0,01	0,046
5	16315	0,01	0,04	0,04	0	0	0	0	0,91	0	0,08	0,09	0,4	0,43	0,04
5	75896	0	0	0,04	0	0,95	0	0	0,01	0	0,96	0	0,04	0	0,034
5	12431	0	0	0,04	0	0,04	0,13	0	0,23	0,56	0	0,31	0,13	0,56	0,006
5	93145	0	0	0,04	0,03	0	0,42	0	0,05	0,46	0	0	0,05	0,95	0,038
5	14655	0	0,09	0,04	0,01	0	0,01	0	0,85	0	0	0,04	0,1	0,86	0,018
5	9369	0	0	0,04	0,01	0	0,07	0	0,1	0,78	0,11	0,04	0,78	0,07	0,018
5	34919	0	0	0,04	0,01	0	0,93	0,02	0	0	0	0,04	0,95	0,01	0,024
5	8718	0	0	0,04	0	0	0,01	0,02	0,93	0	0,01	0,04	0	0,95	0,018
5	52545	0,05	0	0	0	0,06	0,89	0	0	0	0,07	0	0,93	0	0,048
5	54631	0	0	0,04	0,05	0,08	0,82	0,01	0	0	0,12	0,8	0,08	0	0,042
5	52676	0	0	0,04	0	0,46	0,01	0	0,48	0,01	0,01	0,04	0,48	0,47	0,008

5	21845	0	0	0,04	0	0	0,11	0,05	0	0,8	0,11	0,04	0,05	0,8	0,042
5	100000	0	0	0,97	0	0,01	0	0	0,02	0	0,01	0,99	0	0	0,644
5	39340	0	0,02	0	0,08	0,9	0	0	0	0	0,01	0,08	0,91	0	0,038
5	18819	0,07	0	0,01	0	0,9	0	0	0	0,02	0,09	0,91	0	0	0,048
5	19610	0	0,07	0	0,06	0,86	0	0	0,01	0	0,06	0,86	0	0,08	0,048
5	100000	0	0	1	0	0	0	0	0	0	0	0,72	0	0,28	0,372
5	28925	0	0	0,04	0	0,33	0	0	0,59	0,04	0,92	0,04	0	0,04	0,028
5	100000	0,06	0	0,94	0	0	0	0	0	0	0,16	0,63	0	0,21	0,502
5	35239	0	0,01	0	0,06	0,77	0	0,15	0,01	0	0,91	0	0,07	0,02	0,048
5	27706	0,04	0	0	0	0,95	0,01	0	0	0	0,04	0,01	0	0,95	0,036
5	91481	0	0,04	0	0,07	0,5	0,3	0	0	0,09	0	0,11	0,83	0,06	0,046
5	8077	0,04	0	0	0,03	0,84	0	0	0,02	0,07	0,69	0,01	0,09	0,21	0,048
5	74878	0	0,16	0	0	0,83	0	0	0	0,01	0,17	0,83	0	0	0,046
5	7510	0,05	0	0	0	0	0,95	0	0	0	0,05	0	0	0,95	0,044
5	10732	0	0,24	0	0	0,74	0	0	0,01	0,01	0	0,74	0,23	0,03	0,046
5	82262	0	0	0	0,21	0,79	0	0	0	0	0,79	0,21	0	0	0,044
5	9079	0	0	0	0	0,01	0	0,08	0,51	0,4	0,02	0,07	0,4	0,51	0,048
5	31076	0	0,03	0,04	0	0,89	0	0,04	0	0	0,01	0,02	0,94	0,03	0,044
5	33079	0,01	0,06	0	0	0	0,86	0,05	0	0,02	0,06	0,92	0,02	0	0,04
5	20435	0,02	0	0	0	0,93	0	0,04	0,01	0	0,04	0,01	0,02	0,93	0,046
	37209,19	0,01305	0,06605	0,0684	0,02593	0,45618	0,13503	0,00835	0,16223	0,06479	0,22339	0,24149	0,26718	0,26794	0,072059

scoring

b	time_to_c	(C,C)	(C,D)	(C,P)	(D,C)	(D,D)	(D,P)	(P,C)	(P,D)	(P,P)	S(G,G)	S(G,B)	S(B,G)	S(B,B)	
1	24261	0	0	0	0,28	0,72	0	0	0	0	1	0	0	0	0,036
1	11922	0	0,19	0	0,02	0,7	0	0	0,09	0	0	0,72	0,19	0,09	0,044
1	18889	0,04	0,92	0,03	0	0	0	0	0	0,01	0	0,01	0	0,99	0,044
1	19837	0,05	0	0	0,03	0,27	0,22	0	0,43	0	0,32	0,22	0,38	0,08	0,046
1	7289	0,04	0,01	0,03	0,01	0,91	0	0	0	0	0,01	0,04	0,04	0,91	0,048
1	23942	0,01	0	0	0,16	0,83	0	0	0	0	0,83	0,01	0	0,16	0,044
1	18340	0	0	0,04	0	0,89	0,07	0	0	0	0,85	0,04	0,11	0	0,024
1	5009	0,05	0	0	0	0,92	0,02	0	0	0,01	0	0,01	0	0,99	0,046
1	15919	0,04	0,01	0,02	0	0,93	0	0	0	0	0	0,05	0,94	0,01	0,048
1	18294	0	0	0	0,14	0,86	0	0	0	0	0,14	0	0,86	0	0,048

1	10258	0,04	0	0	0,05	0,91	0	0	0	0	0,94	0,04	0,02	0	0,048
1	20712	0	0	0	0,16	0,64	0,2	0	0	0	0,64	0	0,16	0,2	0,038
1	8713	0,05	0	0,01	0	0,72	0,01	0	0,21	0	0	0	0,39	0,61	0,046
1	3974	0	0,05	0,02	0	0,8	0	0	0,13	0	1	0	0	0	0,044
1	5391	0,04	0	0	0	0,93	0,01	0,02	0	0	0,03	0,93	0,04	0	0,048
1	12198	0	0,3	0	0	0,7	0	0	0	0	0	0	0,3	0,7	0,04
1	2975	0	0	0	0,17	0,73	0,1	0	0	0	0,73	0	0,27	0	0,048
1	14543	0,01	0,98	0	0,01	0	0	0	0	0	0	0,01	0,01	0,98	0,034
1	18668	0	0,5	0	0	0,43	0,07	0	0	0	0,07	0,48	0	0,45	0,042
1	18331	0,01	0,06	0	0,03	0,81	0,07	0,02	0	0	0,82	0	0,15	0,03	0,046
1	27784	0,02	0,04	0	0,02	0,85	0	0,01	0,06	0	0,85	0,04	0,04	0,07	0,038
1	25211	0	0,11	0	0	0,85	0	0,01	0,02	0,01	0	0,01	0,98	0,01	0,04
1	32496	0,03	0	0,01	0,03	0,93	0	0	0	0	0	0,97	0	0,03	0,044
1	10764	0,01	0	0,04	0	0,6	0	0,05	0,13	0,17	0,13	0,04	0,6	0,23	0,046
1	7239	0	0,07	0	0,01	0,91	0	0	0,01	0	0,9	0	0,09	0,01	0,046
1	18951	0	0	0	0,05	0,94	0,01	0	0	0	0	0,01	0,05	0,94	0,042
1	8676	0,05	0,02	0	0	0,9	0,03	0	0	0	0	0,08	0	0,92	0,042
1	15753	0	0	0,02	0,08	0,9	0	0	0	0	0	0,86	0,08	0,06	0,048
1	26245	0	0	0	0,07	0,92	0	0	0	0,01	0,01	0,93	0,06	0	0,048
1	31120	0	0	0	0,07	0,93	0	0	0	0	0	0	0,53	0,47	0,04
1	24495	0	0	0	0,05	0	0	0	0,95	0	0	1	0	0	0,04
1	21155	0	0,16	0	0	0,76	0,05	0	0,03	0	0,35	0,08	0,16	0,41	0,048
1	13939	0,02	0,03	0,04	0	0,91	0	0	0	0	0	0,95	0,05	0	0,036
1	25067	0	0	0,04	0	0,9	0	0	0,02	0,04	0	0,04	0,32	0,64	0,01
1	8957	0,02	0,01	0	0,01	0,89	0,03	0,04	0	0	0,01	0,01	0,22	0,76	0,04
1	11245	0,01	0,96	0	0	0,01	0,01	0,01	0	0	0,01	0,02	0,01	0,96	0,046
1	14072	0	0	0,04	0	0,02	0	0	0,94	0	0	0	1	0	0,028
1	37777	0	0,11	0,04	0	0,84	0	0	0	0,01	0,16	0,84	0	0	0,04
1	8863	0	0,08	0	0	0,89	0,01	0	0,02	0	0,49	0,5	0	0,01	0,046
1	30718	0	0	0	0,07	0,88	0	0	0,05	0	0,05	0,88	0,07	0	0,046
1	19880	0,02	0	0	0,05	0,89	0,04	0	0	0	0	0,53	0,43	0,04	0,04
1	10460	0	0	0	0,11	0,89	0	0	0	0	0,11	0	0,89	0	0,048
1	24219	0,04	0,03	0,03	0	0,89	0	0,01	0	0	0	0,1	0,85	0,05	0,048

1	10590	0,03	0,35	0,01	0	0,01	0,03	0	0,57	0	0	0,01	0,03	0,96	0,044
1	10937	0	0,06	0,02	0,01	0,91	0	0	0	0	0,91	0	0,02	0,07	0,048
1	9894	0,01	0	0	0,05	0	0,77	0,01	0,15	0,01	0,18	0,02	0,02	0,78	0,042
1	24967	0	0,14	0	0	0,86	0	0	0	0	0	0	1	0	0,048
1	31839	0,01	0,61	0,03	0,03	0,32	0	0	0	0	0	0,06	0,01	0,93	0,048
1	18627	0,06	0,04	0	0	0,15	0	0	0,74	0,01	0,07	0,23	0,06	0,64	0,046
1	11815	0,04	0	0,02	0	0,05	0,89	0	0	0	0,89	0,02	0,09	0	0,04
1	13555	0,04	0	0	0	0,96	0	0	0	0	0	0,04	0	0,96	0,048
1	9779	0	0	0,01	0,08	0,9	0	0	0	0,01	0,03	0,03	0,93	0,01	0,048
1	12570	0,03	0	0,03	0	0,88	0	0	0,06	0	0,88	0,07	0	0,05	0,048
1	4293	0	0	0	0,1	0,9	0	0	0	0	0	0	0,9	0,1	0,046
1	10587	0	0	0,01	0,17	0,81	0	0	0,01	0	0,8	0,01	0,02	0,17	0,048
1	29534	0	0,12	0	0,03	0,85	0	0	0	0	0	0,85	0,15	0	0,044
1	40956	0	0	0	0,2	0	0	0	0,77	0,03	0,77	0	0	0,23	0,046
1	26280	0,05	0	0	0	0,82	0,11	0,02	0	0	0,02	0,85	0,03	0,1	0,048
1	5140	0,01	0,24	0	0	0,75	0	0	0	0	0,24	0	0,01	0,75	0,048
1	14287	0,02	0,84	0	0	0,14	0	0	0	0	0	0,68	0	0,32	0,046
1	3859	0	0,97	0	0	0	0	0	0,03	0	0,03	0	0,03	0,94	0,042
1	23332	0	0,95	0,04	0	0	0	0,01	0	0	0,01	0,04	0	0,95	0,026
1	10610	0,05	0	0,01	0	0,93	0,01	0	0	0	0	0,94	0,05	0,01	0,04
1	14991	0,04	0,01	0	0	0,01	0,85	0,02	0,07	0	0,04	0,01	0,01	0,94	0,042
1	12588	0	0,04	0	0,13	0,82	0	0	0,01	0	0,86	0,01	0	0,13	0,036
1	10254	0	0	0	0,13	0,85	0,02	0	0	0	0,02	0,01	0,84	0,13	0,048
1	44007	0,05	0	0	0	0,94	0	0,01	0	0	0,94	0	0,06	0	0,046
1	11535	0,06	0	0	0	0	0,94	0	0	0	1	0	0	0	0,044
1	5532	0	0	0,02	0,06	0,64	0	0	0,28	0	0,02	0,64	0	0,34	0,046
1	17214	0,05	0,09	0,01	0	0,85	0	0	0	0	0,14	0	0,01	0,85	0,046
1	14785	0	0,56	0,03	0,02	0,38	0	0	0	0,01	0,06	0,04	0,02	0,88	0,046
1	7048	0,02	0	0	0,03	0,94	0	0	0,01	0	0,04	0,02	0,12	0,82	0,034
1	3455	0	0,04	0	0,1	0,84	0,01	0,01	0	0	0,85	0,04	0,11	0	0,044
1	10039	0	0,22	0,04	0	0,74	0	0	0	0	0	0,02	0,24	0,74	0,042
1	6450	0,01	0,1	0,02	0	0,86	0	0	0,01	0	0	0,02	0,96	0,02	0,044
1	7986	0	0	0,01	0,06	0,91	0,02	0	0	0	0	0,01	0,08	0,91	0,044

1	14607	0	0	0	0,08	0,92	0	0	0	0	0,06	0,94	0	0	0,048
1	5968	0	0	0	0,21	0,78	0,01	0	0	0	0,91	0,05	0,02	0,02	0,042
1	10057	0	0	0	0,18	0,74	0	0	0,08	0	0,78	0	0	0,22	0,04
1	11205	0,06	0	0,04	0	0	0	0	0,9	0	0,02	0,03	0	0,95	0,046
1	11880	0	0	0	0,09	0,91	0	0	0	0	0,42	0	0	0,58	0,048
1	11384	0	0,07	0	0,07	0,83	0,03	0	0	0	0,14	0,83	0,03	0	0,048
1	13875	0,01	0,15	0,02	0	0,81	0	0	0	0,01	0,17	0,81	0	0,02	0,042
1	6233	0,05	0	0	0	0,93	0	0	0	0,02	0,83	0	0	0,17	0,044
1	41822	0	0	0	0,15	0	0,03	0	0,82	0	0,56	0,29	0	0,15	0,048
1	10371	0	0	0	0,06	0,93	0	0	0	0,01	0,06	0	0	0,94	0,048
1	13739	0	0,12	0	0	0,55	0,21	0	0,12	0	0,67	0,03	0	0,3	0,04
1	12483	0,03	0	0,04	0,01	0,92	0	0	0	0	0,01	0,04	0,91	0,04	0,044
1	13798	0	0,98	0	0,02	0	0	0	0	0	0	0	0,02	0,98	0,048
1	11150	0	0	0	0,08	0,44	0,02	0	0,46	0	0,05	0	0,1	0,85	0,046
1	20818	0,01	0,1	0	0	0,75	0,03	0	0,11	0	0,36	0,53	0,1	0,01	0,048
1	24044	0	0,89	0	0,01	0,01	0	0	0,09	0	0,01	0,89	0	0,1	0,048
1	7286	0,05	0	0,01	0	0,94	0	0	0	0	0	0,01	0,94	0,05	0,04
1	23751	0,04	0	0	0,07	0,83	0,02	0	0,04	0	0,76	0,18	0	0,06	0,048
1	17108	0	0,28	0	0	0,7	0	0	0,02	0	0,28	0,02	0	0,7	0,046
1	13501	0,06	0	0	0	0,1	0,84	0	0	0	0,99	0,01	0	0	0,044
1	10541	0,04	0	0,02	0	0,9	0	0,01	0	0,03	0,91	0,02	0,03	0,04	0,046
1	31066	0	0	0	0,14	0,33	0,22	0	0,27	0,04	0,26	0,34	0,26	0,14	0,046
1	11393	0,06	0	0	0	0,93	0,01	0	0	0	0,02	0,88	0,1	0	0,048
1	23405	0	0,29	0	0,03	0,59	0	0	0,08	0,01	0,03	0,34	0,04	0,59	0,044
1,25	20882	0	0	0	0,06	0,68	0,02	0	0,24	0	0	0	0,3	0,7	0,042
1,25	28322	0,02	0	0	0,03	0,95	0	0	0	0	0	0	0,05	0,95	0,048
1,25	10075	0	0	0	0,05	0,92	0,01	0	0,02	0	0	0,02	0,05	0,93	0,048
1,25	22038	0	0	0,04	0	0	0	0	0,96	0	0	0,04	0,96	0	0,02
1,25	37890	0	0,09	0	0,04	0,62	0	0	0,22	0,03	0,22	0,71	0,06	0,01	0,048
1,25	31373	0	0,11	0,01	0	0,87	0	0,01	0	0	0,03	0,09	0,88	0	0,042
1,25	23584	0	0,08	0	0	0	0,92	0	0	0	0,86	0	0,06	0,08	0,048
1,25	17918	0,03	0	0,01	0	0,01	0,74	0,01	0,19	0,01	0,01	0,2	0,76	0,03	0,036
1,25	10056	0	0	0,04	0,11	0,81	0	0	0	0,04	0,78	0,04	0,07	0,11	0,036

1,25	5013	0,03	0,03	0	0,02	0,91	0	0	0	0,01	0,93	0,02	0,02	0,03	0,046
1,25	12915	0,01	0	0	0,07	0	0	0,05	0,87	0	0,12	0,5	0,38	0	0,046
1,25	13101	0	0	0,01	0,17	0,82	0	0	0	0	0,82	0,01	0	0,17	0,046
1,25	15142	0	0,17	0	0,03	0,8	0	0	0	0	0,11	0,01	0,19	0,69	0,046
1,25	16796	0	0,31	0,01	0	0,64	0,02	0	0,02	0	0,1	0,02	0,23	0,65	0,048
1,25	58495	0	1	0	0	0	0	0	0	0	0	1	0	0	0,04
1,25	8717	0	0,2	0	0	0,79	0	0,01	0	0	0,01	0,79	0,2	0	0,044
1,25	6762	0	0,94	0,03	0,03	0	0	0	0	0	0	0,06	0	0,94	0,034
1,25	8805	0,03	0	0	0,01	0,94	0	0,02	0	0	0,03	0	0,95	0,02	0,048
1,25	23318	0,01	0,01	0	0,07	0,9	0	0,01	0	0	0,07	0,07	0,85	0,01	0,048
1,25	10320	0,07	0	0,02	0	0,83	0	0	0,08	0	0	0,1	0	0,9	0,048
1,25	31888	0	0	0	0,03	0,94	0,01	0,02	0	0	0,01	0	0,01	0,98	0,046
1,25	11523	0	0,12	0	0	0	0	0	0,88	0	0,02	0	0,98	0	0,048
1,25	15642	0	0,11	0	0,02	0,85	0,02	0	0	0	0,11	0,04	0,85	0	0,046
1,25	3951	0	0,11	0,02	0	0,79	0	0	0,08	0	0	0,11	0,89	0	0,048
1,25	11589	0,03	0	0	0,07	0,83	0	0	0,07	0	0,83	0	0,03	0,14	0,042
1,25	7473	0	0,3	0	0	0,7	0	0	0	0	0	0,05	0,25	0,7	0,044
1,25	30511	0	0	0	0,06	0,94	0	0	0	0	0,06	0	0	0,94	0,044
1,25	7293	0,02	0,74	0	0	0,24	0	0	0	0	0	0,74	0,01	0,25	0,048
1,25	11387	0,01	0,17	0,04	0,01	0,75	0	0	0,02	0	0,18	0,05	0,03	0,74	0,044
1,25	20634	0,06	0	0	0	0,91	0	0	0,03	0	0,06	0,03	0	0,91	0,048
1,25	2616	0,03	0	0,04	0,01	0,65	0,05	0	0,19	0,03	0,01	0,71	0,27	0,01	0,04
1,25	14818	0	0,1	0	0	0,53	0	0	0,37	0	0,54	0	0,42	0,04	0,044
1,25	39888	0	0	0	0,06	0,94	0	0	0	0	0,06	0	0	0,94	0,048
1,25	19795	0,05	0	0,01	0	0	0	0	0,94	0	0	0,01	0,02	0,97	0,046
1,25	6087	0,01	0,18	0	0,02	0,72	0,07	0	0	0	0,04	0	0,24	0,72	0,048
1,25	19877	0,06	0	0	0	0,6	0,33	0,01	0	0	0	0,93	0,06	0,01	0,048
1,25	15066	0,03	0	0,04	0	0,83	0	0	0,1	0	0,56	0,1	0	0,34	0,048
1,25	13764	0	0,07	0	0	0,93	0	0	0	0	0,93	0,07	0	0	0,048
1,25	9407	0,01	0,19	0	0,02	0	0,04	0,01	0,73	0	0	0,35	0,09	0,56	0,048
1,25	9296	0,06	0	0	0	0,88	0	0,01	0	0,05	0,88	0,01	0,06	0,05	0,048
1,25	18535	0,02	0,08	0	0	0,86	0,02	0,02	0	0	0,1	0,86	0,04	0	0,048
1,25	15812	0	0,16	0	0	0,1	0,02	0	0,7	0,02	0,04	0	0,78	0,18	0,046

1,25	54146	0	0	0,04	0	0,94	0,01	0,01	0	0	0,02	0	0	0,98	0,01
1,25	11640	0,05	0	0	0	0,91	0,04	0	0	0	0,05	0	0,95	0	0,046
1,25	7726	0	0,11	0	0,03	0,81	0	0	0,05	0	0,05	0,03	0,8	0,12	0,048
1,25	11381	0	0,63	0	0	0	0	0	0,37	0	0	0,53	0,04	0,43	0,034
1,25	17968	0,01	0,04	0,02	0,06	0,73	0,14	0	0	0	0,77	0	0,15	0,08	0,046
1,25	16423	0	0	0	0,23	0,76	0	0	0,01	0	0,76	0	0,23	0,01	0,048
1,25	12919	0	0	0,04	0	0,25	0,07	0,07	0,57	0	0,61	0,04	0,03	0,32	0,04
1,25	5824	0,04	0	0,01	0,05	0,86	0,01	0	0,03	0	0,84	0,05	0,1	0,01	0,048
1,25	20151	0,01	0	0	0,06	0,91	0	0	0	0,02	0,03	0,05	0,04	0,88	0,046
1,25	23809	0,02	0,05	0	0	0,92	0	0,01	0	0	0,94	0,03	0	0,03	0,048
1,25	30174	0	0,25	0	0	0,74	0	0	0,01	0	0,1	0	0,16	0,74	0,04
1,25	15344	0,06	0	0	0	0	0	0	0,92	0,02	0	0	0,92	0,08	0,046
1,25	8505	0	0,85	0,03	0	0,03	0,04	0,01	0,04	0	0,03	0,09	0,01	0,87	0,048
1,25	7053	0	0,16	0	0	0,7	0,07	0	0,07	0	0	0	0,93	0,07	0,044
1,25	10449	0,05	0,01	0	0	0,93	0	0	0,01	0	0,36	0,63	0,01	0	0,046
1,25	38721	0	0	0	0,17	0,65	0,14	0,02	0	0,02	0,81	0,02	0,14	0,03	0,044
1,25	17254	0	0,96	0,02	0	0	0,02	0	0	0	0,02	0	0,02	0,96	0,048
1,25	13367	0,01	0,3	0	0	0,68	0,01	0	0	0	0	0	0,18	0,82	0,048
1,25	3998	0,06	0	0	0	0,94	0	0	0	0	0,94	0	0	0,06	0,046
1,25	12720	0	0,96	0,04	0	0	0	0	0	0	0	0,04	0	0,96	0,016
1,25	10907	0	0,13	0	0,04	0,83	0	0	0	0	0,06	0	0,13	0,81	0,036
1,25	4145	0,04	0,29	0,04	0	0,63	0	0	0	0	0,04	0,29	0	0,67	0,046
1,25	6882	0,04	0	0,02	0	0,93	0	0,01	0	0	0,92	0,03	0,03	0,02	0,044
1,25	26992	0,01	0,25	0	0	0,74	0	0	0	0	0,14	0	0,25	0,61	0,046
1,25	23889	0,02	0	0	0,04	0,91	0	0	0,03	0	0,09	0	0,01	0,9	0,048
1,25	14430	0,01	0,09	0	0,04	0,86	0	0	0	0	0,1	0,58	0,03	0,29	0,04
1,25	27079	0	0,23	0	0	0,77	0	0	0	0	0	0,77	0,17	0,06	0,046
1,25	34414	0	0,13	0	0	0,33	0,51	0	0,03	0	0,03	0	0,97	0	0,038
1,25	18612	0,04	0,01	0,01	0	0,93	0	0	0	0,01	0,5	0,04	0,01	0,45	0,046
1,25	37747	0	0,24	0	0,01	0,73	0,02	0	0	0	0,01	0	0,26	0,73	0,04
1,25	5523	0	0,01	0	0,18	0,7	0	0	0,11	0	0,66	0,16	0,17	0,01	0,046
1,25	83999	0,01	0	0,02	0,06	0,57	0	0	0,34	0	0,34	0,58	0,02	0,06	0,038
1,25	4687	0,04	0	0,03	0	0,91	0,01	0,01	0	0	0,05	0,07	0,44	0,44	0,046

1,25	30356	0,05	0	0,02	0	0	0,93	0	0	0	0	0,13	0,82	0,05	0,048
1,25	13173	0,05	0	0	0	0,95	0	0	0	0	0,05	0	0	0,95	0,036
1,25	22024	0	0	0	0,23	0,77	0	0	0	0	0,77	0	0,23	0	0,048
1,25	15398	0,04	0,04	0	0	0,83	0	0	0,08	0,01	0,83	0,06	0,04	0,07	0,046
1,25	33070	0	0	0	0,1	0,89	0,01	0	0	0	0,25	0,74	0	0,01	0,044
1,25	29426	0	0	0,04	0	0,9	0	0,01	0,05	0	0	0,03	0,68	0,29	0,02
1,25	16291	0	0,12	0	0,01	0,87	0	0	0	0	0	0,13	0,87	0	0,048
1,25	10985	0	0	0	0,29	0,67	0,04	0	0	0	0,96	0,04	0	0	0,044
1,25	16711	0	0	0	0,33	0	0	0	0,67	0	0,84	0,16	0	0	0,038
1,25	7603	0	0,97	0	0,02	0	0	0	0	0,01	0	0,01	0,02	0,97	0,04
1,25	6966	0,02	0,06	0,01	0	0	0	0,04	0,87	0	0	0,02	0,11	0,87	0,048
1,25	24118	0	0	0	0,24	0,74	0	0	0,02	0	0,73	0,24	0,02	0,01	0,046
1,25	35737	0,05	0	0	0	0,49	0	0,01	0,45	0	0	0,49	0,01	0,5	0,048
1,25	50154	0	0,31	0	0	0,68	0	0	0	0,01	0,07	0,07	0,25	0,61	0,048
1,25	29442	0	0	0	0,07	0	0	0	0,91	0,02	0	0	0	1	0,048
1,25	12473	0,06	0	0,02	0	0,92	0	0	0	0	0,01	0,02	0,05	0,92	0,044
1,25	13220	0,07	0	0	0	0,91	0	0,01	0	0,01	0	0,01	0,91	0,08	0,048
1,25	38389	0,01	0,93	0	0	0,06	0	0	0	0	0,06	0,01	0	0,93	0,04
1,25	8929	0,06	0	0	0	0,87	0,05	0	0,02	0	0,02	0,87	0,05	0,06	0,044
1,25	9698	0	0,14	0,01	0	0,82	0	0	0,03	0	0,02	0	0,97	0,01	0,046
1,25	24796	0,05	0,01	0	0	0,86	0	0	0,08	0	0	0,95	0	0,05	0,048
1,25	24199	0	0	0	0,14	0,86	0	0	0	0	0,05	0,13	0,82	0	0,048
1,25	36647	0	0,03	0	0,1	0,87	0	0	0	0	0,04	0	0,78	0,18	0,046
1,25	14575	0	0,16	0	0,02	0,81	0	0	0	0,01	0,16	0,81	0,01	0,02	0,04
1,25	14069	0	0	0	0,06	0,94	0	0	0	0	0,06	0,87	0	0,07	0,042
1,5	21994	0,02	0,01	0,03	0	0,07	0,81	0,06	0	0	0,81	0,03	0,08	0,08	0,048
1,5	5463	0,01	0,2	0,01	0	0,75	0	0	0	0,03	0	0,77	0,19	0,04	0,046
1,5	7552	0	0,93	0,04	0	0	0,02	0	0,01	0	0	0,02	0,05	0,93	0,046
1,5	29084	0	0,11	0	0	0,87	0,01	0	0,01	0	0,61	0,27	0,12	0	0,046
1,5	11734	0,05	0	0	0,03	0,85	0,07	0	0	0	0,29	0,05	0,63	0,03	0,046
1,5	23220	0,05	0	0	0	0,86	0	0	0,09	0	0,86	0,05	0	0,09	0,044
1,5	36017	0	0,13	0	0	0,87	0	0	0	0	0	0	1	0	0,042
1,5	21038	0	0	0	0,19	0,8	0	0,01	0	0	0,81	0	0,19	0	0,048

1,5	25706	0	0	0,04	0	0,55	0,39	0,02	0	0	0,06	0	0,94	0	0,022
1,5	14022	0,05	0	0	0	0,31	0,58	0	0,06	0	0	0,31	0,04	0,65	0,048
1,5	41501	0,05	0	0	0	0,91	0	0	0,04	0	0,04	0,01	0,95	0	0,028
1,5	13398	0,01	0	0,04	0,01	0,94	0	0	0	0	0	0,98	0,01	0,01	0,028
1,5	4501	0	0,08	0	0	0	0,91	0	0	0,01	0,91	0	0,09	0	0,044
1,5	26447	0	0,07	0	0	0,92	0	0	0,01	0	0,92	0	0,07	0,01	0,048
1,5	27515	0,02	0	0,04	0	0,21	0,44	0	0,29	0	0,27	0,46	0,24	0,03	0,044
1,5	8555	0	0,09	0	0,06	0,85	0	0	0	0	0	0,85	0,15	0	0,048
1,5	13310	0	0	0	0,08	0,9	0	0	0,02	0	0,1	0,89	0,01	0	0,044
1,5	32220	0,02	0,09	0	0	0,84	0	0	0,01	0,04	0,09	0	0,91	0	0,044
1,5	4668	0,06	0	0	0	0	0	0	0,93	0,01	0	0	0	1	0,048
1,5	10601	0,06	0	0,02	0	0	0	0	0,92	0	0	0,91	0,09	0	0,046
1,5	40340	0,07	0	0	0	0,93	0	0	0	0	0,93	0,07	0	0	0,046
1,5	7342	0,05	0	0	0	0	0	0	0,95	0	0	0	0,97	0,03	0,042
1,5	8076	0,04	0,08	0,01	0	0,87	0	0	0	0	0,08	0,89	0	0,03	0,046
1,5	25245	0,01	0	0,03	0	0,89	0,01	0,06	0	0	0	0,04	0,91	0,05	0,042
1,5	22162	0	0,06	0	0,08	0,8	0,02	0	0,04	0	0,1	0,8	0	0,1	0,048
1,5	52337	0	0,99	0	0	0,01	0	0	0	0	0	0,99	0	0,01	0,046
1,5	8372	0,04	0	0	0	0,94	0	0,02	0	0	0	0,94	0,02	0,04	0,044
1,5	9346	0,05	0	0,04	0	0,86	0	0	0,05	0	0,05	0,9	0,05	0	0,046
1,5	10531	0,05	0	0	0	0,93	0	0	0	0,02	0,02	0,05	0,08	0,85	0,048
1,5	29452	0	0	0	0,23	0,75	0	0	0,02	0	0,76	0,12	0,1	0,02	0,046
1,5	5292	0,04	0,89	0	0	0,07	0	0	0	0	0	0,04	0	0,96	0,046
1,5	5054	0,05	0,01	0	0	0,9	0,02	0	0,02	0	0,88	0	0,06	0,06	0,046
1,5	9978	0,04	0	0,01	0	0,95	0	0	0	0	0,02	0,02	0,96	0	0,048
1,5	18179	0	0,08	0	0,05	0,87	0	0	0	0	0,08	0,87	0,03	0,02	0,048
1,5	17140	0,01	0	0	0,19	0,77	0,03	0	0	0	0,77	0	0,19	0,04	0,048
1,5	36368	0	0,01	0	0,09	0	0,11	0	0,76	0,03	0,12	0,77	0,11	0	0,048
1,5	7371	0	0	0	0,1	0	0,01	0	0,87	0,02	0,22	0,67	0,01	0,1	0,044
1,5	18050	0,01	0,96	0	0	0	0	0	0,03	0	0	0,01	0,03	0,96	0,042
1,5	8145	0	0	0,04	0	0	0,94	0,02	0	0	0,02	0	0,98	0	0,036
1,5	14478	0,01	0,2	0	0,02	0,76	0	0	0	0,01	0,2	0,01	0	0,79	0,046
1,5	41316	0	0	0,04	0	0,91	0	0	0	0,05	0,95	0	0,05	0	0,042

1,5	14337	0,04	0	0,02	0	0,94	0	0	0	0	0,94	0,05	0	0,01	0,048
1,5	10569	0,02	0	0	0,04	0,88	0,02	0	0	0,04	0,1	0	0,02	0,88	0,044
1,5	20991	0	0,08	0	0,06	0,81	0	0	0,05	0	0	0	0,94	0,06	0,038
1,5	9453	0	0,03	0,04	0	0	0	0	0,93	0	0,03	0,04	0,93	0	0,028
1,5	6563	0	0	0	0,19	0,79	0,02	0	0	0	0,74	0	0,19	0,07	0,044
1,5	6814	0	0,2	0	0	0,8	0	0	0	0	0,18	0,8	0	0,02	0,042
1,5	10362	0	0	0	0,06	0,92	0,02	0	0	0	0	0,02	0,13	0,85	0,048
1,5	8224	0,03	0,03	0,03	0	0,81	0	0	0,1	0	0,2	0,03	0,77	0	0,048
1,5	22229	0	0	0	0,24	0,75	0	0	0	0,01	0,75	0,24	0,01	0	0,048
1,5	15376	0	0,1	0,04	0,01	0,84	0	0	0,01	0	0,01	0,05	0,1	0,84	0,028
1,5	22847	0,04	0	0	0	0,15	0	0,01	0,8	0	0	0,22	0,05	0,73	0,042
1,5	17079	0,05	0	0	0,06	0,01	0	0	0,88	0	0,88	0	0,07	0,05	0,046
1,5	18112	0,04	0,02	0,01	0	0,93	0	0	0	0	0,02	0,04	0,94	0	0,048
1,5	8220	0	0,08	0	0	0,92	0	0	0	0	0,92	0	0	0,08	0,046
1,5	9906	0,02	0,96	0,02	0	0	0	0	0	0	0,02	0,04	0	0,94	0,048
1,5	22320	0	0,98	0	0	0,01	0	0	0,01	0	0	0,96	0	0,04	0,022
1,5	12758	0	0,09	0	0,02	0,88	0	0	0,01	0	0	0,01	0,98	0,01	0,046
1,5	24111	0	0,13	0	0,03	0,82	0,02	0	0	0	0	0,82	0,15	0,03	0,038
1,5	26308	0	0,06	0,01	0,08	0,85	0	0	0	0	0,01	0,06	0,93	0	0,048
1,5	16032	0,05	0,02	0	0	0,9	0,01	0	0,01	0,01	0,02	0,42	0,54	0,02	0,048
1,5	8074	0	0,26	0	0,02	0	0,01	0	0,7	0,01	0,02	0,71	0,04	0,23	0,046
1,5	21026	0,04	0	0,03	0	0	0,25	0	0,62	0,06	0,04	0,09	0,87	0	0,048
1,5	4820	0,05	0	0,01	0	0	0,94	0	0	0	0,98	0,01	0	0,01	0,044
1,5	9690	0,04	0	0,04	0	0,91	0	0	0,01	0	0,01	0,08	0,91	0	0,046
1,5	14216	0,06	0	0,01	0	0	0	0	0,93	0	0	0	0,94	0,06	0,048
1,5	8372	0,04	0	0	0,02	0,88	0,05	0	0	0,01	0	0,05	0,88	0,07	0,048
1,5	8674	0,05	0	0	0	0,33	0,62	0	0	0	0,33	0	0,62	0,05	0,044
1,5	10073	0,03	0,12	0	0	0,85	0	0	0	0	0,12	0,85	0	0,03	0,048
1,5	9054	0,05	0	0,04	0,02	0,84	0,04	0	0,01	0	0	0,1	0,85	0,05	0,048
1,5	12257	0,01	0	0	0	0	0,72	0,09	0,18	0	0	0,01	0,99	0	0,048
1,5	9017	0,06	0	0	0	0,94	0	0	0	0	0,02	0,94	0,04	0	0,048
1,5	23142	0,01	0	0,01	0,14	0,78	0,01	0	0,05	0	0,82	0,16	0,02	0	0,046
1,5	24810	0,05	0	0	0,01	0,93	0	0	0,01	0	0,93	0,05	0,01	0,01	0,048

1,5	16774	0	0,09	0,04	0	0,85	0	0	0,02	0	0,15	0,02	0,19	0,64	0,028
1,5	11015	0,01	0	0,01	0,06	0,91	0	0	0	0,01	0,07	0,91	0,01	0,01	0,044
1,5	14540	0	0,12	0	0	0,88	0	0	0	0	0,12	0	0,88	0	0,046
1,5	4221	0,03	0	0,04	0	0,91	0	0	0,02	0	0,02	0	0,06	0,92	0,028
1,5	13826	0,05	0	0,04	0	0,91	0	0	0	0	0,05	0,04	0,91	0	0,048
1,5	31475	0	0,05	0	0,07	0,87	0	0,01	0	0	0,76	0,03	0,13	0,08	0,044
1,5	44891	0	0	0	0,09	0,9	0,01	0	0	0	0,07	0,63	0,3	0	0,046
1,5	10237	0,01	0	0,02	0,05	0,18	0	0	0,74	0	0	0,02	0,19	0,79	0,048
1,5	16255	0	0,11	0	0	0,66	0,23	0	0	0	0,41	0,37	0,22	0	0,044
1,5	21740	0,05	0	0	0,01	0,91	0,03	0	0	0	0,94	0	0,01	0,05	0,046
1,5	12763	0	0	0	0,21	0,79	0	0	0	0	0,76	0,03	0,21	0	0,046
1,5	17006	0	0	0	0,06	0,81	0	0	0,13	0	0,05	0	0,01	0,94	0,046
1,5	4433	0,06	0	0,01	0	0,9	0,01	0	0	0,02	0,01	0,98	0	0,01	0,044
1,5	19362	0	0,12	0	0	0	0,66	0	0,22	0	0,18	0,05	0,58	0,19	0,048
1,5	7572	0,05	0	0,02	0	0,92	0	0	0	0,01	0,06	0,89	0	0,05	0,046
1,5	20678	0,06	0	0	0	0,92	0,02	0	0	0	0,02	0	0,01	0,97	0,046
1,5	32442	0,04	0	0	0	0,47	0,44	0,05	0	0	0,09	0,31	0,6	0	0,046
1,5	33898	0	0,25	0,03	0	0,7	0	0,01	0,01	0	0,04	0	0,26	0,7	0,04
1,5	10067	0	0	0,01	0,22	0,74	0	0	0,03	0	0,78	0,01	0,21	0	0,048
1,5	13660	0	0,91	0	0	0	0,09	0	0	0	0,07	0,02	0	0,91	0,048
1,5	34082	0,05	0	0,04	0	0,89	0,01	0	0	0,01	0,03	0,04	0,03	0,9	0,042
1,5	50320	0	0,24	0	0	0,76	0	0	0	0	0	0,4	0,24	0,36	0,044
1,5	6383	0	0	0,04	0,03	0,91	0	0,02	0	0	0,03	0,06	0,01	0,9	0,044
1,5	16395	0,06	0	0	0	0,89	0	0	0	0,05	0	0,43	0,06	0,51	0,048
1,5	3787	0	0,05	0	0,05	0,9	0	0	0	0	0,9	0	0,05	0,05	0,048
1,5	11502	0,06	0	0,01	0	0,01	0,88	0	0,02	0,02	0	0	0,96	0,04	0,048
1,75	18171	0	0,19	0	0	0,81	0	0	0	0	0	0,77	0,23	0	0,044
1,75	8837	0,05	0,09	0,03	0	0,73	0,1	0	0	0	0	0,17	0,05	0,78	0,048
1,75	11906	0	0,13	0,03	0,03	0,8	0	0,01	0	0	0,03	0,83	0	0,14	0,046
1,75	13122	0,04	0,02	0	0	0,9	0,04	0	0	0	0,02	0,08	0,9	0	0,042
1,75	6826	0	0	0	0,05	0	0,01	0	0,87	0,07	0,01	0,04	0,03	0,92	0,048
1,75	33550	0,01	0	0,04	0,05	0,54	0	0	0,36	0	0,35	0	0,59	0,06	0,044
1,75	9361	0	0	0,04	0,09	0	0,02	0	0,85	0	0,11	0,04	0,85	0	0,046

1,75	15543	0	0,25	0	0,01	0,74	0	0	0	0	0,26	0	0	0,74	0,046
1,75	19832	0	0,12	0,01	0	0,87	0	0	0	0	0	0,04	0,87	0,09	0,048
1,75	14749	0	0,97	0,02	0,01	0	0	0	0	0	0,02	0,12	0,01	0,85	0,048
1,75	26493	0,06	0	0	0,01	0,93	0	0	0	0	0	0	0,94	0,06	0,046
1,75	9037	0	0,29	0	0	0,7	0,01	0	0	0	0	0,01	0,29	0,7	0,046
1,75	19546	0	1	0	0	0	0	0	0	0	0	1	0	0	0,04
1,75	20946	0	0,01	0,01	0,12	0,45	0,41	0	0	0	0,32	0,45	0,11	0,12	0,048
1,75	21666	0	0	0	0,18	0,82	0	0	0	0	0,82	0,04	0,14	0	0,048
1,75	6499	0,05	0	0	0,01	0,82	0	0	0,12	0	0	0	0,01	0,99	0,048
1,75	15746	0,06	0,01	0	0	0	0,92	0	0,01	0	0,94	0,04	0	0,02	0,046
1,75	20393	0	0,91	0	0,03	0,01	0,05	0	0	0	0,01	0	0	0,99	0,048
1,75	25592	0	0	0	0,11	0,89	0	0	0	0	0	0	1	0	0,046
1,75	6133	0,01	0	0	0	0,95	0	0,04	0	0	0	0,88	0,04	0,08	0,048
1,75	13279	0,06	0,01	0	0	0,93	0	0	0	0	0	0,94	0	0,06	0,038
1,75	8689	0,04	0	0,02	0	0,39	0,12	0	0,43	0	0,43	0,4	0	0,17	0,04
1,75	11706	0	0	0	0,11	0,25	0,35	0	0,29	0	0,45	0,35	0	0,2	0,048
1,75	26674	0,05	0	0,01	0	0,94	0	0	0	0	0	0,96	0,04	0	0,038
1,75	6382	0,05	0,01	0,01	0	0,93	0	0	0	0	0,01	0,06	0,93	0	0,048
1,75	33352	0	0,08	0,04	0	0,85	0,03	0	0	0	0	0,11	0,01	0,88	0,004
1,75	16333	0,03	0,03	0	0,03	0,9	0	0	0,01	0	0	0,04	0,91	0,05	0,048
1,75	6924	0,01	0,21	0,03	0	0,74	0	0	0	0,01	0,23	0,03	0,01	0,73	0,046
1,75	24588	0,01	0	0	0,07	0,92	0	0	0	0	0,01	0,44	0,55	0	0,04
1,75	16451	0	0	0	0,12	0,82	0,06	0	0	0	0,13	0	0,87	0	0,048
1,75	30848	0	0	0	0,21	0,65	0,13	0,01	0	0	0,65	0,21	0,13	0,01	0,048
1,75	32259	0,01	0,1	0	0	0,83	0	0,02	0,04	0	0,12	0,86	0,01	0,01	0,04
1,75	7220	0	0	0	0,08	0,91	0,01	0	0	0	0,08	0,91	0	0,01	0,048
1,75	9581	0,05	0	0	0,01	0,9	0,04	0	0	0	0,9	0,04	0,01	0,05	0,044
1,75	31456	0	0,12	0	0	0,88	0	0	0	0	0	0	1	0	0,046
1,75	23666	0,06	0	0	0	0	0,02	0	0,89	0,03	0,03	0,06	0,91	0	0,046
1,75	8118	0,02	0,96	0	0	0	0	0,02	0	0	0	0,02	0,01	0,97	0,048
1,75	11754	0	0	0	0,05	0	0	0	0,95	0	0	0,52	0,05	0,43	0,046
1,75	24903	0	0	0	0,2	0,58	0,04	0	0,18	0	0,58	0	0,42	0	0,046
1,75	5004	0	0,95	0,04	0	0,01	0	0	0	0	0	0,04	0	0,96	0,01

1,75	10111	0	0	0,01	0,04	0	0	0,02	0,93	0	0,04	0	0,01	0,95	0,046
1,75	14456	0,05	0	0,02	0	0,92	0	0	0	0,01	0	0,94	0,01	0,05	0,046
1,75	29169	0	0,21	0	0	0,79	0	0	0	0	0	0,79	0,21	0	0,044
1,75	16333	0,02	0,14	0,04	0	0	0,02	0	0,78	0	0	0,18	0,23	0,59	0,034
1,75	7235	0	0	0,04	0	0,92	0	0	0,01	0,03	0,93	0	0,04	0,03	0,04
1,75	7809	0,04	0	0,03	0	0	0	0,01	0,89	0,03	0,68	0,24	0	0,08	0,048
1,75	40705	0,03	0,39	0	0,01	0,57	0	0	0	0	0,04	0	0,01	0,95	0,048
1,75	29696	0,03	0	0	0,02	0,95	0	0	0	0	0,03	0	0,01	0,96	0,048
1,75	12409	0,03	0	0,03	0	0,94	0	0	0	0	0,94	0,03	0,03	0	0,048
1,75	20548	0,05	0	0	0	0,88	0	0	0,07	0	0	0,07	0,05	0,88	0,046
1,75	13936	0,06	0	0,04	0	0,27	0	0,01	0,62	0	0	0,66	0	0,34	0,042
1,75	6697	0	0	0	0,19	0,71	0,1	0	0	0	0,71	0,19	0,1	0	0,038
1,75	33701	0	0	0	0,07	0,82	0	0	0,11	0	0,11	0,82	0	0,07	0,044
1,75	16754	0,05	0	0,02	0,01	0,91	0,01	0	0	0	0,08	0,01	0	0,91	0,04
1,75	12139	0	0,1	0	0,04	0,86	0	0	0	0	0	0,38	0,62	0	0,048
1,75	18112	0	0,02	0	0,21	0,77	0	0	0	0	0,98	0,01	0	0,01	0,048
1,75	32446	0	0	0	0,05	0,91	0	0,01	0,03	0	0	0,91	0,08	0,01	0,042
1,75	14337	0	0	0,04	0,15	0	0,01	0	0,8	0	0,82	0,03	0	0,15	0,048
1,75	14993	0,02	0,03	0,03	0	0,75	0	0	0,17	0	0,51	0,02	0,43	0,04	0,042
1,75	32645	0,03	0,22	0	0	0,75	0	0	0	0	0,03	0,75	0	0,22	0,046
1,75	8536	0,05	0	0	0	0,94	0	0	0	0,01	0,58	0	0,42	0	0,046
1,75	12100	0,05	0	0	0,01	0,93	0	0	0	0,01	0	0	0,02	0,98	0,048
1,75	13714	0	0,12	0	0	0,88	0	0	0	0	0	0	1	0	0,04
1,75	11455	0,02	0,03	0	0,06	0,89	0	0	0	0	0	0,11	0,89	0	0,044
1,75	19899	0,04	0	0,01	0	0,55	0	0	0,4	0	0	0,57	0,43	0	0,044
1,75	13030	0	0,93	0	0	0,04	0	0	0,03	0	0,03	0,39	0,01	0,57	0,046
1,75	11967	0	0	0,01	0,15	0,84	0	0	0	0	0,84	0	0,01	0,15	0,048
1,75	11933	0	0,08	0,02	0	0,57	0	0	0,28	0,05	0,36	0,07	0,57	0	0,048
1,75	29410	0,04	0	0,02	0,01	0,81	0,12	0	0	0	0,1	0,02	0,85	0,03	0,038
1,75	22407	0	0,28	0,01	0	0,71	0	0	0	0	0,28	0	0,01	0,71	0,046
1,75	14228	0,04	0,02	0	0,02	0,92	0	0	0	0	0	0,04	0,04	0,92	0,044
1,75	29043	0	0,12	0	0	0,87	0	0,01	0	0	0,5	0	0,49	0,01	0,044
1,75	8250	0,07	0	0	0	0,04	0,03	0	0,86	0	0,05	0,04	0,05	0,86	0,048

1,75	12184	0	0	0	0,11	0,89	0	0	0	0	0,47	0,12	0	0,41	0,046
1,75	46134	0,02	0	0,03	0,02	0,88	0,03	0	0	0,02	0,89	0,04	0,07	0	0,048
1,75	34583	0,05	0	0	0	0,8	0,14	0	0	0,01	0,01	0	0,99	0	0,044
1,75	13659	0,05	0	0	0	0,94	0	0,01	0	0	0	0,06	0,94	0	0,046
1,75	11735	0	0,01	0,02	0,05	0,88	0	0	0,04	0	0,18	0,09	0,01	0,72	0,044
1,75	40246	0	0,22	0	0	0,72	0,06	0	0	0	0,06	0	0,32	0,62	0,04
1,75	7860	0	0,08	0	0,02	0,89	0	0,01	0	0	0	0	0,88	0,12	0,046
1,75	17708	0	0,95	0	0,03	0	0	0	0,01	0,01	0,01	0	0,01	0,98	0,042
1,75	17341	0	0,99	0	0	0,01	0	0	0	0	0	1	0	0	0,036
1,75	68969	0	0,23	0	0	0,66	0,11	0	0	0	0,14	0,09	0,26	0,51	0,048
1,75	9781	0,04	0,03	0,02	0	0,9	0	0	0,01	0	0,01	0,05	0,94	0	0,048
1,75	26219	0,06	0	0	0	0,93	0	0	0	0,01	0,97	0	0	0,03	0,046
1,75	36525	0,02	0,05	0	0	0,93	0	0	0	0	0,94	0	0,04	0,02	0,044
1,75	13637	0	0,14	0	0,03	0,79	0	0,01	0,03	0	0,14	0	0,03	0,83	0,046
1,75	7463	0,05	0,05	0	0	0,9	0	0	0	0	0,15	0	0,05	0,8	0,046
1,75	2843	0,05	0	0,02	0	0,67	0,26	0	0	0	0,69	0	0,09	0,22	0,048
1,75	5024	0	0,12	0	0	0,88	0	0	0	0	0,04	0,01	0,95	0	0,048
1,75	5709	0	0,01	0,04	0	0,93	0	0	0,02	0	0	0,03	0,97	0	0,03
1,75	13205	0	0,14	0	0	0,85	0,01	0	0	0	0	0	0,99	0,01	0,046
1,75	51971	0,01	0	0,01	0,17	0,81	0	0	0	0	0,82	0,17	0,01	0	0,042
1,75	14743	0	0	0	0,13	0	0,43	0,01	0,43	0	0,01	0,13	0,86	0	0,042
1,75	10853	0,04	0,01	0,03	0	0,92	0	0	0	0	0,01	0,03	0,92	0,04	0,044
1,75	20984	0	0	0,01	0,19	0,8	0	0	0	0	0,8	0	0,2	0	0,048
1,75	16763	0,02	0,17	0,04	0	0,77	0	0	0	0	0,01	0,78	0,03	0,18	0,046
1,75	7515	0,02	0,04	0,02	0	0,91	0	0,01	0	0	0,49	0,45	0,04	0,02	0,042
1,75	24211	0,02	0	0,04	0	0	0,43	0,02	0,48	0,01	0,36	0,18	0	0,46	0,048
1,75	10906	0	0,09	0	0	0	0	0	0,91	0	0,43	0,56	0,01	0	0,048
2	14492	0,04	0	0	0,02	0,92	0	0	0	0,02	0	0,98	0	0,02	0,044
2	22919	0,01	0,02	0,03	0	0,94	0	0	0	0	0,97	0,01	0,02	0	0,048
2	29776	0	0	0	0,24	0,76	0	0	0	0	0,87	0,01	0,12	0	0,048
2	22155	0,04	0	0,02	0	0,27	0,07	0,02	0,58	0	0	0,07	0,84	0,09	0,048
2	16446	0,06	0	0	0	0,72	0	0	0,22	0	0	0,03	0,69	0,28	0,048
2	18449	0,06	0	0	0	0,94	0	0	0	0	0,96	0	0	0,04	0,048

2	16872	0,05	0	0,03	0,01	0,52	0	0	0,39	0	0,35	0,55	0	0,1	0,048
2	10882	0,05	0	0,03	0	0,79	0	0	0,13	0	0	0,16	0,79	0,05	0,042
2	7381	0	0	0	0,29	0,68	0,03	0	0	0	0,97	0,03	0	0	0,042
2	41682	0	0,19	0	0,01	0,79	0	0,01	0	0	0	0,11	0,21	0,68	0,042
2	6345	0	0,11	0	0,03	0	0,77	0	0,09	0	0,03	0	0,77	0,2	0,044
2	20690	0,04	0,05	0	0	0,91	0	0	0	0	0	0,05	0,91	0,04	0,048
2	14299	0,06	0	0,01	0,01	0	0,14	0	0,78	0	0,01	0	0,14	0,85	0,048
2	20935	0,02	0	0	0,05	0,57	0,36	0	0	0	0	0,36	0,15	0,49	0,048
2	21487	0	0,03	0	0	0	0,83	0,14	0	0	0,83	0,13	0	0,04	0,048
2	7645	0	0	0	0,21	0	0,79	0	0	0	0,76	0	0,17	0,07	0,046
2	38022	0,01	0	0	0,06	0,93	0	0	0	0	0,1	0	0,01	0,89	0,046
2	6636	0	0	0	0,13	0,86	0,01	0	0	0	0	0	0,87	0,13	0,046
2	9099	0,06	0	0	0	0	0,93	0	0,01	0	0,02	0,01	0,93	0,04	0,048
2	9760	0,01	0	0	0,03	0,82	0	0,03	0,11	0	0	0,82	0,07	0,11	0,048
2	12171	0	0,93	0,01	0	0	0,06	0	0	0	0,06	0,01	0	0,93	0,048
2	16242	0,02	0	0	0,05	0,69	0,11	0	0,13	0	0	0,85	0,15	0	0,046
2	23829	0,02	0,05	0	0,02	0,91	0	0	0	0	0,91	0,05	0	0,04	0,048
2	4322	0,02	0	0,01	0,06	0,83	0,01	0	0,07	0	0,13	0,02	0,83	0,02	0,048
2	6799	0	0	0	0,09	0,9	0	0,01	0	0	0	0	0,9	0,1	0,042
2	20156	0,05	0	0	0	0	0,19	0	0,76	0	0	0,95	0,02	0,03	0,04
2	6181	0,04	0,02	0,04	0	0,55	0	0	0,33	0,02	0,37	0,04	0	0,59	0,048
2	9031	0,01	0	0,04	0,05	0,65	0,03	0,01	0,21	0	0,06	0,69	0,04	0,21	0,044
2	13644	0	0	0,04	0	0,54	0,42	0	0	0	0	0,58	0,42	0	0,018
2	12423	0,03	0	0,04	0	0,85	0	0,01	0	0,07	0,1	0,05	0	0,85	0,04
2	33583	0,04	0,04	0	0	0	0,92	0	0	0	0,01	0,02	0,93	0,04	0,044
2	29852	0	0,2	0	0,02	0,76	0,02	0	0	0	0,26	0	0	0,74	0,044
2	21051	0,03	0	0,04	0,01	0,65	0,26	0,01	0	0	0,04	0,04	0,66	0,26	0,048
2	7854	0,05	0	0,04	0	0,88	0	0	0,03	0	0	0,07	0,04	0,89	0,048
2	9264	0	0,01	0	0,09	0,08	0	0	0,78	0,04	0	0,01	0,91	0,08	0,046
2	24946	0	0,23	0	0	0,76	0	0,01	0	0	0	0	0,46	0,54	0,046
2	16246	0,07	0,03	0	0	0,68	0,22	0	0	0	0,07	0,68	0	0,25	0,048
2	25511	0	0,42	0	0,03	0,53	0	0,01	0,01	0	0	0,97	0	0,03	0,048
2	30828	0,04	0,02	0	0,01	0,87	0	0	0,06	0	0,08	0,87	0	0,05	0,048

2	26799	0,02	0	0,04	0	0,77	0	0	0,17	0	0	0,03	0,96	0,01	0,036
2	8188	0	0,17	0	0	0,8	0,03	0	0	0	0	0,49	0,49	0,02	0,048
2	11592	0,05	0	0	0,01	0,9	0	0	0,03	0,01	0,91	0	0,06	0,03	0,046
2	17234	0	0,07	0	0,02	0,9	0	0	0	0,01	0,81	0	0,08	0,11	0,048
2	34931	0	0	0	0,23	0,6	0,17	0	0	0	0,73	0,21	0	0,06	0,042
2	9738	0,05	0	0,02	0	0,91	0	0	0,02	0	0	0,02	0,93	0,05	0,046
2	11730	0,02	0	0	0,06	0,92	0	0	0	0	0	0,92	0,06	0,02	0,046
2	24177	0	0,25	0	0,04	0,39	0	0	0,32	0	0,01	0,56	0	0,43	0,044
2	25140	0,01	0	0	0	0,95	0	0,04	0	0	0,04	0,01	0,01	0,94	0,044
2	25347	0,04	0,96	0	0	0	0	0	0	0	0,01	0	0	0,99	0,048
2	13915	0,05	0	0,03	0	0,07	0	0	0,85	0	0	0,09	0,01	0,9	0,044
2	17844	0,02	0,72	0,03	0,01	0,22	0	0	0	0	0	0,04	0,02	0,94	0,048
2	7008	0,02	0,33	0,02	0	0	0	0,01	0,62	0	0,01	0,02	0,11	0,86	0,046
2	19136	0,01	0,92	0	0	0,04	0	0	0	0,03	0	0	0,04	0,96	0,042
2	24945	0,01	0,38	0	0	0,54	0,06	0	0	0,01	0,08	0,38	0	0,54	0,044
2	6010	0,04	0	0	0	0,95	0	0,01	0	0	0,04	0	0	0,96	0,046
2	6633	0	0	0,03	0,11	0,82	0	0	0	0,04	0,15	0,82	0,02	0,01	0,046
2	20020	0,02	0,45	0,01	0	0,5	0	0,01	0,01	0	0,02	0,4	0,06	0,52	0,044
2	9575	0	0,12	0	0	0,87	0,01	0	0	0	0,12	0	0,88	0	0,048
2	16527	0,01	0,06	0	0	0	0,93	0	0	0	0,99	0	0	0,01	0,044
2	18239	0	0	0	0,12	0,88	0	0	0	0	0,11	0	0,89	0	0,038
2	13592	0	0,11	0,02	0,01	0,85	0,01	0	0	0	0,12	0	0,85	0,03	0,048
2	4195	0,01	0	0	0,09	0,81	0	0,03	0,06	0	0	0,09	0,85	0,06	0,046
2	7443	0,02	0	0,04	0	0	0	0	0,93	0,01	0,9	0,07	0	0,03	0,042
2	15483	0,03	0,01	0	0	0	0,91	0,01	0,04	0	0,85	0	0,11	0,04	0,03
2	24417	0	0,2	0	0	0	0	0	0,8	0	0	0,8	0,2	0	0,048
2	8090	0,06	0	0,03	0	0,91	0	0	0	0	0	0,09	0	0,91	0,046
2	31619	0,06	0	0	0	0	0,1	0	0,84	0	0,84	0	0,1	0,06	0,046
2	39951	0	0	0,03	0,14	0,82	0	0	0,01	0	0,96	0,03	0	0,01	0,046
2	12892	0,02	0,02	0,04	0	0,84	0,01	0	0,03	0,04	0	0,07	0,88	0,05	0,04
2	11892	0,01	0,11	0,01	0	0,86	0	0	0,01	0	0,06	0,01	0,88	0,05	0,046
2	7789	0,04	0,01	0,03	0	0,08	0,01	0	0,83	0	0,09	0,86	0,01	0,04	0,048
2	35262	0,01	0,1	0	0	0,88	0,01	0	0	0	0,03	0,02	0,95	0	0,036

2	9893	0,05	0	0,01	0	0,89	0,05	0	0	0	0	0,01	0,99	0	0,046
2	5801	0,03	0,05	0,04	0	0,03	0,17	0	0,68	0	0	0,92	0,05	0,03	0,042
2	7740	0	0,15	0	0	0,85	0	0	0	0	0	0	0,94	0,06	0,046
2	12220	0	0	0	0,29	0,69	0	0	0,02	0	0,96	0	0,02	0,02	0,046
2	35756	0,06	0	0	0	0	0	0	0,89	0,05	0	0,05	0	0,95	0,046
2	36482	0,05	0	0	0	0,35	0,54	0	0,06	0	0,05	0	0,42	0,53	0,036
2	15220	0	0	0	0,12	0,86	0	0	0,02	0	0,05	0,02	0,93	0	0,046
2	13755	0	0,09	0,02	0,01	0,8	0,08	0	0	0	0	0,09	0,88	0,03	0,046
2	28670	0	0,14	0,01	0,01	0,84	0	0	0	0	0	0	0,84	0,16	0,048
2	11907	0	0	0,01	0,08	0,91	0	0	0	0	0,09	0,89	0,02	0	0,048
2	9878	0	0,56	0,01	0	0	0	0,02	0,41	0	0,02	0	0	0,98	0,044
2	27260	0	0	0	0,06	0,94	0	0	0	0	0,06	0	0	0,94	0,042
2	16790	0,01	0	0,01	0,14	0,68	0,05	0	0,11	0	0,68	0,02	0,16	0,14	0,048
2	11586	0	0,33	0	0,01	0,66	0	0	0	0	0,09	0,66	0	0,25	0,046
2	24056	0,05	0,01	0	0	0,88	0,04	0	0,02	0	0,01	0,11	0,88	0	0,048
2	18186	0	0,02	0	0,13	0,79	0	0	0,06	0	0,87	0	0	0,13	0,04
2	9786	0,01	0,06	0,04	0,01	0,51	0,37	0	0	0	0,06	0,52	0,04	0,38	0,034
2	5809	0	0,06	0	0	0,92	0	0	0	0,02	0,92	0,01	0	0,07	0,038
2	6186	0,06	0	0	0	0,17	0	0	0,75	0,02	0,92	0,02	0,06	0	0,046
2	7170	0	0	0,03	0,1	0,72	0,04	0	0,07	0,04	0,75	0,21	0	0,04	0,042
2	10873	0,04	0	0,01	0	0,02	0,9	0	0,03	0	0,9	0,02	0,05	0,03	0,042
2	29395	0	0,18	0	0	0,24	0,58	0	0	0	0,14	0,18	0,26	0,42	0,046
2	4731	0	0	0,04	0,05	0,9	0	0	0,01	0	0,91	0,08	0,01	0	0,036
2	25508	0	0	0	0,05	0,9	0,04	0,01	0	0	0,05	0,05	0	0,9	0,048
2	20294	0	0	0	0,23	0,74	0	0,01	0,02	0	0,76	0,01	0,23	0	0,044
2	13415	0	0,08	0	0	0,48	0,4	0	0	0,04	0,88	0	0,04	0,08	0,048
2	10540	0	0	0	0,11	0,8	0	0	0,09	0	0	0,11	0,8	0,09	0,046
2	9038	0,01	0,06	0	0	0,92	0	0,01	0	0	0,98	0	0,02	0	0,042
2,25	17225	0,03	0,2	0,02	0	0,1	0,01	0	0,64	0	0,01	0,12	0,2	0,67	0,042
2,25	8677	0	0,16	0	0,03	0,77	0,01	0	0,03	0	0,11	0	0,04	0,85	0,04
2,25	3578	0,05	0	0	0,01	0,87	0,07	0	0	0	0,03	0,91	0,03	0,03	0,04
2,25	23579	0,05	0	0,01	0	0,94	0	0	0	0	0,07	0,88	0,05	0	0,044
2,25	12696	0,03	0,03	0	0,02	0,84	0,08	0	0	0	0,92	0,04	0,02	0,02	0,048

2,25	36432	0	0	0	0,07	0,93	0	0	0	0	0,07	0,92	0	0,01	0,046
2,25	15687	0,01	0	0,04	0	0,93	0	0,02	0	0	0,01	0,97	0,01	0,01	0,03
2,25	13954	0	0	0,04	0	0,94	0,01	0	0	0,01	0,05	0,01	0,94	0	0,028
2,25	29817	0,06	0,08	0,01	0	0,83	0	0	0	0,02	0,02	0,14	0,01	0,83	0,048
2,25	11062	0,03	0,69	0	0	0	0	0	0,28	0	0	0	0,03	0,97	0,042
2,25	23527	0	0	0	0,06	0	0	0	0,94	0	0	1	0	0	0,04
2,25	26606	0,01	0,21	0	0,03	0,74	0	0	0,01	0	0	0,03	0,22	0,75	0,048
2,25	10154	0,01	0	0,02	0,02	0	0,89	0,06	0	0	0	0,05	0,93	0,02	0,048
2,25	23895	0	0,01	0	0,11	0,87	0	0	0,01	0	0,01	0,01	0,98	0	0,044
2,25	24004	0	0,01	0	0,07	0,92	0	0	0	0	0,01	0,92	0	0,07	0,046
2,25	7225	0,04	0	0,01	0,06	0,87	0	0	0,02	0	0	0,03	0,88	0,09	0,036
2,25	16033	0,03	0	0,03	0,01	0,93	0	0	0	0	0,93	0,03	0,03	0,01	0,038
2,25	19212	0	0	0	0,34	0,66	0	0	0	0	1	0	0	0	0,044
2,25	46931	0,04	0	0,01	0	0	0	0,01	0,94	0	0,95	0	0	0,05	0,046
2,25	9989	0,02	0	0,04	0	0,94	0	0	0	0	0,06	0,04	0	0,9	0,026
2,25	29223	0	0,31	0	0	0,65	0	0	0,04	0	0,01	0,04	0,3	0,65	0,044
2,25	32025	0,06	0,07	0	0	0,87	0	0	0	0	0,02	0,06	0	0,92	0,048
2,25	9587	0	0,03	0	0,06	0,91	0	0	0	0	0,08	0,91	0	0,01	0,046
2,25	25760	0,03	0,01	0,03	0	0,85	0	0,02	0,06	0	0,85	0,01	0	0,14	0,048
2,25	9628	0,01	0,09	0	0	0,01	0,75	0	0,03	0,11	0,58	0	0,35	0,07	0,048
2,25	14091	0	0,01	0	0,2	0,75	0,04	0	0	0	0,79	0,18	0	0,03	0,048
2,25	21889	0	0,19	0,04	0	0,76	0,01	0	0	0	0,19	0,04	0	0,77	0,044
2,25	8892	0,05	0,02	0	0	0	0	0,01	0,92	0	0	0,91	0,01	0,08	0,046
2,25	7647	0	0	0	0,1	0,16	0	0	0,73	0,01	0,01	0,8	0,16	0,03	0,044
2,25	6846	0	0,73	0,01	0	0	0	0,04	0,22	0	0	0,01	0,03	0,96	0,048
2,25	17041	0	0,02	0	0,07	0,91	0	0	0	0	0,07	0,93	0	0	0,04
2,25	47701	0	1	0	0	0	0	0	0	0	0	1	0	0	0,024
2,25	38538	0,01	0,37	0,01	0	0,32	0,03	0	0,25	0,01	0,03	0,66	0,01	0,3	0,044
2,25	19031	0,05	0	0	0	0,9	0,04	0,01	0	0	0,06	0,94	0	0	0,048
2,25	10497	0,02	0,19	0	0	0,79	0	0	0	0	0,11	0,79	0,02	0,08	0,046
2,25	4822	0,04	0	0,02	0	0,93	0	0	0	0,01	0,94	0,01	0	0,05	0,048
2,25	8049	0,06	0	0	0	0	0	0	0,94	0	0	0	0,94	0,06	0,042
2,25	17329	0	0,12	0	0	0,84	0,03	0	0,01	0	0,04	0,12	0,84	0	0,046

2,25	15838	0	0,95	0,04	0	0	0	0	0,01	0	0	0,27	0,02	0,71	0,034
2,25	32018	0	0	0	0,12	0,7	0,17	0	0,01	0	0,13	0,17	0,7	0	0,048
2,25	27619	0	0,16	0	0	0,01	0	0	0,83	0	0,01	0,17	0,82	0	0,048
2,25	10113	0	0,09	0	0,01	0,03	0	0,02	0,85	0	0,02	0	0,97	0,01	0,046
2,25	15360	0,05	0	0,01	0	0,73	0,14	0	0,07	0	0,01	0,73	0,21	0,05	0,046
2,25	22491	0,03	0,23	0,01	0	0,71	0	0	0,02	0	0,16	0,01	0,01	0,82	0,046
2,25	5736	0,03	0	0,04	0	0	0,93	0	0	0	0,93	0,03	0,03	0,01	0,046
2,25	31381	0	0	0,02	0,17	0	0,56	0	0,25	0	0,54	0,25	0,17	0,04	0,046
2,25	2636	0,03	0,07	0,01	0	0,88	0	0	0,01	0	0,07	0,89	0,01	0,03	0,038
2,25	18996	0,05	0,01	0	0	0,13	0,01	0,01	0,79	0	0,02	0,02	0,23	0,73	0,042
2,25	6309	0,03	0	0	0,04	0	0	0	0,92	0,01	0,04	0,92	0	0,04	0,048
2,25	16739	0	0	0	0,13	0,87	0	0	0	0	0,64	0	0	0,36	0,048
2,25	8328	0,05	0,01	0,03	0	0,89	0,01	0	0,01	0	0,02	0,92	0	0,06	0,04
2,25	64799	0	0,13	0	0	0,84	0	0	0,02	0,01	0,14	0,39	0,47	0	0,046
2,25	11743	0,06	0,02	0	0	0,02	0	0	0,9	0	0,07	0,02	0	0,91	0,048
2,25	14609	0,04	0,04	0	0	0,9	0	0,02	0	0	0,02	0,93	0,01	0,04	0,04
2,25	5411	0	0	0	0,06	0,91	0	0	0,02	0,01	0,06	0,03	0,01	0,9	0,046
2,25	30341	0	0	0,02	0,11	0,8	0	0	0,07	0	0,61	0,07	0,02	0,3	0,048
2,25	6842	0	0,09	0,02	0,04	0,85	0	0	0	0	0,09	0	0,89	0,02	0,048
2,25	11274	0	0,11	0	0	0,88	0	0,01	0	0	0,59	0,02	0,39	0	0,048
2,25	42636	0	0	0	0,13	0	0,87	0	0	0	0	0	0,91	0,09	0,036
2,25	17193	0	0,43	0,01	0	0	0	0,05	0,51	0	0	0,53	0	0,47	0,042
2,25	22246	0	0	0	0,22	0	0,76	0	0,01	0,01	0,77	0,22	0,01	0	0,048
2,25	10968	0,02	0,22	0	0	0,44	0,07	0	0,25	0	0,25	0	0,09	0,66	0,048
2,25	12662	0	1	0	0	0	0	0	0	0	0	1	0	0	0,042
2,25	12830	0,05	0	0	0,01	0,94	0	0	0	0	0,01	0	0,94	0,05	0,046
2,25	9510	0,06	0	0	0	0,94	0	0	0	0	0,44	0	0,56	0	0,042
2,25	17446	0,06	0,01	0,03	0	0,9	0	0	0	0	0,03	0,91	0,06	0	0,048
2,25	61047	0	0,1	0,02	0	0,75	0,01	0	0,11	0,01	0,58	0,05	0,12	0,25	0,046
2,25	23880	0,04	0,01	0	0	0,94	0	0,01	0	0	0,93	0,02	0,04	0,01	0,048
2,25	14872	0	0,05	0	0,08	0,86	0	0	0	0,01	0,44	0	0,48	0,08	0,044
2,25	26636	0,04	0	0,03	0	0,88	0,05	0	0	0	0	0,12	0,83	0,05	0,046
2,25	28481	0,05	0	0	0	0,01	0,93	0,01	0	0	0,05	0,02	0,93	0	0,046

2,25	19567	0	0	0,01	0,31	0,66	0,02	0	0	0	0,97	0,02	0,01	0	0,04
2,25	47719	0,06	0,02	0	0	0	0,92	0	0	0	0,25	0,58	0,17	0	0,048
2,25	12419	0,05	0,03	0	0	0,92	0	0	0	0	0,8	0,01	0,12	0,07	0,048
2,25	39298	0	0	0,01	0,22	0,63	0,14	0	0	0	0,85	0	0,14	0,01	0,042
2,25	12584	0	0,23	0,02	0	0,08	0	0	0,67	0	0	0,08	0,25	0,67	0,044
2,25	13328	0,05	0	0,02	0	0,91	0,02	0	0	0	0,05	0,89	0	0,06	0,046
2,25	5786	0	0,01	0,04	0	0,5	0,02	0	0,42	0,01	0,42	0,06	0,01	0,51	0,018
2,25	6516	0,03	0,03	0	0,04	0,87	0,03	0	0	0	0,87	0,1	0,03	0	0,048
2,25	23214	0	0	0	0,14	0,01	0,85	0	0	0	0,24	0,02	0,74	0	0,048
2,25	9877	0,05	0	0	0	0,95	0	0	0	0	0	0,95	0,05	0	0,04
2,25	20091	0,07	0	0,04	0	0,89	0	0	0	0	0	0,04	0,07	0,89	0,046
2,25	20498	0,05	0	0	0	0,91	0	0,03	0	0,01	0,97	0	0,01	0,02	0,04
2,25	5707	0,01	0,15	0,01	0	0,7	0	0	0,09	0,04	0,13	0,74	0,11	0,02	0,048
2,25	21644	0	0,17	0	0,01	0,82	0	0	0	0	0,16	0,82	0,01	0,01	0,038
2,25	20753	0,01	0	0,04	0	0,92	0	0	0,01	0,02	0,91	0,04	0,03	0,02	0,036
2,25	11599	0	0	0,01	0,18	0,78	0	0,03	0	0	0,78	0,22	0	0	0,048
2,25	11962	0	0,07	0	0,02	0	0,87	0,02	0,01	0,01	0,96	0,01	0,01	0,02	0,044
2,25	13859	0	0	0,01	0,06	0	0	0	0,93	0	0,01	0,05	0	0,94	0,044
2,25	29522	0,04	0	0	0,06	0,9	0	0	0	0	0,96	0,04	0	0	0,048
2,25	8546	0,01	0,05	0	0,04	0,9	0	0	0	0	0,06	0	0	0,94	0,046
2,25	19772	0,03	0	0	0,05	0,84	0	0,01	0,06	0,01	0,05	0,91	0,04	0	0,048
2,25	11014	0	0	0	0,17	0,77	0	0	0,06	0	0,54	0,4	0,06	0	0,046
2,25	20986	0,05	0	0	0	0,94	0	0	0	0,01	0	0,01	0,06	0,93	0,048
2,25	5290	0,03	0	0	0,09	0,88	0	0	0	0	0,66	0,25	0	0,09	0,048
2,25	8568	0	0	0	0,09	0,9	0,01	0	0	0	0,01	0,93	0,06	0	0,046
2,25	17809	0	0,1	0	0,04	0,85	0,01	0	0	0	0,04	0,09	0,76	0,11	0,036
2,25	16220	0,01	0,24	0,01	0	0,74	0	0	0	0	0,25	0	0	0,75	0,046
2,25	15023	0,05	0	0,01	0	0,93	0,01	0	0	0	0	0,98	0,01	0,01	0,04
2,25	15965	0	1	0	0	0	0	0	0	0	0	1	0	0	0,038
2,5	10090	0,02	0,05	0	0,02	0,91	0	0	0	0	0,07	0	0,91	0,02	0,048
2,5	21805	0	0,03	0,03	0,07	0,87	0	0	0	0	0,05	0,94	0	0,01	0,048
2,5	12643	0,05	0	0,03	0	0,92	0	0	0	0	0	0,95	0,05	0	0,044
2,5	11962	0	0	0	0,11	0,15	0	0	0,74	0	0,01	0,11	0,74	0,14	0,044

2,5	15910	0	0,04	0	0,15	0,78	0,03	0	0	0	0,81	0	0,19	0	0,044
2,5	32059	0	0,16	0	0	0,83	0	0	0	0,01	0,41	0,09	0,08	0,42	0,048
2,5	14376	0,02	0,42	0	0	0,56	0	0	0	0	0,06	0	0	0,94	0,038
2,5	14343	0,04	0,01	0,01	0	0,94	0	0	0	0	0,95	0,01	0	0,04	0,046
2,5	8835	0,05	0,04	0	0	0,05	0,02	0	0,84	0	0,03	0	0,1	0,87	0,042
2,5	12342	0	0,18	0	0,01	0,81	0	0	0	0	0	0,81	0,18	0,01	0,046
2,5	17771	0	0	0	0,06	0,94	0	0	0	0	0,06	0	0	0,94	0,048
2,5	27082	0	0,07	0,01	0	0,92	0	0	0	0	0,92	0,01	0,07	0	0,048
2,5	18904	0,01	0	0,03	0,06	0,9	0	0	0	0	0,03	0,96	0,01	0	0,044
2,5	7351	0	0	0	0,05	0,89	0	0,02	0,04	0	0	0,93	0,05	0,02	0,048
2,5	100000	0,01	0,02	0,97	0	0	0	0	0	0	0,02	0,97	0,01	0	0,926
2,5	12416	0	0	0,02	0,06	0,92	0	0	0	0	0	0	0,08	0,92	0,046
2,5	12653	0	0,25	0,01	0,01	0,66	0,05	0	0,02	0	0,02	0,03	0,27	0,68	0,046
2,5	20789	0	0,56	0	0	0	0	0,03	0,41	0	0	0,56	0	0,44	0,048
2,5	58383	0	0,13	0	0	0,85	0	0,01	0,01	0	0	0	1	0	0,042
2,5	18396	0,06	0	0	0	0	0,77	0	0,13	0,04	0,73	0,27	0	0	0,042
2,5	14818	0	0	0	0,09	0,9	0	0	0	0,01	0	0,71	0,28	0,01	0,038
2,5	7040	0,02	0,04	0,04	0	0	0	0	0,9	0	0	0,01	0,42	0,57	0,046
2,5	17868	0,05	0,01	0	0	0	0,88	0	0,06	0	0,28	0,06	0,6	0,06	0,046
2,5	7540	0,06	0	0	0	0,84	0,04	0	0	0,06	0,04	0,7	0,07	0,19	0,046
2,5	32566	0	0,1	0	0,06	0,83	0	0	0,01	0	0,1	0,29	0,02	0,59	0,048
2,5	12712	0	0,93	0	0,01	0	0,04	0	0,02	0	0,03	0,02	0	0,95	0,048
2,5	6309	0	0	0,04	0,01	0,94	0,01	0	0	0	0	0,06	0,94	0	0,036
2,5	36960	0,04	0	0	0,05	0	0,87	0,01	0,03	0	0,81	0,05	0,12	0,02	0,04
2,5	13124	0,05	0	0,02	0	0,92	0	0	0	0,01	0	0,93	0,01	0,06	0,04
2,5	18524	0	0,14	0	0	0,86	0	0	0	0	0,05	0	0,92	0,03	0,048
2,5	6246	0,04	0	0,02	0	0,94	0	0	0	0	0	0,02	0,94	0,04	0,044
2,5	25301	0	0,57	0	0	0,43	0	0	0	0	0	0	0,16	0,84	0,044
2,5	11161	0	0,14	0	0	0,86	0	0	0	0	0,03	0,11	0,86	0	0,038
2,5	8650	0	1	0	0	0	0	0	0	0	0	1	0	0	0,046
2,5	15982	0	0,22	0	0,03	0,75	0	0	0	0	0	0	0,22	0,78	0,038
2,5	10362	0,01	0	0,04	0,01	0	0	0,03	0,91	0	0	0,03	0,04	0,93	0,044
2,5	18199	0,05	0	0,03	0	0,01	0,68	0	0,07	0,16	0,12	0,08	0,74	0,06	0,046

2,5	11039	0,03	0,01	0	0,06	0,9	0	0	0	0	0,28	0,62	0,09	0,01	0,046
2,5	16185	0	0,02	0	0,07	0,88	0,03	0	0	0	0,07	0	0	0,93	0,046
2,5	6769	0,05	0	0,03	0	0	0,01	0,01	0,79	0,11	0	0	0,85	0,15	0,048
2,5	17963	0,06	0	0	0	0	0,26	0	0,68	0	0,26	0,01	0,68	0,05	0,048
2,5	28507	0	0,13	0	0	0,82	0,01	0	0,04	0	0,01	0	0,99	0	0,048
2,5	6860	0	0	0	0,27	0,67	0,05	0	0,01	0	0,89	0,06	0,05	0	0,048
2,5	20592	0	0	0	0,28	0,72	0	0	0	0	0,97	0	0,03	0	0,048
2,5	11960	0	0	0	0,06	0,94	0	0	0	0	0	0,06	0	0,94	0,046
2,5	23898	0	0	0	0,24	0,74	0	0,02	0	0	0,98	0	0,02	0	0,038
2,5	5567	0	0,13	0	0,01	0,86	0	0	0	0	0,04	0,01	0,95	0	0,046
2,5	12905	0,01	0	0	0,08	0,85	0	0,01	0,05	0	0,08	0,2	0,65	0,07	0,044
2,5	5589	0	0	0	0,07	0,54	0	0	0,39	0	0,03	0,96	0,01	0	0,044
2,5	14400	0,05	0	0,01	0,02	0	0,92	0	0	0	0,99	0,01	0	0	0,046
2,5	22512	0,02	0	0,04	0	0,92	0	0,02	0	0	0,04	0,08	0,65	0,23	0,044
2,5	3146	0	0	0	0,06	0	0	0	0,94	0	0	0	0,06	0,94	0,042
2,5	13081	0	0	0,04	0,03	0,82	0,11	0	0	0	0	0,11	0,86	0,03	0,036
2,5	24679	0	0	0,04	0	0	0,66	0	0,3	0	0,33	0	0,01	0,66	0,012
2,5	5193	0	0,09	0	0,01	0,84	0	0,06	0	0	0,04	0,47	0,15	0,34	0,046
2,5	24014	0	0	0,04	0	0,9	0	0	0,03	0,03	0	0,07	0,9	0,03	0,018
2,5	9286	0	0,11	0	0	0,8	0,07	0	0,02	0	0,18	0,09	0,73	0	0,044
2,5	17009	0	0,02	0,04	0	0,93	0,01	0	0	0	0,96	0	0,01	0,03	0,048
2,5	15406	0,02	0	0,04	0	0,94	0	0	0	0	0	0,04	0,04	0,92	0,024
2,5	6972	0,05	0	0	0,01	0,93	0	0	0	0,01	0,94	0,02	0	0,04	0,048
2,5	12414	0,03	0,08	0	0,03	0,85	0,01	0	0	0	0,09	0,88	0	0,03	0,048
2,5	12146	0,03	0,01	0,01	0,09	0	0,84	0	0	0,02	0,94	0	0	0,06	0,046
2,5	71812	0,05	0	0	0	0,74	0,21	0	0	0	0,78	0,21	0	0,01	0,03
2,5	23452	0,06	0	0	0	0	0	0	0,93	0,01	0,03	0,87	0,06	0,04	0,048
2,5	8790	0	0,38	0,04	0	0	0,01	0	0,57	0	0,03	0,02	0	0,95	0,03
2,5	15149	0,05	0	0	0	0,94	0	0	0	0,01	0,05	0,01	0,94	0	0,044
2,5	12940	0	0	0	0,06	0,92	0,01	0	0,01	0	0,06	0,34	0	0,6	0,044
2,5	17125	0	0	0	0,15	0	0,22	0	0,63	0	0,63	0	0,37	0	0,04
2,5	13283	0	0,13	0,02	0	0,82	0,03	0	0	0	0	0,02	0,95	0,03	0,044
2,5	5317	0	0,99	0,01	0	0	0	0	0	0	0,08	0	0	0,92	0,046

2,5	15835	0,02	0,15	0	0	0,71	0	0,02	0,1	0	0,17	0,7	0,02	0,11	0,046
2,5	12209	0	0,89	0	0	0,06	0,05	0	0	0	0	0,03	0,08	0,89	0,048
2,5	26403	0	0,28	0	0	0,72	0	0	0	0	0	0,11	0,28	0,61	0,046
2,5	16405	0	0,07	0	0	0,81	0	0,03	0,09	0	0,88	0,09	0	0,03	0,048
2,5	28868	0,03	0	0,01	0,04	0,92	0	0	0	0	0,04	0,08	0,85	0,03	0,04
2,5	10800	0,04	0	0	0,01	0,92	0	0,01	0,02	0	0,01	0,05	0,92	0,02	0,046
2,5	39477	0	1	0	0	0	0	0	0	0	0	1	0	0	0,034
2,5	25632	0	0	0	0,05	0,95	0	0	0	0	0,05	0	0	0,95	0,044
2,5	100000	0	0	1	0	0	0	0	0	0	0	1	0	0	0,948
2,5	19898	0	0,72	0,04	0	0,22	0	0,02	0	0	0	0,02	0,03	0,95	0,046
2,5	37900	0	0,06	0,04	0	0	0,87	0	0,03	0	0,35	0	0,61	0,04	0,042
2,5	7634	0	0,17	0	0	0,41	0,26	0	0,16	0	0,16	0,16	0,27	0,41	0,046
2,5	23223	0,06	0	0,02	0	0,53	0,17	0	0,22	0	0,23	0,02	0,22	0,53	0,046
2,5	11583	0,01	0	0	0,16	0,75	0,06	0	0,01	0,01	0,69	0,13	0,18	0	0,046
2,5	19179	0,04	0,02	0	0	0,93	0	0	0,01	0	0	0,01	0,98	0,01	0,048
2,5	3224	0,04	0	0,04	0	0,9	0,01	0	0	0,01	0,02	0,06	0	0,92	0,036
2,5	7557	0,01	0,07	0	0	0,92	0	0	0	0	0,17	0,01	0,82	0	0,042
2,5	22553	0	0	0	0,25	0,75	0	0	0	0	0,73	0,25	0	0,02	0,048
2,5	36844	0,06	0	0	0	0,8	0,14	0	0	0	0	0,14	0,86	0	0,044
2,5	20843	0	0,1	0	0,08	0,78	0,04	0	0	0	0	0,08	0,14	0,78	0,048
2,5	43205	0	0	0	0,1	0,52	0	0	0,38	0	0,39	0,09	0	0,52	0,038
2,5	10571	0	0,12	0	0	0,83	0	0,02	0,03	0	0,02	0,01	0,95	0,02	0,048
2,5	8463	0,03	0	0,02	0,01	0,23	0	0,02	0,67	0,02	0,17	0,02	0,28	0,53	0,042
2,5	6452	0,02	0,3	0	0	0,63	0	0	0,05	0	0,05	0	0,12	0,83	0,044
2,5	7572	0,01	0	0	0,07	0,89	0,03	0	0	0	0,07	0,89	0,01	0,03	0,048
2,5	18131	0	0,01	0	0,09	0,5	0	0,01	0,39	0	0,02	0	0,89	0,09	0,042
2,5	27170	0,05	0,02	0	0	0,91	0	0	0	0,02	0,02	0,03	0,92	0,03	0,042
2,5	11449	0,05	0,11	0	0	0,7	0	0	0,14	0	0	0,19	0	0,81	0,046
2,5	22639	0	0,92	0,03	0,01	0	0	0	0,04	0	0,01	0,04	0,04	0,91	0,044
2,5	34918	0	0,04	0	0,11	0,01	0,84	0	0	0	0,85	0	0,04	0,11	0,042
2,75	16844	0	0	0,04	0	0,72	0,24	0	0	0	0,68	0,06	0	0,26	0,032
2,75	7031	0,04	0,02	0	0	0,81	0,13	0	0	0	0,81	0	0,17	0,02	0,044
2,75	22790	0,07	0	0	0	0	0,93	0	0	0	0,93	0,07	0	0	0,048

2,75	13677	0,03	0,97	0	0	0	0	0	0	0	0	0	0	1	0,038
2,75	12642	0,06	0	0,02	0	0,47	0,08	0	0,37	0	0	0,9	0,02	0,08	0,046
2,75	28475	0,05	0	0	0,01	0,88	0,02	0	0,04	0	0,02	0,93	0,01	0,04	0,044
2,75	16186	0,01	0,19	0	0,01	0,78	0,01	0	0	0	0,26	0	0,02	0,72	0,048
2,75	41490	0	0	0,04	0	0,95	0,01	0	0	0	0,04	0	0	0,96	0,006
2,75	8801	0,01	0	0,03	0,1	0,86	0	0	0	0	0,86	0,11	0	0,03	0,042
2,75	22278	0,06	0	0	0,01	0,9	0,03	0	0	0	0,03	0,97	0	0	0,048
2,75	38075	0,01	0,09	0	0,06	0,84	0	0	0	0	0,08	0,07	0,01	0,84	0,04
2,75	32329	0,05	0	0	0	0,94	0	0	0	0,01	0,99	0	0,01	0	0,046
2,75	55189	0,06	0	0,01	0	0	0	0	0,51	0,42	0	0,72	0,15	0,13	0,048
2,75	7610	0	0,23	0	0	0,77	0	0	0	0	0	0,79	0,17	0,04	0,042
2,75	37877	0	0	0	0,13	0,83	0,02	0	0	0,02	0,13	0,02	0,78	0,07	0,048
2,75	18418	0	0,02	0,04	0,04	0,02	0	0	0,88	0	0	0,06	0,94	0	0,042
2,75	13136	0	0,01	0	0,07	0,92	0	0	0	0	0,1	0,63	0,07	0,2	0,048
2,75	24737	0,02	0	0	0,03	0,51	0,37	0,01	0,06	0	0,12	0	0,09	0,79	0,048
2,75	25384	0	0,09	0	0	0,77	0	0	0,12	0,02	0,77	0	0,03	0,2	0,038
2,75	19951	0,02	0	0,04	0	0,9	0,04	0	0	0	0,02	0,05	0,91	0,02	0,048
2,75	73216	0,05	0	0	0	0,8	0,01	0	0,14	0	0,02	0,13	0	0,85	0,034
2,75	29370	0	0	0	0,04	0,89	0	0,03	0,03	0,01	0,04	0,82	0	0,14	0,048
2,75	16012	0	0,07	0,01	0	0,9	0,01	0	0	0,01	0,94	0,03	0	0,03	0,046
2,75	22762	0	0	0	0,1	0,86	0,03	0,01	0	0	0,1	0,03	0,86	0,01	0,042
2,75	9901	0,01	0	0	0,1	0,88	0	0	0	0,01	0,51	0,03	0	0,46	0,048
2,75	17765	0	0,09	0	0	0,01	0	0,02	0,88	0	0	0,03	0,96	0,01	0,048
2,75	7305	0,03	0	0	0,04	0,88	0,01	0	0,04	0	0,03	0	0,04	0,93	0,044
2,75	25968	0	0,33	0	0	0,67	0	0	0	0	0,17	0	0,06	0,77	0,046
2,75	15176	0	0,17	0,01	0	0,8	0	0	0,02	0	0,03	0,8	0,17	0	0,042
2,75	7715	0,01	0	0,03	0	0,82	0,04	0,1	0	0	0,82	0,07	0,1	0,01	0,04
2,75	7268	0,01	0	0	0,08	0,91	0	0	0	0	0	0	0,92	0,08	0,048
2,75	5823	0,03	0,04	0,04	0	0,53	0,02	0,01	0,33	0	0,13	0,42	0,4	0,05	0,048
2,75	6248	0	0,88	0,04	0	0	0,02	0	0	0,06	0	0,06	0	0,94	0,032
2,75	8237	0,01	0,95	0,04	0	0	0	0	0	0	0,05	0	0	0,95	0,044
2,75	17448	0	0,12	0,03	0	0	0,61	0	0,24	0	0,36	0	0,01	0,63	0,048
2,75	11118	0	0,08	0	0	0,91	0	0	0	0,01	0,92	0,02	0,06	0	0,046

2,75	7776	0	0	0,01	0,06	0,93	0	0	0	0	0	0,06	0,01	0,93	0,048
2,75	6006	0	0	0,03	0,09	0,69	0	0	0,19	0	0,69	0,03	0	0,28	0,044
2,75	7270	0,05	0	0,02	0	0,23	0	0	0,7	0	0,2	0,72	0	0,08	0,034
2,75	7044	0,06	0	0	0	0,92	0,02	0	0	0	0	0,01	0,94	0,05	0,044
2,75	9881	0	0,06	0	0,05	0,82	0,06	0,01	0	0	0,09	0,76	0,05	0,1	0,048
2,75	22405	0	0	0	0,08	0,54	0,03	0	0,35	0	0,03	0,35	0,35	0,27	0,04
2,75	13706	0,01	0	0,04	0,06	0,89	0	0	0	0	0,06	0,03	0	0,91	0,048
2,75	20491	0	0	0	0,35	0	0,65	0	0	0	0,99	0	0	0,01	0,042
2,75	27953	0	0	0	0,12	0,88	0	0	0	0	0,12	0,01	0,87	0	0,036
2,75	31190	0	0	0	0,12	0,88	0	0	0	0	0,12	0	0,88	0	0,042
2,75	25989	0	0,22	0	0	0,66	0,01	0	0,11	0	0	0	0,37	0,63	0,048
2,75	24243	0,05	0,06	0	0	0	0,01	0	0,88	0	0,01	0,01	0,05	0,93	0,044
2,75	16890	0	0,01	0	0,15	0	0,03	0	0,81	0	0,82	0	0	0,18	0,048
2,75	45596	0	0,14	0	0	0,82	0,01	0	0	0,03	0	0,03	0,82	0,15	0,046
2,75	4391	0,03	0	0,02	0	0,83	0	0,03	0,09	0	0	0,05	0,93	0,02	0,042
2,75	5566	0	0	0,04	0	0,95	0	0	0	0,01	0	0,05	0,95	0	0,026
2,75	31575	0	0,09	0	0,02	0,88	0	0,01	0	0	0,01	0	0,99	0	0,044
2,75	7955	0,07	0	0	0	0,93	0	0	0	0	0,07	0	0	0,93	0,048
2,75	15136	0	0,11	0	0	0,47	0	0	0,42	0	0,42	0	0,58	0	0,048
2,75	48330	0	0	0	0,07	0,9	0,01	0	0,02	0	0,07	0,01	0,01	0,91	0,048
2,75	25167	0	0	0	0	0,29	0,61	0,1	0	0	0,08	0,01	0,29	0,62	0,04
2,75	18578	0,06	0,02	0	0	0	0	0	0,9	0,02	0	0,31	0,69	0	0,046
2,75	90184	0,02	0	0,04	0,01	0,92	0,01	0	0	0	0,91	0,07	0,02	0	0,048
2,75	16684	0,04	0	0,03	0,03	0,9	0	0	0	0	0	0,06	0,94	0	0,046
2,75	22576	0,06	0	0	0	0,94	0	0	0	0	0	0	0,06	0,94	0,046
2,75	24573	0	0,01	0	0,11	0,87	0,01	0	0	0	0	0	0,98	0,02	0,048
2,75	21411	0,06	0	0	0	0,48	0,14	0	0,3	0,02	0	0,48	0,08	0,44	0,048
2,75	23881	0,07	0	0	0	0,88	0	0	0,05	0	0	0,05	0	0,95	0,042
2,75	16505	0	0,09	0,01	0	0	0,79	0,01	0,1	0	0,61	0	0,06	0,33	0,048
2,75	13592	0,07	0	0	0	0,86	0,06	0	0,01	0	0	0,07	0,06	0,87	0,046
2,75	22599	0,06	0	0	0	0,94	0	0	0	0	0	0	0,94	0,06	0,046
2,75	12892	0	0	0,01	0,11	0	0	0	0,88	0	0	0,01	0,99	0	0,046
2,75	9119	0,06	0	0	0	0,91	0,03	0	0	0	0	0,94	0,06	0	0,046

2,75	3328	0	0	0	0,11	0,88	0	0	0,01	0	0,01	0	0,99	0	0,046
2,75	12634	0,01	0,19	0	0	0,78	0	0	0	0,02	0,02	0	0,28	0,7	0,042
2,75	30749	0	0,12	0	0	0,88	0	0	0	0	0,3	0,67	0	0,03	0,044
2,75	11179	0,04	0,13	0	0	0	0	0	0,83	0	0	0,83	0	0,17	0,046
2,75	8157	0	0,12	0,01	0	0	0	0	0,87	0	0,01	0	0,99	0	0,048
2,75	20577	0	0,07	0	0	0,93	0	0	0	0	0,97	0	0	0,03	0,046
2,75	27262	0,06	0	0	0	0,86	0,05	0	0	0,03	0,12	0,04	0,84	0	0,044
2,75	8129	0,04	0,01	0	0	0	0,08	0	0,18	0,69	0,08	0,01	0,82	0,09	0,038
2,75	41909	0	0,34	0	0	0,66	0	0	0	0	0,23	0	0	0,77	0,042
2,75	2032	0,05	0	0,01	0	0,71	0	0	0,23	0	0,71	0,01	0,28	0	0,046
2,75	34938	0,05	0,01	0	0,01	0,93	0	0	0	0	0,01	0,01	0,89	0,09	0,046
2,75	9736	0,05	0	0	0	0,91	0,02	0	0	0,02	0,04	0	0,96	0	0,048
2,75	7324	0	0,04	0,03	0	0,88	0	0,05	0	0	0,3	0,65	0,05	0	0,048
2,75	11774	0,06	0	0,01	0	0,81	0,11	0	0,01	0	0,82	0,06	0,01	0,11	0,048
2,75	9595	0,01	0	0	0,12	0	0,87	0	0	0	0,74	0	0,14	0,12	0,038
2,75	22425	0,03	0	0,04	0	0,02	0,7	0	0,2	0,01	0,22	0,05	0,01	0,72	0,034
2,75	12487	0,01	0	0	0,06	0,38	0	0,05	0,5	0	0,5	0,06	0,38	0,06	0,042
2,75	6589	0	0	0	0,06	0,93	0	0,01	0	0	0	0,93	0,06	0,01	0,048
2,75	15477	0	0	0	0,08	0,79	0,1	0	0,03	0	0,07	0,75	0,06	0,12	0,04
2,75	16839	0	0,01	0	0,11	0,87	0	0	0,01	0	0,27	0	0,73	0	0,048
2,75	28036	0	0	0	0,21	0,01	0,77	0	0,01	0	0,77	0,02	0,21	0	0,048
2,75	14176	0,02	0,03	0	0,04	0,88	0,03	0	0	0	0,05	0,04	0,02	0,89	0,034
2,75	20521	0	0	0	0,21	0,7	0,02	0	0,06	0,01	0,69	0,21	0,08	0,02	0,036
2,75	17461	0,05	0	0	0,01	0,15	0,75	0	0,04	0	0,79	0,05	0,01	0,15	0,048
2,75	4304	0	0,09	0,01	0	0,03	0	0,05	0,81	0,01	0,03	0,05	0,09	0,83	0,048
2,75	18804	0,05	0	0	0	0,35	0	0	0,6	0	0,6	0	0	0,4	0,044
2,75	9745	0,06	0	0	0	0,92	0,01	0	0	0,01	0,01	0	0,98	0,01	0,044
2,75	24782	0	0,01	0	0,1	0,87	0,01	0	0	0,01	0,04	0	0,95	0,01	0,046
2,75	19481	0	0,19	0	0	0	0,12	0	0,69	0	0	0	0,72	0,28	0,048
2,75	6140	0,05	0	0	0	0	0,76	0	0,16	0,03	0,01	0,1	0,68	0,21	0,048
2,75	12427	0,03	0	0,01	0,02	0,92	0,01	0,01	0	0	0,05	0,03	0,92	0	0,04
3	7574	0,01	0,01	0,04	0	0,94	0	0	0	0	0,01	0,04	0,95	0	0,022
3	25249	0	0	0	0,07	0,93	0	0	0	0	0,07	0,93	0	0	0,044

3	15512	0,04	0,01	0	0,01	0,93	0,01	0	0	0	0	0,01	0,04	0,95	0,044
3	26449	0	0	0	0,06	0,94	0	0	0	0	0	1	0	0	0,046
3	19145	0,06	0	0	0	0	0	0	0,91	0,03	0,03	0,96	0	0,01	0,048
3	21546	0,06	0	0	0	0	0	0	0,94	0	0	0	1	0	0,044
3	11685	0	0,13	0	0	0,83	0	0	0,04	0	0	0,04	0,96	0	0,046
3	18877	0,04	0	0	0,03	0	0,91	0	0,02	0	0,02	0,07	0	0,91	0,042
3	12749	0	0,09	0	0	0,89	0	0	0,02	0	0,95	0,02	0,03	0	0,042
3	19110	0,06	0	0	0	0,93	0	0	0	0,01	0,94	0	0,06	0	0,044
3	9553	0,02	0,33	0	0	0	0	0	0,65	0	0	0,01	0,12	0,87	0,048
3	30757	0,06	0,01	0	0	0,11	0	0	0,82	0	0,02	0,06	0	0,92	0,048
3	38669	0	0	0	0,12	0,87	0	0	0,01	0	0,06	0,12	0,81	0,01	0,044
3	12333	0	0,21	0	0,02	0	0	0,01	0,76	0	0,21	0,02	0,01	0,76	0,048
3	8206	0,06	0	0,01	0	0,55	0,02	0	0,36	0	0,26	0,02	0,02	0,7	0,044
3	18647	0	0	0	0,07	0,91	0,02	0	0	0	0	0,93	0,07	0	0,044
3	44302	0	0	0,04	0,08	0,88	0	0	0	0	0	0,03	0,69	0,28	0,046
3	15268	0	0,06	0	0,03	0,9	0	0	0	0,01	0,95	0	0,02	0,03	0,046
3	31052	0	0	0,04	0	0,01	0,94	0,01	0	0	0,04	0	0,96	0	0,026
3	31862	0	0	0	0,05	0,94	0	0	0	0,01	0	0	0,06	0,94	0,048
3	12207	0	0	0	0,04	0	0	0,01	0,95	0	0	0,05	0	0,95	0,042
3	17297	0,04	0,05	0	0	0,91	0	0	0	0	0,05	0,04	0,82	0,09	0,048
3	8099	0,05	0	0	0	0	0,83	0	0,12	0	0,12	0,02	0,86	0	0,046
3	17623	0	0,01	0	0,14	0,33	0	0	0,52	0	0,33	0,02	0,64	0,01	0,044
3	24611	0,01	0,66	0	0	0,01	0	0	0,31	0,01	0	0,68	0,04	0,28	0,048
3	8066	0	0,22	0	0	0,76	0	0	0,02	0	0	0,78	0,19	0,03	0,042
3	24887	0,02	0	0,04	0	0,07	0,05	0	0,82	0	0	0	0,82	0,18	0,036
3	21455	0	0	0	0,22	0,74	0,04	0	0	0	0,74	0,04	0,22	0	0,044
3	4339	0,03	0	0,03	0,02	0,92	0	0	0	0	0,02	0,03	0,95	0	0,048
3	13841	0	0,23	0	0	0,75	0	0	0,02	0	0,2	0,77	0	0,03	0,042
3	21840	0	0,08	0	0	0,9	0	0	0,02	0	0,98	0	0,02	0	0,044
3	27875	0,06	0	0	0	0	0,93	0,01	0	0	1	0	0	0	0,046
3	7621	0,05	0,05	0	0	0	0	0	0,9	0	0,05	0,3	0,27	0,38	0,048
3	43028	0	0	0	0,12	0,86	0,02	0	0	0	0,02	0	0,86	0,12	0,048
3	5921	0,02	0,05	0	0	0	0	0,04	0,89	0	0	0,91	0,09	0	0,046

3	15259	0,02	0,97	0	0	0,01	0	0	0	0	0	0	0,02	0,98	0,048
3	41650	0	0,02	0	0,05	0,93	0	0	0	0	0	0,95	0,05	0	0,04
3	26849	0,05	0	0,01	0	0,93	0	0,01	0	0	0	0,95	0	0,05	0,046
3	21991	0,05	0	0,02	0	0,15	0,78	0	0	0	0,02	0,15	0,8	0,03	0,044
3	8979	0	0,95	0	0,04	0	0,01	0	0	0	0	0,01	0	0,99	0,044
3	54519	0	0,04	0	0,02	0,89	0,01	0	0	0,04	0,75	0,15	0,1	0	0,046
3	9367	0,05	0,01	0	0	0,92	0,02	0	0	0	0,88	0,02	0,05	0,05	0,048
3	23833	0,05	0	0	0,04	0,89	0,02	0	0	0	0,91	0,09	0	0	0,046
3	18271	0	0	0,04	0	0,08	0	0	0,88	0	0	0,12	0	0,88	0
3	47160	0	1	0	0	0	0	0	0	0	0,07	0	0	0,93	0,048
3	12548	0	0	0	0,03	0	0,8	0,17	0	0	0,83	0	0	0,17	0,048
3	15879	0	0	0	0,08	0,92	0	0	0	0	0,27	0	0	0,73	0,044
3	40449	0	0,71	0	0	0,19	0	0	0,1	0	0	0	0,11	0,89	0,048
3	28058	0,01	0,97	0	0	0	0	0	0,02	0	0	0	0,03	0,97	0,044
3	18988	0,06	0	0,01	0	0,93	0	0	0	0	0,01	0,06	0,01	0,92	0,044
3	29024	0	0,22	0	0	0,78	0	0	0	0	0	0,78	0,22	0	0,046
3	11569	0,06	0	0	0,03	0,1	0,1	0	0,01	0,7	0,7	0	0,28	0,02	0,044
3	14976	0,01	0	0,03	0,08	0,7	0,18	0	0	0	0,7	0	0,11	0,19	0,042
3	14965	0,05	0	0	0,03	0,37	0,55	0	0	0	0,63	0,37	0	0	0,048
3	12681	0	0	0	0,06	0,94	0	0	0	0	0	1	0	0	0,048
3	13002	0,06	0	0	0	0,74	0,08	0	0,12	0	0,08	0,12	0,74	0,06	0,044
3	19614	0,03	0	0,04	0	0,9	0	0,03	0	0	0,03	0,94	0,03	0	0,046
3	20642	0,02	0	0	0,04	0,03	0	0	0,9	0,01	0	0,02	0,04	0,94	0,048
3	21124	0,06	0	0,02	0	0,85	0,07	0	0	0	0,02	0,85	0,07	0,06	0,048
3	10104	0,02	0	0,04	0	0,91	0	0,03	0	0	0	0,05	0,93	0,02	0,044
3	4822	0,05	0	0,01	0	0,9	0,01	0	0,03	0	0,91	0,01	0	0,08	0,048
3	8839	0,05	0,18	0	0	0,76	0,01	0	0	0	0	0	0	1	0,048
3	24984	0	0,53	0	0	0,47	0	0	0	0	0	0,02	0,21	0,77	0,048
3	20937	0	0,39	0	0	0	0,61	0	0	0	0	0,39	0	0,61	0,042
3	37137	0	0	0	0,21	0,71	0	0	0,08	0	0,88	0	0	0,12	0,04
3	9870	0,02	0	0,04	0,02	0	0,86	0	0,06	0	0,94	0,04	0,02	0	0,046
3	5588	0,06	0	0	0	0	0,39	0	0,55	0	0,52	0,44	0	0,04	0,036
3	13588	0,04	0	0	0	0	0,64	0	0,32	0	0,04	0	0,64	0,32	0,048

3	15397	0	0,1	0,01	0,03	0,85	0,01	0	0	0	0,03	0	0,96	0,01	0,04
3	59215	0,05	0	0,04	0	0	0	0	0,91	0	0,08	0,04	0	0,88	0,046
3	9878	0	0	0,04	0,01	0,6	0	0	0,35	0	0,01	0,64	0,35	0	0,018
3	18120	0	0,05	0	0,06	0,76	0,07	0,02	0,02	0,02	0,07	0,06	0,76	0,11	0,048
3	65490	0,05	0	0	0	0,16	0,74	0	0,03	0,02	0,03	0,08	0,89	0	0,034
3	12173	0	0,11	0	0,03	0,86	0	0	0	0	0,11	0,86	0	0,03	0,046
3	9245	0,06	0	0	0	0,93	0,01	0	0	0	0,01	0,93	0,06	0	0,048
3	12660	0	0,9	0	0	0,01	0,06	0	0	0,03	0,01	0	0,03	0,96	0,048
3	24950	0	0,95	0	0	0	0	0	0	0,05	0	0	0,05	0,95	0,046
3	45537	0	0,99	0	0,01	0	0	0	0	0	0,01	0,68	0	0,31	0,034
3	8812	0	0	0	0,42	0,58	0	0	0	0	1	0	0	0	0,04
3	19338	0,02	0,92	0	0,02	0,04	0	0	0	0	0	0,06	0	0,94	0,046
3	19783	0	0	0	0,23	0,12	0,65	0	0	0	0,77	0,23	0	0	0,048
3	27945	0,02	0	0	0,06	0,54	0,16	0	0,22	0	0,22	0,3	0	0,48	0,048
3	24035	0,01	0	0,04	0,02	0,92	0	0	0,01	0	0,21	0,04	0,02	0,73	0,03
3	21656	0	0	0	0,07	0,91	0	0	0	0,02	0	0	0,07	0,93	0,042
3	22367	0,03	0,05	0	0	0,5	0,13	0	0,29	0	0,82	0	0,18	0	0,046
3	3498	0,01	0,11	0,04	0	0	0	0	0,84	0	0,06	0,46	0,3	0,18	0,048
3	17489	0,02	0	0	0,07	0,9	0	0	0	0,01	0,01	0,07	0,92	0	0,046
3	43038	0,01	0,95	0	0,02	0	0	0	0	0,02	0	0,02	0,03	0,95	0,048
3	5618	0,05	0,01	0,01	0	0,92	0	0	0,01	0	0	0,93	0,06	0,01	0,046
3	21357	0	0,17	0	0,01	0,61	0,18	0,01	0,02	0	0,09	0,01	0,26	0,64	0,044
3	13511	0,01	0	0,04	0,01	0,9	0,04	0	0	0	0,01	0,05	0,94	0	0,034
3	57829	0,02	0	0	0	0,84	0	0,05	0,02	0,07	0,04	0,02	0,07	0,87	0,044
3	4704	0,04	0,06	0	0	0,77	0	0	0,12	0,01	0,07	0,01	0,77	0,15	0,048
3	27194	0	0	0	0,12	0	0,88	0	0	0	0	0	1	0	0,044
3	9583	0	0	0	0,06	0,93	0	0	0,01	0	0	0,18	0	0,82	0,048
3	9476	0	0,13	0	0	0,87	0	0	0	0	0,25	0,75	0	0	0,046
3	12504	0,03	0	0	0,02	0	0,9	0,05	0	0	0,81	0,09	0,03	0,07	0,046
3	17824	0	0	0	0	0,91	0	0,06	0,01	0,02	0,02	0,01	0	0,97	0,048
3	23557	0	0,06	0	0,07	0,03	0,83	0	0,01	0	0	0,08	0,09	0,83	0,048
3	11169	0,05	0,01	0	0	0	0,55	0	0,39	0	0	0,41	0,58	0,01	0,044
3,25	18922	0,03	0	0,03	0	0,92	0	0,02	0	0	0,01	0,03	0,02	0,94	0,042

3,25	7513	0,07	0	0	0	0,91	0	0,01	0	0,01	0	0,02	0,95	0,03	0,048
3,25	18155	0	0	0	0	0,14	0,71	0,12	0,01	0,02	0,41	0,13	0,04	0,42	0,044
3,25	29315	0,02	0	0	0,14	0,8	0,02	0,02	0	0	0,93	0,02	0,01	0,04	0,04
3,25	10428	0	0,09	0	0,02	0,88	0	0	0	0,01	0,8	0,18	0,02	0	0,048
3,25	16376	0	0,3	0	0	0,7	0	0	0	0	0,3	0	0	0,7	0,042
3,25	16746	0	0,97	0	0,02	0	0	0,01	0	0	0	0	0,02	0,98	0,048
3,25	16105	0	0	0	0,25	0	0	0	0,75	0	0,75	0,25	0	0	0,044
3,25	19190	0	0,73	0	0	0,06	0,21	0	0	0	0,02	0,01	0	0,97	0,04
3,25	11499	0	0	0,04	0	0,8	0,16	0	0	0	0,1	0	0,86	0,04	0,022
3,25	35292	0	0,26	0,02	0	0,66	0	0	0,04	0,02	0,02	0	0,28	0,7	0,048
3,25	15610	0	0,1	0	0	0,87	0	0	0,03	0	0,01	0,1	0,87	0,02	0,048
3,25	16345	0	0	0	0,13	0,87	0	0	0	0	0,31	0,69	0	0	0,044
3,25	78587	0,02	0,01	0,04	0	0,91	0	0,02	0	0	0,03	0,06	0	0,91	0,042
3,25	9512	0,05	0	0	0	0,81	0,12	0,01	0	0,01	0,07	0,81	0,04	0,08	0,048
3,25	10588	0,02	0,02	0	0,09	0,76	0,09	0	0,02	0	0,56	0,07	0	0,37	0,046
3,25	10439	0	0	0	0,06	0,9	0,03	0	0,01	0	0,01	0,9	0,04	0,05	0,048
3,25	10783	0,04	0,04	0	0	0,92	0	0	0	0	0,04	0,92	0,04	0	0,044
3,25	10681	0,07	0	0	0	0,02	0,02	0	0,89	0	0,02	0,02	0	0,96	0,046
3,25	8873	0,05	0,01	0	0,03	0,47	0,42	0	0,02	0	0,28	0,47	0	0,25	0,046
3,25	31437	0,06	0	0,02	0	0,92	0	0	0	0	0,08	0,9	0,02	0	0,04
3,25	10655	0	0,3	0	0	0,53	0	0	0,15	0,02	0,03	0	0,3	0,67	0,044
3,25	17243	0	0	0	0,08	0,92	0	0	0	0	0,01	0,91	0,08	0	0,044
3,25	19947	0	0,26	0,04	0	0,7	0	0	0	0	0	0	0,3	0,7	0,048
3,25	49806	0	0,03	0	0,06	0	0	0	0,91	0	0,01	0,9	0,03	0,06	0,044
3,25	10522	0,04	0	0	0,05	0,84	0	0	0,07	0	0,04	0	0,84	0,12	0,048
3,25	14449	0	0,1	0	0,03	0,87	0	0	0	0	0,1	0,03	0,77	0,1	0,044
3,25	27097	0,04	0,04	0,04	0,02	0,78	0,02	0	0,06	0	0,04	0,84	0,08	0,04	0,044
3,25	14928	0,01	0,03	0	0,09	0,12	0	0	0,75	0	0,04	0	0,75	0,21	0,048
3,25	10932	0,05	0,01	0	0	0,93	0	0	0,01	0	0,93	0,05	0,02	0	0,046
3,25	19003	0	0	0	0	0,13	0,54	0,09	0,24	0	0,03	0,36	0,37	0,24	0,04
3,25	9809	0	0,17	0	0	0,82	0,01	0	0	0	0	0,17	0,5	0,33	0,042
3,25	5699	0	0	0,04	0,05	0,15	0,11	0	0,65	0	0	0,76	0	0,24	0,04
3,25	12139	0,04	0	0	0	0,61	0	0,02	0,33	0	0	0,56	0,41	0,03	0,046

3,25	20506	0,02	0,24	0	0,02	0,71	0	0	0,01	0	0,04	0,24	0,01	0,71	0,048
3,25	12778	0	0	0	0,22	0,77	0	0	0	0,01	0,8	0,01	0,15	0,04	0,048
3,25	3509	0	0	0	0	0,87	0	0,13	0	0	0,13	0	0,87	0	0,036
3,25	21059	0	0	0,04	0	0,93	0,02	0,01	0	0	0,21	0,73	0,02	0,04	0,024
3,25	30970	0	0,07	0	0,05	0,77	0	0	0,11	0	0,11	0	0,05	0,84	0,042
3,25	32310	0	0,2	0	0	0,17	0	0	0,63	0	0	0,63	0,2	0,17	0,044
3,25	4811	0,03	0,02	0	0,01	0,85	0,06	0,03	0	0	0,58	0	0,01	0,41	0,034
3,25	4854	0,05	0	0,01	0	0,26	0,16	0	0,52	0	0,05	0,82	0,13	0	0,046
3,25	15362	0	0,15	0	0,02	0,83	0	0	0	0	0,15	0,83	0	0,02	0,036
3,25	45652	0	0,05	0	0,1	0,85	0	0	0	0	0,85	0,05	0	0,1	0,044
3,25	29773	0,01	0	0,04	0,04	0,77	0	0	0,06	0,08	0,1	0,85	0,05	0	0,046
3,25	30791	0,04	0	0	0,06	0	0,88	0,01	0,01	0	0,94	0	0,06	0	0,048
3,25	15907	0,03	0	0	0,05	0,21	0,68	0	0	0,03	0,04	0,18	0,63	0,15	0,042
3,25	22950	0,05	0	0	0	0,68	0,27	0	0	0	0	0,14	0,73	0,13	0,04
3,25	4839	0	0	0,04	0,01	0,72	0	0	0,23	0	0,23	0,23	0,54	0	0,028
3,25	4834	0,05	0	0,03	0	0,92	0	0	0	0	0,01	0,96	0	0,03	0,044
3,25	18688	0,04	0	0	0,01	0,02	0	0	0,93	0	0,45	0,52	0	0,03	0,032
3,25	8627	0,05	0,01	0	0,01	0,83	0	0	0,1	0	0,13	0,01	0,83	0,03	0,042
3,25	8251	0,04	0,06	0	0	0,66	0	0,01	0,23	0	0,01	0,93	0,06	0	0,044
3,25	19657	0,01	0,02	0,04	0	0,03	0,86	0,04	0	0	0	0,07	0,89	0,04	0,048
3,25	34240	0	0,07	0	0,03	0,88	0,02	0	0	0	0,88	0	0,09	0,03	0,048
3,25	18050	0	0	0,04	0	0,79	0	0	0,17	0	0	0	0,04	0,96	0
3,25	30858	0	0	0,02	0,06	0	0,01	0,01	0,89	0,01	0	0,03	0,14	0,83	0,048
3,25	15545	0	0,28	0	0,04	0,68	0	0	0	0	0,14	0	0	0,86	0,048
3,25	33955	0	0,08	0	0,05	0,87	0	0	0	0	0	0,87	0,05	0,08	0,046
3,25	9274	0	0,07	0,01	0	0,89	0,03	0	0	0	0,81	0,12	0,07	0	0,044
3,25	29206	0	0,17	0	0,01	0,81	0	0	0	0,01	0,18	0,81	0	0,01	0,048
3,25	54729	0	0,15	0	0	0,8	0,03	0	0,02	0	0,04	0,15	0,8	0,01	0,044
3,25	15831	0,05	0	0	0,01	0	0,01	0	0,93	0	0	0,84	0,04	0,12	0,048
3,25	42123	0,06	0	0	0	0,94	0	0	0	0	0	0,96	0,04	0	0,048
3,25	14373	0	0,07	0	0,05	0,82	0,06	0	0	0	0,07	0	0,05	0,88	0,044
3,25	11630	0	1	0	0	0	0	0	0	0	0	0	0,07	0,93	0,046
3,25	9225	0,03	0,04	0	0	0,92	0	0	0	0,01	0	0,12	0,81	0,07	0,04

3,25	10077	0,03	0	0,04	0,02	0	0,02	0	0,89	0	0,01	0,04	0	0,95	0,044
3,25	13924	0	0,94	0	0	0	0,03	0	0,03	0	0,03	0	0,03	0,94	0,044
3,25	17191	0,05	0,03	0	0	0,67	0,25	0	0	0	0,05	0	0,25	0,7	0,044
3,25	22167	0,05	0	0	0	0,1	0	0	0,2	0,65	0,84	0,05	0,01	0,1	0,044
3,25	6740	0,04	0,02	0	0	0,92	0	0,02	0	0	0,02	0,02	0	0,96	0,042
3,25	22200	0	0,1	0	0,05	0,82	0	0	0,01	0,02	0	0,34	0	0,66	0,048
3,25	4643	0,01	0	0	0	0,01	0,05	0,18	0,75	0	0,68	0,08	0	0,24	0,048
3,25	13843	0,06	0	0	0	0,34	0	0,01	0,59	0	0,07	0	0	0,93	0,048
3,25	18859	0	0,2	0,02	0	0,78	0	0	0	0	0	0,78	0,22	0	0,048
3,25	6296	0,04	0,01	0,01	0,01	0,48	0,4	0	0	0,05	0,13	0,19	0,37	0,31	0,048
3,25	6919	0,06	0,01	0	0	0,93	0	0	0	0	0,45	0	0	0,55	0,046
3,25	19598	0	0,22	0	0	0,77	0	0	0	0,01	0	0,78	0,22	0	0,044
3,25	21609	0,04	0,04	0,01	0	0	0	0	0,91	0	0	0	0,95	0,05	0,046
3,25	6277	0,03	0	0	0,06	0,85	0	0,02	0	0,04	0,85	0,05	0,05	0,05	0,038
3,25	15183	0,06	0	0	0,01	0,21	0,66	0	0,05	0,01	0,67	0,06	0,06	0,21	0,042
3,25	50011	0	0	0	0,12	0,87	0,01	0	0	0	0,13	0	0,86	0,01	0,038
3,25	10172	0,01	0,15	0	0,01	0	0	0	0,83	0	0	0,84	0,15	0,01	0,048
3,25	14558	0	0,07	0	0	0,93	0	0	0	0	0,93	0	0,05	0,02	0,04
3,25	20327	0	0,01	0	0,2	0,75	0,04	0	0	0	0,79	0,2	0	0,01	0,044
3,25	9738	0,06	0	0	0	0,8	0,01	0	0,13	0	0,37	0,44	0,13	0,06	0,044
3,25	23515	0	0,27	0	0	0,66	0	0	0,07	0	0,07	0	0,27	0,66	0,048
3,25	6007	0,01	0,11	0,02	0	0,83	0	0,02	0	0,01	0,02	0,85	0,12	0,01	0,048
3,25	19580	0,05	0	0,03	0	0,89	0,03	0	0	0	0,06	0,03	0,85	0,06	0,044
3,25	13806	0,06	0	0	0	0,61	0,06	0	0,26	0,01	0,87	0,01	0	0,12	0,04
3,25	4352	0,06	0	0	0	0,01	0,86	0	0,07	0	0,06	0	0,08	0,86	0,048
3,25	8852	0	0	0	0,33	0,63	0,03	0	0	0,01	0,96	0,01	0	0,03	0,048
3,25	10467	0	0,61	0	0,02	0,26	0,09	0	0,02	0	0,02	0,11	0	0,87	0,046
3,25	11011	0	0	0	0,07	0,93	0	0	0	0	0,07	0	0,02	0,91	0,048
3,25	14427	0,04	0	0	0	0,92	0,01	0	0,03	0	0,39	0,01	0,58	0,02	0,044
3,25	25421	0,01	0	0,01	0,06	0,92	0	0	0	0	0,07	0,93	0	0	0,048
3,25	13693	0	0	0,01	0,11	0,88	0	0	0	0	0	0,01	0,99	0	0,034
3,25	13192	0	0	0	0,1	0,87	0	0	0,03	0	0,11	0,87	0,02	0	0,048
3,25	16197	0,01	0,04	0,02	0,03	0,68	0,21	0	0	0,01	0,89	0,05	0,01	0,05	0,042

3,5	14192	0,04	0	0	0,05	0	0,91	0	0	0	0,91	0	0,09	0	0,044
3,5	32680	0	0,97	0	0	0	0	0,03	0	0	0	0,01	0,02	0,97	0,048
3,5	15388	0,05	0	0	0	0,01	0,77	0,01	0,01	0,15	0,14	0	0,79	0,07	0,042
3,5	17634	0,05	0	0	0	0,82	0	0,01	0,12	0	0	0,86	0,05	0,09	0,046
3,5	21185	0	0	0	0,07	0,08	0	0,03	0,82	0	0,1	0,85	0,05	0	0,048
3,5	23519	0,04	0	0	0,03	0	0,03	0	0	0,9	0,01	0	0,05	0,94	0,046
3,5	34448	0	0	0	0,06	0,84	0,02	0	0,08	0	0,07	0,02	0,06	0,85	0,042
3,5	14437	0,01	0,01	0	0,06	0,92	0	0	0	0	0	0,92	0,07	0,01	0,046
3,5	24622	0	0,05	0,04	0	0,05	0,85	0	0	0,01	0	0,06	0,85	0,09	0,038
3,5	18799	0,01	0	0,03	0,08	0,75	0	0	0,13	0	0,14	0	0,78	0,08	0,048
3,5	17579	0	0,03	0,04	0,01	0,81	0,06	0	0,05	0	0,01	0,14	0	0,85	0,02
3,5	14560	0	0,08	0	0	0,71	0,19	0,02	0	0	0,71	0,05	0,19	0,05	0,044
3,5	26243	0,05	0,02	0	0	0,85	0,02	0	0,06	0	0,9	0,03	0	0,07	0,048
3,5	17135	0,06	0	0	0	0,93	0	0	0	0,01	0,99	0,01	0	0	0,04
3,5	15656	0,02	0,08	0	0,03	0	0,01	0	0,61	0,25	0,08	0,1	0,18	0,64	0,036
3,5	41911	0	0	0,01	0,06	0,92	0	0	0	0,01	0	0,97	0,01	0,02	0,048
3,5	24017	0,05	0	0	0	0	0,01	0	0,94	0	0,01	0,89	0	0,1	0,036
3,5	26502	0,01	0	0,04	0	0,91	0	0	0,02	0,02	0,02	0,89	0,04	0,05	0,012
3,5	35533	0,03	0	0,03	0	0,82	0,02	0,02	0,03	0,05	0,87	0,1	0,03	0	0,046
3,5	10813	0,03	0	0,04	0	0,85	0,08	0	0	0	0,09	0,17	0,04	0,7	0,038
3,5	16335	0	0	0	0,14	0,84	0,01	0	0,01	0	0,15	0,11	0,74	0	0,048
3,5	13887	0	0,08	0	0,07	0,85	0	0	0	0	0,06	0,85	0,07	0,02	0,048
3,5	15508	0,05	0	0,01	0	0	0,09	0	0,85	0	0	0,02	0,08	0,9	0,044
3,5	27864	0,01	0	0	0,23	0,09	0,67	0	0	0	0,94	0,01	0,05	0	0,042
3,5	13626	0,03	0	0,01	0	0,93	0	0,03	0	0	0,9	0	0,04	0,06	0,032
3,5	11980	0	0,13	0	0	0,85	0	0,02	0	0	0,02	0,02	0,83	0,13	0,048
3,5	36849	0	0,08	0	0,07	0,23	0,62	0	0	0	0,07	0,08	0,09	0,76	0,046
3,5	13624	0	0	0	0,26	0	0	0	0,74	0	0,73	0,26	0,01	0	0,034
3,5	32523	0	0	0	0,22	0,78	0	0	0	0	0,78	0,22	0	0	0,046
3,5	34277	0	0,24	0	0	0,71	0,05	0	0	0	0	0,52	0,29	0,19	0,046
3,5	36393	0	0,16	0	0,02	0,78	0	0,04	0	0	0,02	0	0,04	0,94	0,038
3,5	11711	0	0	0	0,05	0,89	0	0,04	0,02	0	0,04	0,93	0,02	0,01	0,046
3,5	67077	0,06	0	0	0	0	0,94	0	0	0	1	0	0	0	0,048

3,5	5475	0	0,03	0	0,06	0,1	0	0	0,81	0	0	0,26	0,08	0,66	0,044
3,5	21673	0,01	0	0	0,15	0,65	0,19	0	0	0	0,65	0,19	0,16	0	0,048
3,5	6424	0	0	0	0,17	0	0,65	0	0,18	0	0,35	0,65	0	0	0,036
3,5	21671	0,01	0,07	0	0	0,88	0,02	0	0,02	0	0,91	0,02	0	0,07	0,046
3,5	28548	0	0	0	0,19	0,81	0	0	0	0	0,81	0	0,19	0	0,046
3,5	17434	0	0,07	0	0,02	0,9	0	0,01	0	0	0,91	0	0,01	0,08	0,048
3,5	11609	0,05	0	0	0	0,95	0	0	0	0	0	0,05	0,95	0	0,046
3,5	5013	0,05	0	0	0	0,26	0	0	0,62	0,07	0	0,04	0,92	0,04	0,046
3,5	18163	0,05	0	0	0,01	0,89	0,04	0,01	0	0	0,95	0,05	0	0	0,044
3,5	16618	0,05	0	0,03	0	0,06	0,08	0	0,78	0	0	0,89	0,06	0,05	0,04
3,5	5753	0,04	0	0,02	0,02	0,89	0	0	0,03	0	0,92	0,02	0,04	0,02	0,046
3,5	5391	0	0,21	0	0	0,78	0	0	0	0,01	0,29	0,03	0,19	0,49	0,048
3,5	15389	0	0	0	0,21	0,77	0	0,02	0	0	0,8	0,2	0	0	0,042
3,5	16274	0	0,01	0,04	0	0,35	0	0	0,6	0	0,61	0	0	0,39	0,026
3,5	18902	0,06	0	0	0	0	0	0	0,94	0	0	0	0,94	0,06	0,046
3,5	43507	0,01	0	0	0,09	0,64	0	0	0,26	0	0,63	0,03	0,08	0,26	0,048
3,5	10060	0,06	0	0,01	0	0,93	0	0	0	0	0	0,24	0,75	0,01	0,048
3,5	24061	0	0,29	0	0	0,7	0	0	0,01	0	0,01	0,05	0,24	0,7	0,042
3,5	12558	0	0	0,02	0,15	0,79	0	0	0,01	0,03	0,76	0,22	0	0,02	0,042
3,5	17671	0	0,03	0	0	0	0	0,08	0,89	0	0	0,08	0,92	0	0,048
3,5	18323	0,04	0,04	0	0	0	0,73	0	0,19	0	0,19	0,04	0,73	0,04	0,048
3,5	16951	0	0	0	0,17	0	0,83	0	0	0	0,55	0	0,45	0	0,04
3,5	16590	0,01	0,04	0,04	0	0,85	0,05	0,01	0	0	0,11	0,04	0,85	0	0,046
3,5	42804	0	0,02	0,04	0	0,94	0	0	0	0	0	0,04	0,94	0,02	0,03
3,5	7754	0,05	0	0	0,04	0,91	0	0	0	0	0,96	0,04	0	0	0,048
3,5	23029	0,06	0	0	0	0,23	0,66	0,01	0,01	0,03	0	0,56	0,4	0,04	0,044
3,5	9784	0	0,13	0	0	0,73	0	0	0,14	0	0	0	0,86	0,14	0,046
3,5	23523	0,02	0,12	0	0	0,69	0	0	0,17	0	0,17	0,71	0,12	0	0,044
3,5	14128	0,05	0,06	0	0	0,8	0,01	0	0,08	0	0,09	0	0,05	0,86	0,048
3,5	18694	0	0,97	0	0,02	0	0	0	0	0,01	0,01	0,02	0	0,97	0,046
3,5	14094	0	0,94	0	0,06	0	0	0	0	0	0	0,07	0	0,93	0,048
3,5	9973	0,01	0	0,04	0	0,89	0	0,01	0	0,05	0,89	0,09	0,01	0,01	0,04
3,5	37801	0	0	0,02	0,17	0,78	0,03	0	0	0	0,81	0,02	0,15	0,02	0,042

3,5	16733	0	0,1	0,01	0	0,88	0	0	0	0,01	0	0	0,88	0,12	0,042
3,5	17747	0	0,04	0,02	0,03	0,22	0	0,02	0,67	0	0,07	0,02	0,67	0,24	0,046
3,5	18421	0,03	0	0	0,03	0,91	0,02	0,01	0	0	0,01	0,05	0,03	0,91	0,048
3,5	17074	0,04	0	0,01	0	0	0,94	0,01	0	0	0,94	0	0,05	0,01	0,048
3,5	23417	0	0	0	0,07	0,93	0	0	0	0	0,07	0,93	0	0	0,048
3,5	16200	0,01	0,19	0	0	0	0,02	0,03	0,75	0	0	0,78	0,02	0,2	0,044
3,5	35494	0,05	0	0	0	0,87	0,08	0	0	0	0,05	0,08	0,87	0	0,048
3,5	39150	0	0,1	0,04	0,04	0,82	0	0	0	0	0,1	0,2	0	0,7	0,048
3,5	12227	0	0,02	0	0,1	0,87	0	0	0,01	0	0,11	0,01	0,87	0,01	0,044
3,5	28640	0	0	0	0,23	0,73	0,04	0	0	0	0,77	0	0,23	0	0,048
3,5	42404	0	0	0	0,14	0,85	0	0	0,01	0	0,14	0	0,82	0,04	0,046
3,5	19320	0,06	0	0	0	0,01	0	0	0,91	0,02	0,03	0,94	0,03	0	0,044
3,5	23962	0,07	0	0	0	0,92	0	0	0	0,01	0,07	0,03	0,89	0,01	0,048
3,5	20382	0,05	0	0,02	0	0,91	0	0	0	0,02	0	0,04	0,91	0,05	0,042
3,5	21234	0	0	0,02	0,05	0,93	0	0	0	0	0	0,95	0	0,05	0,042
3,5	16033	0,03	0	0,04	0,03	0,78	0	0	0,12	0	0,02	0,82	0,16	0	0,042
3,5	46022	0	0	0	0,13	0	0,81	0	0,05	0,01	0,16	0	0,82	0,02	0,046
3,5	37701	0,01	0,08	0	0	0,91	0	0	0	0	0,67	0,09	0,24	0	0,048
3,5	18310	0,02	0,1	0	0	0	0,11	0,02	0,75	0	0	0,85	0,04	0,11	0,048
3,5	32482	0,01	0	0,04	0,01	0,87	0	0	0,07	0	0,01	0,05	0,07	0,87	0,024
3,5	20435	0	0,18	0	0	0,73	0	0,01	0,08	0	0,23	0,01	0,13	0,63	0,048
3,5	16073	0,02	0	0	0,05	0,93	0	0	0	0	0,05	0,95	0	0	0,048
3,5	27785	0	0	0,03	0,07	0,85	0	0,05	0	0	0,92	0,02	0,05	0,01	0,046
3,5	29873	0	0,07	0,01	0,05	0,86	0	0,01	0	0	0,08	0,86	0,01	0,05	0,046
3,5	8722	0,04	0,01	0,03	0	0,9	0,01	0,01	0	0	0,06	0,06	0,02	0,86	0,044
3,5	38591	0,05	0,01	0	0	0,15	0	0	0,79	0	0,01	0,79	0	0,2	0,038
3,5	32176	0	0,14	0	0	0,83	0,02	0	0,01	0	0,14	0,02	0,83	0,01	0,048
3,5	8800	0,04	0,03	0	0	0,92	0,01	0	0	0	0	0,03	0,93	0,04	0,048
3,5	8060	0,01	0,08	0,01	0,05	0,85	0	0	0	0	0,01	0,07	0,06	0,86	0,042
3,5	21954	0,01	0,27	0	0	0,72	0	0	0	0	0,01	0	0,27	0,72	0,048
3,5	20772	0	0,28	0	0	0,72	0	0	0	0	0	0	0,28	0,72	0,048
3,5	27225	0	0	0,04	0,06	0,9	0	0	0	0	0,06	0,9	0,03	0,01	0,042
3,5	27605	0,02	0,91	0	0	0,03	0	0	0,04	0	0	0	0,03	0,97	0,038

3,5	28760	0,04	0	0	0,01	0,94	0	0	0,01	0	0,01	0,04	0	0,95	0,042
3,75	36268	0,06	0	0	0	0,94	0	0	0	0	0,93	0,06	0	0,01	0,048
3,75	15403	0,04	0	0	0,03	0,84	0	0	0	0,09	0,11	0,69	0,2	0	0,038
3,75	89825	0	0	0,03	0,06	0,8	0,11	0	0	0	0,01	0,06	0,11	0,82	0,048
3,75	10611	0,04	0	0	0	0,92	0,02	0,02	0	0	0,03	0,92	0,04	0,01	0,044
3,75	25614	0	0	0	0,13	0,09	0,77	0,01	0	0	0,16	0,08	0,75	0,01	0,048
3,75	17315	0,07	0	0	0	0,77	0,01	0	0,15	0	0	0,76	0,15	0,09	0,048
3,75	17445	0,01	0,24	0	0	0,65	0,01	0,03	0,06	0	0	0,69	0,05	0,26	0,048
3,75	11963	0	0,22	0	0,05	0,72	0	0	0	0,01	0	0,22	0,05	0,73	0,048
3,75	6746	0,07	0,02	0	0	0,11	0	0	0,8	0	0	0,07	0,41	0,52	0,044
3,75	21233	0,03	0	0,03	0,02	0	0,92	0	0	0	0,05	0,03	0,92	0	0,044
3,75	30010	0,01	0,08	0,03	0	0,1	0	0	0,78	0	0,34	0,07	0,1	0,49	0,034
3,75	33708	0	0,11	0	0,04	0,84	0,01	0	0	0	0,1	0,84	0,05	0,01	0,042
3,75	11159	0,01	0	0	0,18	0,81	0	0	0	0	0,87	0,01	0,07	0,05	0,044
3,75	40456	0	0	0	0,12	0,88	0	0	0	0	0,12	0	0,88	0	0,044
3,75	8084	0,04	0,02	0,01	0	0	0,92	0	0	0,01	0,96	0,01	0,02	0,01	0,038
3,75	17572	0	0,18	0	0,01	0,79	0	0,02	0	0	0,2	0	0,01	0,79	0,048
3,75	48986	0	0,06	0,01	0,05	0,35	0,26	0	0,26	0,01	0,69	0,01	0,05	0,25	0,03
3,75	26599	0,03	0	0,02	0,02	0,57	0	0,02	0,34	0	0,36	0,57	0,07	0	0,048
3,75	30652	0	0,18	0	0	0,69	0	0	0,13	0	0,13	0,69	0,1	0,08	0,046
3,75	11205	0,03	0,3	0,04	0	0	0	0	0,63	0	0,01	0,34	0,03	0,62	0,046
3,75	7913	0,04	0	0	0	0,94	0	0,01	0	0,01	0	0,25	0,04	0,71	0,046
3,75	8302	0	0,22	0	0	0,73	0	0,04	0	0,01	0,05	0,04	0,01	0,9	0,042
3,75	8736	0	0	0,04	0	0,02	0,01	0	0,93	0	0,85	0,01	0,04	0,1	0,038
3,75	7033	0	0,07	0,01	0	0,9	0	0,01	0	0,01	0,91	0,01	0,08	0	0,048
3,75	9698	0,03	0	0,03	0	0,93	0	0,01	0	0	0,94	0,04	0	0,02	0,048
3,75	15588	0,06	0	0,01	0	0,04	0	0	0,86	0,03	0	0,03	0,87	0,1	0,042
3,75	20927	0	0,07	0	0	0,93	0	0	0	0	0,93	0,07	0	0	0,048
3,75	11479	0,01	0,91	0,03	0,01	0	0	0	0,04	0	0	0,01	0,02	0,97	0,048
3,75	12548	0,06	0,01	0	0	0	0,8	0	0,12	0,01	0,8	0,01	0,07	0,12	0,042
3,75	12369	0,05	0,02	0	0	0,93	0	0	0	0	0,93	0,05	0,01	0,01	0,046
3,75	11841	0,04	0	0	0,01	0,92	0,02	0,01	0	0	0	0,01	0,95	0,04	0,048
3,75	10661	0,01	0	0,03	0,06	0,68	0	0	0,22	0	0	0,74	0,26	0	0,036

3,75	55117	0	0	0	0,06	0,75	0	0	0,19	0	0	0,01	0,05	0,94	0,042
3,75	16322	0,04	0,04	0	0	0,92	0	0	0	0	0,49	0,47	0,04	0	0,042
3,75	21600	0	0,11	0	0	0,87	0,02	0	0	0	0,11	0	0,74	0,15	0,048
3,75	13431	0	0	0	0,09	0,91	0	0	0	0	0,09	0	0,4	0,51	0,048
3,75	6637	0,02	0	0,01	0,06	0,83	0	0,01	0,07	0	0,35	0,01	0,64	0	0,048
3,75	8431	0	0,12	0	0	0,88	0	0	0	0	0	0	1	0	0,042
3,75	14089	0,05	0	0	0,02	0,57	0,34	0	0,02	0	0,67	0,02	0,3	0,01	0,048
3,75	32672	0,01	0	0,04	0	0,86	0	0	0,09	0	0	0	0,13	0,87	0,016
3,75	11624	0,04	0,14	0	0	0,8	0	0,01	0,01	0	0	0,15	0	0,85	0,044
3,75	22388	0	0	0	0,05	0	0,76	0,03	0,16	0	0,08	0	0,16	0,76	0,046
3,75	4986	0,06	0,01	0	0	0,88	0,04	0	0	0,01	0,04	0,88	0,02	0,06	0,048
3,75	59750	0,01	0,08	0	0	0,88	0,01	0	0,02	0	0,89	0,03	0,08	0	0,046
3,75	9431	0,06	0	0	0	0,94	0	0	0	0	0,44	0	0	0,56	0,044
3,75	7807	0,05	0,06	0	0	0	0,02	0	0,87	0	0,04	0,02	0	0,94	0,038
3,75	28095	0	0	0	0,07	0,59	0,34	0	0	0	0	0,34	0,07	0,59	0,038
3,75	8715	0	0	0,04	0	0	0,09	0,05	0,82	0	0	0,07	0,07	0,86	0,042
3,75	10262	0,05	0	0	0	0	0	0	0,79	0,16	0	0,16	0	0,84	0,046
3,75	19923	0,04	0	0,01	0	0	0	0	0,95	0	0,95	0,03	0,01	0,01	0,04
3,75	22373	0,06	0,03	0,03	0	0,72	0,01	0	0,15	0	0	0,04	0,01	0,95	0,048
3,75	4613	0,05	0	0	0,01	0,3	0,2	0	0,43	0,01	0,01	0,01	0,06	0,92	0,048
3,75	19764	0	0	0,01	0,13	0,34	0,51	0	0,01	0	0,34	0,13	0,53	0	0,048
3,75	7427	0,05	0	0	0	0,12	0,83	0	0	0	0,83	0	0,15	0,02	0,046
3,75	12646	0	0	0,04	0,04	0,92	0	0	0	0	0,07	0	0,93	0	0,042
3,75	21824	0	0	0	0,13	0,87	0	0	0	0	0,12	0	0,87	0,01	0,042
3,75	8434	0,02	0	0	0	0,71	0,1	0,12	0,05	0	0,71	0,17	0	0,12	0,048
3,75	31721	0,02	0,06	0,01	0	0,91	0	0	0	0	0,06	0	0,94	0	0,048
3,75	6794	0,04	0,01	0,01	0	0,14	0	0	0,8	0	0,15	0,8	0	0,05	0,038
3,75	7036	0,06	0	0	0,01	0	0	0	0,92	0,01	0,01	0,04	0,88	0,07	0,04
3,75	11619	0,06	0	0	0	0	0	0	0,94	0	0	0	0	1	0,048
3,75	44116	0,05	0,04	0	0	0,1	0,69	0	0,12	0	0	0,15	0,68	0,17	0,046
3,75	18696	0,02	0,95	0	0,03	0	0	0	0	0	0	0	0	1	0,044
3,75	16306	0	0,68	0,03	0	0,06	0,01	0	0,22	0	0,03	0,25	0,05	0,67	0,048
3,75	7102	0,06	0	0	0	0	0	0	0,94	0	0,88	0,05	0,01	0,06	0,046

3,75	19096	0	0	0,04	0	0,02	0	0	0,94	0	0,96	0,04	0	0	0,032
3,75	64578	0	1	0	0	0	0	0	0	0	0	1	0	0	0,042
3,75	19217	0,03	0	0,03	0,04	0,89	0	0,01	0	0	0,94	0,03	0	0,03	0,048
3,75	13375	0	0	0,02	0,13	0,76	0,03	0	0,06	0	0,85	0	0	0,15	0,042
3,75	9974	0,03	0	0,04	0	0	0,1	0	0,83	0	0	0,11	0,83	0,06	0,048
3,75	24714	0	0	0	0,14	0,7	0	0	0,16	0	0,17	0,14	0,69	0	0,046
3,75	85555	0,02	0,18	0	0	0,36	0,42	0	0,02	0	0	0,16	0,28	0,56	0,044
3,75	11305	0,01	0,08	0	0	0,84	0	0,05	0	0,02	0,12	0,85	0,03	0	0,048
3,75	8605	0	0	0	0,07	0,84	0,01	0,08	0	0	0,57	0,28	0	0,15	0,046
3,75	37184	0	0,07	0	0,09	0,82	0	0	0,02	0	0,02	0,19	0,79	0	0,046
3,75	11259	0	0	0	0,06	0,92	0,02	0	0	0	0,04	0,02	0,02	0,92	0,046
3,75	30452	0	0	0	0,09	0	0	0	0,89	0,02	0	0	0,4	0,6	0,048
3,75	19158	0	0,19	0,01	0	0,63	0	0	0,16	0,01	0,2	0,6	0	0,2	0,044
3,75	15855	0	0,19	0	0	0,78	0	0	0,03	0	0,02	0,78	0,19	0,01	0,046
3,75	12280	0,01	0	0	0,05	0,78	0	0	0,16	0	0,16	0	0,01	0,83	0,046
3,75	10301	0,01	0,09	0,02	0	0,77	0	0	0,1	0,01	0,09	0,02	0,78	0,11	0,048
3,75	37169	0,03	0	0,02	0	0,88	0,03	0,02	0	0,02	0,03	0,02	0,93	0,02	0,048
3,75	19666	0,06	0	0	0	0,94	0	0	0	0	0	1	0	0	0,044
3,75	12242	0,03	0	0,02	0,01	0,41	0,02	0,04	0,47	0	0,3	0,66	0,02	0,02	0,048
3,75	10902	0	0	0,04	0	0,79	0,02	0	0,15	0	0,79	0,04	0,15	0,02	0,02
3,75	32186	0,05	0	0	0,01	0,89	0,03	0	0	0,02	0,05	0,05	0,9	0	0,046
3,75	31323	0,01	0,74	0	0	0	0	0,03	0,22	0	0	0,22	0,01	0,77	0,046
3,75	33837	0	0,92	0	0	0,06	0,02	0	0	0	0	0,1	0,06	0,84	0,038
3,75	23444	0,07	0	0	0	0	0	0	0,93	0	0,76	0	0	0,24	0,048
3,75	9555	0	0,76	0,02	0	0	0	0	0,21	0,01	0,01	0,1	0,05	0,84	0,046
3,75	7160	0,06	0	0	0	0,93	0,01	0	0	0	0,85	0,13	0,01	0,01	0,044
3,75	43926	0,01	0,1	0,01	0	0,88	0	0	0	0	0,01	0,09	0,88	0,02	0,038
3,75	25551	0,03	0,08	0	0	0,79	0,02	0,03	0	0,05	0,08	0	0,11	0,81	0,048
3,75	9074	0,03	0,01	0,02	0,01	0,93	0	0	0	0	0,66	0,02	0	0,32	0,046
3,75	30639	0	0,98	0	0	0	0	0	0	0,02	0,02	0	0,05	0,93	0,04
3,75	21291	0	0,19	0	0	0,79	0	0	0	0,02	0,19	0,79	0	0,02	0,046
3,75	5793	0,04	0,02	0,01	0	0	0	0	0,93	0	0,94	0	0,06	0	0,048
3,75	18502	0,02	0,05	0	0	0	0,77	0,04	0,12	0	0,06	0,88	0,04	0,02	0,048

3,75	12906	0,04	0	0,01	0	0	0,04	0	0,91	0	0,82	0,01	0,04	0,13	0,044
3,75	9105	0,05	0	0	0	0,32	0,62	0	0	0,01	0,02	0,37	0	0,61	0,046
4	57678	0,01	0	0,01	0,19	0,75	0	0,01	0	0,03	0,75	0,02	0,19	0,04	0,046
4	8014	0,01	0	0,03	0,07	0,82	0	0	0	0,07	0,01	0,16	0,83	0	0,044
4	29065	0,06	0	0,01	0	0,9	0,03	0	0	0	0,01	0,03	0,91	0,05	0,046
4	12597	0,04	0,02	0	0	0,93	0,01	0	0	0	0,74	0,21	0	0,05	0,048
4	23409	0	0	0	0,1	0,79	0,02	0	0,09	0	0,02	0	0,88	0,1	0,046
4	7409	0,04	0,01	0	0	0,92	0	0	0,03	0	0,01	0	0,92	0,07	0,048
4	31219	0,03	0	0,03	0	0,54	0	0,04	0,36	0	0,36	0,57	0,05	0,02	0,048
4	15470	0,04	0	0,02	0	0,02	0,84	0	0,05	0,03	0	0,02	0,93	0,05	0,046
4	10554	0	0	0	0,13	0,83	0	0	0,04	0	0,13	0,04	0,83	0	0,048
4	13915	0	0	0	0,09	0	0	0	0,91	0	0	0	0,91	0,09	0,046
4	8746	0,05	0	0	0	0,95	0	0	0	0	1	0	0	0	0,04
4	53136	0,03	0	0	0,01	0,94	0	0,02	0	0	0,03	0,01	0,96	0	0,036
4	6867	0,02	0	0,02	0,06	0,87	0,03	0	0	0	0,59	0,03	0,05	0,33	0,048
4	57257	0,06	0	0	0	0	0,94	0	0	0	0	0	0,94	0,06	0,048
4	13394	0,06	0	0	0	0	0,04	0	0,9	0	0	0	0,72	0,28	0,042
4	11579	0	0,04	0,02	0,08	0,76	0,09	0	0,01	0	0,78	0,02	0,15	0,05	0,046
4	11175	0,04	0	0,02	0,01	0,9	0,02	0	0	0,01	0,05	0,03	0,02	0,9	0,046
4	30141	0,01	0	0	0,07	0	0,2	0,01	0,71	0	0,01	0,71	0,28	0	0,048
4	35991	0	0	0	0	0	0	0,06	0,94	0	0	0,09	0	0,91	0,044
4	17652	0,05	0	0	0	0,93	0,02	0	0	0	0,93	0,01	0,02	0,04	0,046
4	34746	0,05	0	0,01	0	0,86	0,08	0	0	0	0,99	0,01	0	0	0,046
4	7577	0	0	0	0,06	0,88	0,04	0,01	0,01	0	0,08	0	0	0,92	0,046
4	21097	0,05	0	0	0,04	0	0,87	0	0,03	0,01	0,88	0,08	0	0,04	0,044
4	41688	0	0	0	0,06	0,78	0,16	0	0	0	0	0	0,06	0,94	0,044
4	9033	0,04	0,01	0,03	0	0,63	0	0	0,29	0	0,01	0,29	0,66	0,04	0,044
4	15539	0	0,01	0	0,09	0,7	0,1	0,01	0	0,09	0,02	0,09	0,89	0	0,044
4	7145	0	0	0	0,06	0,93	0,01	0	0	0	0	0,5	0	0,5	0,046
4	25218	0	0	0	0	0	0,86	0,14	0	0	0	0,05	0,95	0	0,048
4	33506	0,05	0	0,03	0	0,7	0	0	0,22	0	0,05	0,03	0,22	0,7	0,048
4	22416	0	0,12	0	0,04	0,82	0	0	0	0,02	0	0,82	0,17	0,01	0,042
4	12116	0,05	0,01	0	0	0,94	0	0	0	0	0,98	0	0	0,02	0,044

4	17603	0	0,24	0,02	0	0,49	0	0	0,25	0	0	0,52	0,21	0,27	0,036
4	10161	0	0,12	0,04	0	0,05	0	0	0,79	0	0,05	0,16	0	0,79	0,006
4	11849	0,08	0	0	0	0,84	0,08	0	0	0	0,92	0	0,08	0	0,042
4	18701	0,02	0,03	0	0,08	0,87	0	0	0	0	0,9	0,07	0,03	0	0,048
4	20110	0,03	0	0,02	0,09	0,78	0,08	0	0	0	0,88	0,05	0,02	0,05	0,046
4	12721	0	0	0	0,12	0,88	0	0	0	0	0,12	0	0,88	0	0,038
4	26678	0	0,28	0	0	0,62	0,01	0	0,09	0	0	0	0,38	0,62	0,046
4	15934	0,03	0	0,04	0,02	0,81	0,02	0	0,08	0	0,05	0,02	0,09	0,84	0,046
4	17764	0	0,08	0	0	0,92	0	0	0	0	0,84	0	0,06	0,1	0,042
4	20747	0,03	0	0	0,08	0,78	0,11	0	0	0	0,03	0	0,85	0,12	0,048
4	25056	0	0	0	0,29	0,71	0	0	0	0	0,92	0,08	0	0	0,048
4	38516	0	0	0,01	0,12	0,87	0	0	0	0	0	0,01	0,98	0,01	0,036
4	7959	0,05	0,02	0	0	0,12	0,8	0	0,01	0	0,8	0	0,2	0	0,048
4	25278	0	0	0	0,14	0,86	0	0	0	0	0,72	0	0	0,28	0,044
4	28312	0,03	0,02	0,01	0,02	0,89	0	0	0	0,03	0,03	0	0,05	0,92	0,048
4	53306	0	0,13	0	0	0,84	0,03	0	0	0	0	0	1	0	0,048
4	18931	0,02	0,06	0	0	0,89	0	0,02	0	0,01	0	0,06	0,8	0,14	0,036
4	15842	0,04	0,02	0,02	0	0,88	0	0,01	0,03	0	0	0,02	0,96	0,02	0,046
4	18267	0	0	0,01	0,08	0,6	0	0	0,31	0	0,31	0,08	0	0,61	0,042
4	20098	0	0	0,04	0,01	0	0	0	0,95	0	0	0	0,95	0,05	0,032
4	33774	0	0,23	0	0	0,75	0,01	0,01	0	0	0,24	0	0,07	0,69	0,048
4	87688	0	0,1	0	0,02	0,88	0	0	0	0	0	0,1	0,9	0	0,042
4	57468	0	0,05	0,02	0,01	0,91	0	0	0	0,01	0,82	0,01	0,16	0,01	0,048
4	9575	0,04	0,04	0	0	0,9	0,01	0	0	0,01	0,88	0	0,06	0,06	0,046
4	21921	0	0	0	0,2	0	0,79	0	0	0,01	0,8	0	0,2	0	0,044
4	9181	0,01	0,23	0	0	0,76	0	0	0	0	0,29	0	0	0,71	0,048
4	18915	0,01	0	0,02	0,06	0,91	0	0	0	0	0,01	0	0,99	0	0,044
4	19673	0	0,13	0	0	0,83	0,03	0	0,01	0	0,24	0	0,72	0,04	0,046
4	19717	0,03	0,03	0	0	0,11	0,83	0	0	0	0,83	0	0,03	0,14	0,042
4	32952	0	0,03	0,04	0	0,79	0,14	0	0	0	0,79	0,11	0,1	0	0,048
4	18409	0,06	0	0	0	0,89	0,04	0	0,01	0	0,63	0,05	0,26	0,06	0,048
4	4686	0,05	0,02	0,04	0	0	0	0	0,89	0	0	0,01	0,07	0,92	0,046
4	8661	0,04	0	0,01	0,02	0	0	0	0,93	0	0	0,02	0,94	0,04	0,046

4	8330	0	0,08	0,03	0	0,74	0,13	0	0,01	0,01	0,13	0,03	0,84	0	0,038
4	16552	0,05	0	0	0,01	0,94	0	0	0	0	0	0	0,94	0,06	0,046
4	38080	0	0,23	0	0	0,74	0	0,01	0	0,02	0,26	0	0	0,74	0,044
4	18121	0	0	0	0,17	0	0,15	0	0,68	0	0,57	0,31	0	0,12	0,048
4	10505	0	0,11	0,04	0	0,51	0	0	0,34	0	0,01	0	0,83	0,16	0,046
4	24704	0,06	0	0	0	0,66	0	0	0,28	0	0	0,33	0,66	0,01	0,048
4	13260	0,02	0,14	0	0,02	0	0,05	0	0,77	0	0	0,19	0,04	0,77	0,048
4	5335	0,04	0	0	0,07	0,89	0	0	0	0	0,89	0,02	0,09	0	0,044
4	20956	0	0,06	0,01	0	0	0,91	0	0,02	0	0,98	0	0	0,02	0,044
4	18090	0	0	0	0,1	0,57	0	0	0,33	0	0	0	0,9	0,1	0,036
4	39739	0	0	0,04	0	0,54	0,01	0	0,41	0	0,01	0,04	0,54	0,41	0,016
4	27772	0	0	0	0,34	0	0,66	0	0	0	0,97	0	0,03	0	0,048
4	30082	0	0,28	0	0	0	0,01	0	0,71	0	0	0,05	0,28	0,67	0,046
4	10210	0,03	0,97	0	0	0	0	0	0	0	0,01	0	0,02	0,97	0,048
4	8756	0,04	0,05	0	0	0,88	0	0	0,03	0	0,46	0	0,04	0,5	0,046
4	12973	0,05	0,24	0	0	0,71	0	0	0	0	0	0,24	0	0,76	0,044
4	23222	0	0	0,01	0,07	0,92	0	0	0	0	0,01	0,92	0,07	0	0,048
4	36199	0	0,26	0	0,04	0,7	0	0	0	0	0	0	0,26	0,74	0,044
4	7786	0	0,25	0,04	0,04	0	0	0	0,67	0	0	0,29	0	0,71	0,044
4	14999	0	0	0	0,08	0,16	0,05	0	0,7	0,01	0	0,05	0,25	0,7	0,048
4	20273	0,05	0	0	0	0,01	0,94	0	0	0	0,99	0,01	0	0	0,032
4	20039	0	0,11	0	0	0,03	0,17	0	0,69	0	0,17	0,14	0,69	0	0,044
4	7008	0,05	0	0,04	0	0,9	0	0	0	0,01	0,01	0,73	0,21	0,05	0,044
4	33611	0	0,47	0	0,03	0	0	0	0,48	0,02	0,02	0,47	0	0,51	0,044
4	59672	0	0	0	0,06	0	0	0	0,94	0	0	0,01	0,05	0,94	0,036
4	13623	0,05	0,01	0,01	0	0	0,93	0	0	0	0,91	0,01	0,01	0,07	0,042
4	44191	0,06	0	0	0	0,94	0	0	0	0	0,06	0	0,01	0,93	0,046
4	14379	0	0	0,04	0	0,96	0	0	0	0	0,96	0	0,04	0	0,048
4	5309	0,04	0	0	0,02	0,88	0,03	0	0	0,03	0,91	0,09	0	0	0,048
4	12382	0,05	0	0	0,01	0,9	0,01	0	0,03	0	0	0,92	0,05	0,03	0,048
4	34197	0	0,11	0	0,03	0,71	0	0	0,15	0	0	0,04	0,78	0,18	0,044
4	5187	0,06	0	0	0	0	0,94	0	0	0	0	0	1	0	0,04
4	15134	0	0	0	0,07	0,77	0	0	0,16	0	0,16	0	0,05	0,79	0,048

4	7648	0	0,16	0	0	0,73	0	0	0,11	0	0,12	0,73	0,15	0	0,04
4	24703	0,06	0	0	0	0,94	0	0	0	0	0,94	0	0	0,06	0,048
4	40560	0	0,17	0	0	0,03	0	0	0,8	0	0	0	0,8	0,2	0,044
4,25	12955	0	0	0	0,06	0,92	0	0,01	0,01	0	0,05	0,92	0,02	0,01	0,048
4,25	10545	0,05	0	0	0	0,78	0	0	0,17	0	0,19	0,81	0	0	0,042
4,25	8965	0	0,01	0	0,1	0,89	0	0	0	0	0	0,01	0,99	0	0,048
4,25	23291	0,05	0	0,02	0	0,92	0	0	0,01	0	0,02	0,92	0,01	0,05	0,044
4,25	20144	0,06	0,05	0,01	0	0	0	0	0,86	0,02	0,07	0	0,01	0,92	0,048
4,25	14156	0,05	0	0	0	0,93	0	0	0	0,02	0,93	0,05	0,01	0,01	0,038
4,25	16845	0	0,01	0	0,08	0,47	0,02	0	0,42	0	0,01	0,08	0,25	0,66	0,046
4,25	25343	0	0	0	0,09	0,89	0	0	0	0,02	0,09	0,72	0,19	0	0,048
4,25	21692	0	0,93	0	0	0	0,07	0	0	0	0	0,06	0,07	0,87	0,044
4,25	41127	0	0	0	0,07	0,88	0,05	0	0	0	0	0,94	0,06	0	0,044
4,25	8610	0	0,01	0	0,19	0,29	0,01	0	0,47	0,03	0,77	0	0,04	0,19	0,046
4,25	23251	0	0,27	0,03	0	0,67	0,02	0	0,01	0	0,05	0	0,23	0,72	0,042
4,25	49543	0,06	0	0	0	0,93	0	0	0,01	0	0,93	0	0	0,07	0,048
4,25	36938	0,03	0	0,01	0,08	0,8	0,08	0	0	0	0,83	0,08	0,08	0,01	0,046
4,25	35861	0	0	0	0,13	0,74	0,13	0	0	0	0,74	0,03	0,01	0,22	0,042
4,25	30182	0,03	0,11	0	0	0,13	0,28	0	0,45	0	0	0,23	0,48	0,29	0,048
4,25	6439	0,05	0	0,01	0	0,94	0	0	0	0	0,02	0	0,93	0,05	0,042
4,25	30310	0,05	0	0	0	0,94	0	0,01	0	0	0,92	0	0,01	0,07	0,038
4,25	26187	0	0,04	0,04	0	0,6	0	0	0,32	0	0	0,62	0,04	0,34	0,006
4,25	24477	0,01	0	0	0	0,13	0,8	0,06	0	0	0,07	0,8	0,12	0,01	0,044
4,25	13914	0	0,02	0,01	0,06	0,25	0	0	0,66	0	0,01	0,04	0,64	0,31	0,04
4,25	26241	0	0,12	0	0	0,82	0,01	0,05	0	0	0,32	0,5	0,04	0,14	0,048
4,25	7711	0,02	0	0,04	0,02	0,89	0	0	0,03	0	0,08	0,9	0	0,02	0,046
4,25	6080	0,01	0,87	0	0	0,12	0	0	0	0	0	0,02	0,06	0,92	0,046
4,25	8484	0,04	0	0	0	0,95	0	0	0,01	0	0,04	0	0,95	0,01	0,042
4,25	10783	0,03	0,01	0	0	0,01	0	0,03	0,91	0,01	0,02	0,01	0	0,97	0,048
4,25	10507	0	0	0	0,15	0,85	0	0	0	0	0,29	0,15	0,56	0	0,048
4,25	35452	0,06	0	0	0	0,9	0,01	0,03	0	0	0,76	0,09	0,14	0,01	0,04
4,25	32579	0	0	0	0,07	0,92	0,01	0	0	0	0,07	0,93	0	0	0,044
4,25	14608	0	0	0	0,05	0,92	0	0	0,03	0	0	0,03	0,05	0,92	0,038

4,25	24778	0,01	0,01	0	0	0,79	0	0,15	0	0,04	0,83	0,16	0,01	0	0,046
4,25	8783	0,03	0	0	0,14	0,83	0	0	0	0	0,82	0	0,18	0	0,042
4,25	12995	0,03	0	0	0	0,96	0	0,01	0	0	0	0	0,01	0,99	0,046
4,25	8926	0,01	0	0,01	0,11	0,86	0,01	0	0	0	0,75	0,01	0,01	0,23	0,048
4,25	33963	0,05	0	0	0	0,01	0,25	0	0	0,69	0,01	0,68	0,02	0,29	0,046
4,25	29832	0	0,1	0,01	0	0,86	0,03	0	0	0	0,11	0	0,87	0,02	0,048
4,25	12216	0,01	0,12	0,01	0,03	0	0,83	0	0	0	0,12	0	0,05	0,83	0,044
4,25	7480	0,06	0	0	0	0,43	0	0	0,51	0	0,04	0	0,94	0,02	0,04
4,25	7575	0,04	0	0	0,01	0	0	0	0,94	0,01	0,01	0,94	0	0,05	0,044
4,25	17372	0	0,02	0	0,17	0,79	0,02	0	0	0	0,79	0,02	0,19	0	0,044
4,25	19879	0	0,2	0	0	0,79	0	0	0,01	0	0	0,79	0,2	0,01	0,048
4,25	14934	0,05	0	0	0	0,83	0	0	0,12	0	0	0,05	0	0,95	0,042
4,25	29598	0	0	0	0,06	0,93	0	0	0	0,01	0,01	0	0,06	0,93	0,044
4,25	50090	0,04	0	0	0	0,91	0	0,01	0,04	0	0,04	0,91	0,04	0,01	0,044
4,25	5196	0,05	0	0,03	0	0,92	0	0	0	0	0	0,02	0,94	0,04	0,038
4,25	59777	0	0,39	0	0	0	0,61	0	0	0	0	0,39	0	0,61	0,044
4,25	40212	0,01	0	0	0,05	0,85	0,07	0	0,01	0,01	0	0,91	0,02	0,07	0,046
4,25	10676	0,03	0	0	0,03	0,87	0	0,05	0,01	0,01	0,88	0,01	0,04	0,07	0,048
4,25	23793	0	0,96	0	0,03	0	0	0	0,01	0	0,04	0	0,01	0,95	0,048
4,25	19327	0,05	0	0	0	0,57	0	0	0	0,38	0,05	0,95	0	0	0,048
4,25	20607	0,06	0,01	0	0	0,54	0,38	0	0,01	0	0,54	0,06	0,39	0,01	0,048
4,25	4675	0,06	0	0,01	0	0,91	0	0	0,02	0	0,03	0,01	0	0,96	0,046
4,25	36073	0,03	0	0	0,03	0,94	0	0	0	0	0	0,97	0,03	0	0,046
4,25	38544	0	0,23	0	0	0,69	0,01	0,01	0,06	0	0,23	0,02	0	0,75	0,046
4,25	9443	0	0	0	0,04	0,95	0	0,01	0	0	0,01	0,01	0,03	0,95	0,048
4,25	27774	0,04	0,32	0	0	0,3	0	0	0,34	0	0	0,68	0	0,32	0,048
4,25	67819	0,01	0	0,04	0	0,89	0,05	0	0	0,01	0,63	0,1	0,04	0,23	0,036
4,25	23592	0	0,08	0,04	0	0,87	0	0	0	0,01	0,04	0,87	0	0,09	0,004
4,25	21023	0,05	0,01	0	0	0,94	0	0	0	0	0	0,94	0	0,06	0,046
4,25	11905	0,03	0	0,03	0	0,88	0	0	0,06	0	0,68	0,08	0,21	0,03	0,046
4,25	18962	0,02	0,14	0	0,04	0,04	0	0	0,76	0	0,05	0,04	0	0,91	0,048
4,25	10360	0,05	0	0	0	0,9	0	0	0,03	0,02	0	0,03	0,92	0,05	0,048
4,25	11442	0	0	0	0,07	0,68	0,25	0	0	0	0	0,94	0,06	0	0,048

4,25	31909	0,05	0	0,04	0	0,81	0	0	0,08	0,02	0,11	0,08	0	0,81	0,038
4,25	11893	0	0,95	0,02	0	0	0	0,03	0	0	0,02	0,03	0	0,95	0,046
4,25	12869	0,04	0	0	0,02	0,94	0	0	0	0	0,61	0	0,02	0,37	0,044
4,25	33415	0,01	0	0	0	0,93	0,03	0,03	0	0	0,03	0	0	0,97	0,038
4,25	11708	0,05	0	0,01	0	0,87	0,04	0	0,03	0	0,96	0,04	0	0	0,046
4,25	37103	0,02	0	0,04	0,03	0,14	0,76	0,01	0	0	0,91	0,04	0,02	0,03	0,046
4,25	22795	0,05	0	0	0,01	0,65	0	0	0,29	0	0	0,74	0	0,26	0,048
4,25	17025	0,06	0	0	0	0	0,94	0	0	0	0,95	0	0	0,05	0,048
4,25	15379	0,06	0,03	0	0	0,91	0	0	0	0	0,02	0,02	0,05	0,91	0,048
4,25	18999	0	0,08	0	0	0,89	0	0	0,03	0	0,9	0	0	0,1	0,048
4,25	8429	0,03	0,14	0	0	0,6	0,23	0	0	0	0	0,6	0,03	0,37	0,042
4,25	22921	0,05	0	0	0	0,86	0	0	0,08	0,01	0,08	0	0,15	0,77	0,04
4,25	27111	0,05	0,03	0,01	0	0,91	0	0	0	0	0	0,01	0	0,99	0,042
4,25	16611	0,05	0	0	0	0,94	0	0,01	0	0	0	0,04	0,96	0	0,048
4,25	4317	0,04	0	0	0	0	0,06	0,01	0,88	0,01	0,88	0,01	0,05	0,06	0,046
4,25	15190	0	0	0	0,09	0	0	0	0,9	0,01	0	0,83	0,16	0,01	0,042
4,25	4917	0,01	0	0	0,15	0	0	0	0,84	0	0,85	0	0	0,15	0,038
4,25	17348	0	0,06	0	0	0,91	0	0,03	0	0	0,94	0	0,05	0,01	0,038
4,25	4903	0,05	0	0	0	0,94	0,01	0	0	0	0	0,04	0	0,96	0,046
4,25	38410	0,05	0,02	0,01	0	0,91	0,01	0	0	0	0,14	0,02	0,84	0	0,048
4,25	27810	0,03	0	0,03	0	0,85	0,09	0	0	0	0,84	0,09	0	0,07	0,04
4,25	26248	0	0	0,04	0,05	0,84	0	0	0,06	0,01	0,85	0,09	0,06	0	0,04
4,25	5857	0,06	0	0,01	0	0,93	0	0	0	0	0	0,94	0	0,06	0,048
4,25	21816	0,06	0	0	0	0,94	0	0	0	0	0	1	0	0	0,046
4,25	12321	0,06	0	0,02	0	0,44	0,01	0	0,47	0	0	0,03	0,96	0,01	0,048
4,25	16888	0,03	0	0	0	0	0	0,03	0,94	0	0	0,97	0	0,03	0,044
4,25	7665	0	0,25	0,02	0	0,48	0,01	0	0	0,24	0	0,23	0,29	0,48	0,046
4,25	9967	0	0	0	0	0	0,88	0,12	0	0	0	0	0,96	0,04	0,036
4,25	16635	0,01	0,21	0,01	0	0,04	0	0,01	0,7	0,02	0	0,04	0,21	0,75	0,046
4,25	9470	0,02	0	0,04	0,03	0,9	0	0	0,01	0	0,92	0,01	0,02	0,05	0,046
4,25	62291	0	0	0,01	0,17	0,61	0,13	0,02	0,06	0	0,74	0,23	0,03	0	0,048
4,25	22925	0	0	0	0,14	0,5	0,36	0	0	0	0,47	0,02	0,49	0,02	0,048
4,25	31775	0,05	0	0,01	0	0,92	0	0,01	0	0,01	0,01	0,93	0,01	0,05	0,048

4,25	16100	0,04	0	0	0,02	0,94	0	0	0	0	0	0,94	0,04	0,02	0,042
4,25	25116	0	0	0	0,07	0,93	0	0	0	0	0,07	0,93	0	0	0,048
4,25	9406	0	0	0	0,07	0	0,1	0	0,83	0	0,1	0	0	0,9	0,042
4,25	13184	0,04	0,01	0	0	0	0,95	0	0	0	0,03	0,01	0,94	0,02	0,042
4,5	26664	0,02	0	0,04	0	0,18	0	0,06	0,7	0	0,69	0,06	0,06	0,19	0,048
4,5	58808	0	0,21	0	0,01	0,78	0	0	0	0	0	0,79	0,21	0	0,044
4,5	8143	0,01	0,48	0,01	0	0	0	0	0,44	0,06	0,06	0	0,02	0,92	0,044
4,5	23999	0	0,04	0	0,11	0,1	0,01	0	0,74	0	0,44	0,16	0,36	0,04	0,046
4,5	11658	0,04	0	0	0	0,96	0	0	0	0	0	0,96	0,04	0	0,036
4,5	21014	0	0	0	0,11	0,63	0	0	0,26	0	0	0,26	0,74	0	0,038
4,5	14113	0	0	0,04	0,01	0,71	0,08	0	0,16	0	0	0,91	0,09	0	0,016
4,5	6120	0,06	0	0	0,01	0,91	0	0	0,01	0,01	0,01	0	0,98	0,01	0,044
4,5	24738	0	0,24	0	0,04	0,7	0,02	0	0	0	0,01	0,95	0,04	0	0,048
4,5	5267	0,01	0	0	0	0	0,91	0,07	0,01	0	0,01	0,91	0,07	0,01	0,036
4,5	45252	0	0,49	0	0	0,18	0,07	0	0,24	0,02	0	0,36	0,09	0,55	0,036
4,5	36345	0,01	0,01	0	0,12	0,82	0,03	0,01	0	0	0,7	0	0,28	0,02	0,046
4,5	34353	0,02	0,01	0	0,01	0,86	0	0,07	0	0,03	0,53	0,04	0,32	0,11	0,046
4,5	19753	0,02	0	0	0,05	0,93	0	0	0	0	0	0,93	0,05	0,02	0,048
4,5	36897	0	0,11	0	0	0,89	0	0	0	0	0,47	0,52	0,01	0	0,04
4,5	29456	0,03	0	0,01	0,05	0,91	0	0	0	0	0,05	0,04	0,91	0	0,044
4,5	24949	0,04	0	0,01	0,03	0,91	0	0	0	0,01	0	0,02	0,91	0,07	0,048
4,5	17251	0,05	0	0	0,06	0	0,89	0	0	0	0,94	0,06	0	0	0,044
4,5	6798	0,01	0	0	0,21	0,71	0	0	0,06	0,01	0,73	0,21	0	0,06	0,048
4,5	54473	0,05	0	0	0,04	0,37	0,41	0,01	0	0,12	0,84	0,05	0,07	0,04	0,044
4,5	20263	0,05	0	0	0	0,93	0,02	0	0	0	0	0	0,06	0,94	0,04
4,5	27519	0,05	0	0	0	0,82	0,12	0,01	0	0	0,01	0	0,87	0,12	0,046
4,5	21289	0,05	0	0	0	0,95	0	0	0	0	0,79	0,21	0	0	0,038
4,5	21336	0,05	0	0	0	0,93	0,02	0	0	0	0	0,04	0,89	0,07	0,048
4,5	4129	0,03	0	0,02	0,05	0,9	0	0	0	0	0,95	0,02	0,03	0	0,046
4,5	6101	0,05	0,02	0	0	0,93	0	0	0	0	0	0	0,02	0,98	0,046
4,5	10016	0,03	0	0	0,05	0	0	0	0,92	0	0,05	0,87	0,08	0	0,044
4,5	13764	0	0	0	0,12	0,83	0	0	0,05	0	0,12	0,05	0,83	0	0,048
4,5	12919	0	0	0	0,08	0,92	0	0	0	0	0	0,91	0,01	0,08	0,046

4,5	10955	0	0,21	0	0	0,77	0	0	0	0,02	0	0,79	0,21	0	0,048
4,5	23857	0	0	0	0,08	0,92	0	0	0	0	0,02	0,53	0,45	0	0,042
4,5	13024	0	0,02	0	0,06	0,92	0	0	0	0	0,02	0,92	0	0,06	0,048
4,5	13901	0,01	0	0,02	0	0,02	0,06	0,06	0,83	0	0,02	0	0,09	0,89	0,044
4,5	27088	0,01	0	0,03	0,1	0,85	0	0	0	0,01	0,86	0,01	0	0,13	0,046
4,5	19794	0,06	0,01	0	0	0,91	0,02	0	0	0	0	0,03	0	0,97	0,048
4,5	25903	0	0,95	0	0,05	0	0	0	0	0	0	0,05	0	0,95	0,04
4,5	27780	0,03	0,03	0,01	0,01	0,12	0,8	0	0	0	0,81	0	0,15	0,04	0,048
4,5	7294	0,02	0	0,02	0,05	0,91	0	0	0	0	0	0,59	0	0,41	0,048
4,5	15131	0	0	0	0,38	0,62	0	0	0	0	1	0	0	0	0,048
4,5	39442	0	0	0,01	0,07	0,92	0	0	0	0	0,07	0	0,02	0,91	0,048
4,5	13004	0	0,01	0	0	0,07	0	0,1	0,82	0	0	0	0,83	0,17	0,048
4,5	56795	0,04	0	0,02	0	0,94	0	0	0	0	0,96	0	0,04	0	0,046
4,5	24643	0,01	0,02	0	0,02	0,18	0	0,01	0,75	0,01	0,01	0,76	0,01	0,22	0,04
4,5	10298	0,04	0,06	0	0	0,19	0,68	0	0	0,03	0,25	0	0,09	0,66	0,046
4,5	11542	0,05	0	0,04	0	0,85	0	0	0,06	0	0	0,89	0,05	0,06	0,042
4,5	15327	0,01	0,97	0,01	0,01	0	0	0	0	0	0,01	0	0,02	0,97	0,044
4,5	19006	0,05	0	0	0	0	0,11	0	0,84	0	0	1	0	0	0,034
4,5	55674	0	0,1	0	0	0,83	0,01	0	0,02	0,04	0,83	0,1	0,06	0,01	0,048
4,5	20187	0,05	0	0	0	0,95	0	0	0	0	0	0,05	0,95	0	0,046
4,5	22825	0,01	0,13	0	0	0	0,01	0	0,63	0,22	0,15	0,59	0,12	0,14	0,042
4,5	49500	0	0,1	0	0,01	0,87	0,01	0	0,01	0	0,02	0	0,97	0,01	0,042
4,5	10428	0	0,01	0,04	0	0	0,93	0,02	0	0	0	0,06	0,01	0,93	0,016
4,5	17945	0	0	0	0,23	0,77	0	0	0	0	0,77	0,23	0	0	0,048
4,5	15998	0	0	0	0,16	0,84	0	0	0	0	0,58	0,16	0,26	0	0,044
4,5	28821	0,05	0	0	0	0,22	0,61	0	0,1	0,02	0,05	0,76	0,19	0	0,04
4,5	27762	0,05	0	0	0	0,93	0	0	0	0,02	0,94	0,05	0,01	0	0,042
4,5	6141	0,02	0,02	0,02	0	0,94	0	0	0	0	0,96	0,02	0,02	0	0,042
4,5	15136	0	0,23	0	0	0,65	0	0	0,12	0	0,23	0	0,12	0,65	0,044
4,5	3864	0,02	0,02	0,01	0,05	0,9	0	0	0	0	0,04	0,04	0,91	0,01	0,048
4,5	59528	0,01	0	0	0,1	0,88	0,01	0	0	0	0,1	0	0,89	0,01	0,038
4,5	21742	0,02	0,04	0,04	0	0,87	0	0	0,01	0,02	0,04	0,12	0,02	0,82	0,026
4,5	13982	0,01	0,1	0,01	0	0	0,69	0	0,19	0	0,1	0,01	0,89	0	0,046

4,5	16018	0,01	0,46	0	0,01	0,52	0	0	0	0	0,04	0,53	0	0,43	0,042
4,5	15340	0,05	0	0	0	0,94	0	0,01	0	0	0,95	0	0	0,05	0,048
4,5	11702	0	0,23	0	0	0,76	0,01	0	0	0	0,01	0,76	0,23	0	0,04
4,5	9936	0,02	0,08	0	0	0,5	0	0	0,4	0	0,38	0	0,02	0,6	0,048
4,5	23978	0,02	0	0	0,05	0,86	0,05	0	0	0,02	0,39	0,08	0,01	0,52	0,048
4,5	21057	0,01	0,07	0,01	0	0,85	0	0,06	0	0	0	0,86	0,06	0,08	0,048
4,5	26471	0,02	0	0	0,03	0,95	0	0	0	0	0,02	0	0,03	0,95	0,048
4,5	43905	0	0	0	0,27	0,72	0	0,01	0	0	0,95	0	0	0,05	0,048
4,5	7964	0,02	0,07	0,01	0	0,89	0,01	0	0	0	0,07	0,16	0,75	0,02	0,048
4,5	2865	0,06	0	0,04	0	0	0	0	0,9	0	0	0,94	0,06	0	0,046
4,5	23394	0	0	0	0,31	0,68	0	0,01	0	0	1	0	0	0	0,042
4,5	25305	0	0	0	0,12	0,82	0,04	0	0	0,02	0,13	0,04	0,83	0	0,046
4,5	32858	0	0,09	0	0,04	0,87	0	0	0	0	0	0,09	0,91	0	0,044
4,5	24924	0	0,07	0	0,05	0,81	0,07	0	0	0	0,12	0	0,07	0,81	0,046
4,5	19001	0	0,11	0	0	0,53	0,3	0	0,06	0	0,3	0	0,7	0	0,048
4,5	13411	0	0	0,02	0,06	0,73	0,08	0	0,11	0	0,06	0,86	0,08	0	0,044
4,5	67401	0	0	0	0	0,93	0	0,07	0	0	0,07	0,93	0	0	0,048
4,5	12041	0	0,08	0	0	0,85	0,01	0	0,06	0	0,85	0,05	0,08	0,02	0,04
4,5	8620	0,01	0	0	0,1	0,63	0,26	0	0	0	0,28	0	0,63	0,09	0,044
4,5	16557	0	0,14	0	0,02	0	0,1	0	0,72	0,02	0,02	0,2	0,24	0,54	0,04
4,5	12419	0,06	0	0,01	0	0,77	0,11	0	0	0,05	0	0,83	0	0,17	0,048
4,5	27530	0,01	0,83	0	0	0	0	0	0,16	0	0,08	0,01	0	0,91	0,042
4,5	9080	0,01	0	0,04	0	0,94	0	0	0	0,01	0	0,92	0,02	0,06	0,012
4,5	18740	0	0,27	0	0	0,73	0	0	0	0	0,27	0	0	0,73	0,048
4,5	5038	0,03	0	0	0	0,27	0	0,03	0,67	0	0	0,22	0,23	0,55	0,046
4,5	6335	0	0,07	0	0,06	0,87	0	0	0	0	0,03	0,97	0	0	0,042
4,5	11706	0,05	0,01	0	0	0,94	0	0	0	0	0	0,94	0	0,06	0,046
4,5	25276	0	0,45	0	0	0,27	0	0	0,28	0	0	0,3	0,15	0,55	0,048
4,5	12976	0	0,01	0,02	0,05	0,47	0,37	0,02	0	0,06	0,02	0,43	0	0,55	0,042
4,5	69932	0,04	0	0	0,01	0	0,94	0,01	0	0	0,04	0,01	0,92	0,03	0,048
4,5	51424	0	0,29	0	0	0,71	0	0	0	0	0	0,03	0,29	0,68	0,048
4,5	5353	0,05	0	0,01	0,01	0,92	0	0	0,01	0	0,02	0,02	0	0,96	0,044
4,5	6792	0	0,01	0	0,09	0,86	0,01	0	0,03	0	0,12	0	0,87	0,01	0,04

4,5	8838	0,01	0	0,04	0	0,75	0	0	0,2	0	0	0,22	0,73	0,05	0,034
4,5	21747	0,03	0	0,03	0	0,84	0	0	0,09	0,01	0,09	0,03	0,84	0,04	0,046
4,5	10298	0,01	0	0,01	0,05	0,93	0	0	0	0	0,01	0,01	0	0,98	0,048
4,5	39270	0,01	0,14	0,01	0	0,65	0,18	0	0,01	0	0,18	0,61	0,02	0,19	0,046
4,5	9381	0,06	0,01	0	0	0,93	0	0	0	0	0,24	0	0,73	0,03	0,048
4,75	42268	0,03	0,03	0	0,02	0,71	0,2	0	0	0,01	0,03	0,04	0,2	0,73	0,04
4,75	31922	0	0,14	0	0	0	0,86	0	0	0	0,53	0,01	0,14	0,32	0,042
4,75	9555	0	0	0	0,4	0,6	0	0	0	0	0,99	0	0	0,01	0,042
4,75	10577	0,01	0	0	0,04	0,93	0	0,02	0	0	0,04	0,93	0,03	0	0,046
4,75	15855	0	0	0	0,12	0,87	0,01	0	0	0	0,01	0,14	0,85	0	0,048
4,75	46282	0	0,01	0	0,11	0,71	0,17	0	0	0	0,11	0,71	0,18	0	0,038
4,75	4705	0,04	0	0	0,01	0,94	0	0	0	0,01	0,01	0,04	0,22	0,73	0,048
4,75	10049	0,01	0,28	0	0	0,71	0	0	0	0	0,28	0	0	0,72	0,048
4,75	40782	0,06	0	0,01	0	0,91	0	0	0,02	0	0,91	0,01	0	0,08	0,044
4,75	10977	0,03	0	0,01	0,09	0,87	0	0	0	0	0,87	0,01	0,12	0	0,042
4,75	25627	0	0,02	0	0,05	0,93	0	0	0	0	0,05	0	0	0,95	0,048
4,75	27817	0	0,09	0	0	0,02	0	0	0,79	0,1	0,79	0	0,11	0,1	0,044
4,75	22660	0,03	0	0	0,04	0,93	0	0	0	0	0,03	0,93	0,04	0	0,046
4,75	19444	0	0	0	0,12	0,87	0	0	0,01	0	0,12	0,52	0,36	0	0,048
4,75	45566	0,06	0	0	0	0,94	0	0	0	0	0	0	0,94	0,06	0,046
4,75	24251	0,02	0,02	0	0,05	0,91	0	0	0	0	0,07	0,88	0	0,05	0,042
4,75	17979	0,02	0	0	0,04	0,93	0,01	0	0	0	0,14	0,02	0,04	0,8	0,046
4,75	23223	0,05	0,08	0	0	0,86	0,01	0	0	0	0	0,11	0	0,89	0,048
4,75	12141	0,01	0	0	0,1	0,34	0,37	0	0,18	0	0,26	0,38	0,07	0,29	0,046
4,75	19087	0,02	0,06	0	0	0	0,13	0,01	0,78	0	0	0,17	0,81	0,02	0,046
4,75	45132	0	0	0	0,13	0,21	0,65	0	0	0,01	0,22	0,06	0,7	0,02	0,048
4,75	34091	0	0	0	0,14	0,84	0	0	0,01	0,01	0,15	0,26	0,58	0,01	0,048
4,75	43356	0,04	0,07	0,02	0	0,03	0	0	0,83	0,01	0,07	0,02	0,26	0,65	0,048
4,75	64500	0	0,28	0	0	0	0,7	0,02	0	0	0	0,3	0	0,7	0,046
4,75	12280	0	0	0	0,1	0,64	0	0	0,26	0	0	0,1	0,64	0,26	0,046
4,75	23573	0,06	0,05	0	0,01	0,36	0,02	0	0,5	0	0,02	0,5	0,05	0,43	0,048
4,75	7116	0,01	0,59	0	0	0,35	0	0	0,05	0	0	0,4	0,07	0,53	0,046
4,75	8745	0,04	0,01	0,03	0	0	0,01	0	0,91	0	0,01	0,06	0,01	0,92	0,038

4,75	21493	0,04	0,04	0,01	0	0,89	0,01	0,01	0	0	0,02	0,89	0	0,09	0,048
4,75	7820	0,01	0,1	0	0	0,88	0	0	0,01	0	0,01	0	0,99	0	0,042
4,75	25341	0,06	0	0	0	0,7	0,23	0	0,01	0	0,06	0,24	0,68	0,02	0,046
4,75	23618	0,03	0,15	0	0,02	0,65	0	0	0,15	0	0,01	0,04	0,13	0,82	0,044
4,75	35039	0	0,01	0	0,16	0,81	0	0	0,02	0	0,74	0,12	0,01	0,13	0,046
4,75	34167	0	0	0,01	0,11	0,01	0,75	0	0	0,12	0,22	0,56	0,11	0,11	0,046
4,75	29632	0,01	0	0	0,14	0,02	0,39	0	0,44	0	0,65	0,16	0	0,19	0,046
4,75	11378	0,01	0,01	0	0,05	0,92	0	0	0,01	0	0	0,03	0,09	0,88	0,046
4,75	46087	0	0	0,01	0,08	0,91	0	0	0	0	0,01	0	0,91	0,08	0,038
4,75	35736	0	0,02	0	0,07	0,81	0	0	0,09	0,01	0,11	0,02	0,03	0,84	0,046
4,75	33174	0	0,05	0	0,08	0	0	0	0,87	0	0,05	0	0,08	0,87	0,046
4,75	16019	0	0,09	0,02	0,05	0,77	0,07	0	0	0	0,09	0,01	0,12	0,78	0,044
4,75	17692	0,01	0,02	0	0,14	0,78	0,04	0	0	0,01	0,82	0,02	0,02	0,14	0,046
4,75	16169	0,02	0,04	0	0	0,93	0	0	0	0,01	0,94	0	0,04	0,02	0,036
4,75	36815	0,06	0	0	0	0	0	0	0,94	0	0	0,06	0	0,94	0,044
4,75	27920	0,04	0,02	0	0	0,78	0	0,01	0,15	0	0,17	0,78	0,04	0,01	0,048
4,75	14670	0,03	0,03	0	0	0	0,93	0,01	0	0	0,89	0,01	0,06	0,04	0,046
4,75	13258	0	0	0,04	0,02	0,17	0,19	0	0,47	0,11	0,19	0,19	0,51	0,11	0,028
4,75	10409	0	0,2	0	0	0,78	0	0,01	0	0,01	0,02	0,78	0,2	0	0,044
4,75	20561	0	0	0,02	0,12	0,35	0,51	0	0	0	0,36	0,43	0,21	0	0,042
4,75	14817	0	0	0,04	0	0,86	0,02	0,07	0	0,01	0,04	0,01	0,88	0,07	0,04
4,75	18154	0,06	0	0,04	0,01	0,88	0	0	0	0,01	0	0,04	0,05	0,91	0,048
4,75	61748	0,01	0	0	0,05	0,93	0,01	0	0	0	0,06	0,22	0	0,72	0,042
4,75	6548	0,02	0,02	0,01	0	0,23	0,06	0	0,66	0	0,91	0	0,01	0,08	0,048
4,75	26937	0,05	0,01	0	0	0	0,94	0	0	0	0	0,06	0	0,94	0,046
4,75	16255	0,06	0	0	0	0,94	0	0	0	0	0,01	0,05	0	0,94	0,044
4,75	21399	0,03	0,01	0	0,03	0	0	0	0,93	0	0,01	0,31	0,05	0,63	0,046
4,75	15205	0,05	0	0	0,02	0,91	0	0	0	0,02	0,93	0,07	0	0	0,042
4,75	19065	0	0	0	0,06	0,9	0,04	0	0	0	0,08	0,01	0	0,91	0,038
4,75	16230	0	0,1	0	0,04	0,8	0,05	0	0,01	0	0,11	0,59	0,29	0,01	0,046
4,75	20122	0	0	0	0,08	0,71	0	0	0,21	0	0,23	0,69	0,08	0	0,046
4,75	11969	0	0	0	0,09	0,84	0	0,02	0	0,05	0,02	0,09	0,82	0,07	0,042
4,75	12796	0,04	0,01	0,01	0,01	0,05	0	0	0,85	0,03	0,86	0,04	0,04	0,06	0,048

4,75	13740	0	0,78	0	0	0,14	0,08	0	0	0	0,08	0	0	0,92	0,046
4,75	26537	0,05	0	0	0	0,95	0	0	0	0	0,94	0,01	0,05	0	0,048
4,75	11873	0	0,16	0	0,01	0,83	0	0	0	0	0,1	0,73	0,16	0,01	0,048
4,75	13701	0,06	0	0	0	0,9	0,04	0	0	0	0	0,01	0,04	0,95	0,042
4,75	53549	0	0	0,04	0	0,16	0,78	0	0,01	0,01	0,02	0,05	0	0,93	0,006
4,75	25633	0,02	0	0,04	0	0,86	0,03	0	0,01	0,04	0,04	0,03	0,03	0,9	0,03
4,75	30318	0	0,95	0,01	0,02	0	0,02	0	0	0	0,03	0,02	0	0,95	0,042
4,75	10229	0	0,95	0	0	0	0,05	0	0	0	0,02	0	0,03	0,95	0,034
4,75	19017	0	0	0,04	0,01	0,92	0,03	0	0	0	0,04	0,08	0,03	0,85	0,006
4,75	13228	0	0	0,02	0,06	0	0,03	0,01	0,88	0	0	0,86	0,08	0,06	0,04
4,75	9773	0	0,06	0	0,04	0,9	0	0	0	0	0,89	0,1	0,01	0	0,046
4,75	12989	0	0	0	0,15	0	0,18	0	0,67	0	0,12	0,15	0,73	0	0,046
4,75	11246	0	0	0	0,06	0,92	0,02	0	0	0	0	0	0,06	0,94	0,046
4,75	11213	0,01	0,07	0,02	0,02	0	0,86	0	0,01	0,01	0,07	0,03	0,87	0,03	0,046
4,75	13998	0,04	0	0	0	0,92	0,02	0,02	0	0	0,18	0,74	0,01	0,07	0,04
4,75	7831	0,02	0,98	0	0	0	0	0	0	0	0	0	0,02	0,98	0,04
4,75	12869	0,06	0	0	0	0	0	0	0	0,94	0	0,94	0	0,06	0,048
4,75	27662	0,05	0	0	0,01	0,92	0	0	0,01	0,01	0,01	0	0,94	0,05	0,046
4,75	12528	0,02	0	0,04	0,03	0,17	0,3	0	0,44	0	0,21	0,02	0,47	0,3	0,046
4,75	38311	0,01	0	0	0,05	0,92	0,02	0	0	0	0	0,97	0	0,03	0,048
4,75	36776	0,06	0	0,02	0	0,92	0	0	0	0	0,06	0	0,02	0,92	0,042
4,75	10978	0	0	0	0,06	0,89	0,01	0	0,04	0	0,06	0,04	0	0,9	0,046
4,75	7290	0,03	0	0,02	0,03	0,81	0	0,02	0	0,09	0,82	0,17	0	0,01	0,042
4,75	23287	0,05	0	0	0	0,78	0,17	0	0	0	0	0	0,95	0,05	0,036
4,75	15896	0	0	0	0,07	0	0	0	0,93	0	0	0	0,07	0,93	0,048
4,75	11590	0,02	0	0,01	0,05	0	0	0	0,76	0,16	0,05	0,72	0	0,23	0,044
4,75	25920	0	0,06	0,03	0	0,74	0,17	0	0	0	0,92	0	0,06	0,02	0,046
4,75	13068	0,02	0	0	0,14	0,65	0	0	0	0,19	0,65	0,14	0,08	0,13	0,046
4,75	45508	0	0,13	0	0	0,87	0	0	0	0	0	0	0,87	0,13	0,048
4,75	13413	0	0,13	0	0,01	0,8	0,04	0	0	0,02	0,15	0	0,84	0,01	0,046
4,75	15239	0,04	0,95	0	0	0	0	0	0	0,01	0,01	0,04	0	0,95	0,042
4,75	8082	0,04	0	0	0,01	0,94	0,01	0	0	0	0	0	0,01	0,99	0,046
4,75	31717	0	0,05	0	0	0,03	0	0,08	0,84	0	0,04	0,04	0,82	0,1	0,04

4,75	55463	0	0,02	0	0,05	0,93	0	0	0	0	0,05	0	0	0,95	0,038
4,75	19077	0	0	0,04	0	0,02	0,44	0,01	0,41	0,08	0,01	0	0,48	0,51	0,026
4,75	7210	0,01	0	0,04	0	0,94	0	0	0,01	0	0	0,01	0,04	0,95	0,014
4,75	32128	0	0	0,02	0,1	0,43	0	0	0,45	0	0,45	0,1	0	0,45	0,044
4,75	12852	0	0	0,01	0,09	0,89	0	0	0,01	0	0,09	0,91	0	0	0,048
4,75	18383	0	0,26	0	0	0,73	0	0	0	0,01	0,24	0	0,03	0,73	0,048
5	34884	0,01	0	0,04	0,01	0,86	0	0,03	0	0,05	0,08	0,04	0,87	0,01	0,048
5	59962	0,05	0	0	0	0	0,95	0	0	0	0,95	0	0,05	0	0,044
5	20267	0,03	0,05	0,02	0	0	0,06	0	0,84	0	0,05	0	0,87	0,08	0,044
5	23279	0	0,91	0,04	0	0	0	0	0,05	0	0	0,04	0,05	0,91	0,046
5	10805	0,04	0,1	0	0,03	0,77	0,02	0	0	0,04	0,04	0,75	0	0,21	0,048
5	9592	0	0	0,02	0,1	0,77	0,11	0	0	0	0,67	0,11	0,09	0,13	0,048
5	28949	0,05	0	0	0,01	0	0	0	0,94	0	0,01	0,78	0	0,21	0,042
5	40788	0,02	0	0,01	0,12	0,85	0	0	0	0	0,66	0,01	0,27	0,06	0,046
5	28686	0	0,94	0,03	0,01	0,01	0	0,01	0	0	0,01	0,03	0,01	0,95	0,044
5	38512	0,06	0	0	0	0	0	0,02	0,92	0	0,92	0,02	0,06	0	0,048
5	17433	0	0	0	0,23	0,77	0	0	0	0	0,77	0,23	0	0	0,048
5	43236	0,01	0,05	0	0,01	0,93	0	0	0	0	0,95	0,01	0,04	0	0,042
5	21642	0,04	0	0	0,02	0,94	0	0	0	0	0	0	0,98	0,02	0,036
5	4672	0,04	0,02	0	0	0,92	0	0,02	0	0	0	0,02	0,96	0,02	0,044
5	62157	0,04	0,26	0,03	0	0,66	0,01	0	0	0	0,03	0	0	0,97	0,04
5	13122	0,06	0	0	0	0	0,7	0	0,24	0	0	0,94	0,06	0	0,042
5	12133	0,02	0	0	0	0,9	0,03	0,05	0	0	0,36	0,08	0,01	0,55	0,042
5	18242	0,05	0	0	0	0,75	0,01	0	0,19	0	0,02	0,01	0,13	0,84	0,046
5	12915	0,05	0	0	0	0	0,94	0	0	0,01	0,94	0	0,06	0	0,048
5	16934	0,06	0	0	0	0,8	0	0	0	0,14	0,66	0,14	0,14	0,06	0,048
5	11759	0	0	0	0,11	0,84	0,03	0	0	0,02	0,02	0,03	0,95	0	0,046
5	15673	0,02	0	0	0,04	0,92	0	0	0,02	0	0	0,92	0,04	0,04	0,048
5	41647	0	0	0,04	0	0,95	0	0,01	0	0	0	0,01	0,95	0,04	0,018
5	9937	0	0	0	0,08	0,56	0,17	0	0,09	0,1	0,32	0,05	0	0,63	0,044
5	16437	0,01	0	0,02	0,14	0,83	0	0	0	0	0,83	0,03	0,14	0	0,048
5	18876	0,04	0	0	0	0	0,46	0,04	0,46	0	0,28	0,08	0,64	0	0,042
5	25247	0	0	0	0,12	0	0	0	0,88	0	0	0	0,73	0,27	0,048

5	11941	0,04	0,04	0	0	0,92	0	0	0	0	0	0	0,96	0,04	0,046
5	15418	0	0,18	0	0	0,69	0,12	0	0	0,01	0,12	0,69	0,18	0,01	0,046
5	18074	0,01	0	0	0,07	0,75	0	0,01	0,16	0	0,04	0,16	0,8	0	0,046
5	18499	0	0,14	0	0	0,86	0	0	0	0	0	0,49	0,51	0	0,048
5	7215	0,04	0,07	0,02	0	0,85	0,01	0	0	0,01	0,01	0,94	0,01	0,04	0,048
5	20195	0,06	0	0,04	0	0,82	0	0	0,08	0	0,01	0,04	0,08	0,87	0,046
5	8902	0	0	0	0,07	0,93	0	0	0	0	0	0,83	0	0,17	0,044
5	13335	0	0,01	0	0,12	0,87	0	0	0	0	0,29	0,58	0,13	0	0,048
5	8586	0	0	0	0,06	0,93	0	0,01	0	0	0	1	0	0	0,046
5	8695	0,02	0	0	0,07	0,91	0	0	0	0	0	0,02	0,98	0	0,048
5	14171	0,04	0	0,02	0	0	0,03	0	0,9	0,01	0,01	0,02	0,93	0,04	0,044
5	11743	0,04	0	0	0,03	0	0	0	0,93	0	0	0,93	0,04	0,03	0,044
5	19385	0,04	0	0	0	0,89	0	0	0,07	0	0	0,11	0,78	0,11	0,032
5	15370	0,02	0	0,02	0	0,7	0	0,04	0,19	0,03	0,13	0,02	0,02	0,83	0,042
5	20074	0	0,17	0,04	0	0	0,78	0	0	0,01	0	0,13	0	0,87	0,044
5	12190	0,01	0	0,02	0,07	0,03	0,87	0	0	0	0,02	0	0,92	0,06	0,046
5	30496	0	0,16	0	0	0,83	0,01	0	0	0	0	0	1	0	0,048
5	65612	0,03	0	0,01	0,03	0,89	0	0	0	0,04	0,04	0	0,21	0,75	0,042
5	27324	0,06	0	0	0,01	0,93	0	0	0	0	0	0	0,94	0,06	0,048
5	14530	0,04	0,05	0,02	0	0,03	0,86	0	0	0	0	0,56	0,33	0,11	0,042
5	19480	0,06	0	0	0	0,93	0	0	0	0,01	0,91	0,07	0,02	0	0,048
5	27360	0	0,11	0	0,02	0,84	0	0	0,02	0,01	0	0,01	0,95	0,04	0,042
5	12230	0,01	0	0,04	0,03	0,06	0,02	0	0,84	0	0	0,91	0,04	0,05	0,038
5	20583	0	0,17	0	0	0	0	0	0,83	0	0	0,83	0,17	0	0,042
5	15479	0,01	0,06	0	0	0,93	0	0	0	0	0,93	0	0,02	0,05	0,038
5	18770	0,04	0	0,02	0,01	0,93	0	0	0	0	0	0,79	0	0,21	0,044
5	7878	0,03	0	0,02	0	0,91	0	0,04	0	0	0,01	0,01	0,98	0	0,048
5	56061	0	0	0,01	0,14	0,85	0	0	0	0	0,14	0,09	0,77	0	0,042
5	11543	0,02	0,37	0	0,01	0,6	0	0	0	0	0	0,58	0,05	0,37	0,048
5	6888	0,02	0	0	0	0,09	0	0,04	0,85	0	0,04	0,1	0	0,86	0,044
5	12057	0,05	0	0,02	0	0,13	0	0	0,8	0	0	0,02	0	0,98	0,044
5	20728	0,01	0,05	0,03	0	0,9	0	0	0	0,01	0,51	0,43	0,05	0,01	0,048
5	24721	0,03	0	0,01	0,02	0,94	0	0	0	0	0,04	0,94	0	0,02	0,044

5	19608	0,06	0	0	0	0,94	0	0	0	0	0,02	0	0,97	0,01	0,048
5	15320	0,01	0,09	0,01	0	0,87	0,01	0	0,01	0	0,01	0,11	0,88	0	0,046
5	15474	0,01	0,06	0	0	0,92	0,01	0	0	0	0,91	0	0,01	0,08	0,036
5	12250	0,01	0	0	0,11	0,49	0,02	0	0,37	0	0,3	0,7	0	0	0,046
5	5964	0,05	0	0	0	0	0,16	0	0,79	0	0,03	0,1	0,87	0	0,048
5	8114	0,03	0,07	0	0	0,88	0,02	0	0	0	0	0,03	0,88	0,09	0,048
5	10771	0,03	0,97	0	0	0	0	0	0	0	0	0	0,04	0,96	0,044
5	18176	0,03	0	0	0,09	0,87	0	0	0,01	0	0,87	0	0,1	0,03	0,04
5	23859	0	0	0,04	0,05	0,91	0	0	0	0	0,04	0,91	0,05	0	0,042
5	24559	0	0,85	0	0,03	0	0,12	0	0	0	0	0	0	1	0,044
5	3479	0,05	0	0,02	0	0	0	0	0,93	0	0	0,02	0	0,98	0,044
5	19334	0	0	0	0,26	0,73	0	0	0	0,01	0,94	0,04	0,02	0	0,046
5	9159	0	0	0,01	0,06	0,93	0	0	0	0	0,06	0,92	0	0,02	0,044
5	29174	0	0,12	0	0,02	0,61	0,02	0	0,23	0	0	0,25	0,75	0	0,048
5	18703	0,02	0,06	0	0	0,69	0,21	0,01	0,01	0	0,06	0,21	0,7	0,03	0,046
5	16749	0,05	0	0,03	0,01	0,91	0	0	0	0	0,02	0,33	0,61	0,04	0,048
5	26126	0,05	0	0	0	0	0,95	0	0	0	0,95	0,05	0	0	0,044
5	33090	0,01	0,08	0	0	0,8	0	0	0,11	0	0,83	0	0,01	0,16	0,048
5	30397	0,02	0	0	0,27	0,71	0	0	0	0	0,98	0	0,02	0	0,046
5	21455	0	0	0,01	0,08	0,91	0	0	0	0	0,08	0,87	0,05	0	0,048
5	15911	0,06	0	0	0	0	0,94	0	0	0	0,06	0	0,94	0	0,048
5	5245	0	0	0,04	0	0	0,96	0	0	0	0,96	0,04	0	0	0,036
5	40970	0	0,13	0	0	0,81	0,06	0	0	0	0	0,19	0,81	0	0,04
5	18344	0	0,31	0	0	0,69	0	0	0	0	0,16	0,53	0	0,31	0,042
5	8703	0	0,14	0	0	0	0,86	0	0	0	0	0	1	0	0,044
5	9588	0	0,19	0,01	0	0,78	0	0	0	0,02	0	0,79	0,19	0,02	0,046
5	16193	0	0,33	0,01	0	0	0	0,03	0,63	0	0	0,99	0	0,01	0,042
5	24687	0	0	0	0,1	0,43	0,46	0,01	0	0	0,4	0,17	0	0,43	0,042
5	22158	0,04	0	0	0	0,54	0	0,02	0,4	0	0	0	0,55	0,45	0,046
5	27570	0,01	0	0	0	0,35	0,01	0,07	0,41	0,15	0,17	0,03	0,13	0,67	0,046
5	18990	0	0	0	0,25	0,16	0,56	0	0	0,03	0,77	0,18	0	0,05	0,048
5	12780	0,06	0	0	0,04	0	0,81	0	0,09	0	0,81	0	0,1	0,09	0,046
5	21592	0,01	0,06	0	0,06	0,04	0	0	0,83	0	0,82	0,06	0,1	0,02	0,048

5	17080	0	0,15	0,01	0	0,82	0	0	0	0,02	0,01	0,02	0,82	0,15	0,042
5	19017	0	0,09	0	0	0	0,26	0,03	0,61	0,01	0,61	0	0,11	0,28	0,038
5	9832	0	0,04	0	0,07	0,84	0	0	0,05	0	0,05	0,04	0,91	0	0,046
5	28296	0,06	0	0	0	0,33	0,6	0	0	0,01	0,92	0,08	0	0	0,048
5	24138	0,03	0	0	0	0,87	0	0,04	0	0,06	0,01	0,86	0,13	0	0,042
5	7355	0	0	0,01	0,04	0,89	0,05	0,01	0	0	0,07	0,05	0	0,88	0,046
5	74714	0,01	0,08	0,01	0,01	0,68	0,2	0	0	0,01	0,15	0,69	0,16	0	0,036
	19500,47	0,01952	0,0972	0,00929	0,03468	0,59019	0,09482	0,00477	0,14091	0,00861	0,24451	0,22335	0,25598	0,27615	0,044845

stern-judging

b	time_to_c(	(C,C)	(C,D)	(C,P)	(D,C)	(D,D)	(D,P)	(P,C)	(P,D)	(P,P)	S(G,G)	S(G,B)	S(B,G)	S(B,B)	
1	11088	0	0,04	0,01	0	0,95	0	0	0	0	0	0,05	0	0,95	0,008
1	1890	0	0,04	0	0	0,94	0,02	0	0	0	0,94	0,04	0	0,02	0,04
1	1325	0,01	0,04	0	0	0,95	0	0	0	0	0,01	0,21	0,02	0,76	0,022
1	7525	0	0	0	0,16	0,84	0	0	0	0	0,16	0,84	0	0	0,048
1	5950	0	0,04	0	0	0,95	0	0	0,01	0	0,95	0,05	0	0	0,044
1	4285	0	0,04	0	0	0,95	0,01	0	0	0	0,09	0,91	0	0	0,032
1	5261	0	0,04	0	0	0,96	0	0	0	0	0	0,04	0	0,96	0,006
1	2794	0	0,04	0	0	0,95	0	0	0	0,01	0	0,04	0,01	0,95	0,016
1	5254	0	0,04	0	0	0,96	0	0	0	0	0,01	0,99	0	0	0,028
1	5112	0	0	0	0,12	0,87	0	0,01	0	0	0,12	0,88	0	0	0,048
1	2017	0,01	0,04	0	0,01	0,93	0,01	0	0	0	0	0,99	0	0,01	0,04
1	5153	0	0,04	0	0,01	0,95	0	0	0	0	0,94	0,04	0,01	0,01	0,048
1	1806	0	0,04	0,01	0,03	0,88	0,03	0	0	0,01	0,1	0,2	0,01	0,69	0,048
1	4128	0	0,04	0	0,01	0,94	0	0	0	0,01	0	0,05	0,94	0,01	0,04
1	3363	0	0,04	0,01	0	0,89	0,06	0	0	0	0,01	0,04	0,93	0,02	0,04
1	5307	0	0,04	0	0	0,96	0	0	0	0	0	0,04	0	0,96	0,016
1	5662	0	0,04	0	0	0,95	0,01	0	0	0	0	0,04	0,96	0	0,024
1	6432	0	0,04	0,03	0	0	0,01	0	0,92	0	0,03	0,04	0,03	0,9	0,016
1	10443	0	0,04	0	0,01	0,91	0,01	0	0,01	0,02	0,01	0,95	0,03	0,01	0,028
1	5643	0	0,04	0	0,04	0,92	0	0	0	0	0,92	0,04	0,04	0	0,048
1	1485	0	0,04	0	0,06	0,88	0,02	0	0	0	0,02	0,92	0,06	0	0,038
1	1380	0	0,04	0,01	0	0,95	0	0	0	0	0,75	0,25	0	0	0,036
1	9728	0	0	0	0,39	0,61	0	0	0	0	1	0	0	0	0,048

1	5083	0	0,04	0	0	0,94	0	0	0,02	0	0,94	0,04	0,02	0	0,042
1	3120	0	0,04	0,01	0	0,95	0	0	0	0	0	0,95	0,04	0,01	0,024
1	7965	0	0,04	0	0	0,96	0	0	0	0	0	1	0	0	0,018
1	1688	0,01	0,04	0	0	0,94	0	0	0	0,01	0,01	0,75	0,24	0	0,034
1	5039	0	0,04	0	0	0	0,01	0	0,95	0	0	0,04	0,96	0	0,02
1	9847	0	0	0	0,38	0,62	0	0	0	0	1	0	0	0	0,048
1	3783	0	0,04	0	0,04	0,89	0	0,01	0	0,02	0	1	0	0	0,036
1	1544	0	0,01	0,02	0,15	0,81	0	0,01	0	0	0,92	0,05	0,01	0,02	0,046
1	1961	0	0,04	0	0	0,96	0	0	0	0	0	1	0	0	0,03
1	4632	0	0,04	0	0	0,96	0	0	0	0	0	1	0	0	0,022
1	3384	0	0,04	0	0,04	0,91	0,01	0	0	0	0,02	0,95	0,03	0	0,048
1	3763	0	0,03	0	0,15	0,82	0	0	0	0	0,82	0,04	0	0,14	0,046
1	4867	0	0,04	0	0	0,96	0	0	0	0	0	0,04	0,96	0	0,02
1	2030	0	0,04	0	0,01	0,92	0	0,03	0	0	0,04	0,04	0,92	0	0,046
1	5730	0	0,04	0	0	0,94	0	0	0,01	0,01	0,02	0,05	0	0,93	0,006
1	3220	0	0,03	0	0,09	0,88	0	0	0	0	0	0,91	0,09	0	0,048
1	5390	0	0,04	0,01	0	0,95	0	0	0	0	0	0,99	0,01	0	0,022
1	3523	0,01	0,04	0	0,01	0,93	0,01	0	0	0	0,01	0,26	0,01	0,72	0,034
1	1638	0	0,04	0	0	0,96	0	0	0	0	0	0,04	0	0,96	0,008
1	1592	0	0,04	0	0	0,96	0	0	0	0	0	0,23	0	0,77	0,006
1	5249	0	0,04	0	0	0,95	0	0,01	0	0	0,95	0,04	0,01	0	0,034
1	2001	0	0,04	0	0	0,95	0	0	0,01	0	0	0,05	0,95	0	0,018
1	3467	0	0,04	0	0	0,96	0	0	0	0	0	1	0	0	0,034
1	1821	0	0,04	0,01	0	0,95	0	0	0	0	0	0,05	0,01	0,94	0,006
1	3099	0	0,04	0	0	0,93	0	0,01	0,01	0,01	0,01	0,97	0,01	0,01	0,026
1	2371	0	0,04	0	0	0,95	0,01	0	0	0	0	0,05	0	0,95	0,004
1	1396	0,01	0,04	0	0	0,94	0	0,01	0	0	0,02	0,04	0,94	0	0,02
1	1975	0	0,04	0	0	0,96	0	0	0	0	0,04	0,94	0,02	0	0,026
1	1606	0	0,04	0	0	0,96	0	0	0	0	0	0,99	0	0,01	0,02
1	6630	0,01	0,04	0	0	0,93	0	0	0,02	0	0,01	0,04	0,45	0,5	0,026
1	3653	0	0,04	0	0,05	0,91	0	0	0	0	0,91	0,09	0	0	0,038
1	2609	0	0,04	0	0	0,93	0,01	0	0,02	0	0,02	0,04	0	0,94	0,014
1	2891	0	0,04	0	0,03	0,93	0	0	0	0	0,01	0,03	0,92	0,04	0,044

1	30618	0	0	0	0,08	0	0	0	0,92	0	0,18	0	0,08	0,74	0,048
1	6184	0	0,04	0,02	0,03	0,83	0	0	0,08	0	0,05	0,11	0	0,84	0,044
1	1974	0	0,04	0	0	0,96	0	0	0	0	0	0,04	0,96	0	0,014
1	6273	0	0,04	0	0	0,96	0	0	0	0	0	1	0	0	0,02
1	5850	0	0,04	0	0,01	0,95	0	0	0	0	0,01	0,04	0,95	0	0,032
1	5771	0,01	0,04	0,01	0	0,94	0	0	0	0	0,1	0,78	0,11	0,01	0,024
1	2222	0	0,04	0	0,03	0,93	0	0	0	0	0,57	0,43	0	0	0,046
1	2342	0	0,03	0	0,12	0,85	0	0	0	0	0,12	0,03	0,85	0	0,044
1	2670	0	0,04	0,02	0	0,94	0	0	0	0	0,12	0,88	0	0	0,034
1	3739	0	0,04	0	0,02	0,81	0,12	0	0,01	0	0,87	0,05	0,02	0,06	0,04
1	5713	0	0,04	0	0	0,95	0	0	0,01	0	0,01	0,04	0,95	0	0,028
1	13885	0	0,04	0	0	0,77	0	0,01	0,18	0	0	0,04	0,6	0,36	0,028
1	1391	0	0,04	0	0	0,96	0	0	0	0	0	0,04	0,96	0	0,024
1	1524	0	0,04	0,01	0	0,91	0,03	0	0,01	0	0,94	0,04	0,01	0,01	0,048
1	1021	0	0,04	0	0	0,86	0,08	0	0	0,02	0,96	0,04	0	0	0,04
1	1775	0	0,04	0	0	0,96	0	0	0	0	0	1	0	0	0,028
1	2814	0	0,04	0	0	0,94	0,02	0	0	0	0	1	0	0	0,02
1	4162	0	0,04	0	0	0,95	0	0,01	0	0	0	0,05	0,95	0	0,036
1	4032	0,02	0,04	0	0,02	0,92	0	0	0	0	0,02	0,96	0	0,02	0,046
1	4658	0	0,04	0	0	0,95	0	0,01	0	0	0,14	0,11	0	0,75	0,018
1	14864	0	0,04	0,01	0	0,94	0	0,01	0	0	0,01	0,31	0,67	0,01	0,034
1	2022	0	0,04	0	0,01	0,89	0,06	0	0	0	0,02	0,98	0	0	0,026
1	6051	0,02	0,03	0	0,01	0,94	0	0	0	0	0,94	0,03	0,02	0,01	0,036
1	3133	0,01	0,04	0	0	0,94	0,01	0	0	0	0	0	0,02	0,98	0,016
1	3510	0	0,04	0	0	0,96	0	0	0	0	0	0,04	0,96	0	0,016
1	4379	0	0,04	0	0	0,95	0	0,01	0	0	0,01	0,99	0	0	0,03
1	1014	0	0,04	0	0,02	0,94	0	0	0	0	0,1	0,9	0	0	0,032
1	7698	0,01	0,04	0	0	0,93	0	0	0,02	0	0,91	0,06	0,01	0,02	0,044
1	1153	0	0,04	0	0	0,96	0	0	0	0	0	0,89	0,11	0	0,028
1	9428	0	0,04	0,01	0	0,94	0	0,01	0	0	0,01	0,96	0,03	0	0,024
1	3677	0	0,04	0	0	0,95	0	0	0	0,01	0,03	0,02	0	0,95	0,014
1	4781	0	0,04	0	0	0,96	0	0	0	0	0	0,04	0,96	0	0,034
1	7772	0	0,01	0	0,14	0,84	0,01	0	0	0	0,13	0,86	0,01	0	0,048

1	5853	0	0,04	0	0	0,96	0	0	0	0	0	1	0	0	0,028
1	1952	0,01	0,04	0	0,02	0,93	0	0	0	0	0,01	0,99	0	0	0,044
1	8606	0	0,04	0	0	0,96	0	0	0	0	0,04	0	0	0,96	0,012
1	5678	0,01	0,01	0	0,06	0,92	0	0	0	0	0,06	0,01	0	0,93	0,042
1	2624	0	0,04	0,01	0,01	0,93	0	0,01	0	0	0,94	0,05	0	0,01	0,046
1	929	0	0,04	0	0	0,96	0	0	0	0	0	0,06	0	0,94	0,008
1	6678	0	0,04	0	0,01	0,95	0	0	0	0	0	0,04	0,93	0,03	0,022
1	2721	0	0	0	0,13	0,87	0	0	0	0	0,13	0,08	0,79	0	0,042
1	827	0	0,04	0	0	0,96	0	0	0	0	0	1	0	0	0,03
1	2901	0	0,04	0	0,03	0,91	0,01	0	0	0,01	0,04	0,04	0,91	0,01	0,044
1	1921	0,01	0,04	0	0,04	0,91	0	0	0	0	0	0,04	0,92	0,04	0,046
1,25	8973	0	0,04	0	0	0,95	0,01	0	0	0	0	0,04	0,95	0,01	0,026
1,25	2185	0	0,04	0	0,06	0,89	0	0	0	0,01	0,01	0,04	0,95	0	0,044
1,25	1429	0	0,04	0,01	0	0,94	0	0,01	0	0	0	0,99	0,01	0	0,032
1,25	2698	0	0,04	0	0	0,96	0	0	0	0	0,92	0,08	0	0	0,042
1,25	7123	0	0,04	0	0	0,94	0	0,01	0	0,01	0	0,02	0,03	0,95	0,022
1,25	19502	0	0,04	0	0	0	0,27	0	0,69	0	0,01	0,82	0	0,17	0,018
1,25	4821	0,01	0,04	0,02	0	0,93	0	0	0	0	0	0,96	0	0,04	0,042
1,25	3741	0	0,04	0	0	0,96	0	0	0	0	0	0,04	0,96	0	0,03
1,25	11232	0	0,04	0	0	0,96	0	0	0	0	0,96	0,04	0	0	0,04
1,25	2153	0	0,04	0	0	0,96	0	0	0	0	0,01	0,99	0	0	0,024
1,25	5474	0	0,04	0	0	0,96	0	0	0	0	0,01	0,04	0,95	0	0,024
1,25	1638	0	0,04	0	0	0,96	0	0	0	0	0	0,04	0,96	0	0,04
1,25	2089	0	0,04	0	0,01	0,95	0	0	0	0	0,02	0,02	0,95	0,01	0,02
1,25	12903	0,02	0,04	0	0,03	0,9	0	0	0,01	0	0	0,49	0,5	0,01	0,046
1,25	2539	0	0,04	0	0	0,87	0,08	0,01	0	0	0,87	0,12	0	0,01	0,048
1,25	6513	0	0,04	0	0	0,95	0	0	0,01	0	0,01	0,02	0,01	0,96	0,008
1,25	5651	0	0,04	0	0	0,94	0,01	0	0,01	0	0	0,06	0	0,94	0,006
1,25	4416	0	0,04	0	0	0,95	0	0	0,01	0	0,01	0,04	0,95	0	0,022
1,25	1686	0,02	0,03	0	0	0,92	0,02	0	0,01	0	0,96	0,03	0	0,01	0,034
1,25	4337	0	0,04	0,01	0,02	0,92	0	0,01	0	0	0,02	0,04	0,03	0,91	0,04
1,25	16727	0,01	0,04	0	0	0,94	0	0	0,01	0	0,01	0,92	0	0,07	0,044
1,25	2703	0	0,04	0	0,01	0,94	0,01	0	0	0	0	0,89	0,09	0,02	0,032

1,25	1812	0	0,04	0	0	0,96	0	0	0	0	0,81	0,09	0	0,1	0,042
1,25	13122	0	0	0	0,12	0	0,33	0	0,55	0	0,12	0	0,55	0,33	0,046
1,25	1363	0	0,04	0	0	0,96	0	0	0	0	0	1	0	0	0,018
1,25	2384	0	0,04	0,01	0	0,94	0	0	0	0,01	0	0,06	0	0,94	0,018
1,25	3290	0	0,04	0	0,04	0,92	0	0	0	0	0,01	0,95	0,03	0,01	0,048
1,25	27055	0	0	0,02	0,21	0,75	0	0	0,02	0	0,79	0,21	0	0	0,04
1,25	1238	0,01	0,04	0	0	0,95	0	0	0	0	0	0,04	0,01	0,95	0,018
1,25	2582	0	0,04	0	0	0,96	0	0	0	0	0	0,04	0	0,96	0,01
1,25	3592	0	0,04	0	0	0,92	0	0	0,04	0	0,04	0,95	0,01	0	0,022
1,25	1972	0	0,04	0	0	0,94	0	0	0,02	0	0,02	0,04	0	0,94	0,008
1,25	2063	0	0,04	0	0,01	0,95	0	0	0	0	0	0,04	0,01	0,95	0,022
1,25	3553	0	0,04	0	0	0,96	0	0	0	0	0	0,04	0	0,96	0,01
1,25	2656	0	0,04	0	0,02	0,94	0	0	0	0	0,01	0,05	0,94	0	0,034
1,25	6540	0	0,04	0	0,03	0,93	0	0	0	0	0,03	0,97	0	0	0,042
1,25	4379	0	0,04	0	0	0,96	0	0	0	0	0	0	0	1	0,008
1,25	3094	0	0,04	0	0	0,91	0	0	0,05	0	0	1	0	0	0,022
1,25	3131	0	0,04	0,02	0,07	0,83	0,04	0	0	0	0,92	0,03	0,05	0	0,048
1,25	1975	0	0,04	0	0	0,96	0	0	0	0	0	1	0	0	0,022
1,25	6677	0	0,04	0	0	0,96	0	0	0	0	0	0,04	0	0,96	0,008
1,25	1766	0	0,04	0	0	0,86	0,08	0,01	0	0,01	0,95	0,04	0,01	0	0,036
1,25	1374	0	0,04	0,01	0	0,95	0	0	0	0	0,95	0,04	0	0,01	0,036
1,25	4999	0	0,04	0	0,01	0,95	0	0	0	0	0,95	0,05	0	0	0,04
1,25	11307	0,01	0,04	0	0	0,94	0,01	0	0	0	0,94	0,05	0	0,01	0,048
1,25	6236	0	0	0	0,11	0,8	0,09	0	0	0	0,06	0,8	0,05	0,09	0,036
1,25	2719	0	0,04	0	0	0,96	0	0	0	0	0,96	0,04	0	0	0,04
1,25	4184	0	0,04	0	0	0,95	0	0	0,01	0	0,95	0,04	0,01	0	0,046
1,25	1325	0	0,04	0	0	0,96	0	0	0	0	0,96	0,04	0	0	0,032
1,25	5936	0	0,04	0	0	0,96	0	0	0	0	0	0,04	0	0,96	0,006
1,25	3471	0,01	0,04	0,01	0	0,94	0	0	0	0	0	0,06	0,94	0	0,038
1,25	12992	0	0,04	0	0	0,96	0	0	0	0	0,96	0,04	0	0	0,036
1,25	1259	0	0,04	0	0	0,95	0	0,01	0	0	0	0,05	0	0,95	0,02
1,25	1616	0	0,04	0	0	0,95	0,01	0	0	0	0	0,04	0,96	0	0,024
1,25	1743	0	0,04	0	0,01	0,95	0	0	0	0	0	1	0	0	0,02

1,25	3632	0	0,04	0	0	0,96	0	0	0	0	0	0,04	0,96	0	0,026
1,25	3298	0	0,04	0	0	0,95	0,01	0	0	0	0,04	0,03	0,01	0,92	0,008
1,25	2459	0	0,04	0	0	0,95	0	0	0	0,01	0,95	0,04	0,01	0	0,038
1,25	1952	0	0,04	0	0	0,96	0	0	0	0	0,09	0,04	0	0,87	0,014
1,25	1564	0	0,04	0	0	0,96	0	0	0	0	0,96	0,04	0	0	0,028
1,25	13205	0	0,04	0	0	0,96	0	0	0	0	0	0,04	0	0,96	0,006
1,25	1589	0	0,04	0,01	0	0,94	0	0	0	0,01	0	0,06	0,94	0	0,034
1,25	9894	0	0,04	0	0	0,96	0	0	0	0	0,61	0,04	0,35	0	0,042
1,25	4945	0	0,04	0	0	0,96	0	0	0	0	0,02	0,02	0	0,96	0,012
1,25	1495	0	0,04	0,01	0	0,95	0	0	0	0	0	0,99	0,01	0	0,026
1,25	3447	0	0,04	0	0	0,95	0,01	0	0	0	0,42	0,07	0	0,51	0,022
1,25	2095	0	0,04	0	0	0,87	0,09	0	0	0	0,87	0,09	0	0,04	0,032
1,25	1988	0	0,04	0	0	0,96	0	0	0	0	0	0,04	0,96	0	0,028
1,25	4473	0	0,04	0,01	0	0,95	0	0	0	0	0	0,04	0	0,96	0,01
1,25	6629	0	0,04	0	0	0,96	0	0	0	0	0	0,04	0	0,96	0,012
1,25	6481	0	0,04	0	0	0,95	0	0,01	0	0	0,95	0,04	0,01	0	0,044
1,25	1137	0	0,04	0	0	0,96	0	0	0	0	0,82	0,04	0	0,14	0,022
1,25	2540	0	0,04	0	0	0,89	0,02	0	0,05	0	0,94	0,04	0	0,02	0,044
1,25	14649	0,01	0,04	0	0	0	0	0	0,95	0	0,06	0,17	0,77	0	0,048
1,25	1142	0,02	0,03	0	0	0,92	0,03	0	0	0	0,93	0,05	0,01	0,01	0,032
1,25	2181	0	0,04	0	0	0,96	0	0	0	0	0	0,94	0,05	0,01	0,022
1,25	5372	0	0,03	0,03	0	0,94	0	0	0	0	0,94	0,03	0	0,03	0,044
1,25	5781	0	0,04	0,02	0	0,94	0	0	0	0	0,94	0,04	0	0,02	0,048
1,25	1746	0,01	0,04	0	0	0,95	0	0	0	0	0	0,15	0	0,85	0,022
1,25	3371	0	0,04	0	0	0,96	0	0	0	0	0	0,03	0,01	0,96	0,01
1,25	2389	0	0,04	0,01	0	0,95	0	0	0	0	0,11	0,04	0,85	0	0,034
1,25	3929	0	0,04	0,01	0,04	0,81	0	0	0,1	0	0,04	0,03	0,12	0,81	0,046
1,25	12052	0,01	0	0,01	0,12	0,84	0,01	0	0,01	0	0,11	0,03	0,85	0,01	0,048
1,25	2309	0	0,04	0,01	0,01	0,94	0	0	0	0	0,01	0,98	0	0,01	0,044
1,25	3089	0,01	0,04	0	0	0,95	0	0	0	0	0,01	0,98	0	0,01	0,036
1,25	7569	0	0,04	0	0	0,96	0	0	0	0	0	0,02	0	0,98	0,004
1,25	6276	0	0,04	0	0	0,96	0	0	0	0	0	0,94	0	0,06	0,018
1,25	3020	0,01	0,04	0	0,01	0,94	0	0	0	0	0,01	0,04	0,01	0,94	0,03

1,25	7658	0	0	0	0,13	0,87	0	0	0	0	0,13	0,87	0	0	0,044
1,25	25334	0,01	0,04	0	0	0,95	0	0	0	0	0	0,05	0	0,95	0,02
1,25	2440	0	0,04	0	0	0,96	0	0	0	0	0,96	0,04	0	0	0,046
1,25	2961	0	0,04	0	0	0,95	0,01	0	0	0	0,06	0,04	0,89	0,01	0,032
1,25	1452	0	0,04	0	0,01	0,82	0,12	0,01	0	0	0,84	0,06	0	0,1	0,04
1,25	2883	0	0,04	0	0	0,96	0	0	0	0	0	0,04	0	0,96	0,014
1,25	5016	0	0,04	0	0	0,92	0	0	0,04	0	0	0,08	0,92	0	0,032
1,25	2346	0	0,04	0	0	0,96	0	0	0	0	0,96	0,04	0	0	0,034
1,25	3840	0,01	0,04	0	0	0,95	0	0	0	0	0,29	0,03	0	0,68	0,02
1,25	4948	0	0,04	0	0	0,95	0	0,01	0	0	0	0,04	0,02	0,94	0,014
1,25	3374	0	0,04	0	0	0,96	0	0	0	0	0	0,04	0,96	0	0,022
1,25	2120	0	0,04	0	0	0,96	0	0	0	0	0	0,03	0,97	0	0,028
1,5	19528	0	0,02	0	0,21	0,77	0	0	0	0	0,77	0,02	0,21	0	0,04
1,5	11429	0	0,04	0,01	0	0,95	0	0	0	0	0,94	0,05	0	0,01	0,038
1,5	3267	0	0,04	0	0,01	0,95	0	0	0	0	0	0,93	0,01	0,06	0,026
1,5	3019	0	0,04	0	0	0,91	0	0,05	0	0	0,05	0,54	0,41	0	0,048
1,5	4264	0	0,04	0	0	0,95	0,01	0	0	0	0,01	0,97	0,02	0	0,024
1,5	1468	0	0,04	0	0	0,5	0	0,01	0,4	0,05	0,02	0,05	0,5	0,43	0,032
1,5	3580	0	0,04	0	0	0,96	0	0	0	0	0	0,04	0	0,96	0,014
1,5	2837	0,01	0,04	0	0	0,93	0,02	0	0	0	0,15	0,04	0,78	0,03	0,03
1,5	6864	0	0,04	0,01	0	0,95	0	0	0	0	0,95	0,04	0	0,01	0,044
1,5	1489	0,01	0,04	0	0	0,94	0	0	0,01	0	0	0,04	0,87	0,09	0,03
1,5	5027	0	0,04	0	0	0,96	0	0	0	0	0	0,04	0,18	0,78	0,018
1,5	1245	0	0,04	0	0	0,95	0,01	0	0	0	0	0,03	0,97	0	0,016
1,5	4401	0	0,04	0	0,01	0,94	0,01	0	0	0	0	0,15	0,01	0,84	0,008
1,5	5464	0,01	0,04	0	0	0,95	0	0	0	0	0,95	0	0,04	0,01	0,036
1,5	3033	0	0,04	0	0	0,91	0,01	0	0,04	0	0,11	0,04	0,54	0,31	0,018
1,5	1731	0	0,04	0	0	0,96	0	0	0	0	0	1	0	0	0,012
1,5	1798	0	0,04	0	0	0,96	0	0	0	0	0,01	0,04	0	0,95	0,014
1,5	12928	0	0	0	0,13	0,86	0	0	0	0,01	0,13	0,86	0,01	0	0,044
1,5	2314	0	0,04	0	0	0,95	0	0	0,01	0	0,01	0,24	0,75	0	0,014
1,5	2394	0	0,04	0	0	0,93	0,03	0	0	0	0,04	0,04	0,92	0	0,018
1,5	5612	0	0,04	0,01	0	0,92	0	0,03	0	0	0,04	0,05	0,91	0	0,044

1,5	3059	0	0,04	0	0	0,96	0	0	0	0	0	0,88	0,12	0	0,03
1,5	5771	0	0,04	0	0,07	0	0,67	0	0,2	0,02	0,87	0,13	0	0	0,048
1,5	2448	0	0,04	0	0,13	0,83	0	0	0	0	0,95	0,05	0	0	0,044
1,5	5830	0,01	0	0	0,18	0,81	0	0	0	0	0,74	0,08	0	0,18	0,04
1,5	2942	0	0,04	0	0	0,94	0,02	0	0	0	0,81	0,06	0	0,13	0,034
1,5	12483	0	0,04	0	0	0,95	0	0	0	0,01	0,95	0,04	0	0,01	0,022
1,5	3131	0	0,04	0	0	0,96	0	0	0	0	0	0,59	0,41	0	0,032
1,5	2107	0	0,04	0	0	0,96	0	0	0	0	0	0,04	0,96	0	0,03
1,5	5798	0	0,04	0	0	0,96	0	0	0	0	0	0,04	0,72	0,24	0,018
1,5	3534	0	0,04	0	0	0,96	0	0	0	0	0	0,04	0	0,96	0,012
1,5	6374	0	0,04	0	0,01	0,95	0	0	0	0	0	0,99	0,01	0	0,018
1,5	6570	0	0,02	0,04	0,01	0,88	0,05	0	0	0	0,95	0,02	0,02	0,01	0,04
1,5	4162	0	0,04	0	0	0,94	0	0	0,02	0	0,02	0,96	0,02	0	0,026
1,5	3958	0	0,04	0	0	0,96	0	0	0	0	0	1	0	0	0,026
1,5	13772	0	0,04	0,01	0	0,92	0	0	0,03	0	0,06	0	0	0,94	0,018
1,5	2071	0	0,04	0,01	0	0,95	0	0	0	0	0,47	0,53	0	0	0,04
1,5	5610	0	0,04	0	0,01	0,94	0,01	0	0	0	0,01	0,05	0	0,94	0,022
1,5	1891	0	0,04	0	0,01	0,95	0	0	0	0	0,01	0,76	0	0,23	0,028
1,5	4877	0	0,04	0	0	0,96	0	0	0	0	0	0,04	0	0,96	0,006
1,5	2263	0	0,04	0	0	0,95	0	0	0,01	0	0	0,04	0,01	0,95	0,008
1,5	1974	0	0,04	0	0,05	0,89	0	0	0,01	0,01	0,61	0,05	0,29	0,05	0,038
1,5	2059	0	0,04	0	0	0,96	0	0	0	0	0,03	0,04	0	0,93	0,006
1,5	8670	0	0,04	0,01	0	0,93	0	0,02	0	0	0	0,06	0,94	0	0,038
1,5	4796	0	0,04	0	0	0,94	0	0	0,02	0	0	0,06	0,94	0	0,026
1,5	5973	0	0,04	0	0	0,96	0	0	0	0	0	0,04	0	0,96	0,02
1,5	1968	0	0,03	0	0,13	0,81	0	0	0	0,03	0,73	0,03	0,24	0	0,048
1,5	982	0	0,04	0	0	0,96	0	0	0	0	0	0,04	0,96	0	0,034
1,5	1932	0	0,04	0	0	0,96	0	0	0	0	0	0,04	0,01	0,95	0,012
1,5	1709	0	0,04	0	0	0,96	0	0	0	0	0,02	0,02	0	0,96	0,006
1,5	2270	0	0,04	0	0	0,96	0	0	0	0	0,96	0,04	0	0	0,042
1,5	6618	0	0,04	0,01	0	0,95	0	0	0	0	0	0,04	0,01	0,95	0,012
1,5	7691	0	0,04	0	0	0,95	0	0	0,01	0	0,01	0,99	0	0	0,02
1,5	23240	0	0,04	0	0	0,96	0	0	0	0	0,4	0	0	0,6	0,028

1,5	1513	0	0,04	0	0	0,95	0	0	0,01	0	0	0,64	0,36	0	0,032
1,5	7406	0	0,04	0	0	0,95	0	0	0,01	0	0,02	0	0,01	0,97	0,01
1,5	7754	0,01	0,04	0	0	0,95	0	0	0	0	0	1	0	0	0,032
1,5	7409	0	0,04	0	0	0,96	0	0	0	0	0	0,04	0	0,96	0,01
1,5	1830	0	0,04	0	0	0,94	0,02	0	0	0	0,94	0,04	0,02	0	0,036
1,5	1512	0	0,04	0	0	0,94	0,01	0	0,01	0	0,5	0,07	0,43	0	0,024
1,5	1471	0	0,04	0	0	0,96	0	0	0	0	0	0,67	0,33	0	0,02
1,5	4234	0	0,04	0	0	0,96	0	0	0	0	0,54	0,46	0	0	0,026
1,5	5779	0	0,04	0	0	0,94	0,02	0	0	0	0	0,05	0,01	0,94	0,012
1,5	3447	0	0,04	0	0	0,96	0	0	0	0	0	0,04	0	0,96	0,006
1,5	1354	0	0,04	0	0	0,88	0,08	0	0	0	0,09	0,04	0,08	0,79	0,006
1,5	6212	0,02	0,04	0	0	0,94	0	0	0	0	0,96	0,04	0	0	0,046
1,5	3723	0	0,04	0	0	0,95	0	0	0,01	0	0,01	0,04	0,11	0,84	0,024
1,5	1360	0	0,04	0	0	0,95	0	0	0,01	0	0	0,05	0,95	0	0,024
1,5	3043	0,01	0,04	0	0	0,95	0	0	0	0	0,13	0,04	0	0,83	0,022
1,5	3142	0	0,04	0	0	0,95	0	0	0	0,01	0,97	0,03	0	0	0,044
1,5	11574	0,02	0	0	0,09	0,88	0	0	0	0,01	0,09	0,9	0,01	0	0,046
1,5	1959	0	0,04	0,01	0	0,93	0,01	0	0	0,01	0,41	0,06	0,01	0,52	0,032
1,5	6211	0,01	0,04	0	0	0,93	0	0	0	0,02	0,01	0,03	0,95	0,01	0,042
1,5	15186	0	0,04	0	0	0,96	0	0	0	0	0	0,04	0	0,96	0,004
1,5	3165	0	0,04	0	0	0,96	0	0	0	0	0,07	0,02	0	0,91	0,004
1,5	4271	0	0,04	0	0	0,94	0,01	0,01	0	0	0	1	0	0	0,036
1,5	6069	0	0,04	0	0	0,96	0	0	0	0	0,01	0,95	0,04	0	0,02
1,5	3004	0	0,04	0	0	0,96	0	0	0	0	0	0,04	0,96	0	0,034
1,5	3810	0	0,04	0	0	0,96	0	0	0	0	0	0,04	0	0,96	0,008
1,5	6204	0	0,03	0,01	0,06	0,85	0	0,04	0	0,01	0,01	0,09	0,89	0,01	0,048
1,5	1047	0	0,04	0	0	0,95	0	0	0,01	0	0,95	0,03	0,01	0,01	0,046
1,5	2264	0	0,04	0	0,02	0,94	0	0	0	0	0,19	0,04	0	0,77	0,032
1,5	2116	0,01	0,04	0	0	0,94	0,01	0	0	0	0,01	0,05	0,02	0,92	0,02
1,5	10260	0	0,04	0	0	0,96	0	0	0	0	0,01	0,99	0	0	0,032
1,5	8538	0	0,04	0	0	0,96	0	0	0	0	0	0,01	0,03	0,96	0,016
1,5	9713	0,01	0,04	0,01	0	0,94	0	0	0	0	0	0,04	0,94	0,02	0,034
1,5	2334	0	0,04	0,01	0	0,95	0	0	0	0	0,01	0,99	0	0	0,024

1,5	2163	0	0,04	0	0	0,95	0	0	0,01	0	0	0,05	0	0,95	0,01
1,5	8102	0	0,04	0	0	0,95	0	0	0,01	0	0	0,04	0,96	0	0,022
1,5	3232	0	0,04	0,01	0	0,95	0	0	0	0	0	0,04	0,01	0,95	0,016
1,5	4815	0,01	0,04	0	0	0,95	0	0	0	0	0,46	0,06	0	0,48	0,044
1,5	3525	0	0,04	0	0,02	0,94	0	0	0	0	0,94	0,04	0,02	0	0,032
1,5	8931	0	0,04	0	0,01	0,95	0	0	0	0	0	0,99	0	0,01	0,014
1,5	2315	0	0,04	0	0	0,73	0	0	0,23	0	0,23	0,77	0	0	0,026
1,5	2057	0	0,04	0	0,01	0,92	0	0	0,03	0	0,1	0,07	0	0,83	0,024
1,5	6253	0,01	0,04	0	0	0,95	0	0	0	0	0	0,97	0,01	0,02	0,042
1,5	9263	0	0,04	0	0	0,96	0	0	0	0	0,96	0,04	0	0	0,034
1,5	2048	0	0,04	0	0	0,95	0,01	0	0	0	0,02	0,03	0	0,95	0,006
1,5	16668	0	0,04	0	0	0,96	0	0	0	0	0,96	0,04	0	0	0,044
1,5	1560	0	0,04	0	0	0,95	0	0,01	0	0	0,01	0,04	0,95	0	0,026
1,75	8237	0	0,04	0	0,01	0,95	0	0	0	0	0	0,04	0,95	0,01	0,046
1,75	1773	0,01	0,04	0	0	0,95	0	0	0	0	0	0,03	0,48	0,49	0,032
1,75	9283	0,01	0	0	0,14	0,23	0,57	0	0,04	0,01	0,41	0,57	0,02	0	0,038
1,75	4111	0	0,04	0	0,01	0,95	0	0	0	0	0	0,43	0,57	0	0,02
1,75	1626	0	0,04	0	0	0,96	0	0	0	0	0	0,04	0,96	0	0,024
1,75	1962	0	0,04	0	0,01	0,95	0	0	0	0	0	0,02	0,95	0,03	0,024
1,75	4111	0	0,04	0	0	0,96	0	0	0	0	0	0,04	0	0,96	0,008
1,75	2672	0	0,04	0	0	0,92	0	0,01	0,03	0	0,9	0,08	0	0,02	0,036
1,75	11708	0	0,04	0	0,06	0,89	0,01	0	0	0	0,1	0,1	0	0,8	0,046
1,75	1908	0	0,04	0	0	0,94	0,01	0	0	0,01	0	0,4	0,6	0	0,028
1,75	8891	0	0,04	0	0	0,96	0	0	0	0	0	0,04	0,96	0	0,036
1,75	3970	0	0,04	0	0	0,96	0	0	0	0	0	0,46	0,54	0	0,036
1,75	2768	0	0,04	0	0	0,96	0	0	0	0	0,02	0,04	0	0,94	0,002
1,75	12259	0	0,04	0	0	0,93	0	0	0	0,03	0,07	0,04	0,89	0	0,02
1,75	1904	0	0,04	0,01	0	0,94	0	0	0,01	0	0	0,04	0	0,96	0,006
1,75	9359	0	0,04	0	0	0,96	0	0	0	0	0	0,04	0,96	0	0,028
1,75	2744	0	0,04	0	0	0,96	0	0	0	0	0	0,04	0,96	0	0,024
1,75	2332	0	0,04	0	0	0,96	0	0	0	0	0,96	0,04	0	0	0,038
1,75	2096	0	0,04	0	0	0,95	0	0	0	0,01	0	0,01	0,01	0,98	0,01
1,75	13854	0	0,04	0	0,04	0,92	0	0	0	0	0	0,96	0,04	0	0,046

1,75	10244	0	0	0	0,11	0,89	0	0	0	0	0,12	0	0,88	0	0,048
1,75	6158	0	0,04	0	0,11	0,81	0	0	0,03	0,01	0,63	0,24	0,02	0,11	0,044
1,75	4910	0,01	0,04	0	0,08	0,87	0	0	0	0	0	0,04	0,95	0,01	0,044
1,75	12748	0	0,04	0	0	0,94	0,01	0,01	0	0	0	0,99	0	0,01	0,038
1,75	2758	0	0,04	0,01	0,01	0,84	0,09	0	0,01	0	0,06	0,06	0,88	0	0,036
1,75	3981	0,02	0,04	0	0	0,94	0	0	0	0	0,01	0,99	0	0	0,042
1,75	2450	0,01	0,04	0	0	0,94	0,01	0	0	0	0	0,91	0,01	0,08	0,03
1,75	2820	0	0,04	0	0	0,95	0	0,01	0	0	0	0,99	0,01	0	0,034
1,75	2240	0	0,04	0	0	0,94	0	0,02	0	0	0,26	0,7	0	0,04	0,042
1,75	12889	0,01	0,04	0	0	0,95	0	0	0	0	0,01	0,04	0,69	0,26	0,034
1,75	25297	0	0,04	0	0,03	0,9	0,01	0	0,01	0,01	0,02	0,95	0	0,03	0,044
1,75	2289	0	0,04	0	0	0,96	0	0	0	0	0	0,21	0	0,79	0,018
1,75	9061	0,01	0	0	0,12	0,84	0	0,01	0,02	0	0,12	0,83	0,01	0,04	0,046
1,75	6065	0	0,04	0	0	0,96	0	0	0	0	0,96	0,04	0	0	0,046
1,75	5591	0	0,04	0	0	0,96	0	0	0	0	0	1	0	0	0,034
1,75	2685	0,02	0,03	0	0,03	0,91	0,01	0	0	0	0,03	0,03	0,03	0,91	0,046
1,75	7547	0	0,04	0	0	0,95	0	0	0	0,01	0,01	0,04	0	0,95	0,02
1,75	14101	0,01	0,04	0	0	0,95	0	0	0	0	0	0,02	0	0,98	0,014
1,75	2047	0	0,04	0	0,2	0,76	0	0	0	0	0,95	0,04	0	0,01	0,048
1,75	4001	0	0,04	0	0,07	0,82	0	0,01	0,06	0	0,01	0,99	0	0	0,044
1,75	1965	0	0,04	0	0	0,96	0	0	0	0	0	0,04	0,04	0,92	0,008
1,75	4550	0	0,04	0	0	0,96	0	0	0	0	0	0,04	0	0,96	0,012
1,75	10128	0	0,04	0	0,02	0,94	0	0	0	0	0,06	0,04	0,9	0	0,036
1,75	1309	0	0,04	0	0	0,96	0	0	0	0	0	1	0	0	0,024
1,75	3302	0	0,04	0	0	0,95	0	0,01	0	0	0	1	0	0	0,026
1,75	2307	0	0,04	0	0	0,96	0	0	0	0	0	1	0	0	0,022
1,75	2837	0	0,04	0	0,02	0,94	0	0	0	0	0	0,06	0,93	0,01	0,026
1,75	9280	0	0,04	0	0	0,96	0	0	0	0	0,87	0,04	0	0,09	0,042
1,75	1863	0	0,04	0,01	0	0,94	0,01	0	0	0	0,01	0,04	0,01	0,94	0,01
1,75	2372	0	0,04	0	0	0,96	0	0	0	0	0	0,04	0	0,96	0,004
1,75	3175	0	0,04	0	0	0,94	0,02	0	0	0	0,95	0,05	0	0	0,03
1,75	6221	0	0,04	0	0	0,93	0,01	0	0,02	0	0,03	0,04	0,93	0	0,032
1,75	7970	0,01	0,04	0,01	0,01	0,93	0	0	0	0	0,01	0	0	0,99	0,04

1,75	2488	0	0,04	0	0	0,96	0	0	0	0	0	1	0	0	0,03
1,75	10446	0	0,04	0	0	0,95	0	0	0	0,01	0	0,99	0,01	0	0,016
1,75	7138	0	0,04	0	0	0,94	0,01	0,01	0	0	0	0,04	0,93	0,03	0,024
1,75	3637	0	0,04	0	0,05	0,9	0,01	0	0	0	0,3	0,48	0,17	0,05	0,044
1,75	1497	0	0,04	0	0	0,96	0	0	0	0	0	0,04	0	0,96	0,008
1,75	3467	0	0,04	0	0	0,96	0	0	0	0	0	0,08	0	0,92	0,004
1,75	2704	0	0,04	0	0,01	0,95	0	0	0	0	0	0,93	0,05	0,02	0,03
1,75	2111	0	0,03	0,01	0,15	0,81	0	0	0	0	0,77	0,03	0,15	0,05	0,048
1,75	19204	0	0,04	0	0,09	0,25	0,08	0	0,54	0	0,02	0,01	0,86	0,11	0,044
1,75	4028	0	0,04	0	0	0,96	0	0	0	0	0	0,54	0	0,46	0,014
1,75	5386	0	0,04	0	0	0,95	0	0,01	0	0	0	0,04	0,95	0,01	0,028
1,75	4820	0	0,04	0	0	0,96	0	0	0	0	0	1	0	0	0,026
1,75	4700	0	0,04	0	0	0,94	0,01	0,01	0	0	0	0,06	0,94	0	0,032
1,75	9394	0	0,04	0,01	0	0,95	0	0	0	0	0	0,04	0,96	0	0,022
1,75	1235	0	0,04	0,02	0	0,94	0	0	0	0	0,02	0,04	0,94	0	0,042
1,75	2204	0	0,04	0	0	0,96	0	0	0	0	0	0,03	0	0,97	0,012
1,75	2463	0,01	0,04	0	0	0,95	0	0	0	0	0,94	0,06	0	0	0,04
1,75	4855	0	0,04	0	0	0,96	0	0	0	0	0	0,04	0	0,96	0,012
1,75	10004	0	0,04	0	0	0,94	0	0	0,02	0	0,01	0,99	0	0	0,024
1,75	5076	0	0,04	0	0	0,91	0,01	0	0,03	0,01	0,93	0,04	0	0,03	0,04
1,75	5132	0	0,04	0	0	0,96	0	0	0	0	0	0,03	0,01	0,96	0,008
1,75	3994	0,02	0,04	0	0	0,93	0	0,01	0	0	0	0,04	0,96	0	0,048
1,75	1560	0	0,04	0	0	0,95	0	0,01	0	0	0	0,06	0	0,94	0,026
1,75	1183	0	0,04	0	0	0,96	0	0	0	0	0,28	0,04	0	0,68	0,012
1,75	2139	0	0,01	0	0,22	0,76	0	0	0,01	0	0,87	0,11	0,01	0,01	0,038
1,75	1850	0	0,04	0	0	0,96	0	0	0	0	0,02	0,98	0	0	0,026
1,75	3392	0	0,04	0	0	0,96	0	0	0	0	0	1	0	0	0,02
1,75	3048	0	0,04	0	0	0,96	0	0	0	0	0,01	0,03	0	0,96	0,006
1,75	5493	0	0,04	0	0	0,95	0,01	0	0	0	0	0,7	0,3	0	0,03
1,75	11788	0	0,04	0	0	0,95	0	0,01	0	0	0,57	0,43	0	0	0,044
1,75	2405	0	0,04	0	0	0,96	0	0	0	0	0	0,97	0	0,03	0,02
1,75	2745	0	0,04	0	0,01	0,95	0	0	0	0	0	0,03	0,02	0,95	0,014
1,75	2878	0,01	0,04	0	0	0,95	0	0	0	0	0,95	0,05	0	0	0,038

1,75	3036	0	0,04	0	0	0,96	0	0	0	0	0	0,08	0,02	0,9	0,004
1,75	12949	0	0,04	0	0	0,95	0	0,01	0	0	0,02	0,03	0	0,95	0,018
1,75	3448	0	0,04	0	0	0,96	0	0	0	0	0	0,27	0,73	0	0,024
1,75	2729	0	0,04	0	0	0,95	0	0	0,01	0	0	0,92	0	0,08	0,016
1,75	2721	0	0,04	0	0	0,96	0	0	0	0	0,92	0,08	0	0	0,044
1,75	2880	0	0,04	0	0	0,91	0,02	0,01	0,02	0	0,02	0,04	0,79	0,15	0,026
1,75	11330	0,01	0,04	0	0	0,95	0	0	0	0	0,01	0,99	0	0	0,04
1,75	7268	0,02	0,04	0	0	0,93	0	0,01	0	0	0,01	0,04	0,49	0,46	0,036
1,75	2521	0,03	0,03	0	0	0,92	0,01	0	0,01	0	0,01	0,12	0,84	0,03	0,048
1,75	7914	0	0,04	0	0,02	0,84	0	0,01	0,09	0	0,02	0,05	0,03	0,9	0,032
1,75	3516	0	0,04	0	0	0,96	0	0	0	0	0,88	0,03	0,01	0,08	0,046
1,75	5965	0	0,04	0	0	0,96	0	0	0	0	0	0,06	0	0,94	0,006
1,75	3900	0	0,04	0	0	0,92	0,04	0	0	0	0	0,96	0,04	0	0,036
1,75	4950	0	0,04	0	0,01	0,95	0	0	0	0	0	0,04	0,95	0,01	0,026
2	4585	0,01	0,04	0	0	0,95	0	0	0	0	0,87	0,13	0	0	0,034
2	5109	0,02	0,04	0	0	0,93	0	0	0,01	0	0,93	0,05	0	0,02	0,046
2	2174	0	0,04	0	0	0,96	0	0	0	0	0,96	0,04	0	0	0,044
2	2872	0	0,04	0	0	0,91	0	0	0,05	0	0	0,09	0,91	0	0,024
2	19997	0,01	0,04	0,07	0	0	0,19	0	0,69	0	0	0,73	0,26	0,01	0,042
2	16639	0	0,04	0	0	0,96	0	0	0	0	0	0,04	0,96	0	0,028
2	1706	0	0,04	0	0	0,96	0	0	0	0	0	0,04	0,96	0	0,026
2	9884	0	0	0	0,42	0,57	0,01	0	0	0	0,99	0	0	0,01	0,036
2	974	0	0,04	0	0,02	0,9	0,04	0	0	0	0,04	0,96	0	0	0,042
2	7284	0	0,04	0	0	0,96	0	0	0	0	0	1	0	0	0,028
2	4578	0	0,03	0,01	0	0,96	0	0	0	0	0,96	0,04	0	0	0,04
2	3193	0	0,04	0	0,01	0,95	0	0	0	0	0	0,06	0	0,94	0,014
2	6411	0	0	0,03	0,07	0,89	0	0	0,01	0	0,1	0,01	0	0,89	0,048
2	1936	0	0,04	0	0	0,94	0,01	0	0	0,01	0	0,05	0,02	0,93	0,006
2	7504	0,01	0,04	0	0	0,91	0	0	0,04	0	0	0,04	0,91	0,05	0,028
2	14179	0	0,04	0	0	0,96	0	0	0	0	0,95	0,04	0	0,01	0,042
2	1998	0	0,04	0,01	0,06	0,89	0	0	0	0	0,89	0,05	0	0,06	0,028
2	8085	0	0,04	0	0	0,96	0	0	0	0	0	0,03	0,96	0,01	0,022
2	4838	0	0,04	0	0	0,96	0	0	0	0	0	0,04	0	0,96	0,012

2	1959	0	0,04	0	0,01	0,91	0,01	0	0,03	0	0,02	0,93	0,05	0	0,038
2	3528	0	0,04	0	0,07	0,27	0,07	0	0,55	0	0	0,06	0,55	0,39	0,048
2	2543	0,02	0,04	0	0	0,94	0	0	0	0	0	0,16	0,84	0	0,04
2	7873	0	0,04	0	0	0,94	0	0,02	0	0	0,01	0,05	0	0,94	0,03
2	1422	0	0,04	0	0	0,92	0	0,04	0	0	0,91	0,09	0	0	0,042
2	5946	0	0,04	0	0	0,96	0	0	0	0	0,12	0,88	0	0	0,032
2	3274	0	0,04	0	0	0,93	0,03	0	0	0	0,03	0,04	0,93	0	0,024
2	1842	0	0,04	0	0	0,94	0	0,02	0	0	0	0,06	0,94	0	0,036
2	8607	0	0,04	0	0	0,96	0	0	0	0	0	0,04	0	0,96	0
2	2592	0	0,04	0	0	0,96	0	0	0	0	0,01	0,93	0,06	0	0,028
2	3418	0,02	0,04	0	0,04	0,9	0	0	0	0	0,91	0,04	0	0,05	0,042
2	1963	0	0,04	0	0	0,96	0	0	0	0	0	0,04	0,96	0	0,038
2	3529	0,02	0,04	0	0,01	0,89	0,01	0,01	0,01	0,01	0,02	0,95	0,03	0	0,044
2	5575	0	0,04	0	0	0,96	0	0	0	0	0	0,04	0,96	0	0,026
2	12845	0	0,04	0	0	0,96	0	0	0	0	0	0,04	0	0,96	0,008
2	1736	0	0,04	0	0	0,96	0	0	0	0	0	0,04	0,96	0	0,03
2	1529	0,01	0,04	0	0	0,95	0	0	0	0	0,01	0,42	0,56	0,01	0,028
2	4327	0	0,04	0	0	0,92	0,02	0,01	0,01	0	0,01	0	0	0,99	0,02
2	8054	0	0,04	0	0	0,95	0	0,01	0	0	0	0,04	0,95	0,01	0,032
2	8515	0	0,04	0	0	0,96	0	0	0	0	0	0,19	0,8	0,01	0,034
2	11211	0,01	0,04	0	0	0,95	0	0	0	0	0	0,04	0	0,96	0,024
2	8074	0	0,04	0	0	0,96	0	0	0	0	0,97	0,03	0	0	0,034
2	2690	0	0,04	0	0,01	0,94	0,01	0	0	0	0,4	0,04	0,01	0,55	0,024
2	10682	0,01	0,04	0	0	0,94	0	0	0	0,01	0	0,05	0,94	0,01	0,028
2	1839	0	0,04	0	0	0,94	0	0,02	0	0	0,94	0,06	0	0	0,042
2	3733	0	0,04	0	0,01	0,93	0	0	0,02	0	0,01	0,13	0,83	0,03	0,036
2	18428	0	0,04	0	0	0,94	0	0,01	0	0,01	0,01	0,98	0,01	0	0,038
2	2731	0,02	0,04	0	0,04	0,87	0,01	0,01	0	0,01	0,06	0,14	0,8	0	0,048
2	8261	0	0,04	0	0	0,96	0	0	0	0	0	0,18	0,82	0	0,028
2	3754	0	0,04	0	0	0,91	0	0,01	0	0,04	0,01	0,05	0,88	0,06	0,014
2	4067	0	0,04	0	0	0,9	0,04	0,02	0	0	0	0,09	0,01	0,9	0,034
2	1546	0	0,04	0	0,01	0,94	0,01	0	0	0	0,01	0,98	0,01	0	0,03
2	1731	0,01	0,04	0,01	0	0,93	0	0,01	0	0	0,94	0,03	0,01	0,02	0,048

2	4867	0	0,04	0	0	0,96	0	0	0	0	0,33	0,04	0	0,63	0,024
2	11352	0	0,04	0,02	0,03	0,88	0,02	0	0,01	0	0,03	0,04	0,91	0,02	0,036
2	4533	0	0,04	0	0	0,96	0	0	0	0	0	0,04	0,96	0	0,018
2	13457	0	0,04	0,01	0,01	0,93	0	0,01	0	0	0	0,05	0,95	0	0,042
2	1307	0	0,04	0,01	0	0,93	0,02	0	0	0	0	0,97	0,03	0	0,02
2	2931	0	0,04	0	0	0,96	0	0	0	0	0,35	0,04	0,61	0	0,036
2	8664	0	0,04	0	0	0,58	0	0	0,38	0	0,58	0,04	0	0,38	0,028
2	1756	0	0,04	0	0	0,94	0	0	0,01	0,01	0,95	0,05	0	0	0,03
2	2252	0,01	0,04	0	0	0,93	0	0	0,02	0	0,84	0,04	0,11	0,01	0,034
2	1652	0	0,04	0	0,01	0,95	0	0	0	0	0,01	0,03	0,01	0,95	0,018
2	31679	0	0,04	0	0	0,96	0	0	0	0	0,02	0,04	0,94	0	0,014
2	1368	0,01	0,04	0	0	0,95	0	0	0	0	0,12	0,03	0,84	0,01	0,036
2	8460	0	0,04	0	0	0,95	0,01	0	0	0	0,01	0,04	0	0,95	0,006
2	15391	0	0	0	0,13	0,86	0,01	0	0	0	0,11	0,01	0,5	0,38	0,048
2	12393	0	0,04	0,01	0	0,93	0	0	0	0,02	0,01	0,98	0	0,01	0,022
2	2721	0	0,04	0	0	0,96	0	0	0	0	0	0,04	0	0,96	0,01
2	2315	0	0,04	0,01	0	0,94	0	0,01	0	0	0	0,05	0	0,95	0,026
2	2072	0	0,04	0	0	0,96	0	0	0	0	0	0,94	0,06	0	0,026
2	9529	0	0,04	0,01	0	0,94	0	0	0,01	0	0,57	0,04	0	0,39	0,026
2	3196	0,01	0,02	0	0,19	0,78	0	0	0	0	0,78	0,22	0	0	0,048
2	1591	0	0,04	0	0	0,96	0	0	0	0	0	0,04	0,96	0	0,038
2	4114	0	0,04	0	0	0,96	0	0	0	0	0,96	0,04	0	0	0,036
2	11593	0	0,04	0	0	0,96	0	0	0	0	0,94	0,05	0,01	0	0,048
2	26082	0	0,04	0	0,03	0,9	0	0	0,03	0	0,03	0,08	0,89	0	0,032
2	2822	0	0,04	0	0	0,96	0	0	0	0	0	0,05	0,95	0	0,018
2	2794	0	0,04	0	0	0,96	0	0	0	0	0,65	0,04	0	0,31	0,026
2	3190	0	0,04	0	0	0,96	0	0	0	0	0	0,85	0	0,15	0,024
2	2344	0	0,04	0	0	0,96	0	0	0	0	0	0,04	0	0,96	0,01
2	3077	0	0,04	0	0,03	0,92	0	0,01	0	0	0,7	0,26	0,04	0	0,038
2	15644	0	0	0,02	0,07	0,91	0	0	0	0	0,02	0,01	0,09	0,88	0,03
2	6840	0	0,04	0,01	0,02	0,93	0	0	0	0	0	0,04	0,85	0,11	0,048
2	12720	0	0,04	0,01	0,02	0,93	0	0	0	0	0,02	0,04	0,94	0	0,036
2	7402	0	0,04	0	0	0,96	0	0	0	0	0,96	0,01	0	0,03	0,044

2	4641	0,01	0,04	0	0	0,95	0	0	0	0	0	0,03	0,95	0,02	0,024
2	3162	0	0,04	0	0	0,94	0,01	0,01	0	0	0	0,03	0,95	0,02	0,04
2	2911	0	0,04	0	0	0,96	0	0	0	0	0	0,04	0,96	0	0,026
2	2167	0	0,04	0	0	0,96	0	0	0	0	0	0,04	0	0,96	0,006
2	2037	0	0,04	0	0	0,96	0	0	0	0	0	1	0	0	0,024
2	6165	0	0,04	0	0	0,96	0	0	0	0	0	0,04	0,96	0	0,02
2	1533	0,04	0,03	0	0	0,93	0	0	0	0	0,93	0,03	0,04	0	0,046
2	5169	0	0,04	0	0	0,95	0	0,01	0	0	0,4	0,1	0,01	0,49	0,022
2	3987	0,01	0,04	0,01	0	0,93	0	0	0,01	0	0,02	0,06	0,92	0	0,046
2	13736	0	0,04	0,01	0,01	0,87	0,07	0	0	0	0,38	0,03	0,08	0,51	0,028
2	2700	0	0,04	0	0,04	0,92	0	0	0	0	0	0,04	0,87	0,09	0,024
2	1590	0	0,04	0	0	0,96	0	0	0	0	0,96	0,03	0	0,01	0,046
2	6222	0,01	0,04	0	0	0,95	0	0	0	0	0,96	0,03	0,01	0	0,028
2	2338	0	0,04	0	0	0,96	0	0	0	0	0,96	0,04	0	0	0,026
2	3501	0,01	0,04	0	0	0,91	0,02	0	0,02	0	0,92	0,06	0	0,02	0,038
2,25	11160	0	0	0	0,12	0,88	0	0	0	0	0,12	0	0,88	0	0,044
2,25	8471	0	0,04	0	0	0,96	0	0	0	0	0	0,09	0,91	0	0,02
2,25	3396	0	0,04	0	0	0,94	0	0	0,01	0,01	0	0	0,99	0,01	0,026
2,25	5745	0	0,04	0,01	0	0,95	0	0	0	0	0	0,04	0,96	0	0,026
2,25	10851	0	0,04	0,01	0,01	0,94	0	0	0	0	0,86	0,06	0,08	0	0,044
2,25	8450	0	0,04	0,01	0	0,95	0	0	0	0	0	0,04	0,96	0	0,036
2,25	5783	0	0,04	0	0	0,96	0	0	0	0	0,72	0,04	0,18	0,06	0,026
2,25	34709	0,01	0,04	0	0	0,94	0	0	0	0,01	0	0,03	0,03	0,94	0,018
2,25	6299	0,01	0,04	0	0	0,94	0	0,01	0	0	0,01	0,04	0	0,95	0,026
2,25	5069	0,01	0,04	0,01	0	0,91	0	0	0,03	0	0,92	0,04	0,01	0,03	0,046
2,25	1484	0	0,04	0,01	0	0,95	0	0	0	0	0,01	0,04	0,95	0	0,042
2,25	3312	0	0,04	0	0	0,96	0	0	0	0	0	0,04	0	0,96	0
2,25	3169	0	0,04	0	0	0,96	0	0	0	0	0	0,04	0	0,96	0,01
2,25	2519	0,01	0,04	0	0	0,93	0	0	0,02	0	0	0,07	0,93	0	0,04
2,25	5904	0	0	0	0,14	0,47	0,37	0	0	0,02	0,13	0,85	0,02	0	0,046
2,25	7642	0	0,04	0	0	0,95	0	0	0,01	0	0	0,04	0	0,96	0,01
2,25	7283	0	0,04	0	0	0,96	0	0	0	0	0	0,03	0	0,97	0,014
2,25	3667	0	0,04	0	0	0,91	0,05	0	0	0	0	0,04	0,91	0,05	0,022

2,25	10542	0	0,04	0	0	0,96	0	0	0	0	0,03	0,97	0	0	0,024
2,25	28175	0	0,03	0	0,1	0,87	0	0	0	0	0	0,9	0,1	0	0,046
2,25	4067	0	0,04	0	0,02	0,93	0	0	0,01	0	0	0,04	0,95	0,01	0,026
2,25	1897	0	0,04	0	0	0,95	0,01	0	0	0	0	0,05	0,95	0	0,028
2,25	2766	0	0,04	0	0	0,96	0	0	0	0	0	0,98	0,02	0	0,028
2,25	3004	0	0,04	0	0	0,96	0	0	0	0	0,96	0,04	0	0	0,032
2,25	3016	0	0,04	0	0,01	0,95	0	0	0	0	0,03	0,01	0,04	0,92	0,03
2,25	11128	0,01	0,04	0	0	0,95	0	0	0	0	0	0,05	0,91	0,04	0,03
2,25	4298	0	0,04	0	0	0,96	0	0	0	0	0	0,07	0,93	0	0,02
2,25	6186	0,01	0,04	0	0	0,94	0	0	0	0,01	0,95	0,05	0	0	0,038
2,25	7873	0	0,04	0	0	0,95	0	0	0,01	0	0,04	0	0,95	0,01	0,03
2,25	4055	0	0,04	0	0	0,96	0	0	0	0	0	0,04	0,96	0	0,016
2,25	11415	0	0,04	0	0	0,96	0	0	0	0	0,54	0	0	0,46	0,03
2,25	4443	0,04	0,04	0	0	0,92	0	0	0	0	0	0,04	0,95	0,01	0,044
2,25	17327	0,02	0,04	0	0	0,93	0	0	0	0,01	0	1	0	0	0,038
2,25	17517	0,01	0,04	0	0,02	0,93	0	0	0	0	0,01	0,06	0,93	0	0,03
2,25	7728	0	0,04	0	0	0,96	0	0	0	0	0	0,04	0	0,96	0,012
2,25	11053	0	0	0	0,37	0,63	0	0	0	0	0,97	0	0,03	0	0,048
2,25	2598	0	0,04	0	0	0,89	0	0	0,06	0,01	0,89	0,08	0	0,03	0,046
2,25	2419	0	0,04	0	0,02	0,94	0	0	0	0	0	0,06	0,94	0	0,032
2,25	6741	0	0,04	0,01	0	0,95	0	0	0	0	0	0,04	0,65	0,31	0,022
2,25	3469	0	0,04	0	0	0,96	0	0	0	0	0	0,12	0	0,88	0,014
2,25	30982	0	0,04	0	0	0,96	0	0	0	0	0,96	0,04	0	0	0,02
2,25	1936	0	0,04	0	0,02	0,92	0,02	0	0	0	0,03	0,42	0,54	0,01	0,042
2,25	6454	0	0,04	0	0,04	0,9	0	0,02	0	0	0,04	0,94	0	0,02	0,046
2,25	1489	0	0,04	0	0	0,95	0	0	0,01	0	0,9	0,04	0	0,06	0,038
2,25	1692	0	0,04	0,01	0	0,95	0	0	0	0	0	1	0	0	0,036
2,25	1681	0	0,04	0	0	0,96	0	0	0	0	0	0,04	0	0,96	0,012
2,25	8285	0	0,04	0,04	0	0,91	0	0	0	0,01	0	0,08	0	0,92	0,028
2,25	3827	0	0,04	0	0	0,96	0	0	0	0	0,01	0,04	0	0,95	0,01
2,25	5815	0	0,04	0	0,03	0,93	0	0	0	0	0,93	0,07	0	0	0,044
2,25	2016	0	0,04	0	0,04	0,91	0	0	0,01	0	0	0,08	0,91	0,01	0,034
2,25	4965	0	0,04	0	0,03	0,93	0	0	0	0	0,03	0,04	0,58	0,35	0,036

2,25	4241	0	0,04	0	0	0,94	0	0	0,02	0	0,02	0,09	0,88	0,01	0,018
2,25	2054	0	0,04	0	0	0,96	0	0	0	0	0	0,04	0	0,96	0,014
2,25	9930	0	0,04	0	0	0,96	0	0	0	0	0	0,04	0	0,96	0,01
2,25	1229	0	0,04	0	0	0,96	0	0	0	0	0,82	0,18	0	0	0,026
2,25	8726	0	0,04	0	0	0,96	0	0	0	0	0	0,04	0	0,96	0,014
2,25	2966	0	0,04	0	0	0,96	0	0	0	0	0	0,04	0	0,96	0,008
2,25	3458	0	0,04	0	0	0,92	0	0,02	0	0,02	0,92	0,03	0,02	0,03	0,048
2,25	7450	0	0,04	0	0	0,88	0	0	0,08	0	0	0,42	0	0,58	0,008
2,25	1921	0	0,04	0	0	0,92	0	0	0	0,04	0	0,04	0,95	0,01	0,034
2,25	3243	0	0,04	0	0,01	0,94	0	0	0,01	0	0,02	0,04	0	0,94	0,014
2,25	22403	0	0,04	0	0	0,95	0	0	0	0,01	0,02	0,03	0,01	0,94	0,01
2,25	12050	0	0,04	0,01	0	0,94	0,01	0	0	0	0,01	0,98	0	0,01	0,04
2,25	5163	0	0,04	0	0,04	0,91	0	0	0,01	0	0	0,09	0,88	0,03	0,048
2,25	5801	0	0,04	0	0	0,91	0	0,01	0,04	0	0,01	0,06	0,01	0,92	0,014
2,25	1356	0	0,04	0	0	0,96	0	0	0	0	0	0,04	0,96	0	0,024
2,25	5393	0	0,04	0	0,02	0,94	0	0	0	0	0,1	0,04	0,86	0	0,018
2,25	8850	0,01	0,04	0	0	0,95	0	0	0	0	0,01	0,99	0	0	0,036
2,25	6129	0	0,04	0	0	0,94	0	0	0,02	0	0,02	0,04	0,94	0	0,034
2,25	9463	0	0,04	0	0,01	0,95	0	0	0	0	0	0,04	0,01	0,95	0,02
2,25	3099	0	0,04	0,01	0	0,95	0	0	0	0	0,01	0,04	0,95	0	0,034
2,25	21032	0	0,04	0	0	0,95	0,01	0	0	0	0	0,04	0	0,96	0,008
2,25	1827	0	0,04	0	0	0,96	0	0	0	0	0	0,04	0,96	0	0,014
2,25	9859	0	0,04	0	0	0,96	0	0	0	0	0,08	0,04	0,88	0	0,03
2,25	13709	0	0,04	0	0	0,76	0,18	0,01	0,01	0	0,78	0,03	0,18	0,01	0,04
2,25	8991	0	0,04	0	0	0,95	0	0	0,01	0	0	0,04	0,01	0,95	0,004
2,25	4254	0	0,04	0	0	0,95	0	0	0,01	0	0,96	0,04	0	0	0,036
2,25	2348	0	0,04	0	0	0,96	0	0	0	0	0,11	0,03	0,86	0	0,03
2,25	2840	0	0,04	0	0,01	0,94	0	0	0,01	0	0,02	0,03	0,01	0,94	0,028
2,25	9780	0	0,04	0	0	0,96	0	0	0	0	0,01	0,03	0,96	0	0,026
2,25	14678	0	0,04	0,01	0,05	0	0,87	0	0,03	0	0,9	0,02	0,01	0,07	0,04
2,25	6058	0	0,04	0	0	0,96	0	0	0	0	0	0,04	0,96	0	0,034
2,25	3277	0	0,04	0	0	0,94	0	0,02	0	0	0,6	0,04	0,36	0	0,042
2,25	2548	0	0,04	0	0	0,92	0,04	0	0	0	0,92	0,04	0,03	0,01	0,036

2,25	1016	0,01	0,04	0,01	0	0,94	0	0	0	0	0,01	0,94	0,05	0	0,032
2,25	1954	0	0,04	0	0	0,96	0	0	0	0	0	0,05	0	0,95	0,008
2,25	7506	0	0,04	0	0	0,93	0	0	0,02	0,01	0,01	0,06	0	0,93	0,012
2,25	3307	0	0,04	0	0	0,96	0	0	0	0	0,84	0,04	0	0,12	0,036
2,25	4657	0	0,04	0	0	0,96	0	0	0	0	0	0,04	0	0,96	0,01
2,25	4174	0	0,04	0	0	0,96	0	0	0	0	0	1	0	0	0,024
2,25	2710	0	0,04	0,01	0,02	0,92	0	0	0	0,01	0,92	0,03	0,02	0,03	0,048
2,25	14134	0	0,04	0	0	0,95	0	0,01	0	0	0	0,03	0,02	0,95	0,012
2,25	5944	0	0,04	0	0,01	0,92	0,02	0	0	0,01	0	0,04	0,95	0,01	0,026
2,25	11569	0	0,04	0	0	0,95	0	0,01	0	0	0	0,98	0,01	0,01	0,03
2,25	5329	0	0,04	0	0	0,96	0	0	0	0	0	0,04	0	0,96	0,01
2,25	4111	0	0,04	0	0,01	0,95	0	0	0	0	0,95	0,04	0,01	0	0,038
2,25	8608	0	0,02	0	0,1	0,88	0	0	0	0	0,1	0,88	0	0,02	0,046
2,25	5265	0,01	0,04	0	0	0,94	0,01	0	0	0	0,01	0,98	0,01	0	0,042
2,25	17485	0,01	0,04	0,01	0	0,93	0	0,01	0	0	0,02	0,04	0,93	0,01	0,042
2,25	2883	0	0,04	0	0	0,96	0	0	0	0	0,02	0,98	0	0	0,026
2,5	8282	0	0,04	0	0	0,96	0	0	0	0	0	0,04	0	0,96	0,01
2,5	2917	0,01	0,04	0	0	0,93	0	0	0,02	0	0,95	0,04	0	0,01	0,034
2,5	13465	0	0,04	0	0	0,95	0	0	0	0,01	0,01	0,87	0,12	0	0,02
2,5	8240	0	0,04	0	0,01	0,94	0	0,01	0	0	0	0,05	0	0,95	0,026
2,5	9508	0	0,04	0	0	0,96	0	0	0	0	0,01	0,03	0	0,96	0,006
2,5	8125	0	0,04	0	0	0,96	0	0	0	0	0	0	0,04	0,96	0,008
2,5	16403	0	0,04	0	0	0,96	0	0	0	0	0	0,03	0	0,97	0,008
2,5	11274	0	0,01	0	0,17	0,82	0	0	0	0	0,65	0,18	0,17	0	0,048
2,5	3892	0	0,04	0	0	0,81	0,1	0	0,05	0	0	0,09	0,91	0	0,02
2,5	8028	0	0,04	0	0	0,96	0	0	0	0	0	0,04	0,96	0	0,032
2,5	2383	0	0,04	0	0	0,96	0	0	0	0	0,96	0,04	0	0	0,044
2,5	4902	0	0,04	0	0	0,96	0	0	0	0	0	0,04	0,96	0	0,03
2,5	3528	0	0,04	0	0	0,96	0	0	0	0	0,82	0,04	0,14	0	0,044
2,5	9661	0	0,04	0	0	0,95	0,01	0	0	0	0	0,35	0,01	0,64	0,012
2,5	4863	0	0,04	0	0	0,96	0	0	0	0	0,02	0,04	0	0,94	0,006
2,5	4651	0	0,04	0	0	0,93	0	0	0,03	0	0	1	0	0	0,036
2,5	5040	0	0,03	0	0,08	0,88	0	0	0	0,01	0,01	0,9	0,08	0,01	0,046

2,5	28909	0	0,04	0	0	0,95	0,01	0	0	0	0,01	0,35	0,64	0	0,026
2,5	2336	0	0,04	0	0	0,94	0	0	0,02	0	0,02	0,04	0,94	0	0,036
2,5	3568	0	0,04	0	0	0,96	0	0	0	0	0	0,04	0,96	0	0,034
2,5	3642	0	0,04	0	0	0,96	0	0	0	0	0	0,04	0	0,96	0,016
2,5	3924	0	0,04	0	0	0,96	0	0	0	0	0	0,04	0	0,96	0,01
2,5	26833	0	0,04	0	0,11	0,85	0	0	0	0	0,85	0,04	0,06	0,05	0,046
2,5	12205	0	0,04	0	0	0,93	0	0,03	0	0	0,93	0,04	0	0,03	0,048
2,5	1597	0	0,04	0	0	0,96	0	0	0	0	0	0,04	0,96	0	0,02
2,5	8440	0	0,04	0	0,01	0,95	0	0	0	0	0	0,04	0,96	0	0,024
2,5	1505	0	0,04	0	0	0,96	0	0	0	0	0	0,99	0,01	0	0,024
2,5	5102	0	0,04	0	0	0,95	0	0	0	0,01	0	0,07	0,93	0	0,018
2,5	6797	0	0,04	0	0	0,96	0	0	0	0	0,06	0,94	0	0	0,034
2,5	3126	0	0,04	0	0,01	0,95	0	0	0	0	0,01	0,04	0,95	0	0,03
2,5	3072	0	0,04	0	0	0,95	0	0	0	0,01	0	0,99	0,01	0	0,03
2,5	17781	0	0,04	0,01	0	0,95	0	0	0	0	0	0,04	0,95	0,01	0,026
2,5	2373	0	0,04	0	0	0,96	0	0	0	0	0	0,04	0	0,96	0,006
2,5	19825	0	0,04	0	0	0,95	0	0	0	0,01	0,01	0,04	0	0,95	0,008
2,5	5341	0	0,04	0	0	0,94	0,02	0	0	0	0	0,98	0	0,02	0,024
2,5	4477	0	0,04	0	0	0,94	0	0	0,02	0	0,94	0,06	0	0	0,044
2,5	7144	0	0	0	0,15	0,85	0	0	0	0	0,14	0,85	0	0,01	0,044
2,5	7605	0	0,04	0	0	0,96	0	0	0	0	0,93	0,04	0,03	0	0,04
2,5	39334	0	0,04	0	0	0,93	0,02	0,01	0	0	0	0,98	0,01	0,01	0,032
2,5	5106	0	0,04	0	0	0,94	0,02	0	0	0	0,02	0,06	0	0,92	0,012
2,5	7333	0	0,04	0	0,02	0,93	0,01	0	0	0	0,01	0,13	0,84	0,02	0,042
2,5	6447	0,01	0,04	0	0	0,95	0	0	0	0	0	0,04	0	0,96	0,016
2,5	18530	0	0,04	0	0	0,96	0	0	0	0	0	0,04	0,13	0,83	0,012
2,5	3885	0	0,04	0	0	0,92	0,01	0,01	0	0,02	0,46	0,04	0	0,5	0,014
2,5	22291	0	0	0,03	0,12	0,16	0,69	0	0	0	0,03	0	0,97	0	0,04
2,5	6415	0	0	0	0,37	0,62	0	0	0,01	0	1	0	0	0	0,048
2,5	12368	0	0,04	0	0	0,96	0	0	0	0	0	0,01	0	0,99	0,014
2,5	1691	0	0,04	0	0	0,94	0,02	0	0	0	0,01	0,95	0	0,04	0,02
2,5	24428	0	0,04	0	0	0,96	0	0	0	0	0	1	0	0	0,022
2,5	8629	0	0,04	0	0	0,96	0	0	0	0	0	0,04	0	0,96	0,006

2,5	4545	0	0,04	0	0	0,94	0	0	0,02	0	0,33	0,06	0	0,61	0,01
2,5	33481	0	0,04	0	0	0,96	0	0	0	0	0	0,04	0,96	0	0,016
2,5	1443	0	0,02	0	0,11	0,83	0,04	0	0	0	0,4	0,02	0,58	0	0,048
2,5	4194	0	0,04	0,01	0	0,95	0	0	0	0	0	0,04	0	0,96	0,008
2,5	17125	0	0	0	0,13	0,85	0	0	0	0,02	0,1	0,89	0,01	0	0,048
2,5	3100	0	0,04	0	0	0,95	0	0,01	0	0	0,01	0,86	0	0,13	0,012
2,5	1876	0	0,04	0	0	0,96	0	0	0	0	0	0,04	0,96	0	0,024
2,5	7525	0	0,04	0	0,07	0,89	0	0	0	0	0,89	0,04	0,07	0	0,044
2,5	6920	0	0,04	0,01	0,07	0,88	0	0	0	0	0	0,04	0,95	0,01	0,036
2,5	4947	0	0,04	0	0	0,96	0	0	0	0	0	0,04	0	0,96	0,006
2,5	19454	0,01	0,04	0	0	0,94	0	0	0,01	0	0	0,04	0,02	0,94	0,02
2,5	5733	0	0,03	0	0,15	0,82	0	0	0	0	0,82	0,03	0,15	0	0,048
2,5	7337	0	0,04	0	0	0,96	0	0	0	0	0,22	0,45	0	0,33	0,03
2,5	20491	0	0,04	0	0	0,96	0	0	0	0	0	0,04	0,96	0	0,02
2,5	2157	0	0,04	0	0	0,95	0	0	0,01	0	0,96	0,04	0	0	0,036
2,5	1634	0	0,04	0	0	0,95	0,01	0	0	0	0,01	0,18	0	0,81	0,004
2,5	1312	0	0,04	0	0,01	0,94	0	0	0	0,01	0,01	0,05	0	0,94	0,02
2,5	14001	0,01	0,04	0	0	0,95	0	0	0	0	0	0,05	0,95	0	0,038
2,5	5794	0	0,03	0,01	0,08	0,88	0	0	0	0	0	0,01	0,98	0,01	0,048
2,5	18056	0	0,04	0	0,03	0,91	0	0	0	0,02	0,71	0,26	0,03	0	0,046
2,5	4168	0	0,04	0	0	0,95	0	0,01	0	0	0	0,05	0	0,95	0,016
2,5	5499	0	0,04	0	0	0,96	0	0	0	0	0	0,04	0,96	0	0,042
2,5	2252	0	0,04	0	0	0,96	0	0	0	0	0	0,04	0	0,96	0,012
2,5	7143	0	0,04	0	0	0,96	0	0	0	0	0,01	0,03	0,96	0	0,03
2,5	3285	0	0,04	0	0	0,92	0,04	0	0	0	0,04	0,96	0	0	0,042
2,5	1817	0	0,04	0	0	0,96	0	0	0	0	0	0,04	0,13	0,83	0,012
2,5	11543	0	0,04	0	0	0,96	0	0	0	0	0	0,04	0	0,96	0,004
2,5	23940	0	0,04	0	0	0,96	0	0	0	0	0	0,04	0,96	0	0,024
2,5	12112	0	0,04	0	0,01	0,93	0,01	0,01	0	0	0,04	0,02	0,01	0,93	0,022
2,5	3602	0	0,04	0	0,02	0,94	0	0	0	0	0	0,05	0,95	0	0,032
2,5	2291	0	0,04	0	0	0,96	0	0	0	0	0	0,04	0	0,96	0,006
2,5	1827	0	0,04	0	0	0,88	0,07	0,01	0	0	0	0,11	0,35	0,54	0,024
2,5	31753	0	0,04	0	0	0,95	0	0	0,01	0	0,01	0,04	0,95	0	0,022

2,5	10964	0	0,04	0	0,02	0,94	0	0	0	0	0,96	0,04	0	0	0,048
2,5	4490	0,03	0,03	0,06	0	0	0	0	0,88	0	0,02	0,05	0,89	0,04	0,048
2,5	4062	0	0,04	0,01	0,01	0,94	0	0	0	0	0,01	0,05	0	0,94	0,02
2,5	6981	0	0,04	0,01	0	0,94	0	0	0,01	0	0,02	0,97	0	0,01	0,032
2,5	2232	0,01	0,04	0	0	0,95	0	0	0	0	0	0,04	0,95	0,01	0,034
2,5	1585	0	0,04	0	0	0,96	0	0	0	0	0	0,05	0,95	0	0,032
2,5	2673	0,01	0,04	0	0	0,92	0,03	0	0	0	0,96	0,02	0	0,02	0,036
2,5	31233	0	0,04	0	0,04	0,88	0,03	0,01	0	0	0,92	0,08	0	0	0,046
2,5	21698	0	0,04	0	0	0,95	0	0	0	0,01	0,95	0,05	0	0	0,024
2,5	2536	0	0,04	0	0	0,89	0,03	0	0,04	0	0,92	0,02	0	0,06	0,034
2,5	7082	0	0,04	0	0	0,96	0	0	0	0	0	0,04	0	0,96	0,01
2,5	6415	0	0,04	0	0	0,96	0	0	0	0	0	0,04	0,49	0,47	0,014
2,5	5416	0	0,04	0	0	0,96	0	0	0	0	0,96	0,04	0	0	0,04
2,5	2701	0,01	0,04	0	0,01	0,94	0	0	0	0	0,04	0,95	0,01	0	0,048
2,5	1707	0	0,04	0	0,03	0,9	0,01	0	0	0,02	0,92	0,04	0,03	0,01	0,044
2,5	20758	0	0,04	0	0	0,94	0	0	0,02	0	0	0,98	0,01	0,01	0,026
2,5	8257	0,01	0,04	0	0	0,93	0	0	0,01	0,01	0	0,04	0,96	0	0,026
2,75	12960	0	0,04	0	0	0,96	0	0	0	0	0	0,15	0	0,85	0,012
2,75	14888	0,01	0,04	0	0	0,95	0	0	0	0	0,9	0,05	0	0,05	0,042
2,75	2125	0	0,04	0	0	0,96	0	0	0	0	0,92	0,07	0	0,01	0,042
2,75	5677	0	0,04	0	0	0,95	0	0	0	0,01	0,01	0,5	0	0,49	0,016
2,75	1852	0	0,04	0	0	0,96	0	0	0	0	0	0,04	0,96	0	0,028
2,75	3798	0	0,04	0,01	0	0,95	0	0	0	0	0	0,06	0,94	0	0,028
2,75	2223	0	0,04	0	0,01	0,95	0	0	0	0	0,02	0,02	0,43	0,53	0,044
2,75	12098	0	0,04	0,01	0	0,95	0	0	0	0	0	0,05	0	0,95	0,018
2,75	17440	0	0,04	0	0	0,96	0	0	0	0	0,62	0,04	0	0,34	0,036
2,75	2205	0	0,04	0	0	0,91	0	0	0,05	0	0,03	0,02	0,93	0,02	0,024
2,75	8076	0	0,04	0	0	0,96	0	0	0	0	0	0,04	0,96	0	0,032
2,75	27365	0	0,04	0	0	0,94	0,02	0	0	0	0	0,04	0,95	0,01	0,024
2,75	21638	0	0,04	0	0,02	0,93	0,01	0	0	0	0	0,96	0	0,04	0,042
2,75	10908	0	0,04	0	0	0,95	0,01	0	0	0	0,95	0,02	0	0,03	0,036
2,75	26573	0	0,04	0	0	0,95	0	0,01	0	0	0	0,91	0,05	0,04	0,026
2,75	3481	0	0,04	0,01	0	0,95	0	0	0	0	0	0,05	0,95	0	0,03

2,75	8266	0	0,04	0	0	0,95	0	0	0	0,01	0	0,99	0	0,01	0,022
2,75	8375	0	0,04	0	0	0,93	0	0	0,03	0	0	0,04	0,03	0,93	0,014
2,75	12725	0	0,04	0	0	0,96	0	0	0	0	0	0,04	0,91	0,05	0,034
2,75	3286	0	0,04	0	0	0,86	0,1	0	0	0	0,12	0,02	0,86	0	0,022
2,75	4688	0	0,04	0	0	0,96	0	0	0	0	0,08	0,04	0	0,88	0,008
2,75	1889	0	0,04	0	0	0,94	0	0	0,02	0	0,02	0,04	0	0,94	0,002
2,75	26950	0	0,04	0,01	0	0,93	0,01	0	0,01	0	0,01	0,04	0,94	0,01	0,026
2,75	2048	0	0,04	0	0	0,93	0	0,02	0,01	0	0,94	0,04	0	0,02	0,046
2,75	6870	0	0,04	0	0,03	0,87	0,06	0	0	0	0,9	0,1	0	0	0,034
2,75	4649	0	0,04	0	0	0,92	0,04	0	0	0	0,04	0,72	0,24	0	0,024
2,75	12185	0	0,04	0	0	0,96	0	0	0	0	0	0,04	0,96	0	0,024
2,75	9641	0,01	0,04	0	0	0,95	0	0	0	0	0,94	0,04	0,02	0	0,038
2,75	19625	0	0	0,04	0,05	0,57	0	0,01	0,33	0	0,32	0,59	0,07	0,02	0,048
2,75	12312	0	0,04	0	0	0,96	0	0	0	0	0	0,04	0	0,96	0,01
2,75	8180	0	0,04	0	0	0,96	0	0	0	0	0	0,04	0	0,96	0,01
2,75	5548	0	0,04	0	0	0,96	0	0	0	0	0,96	0,04	0	0	0,046
2,75	8374	0	0,04	0	0	0,93	0	0	0,02	0,01	0,04	0,07	0,89	0	0,026
2,75	9390	0	0,04	0	0	0,96	0	0	0	0	0	0,98	0	0,02	0,03
2,75	3129	0,01	0,04	0	0	0,95	0	0	0	0	0,93	0,07	0	0	0,048
2,75	34335	0	0,04	0	0	0,96	0	0	0	0	0,96	0,04	0	0	0,04
2,75	4564	0	0,04	0	0	0,93	0	0	0,03	0	0	0,04	0,03	0,93	0,012
2,75	39438	0	0,04	0	0	0,96	0	0	0	0	0,35	0,63	0	0,02	0,026
2,75	3349	0	0,04	0	0	0,96	0	0	0	0	0	0,98	0	0,02	0,028
2,75	18193	0	0,04	0	0	0,94	0	0	0,02	0	0	0,04	0,96	0	0,024
2,75	4710	0	0,04	0	0	0,96	0	0	0	0	0	0,01	0,99	0	0,038
2,75	1540	0	0,04	0	0	0,96	0	0	0	0	0	0,14	0	0,86	0,008
2,75	8611	0	0,04	0	0	0,95	0	0	0,01	0	0,96	0,04	0	0	0,038
2,75	12046	0,01	0,04	0	0	0,95	0	0	0	0	0	0,04	0,01	0,95	0,03
2,75	4750	0	0,04	0	0,02	0,94	0	0	0	0	0	0,04	0,96	0	0,038
2,75	3246	0	0,04	0	0	0,96	0	0	0	0	0,07	0,93	0	0	0,018
2,75	2297	0	0,04	0	0	0,96	0	0	0	0	0	0,04	0,96	0	0,022
2,75	28774	0	0,04	0,01	0	0,95	0	0	0	0	0	0,04	0	0,96	0,026
2,75	16115	0	0,01	0,01	0,14	0,31	0,07	0	0,43	0,03	0,5	0,15	0,34	0,01	0,042

2,75	13442	0	0,04	0	0	0,95	0	0,01	0	0	0,04	0,01	0	0,95	0,012
2,75	2406	0	0,04	0,01	0	0,95	0	0	0	0	0	0,04	0,96	0	0,014
2,75	6510	0	0,04	0	0	0,95	0	0,01	0	0	0,02	0,96	0,01	0,01	0,026
2,75	7812	0	0,04	0	0	0,96	0	0	0	0	0,08	0,45	0	0,47	0,01
2,75	6663	0	0,04	0	0	0,96	0	0	0	0	0	0,04	0,07	0,89	0,006
2,75	12507	0	0,04	0,01	0,03	0,92	0	0	0	0	0,95	0,05	0	0	0,042
2,75	2911	0	0,04	0	0	0,96	0	0	0	0	0	0,04	0,96	0	0,018
2,75	12294	0	0,04	0	0,07	0,78	0,1	0	0	0,01	0,79	0,14	0,06	0,01	0,042
2,75	1503	0	0,04	0	0	0,96	0	0	0	0	0	0,05	0,95	0	0,024
2,75	13214	0	0,04	0	0	0,96	0	0	0	0	0,14	0,04	0	0,82	0,006
2,75	1649	0	0,04	0	0	0,96	0	0	0	0	0	0,04	0,12	0,84	0,012
2,75	2407	0	0,04	0	0	0,95	0	0	0	0,01	0	0,04	0,96	0	0,026
2,75	1412	0	0,04	0	0	0,96	0	0	0	0	0	0,04	0,96	0	0,022
2,75	1586	0	0,04	0	0	0,96	0	0	0	0	0,48	0,04	0,48	0	0,024
2,75	3579	0	0,04	0	0	0,96	0	0	0	0	0	0,04	0,59	0,37	0,026
2,75	1611	0	0,04	0	0	0,94	0	0,02	0	0	0	0,02	0,95	0,03	0,02
2,75	4745	0	0,04	0	0	0,96	0	0	0	0	0	1	0	0	0,044
2,75	1813	0	0,04	0	0,02	0,94	0	0	0	0	0	0,04	0,94	0,02	0,036
2,75	2863	0	0,04	0	0	0,96	0	0	0	0	0	0,03	0,01	0,96	0,006
2,75	5958	0	0,04	0	0	0,96	0	0	0	0	0,96	0,04	0	0	0,04
2,75	12565	0	0,04	0	0	0,96	0	0	0	0	0	0,04	0,96	0	0,028
2,75	3653	0,01	0,04	0	0,09	0,86	0	0	0	0	0,96	0,04	0	0	0,046
2,75	6762	0	0,04	0	0	0,96	0	0	0	0	0,96	0,04	0	0	0,042
2,75	13180	0	0,04	0	0	0,95	0,01	0	0	0	0,01	0,04	0,01	0,94	0,008
2,75	36037	0	0,04	0	0	0,96	0	0	0	0	0	1	0	0	0,036
2,75	8945	0	0,04	0	0,01	0,95	0	0	0	0	0,95	0,02	0,01	0,02	0,034
2,75	4817	0,01	0,04	0	0	0,95	0	0	0	0	0	0,02	0,96	0,02	0,028
2,75	4164	0	0,04	0	0	0,95	0	0	0,01	0	0	0,01	0,04	0,95	0,01
2,75	11440	0	0,04	0	0	0,95	0	0	0,01	0	0,02	0,04	0,61	0,33	0,024
2,75	1318	0	0,04	0	0	0,95	0	0	0	0,01	0	0,04	0,96	0	0,022
2,75	5049	0	0,04	0	0	0,94	0	0	0,02	0	0	0,04	0,02	0,94	0,008
2,75	4995	0	0,04	0	0,01	0,95	0	0	0	0	0	0,04	0,01	0,95	0,02
2,75	1357	0	0,04	0	0	0,96	0	0	0	0	0,7	0,04	0,26	0	0,028

2,75	7462	0	0,04	0	0	0,95	0,01	0	0	0	0,9	0,05	0,05	0	0,046
2,75	4015	0,01	0,04	0	0	0,94	0	0,01	0	0	0	0,05	0,95	0	0,044
2,75	6527	0,01	0,04	0	0	0,95	0	0	0	0	0,35	0,04	0,61	0	0,042
2,75	11603	0	0,04	0	0	0,96	0	0	0	0	0	0,04	0	0,96	0,01
2,75	2553	0	0,04	0	0	0,95	0	0	0,01	0	0,95	0,04	0,01	0	0,038
2,75	12435	0	0,04	0,01	0,01	0,94	0	0	0	0	0,94	0,04	0,01	0,01	0,04
2,75	4003	0	0,04	0	0	0,96	0	0	0	0	0,96	0,04	0	0	0,036
2,75	10537	0	0,04	0	0,01	0,92	0,01	0	0,02	0	0	0,06	0,92	0,02	0,026
2,75	5360	0	0,04	0	0	0,95	0	0	0,01	0	0	0,99	0,01	0	0,02
2,75	10216	0	0,04	0	0	0,95	0	0	0,01	0	0,01	0,04	0,95	0	0,03
2,75	1895	0	0,04	0,01	0	0,95	0	0	0	0	0,01	0,05	0,94	0	0,024
2,75	13478	0	0,03	0	0,06	0,91	0	0	0	0	0,83	0,17	0	0	0,028
2,75	51513	0	0,04	0	0	0,96	0	0	0	0	0,01	0	0,96	0,03	0,018
2,75	8610	0	0,04	0	0,01	0,93	0,02	0	0	0	0,94	0,04	0,02	0	0,038
2,75	1363	0	0,04	0	0	0,94	0	0	0,02	0	0,02	0,04	0	0,94	0,008
2,75	6604	0	0,04	0	0	0,96	0	0	0	0	0	0,04	0,96	0	0,016
2,75	1486	0	0,04	0	0,05	0,9	0	0	0	0,01	0,05	0,04	0,9	0,01	0,034
2,75	10127	0	0,04	0,01	0	0,88	0	0	0,06	0,01	0,01	0,06	0,08	0,85	0,018
3	5914	0	0,04	0,01	0	0,92	0,03	0	0	0	0	0,99	0	0,01	0,024
3	6680	0	0,04	0	0	0,95	0	0	0	0,01	0	0,04	0,96	0	0,026
3	6747	0	0,04	0	0,01	0,95	0	0	0	0	0,01	0,04	0,95	0	0,024
3	6726	0	0,04	0	0	0,96	0	0	0	0	0,03	0,06	0,83	0,08	0,028
3	16970	0	0,04	0	0	0,96	0	0	0	0	0	0,04	0	0,96	0,012
3	5373	0	0,04	0	0	0,96	0	0	0	0	0	0,04	0,96	0	0,026
3	4400	0	0,04	0	0	0,96	0	0	0	0	0	0,04	0,96	0	0,026
3	3599	0,01	0,04	0	0,02	0,93	0	0	0	0	0	0,04	0,96	0	0,04
3	21100	0	0,04	0	0	0,96	0	0	0	0	0	0,04	0	0,96	0,014
3	2717	0	0,04	0	0	0,95	0	0	0	0,01	0	0,04	0,95	0,01	0,024
3	13719	0	0	0	0,08	0,92	0	0	0	0	0,08	0	0	0,92	0,046
3	20446	0	0,04	0	0,01	0,95	0	0	0	0	0,5	0,5	0	0	0,016
3	25926	0	0,04	0	0	0,9	0	0	0,06	0	0	0,1	0	0,9	0,004
3	5355	0	0,04	0	0	0,96	0	0	0	0	0,47	0,01	0,52	0	0,024
3	10464	0	0,04	0	0,03	0,91	0	0,02	0	0	0,91	0,07	0	0,02	0,048

3	11378	0	0,04	0,01	0	0,93	0,01	0	0,01	0	0,02	0,02	0,01	0,95	0,018
3	8083	0	0,04	0	0,01	0,94	0	0	0	0,01	0	0,04	0	0,96	0,024
3	14137	0	0,04	0	0	0,72	0	0	0,24	0	0	0,04	0	0,96	0,012
3	9389	0	0,04	0	0	0,95	0	0	0	0,01	0	0,05	0	0,95	0,014
3	3093	0	0,04	0	0	0,96	0	0	0	0	0	0,04	0	0,96	0,014
3	2496	0	0,04	0	0,01	0,95	0	0	0	0	0,01	0,04	0	0,95	0,022
3	11890	0	0,04	0	0	0,96	0	0	0	0	0	0,96	0,04	0	0,034
3	6825	0,01	0,04	0	0	0,94	0	0,01	0	0	0,01	0,04	0,94	0,01	0,04
3	4097	0	0,04	0	0	0,94	0,01	0	0,01	0	0,16	0,26	0,58	0	0,032
3	14821	0	0,04	0	0	0,91	0,01	0	0,01	0,03	0,03	0,04	0,93	0	0,022
3	3223	0	0,04	0	0	0,9	0,02	0	0,04	0	0	0,95	0,03	0,02	0,028
3	22459	0	0,04	0	0,01	0,95	0	0	0	0	0,56	0,05	0,39	0	0,044
3	14852	0,01	0,04	0	0	0,95	0	0	0	0	0,17	0,04	0,79	0	0,038
3	4134	0	0,04	0	0	0,95	0	0	0	0,01	0	0,04	0	0,96	0,006
3	22520	0	0,04	0	0	0,96	0	0	0	0	0	0,03	0,97	0	0,024
3	2235	0	0,04	0	0	0,96	0	0	0	0	0	0,04	0	0,96	0,006
3	5815	0	0,04	0	0	0,95	0,01	0	0	0	0,01	0,04	0	0,95	0,012
3	7485	0,01	0,04	0,02	0,02	0,91	0	0	0	0	0,01	0,04	0,91	0,04	0,048
3	1136	0,01	0,04	0	0	0,93	0,01	0	0	0,01	0	0,98	0,02	0	0,034
3	5594	0	0,04	0	0	0,93	0,03	0	0	0	0	0,04	0	0,96	0,012
3	6414	0,01	0,04	0	0	0,95	0	0	0	0	0	0,96	0,01	0,03	0,036
3	3810	0	0,04	0	0	0,96	0	0	0	0	0,96	0,04	0	0	0,02
3	11713	0	0	0	0,14	0,79	0,07	0	0	0	0,12	0,06	0,8	0,02	0,032
3	6269	0	0,03	0	0,12	0,76	0,08	0,01	0	0	0,73	0,14	0,09	0,04	0,048
3	22348	0	0,04	0	0	0,95	0,01	0	0	0	0,01	0,04	0,94	0,01	0,036
3	9007	0	0,04	0	0	0,96	0	0	0	0	0,01	0,3	0,06	0,63	0,01
3	10356	0	0,04	0	0,01	0,94	0	0	0	0,01	0,04	0,04	0,9	0,02	0,032
3	8242	0	0,04	0	0	0,96	0	0	0	0	0	0,04	0	0,96	0,01
3	3670	0	0,04	0	0	0,94	0	0,02	0	0	0,94	0,04	0,02	0	0,044
3	2579	0	0,04	0,01	0	0,94	0	0	0	0,01	0,01	0,09	0,01	0,89	0,012
3	6404	0	0,04	0	0	0,96	0	0	0	0	0	0,03	0,97	0	0,018
3	12715	0	0,04	0	0	0,89	0,07	0	0	0	0	1	0	0	0,024
3	8564	0	0,04	0,01	0	0,95	0	0	0	0	0,94	0,05	0,01	0	0,044

3	5613	0,01	0,04	0	0	0,95	0	0	0	0	0,01	0,04	0	0,95	0,018
3	25089	0	0,04	0	0	0,95	0	0,01	0	0	0,01	0,04	0	0,95	0,016
3	3281	0,01	0,02	0	0,07	0,9	0	0	0	0	0,07	0,56	0,37	0	0,034
3	23210	0	0,04	0	0	0,91	0	0	0,05	0	0	0,09	0	0,91	0,008
3	3251	0	0,04	0	0	0,96	0	0	0	0	0	0,04	0,96	0	0,016
3	15266	0	0,04	0	0	0,94	0,01	0	0	0,01	0	1	0	0	0,024
3	4790	0	0,04	0	0	0,96	0	0	0	0	0,95	0,05	0	0	0,028
3	25623	0	0,04	0	0,01	0,95	0	0	0	0	0	0,98	0,01	0,01	0,034
3	26592	0	0,04	0,01	0	0,94	0	0	0,01	0	0	0,57	0,41	0,02	0,028
3	5686	0	0,04	0,01	0	0,95	0	0	0	0	0	0,05	0,95	0	0,032
3	21042	0	0,04	0	0	0,96	0	0	0	0	0	0,04	0,24	0,72	0,006
3	13108	0,01	0,04	0	0	0,95	0	0	0	0	0	0,07	0,93	0	0,026
3	6827	0	0,04	0	0	0,96	0	0	0	0	0,01	0,03	0	0,96	0,004
3	2378	0	0,04	0	0	0,93	0,03	0	0	0	0,03	0,04	0,93	0	0,034
3	3055	0	0,04	0	0	0,95	0,01	0	0	0	0,01	0,04	0	0,95	0,012
3	2318	0	0,04	0	0	0,95	0	0	0	0,01	0	0,04	0,95	0,01	0,018
3	4765	0	0	0,01	0,33	0,66	0	0	0	0	0,99	0,01	0	0	0,04
3	2695	0	0,04	0	0	0,96	0	0	0	0	0	0,04	0	0,96	0,002
3	9673	0	0,04	0	0	0,96	0	0	0	0	0	0,02	0,51	0,47	0,014
3	9751	0	0,04	0	0,16	0,79	0	0	0	0,01	0,81	0,17	0,01	0,01	0,044
3	2826	0	0,04	0,01	0	0,92	0	0,02	0	0,01	0,01	0,04	0,94	0,01	0,044
3	5040	0	0,04	0	0	0,96	0	0	0	0	0,03	0,04	0	0,93	0,012
3	2429	0	0,04	0	0,01	0,95	0	0	0	0	0,18	0,04	0,77	0,01	0,032
3	6796	0	0,04	0	0	0,96	0	0	0	0	0	0,03	0,96	0,01	0,014
3	4111	0	0,04	0	0	0,96	0	0	0	0	0	0,04	0,15	0,81	0,024
3	16416	0	0,04	0	0,06	0,9	0	0	0	0	0,14	0,09	0,76	0,01	0,046
3	8679	0,01	0,04	0	0	0,93	0	0,02	0	0	0,02	0,04	0,93	0,01	0,038
3	9069	0	0,04	0	0	0,96	0	0	0	0	0	1	0	0	0,026
3	10148	0	0,04	0	0,01	0,95	0	0	0	0	0,01	0,03	0	0,96	0,024
3	19467	0	0,04	0	0,07	0,89	0	0	0	0	0,1	0,9	0	0	0,048
3	25421	0	0,04	0	0,01	0,93	0	0	0,02	0	0	0,06	0,01	0,93	0,012
3	1491	0	0,04	0	0	0,94	0,02	0	0	0	0	0,06	0,94	0	0,03
3	29785	0	0,04	0	0	0,84	0	0,1	0,02	0	0,94	0,04	0,02	0	0,042

3	7619	0	0,04	0	0	0,95	0	0,01	0	0	0	1	0	0	0,022
3	3466	0	0,04	0	0,02	0,91	0,03	0	0	0	0,32	0,04	0,03	0,61	0,04
3	2478	0	0,04	0	0	0,96	0	0	0	0	0	0,03	0,97	0	0,026
3	14690	0	0,04	0	0,01	0,95	0	0	0	0	0,68	0,04	0	0,28	0,026
3	26827	0	0,04	0	0,02	0,94	0	0	0	0	0	0,04	0,94	0,02	0,02
3	6699	0,01	0,04	0	0	0,95	0	0	0	0	0,01	0,04	0,95	0	0,036
3	11000	0	0,04	0	0	0,96	0	0	0	0	0	0,04	0	0,96	0,008
3	4337	0,01	0,04	0	0	0,95	0	0	0	0	0,01	0,03	0,94	0,02	0,028
3	5358	0	0,04	0	0	0,96	0	0	0	0	0	0,11	0	0,89	0,006
3	2457	0	0,04	0	0,02	0,94	0	0	0	0	0	0,04	0	0,96	0,018
3	5264	0	0,04	0	0	0,92	0	0	0,04	0	0	0,04	0,96	0	0,026
3	15131	0	0,04	0	0	0,95	0	0,01	0	0	0,01	0,04	0,95	0	0,026
3	2289	0	0,04	0	0	0,96	0	0	0	0	0,07	0,93	0	0	0,012
3	34126	0	0,04	0	0,01	0,95	0	0	0	0	0	0,05	0,95	0	0,018
3	26651	0	0,04	0	0	0,95	0	0	0	0,01	0	0	1	0	0,03
3	7124	0	0,04	0	0	0,96	0	0	0	0	0	0,08	0	0,92	0,01
3	17959	0	0,04	0	0	0,92	0	0,04	0	0	0,03	0,05	0,92	0	0,034
3	10427	0	0,04	0	0	0,96	0	0	0	0	0	0,04	0,96	0	0,03
3	15685	0	0,04	0	0	0,95	0	0	0,01	0	0,01	0,99	0	0	0,024
3,25	3513	0	0,04	0	0,04	0,92	0	0	0	0	0	0,04	0,96	0	0,046
3,25	5018	0	0,04	0	0	0,96	0	0	0	0	0	0,04	0,96	0	0,026
3,25	22232	0	0,04	0,01	0	0,95	0	0	0	0	0,03	0,02	0,95	0	0,032
3,25	2731	0	0,04	0	0	0,96	0	0	0	0	0	0,04	0	0,96	0,01
3,25	14204	0	0,04	0	0	0,94	0	0,02	0	0	0	0,78	0	0,22	0,026
3,25	3343	0	0,04	0	0	0,96	0	0	0	0	0,15	0,85	0	0	0,022
3,25	15856	0	0,04	0	0	0,96	0	0	0	0	0,01	0,44	0	0,55	0,018
3,25	4883	0	0,04	0	0	0,83	0,01	0	0,12	0	0,13	0,77	0,1	0	0,024
3,25	19823	0	0,04	0	0	0,95	0	0	0	0,01	0	0,04	0,96	0	0,022
3,25	26189	0	0,04	0	0	0,96	0	0	0	0	0,96	0,04	0	0	0,048
3,25	3043	0	0,04	0,01	0,05	0,89	0	0,01	0	0	0,05	0,94	0	0,01	0,048
3,25	1684	0	0,04	0	0	0,92	0	0	0,04	0	0	1	0	0	0,024
3,25	39072	0	0,04	0	0	0,96	0	0	0	0	0	0,13	0,87	0	0,02
3,25	7378	0,01	0,04	0,02	0	0,93	0	0	0	0	0,19	0,06	0,49	0,26	0,028

3,25	12697	0	0,04	0,01	0	0,95	0	0	0	0	0,95	0,05	0	0	0,048
3,25	3181	0	0,04	0	0	0,95	0	0,01	0	0	0,06	0,03	0,02	0,89	0,016
3,25	16001	0	0,04	0	0	0,96	0	0	0	0	0	0,08	0	0,92	0,006
3,25	6755	0	0,04	0	0	0,96	0	0	0	0	0	0,04	0,96	0	0,026
3,25	9779	0	0,04	0	0	0,96	0	0	0	0	0,32	0,68	0	0	0,03
3,25	3254	0	0,04	0	0,01	0,95	0	0	0	0	0,01	0,04	0,95	0	0,036
3,25	2268	0	0,04	0	0	0,96	0	0	0	0	0	0,12	0	0,88	0,014
3,25	4592	0	0,04	0	0	0,96	0	0	0	0	0,96	0,04	0	0	0,03
3,25	19545	0	0,04	0	0,01	0,95	0	0	0	0	0,02	0,03	0,95	0	0,024
3,25	1804	0	0,04	0,01	0	0,95	0	0	0	0	0,02	0,05	0	0,93	0,006
3,25	5042	0	0,04	0	0,02	0,92	0	0	0,01	0,01	0	0,97	0,02	0,01	0,028
3,25	4421	0	0,04	0	0	0,95	0	0	0	0,01	0,01	0,04	0,95	0	0,02
3,25	5849	0	0,04	0	0	0,96	0	0	0	0	0,01	0,99	0	0	0,022
3,25	63844	0	0,04	0	0	0,96	0	0	0	0	0	0,99	0,01	0	0,03
3,25	2317	0	0,04	0	0	0,96	0	0	0	0	0	0,04	0,04	0,92	0,02
3,25	21492	0	0,04	0	0	0,95	0	0,01	0	0	0,95	0,04	0	0,01	0,042
3,25	5652	0	0,04	0,01	0	0,95	0	0	0	0	0	0,04	0,95	0,01	0,032
3,25	8087	0	0,04	0	0	0,9	0	0	0,06	0	0	0,04	0,9	0,06	0,032
3,25	22988	0	0,04	0	0,01	0,95	0	0	0	0	0	0,05	0	0,95	0,016
3,25	30445	0	0,04	0	0,01	0,89	0	0	0,06	0	0,08	0,62	0,01	0,29	0,028
3,25	4606	0,01	0,04	0	0,03	0,92	0	0	0	0	0,19	0,05	0,74	0,02	0,042
3,25	7688	0	0,04	0	0	0,96	0	0	0	0	0	0,1	0,04	0,86	0,01
3,25	5632	0	0,04	0	0	0,96	0	0	0	0	0,48	0,03	0,49	0	0,02
3,25	5114	0	0,04	0	0	0,94	0,01	0,01	0	0	0,01	0,04	0,01	0,94	0,018
3,25	28176	0	0,04	0	0	0,96	0	0	0	0	0	0,04	0	0,96	0,008
3,25	4612	0	0,04	0	0	0,96	0	0	0	0	0	0,04	0,96	0	0,024
3,25	13770	0	0,04	0	0,03	0,92	0	0,01	0	0	0	0,04	0,92	0,04	0,036
3,25	6813	0	0,04	0,02	0	0,92	0,02	0	0	0	0,92	0,03	0,05	0	0,042
3,25	40381	0	0,04	0	0	0,96	0	0	0	0	0	0,04	0,03	0,93	0,012
3,25	11774	0	0,04	0	0	0,96	0	0	0	0	0	0,04	0	0,96	0,004
3,25	7287	0	0,01	0,01	0,06	0,92	0	0	0	0	0,06	0	0	0,94	0,046
3,25	5038	0	0,03	0	0,08	0,89	0	0	0	0	0	0,91	0,09	0	0,046
3,25	13193	0	0,01	0	0,06	0,91	0	0	0,02	0	0,06	0,01	0,01	0,92	0,04

3,25	19227	0	0,04	0	0	0,83	0	0	0,13	0	0	0,99	0	0,01	0,034
3,25	5151	0	0,04	0	0	0,96	0	0	0	0	0	0,04	0,96	0	0,03
3,25	1301	0	0,04	0	0,01	0,95	0	0	0	0	0,01	0,99	0	0	0,03
3,25	21476	0	0,04	0	0	0,95	0,01	0	0	0	0	0,01	0,95	0,04	0,016
3,25	1077	0	0,04	0	0	0,96	0	0	0	0	0,49	0,04	0	0,47	0,02
3,25	19673	0	0,04	0	0	0,96	0	0	0	0	0	0,04	0	0,96	0,012
3,25	16550	0	0	0	0,1	0	0,9	0	0	0	0,1	0,7	0,07	0,13	0,048
3,25	3543	0	0,04	0	0	0,96	0	0	0	0	0	0,04	0	0,96	0,02
3,25	1994	0	0,04	0	0	0,93	0,03	0	0	0	0,93	0,07	0	0	0,034
3,25	1509	0	0,04	0	0	0,96	0	0	0	0	0	0,04	0	0,96	0,006
3,25	5519	0	0,04	0	0	0,95	0	0	0	0,01	0,01	0,99	0	0	0,026
3,25	3596	0	0,04	0	0	0,95	0	0,01	0	0	0	0,04	0,01	0,95	0,008
3,25	3088	0,01	0,04	0	0	0,93	0	0	0,02	0	0,02	0,03	0,94	0,01	0,03
3,25	14089	0	0,04	0	0	0,93	0	0,01	0,02	0	0	0,98	0,02	0	0,028
3,25	2302	0	0,04	0	0,01	0,95	0	0	0	0	0	0,04	0,01	0,95	0,02
3,25	8883	0	0,04	0	0	0,95	0	0	0	0,01	0	0,02	0	0,98	0,014
3,25	4683	0	0,04	0	0	0,95	0	0	0,01	0	0	0,04	0	0,96	0,008
3,25	3648	0	0,04	0,01	0	0,93	0	0	0,02	0	0,01	0,05	0,01	0,93	0,006
3,25	2735	0	0,04	0	0	0,96	0	0	0	0	0	1	0	0	0,026
3,25	19406	0	0,04	0,01	0	0,94	0	0	0,01	0	0	0,05	0,94	0,01	0,028
3,25	13190	0,01	0,04	0	0	0,94	0,01	0	0	0	0,94	0,04	0	0,02	0,044
3,25	3873	0	0,04	0	0	0,89	0	0,01	0,06	0	0,86	0,11	0	0,03	0,042
3,25	9223	0	0,04	0	0	0,95	0,01	0	0	0	0	0,04	0,5	0,46	0,024
3,25	16479	0,01	0,04	0,01	0	0,94	0	0	0	0	0	0,04	0,02	0,94	0,012
3,25	2018	0	0,04	0	0	0,96	0	0	0	0	0,2	0,13	0,66	0,01	0,018
3,25	3388	0,01	0,04	0	0	0,93	0,01	0	0	0,01	0,01	0,98	0	0,01	0,03
3,25	13907	0,01	0,04	0	0	0,95	0	0	0	0	0	0,05	0	0,95	0,022
3,25	3381	0	0,04	0,01	0,03	0,92	0	0	0	0	0	0,07	0,21	0,72	0,044
3,25	15906	0	0,04	0	0	0,96	0	0	0	0	0	1	0	0	0,02
3,25	5465	0	0,04	0	0	0,95	0	0	0,01	0	0,01	0,04	0	0,95	0,006
3,25	11010	0,06	0	0	0	0,89	0,05	0	0	0	0,95	0	0,05	0	0,046
3,25	22771	0,01	0,04	0	0	0,94	0,01	0	0	0	0,97	0,03	0	0	0,048
3,25	1803	0	0,04	0	0	0,96	0	0	0	0	0	0,04	0,96	0	0,03

3,25	10541	0	0,04	0	0	0,96	0	0	0	0	0	0,02	0,95	0,03	0,034
3,25	10971	0,02	0,04	0	0	0,82	0,1	0	0	0,02	0,82	0,04	0,12	0,02	0,03
3,25	6273	0,02	0,04	0	0	0,9	0,04	0	0	0	0	0,14	0,02	0,84	0,022
3,25	7068	0,01	0,04	0	0	0,95	0	0	0	0	0,01	0,99	0	0	0,034
3,25	2879	0	0,04	0	0	0,94	0	0	0,01	0,01	0,01	0,29	0,68	0,02	0,02
3,25	22203	0,01	0,04	0	0	0,93	0,02	0	0	0	0,04	0,15	0,8	0,01	0,028
3,25	4802	0	0,04	0	0,06	0,9	0	0	0	0	0,9	0,04	0,06	0	0,046
3,25	10503	0	0,04	0	0	0,96	0	0	0	0	0	0,04	0	0,96	0,006
3,25	6202	0	0,04	0	0	0,95	0	0	0	0,01	0	0,05	0	0,95	0,008
3,25	2661	0	0,04	0	0	0,95	0	0	0	0,01	0,01	0,04	0,95	0	0,022
3,25	6884	0	0,04	0,01	0	0,95	0	0	0	0	0,01	0,04	0	0,95	0,004
3,25	8376	0	0,04	0	0,02	0,93	0,01	0	0	0	0,29	0,05	0,66	0	0,022
3,25	2290	0	0,04	0	0	0,96	0	0	0	0	0,05	0,04	0,91	0	0,016
3,25	9678	0	0,04	0	0	0,96	0	0	0	0	0,96	0,04	0	0	0,042
3,25	2996	0	0,04	0	0	0,96	0	0	0	0	0,02	0,04	0	0,94	0,006
3,25	10772	0	0,04	0	0	0,96	0	0	0	0	0	0,05	0,95	0	0,032
3,25	17088	0,01	0,04	0	0	0,94	0	0	0,01	0	0,33	0,05	0,01	0,61	0,032
3,25	2558	0	0,04	0,04	0	0,92	0	0	0	0	0,03	0,28	0,69	0	0,046
3,25	11043	0	0,04	0	0	0,96	0	0	0	0	0,01	0,03	0	0,96	0,01
3,25	3776	0	0,04	0	0	0,95	0,01	0	0	0	0	0,04	0,95	0,01	0,034
3,5	11208	0	0,04	0	0	0,96	0	0	0	0	0,04	0,04	0	0,92	0,014
3,5	2501	0,02	0,04	0	0	0,89	0,02	0	0,03	0	0,02	0,06	0,03	0,89	0,036
3,5	44038	0,01	0,04	0	0	0,95	0	0	0	0	0	0,05	0,95	0	0,04
3,5	9407	0	0,04	0	0	0,96	0	0	0	0	0	0,03	0,96	0,01	0,012
3,5	5239	0	0,04	0	0	0,96	0	0	0	0	0	0,04	0	0,96	0,01
3,5	31327	0	0,04	0	0	0,96	0	0	0	0	0	1	0	0	0,02
3,5	12126	0	0,04	0	0	0,95	0	0	0,01	0	0	0,04	0,01	0,95	0,01
3,5	4095	0	0,04	0,01	0	0,93	0	0	0,02	0	0,03	0,01	0,96	0	0,028
3,5	4123	0,02	0,04	0	0	0,94	0	0	0	0	0	0,06	0,92	0,02	0,034
3,5	9377	0	0,04	0,04	0	0,91	0,01	0	0	0	0,94	0,05	0,01	0	0,04
3,5	26540	0	0,04	0,01	0	0,95	0	0	0	0	0	0,06	0,94	0	0,02
3,5	6416	0,03	0,04	0	0,01	0,92	0	0	0	0	0,03	0,04	0,93	0	0,044
3,5	17486	0	0,04	0	0	0,93	0,03	0	0	0	0,93	0,07	0	0	0,036

3,5	31945	0	0,04	0	0	0,94	0	0,02	0	0	0	0,56	0,02	0,42	0,018
3,5	7415	0	0,04	0	0	0,95	0	0	0	0,01	0,01	0,99	0	0	0,032
3,5	10758	0	0,04	0	0,05	0,78	0,12	0	0	0,01	0,9	0,09	0,01	0	0,04
3,5	1834	0	0,04	0	0	0,95	0	0	0	0,01	0	0,05	0	0,95	0,012
3,5	13616	0	0,04	0	0	0,96	0	0	0	0	0	0,04	0	0,96	0,01
3,5	6833	0,01	0,04	0	0	0,94	0	0,01	0	0	0	0,02	0,98	0	0,034
3,5	8437	0	0,04	0	0	0,95	0	0	0	0,01	0,01	0,04	0	0,95	0,018
3,5	6258	0	0,04	0	0	0,96	0	0	0	0	0	0,04	0	0,96	0,008
3,5	1413	0,01	0,04	0	0	0,94	0	0	0	0,01	0	0,1	0,01	0,89	0,022
3,5	11765	0	0,01	0	0,11	0,75	0,01	0	0,12	0	0	0,02	0,87	0,11	0,04
3,5	1982	0	0,04	0	0	0,96	0	0	0	0	0	1	0	0	0,022
3,5	6017	0	0,04	0	0	0,95	0,01	0	0	0	0	0,06	0,94	0	0,034
3,5	2613	0	0,04	0	0,01	0,95	0	0	0	0	0	0,04	0,07	0,89	0,008
3,5	6257	0,01	0,04	0	0	0,86	0	0	0,09	0	0,03	0,04	0,89	0,04	0,038
3,5	13448	0	0,04	0	0	0,96	0	0	0	0	0	0,01	0,96	0,03	0,028
3,5	7231	0	0,04	0	0,02	0,94	0	0	0	0	0	0,04	0,95	0,01	0,032
3,5	24387	0	0,01	0,01	0,13	0,83	0,01	0	0,01	0	0,16	0,84	0	0	0,048
3,5	20656	0,01	0,03	0,01	0,06	0,86	0	0,03	0	0	0,86	0,08	0,06	0	0,048
3,5	3314	0	0,04	0,01	0,01	0,93	0	0,01	0	0	0,02	0,04	0,38	0,56	0,032
3,5	10456	0,01	0	0,03	0,14	0,07	0,75	0	0	0	0,96	0,03	0,01	0	0,046
3,5	15799	0	0,04	0	0	0,96	0	0	0	0	0	0,04	0	0,96	0,014
3,5	17665	0	0,04	0	0	0,96	0	0	0	0	0	0,04	0,96	0	0,034
3,5	30208	0	0	0	0,07	0,91	0,01	0,01	0	0	0,01	0,01	0,07	0,91	0,044
3,5	10340	0	0,04	0	0	0,94	0,02	0	0	0	0,01	0,98	0	0,01	0,018
3,5	12014	0,01	0,01	0,01	0,1	0,82	0,03	0	0,02	0	0,82	0,01	0,14	0,03	0,044
3,5	4429	0	0,04	0	0	0,94	0	0	0,02	0	0	0,04	0,94	0,02	0,024
3,5	4071	0	0,04	0	0	0,96	0	0	0	0	0,96	0,04	0	0	0,036
3,5	8599	0,01	0,04	0	0,02	0,85	0,07	0	0	0,01	0,01	0,09	0,84	0,06	0,034
3,5	7235	0	0,04	0,01	0,07	0,87	0,01	0	0	0	0,01	0,05	0,94	0	0,042
3,5	23011	0	0	0	0,1	0,24	0,63	0,03	0	0	0,18	0,58	0,24	0	0,048
3,5	12356	0	0,04	0	0,02	0,94	0	0	0	0	0	0,98	0	0,02	0,032
3,5	2708	0	0,04	0	0,02	0,93	0	0,01	0	0	0	0,97	0,02	0,01	0,046
3,5	2407	0	0,04	0	0	0,96	0	0	0	0	0	1	0	0	0,032

3,5	37318	0	0,04	0	0	0,89	0	0,07	0	0	0	0,04	0,96	0	0,046
3,5	6439	0	0	0	0,43	0,54	0,02	0	0	0,01	0,99	0	0	0,01	0,046
3,5	37129	0,02	0,04	0	0	0,93	0	0	0,01	0	0,02	0,04	0	0,94	0,03
3,5	9971	0	0,04	0	0,08	0,88	0	0	0	0	0	0,04	0,96	0	0,042
3,5	8275	0	0,04	0	0	0,96	0	0	0	0	0	0,32	0	0,68	0,012
3,5	2947	0	0,04	0	0	0,93	0	0,02	0	0,01	0,21	0,12	0	0,67	0,016
3,5	2632	0	0,04	0	0	0,95	0	0,01	0	0	0	0,04	0	0,96	0,018
3,5	9954	0	0,04	0	0	0,95	0,01	0	0	0	0,01	0,04	0	0,95	0,01
3,5	8466	0	0,04	0	0	0,96	0	0	0	0	0	0,03	0	0,97	0,01
3,5	4739	0	0,04	0	0	0,96	0	0	0	0	0	0,04	0	0,96	0,006
3,5	19907	0	0,04	0	0,01	0,95	0	0	0	0	0	0,01	0,99	0	0,028
3,5	5054	0	0,04	0	0	0,95	0	0	0	0,01	0	0,04	0	0,96	0,008
3,5	3786	0,01	0,04	0	0	0,94	0	0	0	0,01	0,01	0,05	0	0,94	0,014
3,5	1478	0	0,04	0	0	0,95	0	0,01	0	0	0,01	0,99	0	0	0,03
3,5	12147	0	0,04	0	0	0,96	0	0	0	0	0,25	0,04	0,06	0,65	0,022
3,5	25474	0	0,04	0	0	0,96	0	0	0	0	0	0,54	0	0,46	0,024
3,5	6874	0	0,04	0	0	0,94	0	0,01	0,01	0	0,01	0,02	0,03	0,94	0,014
3,5	2437	0	0,04	0,01	0	0,93	0	0,01	0	0,01	0,93	0,06	0,01	0	0,044
3,5	1813	0	0,04	0	0	0,96	0	0	0	0	0	0,04	0,91	0,05	0,016
3,5	1849	0	0,04	0	0	0,89	0	0	0,06	0,01	0,01	0,1	0	0,89	0,008
3,5	14786	0	0,04	0	0	0,95	0	0,01	0	0	0,01	0,04	0,01	0,94	0,016
3,5	13654	0	0,04	0	0	0,96	0	0	0	0	0,96	0,04	0	0	0,036
3,5	22443	0	0,04	0	0	0,96	0	0	0	0	0	0,04	0	0,96	0,016
3,5	7123	0,01	0,04	0	0	0,95	0	0	0	0	0	0,05	0	0,95	0,018
3,5	2024	0	0,04	0	0	0,95	0	0	0,01	0	0,95	0,04	0	0,01	0,038
3,5	11319	0	0,04	0	0	0,96	0	0	0	0	0	0,04	0	0,96	0,008
3,5	17716	0	0,01	0	0,11	0,83	0,03	0,02	0	0	0,13	0,01	0,86	0	0,048
3,5	19311	0	0,04	0	0	0,95	0,01	0	0	0	0	0,04	0,95	0,01	0,036
3,5	16639	0	0,03	0,01	0,03	0,85	0	0,02	0,06	0	0,03	0,09	0,86	0,02	0,038
3,5	4169	0,01	0,04	0	0,01	0,94	0	0	0	0	0	0,02	0,01	0,97	0,018
3,5	3540	0	0,04	0	0	0,95	0,01	0	0	0	0,95	0,04	0	0,01	0,04
3,5	4645	0	0,04	0	0,01	0,84	0	0	0,11	0	0,02	0,92	0,06	0	0,038
3,5	3254	0	0,04	0	0	0,95	0	0	0,01	0	0	0,04	0,95	0,01	0,024

3,5	4929	0	0,04	0	0	0,96	0	0	0	0	0	0,04	0	0,96	0,008
3,5	8610	0	0,04	0	0	0,96	0	0	0	0	0,14	0,04	0	0,82	0,012
3,5	4216	0	0,04	0,02	0	0,94	0	0	0	0	0,02	0,84	0,14	0	0,03
3,5	13059	0	0,04	0	0	0,96	0	0	0	0	0	0,94	0,06	0	0,018
3,5	7250	0	0,04	0	0	0,96	0	0	0	0	0,12	0,04	0	0,84	0,016
3,5	6898	0	0,04	0	0,01	0,95	0	0	0	0	0	0,05	0	0,95	0,014
3,5	8176	0	0,04	0	0	0,95	0	0,01	0	0	0	0,04	0	0,96	0,018
3,5	11912	0	0,04	0,01	0	0,95	0	0	0	0	0,01	0,04	0,95	0	0,03
3,5	34217	0	0,04	0	0,03	0,93	0	0	0	0	0	0,04	0,96	0	0,044
3,5	8900	0	0,04	0	0	0,96	0	0	0	0	0	0,46	0,54	0	0,018
3,5	38745	0,01	0,04	0	0,01	0,93	0	0	0	0,01	0	0,05	0,94	0,01	0,048
3,5	2324	0,01	0,04	0	0	0,95	0	0	0	0	0	0,05	0,95	0	0,034
3,5	4426	0	0,04	0	0,01	0,94	0,01	0	0	0	0	0,97	0,01	0,02	0,032
3,5	9547	0	0,04	0	0,01	0,9	0	0	0,05	0	0,03	0	0,97	0	0,012
3,5	7756	0,01	0,04	0	0	0,93	0,01	0,01	0	0	0,93	0,06	0,01	0	0,048
3,5	2093	0,02	0,04	0	0	0,93	0	0	0,01	0	0	0,9	0,1	0	0,048
3,5	9211	0	0,04	0	0	0,95	0	0	0,01	0	0	0,98	0	0,02	0,02
3,5	8384	0,01	0,04	0	0	0,95	0	0	0	0	0	0,04	0,03	0,93	0,032
3,5	4964	0	0,04	0	0,01	0,94	0	0	0,01	0	0,01	0,04	0,01	0,94	0,018
3,5	6575	0	0,04	0,02	0	0,94	0	0	0	0	0,96	0,01	0,03	0	0,048
3,5	21400	0,02	0,04	0	0	0,94	0	0	0	0	0	0,04	0,96	0	0,042
3,75	1664	0	0,04	0	0	0,96	0	0	0	0	0	0,34	0,66	0	0,018
3,75	5535	0	0,03	0	0,09	0,88	0	0	0	0	0,36	0,12	0,02	0,5	0,046
3,75	36951	0	0,04	0	0	0,95	0,01	0	0	0	0,07	0,92	0	0,01	0,04
3,75	16996	0	0,04	0	0	0,96	0	0	0	0	0	0,04	0	0,96	0,028
3,75	2336	0	0,04	0	0	0,95	0	0	0	0,01	0	0,02	0,77	0,21	0,014
3,75	9887	0	0,04	0	0,1	0,76	0,1	0	0	0	0,76	0,04	0,01	0,19	0,048
3,75	3752	0	0,04	0	0	0,96	0	0	0	0	0	0,04	0,96	0	0,024
3,75	2728	0	0,04	0	0	0,96	0	0	0	0	0	0,03	0,96	0,01	0,028
3,75	10332	0	0	0	0,36	0	0,64	0	0	0	1	0	0	0	0,046
3,75	3237	0	0,04	0	0	0,96	0	0	0	0	0	0,08	0,39	0,53	0,016
3,75	2710	0	0,04	0	0,01	0,93	0	0	0,02	0	0,02	0,04	0,01	0,93	0,012
3,75	8018	0	0,04	0	0	0,96	0	0	0	0	0,93	0,04	0,03	0	0,048

3,75	2952	0	0,04	0	0	0,96	0	0	0	0	0	1	0	0	0,024
3,75	23363	0	0,04	0	0,01	0,94	0	0	0	0,01	0,94	0,04	0,02	0	0,038
3,75	10081	0	0,04	0	0	0,96	0	0	0	0	0	0,04	0,96	0	0,02
3,75	19200	0	0,04	0	0	0,96	0	0	0	0	0	0,04	0,96	0	0,024
3,75	6519	0	0,04	0,03	0,02	0	0	0	0,81	0,1	0,05	0,14	0,81	0	0,038
3,75	16129	0	0,04	0	0,1	0,83	0	0	0,03	0	0,13	0,01	0,83	0,03	0,042
3,75	13138	0	0,04	0,02	0	0,92	0	0	0	0,02	0,07	0,04	0,89	0	0,032
3,75	16690	0,02	0,04	0	0	0,94	0	0	0	0	0	0,06	0,94	0	0,042
3,75	7209	0	0,04	0	0	0,96	0	0	0	0	0,9	0,04	0,06	0	0,042
3,75	8684	0	0,04	0	0	0,96	0	0	0	0	0	0,04	0	0,96	0,006
3,75	6268	0	0,04	0	0	0,96	0	0	0	0	0	0,05	0,83	0,12	0,018
3,75	3581	0	0,04	0	0,03	0,93	0	0	0	0	0,03	0,04	0,93	0	0,042
3,75	5176	0	0,04	0	0	0,82	0,14	0	0	0	0,82	0,04	0,14	0	0,036
3,75	18777	0	0,04	0,01	0	0,92	0	0	0,03	0	0	0,03	0,92	0,05	0,032
3,75	25116	0	0,04	0,01	0,04	0,91	0	0	0	0	0,91	0,08	0	0,01	0,028
3,75	6703	0	0,04	0	0,07	0,87	0,01	0,01	0	0	0,05	0,07	0,88	0	0,048
3,75	15579	0	0,03	0	0,2	0,77	0	0	0	0	0,77	0,03	0,2	0	0,044
3,75	6364	0	0,04	0	0	0,96	0	0	0	0	0	0,04	0,13	0,83	0,01
3,75	8915	0	0,04	0,01	0	0,92	0	0	0,03	0	0,01	0,68	0	0,31	0,02
3,75	14086	0	0,04	0,01	0,01	0,94	0	0	0	0	0,07	0	0	0,93	0,014
3,75	21703	0,02	0,04	0	0	0,94	0	0	0	0	0,78	0,05	0	0,17	0,046
3,75	13031	0	0,04	0	0	0,96	0	0	0	0	0	0,04	0,43	0,53	0,018
3,75	2330	0	0,04	0	0	0,96	0	0	0	0	0,96	0,04	0	0	0,026
3,75	9867	0	0,04	0	0	0,96	0	0	0	0	0,08	0,06	0,86	0	0,03
3,75	8260	0	0,04	0	0	0,94	0	0,01	0	0,01	0	0,04	0,01	0,95	0,022
3,75	28860	0	0,04	0	0,01	0,93	0,02	0	0	0	0,95	0,04	0	0,01	0,042
3,75	2527	0	0,04	0	0	0,96	0	0	0	0	0	0,03	0,96	0,01	0,024
3,75	3153	0,01	0,04	0	0	0,9	0	0	0,05	0	0,05	0,04	0	0,91	0,024
3,75	3994	0	0,04	0,01	0	0,95	0	0	0	0	0	0,05	0,87	0,08	0,03
3,75	5659	0,01	0,04	0,01	0	0,94	0	0	0	0	0,01	0,97	0,02	0	0,028
3,75	14730	0	0,04	0	0	0,95	0	0,01	0	0	0	0,04	0,01	0,95	0,014
3,75	3351	0	0,04	0	0	0,96	0	0	0	0	0	0,63	0	0,37	0,01
3,75	3721	0	0,04	0	0	0,9	0	0	0,06	0	0,06	0,02	0	0,92	0,006

3,75	1059	0	0,04	0	0,02	0,92	0	0	0,02	0	0	0,06	0,94	0	0,03
3,75	2566	0	0,04	0	0	0,94	0	0	0	0,02	0,02	0,04	0,94	0	0,026
3,75	5492	0	0,04	0	0	0,94	0	0,01	0,01	0	0	0,04	0,01	0,95	0,014
3,75	2335	0	0,04	0	0	0,96	0	0	0	0	0	0,04	0	0,96	0,016
3,75	2645	0	0,04	0	0	0,95	0	0,01	0	0	0	0,42	0	0,58	0,012
3,75	15506	0	0,04	0	0	0,96	0	0	0	0	0	0,04	0	0,96	0,014
3,75	5814	0	0,04	0	0,01	0,89	0	0	0,06	0	0	0,03	0,06	0,91	0,016
3,75	28772	0	0,04	0	0	0,96	0	0	0	0	0	0,04	0	0,96	0,002
3,75	27746	0	0,04	0	0	0,95	0	0,01	0	0	0	0,99	0	0,01	0,032
3,75	15463	0	0,04	0	0	0,96	0	0	0	0	0	0,97	0	0,03	0,024
3,75	12401	0	0,04	0	0	0,96	0	0	0	0	0,05	0,04	0,9	0,01	0,032
3,75	2016	0	0,04	0	0	0,94	0	0	0,02	0	0	0,04	0,02	0,94	0,012
3,75	1777	0	0,04	0	0	0,95	0	0	0	0,01	0	0,05	0,95	0	0,034
3,75	8331	0	0,04	0	0	0,96	0	0	0	0	0	0,04	0	0,96	0,02
3,75	20232	0	0	0	0,14	0,85	0,01	0	0	0	0,15	0	0,85	0	0,046
3,75	2755	0	0,04	0,01	0	0,9	0	0	0,05	0	0	0,05	0,05	0,9	0,016
3,75	2921	0	0,04	0	0	0,95	0,01	0	0	0	0	0,03	0,96	0,01	0,022
3,75	6012	0	0,04	0	0	0,96	0	0	0	0	0	0,04	0	0,96	0,01
3,75	7495	0	0,04	0,01	0	0,94	0	0	0,01	0	0,02	0,04	0,94	0	0,03
3,75	14393	0,01	0,04	0	0	0,95	0	0	0	0	0,96	0,03	0,01	0	0,044
3,75	1325	0,01	0,04	0	0	0,92	0,01	0	0,02	0	0,16	0,04	0,03	0,77	0,024
3,75	3900	0	0,04	0	0,01	0,95	0	0	0	0	0	0,04	0,96	0	0,022
3,75	9247	0	0,04	0	0	0,95	0	0,01	0	0	0	0,05	0	0,95	0,016
3,75	8146	0	0,04	0	0,05	0,9	0	0	0,01	0	0,68	0,05	0,05	0,22	0,04
3,75	11158	0	0,04	0,01	0,01	0,94	0	0	0	0	0,03	0,02	0,94	0,01	0,03
3,75	4544	0	0,04	0,01	0,01	0,94	0	0	0	0	0,06	0	0	0,94	0,03
3,75	13870	0,02	0,03	0	0	0,93	0,01	0	0	0,01	0,94	0,03	0,02	0,01	0,04
3,75	8086	0	0,04	0	0,01	0,95	0	0	0	0	0,02	0,05	0	0,93	0,02
3,75	18019	0	0,04	0,01	0,01	0,94	0	0	0	0	0,01	0,04	0	0,95	0,014
3,75	14710	0	0,04	0	0	0,95	0	0	0	0,01	0	0,04	0	0,96	0,006
3,75	41798	0	0,03	0	0,16	0,8	0	0	0	0,01	0,8	0,04	0,16	0	0,048
3,75	5799	0	0,04	0	0	0,96	0	0	0	0	0,95	0,04	0	0,01	0,03
3,75	2778	0	0,04	0	0	0,96	0	0	0	0	0	0,06	0	0,94	0,008

3,75	23203	0	0,04	0,01	0	0,86	0	0,02	0,07	0	0,03	0,03	0,89	0,05	0,036
3,75	9103	0	0	0	0,1	0,79	0	0	0,11	0	0,1	0,76	0	0,14	0,048
3,75	4503	0	0,04	0	0,01	0,95	0	0	0	0	0	0,04	0,96	0	0,042
3,75	1763	0	0,04	0	0	0,96	0	0	0	0	0	0,04	0,96	0	0,028
3,75	7328	0	0,04	0	0	0,95	0,01	0	0	0	0	0,04	0	0,96	0,008
3,75	11271	0	0,04	0	0	0,96	0	0	0	0	0,96	0	0	0,04	0,034
3,75	18888	0	0,04	0	0,01	0,94	0	0,01	0	0	0,01	0,22	0,18	0,59	0,024
3,75	8408	0,01	0,04	0	0,04	0,91	0	0	0	0	0,01	0,08	0	0,91	0,038
3,75	13335	0	0,04	0	0,01	0,92	0	0	0,03	0	0,04	0,04	0	0,92	0,016
3,75	18068	0	0,04	0	0,01	0,95	0	0	0	0	0,05	0,12	0,18	0,65	0,036
3,75	1935	0	0,04	0	0,01	0,95	0	0	0	0	0	0,04	0,52	0,44	0,026
3,75	28458	0	0,04	0	0	0,95	0	0	0	0,01	0	0,03	0,01	0,96	0,014
3,75	26224	0,01	0,04	0,04	0	0,9	0	0	0,01	0	0,72	0,06	0,02	0,2	0,044
3,75	9947	0	0,04	0	0	0,93	0	0,02	0	0,01	0,28	0,06	0,66	0	0,036
3,75	39919	0	0,04	0,01	0	0,95	0	0	0	0	0	0,04	0	0,96	0,01
3,75	2752	0	0,04	0,02	0	0,94	0	0	0	0	0,94	0,04	0	0,02	0,04
3,75	42509	0,01	0,04	0	0	0,95	0	0	0	0	0	0,13	0	0,87	0,02
3,75	16423	0	0,04	0	0	0,93	0,01	0	0,01	0,01	0	0,06	0,94	0	0,03
3,75	4624	0	0,03	0	0,13	0,51	0,3	0,03	0	0	0,51	0,33	0,16	0	0,048
3,75	48114	0	0,04	0	0	0,96	0	0	0	0	0	0,04	0,06	0,9	0,008
3,75	11105	0	0,04	0	0	0,93	0	0	0	0,03	0	0,04	0,96	0	0,024
3,75	2577	0	0,04	0	0,02	0,94	0	0	0	0	0	0,06	0,58	0,36	0,018
4	12133	0	0,04	0	0	0,96	0	0	0	0	0	0,04	0	0,96	0,008
4	2729	0	0,04	0	0	0,92	0	0	0,04	0	0	0,13	0,86	0,01	0,016
4	5624	0	0,04	0	0	0,96	0	0	0	0	0	0,04	0,96	0	0,014
4	21924	0	0,04	0	0	0,96	0	0	0	0	0	0,04	0,96	0	0,028
4	13820	0,01	0,04	0	0	0,93	0,01	0,01	0	0	0,64	0,06	0,3	0	0,042
4	3809	0	0,02	0,02	0,1	0,83	0,01	0	0,02	0	0,85	0,02	0	0,13	0,048
4	3605	0	0,04	0	0	0,96	0	0	0	0	0	0,04	0	0,96	0,012
4	25030	0	0,04	0	0	0,95	0	0	0,01	0	0,01	0,02	0	0,97	0,01
4	9490	0	0,04	0	0	0,96	0	0	0	0	0,96	0,04	0	0	0,034
4	4524	0	0,04	0	0	0,94	0	0	0,02	0	0	0,04	0,01	0,95	0,008
4	29233	0,01	0,04	0	0	0,95	0	0	0	0	0	0,04	0,95	0,01	0,034

4	31736	0	0,04	0	0	0,95	0	0	0,01	0	0,01	0,04	0	0,95	0,01
4	1420	0	0,04	0	0	0,96	0	0	0	0	0	1	0	0	0,02
4	7753	0	0,04	0	0	0,95	0,01	0	0	0	0,02	0,01	0,01	0,96	0,01
4	15566	0	0,04	0	0	0,96	0	0	0	0	0	0,04	0	0,96	0,008
4	5027	0	0,04	0,04	0	0,92	0	0	0	0	0	0,05	0	0,95	0,022
4	1964	0	0,04	0	0	0,95	0,01	0	0	0	0,01	0,03	0,56	0,4	0,012
4	2341	0	0,04	0,01	0	0,95	0	0	0	0	0	0,04	0,01	0,95	0,01
4	35229	0	0,04	0	0	0,95	0	0	0,01	0	0	0,04	0,95	0,01	0,018
4	9345	0,02	0,04	0	0,01	0,93	0	0	0	0	0	0,04	0,96	0	0,048
4	12112	0	0,04	0	0	0,96	0	0	0	0	0	0,13	0	0,87	0,012
4	5136	0	0,04	0	0	0,96	0	0	0	0	0	0,04	0,96	0	0,03
4	16933	0	0,04	0	0	0,96	0	0	0	0	0	0,04	0	0,96	0,01
4	4926	0	0,04	0	0	0,96	0	0	0	0	0	0	0,97	0,03	0,024
4	24823	0	0,04	0	0	0,96	0	0	0	0	0,96	0,04	0	0	0,026
4	10882	0	0,04	0	0,09	0,85	0	0	0,02	0	0,94	0,06	0	0	0,042
4	11389	0	0,04	0	0,01	0,95	0	0	0	0	0	0,04	0,95	0,01	0,028
4	17029	0	0,04	0,01	0,02	0,93	0	0	0	0	0,03	0,04	0	0,93	0,034
4	5193	0	0,04	0	0	0,96	0	0	0	0	0	1	0	0	0,024
4	5977	0	0,04	0,01	0	0,95	0	0	0	0	0	0,04	0,96	0	0,022
4	22503	0	0,04	0	0	0,95	0	0,01	0	0	0,02	0,02	0,96	0	0,04
4	9825	0	0,04	0	0	0,96	0	0	0	0	0	0,04	0	0,96	0,006
4	12002	0	0,04	0	0,03	0,93	0	0	0	0	0	0,07	0,93	0	0,048
4	61091	0	0,04	0,02	0	0,93	0	0,01	0	0	0,06	0	0	0,94	0,022
4	17382	0	0,04	0	0	0,95	0	0,01	0	0	0,22	0,05	0	0,73	0,016
4	8417	0	0,04	0	0	0,96	0	0	0	0	0	0,09	0	0,91	0,01
4	14894	0	0,04	0	0	0,94	0	0,02	0	0	0	0,01	0,92	0,07	0,036
4	15965	0	0,04	0	0	0,96	0	0	0	0	0	0,6	0,4	0	0,018
4	40167	0	0,04	0	0	0,96	0	0	0	0	0	0,04	0	0,96	0,006
4	3585	0	0,04	0	0	0	0,22	0	0,74	0	0,22	0,78	0	0	0,022
4	12933	0	0,04	0	0	0,96	0	0	0	0	0	0,04	0	0,96	0,008
4	10681	0	0,04	0	0,01	0,95	0	0	0	0	0,04	0	0,96	0	0,02
4	21456	0,01	0,04	0	0	0,95	0	0	0	0	0	0,04	0,95	0,01	0,04
4	1586	0	0,04	0	0	0,96	0	0	0	0	0	0,04	0,96	0	0,02

4	28124	0	0,04	0	0	0,96	0	0	0	0	0	0,04	0,96	0	0,03
4	14577	0,01	0,04	0	0	0,95	0	0	0	0	0,95	0,04	0,01	0	0,044
4	8310	0	0,04	0	0	0,96	0	0	0	0	0	1	0	0	0,036
4	34738	0	0,04	0	0	0,94	0	0	0,02	0	0	0,04	0	0,96	0,004
4	21034	0,01	0,04	0,02	0	0,92	0	0	0,01	0	0,01	0,05	0,02	0,92	0,018
4	19544	0	0,04	0	0,14	0,81	0,01	0	0	0	0,81	0,05	0,14	0	0,042
4	9433	0	0,04	0,01	0	0,94	0,01	0	0	0	0,28	0,04	0,67	0,01	0,036
4	20752	0	0,04	0	0	0,78	0	0	0,18	0	0	0,04	0,18	0,78	0,022
4	48818	0	0,04	0	0	0,96	0	0	0	0	0	0,04	0	0,96	0,012
4	17450	0	0,04	0	0,01	0,93	0	0	0,01	0,01	0	0,05	0,94	0,01	0,024
4	27862	0,02	0,04	0	0	0,94	0	0	0	0	0,02	0,98	0	0	0,046
4	31333	0	0,04	0	0	0,96	0	0	0	0	0	0,04	0	0,96	0,01
4	3519	0	0,04	0	0	0,96	0	0	0	0	0,03	0,01	0	0,96	0,012
4	11798	0	0,04	0	0	0,96	0	0	0	0	0	0,05	0	0,95	0,01
4	9066	0	0,04	0	0	0,94	0,01	0	0,01	0	0	0,04	0,02	0,94	0,004
4	4583	0	0,04	0	0	0,95	0,01	0	0	0	0	0,04	0,95	0,01	0,026
4	2440	0	0,04	0	0	0,94	0,01	0	0,01	0	0,03	0,02	0	0,95	0,008
4	1987	0	0,04	0	0,01	0,95	0	0	0	0	0	0,12	0,18	0,7	0,026
4	16027	0	0,04	0,01	0	0,95	0	0	0	0	0,81	0,05	0	0,14	0,046
4	13351	0	0,04	0	0	0,89	0,03	0	0,02	0,02	0,02	0,93	0,04	0,01	0,024
4	7861	0	0,04	0,01	0	0,95	0	0	0	0	0	0,04	0	0,96	0,006
4	1380	0	0,04	0,01	0	0,95	0	0	0	0	0,22	0,04	0,02	0,72	0,022
4	1647	0	0,04	0	0	0,96	0	0	0	0	0	0,04	0	0,96	0,014
4	18741	0	0,04	0	0,02	0,93	0	0	0,01	0	0,02	0,05	0,93	0	0,038
4	37967	0	0,04	0	0	0,96	0	0	0	0	0	0,04	0	0,96	0,008
4	14631	0	0,04	0	0	0,96	0	0	0	0	0	1	0	0	0,032
4	8467	0	0,04	0,01	0	0,93	0	0	0	0,02	0,01	0,03	0,02	0,94	0,002
4	30478	0	0,04	0	0	0,95	0,01	0	0	0	0,01	0,03	0,12	0,84	0,024
4	20328	0	0,04	0	0,01	0,95	0	0	0	0	0,01	0,99	0	0	0,02
4	5587	0	0,04	0	0	0,94	0	0	0	0,02	0	0,97	0,01	0,02	0,032
4	7158	0	0,04	0	0	0,89	0	0	0,07	0	0	0,11	0,89	0	0,022
4	9121	0,01	0,04	0	0	0,95	0	0	0	0	0,01	0,03	0,06	0,9	0,018
4	23028	0	0,04	0,01	0	0,95	0	0	0	0	0	0,05	0,84	0,11	0,022

4	9191	0	0,04	0	0,01	0,95	0	0	0	0	0,19	0,22	0,01	0,58	0,012
4	16075	0	0,04	0	0	0,95	0	0	0,01	0	0	0,05	0	0,95	0,006
4	2235	0	0,04	0	0	0,96	0	0	0	0	0,09	0,04	0,64	0,23	0,01
4	2597	0	0,04	0,05	0	0,9	0	0	0	0,01	0	0,1	0,9	0	0,042
4	3517	0	0,04	0	0	0,95	0,01	0	0	0	0,01	0,04	0	0,95	0,004
4	23476	0	0,04	0	0,02	0,88	0,01	0,05	0	0	0,94	0,05	0,01	0	0,048
4	4130	0	0,04	0	0	0,92	0	0	0	0,04	0,97	0,03	0	0	0,048
4	6506	0	0,04	0	0,11	0,85	0	0	0	0	0,72	0,15	0,13	0	0,046
4	38565	0	0,04	0	0	0,92	0	0	0,03	0,01	0,03	0,04	0,01	0,92	0,014
4	10192	0,01	0,04	0,04	0	0,9	0	0,01	0	0	0,01	0,04	0,05	0,9	0,03
4	23542	0	0,04	0	0	0,92	0	0	0,04	0	0,04	0,73	0,01	0,22	0,01
4	3707	0	0,04	0	0	0,96	0	0	0	0	0	0,04	0	0,96	0,002
4	23001	0	0,04	0	0	0,94	0,02	0	0	0	0,97	0,02	0,01	0	0,04
4	27043	0,01	0,04	0,01	0	0,94	0	0	0	0	0	0,04	0,95	0,01	0,038
4	10255	0	0,04	0	0	0,96	0	0	0	0	0	0,04	0	0,96	0,004
4	17970	0	0,04	0	0	0,93	0	0	0,03	0	0,82	0,04	0,11	0,03	0,036
4	13041	0	0,04	0	0,05	0,01	0	0	0,9	0	0	0,04	0,95	0,01	0,044
4	2298	0	0,04	0	0	0,95	0,01	0	0	0	0	0,03	0	0,97	0,012
4	14692	0	0,04	0	0	0,95	0,01	0	0	0	0,01	0,04	0	0,95	0,02
4	2123	0	0,01	0	0,28	0,7	0	0,01	0	0	0,96	0,01	0,02	0,01	0,046
4	10061	0	0,04	0	0	0,93	0,03	0	0	0	0,04	0	0,93	0,03	0,026
4	8459	0	0,04	0	0	0,96	0	0	0	0	0,66	0,04	0	0,3	0,03
4	6945	0,01	0,04	0,02	0	0,9	0,02	0,01	0	0	0	0,04	0,03	0,93	0,024
4,25	8133	0	0,04	0	0	0,94	0,02	0	0	0	0,02	0,06	0	0,92	0,002
4,25	12557	0	0,04	0	0	0,95	0	0,01	0	0	0	0,04	0,04	0,92	0,024
4,25	7734	0	0,04	0	0	0,95	0	0	0,01	0	0	0,97	0,02	0,01	0,028
4,25	14896	0	0,04	0	0	0,96	0	0	0	0	0	0,98	0	0,02	0,02
4,25	9632	0	0,04	0	0	0,96	0	0	0	0	0	0,04	0,96	0	0,024
4,25	20060	0	0,04	0	0,05	0,89	0,01	0	0,01	0	0	0,1	0,9	0	0,044
4,25	4528	0	0,04	0,01	0	0,92	0	0	0,03	0	0	0,04	0,01	0,95	0,01
4,25	43854	0	0,04	0	0,01	0,94	0,01	0	0	0	0,01	0,05	0	0,94	0,016
4,25	2975	0	0,04	0	0	0,87	0	0	0,09	0	0,07	0,04	0	0,89	0,016
4,25	3616	0	0,04	0,01	0	0,94	0	0	0,01	0	0	0,03	0	0,97	0,01

4,25	17903	0	0,04	0	0	0,95	0	0	0,01	0	0	0,05	0,95	0	0,026
4,25	2763	0	0,04	0	0	0,96	0	0	0	0	0	0,04	0	0,96	0,004
4,25	2504	0,02	0,04	0	0	0,9	0,04	0	0	0	0,41	0,06	0,29	0,24	0,046
4,25	4802	0	0,04	0,02	0	0,91	0	0,02	0	0,01	0,93	0,06	0,01	0	0,028
4,25	15705	0	0,04	0	0,03	0,93	0	0	0	0	0	0,98	0	0,02	0,044
4,25	9579	0	0,04	0	0	0,96	0	0	0	0	0	1	0	0	0,03
4,25	15563	0	0,02	0	0,12	0,81	0	0	0,05	0	0,16	0,81	0	0,03	0,04
4,25	20753	0	0,04	0	0,03	0,93	0	0	0	0	0,03	0,04	0,93	0	0,038
4,25	6297	0	0,04	0	0	0,95	0	0	0,01	0	0	0,04	0,95	0,01	0,026
4,25	10799	0	0,04	0	0	0,96	0	0	0	0	0	0,02	0,96	0,02	0,026
4,25	17730	0,01	0,04	0,03	0	0,92	0	0	0	0	0	0,99	0,01	0	0,048
4,25	4765	0	0,04	0,01	0	0,94	0	0	0	0,01	0	0,05	0,01	0,94	0,006
4,25	8181	0	0,04	0	0	0,94	0,01	0	0	0,01	0	0,04	0,01	0,95	0,008
4,25	3073	0	0,04	0,01	0	0,95	0	0	0	0	0,17	0,04	0,79	0	0,034
4,25	13821	0	0,04	0	0	0,96	0	0	0	0	0	0,33	0,67	0	0,018
4,25	6182	0	0,04	0	0	0,91	0	0	0,05	0	0,05	0,04	0	0,91	0,002
4,25	31909	0	0,04	0	0,03	0,9	0	0	0	0,03	0,03	0,07	0,9	0	0,046
4,25	16383	0	0,04	0	0	0,94	0,02	0	0	0	0	0,04	0	0,96	0,014
4,25	10664	0	0,04	0	0	0,96	0	0	0	0	0	0,04	0	0,96	0,014
4,25	5774	0	0,04	0	0	0,95	0	0	0,01	0	0	0,04	0	0,96	0,01
4,25	20261	0,01	0,04	0	0	0,94	0,01	0	0	0	0,02	0,04	0,94	0	0,028
4,25	21178	0	0,04	0	0	0,86	0	0	0,1	0	0,48	0,04	0,1	0,38	0,026
4,25	30455	0	0,04	0	0,02	0,85	0	0,01	0,08	0	0,08	0,05	0,86	0,01	0,046
4,25	6849	0	0,04	0	0	0,9	0	0,01	0,05	0	0,01	0,04	0,06	0,89	0,016
4,25	8412	0	0,04	0	0	0,96	0	0	0	0	0	0,04	0	0,96	0,008
4,25	23988	0	0,04	0	0	0,93	0,02	0,01	0	0	0	0,99	0,01	0	0,016
4,25	3161	0	0,04	0,01	0,02	0,91	0,02	0	0	0	0	0,05	0,04	0,91	0,03
4,25	14710	0	0,04	0,02	0	0,93	0	0	0,01	0	0,93	0,05	0,02	0	0,04
4,25	6106	0,02	0,04	0	0	0,9	0	0,04	0	0	0,01	0,97	0	0,02	0,048
4,25	48776	0	0,04	0	0	0,96	0	0	0	0	0	0,03	0,96	0,01	0,022
4,25	14183	0	0,04	0	0	0,96	0	0	0	0	0	0	0,04	0,96	0,012
4,25	22930	0	0,04	0,01	0,02	0,93	0	0	0	0	0,03	0,03	0,01	0,93	0,016
4,25	23812	0	0,04	0	0	0,96	0	0	0	0	0	0,04	0	0,96	0,006

4,25	4426	0	0,04	0	0	0,96	0	0	0	0	0	0,04	0	0,96	0,012
4,25	5510	0	0,04	0	0	0,96	0	0	0	0	0	0,01	0,99	0	0,024
4,25	48662	0	0,04	0	0	0,96	0	0	0	0	0	0,04	0	0,96	0,01
4,25	11681	0	0,04	0	0	0,93	0,02	0,01	0	0	0,02	0,04	0,01	0,93	0,02
4,25	5750	0	0,04	0	0,01	0,95	0	0	0	0	0,37	0,05	0,28	0,3	0,028
4,25	18566	0	0,04	0	0	0,95	0	0	0,01	0	0	0,95	0,01	0,04	0,024
4,25	23918	0	0,04	0	0,01	0,95	0	0	0	0	0	0,04	0,34	0,62	0,024
4,25	5639	0	0,04	0,01	0	0,95	0	0	0	0	0	0,56	0,43	0,01	0,028
4,25	7000	0	0,04	0	0	0,96	0	0	0	0	0	0,04	0,96	0	0,022
4,25	8828	0	0,04	0,01	0	0,95	0	0	0	0	0	0,04	0	0,96	0,014
4,25	15499	0	0,04	0	0	0,96	0	0	0	0	0	0,04	0	0,96	0,006
4,25	17613	0	0,04	0,01	0	0,95	0	0	0	0	0,03	0,04	0	0,93	0,024
4,25	16697	0	0,04	0	0,05	0,91	0	0	0	0	0	0,09	0,91	0	0,044
4,25	20993	0	0,03	0	0,15	0,76	0,03	0,03	0	0	0,78	0,03	0,19	0	0,046
4,25	33155	0	0,04	0	0	0,95	0	0	0,01	0	0,95	0,03	0	0,02	0,044
4,25	4947	0	0,04	0	0	0,96	0	0	0	0	0	0,02	0,04	0,94	0,004
4,25	10693	0,01	0,04	0	0	0,95	0	0	0	0	0,99	0,01	0	0	0,044
4,25	8191	0	0,04	0	0	0,91	0,05	0	0	0	0	0,92	0,03	0,05	0,032
4,25	10300	0	0,04	0,01	0	0,95	0	0	0	0	0,01	0,06	0,93	0	0,018
4,25	12231	0	0,04	0	0	0,96	0	0	0	0	0	0,04	0,96	0	0,016
4,25	1606	0	0,04	0	0,01	0,95	0	0	0	0	0	0,03	0,96	0,01	0,024
4,25	72992	0	0,01	0	0,12	0,8	0,01	0	0,06	0	0,13	0	0,8	0,07	0,046
4,25	4739	0	0	0	0,12	0,87	0,01	0	0	0	0,12	0,88	0	0	0,044
4,25	9142	0	0,04	0	0	0,9	0	0	0,06	0	0,8	0	0,06	0,14	0,044
4,25	5017	0	0,04	0	0	0,96	0	0	0	0	0,51	0,02	0,47	0	0,012
4,25	2929	0	0,04	0	0	0,96	0	0	0	0	0	0,04	0	0,96	0,01
4,25	9746	0	0,04	0	0	0,96	0	0	0	0	0,96	0,03	0,01	0	0,044
4,25	31225	0	0,03	0	0,07	0,65	0,23	0	0,02	0	0,07	0	0,64	0,29	0,042
4,25	27716	0	0,04	0	0	0,96	0	0	0	0	0,08	0,91	0	0,01	0,022
4,25	3954	0	0,04	0	0	0,96	0	0	0	0	0,02	0,01	0,5	0,47	0,018
4,25	19148	0	0,02	0	0,1	0,8	0,08	0	0	0	0,03	0,86	0,11	0	0,046
4,25	18787	0	0	0	0,12	0,86	0,02	0	0	0	0,12	0,02	0,86	0	0,042
4,25	38086	0	0,04	0	0	0,96	0	0	0	0	0	0,03	0,97	0	0,026

4,25	19545	0	0,04	0,01	0	0,94	0	0	0	0,01	0,06	0,04	0,9	0	0,042
4,25	2022	0,01	0,04	0	0	0,94	0	0,01	0	0	0	0,06	0,01	0,93	0,02
4,25	4276	0	0,04	0	0	0,96	0	0	0	0	0	0,04	0	0,96	0,014
4,25	1328	0	0,04	0	0	0,94	0	0	0,02	0	0	0,06	0	0,94	0,016
4,25	12475	0	0,04	0	0	0,95	0	0,01	0	0	0,42	0,04	0,01	0,53	0,028
4,25	17115	0,01	0,04	0	0	0,95	0	0	0	0	0	0,04	0,01	0,95	0,018
4,25	2531	0	0,04	0	0	0,95	0	0	0,01	0	0	0,05	0	0,95	0,008
4,25	29751	0	0,04	0	0	0,96	0	0	0	0	0	0,03	0	0,97	0,012
4,25	22759	0	0,04	0	0	0,96	0	0	0	0	0	0,04	0,96	0	0,026
4,25	7340	0	0,04	0	0	0,96	0	0	0	0	0	0,04	0	0,96	0,008
4,25	13450	0	0,04	0	0	0,96	0	0	0	0	0	0,04	0	0,96	0,008
4,25	3761	0	0,04	0	0	0,96	0	0	0	0	0	0,04	0,06	0,9	0,014
4,25	7655	0	0,04	0	0	0,94	0	0	0,02	0	0,15	0,04	0,02	0,79	0,012
4,25	3684	0	0,04	0	0	0,95	0	0	0,01	0	0,01	0,26	0,73	0	0,024
4,25	46011	0	0,04	0	0,01	0,94	0	0	0,01	0	0,02	0,03	0,01	0,94	0,028
4,25	13994	0	0,04	0	0	0,96	0	0	0	0	0	0,04	0	0,96	0,01
4,25	3761	0	0,04	0	0	0,91	0,03	0,02	0	0	0,91	0,08	0,01	0	0,046
4,25	16599	0	0,04	0	0	0,94	0	0,02	0	0	0,96	0,01	0,03	0	0,038
4,25	1558	0	0,04	0	0	0,95	0	0	0	0,01	0	0,78	0,02	0,2	0,014
4,25	13549	0	0,04	0	0,05	0,91	0	0	0	0	0,01	0,95	0,04	0	0,04
4,25	13876	0	0,03	0,01	0,08	0	0,87	0	0,01	0	0,05	0,26	0,69	0	0,048
4,25	11063	0	0,04	0	0	0,94	0	0,01	0	0,01	0,02	0,04	0,94	0	0,03
4,25	2159	0	0,04	0,01	0,01	0,92	0	0	0,02	0	0	0,04	0,93	0,03	0,042
4,25	38371	0	0,04	0	0,02	0,93	0	0,01	0	0	0,02	0,03	0,94	0,01	0,028
4,5	4054	0	0,04	0	0	0,96	0	0	0	0	0	0,16	0	0,84	0,016
4,5	11519	0,01	0,04	0,01	0	0,94	0	0	0	0	0	0,05	0,95	0	0,022
4,5	47408	0,01	0,04	0	0,01	0,94	0	0	0	0	0,2	0,03	0,75	0,02	0,042
4,5	4201	0	0,04	0	0	0,88	0,08	0	0	0	0,75	0,04	0,21	0	0,036
4,5	46660	0	0,04	0	0,02	0,94	0	0	0	0	0,95	0,04	0	0,01	0,036
4,5	2640	0	0,04	0	0	0,95	0	0	0,01	0	0	1	0	0	0,032
4,5	1660	0	0,04	0	0	0,96	0	0	0	0	0	0,04	0,96	0	0,032
4,5	28788	0,02	0,04	0	0,01	0,9	0,01	0	0,02	0	0,01	0,13	0,86	0	0,046
4,5	4366	0	0,04	0	0	0,96	0	0	0	0	0	0,04	0,96	0	0,038

4,5	30503	0	0,04	0	0	0,93	0	0	0,03	0	0	0,04	0,03	0,93	0,006
4,5	10988	0	0,04	0,01	0	0,95	0	0	0	0	0	0,03	0,97	0	0,022
4,5	6058	0,01	0,04	0,01	0	0,94	0	0	0	0	0	0,98	0,01	0,01	0,042
4,5	1849	0	0,04	0	0	0,96	0	0	0	0	0	0,04	0,96	0	0,022
4,5	4085	0	0,04	0	0	0,96	0	0	0	0	0	0,04	0	0,96	0,01
4,5	6053	0	0,04	0	0,01	0,95	0	0	0	0	0	0,04	0,96	0	0,03
4,5	12700	0	0,04	0	0,01	0,73	0,22	0	0	0	0,73	0,2	0,02	0,05	0,048
4,5	12661	0	0,04	0	0	0,96	0	0	0	0	0	0,04	0	0,96	0,006
4,5	3883	0	0,04	0	0	0,95	0	0,01	0	0	0,11	0,05	0	0,84	0,024
4,5	39699	0,01	0,04	0	0	0,95	0	0	0	0	0,95	0,05	0	0	0,046
4,5	2939	0,01	0,04	0	0	0,93	0	0,01	0,01	0	0,03	0,02	0,88	0,07	0,02
4,5	11092	0	0,04	0	0,08	0,88	0	0	0	0	0,11	0,85	0,01	0,03	0,046
4,5	18018	0,02	0,04	0	0	0,94	0	0	0	0	0,55	0,06	0	0,39	0,042
4,5	3672	0,01	0,04	0	0	0,95	0	0	0	0	0	0,01	0,03	0,96	0,022
4,5	9119	0	0,04	0	0,03	0,93	0	0	0	0	0	0,48	0	0,52	0,038
4,5	3116	0	0,04	0	0,02	0,92	0	0	0,01	0,01	0,03	0,05	0	0,92	0,03
4,5	37131	0	0,04	0	0	0,96	0	0	0	0	0	0,04	0	0,96	0,006
4,5	11288	0	0,04	0	0	0,96	0	0	0	0	0	0,04	0,96	0	0,024
4,5	42657	0	0,04	0	0,03	0,93	0	0	0	0	0,03	0	0,93	0,04	0,044
4,5	2246	0	0,04	0,01	0	0,95	0	0	0	0	0	0,12	0,88	0	0,03
4,5	14063	0,01	0,04	0	0	0,95	0	0	0	0	0,04	0	0,01	0,95	0,032
4,5	5580	0	0,04	0,02	0	0,94	0	0	0	0	0	0,06	0,93	0,01	0,042
4,5	13388	0,02	0,04	0	0	0,89	0,05	0	0	0	0	0,11	0,68	0,21	0,034
4,5	3498	0	0,04	0	0	0,96	0	0	0	0	0	0,04	0,96	0	0,018
4,5	5103	0	0,04	0	0	0,96	0	0	0	0	0	0,2	0,79	0,01	0,028
4,5	64960	0	0,04	0,01	0	0,95	0	0	0	0	0	0,04	0,95	0,01	0,034
4,5	53887	0	0,04	0	0	0,96	0	0	0	0	0	0,04	0	0,96	0,016
4,5	24825	0	0,04	0	0	0,94	0,02	0	0	0	0,89	0,04	0,07	0	0,032
4,5	32290	0	0,04	0	0,06	0,88	0	0	0,02	0	0,06	0	0,06	0,88	0,044
4,5	16573	0	0,04	0	0	0,96	0	0	0	0	0	0,04	0,44	0,52	0,02
4,5	26919	0	0,04	0	0	0,95	0	0	0	0,01	0,18	0,04	0,78	0	0,03
4,5	20286	0	0,04	0	0	0,96	0	0	0	0	0,03	0,04	0	0,93	0,008
4,5	61254	0,01	0,04	0	0	0,95	0	0	0	0	0,01	0,1	0,01	0,88	0,022

4,5	16933	0	0,04	0	0	0,95	0	0	0,01	0	0,97	0,01	0	0,02	0,038
4,5	4478	0	0,04	0	0	0,9	0,06	0	0	0	0	0,04	0,9	0,06	0,026
4,5	1318	0	0,01	0	0,1	0,84	0	0	0,05	0	0,05	0,01	0,81	0,13	0,044
4,5	9993	0,01	0,04	0,01	0	0,86	0	0	0,08	0	0	0,04	0,02	0,94	0,036
4,5	26913	0	0,04	0	0,01	0,92	0	0	0	0,03	0,01	0,05	0,94	0	0,024
4,5	19032	0	0,04	0,01	0	0,95	0	0	0	0	0,95	0,04	0,01	0	0,04
4,5	10457	0	0,04	0	0,01	0,95	0	0	0	0	0	0,04	0,95	0,01	0,04
4,5	2691	0	0,04	0	0	0,96	0	0	0	0	0	0,04	0	0,96	0,008
4,5	16017	0	0,04	0	0	0,95	0	0	0,01	0	0	0,96	0,04	0	0,022
4,5	2170	0	0,04	0	0,04	0,89	0	0	0,03	0	0,04	0,04	0,89	0,03	0,048
4,5	2301	0	0,04	0,01	0	0,95	0	0	0	0	0	0,05	0,85	0,1	0,03
4,5	6249	0,01	0,04	0	0	0,94	0	0	0,01	0	0,01	0	0	0,99	0,016
4,5	12393	0	0,04	0,02	0,06	0,88	0	0	0	0	0	0,12	0,84	0,04	0,044
4,5	29415	0	0,04	0	0	0,94	0,01	0	0,01	0	0,01	0	0,99	0	0,022
4,5	3698	0	0,04	0,04	0,01	0,91	0	0	0	0	0,01	0,07	0,91	0,01	0,046
4,5	2409	0	0,04	0	0	0,96	0	0	0	0	0	0,01	0,89	0,1	0,026
4,5	9519	0	0,04	0	0	0,96	0	0	0	0	0,96	0,04	0	0	0,032
4,5	4988	0,01	0,04	0	0	0,74	0,1	0	0,11	0	0,12	0,13	0,75	0	0,03
4,5	3185	0	0,04	0	0	0,95	0	0	0	0,01	0,01	0,04	0	0,95	0,014
4,5	4459	0	0,04	0,01	0	0,91	0,01	0	0,03	0	0	0,04	0	0,96	0,008
4,5	47435	0	0,04	0	0	0,96	0	0	0	0	0	0	0,96	0,04	0,028
4,5	22350	0	0,04	0	0	0,96	0	0	0	0	0	0,02	0,02	0,96	0
4,5	7892	0	0,04	0	0	0,96	0	0	0	0	0	1	0	0	0,034
4,5	7852	0	0,04	0	0	0,96	0	0	0	0	0	0,04	0,01	0,95	0,008
4,5	8532	0,01	0,04	0	0	0,94	0	0	0,01	0	0,01	0,04	0,95	0	0,03
4,5	3123	0,01	0,04	0	0,08	0,87	0	0	0	0	0,03	0,89	0,08	0	0,046
4,5	3269	0	0,04	0	0	0,95	0	0	0,01	0	0,44	0,42	0,04	0,1	0,032
4,5	6790	0	0,04	0	0	0,95	0,01	0	0	0	0	0,05	0	0,95	0,012
4,5	10509	0	0,04	0,04	0	0,89	0,01	0,02	0	0	0,03	0,04	0,91	0,02	0,046
4,5	13466	0	0,04	0	0	0,94	0,02	0	0	0	0,94	0,04	0,02	0	0,034
4,5	16630	0,01	0,03	0	0,07	0,89	0	0	0	0	0,02	0,03	0,89	0,06	0,04
4,5	22975	0	0,04	0	0,01	0,94	0	0	0	0,01	0	0,04	0,95	0,01	0,024
4,5	6705	0	0,04	0	0	0,96	0	0	0	0	0	0,04	0,96	0	0,02

4,5	8650	0	0,04	0	0	0,96	0	0	0	0	0	0,04	0,03	0,93	0,01
4,5	16579	0,01	0,04	0	0	0,93	0,01	0	0,01	0	0	0,04	0,96	0	0,028
4,5	2648	0,01	0,04	0,01	0	0,93	0	0,01	0	0	0,91	0,05	0	0,04	0,048
4,5	20442	0	0,04	0	0	0,94	0,02	0	0	0	0,62	0,05	0	0,33	0,034
4,5	9212	0	0,04	0	0	0,91	0	0	0,05	0	0,01	0,08	0	0,91	0,006
4,5	7200	0	0,04	0,01	0	0,95	0	0	0	0	0	0,05	0,95	0	0,04
4,5	22540	0,01	0,04	0	0	0,95	0	0	0	0	0	0,04	0,95	0,01	0,04
4,5	15010	0	0,04	0	0	0,94	0,02	0	0	0	0,68	0,32	0	0	0,032
4,5	10196	0	0,04	0	0,01	0,92	0	0	0	0,03	0,73	0,1	0,17	0	0,048
4,5	10962	0,01	0	0	0,13	0,01	0	0	0,85	0	0,17	0,66	0,17	0	0,046
4,5	50435	0	0,04	0	0	0,95	0	0,01	0	0	0,44	0,51	0,01	0,04	0,032
4,5	40585	0	0,04	0	0,01	0,94	0	0,01	0	0	0,21	0,04	0,01	0,74	0,036
4,5	17547	0	0,04	0	0	0,92	0	0	0,04	0	0	0,01	0,99	0	0,016
4,5	20152	0	0,04	0	0,03	0,93	0	0	0	0	0	0,07	0,71	0,22	0,028
4,5	4582	0	0,04	0	0	0,94	0	0	0,01	0,01	0	0,98	0	0,02	0,022
4,5	18686	0	0,04	0	0	0,96	0	0	0	0	0	0,04	0	0,96	0,006
4,5	5070	0	0,04	0,01	0	0,95	0	0	0	0	0	0,04	0,01	0,95	0,008
4,5	2047	0	0,04	0	0	0,96	0	0	0	0	0	0,05	0	0,95	0,01
4,5	20039	0	0,04	0	0,03	0,93	0	0	0	0	0,04	0	0,94	0,02	0,046
4,5	9599	0	0,04	0	0,01	0,95	0	0	0	0	0	0,04	0	0,96	0,02
4,5	8488	0,01	0,04	0	0	0,95	0	0	0	0	0	0,05	0,95	0	0,042
4,5	7522	0	0,04	0,01	0	0,87	0,02	0	0,06	0	0,06	0,07	0,85	0,02	0,02
4,5	11237	0	0,04	0	0	0,96	0	0	0	0	0,17	0,79	0	0,04	0,026
4,5	15707	0	0,04	0	0	0,96	0	0	0	0	0	0,05	0,01	0,94	0,01
4,5	32986	0	0,04	0	0	0,95	0,01	0	0	0	0	0,96	0,04	0	0,026
4,75	10005	0,02	0,04	0,02	0	0,89	0	0	0	0,03	0,01	0,07	0,03	0,89	0,04
4,75	28322	0	0,04	0	0	0,95	0	0,01	0	0	0,23	0,04	0,73	0	0,034
4,75	25629	0	0,04	0,01	0	0,95	0	0	0	0	0,39	0,61	0	0	0,044
4,75	35878	0	0,04	0	0	0,95	0	0	0	0,01	0	0,05	0,95	0	0,036
4,75	42619	0	0,04	0	0,01	0,95	0	0	0	0	0,96	0,04	0	0	0,04
4,75	20665	0	0,04	0	0	0,96	0	0	0	0	0	1	0	0	0,024
4,75	39842	0	0,04	0,01	0	0,94	0	0	0	0,01	0	0,04	0,01	0,95	0,004
4,75	38143	0	0,04	0	0	0,89	0,01	0,01	0,04	0,01	0,05	0,79	0,15	0,01	0,034

4,75	12786	0	0,04	0,01	0	0,95	0	0	0	0	0,01	0,68	0	0,31	0,032
4,75	14673	0	0,04	0	0	0,95	0	0	0	0,01	0	0,03	0,96	0,01	0,04
4,75	19813	0	0,04	0	0	0,95	0	0	0,01	0	0	0,04	0,96	0	0,026
4,75	3509	0	0,04	0	0	0,95	0,01	0	0	0	0	0,04	0,81	0,15	0,022
4,75	16673	0	0,04	0	0	0,95	0,01	0	0	0	0	0,04	0,84	0,12	0,028
4,75	13248	0	0,04	0	0,02	0,92	0,02	0	0	0	0,02	0,06	0,92	0	0,032
4,75	49863	0	0,04	0,01	0	0,92	0,01	0	0	0,02	0,02	0,06	0,92	0	0,02
4,75	19334	0	0,04	0,01	0,01	0,94	0	0	0	0	0	0,04	0	0,96	0,024
4,75	1916	0	0,04	0	0	0,96	0	0	0	0	0	0,03	0,96	0,01	0,02
4,75	24645	0	0,04	0	0	0,96	0	0	0	0	0	0,04	0,91	0,05	0,026
4,75	4744	0	0,04	0	0	0,96	0	0	0	0	0,02	0,02	0	0,96	0,01
4,75	16794	0	0,03	0	0	0,81	0,12	0,02	0	0,02	0,94	0,04	0	0,02	0,036
4,75	13804	0	0,04	0	0,01	0,95	0	0	0	0	0,01	0,03	0,01	0,95	0,02
4,75	5074	0	0,04	0,01	0,02	0,93	0	0	0	0	0,93	0,06	0	0,01	0,04
4,75	5499	0	0,04	0	0	0,95	0	0	0	0,01	0	0,04	0,95	0,01	0,03
4,75	1632	0	0,04	0	0	0,95	0	0	0,01	0	0	0,99	0,01	0	0,026
4,75	6312	0	0,04	0	0	0,96	0	0	0	0	0,03	0,96	0	0,01	0,032
4,75	3582	0	0,04	0	0,05	0,88	0	0	0,03	0	0,05	0,07	0,88	0	0,046
4,75	4901	0	0,04	0,02	0	0,92	0	0,02	0	0	0,02	0,96	0,02	0	0,036
4,75	6284	0	0,04	0	0	0,96	0	0	0	0	0	0,44	0,01	0,55	0,012
4,75	29786	0	0,04	0	0	0,96	0	0	0	0	0	1	0	0	0,026
4,75	3551	0	0,04	0	0	0,96	0	0	0	0	0	0,04	0	0,96	0,002
4,75	35653	0	0,04	0	0	0,87	0,06	0	0,02	0,01	0,94	0,04	0,02	0	0,036
4,75	17490	0	0,04	0	0	0,96	0	0	0	0	0	0,04	0,96	0	0,024
4,75	11311	0	0,04	0	0	0,96	0	0	0	0	0	0,04	0	0,96	0,006
4,75	31938	0	0,04	0	0	0,96	0	0	0	0	0	0,12	0,88	0	0,03
4,75	3515	0	0,04	0	0	0,95	0	0	0	0,01	0,01	0,04	0,69	0,26	0,026
4,75	2165	0	0,04	0	0	0,9	0	0	0,06	0	0	0,03	0,07	0,9	0,008
4,75	9313	0,01	0,04	0	0	0,92	0,01	0,02	0	0	0,01	0,02	0,94	0,03	0,044
4,75	9353	0,01	0,04	0	0	0,95	0	0	0	0	0	0,02	0,96	0,02	0,024
4,75	2794	0	0,04	0	0	0,94	0	0,01	0	0,01	0	0,04	0,96	0	0,024
4,75	42473	0	0,04	0	0,01	0,94	0	0	0	0,01	0,01	0,03	0,94	0,02	0,034
4,75	15544	0	0,04	0	0,02	0,91	0	0,03	0	0	0,02	0,04	0,91	0,03	0,04

4,75	16104	0	0,04	0	0	0,96	0	0	0	0	0	0,66	0	0,34	0,018
4,75	17760	0	0,04	0	0	0,86	0	0	0,1	0	0,11	0,03	0	0,86	0,006
4,75	24139	0	0,04	0	0	0,96	0	0	0	0	0,01	0,99	0	0	0,026
4,75	7366	0	0,04	0	0	0,96	0	0	0	0	0,01	0,04	0,95	0	0,034
4,75	6842	0	0,04	0	0	0,95	0	0	0,01	0	0,87	0	0,01	0,12	0,034
4,75	9590	0	0,04	0,01	0	0,95	0	0	0	0	0	0,95	0,01	0,04	0,028
4,75	3889	0,03	0,04	0	0	0,87	0	0	0,05	0,01	0,03	0,91	0	0,06	0,04
4,75	9578	0	0,04	0	0	0,95	0	0	0	0,01	0	0,05	0	0,95	0,012
4,75	64445	0,01	0	0	0,07	0,64	0	0	0,28	0	0,07	0,5	0,15	0,28	0,03
4,75	3872	0	0,04	0	0	0,95	0,01	0	0	0	0	0,04	0,95	0,01	0,022
4,75	17908	0	0,04	0	0	0,96	0	0	0	0	0	0,04	0	0,96	0,02
4,75	49401	0,03	0,04	0,02	0	0,89	0,02	0	0	0	0,06	0,03	0,91	0	0,042
4,75	41692	0	0,04	0	0	0,96	0	0	0	0	0	0,01	0,99	0	0,026
4,75	23473	0	0,04	0	0	0,96	0	0	0	0	0	0,05	0,95	0	0,02
4,75	17774	0,01	0,03	0	0,13	0,83	0	0	0	0	0,83	0	0,16	0,01	0,044
4,75	29322	0	0	0	0,48	0,52	0	0	0	0	1	0	0	0	0,046
4,75	17481	0	0,04	0	0,05	0,88	0,03	0	0	0	0,06	0,03	0,91	0	0,046
4,75	11490	0	0	0	0,14	0,86	0	0	0	0	0,14	0,84	0	0,02	0,044
4,75	9051	0	0,04	0	0	0,63	0	0	0,33	0	0	0,33	0,04	0,63	0,016
4,75	10301	0	0,04	0	0	0,96	0	0	0	0	0	0,04	0,96	0	0,024
4,75	23013	0	0,04	0	0	0,96	0	0	0	0	0	0,04	0,96	0	0,028
4,75	5333	0	0,04	0	0,01	0,88	0,05	0,02	0	0	0,02	0,09	0,89	0	0,034
4,75	24892	0	0,04	0	0	0,96	0	0	0	0	0,54	0,46	0	0	0,036
4,75	7253	0,01	0,04	0	0	0,83	0,11	0	0	0,01	0,12	0,04	0,81	0,03	0,026
4,75	22348	0	0,04	0	0	0,96	0	0	0	0	0,04	0	0	0,96	0,004
4,75	14252	0	0,04	0	0	0,93	0,03	0	0	0	0,21	0,04	0,75	0	0,024
4,75	19602	0	0,04	0	0	0,93	0	0	0,03	0	0	0,04	0,03	0,93	0,004
4,75	4430	0	0,04	0	0	0,96	0	0	0	0	0	0,03	0,96	0,01	0,022
4,75	2822	0	0,04	0	0,05	0,91	0	0	0	0	0	0,11	0,89	0	0,048
4,75	30624	0	0,04	0	0	0,96	0	0	0	0	0,8	0,04	0	0,16	0,026
4,75	37914	0,01	0,04	0	0,01	0,94	0	0	0	0	0,04	0,01	0,95	0	0,028
4,75	14024	0	0,02	0,04	0,05	0,89	0	0	0	0	0	0,07	0,93	0	0,048
4,75	3864	0,01	0,04	0	0	0,95	0	0	0	0	0,01	0,04	0	0,95	0,02

4,75	10831	0	0,04	0,01	0	0,95	0	0	0	0	0	0,04	0	0,96	0,018
4,75	41616	0	0,04	0	0	0,96	0	0	0	0	0	0,36	0,05	0,59	0,016
4,75	6567	0	0,04	0	0	0,96	0	0	0	0	0	0,04	0	0,96	0,01
4,75	14970	0	0,04	0	0	0,96	0	0	0	0	0	0,96	0	0,04	0,03
4,75	5655	0,01	0,04	0	0	0,95	0	0	0	0	0,01	0,91	0	0,08	0,026
4,75	7569	0	0,04	0	0	0,95	0,01	0	0	0	0	0	0	1	0,01
4,75	28678	0	0,04	0	0	0,95	0	0	0	0,01	0,19	0,04	0	0,77	0,008
4,75	2655	0	0,04	0	0	0,94	0,02	0	0	0	0	0,04	0,96	0	0,026
4,75	32549	0	0,04	0	0	0,93	0,02	0,01	0	0	0,94	0,05	0,01	0	0,036
4,75	19618	0	0,04	0	0	0,96	0	0	0	0	0	0,04	0	0,96	0,008
4,75	2566	0	0,04	0	0	0,96	0	0	0	0	0,02	0,02	0	0,96	0,01
4,75	8049	0	0,04	0	0	0,96	0	0	0	0	0,01	0,27	0,72	0	0,026
4,75	26093	0	0,04	0	0	0,94	0	0	0,02	0	0	0,05	0	0,95	0,01
4,75	1847	0,01	0,04	0	0	0,95	0	0	0	0	0,02	0,19	0,79	0	0,024
4,75	5068	0	0,04	0	0	0,85	0,01	0	0,1	0	0,01	0,04	0	0,95	0,004
4,75	18629	0	0,04	0	0	0,96	0	0	0	0	0	0	0,04	0,96	0,012
4,75	24325	0	0,04	0	0	0,93	0	0,01	0	0,02	0,01	0,02	0,93	0,04	0,046
4,75	8309	0	0,04	0	0	0,94	0	0	0,01	0,01	0,01	0,05	0,94	0	0,024
4,75	6237	0	0,04	0	0	0,96	0	0	0	0	0,02	0,02	0	0,96	0,004
4,75	22507	0	0,04	0	0	0,96	0	0	0	0	0	0,05	0,01	0,94	0,012
4,75	44076	0	0,04	0	0	0,96	0	0	0	0	0,01	0,51	0	0,48	0,018
4,75	7909	0	0,04	0	0	0,94	0	0	0,01	0,01	0	0,06	0	0,94	0,02
4,75	4012	0	0,04	0	0,01	0,94	0,01	0	0	0	0,94	0,04	0,01	0,01	0,046
4,75	5050	0	0,04	0	0	0,94	0	0,02	0	0	0	0,04	0,95	0,01	0,022
4,75	16634	0	0	0	0,13	0,86	0	0,01	0	0	0,01	0,13	0,86	0	0,048
4,75	14192	0	0,04	0	0,02	0,94	0	0	0	0	0	0,04	0,94	0,02	0,032
5	21568	0	0,04	0	0	0,93	0	0	0	0,03	0	0,04	0,93	0,03	0,018
5	2001	0	0,04	0	0	0,96	0	0	0	0	0	0,93	0,01	0,06	0,022
5	3018	0	0,04	0	0	0,94	0,02	0	0	0	0	0,04	0,96	0	0,028
5	31894	0	0,04	0	0	0,96	0	0	0	0	0	0,04	0	0,96	0,002
5	17185	0	0,04	0	0	0,95	0,01	0	0	0	0,01	0,04	0	0,95	0,006
5	9193	0	0,04	0	0	0,94	0	0	0,01	0,01	0,01	0,98	0	0,01	0,02
5	36975	0	0,04	0	0,02	0,94	0	0	0	0	0	0,83	0	0,17	0,04

5	5120	0	0,04	0	0	0,96	0	0	0	0	0,97	0,02	0	0,01	0,038
5	12467	0,01	0,03	0,02	0,05	0,89	0	0	0	0	0,01	0,92	0,07	0	0,048
5	5616	0	0,04	0	0,01	0,94	0	0	0	0,01	0,01	0,05	0,01	0,93	0,016
5	7209	0	0,04	0	0	0,96	0	0	0	0	0	0,04	0	0,96	0,014
5	41744	0	0,04	0	0	0,96	0	0	0	0	0,11	0,04	0	0,85	0,008
5	14075	0	0	0,01	0,16	0,11	0,71	0	0	0,01	0,67	0	0,16	0,17	0,046
5	63648	0	0,04	0,01	0	0,95	0	0	0	0	0	0,92	0,01	0,07	0,026
5	3138	0	0,04	0,02	0	0,94	0	0	0	0	0	0,04	0,96	0	0,036
5	58082	0	0,04	0	0,01	0,94	0	0	0,01	0	0,36	0	0,01	0,63	0,022
5	31469	0	0,04	0,01	0	0,95	0	0	0	0	0,01	0,99	0	0	0,022
5	20869	0	0,04	0	0	0,88	0	0	0,08	0	0	0,04	0,72	0,24	0,024
5	5173	0	0,04	0,01	0	0,94	0,01	0	0	0	0	0,05	0,8	0,15	0,03
5	19571	0	0,04	0	0	0,96	0	0	0	0	0,28	0,04	0	0,68	0,01
5	2800	0	0,04	0	0	0,96	0	0	0	0	0	0,04	0	0,96	0,012
5	19740	0	0,04	0	0,02	0,94	0	0	0	0	0	0,01	0,05	0,94	0,028
5	2088	0	0,04	0,01	0	0,95	0	0	0	0	0	0,04	0	0,96	0,014
5	2528	0	0,04	0	0	0,95	0	0	0,01	0	0,01	0,86	0,13	0	0,028
5	2586	0	0,04	0	0	0,96	0	0	0	0	0	0,04	0,96	0	0,02
5	25804	0	0,04	0	0	0,95	0	0	0,01	0	0	0,05	0	0,95	0,004
5	25782	0	0,04	0	0	0,96	0	0	0	0	0	0,05	0,95	0	0,018
5	43266	0	0,04	0,01	0	0,89	0,03	0	0	0,03	0,03	0,07	0,01	0,89	0,02
5	15677	0	0,04	0	0	0,95	0,01	0	0	0	0,02	0,05	0,92	0,01	0,02
5	13039	0	0,04	0	0	0,94	0	0	0,01	0,01	0	0,05	0,95	0	0,034
5	3098	0	0,03	0	0,25	0,72	0	0	0	0	0,92	0,03	0	0,05	0,044
5	21157	0	0,04	0	0	0,96	0	0	0	0	0	0,04	0	0,96	0,008
5	6718	0,03	0	0	0,1	0,23	0	0	0,53	0,11	0,53	0,32	0,01	0,14	0,048
5	3419	0	0,04	0	0,01	0,95	0	0	0	0	0	0,03	0,95	0,02	0,022
5	6511	0	0,04	0	0,01	0,89	0	0	0,06	0	0,02	0,03	0,95	0	0,032
5	9549	0	0,04	0,02	0	0,94	0	0	0	0	0	0,04	0,96	0	0,028
5	2972	0	0,04	0,01	0	0,81	0,01	0,01	0,12	0	0,02	0,04	0,12	0,82	0,018
5	40793	0	0,04	0	0	0,96	0	0	0	0	0	0	0,01	0,99	0,01
5	6538	0	0,04	0	0	0,96	0	0	0	0	0	0,04	0,96	0	0,03
5	19662	0	0,04	0	0	0,94	0,01	0	0,01	0	0	0,55	0,44	0,01	0,028

5	36679	0	0,04	0	0	0,95	0,01	0	0	0	0,01	0,04	0	0,95	0,01
5	10198	0	0,04	0	0	0,95	0,01	0	0	0	0,03	0	0,97	0	0,026
5	16626	0	0,04	0,02	0	0,91	0	0	0,03	0	0,01	0,92	0,03	0,04	0,03
5	11497	0	0	0	0,23	0,77	0	0	0	0	0,82	0,01	0,17	0	0,032
5	23110	0	0,04	0	0,01	0,94	0	0	0	0,01	0,01	0,05	0,94	0	0,028
5	40281	0	0,04	0	0	0,94	0	0	0,01	0,01	0,45	0,04	0,51	0	0,03
5	14717	0	0,04	0	0	0,95	0,01	0	0	0	0	0,31	0,69	0	0,022
5	12588	0	0,04	0	0	0,94	0,02	0	0	0	0	0,05	0,94	0,01	0,03
5	2639	0	0,04	0	0	0,96	0	0	0	0	0	0,04	0,01	0,95	0,01
5	38440	0,01	0,04	0	0	0,93	0	0,02	0	0	0,01	0,03	0,02	0,94	0,038
5	12734	0	0,04	0	0	0,96	0	0	0	0	0	0,04	0	0,96	0,012
5	32818	0	0,04	0	0	0,94	0	0	0	0,02	0,53	0,02	0,04	0,41	0,028
5	2538	0	0,04	0	0	0,96	0	0	0	0	0	0,07	0,93	0	0,03
5	33631	0,01	0,04	0	0,01	0,94	0	0	0	0	0	0,99	0	0,01	0,03
5	2771	0	0,04	0	0	0,94	0	0	0	0,02	0,64	0,34	0	0,02	0,038
5	5621	0	0,04	0	0	0,96	0	0	0	0	0	0,05	0	0,95	0,01
5	11665	0	0,04	0	0,01	0,95	0	0	0	0	0,05	0,04	0,01	0,9	0,02
5	17482	0	0,04	0	0,01	0,95	0	0	0	0	0,48	0,52	0	0	0,03
5	22810	0,01	0,03	0	0,1	0,8	0,02	0	0,03	0,01	0,11	0,03	0,81	0,05	0,046
5	9534	0	0,04	0	0,01	0,95	0	0	0	0	0,01	0,04	0,95	0	0,032
5	13473	0	0,04	0	0	0,95	0	0,01	0	0	0	0,04	0,95	0,01	0,024
5	2153	0	0,04	0	0	0,96	0	0	0	0	0	0,04	0,96	0	0,018
5	7448	0	0,04	0	0	0,96	0	0	0	0	0	0,04	0	0,96	0,012
5	5708	0	0,04	0	0	0,96	0	0	0	0	0	0,04	0	0,96	0,01
5	30957	0,01	0,04	0	0	0,94	0	0,01	0	0	0,02	0,04	0,94	0	0,04
5	8162	0	0,04	0	0	0,87	0	0	0,08	0,01	0,01	0,12	0,87	0	0,024
5	34434	0	0,04	0	0,01	0,92	0	0	0,03	0	0,93	0,03	0	0,04	0,034
5	2413	0,01	0,04	0	0	0,85	0,01	0	0,09	0	0,06	0,06	0,86	0,02	0,048
5	18379	0,01	0,04	0	0	0,92	0,01	0	0,02	0	0	0,02	0,04	0,94	0,016
5	3843	0	0,04	0	0	0,96	0	0	0	0	0,02	0,02	0	0,96	0,018
5	5726	0	0,04	0,01	0	0,88	0,07	0	0	0	0,07	0,04	0,88	0,01	0,02
5	38606	0	0,04	0	0	0,96	0	0	0	0	0	0,04	0	0,96	0,014
5	23199	0	0,04	0	0	0,96	0	0	0	0	0	0,04	0	0,96	0,014

5	3810	0	0,04	0	0	0,94	0,01	0	0,01	0	0	0,05	0,95	0	0,016
5	8945	0	0,04	0	0,01	0,93	0	0	0	0,02	0	0,04	0,93	0,03	0,024
5	8153	0	0,04	0	0	0,88	0	0	0,08	0	0	0,01	0,96	0,03	0,024
5	18964	0	0,04	0	0	0,96	0	0	0	0	0	0	0,04	0,96	0,018
5	30856	0	0,04	0	0	0,93	0,02	0	0,01	0	0	0,05	0	0,95	0,012
5	41240	0	0,01	0	0,14	0,75	0,03	0	0,07	0	0,2	0,03	0,76	0,01	0,044
5	5629	0	0,04	0	0	0,96	0	0	0	0	0	0,99	0	0,01	0,018
5	10670	0	0	0	0,15	0,85	0	0	0	0	0,14	0	0,86	0	0,046
5	21775	0	0,04	0	0	0,94	0	0	0,01	0,01	0,02	0,04	0,94	0	0,014
5	21974	0	0,04	0,01	0	0,94	0	0	0,01	0	0,02	0,04	0,94	0	0,026
5	15884	0	0,04	0	0	0,95	0,01	0	0	0	0,71	0,28	0	0,01	0,04
5	10134	0	0,04	0	0	0,94	0	0	0,02	0	0,03	0	0,03	0,94	0,008
5	11816	0	0,04	0	0	0,95	0	0	0,01	0	0	0,04	0	0,96	0
5	5485	0	0,04	0	0	0,94	0,02	0	0	0	0	0,04	0,02	0,94	0,008
5	23010	0	0,04	0	0	0,96	0	0	0	0	0	0,04	0	0,96	0,006
5	3511	0	0,04	0	0	0,96	0	0	0	0	0	0,04	0,96	0	0,022
5	11567	0	0,04	0	0	0,95	0	0	0,01	0	0	0,04	0,02	0,94	0,006
5	16142	0	0	0,01	0,12	0	0,06	0	0,58	0,23	0,24	0,76	0	0	0,038
5	5970	0	0,04	0	0	0,96	0	0	0	0	0	0,04	0	0,96	0,008
5	3926	0	0,04	0,01	0	0,95	0	0	0	0	0	0,96	0,01	0,03	0,018
5	3094	0	0,04	0	0	0,95	0	0,01	0	0	0	0,04	0,95	0,01	0,024
5	78684	0	0,04	0	0	0,96	0	0	0	0	0	0,04	0	0,96	0,014
5	4632	0	0,04	0	0	0,92	0	0	0,02	0,02	0,29	0,71	0	0	0,04
5	14277	0,01	0,04	0	0	0,95	0	0	0	0	0	0,04	0	0,96	0,014
5	12199	0	0,04	0	0	0,96	0	0	0	0	0,04	0	0,05	0,91	0,026
5	23064	0	0,03	0	0,1	0,86	0	0,01	0	0	0,09	0,03	0,87	0,01	0,046
5	12871	0	0,04	0	0	0,96	0	0	0	0	0,02	0,04	0	0,94	0,008
	10058,27	0,00167	0,03812	0,00193	0,01349	0,91789	0,00989	0,00158	0,01372	0,00169	0,1762	0,20484	0,30712	0,31185	0,026989
simple-standing															
b	time_to_cc	(C,C)	(C,D)	(C,P)	(D,C)	(D,D)	(D,P)	(P,C)	(P,D)	(P,P)	S(G,G)	S(G,B)	S(B,G)	S(B,B)	
1	2167	0	0,04	0	0	0,95	0	0,01	0	0	0,01	0,04	0	0,95	0,02
1	1292	0	0,04	0	0,01	0,95	0	0	0	0	0,95	0,04	0	0,01	0,048
1	4178	0	0,04	0	0	0,96	0	0	0	0	0,96	0,04	0	0	0,042

1	6573	0	0	0	0,13	0,86	0	0	0	0,01	0,13	0	0,87	0	0,038
1	3166	0	0,04	0	0	0,96	0	0	0	0	0,01	0,03	0,96	0	0,02
1	10636	0	0,04	0	0,01	0,95	0	0	0	0	0	0,99	0,01	0	0,04
1	2095	0	0,04	0	0	0,94	0	0	0,02	0	0	0,98	0	0,02	0,024
1	12388	0	0,04	0	0,11	0,85	0	0	0	0	0,01	0,99	0	0	0,046
1	1418	0	0,04	0	0	0,94	0	0,01	0	0,01	0,01	0,04	0	0,95	0,026
1	5595	0,01	0,03	0,01	0,15	0,8	0	0	0	0	0,8	0,04	0,16	0	0,048
1	4756	0	0,04	0	0,05	0,91	0	0	0	0	0	0,95	0	0,05	0,038
1	2289	0,01	0,04	0	0	0,95	0	0	0	0	0,01	0,04	0,11	0,84	0,028
1	10625	0	0	0	0,3	0,64	0,03	0,03	0	0	0,64	0,33	0	0,03	0,048
1	3678	0	0	0	0,28	0,7	0	0	0,01	0,01	0,7	0,29	0	0,01	0,048
1	6479	0	0,04	0	0	0,96	0	0	0	0	0	0,04	0	0,96	0,006
1	2442	0	0,04	0	0	0,94	0,01	0	0,01	0	0,01	0,04	0,95	0	0,032
1	3116	0,01	0,04	0	0	0,95	0	0	0	0	0	0,03	0,01	0,96	0,026
1	9351	0	0	0	0,1	0,9	0	0	0	0	0,07	0	0,2	0,73	0,046
1	2401	0	0,04	0,01	0	0,95	0	0	0	0	0,01	0,99	0	0	0,032
1	4872	0,01	0,04	0	0	0,93	0	0	0	0,02	0	0,07	0,31	0,62	0,028
1	16998	0,01	0	0	0,11	0,86	0,01	0,01	0	0	0,14	0,85	0,01	0	0,04
1	10330	0	0	0	0,13	0,87	0	0	0	0	0,13	0,67	0,14	0,06	0,042
1	2467	0	0,04	0	0	0,96	0	0	0	0	0,98	0,02	0	0	0,044
1	6540	0	0,04	0,01	0,08	0,87	0	0	0	0	0	0,98	0,01	0,01	0,044
1	9574	0	0	0	0,23	0,77	0	0	0	0	0,77	0	0,23	0	0,048
1	8626	0,01	0,04	0	0	0,94	0	0	0,01	0	0	0,01	0,98	0,01	0,038
1	1317	0	0,04	0	0	0,95	0	0,01	0	0	0,28	0,72	0	0	0,034
1	10020	0	0	0	0,19	0,8	0,01	0	0	0	0,29	0,71	0	0	0,046
1	6244	0	0,04	0,01	0	0,94	0,01	0	0	0	0,01	0,99	0	0	0,032
1	10231	0	0,04	0	0	0,84	0,11	0	0	0,01	0,84	0,16	0	0	0,026
1	11082	0	0,04	0	0	0,96	0	0	0	0	0	0,04	0	0,96	0,01
1	3172	0	0,04	0	0	0,96	0	0	0	0	0	0,04	0	0,96	0,002
1	5399	0	0,04	0	0,01	0,95	0	0	0	0	0	0,04	0,8	0,16	0,024
1	2122	0	0,04	0	0	0,96	0	0	0	0	0	1	0	0	0,026
1	7124	0	0,04	0	0	0,95	0	0	0	0,01	0	0,05	0	0,95	0,006
1	10672	0	0	0	0,15	0,84	0	0	0	0,01	0,12	0	0,85	0,03	0,04

1	10253	0	0,04	0	0,01	0,91	0,04	0	0	0	0,92	0,08	0	0	0,04
1	8897	0	0,04	0	0	0,96	0	0	0	0	0,95	0,03	0,01	0,01	0,044
1	4003	0	0,04	0	0,02	0,93	0	0	0,01	0	0	0,94	0,03	0,03	0,046
1	4348	0	0,04	0	0	0,94	0,01	0	0,01	0	0,83	0,03	0,12	0,02	0,046
1	1762	0	0,04	0	0	0,96	0	0	0	0	0,96	0,04	0	0	0,04
1	12272	0	0	0	0,26	0,58	0,11	0,05	0	0	0,83	0,11	0	0,06	0,048
1	2026	0	0,04	0	0	0,95	0	0,01	0	0	0,92	0,07	0	0,01	0,044
1	14290	0	0,04	0	0	0,96	0	0	0	0	0,96	0,04	0	0	0,048
1	5994	0	0	0	0,1	0,9	0	0	0	0	0	0	0,96	0,04	0,032
1	3894	0	0,04	0	0,08	0,87	0,01	0	0	0	0	0,91	0	0,09	0,046
1	5043	0	0,04	0	0	0,95	0	0	0	0,01	0	0,04	0,96	0	0,034
1	19408	0,01	0	0,01	0,06	0,88	0,01	0,02	0,01	0	0,07	0,04	0,88	0,01	0,048
1	4420	0	0,04	0	0	0,92	0	0,01	0	0,03	0,13	0,05	0,81	0,01	0,026
1	7760	0	0	0	0,13	0,87	0	0	0	0	0	1	0	0	0,048
1	1805	0	0,04	0	0,1	0,86	0	0	0	0	0	0,04	0,87	0,09	0,042
1	2936	0,02	0,04	0	0,02	0,92	0	0	0	0	0,01	0,07	0,92	0	0,046
1	1746	0	0,04	0	0	0,96	0	0	0	0	0	0,04	0,89	0,07	0,02
1	1242	0	0,04	0,01	0	0,95	0	0	0	0	0,96	0,04	0	0	0,032
1	2784	0,01	0,04	0	0	0,95	0	0	0	0	0,96	0,03	0,01	0	0,042
1	5994	0	0,04	0	0	0,96	0	0	0	0	0	1	0	0	0,026
1	2510	0	0,04	0	0	0,96	0	0	0	0	0	0,04	0,96	0	0,038
1	4431	0	0,04	0	0	0,96	0	0	0	0	0,96	0,03	0,01	0	0,04
1	1778	0	0,04	0	0	0,95	0,01	0	0	0	0	0,06	0,94	0	0,022
1	13155	0	0	0	0,12	0,88	0	0	0	0	0	0,88	0,12	0	0,048
1	2545	0	0,04	0	0	0,96	0	0	0	0	0	1	0	0	0,022
1	7435	0	0,04	0	0	0,94	0	0,01	0	0,01	0	1	0	0	0,026
1	8022	0	0,04	0	0	0,94	0,01	0	0	0,01	0,01	0,05	0	0,94	0,02
1	17727	0	0,04	0	0	0,95	0	0	0	0,01	0	0,05	0,01	0,94	0,012
1	6527	0	0,03	0	0,15	0,74	0,03	0,05	0	0	0,75	0,16	0	0,09	0,044
1	2540	0	0,04	0,01	0	0,92	0	0	0,03	0	0,03	0,05	0,92	0	0,028
1	14082	0	0,04	0	0	0,96	0	0	0	0	0,04	0,04	0,13	0,79	0,01
1	5498	0	0,04	0	0	0,91	0,01	0	0,03	0,01	0,02	0,05	0,92	0,01	0,022
1	1922	0	0,04	0	0	0,96	0	0	0	0	0	0,03	0,01	0,96	0,01

1	3117	0,01	0	0	0,12	0,84	0,03	0	0	0	0,16	0	0,84	0	0,04
1	2431	0	0,04	0,01	0	0,95	0	0	0	0	0	0,04	0,95	0,01	0,03
1	13307	0	0	0	0,15	0,85	0	0	0	0	0,15	0,85	0	0	0,046
1	1430	0	0,04	0	0	0,96	0	0	0	0	0	0,04	0,88	0,08	0,022
1	17868	0	0	0	0,22	0,78	0	0	0	0	0,78	0	0,22	0	0,046
1	18764	0	0,04	0	0,02	0,93	0,01	0	0	0	0	0,06	0	0,94	0,03
1	6734	0	0,04	0	0,01	0,93	0	0	0,02	0	0,06	0	0,01	0,93	0,02
1	8356	0	0,04	0	0	0,96	0	0	0	0	0	0,04	0	0,96	0,006
1	7117	0	0	0	0,17	0,83	0	0	0	0	0,17	0,83	0	0	0,042
1	4030	0	0,04	0	0,01	0,86	0,09	0	0	0	0,94	0,04	0	0,02	0,034
1	6298	0	0,04	0	0	0,95	0	0	0,01	0	0	0,04	0,01	0,95	0,01
1	9619	0	0	0	0,07	0	0,15	0,01	0,77	0	0,04	0,84	0,01	0,11	0,044
1	5273	0	0,04	0	0	0,96	0	0	0	0	0,01	0,03	0	0,96	0,014
1	6372	0	0,04	0,01	0	0,95	0	0	0	0	0,38	0,05	0	0,57	0,032
1	3792	0,01	0,04	0,01	0	0,94	0	0	0	0	0	0,05	0	0,95	0,016
1	1275	0	0,04	0	0,01	0,95	0	0	0	0	0	0,04	0,96	0	0,024
1	7412	0	0,04	0	0	0,95	0	0	0,01	0	0,02	0,03	0	0,95	0,012
1	3115	0	0,04	0	0	0,91	0	0	0,04	0,01	0,04	0,05	0	0,91	0,01
1	19625	0	0,04	0	0	0,96	0	0	0	0	0,17	0,04	0	0,79	0,012
1	9784	0	0,04	0	0	0,86	0,1	0	0	0	0	1	0	0	0,026
1	1756	0	0,04	0,02	0	0,93	0	0	0	0,01	0,93	0,04	0,03	0	0,04
1	8838	0	0	0	0,12	0,88	0	0	0	0	0,06	0,93	0,01	0	0,048
1	14097	0	0,04	0	0,01	0,95	0	0	0	0	0	0,04	0,01	0,95	0,018
1	4866	0	0,04	0	0,02	0	0,01	0	0,91	0,02	0,01	0,05	0,03	0,91	0,026
1	1773	0,01	0,03	0	0,08	0,88	0	0	0	0	0,09	0,13	0,78	0	0,048
1	7721	0	0	0	0,15	0,68	0	0	0,17	0	0,26	0,17	0,57	0	0,042
1	2402	0	0,04	0	0	0,9	0	0,01	0,05	0	0	0,04	0,96	0	0,032
1	2053	0	0,04	0	0,01	0,95	0	0	0	0	0	0,06	0,93	0,01	0,018
1	1662	0	0,04	0	0	0,96	0	0	0	0	0	0,04	0,96	0	0,018
1	7468	0,01	0,04	0	0,02	0,92	0	0	0,01	0	0,02	0,95	0,02	0,01	0,048
1	3397	0	0,04	0,01	0	0,95	0	0	0	0	0	0,04	0,28	0,68	0,022
1,25	3185	0,01	0,04	0	0	0,95	0	0	0	0	0,18	0,8	0	0,02	0,04
1,25	8399	0,01	0,03	0,01	0,04	0,89	0	0	0,02	0	0,05	0,03	0,9	0,02	0,046

1,25	11517	0	0,04	0	0	0,96	0	0	0	0	0	0,03	0,96	0,01	0,034
1,25	17669	0	0,04	0	0,01	0,93	0	0,01	0,01	0	0	0,05	0,93	0,02	0,042
1,25	6347	0	0,04	0	0	0,96	0	0	0	0	0	0,07	0,93	0	0,03
1,25	11187	0	0,04	0	0,01	0,94	0,01	0	0	0	0	0,99	0	0,01	0,024
1,25	3703	0	0,04	0	0,03	0,88	0	0	0,04	0,01	0	0,95	0,03	0,02	0,034
1,25	8242	0	0,04	0	0,17	0,77	0	0	0,01	0,01	0,79	0,01	0,2	0	0,038
1,25	4694	0	0,04	0	0,01	0,94	0	0	0,01	0	0,77	0,06	0	0,17	0,038
1,25	1693	0	0,04	0	0	0,96	0	0	0	0	0	0,15	0	0,85	0,014
1,25	7502	0	0,04	0	0	0,96	0	0	0	0	0	0,04	0,01	0,95	0,008
1,25	3629	0	0	0	0,11	0,89	0	0	0	0	0,01	0	0,93	0,06	0,048
1,25	1413	0	0,04	0	0,1	0,83	0	0	0,03	0	0,86	0,04	0,1	0	0,048
1,25	3155	0,01	0,04	0	0	0,95	0	0	0	0	0	0,04	0	0,96	0,022
1,25	7477	0	0,02	0	0,1	0,81	0,02	0,01	0,04	0	0,04	0,02	0,91	0,03	0,046
1,25	5869	0	0	0	0,26	0,71	0	0	0	0,03	0,74	0,26	0	0	0,048
1,25	2713	0	0,04	0	0	0,96	0	0	0	0	0,04	0,03	0,01	0,92	0,006
1,25	4030	0	0,04	0	0	0,95	0	0,01	0	0	0	0,05	0,95	0	0,03
1,25	5344	0	0	0	0,13	0,86	0,01	0	0	0	0,01	0	0,99	0	0,048
1,25	1665	0	0,04	0	0	0,96	0	0	0	0	0	0,3	0	0,7	0,014
1,25	11404	0	0	0	0,38	0,62	0	0	0	0	1	0	0	0	0,044
1,25	25501	0	0,04	0	0	0,94	0,02	0	0	0	0	0,98	0,01	0,01	0,026
1,25	1876	0	0,04	0	0	0,93	0,01	0	0,01	0,01	0	0,98	0,02	0	0,036
1,25	6627	0	0,04	0	0	0,96	0	0	0	0	0	0	1	0	0,026
1,25	5857	0	0,04	0	0	0,95	0	0	0	0,01	0	0,05	0,95	0	0,02
1,25	5552	0	0,04	0	0	0,96	0	0	0	0	0	1	0	0	0,034
1,25	12645	0	0,04	0	0	0,96	0	0	0	0	0	0,04	0,96	0	0,028
1,25	1540	0	0,04	0,01	0	0,95	0	0	0	0	0	0,04	0,96	0	0,03
1,25	23953	0	0	0	0,14	0,86	0	0	0	0	0,14	0,86	0	0	0,038
1,25	10801	0	0,04	0	0,02	0,93	0	0,01	0	0	0	0,04	0,95	0,01	0,028
1,25	9708	0	0	0	0,37	0	0,62	0	0	0,01	0,9	0,1	0	0	0,048
1,25	9207	0	0,04	0	0,07	0,53	0	0	0,36	0	0	0,47	0	0,53	0,044
1,25	2300	0	0,04	0	0	0,96	0	0	0	0	0	0,04	0,96	0	0,018
1,25	11044	0,01	0,04	0	0,04	0,88	0	0	0,02	0,01	0,01	0,07	0,9	0,02	0,046
1,25	5834	0	0	0	0,23	0,77	0	0	0	0	0,77	0	0,23	0	0,048

1,25	8041	0	0,04	0	0	0,96	0	0	0	0	0	0,05	0,06	0,89	0,018
1,25	11438	0	0,01	0	0,1	0,89	0	0	0	0	0,05	0,95	0	0	0,048
1,25	4155	0	0,04	0	0	0,82	0,03	0	0,11	0	0	0,89	0,11	0	0,044
1,25	1735	0	0,04	0	0,02	0,93	0	0	0	0,01	0	0,06	0,01	0,93	0,026
1,25	11188	0	0	0	0,12	0,87	0,01	0	0	0	0	0,87	0,13	0	0,044
1,25	1382	0,01	0,02	0	0,16	0,74	0,07	0	0	0	0,9	0,01	0,07	0,02	0,044
1,25	1322	0	0,04	0	0	0,96	0	0	0	0	0	1	0	0	0,028
1,25	6791	0	0,04	0	0	0,96	0	0	0	0	0	0,04	0	0,96	0,006
1,25	2929	0,01	0,04	0	0,02	0,66	0,01	0,02	0,24	0	0,25	0,04	0,6	0,11	0,046
1,25	1109	0	0,03	0	0,14	0,8	0	0	0,03	0	0,71	0,03	0	0,26	0,044
1,25	8883	0	0,04	0	0	0,93	0	0,01	0,02	0	0,01	0,04	0,02	0,93	0,012
1,25	2190	0	0,04	0	0	0,96	0	0	0	0	0	0,04	0,96	0	0,032
1,25	7868	0	0,04	0	0,01	0,95	0	0	0	0	0,01	0,05	0,94	0	0,034
1,25	5431	0	0,03	0	0,12	0,68	0,17	0	0	0	0,13	0,74	0	0,13	0,046
1,25	6293	0	0	0	0,22	0,75	0	0,01	0	0,02	0,75	0,02	0,22	0,01	0,046
1,25	1254	0,01	0,04	0	0	0,95	0	0	0	0	0,01	0,04	0,95	0	0,034
1,25	1900	0	0,04	0	0	0,88	0	0	0,08	0	0	0,04	0,88	0,08	0,014
1,25	8108	0	0,04	0	0	0,96	0	0	0	0	0,06	0	0,94	0	0,03
1,25	8128	0	0,04	0	0	0,95	0	0	0,01	0	0	0,04	0,01	0,95	0,002
1,25	2525	0	0,04	0	0	0,94	0	0,02	0	0	0	0,07	0	0,93	0,032
1,25	3556	0	0,04	0	0	0,96	0	0	0	0	0	0,04	0	0,96	0,01
1,25	2898	0	0,04	0	0	0,94	0,01	0,01	0	0	0,94	0,01	0,04	0,01	0,048
1,25	923	0	0,04	0	0	0,96	0	0	0	0	0,96	0,04	0	0	0,04
1,25	19707	0	0,04	0	0,01	0,95	0	0	0	0	0,61	0,39	0	0	0,04
1,25	9382	0	0	0	0,14	0,82	0,04	0	0	0	0,1	0,05	0,82	0,03	0,046
1,25	6090	0,01	0,02	0	0,09	0,88	0	0	0	0	0	0,12	0,88	0	0,04
1,25	7944	0	0,04	0	0	0,95	0	0	0,01	0	0,89	0,04	0,07	0	0,036
1,25	4725	0	0	0	0,09	0,91	0	0	0	0	0,09	0,39	0	0,52	0,048
1,25	11583	0	0,04	0	0	0,96	0	0	0	0	0	0,04	0,96	0	0,018
1,25	9757	0	0,04	0,01	0	0,89	0,03	0	0,03	0	0,78	0,06	0,16	0	0,038
1,25	16545	0	0,04	0	0,1	0,82	0,04	0	0	0	0,13	0,86	0	0,01	0,048
1,25	10256	0	0,04	0	0	0,95	0	0	0	0,01	0,28	0,04	0,01	0,67	0,024
1,25	42494	0	0,04	0	0,01	0,95	0	0	0	0	0,02	0,04	0,03	0,91	0,02

1,25	13854	0	0,04	0	0	0,94	0,02	0	0	0	0	0,91	0,07	0,02	0,026
1,25	7133	0	0	0	0,08	0,92	0	0	0	0	0	0	0,08	0,92	0,046
1,25	10903	0	0	0	0,34	0,66	0	0	0	0	0,86	0,14	0	0	0,046
1,25	9211	0,01	0,04	0	0	0,95	0	0	0	0	0,63	0,05	0,03	0,29	0,046
1,25	1456	0	0,04	0	0,17	0,79	0	0	0	0	0,79	0,21	0	0	0,048
1,25	6127	0	0,04	0,01	0	0,95	0	0	0	0	0	0,04	0,01	0,95	0,006
1,25	8017	0	0,04	0	0	0,94	0	0	0,02	0	0	0,13	0	0,87	0,004
1,25	2047	0	0,04	0	0	0,96	0	0	0	0	0,91	0,04	0	0,05	0,044
1,25	8307	0,01	0,04	0	0,02	0,93	0	0	0	0	0,02	0,05	0,89	0,04	0,038
1,25	8452	0	0,04	0	0	0,96	0	0	0	0	0	0,04	0,96	0	0,028
1,25	1929	0	0,04	0	0	0,96	0	0	0	0	0	0,04	0,96	0	0,02
1,25	9965	0,05	0	0	0	0,84	0,11	0	0	0	0,88	0,1	0	0,02	0,038
1,25	2924	0	0,04	0,02	0	0,93	0	0	0,01	0	0,95	0,04	0	0,01	0,046
1,25	2566	0	0,04	0	0	0,96	0	0	0	0	0	0,04	0	0,96	0,012
1,25	2120	0	0,04	0	0	0,96	0	0	0	0	0	0,86	0,14	0	0,026
1,25	2594	0	0,04	0	0,01	0,95	0	0	0	0	0	0,05	0,95	0	0,03
1,25	8735	0	0,04	0	0	0,96	0	0	0	0	0	0,04	0	0,96	0,01
1,25	1422	0	0,04	0	0	0,96	0	0	0	0	0	0,04	0,96	0	0,042
1,25	2284	0	0,04	0	0	0,96	0	0	0	0	0	0,99	0,01	0	0,026
1,25	1807	0,01	0,04	0	0	0,9	0	0,02	0,03	0	0,01	0,09	0,02	0,88	0,024
1,25	2931	0	0,04	0	0,01	0,94	0	0	0	0,01	0,01	0,04	0	0,95	0,01
1,25	7728	0	0	0	0,16	0,84	0	0	0	0	0,25	0,16	0,59	0	0,042
1,25	11357	0	0,04	0	0	0,94	0	0,02	0	0	0	0,05	0,94	0,01	0,028
1,25	9969	0	0,04	0	0	0,91	0,04	0	0	0,01	0,91	0,04	0,05	0	0,03
1,25	1874	0	0,04	0	0,01	0,95	0	0	0	0	0,12	0,04	0,83	0,01	0,032
1,25	2798	0	0,04	0	0	0,96	0	0	0	0	0	0,04	0	0,96	0,01
1,25	9420	0	0,04	0	0	0,95	0	0	0	0,01	0,01	0,04	0	0,95	0,01
1,25	15734	0	0,04	0,02	0,01	0,92	0,01	0	0	0	0,02	0,39	0,57	0,02	0,026
1,25	6982	0	0,04	0	0	0,96	0	0	0	0	0	0,04	0,96	0	0,02
1,25	4433	0	0,04	0	0	0,95	0	0,01	0	0	0,82	0,18	0	0	0,036
1,25	2547	0	0,04	0	0,02	0,94	0	0	0	0	0	0,04	0	0,96	0,018
1,25	6739	0	0,04	0	0	0,93	0	0	0,02	0,01	0	0,17	0	0,83	0,004
1,5	6084	0	0,04	0	0	0,96	0	0	0	0	0	0,04	0,96	0	0,028

1,5	10831	0	0,04	0,02	0,02	0,91	0	0	0	0,01	0,9	0,04	0,04	0,02	0,046
1,5	1085	0	0,04	0	0	0,92	0	0,04	0	0	0,96	0,04	0	0	0,042
1,5	12721	0	0,04	0	0	0,95	0	0	0,01	0	0	0,05	0,95	0	0,022
1,5	20296	0	0,04	0	0,02	0,92	0	0	0,01	0,01	0,01	0,97	0	0,02	0,04
1,5	4307	0,01	0,04	0	0	0,94	0	0,01	0	0	0,94	0,04	0,01	0,01	0,04
1,5	1973	0	0,04	0	0	0,96	0	0	0	0	0	0,04	0	0,96	0,02
1,5	4274	0	0,04	0,01	0	0,95	0	0	0	0	0,01	0,04	0,95	0	0,018
1,5	17015	0,05	0	0	0	0,68	0,27	0	0	0	0	0,27	0,04	0,69	0,044
1,5	9290	0,01	0,04	0	0	0,95	0	0	0	0	0,01	0,03	0,95	0,01	0,016
1,5	1519	0	0,04	0	0	0,94	0	0,01	0,01	0	0	0,05	0,94	0,01	0,024
1,5	11695	0	0	0,01	0,12	0,68	0,19	0	0	0	0,25	0,55	0	0,2	0,048
1,5	5771	0	0,04	0,01	0	0,95	0	0	0	0	0,01	0,02	0,02	0,95	0,01
1,5	21473	0	0	0	0,31	0,68	0	0,01	0	0	0,99	0	0,01	0	0,048
1,5	20796	0	0,04	0	0,03	0,93	0	0	0	0	0,93	0,04	0,03	0	0,048
1,5	4902	0	0,04	0	0	0,96	0	0	0	0	0	1	0	0	0,026
1,5	28263	0	0	0	0,11	0,77	0	0	0,12	0	0,02	0,87	0,11	0	0,048
1,5	2115	0	0,04	0	0	0,93	0,03	0	0	0	0,01	0,04	0	0,95	0,004
1,5	3385	0	0,04	0	0,04	0,91	0	0	0,01	0	0,66	0,25	0,05	0,04	0,042
1,5	23711	0	0,04	0	0	0,91	0,04	0	0,01	0	0,77	0,22	0,01	0	0,048
1,5	7395	0	0,04	0	0	0,96	0	0	0	0	0	0,04	0,96	0	0,018
1,5	8022	0	0,03	0,02	0,06	0,88	0	0,01	0	0	0,03	0,01	0,9	0,06	0,044
1,5	8052	0	0	0	0,15	0,84	0	0	0	0,01	0	0,09	0,91	0	0,048
1,5	2136	0	0	0	0,17	0,83	0	0	0	0	0,17	0	0,83	0	0,048
1,5	2692	0	0,04	0	0	0,96	0	0	0	0	0	0,04	0,92	0,04	0,034
1,5	14075	0,01	0,04	0	0	0,95	0	0	0	0	0	0,04	0,96	0	0,028
1,5	3747	0	0,04	0	0	0,94	0,01	0	0,01	0	0	0,99	0	0,01	0,036
1,5	29459	0	0,04	0	0	0,96	0	0	0	0	0	0,04	0,96	0	0,036
1,5	14329	0	0	0	0,13	0,58	0,29	0	0	0	0	0,29	0,52	0,19	0,042
1,5	15603	0,01	0,04	0	0,02	0,92	0	0	0,01	0	0,03	0,96	0,01	0	0,04
1,5	7333	0	0,01	0	0,15	0,72	0	0	0,07	0,05	0,72	0,01	0,05	0,22	0,048
1,5	15544	0	0	0	0,14	0,86	0	0	0	0	0	0,84	0,16	0	0,046
1,5	15291	0	0,04	0	0	0,95	0,01	0	0	0	0,01	0,04	0	0,95	0,014
1,5	6757	0	0,04	0	0,02	0,92	0,01	0	0	0,01	0,01	0,96	0	0,03	0,036

1,5	23922	0	0,04	0,01	0	0,94	0	0	0,01	0	0,95	0,05	0	0	0,044
1,5	14764	0	0,04	0	0	0,96	0	0	0	0	0	1	0	0	0,024
1,5	10989	0	0,04	0	0,01	0,95	0	0	0	0	0	0,01	0,9	0,09	0,016
1,5	8181	0	0	0	0,07	0,92	0	0,01	0	0	0,01	0,06	0	0,93	0,048
1,5	24586	0	0,04	0	0	0,96	0	0	0	0	0	0,04	0,96	0	0,03
1,5	2999	0	0,04	0	0	0,95	0,01	0	0	0	0,95	0,04	0,01	0	0,04
1,5	29708	0	0,04	0,01	0,01	0,94	0	0	0	0	0,94	0,05	0	0,01	0,044
1,5	10069	0	0,03	0	0,07	0,87	0	0	0,03	0	0,04	0,02	0,07	0,87	0,048
1,5	12999	0	0,04	0	0,02	0,94	0	0	0	0	0,8	0,18	0	0,02	0,046
1,5	19348	0	0,04	0	0	0,95	0,01	0	0	0	0,01	0,03	0	0,96	0,004
1,5	4260	0	0,04	0	0	0,96	0	0	0	0	0	0,79	0,16	0,05	0,022
1,5	3818	0	0,04	0	0	0,96	0	0	0	0	0	0,04	0,95	0,01	0,032
1,5	3489	0	0	0	0,14	0,86	0	0	0	0	0,14	0,86	0	0	0,038
1,5	4143	0	0	0	0,39	0,61	0	0	0	0	0,99	0	0,01	0	0,04
1,5	5440	0	0,04	0	0	0,94	0,01	0,01	0	0	0	0,05	0,94	0,01	0,03
1,5	2495	0	0,04	0	0	0,96	0	0	0	0	0	1	0	0	0,026
1,5	5931	0	0,04	0	0	0,96	0	0	0	0	0,98	0,01	0	0,01	0,038
1,5	3931	0	0,04	0	0	0,96	0	0	0	0	0,45	0,04	0,26	0,25	0,036
1,5	6966	0	0	0	0,11	0,89	0	0	0	0	0	0,11	0,89	0	0,048
1,5	18007	0,01	0	0	0,07	0	0,92	0	0	0	0,26	0	0,07	0,67	0,038
1,5	13051	0,01	0,04	0	0	0,94	0	0	0,01	0	0,01	0	0,05	0,94	0,008
1,5	7045	0	0,04	0	0	0,93	0	0	0,02	0,01	0	0,93	0,07	0	0,012
1,5	10293	0	0	0	0,1	0,89	0	0	0	0,01	0,1	0,01	0,55	0,34	0,036
1,5	2447	0	0,04	0	0	0,94	0	0	0,02	0	0,02	0,04	0,94	0	0,04
1,5	13221	0	0	0	0,31	0,69	0	0	0	0	0,8	0,2	0	0	0,048
1,5	10350	0	0,04	0	0	0,96	0	0	0	0	0	0,96	0,04	0	0,024
1,5	18465	0	0	0	0,18	0,82	0	0	0	0	0,18	0,81	0,01	0	0,048
1,5	4121	0	0,03	0	0,2	0,76	0	0	0,01	0	0,76	0,05	0	0,19	0,048
1,5	27800	0	0,04	0	0	0,92	0	0,01	0,03	0	0	0,97	0	0,03	0,024
1,5	2470	0	0,04	0,01	0	0,95	0	0	0	0	0	0,03	0,14	0,83	0,014
1,5	25288	0	0	0	0,07	0,91	0	0	0,02	0	0	0	0,09	0,91	0,048
1,5	9090	0	0,04	0	0,04	0,92	0	0	0	0	0	0,51	0,49	0	0,044
1,5	2796	0	0,04	0	0	0,92	0,04	0	0	0	0	0,13	0,83	0,04	0,03

1,5	5932	0	0,04	0	0	0,93	0	0,03	0	0	0,02	0,05	0	0,93	0,036
1,5	8867	0	0,04	0	0,03	0,9	0,03	0	0	0	0,06	0,9	0,04	0	0,038
1,5	21059	0	0,04	0	0	0,93	0	0	0,03	0	0	0,04	0,03	0,93	0,004
1,5	5534	0,01	0,04	0	0,01	0,94	0	0	0	0	0,68	0,04	0	0,28	0,038
1,5	3000	0	0,04	0	0	0,94	0	0	0,01	0,01	0	0,05	0,94	0,01	0,028
1,5	29596	0	0,04	0	0	0,96	0	0	0	0	0,96	0,04	0	0	0,034
1,5	21110	0	0	0	0,16	0,8	0,04	0	0	0	0,16	0,8	0	0,04	0,044
1,5	11981	0	0,04	0	0	0,96	0	0	0	0	0	0,96	0	0,04	0,022
1,5	14660	0	0,04	0	0	0,72	0,05	0	0,19	0	0,19	0,72	0,09	0	0,032
1,5	7750	0	0	0	0,35	0,63	0,01	0	0,01	0	0,96	0,02	0,01	0,01	0,048
1,5	7242	0	0,04	0	0,06	0,89	0,01	0	0	0	0,03	0,01	0,76	0,2	0,038
1,5	6099	0	0	0,01	0,1	0,88	0	0	0,01	0	0,19	0,01	0	0,8	0,034
1,5	8452	0	0,04	0	0,01	0,95	0	0	0	0	0	0,05	0,95	0	0,032
1,5	11695	0	0	0	0,43	0,57	0	0	0	0	1	0	0	0	0,048
1,5	1902	0	0,04	0	0	0,95	0,01	0	0	0	0	0,04	0,95	0,01	0,026
1,5	6092	0	0,04	0	0	0,9	0,01	0	0,05	0	0,07	0,04	0	0,89	0,008
1,5	3530	0	0,04	0	0	0,95	0	0,01	0	0	0,01	0,04	0	0,95	0,018
1,5	34131	0	0,04	0	0,02	0,94	0	0	0	0	0	0,04	0,94	0,02	0,038
1,5	8947	0	0,04	0	0	0,96	0	0	0	0	0	0,04	0	0,96	0,012
1,5	5418	0	0,04	0	0	0,96	0	0	0	0	0,01	0,03	0,47	0,49	0,016
1,5	12941	0	0,04	0	0	0,95	0	0	0	0,01	0	0,05	0,95	0	0,028
1,5	17371	0	0,04	0	0	0,96	0	0	0	0	0	0,04	0,96	0	0,016
1,5	23304	0	0,02	0	0,11	0,87	0	0	0	0	0,11	0,89	0	0	0,034
1,5	2119	0	0,04	0	0	0,96	0	0	0	0	0	1	0	0	0,022
1,5	8566	0	0,04	0	0	0,93	0,03	0	0	0	0,69	0,07	0	0,24	0,034
1,5	16283	0	0	0	0,32	0,65	0,02	0	0	0,01	0,98	0	0,02	0	0,036
1,5	8124	0	0,04	0	0	0,96	0	0	0	0	0	0,04	0	0,96	0,012
1,5	2661	0	0,04	0	0	0,95	0,01	0	0	0	0	0,04	0,01	0,95	0,008
1,5	13485	0	0,04	0	0,03	0,91	0	0,02	0	0	0	0,95	0,03	0,02	0,04
1,5	8958	0	0	0	0,2	0,79	0,01	0	0	0	0,79	0,01	0,19	0,01	0,048
1,5	14864	0,01	0,04	0	0	0,95	0	0	0	0	0,01	0,98	0	0,01	0,046
1,5	2977	0,02	0,04	0	0,01	0,88	0	0,01	0,04	0	0,01	0,09	0,89	0,01	0,038
1,5	10648	0	0,04	0	0	0,92	0,04	0	0	0	0	0,08	0,92	0	0,026

1,75	14957	0	0,04	0	0	0,96	0	0	0	0	0	0,04	0	0,96	0,008
1,75	9057	0	0	0	0,15	0,8	0,01	0	0,04	0	0,19	0,01	0,8	0	0,044
1,75	44271	0	0,04	0	0	0,04	0,5	0,02	0,4	0	0	0,42	0,5	0,08	0,034
1,75	9563	0	0,04	0	0,02	0,94	0	0	0	0	0,94	0,05	0,01	0	0,028
1,75	13291	0	0,04	0	0	0,96	0	0	0	0	0	0,53	0	0,47	0,024
1,75	3869	0	0,04	0	0,03	0,92	0	0	0	0,01	0,92	0,07	0,01	0	0,046
1,75	11971	0	0	0	0,11	0,8	0,08	0,01	0	0	0,04	0,81	0,08	0,07	0,04
1,75	13964	0,01	0	0,01	0,05	0,93	0	0	0	0	0,06	0	0	0,94	0,046
1,75	15942	0	0,04	0	0	0,92	0,01	0,03	0	0	0,04	0,93	0	0,03	0,042
1,75	61307	0	0	0	0,12	0,88	0	0	0	0	0	0,39	0,61	0	0,046
1,75	49329	0	0,04	0	0,01	0,93	0,01	0	0	0,01	0,92	0,05	0,02	0,01	0,028
1,75	8675	0	0,04	0	0	0,94	0,01	0	0,01	0	0,02	0,04	0,28	0,66	0,018
1,75	37351	0	0,04	0,02	0	0,93	0	0	0	0,01	0,04	0	0,94	0,02	0,032
1,75	11124	0	0,04	0	0	0,96	0	0	0	0	0	0,04	0,02	0,94	0,012
1,75	2917	0	0,04	0,01	0,05	0,87	0	0	0,03	0	0,87	0,06	0,03	0,04	0,046
1,75	4945	0	0,04	0	0	0,95	0	0	0	0,01	0,01	0,04	0,12	0,83	0,012
1,75	11547	0	0,04	0,01	0,01	0,94	0	0	0	0	0,95	0,05	0	0	0,032
1,75	74193	0	0	0	0,15	0,83	0	0	0,02	0	0	0,83	0,15	0,02	0,046
1,75	16580	0	0	0	0,34	0,58	0,08	0	0	0	0,84	0,08	0,08	0	0,048
1,75	49165	0,02	0,04	0	0	0,41	0	0	0,53	0	0,04	0,02	0,41	0,53	0,04
1,75	12437	0	0,04	0	0	0,76	0,17	0	0,03	0	0,76	0,04	0,03	0,17	0,034
1,75	4621	0	0,04	0	0	0,95	0	0	0	0,01	0,74	0,04	0,01	0,21	0,016
1,75	35810	0	0,03	0	0,1	0,87	0	0	0	0	0,26	0,23	0,51	0	0,044
1,75	18156	0	0	0	0,09	0,02	0,15	0	0,74	0	0,02	0,24	0,13	0,61	0,044
1,75	33428	0	0,04	0	0	0,95	0,01	0	0	0	0	0,05	0	0,95	0,01
1,75	26792	0	0,04	0	0	0,95	0	0	0,01	0	0,95	0,03	0	0,02	0,036
1,75	11581	0	0,04	0	0,01	0,95	0	0	0	0	0	0,04	0,96	0	0,018
1,75	17374	0	0,04	0	0	0,95	0,01	0	0	0	0,71	0,04	0,25	0	0,038
1,75	19419	0,01	0,04	0	0	0,91	0,03	0	0	0,01	0,92	0,04	0,01	0,03	0,048
1,75	18882	0	0,04	0	0	0,96	0	0	0	0	0	0,02	0,02	0,96	0,014
1,75	20564	0,02	0,04	0	0	0,94	0	0	0	0	0,02	0,43	0,55	0	0,04
1,75	9245	0	0	0	0,12	0,88	0	0	0	0	0	0,88	0,12	0	0,048
1,75	48468	0	0,04	0	0	0,96	0	0	0	0	0	1	0	0	0,016

1,75	17439	0	0,04	0	0	0,96	0	0	0	0	0,03	0,92	0,01	0,04	0,028
1,75	24869	0	0,04	0	0	0,91	0,02	0	0	0,03	0,19	0,77	0,03	0,01	0,032
1,75	77325	0	0,04	0	0	0,96	0	0	0	0	0,31	0,67	0,02	0	0,03
1,75	22301	0	0,04	0	0,05	0,89	0,02	0	0	0	0,05	0,04	0,89	0,02	0,042
1,75	58491	0	0	0	0,11	0,88	0	0,01	0	0	0	0	0,99	0,01	0,048
1,75	25159	0	0,04	0	0,01	0,95	0	0	0	0	0	0,05	0,95	0	0,028
1,75	6830	0	0,04	0	0	0,95	0	0	0,01	0	0,03	0,01	0,51	0,45	0,03
1,75	12682	0	0,04	0	0	0,96	0	0	0	0	0	0,05	0	0,95	0,008
1,75	1417	0	0,04	0	0,02	0,94	0	0	0	0	0,06	0,04	0,9	0	0,026
1,75	20842	0	0,04	0	0	0,94	0	0	0,02	0	0	0,08	0	0,92	0,01
1,75	18092	0	0,04	0	0,02	0,89	0,05	0	0	0	0,88	0,09	0,03	0	0,036
1,75	16545	0	0,04	0,01	0	0,93	0	0	0,01	0,01	0,02	0,06	0	0,92	0,014
1,75	26814	0	0,04	0	0,02	0,94	0	0	0	0	0	0,87	0,13	0	0,042
1,75	58238	0	0,04	0	0	0,96	0	0	0	0	0	0,99	0	0,01	0,02
1,75	28502	0	0,04	0	0	0,96	0	0	0	0	0	1	0	0	0,02
1,75	26633	0	0,03	0	0,06	0,91	0	0	0	0	0	0,04	0,05	0,91	0,036
1,75	19097	0	0,04	0	0	0,95	0	0	0	0,01	0	0,05	0,95	0	0,016
1,75	11926	0,01	0,04	0,01	0	0	0	0,02	0,92	0	0,01	0,06	0,01	0,92	0,04
1,75	9812	0	0,04	0	0	0,95	0	0	0	0,01	0,95	0	0,01	0,04	0,03
1,75	8371	0	0,04	0	0,03	0,93	0	0	0	0	0	0,04	0,93	0,03	0,03
1,75	17685	0,01	0,04	0	0	0,95	0	0	0	0	0,53	0,06	0,4	0,01	0,04
1,75	10757	0	0,04	0	0	0,94	0	0,01	0	0,01	0,13	0	0,86	0,01	0,026
1,75	2472	0	0,04	0	0	0,94	0,02	0	0	0	0	0,06	0,94	0	0,034
1,75	1879	0	0,04	0	0	0,96	0	0	0	0	0	0,04	0,83	0,13	0,038
1,75	18580	0	0,04	0	0	0,96	0	0	0	0	0	0,03	0,96	0,01	0,022
1,75	4183	0	0,04	0	0	0,96	0	0	0	0	0	0,04	0,92	0,04	0,028
1,75	2970	0,02	0,04	0	0,03	0,91	0	0	0	0	0	0,05	0,03	0,92	0,042
1,75	15793	0	0,04	0	0	0,96	0	0	0	0	0,13	0,38	0,49	0	0,012
1,75	18348	0	0,02	0	0,08	0,88	0	0	0,02	0	0,11	0,02	0	0,87	0,048
1,75	12573	0,03	0,03	0,01	0	0,93	0	0	0	0	0,04	0,03	0,93	0	0,044
1,75	4948	0	0,04	0,01	0	0,9	0,03	0	0,02	0	0	0,07	0,91	0,02	0,032
1,75	5274	0	0,04	0	0,04	0,92	0	0	0	0	0,04	0,96	0	0	0,046
1,75	30861	0,01	0,04	0	0,02	0,93	0	0	0	0	0,01	0,04	0,02	0,93	0,046

1,75	5296	0	0,04	0	0	0,95	0,01	0	0	0	0	0,04	0,96	0	0,024
1,75	2696	0	0,04	0	0,01	0,95	0	0	0	0	0	0,04	0,79	0,17	0,03
1,75	3162	0,01	0,03	0	0,09	0,87	0	0	0	0	0,87	0,03	0	0,1	0,042
1,75	11446	0	0,04	0	0	0,96	0	0	0	0	0	0,04	0	0,96	0,014
1,75	7346	0,01	0,04	0	0	0,95	0	0	0	0	0,01	0	0,98	0,01	0,038
1,75	7295	0	0,03	0	0,15	0,82	0	0	0	0	0,82	0,03	0,15	0	0,046
1,75	14881	0,03	0,04	0	0	0,91	0	0,01	0,01	0	0,03	0,95	0,01	0,01	0,042
1,75	18046	0	0,04	0	0	0,96	0	0	0	0	0	0,03	0,91	0,06	0,032
1,75	25411	0	0,04	0	0	0,96	0	0	0	0	0	0	0	1	0,006
1,75	7728	0	0,01	0	0,11	0,81	0	0	0,07	0	0	0,13	0,87	0	0,046
1,75	6604	0	0,04	0	0,07	0,89	0	0	0	0	0	0,93	0,07	0	0,038
1,75	9925	0	0,03	0	0,07	0	0	0	0,82	0,08	0,08	0,03	0,87	0,02	0,036
1,75	5451	0	0,04	0	0	0,95	0	0	0,01	0	0,01	0,04	0,95	0	0,028
1,75	6758	0,03	0,03	0,01	0	0,92	0	0,01	0	0	0,07	0,04	0,89	0	0,046
1,75	4042	0	0,04	0	0,05	0,88	0,03	0	0	0	0	0,01	0,93	0,06	0,042
1,75	15685	0	0,04	0,01	0	0,95	0	0	0	0	0,01	0,04	0,94	0,01	0,026
1,75	6292	0	0,01	0	0,32	0,64	0,02	0,01	0	0	0,97	0	0,02	0,01	0,048
1,75	4851	0	0,04	0	0,05	0,9	0	0	0	0,01	0	0,94	0,06	0	0,048
1,75	2583	0	0,04	0	0	0,96	0	0	0	0	0	1	0	0	0,032
1,75	18007	0	0,01	0	0,24	0,75	0	0	0	0	0,75	0,01	0,24	0	0,042
1,75	13109	0	0	0	0,13	0,86	0	0	0,01	0	0	0,86	0,13	0,01	0,042
1,75	24812	0	0,03	0	0,16	0,81	0	0	0	0	0,84	0	0,16	0	0,046
1,75	27811	0	0,04	0	0	0,95	0	0	0,01	0	0	0,05	0	0,95	0,01
1,75	16920	0	0,04	0	0	0,96	0	0	0	0	0	0,04	0,96	0	0,032
1,75	13079	0	0,04	0	0	0,95	0	0	0	0,01	0	0,04	0,01	0,95	0,008
1,75	45616	0	0,04	0	0	0,96	0	0	0	0	0	0,04	0,96	0	0,032
1,75	8600	0	0,04	0,01	0	0,91	0,02	0	0	0,02	0,01	0,06	0	0,93	0,018
1,75	13980	0,01	0	0,02	0,05	0,87	0	0	0,02	0,03	0,05	0,03	0	0,92	0,048
1,75	26756	0	0,04	0	0	0,93	0,01	0,02	0	0	0,73	0,26	0	0,01	0,034
1,75	13802	0,03	0,04	0	0	0,85	0,08	0	0	0	0,85	0,07	0,08	0	0,046
1,75	9435	0	0,04	0	0	0,95	0,01	0	0	0	0,01	0	0	0,99	0,01
1,75	5181	0	0,04	0	0,02	0,92	0	0,02	0	0	0,02	0,04	0,94	0	0,038
1,75	3706	0	0,04	0	0	0,95	0	0	0,01	0	0	0,03	0,97	0	0,016

1,75	4378	0	0,04	0	0	0,94	0,01	0,01	0	0	0,78	0,04	0	0,18	0,034
2	5167	0	0,04	0	0	0,96	0	0	0	0	0	0,04	0,96	0	0,03
2	48569	0	0,04	0	0	0,94	0	0	0,02	0	0	0,05	0,02	0,93	0,004
2	16376	0	0	0	0,37	0,63	0	0	0	0	1	0	0	0	0,048
2	32003	0	0,04	0	0	0,96	0	0	0	0	0,02	0,01	0,01	0,96	0,014
2	3102	0	0,04	0	0	0,93	0	0,01	0,02	0	0	0,06	0,93	0,01	0,022
2	13278	0	0,04	0	0	0,94	0,02	0	0	0	0,01	0,88	0	0,11	0,022
2	6525	0	0	0	0,25	0,75	0	0	0	0	0,75	0,25	0	0	0,048
2	21952	0	0,04	0	0	0,9	0	0,04	0	0,02	0	0,06	0,01	0,93	0,048
2	20369	0	0,04	0	0	0,96	0	0	0	0	0	0,19	0,01	0,8	0,012
2	16245	0	0,04	0	0,14	0,77	0,05	0	0	0	0,56	0,04	0,26	0,14	0,048
2	1554	0	0,04	0	0	0,95	0	0	0	0,01	0	0,04	0,95	0,01	0,028
2	23861	0	0,04	0,03	0	0,92	0	0	0,01	0	0,29	0,05	0	0,66	0,026
2	24798	0	0,04	0	0	0,93	0,03	0	0	0	0,44	0,55	0	0,01	0,042
2	7421	0	0,04	0	0	0,95	0	0	0,01	0	0,95	0,03	0,02	0	0,046
2	11681	0,03	0,03	0	0	0,89	0,03	0	0,02	0	0,92	0,03	0,02	0,03	0,044
2	64358	0	0,04	0	0	0,96	0	0	0	0	0	0,04	0,96	0	0,026
2	3722	0	0,04	0	0	0,95	0	0	0	0,01	0	0,04	0	0,96	0,008
2	10337	0	0,04	0	0	0,96	0	0	0	0	0	0,04	0	0,96	0,008
2	26598	0	0,04	0	0	0,93	0,03	0	0	0	0,9	0,1	0	0	0,046
2	9342	0	0,04	0,01	0	0,79	0	0	0,16	0	0	0,15	0,02	0,83	0,012
2	20632	0	0,04	0	0	0,96	0	0	0	0	0	0,04	0	0,96	0,002
2	20545	0	0	0	0,07	0,93	0	0	0	0	0,06	0,06	0	0,88	0,042
2	10467	0,01	0,03	0	0,08	0,88	0	0	0	0	0,01	0,1	0,89	0	0,036
2	2947	0,01	0,02	0,01	0,06	0,89	0	0,01	0	0	0	0,92	0,06	0,02	0,046
2	38268	0,01	0,04	0	0,01	0,91	0,01	0,01	0,01	0	0,02	0,94	0,04	0	0,044
2	9048	0	0,04	0	0	0,96	0	0	0	0	0	0,01	0	0,99	0,004
2	28304	0	0,04	0	0	0,96	0	0	0	0	0	0,04	0,92	0,04	0,022
2	2673	0	0,04	0	0	0,96	0	0	0	0	0	0,06	0	0,94	0,012
2	7964	0,03	0	0,01	0	0,93	0	0,03	0	0	0,06	0	0	0,94	0,046
2	17484	0	0,03	0	0,11	0,86	0	0	0	0	0,83	0,03	0,03	0,11	0,044
2	3340	0	0,02	0	0,1	0,88	0	0	0	0	0	0,88	0,1	0,02	0,044
2	2990	0	0,04	0	0	0,96	0	0	0	0	0	0,04	0	0,96	0,006

2	47661	0	0,04	0	0	0,96	0	0	0	0	0	0,04	0,96	0	0,024
2	10563	0	0,03	0	0,11	0,62	0,24	0	0	0	0,27	0	0,62	0,11	0,048
2	4860	0	0,04	0	0	0,83	0	0	0,13	0	0	0,04	0,13	0,83	0,012
2	67264	0	0	0	0,36	0,62	0	0	0	0,02	0,99	0	0,01	0	0,048
2	10213	0	0,04	0	0	0,96	0	0	0	0	0,01	0,04	0	0,95	0,002
2	27437	0,01	0	0	0,12	0,87	0	0	0	0	0,12	0,01	0,87	0	0,048
2	29868	0	0	0	0,07	0	0	0	0,93	0	0	0	0,07	0,93	0,048
2	8916	0	0,04	0	0,01	0,94	0,01	0	0	0	0,1	0,05	0,85	0	0,04
2	6906	0	0,04	0	0	0,96	0	0	0	0	0	0,04	0,96	0	0,028
2	35456	0	0,04	0	0	0,94	0	0,02	0	0	0,94	0,04	0,01	0,01	0,046
2	20166	0	0,04	0	0	0,95	0	0,01	0	0	0	0,04	0,01	0,95	0,012
2	9580	0	0,04	0	0	0,94	0	0	0	0,02	0,01	0,06	0,83	0,1	0,014
2	7577	0	0,04	0	0	0,96	0	0	0	0	0	0,04	0	0,96	0,01
2	22061	0	0	0	0,07	0,85	0,08	0	0	0	0	0,25	0,05	0,7	0,04
2	7820	0	0,04	0	0	0,96	0	0	0	0	0	0,97	0	0,03	0,024
2	20415	0	0,04	0	0	0,96	0	0	0	0	0,03	0,25	0	0,72	0,014
2	12112	0	0,04	0	0	0,39	0,08	0	0,49	0	0,02	0,01	0,58	0,39	0,018
2	29324	0	0,04	0	0	0,94	0	0	0,02	0	0	0,04	0	0,96	0,004
2	18167	0	0,04	0	0,04	0,91	0,01	0	0	0	0	0,95	0,05	0	0,048
2	4211	0	0,04	0	0	0,94	0	0,01	0	0,01	0	0,04	0,95	0,01	0,032
2	27119	0	0,04	0,01	0	0,94	0	0	0,01	0	0,01	0,05	0,94	0	0,036
2	7868	0	0	0	0,23	0,73	0	0	0,04	0	0,73	0,01	0,26	0	0,044
2	3360	0	0,04	0	0	0,96	0	0	0	0	0	0,03	0	0,97	0,008
2	19295	0	0	0	0,24	0,75	0	0	0,01	0	0,76	0	0,24	0	0,046
2	79845	0	0	0	0,12	0,88	0	0	0	0	0,12	0,53	0	0,35	0,04
2	50064	0	0,04	0	0	0,95	0	0	0,01	0	0,01	0,96	0,03	0	0,028
2	12705	0	0,04	0	0	0,95	0	0	0	0,01	0	0,04	0	0,96	0,012
2	26184	0	0,04	0	0,13	0,83	0	0	0	0	0,83	0,04	0,13	0	0,048
2	19943	0	0	0	0,39	0,59	0,02	0	0	0	1	0	0	0	0,048
2	35685	0	0,04	0	0	0,95	0	0	0	0,01	0,93	0,06	0,01	0	0,026
2	23890	0	0,02	0	0,09	0,88	0	0	0,01	0	0,09	0,02	0,88	0,01	0,048
2	10251	0	0,04	0	0	0,96	0	0	0	0	0,96	0	0,04	0	0,036
2	10658	0	0,04	0	0,04	0,92	0	0	0	0	0,02	0,98	0	0	0,038

2	7240	0	0,03	0,01	0	0,96	0	0	0	0	0,97	0,03	0	0	0,036
2	9589	0	0,04	0	0	0,95	0,01	0	0	0	0	0,04	0	0,96	0,01
2	3753	0	0,04	0	0	0,95	0	0,01	0	0	0	0	0,99	0,01	0,03
2	7986	0	0,04	0	0	0,95	0,01	0	0	0	0	0,39	0,6	0,01	0,028
2	20916	0	0,04	0	0	0,91	0	0	0,05	0	0,03	0,03	0,03	0,91	0,022
2	34926	0	0,04	0	0	0,94	0,01	0	0	0,01	0	0,04	0,95	0,01	0,026
2	10632	0	0,04	0	0,03	0,92	0,01	0	0	0	0	0,97	0,01	0,02	0,042
2	14884	0	0,04	0	0,01	0,94	0	0	0	0,01	0,02	0,04	0,94	0	0,022
2	56408	0	0,03	0	0,08	0,86	0,02	0,01	0	0	0	0,03	0,96	0,01	0,048
2	21777	0	0,04	0	0	0,95	0	0	0,01	0	0	0,05	0,95	0	0,024
2	13700	0	0,04	0	0	0,86	0,02	0	0,08	0	0	0,04	0,94	0,02	0,032
2	6640	0	0,04	0	0,01	0,95	0	0	0	0	0	0,04	0,95	0,01	0,022
2	15378	0	0,04	0	0	0,95	0,01	0	0	0	0	0,01	0,95	0,04	0,026
2	14663	0	0	0	0,15	0,85	0	0	0	0	0,65	0	0	0,35	0,048
2	42449	0,01	0,04	0	0	0,94	0,01	0	0	0	0,89	0,1	0,01	0	0,046
2	10075	0	0,04	0	0	0,96	0	0	0	0	0	0,04	0,96	0	0,018
2	4944	0,03	0,04	0	0	0,93	0	0	0	0	0	0,45	0	0,55	0,044
2	2599	0	0,04	0	0	0,96	0	0	0	0	0	0,04	0,96	0	0,018
2	27057	0,01	0,04	0	0	0,95	0	0	0	0	0	0,04	0,95	0,01	0,024
2	1096	0	0,04	0	0	0,89	0,01	0	0,06	0	0,34	0,04	0,6	0,02	0,024
2	18682	0	0,04	0	0,03	0,92	0,01	0	0	0	0,15	0,04	0,8	0,01	0,046
2	30291	0	0	0	0,13	0,73	0,14	0	0	0	0,13	0,14	0,73	0	0,046
2	22355	0	0,04	0	0	0,96	0	0	0	0	0	0,04	0	0,96	0,006
2	9277	0,01	0,04	0	0	0,95	0	0	0	0	0	0	0,01	0,99	0,026
2	4113	0	0,04	0	0	0,96	0	0	0	0	0	0,04	0,86	0,1	0,028
2	25575	0	0,04	0	0	0,96	0	0	0	0	0	0,04	0,94	0,02	0,03
2	13981	0,01	0,04	0	0	0,95	0	0	0	0	0,46	0,5	0,04	0	0,042
2	2499	0	0,04	0	0	0,96	0	0	0	0	0	1	0	0	0,028
2	25593	0	0,04	0,02	0	0	0	0	0,93	0,01	0	0,2	0,78	0,02	0,042
2	9267	0	0	0	0,13	0	0,04	0	0,83	0	0,02	0,14	0,81	0,03	0,048
2	31992	0	0	0	0,2	0	0,79	0	0	0,01	0,79	0,01	0,2	0	0,048
2	7642	0	0,04	0	0	0,96	0	0	0	0	0,96	0,04	0	0	0,036
2	19432	0	0,04	0	0	0,9	0	0	0,06	0	0,06	0,01	0,9	0,03	0,03

2	32200	0	0,04	0,01	0	0,95	0	0	0	0	0,04	0	0,01	0,95	0,012
2	27685	0	0,04	0	0	0,96	0	0	0	0	0,09	0,04	0	0,87	0,004
2,25	17157	0,01	0,04	0	0,05	0,9	0	0	0	0	0,91	0,04	0	0,05	0,046
2,25	8431	0	0,04	0	0	0,95	0	0	0,01	0	0,01	0,04	0	0,95	0,006
2,25	8661	0	0,04	0	0,09	0,87	0	0	0	0	0,87	0,09	0,03	0,01	0,048
2,25	47141	0	0	0	0,21	0,79	0	0	0	0	0,79	0	0,21	0	0,038
2,25	59972	0	0	0,01	0,13	0	0	0	0,86	0	0	0,99	0	0,01	0,048
2,25	31404	0,01	0,04	0	0	0,94	0	0	0,01	0	0,01	0,04	0,01	0,94	0,028
2,25	80648	0	0,04	0	0	0,88	0,08	0	0	0	0	0,04	0,88	0,08	0,02
2,25	21258	0	0,04	0	0	0,96	0	0	0	0	0	0,04	0	0,96	0,01
2,25	14086	0	0,04	0	0	0,91	0	0	0,05	0	0	0,1	0,2	0,7	0,022
2,25	11752	0	0	0	0,28	0	0,69	0,02	0	0,01	0,93	0	0,05	0,02	0,046
2,25	12629	0	0,04	0	0	0,95	0	0	0	0,01	0	0,05	0	0,95	0,018
2,25	12315	0	0,04	0	0	0,94	0	0	0,01	0,01	0,02	0,96	0	0,02	0,03
2,25	32472	0	0,04	0	0	0,96	0	0	0	0	0	1	0	0	0,032
2,25	11121	0	0,04	0	0,02	0,94	0	0	0	0	0	0,05	0,95	0	0,03
2,25	13480	0	0	0	0,16	0,84	0	0	0	0	0,16	0,84	0	0	0,048
2,25	11901	0	0,04	0,01	0	0,95	0	0	0	0	0,92	0,05	0,03	0	0,046
2,25	46695	0	0	0	0,07	0,93	0	0	0	0	0,07	0	0	0,93	0,042
2,25	41155	0	0,04	0	0,01	0,95	0	0	0	0	0,01	0,04	0,95	0	0,032
2,25	9654	0	0	0,01	0,1	0,84	0,03	0	0,02	0	0,04	0	0,84	0,12	0,048
2,25	4839	0,01	0,04	0	0	0,95	0	0	0	0	0	0,04	0	0,96	0,02
2,25	45223	0	0,04	0	0	0,96	0	0	0	0	0	0,04	0,96	0	0,028
2,25	34671	0	0,04	0,01	0	0,93	0	0	0,02	0	0	0,03	0,04	0,93	0,012
2,25	9937	0	0,04	0	0,03	0,93	0	0	0	0	0,03	0,04	0,93	0	0,04
2,25	12332	0	0,04	0,01	0,01	0,94	0	0	0	0	0	0,97	0,01	0,02	0,04
2,25	42089	0	0,04	0	0,03	0,93	0	0	0	0	0,03	0,04	0,93	0	0,048
2,25	7499	0	0,04	0	0,01	0,95	0	0	0	0	0	0,04	0	0,96	0,016
2,25	9353	0,01	0,04	0	0,01	0,93	0	0,01	0	0	0,38	0,04	0,57	0,01	0,042
2,25	28027	0	0,04	0	0	0,95	0,01	0	0	0	0	0,05	0	0,95	0,012
2,25	11782	0	0,04	0	0	0,96	0	0	0	0	0	0,04	0	0,96	0,008
2,25	17298	0,01	0,04	0	0	0,91	0	0	0,04	0	0,05	0,04	0,91	0	0,032
2,25	2042	0	0,04	0	0,18	0,78	0	0	0	0	0,78	0,04	0,18	0	0,046

2,25	7322	0	0	0	0,09	0,91	0	0	0	0	0,09	0,29	0,37	0,25	0,036
2,25	14396	0	0,04	0	0	0,96	0	0	0	0	0	0,04	0,93	0,03	0,026
2,25	9136	0	0,04	0	0	0,96	0	0	0	0	0	0,03	0,95	0,02	0,026
2,25	13187	0	0,04	0	0	0,87	0	0,06	0,03	0	0,9	0,04	0,01	0,05	0,038
2,25	100000	0	0,96	0	0	0,02	0	0,01	0,01	0	0,01	0,01	0,02	0,96	0,23
2,25	20951	0	0,04	0	0,05	0,89	0	0	0,01	0,01	0,9	0,03	0,02	0,05	0,036
2,25	80217	0	0,04	0	0	0,96	0	0	0	0	0	0,04	0	0,96	0,002
2,25	80150	0,01	0,04	0	0	0,91	0,04	0	0	0	0,91	0,04	0,04	0,01	0,048
2,25	38121	0	0,04	0	0,09	0,87	0	0	0	0	0,04	0,87	0,09	0	0,044
2,25	35293	0	0	0	0,08	0,92	0	0	0	0	0,07	0	0	0,93	0,048
2,25	10538	0	0,04	0	0	0,93	0,03	0	0	0	0,93	0,06	0	0,01	0,04
2,25	33932	0	0,04	0	0,03	0,93	0	0	0	0	0,03	0,04	0	0,93	0,032
2,25	26526	0	0,04	0	0	0,96	0	0	0	0	0	0,04	0,96	0	0,02
2,25	76806	0	0,04	0	0	0,96	0	0	0	0	0	0,4	0,57	0,03	0,02
2,25	22430	0	0,04	0	0,01	0,93	0,02	0	0	0	0,83	0,12	0,02	0,03	0,036
2,25	28316	0	0	0	0,38	0	0,62	0	0	0	1	0	0	0	0,042
2,25	9150	0	0,04	0	0,09	0,87	0	0	0	0	0	0,04	0,96	0	0,044
2,25	17997	0	0,04	0	0	0,96	0	0	0	0	0	0	0	1	0,008
2,25	30272	0,04	0,04	0	0	0,51	0,41	0	0	0	0,04	0,04	0,92	0	0,04
2,25	15741	0	0,04	0	0	0,95	0	0,01	0	0	0,93	0,05	0,01	0,01	0,044
2,25	10295	0,01	0,04	0	0	0,95	0	0	0	0	0,29	0,03	0,01	0,67	0,028
2,25	40445	0	0,04	0	0,09	0,85	0,01	0	0,01	0	0,05	0	0,94	0,01	0,048
2,25	13363	0	0,04	0	0	0,96	0	0	0	0	0	0,04	0,96	0	0,026
2,25	78760	0	0,04	0	0	0,96	0	0	0	0	0	0,57	0,43	0	0,024
2,25	7386	0	0,01	0	0,06	0,92	0	0,01	0	0	0,09	0,01	0,07	0,83	0,04
2,25	42941	0,08	0	0	0	0,01	0,91	0	0	0	0,97	0,01	0,01	0,01	0,048
2,25	23266	0	0,04	0	0	0,93	0	0	0,02	0,01	0	0,05	0	0,95	0,01
2,25	10521	0	0,04	0	0	0,95	0	0,01	0	0	0,95	0	0	0,05	0,046
2,25	20776	0	0,04	0	0	0,96	0	0	0	0	0	0,02	0,96	0,02	0,038
2,25	2281	0	0,04	0	0	0,96	0	0	0	0	0	0,04	0	0,96	0,012
2,25	14727	0,01	0,04	0	0	0,93	0	0,02	0	0	0	0,31	0,03	0,66	0,034
2,25	38923	0	0,04	0	0	0,96	0	0	0	0	0	0,04	0	0,96	0,01
2,25	74750	0,03	0,04	0,01	0	0,91	0,01	0	0	0	0,02	0,06	0	0,92	0,03

2,25	16523	0,01	0,04	0	0	0,95	0	0	0	0	0	0,01	0	0,99	0,026
2,25	4224	0	0,04	0	0	0,96	0	0	0	0	0	1	0	0	0,03
2,25	35528	0,02	0	0	0,05	0,47	0	0,02	0,38	0,06	0,06	0	0,45	0,49	0,048
2,25	12291	0	0,04	0	0	0,96	0	0	0	0	0	0,04	0	0,96	0,008
2,25	17799	0	0,01	0	0,12	0,87	0	0	0	0	0	0	1	0	0,048
2,25	4318	0	0,04	0	0,05	0,91	0	0	0	0	0,95	0,05	0	0	0,038
2,25	40411	0	0	0	0,11	0,81	0	0,01	0,01	0,06	0,02	0,95	0	0,03	0,04
2,25	20369	0	0,04	0	0	0,95	0	0	0	0,01	0,01	0,04	0	0,95	0,01
2,25	30586	0	0,04	0	0	0,96	0	0	0	0	0	0,04	0,96	0	0,028
2,25	4492	0,01	0,04	0	0	0,94	0	0,01	0	0	0,58	0,04	0,38	0	0,044
2,25	33434	0	0,04	0	0	0,91	0	0	0,05	0	0,79	0,07	0,14	0	0,036
2,25	8505	0	0,04	0	0	0,96	0	0	0	0	0	0,04	0,96	0	0,026
2,25	11587	0	0,04	0	0	0,96	0	0	0	0	0	0,04	0,96	0	0,026
2,25	15367	0	0,01	0	0,12	0,86	0	0	0,01	0	0	0,01	0,98	0,01	0,046
2,25	62526	0	0,04	0	0,08	0,73	0,14	0	0	0,01	0,73	0,18	0,01	0,08	0,04
2,25	24026	0	0,04	0	0	0,92	0,01	0,02	0	0,01	0,94	0,04	0,01	0,01	0,042
2,25	12034	0	0,04	0	0,03	0,93	0	0	0	0	0	0,04	0,96	0	0,044
2,25	32320	0	0,04	0	0,06	0,9	0	0	0	0	0	0,04	0,9	0,06	0,042
2,25	3418	0,01	0,04	0	0	0,94	0	0	0	0,01	0,28	0,04	0	0,68	0,044
2,25	9544	0,01	0,04	0	0	0,9	0,05	0	0	0	0	0,09	0,87	0,04	0,034
2,25	12923	0	0,04	0	0,08	0,87	0,01	0	0	0	0,01	0,04	0,88	0,07	0,034
2,25	41753	0,01	0	0	0,09	0,9	0	0	0	0	0,01	0,9	0,09	0	0,03
2,25	33900	0	0,04	0	0,02	0,93	0	0	0	0,01	0	0,03	0,03	0,94	0,024
2,25	10356	0	0	0,02	0,07	0	0,27	0	0,64	0	0,05	0,03	0,02	0,9	0,048
2,25	55998	0	0,04	0	0,02	0,94	0	0	0	0	0,94	0	0,02	0,04	0,046
2,25	13478	0	0,04	0	0	0,96	0	0	0	0	0	0,03	0,57	0,4	0,024
2,25	34483	0	0,04	0	0	0,95	0	0,01	0	0	0	0,03	0,01	0,96	0,02
2,25	20200	0,01	0,04	0	0	0,95	0	0	0	0	0	0,04	0,27	0,69	0,038
2,25	19021	0	0,04	0	0	0,96	0	0	0	0	0,01	0,01	0	0,98	0,004
2,25	5690	0	0,04	0	0	0,95	0	0	0	0,01	0,95	0,04	0,01	0	0,04
2,25	15061	0	0,04	0	0	0,92	0	0	0,04	0	0	0,04	0,96	0	0,026
2,25	42810	0	0,04	0	0	0,94	0	0	0,02	0	0	0,41	0,58	0,01	0,024
2,25	15040	0	0	0	0,07	0,91	0,01	0	0,01	0	0,07	0	0	0,93	0,046

2,25	6352	0	0	0	0,13	0,85	0	0	0,02	0	0,06	0,82	0,12	0	0,042
2,25	55993	0	0,04	0	0,06	0,89	0	0	0	0,01	0	0,04	0,9	0,06	0,048
2,25	47813	0,01	0,04	0,01	0	0,94	0	0	0	0	0,01	0,99	0	0	0,04
2,5	6961	0	0,04	0	0	0,96	0	0	0	0	0	0,04	0,96	0	0,022
2,5	20178	0,03	0,04	0	0	0,93	0	0	0	0	0,03	0,04	0,93	0	0,042
2,5	18463	0	0,04	0	0	0,94	0	0	0,01	0,01	0,01	0,03	0	0,96	0,008
2,5	100000	0	0,96	0,01	0	0,01	0	0,02	0	0	0,03	0,97	0	0	0,924
2,5	65868	0	0,04	0	0	0,96	0	0	0	0	0	0,04	0,03	0,93	0,012
2,5	8270	0	0,01	0,01	0,11	0,86	0	0	0	0,01	0,01	0,98	0	0,01	0,04
2,5	11081	0,01	0,04	0	0	0,67	0,1	0,01	0,17	0	0,02	0,86	0,12	0	0,036
2,5	100000	0	0,95	0,01	0	0	0,03	0	0,01	0	0,01	0,98	0,01	0	0,824
2,5	100000	0	0,92	0	0,06	0	0	0,02	0	0	0	0,92	0	0,08	0,69
2,5	27201	0	0,04	0	0	0,94	0	0,02	0	0	0,02	0,04	0,94	0	0,028
2,5	46550	0	0,04	0	0	0,95	0,01	0	0	0	0	0,04	0,92	0,04	0,024
2,5	19700	0	0,01	0	0,21	0,77	0	0	0	0,01	0,77	0,22	0,01	0	0,048
2,5	14304	0	0,04	0	0,01	0,94	0	0	0	0,01	0	0,1	0,89	0,01	0,028
2,5	64577	0	0,04	0,01	0,02	0,93	0	0	0	0	0,01	0,76	0,22	0,01	0,036
2,5	40392	0	0,04	0	0	0,96	0	0	0	0	0	0,04	0	0,96	0,004
2,5	10255	0	0,04	0	0	0,96	0	0	0	0	0,01	0	0,03	0,96	0,012
2,5	36214	0	0	0	0,43	0,57	0	0	0	0	1	0	0	0	0,048
2,5	10381	0	0,04	0,01	0	0,94	0	0	0	0,01	0,12	0,04	0,04	0,8	0,016
2,5	19437	0	0,04	0,01	0	0,94	0,01	0	0	0	0	0,04	0,42	0,54	0,018
2,5	24791	0	0,04	0	0	0,96	0	0	0	0	0	0,04	0,96	0	0,042
2,5	3800	0	0,04	0	0	0,96	0	0	0	0	0,08	0,04	0,77	0,11	0,018
2,5	22752	0	0,04	0	0	0,95	0	0	0,01	0	0	0,02	0,97	0,01	0,028
2,5	41415	0	0,04	0	0	0,95	0	0	0	0,01	0	0,96	0	0,04	0,03
2,5	8030	0	0	0	0,14	0,86	0	0	0	0	0,14	0,84	0	0,02	0,048
2,5	16359	0,04	0,04	0	0	0,92	0	0	0	0	0,02	0,96	0	0,02	0,048
2,5	18186	0	0	0	0,13	0,86	0	0	0,01	0	0	0,87	0,13	0	0,048
2,5	30589	0	0	0	0,2	0,77	0,01	0	0	0,02	0,77	0	0,23	0	0,048
2,5	24424	0	0,04	0	0	0,96	0	0	0	0	0	0,04	0,96	0	0,026
2,5	15246	0	0	0	0,12	0,84	0	0	0,04	0	0	0	0,96	0,04	0,038
2,5	6127	0	0,04	0	0	0,96	0	0	0	0	0	0	0	1	0,014

2,5	80814	0	0,04	0	0,01	0,94	0,01	0	0	0	0,01	0,01	0	0,98	0,01
2,5	8565	0	0,04	0,01	0	0,95	0	0	0	0	0	0,04	0	0,96	0,012
2,5	42129	0	0,04	0	0,02	0,93	0	0,01	0	0	0	0,04	0,93	0,03	0,036
2,5	18540	0	0,04	0	0	0,96	0	0	0	0	0	0,04	0	0,96	0,016
2,5	56935	0	0,04	0,01	0	0,95	0	0	0	0	0	0	0,04	0,96	0,006
2,5	15353	0	0,04	0	0,01	0,95	0	0	0	0	0,15	0,01	0	0,84	0,032
2,5	11641	0	0	0,01	0,19	0,78	0	0	0	0,02	0,78	0	0,2	0,02	0,044
2,5	22111	0,05	0	0	0	0,95	0	0	0	0	0,96	0,04	0	0	0,048
2,5	8184	0,03	0	0	0,09	0,83	0	0	0,04	0,01	0,84	0,03	0,13	0	0,048
2,5	3246	0	0,04	0	0	0,95	0	0	0,01	0	0	0,04	0,96	0	0,018
2,5	29605	0	0,04	0	0,01	0,94	0,01	0	0	0	0	0,04	0,01	0,95	0,024
2,5	32124	0	0,04	0	0	0,92	0,02	0	0,01	0,01	0,04	0,01	0,95	0	0,022
2,5	23838	0	0,04	0	0	0,9	0	0	0,06	0	0,03	0,1	0,87	0	0,028
2,5	8327	0	0,04	0	0,04	0,91	0	0,01	0	0	0	0,95	0,04	0,01	0,042
2,5	24194	0	0	0	0,12	0,84	0	0	0,02	0,02	0	0,84	0,16	0	0,048
2,5	44454	0	0,04	0	0,01	0,94	0	0	0	0,01	0,42	0,57	0	0,01	0,028
2,5	17063	0,01	0	0	0,06	0,84	0,07	0	0	0,02	0,03	0,07	0	0,9	0,046
2,5	39345	0	0,04	0	0	0,96	0	0	0	0	0	0,04	0	0,96	0,008
2,5	10442	0	0,04	0	0,02	0,94	0	0	0	0	0	0,04	0,94	0,02	0,036
2,5	26223	0	0	0	0,13	0,86	0	0	0	0,01	0	0,99	0,01	0	0,042
2,5	32492	0	0,01	0	0,21	0,7	0	0	0,07	0,01	0,71	0,04	0,21	0,04	0,048
2,5	24093	0	0,04	0,02	0	0,92	0,02	0	0	0	0,03	0,04	0,92	0,01	0,03
2,5	13812	0	0,04	0	0	0,94	0,01	0	0	0,01	0,01	0,94	0	0,05	0,016
2,5	35822	0	0,02	0	0,1	0,88	0	0	0	0	0	0,09	0,91	0	0,048
2,5	23258	0	0,04	0	0	0,85	0,09	0,02	0	0	0,86	0,04	0,1	0	0,044
2,5	8504	0	0	0	0,15	0,14	0	0	0,71	0	0	0,14	0,86	0	0,048
2,5	16320	0	0,04	0,01	0	0,95	0	0	0	0	0,03	0,02	0,05	0,9	0,016
2,5	12179	0	0,04	0	0	0,96	0	0	0	0	0,01	0,03	0,31	0,65	0,01
2,5	2867	0	0,04	0	0,01	0,93	0,01	0	0	0,01	0,94	0,04	0	0,02	0,044
2,5	12239	0,01	0	0	0,11	0,88	0	0	0	0	0,01	0,11	0,88	0	0,048
2,5	7963	0	0,04	0	0	0,96	0	0	0	0	0	1	0	0	0,022
2,5	8323	0	0,04	0	0	0,95	0	0	0	0,01	0,01	0,05	0,94	0	0,026
2,5	18742	0	0	0	0,27	0,71	0,02	0	0	0	0,71	0,02	0,27	0	0,048

2,5	28738	0	0	0	0,15	0,84	0	0	0,01	0	0,16	0,84	0	0	0,042
2,5	2806	0,01	0,04	0	0	0,92	0	0,01	0,02	0	0,92	0,07	0	0,01	0,048
2,5	18217	0	0,04	0	0	0,95	0	0,01	0	0	0	0,04	0,91	0,05	0,032
2,5	61914	0,04	0	0	0	0,76	0,17	0,03	0	0	0,04	0,03	0,18	0,75	0,048
2,5	18262	0	0	0	0,13	0,72	0,13	0	0,01	0,01	0,07	0,06	0,75	0,12	0,046
2,5	9555	0	0,04	0	0	0,96	0	0	0	0	0	0,04	0,96	0	0,03
2,5	16566	0	0,04	0,01	0	0,95	0	0	0	0	0,96	0,03	0,01	0	0,044
2,5	68249	0	0,04	0	0	0,94	0	0	0,01	0,01	0	0,04	0,86	0,1	0,02
2,5	27421	0	0,04	0,01	0	0,95	0	0	0	0	0,95	0,01	0,04	0	0,042
2,5	15468	0	0,04	0,01	0,02	0,93	0	0	0	0	0,93	0,03	0	0,04	0,044
2,5	55385	0	0	0,01	0,12	0,83	0,04	0	0	0	0,01	0,79	0,16	0,04	0,046
2,5	22628	0	0	0	0,07	0,92	0	0,01	0	0	0,07	0	0	0,93	0,046
2,5	63079	0	0	0	0,09	0,85	0,06	0	0	0	0,11	0,01	0	0,88	0,048
2,5	5061	0	0,04	0,01	0	0,94	0	0	0,01	0	0	0,05	0,92	0,03	0,032
2,5	46074	0	0,04	0	0	0,96	0	0	0	0	0	0,04	0	0,96	0,008
2,5	2054	0	0,04	0	0	0,96	0	0	0	0	0	0,04	0,96	0	0,036
2,5	66443	0,01	0,04	0	0	0,95	0	0	0	0	0	0,05	0,95	0	0,032
2,5	21139	0	0,04	0,01	0,02	0,92	0,01	0	0	0	0	0,05	0,94	0,01	0,03
2,5	3222	0	0,02	0	0,1	0,87	0	0	0,01	0	0	0,9	0	0,1	0,046
2,5	39527	0	0	0	0,17	0,53	0,3	0	0	0	0,19	0,53	0,28	0	0,048
2,5	12715	0	0,04	0	0,01	0,95	0	0	0	0	0,95	0,04	0,01	0	0,04
2,5	28380	0	0,04	0	0,03	0,92	0	0	0,01	0	0	0,27	0,73	0	0,048
2,5	36990	0	0,04	0	0	0,95	0	0,01	0	0	0,01	0,04	0,32	0,63	0,022
2,5	24865	0	0,04	0	0	0,96	0	0	0	0	0,96	0,02	0	0,02	0,036
2,5	74585	0	0,04	0,01	0,01	0,89	0	0,01	0,04	0	0	0,09	0,9	0,01	0,028
2,5	29740	0	0	0	0,23	0,28	0,49	0	0	0	0,49	0,23	0,28	0	0,044
2,5	54276	0	0,04	0	0	0,96	0	0	0	0	0	0,04	0	0,96	0,008
2,5	86352	0	0,04	0	0	0,95	0	0	0	0,01	0,01	0,04	0	0,95	0,008
2,5	11156	0	0,04	0	0	0,83	0,06	0	0,05	0,02	0,78	0,08	0,09	0,05	0,038
2,5	73821	0	0	0	0,17	0,83	0	0	0	0	0,17	0,83	0	0	0,048
2,5	38735	0	0,04	0	0	0,94	0,01	0	0,01	0	0,01	0,94	0,01	0,04	0,024
2,5	12180	0	0,04	0	0,01	0,95	0	0	0	0	0	0,04	0,95	0,01	0,038
2,5	19213	0	0	0,01	0,33	0,62	0,04	0	0	0	0,96	0,04	0	0	0,046

2,5	28409	0,01	0,04	0	0,03	0,92	0	0	0	0	0,03	0	0,93	0,04	0,048
2,5	21931	0	0,04	0	0	0,96	0	0	0	0	0	0,05	0,95	0	0,032
2,5	59251	0	0,04	0	0	0,96	0	0	0	0	0,95	0,04	0,01	0	0,038
2,5	100000	0,18	0,78	0	0	0	0,04	0	0	0	0,09	0,87	0,04	0	0,836
2,75	6673	0	0,01	0	0,08	0,91	0	0	0	0	0,08	0,01	0	0,91	0,048
2,75	17607	0	0	0	0,12	0,87	0	0	0	0,01	0,12	0	0,84	0,04	0,044
2,75	12731	0	0,04	0	0	0,96	0	0	0	0	0,12	0,04	0,84	0	0,026
2,75	44147	0,01	0,01	0	0,07	0,91	0	0	0	0	0	0,1	0,08	0,82	0,048
2,75	22849	0	0,03	0	0,08	0	0	0	0,83	0,06	0,07	0,86	0,07	0	0,048
2,75	9568	0	0,02	0	0,13	0,84	0	0	0,01	0	0,12	0,13	0,75	0	0,048
2,75	30107	0	0,04	0	0	0,96	0	0	0	0	0	0,04	0,96	0	0,022
2,75	21263	0	0,04	0	0	0,92	0,01	0	0,02	0,01	0,05	0,91	0	0,04	0,026
2,75	48482	0	0	0	0,13	0,87	0	0	0	0	0,13	0,71	0	0,16	0,048
2,75	34742	0	0	0	0,11	0,76	0,13	0	0	0	0	0,85	0,05	0,1	0,048
2,75	1774	0	0,04	0	0	0,91	0,04	0,01	0	0	0,57	0,04	0,37	0,02	0,046
2,75	17676	0	0	0	0,11	0,69	0,19	0	0	0,01	0,01	0	0,87	0,12	0,038
2,75	49001	0	0,04	0	0,01	0,95	0	0	0	0	0	0,04	0,96	0	0,024
2,75	42443	0	0,04	0	0	0,96	0	0	0	0	0	0,04	0	0,96	0,008
2,75	10208	0	0,04	0	0,03	0,93	0	0	0	0	0,05	0,04	0,91	0	0,032
2,75	61043	0	0,04	0,01	0	0,94	0	0,01	0	0	0,57	0,07	0,36	0	0,046
2,75	46271	0	0,04	0	0	0,96	0	0	0	0	0,96	0,04	0	0	0,04
2,75	4007	0	0,04	0	0	0,96	0	0	0	0	0	0,04	0	0,96	0,006
2,75	69315	0	0,04	0	0	0,96	0	0	0	0	0	0,04	0,16	0,8	0,022
2,75	8318	0,01	0,04	0	0	0,93	0	0	0	0,02	0,19	0,04	0,77	0	0,034
2,75	47322	0	0	0	0,16	0,83	0	0	0,01	0	0,18	0,81	0,01	0	0,048
2,75	13251	0	0,02	0	0,11	0,69	0	0	0	0,18	0	0,11	0,69	0,2	0,044
2,75	12179	0	0	0	0,31	0,69	0	0	0	0	0,97	0	0,03	0	0,042
2,75	81413	0,02	0,04	0	0	0	0,14	0,01	0,79	0	0,06	0,15	0,79	0	0,036
2,75	50049	0	0,04	0,02	0	0,94	0	0	0	0	0,94	0,04	0,02	0	0,046
2,75	38004	0	0,04	0	0	0,95	0	0	0,01	0	0,04	0	0	0,96	0,012
2,75	5964	0	0,04	0	0,02	0,91	0,01	0,01	0,01	0	0,97	0,01	0,01	0,01	0,038
2,75	26666	0	0,04	0	0	0,89	0,02	0	0,04	0,01	0,88	0,03	0,04	0,05	0,032
2,75	49503	0	0	0	0,13	0,74	0	0	0,12	0,01	0,14	0,84	0,01	0,01	0,04

2,75	100000	0	0,99	0	0	0	0	0	0,01	0	0	0,98	0,01	0,01	0,904
2,75	11091	0,01	0,03	0	0,09	0,85	0	0	0,01	0,01	0	0,13	0,85	0,02	0,046
2,75	36727	0	0,04	0	0	0,96	0	0	0	0	0,01	0,03	0,08	0,88	0,012
2,75	70367	0	0	0	0,12	0,02	0	0,06	0,75	0,05	0,74	0,02	0,18	0,06	0,048
2,75	9923	0	0,04	0	0	0,96	0	0	0	0	0	0,04	0	0,96	0,008
2,75	29902	0	0,04	0	0	0,92	0,04	0	0	0	0,92	0,04	0	0,04	0,03
2,75	18699	0	0,04	0	0	0,95	0	0,01	0	0	0,01	0,04	0	0,95	0,016
2,75	11865	0	0,04	0	0	0,95	0,01	0	0	0	0	0,99	0	0,01	0,014
2,75	22885	0	0,04	0	0,05	0,89	0	0	0	0,02	0	0	0,51	0,49	0,042
2,75	7118	0	0	0	0,08	0,92	0	0	0	0	0	0	0,08	0,92	0,046
2,75	21815	0,03	0,03	0	0	0,94	0	0	0	0	0,96	0,01	0,03	0	0,044
2,75	14907	0,01	0,04	0	0	0,94	0	0,01	0	0	0	0,05	0,94	0,01	0,036
2,75	10648	0,01	0,04	0,02	0	0,88	0	0	0	0,05	0,59	0,11	0,3	0	0,046
2,75	24792	0	0,04	0	0,02	0,94	0	0	0	0	0,03	0,94	0,02	0,01	0,034
2,75	68302	0	0,04	0	0	0,96	0	0	0	0	0	0	0	1	0,01
2,75	33677	0,06	0	0	0	0,94	0	0	0	0	0,06	0,02	0,92	0	0,048
2,75	75699	0	0	0	0,14	0,71	0	0	0,15	0	0,13	0,12	0,75	0	0,044
2,75	29293	0	0,04	0	0	0,95	0	0,01	0	0	0,01	0,04	0	0,95	0,012
2,75	3988	0	0,04	0	0,02	0,91	0,03	0	0	0	0,1	0,06	0,83	0,01	0,032
2,75	30754	0	0,04	0	0,03	0,93	0	0	0	0	0,87	0,03	0,03	0,07	0,038
2,75	16576	0	0,04	0	0	0,96	0	0	0	0	0	1	0	0	0,034
2,75	22658	0	0,04	0	0	0,96	0	0	0	0	0	0,04	0	0,96	0,01
2,75	100000	0,21	0,78	0	0	0	0	0	0	0,01	0	0,99	0	0,01	0,952
2,75	45410	0	0,04	0	0	0,95	0	0	0	0,01	0,01	0,04	0	0,95	0,01
2,75	61123	0	0,02	0,02	0,09	0,87	0	0	0	0	0,02	0,02	0,87	0,09	0,048
2,75	23561	0	0	0	0,13	0,85	0	0	0	0,02	0,09	0	0,91	0	0,044
2,75	51728	0	0,04	0	0	0,96	0	0	0	0	0,02	0,04	0,94	0	0,032
2,75	64155	0,01	0,04	0	0	0,93	0,02	0	0	0	0	0,01	0,01	0,98	0,014
2,75	51417	0,01	0,04	0	0,01	0,93	0	0,01	0	0	0	0,05	0,95	0	0,032
2,75	100000	0	1	0	0	0	0	0	0	0	0,04	0,96	0	0	0,954
2,75	27775	0,01	0,04	0	0	0,95	0	0	0	0	0,01	0,04	0	0,95	0,016
2,75	22116	0	0,04	0	0,1	0,86	0	0	0	0	0,15	0,78	0,02	0,05	0,048
2,75	52559	0	0,04	0	0	0,95	0,01	0	0	0	0	0,96	0,03	0,01	0,024

2,75	60765	0	0	0	0,35	0,64	0,01	0	0	0	0,99	0	0	0,01	0,048
2,75	36621	0	0,02	0,01	0,1	0,87	0	0	0	0	0	0,89	0,11	0	0,048
2,75	72300	0	0,03	0	0,14	0,75	0,03	0	0,05	0	0,92	0	0	0,08	0,038
2,75	44761	0	0,04	0	0	0,92	0	0,03	0	0,01	0,92	0	0,03	0,05	0,042
2,75	100000	0	0,98	0,02	0	0	0	0	0	0	0	0,98	0,02	0	0,868
2,75	31578	0,01	0,04	0	0	0,95	0	0	0	0	0	0,04	0,96	0	0,03
2,75	31944	0	0	0	0,09	0,91	0	0	0	0	0,33	0,09	0	0,58	0,042
2,75	25337	0	0,04	0	0	0,96	0	0	0	0	0,04	0	0	0,96	0,008
2,75	19764	0	0,04	0	0	0,96	0	0	0	0	0	0,04	0,24	0,72	0,02
2,75	35816	0	0,04	0	0	0,95	0	0	0,01	0	0	0,98	0	0,02	0,016
2,75	57124	0,01	0,04	0	0,04	0,76	0,14	0	0,01	0	0,05	0,04	0,9	0,01	0,046
2,75	23362	0	0,04	0	0,01	0,94	0	0	0	0,01	0	0,05	0,95	0	0,03
2,75	67229	0	0,04	0,01	0	0,94	0	0	0,01	0	0,01	0,03	0,94	0,02	0,02
2,75	44399	0,03	0,04	0	0	0	0,89	0,01	0	0,03	0,75	0,08	0,17	0	0,048
2,75	70062	0	0,04	0	0	0,96	0	0	0	0	0	0,04	0,96	0	0,032
2,75	10664	0,01	0,04	0	0,06	0,88	0,01	0	0	0	0,02	0,04	0,88	0,06	0,048
2,75	49470	0	0	0	0,09	0	0,45	0	0,45	0,01	0,01	0,51	0,45	0,03	0,044
2,75	25391	0	0,01	0	0,13	0,85	0,01	0	0	0	0	0,83	0,17	0	0,048
2,75	72098	0,01	0,04	0	0	0,8	0	0	0,15	0	0,15	0,04	0	0,81	0,018
2,75	28119	0	0	0,01	0,2	0,73	0,06	0	0	0	0,73	0,21	0,06	0	0,048
2,75	20647	0	0	0	0,28	0	0,71	0	0	0,01	0,7	0,29	0,01	0	0,048
2,75	65281	0	0	0	0,19	0,78	0	0,02	0,01	0	0,73	0,02	0,25	0	0,046
2,75	36216	0,03	0,03	0	0	0,92	0	0	0,02	0	0,02	0,91	0,07	0	0,034
2,75	67426	0	0,04	0	0	0,95	0	0	0	0,01	0,01	0,97	0,02	0	0,032
2,75	35227	0	0	0	0,14	0	0,3	0	0,56	0	0,56	0,1	0,14	0,2	0,048
2,75	100000	0,41	0,59	0	0	0	0	0	0	0	0,41	0,59	0	0	0,992
2,75	94033	0	0,04	0	0,01	0,92	0	0,03	0	0	0	0,15	0,84	0,01	0,044
2,75	9178	0	0,04	0	0	0,96	0	0	0	0	0	0,04	0	0,96	0,01
2,75	6268	0	0,04	0	0	0,96	0	0	0	0	0,04	0,03	0,92	0,01	0,018
2,75	21074	0	0,04	0	0	0,96	0	0	0	0	0,88	0,03	0,09	0	0,024
2,75	43373	0,01	0,04	0,01	0	0,94	0	0	0	0	0,71	0	0,29	0	0,038
2,75	100000	0,01	0,97	0	0,02	0	0	0	0	0	0,02	0,97	0	0,01	0,93
2,75	12895	0	0,01	0	0,31	0,68	0	0	0	0	0,94	0,02	0,01	0,03	0,046

2,75	25146	0	0,04	0	0	0,91	0	0	0,05	0	0,92	0,04	0	0,04	0,034
2,75	50578	0	0,02	0,03	0,07	0,52	0,35	0	0,01	0	0,07	0,54	0,36	0,03	0,042
2,75	26153	0	0,04	0	0	0,96	0	0	0	0	0,85	0,11	0	0,04	0,04
2,75	3259	0	0,04	0,01	0	0,91	0	0	0,04	0	0,04	0,04	0	0,92	0,014
2,75	16556	0	0,04	0	0,01	0,91	0	0	0,04	0	0,03	0,06	0,69	0,22	0,034
3	20635	0	0,04	0,01	0	0,95	0	0	0	0	0,03	0,95	0,02	0	0,036
3	20684	0,01	0,01	0	0,12	0,86	0	0	0	0	0	0,13	0,86	0,01	0,048
3	9074	0	0,04	0	0	0,95	0	0	0,01	0	0,02	0,1	0	0,88	0,008
3	23414	0	0,04	0	0	0,96	0	0	0	0	0	0	0,44	0,56	0,014
3	21515	0	0,01	0	0,1	0,67	0	0	0,22	0	0	0,01	0,77	0,22	0,046
3	77567	0,01	0,04	0	0,06	0,87	0,02	0	0	0	0	0,04	0,91	0,05	0,04
3	55817	0	0,04	0,01	0	0,95	0	0	0	0	0,01	0,96	0	0,03	0,026
3	12664	0	0,04	0	0,01	0,9	0,03	0	0	0,02	0,95	0,02	0	0,03	0,044
3	11745	0	0,04	0	0	0,92	0	0	0,04	0	0,04	0,04	0	0,92	0,004
3	40177	0	0	0	0,09	0,49	0,12	0	0,3	0	0,09	0	0,09	0,82	0,042
3	9475	0,06	0	0	0	0,02	0	0	0,92	0	0	0	0,94	0,06	0,046
3	82304	0	0,03	0	0,1	0,82	0	0	0,05	0	0,09	0,69	0,22	0	0,046
3	20736	0	0,04	0	0	0,96	0	0	0	0	0,96	0	0	0,04	0,042
3	33416	0	0	0	0,17	0,82	0,01	0	0	0	0,08	0	0,92	0	0,048
3	52164	0	0,04	0	0	0,96	0	0	0	0	0,59	0,38	0,03	0	0,028
3	15284	0	0,04	0	0	0,96	0	0	0	0	0	0,03	0,01	0,96	0,016
3	41017	0,05	0	0	0	0,95	0	0	0	0	0,05	0,95	0	0	0,046
3	27401	0	0	0	0,12	0,88	0	0	0	0	0,02	0	0,98	0	0,036
3	12146	0	0,04	0	0	0,96	0	0	0	0	0,96	0	0,01	0,03	0,028
3	35055	0	0,04	0,01	0	0,95	0	0	0	0	0	0,96	0,01	0,03	0,026
3	22311	0,03	0	0,01	0	0,32	0,45	0,03	0,15	0,01	0,03	0,36	0,15	0,46	0,048
3	26500	0	0,04	0	0,01	0,93	0,02	0	0	0	0	0,03	0,96	0,01	0,022
3	5919	0,05	0,03	0	0	0,6	0,31	0	0,01	0	0,01	0,04	0	0,95	0,046
3	22933	0	0,02	0	0,13	0,84	0	0	0,01	0	0,84	0,01	0	0,15	0,042
3	68895	0	0,04	0	0	0,95	0	0	0,01	0	0	0	0,99	0,01	0,026
3	14257	0	0,04	0	0,14	0,82	0	0	0	0	0,82	0	0,14	0,04	0,042
3	23621	0	0,04	0	0	0,96	0	0	0	0	0,87	0	0	0,13	0,02
3	7076	0	0,04	0	0	0,96	0	0	0	0	0,04	0	0	0,96	0,012

3	37104	0,01	0	0	0,22	0,77	0	0	0	0	0,78	0	0,22	0	0,044
3	21251	0,02	0,04	0	0,03	0	0	0	0,91	0	0	0	0,91	0,09	0,044
3	7046	0	0,04	0	0	0,92	0	0	0,04	0	0	0,92	0,04	0,04	0,034
3	66938	0,04	0,04	0	0	0,91	0,01	0	0	0	0,01	0,84	0,1	0,05	0,034
3	9748	0	0,04	0	0	0,96	0	0	0	0	0,02	0,03	0,01	0,94	0,014
3	13800	0	0,04	0	0	0,95	0,01	0	0	0	0,03	0	0	0,97	0,008
3	100000	0,07	0,91	0,02	0	0	0	0	0	0	0	0,91	0	0,09	0,766
3	32477	0,02	0,04	0	0	0,93	0	0	0,01	0	0,01	0,04	0,95	0	0,036
3	8781	0	0	0	0,12	0,8	0,08	0	0	0	0,12	0	0,8	0,08	0,048
3	9590	0	0,04	0	0	0,96	0	0	0	0	0	0	0,12	0,88	0,014
3	12112	0	0,01	0	0,12	0,7	0,17	0	0	0	0,12	0	0,88	0	0,044
3	6882	0,02	0,04	0,01	0	0,93	0	0	0	0	0	0,04	0,96	0	0,04
3	12607	0	0,03	0,02	0,06	0,89	0	0	0	0	0,91	0,01	0	0,08	0,048
3	56863	0	0,04	0	0	0,95	0	0	0,01	0	0,01	0,12	0,87	0	0,034
3	39766	0,05	0	0	0	0	0,94	0	0	0,01	0,87	0,05	0,07	0,01	0,048
3	33749	0,01	0,04	0	0	0,91	0	0,03	0	0,01	0,07	0,13	0,01	0,79	0,036
3	41206	0,01	0	0	0,19	0,79	0	0	0	0,01	0,8	0,01	0	0,19	0,038
3	8205	0	0,04	0	0	0,95	0	0,01	0	0	0,04	0,01	0,15	0,8	0,026
3	6869	0	0,04	0	0	0,95	0	0	0	0,01	0	0,03	0,95	0,02	0,018
3	18983	0	0,04	0	0,03	0,9	0	0	0,03	0	0	0,01	0,93	0,06	0,018
3	48054	0	0,04	0	0,01	0,95	0	0	0	0	0,01	0,14	0,85	0	0,024
3	27711	0	0,04	0	0	0	0,01	0	0,76	0,19	0	0,76	0,05	0,19	0,016
3	65048	0,01	0,04	0	0	0,87	0	0	0,08	0	0,01	0,07	0,01	0,91	0,024
3	8810	0	0,04	0,01	0,01	0,94	0	0	0	0	0	0,05	0,01	0,94	0,022
3	8171	0	0,04	0,01	0	0,95	0	0	0	0	0	0,05	0,95	0	0,024
3	96597	0	0,04	0	0	0,96	0	0	0	0	0,96	0,03	0	0,01	0,032
3	32824	0	0,04	0	0	0,96	0	0	0	0	0	0,04	0,96	0	0,024
3	100000	0	0,94	0	0	0,02	0	0	0,03	0,01	0,02	0,93	0,01	0,04	0,742
3	20435	0	0,04	0	0	0,96	0	0	0	0	0,96	0,04	0	0	0,036
3	2203	0	0,04	0	0	0,96	0	0	0	0	0	0,05	0	0,95	0,006
3	11797	0	0	0	0,37	0,62	0	0,01	0	0	0,97	0,02	0,01	0	0,048
3	64650	0	0,04	0	0	0,96	0	0	0	0	0	0,04	0,96	0	0,022
3	19516	0	0,04	0,01	0	0,9	0	0	0,04	0,01	0,01	0,08	0,87	0,04	0,026

3	87743	0	0,04	0	0,01	0,95	0	0	0	0	0	0,99	0	0,01	0,026
3	27917	0	0,04	0	0	0,96	0	0	0	0	0,96	0	0	0,04	0,04
3	83587	0	0,04	0,02	0	0,94	0	0	0	0	0	0,96	0	0,04	0,042
3	38209	0,03	0,03	0	0,02	0,92	0	0	0	0	0	0,97	0,01	0,02	0,046
3	44407	0	0	0	0,21	0,77	0,02	0	0	0	0,37	0,61	0,02	0	0,046
3	20060	0,02	0,04	0,01	0	0,91	0	0	0	0,02	0	0,93	0,01	0,06	0,042
3	56688	0	0,04	0	0	0,96	0	0	0	0	0	0,04	0,96	0	0,024
3	36117	0,01	0,04	0	0	0,94	0,01	0	0	0	0	0,06	0,1	0,84	0,026
3	37080	0	0,04	0	0	0,96	0	0	0	0	0	0,96	0,04	0	0,028
3	23247	0	0,04	0	0	0,96	0	0	0	0	0,11	0,04	0,85	0	0,026
3	21747	0	0,04	0	0	0,95	0,01	0	0	0	0	0,03	0	0,97	0,022
3	24001	0	0,04	0	0,1	0,86	0	0	0	0	0,7	0,04	0,26	0	0,048
3	85135	0	0,04	0	0	0,95	0	0	0	0,01	0,01	0,04	0,95	0	0,018
3	15878	0	0,04	0	0	0,93	0	0	0,03	0	0,01	0,03	0	0,96	0,008
3	75835	0	0,03	0,01	0,06	0,9	0	0	0	0	0,03	0,02	0,04	0,91	0,04
3	34661	0	0,04	0	0	0,96	0	0	0	0	0,04	0	0	0,96	0,016
3	39384	0,01	0	0	0,14	0,62	0,21	0	0	0,02	0,33	0,67	0	0	0,04
3	77565	0	0,04	0	0	0,95	0	0	0,01	0	0	0,05	0	0,95	0,014
3	36636	0	0	0	0,11	0,81	0,08	0	0	0	0,11	0,81	0	0,08	0,036
3	20701	0	0,04	0	0	0,94	0	0	0,02	0	0	0,05	0,95	0	0,016
3	17760	0	0,04	0	0	0,96	0	0	0	0	0,04	0	0	0,96	0,012
3	32889	0	0,04	0	0	0,93	0,02	0	0,01	0	0,02	0,03	0,02	0,93	0,01
3	22935	0,01	0	0	0,08	0,85	0,04	0,01	0,01	0	0,04	0,01	0,94	0,01	0,038
3	26905	0	0,04	0	0	0,95	0	0,01	0	0	0,08	0,8	0,08	0,04	0,036
3	14021	0	0,04	0	0,01	0,94	0	0	0,01	0	0,01	0,04	0,65	0,3	0,036
3	100000	0,1	0,86	0	0,01	0	0	0	0,03	0	0,04	0,86	0,1	0	0,752
3	13667	0	0,04	0	0	0,95	0	0,01	0	0	0	0,01	0,99	0	0,042
3	15944	0	0,04	0	0,01	0,95	0	0	0	0	0,01	0,02	0,02	0,95	0,018
3	100000	0,01	0,96	0	0	0,01	0,01	0	0,01	0	0,01	0,97	0	0,02	0,856
3	24778	0	0	0	0,12	0,81	0	0	0,06	0,01	0,03	0,63	0,11	0,23	0,046
3	12943	0	0,04	0	0	0,96	0	0	0	0	0	0,03	0	0,97	0,004
3	6438	0	0,04	0	0	0,9	0,05	0	0,01	0	0	0,09	0,91	0	0,034
3	54882	0,04	0,03	0	0	0,92	0,01	0	0	0	0,91	0,08	0,01	0	0,048

3	16355	0,01	0,04	0	0	0,94	0	0	0	0,01	0,05	0,89	0,06	0	0,032
3	28511	0	0,02	0	0,08	0,86	0,04	0	0	0	0,05	0,05	0,05	0,85	0,048
3	71385	0,01	0,04	0	0	0,84	0,1	0	0,01	0	0	0,03	0,95	0,02	0,028
3	17858	0	0,04	0	0	0,96	0	0	0	0	0	0,04	0,93	0,03	0,022
3	39584	0	0,04	0	0	0,96	0	0	0	0	0	1	0	0	0,022
3	13697	0	0,04	0	0	0,96	0	0	0	0	0	0,57	0,39	0,04	0,022
3,25	14667	0	0	0	0,28	0,65	0,07	0	0	0	0,87	0	0	0,13	0,04
3,25	47417	0	0	0	0,09	0,85	0,06	0	0	0	0	0	0,14	0,86	0,048
3,25	91938	0	0,04	0,01	0	0,95	0	0	0	0	0,01	0	0,03	0,96	0,008
3,25	7102	0	0,04	0	0,01	0,95	0	0	0	0	0,01	0,03	0,96	0	0,018
3,25	24695	0,03	0,03	0	0	0,94	0	0	0	0	0	0,03	0,97	0	0,046
3,25	100000	0	1	0	0	0	0	0	0	0	0	0,79	0	0,21	0,546
3,25	26059	0,02	0,04	0	0,03	0,46	0	0	0,45	0	0,06	0	0,78	0,16	0,038
3,25	31699	0	0,04	0	0	0,96	0	0	0	0	0	0	0,04	0,96	0,014
3,25	10794	0	0,04	0	0	0,88	0,01	0	0,07	0	0	0,06	0,01	0,93	0,01
3,25	43567	0	0	0,01	0,09	0,61	0,22	0	0,07	0	0	0,61	0,09	0,3	0,03
3,25	9299	0	0,04	0	0	0,96	0	0	0	0	0	0,04	0	0,96	0,002
3,25	5307	0,01	0,04	0	0	0,95	0	0	0	0	0	0,04	0	0,96	0,034
3,25	51693	0	0,04	0	0	0,94	0	0	0	0,02	0,01	0,04	0	0,95	0,004
3,25	72807	0	0,04	0	0,01	0,95	0	0	0	0	0,01	0,04	0,01	0,94	0,022
3,25	23633	0	0,04	0	0	0,96	0	0	0	0	0,04	0	0,11	0,85	0,006
3,25	25366	0	0,04	0	0,01	0,95	0	0	0	0	0,02	0,04	0,02	0,92	0,026
3,25	42944	0	0,04	0	0	0,96	0	0	0	0	0	0,17	0	0,83	0,008
3,25	7685	0,01	0,01	0	0,14	0,83	0	0,01	0	0	0,81	0,05	0	0,14	0,038
3,25	37597	0,01	0,04	0	0	0	0,02	0	0,88	0,05	0,92	0,03	0	0,05	0,044
3,25	17018	0	0,04	0	0,01	0,94	0	0,01	0	0	0,07	0,03	0,01	0,89	0,024
3,25	49823	0	0,04	0	0,01	0,94	0	0	0	0,01	0	0,04	0,01	0,95	0,016
3,25	100000	0	0,97	0,03	0	0	0	0	0	0	0,11	0,82	0,03	0,04	0,76
3,25	24428	0	0,01	0,01	0,15	0,83	0	0	0	0	0,74	0,01	0,15	0,1	0,048
3,25	33256	0,01	0	0	0,05	0,91	0	0	0,03	0	0	0,04	0,05	0,91	0,036
3,25	5382	0	0,04	0	0	0,95	0	0	0	0,01	0	0,04	0,95	0,01	0,022
3,25	60820	0	0,04	0	0	0,95	0,01	0	0	0	0,01	0	0,09	0,9	0,018
3,25	22906	0	0,04	0	0	0,96	0	0	0	0	0	0	0,96	0,04	0,02

3,25	10575	0	0,04	0	0	0,95	0	0	0,01	0	0,01	0	0,04	0,95	0,014
3,25	8552	0	0,04	0	0	0,95	0	0,01	0	0	0	0,01	0,94	0,05	0,032
3,25	31866	0	0,04	0	0	0,96	0	0	0	0	0	0,04	0	0,96	0,01
3,25	42016	0	0,04	0	0	0,95	0	0	0,01	0	0	0,2	0,02	0,78	0,018
3,25	72024	0	0,04	0	0	0,95	0	0,01	0	0	0	0,04	0,01	0,95	0,022
3,25	6884	0	0,04	0	0	0,94	0	0	0	0,02	0,02	0,02	0,95	0,01	0,028
3,25	22494	0	0	0	0,08	0,92	0	0	0	0	0,08	0,01	0	0,91	0,046
3,25	32957	0,04	0	0	0	0,96	0	0	0	0	0,04	0,22	0,74	0	0,048
3,25	61993	0	0,04	0	0	0,96	0	0	0	0	0,37	0,05	0	0,58	0,014
3,25	51126	0	0	0,06	0	0,18	0,7	0,06	0	0	0,28	0,7	0	0,02	0,042
3,25	11915	0,01	0	0	0,1	0,89	0	0	0	0	0,01	0,89	0,1	0	0,04
3,25	100000	0,02	0,96	0	0	0	0,02	0	0	0	0,3	0,68	0,02	0	0,902
3,25	100000	0,04	0,96	0	0	0	0	0	0	0	0	0,96	0,04	0	0,85
3,25	23398	0	0,04	0	0	0,96	0	0	0	0	0	0,4	0,6	0	0,018
3,25	10050	0,04	0,02	0	0	0,92	0	0,02	0	0	0,06	0,93	0,01	0	0,046
3,25	100000	0,72	0,23	0	0	0,05	0	0	0	0	0,77	0,23	0	0	0,948
3,25	55068	0,01	0,04	0	0	0,95	0	0	0	0	0	0,02	0,03	0,95	0,014
3,25	81954	0,01	0,04	0	0	0,93	0	0	0,01	0,01	0,94	0,05	0,01	0	0,048
3,25	17496	0	0,04	0	0	0,96	0	0	0	0	0,96	0,04	0	0	0,036
3,25	60686	0,01	0,04	0	0	0,95	0	0	0	0	0	0,99	0,01	0	0,042
3,25	53638	0	0,03	0	0,08	0,7	0,19	0	0	0	0,08	0,03	0,89	0	0,04
3,25	79577	0,01	0,04	0	0,01	0,93	0	0	0	0,01	0,01	0	0	0,99	0,02
3,25	48398	0	0,04	0	0,01	0,94	0	0,01	0	0	0,02	0,07	0,91	0	0,032
3,25	51468	0	0	0	0,13	0,69	0,18	0	0	0	0,14	0,18	0,68	0	0,048
3,25	46556	0	0,04	0	0,08	0,88	0	0	0	0	0,12	0,88	0	0	0,036
3,25	100000	0,15	0,27	0,01	0	0,49	0	0,08	0	0	0,09	0,36	0,06	0,49	0,272
3,25	12884	0	0,04	0	0	0,94	0	0	0	0,02	0,01	0,01	0,94	0,04	0,012
3,25	100000	0	0,99	0	0	0	0	0	0,01	0	0,04	0,95	0	0,01	0,906
3,25	61441	0,01	0,03	0	0,02	0,93	0	0,01	0	0	0,01	0,07	0,45	0,47	0,048
3,25	22226	0,05	0,03	0	0	0,92	0	0	0	0	0,16	0,03	0	0,81	0,048
3,25	6776	0	0,04	0	0	0,95	0,01	0	0	0	0,01	0,03	0	0,96	0,006
3,25	49680	0	0,04	0	0	0,96	0	0	0	0	0,04	0,96	0	0	0,026
3,25	4729	0,01	0,04	0	0,04	0,91	0	0	0	0	0,91	0,04	0	0,05	0,048

3,25	63642	0	0,04	0	0	0,96	0	0	0	0	0	0,04	0,96	0	0,016
3,25	18025	0,01	0,04	0	0	0,94	0	0	0,01	0	0,95	0,04	0	0,01	0,044
3,25	90022	0	0,01	0,06	0,03	0,69	0,21	0	0	0	0,28	0,69	0,03	0	0,048
3,25	37067	0	0,04	0	0	0,95	0	0	0	0,01	0,05	0	0,95	0	0,03
3,25	10290	0	0,03	0	0,08	0,85	0,01	0	0,01	0,02	0,01	0,9	0	0,09	0,046
3,25	33912	0	0,04	0	0	0,87	0,06	0	0,02	0,01	0,02	0	0,93	0,05	0,024
3,25	62858	0,01	0,04	0	0,03	0,92	0	0	0	0	0,38	0,04	0,02	0,56	0,04
3,25	3868	0	0,04	0	0,01	0,94	0	0,01	0	0	0,04	0,05	0,08	0,83	0,014
3,25	100000	0	0,99	0	0	0	0	0,01	0	0	0,01	0,99	0	0	0,932
3,25	100000	0	0,97	0	0,02	0	0	0,01	0	0	0	0,97	0,03	0	0,812
3,25	24340	0	0,04	0	0	0,96	0	0	0	0	0	0,01	0,13	0,86	0,016
3,25	24612	0	0,04	0	0	0,76	0,2	0	0	0	0,24	0	0,22	0,54	0,018
3,25	48646	0	0	0	0,12	0,49	0,39	0	0	0	0,12	0	0,87	0,01	0,048
3,25	25201	0	0,04	0	0,11	0,85	0	0	0	0	0	0,89	0,1	0,01	0,048
3,25	12491	0,01	0,04	0	0	0,95	0	0	0	0	0,39	0	0,56	0,05	0,04
3,25	100000	0	1	0	0	0	0	0	0	0	0	1	0	0	0,96
3,25	80027	0	0,04	0	0	0,96	0	0	0	0	0	0	0,96	0,04	0,026
3,25	72317	0	0,04	0	0,01	0,9	0,02	0	0,03	0	0,92	0,04	0,04	0	0,044
3,25	8746	0,01	0,01	0	0,08	0,9	0	0	0	0	0	0,44	0,56	0	0,046
3,25	61935	0	0,04	0	0	0,95	0	0,01	0	0	0	0,04	0,96	0	0,026
3,25	45009	0	0,04	0,01	0	0	0,02	0	0,93	0	0,01	0,85	0	0,14	0,024
3,25	44621	0	0,04	0	0	0,95	0	0	0,01	0	0,01	0	0,8	0,19	0,03
3,25	16322	0	0,04	0	0,01	0,93	0	0	0	0,02	0,01	0,04	0,02	0,93	0,012
3,25	8828	0	0	0	0,14	0,86	0	0	0	0	0	0,86	0,14	0	0,044
3,25	37140	0	0,04	0	0	0,94	0	0,02	0	0	0,03	0,04	0,93	0	0,034
3,25	100000	0,28	0,71	0	0	0	0	0	0,01	0	0,28	0,71	0	0,01	0,954
3,25	22458	0	0,04	0	0	0,96	0	0	0	0	0,96	0	0	0,04	0,034
3,25	20454	0	0,04	0	0	0,96	0	0	0	0	0	0	0,96	0,04	0,02
3,25	100000	0	0,98	0,02	0	0	0	0	0	0	0	0,91	0	0,09	0,718
3,25	8480	0	0	0	0,26	0,2	0,54	0	0	0	0,8	0	0	0,2	0,044
3,25	87154	0	0,04	0	0	0,96	0	0	0	0	0	0,03	0	0,97	0,008
3,25	92103	0	0,04	0	0	0,96	0	0	0	0	0	0,04	0	0,96	0,01
3,25	46290	0	0,04	0	0	0,95	0	0	0	0,01	0,03	0,01	0	0,96	0,012

3,25	18785	0	0,04	0	0,03	0,93	0	0	0	0	0	0,03	0,03	0,94	0,034
3,25	90958	0	0,04	0	0	0,96	0	0	0	0	0	0,04	0,96	0	0,024
3,25	72326	0	0,04	0,01	0	0,95	0	0	0	0	0	0	0,95	0,05	0,03
3,25	100000	0	0,96	0,02	0	0	0,01	0	0,01	0	0,03	0,96	0,01	0	0,906
3,25	12950	0,06	0	0	0,02	0,92	0	0	0	0	0,84	0,1	0	0,06	0,048
3,25	14951	0	0,04	0	0	0,96	0	0	0	0	0,03	0	0	0,97	0,016
3,25	24500	0,03	0,02	0	0	0,93	0	0,01	0	0,01	0,93	0,05	0,01	0,01	0,046
3,5	27809	0	0,04	0	0	0,96	0	0	0	0	0	0	0,04	0,96	0,004
3,5	70590	0	0,04	0	0	0,95	0	0	0,01	0	0,01	0,04	0,95	0	0,03
3,5	100000	0	1	0	0	0	0	0	0	0	0	0,78	0	0,22	0,602
3,5	23465	0	0,04	0	0,04	0,92	0	0	0	0	0	0,92	0	0,08	0,04
3,5	22805	0,03	0,04	0	0	0,9	0,01	0	0,02	0	0,54	0,04	0,4	0,02	0,048
3,5	46386	0	0,04	0	0,08	0,88	0	0	0	0	0	0,04	0,08	0,88	0,048
3,5	75628	0,01	0,04	0	0,01	0,93	0	0,01	0	0	0	0,16	0,82	0,02	0,038
3,5	69916	0	0,04	0,01	0,01	0,94	0	0	0	0	0	0,03	0,03	0,94	0,024
3,5	18607	0	0,04	0	0,02	0,93	0	0	0,01	0	0,93	0,07	0	0	0,04
3,5	9689	0,05	0,04	0	0	0,91	0	0	0	0	0,04	0,05	0	0,91	0,048
3,5	77386	0	0,04	0	0	0,92	0	0	0,04	0	0	0,08	0	0,92	0,012
3,5	21374	0	0,03	0	0,14	0,5	0,33	0	0	0	0,36	0,64	0	0	0,044
3,5	7046	0	0,04	0	0,03	0,85	0,08	0	0	0	0	0,1	0,9	0	0,032
3,5	50592	0,01	0,04	0	0	0,93	0,01	0,01	0	0	0	0,97	0	0,03	0,036
3,5	7035	0	0	0	0,21	0,72	0	0,01	0,06	0	0,72	0,01	0,2	0,07	0,046
3,5	100000	0	0,98	0	0,01	0	0	0	0	0,01	0	0,98	0,01	0,01	0,86
3,5	10723	0,01	0,04	0	0	0,94	0	0,01	0	0	0	0,01	0,04	0,95	0,024
3,5	89419	0,03	0	0,02	0	0,93	0	0,02	0	0	0,03	0,94	0,03	0	0,034
3,5	25992	0	0,04	0,01	0	0,92	0	0	0,03	0	0,02	0,03	0,93	0,02	0,028
3,5	13008	0	0,04	0	0	0,81	0	0,03	0,12	0	0,03	0,04	0,81	0,12	0,044
3,5	72632	0,05	0	0	0	0,81	0	0	0,14	0	0,17	0,02	0,81	0	0,036
3,5	14553	0	0,04	0	0	0,96	0	0	0	0	0,24	0,04	0	0,72	0,028
3,5	35503	0	0,04	0,01	0	0,94	0	0	0,01	0	0	0,04	0,01	0,95	0,012
3,5	4432	0	0,04	0	0	0,96	0	0	0	0	0,01	0,03	0,96	0	0,03
3,5	100000	0	0,95	0	0	0,01	0,02	0,02	0	0	0,04	0,96	0	0	0,86
3,5	50277	0,05	0	0,01	0	0,93	0	0,01	0	0	0	0,1	0,87	0,03	0,048

3,5	12513	0	0,04	0	0	0,96	0	0	0	0	0	0	0,96	0,04	0,012
3,5	7788	0	0,04	0	0	0,96	0	0	0	0	0,96	0,04	0	0	0,046
3,5	73491	0,04	0,02	0	0,01	0,93	0	0	0	0	0,98	0,01	0,01	0	0,048
3,5	43231	0	0,04	0	0,02	0,94	0	0	0	0	0,66	0,05	0	0,29	0,042
3,5	44378	0	0,04	0	0,01	0,86	0,01	0,03	0,05	0	0,91	0,06	0,01	0,02	0,046
3,5	100000	0	1	0	0	0	0	0	0	0	0	1	0	0	0,97
3,5	11632	0	0,04	0	0	0,96	0	0	0	0	0	0,04	0,96	0	0,024
3,5	35644	0	0,04	0	0	0,93	0,02	0	0	0,01	0,81	0,02	0,16	0,01	0,034
3,5	27879	0	0,04	0	0	0,93	0	0,03	0	0	0	0,04	0	0,96	0,034
3,5	2544	0	0,03	0	0,11	0,83	0,03	0	0	0	0,61	0,05	0	0,34	0,044
3,5	15115	0	0,04	0	0	0,94	0	0	0,02	0	0,02	0,04	0	0,94	0,014
3,5	44729	0,02	0,03	0	0	0,9	0,02	0,01	0,02	0	0,9	0,05	0	0,05	0,048
3,5	100000	0,11	0,89	0	0	0	0	0	0	0	0,1	0,9	0	0	0,978
3,5	92713	0	0	0	0,11	0,12	0,76	0	0	0,01	0,12	0,5	0,38	0	0,048
3,5	100000	0	0,99	0	0,01	0	0	0	0	0	0	1	0	0	0,926
3,5	75330	0,05	0	0,01	0	0,31	0,59	0	0,04	0	0,06	0,01	0,31	0,62	0,042
3,5	39383	0	0,04	0	0	0,94	0	0	0,02	0	0	0	0	1	0,012
3,5	16502	0	0,04	0,01	0,01	0,91	0	0,02	0	0,01	0,91	0,02	0,04	0,03	0,034
3,5	26476	0,05	0	0	0	0	0,94	0	0	0,01	0,91	0,05	0	0,04	0,046
3,5	17826	0	0,04	0	0	0,95	0	0,01	0	0	0	0,04	0,05	0,91	0,02
3,5	33039	0	0,04	0	0	0,93	0	0,02	0	0,01	0,06	0	0,93	0,01	0,022
3,5	72631	0	0,04	0	0	0,96	0	0	0	0	0	0,04	0	0,96	0,012
3,5	25830	0	0,04	0,02	0	0,94	0	0	0	0	0	0,04	0,94	0,02	0,036
3,5	36159	0,01	0,04	0	0,01	0,94	0	0	0	0	0	0,05	0,95	0	0,032
3,5	95265	0	0,04	0	0,01	0,94	0	0	0	0,01	0,01	0,03	0,95	0,01	0,034
3,5	29103	0	0	0	0,17	0,82	0,01	0	0	0	0,37	0	0,63	0	0,048
3,5	85951	0,01	0,04	0	0	0,92	0,02	0	0	0,01	0	0	0,98	0,02	0,022
3,5	95997	0	0,04	0	0	0,96	0	0	0	0	0	0,04	0,96	0	0,032
3,5	51679	0	0,03	0	0,09	0,87	0	0,01	0	0	0,21	0,33	0,37	0,09	0,046
3,5	10136	0	0,04	0,01	0	0,92	0	0,03	0	0	0,95	0,01	0,03	0,01	0,048
3,5	63005	0	0,04	0	0,09	0,85	0,01	0	0,01	0	0,12	0,04	0,84	0	0,044
3,5	14955	0	0,04	0	0	0,96	0	0	0	0	0	1	0	0	0,034
3,5	100000	0	0,99	0	0	0	0	0	0	0,01	0	0,99	0,01	0	0,898

3,5	26585	0	0,03	0	0,07	0,89	0	0,01	0	0	0,07	0,03	0,26	0,64	0,048
3,5	33400	0	0	0	0,1	0,63	0,27	0	0	0	0,24	0	0,1	0,66	0,046
3,5	81596	0	0,04	0	0	0,96	0	0	0	0	0,01	0,03	0,96	0	0,03
3,5	17043	0	0,04	0,03	0,02	0,81	0,04	0	0,06	0	0,04	0,12	0,82	0,02	0,044
3,5	89701	0	0,04	0	0	0,96	0	0	0	0	0	0	0,89	0,11	0,016
3,5	41884	0	0,04	0	0	0,96	0	0	0	0	0,96	0,02	0	0,02	0,044
3,5	47229	0,05	0,01	0	0	0,93	0	0,01	0	0	0,03	0,96	0,01	0	0,048
3,5	21323	0	0	0	0	0	0,87	0,13	0	0	0,24	0,76	0	0	0,048
3,5	51566	0	0	0	0,08	0,01	0,24	0	0,67	0	0	0,08	0,25	0,67	0,046
3,5	27185	0	0,04	0	0	0,96	0	0	0	0	0,96	0	0	0,04	0,036
3,5	6286	0	0,04	0	0	0,95	0	0	0	0,01	0,28	0,04	0,68	0	0,026
3,5	29344	0	0,04	0	0	0,95	0,01	0	0	0	0,01	0,04	0,95	0	0,018
3,5	13732	0	0,04	0	0,05	0	0,02	0	0,89	0	0,02	0	0,07	0,91	0,046
3,5	82154	0	0,04	0	0	0,96	0	0	0	0	0	0,04	0	0,96	0,014
3,5	18555	0	0,04	0	0	0,96	0	0	0	0	0	0,01	0,46	0,53	0,008
3,5	14024	0	0,04	0	0	0,95	0	0,01	0	0	0,01	0,01	0	0,98	0,014
3,5	100000	0,01	0,77	0	0,22	0	0	0	0	0	0,77	0	0,23	0	0,682
3,5	20912	0	0,04	0	0	0,96	0	0	0	0	0,02	0,02	0	0,96	0,008
3,5	46707	0	0,01	0	0,34	0,64	0	0	0,01	0	0,99	0	0	0,01	0,04
3,5	41528	0	0,01	0	0,2	0,01	0,05	0	0,73	0	0,73	0,05	0,21	0,01	0,046
3,5	100000	0,13	0,8	0,07	0	0	0	0	0	0	0,2	0,8	0	0	0,982
3,5	95252	0	0,04	0	0	0,96	0	0	0	0	0,96	0,04	0	0	0,036
3,5	22936	0	0	0	0,11	0,87	0	0	0,02	0	0	0,13	0,87	0	0,044
3,5	41589	0,01	0,04	0	0,01	0,94	0	0	0	0	0	0,04	0,01	0,95	0,032
3,5	21353	0	0,04	0	0	0,96	0	0	0	0	0,01	0	0,26	0,73	0,014
3,5	41258	0	0	0	0,15	0,85	0	0	0	0	0,15	0	0,85	0	0,042
3,5	77082	0	0,04	0	0,03	0,79	0,05	0,01	0,04	0,04	0,48	0,04	0	0,48	0,032
3,5	12763	0	0,04	0	0	0,92	0,04	0	0	0	0,01	0,14	0,82	0,03	0,038
3,5	44375	0	0,04	0	0	0,96	0	0	0	0	0	0,07	0	0,93	0,006
3,5	19600	0,01	0,01	0,04	0,03	0,37	0,01	0	0,53	0	0,01	0,16	0,46	0,37	0,046
3,5	38180	0	0	0	0,14	0,81	0,01	0	0,04	0	0,32	0,19	0,49	0	0,048
3,5	21331	0	0,04	0	0	0,96	0	0	0	0	0	0,04	0	0,96	0,006
3,5	16881	0	0,04	0	0	0,96	0	0	0	0	0,21	0,04	0,75	0	0,028

3,5	88504	0,06	0	0	0	0,82	0,12	0	0	0	0,17	0	0,82	0,01	0,048
3,5	21656	0	0,01	0	0,07	0,49	0,02	0	0,38	0,03	0,02	0,05	0	0,93	0,046
3,5	67174	0	0,04	0	0	0,96	0	0	0	0	0,01	0,02	0,02	0,95	0,008
3,5	42731	0	0,04	0	0	0,96	0	0	0	0	0	0,03	0,97	0	0,018
3,5	26286	0	0,04	0	0	0,96	0	0	0	0	0	0,03	0	0,97	0,006
3,5	50336	0	0,04	0	0	0,95	0	0	0	0,01	0	0	0,01	0,99	0,016
3,5	15910	0,01	0,04	0	0	0,94	0	0,01	0	0	0	0,58	0,4	0,02	0,038
3,5	18395	0	0,04	0	0	0,96	0	0	0	0	0,01	0,03	0,72	0,24	0,012
3,75	47278	0	0,04	0	0	0,95	0	0	0	0,01	0	0,05	0,95	0	0,032
3,75	65804	0,01	0,04	0,01	0	0,94	0	0	0	0	0,36	0,64	0	0	0,044
3,75	13611	0,01	0,03	0	0,17	0,79	0	0	0	0	0,96	0,02	0,02	0	0,048
3,75	8902	0	0	0	0,35	0,61	0,03	0,01	0	0	0,97	0,03	0	0	0,048
3,75	20521	0,02	0,04	0	0,06	0,88	0	0	0	0	0,09	0,05	0,8	0,06	0,044
3,75	52204	0	0	0	0,11	0,05	0	0	0,84	0	0,14	0,79	0	0,07	0,046
3,75	82400	0	0,04	0	0	0,93	0,01	0,01	0	0,01	0,01	0,05	0	0,94	0,014
3,75	4793	0	0,04	0	0	0,96	0	0	0	0	0,03	0,21	0,01	0,75	0,014
3,75	26695	0,01	0,04	0,01	0	0,94	0	0	0	0	0,9	0,04	0,01	0,05	0,042
3,75	20998	0	0,04	0	0	0,96	0	0	0	0	0	0,03	0,01	0,96	0,006
3,75	36107	0	0	0	0,08	0,85	0,04	0	0,03	0	0,04	0,11	0	0,85	0,048
3,75	100000	0	0	0	1	0	0	0	0	0	0	0	1	0	0,468
3,75	16513	0	0,01	0,02	0,2	0,77	0	0	0	0	0,97	0,01	0,02	0	0,048
3,75	14640	0	0,04	0	0	0,96	0	0	0	0	0	0,66	0	0,34	0,01
3,75	53554	0	0,04	0	0,1	0,67	0	0	0,19	0	0,65	0,06	0,1	0,19	0,048
3,75	100000	0	1	0	0	0	0	0	0	0	0	1	0	0	0,978
3,75	100000	0	0,9	0,07	0,03	0	0	0	0	0	0,03	0,93	0	0,04	0,77
3,75	100000	0	0,93	0,07	0	0	0	0	0	0	0	0,93	0	0,07	0,736
3,75	100000	0	0,98	0	0,01	0	0	0	0	0,01	0,01	0,98	0	0,01	0,896
3,75	56268	0	0,04	0	0	0,96	0	0	0	0	0,96	0,03	0	0,01	0,034
3,75	100000	0	0,97	0	0	0,01	0	0	0	0,02	0,01	0,97	0,02	0	0,846
3,75	25062	0,06	0	0	0	0,66	0,25	0,01	0	0,02	0,66	0,07	0,26	0,01	0,046
3,75	56409	0,01	0,01	0	0,12	0,78	0,08	0	0	0	0,58	0,01	0	0,41	0,046
3,75	14901	0	0	0,01	0,18	0,81	0	0	0	0	0,81	0	0,18	0,01	0,044
3,75	8905	0,02	0,04	0	0	0,91	0,01	0,01	0	0,01	0,02	0,03	0,91	0,04	0,042

3,75	56192	0	0	0	0,3	0,67	0	0,02	0	0,01	0,96	0,01	0,03	0	0,048
3,75	40744	0,02	0,04	0	0	0,92	0	0	0,02	0	0	0,04	0	0,96	0,024
3,75	29042	0,04	0,03	0	0	0,92	0	0,01	0	0	0,04	0,04	0	0,92	0,04
3,75	22454	0,05	0,03	0	0,01	0,91	0	0	0	0	0	0,04	0,9	0,06	0,046
3,75	34441	0	0	0	0,22	0	0,78	0	0	0	0,71	0	0,29	0	0,046
3,75	28476	0	0	0	0,11	0,78	0,1	0,01	0	0	0,18	0,72	0	0,1	0,048
3,75	37738	0	0,04	0,01	0,02	0,93	0	0	0	0	0	0,04	0,61	0,35	0,034
3,75	15563	0	0,04	0	0,01	0,94	0,01	0	0	0	0,96	0,01	0,02	0,01	0,044
3,75	8876	0	0,04	0	0	0,96	0	0	0	0	0	0,04	0,96	0	0,022
3,75	66437	0,01	0,04	0	0	0,94	0	0	0,01	0	0,05	0,01	0	0,94	0,01
3,75	21313	0,01	0,04	0	0	0,94	0	0	0,01	0	0,96	0,02	0	0,02	0,042
3,75	43862	0	0	0	0,19	0	0	0	0,8	0,01	0,8	0	0,01	0,19	0,034
3,75	16738	0	0,04	0	0,03	0,93	0	0	0	0	0	0,96	0,04	0	0,034
3,75	100000	0,25	0,74	0	0	0	0,01	0	0	0	0,01	0,74	0	0,25	0,654
3,75	10032	0,01	0,04	0	0	0,95	0	0	0	0	0	0,02	0,04	0,94	0,024
3,75	24669	0	0,04	0	0	0,89	0,04	0	0,03	0	0,08	0,02	0,88	0,02	0,028
3,75	4828	0,05	0,01	0	0	0,94	0	0	0	0	0,98	0,02	0	0	0,046
3,75	100000	0,14	0,86	0	0	0	0	0	0	0	0,14	0,86	0	0	0,97
3,75	67693	0	0,03	0,01	0,09	0,73	0	0	0,14	0	0,76	0	0,15	0,09	0,046
3,75	13572	0	0,04	0,01	0	0,95	0	0	0	0	0	0,04	0,95	0,01	0,02
3,75	18635	0	0	0,03	0,1	0,22	0	0	0,65	0	0,25	0,08	0,67	0	0,048
3,75	31776	0	0,04	0	0	0,92	0,04	0	0	0	0,03	0	0,05	0,92	0,014
3,75	67549	0	0	0	0,14	0,77	0,09	0	0	0	0	0,09	0,91	0	0,048
3,75	6684	0	0,04	0	0	0,96	0	0	0	0	0	0,01	0	0,99	0,008
3,75	100000	0	0,99	0	0	0,01	0	0	0	0	0	0,98	0,01	0,01	0,88
3,75	10890	0	0,04	0	0	0,93	0	0,01	0	0,02	0,94	0,03	0,03	0	0,044
3,75	93302	0	0,04	0	0	0,95	0	0	0,01	0	0,04	0,09	0	0,87	0,014
3,75	83356	0	0	0	0,3	0	0,7	0	0	0	0,7	0,3	0	0	0,042
3,75	50199	0,03	0,03	0,01	0	0,93	0	0	0	0	0,42	0,05	0,5	0,03	0,03
3,75	25777	0,01	0,04	0	0	0,94	0,01	0	0	0	0,01	0,05	0	0,94	0,022
3,75	85244	0	0	0,01	0,11	0	0,03	0	0,79	0,06	0,1	0	0,9	0	0,048
3,75	100000	0	0	0	1	0	0	0	0	0	1	0	0	0	0,162
3,75	24395	0	0,04	0	0	0,95	0,01	0	0	0	0	0,01	0,99	0	0,022

3,75	66437	0	0	0	0,09	0,56	0,3	0	0,05	0	0,34	0	0,09	0,57	0,04
3,75	29811	0	0,04	0	0	0,96	0	0	0	0	0	0	0,94	0,06	0,03
3,75	47084	0	0	0	0,19	0	0,81	0	0	0	0,81	0	0	0,19	0,042
3,75	34347	0	0	0	0,18	0,33	0,48	0,01	0	0	0,51	0,49	0	0	0,046
3,75	72956	0	0,03	0	0,08	0,89	0	0	0	0	0	0,48	0	0,52	0,046
3,75	32682	0	0,04	0	0	0,96	0	0	0	0	0,93	0,04	0	0,03	0,044
3,75	52059	0	0,04	0	0	0,29	0,66	0	0	0,01	0,38	0,54	0,05	0,03	0,03
3,75	41983	0	0,04	0	0	0,95	0,01	0	0	0	0,04	0	0,01	0,95	0,01
3,75	69943	0,01	0,04	0	0,03	0,92	0	0	0	0	0	0,07	0,93	0	0,048
3,75	28721	0,05	0	0	0,02	0,92	0	0	0	0,01	0,17	0	0,82	0,01	0,048
3,75	37705	0	0	0	0,14	0,78	0,01	0	0,07	0	0,11	0,03	0,74	0,12	0,046
3,75	40955	0	0,04	0	0	0,94	0,01	0	0,01	0	0,01	0	0	0,99	0,006
3,75	15110	0	0,04	0	0,04	0,92	0	0	0	0	0,92	0,04	0,04	0	0,042
3,75	21908	0	0,04	0	0,17	0,77	0	0	0,02	0	0,78	0,04	0,02	0,16	0,048
3,75	10478	0	0	0	0,4	0,59	0,01	0	0	0	0,99	0	0,01	0	0,048
3,75	62752	0	0,04	0,01	0,02	0,7	0,22	0	0,01	0	0,87	0,11	0	0,02	0,044
3,75	33765	0	0,04	0	0,09	0,78	0	0	0,09	0	0,06	0,9	0	0,04	0,032
3,75	100000	0,1	0,88	0	0	0,01	0,01	0	0	0	0	0,89	0,01	0,1	0,71
3,75	21286	0	0,04	0	0	0,95	0	0	0,01	0	0,95	0,05	0	0	0,036
3,75	45758	0	0,04	0	0	0,96	0	0	0	0	0	0	0,04	0,96	0,004
3,75	82949	0	0,04	0	0	0,96	0	0	0	0	0,07	0	0	0,93	0,018
3,75	29622	0	0,04	0	0	0,96	0	0	0	0	0,96	0,04	0	0	0,034
3,75	100000	0	0,83	0,15	0	0,02	0	0	0	0	0,16	0,82	0,02	0	0,894
3,75	53367	0,02	0,04	0	0	0,94	0	0	0	0	0,04	0,92	0,04	0	0,038
3,75	1862	0	0,04	0	0	0,94	0,01	0	0,01	0	0,01	0,05	0,94	0	0,01
3,75	62144	0	0,04	0	0,01	0,93	0	0	0	0,02	0	0,06	0	0,94	0,018
3,75	100000	0	0,96	0,01	0	0,01	0,01	0	0,01	0	0,63	0,34	0,01	0,02	0,924
3,75	16167	0	0	0	0,14	0,81	0,02	0,02	0	0,01	0,02	0,01	0,97	0	0,048
3,75	35611	0	0,03	0	0,26	0,71	0	0	0	0	1	0	0	0	0,046
3,75	33199	0,01	0,04	0	0	0,93	0,02	0	0	0	0	0,02	0,93	0,05	0,032
3,75	35525	0	0,04	0	0	0,96	0	0	0	0	0	0,04	0,96	0	0,032
3,75	40274	0	0,04	0	0	0,96	0	0	0	0	0,96	0	0	0,04	0,042
3,75	9112	0	0,02	0	0,09	0	0,49	0	0,39	0,01	0	0,05	0,51	0,44	0,048

3,75	98219	0	0,04	0	0	0,95	0	0	0,01	0	0	0,01	0,99	0	0,026
3,75	37172	0	0,04	0	0	0,96	0	0	0	0	0	0,04	0,12	0,84	0,026
3,75	79431	0	0,04	0	0	0,94	0,01	0	0,01	0	0	0,04	0,23	0,73	0,01
3,75	55078	0	0,04	0,01	0	0,94	0	0,01	0	0	0,05	0,02	0	0,93	0,022
3,75	6896	0	0	0	0,1	0,81	0,09	0	0	0	0,1	0	0,27	0,63	0,04
3,75	22950	0	0,04	0	0	0,96	0	0	0	0	0	0	0,99	0,01	0,014
3,75	10557	0	0,04	0	0	0,95	0	0	0,01	0	0,42	0,01	0	0,57	0,02
3,75	30673	0	0,04	0	0,03	0	0,93	0	0	0	0,97	0	0,03	0	0,038
3,75	94230	0	0,04	0	0,07	0,86	0,03	0	0	0	0	0,06	0,94	0	0,046
4	74813	0	0	0	0,09	0,89	0,01	0	0	0,01	0	0,21	0,1	0,69	0,046
4	28764	0	0,04	0	0	0,96	0	0	0	0	0,04	0	0	0,96	0,012
4	74685	0	0,04	0	0	0,84	0	0,01	0,11	0	0,15	0	0,08	0,77	0,02
4	45917	0,01	0,01	0	0,08	0,9	0	0	0	0	0	0,92	0	0,08	0,046
4	34017	0	0,04	0,02	0	0,89	0,01	0,04	0	0	0,89	0,02	0,02	0,07	0,048
4	48334	0	0,04	0	0	0,56	0,05	0,02	0,33	0	0	0,6	0,07	0,33	0,042
4	68427	0	0,04	0	0	0,96	0	0	0	0	0,02	0,02	0,96	0	0,032
4	37256	0,01	0,04	0	0	0,94	0	0,01	0	0	0,12	0,04	0,84	0	0,046
4	58207	0	0,04	0	0	0,96	0	0	0	0	0,6	0	0,4	0	0,02
4	9532	0	0,04	0,01	0	0,91	0	0	0,04	0	0	0,95	0,04	0,01	0,022
4	24447	0,06	0	0	0	0,93	0	0	0,01	0	0,83	0,17	0	0	0,048
4	100000	0	1	0	0	0	0	0	0	0	0	1	0	0	0,964
4	8817	0,01	0,04	0	0	0,91	0	0	0,02	0,02	0,26	0,04	0,69	0,01	0,046
4	29159	0	0,04	0	0	0,94	0,02	0	0	0	0,96	0	0,04	0	0,04
4	27829	0	0	0	0,11	0,89	0	0	0	0	0	0	1	0	0,048
4	31394	0,01	0	0	0,07	0,91	0	0	0,01	0	0,07	0	0,02	0,91	0,048
4	17042	0,02	0,04	0	0	0,94	0	0	0	0	0,02	0,04	0,94	0	0,04
4	55564	0	0,04	0	0,02	0,94	0	0	0	0	0,94	0,04	0,01	0,01	0,048
4	23755	0	0,04	0	0,01	0,94	0,01	0	0	0	0,01	0	0,45	0,54	0,036
4	67203	0	0,04	0,01	0	0,95	0	0	0	0	0,02	0,03	0	0,95	0,012
4	100000	0	1	0	0	0	0	0	0	0	0,03	0,97	0	0	0,948
4	78229	0	0,04	0	0	0,96	0	0	0	0	0	0,03	0,97	0	0,022
4	15071	0	0,04	0	0,02	0,92	0	0	0,01	0,01	0	0,06	0,02	0,92	0,016
4	22565	0	0,04	0	0	0,96	0	0	0	0	0	0	1	0	0,02

4	59872	0	0,04	0	0	0,96	0	0	0	0	0	0,04	0,02	0,94	0,006
4	75434	0,05	0	0	0,03	0,92	0	0	0	0	0,05	0,92	0	0,03	0,048
4	90152	0	0,04	0	0,06	0,87	0	0	0,03	0	0	0,08	0,9	0,02	0,042
4	40915	0	0,04	0	0	0,91	0	0,01	0,03	0,01	0,03	0,01	0,04	0,92	0,016
4	18573	0	0,04	0	0,03	0,9	0,01	0	0,02	0	0,54	0,4	0	0,06	0,048
4	44933	0	0,04	0	0	0,96	0	0	0	0	0	0,02	0	0,98	0,006
4	27925	0,02	0,04	0	0	0,93	0	0	0	0,01	0,86	0,05	0,07	0,02	0,044
4	36144	0,06	0	0	0	0,93	0	0	0,01	0	0	0,93	0,07	0	0,048
4	29992	0	0,04	0	0,01	0,95	0	0	0	0	0,99	0,01	0	0	0,032
4	43410	0	0,04	0	0	0,94	0,01	0	0	0,01	0,01	0,05	0,82	0,12	0,028
4	41551	0	0,04	0	0	0	0,03	0	0,93	0	0,04	0	0	0,96	0,004
4	3586	0,01	0	0	0,05	0,89	0	0,05	0	0	0,03	0,08	0,89	0	0,048
4	46173	0	0,04	0	0	0,96	0	0	0	0	0,96	0,04	0	0	0,022
4	25774	0	0,04	0	0	0,94	0,02	0	0	0	0	0,04	0,13	0,83	0,018
4	26967	0,02	0,02	0,01	0,06	0,76	0,13	0	0	0	0,01	0,16	0,76	0,07	0,048
4	35734	0,05	0	0	0	0,92	0	0	0,02	0,01	0	0,06	0,92	0,02	0,046
4	59540	0,01	0	0,08	0	0,89	0,01	0	0	0,01	0,87	0	0,01	0,12	0,044
4	50547	0	0,04	0	0,07	0,88	0	0	0,01	0	0,1	0	0,89	0,01	0,048
4	55909	0,01	0,04	0	0,01	0,92	0,01	0,01	0	0	0,02	0,35	0,58	0,05	0,04
4	46667	0	0,04	0	0	0,96	0	0	0	0	0	0,04	0	0,96	0,012
4	22349	0	0,04	0	0	0,95	0,01	0	0	0	0	0,95	0,04	0,01	0,02
4	100000	0,06	0,94	0	0	0	0	0	0	0	0	0,96	0	0,04	0,85
4	100000	0,08	0,91	0	0	0	0	0	0	0,01	0,01	0,75	0,15	0,09	0,602
4	17442	0	0	0	0,26	0	0	0	0	0,74	0,69	0,31	0	0	0,042
4	61736	0	0,04	0	0,04	0,92	0	0	0	0	0	0,91	0,09	0	0,046
4	52227	0,05	0	0	0	0	0,02	0	0,93	0	0	0,98	0	0,02	0,044
4	23022	0	0,04	0	0	0,95	0,01	0	0	0	0,01	0,47	0,49	0,03	0,016
4	20651	0	0,04	0	0	0,95	0	0	0	0,01	0,02	0,02	0,01	0,95	0,012
4	13733	0	0,04	0	0	0,96	0	0	0	0	0	0,96	0,01	0,03	0,026
4	36886	0	0,04	0,01	0	0,01	0	0	0,91	0,03	0,02	0,91	0,03	0,04	0,026
4	13555	0	0	0	0	0,78	0	0,12	0	0,1	0,1	0	0,88	0,02	0,048
4	63518	0	0	0,02	0,12	0,83	0	0	0	0,03	0	0,84	0,13	0,03	0,046
4	17277	0	0,04	0,03	0	0,93	0	0	0	0	0,82	0,04	0,14	0	0,046

4	73313	0	0,04	0	0	0,95	0	0	0,01	0	0	0,04	0	0,96	0,018
4	100000	0	0,97	0,02	0,01	0	0	0	0	0	0,02	0,97	0,01	0	0,916
4	100000	0,26	0,74	0	0	0	0	0	0	0	0	1	0	0	0,962
4	92168	0	0	0	0	0,83	0,09	0,07	0,01	0	0	0,16	0,01	0,83	0,048
4	31652	0	0,02	0	0,08	0,9	0	0	0	0	0	0,92	0,08	0	0,042
4	23585	0,02	0,04	0	0	0,94	0	0	0	0	0,94	0,04	0,02	0	0,044
4	6144	0,03	0,04	0	0,03	0,9	0	0	0	0	0,9	0,07	0	0,03	0,048
4	15713	0,02	0,04	0	0	0,93	0,01	0	0	0	0	0	0,97	0,03	0,036
4	18518	0	0	0	0,26	0,59	0,02	0	0,13	0	0,72	0,26	0	0,02	0,048
4	42023	0	0,04	0	0	0,93	0	0	0,03	0	0	0,04	0,95	0,01	0,016
4	24179	0	0,04	0	0	0,95	0	0	0	0,01	0	0,02	0,95	0,03	0,024
4	22227	0,01	0	0	0,25	0	0,74	0	0	0	0,98	0,02	0	0	0,048
4	31234	0	0,04	0	0	0,96	0	0	0	0	0	0,96	0,04	0	0,022
4	40160	0	0	0,01	0,07	0	0	0	0	0,92	0,05	0,94	0,01	0	0,048
4	19398	0	0,04	0	0	0,92	0	0	0,04	0	0	0	0,08	0,92	0,016
4	54553	0	0,04	0	0	0,96	0	0	0	0	0	0,96	0	0,04	0,028
4	31305	0	0,03	0	0,06	0	0,35	0	0,56	0	0	0,21	0,65	0,14	0,048
4	23369	0	0,04	0	0	0,96	0	0	0	0	0	0,03	0,96	0,01	0,024
4	22825	0	0	0,01	0,2	0,79	0	0	0	0	0,69	0	0,31	0	0,046
4	21575	0	0,04	0	0	0,96	0	0	0	0	0,96	0	0	0,04	0,046
4	33017	0	0,04	0	0	0,96	0	0	0	0	0	0	0	1	0,01
4	8425	0	0,01	0	0,4	0,52	0,07	0	0	0	0,99	0,01	0	0	0,048
4	45444	0,02	0,04	0	0	0,93	0	0	0,01	0	0,95	0,04	0	0,01	0,046
4	21743	0,03	0	0	0,07	0,82	0,03	0	0	0,05	0,07	0,88	0,05	0	0,048
4	70412	0	0,04	0	0,01	0,95	0	0	0	0	0	0	0,96	0,04	0,03
4	72917	0	0	0	0,13	0,59	0,27	0	0,01	0	0,12	0,85	0,02	0,01	0,044
4	5996	0	0,04	0,01	0	0,95	0	0	0	0	0,01	0,04	0,95	0	0,034
4	49015	0,01	0	0	0,11	0	0,05	0	0,83	0	0,07	0,88	0	0,05	0,048
4	18156	0	0,04	0	0	0,96	0	0	0	0	0,04	0	0,95	0,01	0,018
4	18740	0	0	0,04	0,09	0	0	0	0,54	0,33	0,01	0,37	0,58	0,04	0,04
4	14713	0,02	0	0,04	0	0	0,03	0,04	0,85	0,02	0,04	0,56	0,06	0,34	0,048
4	100000	0	0,98	0	0,01	0	0,01	0	0	0	0,01	0,99	0	0	0,908
4	30612	0	0	0	0,16	0,82	0	0,01	0,01	0	0,64	0,05	0	0,31	0,046

4	48349	0	0,04	0	0	0,95	0	0,01	0	0	0	0,94	0,01	0,05	0,02
4	56661	0	0,04	0	0,02	0	0	0	0,94	0	0	0,04	0,03	0,93	0,03
4	13671	0	0,04	0,01	0	0,95	0	0	0	0	0	0,99	0	0,01	0,018
4	51800	0	0,04	0	0	0,96	0	0	0	0	0	0,96	0	0,04	0,022
4	10756	0	0,04	0	0	0,95	0	0,01	0	0	0	0,04	0,95	0,01	0,028
4	28809	0	0	0	0,2	0,8	0	0	0	0	0,71	0	0,2	0,09	0,046
4	20690	0	0	0	0,12	0,87	0	0,01	0	0	0	0,13	0,87	0	0,048
4	14676	0	0,04	0	0	0,96	0	0	0	0	0,03	0,04	0	0,93	0,01
4	40659	0,06	0	0,01	0	0	0,63	0	0,3	0	0,54	0	0	0,46	0,042
4	68211	0	0,04	0	0	0,96	0	0	0	0	0	0,04	0	0,96	0,012
4,25	64972	0	0	0	0,08	0,01	0	0	0,91	0	0,01	0,91	0,08	0	0,042
4,25	47574	0	0,04	0	0	0	0	0	0,96	0	0	0,8	0,2	0	0,028
4,25	7535	0	0,04	0	0	0,93	0	0	0,03	0	0,03	0,04	0	0,93	0,01
4,25	55996	0	0,03	0	0,09	0,74	0,13	0	0,01	0	0,03	0,22	0,75	0	0,044
4,25	87452	0	0	0	0,14	0,86	0	0	0	0	0	0,86	0,14	0	0,046
4,25	18682	0,01	0,04	0	0	0,94	0,01	0	0	0	0,42	0	0,05	0,53	0,03
4,25	26527	0	0	0	0,07	0,93	0	0	0	0	0,07	0	0	0,93	0,048
4,25	11848	0	0,04	0,01	0	0,95	0	0	0	0	0	0,04	0,94	0,02	0,022
4,25	65948	0	0,04	0,01	0,06	0,87	0	0,01	0,01	0	0,1	0,06	0,02	0,82	0,048
4,25	74692	0	0,04	0	0	0,95	0	0	0	0,01	0,03	0,01	0,95	0,01	0,026
4,25	19796	0	0,04	0,01	0	0	0	0,01	0,94	0	0	0,36	0,05	0,59	0,016
4,25	33994	0,05	0,02	0	0	0,91	0,02	0	0	0	0,02	0,05	0,89	0,04	0,048
4,25	5350	0	0,04	0	0	0,95	0	0	0	0,01	0	0,05	0	0,95	0,01
4,25	55743	0	0,04	0,01	0	0,94	0,01	0	0	0	0	0,05	0	0,95	0,01
4,25	36198	0	0,04	0	0	0,96	0	0	0	0	0	0,01	0,05	0,94	0,01
4,25	56876	0	0	0	0,21	0,78	0	0,01	0	0	0,81	0	0,18	0,01	0,048
4,25	54039	0	0,04	0	0,01	0,94	0	0,01	0	0	0,04	0,01	0,94	0,01	0,03
4,25	18325	0	0,04	0	0	0,93	0	0	0,03	0	0,93	0	0,07	0	0,032
4,25	100000	0,17	0,79	0	0	0,01	0,01	0,02	0	0	0,05	0,75	0,05	0,15	0,66
4,25	53498	0	0,01	0	0,14	0,84	0,01	0	0	0	0,15	0,85	0	0	0,042
4,25	100000	0	0,88	0,01	0,1	0,01	0	0	0	0	0,01	0,87	0,12	0	0,7
4,25	41350	0	0,04	0	0,02	0,91	0	0	0	0,03	0,01	0,07	0,02	0,9	0,022
4,25	2626	0	0,04	0	0	0,95	0	0	0,01	0	0	0	0,05	0,95	0,008

4,25	45245	0	0,04	0	0	0,96	0	0	0	0	0,96	0,01	0,03	0	0,034
4,25	21811	0	0,04	0	0,01	0,95	0	0	0	0	0	0,04	0,95	0,01	0,04
4,25	53092	0	0,04	0	0	0,91	0,03	0	0,02	0	0,91	0,03	0,03	0,03	0,038
4,25	37402	0	0	0	0,16	0,81	0	0	0,03	0	0,2	0,04	0,76	0	0,042
4,25	13059	0,02	0,03	0	0	0,69	0,21	0,04	0	0,01	0,76	0	0,21	0,03	0,042
4,25	72370	0	0,04	0	0,01	0,95	0	0	0	0	0	0	0,96	0,04	0,032
4,25	31619	0	0,04	0	0	0,95	0	0	0	0,01	0	0,04	0,01	0,95	0,016
4,25	100000	0	1	0	0	0	0	0	0	0	0	0,23	0	0,77	0,27
4,25	76159	0,01	0,04	0	0	0,93	0,01	0	0,01	0	0,01	0	0,05	0,94	0,018
4,25	70605	0	0,04	0	0	0,96	0	0	0	0	0,04	0	0,96	0	0,03
4,25	52433	0	0,04	0	0	0,95	0,01	0	0	0	0	0,02	0,98	0	0,016
4,25	29108	0	0,04	0	0	0,94	0	0	0,02	0	0,04	0	0,96	0	0,028
4,25	26766	0	0,04	0	0	0,89	0,04	0	0	0,03	0,15	0,04	0,08	0,73	0,024
4,25	31802	0	0	0	0,07	0,7	0,16	0	0,01	0,06	0	0,22	0,01	0,77	0,042
4,25	100000	0,01	0,93	0,03	0,01	0	0	0,02	0	0	0	0,93	0,01	0,06	0,74
4,25	58164	0,01	0	0	0,14	0,06	0,45	0	0,34	0	0,34	0,65	0,01	0	0,048
4,25	33233	0	0,04	0	0	0,96	0	0	0	0	0	0,04	0	0,96	0,008
4,25	68752	0	0,04	0	0	0,94	0	0	0,02	0	0	0,94	0	0,06	0,02
4,25	81390	0,01	0,04	0	0	0,95	0	0	0	0	0,95	0,04	0	0,01	0,038
4,25	10642	0,05	0,01	0	0	0,94	0	0	0	0	0,14	0,86	0	0	0,036
4,25	18335	0	0,04	0	0,02	0,93	0	0	0,01	0	0	0,02	0,98	0	0,026
4,25	100000	0	0,98	0,01	0	0	0,01	0	0	0	0	0,98	0,02	0	0,892
4,25	80213	0	0	0	0,27	0,71	0	0	0,02	0	0,87	0,01	0,12	0	0,04
4,25	63952	0	0,04	0	0	0,94	0,01	0	0	0,01	0,01	0,01	0,94	0,04	0,022
4,25	2183	0	0,04	0	0	0,96	0	0	0	0	0,96	0,04	0	0	0,038
4,25	35400	0	0,04	0	0	0,96	0	0	0	0	0	0,04	0,08	0,88	0,012
4,25	41883	0	0	0,02	0,16	0,72	0,1	0	0	0	0,18	0,07	0,75	0	0,042
4,25	18145	0,02	0,04	0	0	0,93	0,01	0	0	0	0,01	0,04	0	0,95	0,034
4,25	63330	0,02	0	0	0,06	0	0,33	0	0,58	0,01	0,01	0,06	0,33	0,6	0,038
4,25	38113	0,05	0	0	0	0,71	0,23	0,01	0	0	0,96	0,02	0	0,02	0,048
4,25	53059	0	0,04	0	0	0,93	0,03	0	0	0	0,96	0,04	0	0	0,036
4,25	51819	0	0,03	0	0,04	0,91	0	0,02	0	0	0,93	0,04	0	0,03	0,044
4,25	12304	0	0,04	0,01	0,08	0,87	0	0	0	0	0,08	0,87	0,02	0,03	0,044

4,25	21604	0	0,02	0	0,1	0,84	0,04	0	0	0	0,02	0,04	0,94	0	0,046
4,25	81632	0	0,04	0	0	0,95	0	0	0,01	0	0,36	0,01	0,04	0,59	0,014
4,25	9732	0	0,04	0	0	0,94	0,02	0	0	0	0,69	0,05	0,25	0,01	0,04
4,25	65214	0	0,04	0	0	0,95	0	0	0	0,01	0,01	0	0	0,99	0,006
4,25	51364	0	0,04	0	0	0,92	0	0	0,04	0	0	0,04	0,92	0,04	0,018
4,25	41430	0	0,03	0	0,05	0,92	0	0	0	0	0,01	0,07	0	0,92	0,038
4,25	15465	0	0,01	0	0,37	0,62	0	0	0	0	0,99	0,01	0	0	0,046
4,25	14427	0,01	0,04	0	0	0,95	0	0	0	0	0	0,97	0,01	0,02	0,044
4,25	54126	0	0,04	0	0	0,9	0	0,01	0,04	0,01	0,28	0,01	0,02	0,69	0,03
4,25	32165	0	0	0	0,11	0	0,34	0	0,54	0,01	0,32	0,11	0	0,57	0,046
4,25	8930	0,02	0,04	0	0,01	0,92	0	0	0,01	0	0,04	0	0	0,96	0,022
4,25	44901	0	0,04	0,01	0	0,84	0	0,01	0,1	0	0,6	0,01	0,05	0,34	0,038
4,25	53961	0	0,04	0	0	0,85	0	0	0,1	0,01	0,09	0,89	0	0,02	0,036
4,25	20291	0	0	0	0,11	0,7	0,18	0	0	0,01	0	0,86	0,02	0,12	0,044
4,25	56285	0	0,02	0	0,07	0,9	0	0	0,01	0	0,07	0,01	0,03	0,89	0,044
4,25	54894	0,03	0	0	0,09	0,88	0	0	0	0	0,91	0	0,09	0	0,038
4,25	100000	0	0,94	0,01	0	0,02	0,01	0	0	0,02	0,01	0,94	0,03	0,02	0,778
4,25	100000	0	1	0	0	0	0	0	0	0	0	0,99	0,01	0	0,938
4,25	15776	0	0,04	0	0,07	0,89	0	0	0	0	0	0,08	0,92	0	0,042
4,25	6316	0,01	0,04	0	0	0,08	0	0	0,82	0,05	0,82	0,14	0,04	0	0,04
4,25	100000	0,53	0,38	0,09	0	0	0	0	0	0	0	0,77	0,21	0,02	0,822
4,25	46808	0	0,04	0	0	0,95	0,01	0	0	0	0	0,04	0,59	0,37	0,032
4,25	18989	0	0,04	0	0	0,96	0	0	0	0	0,01	0	0,03	0,96	0,014
4,25	17594	0	0,04	0	0	0,96	0	0	0	0	0	0,04	0,96	0	0,024
4,25	20680	0,01	0,04	0,01	0,02	0,82	0,1	0	0	0	0,27	0,06	0,65	0,02	0,036
4,25	11059	0	0,01	0	0,15	0,83	0,01	0	0	0	0,13	0,26	0,61	0	0,048
4,25	47034	0	0,04	0	0	0,96	0	0	0	0	0,96	0	0	0,04	0,042
4,25	100000	0,04	0,95	0	0,01	0	0	0	0	0	0	0,96	0	0,04	0,81
4,25	27931	0,01	0,04	0	0	0,94	0	0	0	0,01	0,01	0,74	0,01	0,24	0,04
4,25	75623	0	0,04	0	0,01	0,95	0	0	0	0	0	0,62	0,34	0,04	0,034
4,25	100000	0,07	0,88	0,03	0	0	0,02	0	0	0	0,02	0,91	0	0,07	0,732
4,25	32665	0	0,04	0	0	0,96	0	0	0	0	0,03	0,22	0	0,75	0,01
4,25	28724	0	0,04	0	0	0,96	0	0	0	0	0	0,96	0,04	0	0,018

4,25	83408	0	0	0	0,02	0,48	0,42	0,08	0	0	0	0,92	0	0,08	0,046
4,25	4156	0	0,04	0	0	0,95	0,01	0	0	0	0	0,04	0	0,96	0,004
4,25	16017	0	0	0,03	0,07	0,66	0,13	0	0,09	0,02	0	0,78	0,09	0,13	0,044
4,25	8593	0,06	0,01	0	0	0,91	0	0	0	0,02	0	0,08	0,92	0	0,046
4,25	15260	0	0,04	0	0	0,93	0,02	0,01	0	0	0,05	0,02	0,93	0	0,03
4,25	100000	0,05	0,62	0	0,27	0	0,06	0	0	0	0,09	0,62	0,27	0,02	0,542
4,25	25685	0	0	0	0,38	0,61	0	0	0,01	0	0,77	0,21	0,01	0,01	0,048
4,25	37191	0	0,04	0	0	0,95	0	0	0,01	0	0	0,04	0,01	0,95	0,006
4,25	16981	0	0,04	0	0	0,05	0	0	0,91	0	0,05	0,04	0	0,91	0,006
4,25	32259	0	0	0,01	0,08	0,71	0	0	0	0,2	0	0	0	1	0,048
4,25	9876	0	0,03	0	0,01	0	0,21	0,07	0,68	0	0	0,3	0,48	0,22	0,042
4,5	100000	0	0,98	0	0,01	0,01	0	0	0	0	0	0,64	0	0,36	0,394
4,5	59381	0	0	0	0,07	0,93	0	0	0	0	0,07	0	0	0,93	0,044
4,5	31344	0	0,03	0,01	0,1	0,86	0	0	0	0	0,99	0,01	0	0	0,046
4,5	22517	0	0,04	0	0	0,96	0	0	0	0	0,01	0,95	0,04	0	0,038
4,5	49080	0	0,04	0	0	0,96	0	0	0	0	0,96	0,03	0,01	0	0,034
4,5	40577	0,02	0	0,08	0	0,19	0,41	0	0,3	0	0,38	0,19	0,02	0,41	0,048
4,5	31936	0	0,04	0,01	0	0,29	0	0	0,66	0	0,1	0,42	0,04	0,44	0,018
4,5	27918	0	0,04	0	0	0,96	0	0	0	0	0,89	0,04	0	0,07	0,046
4,5	72518	0	0,04	0	0	0,96	0	0	0	0	0	0,04	0	0,96	0,006
4,5	75232	0	0	0,05	0	0	0,89	0,05	0	0,01	0,05	0	0,05	0,9	0,042
4,5	6781	0	0,04	0,01	0	0,92	0	0,03	0	0	0,01	0,02	0,92	0,05	0,028
4,5	100000	0,25	0,73	0,01	0	0	0,01	0	0	0	0	0,74	0	0,26	0,686
4,5	29419	0	0,04	0	0,07	0	0,04	0	0,84	0,01	0	0	0,96	0,04	0,046
4,5	19428	0	0	0	0,28	0,71	0	0,01	0	0	0,69	0,28	0,01	0,02	0,048
4,5	28843	0	0,04	0	0,02	0,93	0	0	0	0,01	0	0,04	0,93	0,03	0,036
4,5	18391	0,01	0,04	0	0	0,95	0	0	0	0	0	0,99	0	0,01	0,034
4,5	74046	0	0,04	0	0	0,92	0,03	0,01	0	0	0	0,11	0,87	0,02	0,032
4,5	95534	0,06	0	0	0	0,81	0,12	0	0	0,01	0,12	0,07	0	0,81	0,048
4,5	34592	0	0,04	0	0	0,96	0	0	0	0	0,96	0,01	0,03	0	0,024
4,5	58826	0	0,04	0	0	0,96	0	0	0	0	0,03	0,01	0	0,96	0,01
4,5	63768	0,06	0,01	0	0	0,93	0	0	0	0	0	0,6	0,4	0	0,048
4,5	93819	0	0,04	0	0	0,95	0	0	0	0,01	0,01	0	0,95	0,04	0,032

4,5	62856	0	0,04	0	0	0,96	0	0	0	0	0,04	0,96	0	0	0,03
4,5	16344	0	0,03	0	0,08	0,85	0	0	0,04	0	0	0,03	0,77	0,2	0,044
4,5	30259	0	0,04	0	0	0,7	0,26	0	0	0	0,23	0,01	0,06	0,7	0,014
4,5	40738	0	0,04	0	0,01	0,92	0	0	0,03	0	0,16	0,04	0,08	0,72	0,016
4,5	33242	0,04	0,04	0	0	0,92	0	0	0	0	0,03	0,04	0,01	0,92	0,048
4,5	14674	0	0	0	0,13	0	0	0	0,63	0,24	0	0,76	0,24	0	0,044
4,5	65248	0	0,04	0	0	0,96	0	0	0	0	1	0	0	0	0,042
4,5	86075	0,03	0	0	0	0	0,5	0,04	0,29	0,14	0,11	0,32	0	0,57	0,042
4,5	9985	0	0,04	0	0,03	0,92	0	0	0,01	0	0,07	0	0,9	0,03	0,034
4,5	22103	0	0,04	0	0	0,96	0	0	0	0	0	0,01	0,04	0,95	0,006
4,5	71758	0,01	0,03	0,01	0,01	0,92	0	0,02	0	0	0	0,03	0,93	0,04	0,04
4,5	100000	0	0,99	0	0	0	0,01	0	0	0	0	0	0,01	0,99	0,198
4,5	55119	0,04	0,03	0	0	0,92	0	0,01	0	0	0	0,08	0,92	0	0,048
4,5	100000	0	0,98	0	0	0	0	0	0,02	0	0,04	0,96	0	0	0,936
4,5	27763	0	0,04	0	0	0,96	0	0	0	0	0	1	0	0	0,022
4,5	100000	0	0,97	0,03	0	0	0	0	0	0	0	1	0	0	0,978
4,5	100000	0,11	0,87	0	0	0	0	0,02	0	0	0	0,87	0,02	0,11	0,662
4,5	65603	0,02	0,04	0	0	0,87	0	0	0,07	0	0,86	0,09	0,01	0,04	0,044
4,5	7522	0	0,04	0	0	0,95	0	0,01	0	0	0	0,04	0,96	0	0,032
4,5	52893	0	0,04	0	0	0,94	0	0,02	0	0	0,94	0,02	0,04	0	0,036
4,5	100000	0	1	0	0	0	0	0	0	0	0	1	0	0	0,982
4,5	48737	0,01	0,04	0	0	0,94	0,01	0	0	0	0	0,06	0,94	0	0,036
4,5	23776	0	0,04	0	0	0,96	0	0	0	0	0,09	0	0	0,91	0,008
4,5	45686	0	0,04	0	0	0,86	0,1	0	0	0	0	0,9	0,1	0	0,014
4,5	68405	0	0,04	0	0	0,96	0	0	0	0	0,94	0	0,04	0,02	0,036
4,5	100000	0	1	0	0	0	0	0	0	0	0	1	0	0	0,974
4,5	100000	0,02	0,98	0	0	0	0	0	0	0	0	1	0	0	0,94
4,5	55978	0	0	0	0,36	0,64	0	0	0	0	0,92	0,08	0	0	0,042
4,5	15847	0	0,04	0,01	0,02	0,9	0,01	0	0,02	0	0,03	0,04	0,93	0	0,046
4,5	89636	0	0,04	0	0,06	0,9	0	0	0	0	0,81	0,13	0	0,06	0,046
4,5	27989	0,03	0	0,07	0	0	0	0	0,9	0	0	0,93	0	0,07	0,046
4,5	100000	0	1	0	0	0	0	0	0	0	0,14	0,86	0	0	0,976
4,5	19311	0	0,04	0	0	0,94	0	0	0,02	0	0,01	0,04	0,44	0,51	0,014

4,5	99299	0,01	0,04	0	0	0,94	0	0	0,01	0	0	0,01	0,02	0,97	0,022
4,5	68435	0,06	0	0	0	0,61	0,31	0	0	0,02	0,66	0	0	0,34	0,048
4,5	14352	0	0,04	0	0,03	0,93	0	0	0	0	0	0,08	0,89	0,03	0,046
4,5	13003	0	0,04	0	0	0,96	0	0	0	0	0	0	0,95	0,05	0,028
4,5	58098	0	0	0	0,11	0,59	0,19	0	0	0,11	0	0,36	0,59	0,05	0,036
4,5	14919	0	0,04	0	0	0,96	0	0	0	0	0	0,02	0,96	0,02	0,026
4,5	100000	0,2	0,65	0	0,11	0,04	0	0	0	0	0	0,69	0,12	0,19	0,572
4,5	71022	0	0,04	0	0,01	0,95	0	0	0	0	0	0,01	0,95	0,04	0,032
4,5	38036	0	0,04	0	0	0,91	0	0,03	0,02	0	0	0,92	0,03	0,05	0,03
4,5	11096	0	0,04	0	0	0,96	0	0	0	0	0,61	0,04	0	0,35	0,024
4,5	11158	0	0,04	0,01	0,02	0,88	0	0,01	0	0,04	0	0,05	0,88	0,07	0,042
4,5	82742	0	0,04	0	0	0,96	0	0	0	0	0	0,92	0,06	0,02	0,024
4,5	35854	0	0,04	0	0	0,96	0	0	0	0	0,03	0,01	0,96	0	0,03
4,5	34151	0	0,04	0	0,01	0,95	0	0	0	0	0,02	0,07	0,13	0,78	0,02
4,5	6151	0	0	0	0,13	0,85	0,01	0,01	0	0	0	0,02	0,98	0	0,046
4,5	47390	0,01	0,04	0	0	0,94	0,01	0	0	0	0,05	0	0,01	0,94	0,026
4,5	35681	0	0,04	0	0	0,83	0	0	0,13	0	0	0,02	0	0,98	0,014
4,5	26172	0	0,04	0	0	0,96	0	0	0	0	0	0,11	0	0,89	0,014
4,5	26068	0	0,04	0	0,01	0,95	0	0	0	0	0,04	0,01	0	0,95	0,022
4,5	100000	0	0,99	0,01	0	0	0	0	0	0	0	0,99	0	0,01	0,936
4,5	76546	0	0,04	0	0	0,95	0	0	0	0,01	0	0,97	0,01	0,02	0,02
4,5	75555	0	0	0	0,42	0,58	0	0	0	0	0,99	0	0,01	0	0,044
4,5	24719	0,01	0,04	0	0,05	0,88	0	0	0	0,02	0,05	0,04	0,01	0,9	0,046
4,5	100000	0	0,99	0	0	0	0,01	0	0	0	0	0,99	0,01	0	0,908
4,5	68306	0	0	0,03	0,12	0,52	0,29	0	0	0,04	0,04	0,96	0	0	0,042
4,5	17001	0	0,04	0	0	0,96	0	0	0	0	0	0,98	0,02	0	0,032
4,5	100000	0	1	0	0	0	0	0	0	0	0	1	0	0	0,974
4,5	70339	0	0,04	0	0	0,86	0,1	0	0	0	0,81	0,09	0,1	0	0,032
4,5	100000	0,42	0,56	0	0	0	0,01	0	0	0,01	0	0,57	0,42	0,01	0,77
4,5	30458	0	0,04	0	0,02	0,93	0	0,01	0	0	0,02	0,05	0,93	0	0,038
4,5	10908	0	0	0	0,14	0,65	0	0,01	0,2	0	0,15	0,85	0	0	0,046
4,5	40414	0	0,04	0,01	0,03	0,81	0,01	0	0,1	0	0	0,01	0,08	0,91	0,042
4,5	8111	0,01	0,04	0	0	0,95	0	0	0	0	0	0,04	0	0,96	0,026

4,5	94677	0	0,04	0	0	0,89	0	0	0,07	0	0,07	0	0	0,93	0,014
4,5	21050	0,02	0,03	0	0	0,62	0,21	0,02	0,1	0	0,4	0,25	0,1	0,25	0,046
4,5	74456	0	0,04	0	0,02	0,94	0	0	0	0	0	0,06	0,94	0	0,032
4,5	24367	0	0,04	0	0,03	0,92	0	0,01	0	0	0,03	0,93	0,04	0	0,042
4,5	29444	0,02	0,04	0	0	0,93	0	0,01	0	0	0,92	0,03	0	0,05	0,04
4,5	41849	0	0,04	0	0	0,95	0	0	0,01	0	0	0,95	0,01	0,04	0,018
4,5	29257	0	0,04	0	0	0,95	0	0	0	0,01	0,01	0,03	0,95	0,01	0,034
4,5	100000	0	1	0	0	0	0	0	0	0	1	0	0	0	0,994
4,5	80694	0	0,04	0	0,02	0,94	0	0	0	0	0,02	0	0,04	0,94	0,022
4,5	71239	0	0,04	0	0	0,95	0	0,01	0	0	0,97	0	0,03	0	0,044
4,5	21995	0	0,04	0	0,01	0,95	0	0	0	0	0,01	0	0,96	0,03	0,028
4,5	13887	0	0,04	0,01	0,01	0,94	0	0	0	0	0,55	0	0,02	0,43	0,042
4,75	32334	0	0,01	0	0,1	0,83	0,02	0	0,04	0	0,09	0,84	0,02	0,05	0,048
4,75	37990	0	0,04	0	0	0,95	0	0	0	0,01	0,01	0,02	0,02	0,95	0,012
4,75	45712	0	0,04	0	0	0,96	0	0	0	0	0,06	0	0,9	0,04	0,026
4,75	80892	0	0,04	0	0,01	0,95	0	0	0	0	0,01	0,04	0	0,95	0,02
4,75	48870	0	0	0	0,08	0,84	0,08	0	0	0	0	0,08	0	0,92	0,048
4,75	100000	0	0,98	0	0	0	0	0	0,02	0	0,02	0,98	0	0	0,89
4,75	19004	0,01	0,04	0	0	0,95	0	0	0	0	0,34	0,05	0	0,61	0,034
4,75	63562	0	0,04	0	0	0,96	0	0	0	0	0	0,01	0	0,99	0,004
4,75	40551	0	0,04	0	0	0,96	0	0	0	0	0	0	1	0	0,022
4,75	50989	0	0,04	0,01	0	0,94	0	0	0,01	0	0,01	0,01	0	0,98	0,016
4,75	41989	0	0	0	0,37	0	0,61	0	0,01	0,01	0,99	0	0	0,01	0,046
4,75	60734	0	0,04	0	0	0,96	0	0	0	0	0,97	0,03	0	0	0,04
4,75	27185	0,01	0	0	0,11	0,82	0,06	0	0	0	0	0,82	0,12	0,06	0,046
4,75	100000	0	0,94	0,05	0	0	0	0	0,01	0	0	1	0	0	0,954
4,75	25412	0	0	0	0	0	0	0,08	0,03	0,89	0,08	0,92	0	0	0,046
4,75	37333	0	0,04	0	0	0,96	0	0	0	0	0	0,14	0	0,86	0,02
4,75	12493	0,04	0,02	0	0,01	0,91	0	0	0,01	0,01	0,77	0,08	0	0,15	0,04
4,75	77233	0	0	0	0,4	0,6	0	0	0	0	0,96	0,04	0	0	0,042
4,75	3299	0,02	0,04	0	0	0,94	0	0	0	0	0	0,04	0,94	0,02	0,04
4,75	11342	0	0,04	0	0	0,96	0	0	0	0	0	0	0	1	0,008
4,75	54546	0	0	0	0,12	0,87	0,01	0	0	0	0,01	0,95	0	0,04	0,04

4,75	4374	0	0,04	0	0	0,96	0	0	0	0	0	0	0,77	0,23	0,028
4,75	18684	0	0,04	0	0	0,89	0,05	0	0,02	0	0,94	0	0,04	0,02	0,048
4,75	53735	0,01	0,04	0	0	0,95	0	0	0	0	0	0,09	0,04	0,87	0,026
4,75	92992	0	0,04	0	0	0,96	0	0	0	0	0,98	0	0,02	0	0,036
4,75	16326	0	0,04	0	0	0,94	0	0	0,02	0	0,02	0	0,72	0,26	0,02
4,75	47144	0	0,04	0	0	0,9	0	0	0,06	0	0,13	0,08	0	0,79	0,02
4,75	60882	0	0	0	0,17	0,83	0	0	0	0	0,17	0,83	0	0	0,046
4,75	83435	0	0	0	0,09	0,77	0,1	0	0,04	0	0,06	0	0,13	0,81	0,046
4,75	57973	0	0,04	0	0,01	0,9	0	0	0,04	0,01	0,06	0	0,73	0,21	0,024
4,75	100000	0	1	0	0	0	0	0	0	0	0	1	0	0	0,976
4,75	79084	0	0,03	0	0,09	0,83	0	0	0,05	0	0,01	0,85	0,07	0,07	0,044
4,75	81228	0	0,04	0	0,06	0,85	0,05	0	0	0	0,85	0,01	0,09	0,05	0,044
4,75	30395	0,02	0,04	0	0	0	0,94	0	0	0	0,04	0	0,89	0,07	0,044
4,75	59271	0	0,04	0	0	0,94	0	0	0	0,02	0	0,3	0	0,7	0,01
4,75	26576	0	0,04	0	0	0,96	0	0	0	0	0	0,02	0,02	0,96	0,012
4,75	73456	0,05	0,02	0	0	0,91	0,02	0	0	0	0,02	0,02	0,96	0	0,048
4,75	35428	0	0,04	0,02	0	0,94	0	0	0	0	0	0,04	0,94	0,02	0,038
4,75	34758	0	0	0	0,07	0,62	0,25	0	0,01	0,05	0,02	0,36	0	0,62	0,042
4,75	55285	0	0,04	0	0,01	0,95	0	0	0	0	0	0,04	0,95	0,01	0,034
4,75	31975	0	0,04	0	0	0,95	0,01	0	0	0	0,01	0,02	0	0,97	0,02
4,75	76699	0	0,04	0	0	0,96	0	0	0	0	0,13	0	0,83	0,04	0,026
4,75	36737	0	0,04	0	0,01	0,95	0	0	0	0	0,01	0,96	0,03	0	0,036
4,75	37271	0,01	0,04	0	0,04	0,91	0	0	0	0	0,01	0,04	0,08	0,87	0,048
4,75	100000	0	0,98	0,02	0	0	0	0	0	0	0	0,98	0	0,02	0,898
4,75	18759	0,06	0,01	0	0	0,92	0	0	0	0,01	0	0,02	0,98	0	0,046
4,75	55644	0	0,04	0	0	0,94	0	0	0,02	0	0	0,03	0,37	0,6	0,016
4,75	18292	0	0,04	0	0,02	0,94	0	0	0	0	0	0,06	0,94	0	0,03
4,75	44553	0,01	0,04	0	0	0,95	0	0	0	0	0,95	0,05	0	0	0,04
4,75	10805	0,04	0,03	0	0	0,76	0,15	0	0,02	0	0,04	0,23	0,73	0	0,044
4,75	27215	0	0,04	0	0,01	0,94	0,01	0	0	0	0,07	0,02	0	0,91	0,026
4,75	12775	0	0,04	0	0	0,96	0	0	0	0	0	0,04	0	0,96	0,01
4,75	18234	0	0,04	0,01	0,02	0,93	0	0	0	0	0	0,05	0,94	0,01	0,038
4,75	80742	0	0,04	0	0	0,96	0	0	0	0	0	0,04	0,04	0,92	0,01

4,75	16916	0	0,04	0	0,01	0,95	0	0	0	0	0,01	0,98	0	0,01	0,038
4,75	25452	0	0,04	0	0	0,96	0	0	0	0	0,55	0	0,41	0,04	0,03
4,75	56127	0	0,04	0,01	0	0,94	0	0	0,01	0	0	0,01	0,01	0,98	0,006
4,75	100000	0	0,88	0	0	0,09	0,02	0	0,01	0	0	0,44	0,09	0,47	0,298
4,75	39197	0,01	0,01	0	0,24	0,72	0,01	0	0,01	0	0,95	0	0,01	0,04	0,032
4,75	13130	0	0,04	0	0	0,96	0	0	0	0	0	0,02	0,01	0,97	0,004
4,75	50627	0,04	0,01	0	0,03	0,92	0	0	0	0	0,04	0,04	0,92	0	0,044
4,75	71496	0,06	0,03	0	0	0,89	0,01	0	0	0,01	0,02	0,06	0,03	0,89	0,044
4,75	40162	0,02	0	0	0,06	0,91	0	0,01	0	0	0,03	0,94	0,03	0	0,046
4,75	12819	0	0,04	0	0	0,86	0	0	0,07	0,03	0,03	0	0,93	0,04	0,03
4,75	24687	0,06	0	0	0	0,54	0	0,01	0	0,39	0,4	0,54	0,06	0	0,048
4,75	50073	0	0	0,01	0,12	0,87	0	0	0	0	0,12	0,88	0	0	0,046
4,75	23714	0,05	0	0	0	0,71	0	0	0	0,24	0	0,15	0	0,85	0,046
4,75	26749	0,02	0,04	0	0	0,93	0	0	0	0,01	0,02	0	0,93	0,05	0,04
4,75	37084	0	0	0	0,09	0,86	0	0,04	0,01	0	0	0,04	0,96	0	0,048
4,75	22319	0	0,04	0	0,02	0,94	0	0	0	0	0	0,98	0,02	0	0,028
4,75	48168	0	0	0	0,12	0,74	0,14	0	0	0	0	0,41	0,59	0	0,048
4,75	10910	0	0	0	0,32	0,68	0	0	0	0	0,97	0	0,03	0	0,048
4,75	12917	0	0	0	0,15	0,85	0	0	0	0	0,04	0,84	0,11	0,01	0,046
4,75	61053	0,01	0,04	0	0	0,94	0,01	0	0	0	0,14	0,04	0,81	0,01	0,048
4,75	39883	0	0,04	0	0	0,96	0	0	0	0	0,03	0,01	0	0,96	0,01
4,75	22909	0,01	0,04	0	0	0,95	0	0	0	0	0	0,95	0,04	0,01	0,04
4,75	100000	0	0,99	0	0	0	0	0,01	0	0	0	1	0	0	0,932
4,75	34237	0	0,04	0	0	0,91	0	0	0,05	0	0,1	0,04	0,81	0,05	0,034
4,75	25992	0	0,04	0	0,05	0,91	0	0	0	0	0,02	0,91	0	0,07	0,048
4,75	26127	0	0,04	0	0	0,96	0	0	0	0	0	0,36	0	0,64	0,02
4,75	64662	0	0,01	0	0,09	0,55	0,01	0	0,33	0,01	0,01	0,9	0,09	0	0,044
4,75	12167	0	0,04	0	0	0,96	0	0	0	0	0	0,04	0,96	0	0,024
4,75	12475	0,01	0,04	0	0	0,91	0	0	0,03	0,01	0,05	0,03	0,92	0	0,03
4,75	12577	0	0,04	0	0	0,95	0	0	0,01	0	0	0,04	0	0,96	0,01
4,75	77208	0,06	0	0	0	0,94	0	0	0	0	0,98	0	0	0,02	0,046
4,75	78675	0	0,04	0	0	0,96	0	0	0	0	0	0,04	0	0,96	0,01
4,75	19716	0	0,04	0	0,01	0,92	0	0,03	0	0	0,74	0,01	0,07	0,18	0,04

4,75	18963	0	0,01	0	0,27	0,72	0	0	0	0	0,99	0	0,01	0	0,048
4,75	46185	0	0,04	0	0	0,95	0	0	0	0,01	0,04	0	0,96	0	0,026
4,75	57357	0,01	0,04	0	0,01	0,93	0	0,01	0	0	0,01	0,01	0,98	0	0,034
4,75	100000	0,02	0,84	0	0	0,09	0,04	0	0	0,01	0,01	0,85	0,03	0,11	0,484
4,75	27632	0	0,04	0	0,05	0,91	0	0	0	0	0,44	0,05	0	0,51	0,034
4,75	100000	0	0,99	0	0	0	0	0	0,01	0	0	0,99	0,01	0	0,872
4,75	9786	0,01	0,04	0	0	0,92	0	0,03	0	0	0,02	0,04	0,92	0,02	0,046
4,75	46549	0	0,04	0	0,12	0,84	0	0	0	0	0,84	0,03	0	0,13	0,042
4,75	13497	0	0	0,02	0,1	0	0,01	0	0,87	0	0	0,97	0,03	0	0,048
4,75	45082	0,06	0	0,01	0	0,83	0,06	0	0,04	0	0,04	0,12	0,84	0	0,048
4,75	11454	0,05	0,01	0	0	0,85	0,09	0	0	0	0,05	0,1	0	0,85	0,042
4,75	100000	0	0,42	0	0	0,53	0	0	0,05	0	0	0,95	0	0,05	0,206
4,75	71699	0	0,04	0	0	0,96	0	0	0	0	0	0	0	1	0,016
5	22766	0,05	0	0	0	0,95	0	0	0	0	0,05	0,95	0	0	0,046
5	7453	0,05	0	0	0	0,64	0,3	0	0	0,01	0,01	0,05	0,94	0	0,036
5	34644	0,05	0	0	0	0,95	0	0	0	0	0	1	0	0	0,048
5	40199	0	0,04	0	0	0,95	0	0	0	0,01	0,01	0,04	0	0,95	0,014
5	30781	0,02	0	0,04	0	0,74	0	0,01	0,19	0	0,19	0,07	0,74	0	0,042
5	76704	0	0,04	0	0	0,94	0	0	0,02	0	0	0,03	0,97	0	0,032
5	14591	0	0	0,02	0,14	0,76	0	0,02	0,04	0,02	0,2	0,56	0,21	0,03	0,042
5	20731	0	0,04	0	0	0,96	0	0	0	0	0	0,96	0	0,04	0,016
5	100000	0	0,96	0	0,03	0	0	0,01	0	0	0,04	0,96	0	0	0,9
5	52716	0	0,04	0	0	0,96	0	0	0	0	0	0,96	0	0,04	0,034
5	50742	0	0,01	0	0,11	0,85	0,03	0	0	0	0	0,89	0,11	0	0,048
5	100000	0	1	0	0	0	0	0	0	0	0	0,97	0	0,03	0,868
5	4987	0	0,04	0	0	0,95	0	0	0,01	0	0	0,03	0,01	0,96	0,008
5	16689	0	0,04	0	0	0,96	0	0	0	0	0,28	0,68	0	0,04	0,016
5	82191	0	0,04	0	0	0,96	0	0	0	0	0,04	0,95	0,01	0	0,026
5	100000	0	0,99	0	0	0	0	0,01	0	0	0,01	0,99	0	0	0,926
5	18870	0	0,04	0	0	0,95	0	0	0	0,01	0,16	0,03	0,81	0	0,022
5	71780	0	0,04	0,02	0	0,92	0,02	0	0	0	0,94	0,01	0,02	0,03	0,048
5	22132	0	0,04	0	0,04	0,03	0	0,07	0,82	0	0,27	0,58	0,11	0,04	0,048
5	100000	0,01	0,97	0	0	0	0	0	0,02	0	0	0,98	0	0,02	0,832

5	18910	0	0,01	0	0,11	0,67	0,21	0	0	0	0,14	0	0,79	0,07	0,046
5	24773	0	0	0	0,35	0,65	0	0	0	0	0,98	0	0	0,02	0,046
5	72982	0	0	0,07	0,03	0,88	0,01	0,01	0	0	0,05	0,81	0,05	0,09	0,04
5	100000	0	0,99	0	0	0	0,01	0	0	0	0	0,97	0,02	0,01	0,888
5	54104	0	0,04	0	0	0,83	0,13	0	0	0	0,87	0	0,13	0	0,04
5	25011	0	0,01	0	0,16	0,83	0	0	0	0	0,16	0	0,84	0	0,048
5	38667	0	0,04	0,03	0	0,87	0,06	0	0	0	0	0,03	0	0,97	0,022
5	100000	0	0,95	0,04	0	0	0	0	0,01	0	0,42	0,52	0,06	0	0,876
5	29610	0	0,04	0	0	0,96	0	0	0	0	0	0	1	0	0,024
5	55362	0	0,04	0	0	0,96	0	0	0	0	0	0,04	0,95	0,01	0,014
5	12072	0,01	0,03	0	0,06	0,89	0	0,01	0	0	0	0	0,99	0,01	0,046
5	54403	0	0	0	0,12	0,7	0,18	0	0	0	0,01	0,01	0,81	0,17	0,048
5	57094	0,01	0,04	0,02	0,01	0,92	0	0	0	0	0,02	0,8	0,04	0,14	0,04
5	39991	0	0,04	0	0	0,95	0	0	0,01	0	0,01	0,04	0,19	0,76	0,026
5	15063	0,05	0	0	0	0,18	0,62	0,02	0,12	0,01	0,2	0	0,24	0,56	0,044
5	27307	0	0,04	0	0	0,96	0	0	0	0	0	0	0,01	0,99	0,002
5	46630	0	0,04	0	0,06	0,9	0	0	0	0	0,04	0	0,01	0,95	0,048
5	38430	0	0,04	0	0	0,94	0	0	0,01	0,01	0,01	0,05	0	0,94	0,01
5	81257	0	0,04	0	0,01	0,88	0	0	0,07	0	0	0,04	0,89	0,07	0,032
5	61616	0	0	0,01	0,13	0,6	0,26	0	0	0	0,13	0,6	0,26	0,01	0,046
5	32614	0	0,04	0	0	0,9	0,04	0	0,02	0	0	0,04	0,9	0,06	0,038
5	11493	0,01	0,02	0,05	0	0,82	0,03	0,05	0,02	0	0,1	0,04	0,86	0	0,046
5	77044	0	0,04	0	0	0,94	0,01	0,01	0	0	0,02	0,03	0,95	0	0,03
5	37576	0,01	0	0	0,08	0,6	0	0	0,31	0	0	0,09	0,91	0	0,048
5	24273	0	0,04	0	0,06	0,89	0	0	0	0,01	0,92	0,05	0,03	0	0,044
5	24075	0	0,04	0	0	0,86	0	0	0,08	0,02	0,06	0	0	0,94	0,01
5	43093	0,01	0,04	0	0	0,63	0,06	0	0,26	0	0	0,04	0,01	0,95	0,022
5	45582	0	0,04	0	0	0,94	0,01	0	0,01	0	0	0,02	0	0,98	0,006
5	20922	0	0,04	0	0	0,92	0,01	0,03	0	0	0,93	0,04	0,03	0	0,048
5	9619	0,04	0,03	0	0	0,92	0	0	0,01	0	0,04	0	0,93	0,03	0,042
5	52487	0	0	0,01	0,11	0,77	0,09	0	0	0,02	0,17	0,54	0,23	0,06	0,046
5	43717	0	0,04	0	0	0,95	0	0,01	0	0	0	0	0,96	0,04	0,026
5	18977	0	0	0	0,12	0	0,86	0,02	0	0	0,12	0	0,88	0	0,046

5	22732	0	0,04	0	0,03	0,91	0	0,02	0	0	0,93	0	0,04	0,03	0,04
5	24486	0	0	0	0,08	0,81	0,11	0	0	0	0,09	0	0,1	0,81	0,04
5	17029	0	0	0	0,17	0,71	0,11	0	0	0,01	0,71	0,01	0,17	0,11	0,04
5	19835	0	0,04	0	0	0,96	0	0	0	0	0	0,04	0,01	0,95	0,014
5	27375	0	0,03	0,01	0,05	0,9	0,01	0	0	0	0,01	0,09	0	0,9	0,04
5	3631	0	0,04	0	0	0,96	0	0	0	0	0	0,04	0,96	0	0,028
5	14345	0	0,04	0	0,01	0,93	0	0,01	0,01	0	0,01	0,05	0	0,94	0,038
5	100000	0	0,94	0	0	0,02	0	0	0,02	0,02	0,22	0,77	0	0,01	0,852
5	14338	0,06	0	0	0	0,53	0	0	0	0,41	0	0,45	0,14	0,41	0,04
5	65560	0	0	0	0,2	0,8	0	0	0	0	0,78	0,03	0	0,19	0,044
5	32638	0,07	0	0	0	0,77	0	0	0,16	0	0,77	0,06	0,17	0	0,048
5	55689	0,06	0	0	0	0	0	0	0,94	0	0	1	0	0	0,038
5	100000	0	0,98	0	0	0	0,01	0	0,01	0	0,01	0,99	0	0	0,926
5	40083	0	0,04	0	0	0,96	0	0	0	0	0	0,65	0,35	0	0,024
5	80591	0,04	0,01	0,01	0	0,7	0,24	0	0	0	0,27	0,03	0,31	0,39	0,048
5	78443	0	0,04	0,01	0	0,95	0	0	0	0	0	0,01	0,99	0	0,02
5	62988	0	0	0	0,08	0	0,34	0	0,58	0	0,01	0,07	0,01	0,91	0,044
5	27848	0	0	0	0,12	0,88	0	0	0	0	0,12	0	0,88	0	0,048
5	9330	0	0,04	0	0	0,96	0	0	0	0	0	0,04	0	0,96	0,006
5	22086	0	0,04	0,01	0,07	0	0	0	0,88	0	0	0,79	0,12	0,09	0,044
5	9179	0	0,04	0	0	0,96	0	0	0	0	0	0,04	0	0,96	0,008
5	100000	0	0,98	0,02	0	0	0	0	0	0	0	1	0	0	0,952
5	30859	0	0,04	0	0	0,96	0	0	0	0	0	0,04	0	0,96	0,008
5	34699	0	0,04	0	0,04	0,92	0	0	0	0	0,04	0,02	0	0,94	0,044
5	100000	0	0,98	0	0	0,02	0	0	0	0	0	0,89	0,09	0,02	0,712
5	44191	0	0	0,09	0,03	0	0,28	0	0,6	0	0,17	0,52	0,09	0,22	0,044
5	68194	0	0,04	0,05	0	0,84	0	0	0,07	0	0,04	0,06	0,84	0,06	0,038
5	23009	0	0	0	0,12	0,88	0	0	0	0	0	0,12	0,88	0	0,038
5	56547	0	0,04	0,02	0	0,9	0	0	0,02	0,02	0,06	0,02	0,9	0,02	0,028
5	69678	0,04	0,02	0	0	0,9	0	0	0,04	0	0,89	0,07	0,01	0,03	0,044
5	98253	0,01	0	0,02	0,09	0,85	0	0,02	0	0,01	0,81	0,02	0,03	0,14	0,048
5	39712	0	0,04	0	0	0,96	0	0	0	0	0,04	0	0	0,96	0,008
5	100000	0	0,98	0	0	0	0	0	0,02	0	0	0,72	0,09	0,19	0,548

5	12583	0,02	0,04	0	0,02	0,9	0,02	0	0	0	0	0,01	0,96	0,03	0,044
5	63202	0	0,04	0	0	0,79	0,17	0	0	0	0,08	0,02	0,78	0,12	0,028
5	45612	0,02	0,04	0	0	0,93	0,01	0	0	0	0,99	0	0,01	0	0,048
5	57586	0	0,04	0	0	0,96	0	0	0	0	0	0,04	0,96	0	0,024
5	15170	0,07	0	0	0	0,93	0	0	0	0	0,12	0,88	0	0	0,048
5	31776	0,01	0,04	0	0	0,95	0	0	0	0	0,9	0,05	0,01	0,04	0,038
5	100000	0	1	0	0	0	0	0	0	0	0	1	0	0	0,948
5	81654	0	0	0	0,08	0	0,53	0	0,39	0	0,08	0	0,39	0,53	0,032
5	37778	0,04	0,03	0	0	0,93	0	0	0	0	0	0,03	0,9	0,07	0,04
5	55080	0,05	0,02	0	0	0,93	0	0	0	0	0,02	0	0,98	0	0,048
5	100000	0,1	0,87	0,02	0	0	0	0	0	0,01	0	0,87	0,1	0,03	0,716
5	16266	0	0,04	0	0	0,9	0,02	0	0,04	0	0,9	0,04	0,05	0,01	0,036
5	100000	0	1	0	0	0	0	0	0	0	0	1	0	0	0,962
5	4325	0	0,04	0	0	0,95	0,01	0	0	0	0	1	0	0	0,03
	32500,36	0,00713	0,08438	0,00228	0,0389	0,79416	0,02935	0,00246	0,03608	0,00525	0,20654	0,23841	0,29198	0,26307	0,080812
