# Supplementary material for: Deception Undermines the Stability of Cooperation in Games of Indirect Reciprocity
Source: PLoS One. 2016 Jan 29;11(1):e0147623. doi: 10.1371/journal.pone.0147623 (PMC4732813; doi:10.1371/journal.pone.0147623)
Supplement: S3 Table — (PDF) [file pone.0147623.s007.pdf]

S3 Table. Results of the Beta=6 scenario.

shunning

b	time_to_c	(C,C)	(C,D)	(C,P)	(D,C)	(D,D)	(D,P)	(P,C)	(P,D)	(P,P)	S(G,G)	S(G,B)	S(B,G)	S(B,B)	cooperatio
1	22473	0	0	0,04	0	0,95	0,01	0	0	0	0	0,04	0	0,96	0,002
1	6263	0	0	0,04	0,01	0,11	0,8	0	0,04	0	0,12	0	0,04	0,84	0,018
1	42903	0,03	0,02	0,04	0	0,89	0	0,02	0	0	0,05	0,04	0	0,91	0,046
1	42162	0	0,1	0	0	0,89	0	0,01	0	0	0,75	0,15	0,1	0	0,048
1	39187	0	0,08	0,01	0,07	0,01	0,83	0	0	0	0,01	0,07	0,08	0,84	0,048
1	17667	0,01	0,07	0	0	0,91	0	0,01	0	0	0,6	0,33	0,07	0	0,048
1	14155	0	0,1	0	0	0,15	0,66	0	0	0,09	0,75	0,01	0	0,24	0,048
1	47541	0	0,07	0	0,06	0,32	0	0	0,55	0	0,34	0,58	0,07	0,01	0,046
1	78187	0	0,09	0,02	0	0,87	0	0,01	0	0,01	0,02	0,02	0,87	0,09	0,046
1	42668	0,02	0	0	0,05	0,93	0	0	0	0	0,02	0,91	0,07	0	0,048
1	12881	0	0,56	0,03	0	0	0	0,03	0,28	0,1	0	0,16	0	0,84	0,04
1	68858	0	0,13	0,02	0	0,85	0	0	0	0	0	0	1	0	0,048
1	25617	0	0,44	0,04	0	0	0	0	0,04	0,48	0,02	0,06	0	0,92	0,046
1	44753	0,03	0,92	0,03	0	0	0	0	0	0,02	0	0,05	0,03	0,92	0,048
1	18512	0	0	0,04	0	0,47	0,42	0,03	0,04	0	0,46	0,07	0,47	0	0,034
1	11777	0,01	0,41	0,01	0	0	0,5	0	0,07	0	0,01	0,09	0,03	0,87	0,046
1	9868	0	0,16	0	0	0,21	0,62	0	0,01	0	0,16	0	0,63	0,21	0,046
1	41685	0,02	0,02	0,04	0	0,25	0,67	0	0	0	0	0,29	0,67	0,04	0,04
1	13339	0	0	0,04	0	0,77	0	0	0	0,19	0	0,04	0,96	0	0,022
1	14692	0	0	0	0,11	0,76	0	0	0,12	0,01	0,19	0,01	0,69	0,11	0,032
1	18092	0,02	0	0,04	0,02	0	0	0	0,9	0,02	0	0,06	0	0,94	0,046
1	61085	0	0	0,03	0	0,94	0	0,03	0	0	0,04	0,04	0,92	0	0,032
1	8980	0	0	0,04	0	0,01	0	0,06	0,88	0,01	0,07	0,88	0,05	0	0,044
1	15731	0,01	0,04	0,04	0,01	0	0,9	0	0	0	0,05	0	0,01	0,94	0,03
1	3272	0	0	0,04	0,02	0,94	0	0	0	0	0	0,06	0,94	0	0,038
1	10458	0	0,13	0,04	0	0,79	0	0,03	0	0,01	0	0,86	0,14	0	0,044
1	100000	0	0	0,87	0	0	0	0,05	0,02	0,06	0	0,87	0,13	0	0,476
1	17416	0	0	0,04	0	0,64	0,01	0	0,31	0	0,65	0,04	0	0,31	0,03
1	37761	0	0	0,04	0	0,95	0	0	0	0,01	0,03	0,06	0,9	0,01	0,026
1	13582	0,06	0,01	0	0	0,92	0	0	0	0,01	0,07	0,01	0	0,92	0,048

1	12973	0,05	0	0,03	0	0,85	0	0,01	0,05	0,01	0,09	0,87	0,01	0,03	0,046
1	29770	0	0,07	0,04	0,01	0,88	0	0	0	0	0,12	0	0,88	0	0,044
1	43544	0,06	0	0	0	0,93	0	0	0	0,01	0,93	0	0,07	0	0,046
1	6941	0	0	0,04	0,01	0	0,93	0,02	0	0	0,01	0,06	0,02	0,91	0,024
1	65973	0,01	0,09	0,04	0	0,72	0	0	0	0,14	0	0,13	0,73	0,14	0,048
1	54385	0	0,51	0	0,03	0,3	0	0	0,13	0,03	0,03	0,33	0,01	0,63	0,036
1	30184	0	0	0,04	0	0	0	0	0,96	0	0	0,04	0,96	0	0,024
1	18242	0	0	0,04	0,02	0	0	0,01	0,93	0	0	0,04	0,93	0,03	0,034
1	23144	0	0,06	0	0	0,94	0	0	0	0	0,94	0	0,06	0	0,032
1	4966	0	0,1	0	0	0,51	0,39	0	0	0	0,61	0	0,39	0	0,048
1	3392	0,02	0	0,04	0,02	0,01	0,51	0	0,4	0	0,1	0,05	0,85	0	0,04
1	14128	0,03	0	0	0,05	0,92	0	0	0	0	0,02	0	0,92	0,06	0,048
1	6074	0	0	0	0,15	0,83	0,01	0	0	0,01	0,52	0	0,46	0,02	0,046
1	17792	0,05	0	0,01	0	0,94	0	0	0	0	0,07	0,93	0	0	0,048
1	27944	0,05	0	0,01	0	0,94	0	0	0	0	0,95	0,01	0,01	0,03	0,044
1	15613	0,02	0	0,04	0	0	0,83	0,03	0,03	0,05	0,72	0,02	0,21	0,05	0,044
1	20650	0	0	0,04	0	0,06	0,27	0	0,63	0	0,64	0,06	0,27	0,03	0,032
1	13254	0	0,03	0	0,1	0,2	0	0,01	0,66	0	0,21	0,13	0,66	0	0,042
1	4258	0	0	0	0,17	0,83	0	0	0	0	0,83	0	0,17	0	0,042
1	15412	0	0	0,04	0	0,01	0,03	0,03	0	0,89	0,04	0	0,03	0,93	0,03
1	11974	0	0	0,04	0	0,55	0	0,01	0,4	0	0	0,04	0,95	0,01	0,018
1	3508	0,05	0	0,02	0	0,93	0	0	0	0	0	0,07	0,93	0	0,046
1	23029	0,02	0,02	0,03	0	0,65	0	0,03	0,25	0	0,27	0,03	0,68	0,02	0,04
1	2411	0	0	0,04	0	0	0	0,08	0,84	0,04	0	0,12	0,88	0	0,03
1	4301	0	0,12	0	0	0,52	0	0	0,36	0	0	0,12	0,84	0,04	0,04
1	3377	0	0	0,04	0	0,95	0	0	0,01	0	0	0,04	0,01	0,95	0
1	30586	0	0,07	0,01	0,01	0,91	0	0	0	0	0,92	0,01	0,07	0	0,048
1	78370	0,01	0,03	0,04	0	0	0	0,03	0,89	0	0,01	0,08	0	0,91	0,038
1	43479	0,06	0	0	0	0	0	0	0,94	0	0	0,06	0	0,94	0,044
1	19380	0	0,51	0,04	0	0	0	0	0,45	0	0	0,99	0,01	0	0,046
1	54694	0	0	0,04	0	0,96	0	0	0	0	0,3	0,04	0,22	0,44	0,018
1	65365	0,03	0,95	0,02	0	0	0	0	0	0	0,03	0,02	0	0,95	0,046
1	3978	0	0	0,04	0,01	0,27	0	0	0	0,68	0,37	0,05	0	0,58	0,036

1	18573	0	0	0,02	0,05	0	0,23	0,05	0	0,65	0,02	0,69	0,29	0	0,044
1	50323	0	0,36	0	0,02	0,6	0,01	0	0,01	0	0,14	0,01	0	0,85	0,04
1	18085	0,06	0	0,02	0,01	0	0,04	0	0,87	0	0,01	0,74	0,21	0,04	0,048
1	28795	0,02	0	0,04	0	0	0	0	0,94	0	0,96	0	0,02	0,02	0,044
1	8733	0	0,14	0,01	0	0,85	0	0	0	0	0,1	0,01	0,85	0,04	0,046
1	52382	0,06	0	0	0	0,89	0,05	0	0	0	0,89	0,06	0	0,05	0,044
1	78930	0	0,04	0,04	0	0	0	0	0,92	0	0	0,04	0,92	0,04	0,034
1	100000	0,04	0	0,95	0	0,01	0	0	0	0	0,81	0,19	0	0	0,954
1	12262	0,03	0	0,03	0,05	0,83	0,04	0	0,02	0	0,02	0,03	0,95	0	0,044
1	34545	0	0,01	0,03	0,11	0,85	0	0	0	0	0,85	0,11	0,01	0,03	0,044
1	5586	0	0,12	0	0	0,66	0	0	0,21	0,01	0	0	0,88	0,12	0,042
1	12226	0	0	0	0,07	0,7	0	0	0,23	0	0,23	0	0	0,77	0,042
1	33698	0	0	0	0,37	0,63	0	0	0	0	1	0	0	0	0,048
1	74072	0,02	0,02	0	0,05	0,9	0	0	0	0,01	0,05	0,9	0,03	0,02	0,044
1	3313	0	0	0	0,15	0,81	0	0,02	0,02	0	0,81	0,01	0,02	0,16	0,034
1	15447	0,06	0	0	0	0,55	0	0	0,39	0	0,01	0,58	0,02	0,39	0,044
1	36897	0,05	0	0,04	0	0,05	0	0	0,86	0	0,01	0,04	0,9	0,05	0,048
1	17725	0	0	0,04	0	0,96	0	0	0	0	0,61	0,04	0,12	0,23	0,032
1	32735	0	0	0	0,28	0	0,69	0,02	0,01	0	0,95	0,05	0	0	0,044
1	19922	0	0,96	0	0	0	0	0	0	0,04	0,01	0,68	0	0,31	0,038
1	59898	0,06	0	0	0	0,07	0,87	0	0	0	0,93	0	0	0,07	0,042
1	5129	0,01	0,06	0	0	0,93	0	0	0	0	0,79	0	0,21	0	0,046
1	26577	0,01	0,19	0,04	0	0,1	0	0,01	0,65	0	0,04	0,3	0,04	0,62	0,036
1	55749	0	0	0	0,05	0	0,85	0,01	0	0,09	0,01	0,05	0,08	0,86	0,046
1	34779	0	0,1	0	0,02	0,79	0,09	0	0	0	0,48	0,33	0,1	0,09	0,044
1	60507	0	0,01	0,04	0	0	0,72	0,01	0	0,22	0,19	0,05	0,76	0	0,022
1	29367	0	0	0	0,12	0	0,57	0,05	0,01	0,25	0,57	0,26	0,12	0,05	0,042
1	78826	0,05	0	0	0	0	0,89	0	0	0,06	0,82	0,18	0	0	0,046
1	8663	0,03	0	0,03	0,07	0,82	0,01	0	0,01	0,03	0,1	0,01	0,82	0,07	0,048
1	18183	0	0,28	0	0	0	0	0	0,72	0	0,01	0	0,28	0,71	0,046
1	8889	0	0,08	0,03	0	0,84	0,04	0	0,01	0	0,07	0	0,92	0,01	0,042
1	47269	0	0	0	0,07	0,92	0	0	0	0,01	0	0,92	0,07	0,01	0,04
1	50935	0,06	0	0	0	0,94	0	0	0	0	0,06	0	0	0,94	0,048

1	48485	0,01	0	0	0,05	0	0	0	0,93	0,01	0,02	0,69	0,01	0,28	0,044
1	8920	0,05	0	0,01	0	0,93	0	0,01	0	0	0,05	0,01	0,93	0,01	0,046
1	100000	0	0	0,99	0,01	0	0	0	0	0	0,01	0,99	0	0	0,714
1	28079	0,03	0	0,01	0	0,82	0	0,1	0	0,04	0,79	0,05	0,06	0,1	0,048
1,25	26540	0	0	0	0,09	0	0,55	0	0	0,36	0,01	0,08	0,55	0,36	0,046
1,25	58388	0	0,05	0	0,06	0,88	0	0	0,01	0	0,05	0,07	0	0,88	0,048
1,25	3761	0,03	0	0	0	0,63	0	0,03	0,31	0	0,31	0,56	0,13	0	0,036
1,25	94971	0	0,05	0,04	0	0,76	0,01	0	0,01	0,13	0,02	0,04	0,02	0,92	0,01
1,25	21773	0,01	0	0,01	0,14	0,69	0,02	0	0,13	0	0,79	0,01	0,02	0,18	0,048
1,25	59164	0,01	0,19	0	0	0,8	0	0	0	0	0	0,8	0,2	0	0,042
1,25	19392	0,04	0	0,04	0	0,72	0,18	0	0,02	0	0,23	0,24	0,53	0	0,04
1,25	14098	0	0	0,04	0,05	0,15	0	0	0,64	0,12	0	0,03	0,94	0,03	0,046
1,25	36058	0	0	0,04	0	0,92	0,02	0,02	0	0	0,73	0,25	0,02	0	0,038
1,25	100000	0,01	0	0,99	0	0	0	0	0	0	0	1	0	0	0,648
1,25	59294	0,02	0	0,02	0,02	0,68	0	0,02	0	0,24	0,04	0,02	0,94	0	0,044
1,25	6925	0	0,01	0,04	0	0,82	0	0,02	0,09	0,02	0	0,11	0,82	0,07	0,042
1,25	18415	0,06	0	0	0	0	0	0	0,94	0	0,94	0	0	0,06	0,048
1,25	64626	0	0	0,04	0,06	0,71	0	0	0	0,19	0	0,7	0,11	0,19	0,044
1,25	89770	0,01	0,09	0	0	0,5	0,39	0,01	0	0	0,42	0,01	0,56	0,01	0,048
1,25	29977	0	0,17	0	0,02	0,81	0	0	0	0	0,02	0,81	0,17	0	0,042
1,25	6804	0	0	0,04	0	0,02	0,71	0	0,23	0	0,02	0,19	0,46	0,33	0,022
1,25	83249	0,02	0,98	0	0	0	0	0	0	0	0	1	0	0	0,048
1,25	15346	0	0,14	0,01	0,03	0,75	0	0	0,07	0	0,03	0,82	0,15	0	0,046
1,25	58105	0	0,01	0,04	0,07	0,25	0	0	0,63	0	0,67	0,01	0,25	0,07	0,03
1,25	57901	0	0,07	0	0,01	0,92	0	0	0	0	0,92	0	0,07	0,01	0,044
1,25	95686	0	0,03	0,04	0	0,02	0,69	0	0	0,22	0,69	0,14	0	0,17	0,046
1,25	5647	0	0	0,04	0	0	0	0	0,95	0,01	0	0,03	0,02	0,95	0,002
1,25	27897	0	0,03	0,03	0,06	0,72	0	0	0,15	0,01	0,06	0,18	0,03	0,73	0,046
1,25	5349	0,04	0	0,03	0	0	0	0,02	0,09	0,82	0,13	0,03	0,84	0	0,046
1,25	85644	0	0	0,04	0	0,89	0,03	0,03	0,01	0	0	0,92	0,04	0,04	0,028
1,25	29916	0	0	0,04	0,06	0	0	0	0,89	0,01	0	0,92	0	0,08	0,044
1,25	12088	0	0	0,04	0	0,01	0,07	0	0,88	0	0,01	0,12	0,87	0	0,018
1,25	16577	0	0	0	0,3	0,7	0	0	0	0	0,99	0	0,01	0	0,042

1,25	24199	0	0,01	0,02	0	0,4	0	0,09	0,48	0	0	0,5	0,5	0	0,046
1,25	21304	0	0,21	0	0	0,79	0	0	0	0	0	0,79	0,21	0	0,048
1,25	30797	0,01	0,86	0	0	0	0	0	0,13	0	0,01	0,99	0	0	0,046
1,25	38634	0	0,97	0,01	0,02	0	0	0	0	0	0,02	0,01	0,01	0,96	0,04
1,25	13814	0,05	0,01	0	0	0	0,8	0	0,09	0,05	0,85	0,06	0,04	0,05	0,048
1,25	3774	0,07	0	0	0	0,91	0	0	0,02	0	0,96	0,02	0,02	0	0,048
1,25	100000	0	0	0,96	0,03	0	0	0,01	0	0	0	0,96	0,03	0,01	0,556
1,25	21326	0	0	0	0,21	0,79	0	0	0	0	0,79	0,02	0	0,19	0,046
1,25	43058	0,05	0	0,03	0	0	0,02	0	0,39	0,51	0	0,1	0	0,9	0,046
1,25	71030	0	0,01	0	0,09	0,9	0	0	0	0	0,09	0	0,9	0,01	0,032
1,25	11774	0	0	0,03	0,03	0	0,8	0,13	0,01	0	0,81	0,06	0,13	0	0,038
1,25	45223	0,03	0	0,02	0	0	0	0,05	0,17	0,73	0,25	0,01	0,01	0,73	0,048
1,25	28881	0	0,09	0	0,07	0,83	0	0	0	0,01	0,09	0,9	0	0,01	0,048
1,25	4018	0	0	0,04	0	0	0,18	0	0,02	0,76	0,01	0,15	0,11	0,73	0,018
1,25	22904	0	0,12	0	0	0,02	0,62	0	0,06	0,18	0,02	0,1	0,8	0,08	0,042
1,25	29400	0	0,02	0,04	0,03	0,36	0	0	0,52	0,03	0,39	0,05	0	0,56	0,046
1,25	68868	0	0	0,03	0,09	0,02	0,16	0	0,7	0	0,47	0,19	0,25	0,09	0,04
1,25	65486	0	0,2	0,04	0	0	0	0	0,76	0	0,1	0,04	0,1	0,76	0,036
1,25	41179	0	0	0	0,08	0,61	0	0	0,31	0	0,08	0	0	0,92	0,048
1,25	7580	0	0,14	0	0,01	0,83	0	0	0,01	0,01	0,01	0,04	0,94	0,01	0,048
1,25	12929	0	0,27	0,04	0	0,11	0	0	0,58	0	0	0,04	0,27	0,69	0,03
1,25	10426	0	0	0,04	0	0,93	0	0,03	0	0	0	0,01	0,04	0,95	0,026
1,25	15062	0,01	0,22	0,03	0	0,55	0	0,03	0	0,16	0	0,06	0,17	0,77	0,044
1,25	19655	0	0	0,04	0	0	0	0	0	0,96	0,09	0,86	0,05	0	0,028
1,25	37930	0	0,02	0,03	0,08	0,47	0,24	0	0,08	0,08	0,32	0,5	0,1	0,08	0,046
1,25	28051	0	0,21	0	0	0	0,78	0,01	0	0	0	0,78	0,02	0,2	0,046
1,25	15366	0	0	0,04	0	0	0,01	0,01	0,94	0	0	0,44	0,01	0,55	0,008
1,25	9375	0	0,22	0	0	0,78	0	0	0	0	0	0,78	0,22	0	0,038
1,25	6352	0	0,04	0,04	0	0,01	0,9	0,01	0	0	0,04	0,31	0,64	0,01	0,046
1,25	31959	0	0,02	0	0	0	0,29	0,1	0,57	0,02	0,26	0	0,57	0,17	0,044
1,25	39416	0	0,01	0	0	0	0,87	0,12	0	0	0	0,87	0,13	0	0,046
1,25	100000	0	0	0,98	0	0	0	0	0	0,02	0	0,98	0,02	0	0,494
1,25	54704	0	0,19	0	0	0,81	0	0	0	0	0,03	0	0,74	0,23	0,042

1,25	62338	0,02	0	0,04	0	0,92	0	0,01	0,01	0	0,93	0,04	0,02	0,01	0,046
1,25	29715	0	0,01	0,04	0	0,94	0	0,01	0	0	0,1	0,27	0	0,63	0,024
1,25	17299	0	0	0	0	0,01	0	0,1	0,89	0	0	0	0,9	0,1	0,046
1,25	46715	0	0	0,04	0	0,74	0	0	0,16	0,06	0,05	0,04	0,01	0,9	0,006
1,25	16953	0,06	0	0	0	0	0,62	0	0,3	0,02	0	0,11	0,81	0,08	0,046
1,25	56721	0	0,02	0	0,08	0,83	0,07	0	0	0	0	0,09	0,91	0	0,048
1,25	3049	0	0	0,04	0	0	0,03	0,01	0,92	0	0,03	0,5	0,01	0,46	0,012
1,25	22871	0,03	0,04	0,01	0,03	0,89	0	0	0	0	0,04	0,89	0,03	0,04	0,048
1,25	49605	0	0	0,04	0	0,96	0	0	0	0	0	0,1	0,9	0	0,018
1,25	5514	0	0,12	0	0	0,86	0	0,02	0	0	0,02	0	0,86	0,12	0,048
1,25	22228	0	0	0,04	0	0	0	0,02	0,7	0,24	0,02	0,04	0	0,94	0,028
1,25	6875	0	0,05	0,04	0	0,79	0,09	0	0	0,03	0,17	0,04	0,79	0	0,03
1,25	18457	0,04	0	0,03	0,01	0	0,02	0	0,9	0	0,03	0,03	0,04	0,9	0,046
1,25	11278	0,02	0	0	0	0	0,16	0,06	0,66	0,1	0,14	0,06	0,17	0,63	0,046
1,25	8775	0	0	0,04	0	0,1	0	0	0,33	0,53	0,15	0,57	0,18	0,1	0,024
1,25	16499	0	0	0	0,07	0,93	0	0	0	0	0	0,88	0,12	0	0,046
1,25	6030	0	0	0	0	0,94	0	0,06	0	0	0	0,94	0,06	0	0,04
1,25	57722	0	0	0,04	0	0,01	0,07	0	0,08	0,8	0,01	0,19	0	0,8	0,002
1,25	11142	0	0	0,01	0,07	0,92	0	0	0	0	0	0,01	0,1	0,89	0,046
1,25	17263	0	0	0,04	0	0,96	0	0	0	0	0,96	0,04	0	0	0,036
1,25	3686	0	0,08	0,03	0,02	0,81	0,06	0	0	0	0,09	0,06	0,85	0	0,046
1,25	7632	0,02	0	0,01	0,06	0,02	0	0,01	0,88	0	0,07	0,88	0,05	0	0,048
1,25	24720	0	0,08	0	0,05	0,86	0,01	0	0	0	0,12	0,01	0,01	0,86	0,046
1,25	78489	0,01	0,01	0,04	0	0,65	0	0	0	0,29	0,1	0,06	0,19	0,65	0,04
1,25	8620	0	0	0	0,12	0	0	0	0,88	0	0	0	0,88	0,12	0,042
1,25	36221	0	0	0	0,06	0,49	0	0	0,45	0	0	0,54	0,03	0,43	0,048
1,25	26335	0	0	0,04	0	0	0,01	0	0	0,95	0,01	0,04	0	0,95	0,008
1,25	10608	0	0,18	0,01	0	0	0,24	0	0,57	0	0,07	0,78	0,15	0	0,048
1,25	100000	0	0	0,96	0	0	0	0	0	0,04	0,22	0,74	0,04	0	0,678
1,25	29824	0,02	0,93	0,04	0	0,01	0	0	0	0	0	0,36	0,02	0,62	0,04
1,25	23718	0,01	0	0,04	0	0,13	0,71	0	0,1	0,01	0,72	0,09	0,19	0	0,048
1,25	31102	0	0,24	0,04	0	0,72	0	0	0	0	0,23	0,02	0	0,75	0,042
1,25	65292	0	0	0,02	0,06	0,65	0	0	0,27	0	0	0,24	0,11	0,65	0,044

1,25	57972	0	0	0,04	0	0	0,06	0	0,02	0,88	0,07	0,01	0,33	0,59	0,012
1,25	21977	0	0	0,01	0	0,77	0	0,22	0	0	0,77	0,01	0,22	0	0,048
1,25	40354	0,04	0	0,04	0	0	0,92	0	0	0	0	0,04	0,94	0,02	0,048
1,25	19360	0,04	0	0	0	0	0	0,02	0,94	0	0	0,06	0,47	0,47	0,046
1,25	69613	0	0,07	0,01	0,04	0	0	0	0,88	0	0,04	0,07	0,88	0,01	0,048
1,5	32157	0,01	0	0,04	0	0,95	0	0	0	0	0	0,03	0,96	0,01	0,02
1,5	86756	0	0,99	0	0	0	0	0	0,01	0	0,01	0,99	0	0	0,032
1,5	44500	0	0	0	0,2	0,8	0	0	0	0	0,8	0,13	0	0,07	0,046
1,5	6095	0	0	0	0,3	0	0,68	0,01	0,01	0	0,98	0	0,01	0,01	0,04
1,5	74029	0	0,02	0,04	0,01	0,62	0	0	0,01	0,3	0,31	0,04	0,03	0,62	0,032
1,5	10756	0,01	0	0,04	0	0,77	0	0	0,16	0,02	0,06	0,77	0,17	0	0,032
1,5	28977	0	0,06	0,02	0	0,89	0,01	0	0,02	0	0,92	0,07	0,01	0	0,048
1,5	7920	0	0,01	0,04	0,01	0,58	0	0	0,04	0,32	0,35	0,59	0	0,06	0,032
1,5	5561	0	0	0	0,12	0,77	0	0,11	0	0	0,86	0,02	0,12	0	0,038
1,5	36364	0	0,01	0	0,08	0	0	0	0,91	0	0	0,01	0,91	0,08	0,042
1,5	14891	0,01	0,03	0,04	0	0,91	0	0,01	0	0	0	0,91	0,05	0,04	0,026
1,5	34744	0,01	0,03	0,01	0	0,94	0	0,01	0	0	0,97	0,01	0,02	0	0,044
1,5	18411	0	0,12	0,01	0	0,83	0	0	0	0,04	0,56	0,01	0,39	0,04	0,044
1,5	19168	0	0	0	0,1	0,88	0	0	0	0,02	0,02	0	0,98	0	0,036
1,5	37930	0	0,14	0,04	0	0	0	0,01	0	0,81	0	0,12	0	0,88	0,048
1,5	37098	0	0	0	0,1	0	0,84	0,01	0	0,05	0,02	0,92	0,05	0,01	0,044
1,5	18568	0	0	0,03	0,15	0,44	0	0	0,33	0,05	0,89	0,02	0,01	0,08	0,038
1,5	71407	0	0	0	0,11	0,83	0	0	0,06	0	0,11	0,06	0,83	0	0,048
1,5	74585	0	1	0	0	0	0	0	0	0	0,08	0	0	0,92	0,048
1,5	13987	0	0	0,04	0,02	0,12	0	0,01	0,81	0	0,02	0,96	0,01	0,01	0,042
1,5	73519	0	0,01	0	0,06	0,93	0	0	0	0	0,07	0,93	0	0	0,032
1,5	40142	0	0	0	0,07	0,92	0	0	0,01	0	0,07	0,92	0,01	0	0,048
1,5	100000	0	0	1	0	0	0	0	0	0	0	1	0	0	0,706
1,5	21937	0	0	0,04	0,06	0	0,07	0	0,83	0	0,01	0,03	0	0,96	0,046
1,5	36572	0,05	0,11	0	0	0,84	0	0	0	0	0,05	0	0,11	0,84	0,048
1,5	15765	0	0,02	0,04	0	0	0	0	0	0,94	0,02	0,04	0,94	0	0,028
1,5	37207	0,05	0,04	0	0	0	0	0	0,15	0,76	0,01	0,14	0,04	0,81	0,038
1,5	6576	0	0	0,04	0	0,73	0,17	0,06	0	0	0,31	0,65	0,04	0	0,034

1,5	56759	0,06	0	0,01	0	0,92	0	0	0	0,01	0,06	0,02	0,92	0	0,04
1,5	15097	0	0,07	0,01	0,01	0,76	0,02	0	0,13	0	0,77	0,04	0,19	0	0,046
1,5	54057	0,05	0	0	0,03	0,88	0	0	0	0,04	0	0	0,96	0,04	0,048
1,5	68849	0	0,17	0,01	0	0	0,82	0	0	0	0,17	0,66	0,01	0,16	0,048
1,5	38475	0	0,32	0	0	0,67	0	0,01	0	0	0	0	0,33	0,67	0,048
1,5	46922	0,06	0	0	0	0	0,93	0	0,01	0	0,06	0,94	0	0	0,048
1,5	15519	0	0	0,04	0	0,69	0	0	0,27	0	0	0,05	0,95	0	0,02
1,5	9487	0	0,35	0	0	0,65	0	0	0	0	0	0,05	0,3	0,65	0,046
1,5	46482	0,01	0,06	0,04	0	0,86	0	0	0	0,03	0,02	0,01	0,06	0,91	0,012
1,5	19551	0	0	0	0,08	0,22	0,7	0	0	0	0	0,67	0,11	0,22	0,048
1,5	33904	0	0	0	0,1	0	0	0	0,9	0	0	0	0,9	0,1	0,048
1,5	14957	0	0,05	0	0,09	0,67	0,19	0	0	0	0,67	0	0,03	0,3	0,044
1,5	7602	0	0	0,04	0	0,14	0,12	0	0	0,7	0,06	0,04	0,9	0	0,036
1,5	5659	0	0	0,04	0	0	0,04	0	0	0,92	0	0,08	0	0,92	0,006
1,5	65043	0	0	0,04	0,07	0,88	0,01	0	0	0	0,15	0	0,16	0,69	0,048
1,5	6831	0,06	0	0,02	0	0,85	0,07	0	0	0	0,05	0,1	0,01	0,84	0,048
1,5	27673	0	0,13	0	0	0,78	0,08	0,01	0	0	0,09	0,13	0,78	0	0,048
1,5	92236	0,01	0	0	0,06	0,93	0	0	0	0	0,06	0,93	0	0,01	0,046
1,5	90908	0	0,06	0	0,07	0	0	0	0,87	0	0,06	0,87	0,01	0,06	0,048
1,5	42531	0	0	0	0,13	0,87	0	0	0	0	0	0	1	0	0,044
1,5	47940	0	0,22	0	0	0,78	0	0	0	0	0	0,78	0,22	0	0,046
1,5	100000	0,02	0,01	0,97	0	0	0	0	0	0	0	0,98	0	0,02	0,504
1,5	36511	0	0,01	0	0,11	0,04	0	0	0,84	0	0,05	0	0,84	0,11	0,048
1,5	16245	0,06	0	0	0	0,87	0,05	0	0,02	0	0,06	0,11	0	0,83	0,046
1,5	52681	0	0	0,04	0,06	0,44	0,46	0	0	0	0,44	0,04	0,52	0	0,042
1,5	55128	0,06	0	0,01	0	0,93	0	0	0	0	0,01	0	0,99	0	0,046
1,5	31566	0	0	0,04	0	0	0	0,01	0,93	0,02	0	0,26	0,68	0,06	0,026
1,5	48388	0	0,96	0	0,03	0,01	0	0	0	0	0,01	0	0,03	0,96	0,048
1,5	35835	0	0	0,04	0	0	0,04	0	0,81	0,11	0	0,11	0,08	0,81	0,01
1,5	43900	0	0,25	0	0	0,73	0	0	0,02	0	0,25	0,23	0,02	0,5	0,044
1,5	30851	0	0,92	0,02	0	0	0	0,02	0,04	0	0,04	0,03	0,01	0,92	0,048
1,5	61469	0,06	0	0	0	0	0,86	0	0,08	0	0,27	0,08	0,65	0	0,046
1,5	17544	0,01	0,08	0,02	0	0,86	0	0	0,03	0	0,11	0	0,89	0	0,044

1,5	39331	0	0,02	0,04	0	0,93	0,01	0	0	0	0,02	0,01	0,02	0,95	0
1,5	14293	0,04	0	0,04	0	0,44	0,48	0	0	0	0,48	0,04	0	0,48	0,044
1,5	84831	0	0,07	0	0,07	0,86	0	0	0	0	0,07	0	0,86	0,07	0,048
1,5	7969	0,05	0	0	0	0,02	0	0	0,93	0	0	0,95	0	0,05	0,044
1,5	10131	0	0,17	0,04	0,02	0,01	0	0	0	0,76	0	0,21	0,02	0,77	0,042
1,5	69573	0	0	0,04	0	0,01	0,02	0	0,93	0	0	0,12	0,48	0,4	0,006
1,5	20337	0,04	0	0,03	0	0,77	0,11	0,01	0,04	0	0,77	0,12	0,11	0	0,044
1,5	19909	0,04	0	0,04	0	0,91	0	0,01	0	0	0,04	0,05	0,91	0	0,048
1,5	41898	0,01	0	0	0	0,86	0,02	0,06	0	0,05	0	0,91	0,08	0,01	0,044
1,5	79408	0,04	0	0	0,05	0,38	0,32	0	0,09	0,12	0,09	0,32	0,59	0	0,048
1,5	42989	0	0,25	0	0	0,75	0	0	0	0	0	0,75	0,25	0	0,048
1,5	19840	0	0,99	0	0	0	0	0	0,01	0	0	0,98	0,02	0	0,046
1,5	8034	0,05	0	0	0,01	0,2	0	0	0,73	0,01	0,13	0,86	0	0,01	0,048
1,5	11585	0	0,04	0,04	0,02	0	0	0	0	0,9	0,02	0,09	0,06	0,83	0,024
1,5	49393	0	0,04	0	0,06	0	0,06	0,08	0,76	0	0,84	0,02	0,14	0	0,038
1,5	38668	0	0,25	0,02	0	0,52	0	0	0,21	0	0	0,22	0,26	0,52	0,04
1,5	2783	0	0	0,04	0	0	0	0	0,96	0	0	0,03	0,01	0,96	0
1,5	78603	0	0	0,04	0	0,07	0	0	0,74	0,15	0,07	0,61	0,17	0,15	0,014
1,5	16246	0	0,01	0,04	0	0	0	0	0,95	0	0,03	0	0,01	0,96	0,002
1,5	4862	0	0	0	0,14	0,82	0	0,04	0	0	0,82	0	0,18	0	0,048
1,5	41475	0	0	0,04	0	0,95	0,01	0	0	0	0,05	0,01	0,91	0,03	0,024
1,5	20439	0	0	0	0,33	0,66	0	0	0	0,01	0,99	0	0,01	0	0,04
1,5	35540	0	0	0,04	0	0,74	0	0	0,22	0	0	0,04	0,74	0,22	0,01
1,5	39522	0,03	0	0	0,07	0,73	0	0	0,17	0	0,68	0	0,29	0,03	0,036
1,5	31701	0,02	0	0,04	0	0,23	0	0	0,71	0	0,01	0,14	0,59	0,26	0,04
1,5	57052	0	0,12	0,04	0	0,82	0,02	0	0	0	0,41	0,12	0	0,47	0,048
1,5	19587	0,01	0,02	0,02	0,02	0,53	0,36	0,03	0,01	0	0,5	0,39	0,07	0,04	0,042
1,5	17020	0	0	0	0,26	0,73	0	0	0	0,01	0,93	0,01	0	0,06	0,048
1,5	100000	0	0	0,98	0	0,01	0	0,01	0	0	0	0,99	0	0,01	0,654
1,5	14011	0	0	0,04	0	0,32	0	0,03	0,03	0,58	0,03	0,04	0,58	0,35	0,04
1,5	43502	0	0	0,04	0	0,92	0,04	0	0	0	0,04	0,04	0,92	0	0,018
1,5	14740	0,06	0	0	0	0	0	0	0,93	0,01	0	0,99	0,01	0	0,048
1,5	100000	0	0,01	0,97	0,01	0	0,01	0	0	0	0,33	0,67	0	0	0,938

1,5	52792	0	0	0,02	0,09	0,09	0	0	0,43	0,37	0	0,14	0,45	0,41	0,046
1,5	64983	0	0	0,04	0	0	0,58	0	0	0,38	0	0,62	0,03	0,35	0,02
1,5	19904	0	0,25	0	0	0,73	0,01	0	0,01	0	0,18	0,57	0	0,25	0,048
1,5	12921	0	0,95	0,01	0,01	0	0	0	0	0,03	0,02	0	0,03	0,95	0,042
1,5	100000	0	0,09	0,87	0	0	0,04	0	0	0	0,08	0,88	0,04	0	0,6
1,5	7525	0	0	0,04	0	0	0	0	0,96	0	0,03	0	0,01	0,96	0,004
1,75	38475	0,01	0,11	0	0,04	0	0,04	0	0,8	0	0,04	0,84	0,04	0,08	0,048
1,75	19376	0,01	0	0,04	0,04	0,02	0,89	0	0	0	0,04	0	0,01	0,95	0,048
1,75	100000	0	0	0,98	0,02	0	0	0	0	0	0,02	0,98	0	0	0,738
1,75	34813	0,05	0	0	0	0	0,94	0	0	0,01	0,03	0,95	0	0,02	0,048
1,75	12020	0,01	0	0,04	0	0	0	0,01	0,21	0,73	0	0,05	0,21	0,74	0,022
1,75	14342	0	0,27	0	0,01	0,72	0	0	0	0	0	0,01	0,26	0,73	0,046
1,75	48364	0	0,01	0,04	0,01	0,94	0	0	0	0	0,06	0	0,94	0	0,028
1,75	100000	0	0,02	0,96	0	0,01	0	0,01	0	0	0,03	0,95	0,02	0	0,598
1,75	23779	0	0	0	0,14	0,77	0,08	0,01	0	0	0,78	0	0,06	0,16	0,042
1,75	57304	0	0,01	0	0,21	0,76	0	0,01	0,01	0	0,77	0,22	0,01	0	0,048
1,75	15687	0	0	0,03	0,08	0,02	0,01	0	0,86	0	0,03	0,01	0,88	0,08	0,032
1,75	54949	0	0,19	0,04	0	0	0	0	0,77	0	0,19	0,04	0	0,77	0,044
1,75	47993	0	0,3	0	0	0,69	0	0	0	0,01	0,01	0,05	0,25	0,69	0,038
1,75	15540	0	0	0,04	0	0,87	0	0,09	0	0	0,09	0,82	0,09	0	0,038
1,75	5864	0	0	0,04	0	0	0	0	0,96	0	0,96	0,04	0	0	0,038
1,75	16425	0,04	0	0,04	0	0,5	0,42	0	0	0	0,77	0,06	0,17	0	0,048
1,75	21363	0	0	0,04	0	0,92	0	0	0,04	0	0	0	1	0	0,026
1,75	5305	0,01	0,94	0,01	0,03	0,01	0	0	0	0	0,01	0,01	0,01	0,97	0,046
1,75	62311	0,06	0	0	0	0	0,82	0	0	0,12	0,18	0	0,82	0	0,048
1,75	28619	0	0	0,04	0,01	0	0,85	0	0	0,1	0	0,05	0,85	0,1	0,038
1,75	5380	0	0	0	0,07	0,91	0,02	0	0	0	0,09	0,91	0	0	0,046
1,75	98565	0,06	0	0	0	0,11	0,24	0	0,59	0	0,07	0,09	0,84	0	0,046
1,75	61463	0	0	0,03	0,03	0,91	0	0,03	0	0	0,03	0	0	0,97	0,046
1,75	100000	0	0	0,99	0,01	0	0	0	0	0	0,01	0,99	0	0	0,69
1,75	54733	0,01	0	0	0,08	0	0,53	0	0	0,38	0,05	0,35	0	0,6	0,044
1,75	84684	0	0	0	0,06	0,8	0,14	0	0	0	0,06	0,54	0,01	0,39	0,046
1,75	42810	0,04	0	0	0,04	0,58	0,19	0	0,04	0,11	0,19	0	0,73	0,08	0,046

1,75	13700	0	0,72	0	0,03	0	0	0	0,2	0,05	0	0,75	0	0,25	0,044
1,75	53703	0,04	0,01	0,03	0	0,9	0,01	0,01	0	0	0,04	0,03	0,88	0,05	0,048
1,75	20653	0,02	0	0	0,14	0,84	0	0	0	0	0,86	0,14	0	0	0,044
1,75	78392	0	0	0	0	0,78	0,01	0,12	0,08	0,01	0,01	0,01	0,9	0,08	0,04
1,75	100000	0	0,05	0,91	0	0	0	0,01	0,03	0	0	0,96	0,01	0,03	0,482
1,75	18597	0	0,93	0	0,01	0	0	0	0,02	0,04	0,02	0,01	0,04	0,93	0,042
1,75	10284	0	0,05	0,03	0,04	0	0,56	0	0	0,32	0	0,22	0,32	0,46	0,048
1,75	13471	0	0	0,01	0	0,1	0,81	0,08	0	0	0,08	0	0,82	0,1	0,046
1,75	89509	0	0	0,04	0,02	0,85	0	0	0,09	0	0	0,13	0	0,87	0,022
1,75	40170	0,04	0,06	0	0	0,78	0	0	0	0,12	0	0,79	0,05	0,16	0,036
1,75	18805	0	0,11	0	0	0,53	0,19	0	0,15	0,02	0,21	0,26	0,53	0	0,046
1,75	33229	0	0,2	0,04	0	0,42	0	0	0,34	0	0,17	0,63	0	0,2	0,04
1,75	7496	0,04	0	0,02	0	0	0	0	0,91	0,03	0	0,09	0,91	0	0,044
1,75	32248	0	0	0,04	0	0	0	0	0,96	0	0,6	0,13	0,04	0,23	0,034
1,75	4185	0,01	0	0,04	0,01	0,78	0	0,01	0,15	0	0,01	0,82	0,01	0,16	0,018
1,75	31800	0,04	0	0,04	0	0	0,05	0	0,87	0	0,05	0,04	0,04	0,87	0,042
1,75	7437	0,05	0	0,01	0	0	0	0	0,94	0	0	0,99	0	0,01	0,032
1,75	23657	0	0	0,04	0,05	0,71	0,2	0	0	0	0,9	0,06	0,01	0,03	0,048
1,75	63757	0	0	0	0,13	0,87	0	0	0	0	0,29	0	0,71	0	0,04
1,75	17625	0	0	0,04	0	0	0,03	0	0,78	0,15	0,78	0	0,15	0,07	0,028
1,75	24671	0	0,03	0,01	0	0	0	0,09	0,87	0	0	0,04	0,87	0,09	0,046
1,75	39690	0,06	0	0	0	0,88	0,06	0	0	0	0,93	0	0	0,07	0,048
1,75	44772	0	0	0,04	0	0,06	0	0	0,9	0	0	0,1	0,9	0	0,024
1,75	34982	0	0	0,01	0,12	0,03	0,03	0	0,81	0	0,01	0,02	0,9	0,07	0,048
1,75	20022	0	0,04	0	0	0,74	0	0,12	0,1	0	0,86	0	0,14	0	0,046
1,75	8226	0,01	0,01	0,03	0,07	0,88	0	0	0	0	0,89	0,02	0,01	0,08	0,048
1,75	100000	0	0	0,97	0	0	0	0,01	0,01	0,01	0	0,97	0,02	0,01	0,508
1,75	10162	0	0	0,02	0,05	0,93	0	0	0	0	0,01	0,05	0	0,94	0,038
1,75	14757	0,05	0	0,04	0	0,85	0,06	0	0	0	0	0,04	0,05	0,91	0,046
1,75	16136	0,03	0	0	0,13	0	0,83	0	0,01	0	0,83	0,16	0	0,01	0,048
1,75	11024	0	0	0,04	0,01	0	0,14	0	0,79	0,02	0,21	0,04	0,37	0,38	0,034
1,75	100000	0	0,07	0,92	0	0	0	0	0	0,01	0,07	0,93	0	0	0,754
1,75	20154	0	0	0	0,19	0,65	0,14	0	0,02	0	0,76	0	0	0,24	0,044

1,75	19014	0	0,06	0,04	0	0,25	0	0	0	0,65	0	0,38	0	0,62	0,022
1,75	7387	0	0,02	0,03	0,04	0,62	0	0	0,29	0	0,29	0,07	0	0,64	0,042
1,75	27325	0	0,26	0,01	0	0,73	0	0	0	0	0	0,73	0,27	0	0,042
1,75	29267	0,05	0	0	0	0,95	0	0	0	0	0	0,32	0,67	0,01	0,04
1,75	6425	0	0	0,04	0	0	0	0	0,34	0,62	0,01	0,19	0	0,8	0,008
1,75	100000	0,43	0,05	0,12	0	0	0,14	0	0,07	0,19	0,47	0,21	0,22	0,1	0,514
1,75	27786	0,04	0	0	0,04	0,87	0,05	0	0	0	0,08	0,05	0,87	0	0,044
1,75	34172	0	0,05	0	0	0,91	0	0,04	0	0	0,04	0	0,91	0,05	0,046
1,75	60704	0	0,08	0	0	0,87	0,03	0,01	0	0,01	0,95	0,01	0	0,04	0,044
1,75	100000	0	0	0,99	0	0	0	0	0,01	0	0	0,99	0,01	0	0,594
1,75	17956	0	0	0,04	0	0,47	0,49	0	0	0	0,49	0	0	0,51	0,016
1,75	49553	0	0,99	0	0	0	0	0	0	0,01	0	0,99	0,01	0	0,04
1,75	6688	0	0	0	0,09	0,9	0,01	0	0	0	0,08	0,02	0,9	0	0,044
1,75	81901	0,04	0	0,04	0	0,92	0	0	0	0	0	0,04	0,96	0	0,048
1,75	57838	0	0	0,04	0,03	0,75	0,15	0	0	0,03	0,15	0,75	0,07	0,03	0,04
1,75	98073	0,07	0	0	0	0,93	0	0	0	0	1	0	0	0	0,048
1,75	7485	0,02	0,01	0	0,04	0	0,02	0,03	0,88	0	0,05	0,01	0,89	0,05	0,048
1,75	15644	0	0	0	0,12	0,88	0	0	0	0	0,12	0	0,88	0	0,044
1,75	17175	0	0	0	0,1	0	0	0,1	0,66	0,14	0,74	0,14	0	0,12	0,042
1,75	62553	0	0,05	0,03	0	0,46	0,18	0,07	0	0,21	0,64	0,1	0,17	0,09	0,048
1,75	21578	0	0,21	0	0	0,78	0,01	0	0	0	0,21	0,78	0,01	0	0,048
1,75	17348	0	0,01	0,03	0,06	0	0	0	0	0,9	0	0,03	0,01	0,96	0,046
1,75	22123	0	0,21	0,02	0,01	0,76	0	0	0	0	0	0,47	0,23	0,3	0,048
1,75	46529	0	0,2	0,01	0,03	0	0,01	0	0,75	0	0	0,04	0,23	0,73	0,046
1,75	24601	0	0,12	0	0	0,88	0	0	0	0	0,11	0	0,89	0	0,048
1,75	17966	0,01	0	0,04	0	0,94	0	0	0	0,01	0,95	0	0,05	0	0,046
1,75	5592	0,04	0	0	0	0	0	0,03	0,93	0	0,03	0,04	0,93	0	0,044
1,75	18020	0	0	0,04	0	0,17	0,57	0	0	0,22	0,6	0,08	0,22	0,1	0,032
1,75	27974	0	0	0,04	0,02	0	0	0	0	0,94	0,01	0,04	0,95	0	0,044
1,75	38197	0	0,06	0	0	0	0,87	0,04	0,01	0,02	0,06	0,43	0,48	0,03	0,042
1,75	58494	0	0,07	0	0,02	0,01	0,4	0	0,5	0	0,4	0,03	0,53	0,04	0,038
1,75	21092	0,01	0	0	0,16	0,79	0	0	0,04	0	0,82	0,1	0	0,08	0,046
1,75	35328	0	0	0	0,23	0,76	0,01	0	0	0	0,76	0	0,23	0,01	0,048

1,75	78234	0	0,2	0	0	0,76	0,03	0	0,01	0	0,27	0	0,21	0,52	0,04
1,75	4353	0	0	0,04	0	0	0	0	0,75	0,21	0	0,04	0	0,96	0,008
1,75	34124	0,03	0	0,01	0,04	0,92	0	0	0	0	0,03	0,02	0,92	0,03	0,04
1,75	12953	0,01	0	0	0,05	0,87	0,05	0	0	0,02	0,05	0,01	0,08	0,86	0,04
1,75	38631	0	0,04	0	0,05	0,91	0	0	0	0	0	0,04	0,03	0,93	0,046
1,75	20182	0	0,1	0	0	0,88	0,02	0	0	0	0,77	0	0,12	0,11	0,044
1,75	100000	0	0	1	0	0	0	0	0	0	0	0,89	0,11	0	0,518
2	97350	0	0	0	0,12	0,88	0	0	0	0	0,12	0	0,88	0	0,044
2	2021	0	0	0,04	0	0,9	0	0	0,06	0	0,9	0,1	0	0	0,04
2	100000	0	0	0,99	0,01	0	0	0	0	0	0	0,96	0,01	0,03	0,566
2	88782	0,06	0	0	0	0,94	0	0	0	0	1	0	0	0	0,042
2	85513	0	0,32	0	0	0,67	0	0	0	0,01	0	0	0,33	0,67	0,048
2	72410	0,05	0,01	0	0	0,01	0,9	0,02	0,01	0	0,06	0,88	0,06	0	0,046
2	66821	0	0	0,04	0,03	0	0	0	0,93	0	0	0,06	0,01	0,93	0,032
2	5579	0	0,02	0,04	0,02	0,24	0,06	0	0,62	0	0,29	0,04	0,06	0,61	0,02
2	50850	0	0,01	0,04	0	0,94	0	0	0,01	0	0,04	0	0,22	0,74	0,014
2	68540	0,04	0,03	0,01	0,01	0,91	0	0	0	0	0,01	0,03	0,94	0,02	0,048
2	14992	0	0	0,04	0	0	0,09	0,05	0	0,82	0,05	0,04	0,86	0,05	0,048
2	13362	0,04	0	0	0	0,72	0	0,07	0,17	0	0,72	0,28	0	0	0,048
2	18137	0	0	0,04	0,01	0	0,95	0	0	0	0	0,05	0	0,95	0,012
2	3378	0,04	0	0,04	0	0,09	0	0	0,75	0,08	0,01	0,08	0,84	0,07	0,046
2	11285	0	0	0,02	0,08	0,49	0	0	0,41	0	0	0	0,59	0,41	0,048
2	52843	0	0,13	0	0	0,86	0	0,01	0	0	0	0,01	0,99	0	0,048
2	100000	0	0,03	0,97	0	0	0	0	0	0	0	1	0	0	0,742
2	23758	0	0	0	0	0,53	0,36	0,11	0	0	0,36	0	0,31	0,33	0,048
2	65687	0	0,29	0	0	0,69	0	0	0,02	0	0,31	0	0	0,69	0,048
2	61753	0,05	0	0	0	0	0,95	0	0	0	0,05	0	0,95	0	0,046
2	17893	0	0,25	0	0	0,75	0	0	0	0	0	0,82	0,18	0	0,048
2	31417	0	0,03	0,03	0	0,63	0,01	0,06	0,24	0	0,9	0,01	0,06	0,03	0,048
2	12389	0	0,07	0,04	0	0,02	0	0	0,87	0	0	0,07	0,83	0,1	0,048
2	22253	0	0	0,04	0,01	0,95	0	0	0	0	0,01	0,04	0,23	0,72	0,022
2	7370	0	0	0	0,1	0,83	0,07	0	0	0	0	0,1	0,83	0,07	0,048
2	24168	0,02	0,01	0,04	0	0	0	0,01	0,92	0	0,01	0,07	0,21	0,71	0,042

2	42895	0	0	0,04	0	0	0	0	0,96	0	0	0,04	0	0,96	0
2	45203	0	0,16	0	0	0,19	0	0	0,4	0,25	0,03	0	0,81	0,16	0,044
2	45321	0	0	0	0,06	0	0,75	0,17	0,02	0	0,75	0,23	0	0,02	0,046
2	24998	0,03	0,01	0,04	0	0,05	0,6	0	0,27	0	0,03	0	0,9	0,07	0,048
2	64910	0	0,04	0	0,08	0,84	0	0,04	0	0	0,9	0,08	0	0,02	0,042
2	100000	0	0	0,96	0	0	0,02	0	0,02	0	0,02	0,96	0	0,02	0,554
2	24009	0	0,08	0,04	0	0,01	0	0	0,01	0,86	0	0,02	0,11	0,87	0,026
2	9136	0	0,04	0	0,11	0,85	0	0	0	0	0,52	0,04	0,44	0	0,042
2	8406	0	0,1	0	0,02	0,88	0	0	0	0	0	0,07	0,93	0	0,04
2	100000	0	0	0,99	0	0	0	0	0	0,01	0	0,99	0,01	0	0,632
2	19600	0	0,03	0	0,18	0,79	0	0	0	0	0,76	0,06	0,18	0	0,046
2	13711	0,02	0	0	0,04	0,89	0,01	0	0,04	0	0	0,89	0,07	0,04	0,048
2	11487	0	0	0,01	0	0,85	0	0,08	0,06	0	0,01	0,04	0,29	0,66	0,048
2	60743	0,01	0	0	0	0	0	0,12	0,87	0	0	0,12	0,88	0	0,042
2	19245	0,07	0,02	0,02	0	0,89	0	0	0	0	0,07	0,92	0	0,01	0,048
2	52484	0	0,06	0	0,08	0	0,65	0	0,21	0	0,09	0,14	0,77	0	0,036
2	11543	0	0,19	0	0	0,8	0	0,01	0	0	0	0,79	0,19	0,02	0,044
2	85762	0	0,22	0	0	0,64	0,12	0	0	0,02	0,24	0,14	0	0,62	0,04
2	12553	0	0,23	0,04	0	0,73	0	0	0	0	0,24	0,03	0	0,73	0,03
2	32155	0	0,29	0	0	0,71	0	0	0	0	0	0	0,29	0,71	0,034
2	100000	0	0	0	1	0	0	0	0	0	1	0	0	0	0,182
2	92882	0,01	0	0,04	0	0,1	0,01	0,01	0,83	0	0	0,87	0,03	0,1	0,028
2	65033	0,03	0,07	0,04	0	0,81	0	0	0	0,05	0	0,1	0,56	0,34	0,048
2	20064	0,02	0	0,04	0	0,67	0	0	0,27	0	0,02	0,6	0,11	0,27	0,026
2	26632	0	0,19	0,03	0	0,47	0,02	0	0	0,29	0	0,94	0,06	0	0,044
2	27929	0,01	0,06	0	0	0,06	0	0	0,87	0	0,93	0	0,07	0	0,048
2	10997	0,04	0	0,03	0	0,08	0	0,01	0	0,84	0,04	0,03	0	0,93	0,044
2	7251	0	0,08	0	0	0,07	0,85	0	0	0	0,93	0	0,07	0	0,044
2	6770	0,04	0	0,01	0,01	0,93	0	0	0	0,01	0,94	0,02	0,04	0	0,036
2	100000	0	0	0,99	0	0,01	0	0	0	0	1	0	0	0	0,974
2	59828	0,02	0	0,04	0	0,86	0,08	0	0	0	0,01	0,07	0,9	0,02	0,044
2	15225	0	0	0,04	0	0,89	0,02	0,01	0	0,04	0,02	0,93	0,01	0,04	0,018
2	100000	0	0	0,99	0	0,01	0	0	0	0	0,01	0,99	0	0	0,724

2	61268	0	0	0,03	0	0	0,01	0,06	0,9	0	0,06	0,03	0,01	0,9	0,042
2	63976	0	0,12	0	0	0,86	0,02	0	0	0	0,08	0,02	0,9	0	0,046
2	14021	0	0,05	0,01	0	0,85	0	0,05	0,04	0	0	0,04	0,9	0,06	0,046
2	100000	0,14	0	0,85	0	0	0	0,01	0	0	0	1	0	0	0,682
2	56131	0,02	0	0,04	0	0,94	0	0	0	0	0,06	0,36	0	0,58	0,034
2	20063	0	0	0,04	0	0,81	0,1	0	0	0,05	0,04	0,11	0	0,85	0
2	26301	0	0	0	0,07	0	0	0	0,93	0	0,04	0,93	0	0,03	0,042
2	22980	0	0	0,04	0	0,69	0,18	0,06	0	0,03	0,26	0,01	0,54	0,19	0,04
2	38276	0	0,02	0,04	0	0	0,87	0,07	0	0	0,01	0,29	0,59	0,11	0,048
2	92065	0	0	0,04	0	0,72	0	0,05	0,19	0	0,19	0,04	0,05	0,72	0,044
2	19574	0	0	0	0	0,81	0	0,12	0	0,07	0	0	1	0	0,042
2	3639	0,02	0,27	0,01	0	0	0	0	0,62	0,08	0	0,01	0,35	0,64	0,048
2	67728	0	0,2	0	0	0,44	0,35	0	0	0,01	0,16	0,22	0,17	0,45	0,048
2	100000	0,37	0,03	0,6	0	0	0	0	0	0	0,22	0,61	0,17	0	0,806
2	40688	0,04	0	0,04	0	0	0	0	0,92	0	0,04	0,92	0	0,04	0,038
2	68206	0	0,04	0,04	0	0,38	0,11	0	0,43	0	0	0,07	0,65	0,28	0,034
2	34774	0	0,04	0,04	0	0	0,49	0	0,43	0	0,04	0	0,92	0,04	0,042
2	19163	0,02	0,96	0,01	0	0	0	0,01	0	0	0,01	0	0,01	0,98	0,032
2	37189	0	0	0	0,07	0	0,93	0	0	0	0,05	0,07	0	0,88	0,046
2	18672	0	0	0	0	0	0,79	0,17	0	0,04	0,79	0	0,17	0,04	0,048
2	17231	0	0	0,04	0	0,94	0	0	0,02	0	0	0,01	0,07	0,92	0,004
2	15329	0,04	0,02	0,03	0	0,27	0,23	0	0,16	0,25	0,2	0,53	0,02	0,25	0,048
2	22739	0,06	0	0	0	0	0,94	0	0	0	0	0	0,04	0,96	0,044
2	100000	0	0,03	0,93	0	0	0,01	0,03	0	0	0,01	0,98	0,01	0	0,522
2	17204	0,04	0,06	0	0	0	0,8	0,02	0	0,08	0,04	0,66	0,22	0,08	0,046
2	76331	0	0	0	0	0,67	0	0,09	0,24	0	0,28	0	0	0,72	0,046
2	72401	0,04	0,05	0,04	0	0,34	0,53	0	0	0	0,01	0,37	0,09	0,53	0,042
2	41212	0	0,1	0	0,03	0,87	0	0	0	0	0	0,03	0,9	0,07	0,044
2	30427	0,02	0,05	0	0	0,9	0,02	0	0,01	0	0,94	0,03	0	0,03	0,048
2	32557	0	0	0	0,08	0,82	0	0	0,1	0	0	0,88	0,1	0,02	0,046
2	7953	0	0	0,04	0	0	0,05	0	0,38	0,53	0,84	0,16	0	0	0,046
2	52641	0,05	0	0,01	0	0,02	0	0	0,92	0	0	0,06	0,94	0	0,044
2	34053	0	0,07	0	0	0,81	0,08	0,04	0	0	0,85	0	0,08	0,07	0,048

2	73586	0	0	0,04	0,01	0,01	0	0,06	0,85	0,03	0,2	0,1	0,02	0,68	0,046
2	100000	0	0	0,99	0	0	0	0	0	0,01	0	1	0	0	0,606
2	50771	0,01	0	0	0,03	0,95	0	0,01	0	0	0,01	0	0	0,99	0,044
2	100000	0,01	0	0,99	0	0	0	0	0	0	0	0,99	0,01	0	0,604
2	22222	0	0	0,03	0,06	0,36	0	0,01	0,04	0,5	0,06	0,32	0,04	0,58	0,048
2	28905	0	0	0,04	0	0,32	0,12	0,01	0,13	0,38	0,01	0,5	0,05	0,44	0,018
2	100000	0	0,06	0,91	0	0	0,03	0	0	0	0,05	0,91	0,01	0,03	0,584
2	23520	0	0	0,04	0	0	0,32	0	0,02	0,62	0,05	0,04	0,59	0,32	0,026
2,25	89333	0	0,12	0	0,03	0,85	0	0	0	0	0,15	0,85	0	0	0,044
2,25	11823	0,05	0,03	0	0	0,59	0	0	0,1	0,23	0,08	0,23	0,09	0,6	0,048
2,25	9860	0,02	0,19	0	0	0,79	0	0	0	0	0,02	0	0,19	0,79	0,046
2,25	24543	0,03	0	0	0,04	0,77	0	0	0,16	0	0,17	0,55	0,24	0,04	0,044
2,25	78302	0	0	0,04	0	0,11	0,85	0	0	0	0,85	0,02	0,09	0,04	0,03
2,25	15092	0,01	0,02	0,04	0,01	0	0	0	0	0,92	0,01	0,06	0	0,93	0,028
2,25	60521	0	0	0,04	0	0	0,93	0,03	0	0	0	0,02	0,05	0,93	0,036
2,25	20077	0	0	0,04	0	0,01	0	0	0,01	0,94	0	0,04	0,01	0,95	0
2,25	13819	0	0	0	0,01	0	0	0,05	0,94	0	0	0,06	0	0,94	0,046
2,25	26439	0,02	0	0,03	0,03	0	0	0	0,92	0	0,04	0,13	0,02	0,81	0,048
2,25	28989	0,05	0	0	0	0	0,32	0,01	0,52	0,1	0	0	0,48	0,52	0,048
2,25	17568	0	0	0	0,11	0,89	0	0	0	0	0	0,11	0,89	0	0,046
2,25	12591	0	0	0,04	0,02	0	0	0	0	0,94	0,01	0,02	0,02	0,95	0,028
2,25	25653	0	0,02	0,04	0	0,4	0,53	0	0	0,01	0	0,04	0,96	0	0,032
2,25	62680	0	0,1	0	0	0,9	0	0	0	0	0,52	0	0,48	0	0,042
2,25	54327	0	0,97	0,02	0,01	0	0	0	0	0	0	0	0,03	0,97	0,042
2,25	37359	0	0	0,04	0,01	0,75	0,13	0	0,07	0	0,72	0,19	0,07	0,02	0,042
2,25	17748	0	0	0,04	0	0,63	0	0	0	0,33	0,65	0,03	0,06	0,26	0,034
2,25	9313	0	0	0,04	0	0,96	0	0	0	0	0	0,04	0,96	0	0,03
2,25	28913	0	0,01	0,02	0,15	0,8	0	0,02	0	0	0,92	0	0,03	0,05	0,044
2,25	13667	0	0	0	0,11	0,89	0	0	0	0	0	0,11	0,89	0	0,048
2,25	7081	0	0	0,03	0	0,09	0,19	0,09	0,6	0	0	0,63	0,37	0	0,044
2,25	77254	0	0,88	0,01	0	0,01	0	0	0,04	0,06	0,04	0,07	0,01	0,88	0,044
2,25	71462	0,04	0	0,02	0	0,94	0	0	0	0	0,97	0,02	0	0,01	0,048
2,25	11237	0	0	0,04	0	0,43	0	0	0,34	0,19	0,34	0,11	0,55	0	0,02

2,25	25370	0,01	0	0,03	0	0,93	0	0,03	0	0	0,01	0,03	0,03	0,93	0,036
2,25	17617	0	0	0	0,12	0,83	0,05	0	0	0	0,12	0	0,83	0,05	0,044
2,25	5486	0,01	0	0,04	0	0	0	0	0	0,95	0,01	0,04	0	0,95	0,018
2,25	17897	0,03	0	0,04	0,01	0,86	0,06	0	0	0	0,01	0,89	0,09	0,01	0,038
2,25	18734	0	0	0,04	0	0,96	0	0	0	0	0,96	0,04	0	0	0,038
2,25	11111	0,05	0	0,02	0	0,01	0,91	0,01	0	0	0	0	0,94	0,06	0,046
2,25	18280	0	0	0,04	0	0	0,73	0	0,23	0	0,04	0	0,77	0,19	0,022
2,25	22398	0	0,4	0,04	0	0,13	0	0	0	0,43	0	0,08	0	0,92	0,046
2,25	22042	0	0,01	0	0,1	0,89	0	0	0	0	0,1	0,01	0,89	0	0,044
2,25	26054	0	0	0,04	0	0,94	0	0	0,02	0	0,04	0	0,96	0	0,014
2,25	27517	0	0	0	0,06	0,94	0	0	0	0	0,06	0,94	0	0	0,044
2,25	36845	0	0,05	0,04	0	0	0,02	0	0,11	0,78	0,02	0,17	0,79	0,02	0,046
2,25	34817	0	0	0,04	0,04	0	0	0	0,92	0	0,1	0,03	0,86	0,01	0,044
2,25	100000	0	0	0,98	0	0	0	0,01	0	0,01	0	0,98	0,01	0,01	0,508
2,25	12405	0	0	0	0,33	0,67	0	0	0	0	1	0	0	0	0,044
2,25	90203	0	0	0,04	0	0	0,63	0	0,33	0	0,02	0,28	0,08	0,62	0,016
2,25	5130	0	0	0,04	0	0,92	0,03	0,01	0	0	0	0,94	0,06	0	0,016
2,25	8237	0	0	0,04	0	0,95	0	0,01	0	0	0,63	0	0,37	0	0,028
2,25	21331	0	0	0,04	0	0,92	0	0	0,04	0	0,92	0	0,04	0,04	0,038
2,25	39403	0	0,14	0	0,03	0,04	0,02	0	0,77	0	0,13	0,56	0,02	0,29	0,044
2,25	11278	0	0,01	0,04	0	0,01	0	0	0,92	0,02	0,01	0,01	0,02	0,96	0
2,25	33080	0	0,13	0,01	0	0	0,01	0	0,84	0,01	0,33	0	0,35	0,32	0,048
2,25	61142	0	0	0	0,18	0,6	0,22	0	0	0	0,64	0,13	0	0,23	0,048
2,25	52619	0,03	0,05	0,01	0	0,02	0,87	0,02	0	0	0,03	0,89	0,02	0,06	0,044
2,25	40191	0	0	0	0,12	0,85	0,03	0	0	0	0,32	0,64	0,03	0,01	0,04
2,25	64122	0	0,94	0	0,03	0,03	0	0	0	0	0,03	0,33	0	0,64	0,048
2,25	60473	0	0	0,04	0	0,07	0	0	0	0,89	0	0,04	0	0,96	0,016
2,25	51592	0,06	0	0	0,01	0	0,88	0	0,05	0	0,06	0,39	0,5	0,05	0,042
2,25	90188	0	0,02	0	0	0,09	0,07	0,08	0,65	0,09	0,02	0,18	0,7	0,1	0,048
2,25	73210	0	0	0	0,17	0,69	0,02	0	0,12	0	0,71	0,17	0	0,12	0,042
2,25	42603	0	0	0	0,1	0,89	0	0	0,01	0	0,45	0,1	0,37	0,08	0,036
2,25	16971	0	0	0	0,23	0,77	0	0	0	0	0,77	0	0,23	0	0,046
2,25	37292	0	0	0	0,11	0	0,06	0	0,83	0	0	0,11	0,62	0,27	0,042

2,25	61537	0,04	0,07	0,04	0	0,72	0,01	0	0	0,12	0,04	0,23	0,24	0,49	0,048
2,25	100000	0	0	1	0	0	0	0	0	0	0	1	0	0	0,636
2,25	29548	0,01	0	0,04	0,01	0,02	0,64	0	0	0,28	0,05	0,15	0,02	0,78	0,026
2,25	33397	0,05	0,05	0	0	0	0,29	0	0,61	0	0,05	0	0,05	0,9	0,044
2,25	100000	0	0,42	0	0	0,22	0,31	0,05	0	0	0,44	0,1	0,46	0	0,31
2,25	2580	0	0	0,03	0	0,02	0	0,09	0,8	0,06	0	0,07	0,86	0,07	0,036
2,25	20745	0	0,01	0	0,05	0,07	0	0,05	0,82	0	0,11	0,01	0,71	0,17	0,036
2,25	17402	0	0	0	0,1	0	0,18	0	0,67	0,05	0,49	0,1	0	0,41	0,036
2,25	7875	0	0,06	0	0,03	0,9	0	0	0	0,01	0,9	0,03	0,06	0,01	0,048
2,25	6106	0	0,14	0,02	0	0,46	0,11	0	0	0,27	0,48	0	0,17	0,35	0,048
2,25	17442	0	0	0,03	0,07	0,01	0	0,02	0	0,87	0,07	0,04	0,02	0,87	0,044
2,25	52449	0	0,46	0,01	0,03	0,49	0	0,01	0	0	0,03	0,47	0,01	0,49	0,048
2,25	31452	0,01	0	0	0,03	0,93	0,02	0,01	0	0	0	0,03	0	0,97	0,032
2,25	26145	0	0	0	0,16	0	0,84	0	0	0	0,16	0	0,84	0	0,048
2,25	14621	0	0	0,04	0,03	0	0	0,01	0,92	0	0,01	0,03	0,03	0,93	0,042
2,25	69609	0,01	0	0,04	0	0,94	0	0	0,01	0	0	0,05	0,94	0,01	0,024
2,25	6725	0,01	0	0,03	0,04	0,7	0,14	0,01	0,05	0,02	0,04	0,73	0,16	0,07	0,046
2,25	15497	0,01	0	0,04	0,05	0,9	0	0	0	0	0,05	0,44	0,51	0	0,042
2,25	3769	0	0	0,04	0	0	0	0	0	0,96	0	0,03	0,01	0,96	0,012
2,25	32594	0,01	0	0,01	0,04	0,91	0	0	0,02	0,01	0	0,07	0,02	0,91	0,048
2,25	10114	0,01	0,76	0,04	0	0,19	0	0	0	0	0,04	0,17	0,03	0,76	0,032
2,25	29684	0	0	0,04	0	0	0	0,01	0,95	0	0	0	0,05	0,95	0,012
2,25	20496	0	0,01	0	0,17	0,82	0	0	0	0	0,82	0	0,18	0	0,048
2,25	28661	0,05	0	0	0	0,31	0,14	0	0,5	0	0,5	0	0	0,5	0,04
2,25	18969	0	0	0,03	0,11	0,46	0,1	0	0,3	0	0,11	0,01	0,79	0,09	0,042
2,25	100000	0	0	0,99	0	0	0	0	0	0,01	0	1	0	0	0,712
2,25	15013	0	0,89	0,02	0	0,01	0	0,04	0,04	0	0	0,11	0	0,89	0,044
2,25	2797	0	0	0,04	0	0,02	0	0	0,93	0,01	0,02	0,96	0	0,02	0,004
2,25	59887	0	0	0	0,12	0,86	0,02	0	0	0	0,02	0	0,95	0,03	0,044
2,25	1872	0	0,02	0,04	0	0	0	0,03	0,9	0,01	0	0,04	0,01	0,95	0,036
2,25	17714	0	0,3	0	0	0,02	0	0	0,68	0	0	0	0,38	0,62	0,048
2,25	36014	0	0	0	0,22	0,78	0	0	0	0	0,78	0,22	0	0	0,046
2,25	36155	0	0	0,02	0,1	0	0,76	0,03	0	0,09	0,05	0,14	0,66	0,15	0,048

2,25	28125	0	0	0,04	0,09	0,85	0,02	0	0	0	0	0,15	0,79	0,06	0,04
2,25	30792	0	0,1	0	0,02	0,88	0	0	0	0	0,1	0	0,89	0,01	0,048
2,25	34441	0	0,05	0,02	0,04	0,85	0,02	0,01	0	0,01	0,9	0,02	0,03	0,05	0,044
2,25	36937	0	0,25	0	0,02	0,72	0	0	0	0,01	0,25	0,02	0,02	0,71	0,048
2,25	100000	0,14	0	0,76	0,07	0	0	0,03	0	0	0,07	0,76	0,14	0,03	0,604
2,25	30477	0	0	0,04	0	0,94	0	0,02	0	0	0,04	0,02	0,94	0	0,044
2,25	52266	0	0	0	0,06	0,32	0	0	0,62	0	0	0,31	0,07	0,62	0,048
2,25	25030	0	0,31	0	0	0,67	0	0	0,02	0	0	0,02	0,31	0,67	0,04
2,25	59652	0,04	0	0	0,04	0,92	0	0	0	0	0,06	0,04	0,9	0	0,048
2,5	52951	0,06	0,02	0	0	0,86	0,06	0	0	0	0,04	0,04	0,86	0,06	0,048
2,5	38434	0	0	0	0	0,85	0	0,11	0,04	0	0	0	0,89	0,11	0,042
2,5	100000	0,04	0	0,95	0	0,01	0	0	0	0	0,03	0,95	0,01	0,01	0,652
2,5	35621	0	0,42	0	0,01	0,52	0	0,01	0,04	0	0,04	0,52	0,01	0,43	0,034
2,5	51335	0,06	0	0	0	0,54	0,4	0	0	0	0	0,94	0	0,06	0,048
2,5	41839	0	0	0,04	0,07	0,64	0,25	0	0	0	0,96	0,04	0	0	0,032
2,5	3405	0,06	0	0,03	0	0	0,13	0	0	0,78	0,06	0,16	0	0,78	0,044
2,5	48846	0	0,28	0,01	0	0,7	0	0	0	0,01	0,16	0,01	0,12	0,71	0,048
2,5	17549	0,06	0	0	0	0,1	0,84	0	0	0	0,82	0	0,06	0,12	0,048
2,5	80309	0	0	0,04	0	0	0	0	0	0,96	0	0,04	0	0,96	0,006
2,5	33058	0,02	0,11	0	0	0,85	0,02	0	0	0	0,04	0	0,85	0,11	0,048
2,5	26822	0	0,11	0	0	0,49	0	0,04	0,34	0,02	0,36	0	0,56	0,08	0,046
2,5	18709	0	0,02	0	0,16	0,8	0,02	0	0	0	0,8	0,18	0,02	0	0,048
2,5	27385	0	0	0	0,35	0,64	0,01	0	0	0	0,99	0	0,01	0	0,044
2,5	100000	0	0	1	0	0	0	0	0	0	0	1	0	0	0,782
2,5	2846	0	0	0	0,02	0,6	0,07	0,31	0	0	0,98	0,02	0	0	0,048
2,5	21033	0,04	0	0,04	0	0,91	0	0,01	0	0	0,05	0,93	0,01	0,01	0,048
2,5	13510	0	0,17	0	0,02	0,31	0,39	0	0,11	0	0,3	0,12	0,03	0,55	0,048
2,5	1923	0	0	0,04	0	0,76	0	0,06	0,14	0	0,08	0	0,1	0,82	0,046
2,5	11779	0,05	0	0	0,01	0,87	0,02	0	0,05	0	0,87	0,02	0,1	0,01	0,044
2,5	12581	0	0,1	0	0	0,87	0,01	0	0,02	0	0,59	0	0,2	0,21	0,048
2,5	28932	0	0	0	0,06	0,92	0,02	0	0	0	0,02	0,02	0,06	0,9	0,046
2,5	22370	0	0,79	0,04	0	0	0	0	0	0,17	0	0,04	0	0,96	0,028
2,5	71487	0	0	0	0,03	0,92	0,01	0,03	0	0,01	0,03	0,46	0,04	0,47	0,048

2,5	20601	0	0	0,04	0	0,96	0	0	0	0	0,04	0	0,96	0	0,028
2,5	88991	0,01	0,06	0,04	0	0,85	0	0	0,04	0	0,04	0,87	0,09	0	0,028
2,5	48950	0,06	0	0	0	0,29	0,65	0	0	0	0,35	0	0	0,65	0,048
2,5	66819	0	0,18	0	0	0,69	0	0,01	0,12	0	0,18	0,82	0	0	0,044
2,5	100000	0	0	0,99	0,01	0	0	0	0	0	0	0,99	0	0,01	0,618
2,5	35361	0	0	0,04	0	0,44	0	0,04	0	0,48	0,44	0,08	0	0,48	0,048
2,5	11565	0,04	0	0,02	0	0,92	0,01	0,01	0	0	0,04	0,95	0,01	0	0,044
2,5	15612	0	0,01	0,04	0	0	0,81	0,01	0,13	0	0,13	0,49	0,38	0	0,036
2,5	25373	0	0,04	0,04	0,01	0,9	0	0,01	0	0	0,01	0,4	0,59	0	0,042
2,5	99633	0	0,07	0	0	0,77	0,11	0	0,05	0	0,89	0,11	0	0	0,048
2,5	59948	0	0	0	0,02	0,39	0,12	0,05	0,23	0,19	0,02	0,24	0,16	0,58	0,046
2,5	21169	0,01	0	0,04	0	0	0,02	0	0,93	0	0,01	0,04	0,94	0,01	0,024
2,5	36934	0	0	0,04	0,07	0,53	0	0	0	0,36	0,07	0,55	0,02	0,36	0,04
2,5	7021	0,06	0	0	0	0,86	0,01	0	0	0,07	0,07	0	0,92	0,01	0,042
2,5	100000	0,01	0	0,99	0	0	0	0	0	0	0	1	0	0	0,732
2,5	57293	0	0,19	0	0	0,81	0	0	0	0	0,01	0,45	0,47	0,07	0,046
2,5	16099	0	0	0,04	0	0	0	0,01	0,95	0	0	0,01	0,59	0,4	0,02
2,5	23567	0	0,03	0,04	0	0,87	0	0	0	0,06	0,13	0,07	0,8	0	0,044
2,5	100000	0,01	0,02	0,97	0	0	0	0	0	0	0	0,97	0	0,03	0,538
2,5	49285	0,02	0	0	0,04	0,11	0	0	0,83	0	0,03	0	0,04	0,93	0,042
2,5	46373	0,04	0,01	0,04	0	0,91	0	0	0	0	0,05	0,83	0	0,12	0,04
2,5	61741	0	0	0	0,12	0	0,88	0	0	0	0	0,12	0,88	0	0,036
2,5	12614	0,03	0,06	0,04	0	0,01	0,84	0	0	0,02	0,11	0	0	0,89	0,046
2,5	31827	0	0	0,04	0	0,89	0	0,06	0,01	0	0,89	0	0,02	0,09	0,048
2,5	49484	0	0,03	0,04	0	0,77	0,14	0	0	0,02	0	0,21	0,77	0,02	0,034
2,5	45323	0	0,14	0,01	0	0,62	0,2	0,03	0	0	0,15	0,69	0	0,16	0,034
2,5	12148	0	0	0,04	0	0	0,02	0	0,89	0,05	0	0,04	0,89	0,07	0,022
2,5	22626	0	1	0	0	0	0	0	0	0	0	0	0,09	0,91	0,048
2,5	14251	0,04	0	0	0	0	0,23	0	0,73	0	0,04	0,73	0	0,23	0,04
2,5	6421	0	0	0,04	0	0,96	0	0	0	0	0,68	0,04	0	0,28	0,03
2,5	17374	0	0,91	0	0,03	0	0,03	0	0,03	0	0,03	0	0	0,97	0,048
2,5	63923	0,01	0,05	0,02	0	0,91	0	0,01	0	0	0,91	0,03	0,06	0	0,04
2,5	5390	0	0,05	0,04	0	0,72	0	0	0	0,19	0,71	0,18	0,05	0,06	0,044

2,5	43897	0	0,06	0,04	0	0,79	0,01	0	0,01	0,09	0	0,13	0,86	0,01	0,034
2,5	32653	0,04	0	0,02	0	0,93	0	0,01	0	0	0,93	0,06	0,01	0	0,048
2,5	15266	0	0	0,03	0,08	0	0,87	0,01	0,01	0	0,01	0	0,86	0,13	0,048
2,5	9104	0,05	0	0	0	0	0,23	0	0,72	0	0,07	0	0,03	0,9	0,04
2,5	34374	0,01	0,01	0	0	0,13	0	0,1	0,75	0	0,02	0,1	0,88	0	0,048
2,5	42689	0	0	0	0,17	0,46	0,37	0	0	0	0,37	0,46	0,17	0	0,038
2,5	32888	0,05	0,04	0	0	0,9	0,01	0	0	0	0	0,94	0,06	0	0,048
2,5	70345	0	0	0	0,22	0	0,78	0	0	0	0,78	0	0,22	0	0,048
2,5	18884	0	0	0,01	0,09	0,89	0	0,01	0	0	0,1	0,01	0,89	0	0,048
2,5	40222	0,06	0	0	0	0	0	0	0,94	0	0,08	0	0,32	0,6	0,048
2,5	67033	0	0,1	0	0	0	0,69	0,04	0,17	0	0,12	0	0,19	0,69	0,048
2,5	3199	0,01	0	0,04	0	0	0,01	0,01	0,55	0,38	0,01	0,06	0	0,93	0,028
2,5	41737	0	0	0,04	0	0	0,95	0	0,01	0	0	0,05	0	0,95	0,014
2,5	22075	0,03	0,04	0	0	0	0,93	0	0	0	0,03	0,04	0,93	0	0,046
2,5	37181	0	0	0,04	0	0	0	0	0,96	0	0	0	0,96	0,04	0,03
2,5	100000	0	0,12	0,87	0	0	0	0	0,01	0	0,37	0,5	0,13	0	0,776
2,5	33255	0	0,14	0	0	0,86	0	0	0	0	0,02	0,12	0,86	0	0,048
2,5	55791	0,03	0	0,04	0	0	0,82	0	0,11	0	0	0,08	0,89	0,03	0,048
2,5	16102	0	0,26	0,02	0,01	0,7	0	0,01	0	0	0	0,01	0,24	0,75	0,044
2,5	56050	0	0	0,04	0	0,22	0	0	0	0,74	0	0	0,23	0,77	0,006
2,5	50606	0,01	0	0	0	0,94	0	0,05	0	0	0,06	0	0	0,94	0,044
2,5	21549	0	0	0,04	0	0	0,91	0	0	0,05	0,9	0,05	0,05	0	0,038
2,5	54958	0	0,01	0	0,01	0,91	0	0,06	0	0,01	0	0,98	0,01	0,01	0,046
2,5	38333	0,01	0	0,03	0	0,89	0	0,07	0	0	0	0,03	0,91	0,06	0,044
2,5	56314	0	0	0	0,14	0,79	0	0	0,07	0	0,4	0,53	0	0,07	0,046
2,5	52458	0	0	0,04	0	0,19	0,14	0,01	0,62	0	0,14	0,21	0,65	0	0,034
2,5	9703	0	0,22	0	0	0,77	0,01	0	0	0	0	0,77	0,22	0,01	0,036
2,5	18797	0	0	0	0	0,88	0	0,11	0,01	0	0,12	0,05	0,83	0	0,048
2,5	21416	0	0,28	0	0	0	0	0	0,72	0	0,19	0	0,09	0,72	0,048
2,5	100000	0,01	0	0,96	0,01	0	0	0,01	0,01	0	0,01	0,96	0,02	0,01	0,588
2,5	28645	0	0	0,04	0	0,96	0	0	0	0	0	1	0	0	0,004
2,5	40390	0	0,02	0	0,17	0,79	0,02	0	0	0	0,79	0	0,2	0,01	0,042
2,5	43899	0,05	0	0	0,01	0,89	0,03	0,01	0,01	0	0,04	0,05	0,01	0,9	0,046

2,5	31755	0	0	0,04	0	0,8	0	0	0	0,16	0	0,06	0	0,94	0,004
2,5	7358	0	0,85	0	0,04	0	0	0	0,11	0	0,02	0,11	0	0,87	0,04
2,5	100000	0	0,04	0,93	0	0	0	0	0	0,03	0,03	0,94	0	0,03	0,566
2,5	24058	0	0	0,04	0	0,39	0,54	0	0	0,03	0,03	0,92	0,04	0,01	0,02
2,5	50736	0,02	0,02	0,04	0	0,57	0,05	0	0,3	0	0	0,07	0,34	0,59	0,03
2,5	18172	0	0	0	0,06	0,94	0	0	0	0	0	0,06	0	0,94	0,048
2,5	14754	0,05	0	0,03	0,01	0,82	0,09	0	0	0	0,04	0,62	0,01	0,33	0,048
2,5	35857	0	0,05	0	0	0,4	0	0,07	0	0,48	0,38	0,09	0,48	0,05	0,048
2,5	68218	0	0	0	0	0	0	0,06	0,94	0	0	1	0	0	0,048
2,5	68517	0	0,02	0,03	0,05	0,39	0,1	0	0,41	0	0,01	0,44	0,55	0	0,048
2,75	25112	0	0	0,04	0	0,46	0	0	0,49	0,01	0,01	0,53	0	0,46	0
2,75	27162	0	0	0,04	0	0	0	0	0	0,96	0	0,04	0	0,96	0,008
2,75	15493	0,03	0,03	0,01	0,01	0,92	0	0	0	0	0,01	0,07	0,89	0,03	0,046
2,75	4581	0	0	0,04	0	0	0	0	0	0,96	0,08	0,04	0	0,88	0,014
2,75	84578	0	0,04	0,04	0	0,01	0	0	0,26	0,65	0,04	0,01	0,69	0,26	0,03
2,75	37839	0	0	0,04	0	0,02	0,89	0	0,05	0	0	0,94	0,06	0	0,024
2,75	30972	0,05	0	0	0	0,95	0	0	0	0	0,05	0	0,95	0	0,048
2,75	30481	0	0,01	0	0,11	0,86	0,02	0	0	0	0,02	0	0,97	0,01	0,048
2,75	3131	0	0	0,04	0	0,89	0,02	0,02	0,01	0,02	0,04	0,08	0,86	0,02	0,032
2,75	27791	0	0,92	0,02	0,02	0,04	0	0	0	0	0,05	0	0,01	0,94	0,048
2,75	25818	0,02	0	0,04	0,02	0,4	0,38	0,01	0,13	0	0	0,5	0,09	0,41	0,046
2,75	29621	0,02	0	0	0,14	0,84	0	0	0	0	0,98	0	0	0,02	0,034
2,75	100000	0	0	0,96	0	0	0	0	0,04	0	0,13	0,84	0,02	0,01	0,638
2,75	50505	0,04	0	0,04	0,01	0	0,87	0	0,02	0,02	0,04	0,85	0,08	0,03	0,038
2,75	13735	0,05	0	0,04	0	0	0	0	0,41	0,5	0,07	0	0,52	0,41	0,046
2,75	15750	0	0	0,04	0,02	0,92	0	0	0	0,02	0,93	0	0,07	0	0,046
2,75	90340	0,02	0,01	0,04	0	0,93	0	0	0	0	0,91	0,01	0,05	0,03	0,04
2,75	42991	0	0	0,04	0,02	0,49	0,44	0	0,01	0	0,49	0,01	0,06	0,44	0,042
2,75	8797	0	0	0	0,05	0,79	0	0,02	0,13	0,01	0	0,07	0,02	0,91	0,048
2,75	30720	0	0,01	0,04	0	0,9	0	0,04	0	0,01	0,92	0	0,01	0,07	0,038
2,75	100000	0	0	1	0	0	0	0	0	0	0	1	0	0	0,626
2,75	13742	0	0	0,04	0	0,95	0	0	0	0,01	0,64	0,04	0,31	0,01	0,032
2,75	98586	0	0,14	0,02	0	0,84	0	0	0	0	0,16	0	0,82	0,02	0,04

2,75	55358	0	0	0,04	0	0,95	0	0,01	0	0	0,04	0	0,96	0	0,022
2,75	100000	0,01	0,08	0,57	0	0,01	0,08	0	0	0,25	0,05	0,66	0,08	0,21	0,266
2,75	28644	0	0,01	0,04	0	0,95	0	0	0	0	0	0,01	0,98	0,01	0,026
2,75	17745	0	0,9	0	0	0	0,06	0	0	0,04	0,04	0,11	0	0,85	0,046
2,75	39810	0,06	0	0	0	0,93	0	0	0	0,01	0	0,01	0,99	0	0,046
2,75	55812	0	0	0,04	0	0	0	0	0	0,96	0,02	0,94	0,04	0	0,022
2,75	20126	0	0	0,04	0	0	0	0	0,96	0	0	0,86	0	0,14	0,004
2,75	49331	0,03	0,01	0,04	0	0,54	0,38	0	0	0	0,01	0,57	0,42	0	0,048
2,75	76711	0	0	0	0,06	0,94	0	0	0	0	0,06	0	0	0,94	0,048
2,75	100000	0	0	0,78	0,02	0	0,05	0,13	0,01	0,01	0,05	0,83	0,11	0,01	0,496
2,75	100000	0	0,01	0,99	0	0	0	0	0	0	0,01	0,99	0	0	0,664
2,75	44379	0	0	0	0,05	0,95	0	0	0	0	0,05	0	0	0,95	0,04
2,75	19509	0	0,1	0	0	0,86	0	0	0,04	0	0,87	0	0	0,13	0,048
2,75	24554	0,03	0	0	0,03	0,91	0,02	0	0	0,01	0,01	0,04	0,04	0,91	0,048
2,75	10384	0,03	0,03	0	0,02	0,81	0	0,04	0,07	0	0,04	0,84	0,1	0,02	0,048
2,75	29479	0	0	0	0,09	0,88	0,01	0	0,02	0	0,05	0,8	0,13	0,02	0,046
2,75	57608	0,05	0	0,01	0	0,93	0	0,01	0	0	0,05	0,91	0,04	0	0,046
2,75	25428	0	0	0,04	0	0,93	0,01	0,02	0	0	0	0,06	0,01	0,93	0,004
2,75	20208	0	0,02	0,04	0	0,48	0	0	0	0,46	0	0,04	0,13	0,83	0,02
2,75	5758	0,01	0	0,01	0,1	0,81	0	0,03	0	0,04	0,74	0,07	0,15	0,04	0,048
2,75	87154	0	0,01	0	0,04	0,9	0	0,04	0	0,01	0,3	0	0,05	0,65	0,044
2,75	18179	0	0,01	0	0,08	0,89	0,01	0	0	0,01	0,08	0,89	0,01	0,02	0,048
2,75	1027	0	0	0,04	0,01	0,49	0	0,06	0,39	0,01	0,39	0,51	0,08	0,02	0,044
2,75	10507	0	0,2	0,04	0	0	0	0	0	0,76	0	0,24	0	0,76	0,048
2,75	3430	0	0,29	0,01	0	0,44	0,04	0	0,17	0,05	0	0,95	0,05	0	0,048
2,75	22807	0,03	0	0,04	0	0,93	0	0	0	0	0,33	0,67	0	0	0,048
2,75	39759	0	0	0,04	0	0,01	0,04	0,03	0,88	0	0,01	0,04	0,91	0,04	0,046
2,75	31059	0,03	0,16	0	0	0,05	0	0	0	0,76	0,16	0,04	0	0,8	0,048
2,75	33824	0	0,17	0	0	0,58	0	0	0,23	0,02	0	0,23	0,75	0,02	0,048
2,75	51907	0	0	0	0,24	0,2	0,21	0	0,25	0,1	0,75	0,25	0	0	0,044
2,75	100000	0	0	1	0	0	0	0	0	0	0	1	0	0	0,756
2,75	15626	0,04	0,02	0	0	0,26	0,68	0	0	0	0,64	0,06	0,3	0	0,044
2,75	7643	0	0,04	0,02	0,03	0,06	0	0,07	0,78	0	0,8	0,06	0,03	0,11	0,048

2,75	70675	0	0	0	0	0	0,77	0,15	0,05	0,03	0,46	0,35	0,15	0,04	0,046
2,75	3238	0	0	0,04	0	0	0	0,03	0,93	0	0	0,55	0,45	0	0,044
2,75	9354	0	0	0,01	0	0,07	0,3	0,19	0,43	0	0,78	0,01	0,02	0,19	0,046
2,75	94792	0	0,01	0,04	0,03	0	0,18	0,02	0	0,72	0,21	0,6	0,03	0,16	0,042
2,75	28976	0	0,2	0	0	0,73	0	0	0,07	0	0	0,79	0,21	0	0,04
2,75	100000	0,01	0	0,98	0	0	0	0,01	0	0	0	0,99	0,01	0	0,528
2,75	8751	0	0,2	0	0	0,8	0	0	0	0	0,44	0	0	0,56	0,048
2,75	73692	0	0	0	0,13	0,82	0,01	0,04	0	0	0,81	0	0	0,19	0,042
2,75	12268	0	0	0,04	0	0,02	0	0	0,01	0,93	0	0,06	0,02	0,92	0,016
2,75	8018	0	0	0,04	0	0	0	0,07	0	0,89	0	0,06	0,94	0	0,048
2,75	21444	0	0	0,04	0	0	0	0,06	0,88	0,02	0	0,06	0,92	0,02	0,042
2,75	12504	0	0,11	0	0	0,48	0,01	0,01	0,39	0	0,18	0,01	0,81	0	0,046
2,75	23898	0	0	0	0,05	0,95	0	0	0	0	0	0	0,05	0,95	0,036
2,75	20376	0	0,07	0	0	0,01	0,92	0	0	0	1	0	0	0	0,044
2,75	15803	0	0,01	0,04	0	0,01	0	0	0,94	0	0	0,05	0,3	0,65	0,018
2,75	9680	0	0,02	0	0,13	0,17	0,68	0	0	0	0,29	0,02	0,69	0	0,048
2,75	33924	0	0	0,04	0	0,83	0,13	0	0	0	0,01	0,95	0	0,04	0,006
2,75	100000	0	0	1	0	0	0	0	0	0	0	1	0	0	0,7
2,75	7177	0	0	0,04	0,04	0,89	0	0	0,01	0,02	0,89	0,05	0,02	0,04	0,044
2,75	30017	0,01	0,01	0	0,05	0	0	0	0,93	0	0	0	0	1	0,046
2,75	51889	0,03	0	0,04	0	0,89	0,04	0	0	0	0,04	0,93	0,03	0	0,044
2,75	30816	0	0	0,04	0	0,75	0	0	0,02	0,19	0,75	0,04	0,21	0	0,034
2,75	53793	0,01	0	0,04	0	0,03	0	0,02	0,04	0,86	0,07	0,04	0,87	0,02	0,032
2,75	100000	0	0	1	0	0	0	0	0	0	0	1	0	0	0,65
2,75	39713	0	0,06	0,01	0,07	0,85	0	0	0	0,01	0,64	0,01	0,32	0,03	0,048
2,75	25523	0,03	0,04	0	0	0,02	0,78	0,02	0,11	0	0,78	0,11	0,06	0,05	0,048
2,75	5767	0,05	0	0	0	0	0	0	0,75	0,2	0	0,21	0	0,79	0,04
2,75	12088	0,02	0	0,04	0	0,83	0,09	0	0,02	0	0,34	0,04	0,61	0,01	0,042
2,75	4333	0,06	0	0	0,01	0,9	0	0	0,03	0	0,09	0,91	0	0	0,044
2,75	21981	0	0,1	0	0,03	0,54	0,33	0	0	0	0,36	0,07	0,06	0,51	0,044
2,75	5424	0	0,21	0	0	0,77	0,01	0	0,01	0	0	0,77	0,22	0,01	0,048
2,75	92015	0	0	0,04	0,06	0	0	0	0,84	0,06	0	0,88	0	0,12	0,036
2,75	43519	0,01	0	0,04	0,04	0,73	0	0	0	0,18	0,01	0,14	0,18	0,67	0,038

2,75	25714	0	0,02	0,04	0	0	0,67	0	0,27	0	0,69	0,25	0,06	0	0,044
2,75	52782	0	0	0,04	0	0	0,43	0	0,08	0,45	0	0,47	0,25	0,28	0,018
2,75	82424	0,03	0	0,03	0,07	0,87	0	0	0	0	0,89	0,03	0,04	0,04	0,048
2,75	3775	0	0	0,04	0	0	0	0,03	0,93	0	0	0,01	0	0,99	0,036
2,75	75913	0,03	0,14	0	0	0,07	0,61	0	0,15	0	0	0,14	0,2	0,66	0,048
2,75	100000	0	0	1	0	0	0	0	0	0	0	1	0	0	0,592
2,75	21879	0,06	0	0,03	0	0,85	0,05	0	0,01	0	0,08	0,06	0,01	0,85	0,048
2,75	85668	0	0,12	0,01	0	0,86	0,01	0	0	0	0,09	0,13	0,78	0	0,044
2,75	63727	0	0,01	0	0,12	0,56	0,31	0	0	0	0,32	0	0,67	0,01	0,048
2,75	4727	0	0,01	0,02	0,08	0	0,06	0	0	0,83	0,09	0,08	0	0,83	0,048
2,75	81016	0,02	0	0,04	0	0,46	0	0	0	0,48	0,02	0,5	0	0,48	0,034
3	44261	0	0	0	0,14	0,86	0	0	0	0	0,77	0,03	0,11	0,09	0,036
3	6700	0,04	0	0,04	0	0,87	0	0	0,05	0	0	0,04	0,72	0,24	0,046
3	39407	0,04	0,05	0	0	0	0,91	0	0	0	0	0,91	0,09	0	0,048
3	75401	0,05	0	0	0	0	0	0	0,95	0	0	0,01	0	0,99	0,036
3	15148	0	0	0,04	0	0	0,96	0	0	0	0,04	0	0	0,96	0,004
3	38444	0	0	0,04	0	0,94	0	0,02	0	0	0,1	0,04	0,02	0,84	0,028
3	8791	0	0	0,04	0	0	0	0	0,37	0,59	0,02	0,46	0	0,52	0,01
3	14214	0	0	0	0,11	0,01	0	0	0,88	0	0,02	0,87	0,11	0	0,048
3	86247	0	0,15	0	0	0,81	0,04	0	0	0	0	0,25	0,75	0	0,046
3	17556	0	0,01	0	0,14	0,85	0	0	0	0	0,85	0	0,15	0	0,036
3	45535	0	0	0,04	0,01	0,85	0	0	0,1	0	0,04	0,15	0,02	0,79	0,012
3	82770	0,06	0	0	0	0,63	0,29	0	0,02	0	0,07	0,01	0,86	0,06	0,04
3	17590	0	0	0,01	0,02	0,91	0	0,03	0,03	0	0,04	0	0,02	0,94	0,044
3	89018	0,02	0	0	0,01	0,81	0	0,07	0,09	0	0	0,02	0,98	0	0,036
3	100000	0	0,27	0	0,06	0,01	0,25	0	0,26	0,15	0,32	0,06	0,62	0	0,172
3	4181	0,03	0	0,04	0	0,43	0	0	0	0,5	0,01	0,04	0,1	0,85	0,034
3	66480	0	0	0	0	0	0	0,13	0,37	0,5	0,5	0	0	0,5	0,048
3	45783	0	0,03	0	0,11	0	0,47	0	0,39	0	0,47	0,14	0,39	0	0,048
3	63738	0	0	0,04	0	0,88	0	0	0	0,08	0	0,04	0,96	0	0,018
3	35037	0	0	0,04	0,01	0	0,94	0,01	0	0	0,37	0	0,58	0,05	0,038
3	48937	0	0,01	0	0,1	0,57	0,32	0	0	0	0,49	0,01	0	0,5	0,048
3	79483	0	0,07	0,01	0	0	0	0	0,92	0	0,92	0,01	0,07	0	0,044

3	8322	0	0,1	0	0	0,88	0	0,01	0,01	0	0,11	0,01	0,88	0	0,038
3	19263	0,06	0	0	0,02	0,89	0,03	0	0	0	0,91	0	0,09	0	0,044
3	13921	0	0,01	0,04	0	0	0,13	0,04	0,78	0	0,69	0,05	0,26	0	0,046
3	71371	0	0	0,04	0	0	0	0	0,95	0,01	0	0,26	0,73	0,01	0,022
3	52253	0,04	0	0,03	0	0	0	0	0,04	0,89	0,91	0,06	0	0,03	0,048
3	32496	0	0,98	0	0	0,01	0	0,01	0	0	0,05	0	0,02	0,93	0,042
3	1986	0	0	0,04	0	0,88	0,01	0	0	0,07	0,08	0,04	0,88	0	0,018
3	87014	0	0,83	0,02	0,02	0,04	0	0	0,08	0,01	0,02	0,05	0,03	0,9	0,042
3	71201	0	0	0,04	0	0,02	0	0	0,82	0,12	0,02	0,01	0,82	0,15	0,028
3	53679	0,04	0	0,02	0	0,82	0,03	0	0,09	0	0,13	0,84	0	0,03	0,04
3	87063	0	0,08	0	0	0,88	0	0	0,01	0,03	0,88	0,01	0,09	0,02	0,04
3	11971	0	0	0	0,1	0,31	0	0	0	0,59	0	0,31	0,1	0,59	0,048
3	31424	0,03	0,05	0,01	0	0,89	0	0,02	0	0	0,05	0,01	0,01	0,93	0,04
3	91687	0	0,03	0	0	0	0,85	0,09	0	0,03	0,03	0	0,88	0,09	0,048
3	100000	0	0,01	0,96	0	0	0,03	0	0	0	0	0,96	0,03	0,01	0,492
3	49738	0	0,1	0,04	0	0,32	0	0	0	0,54	0	0,04	0,54	0,42	0,03
3	100000	0,11	0	0,71	0	0,12	0,05	0	0,01	0	0,02	0,71	0	0,27	0,378
3	25209	0,04	0	0	0	0,91	0	0	0	0,05	1	0	0	0	0,044
3	46291	0	0,26	0	0	0,74	0	0	0	0	0	0,74	0,26	0	0,048
3	79649	0	0	0	0,13	0,19	0	0	0,68	0	0,13	0,19	0,68	0	0,044
3	35703	0	0	0	0,07	0,93	0	0	0	0	0,06	0,93	0,01	0	0,042
3	7724	0	0	0,04	0	0,93	0	0	0	0,03	0,55	0,04	0,41	0	0,026
3	11503	0,02	0	0,04	0	0,79	0	0,03	0,12	0	0	0,02	0,08	0,9	0,046
3	12997	0,06	0	0	0	0,16	0	0	0,78	0	0,06	0	0,16	0,78	0,044
3	10589	0	0	0,04	0,01	0	0,1	0,01	0,68	0,16	0,06	0,04	0,84	0,06	0,034
3	42516	0	0	0,03	0,14	0	0	0	0,52	0,31	0,65	0,03	0,01	0,31	0,048
3	43176	0,05	0	0	0,03	0,17	0,75	0	0	0	0,05	0,82	0,03	0,1	0,048
3	34914	0	0	0	0,24	0,76	0	0	0	0	0,76	0,24	0	0	0,046
3	45209	0,01	0	0,04	0	0	0,58	0	0	0,37	0,01	0,04	0,95	0	0,03
3	49198	0,04	0	0	0	0,15	0	0,1	0,71	0	0,71	0,25	0,04	0	0,042
3	22492	0,07	0	0	0	0,93	0	0	0	0	1	0	0	0	0,046
3	23462	0	0,97	0	0	0	0	0	0,01	0,02	0,01	0,98	0	0,01	0,046
3	10361	0	0	0,04	0	0,04	0,46	0,08	0	0,38	0	0,27	0,39	0,34	0,046

3	88811	0	0,16	0,04	0	0,28	0	0	0	0,52	0	0,04	0,28	0,68	0,036
3	49394	0	0	0,04	0	0	0,06	0,02	0,88	0	0,94	0,04	0,02	0	0,038
3	100000	0	0,2	0,77	0,02	0	0,01	0	0	0	0	0,79	0,21	0	0,454
3	35771	0	0	0,02	0,08	0,82	0,02	0,01	0	0,05	0,02	0,58	0,36	0,04	0,044
3	14540	0	0,04	0	0,11	0,78	0	0	0,01	0,06	0	0,05	0,82	0,13	0,044
3	78540	0	0,26	0,01	0,01	0,67	0	0	0,05	0	0,05	0,01	0,27	0,67	0,048
3	29270	0	0,15	0,01	0	0,6	0	0	0,24	0	0,24	0,69	0,07	0	0,042
3	9512	0,01	0	0,04	0,01	0,93	0	0	0,01	0	0	0,05	0,88	0,07	0,046
3	5379	0	0	0,04	0	0,93	0	0,03	0	0	0	0,07	0,93	0	0,048
3	13161	0,05	0	0,04	0	0	0,14	0	0	0,77	0,02	0,05	0,03	0,9	0,048
3	44344	0,02	0,04	0	0	0,78	0,16	0	0	0	0,8	0,04	0,16	0	0,048
3	100000	0	0	1	0	0	0	0	0	0	0	1	0	0	0,65
3	42158	0,02	0,05	0	0,03	0,03	0,75	0	0,12	0	0,29	0,56	0,1	0,05	0,046
3	66910	0	0,08	0,01	0	0	0,91	0	0	0	0,81	0,08	0,11	0	0,046
3	26474	0	0	0,01	0,08	0,7	0	0	0	0,21	0	0,71	0	0,29	0,044
3	30035	0	0,11	0	0	0	0,85	0,04	0	0	0,04	0,96	0	0	0,048
3	98251	0	0	0,04	0,01	0,77	0,02	0	0,16	0	0,02	0	0,17	0,81	0,018
3	21401	0	0	0	0,13	0	0	0	0,87	0	0,12	0,25	0,63	0	0,046
3	5074	0	0	0,01	0,03	0,91	0,01	0,02	0,02	0	0,03	0,02	0,04	0,91	0,044
3	43755	0,05	0	0	0	0,93	0,02	0	0	0	0,04	0,93	0,03	0	0,044
3	42523	0	0	0	0,08	0,92	0	0	0	0	0	0,92	0,08	0	0,048
3	21871	0	0,04	0,04	0	0,73	0,06	0	0,13	0	0,77	0,08	0,12	0,03	0,046
3	28534	0	0	0	0,13	0	0	0	0,87	0	0,05	0	0,82	0,13	0,048
3	62774	0,03	0	0,04	0	0,9	0	0,01	0	0,02	0,93	0,04	0,03	0	0,048
3	32574	0,04	0	0,03	0	0,73	0,02	0	0	0,18	0,91	0,03	0,04	0,02	0,042
3	87127	0,01	0	0,04	0	0,95	0	0	0	0	0	0,99	0,01	0	0,014
3	19256	0	0	0,04	0	0,75	0,15	0	0,01	0,05	0	0,18	0,78	0,04	0,032
3	10923	0	0	0,04	0	0,9	0,01	0,02	0,03	0	0,04	0,89	0,02	0,05	0,016
3	77464	0,04	0	0,04	0	0,01	0,03	0	0,88	0	0,03	0,04	0,04	0,89	0,038
3	24019	0	0,02	0,04	0	0,76	0,06	0,1	0	0,02	0,09	0	0,06	0,85	0,046
3	17872	0,06	0	0	0	0,93	0	0	0,01	0	0,92	0	0,07	0,01	0,038
3	36870	0,01	0	0,01	0,1	0,86	0	0	0,02	0	0,01	0	0,99	0	0,044
3	94686	0	0	0,04	0	0,05	0	0	0	0,91	0	0,04	0,14	0,82	0,016

3	8714	0	0,29	0	0	0,64	0,07	0	0	0	0	0,07	0,31	0,62	0,042
3	37680	0	0,03	0,01	0	0,01	0,46	0,07	0,42	0	0,03	0,34	0,3	0,33	0,042
3	5395	0	0	0,04	0	0	0	0,02	0	0,94	0,02	0,04	0	0,94	0,022
3	19447	0	0	0	0,23	0	0	0	0,77	0	0,77	0,13	0	0,1	0,046
3	7654	0	0,48	0,03	0	0,35	0	0	0,14	0	0,14	0,03	0	0,83	0,048
3	100000	0,01	0	0,99	0	0	0	0	0	0	0	0,99	0,01	0	0,644
3	100000	0	0	0,98	0	0	0,02	0	0	0	0	0,98	0	0,02	0,562
3	16691	0,07	0	0	0	0,1	0,83	0	0	0	0	0	0,83	0,17	0,048
3	46838	0	0,01	0	0,28	0,68	0	0	0,03	0	0,98	0,02	0	0	0,046
3	27719	0	0	0	0,12	0,64	0,01	0	0,23	0	0,05	0,18	0,77	0	0,048
3	17478	0	0	0	0,08	0,89	0	0	0,03	0	0,11	0,89	0	0	0,046
3	10356	0	0,16	0	0	0,46	0,37	0	0,01	0	0	0,47	0,53	0	0,048
3,25	57526	0,01	0,98	0	0	0	0	0	0,01	0	0,02	0,56	0	0,42	0,03
3,25	81984	0,05	0	0,04	0	0,6	0	0	0,31	0	0,28	0,67	0,02	0,03	0,046
3,25	14277	0	0	0,04	0	0	0,95	0	0,01	0	0	0,03	0,01	0,96	0,006
3,25	3411	0,01	0	0	0	0,94	0	0,05	0	0	0,01	0,94	0	0,05	0,044
3,25	42807	0	0,21	0	0,05	0,01	0	0	0,73	0	0,03	0,03	0	0,94	0,048
3,25	7480	0	0	0,04	0	0,01	0	0,04	0,3	0,61	0,3	0,09	0,61	0	0,032
3,25	9363	0,02	0,21	0	0	0,76	0	0	0,01	0	0,21	0,01	0,01	0,77	0,044
3,25	33160	0	0	0,01	0,17	0,82	0	0	0	0	0,82	0,01	0	0,17	0,048
3,25	37782	0	0,37	0,04	0,03	0,03	0	0	0,53	0	0	0,17	0,03	0,8	0,042
3,25	19267	0,06	0	0	0	0	0	0,01	0,93	0	0,06	0	0	0,94	0,046
3,25	24859	0,04	0,05	0	0	0	0	0	0,02	0,89	0	0,95	0,05	0	0,048
3,25	14352	0,03	0	0,01	0,03	0,93	0	0	0	0	0	0,93	0,04	0,03	0,046
3,25	14547	0,05	0,01	0	0	0,91	0	0	0	0,03	0,97	0,03	0	0	0,046
3,25	18282	0,05	0	0	0	0,71	0	0	0,24	0	0,05	0,71	0,24	0	0,046
3,25	36760	0,02	0,01	0,01	0,04	0	0	0	0,92	0	0,06	0,08	0,7	0,16	0,048
3,25	17446	0,02	0	0,04	0	0,94	0	0	0	0	0,95	0,04	0,01	0	0,036
3,25	13332	0,02	0	0,04	0	0,63	0	0	0	0,31	0	0,66	0,31	0,03	0,028
3,25	92046	0,01	0	0,04	0,01	0,92	0	0,01	0,01	0	0,98	0	0	0,02	0,046
3,25	59914	0	0,05	0,03	0	0,92	0	0	0	0	0,92	0,03	0,02	0,03	0,04
3,25	28521	0,02	0	0,01	0	0,86	0	0,04	0,07	0	0	0,11	0,86	0,03	0,038
3,25	65229	0	0,22	0	0,01	0,73	0,03	0	0	0,01	0,25	0,01	0	0,74	0,04

3,25	11065	0,01	0	0	0,07	0,86	0,04	0,02	0	0	0	0	0,88	0,12	0,048
3,25	24558	0	0,51	0	0	0	0	0,02	0,01	0,46	0	0,02	0,01	0,97	0,046
3,25	13880	0	0	0,04	0	0	0,87	0	0,09	0	0,29	0,11	0,6	0	0,04
3,25	9447	0	0	0,04	0	0	0,39	0	0,53	0,04	0,61	0,04	0	0,35	0,024
3,25	67600	0	0	0	0,23	0,77	0	0	0	0	0,77	0,23	0	0	0,048
3,25	81526	0,01	0,24	0	0	0,7	0,05	0	0	0	0,03	0,02	0,25	0,7	0,048
3,25	4908	0	0	0	0,13	0	0	0,01	0,74	0,12	0,01	0,11	0,74	0,14	0,046
3,25	42981	0	0,07	0	0,05	0,88	0	0	0	0	0,88	0	0,02	0,1	0,044
3,25	3323	0	0	0,04	0	0	0,1	0	0	0,86	0	0,04	0	0,96	0,006
3,25	16008	0	0,97	0	0	0	0,03	0	0	0	0	0,97	0	0,03	0,042
3,25	18813	0	0,07	0	0	0,93	0	0	0	0	0,91	0	0,07	0,02	0,046
3,25	97906	0	0	0,04	0	0,96	0	0	0	0	0	0,01	0,96	0,03	0,02
3,25	59407	0	0,04	0,02	0,06	0	0,2	0,01	0	0,67	0,06	0,06	0,43	0,45	0,048
3,25	82954	0	0,06	0	0,06	0	0,86	0	0,02	0	0,11	0	0	0,89	0,046
3,25	19407	0	0,21	0	0	0,7	0	0	0,09	0	0,17	0,7	0,09	0,04	0,044
3,25	100000	0	0	0,29	0	0	0	0	0	0,71	0,07	0,27	0,02	0,64	0,066
3,25	23203	0	0	0,04	0,01	0,09	0,79	0	0	0,07	0	0,08	0,92	0	0,028
3,25	26211	0,05	0	0,02	0	0,86	0	0	0,07	0	0,12	0,88	0	0	0,048
3,25	52896	0,01	0,96	0	0,03	0	0	0	0	0	0	0,03	0,01	0,96	0,048
3,25	76419	0	0,07	0	0,05	0,74	0	0	0,13	0,01	0,09	0,18	0,73	0	0,044
3,25	43018	0	0	0,03	0,07	0,02	0,08	0	0,8	0	0,03	0	0,12	0,85	0,048
3,25	44714	0,02	0	0,01	0,04	0,93	0	0	0	0	0,01	0,8	0	0,19	0,048
3,25	14323	0,01	0,07	0,03	0	0,85	0,01	0,01	0,02	0	0,04	0,03	0,92	0,01	0,044
3,25	79949	0	0,92	0	0	0	0	0	0	0,08	0	0	0,09	0,91	0,044
3,25	16533	0	0	0,04	0,02	0,94	0	0	0	0	0	0,92	0,08	0	0,022
3,25	40662	0,05	0	0	0	0,9	0,04	0	0	0,01	0,01	0,04	0,95	0	0,044
3,25	48397	0	0,04	0	0,09	0,87	0	0	0	0	0,04	0,08	0,69	0,19	0,042
3,25	35294	0	0,13	0	0	0,87	0	0	0	0	0	0	0,99	0,01	0,042
3,25	96547	0	0	0	0,06	0,89	0	0	0,05	0	0,06	0,05	0	0,89	0,046
3,25	43378	0	0,01	0,04	0	0	0,14	0	0	0,81	0	0,14	0,82	0,04	0,028
3,25	51285	0,03	0	0	0	0,66	0,26	0,05	0	0	0,66	0	0,05	0,29	0,044
3,25	22843	0,05	0,01	0,01	0	0,74	0,16	0	0,03	0	0,22	0,01	0,03	0,74	0,048
3,25	66403	0	0	0	0,12	0,88	0	0	0	0	0,12	0	0,88	0	0,044

3,25	100000	0	0	0,99	0	0	0	0	0,01	0	0	0,99	0,01	0	0,656
3,25	10217	0	0,24	0	0	0,74	0	0,01	0	0,01	0,25	0	0	0,75	0,048
3,25	39063	0	0	0	0,12	0,37	0	0	0,49	0,02	0,41	0,46	0,02	0,11	0,048
3,25	24189	0,06	0	0	0	0	0,92	0,01	0,01	0	0,98	0	0,01	0,01	0,044
3,25	6306	0,01	0,12	0	0	0,83	0	0	0,04	0	0,14	0	0,84	0,02	0,048
3,25	74839	0	0,07	0	0,04	0,68	0,16	0	0	0,05	0,85	0,06	0	0,09	0,048
3,25	37404	0	0	0	0,06	0,07	0,01	0	0,86	0	0,01	0,13	0	0,86	0,048
3,25	38541	0	0,01	0	0,17	0,76	0,06	0	0	0	0,77	0,18	0	0,05	0,044
3,25	46109	0	0	0,01	0,15	0,84	0	0	0	0	0,89	0,01	0,02	0,08	0,048
3,25	22743	0,01	0	0,04	0	0,82	0,13	0	0	0	0	0,04	0,14	0,82	0,02
3,25	30082	0	0	0,04	0,02	0	0	0	0,94	0	0,21	0	0,51	0,28	0,04
3,25	28124	0	0,95	0	0,05	0	0	0	0	0	0	0,01	0	0,99	0,048
3,25	57457	0	0	0,04	0,05	0,02	0,89	0	0	0	0	0	0,89	0,11	0,042
3,25	67778	0	0,07	0	0	0,93	0	0	0	0	0,95	0	0,05	0	0,036
3,25	43740	0,02	0	0,04	0,01	0,25	0,17	0	0	0,51	0,02	0,06	0,28	0,64	0,036
3,25	20468	0,01	0,84	0,03	0,01	0,01	0,1	0	0	0	0,04	0,01	0	0,95	0,048
3,25	13142	0,06	0	0	0	0	0,67	0	0,24	0,03	0	0	0,76	0,24	0,048
3,25	44033	0	0,84	0	0	0	0	0,03	0,13	0	0,03	0,13	0,01	0,83	0,048
3,25	67184	0	0	0	0,12	0,79	0	0	0,09	0	0	0,01	0,99	0	0,046
3,25	4943	0	0	0	0	0,81	0	0,13	0	0,06	0,06	0,05	0,89	0	0,046
3,25	44935	0	0,14	0,04	0	0,2	0,21	0	0,41	0	0	0,51	0,41	0,08	0,046
3,25	11068	0,01	0	0	0,05	0,91	0,01	0	0,02	0	0,07	0,91	0	0,02	0,042
3,25	33412	0	0,04	0,01	0,06	0,7	0,17	0,02	0	0	0	0,59	0,35	0,06	0,044
3,25	81106	0,05	0	0	0,02	0	0,92	0,01	0	0	0,95	0	0,05	0	0,048
3,25	36491	0,01	0	0,04	0,04	0	0	0	0,06	0,85	0,01	0,13	0,85	0,01	0,048
3,25	14728	0,02	0	0,01	0,06	0,87	0,04	0	0	0	0,03	0,1	0,87	0	0,044
3,25	45496	0,01	0	0,04	0	0	0	0,06	0,89	0	0,03	0,87	0,1	0	0,048
3,25	89352	0,01	0	0	0,08	0,01	0,88	0	0	0,02	0,05	0	0,87	0,08	0,046
3,25	19650	0	0,01	0,04	0,03	0	0,28	0,02	0,62	0	0,01	0,01	0,15	0,83	0,044
3,25	75626	0	0,01	0,04	0	0	0	0	0,95	0	0,95	0,01	0	0,04	0,046
3,25	64806	0,01	0	0	0,14	0	0,08	0,01	0	0,76	0,76	0,08	0,01	0,15	0,048
3,25	16468	0,04	0	0,01	0,01	0,94	0	0	0	0	0,18	0	0,8	0,02	0,046
3,25	100000	0,52	0	0,47	0	0	0,01	0	0	0	0,52	0,47	0	0,01	0,962

3,25	100000	0	0	0,97	0,02	0	0	0	0,01	0	0	0,99	0,01	0	0,504
3,25	100000	0	0	0,96	0,02	0,02	0	0	0	0	0,01	0,98	0	0,01	0,606
3,25	69098	0	0,22	0,01	0	0,74	0	0	0	0,03	0,24	0	0,03	0,73	0,042
3,25	54532	0	0	0	0,11	0,86	0,02	0,01	0	0	0,11	0,02	0,87	0	0,046
3,25	13092	0,03	0	0,04	0,01	0,18	0,74	0	0	0	0,21	0,01	0,72	0,06	0,034
3,25	100000	0	0,02	0,95	0	0	0	0,01	0	0,02	0	0,97	0,03	0	0,53
3,25	10359	0	0,04	0,01	0	0,8	0,01	0,09	0	0,05	0,84	0,01	0,1	0,05	0,048
3,25	43464	0	0,11	0,03	0	0,84	0,01	0,01	0	0	0	0,03	0,96	0,01	0,044
3,25	35076	0,02	0	0,04	0,02	0,89	0,02	0,01	0	0	0,02	0	0,96	0,02	0,042
3,25	14992	0,02	0	0,04	0	0	0	0	0,94	0	0,02	0	0,03	0,95	0,022
3,25	12544	0	0,26	0	0	0	0,69	0	0,02	0,03	0,01	0,68	0,02	0,29	0,048
3,25	42644	0,05	0	0,04	0	0,91	0	0	0	0	0,15	0	0	0,85	0,048
3,25	60240	0	0	0,01	0,06	0,88	0,02	0,03	0	0	0,01	0	0,97	0,02	0,046
3,5	25681	0,01	0	0,04	0	0,29	0,01	0,08	0	0,57	0,65	0,14	0,2	0,01	0,048
3,5	43222	0	0	0,04	0,01	0,9	0,05	0	0	0	0	0	0,94	0,06	0,038
3,5	21217	0	0,16	0	0	0,83	0	0	0	0,01	0,17	0,66	0,17	0	0,044
3,5	62206	0	0,31	0,01	0	0,68	0	0	0	0	0,02	0,01	0,29	0,68	0,046
3,5	37360	0	0,11	0	0,04	0	0,85	0	0	0	0	0,11	0,03	0,86	0,046
3,5	33105	0	0	0,04	0	0,9	0,06	0	0	0	0,06	0	0,9	0,04	0,02
3,5	9448	0	0	0,04	0	0,72	0,03	0	0,12	0,09	0	0,75	0,22	0,03	0,014
3,5	26778	0	0,01	0,04	0	0	0	0,05	0	0,9	0,05	0,05	0	0,9	0,044
3,5	71823	0	0,05	0,04	0	0	0	0	0,88	0,03	0	0,21	0	0,79	0,008
3,5	13822	0	0,04	0	0,08	0,79	0,09	0	0	0	0,83	0,03	0,02	0,12	0,04
3,5	13032	0,05	0	0,04	0	0,12	0	0	0,79	0	0,22	0,03	0,18	0,57	0,048
3,5	63492	0	0,13	0	0,01	0,86	0	0	0	0	0,01	0	0,99	0	0,048
3,5	9681	0	0,98	0	0	0	0	0	0,01	0,01	0	0,98	0,01	0,01	0,04
3,5	9615	0,03	0	0	0,02	0,94	0	0,01	0	0	0,01	0,03	0,01	0,95	0,046
3,5	67055	0	0	0,04	0	0,93	0	0	0,03	0	0,03	0,04	0	0,93	0
3,5	6441	0	0	0,04	0	0	0	0,01	0	0,95	0	0,04	0	0,96	0,014
3,5	54293	0	0	0	0,14	0,86	0	0	0	0	0,62	0,38	0	0	0,048
3,5	72696	0	0	0,04	0	0	0,05	0	0,89	0,02	0	0,89	0,05	0,06	0,01
3,5	2026	0	0	0,04	0	0	0	0	0,95	0,01	0,01	0,05	0	0,94	0
3,5	50145	0	0,12	0,02	0	0,79	0	0,04	0,03	0	0,15	0,61	0,04	0,2	0,048

3,5	9887	0,03	0	0,04	0	0,88	0	0,03	0	0,02	0,03	0,92	0,05	0	0,048
3,5	5463	0,01	0,11	0,04	0,01	0	0	0	0,56	0,27	0,01	0,04	0	0,95	0,04
3,5	16452	0	0,08	0,02	0	0,86	0	0,04	0	0	0	0,02	0,28	0,7	0,048
3,5	100000	0,46	0	0,45	0	0,02	0	0,07	0	0	0,46	0,5	0,04	0	0,872
3,5	25172	0,05	0	0	0	0,95	0	0	0	0	0,05	0	0	0,95	0,048
3,5	79328	0	0	0	0,21	0,79	0	0	0	0	0,79	0	0,21	0	0,048
3,5	10654	0,02	0	0,04	0,01	0	0	0	0,93	0	0	0,95	0,04	0,01	0,036
3,5	98822	0	0,23	0	0	0,49	0	0	0,27	0,01	0,01	0	0,45	0,54	0,046
3,5	75269	0	0,28	0	0	0,72	0	0	0	0	0	0	0,28	0,72	0,048
3,5	6554	0	0,04	0,04	0	0,85	0,07	0	0	0	0	0	0,08	0,92	0,01
3,5	100000	0,8	0	0,02	0	0	0,18	0	0	0	0,79	0,08	0,13	0	0,818
3,5	76997	0	0	0,04	0	0,67	0	0	0,28	0,01	0,2	0,09	0,51	0,2	0,022
3,5	38040	0	0,03	0,04	0	0,39	0,1	0	0,1	0,34	0,25	0,34	0	0,41	0,03
3,5	100000	0,17	0,04	0,68	0	0	0	0	0	0,11	0,11	0,85	0	0,04	0,59
3,5	100000	0	0	1	0	0	0	0	0	0	0	1	0	0	0,646
3,5	21791	0	0	0,04	0	0	0	0	0,87	0,09	0	0,04	0,87	0,09	0,026
3,5	40201	0	0,26	0	0,01	0,72	0,01	0	0	0	0	0	0,31	0,69	0,048
3,5	6570	0	0	0,04	0	0,73	0	0,01	0,22	0	0	0,78	0,22	0	0,03
3,5	30299	0	0	0,04	0	0	0,87	0	0	0,09	0,66	0,21	0,09	0,04	0,03
3,5	20882	0,01	0,34	0	0	0,61	0	0,03	0	0,01	0	0,63	0,03	0,34	0,042
3,5	15515	0	0,01	0,04	0	0,94	0	0,01	0	0	0	0,02	0,04	0,94	0,008
3,5	24106	0	0	0,04	0	0	0,49	0	0,46	0,01	0,46	0,5	0	0,04	0,024
3,5	74588	0	0,01	0	0,06	0,92	0,01	0	0	0	0,06	0,01	0	0,93	0,044
3,5	2626	0,04	0	0,04	0	0,91	0,01	0	0	0	0	0	0,96	0,04	0,034
3,5	41985	0	0	0,01	0,07	0,89	0,02	0,01	0	0	0,01	0,89	0,08	0,02	0,04
3,5	25173	0	0,25	0	0,03	0,72	0	0	0	0	0,25	0,01	0	0,74	0,042
3,5	16489	0,04	0	0,04	0	0	0,15	0	0	0,77	0,06	0,17	0	0,77	0,046
3,5	9149	0,02	0	0,03	0	0,91	0	0,04	0	0	0,03	0,03	0,91	0,03	0,04
3,5	98142	0	0,87	0	0	0,02	0	0,01	0,01	0,09	0,02	0,37	0	0,61	0,046
3,5	27919	0	0	0,01	0	0,14	0,61	0,2	0,04	0	0,76	0	0,19	0,05	0,046
3,5	8957	0	0	0,01	0	0,88	0,04	0,07	0	0	0	0,05	0,06	0,89	0,046
3,5	17135	0,01	0	0,01	0,06	0,9	0,02	0	0	0	0,03	0,01	0,12	0,84	0,042
3,5	7459	0	0	0,04	0	0,71	0	0	0,12	0,13	0,24	0,05	0,71	0	0,028

3,5	4505	0	0	0,04	0	0,14	0,41	0,01	0,34	0,06	0,88	0,04	0,01	0,07	0,044
3,5	12055	0	0	0,04	0,01	0,06	0	0	0,89	0	0	0,04	0	0,96	0,012
3,5	82173	0	0,04	0,03	0,04	0,89	0	0	0	0	0,93	0,03	0,04	0	0,04
3,5	31429	0,04	0,01	0,01	0,03	0	0,16	0	0,75	0	0	0,05	0,79	0,16	0,044
3,5	34994	0	0,96	0	0,04	0	0	0	0	0	0	1	0	0	0,046
3,5	100000	0,17	0	0,15	0	0	0,15	0	0,47	0,06	0	0,3	0,64	0,06	0,224
3,5	31332	0	0,15	0	0,02	0,83	0	0	0	0	0,15	0,83	0,02	0	0,048
3,5	20816	0	0,4	0	0,01	0,23	0,01	0	0,34	0,01	0,01	0,01	0,23	0,75	0,046
3,5	12081	0,05	0	0	0,05	0,65	0,14	0	0,11	0	0,7	0,25	0,05	0	0,044
3,5	11207	0,05	0,04	0	0	0,21	0	0	0	0,7	0	0,09	0,27	0,64	0,046
3,5	19491	0	0	0,04	0,02	0,94	0	0	0	0	0,04	0	0,94	0,02	0,032
3,5	50969	0	0,12	0	0	0,4	0,48	0	0	0	0,52	0	0,48	0	0,048
3,5	3355	0	0	0,04	0	0	0	0	0,96	0	0	0,04	0	0,96	0,002
3,5	18238	0	0	0,02	0,15	0,78	0	0	0,05	0	0,82	0	0,17	0,01	0,04
3,5	24683	0	0,07	0	0,06	0,87	0	0	0	0	0,46	0	0,47	0,07	0,044
3,5	37451	0	0,11	0	0,05	0,84	0	0	0	0	0	0	0,16	0,84	0,042
3,5	14609	0,06	0	0,01	0	0	0,02	0	0,23	0,68	0,25	0	0,69	0,06	0,042
3,5	18608	0	0	0,04	0	0	0,12	0	0,67	0,17	0	0	0,33	0,67	0,016
3,5	46317	0	0	0,04	0	0,83	0	0,04	0,01	0,08	0,01	0,87	0,12	0	0,04
3,5	40583	0	0,01	0,04	0	0	0,18	0	0,72	0,05	0,72	0,04	0	0,24	0,03
3,5	100000	0,02	0,21	0,75	0	0	0,01	0	0	0,01	0,75	0,21	0,01	0,03	0,924
3,5	22212	0	0,02	0,04	0,01	0	0	0	0	0,93	0,02	0,67	0,04	0,27	0,038
3,5	29332	0	0,13	0	0	0,79	0	0	0,08	0	0	0,2	0,8	0	0,048
3,5	62935	0,01	0	0,03	0	0	0	0,04	0,92	0	0,01	0,95	0,04	0	0,044
3,5	46996	0,01	0	0,04	0	0	0	0	0,9	0,05	0	0,02	0,01	0,97	0,016
3,5	41262	0,06	0	0	0	0	0,91	0	0	0,03	0,14	0	0	0,86	0,046
3,5	18787	0,01	0,11	0	0,02	0	0	0	0,83	0,03	0,13	0,02	0,83	0,02	0,044
3,5	13150	0	0	0	0,11	0,31	0	0	0,58	0	0,3	0	0,11	0,59	0,044
3,5	20684	0,03	0	0	0,03	0,01	0	0	0,93	0	0,03	0	0,01	0,96	0,042
3,5	53163	0,02	0	0,04	0	0	0	0	0,32	0,62	0	0,05	0,63	0,32	0,038
3,5	22803	0	0	0	0,23	0,77	0	0	0	0	0,77	0,23	0	0	0,044
3,5	16753	0	0	0	0,15	0,77	0,01	0	0,01	0,06	0,79	0	0,01	0,2	0,048
3,5	5176	0,01	0	0,02	0,07	0,82	0	0	0,08	0	0,07	0,02	0,89	0,02	0,032

3,5	7784	0,03	0	0	0,01	0,2	0,1	0,02	0,64	0	0,03	0,01	0,84	0,12	0,044
3,5	19765	0,05	0	0,01	0,02	0,71	0	0,01	0	0,2	0,07	0,72	0,2	0,01	0,048
3,5	17616	0,01	0	0	0	0,68	0,22	0,08	0,01	0	0	0,68	0,23	0,09	0,048
3,5	7651	0	0	0,04	0	0	0,25	0	0,71	0	0,71	0,04	0,08	0,17	0,034
3,5	21831	0	0,6	0	0,02	0,31	0	0	0	0,07	0,02	0,66	0,01	0,31	0,044
3,5	15060	0	0	0	0	0,92	0	0,08	0	0	0	1	0	0	0,048
3,5	37091	0,05	0,02	0,04	0	0,01	0	0	0,88	0	0,03	0,04	0,05	0,88	0,048
3,5	72823	0	0	0	0	0,53	0	0,11	0	0,36	0,53	0	0	0,47	0,046
3,5	18306	0	0	0	0,13	0,15	0,47	0	0,25	0	0	0,75	0,25	0	0,044
3,5	92665	0	0,05	0,04	0	0	0,38	0	0	0,53	0	0	0,61	0,39	0,034
3,5	74402	0,05	0	0,02	0	0,86	0,02	0	0	0,05	0,86	0,08	0,03	0,03	0,04
3,5	54055	0	0,01	0	0,12	0	0,86	0	0	0,01	0,01	0,87	0,12	0	0,048
3,5	42429	0,02	0,01	0,02	0,03	0,01	0	0	0,91	0	0,05	0,02	0,06	0,87	0,042
3,5	7808	0,05	0,19	0	0	0,44	0,32	0	0	0	0,04	0,23	0,01	0,72	0,044
3,75	16889	0	0	0,01	0,08	0,44	0,47	0	0	0	0,06	0,47	0	0,47	0,048
3,75	21273	0,02	0	0	0,08	0,87	0,01	0	0	0,02	0,03	0	0,95	0,02	0,048
3,75	7737	0	0,19	0,03	0	0,11	0,67	0	0	0	0,14	0	0,19	0,67	0,048
3,75	15914	0,01	0,02	0	0,02	0,92	0	0,03	0	0	0	0,02	0,06	0,92	0,048
3,75	100000	0	0	0,75	0	0,03	0	0	0,07	0,15	0	0	0,22	0,78	0,212
3,75	38132	0,03	0,04	0,01	0	0,67	0,22	0	0,02	0,01	0,27	0,02	0,67	0,04	0,048
3,75	12432	0,01	0,23	0,01	0,02	0,72	0,01	0	0	0	0,01	0,01	0,23	0,75	0,04
3,75	27200	0	0	0,04	0	0,96	0	0	0	0	0,73	0	0,27	0	0,04
3,75	28993	0	0	0,04	0	0	0	0	0,85	0,11	0	0,04	0,85	0,11	0,024
3,75	11282	0	0,1	0	0	0,76	0,14	0	0	0	0,86	0	0	0,14	0,044
3,75	100000	0	0,03	0,95	0	0	0	0,02	0	0	0	1	0	0	0,754
3,75	36308	0	0	0,04	0	0,88	0	0,08	0	0	0	0,01	0,91	0,08	0,048
3,75	41177	0	0	0,04	0	0,95	0	0	0,01	0	0	0,05	0,95	0	0,014
3,75	31175	0	0	0,04	0	0,75	0,15	0	0	0,06	0,06	0	0	0,94	0,008
3,75	16025	0	0	0	0,07	0,91	0	0	0,02	0	0	0,02	0,06	0,92	0,048
3,75	84607	0	0	0,04	0,07	0,85	0	0	0,04	0	0	0,1	0,85	0,05	0,046
3,75	13305	0	0	0,04	0,08	0,85	0,01	0,01	0	0,01	0,02	0,01	0,95	0,02	0,044
3,75	100000	0	0	1	0	0	0	0	0	0	0	1	0	0	0,672
3,75	38026	0	0	0,04	0	0,95	0	0	0,01	0	0,95	0,01	0	0,04	0,046

3,75	75136	0,05	0	0,03	0	0,62	0	0	0,3	0	0,01	0,33	0	0,66	0,048
3,75	3020	0	0,12	0,04	0	0	0	0,02	0	0,82	0	0,09	0,09	0,82	0,036
3,75	10862	0	0,02	0,04	0	0,91	0	0	0	0,03	0	0	0,04	0,96	0,002
3,75	43800	0,01	0,05	0,04	0	0	0,01	0	0	0,89	0	0,05	0,06	0,89	0,026
3,75	24006	0,02	0,25	0	0	0,73	0	0	0	0	0,26	0,05	0	0,69	0,048
3,75	49615	0,04	0,03	0	0	0,72	0,01	0	0,2	0	0,03	0,72	0,05	0,2	0,04
3,75	48241	0	0,07	0,03	0,05	0,33	0,48	0	0	0,04	0,01	0,38	0,09	0,52	0,048
3,75	16145	0	0,05	0	0,16	0,74	0	0	0,03	0,02	0,9	0	0,08	0,02	0,046
3,75	42676	0,01	0	0	0	0,91	0,01	0,06	0,01	0	0	0,08	0,35	0,57	0,046
3,75	18365	0	0	0,01	0,04	0,03	0	0,01	0	0,91	0,04	0,04	0	0,92	0,048
3,75	52286	0,01	0	0	0	0,51	0,3	0,08	0,1	0	0,28	0	0,1	0,62	0,044
3,75	3888	0,06	0	0,01	0	0,82	0	0	0	0,11	0,17	0,01	0,82	0	0,038
3,75	26134	0,06	0	0	0,01	0	0,93	0	0	0	0,97	0	0	0,03	0,046
3,75	20345	0,05	0	0	0,01	0,92	0	0	0	0,02	0,05	0,01	0,02	0,92	0,048
3,75	80046	0	0	0,04	0	0,62	0,28	0	0	0,06	0,34	0,03	0,63	0	0,032
3,75	11607	0	0,09	0	0,04	0,78	0	0	0,09	0	0	0,11	0,89	0	0,044
3,75	35159	0	0	0,03	0,14	0	0,79	0,04	0	0	0,79	0,04	0,17	0	0,048
3,75	60581	0	0	0	0	0,93	0	0,07	0	0	0	0	0,07	0,93	0,046
3,75	100000	0	0,39	0,04	0,47	0,01	0	0,04	0	0,05	0,45	0,04	0	0,51	0,512
3,75	2784	0	0	0,04	0	0	0	0	0	0,96	0	0,04	0	0,96	0,006
3,75	22165	0,06	0	0	0	0,89	0,04	0,01	0	0	0,07	0,89	0,04	0	0,048
3,75	7762	0	0	0	0,05	0,03	0	0	0,92	0	0	0,97	0	0,03	0,048
3,75	7769	0,01	0,08	0	0	0	0,91	0	0	0	0	0,93	0,01	0,06	0,048
3,75	26653	0	0	0,04	0	0,08	0,05	0	0	0,83	0	0,12	0	0,88	0,002
3,75	34429	0	0,15	0	0	0,59	0,23	0,03	0	0	0,21	0,02	0,18	0,59	0,044
3,75	47498	0	0,11	0	0,04	0,69	0	0	0	0,16	0,04	0,11	0	0,85	0,04
3,75	19323	0,04	0	0,02	0,02	0,92	0	0	0	0	0,92	0,06	0,02	0	0,048
3,75	7617	0,01	0	0,01	0,08	0,89	0,01	0	0	0	0,01	0	0,98	0,01	0,046
3,75	51048	0	0,01	0,04	0,07	0,87	0	0,01	0	0	0,87	0	0,03	0,1	0,042
3,75	7624	0,06	0	0	0	0,94	0	0	0	0	0,05	0	0	0,95	0,048
3,75	34295	0,03	0,03	0	0,02	0	0,33	0	0,02	0,57	0,34	0,02	0,03	0,61	0,046
3,75	100000	0	0	0,96	0,01	0	0,02	0,01	0	0	0	0,96	0,04	0	0,58
3,75	28606	0	0	0	0,1	0	0,04	0,01	0,85	0	0,03	0,58	0,36	0,03	0,042

3,75	26293	0,01	0,07	0	0	0,91	0	0	0,01	0	0,73	0,19	0,08	0	0,044
3,75	14672	0	0,01	0,02	0,07	0,59	0	0	0,31	0	0,18	0,31	0,07	0,44	0,042
3,75	27132	0,01	0	0,04	0	0	0,95	0	0	0	0	0,05	0	0,95	0,018
3,75	38414	0	0	0,04	0	0,39	0,01	0	0,24	0,32	0,01	0,04	0,95	0	0,022
3,75	19273	0	0,01	0,03	0	0,86	0	0,07	0,03	0	0,89	0,03	0,08	0	0,042
3,75	100000	0	0	0,98	0	0,02	0	0	0	0	0	0,98	0,02	0	0,588
3,75	15779	0	0	0,04	0	0	0,26	0	0,61	0,09	0,07	0,04	0,88	0,01	0,02
3,75	3396	0	0,01	0,04	0	0	0	0	0	0,95	0	0,33	0,67	0	0,036
3,75	77884	0	0	0,04	0,04	0,84	0	0	0,06	0,02	0,02	0,9	0,04	0,04	0,034
3,75	77398	0	0,01	0,04	0	0,16	0,06	0,02	0,71	0	0,16	0,02	0,72	0,1	0,04
3,75	43100	0	0	0,04	0	0	0,12	0	0,84	0	0	0,04	0,84	0,12	0,036
3,75	34582	0	0	0,04	0	0,71	0	0	0,03	0,22	0,03	0,04	0,93	0	0,028
3,75	16763	0	0,09	0	0,01	0	0	0,02	0,87	0,01	0,11	0,87	0,01	0,01	0,044
3,75	16326	0	0,01	0,04	0,09	0,86	0	0	0	0	0,11	0,03	0,81	0,05	0,046
3,75	6359	0	0,02	0,01	0	0,01	0,72	0,11	0,13	0	0,13	0,72	0,11	0,04	0,044
3,75	11310	0,03	0,96	0	0	0	0	0	0,01	0	0	0,01	0,03	0,96	0,04
3,75	94045	0	0	0,04	0	0,93	0	0,03	0	0	0,04	0	0,96	0	0,046
3,75	5524	0	0	0,04	0	0	0	0	0,96	0	0	0,04	0,96	0	0,032
3,75	9245	0,02	0	0	0,09	0,69	0	0	0	0,2	0,69	0,02	0,08	0,21	0,044
3,75	10755	0	0	0	0,21	0,77	0	0,02	0	0	0,79	0	0,21	0	0,044
3,75	8350	0,05	0	0	0	0,82	0	0,01	0,07	0,05	0,8	0,13	0,07	0	0,044
3,75	54205	0	0,06	0,04	0	0	0,01	0	0,87	0,02	0	0,1	0,02	0,88	0
3,75	6443	0,05	0	0,02	0	0	0	0	0,93	0	0	0,12	0,07	0,81	0,048
3,75	100000	0,17	0	0,57	0	0	0	0	0	0,26	0,26	0,57	0	0,17	0,484
3,75	100000	0,01	0	0,96	0	0,01	0	0,01	0,01	0	0	0,95	0,03	0,02	0,522
3,75	51740	0,04	0,03	0	0	0	0,92	0	0,01	0	0,07	0	0,92	0,01	0,042
3,75	14872	0	0,01	0,04	0	0,08	0,21	0	0,3	0,36	0,22	0,07	0,05	0,66	0,016
3,75	100000	0,06	0,04	0,89	0	0	0	0,01	0	0	0,07	0,82	0,05	0,06	0,53
3,75	9362	0,06	0	0,01	0	0,93	0	0	0	0	0,04	0,94	0,02	0	0,048
3,75	4026	0	0	0,04	0	0	0	0,04	0,85	0,07	0,03	0,04	0,9	0,03	0,036
3,75	17599	0,02	0,04	0,04	0,01	0	0,21	0	0	0,68	0,02	0,22	0,04	0,72	0,038
3,75	10230	0	0,95	0	0	0,01	0	0,03	0,01	0	0	0,01	0,04	0,95	0,046
3,75	57233	0,01	0,01	0	0,07	0,91	0	0	0	0	0,69	0	0,27	0,04	0,046

3,75	20745	0,02	0	0,04	0	0,69	0	0	0,25	0	0,02	0,25	0,73	0	0,038
3,75	19618	0,01	0,1	0	0	0,5	0	0,02	0,37	0	0,01	0	0,96	0,03	0,048
3,75	27135	0,02	0	0,04	0	0,45	0	0	0,04	0,45	0,41	0	0,08	0,51	0,028
3,75	29414	0	0	0	0,33	0,67	0	0	0	0	1	0	0	0	0,04
3,75	32874	0,03	0,09	0	0	0,55	0,02	0,01	0,29	0,01	0,32	0,08	0,07	0,53	0,048
3,75	46552	0	0,21	0,01	0	0,63	0,07	0	0,01	0,07	0,29	0,01	0	0,7	0,042
3,75	43745	0	0	0	0,09	0,88	0	0,03	0	0	0,06	0,06	0,88	0	0,048
3,75	19320	0,05	0	0	0	0,06	0	0	0,89	0	0	0	0	1	0,04
3,75	37744	0	0,15	0,01	0,01	0,82	0	0	0,01	0	0,15	0,85	0	0	0,044
3,75	29719	0	0,15	0	0	0,85	0	0	0	0	0	0	1	0	0,048
3,75	100000	0	0	1	0	0	0	0	0	0	0	1	0	0	0,646
3,75	24390	0,01	0	0	0,05	0,65	0,22	0	0,07	0	0,04	0,23	0,01	0,72	0,036
3,75	9469	0	0	0,04	0,07	0,04	0	0	0,85	0	0,41	0,06	0	0,53	0,042
3,75	20997	0	0	0,04	0,03	0	0	0	0,93	0	0	0,06	0,94	0	0,04
3,75	19224	0,03	0	0,02	0,06	0,01	0,87	0	0	0,01	0,12	0	0,88	0	0,048
4	36649	0	0,12	0,04	0	0	0,63	0	0	0,21	0,12	0,04	0,63	0,21	0,034
4	32183	0	0	0,04	0	0,01	0,46	0,06	0,43	0	0	0,1	0,37	0,53	0,046
4	48470	0	0,05	0,03	0	0,51	0	0	0,4	0,01	0,93	0,01	0,06	0	0,048
4	28750	0	0	0	0,14	0,53	0	0	0,33	0	0,45	0,02	0	0,53	0,048
4	5141	0	0	0	0,06	0,94	0	0	0	0	0	0,94	0	0,06	0,048
4	17136	0	0,05	0	0,06	0,87	0,01	0	0,01	0	0,05	0,01	0,71	0,23	0,048
4	17643	0	0	0	0,11	0,88	0	0,01	0	0	0,27	0,62	0,11	0	0,048
4	19143	0,02	0,04	0	0	0,91	0	0,03	0	0	0,94	0	0,02	0,04	0,046
4	6520	0,01	0,96	0	0,03	0	0	0	0	0	0	0	0,03	0,97	0,048
4	46302	0	0	0,04	0	0,6	0	0	0,36	0	0,57	0	0,04	0,39	0,03
4	38178	0	0	0	0,13	0,85	0	0	0,02	0	0,15	0	0,8	0,05	0,048
4	22866	0	0,06	0	0	0	0,93	0,01	0	0	0,93	0	0,07	0	0,04
4	42875	0	0,1	0	0,02	0,81	0,07	0	0	0	0,12	0	0,8	0,08	0,044
4	8418	0	0,03	0	0,17	0,01	0	0,03	0,75	0,01	0,77	0	0,17	0,06	0,048
4	83404	0	0,01	0,02	0	0,02	0,02	0,06	0,87	0	0,07	0,08	0,85	0	0,032
4	50841	0	0	0,04	0	0,92	0,04	0	0	0	0,04	0,04	0,92	0	0,022
4	43470	0	0	0	0,23	0,76	0	0	0	0,01	0,76	0	0,23	0,01	0,048
4	8894	0,01	0	0	0,01	0	0	0,04	0,94	0	0,01	0	0	0,99	0,046

4	24315	0,03	0	0	0	0,94	0	0,03	0	0	0	0,03	0	0,97	0,048
4	9802	0,03	0	0,04	0	0	0,93	0	0	0	0,03	0,04	0,19	0,74	0,044
4	14731	0	0,15	0	0	0,8	0,04	0,01	0	0	0,15	0,78	0,03	0,04	0,046
4	63610	0,02	0,31	0	0	0,08	0	0	0	0,59	0,02	0	0,03	0,95	0,048
4	10879	0,06	0	0	0	0	0,94	0	0	0	0	0	1	0	0,048
4	27073	0,01	0	0	0,04	0,23	0	0	0,72	0	0,03	0,2	0,05	0,72	0,048
4	44520	0	0,84	0	0,04	0,01	0	0	0,11	0	0	0,15	0	0,85	0,048
4	60779	0	0,95	0	0,03	0	0	0,01	0,01	0	0,02	0,04	0	0,94	0,04
4	12447	0	0,01	0	0,13	0,16	0,69	0	0,01	0	0,69	0	0,14	0,17	0,048
4	55317	0	0	0	0	0	0,26	0,07	0,67	0	0,02	0,07	0,24	0,67	0,046
4	91770	0,04	0	0	0	0,61	0,01	0,01	0	0,33	0,35	0,04	0,61	0	0,046
4	100000	0,09	0	0,86	0	0,04	0	0,01	0	0	0,34	0,53	0,04	0,09	0,664
4	18454	0	0,01	0,04	0	0,26	0,12	0	0,34	0,23	0,34	0,14	0	0,52	0,026
4	7793	0	0,9	0	0	0	0,02	0	0,08	0	0	0	0,08	0,92	0,046
4	14039	0,05	0	0	0,02	0,93	0	0	0	0	0,91	0,07	0	0,02	0,048
4	31355	0	0	0	0,24	0,7	0	0	0,06	0	0,75	0,24	0	0,01	0,044
4	100000	0,02	0	0,98	0	0	0	0	0	0	0	0,98	0,02	0	0,616
4	34927	0,07	0,01	0	0	0	0,03	0	0,89	0	0,03	0,04	0,9	0,03	0,048
4	98611	0	0,22	0	0,04	0,74	0	0	0	0	0	0,22	0,14	0,64	0,048
4	45312	0,01	0,06	0,02	0,03	0,83	0,04	0	0	0,01	0,05	0,14	0,72	0,09	0,044
4	35757	0,06	0	0	0	0	0,76	0	0,15	0,03	0,08	0,13	0,79	0	0,042
4	82054	0,03	0	0	0	0,65	0,06	0,05	0,11	0,1	0,03	0,15	0,71	0,11	0,048
4	37593	0	0,1	0,01	0,02	0,68	0,17	0	0,02	0	0,02	0,3	0,67	0,01	0,042
4	42611	0	0,03	0,01	0,1	0,46	0,18	0	0,01	0,21	0,65	0,04	0,01	0,3	0,04
4	25635	0	0	0,03	0,07	0,71	0	0	0,19	0	0,26	0,02	0	0,72	0,048
4	2770	0	0,01	0,04	0	0,35	0,02	0,02	0	0,56	0,06	0,04	0,34	0,56	0,038
4	40905	0,03	0,02	0	0,04	0,02	0,89	0	0	0	0,03	0,81	0,04	0,12	0,046
4	39223	0,03	0	0,03	0	0	0,52	0,01	0,25	0,16	0,02	0,27	0,55	0,16	0,042
4	62894	0	0	0	0,12	0,87	0	0	0,01	0	0	0	0,9	0,1	0,044
4	16795	0	0,3	0	0	0,7	0	0	0	0	0	0	0,33	0,67	0,044
4	3184	0	0,02	0,03	0,05	0,87	0	0	0,03	0	0,08	0,31	0,61	0	0,048
4	28067	0	0,13	0	0	0,87	0	0	0	0	0	0,01	0,87	0,12	0,048
4	3427	0	0	0,03	0	0	0,64	0,12	0,08	0,13	0,61	0,19	0,12	0,08	0,04

4	41437	0	0	0,04	0	0,02	0	0	0	0,94	0	0,04	0,94	0,02	0,018
4	40003	0	0	0,04	0,01	0,36	0	0	0,01	0,58	0,01	0,29	0,12	0,58	0,024
4	16222	0,05	0	0	0	0,79	0,02	0,01	0,12	0,01	0,13	0,01	0,05	0,81	0,046
4	64938	0	0	0,01	0,07	0,92	0	0	0	0	0	0,92	0,08	0	0,048
4	62940	0	0,01	0,04	0,01	0,17	0,63	0	0,14	0	0,01	0,21	0,77	0,01	0,046
4	61540	0,01	0	0,04	0	0,03	0	0	0,79	0,13	0,18	0,69	0,13	0	0,032
4	8117	0,01	0,22	0,01	0	0,73	0	0,03	0	0	0,01	0,01	0,16	0,82	0,044
4	10350	0,03	0	0,04	0,01	0,92	0	0	0	0	0,92	0,04	0,04	0	0,046
4	20310	0	0	0	0,12	0,42	0	0	0,26	0,2	0,42	0,26	0,12	0,2	0,046
4	100000	0	0	0,74	0	0	0,03	0	0,16	0,07	0	0,55	0,45	0	0,308
4	100000	0	0	1	0	0	0	0	0	0	0	0,05	0	0,95	0,174
4	65305	0	0,93	0	0	0,01	0	0	0,06	0	0	0,5	0,07	0,43	0,044
4	48157	0	0	0	0,1	0,88	0	0,01	0	0,01	0	0,11	0,88	0,01	0,048
4	73592	0	0	0,04	0	0,94	0,01	0	0	0,01	0,01	0,01	0,02	0,96	0,002
4	96191	0	0	0,04	0	0	0,32	0	0	0,64	0	0,04	0,96	0	0,03
4	81127	0	0,01	0	0	0,42	0,5	0,07	0	0	0	0	0,08	0,92	0,048
4	17206	0	0,27	0	0	0	0	0	0,73	0	0,26	0	0	0,74	0,04
4	19756	0	0	0,04	0	0,95	0	0,01	0	0	0,01	0	0,99	0	0,028
4	24630	0	0	0,04	0	0	0	0	0,91	0,05	0,01	0,02	0,07	0,9	0,004
4	24759	0,01	0,01	0,04	0	0	0,26	0	0,68	0	0	0,6	0,39	0,01	0,04
4	12333	0	0,96	0	0	0,04	0	0	0	0	0,02	0,98	0	0	0,042
4	69087	0	0,15	0,03	0	0,63	0	0	0,13	0,06	0,01	0,47	0,23	0,29	0,044
4	12793	0,03	0	0,02	0	0,58	0,03	0,04	0,27	0,03	0,64	0,29	0,01	0,06	0,042
4	4215	0	0	0,04	0	0,03	0,74	0	0,14	0,05	0	0,18	0,77	0,05	0,026
4	15757	0	0	0	0	0,66	0	0,33	0,01	0	0,94	0,04	0	0,02	0,044
4	53786	0,05	0	0,04	0	0	0,05	0	0	0,86	0,05	0	0,9	0,05	0,042
4	98284	0	0	0	0,09	0	0,02	0	0,85	0,04	0	0,87	0,09	0,04	0,04
4	19463	0	0	0	0,06	0	0	0	0,94	0	0	0,06	0	0,94	0,042
4	27452	0,02	0,06	0	0,02	0	0	0	0,9	0	0,23	0,02	0,75	0	0,048
4	53517	0	0,95	0	0	0	0	0	0	0,05	0	0	0,05	0,95	0,044
4	100000	0	0	0,23	0,2	0,5	0	0	0,07	0	0,09	0,14	0	0,77	0,214
4	32911	0	0,1	0	0	0,9	0	0	0	0	0,13	0,03	0,84	0	0,042
4	16836	0,06	0	0	0	0,94	0	0	0	0	0,05	0,02	0,93	0	0,048

4	36667	0	0	0,04	0	0	0,01	0	0,53	0,42	0	0,05	0	0,95	0,006
4	17893	0	0,07	0	0,06	0,72	0,08	0	0,03	0,04	0,18	0,78	0	0,04	0,046
4	32853	0,04	0	0,02	0	0,85	0,01	0,01	0	0,07	0,9	0,03	0,07	0	0,048
4	34789	0,05	0	0	0,02	0,16	0,68	0	0	0,09	0,18	0	0,82	0	0,038
4	5497	0,06	0	0	0	0	0,54	0	0,34	0,06	0,62	0,06	0	0,32	0,048
4	19430	0,03	0	0,04	0	0	0	0	0,11	0,82	0,01	0,04	0	0,95	0,038
4	12302	0	0,02	0	0,06	0,91	0	0	0,01	0	0,06	0,9	0	0,04	0,048
4	15527	0,01	0	0	0,05	0,93	0	0	0,01	0	0	0,99	0	0,01	0,048
4	56646	0	0,18	0,01	0	0,52	0,17	0	0,12	0	0,17	0,15	0,27	0,41	0,048
4	91997	0,05	0	0,03	0	0,61	0,15	0	0,16	0	0,16	0,03	0,05	0,76	0,038
4	35170	0	0	0,04	0	0,95	0	0,01	0	0	0	0	0,8	0,2	0,034
4	35026	0	0	0	0,06	0,88	0	0,05	0,01	0	0	0,11	0,89	0	0,048
4	24852	0	0,94	0	0,01	0	0,05	0	0	0	0,01	0	0,05	0,94	0,048
4	99900	0	0,01	0,04	0	0	0	0	0,15	0,8	0,11	0,04	0,01	0,84	0,012
4	6483	0	0,05	0,04	0,03	0,15	0,02	0	0	0,71	0,21	0,06	0,02	0,71	0,044
4	20360	0	0,04	0	0,1	0,85	0	0,01	0	0	0,2	0,01	0,79	0	0,036
4,25	15187	0	0	0,04	0,01	0,01	0	0	0,94	0	0	0	0,05	0,95	0,01
4,25	9043	0	0	0,04	0	0,64	0	0	0,01	0,31	0	0,05	0,31	0,64	0,01
4,25	37373	0	0,27	0	0	0,63	0,01	0,04	0,04	0,01	0	0,94	0,01	0,05	0,046
4,25	17342	0,03	0,06	0	0	0,6	0,04	0,04	0,22	0,01	0,01	0,03	0,7	0,26	0,042
4,25	49395	0	0,11	0	0,05	0	0	0	0,84	0	0	0,05	0,11	0,84	0,036
4,25	43243	0	0,12	0	0,01	0,87	0	0	0	0	0	0	1	0	0,038
4,25	50721	0	0	0,04	0	0,25	0,69	0,02	0	0	0,21	0,73	0,04	0,02	0,046
4,25	31226	0	0,09	0	0,01	0	0,89	0	0,01	0	0,89	0	0,1	0,01	0,042
4,25	50886	0,03	0,06	0	0,01	0,81	0	0,01	0,08	0	0,08	0,83	0,09	0	0,042
4,25	71065	0	0	0	0	0,88	0	0,12	0	0	0,01	0	0,99	0	0,044
4,25	5010	0	0	0,04	0,01	0	0,65	0	0,3	0	0	0,15	0,84	0,01	0,032
4,25	56872	0,06	0	0	0	0,03	0	0	0,68	0,23	0,03	0,22	0,72	0,03	0,046
4,25	66679	0,04	0	0,04	0	0	0	0	0	0,92	0	0,08	0,88	0,04	0,048
4,25	10566	0	0	0,04	0	0	0,82	0	0,08	0,06	0,96	0	0	0,04	0,04
4,25	7965	0	0	0	0,09	0,86	0	0,05	0	0	0	0,13	0,87	0	0,048
4,25	50478	0,01	0	0	0,04	0,07	0,86	0,02	0	0	0,05	0,02	0	0,93	0,048
4,25	61135	0	0,93	0,04	0,01	0	0	0,01	0,01	0	0,01	0,05	0,01	0,93	0,038

4,25	9762	0	0	0,04	0	0,03	0,17	0	0	0,76	0	0,07	0,14	0,79	0,022
4,25	30238	0	0,07	0	0,02	0,75	0,06	0,01	0,06	0,03	0,82	0,06	0,06	0,06	0,044
4,25	14914	0,02	0	0,01	0,01	0,72	0,09	0,03	0,1	0,02	0,01	0,85	0,04	0,1	0,036
4,25	13775	0,02	0	0,04	0	0	0	0	0	0,94	0	0,04	0,96	0	0,044
4,25	38305	0	0	0,04	0,01	0,12	0	0,03	0,72	0,08	0,01	0,17	0,81	0,01	0,048
4,25	19032	0,01	0,01	0,04	0	0,93	0	0	0	0,01	0	0,01	0,94	0,05	0,024
4,25	33785	0	0	0	0,06	0	0	0	0,94	0	0	0,06	0	0,94	0,048
4,25	38601	0	0	0,04	0,01	0	0,95	0	0	0	0	0,03	0,97	0	0,034
4,25	16089	0,05	0,02	0	0	0,93	0	0	0	0	0,95	0	0	0,05	0,042
4,25	100000	0	0	0,98	0,01	0	0	0,01	0	0	0,01	0,98	0	0,01	0,59
4,25	100000	0	0	0,98	0	0	0	0	0,02	0	0	0,98	0,02	0	0,596
4,25	19472	0,03	0	0	0	0,02	0,88	0,07	0	0	0,09	0	0,91	0	0,04
4,25	29509	0	0,13	0	0	0,86	0,01	0	0	0	0,14	0	0,86	0	0,042
4,25	30715	0,05	0,01	0,01	0,01	0,76	0	0	0	0,16	0,82	0,01	0,1	0,07	0,048
4,25	100000	0	0	0,99	0,01	0	0	0	0	0	0	0,93	0,07	0	0,5
4,25	100000	0,56	0	0,34	0,03	0,07	0	0	0	0	0,03	0,9	0,07	0	0,74
4,25	8319	0	0	0,04	0	0,92	0	0	0	0,04	0	0,78	0,14	0,08	0,008
4,25	30615	0	0	0,01	0,08	0	0,38	0	0,51	0,02	0,02	0,01	0,47	0,5	0,048
4,25	78903	0	0,14	0	0,03	0	0,49	0	0	0,34	0	0,16	0,35	0,49	0,042
4,25	74601	0,04	0	0,04	0	0	0,9	0	0	0,02	0,06	0,04	0	0,9	0,042
4,25	45325	0	0,03	0,04	0	0	0	0	0,76	0,17	0	0,07	0,01	0,92	0,004
4,25	2155	0	0	0,04	0,01	0	0,29	0	0,55	0,11	0,03	0,56	0,41	0	0,024
4,25	100000	0	0,01	0,99	0	0	0	0	0	0	0	1	0	0	0,61
4,25	28992	0,05	0	0	0	0,13	0	0	0,82	0	0,03	0	0,19	0,78	0,046
4,25	78644	0	0	0	0	0,95	0	0,05	0	0	0	1	0	0	0,038
4,25	31391	0	0,01	0,04	0	0	0	0	0,95	0	0,83	0,13	0,04	0	0,04
4,25	43618	0	0,1	0,01	0	0	0,25	0	0,63	0,01	0,08	0,01	0,91	0	0,046
4,25	70496	0,06	0	0	0	0	0,92	0	0	0,02	0	0,56	0,06	0,38	0,046
4,25	16925	0,05	0	0	0	0,95	0	0	0	0	0	0	1	0	0,04
4,25	13452	0,06	0	0	0	0	0,89	0,03	0,02	0	0,89	0,08	0,03	0	0,044
4,25	15922	0,01	0,1	0	0	0,86	0,01	0	0,01	0,01	0,59	0,29	0,11	0,01	0,048
4,25	52625	0,04	0	0,04	0	0,03	0,89	0	0	0	0,04	0,04	0,16	0,76	0,048
4,25	18856	0,04	0	0,03	0	0	0,67	0	0,26	0	0,71	0,17	0	0,12	0,048

4,25	16730	0	0	0,01	0,07	0	0,92	0	0	0	0,05	0,02	0	0,93	0,04
4,25	32638	0	0	0,04	0	0,9	0	0,01	0	0,05	0,9	0,1	0	0	0,046
4,25	49065	0,01	0	0,04	0	0	0	0	0,82	0,13	0,13	0	0,82	0,05	0,03
4,25	34742	0,01	0	0,04	0	0	0,94	0	0,01	0	0,01	0,04	0,94	0,01	0,03
4,25	100000	0,02	0	0,95	0	0,03	0	0	0	0	0	0,96	0	0,04	0,538
4,25	47610	0	0,04	0	0,06	0,9	0	0	0	0	0,04	0,06	0,64	0,26	0,038
4,25	23137	0,02	0,07	0	0	0,88	0,02	0	0	0,01	0,11	0	0,88	0,01	0,044
4,25	2070	0	0	0	0,13	0,87	0	0	0	0	0	0	1	0	0,042
4,25	35327	0,05	0	0	0,02	0	0,85	0	0	0,08	0,15	0,85	0	0	0,048
4,25	38230	0	0	0,04	0,05	0	0,7	0	0,14	0,07	0,13	0,05	0,82	0	0,046
4,25	59375	0	0,08	0,04	0	0	0	0	0	0,88	0	0,04	0	0,96	0,024
4,25	7107	0	0,02	0,04	0	0	0	0	0,52	0,42	0	0,57	0,42	0,01	0,024
4,25	10364	0,04	0,08	0	0	0,88	0	0	0	0	0,05	0,93	0,02	0	0,044
4,25	41637	0	0	0	0,15	0,81	0	0,02	0,02	0	0,74	0,02	0,22	0,02	0,046
4,25	45571	0	0,16	0,01	0	0,29	0	0	0,54	0	0	0,7	0,3	0	0,048
4,25	24971	0	0	0,04	0	0	0,1	0,09	0,5	0,27	0,55	0,05	0,34	0,06	0,048
4,25	42662	0	0,26	0	0	0,74	0	0	0	0	0,26	0	0	0,74	0,048
4,25	50896	0	0	0,04	0,01	0,15	0,78	0,02	0	0	0	0,04	0,79	0,17	0,036
4,25	12637	0	0	0,04	0	0,03	0,86	0,07	0	0	0	0,04	0,89	0,07	0,042
4,25	100000	0	0	0,97	0	0	0,03	0	0	0	0	0,97	0	0,03	0,526
4,25	8904	0	0	0,04	0	0	0,03	0	0,74	0,19	0	0,04	0,74	0,22	0,034
4,25	100000	0,45	0,01	0,51	0,02	0,01	0	0	0	0	0,02	0,97	0	0,01	0,768
4,25	4537	0,02	0	0,04	0	0,29	0,57	0	0,08	0	0,29	0,04	0,57	0,1	0,046
4,25	34904	0	0	0,04	0	0	0,03	0	0	0,93	0,03	0,04	0	0,93	0,014
4,25	20188	0	0	0,04	0	0,95	0	0,01	0	0	0,05	0,01	0	0,94	0,01
4,25	16196	0,01	0,07	0	0	0,92	0	0	0	0	0,92	0	0,01	0,07	0,048
4,25	25253	0	0,04	0,03	0	0,88	0	0,02	0	0,03	0,88	0,09	0,01	0,02	0,044
4,25	67743	0	0,69	0,01	0	0	0	0	0	0,3	0,04	0,08	0	0,88	0,048
4,25	8566	0	0,08	0	0,04	0	0,88	0	0	0	0	0,04	0,96	0	0,046
4,25	48494	0,05	0	0,01	0	0	0	0	0,05	0,89	0	0,08	0,01	0,91	0,042
4,25	24840	0	0	0,04	0	0,12	0,72	0,11	0,01	0	0,95	0,04	0	0,01	0,048
4,25	39268	0	0	0	0,1	0,87	0	0	0,01	0,02	0,08	0,05	0,87	0	0,04
4,25	3548	0	0,04	0,04	0	0,66	0	0	0	0,26	0,29	0,22	0	0,49	0,032

4,25	53207	0,06	0,04	0	0	0,8	0	0	0	0,1	0,08	0,04	0,79	0,09	0,042
4,25	17359	0	0	0	0,07	0,87	0	0	0,05	0,01	0,01	0,97	0,02	0	0,048
4,25	8161	0	0,09	0	0,07	0,26	0	0	0,54	0,04	0,09	0,3	0,07	0,54	0,048
4,25	9327	0	0	0	0,12	0	0,87	0	0	0,01	0,01	0,99	0	0	0,048
4,25	49904	0	0,49	0,01	0	0,49	0	0,01	0	0	0,01	0,49	0,1	0,4	0,048
4,25	25284	0	0,07	0,04	0	0	0	0	0	0,89	0,04	0,07	0,02	0,87	0,014
4,25	33820	0	0,25	0	0,02	0,73	0	0	0	0	0	0,02	0,25	0,73	0,048
4,25	32706	0,06	0	0	0	0	0,94	0	0	0	0,25	0,69	0,06	0	0,042
4,25	100000	0	0	1	0	0	0	0	0	0	0	1	0	0	0,658
4,25	5000	0	0	0,04	0	0,01	0,95	0	0	0	0	0,16	0,01	0,83	0,01
4,25	50889	0,02	0,01	0	0,03	0	0	0	0,94	0	0	0,92	0	0,08	0,044
4,25	20422	0	0,08	0,04	0	0,81	0	0	0	0,07	0	0,74	0,18	0,08	0,032
4,25	42729	0	0,15	0	0	0,85	0	0	0	0	0	0	1	0	0,048
4,25	93306	0	0,9	0,04	0	0	0,05	0,01	0	0	0	0,29	0,01	0,7	0,044
4,25	55836	0	0,01	0,03	0,09	0,45	0	0	0,42	0	0,87	0,1	0	0,03	0,04
4,25	48049	0	0,2	0,04	0	0	0	0	0,7	0,06	0,08	0	0,05	0,87	0,044
4,25	20971	0,06	0	0	0	0,89	0,03	0,01	0,01	0	0,76	0,2	0,04	0	0,048
4,5	85844	0,01	0,98	0	0,01	0	0	0	0	0	0	0	0,06	0,94	0,044
4,5	29979	0	0	0	0,11	0	0,86	0,02	0,01	0	0,11	0	0,87	0,02	0,044
4,5	25042	0	0	0,04	0	0	0,26	0	0,7	0	0,07	0	0,04	0,89	0,01
4,5	100000	0	0	1	0	0	0	0	0	0	0	1	0	0	0,61
4,5	58977	0	0	0	0,09	0,19	0	0	0,16	0,56	0	0,56	0,16	0,28	0,046
4,5	20126	0	0	0,04	0	0,96	0	0	0	0	0	1	0	0	0,002
4,5	70594	0	0,09	0	0,03	0,85	0,01	0,02	0	0	0,12	0,85	0,03	0	0,036
4,5	24554	0	0,02	0	0	0	0	0,07	0,91	0	0	0,14	0,07	0,79	0,044
4,5	26056	0,04	0,03	0,01	0	0,02	0	0,01	0,24	0,65	0,28	0,65	0,04	0,03	0,048
4,5	38223	0,01	0	0,04	0,01	0,03	0,91	0	0	0	0,01	0	0,94	0,05	0,024
4,5	36358	0,06	0	0	0	0,01	0	0	0,93	0	0,05	0,73	0,22	0	0,044
4,5	34439	0	0	0,04	0	0,5	0,05	0	0	0,41	0,41	0	0,59	0	0,03
4,5	13274	0,03	0,01	0,04	0	0	0	0	0	0,92	0	0,96	0,01	0,03	0,048
4,5	7001	0	0,13	0	0,04	0,61	0	0	0,22	0	0,04	0	0,22	0,74	0,048
4,5	80197	0,03	0	0,04	0	0,93	0	0	0	0	0,06	0	0,94	0	0,048
4,5	11429	0	0	0,04	0	0	0	0	0,01	0,95	0,01	0,04	0	0,95	0,014

4,5	21622	0	0,08	0,02	0	0,12	0,77	0,01	0	0	0,08	0,14	0,78	0	0,044
4,5	14537	0,03	0,04	0	0	0	0,89	0	0,03	0,01	0,04	0,96	0	0	0,044
4,5	94540	0	0	0	0,06	0	0	0	0,94	0	0	0,94	0	0,06	0,046
4,5	88717	0	0	0,04	0	0,14	0,31	0	0	0,51	0	0,49	0,51	0	0,028
4,5	48733	0	0,03	0,04	0	0,12	0	0	0	0,81	0	0,07	0,81	0,12	0,048
4,5	34627	0	0	0,04	0	0	0,89	0,05	0,02	0	0,08	0,69	0,21	0,02	0,046
4,5	17887	0,06	0	0	0	0,91	0	0	0,03	0	0,06	0	0,03	0,91	0,04
4,5	37822	0	0	0,01	0,12	0,03	0	0	0,74	0,1	0,01	0,02	0,49	0,48	0,048
4,5	16079	0	0,01	0	0,09	0,9	0	0	0	0	0	0,64	0,36	0	0,048
4,5	29616	0,05	0	0,03	0	0	0,81	0	0	0,11	0,81	0,05	0,14	0	0,044
4,5	9843	0	0,02	0	0,09	0,89	0	0	0	0	0,08	0,02	0,89	0,01	0,038
4,5	41725	0,06	0	0	0	0,92	0,02	0	0	0	0	0	0,98	0,02	0,048
4,5	5286	0	0	0,04	0	0,43	0	0	0	0,53	0,43	0,04	0,53	0	0,038
4,5	14396	0	0	0,02	0,05	0	0	0	0,93	0	0,02	0	0	0,98	0,044
4,5	22459	0	0,03	0,04	0	0	0	0	0,42	0,51	0,07	0,04	0,89	0	0,032
4,5	56340	0	0,2	0	0,02	0,75	0,03	0	0	0	0,23	0	0	0,77	0,048
4,5	19855	0	0	0	0,07	0,93	0	0	0	0	0,07	0,93	0	0	0,046
4,5	53483	0,05	0	0	0	0,92	0,03	0	0	0	0,03	0	0	0,97	0,036
4,5	11593	0	0	0,04	0,03	0,1	0	0	0	0,83	0,15	0,14	0,69	0,02	0,04
4,5	35506	0	0,09	0,04	0	0,85	0	0	0	0,02	0,1	0,58	0,27	0,05	0,046
4,5	35624	0,03	0,03	0,03	0,02	0	0,01	0	0	0,88	0,29	0,04	0,06	0,61	0,04
4,5	34748	0	0,01	0,04	0	0,18	0	0	0	0,77	0,18	0,77	0	0,05	0,024
4,5	49442	0	0,07	0	0	0,91	0	0,02	0	0	0,97	0	0	0,03	0,048
4,5	9786	0	0	0	0,09	0	0	0,02	0,88	0,01	0	0,01	0,9	0,09	0,048
4,5	11797	0	0	0,03	0,12	0,85	0	0	0	0	0,75	0	0,12	0,13	0,048
4,5	51051	0	0	0,01	0,01	0,22	0,51	0,09	0	0,16	0,09	0	0,68	0,23	0,044
4,5	19300	0,01	0,14	0	0	0	0,08	0	0,77	0	0,13	0,78	0,01	0,08	0,046
4,5	100000	0	0	1	0	0	0	0	0	0	0	0,99	0,01	0	0,552
4,5	27422	0	0	0	0	0,89	0	0,11	0	0	0	0,11	0,89	0	0,046
4,5	100000	0	0	1	0	0	0	0	0	0	0	1	0	0	0,62
4,5	16510	0	0	0,04	0	0,31	0	0	0,01	0,64	0,31	0,05	0	0,64	0,022
4,5	62192	0	0,11	0	0	0,85	0	0,02	0,01	0,01	0	0	0,88	0,12	0,044
4,5	21503	0	0,05	0	0,05	0,75	0	0	0	0,15	0,02	0,05	0,15	0,78	0,046

4,5	41026	0	0,15	0	0	0	0,81	0	0,04	0	0,4	0	0	0,6	0,048
4,5	30415	0,06	0	0	0	0,66	0,05	0	0,23	0	0,11	0,66	0,23	0	0,036
4,5	31027	0	0	0	0,07	0,91	0,01	0	0,01	0	0	0,92	0,07	0,01	0,046
4,5	22290	0	0	0,04	0,01	0,02	0	0	0,86	0,07	0,05	0,1	0,85	0	0,028
4,5	19460	0	0	0,04	0,08	0,01	0,77	0,01	0	0,09	0	0,02	0,89	0,09	0,044
4,5	39529	0	0	0	0,28	0,72	0	0	0	0	1	0	0	0	0,046
4,5	12313	0	0	0	0,11	0,89	0	0	0	0	0	0,11	0,87	0,02	0,046
4,5	38032	0,06	0	0	0	0	0	0	0,94	0	0	0	1	0	0,048
4,5	100000	0,16	0	0,84	0	0	0	0	0	0	0	0,96	0,04	0	0,584
4,5	16125	0	0,11	0	0	0,86	0	0	0,02	0,01	0,48	0	0,52	0	0,046
4,5	26440	0	0	0,04	0	0	0,83	0,03	0,03	0,07	0,03	0	0,1	0,87	0,03
4,5	7507	0,02	0	0,01	0	0,93	0	0,04	0	0	0	0	0,96	0,04	0,046
4,5	54566	0	0,11	0	0	0,12	0,74	0,02	0	0,01	0,1	0,41	0,46	0,03	0,04
4,5	38247	0	0	0,04	0	0,01	0	0	0,7	0,25	0,02	0,26	0	0,72	0,004
4,5	21797	0,01	0,02	0,04	0	0,21	0,15	0,02	0,45	0,1	0,25	0,03	0,69	0,03	0,048
4,5	15465	0,04	0	0,04	0	0	0,91	0	0,01	0	0	0,01	0,21	0,78	0,044
4,5	61723	0	0,35	0,04	0	0	0	0,01	0,6	0	0,01	0,35	0,04	0,6	0,04
4,5	52137	0	0	0	0	0,28	0	0,09	0,63	0	0	0	0,72	0,28	0,042
4,5	46144	0,01	0	0,01	0,13	0	0	0	0,85	0	0,02	0	0,98	0	0,048
4,5	18908	0	0,08	0	0,02	0,9	0	0	0	0	0,08	0,33	0,57	0,02	0,044
4,5	19190	0	0,15	0,04	0	0	0,01	0	0,28	0,52	0,14	0,06	0,02	0,78	0,04
4,5	100000	0,38	0	0,59	0	0	0	0	0,03	0	0,4	0,57	0,03	0	0,876
4,5	13822	0,02	0	0,04	0	0,72	0	0	0,1	0,12	0,82	0,06	0,12	0	0,048
4,5	67051	0,04	0	0	0,02	0,94	0	0	0	0	0	0,06	0,94	0	0,038
4,5	51078	0	0,08	0,03	0	0,71	0,04	0	0,14	0	0,19	0,08	0,73	0	0,044
4,5	18217	0	0,12	0	0	0,86	0	0,01	0,01	0	0,34	0,64	0	0,02	0,038
4,5	37627	0	0	0	0	0	0,9	0,1	0	0	0	0,9	0,1	0	0,044
4,5	64345	0,04	0	0	0,03	0,53	0	0	0,4	0	0,01	0	0,47	0,52	0,044
4,5	14790	0	0	0,02	0,01	0,79	0,01	0,07	0	0,1	0,21	0,01	0,09	0,69	0,046
4,5	72294	0	0,01	0,01	0,03	0,09	0,61	0,06	0,03	0,16	0,09	0,17	0,17	0,57	0,048
4,5	47668	0	0	0	0,25	0	0	0	0,75	0	0,7	0,25	0,05	0	0,048
4,5	47694	0	0,82	0	0	0,02	0	0,02	0,14	0	0,02	0	0,02	0,96	0,046
4,5	65679	0	0,09	0,04	0	0,24	0,63	0	0	0	0,12	0,24	0	0,64	0,03

4,5	24024	0,03	0,09	0	0	0	0,01	0,01	0,86	0	0,12	0,02	0	0,86	0,046
4,5	29725	0	0	0,04	0	0,56	0,35	0	0,05	0	0,35	0,56	0,09	0	0,022
4,5	34559	0	0	0,04	0,02	0	0,44	0	0	0,5	0,47	0	0	0,53	0,034
4,5	19545	0,02	0,83	0,03	0	0	0	0	0,09	0,03	0	0,03	0,02	0,95	0,046
4,5	65087	0	0,01	0	0,17	0,82	0	0	0	0	0,82	0,1	0,07	0,01	0,048
4,5	50368	0	0,12	0	0	0,2	0	0	0	0,68	0,14	0	0,86	0	0,046
4,5	74446	0,01	0	0	0,05	0,9	0,04	0	0	0	0	0	0,1	0,9	0,04
4,5	47391	0	0	0,04	0	0,53	0,13	0	0	0,3	0,53	0,04	0,13	0,3	0,028
4,5	42688	0,01	0,04	0	0,05	0,9	0	0	0	0	0	0,88	0,08	0,04	0,042
4,5	100000	0	0	0,96	0	0	0	0,01	0,01	0,02	0,01	0,55	0,38	0,06	0,486
4,5	100000	0	0,38	0,02	0,29	0,01	0,08	0	0,22	0	0,02	0,31	0,38	0,29	0,31
4,5	22167	0,04	0	0,04	0	0	0	0	0,92	0	0,04	0,04	0	0,92	0,044
4,5	94394	0,01	0,11	0	0	0	0,05	0	0,83	0	0,05	0,17	0,78	0	0,044
4,5	26697	0	0,02	0,04	0	0,94	0	0	0	0	0,02	0	0,98	0	0,012
4,5	7701	0	0,01	0	0,12	0,86	0,01	0	0	0	0,01	0,12	0,87	0	0,048
4,5	64492	0,04	0,01	0,04	0	0,9	0,01	0	0	0	0,05	0,04	0	0,91	0,044
4,5	31643	0,02	0	0,04	0	0	0,49	0	0,36	0,09	0,48	0,05	0,36	0,11	0,044
4,5	27260	0,03	0	0,04	0,02	0	0,04	0	0	0,87	0,02	0,07	0,08	0,83	0,044
4,75	41133	0	0,02	0,04	0	0,94	0	0	0	0	0,02	0	0,94	0,04	0,028
4,75	7034	0,06	0	0	0	0,12	0,59	0	0,23	0	0,01	0	0,87	0,12	0,048
4,75	36045	0,01	0,17	0	0	0,13	0,35	0	0,27	0,07	0	0,48	0,33	0,19	0,048
4,75	31612	0,01	0,97	0,01	0	0	0	0	0	0,01	0,05	0	0	0,95	0,034
4,75	51282	0	0,95	0,01	0,03	0	0	0,01	0	0	0	0,04	0,01	0,95	0,046
4,75	100000	0	0	0,97	0	0	0	0,03	0	0	0	0,97	0,03	0	0,522
4,75	55366	0,05	0	0,01	0	0,49	0,02	0	0,43	0	0,43	0,48	0,07	0,02	0,048
4,75	45668	0	0	0,04	0	0	0,01	0,04	0,02	0,89	0,07	0,89	0	0,04	0,046
4,75	19638	0,02	0	0,02	0	0,9	0	0,06	0	0	0,02	0	0,96	0,02	0,046
4,75	21071	0	0	0,04	0	0	0,03	0	0	0,93	0,12	0,04	0,84	0	0,032
4,75	48145	0	0	0,04	0	0,01	0	0	0,95	0	0,96	0	0,04	0	0,042
4,75	61608	0,04	0,65	0	0	0,31	0	0	0	0	0	0,99	0	0,01	0,048
4,75	78541	0	0,49	0	0	0,39	0	0	0	0,12	0,12	0,04	0,01	0,83	0,044
4,75	55742	0,03	0,39	0	0,01	0,48	0	0	0,09	0	0,03	0,48	0	0,49	0,046
4,75	58962	0	0	0	0,08	0	0,09	0,05	0,78	0	0,08	0	0,46	0,46	0,046

4,75	4503	0,04	0,01	0,02	0	0,52	0,38	0	0	0,03	0,54	0,01	0	0,45	0,044
4,75	17571	0	0,15	0	0	0,81	0	0	0,04	0	0	0	0,8	0,2	0,04
4,75	84517	0	0,01	0,01	0,09	0,41	0	0	0,48	0	0,01	0,02	0,89	0,08	0,04
4,75	42867	0	0	0,04	0,04	0,04	0,03	0	0	0,85	0,16	0,07	0	0,77	0,03
4,75	57529	0	0,03	0,01	0,06	0,12	0,57	0,04	0,02	0,15	0,58	0,11	0,03	0,28	0,048
4,75	28502	0,03	0,02	0,03	0	0,92	0	0	0	0	0,03	0,02	0,95	0	0,044
4,75	24970	0	0,92	0	0	0	0	0	0,08	0	0,08	0,03	0	0,89	0,046
4,75	30465	0,04	0	0	0	0,67	0,05	0,01	0,15	0,08	0	0,09	0,15	0,76	0,048
4,75	23785	0	0	0,04	0	0,77	0,04	0	0	0,15	0	0,21	0,02	0,77	0,004
4,75	13070	0,02	0	0	0,05	0,93	0	0	0	0	0,01	0,93	0,05	0,01	0,048
4,75	41882	0,03	0	0,02	0,03	0,89	0	0	0,02	0,01	0,94	0	0,02	0,04	0,046
4,75	41037	0	0,04	0,01	0,05	0,83	0	0,02	0,05	0	0,87	0,05	0,02	0,06	0,046
4,75	23426	0,01	0,01	0,02	0	0,91	0	0,05	0	0	0,01	0,02	0,96	0,01	0,042
4,75	50693	0,02	0	0,04	0	0,04	0	0,02	0,24	0,64	0	0,3	0,02	0,68	0,036
4,75	44837	0	0	0,04	0	0	0	0	0	0,96	0	0,02	0,02	0,96	0,01
4,75	70007	0	0	0,04	0	0,94	0	0,01	0	0,01	0	0,95	0,01	0,04	0,012
4,75	59261	0,01	0,05	0	0,05	0,89	0	0	0	0	0	0	0,51	0,49	0,048
4,75	52173	0,01	0,1	0	0	0,01	0,79	0,01	0,05	0,03	0,1	0,04	0,86	0	0,048
4,75	31692	0	0	0,04	0	0,95	0,01	0	0	0	0	0	0,95	0,05	0,016
4,75	23258	0,04	0	0,01	0,03	0,46	0	0	0,37	0,09	0,08	0,01	0,82	0,09	0,048
4,75	60046	0	0	0	0,2	0,8	0	0	0	0	0,8	0	0	0,2	0,048
4,75	20647	0	0	0	0,08	0,92	0	0	0	0	0	0,9	0,07	0,03	0,046
4,75	61157	0	0,2	0	0	0,47	0	0	0,33	0	0,19	0,56	0,01	0,24	0,038
4,75	86749	0	0,2	0	0	0,45	0,17	0	0	0,18	0,2	0	0,18	0,62	0,046
4,75	56276	0	0,24	0,04	0	0,65	0,04	0	0	0,03	0	0,89	0,07	0,04	0,044
4,75	72709	0,02	0,01	0,04	0	0	0	0	0,93	0	0,01	0	0,04	0,95	0,026
4,75	18913	0	0	0,04	0	0,01	0,23	0,03	0,01	0,68	0,2	0,08	0,04	0,68	0,042
4,75	90334	0,04	0	0,04	0	0	0	0	0,92	0	0,08	0,02	0,9	0	0,046
4,75	22352	0	0	0	0,06	0,51	0,23	0,13	0	0,07	0,57	0	0,38	0,05	0,048
4,75	75215	0,03	0	0	0,02	0,29	0,66	0	0	0	0,03	0,02	0,29	0,66	0,04
4,75	18130	0	0,96	0,04	0	0	0	0	0	0	0	0	0,04	0,96	0,044
4,75	47790	0,02	0,04	0	0	0	0,86	0,02	0	0,06	0	0	1	0	0,048
4,75	39477	0	0,04	0	0,09	0,87	0	0	0	0	0	0	0,91	0,09	0,048

4,75	26998	0	0	0,04	0	0	0,19	0	0	0,77	0,15	0	0,04	0,81	0,008
4,75	77314	0	0	0	0	0,2	0	0,13	0,12	0,55	0,12	0,55	0,33	0	0,048
4,75	49746	0	0,29	0,01	0	0,7	0	0	0	0	0	0,08	0,3	0,62	0,046
4,75	27182	0	0	0	0,11	0,01	0,84	0	0	0,04	0,1	0,83	0,04	0,03	0,038
4,75	8285	0	0	0,04	0	0	0	0,02	0	0,94	0	0,04	0	0,96	0,03
4,75	65502	0	0	0	0,03	0,16	0	0,03	0,78	0	0,03	0	0,19	0,78	0,046
4,75	41008	0	0,05	0	0,04	0,84	0,07	0	0	0	0,84	0	0,12	0,04	0,036
4,75	22213	0,04	0	0	0,03	0,07	0,01	0	0,85	0	0,01	0,12	0,8	0,07	0,048
4,75	38685	0	0,08	0,03	0,01	0	0,2	0	0,68	0	0,08	0,13	0,7	0,09	0,046
4,75	77799	0,04	0,03	0,01	0	0,44	0	0	0,04	0,44	0,04	0,04	0,44	0,48	0,048
4,75	6234	0	0	0,04	0	0	0,1	0,01	0,2	0,65	0,09	0,12	0	0,79	0,024
4,75	15857	0,03	0	0,02	0,02	0,93	0	0	0	0	0,05	0,93	0	0,02	0,044
4,75	100000	0,05	0	0,94	0	0	0	0	0,01	0	0	0,95	0,05	0	0,572
4,75	44867	0,02	0,03	0,02	0,01	0	0	0	0,92	0	0,91	0,06	0	0,03	0,046
4,75	100000	0	0	0,88	0,01	0	0	0	0	0,11	0,03	0,85	0,01	0,11	0,388
4,75	86912	0	0	0	0,06	0,6	0,01	0,01	0,32	0	0,01	0,92	0,06	0,01	0,046
4,75	48338	0	0,04	0	0,08	0,06	0,82	0	0	0	0,12	0	0,07	0,81	0,042
4,75	6477	0	0,15	0,04	0,01	0	0	0	0,26	0,54	0,15	0,31	0	0,54	0,044
4,75	40095	0,05	0,02	0	0	0,93	0	0	0	0	0,07	0,04	0,04	0,85	0,04
4,75	27667	0	0	0,02	0,11	0	0,84	0	0	0,03	0,08	0,06	0,86	0	0,048
4,75	3286	0	0,01	0	0,1	0,89	0	0	0	0	0	0,01	0,89	0,1	0,032
4,75	63031	0	0	0,04	0	0,19	0,37	0,1	0,3	0	0,41	0,33	0,24	0,02	0,046
4,75	14241	0	0,01	0	0,06	0,92	0,01	0	0	0	0,01	0,93	0,06	0	0,048
4,75	31851	0,04	0	0	0,03	0,91	0,02	0	0	0	0,05	0,01	0,03	0,91	0,044
4,75	18356	0	0	0,04	0	0,96	0	0	0	0	0,93	0,03	0,04	0	0,032
4,75	43525	0,06	0	0,02	0	0,91	0	0	0,01	0	0,07	0	0,02	0,91	0,04
4,75	100000	0,04	0	0	0,47	0,17	0,32	0	0	0	0,43	0,1	0,47	0	0,234
4,75	40008	0,01	0,06	0	0	0	0	0,02	0,91	0	0,09	0,88	0,03	0	0,03
4,75	24212	0	0,09	0,04	0	0	0	0	0	0,87	0	0,04	0,04	0,92	0,036
4,75	6387	0	0	0	0,11	0,82	0,06	0	0,01	0	0	0,01	0,63	0,36	0,048
4,75	31796	0	0	0	0,07	0,93	0	0	0	0	0,07	0,93	0	0	0,044
4,75	50327	0	0,18	0	0	0,81	0	0,01	0	0	0,08	0,74	0,17	0,01	0,046
4,75	10298	0	0,02	0,04	0	0	0	0,03	0,86	0,05	0	0,94	0,05	0,01	0,04

4,75	17879	0,04	0,02	0	0	0,94	0	0	0	0	0,16	0,04	0,78	0,02	0,038
4,75	19389	0	0,13	0	0,03	0,82	0	0,01	0,01	0	0,01	0,01	0,16	0,82	0,044
4,75	100000	0	0	0,98	0	0	0,01	0	0,01	0	0	0,99	0	0,01	0,616
4,75	28162	0	0	0	0,16	0,68	0,15	0	0	0,01	0,31	0,01	0,68	0	0,048
4,75	36822	0,04	0	0,02	0	0,91	0,03	0	0	0	0,93	0,01	0	0,06	0,046
4,75	22246	0,01	0,02	0	0,04	0,92	0	0,01	0	0	0	0	0,05	0,95	0,042
4,75	57950	0	0	0	0,11	0,87	0	0	0	0,02	0	0	0,98	0,02	0,048
4,75	100000	0	0	1	0	0	0	0	0	0	0	1	0	0	0,638
4,75	18406	0	0	0	0,12	0,88	0	0	0	0	0	0,12	0,87	0,01	0,044
4,75	100000	0	0	0,39	0	0	0	0,02	0,17	0,42	0,14	0,39	0,16	0,31	0,176
4,75	42745	0,01	0,1	0,02	0	0,76	0	0	0,1	0,01	0,13	0	0,86	0,01	0,042
4,75	19469	0,04	0	0,03	0	0	0	0	0	0,93	0,05	0,03	0,92	0	0,048
4,75	92497	0	0,17	0	0	0	0,01	0	0,82	0	0	0	0,83	0,17	0,044
4,75	17788	0	0	0	0,12	0,88	0	0	0	0	0	0,12	0,88	0	0,044
4,75	20724	0,02	0,09	0	0	0	0,89	0	0	0	0,1	0	0,9	0	0,048
4,75	100000	0	0	0,94	0	0	0,04	0,02	0	0	0,04	0,94	0	0,02	0,512
4,75	36351	0,06	0,03	0,01	0	0,88	0	0	0,02	0	0,08	0,8	0,01	0,11	0,046
4,75	50158	0	0	0,04	0	0,1	0,8	0,06	0	0	0	0,16	0,8	0,04	0,038
4,75	24248	0,01	0,98	0	0	0	0	0,01	0	0	0,01	0,99	0	0	0,046
5	83164	0,01	0,09	0	0,01	0,88	0	0	0	0,01	0,06	0	0,89	0,05	0,048
5	44378	0	0	0,04	0,02	0,85	0	0	0,03	0,06	0,85	0,03	0,12	0	0,028
5	67146	0	0	0	0,13	0,29	0	0	0,58	0	0,33	0,58	0	0,09	0,04
5	100000	0	0	0,92	0	0	0	0,08	0	0	0,09	0,9	0,01	0	0,668
5	8916	0,05	0	0	0,02	0	0,17	0	0,76	0	0,93	0	0,05	0,02	0,046
5	27656	0	0	0	0,33	0,67	0	0	0	0	0,94	0,06	0	0	0,048
5	17872	0,02	0	0,03	0,05	0,66	0,1	0	0	0,14	0,14	0,53	0,14	0,19	0,048
5	21250	0	0,13	0,04	0	0	0	0	0	0,83	0,13	0,04	0	0,83	0,044
5	13066	0	0	0	0,11	0,88	0	0	0	0,01	0,04	0,36	0,6	0	0,03
5	12123	0	0	0,04	0	0,74	0	0	0,22	0	0,78	0,22	0	0	0,034
5	3263	0	0	0,04	0,06	0	0	0,01	0,04	0,85	0,01	0,08	0,91	0	0,048
5	4288	0	0	0,01	0	0,02	0,01	0,11	0,85	0	0,01	0	0,96	0,03	0,048
5	33134	0	0,09	0,04	0	0	0,41	0,03	0	0,43	0	0,13	0,11	0,76	0,032
5	62200	0,03	0	0,03	0	0,88	0,06	0	0	0	0,9	0,03	0,07	0	0,048

5	9498	0,01	0	0,04	0,06	0,83	0,05	0	0,01	0	0,83	0,05	0,04	0,08	0,048
5	8086	0	0	0,04	0	0	0,68	0	0	0,28	0,6	0,04	0,36	0	0,024
5	7264	0,01	0,2	0	0	0	0,46	0	0,07	0,26	0,2	0,26	0,08	0,46	0,044
5	80224	0,02	0,21	0,02	0	0,75	0	0	0	0	0,21	0,02	0	0,77	0,04
5	33828	0	0,12	0	0	0	0,88	0	0	0	0	0,12	0,88	0	0,036
5	11399	0	0	0,04	0,04	0,71	0	0	0	0,21	0,01	0,01	0,24	0,74	0,042
5	36275	0,01	0	0,04	0,07	0	0	0	0	0,88	0	0,02	0,91	0,07	0,044
5	28603	0	0,12	0,01	0	0,17	0,67	0	0,03	0	0,2	0	0,8	0	0,046
5	24651	0,06	0	0	0,04	0	0	0	0,79	0,11	0,85	0,15	0	0	0,048
5	11018	0	0,03	0,01	0,02	0	0	0,04	0,9	0	0,08	0	0,22	0,7	0,048
5	30239	0	0,49	0	0	0	0,01	0	0,46	0,04	0,07	0,44	0,03	0,46	0,048
5	8121	0,05	0	0	0,01	0,3	0,59	0	0	0,05	0,06	0,64	0,3	0	0,046
5	100000	0,01	0	0,98	0	0,01	0	0	0	0	0,01	0,98	0,01	0	0,614
5	51355	0,01	0,52	0	0	0	0,07	0	0,4	0	0	0,41	0,07	0,52	0,046
5	41403	0	0,28	0	0	0,72	0	0	0	0	0	0	0,28	0,72	0,042
5	45612	0,01	0,13	0	0	0,86	0	0	0	0	0	0,01	0,92	0,07	0,044
5	14913	0	0	0	0,22	0	0	0	0,78	0	0,78	0	0,22	0	0,048
5	6855	0	0	0,02	0,05	0,01	0,26	0	0	0,66	0,01	0	0,27	0,72	0,042
5	9754	0,05	0	0	0	0,73	0	0,01	0	0,21	0,22	0,05	0,73	0	0,046
5	100000	0,12	0	0,88	0	0	0	0	0	0	0,12	0,88	0	0	0,894
5	11292	0	0	0,03	0	0,01	0,01	0,06	0,87	0,02	0	0,03	0,08	0,89	0,04
5	32226	0	0	0,04	0	0	0,96	0	0	0	0	0,03	0,01	0,96	0,004
5	73161	0,01	0,04	0,04	0,03	0,8	0,06	0	0,02	0	0,03	0,06	0,89	0,02	0,044
5	90455	0,03	0	0,03	0	0,69	0	0,03	0,22	0	0,25	0,03	0,69	0,03	0,04
5	72768	0	0	0,04	0	0,01	0,18	0	0,77	0	0,86	0,03	0	0,11	0,036
5	38836	0	0	0,04	0	0,95	0	0	0	0,01	0,52	0,03	0,45	0	0,024
5	21481	0	0	0,02	0	0,87	0	0,11	0	0	0	0	0,97	0,03	0,046
5	22405	0	0	0,02	0,01	0	0	0,09	0	0,88	0,09	0,02	0,76	0,13	0,046
5	35685	0	0,29	0	0	0,71	0	0	0	0	0,29	0	0	0,71	0,048
5	47910	0	0,01	0,03	0,16	0	0,51	0	0,28	0,01	0,66	0,05	0,28	0,01	0,046
5	22113	0,06	0	0,02	0	0,11	0	0	0,81	0	0,01	0,12	0,04	0,83	0,042
5	88033	0,02	0	0,03	0	0,9	0,01	0,04	0	0	0,9	0,03	0,04	0,03	0,048
5	21657	0,05	0	0,04	0	0,83	0,08	0	0	0	0,05	0,23	0,08	0,64	0,046

5	32104	0,03	0,09	0	0	0,06	0,65	0	0,17	0	0,05	0,05	0,64	0,26	0,048
5	73657	0,01	0,04	0	0,09	0	0,86	0	0	0	0,9	0,01	0	0,09	0,048
5	100000	0,11	0	0,41	0,07	0	0	0	0,41	0	0,53	0,02	0,44	0,01	0,424
5	38848	0	0,1	0	0	0	0,22	0	0,6	0,08	0,61	0,08	0,1	0,21	0,042
5	27029	0,03	0,06	0,02	0	0	0,51	0	0	0,38	0	0,11	0,51	0,38	0,042
5	25181	0,03	0	0,04	0,02	0,43	0	0	0,48	0	0,04	0,13	0,4	0,43	0,046
5	25167	0	0	0,02	0,17	0,81	0	0	0	0	0,83	0	0,17	0	0,048
5	31919	0	0	0	0,03	0,84	0	0,1	0,03	0	0,1	0	0,9	0	0,048
5	49399	0	0,01	0,03	0,06	0,79	0,03	0	0,08	0	0,03	0,83	0,08	0,06	0,046
5	12001	0	0,08	0	0,01	0,91	0	0	0	0	0,92	0,01	0	0,07	0,048
5	62740	0	0,09	0,04	0	0,16	0	0	0,71	0	0	0,11	0,76	0,13	0,046
5	24608	0,02	0,08	0	0	0,17	0,69	0	0,04	0	0,71	0,08	0,04	0,17	0,042
5	15346	0,01	0	0	0	0,03	0,23	0,07	0	0,66	0,08	0	0,14	0,78	0,046
5	19451	0	0	0	0,12	0,38	0,03	0	0,47	0	0,03	0	0,88	0,09	0,048
5	13978	0	0	0	0,16	0,83	0	0	0	0,01	0,83	0	0	0,17	0,046
5	16743	0,01	0	0,01	0	0,03	0	0,08	0,87	0	0	0,01	0,91	0,08	0,03
5	4370	0,05	0	0	0	0	0	0	0,95	0	0,05	0	0	0,95	0,048
5	48152	0	0,07	0,01	0,01	0,88	0	0,02	0,01	0	0,95	0,02	0,03	0	0,048
5	49111	0	0,01	0	0,08	0,88	0	0,01	0	0,02	0,02	0,01	0,91	0,06	0,048
5	41184	0	0	0,04	0,06	0,09	0,52	0,01	0	0,28	0	0,61	0,17	0,22	0,046
5	18958	0	0	0,02	0,09	0,56	0,03	0	0,3	0	0,26	0,58	0,13	0,03	0,048
5	28143	0	0,1	0	0,01	0,87	0	0,02	0	0	0	0	0,97	0,03	0,044
5	14483	0,01	0,1	0	0,04	0,71	0	0	0,14	0	0,33	0	0,01	0,66	0,048
5	9320	0	0	0	0,23	0,76	0,01	0	0	0	0,76	0,23	0,01	0	0,048
5	25675	0,02	0	0	0,09	0	0,89	0	0	0	0,02	0,89	0,09	0	0,046
5	5394	0	0	0,04	0	0	0,68	0	0	0,28	0,28	0,04	0	0,68	0,01
5	47712	0,01	0,06	0,01	0,03	0	0	0	0,12	0,77	0,03	0,07	0,58	0,32	0,044
5	16312	0,04	0,06	0	0	0,11	0	0	0,79	0	0,04	0	0,11	0,85	0,044
5	100000	0	0	0,99	0	0,01	0	0	0	0	0	0,99	0,01	0	0,578
5	42287	0	0,12	0	0,05	0,48	0	0	0,35	0	0,04	0	0,48	0,48	0,046
5	14135	0	0	0,04	0,01	0	0	0,01	0,93	0,01	0	0,06	0,09	0,85	0,02
5	59983	0	0,01	0,04	0	0,18	0,01	0	0,12	0,64	0	0,34	0,02	0,64	0,01
5	35824	0	0	0,04	0,05	0	0	0	0	0,91	0	0,04	0	0,96	0,038

5	48068	0,06	0	0	0	0,87	0	0	0,07	0	0,1	0,87	0,03	0	0,046
5	61213	0,04	0	0,04	0	0,9	0	0	0,02	0	0,04	0,04	0,92	0	0,048
5	14934	0	0	0	0,16	0,66	0,1	0	0	0,08	0,66	0,12	0,16	0,06	0,048
5	57682	0	0,07	0	0,05	0,87	0	0,01	0	0	0	0,05	0,94	0,01	0,048
5	47326	0	0	0,04	0	0	0	0	0	0,96	0	0,96	0	0,04	0,022
5	14768	0,05	0	0	0,01	0,16	0	0	0,78	0	0,78	0,11	0,05	0,06	0,044
5	11051	0,03	0	0,01	0,05	0,81	0,05	0	0	0,05	0	0	0,95	0,05	0,048
5	21973	0	0,01	0,04	0	0	0	0	0,01	0,94	0	0,06	0	0,94	0,01
5	26756	0	0	0,04	0	0,01	0,73	0	0,22	0	0,22	0,01	0,04	0,73	0,008
5	16235	0,04	0	0,04	0	0,24	0,01	0	0,66	0,01	0,01	0,04	0,28	0,67	0,048
5	29139	0	0	0,04	0	0	0	0	0	0,96	0	0,04	0	0,96	0,01
5	50106	0	0	0,04	0,04	0,87	0	0,05	0	0	0,87	0,09	0	0,04	0,046
5	23421	0	0,07	0	0	0,73	0	0,05	0,15	0	0,17	0,1	0,15	0,58	0,048
5	36422	0	0	0	0,07	0,93	0	0	0	0	0,07	0,93	0	0	0,044
5	15742	0,01	0,95	0,04	0	0	0	0	0	0	0	0,01	0,04	0,95	0,046
5	9786	0	0	0,04	0	0	0	0	0,95	0,01	0,04	0,01	0	0,95	0,004
5	53612	0,02	0	0,03	0,05	0,9	0	0	0	0	0,02	0,94	0	0,04	0,048
5	35736	0	0	0,04	0	0	0,04	0,08	0	0,84	0,03	0,09	0,84	0,04	0,048
5	25516	0	0	0,04	0	0,52	0,43	0	0	0,01	0,52	0,04	0,44	0	0,03
5	17791	0	0,02	0,04	0	0	0,65	0	0,29	0	0,02	0,65	0,29	0,04	0,034
	38291,2	0,01344	0,07256	0,07521	0,02733	0,41383	0,12457	0,01035	0,16995	0,092776	0,19905	0,242765	0,280065	0,278118	0,078204
scoring															
b	time_to_co	(C,C)	(C,D)	(C,P)	(D,C)	(D,D)	(D,P)	(P,C)	(P,D)	(P,P)	S(G,G)	S(G,B)	S(B,G)	S(B,B)	
1	7358	0	0,13	0	0,03	0,73	0	0	0,11	0	0,13	0,85	0,02	0	0,048
1	14119	0,01	0	0,04	0,02	0,93	0	0	0	0	0,01	0,04	0,95	0	0,04
1	9127	0,06	0	0	0,03	0	0,17	0	0,74	0	0,77	0	0	0,23	0,048
1	24654	0	0,99	0	0	0,01	0	0	0	0	0	0,99	0	0,01	0,044
1	28129	0,01	0	0	0,04	0,71	0	0,06	0,18	0	0,24	0	0,76	0	0,048
1	5163	0,05	0	0,03	0	0,92	0	0	0	0	0	0,08	0,91	0,01	0,044
1	9139	0,01	0	0,04	0	0,95	0	0	0	0	0	0,04	0,96	0	0,032
1	3566	0,03	0,01	0	0	0,96	0	0	0	0	0,01	0	0,98	0,01	0,048
1	21476	0,03	0,02	0	0	0,88	0,01	0,02	0,04	0	0,09	0,02	0	0,89	0,042
1	10819	0	0,19	0	0	0,78	0	0,01	0,02	0	0,15	0,84	0,01	0	0,042

1	35873	0	0	0	0,06	0,94	0	0	0	0	0	0	0,07	0,93	0,042
1	28019	0	0	0	0,12	0,88	0	0	0	0	0,12	0	0,88	0	0,048
1	4879	0,04	0	0,01	0	0,93	0	0,02	0	0	0,04	0	0,93	0,03	0,048
1	17112	0,05	0	0	0	0,87	0	0,01	0,07	0	0	0,09	0,83	0,08	0,046
1	13008	0	0,03	0,01	0,06	0,88	0	0	0,02	0	0,05	0,01	0	0,94	0,048
1	8779	0,04	0	0	0	0,01	0,1	0,05	0,8	0	0,8	0	0,09	0,11	0,044
1	12472	0	0	0,02	0,07	0,9	0,01	0	0	0	0,07	0,06	0,03	0,84	0,048
1	17568	0,03	0	0	0,03	0	0,01	0	0,93	0	0	0,02	0,03	0,95	0,044
1	36020	0	0	0	0,08	0,92	0	0	0	0	0,2	0,77	0,03	0	0,046
1	26018	0,01	0,96	0	0	0	0,01	0,02	0	0	0	0,01	0,02	0,97	0,032
1	9989	0	0,15	0,02	0,06	0,04	0	0	0,73	0	0	0,06	0	0,94	0,048
1	10760	0,03	0	0	0	0,81	0,01	0,15	0	0	0,96	0,03	0	0,01	0,048
1	16116	0	0	0	0,08	0,92	0	0	0	0	0,19	0,81	0	0	0,046
1	43710	0,01	0,94	0,01	0	0	0,01	0,01	0,02	0	0,02	0,03	0,01	0,94	0,044
1	14761	0,06	0	0	0	0	0	0	0,49	0,45	0	0,51	0,49	0	0,044
1	32837	0,06	0	0,02	0	0	0	0	0,91	0,01	0,02	0,05	0,91	0,02	0,042
1	21005	0,06	0	0	0	0	0	0	0,94	0	0,61	0,33	0	0,06	0,042
1	4097	0	0,01	0,01	0,1	0,88	0	0	0	0	0	0,01	0,98	0,01	0,04
1	5890	0,02	0	0,03	0	0	0,09	0,09	0,77	0	0,81	0,08	0,11	0	0,048
1	7347	0	0	0	0,06	0,94	0	0	0	0	0	0,03	0,03	0,94	0,042
1	24352	0	0,06	0,03	0	0	0,91	0	0	0	0,09	0	0,91	0	0,048
1	20996	0	0	0	0,05	0	0,26	0	0,69	0	0,01	0	0,05	0,94	0,042
1	6355	0,05	0	0,04	0	0	0	0	0,9	0,01	0,01	0,94	0,04	0,01	0,048
1	10374	0,05	0,03	0,01	0	0,9	0,01	0	0	0	0	0,04	0,1	0,86	0,048
1	9771	0	0,24	0	0	0,75	0	0	0,01	0	0,01	0,44	0,31	0,24	0,048
1	2753	0,03	0,65	0,03	0	0,29	0	0	0	0	0,04	0,03	0	0,93	0,046
1	9952	0,04	0	0,01	0	0,95	0	0	0	0	0,56	0,01	0,43	0	0,048
1	18580	0,06	0	0	0	0,92	0	0	0,01	0,01	0,01	0	0,17	0,82	0,046
1	29557	0	0,13	0	0,02	0,81	0,01	0	0	0,03	0,12	0,81	0,06	0,01	0,046
1	11640	0	0,06	0	0,01	0,09	0	0	0,83	0,01	0,84	0,1	0,06	0	0,046
1	5088	0	0,6	0,04	0	0,36	0	0	0	0	0	0	0,08	0,92	0,046
1	31879	0,06	0	0	0	0,9	0,02	0	0	0,02	0,08	0,4	0,52	0	0,048
1	8054	0,01	0	0,04	0	0,04	0	0	0,91	0	0,04	0,94	0,02	0	0,022

1	10934	0,04	0	0,03	0	0,91	0,02	0	0	0	0,95	0,04	0	0,01	0,048
1	11611	0,05	0,18	0	0	0,72	0,05	0	0	0	0,05	0,18	0,01	0,76	0,042
1	19299	0	0	0	0,24	0,76	0	0	0	0	0,76	0,24	0	0	0,048
1	13608	0	0,26	0	0,01	0,7	0	0	0,03	0	0,01	0,01	0,25	0,73	0,046
1	27590	0	0	0,02	0,15	0,83	0	0	0	0	0,83	0,17	0	0	0,03
1	28763	0	0,08	0	0	0,01	0,89	0	0	0,02	0,99	0,01	0	0	0,046
1	9052	0,01	0	0,01	0	0,93	0	0,05	0	0	0	0,93	0,01	0,06	0,048
1	49685	0	0	0	0,24	0,76	0	0	0	0	0,76	0,07	0,17	0	0,044
1	15259	0	0,14	0	0	0	0,01	0	0,83	0,02	0,02	0	0,98	0	0,048
1	31803	0,06	0	0	0	0	0,01	0	0,93	0	0	0,93	0	0,07	0,048
1	5665	0,02	0	0	0,02	0,94	0,01	0,01	0	0	0,02	0,01	0,01	0,96	0,048
1	9645	0,04	0,92	0	0	0,02	0	0	0,02	0	0	0,02	0	0,98	0,044
1	17250	0	0,19	0	0	0,66	0	0	0,15	0	0,15	0	0,66	0,19	0,048
1	5290	0,02	0,05	0	0	0,92	0	0,01	0	0	0,92	0	0,08	0	0,044
1	30412	0,02	0,75	0	0	0	0	0	0,23	0	0	0	0,05	0,95	0,048
1	18090	0,05	0	0	0,02	0,88	0	0	0,05	0	0,66	0,26	0,02	0,06	0,046
1	14482	0,05	0,01	0,03	0	0	0,1	0	0,81	0	0,04	0,82	0,04	0,1	0,048
1	11154	0,04	0	0,01	0	0,01	0,94	0	0	0	0	0,01	0,94	0,05	0,032
1	10325	0,02	0,9	0,04	0	0,03	0	0	0,01	0	0,04	0,04	0	0,92	0,046
1	11857	0,02	0	0	0,07	0,69	0,13	0	0,09	0	0,28	0,43	0,09	0,2	0,046
1	26082	0,03	0,03	0	0,02	0,83	0,09	0	0	0	0,02	0	0,86	0,12	0,048
1	45880	0	0	0,04	0,02	0,89	0	0,04	0,01	0	0,04	0,01	0,95	0	0,044
1	37990	0,05	0	0,01	0	0	0,42	0,02	0,49	0,01	0,49	0,03	0,01	0,47	0,046
1	14349	0	0,94	0	0	0	0	0	0,06	0	0	0,94	0	0,06	0,028
1	7909	0	0,16	0	0	0,83	0	0	0	0,01	0	0,01	0,99	0	0,046
1	4179	0	0,22	0,02	0	0,76	0	0	0	0	0	0,78	0,22	0	0,048
1	6253	0	0,11	0,03	0,02	0	0,37	0	0,47	0	0,03	0,15	0,62	0,2	0,048
1	18960	0,06	0	0	0	0,93	0,01	0	0	0	0,01	0,06	0	0,93	0,048
1	19396	0	0	0	0,19	0,76	0,05	0	0	0	0,76	0,01	0,23	0	0,044
1	59185	0,06	0	0	0	0,84	0	0	0,09	0,01	0,84	0,07	0,01	0,08	0,048
1	5075	0	1	0	0	0	0	0	0	0	0	1	0	0	0,034
1	19031	0	0,95	0	0	0,03	0	0	0,02	0	0	0,6	0,03	0,37	0,034
1	42248	0,05	0	0,01	0	0,85	0	0	0,09	0	0	0,14	0,01	0,85	0,046

1	17124	0	0,03	0	0,06	0,86	0,04	0	0,01	0	0,03	0,87	0,09	0,01	0,04
1	27989	0,06	0	0	0	0,73	0,2	0,01	0	0	0,97	0	0,02	0,01	0,046
1	25779	0	0,16	0	0	0,84	0	0	0	0	0,04	0,17	0,46	0,33	0,032
1	12521	0	0,01	0	0,11	0,88	0	0	0	0	0,08	0	0,89	0,03	0,048
1	13949	0	0,05	0	0,01	0,65	0,01	0,01	0,26	0,01	0,92	0,02	0,01	0,05	0,038
1	14173	0	0,28	0,02	0	0,7	0	0	0	0	0,28	0,02	0,01	0,69	0,038
1	11378	0,03	0,17	0	0	0,8	0	0	0	0	0,11	0,03	0	0,86	0,04
1	9832	0	0,99	0	0	0	0	0	0,01	0	0	0,99	0	0,01	0,036
1	17551	0,05	0	0	0	0	0,46	0	0,49	0	0,39	0	0,56	0,05	0,046
1	34725	0,01	0,08	0,01	0	0,8	0,09	0	0	0,01	0,17	0,01	0,8	0,02	0,038
1	12119	0	0,1	0	0,04	0,02	0,01	0	0,83	0	0,13	0,83	0	0,04	0,046
1	16512	0	0,09	0	0	0,9	0	0	0	0,01	0,9	0	0	0,1	0,048
1	12227	0,05	0	0	0,02	0,93	0	0	0	0	0,97	0,02	0	0,01	0,048
1	20568	0,01	0	0,04	0	0	0	0	0,95	0	0,07	0,89	0	0,04	0,016
1	15190	0	0,03	0	0,09	0,82	0,04	0	0,01	0,01	0	0,16	0,82	0,02	0,036
1	15328	0,05	0	0,03	0	0,91	0,01	0	0	0	0	0,91	0,05	0,04	0,048
1	18642	0,05	0	0	0,01	0,9	0,01	0,03	0	0	0,92	0,05	0,03	0	0,048
1	11514	0	0	0,01	0,11	0	0	0	0,88	0	0,21	0,68	0	0,11	0,048
1	26198	0,05	0,01	0	0	0,61	0,33	0	0	0	0,38	0	0,33	0,29	0,048
1	9383	0	0,14	0	0,01	0,46	0,36	0	0,03	0	0,01	0,37	0,6	0,02	0,048
1	6083	0,03	0	0,04	0	0,86	0	0	0,06	0,01	0	0,94	0,03	0,03	0,034
1	27967	0	0,03	0	0,11	0,86	0	0	0	0	0,89	0	0,11	0	0,048
1	42558	0	0	0	0,28	0,72	0	0	0	0	1	0	0	0	0,046
1	12515	0,05	0,03	0	0	0,26	0	0	0,66	0	0,29	0	0	0,71	0,046
1,25	12594	0	0,07	0,01	0	0,91	0	0,01	0	0	0,92	0	0	0,08	0,048
1,25	3933	0,04	0,08	0,04	0,01	0	0	0	0,83	0	0,09	0,04	0	0,87	0,048
1,25	50183	0	0	0	0,07	0,93	0	0	0	0	0	0	0,05	0,95	0,048
1,25	5954	0,02	0,04	0,04	0	0	0	0	0,89	0,01	0,2	0,75	0,04	0,01	0,046
1,25	29302	0,06	0	0	0	0,18	0,64	0	0,11	0,01	0,73	0,06	0,06	0,15	0,048
1,25	36265	0,01	0,94	0	0	0,05	0	0	0	0	0	0,01	0,05	0,94	0,04
1,25	8351	0,05	0,01	0	0	0	0	0	0,94	0	0,06	0	0,05	0,89	0,048
1,25	35527	0	0	0	0,16	0,81	0,03	0	0	0	0,8	0	0,16	0,04	0,044
1,25	23324	0	0	0,02	0,09	0,87	0	0,02	0	0	0,11	0	0,89	0	0,048

1,25	13599	0,03	0	0,04	0	0	0	0	0,93	0	0,91	0,03	0,05	0,01	0,048
1,25	8578	0,02	0	0,02	0,01	0	0	0,02	0,93	0	0	0,6	0,39	0,01	0,044
1,25	5883	0,03	0,27	0	0	0,69	0,01	0	0	0	0	0,96	0	0,04	0,044
1,25	11136	0	0,08	0	0	0,83	0	0,04	0,03	0,02	0,84	0	0,12	0,04	0,046
1,25	55347	0,01	0,23	0,01	0	0,75	0	0	0	0	0,23	0	0,01	0,76	0,048
1,25	10525	0,03	0	0,04	0	0	0,04	0,02	0,87	0	0	0,06	0,03	0,91	0,044
1,25	14577	0,04	0	0	0,07	0,89	0	0	0	0	0,89	0,02	0	0,09	0,046
1,25	21050	0	0,04	0,02	0,07	0,87	0	0	0	0	0,06	0,72	0,22	0	0,046
1,25	23927	0	0,18	0,01	0	0,46	0,35	0	0	0	0	0,64	0,35	0,01	0,042
1,25	24734	0	0	0,01	0,3	0,69	0	0	0	0	0,99	0	0	0,01	0,048
1,25	7052	0,05	0	0	0	0	0,26	0	0,69	0	0,92	0	0,03	0,05	0,04
1,25	32210	0,03	0	0	0,05	0,92	0	0	0	0	0,61	0,03	0	0,36	0,046
1,25	62778	0,01	0,16	0,01	0	0,82	0	0	0	0	0,15	0,82	0,02	0,01	0,046
1,25	24697	0,02	0	0	0,04	0,94	0	0	0	0	0	0,06	0,06	0,88	0,048
1,25	9886	0,05	0	0	0	0,95	0	0	0	0	0,95	0	0	0,05	0,042
1,25	4292	0,02	0,02	0	0	0,91	0	0,05	0	0	0	0,93	0,07	0	0,048
1,25	16305	0,02	0,22	0	0	0,71	0,03	0,01	0	0,01	0	0,96	0,02	0,02	0,048
1,25	26018	0	0,01	0	0,21	0,78	0	0	0	0	0,94	0,05	0,01	0	0,042
1,25	20971	0	0	0	0,11	0,84	0,04	0	0,01	0	0,05	0,42	0,53	0	0,048
1,25	8322	0,03	0,01	0	0	0,93	0	0,03	0	0	0,93	0,06	0	0,01	0,048
1,25	14012	0	0,02	0	0	0,6	0,07	0,09	0,14	0,08	0,1	0,8	0,04	0,06	0,048
1,25	32137	0	0	0,04	0	0	0	0	0,96	0	0	0,01	0	0,99	0
1,25	24615	0	0	0	0,05	0,93	0	0	0	0,02	0,07	0	0	0,93	0,04
1,25	71771	0	0	0	0,11	0,86	0,01	0,01	0	0,01	0	0	0,97	0,03	0,048
1,25	15168	0,05	0	0,03	0	0,9	0	0,01	0	0,01	0,01	0,08	0,03	0,88	0,048
1,25	28347	0	0	0	0,12	0,88	0	0	0	0	0,11	0	0,89	0	0,048
1,25	22853	0	0	0	0,1	0,9	0	0	0	0	0	0	1	0	0,04
1,25	34450	0	0	0	0,06	0,94	0	0	0	0	0,06	0	0	0,94	0,044
1,25	9426	0,01	0,06	0,01	0	0	0,92	0	0	0	0,98	0,01	0	0,01	0,048
1,25	14048	0	0,11	0	0	0,02	0,78	0	0	0,09	0	0,11	0,89	0	0,046
1,25	8940	0,02	0	0	0,1	0,73	0,05	0,02	0,08	0	0,63	0,27	0,1	0	0,048
1,25	3604	0,01	0	0	0,13	0,86	0	0	0	0	0,58	0,42	0	0	0,048
1,25	47595	0,01	0,01	0,01	0,16	0	0	0	0,81	0	0,81	0,01	0,16	0,02	0,044

1,25	12049	0,04	0	0	0,01	0	0	0	0,95	0	0	0,28	0	0,72	0,044
1,25	6455	0,04	0,05	0	0	0,89	0,02	0	0	0	0	0,07	0,93	0	0,048
1,25	38865	0	0	0	0,11	0,89	0	0	0	0	0,11	0	0,45	0,44	0,046
1,25	12138	0	0,08	0	0	0,92	0	0	0	0	0,92	0	0,08	0	0,048
1,25	16256	0	0	0	0,05	0,7	0,09	0,03	0,13	0	0,05	0,13	0,09	0,73	0,048
1,25	12746	0,07	0	0	0	0,83	0,09	0	0,01	0	0	0,17	0,83	0	0,048
1,25	15856	0	0	0,01	0,1	0,88	0,01	0	0	0	0,01	0	0,88	0,11	0,044
1,25	8918	0,05	0	0	0	0,95	0	0	0	0	0	0,95	0,03	0,02	0,042
1,25	14172	0,03	0,02	0,04	0,02	0,89	0	0	0	0	0,01	0,07	0	0,92	0,044
1,25	5577	0,02	0,11	0	0	0,87	0	0	0	0	0	0	0,87	0,13	0,048
1,25	22137	0,04	0	0,02	0	0,88	0,03	0,03	0	0	0,83	0,14	0	0,03	0,04
1,25	3292	0,06	0	0	0	0,94	0	0	0	0	0	0	0	1	0,046
1,25	23492	0,03	0	0	0,02	0,94	0,01	0	0	0	0	0,03	0,01	0,96	0,048
1,25	25241	0	0	0	0,06	0,93	0	0	0,01	0	0,01	0,06	0,06	0,87	0,048
1,25	10283	0,06	0	0	0	0,91	0	0	0,03	0	0	0,06	0	0,94	0,048
1,25	7820	0,02	0	0	0,07	0,91	0	0	0	0	0,07	0	0,93	0	0,048
1,25	10735	0	0,44	0	0,01	0,54	0	0,01	0	0	0,08	0,47	0	0,45	0,048
1,25	9985	0,03	0	0,02	0	0,95	0	0	0	0	0,97	0	0,03	0	0,044
1,25	14089	0,05	0	0	0,01	0,94	0	0	0	0	0	0,06	0,94	0	0,044
1,25	23744	0,07	0	0	0	0	0,93	0	0	0	0	0	1	0	0,048
1,25	21228	0,01	0,01	0	0,08	0,76	0	0	0,14	0	0,02	0,01	0,89	0,08	0,048
1,25	13598	0	0,18	0	0,04	0,78	0	0	0	0	0,04	0	0,18	0,78	0,044
1,25	8118	0	0	0	0,07	0	0	0,02	0,91	0	0	0,92	0,07	0,01	0,046
1,25	8360	0	0	0	0,13	0	0	0	0,87	0	0	0	0,87	0,13	0,048
1,25	23114	0,06	0	0	0	0,9	0	0	0,01	0,03	0,9	0	0,07	0,03	0,048
1,25	8839	0	0,09	0	0	0,86	0,04	0	0	0,01	0,86	0	0,13	0,01	0,042
1,25	3205	0	0,01	0,04	0	0,68	0	0	0,27	0	0,27	0,02	0,02	0,69	0,02
1,25	7229	0,02	0,96	0	0,02	0	0	0	0	0	0	0,04	0	0,96	0,046
1,25	14577	0	0,95	0	0,04	0	0,01	0	0	0	0	0,29	0	0,71	0,046
1,25	28139	0,04	0,96	0	0	0	0	0	0	0	0	0,04	0	0,96	0,042
1,25	7750	0	0	0	0,08	0,92	0	0	0	0	0	0	0,24	0,76	0,048
1,25	8375	0,06	0,01	0	0	0,93	0	0	0	0	0,94	0	0,06	0	0,046
1,25	10932	0	0,03	0	0,08	0,89	0	0	0	0	0,04	0,84	0,06	0,06	0,044

1,25	22287	0	0	0	0,14	0,86	0	0	0	0	0,14	0	0,86	0	0,048
1,25	40489	0,05	0	0	0	0,03	0,72	0	0	0,2	0,75	0,02	0,23	0	0,036
1,25	7538	0	0,98	0	0	0	0	0	0,02	0	0	0,02	0,07	0,91	0,042
1,25	15140	0,05	0	0,01	0	0,93	0,01	0	0	0	0,05	0,01	0,91	0,03	0,046
1,25	5661	0	0,17	0,04	0	0	0	0	0,79	0	0	0,21	0	0,79	0,01
1,25	16617	0	0,93	0	0	0,01	0,05	0,01	0	0	0,03	0,01	0,02	0,94	0,046
1,25	19153	0,05	0	0,04	0	0	0,91	0	0	0	0,07	0	0,88	0,05	0,044
1,25	26813	0,04	0,01	0	0	0	0	0	0,95	0	0	0,07	0,93	0	0,044
1,25	12056	0,01	0	0,02	0,15	0,79	0	0,03	0	0	0,94	0,02	0,03	0,01	0,048
1,25	4909	0,05	0	0,03	0	0,86	0,04	0	0,02	0	0	0,07	0,86	0,07	0,044
1,25	36379	0,04	0	0	0,01	0,95	0	0	0	0	0,04	0,01	0	0,95	0,048
1,25	7625	0,04	0,03	0	0	0,89	0,01	0	0,03	0	0,02	0,04	0,01	0,93	0,046
1,25	14175	0	0,07	0	0,06	0,83	0	0	0	0,04	0	0,17	0	0,83	0,048
1,25	28881	0	0	0	0,08	0	0,92	0	0	0	0	0	0,08	0,92	0,046
1,25	21558	0	0	0	0,04	0,17	0	0,01	0,78	0	0	0	0,01	0,99	0,046
1,25	11229	0,04	0,03	0	0	0,93	0	0	0	0	1	0	0	0	0,042
1,25	17405	0	0,12	0,03	0	0,75	0,02	0	0,08	0	0,12	0	0,43	0,45	0,044
1,25	27806	0,05	0,04	0	0	0,7	0	0	0,21	0	0	0,79	0	0,21	0,04
1,25	14684	0,02	0	0,02	0,07	0	0	0	0,87	0,02	0,02	0,02	0,89	0,07	0,048
1,25	25104	0,06	0,08	0	0	0,68	0	0	0,18	0	0,2	0	0,09	0,71	0,044
1,25	53867	0,06	0	0,01	0	0,93	0	0	0	0	0	0,94	0	0,06	0,042
1,25	10436	0,03	0	0,03	0	0,94	0	0	0	0	0,94	0,03	0	0,03	0,044
1,25	10396	0	0	0	0,24	0,67	0,08	0	0	0,01	0,71	0,2	0,09	0	0,048
1,25	8082	0	0	0,04	0	0,01	0	0,01	0,94	0	0	0,04	0,29	0,67	0,022
1,25	11085	0,02	0	0,04	0	0,94	0	0	0	0	0,01	0,03	0,96	0	0,042
1,5	24339	0,05	0	0,02	0	0,89	0	0,01	0,01	0,02	0,09	0,86	0	0,05	0,048
1,5	8995	0,05	0	0	0	0,95	0	0	0	0	0,72	0,28	0	0	0,04
1,5	10380	0	0	0,03	0,13	0,79	0,05	0	0	0	0,92	0	0,08	0	0,048
1,5	2906	0,04	0,04	0	0	0,92	0	0	0	0	0,04	0	0,92	0,04	0,042
1,5	14737	0,04	0,96	0	0	0	0	0	0	0	0	0,12	0	0,88	0,044
1,5	8450	0,03	0	0	0,02	0,95	0	0	0	0	0,02	0	0,04	0,94	0,042
1,5	4852	0,04	0,02	0,03	0	0,77	0	0	0,14	0	0,78	0,15	0,04	0,03	0,048
1,5	13673	0,04	0,02	0,01	0,01	0	0	0,01	0,9	0,01	0,02	0	0,02	0,96	0,048

1,5	53072	0	0,08	0	0	0,9	0	0	0,01	0,01	0,91	0	0,08	0,01	0,044
1,5	6913	0	0	0	0,11	0,89	0	0	0	0	0	0,08	0,81	0,11	0,048
1,5	4243	0,02	0	0,04	0,01	0,9	0	0	0,03	0	0,04	0,04	0,9	0,02	0,042
1,5	52834	0,04	0	0	0,02	0,8	0	0	0,14	0	0,04	0,15	0,03	0,78	0,048
1,5	16273	0,02	0,14	0,01	0	0,24	0,01	0	0,58	0	0,24	0,01	0,14	0,61	0,04
1,5	32232	0	0	0,04	0	0	0	0	0,96	0	0	0,04	0	0,96	0
1,5	14788	0	0,25	0	0	0,69	0,06	0	0	0	0	0,75	0,15	0,1	0,042
1,5	28544	0,05	0	0,01	0	0,94	0	0	0	0	0,99	0,01	0	0	0,044
1,5	11279	0,05	0,01	0	0	0,94	0	0	0	0	0	0	0,97	0,03	0,04
1,5	18408	0,03	0	0	0,02	0,95	0	0	0	0	0	0,46	0	0,54	0,036
1,5	18388	0	1	0	0	0	0	0	0	0	0	1	0	0	0,016
1,5	18775	0,06	0	0	0	0,02	0,92	0	0	0	0,98	0	0,02	0	0,044
1,5	13957	0,05	0	0	0	0,95	0	0	0	0	0	1	0	0	0,044
1,5	32423	0,04	0	0,01	0	0,93	0	0,02	0	0	0,02	0,93	0,01	0,04	0,048
1,5	25651	0	0,03	0,01	0	0,01	0,82	0,11	0,02	0	0,65	0,14	0,21	0	0,046
1,5	12538	0	0	0	0,24	0,72	0,03	0	0,01	0	0,9	0	0,03	0,07	0,042
1,5	12538	0,02	0,03	0	0	0,94	0,01	0	0	0	0,97	0	0	0,03	0,042
1,5	5943	0,03	0	0,04	0	0,84	0	0,02	0,07	0	0,02	0,11	0,85	0,02	0,046
1,5	9464	0,04	0,01	0	0	0,66	0,24	0	0,05	0	0,01	0,04	0,92	0,03	0,046
1,5	7647	0,03	0	0,04	0,02	0	0,51	0,01	0,39	0	0,04	0,05	0,4	0,51	0,046
1,5	17544	0	0	0	0	0,5	0	0,06	0	0,44	0,06	0	0	0,94	0,048
1,5	12714	0	0,21	0	0	0,79	0	0	0	0	0	0,79	0,21	0	0,046
1,5	7239	0,01	0,01	0,03	0,1	0	0,76	0	0,02	0,07	0,77	0,06	0,14	0,03	0,04
1,5	22802	0,02	0,04	0	0,02	0	0,86	0	0	0,06	0,94	0	0,06	0	0,042
1,5	7579	0	0,12	0	0	0,88	0	0	0	0	0,12	0	0,88	0	0,048
1,5	16979	0,01	0	0,01	0,11	0	0,84	0	0,03	0	0,28	0,04	0,68	0	0,044
1,5	23402	0,01	0,07	0	0	0,91	0,01	0	0	0	0,82	0,11	0	0,07	0,038
1,5	5074	0,05	0	0,03	0	0,67	0,11	0	0,14	0	0,03	0,08	0,4	0,49	0,044
1,5	8278	0	0,13	0	0	0,87	0	0	0	0	0	0,13	0,87	0	0,042
1,5	10996	0,02	0,97	0	0	0,01	0	0	0	0	0	0	0,03	0,97	0,046
1,5	18072	0	0	0,04	0	0,96	0	0	0	0	0	0,04	0,96	0	0,028
1,5	9638	0,01	0,03	0	0,04	0,9	0,02	0	0	0	0,04	0,01	0,04	0,91	0,048
1,5	15759	0,03	0,02	0	0	0,93	0	0	0,02	0	0,93	0	0,05	0,02	0,046

1,5	17150	0	0,98	0	0	0,02	0	0	0	0	0	0,98	0	0,02	0,036
1,5	39821	0	0	0	0,06	0,9	0	0	0	0,04	0	0	0,2	0,8	0,042
1,5	18501	0	0,81	0	0	0	0	0,01	0,18	0	0	0,81	0	0,19	0,04
1,5	18479	0,06	0	0,01	0	0,05	0,85	0	0,03	0	0	0,01	0,91	0,08	0,048
1,5	9580	0,04	0,01	0,02	0,02	0	0,91	0	0	0	0,04	0,01	0,93	0,02	0,04
1,5	7097	0	0,9	0,01	0	0	0	0,03	0,06	0	0,01	0,09	0	0,9	0,04
1,5	4580	0,05	0	0	0,01	0,42	0	0	0,52	0	0,43	0,05	0	0,52	0,044
1,5	5958	0,04	0	0,03	0	0,92	0,01	0	0	0	0	0,92	0,05	0,03	0,04
1,5	10016	0,01	0	0	0	0,64	0,26	0,08	0	0,01	0,26	0,01	0	0,73	0,046
1,5	8240	0,06	0	0	0	0,91	0	0	0	0,03	0	0,04	0,18	0,78	0,048
1,5	7100	0	0,04	0	0,04	0,59	0,01	0,02	0,3	0	0,07	0	0,04	0,89	0,046
1,5	3632	0,05	0	0,03	0	0,92	0	0	0	0	0,01	0,96	0	0,03	0,042
1,5	4001	0,05	0	0,03	0	0,05	0	0	0,81	0,06	0,06	0,13	0	0,81	0,046
1,5	20331	0	0	0,04	0,04	0	0,92	0	0	0	0,04	0	0	0,96	0,032
1,5	11186	0	0	0	0,06	0,94	0	0	0	0	0,07	0	0	0,93	0,044
1,5	3550	0,06	0	0,01	0	0,88	0,05	0	0	0	0,12	0,09	0,73	0,06	0,04
1,5	12266	0,05	0	0	0	0,8	0	0	0,15	0	0,08	0,07	0,05	0,8	0,034
1,5	4953	0,04	0	0,01	0	0,92	0,03	0	0	0	0,93	0,04	0,03	0	0,048
1,5	6110	0,06	0	0	0	0,9	0,03	0,01	0	0	0,92	0,07	0,01	0	0,048
1,5	15783	0,01	0	0	0,08	0,91	0	0	0	0	0,29	0,7	0,01	0	0,04
1,5	26471	0,03	0,13	0,02	0	0	0,77	0,01	0	0,04	0,02	0,13	0,01	0,84	0,046
1,5	18760	0,05	0	0,02	0,01	0,9	0	0	0,02	0	0,01	0,9	0,05	0,04	0,042
1,5	13156	0,04	0	0	0	0,14	0,82	0	0	0	0,05	0	0,77	0,18	0,028
1,5	2182	0,05	0	0	0	0,88	0,06	0,01	0	0	0,04	0,89	0,07	0	0,046
1,5	40745	0	0	0	0,17	0,8	0,02	0	0	0,01	0,77	0,02	0,08	0,13	0,042
1,5	16709	0,02	0	0	0,08	0,01	0,55	0	0,34	0	0,02	0,34	0,63	0,01	0,046
1,5	26482	0,04	0	0	0	0,94	0	0,02	0	0	0,04	0	0,96	0	0,038
1,5	16370	0,05	0	0	0	0,82	0,1	0,03	0	0	0,01	0	0,92	0,07	0,046
1,5	17490	0	0	0	0,06	0,79	0	0	0,15	0	0,01	0,05	0	0,94	0,044
1,5	19892	0,07	0	0	0	0	0,78	0	0,15	0	0,56	0	0,08	0,36	0,044
1,5	3867	0	0	0,04	0	0,95	0	0	0	0,01	0,01	0,02	0,8	0,17	0,024
1,5	22248	0	0	0	0,15	0,47	0,23	0,01	0,14	0	0,47	0,19	0,29	0,05	0,048
1,5	15574	0,01	0,03	0	0,09	0,87	0	0	0	0	0,88	0	0,03	0,09	0,044

1,5	12848	0,02	0,55	0,03	0	0,39	0	0	0	0,01	0,07	0,01	0	0,92	0,046
1,5	20910	0,05	0,04	0	0	0,8	0	0	0,11	0	0,01	0,11	0,82	0,06	0,044
1,5	11578	0	0,05	0	0,01	0,94	0	0	0	0	0,98	0,01	0,01	0	0,046
1,5	22598	0	0,14	0	0	0,85	0,01	0	0	0	0	0,14	0,86	0	0,048
1,5	9868	0	0,01	0,03	0,07	0,65	0	0	0,22	0,02	0	0,04	0,73	0,23	0,048
1,5	40212	0	0,2	0	0,04	0,76	0	0	0	0	0,2	0	0	0,8	0,046
1,5	71907	0,03	0	0	0,04	0,88	0,01	0,01	0,03	0	0	0,07	0,89	0,04	0,046
1,5	25459	0	0,06	0	0	0,84	0	0,1	0	0	0,9	0	0	0,1	0,038
1,5	41536	0	0,09	0,03	0	0,85	0	0	0,01	0,02	0,12	0,02	0,85	0,01	0,048
1,5	6479	0,01	0,94	0,04	0	0	0	0	0	0,01	0	0,04	0,02	0,94	0,046
1,5	10162	0	0	0	0,07	0,89	0	0	0,04	0	0,01	0	0,1	0,89	0,044
1,5	8269	0,01	0,96	0	0,03	0	0	0	0	0	0,01	0,01	0	0,98	0,046
1,5	5923	0,01	0,13	0,01	0	0,85	0	0	0	0	0,15	0,71	0,1	0,04	0,042
1,5	21744	0,04	0,04	0	0,01	0,75	0,16	0	0	0	0,17	0,79	0,04	0	0,048
1,5	26646	0	0	0,04	0	0,88	0,03	0	0,05	0	0,03	0,04	0,93	0	0,026
1,5	9162	0,01	0,01	0	0	0,79	0,14	0,05	0	0	0,06	0,41	0,14	0,39	0,048
1,5	17121	0,05	0	0,04	0	0,79	0	0	0,11	0,01	0,04	0,81	0,01	0,14	0,042
1,5	13835	0,06	0	0	0	0,94	0	0	0	0	0,02	0,94	0	0,04	0,048
1,5	16432	0,03	0,01	0,04	0	0,9	0,02	0	0	0	0	0,07	0,93	0	0,038
1,5	4459	0,02	0	0,04	0	0	0	0	0,94	0	0	0,03	0,02	0,95	0,02
1,5	10565	0	0,17	0	0,01	0,78	0	0	0,04	0	0	0,01	0,63	0,36	0,048
1,5	11990	0	1	0	0	0	0	0	0	0	0	1	0	0	0,046
1,5	19325	0,06	0	0	0	0,94	0	0	0	0	0	0,05	0	0,95	0,048
1,5	20868	0,02	0	0	0,07	0,89	0	0	0,02	0	0	0	0,98	0,02	0,048
1,5	19623	0	0	0,04	0	0	0	0	0,75	0,21	0,19	0	0,78	0,03	0,022
1,5	10997	0,06	0	0	0	0,94	0	0	0	0	0	0	0	1	0,048
1,75	15683	0,03	0,09	0	0	0,52	0,29	0	0,07	0	0	0,61	0,36	0,03	0,044
1,75	35562	0	1	0	0	0	0	0	0	0	0	1	0	0	0,034
1,75	8269	0,03	0	0,03	0	0,12	0,81	0,01	0	0	0,1	0,03	0,02	0,85	0,038
1,75	4026	0,05	0	0,01	0	0,93	0	0	0,01	0	0	0,7	0	0,3	0,044
1,75	14605	0,06	0	0	0	0	0,38	0	0,56	0	0	0,38	0,59	0,03	0,048
1,75	9544	0,05	0	0	0	0,02	0,93	0	0	0	0	0	0,02	0,98	0,048
1,75	13214	0	0	0,04	0,02	0	0	0	0,94	0	0	0,98	0	0,02	0,016

1,75	12761	0	0,08	0	0	0,92	0	0	0	0	0,92	0	0,08	0	0,048
1,75	18469	0	0,24	0	0	0,64	0	0	0,04	0,08	0,24	0	0,12	0,64	0,046
1,75	29291	0	0,25	0	0,01	0,69	0,05	0	0	0	0,21	0	0,06	0,73	0,046
1,75	15701	0	0,06	0,01	0,01	0,76	0,12	0,04	0	0	0,9	0,01	0,07	0,02	0,04
1,75	6348	0,01	0	0	0	0,94	0	0,05	0	0	0,01	0,01	0,04	0,94	0,044
1,75	10106	0	0	0	0,25	0,75	0	0	0	0	0,91	0,09	0	0	0,046
1,75	12725	0	0,64	0	0	0,36	0	0	0	0	0,06	0	0,08	0,86	0,046
1,75	6697	0,02	0	0,03	0	0	0,12	0,05	0,78	0	0	0,16	0,84	0	0,044
1,75	8363	0	0	0	0,19	0,8	0	0	0	0,01	0,8	0,18	0	0,02	0,046
1,75	3815	0	0	0,04	0	0,91	0	0,01	0,04	0	0	0,04	0,95	0,01	0,02
1,75	18578	0,05	0	0	0	0,94	0	0	0	0,01	0	0,05	0,01	0,94	0,046
1,75	7204	0,06	0	0	0	0,92	0	0	0,01	0,01	0,92	0,01	0,06	0,01	0,044
1,75	5221	0,02	0	0,02	0,04	0,92	0	0	0	0	0,05	0,02	0	0,93	0,048
1,75	3456	0,02	0,05	0,04	0	0,8	0	0	0	0,09	0,15	0,76	0,08	0,01	0,04
1,75	42625	0	0,03	0	0,07	0,67	0	0	0,23	0	0,23	0,74	0,03	0	0,042
1,75	9111	0,03	0,01	0	0	0	0,01	0,04	0,91	0	0,06	0,01	0,92	0,01	0,046
1,75	35727	0,01	0,97	0	0	0	0	0	0	0,02	0	0,21	0	0,79	0,036
1,75	13886	0	0,12	0	0	0,87	0	0	0,01	0	0,01	0,02	0,97	0	0,044
1,75	17789	0	0,15	0	0	0,52	0,33	0	0	0	0	0,52	0,48	0	0,044
1,75	26490	0	0	0	0,06	0,93	0	0	0,01	0	0,06	0,94	0	0	0,044
1,75	11057	0,03	0,02	0	0	0,04	0	0,03	0,88	0	0	0,02	0,03	0,95	0,046
1,75	16179	0	0,02	0,02	0,07	0,84	0	0,03	0	0,02	0,09	0,04	0,85	0,02	0,046
1,75	11099	0,04	0	0	0,08	0,87	0	0	0,01	0	0,98	0,02	0	0	0,044
1,75	51400	0	0	0	0,2	0,79	0,01	0	0	0	0,79	0	0,01	0,2	0,046
1,75	39655	0	0,02	0,04	0	0,56	0,13	0,05	0,1	0,1	0	0,63	0,33	0,04	0,032
1,75	33142	0,01	0	0	0,11	0	0,01	0	0,87	0	0,16	0,03	0,79	0,02	0,048
1,75	10404	0,03	0	0	0,03	0,93	0,01	0	0	0	0,01	0,05	0,64	0,3	0,04
1,75	5377	0,03	0	0	0	0	0,93	0,04	0	0	0,03	0,04	0,93	0	0,042
1,75	23304	0	0	0	0,21	0	0,25	0	0,54	0	0,54	0,21	0,25	0	0,048
1,75	15262	0,04	0,14	0	0	0,79	0,02	0,01	0	0	0,01	0,16	0,01	0,82	0,048
1,75	24816	0,03	0,63	0	0	0,33	0	0,01	0	0	0	0,36	0	0,64	0,048
1,75	52442	0,05	0,01	0,01	0	0,76	0	0	0,17	0	0,01	0,06	0,17	0,76	0,044
1,75	6115	0,03	0,95	0	0	0,01	0	0	0,01	0	0	0,02	0,01	0,97	0,044

1,75	12895	0,03	0	0,03	0,04	0,9	0	0	0	0	0,07	0,01	0,04	0,88	0,048
1,75	6593	0,06	0	0,03	0	0,91	0	0	0	0	0	0,94	0,05	0,01	0,04
1,75	45825	0	1	0	0	0	0	0	0	0	0	1	0	0	0,026
1,75	13055	0,06	0	0	0	0,93	0	0	0,01	0	0,94	0	0,06	0	0,044
1,75	17663	0,04	0	0	0	0,91	0,05	0	0	0	0,58	0,04	0	0,38	0,034
1,75	8880	0,06	0,06	0	0	0,88	0	0	0	0	0,07	0	0	0,93	0,048
1,75	38675	0,05	0,01	0	0	0,73	0,15	0	0,06	0	0,84	0,01	0,15	0	0,038
1,75	14377	0	0,28	0	0	0,71	0	0	0,01	0	0,29	0	0	0,71	0,04
1,75	29704	0	0,36	0	0,06	0	0	0	0,58	0	0	0,42	0	0,58	0,048
1,75	20958	0,05	0	0	0	0,09	0,24	0,01	0,61	0	0	0,14	0,72	0,14	0,048
1,75	10679	0	0	0	0,06	0,85	0	0	0,09	0	0,06	0,09	0	0,85	0,048
1,75	36157	0	0	0	0,05	0,94	0	0,01	0	0	0,05	0,95	0	0	0,038
1,75	20705	0	0	0,02	0,15	0,83	0	0	0	0	0,81	0	0,17	0,02	0,038
1,75	42601	0,01	0	0,04	0	0,09	0	0	0,86	0	0,09	0,9	0,01	0	0,02
1,75	6341	0,05	0	0,02	0	0,92	0	0	0,01	0	0,93	0,02	0,05	0	0,048
1,75	26770	0	0,96	0,04	0	0	0	0	0	0	0	0,04	0	0,96	0,026
1,75	7261	0,06	0	0	0	0,93	0	0	0,01	0	0,06	0	0,93	0,01	0,044
1,75	7416	0,05	0	0	0,01	0	0	0	0,87	0,07	0,12	0	0,87	0,01	0,042
1,75	8893	0,03	0	0,04	0	0,7	0	0	0,23	0	0,23	0,02	0,75	0	0,04
1,75	15108	0,04	0	0,02	0	0,81	0,06	0,03	0,04	0	0	0,85	0,11	0,04	0,048
1,75	35320	0	1	0	0	0	0	0	0	0	0	1	0	0	0,038
1,75	20311	0,01	0	0	0	0,01	0,5	0,08	0,4	0	0,23	0,51	0,18	0,08	0,048
1,75	32599	0,05	0	0	0	0,92	0	0,03	0	0	0,06	0	0,91	0,03	0,046
1,75	7502	0,01	0	0,04	0	0,39	0	0,03	0,4	0,13	0,2	0,43	0,01	0,36	0,048
1,75	20297	0,04	0,01	0	0	0,93	0	0,02	0	0	0	0	0,97	0,03	0,04
1,75	15895	0	0,05	0,03	0	0,92	0	0	0	0	0,92	0,04	0,04	0	0,038
1,75	5981	0,04	0,03	0	0	0,41	0,32	0	0,2	0	0,44	0,47	0,07	0,02	0,048
1,75	9917	0,04	0,01	0,04	0	0,25	0,66	0	0	0	0	0,05	0,04	0,91	0,044
1,75	22412	0,03	0	0,03	0	0,93	0,01	0	0	0	0,24	0,03	0,73	0	0,044
1,75	17718	0,05	0	0	0	0,88	0,07	0	0	0	0,95	0	0	0,05	0,044
1,75	16081	0	0	0	0,27	0,72	0,01	0	0	0	0,96	0	0	0,04	0,038
1,75	11502	0,05	0	0	0,01	0	0	0	0,93	0,01	0,02	0,04	0,94	0	0,046
1,75	10959	0,04	0	0,01	0	0,95	0	0	0	0	0,95	0,05	0	0	0,048

1,75	1245	0	0	0,04	0,02	0,1	0	0,01	0,37	0,46	0,47	0,2	0,31	0,02	0,046
1,75	16824	0,06	0	0	0	0,91	0,02	0	0,01	0	0	0,92	0,06	0,02	0,044
1,75	43555	0,03	0,02	0,01	0	0,58	0,36	0	0	0	0,9	0,04	0,05	0,01	0,048
1,75	25344	0	0,06	0	0,06	0	0,06	0	0,82	0	0,06	0,7	0,01	0,23	0,04
1,75	20861	0	0,14	0	0	0,86	0	0	0	0	0,01	0	0,99	0	0,044
1,75	17505	0,05	0	0,03	0	0,92	0	0	0	0	0,01	0,03	0,91	0,05	0,042
1,75	12122	0,06	0,01	0	0	0,79	0	0,04	0,1	0	0,06	0	0,9	0,04	0,046
1,75	11750	0,01	0	0	0,05	0,44	0,06	0	0,44	0	0	0,44	0,11	0,45	0,048
1,75	7927	0,03	0,97	0	0	0	0	0	0	0	0	0	0,02	0,98	0,046
1,75	13188	0	0,07	0,01	0,04	0,88	0	0	0	0	0,11	0	0,89	0	0,044
1,75	5361	0	0	0,04	0	0,92	0	0,02	0,02	0	0	0,08	0,92	0	0,036
1,75	12471	0	0	0	0,14	0,8	0	0	0,06	0	0,43	0	0,51	0,06	0,048
1,75	10382	0	0,02	0	0,05	0,53	0	0,02	0,38	0	0,02	0,03	0,38	0,57	0,046
1,75	16852	0,06	0	0	0	0,9	0,04	0	0	0	0	0,06	0,05	0,89	0,044
1,75	28614	0,04	0,08	0	0,01	0,87	0	0	0	0	0,01	0	0,12	0,87	0,044
1,75	19683	0	0,96	0	0	0	0,04	0	0	0	0,04	0	0	0,96	0,044
1,75	32893	0,01	0	0	0,06	0,02	0	0,03	0,88	0	0,06	0,02	0,73	0,19	0,04
1,75	11837	0,02	0,09	0	0,01	0	0	0,02	0,85	0,01	0	0,01	0,11	0,88	0,042
1,75	23643	0	0	0	0,15	0,67	0,14	0,01	0,03	0	0,56	0	0,41	0,03	0,044
1,75	8244	0	0,05	0,04	0,01	0,17	0,73	0	0	0	0,78	0	0,21	0,01	0,046
1,75	10982	0	0,16	0	0	0,36	0,33	0	0,15	0	0,16	0,48	0,36	0	0,048
1,75	18310	0,05	0	0,02	0	0,92	0	0	0,01	0	0,92	0	0	0,08	0,046
1,75	9631	0,05	0	0,02	0	0	0	0	0,9	0,03	0	0,05	0,95	0	0,044
1,75	6901	0	0,3	0	0	0,7	0	0	0	0	0	0	0,3	0,7	0,03
1,75	12368	0,06	0	0	0	0,94	0	0	0	0	0	0,06	0	0,94	0,046
1,75	14712	0,05	0,01	0	0	0,42	0,22	0	0,3	0	0,65	0,13	0,22	0	0,046
1,75	8599	0,04	0	0	0	0,9	0	0,02	0	0,04	0,04	0,92	0,04	0	0,044
2	29393	0,01	0	0,02	0,15	0,19	0,63	0	0	0	0,65	0,34	0	0,01	0,048
2	7855	0,03	0,02	0,03	0	0,69	0,02	0	0	0,21	0,21	0,76	0,03	0	0,04
2	29736	0,04	0	0,03	0	0,91	0,01	0,01	0	0	0	0	0,96	0,04	0,048
2	55489	0,02	0	0	0,07	0,91	0	0	0	0	0,31	0,07	0,62	0	0,048
2	5332	0,05	0	0,02	0,01	0	0	0	0,92	0	0	0	0	1	0,044
2	10722	0,06	0,06	0	0	0,78	0,01	0	0,09	0	0,1	0,11	0,01	0,78	0,046

2	6636	0,02	0	0,03	0	0	0,5	0,04	0,26	0,15	0,31	0,05	0,17	0,47	0,044
2	28205	0,05	0,02	0	0	0,44	0	0	0,49	0	0,02	0,05	0,49	0,44	0,046
2	34529	0	0,97	0	0	0	0	0	0,03	0	0	0,97	0	0,03	0,032
2	10687	0,06	0,01	0	0	0,93	0	0	0	0	0,03	0	0,9	0,07	0,046
2	3875	0	0	0	0,14	0,85	0,01	0	0	0	0,67	0,14	0	0,19	0,048
2	8491	0	0	0	0,29	0,67	0,03	0,01	0	0	0,99	0	0,01	0	0,046
2	10308	0,03	0	0	0,06	0,9	0	0	0	0,01	0,8	0,16	0	0,04	0,04
2	3575	0	0	0,04	0	0	0	0	0,92	0,04	0,04	0,03	0,22	0,71	0,012
2	23127	0	0	0,04	0	0	0	0,04	0,91	0,01	0	0	0,02	0,98	0,026
2	6375	0,06	0	0	0	0,92	0	0,02	0	0	0,92	0,01	0,07	0	0,048
2	6993	0,07	0	0	0	0,84	0	0	0,07	0,02	0	0,08	0,03	0,89	0,048
2	5074	0	0,07	0	0	0,92	0	0,01	0	0	0,93	0	0	0,07	0,042
2	11354	0,04	0,88	0	0	0,08	0	0	0	0	0	0,02	0	0,98	0,048
2	15166	0	0	0	0,27	0,68	0	0	0	0,05	1	0	0	0	0,048
2	22183	0,04	0	0,04	0	0,92	0	0	0	0	0	0,04	0,96	0	0,048
2	33976	0,04	0	0,03	0,01	0	0,02	0	0,9	0	0,02	0,03	0,02	0,93	0,048
2	39150	0	0,14	0	0	0,86	0	0	0	0	0	0	1	0	0,042
2	7458	0,03	0,09	0	0	0,15	0	0,02	0,71	0	0,1	0,01	0	0,89	0,044
2	20409	0	0,05	0,04	0	0,87	0	0,01	0,03	0	0,27	0,04	0,65	0,04	0,046
2	21946	0	0,09	0,04	0	0,77	0	0	0	0,1	0	0,95	0,04	0,01	0,034
2	14353	0	0,01	0	0,11	0,88	0	0	0	0	0,03	0,01	0,95	0,01	0,046
2	12307	0	0	0,04	0,03	0,91	0	0	0,01	0,01	0,01	0,95	0,02	0,02	0,028
2	16799	0	0,08	0	0	0	0	0	0,91	0,01	0,73	0,23	0,03	0,01	0,038
2	61760	0	0,09	0	0	0,85	0	0	0,06	0	0,91	0	0,09	0	0,042
2	22083	0	0,01	0	0,06	0,84	0	0	0,09	0	0	0	0,06	0,94	0,046
2	28935	0	0,15	0	0,06	0,74	0,03	0	0	0,02	0	0,18	0,02	0,8	0,048
2	8769	0,03	0	0	0,03	0,94	0	0	0	0	0	0,94	0,06	0	0,042
2	21629	0	0,12	0	0	0,87	0	0	0,01	0	0,01	0	0,99	0	0,044
2	70296	0,05	0	0,01	0	0,94	0	0	0	0	0	0,01	0,94	0,05	0,048
2	32244	0,02	0	0,01	0,07	0	0,29	0	0,61	0	0,43	0,54	0,03	0	0,042
2	3945	0,05	0	0,04	0	0,77	0	0	0,14	0	0	0,98	0,02	0	0,048
2	18711	0,05	0	0,04	0	0,89	0	0	0,02	0	0,05	0,04	0,06	0,85	0,042
2	11218	0,05	0,01	0	0	0,76	0,17	0	0	0,01	0,98	0	0,02	0	0,046

2	18008	0	0,12	0	0	0	0,86	0	0	0,02	0,11	0,55	0,33	0,01	0,046
2	46404	0,05	0	0	0	0,94	0	0,01	0	0	0,9	0	0	0,1	0,042
2	6733	0,06	0	0	0,01	0,66	0	0	0,27	0	0,27	0	0,02	0,71	0,046
2	11649	0,03	0,95	0,02	0	0	0	0	0	0	0	0,18	0	0,82	0,036
2	18254	0	0	0,04	0,04	0,92	0	0	0	0	0,94	0,03	0,03	0	0,036
2	14476	0	0,01	0	0,1	0,03	0,01	0	0,83	0,02	0,01	0,88	0,11	0	0,048
2	26960	0	0,12	0	0,02	0,83	0,03	0	0	0	0,02	0,12	0,85	0,01	0,044
2	13973	0,05	0,01	0	0	0,91	0	0,02	0,01	0	0,86	0,01	0,02	0,11	0,048
2	6050	0,04	0,01	0	0	0,59	0	0,01	0,35	0	0,39	0,56	0,04	0,01	0,042
2	25103	0,05	0,03	0	0	0,9	0	0	0	0,02	0,03	0,9	0,05	0,02	0,046
2	20664	0,02	0	0,03	0,04	0	0	0	0,83	0,08	0	0,13	0,74	0,13	0,048
2	46258	0	0	0	0,07	0,92	0	0	0,01	0	0	0,01	0,06	0,93	0,042
2	8860	0,04	0	0	0,01	0,95	0	0	0	0	0,04	0	0,96	0	0,03
2	17471	0	0	0	0,08	0,51	0,05	0	0,36	0	0	0,02	0,44	0,54	0,04
2	6760	0	0	0	0,2	0,8	0	0	0	0	0,8	0,2	0	0	0,048
2	21271	0	0,05	0,03	0,01	0,04	0,86	0,01	0	0	0,96	0	0,04	0	0,048
2	16845	0	0,14	0	0	0,01	0,06	0	0,79	0	0	0	0,86	0,14	0,048
2	10063	0	0	0	0,05	0,95	0	0	0	0	0,01	0,95	0	0,04	0,044
2	20638	0	0,12	0	0	0,84	0	0	0,02	0,02	0,33	0,63	0,04	0	0,038
2	7759	0,05	0	0	0,05	0	0,87	0	0	0,03	0,36	0,18	0,46	0	0,036
2	13878	0,02	0,15	0	0	0,82	0	0	0,01	0	0,01	0,84	0,15	0	0,048
2	20685	0	0	0,04	0	0,91	0	0,01	0	0,04	0,94	0,01	0,05	0	0,038
2	31854	0,04	0	0	0,01	0,79	0,16	0	0	0	0,79	0	0,21	0	0,038
2	8642	0,05	0	0	0,04	0,89	0,02	0	0	0	0,89	0,02	0,04	0,05	0,048
2	7507	0,03	0,06	0	0,01	0,9	0	0	0	0	0	0,9	0,06	0,04	0,048
2	50982	0,02	0,19	0	0	0,78	0	0	0,01	0	0,15	0	0,04	0,81	0,046
2	20884	0	0,05	0	0,09	0,8	0	0	0,06	0	0,8	0	0,12	0,08	0,048
2	12348	0,04	0	0	0	0,12	0,4	0	0,43	0,01	0,13	0,4	0	0,47	0,034
2	7550	0,01	0	0,04	0,01	0,06	0,83	0	0,05	0	0,83	0,1	0,06	0,01	0,046
2	52308	0,03	0	0	0,07	0,8	0	0	0,08	0,02	0,2	0,78	0,02	0	0,046
2	17205	0,05	0,02	0,02	0	0,01	0,87	0	0	0,03	0,04	0,02	0,04	0,9	0,048
2	27022	0,04	0	0	0	0,82	0	0,02	0,04	0,08	0,04	0,92	0,01	0,03	0,048
2	6388	0,05	0	0,02	0	0,83	0,1	0	0	0	0,1	0,02	0,87	0,01	0,042

2	17272	0	0,98	0	0	0	0,02	0	0	0	0,02	0,72	0	0,26	0,042
2	23841	0,06	0	0	0	0,45	0,49	0	0	0	0,48	0,02	0,09	0,41	0,048
2	10187	0,01	0,01	0	0,09	0,02	0,86	0	0,01	0	0,01	0,01	0,89	0,09	0,048
2	4331	0,02	0	0,04	0	0,08	0	0,01	0,82	0,03	0,01	0,06	0,87	0,06	0,046
2	7110	0,07	0	0	0	0,92	0	0	0	0,01	0,01	0,91	0,08	0	0,046
2	81144	0,03	0,94	0	0	0	0	0	0,03	0	0,03	0	0	0,97	0,04
2	6090	0,07	0,03	0	0	0,9	0	0	0	0	0,03	0	0,97	0	0,048
2	9956	0	0	0	0,23	0	0,62	0,02	0,02	0,11	0,63	0,13	0,24	0	0,048
2	18127	0,02	0	0	0,04	0	0,03	0	0,91	0	0	0	0,07	0,93	0,046
2	11626	0,03	0,01	0,02	0	0,9	0	0	0,04	0	0,91	0,02	0,04	0,03	0,046
2	14312	0,06	0	0	0	0,9	0	0	0,04	0	0,02	0	0,02	0,96	0,044
2	22697	0	0,05	0,04	0,04	0,87	0	0	0	0	0,02	0	0,07	0,91	0,042
2	25988	0	0	0	0,06	0,94	0	0	0	0	0	0,99	0	0,01	0,044
2	13852	0,01	0,96	0,02	0	0	0	0,01	0	0	0,02	0,15	0,01	0,82	0,048
2	11310	0	0	0,02	0,21	0,76	0	0	0,01	0	0,97	0,03	0	0	0,046
2	15035	0,02	0	0,04	0	0,88	0,06	0	0	0	0,06	0,14	0	0,8	0,02
2	20907	0	0,25	0	0	0,12	0	0	0,63	0	0,25	0,12	0	0,63	0,03
2	6555	0,05	0	0	0	0,94	0	0	0,01	0	0	0,05	0	0,95	0,048
2	33921	0	0,07	0	0,05	0,1	0	0,01	0,77	0	0,06	0,11	0,05	0,78	0,048
2	8312	0,03	0	0,04	0	0,93	0	0	0	0	0,87	0,04	0,09	0	0,04
2	9252	0	0,41	0	0	0,59	0	0	0	0	0	0	0,19	0,81	0,048
2	12703	0	0	0	0,07	0,93	0	0	0	0	0,07	0,93	0	0	0,04
2	27295	0,05	0	0	0	0,95	0	0	0	0	0	0,05	0	0,95	0,046
2	20702	0	0,04	0	0,07	0,89	0	0	0	0	0,32	0,62	0	0,06	0,034
2	15868	0	0	0,04	0	0	0,82	0	0	0,14	0,14	0	0,86	0	0,02
2	9310	0	0,02	0	0,08	0,89	0	0,01	0	0	0,08	0,03	0,75	0,14	0,048
2	4218	0	0,19	0,02	0	0,52	0	0	0,23	0,04	0	0,54	0,19	0,27	0,048
2	3363	0	0	0	0,07	0,22	0	0	0,71	0	0	0	0,22	0,78	0,046
2,25	31477	0	0,01	0	0,07	0,91	0	0	0,01	0	0,07	0,01	0	0,92	0,04
2,25	3486	0,01	0	0,03	0,02	0,23	0	0,02	0,65	0,04	0,02	0,24	0,05	0,69	0,044
2,25	11214	0,05	0	0	0,02	0,02	0	0	0,89	0,02	0	0,04	0,89	0,07	0,042
2,25	4137	0	0,17	0	0	0	0	0	0,83	0	0	0,83	0,16	0,01	0,044
2,25	12849	0,04	0	0,02	0	0,92	0	0,02	0	0	0,89	0,05	0,06	0	0,048

2,25	32265	0,06	0,02	0	0	0,91	0	0	0,01	0	0,01	0,08	0	0,91	0,046
2,25	26317	0	0,37	0,02	0	0,58	0	0	0,03	0	0,01	0	0,23	0,76	0,048
2,25	10893	0,01	0	0	0,07	0	0,92	0	0	0	0,01	0	0	0,99	0,048
2,25	19281	0,05	0	0	0	0	0,81	0	0,14	0	0,14	0	0,05	0,81	0,046
2,25	7255	0	0,13	0	0	0,86	0	0,01	0	0	0	0	0,99	0,01	0,036
2,25	10010	0	0,35	0	0	0,64	0	0	0	0,01	0,21	0,01	0	0,78	0,038
2,25	8278	0	0,04	0	0,1	0,86	0	0	0	0	0,18	0	0,74	0,08	0,046
2,25	41258	0	0,01	0	0,11	0,88	0	0	0	0	0,01	0	0,88	0,11	0,044
2,25	7128	0,01	0,11	0	0	0,88	0	0	0	0	0,11	0	0,88	0,01	0,046
2,25	21436	0,06	0	0	0	0,92	0	0,01	0,01	0	0	0,92	0,07	0,01	0,048
2,25	20003	0	0	0	0,05	0,95	0	0	0	0	0,07	0	0	0,93	0,04
2,25	5709	0	0	0,04	0	0,91	0	0,05	0	0	0	0,02	0,07	0,91	0,044
2,25	15549	0,05	0	0	0	0,93	0	0	0	0,02	0,93	0	0,02	0,05	0,046
2,25	25107	0	0,19	0	0	0,8	0	0,01	0	0	0,09	0,81	0,1	0	0,048
2,25	18439	0	0,29	0	0,01	0	0,08	0	0,62	0	0	0,01	0,38	0,61	0,048
2,25	30628	0,05	0	0	0	0,83	0	0	0,12	0	0	0	0,95	0,05	0,036
2,25	5989	0,01	0,15	0	0	0,01	0	0	0,83	0	0,01	0	0,69	0,3	0,046
2,25	20004	0	0	0,01	0,08	0,9	0,01	0	0	0	0	0,98	0,02	0	0,042
2,25	18073	0	0	0,04	0,01	0,93	0	0,02	0	0	0,02	0,93	0,05	0	0,044
2,25	47881	0	0,25	0	0,03	0	0	0	0,72	0	0	0	0,03	0,97	0,038
2,25	15162	0,05	0	0	0	0,89	0,04	0	0,02	0	0,05	0	0,95	0	0,04
2,25	22512	0,06	0	0	0	0,94	0	0	0	0	0,01	0	0,93	0,06	0,048
2,25	53496	0	0	0	0,14	0	0,54	0	0,31	0,01	0,46	0,46	0	0,08	0,046
2,25	8419	0,05	0	0	0	0,95	0	0	0	0	0,95	0	0	0,05	0,046
2,25	20379	0,05	0,02	0	0,07	0,86	0	0	0	0	0,86	0,12	0	0,02	0,048
2,25	21495	0,04	0	0,03	0	0,53	0,03	0,01	0,36	0	0,07	0,5	0,39	0,04	0,042
2,25	35792	0,01	0,91	0,02	0	0	0	0	0,01	0,05	0	0,63	0	0,37	0,048
2,25	9343	0	1	0	0	0	0	0	0	0	0	1	0	0	0,044
2,25	29037	0,01	0,12	0	0	0,82	0	0	0,05	0	0	0	0,95	0,05	0,046
2,25	14837	0	0,24	0	0	0,76	0	0	0	0	0	0,63	0,24	0,13	0,03
2,25	14268	0,04	0	0,01	0	0,78	0,14	0,03	0	0	0,69	0,18	0	0,13	0,046
2,25	14008	0,01	0	0	0,06	0,01	0,01	0	0,91	0	0,07	0,01	0	0,92	0,048
2,25	11387	0,02	0,08	0	0	0,15	0,71	0,01	0	0,03	0,09	0,24	0,65	0,02	0,04

2,25	35035	0,01	0,86	0	0	0,05	0	0	0,07	0,01	0,04	0,01	0,02	0,93	0,046
2,25	18668	0,03	0	0,02	0,02	0,88	0	0,01	0	0,04	0,09	0,89	0	0,02	0,046
2,25	12150	0	0,02	0,04	0	0,03	0,91	0	0	0	0,96	0,04	0	0	0,04
2,25	22391	0	0	0	0,07	0,86	0	0	0,07	0	0,07	0,87	0,06	0	0,038
2,25	17697	0	0,06	0	0,01	0,91	0	0,01	0	0,01	0,92	0,01	0,06	0,01	0,044
2,25	17143	0,03	0,95	0	0	0	0,02	0	0	0	0	0,02	0,03	0,95	0,04
2,25	5047	0	0	0	0	0	0	0,12	0,88	0	0,12	0	0,88	0	0,046
2,25	18031	0	0,04	0	0,07	0,88	0	0	0	0,01	0,06	0	0,05	0,89	0,046
2,25	9487	0,04	0,05	0	0	0,88	0,03	0	0	0	0,04	0,03	0,88	0,05	0,042
2,25	16483	0	0	0	0,06	0,91	0	0	0,03	0	0,05	0,49	0,01	0,45	0,048
2,25	39921	0,06	0,02	0	0	0,92	0	0	0	0	0,02	0,18	0	0,8	0,048
2,25	23173	0,05	0	0	0	0,06	0	0,01	0,88	0	0	0,83	0,01	0,16	0,046
2,25	10452	0,03	0,15	0,01	0,01	0,8	0	0	0	0	0	0,01	0,18	0,81	0,046
2,25	18881	0	0	0	0,04	0,94	0,01	0,01	0	0	0,04	0,94	0	0,02	0,042
2,25	21672	0	0	0	0,1	0,47	0,26	0	0,17	0	0,11	0,17	0,46	0,26	0,048
2,25	24912	0,05	0	0,01	0	0,94	0	0	0	0	0,01	0,33	0,65	0,01	0,048
2,25	10866	0	0	0,01	0,05	0,89	0	0,01	0,02	0,02	0,01	0,03	0,05	0,91	0,048
2,25	24152	0	0	0,04	0	0,02	0	0	0,94	0	0	0,02	0,98	0	0,036
2,25	18872	0,01	0	0,03	0,11	0,85	0	0	0	0	0,77	0,16	0,06	0,01	0,048
2,25	10139	0,05	0	0	0	0,95	0	0	0	0	0	0,04	0	0,96	0,048
2,25	8913	0	0,02	0	0,07	0,9	0	0,01	0	0	0,09	0	0,9	0,01	0,048
2,25	2386	0,04	0	0	0	0,28	0	0,03	0,65	0	0,03	0,93	0,04	0	0,048
2,25	30131	0,02	0	0	0	0,03	0,78	0,16	0	0,01	0,78	0,16	0,05	0,01	0,042
2,25	19411	0,04	0	0,02	0	0,92	0	0,01	0,01	0	0,06	0,04	0,9	0	0,046
2,25	7785	0,03	0,03	0	0,02	0,92	0	0	0	0	0,06	0	0,92	0,02	0,044
2,25	23954	0,05	0	0	0	0,92	0	0,01	0,02	0	0,02	0,01	0,92	0,05	0,048
2,25	2665	0,01	0	0	0	0	0	0,05	0,94	0	0	0,01	0,01	0,98	0,046
2,25	2818	0,02	0	0,04	0	0,9	0	0	0,04	0	0	0,04	0,94	0,02	0,03
2,25	22223	0	0	0	0,28	0,72	0	0	0	0	1	0	0	0	0,038
2,25	22219	0	0,95	0,01	0	0,01	0,03	0	0	0	0,03	0,01	0,01	0,95	0,042
2,25	25706	0	0,38	0	0	0,59	0,01	0,02	0	0	0,07	0,39	0,02	0,52	0,046
2,25	14587	0	0	0	0,06	0,93	0	0,01	0	0	0	0,93	0,07	0	0,046
2,25	31609	0	0,11	0,03	0	0,8	0	0,06	0	0	0	0,86	0	0,14	0,048

2,25	14200	0	0,15	0	0	0,65	0,2	0	0	0	0,22	0,13	0,31	0,34	0,042
2,25	30943	0	0	0	0,11	0,87	0	0	0,02	0	0,02	0	0,98	0	0,04
2,25	15359	0,05	0	0	0	0,91	0,04	0	0	0	0,95	0	0	0,05	0,038
2,25	53717	0	0	0	0,07	0	0	0	0,93	0	0	1	0	0	0,046
2,25	9991	0,06	0	0	0	0,1	0	0	0,79	0,05	0,84	0,09	0,04	0,03	0,044
2,25	11047	0,02	0,03	0	0,03	0,92	0	0	0	0	0,02	0,02	0,92	0,04	0,042
2,25	6825	0,03	0,01	0	0,05	0,91	0	0	0	0	0,03	0	0,96	0,01	0,048
2,25	6585	0,06	0	0,01	0	0,91	0,02	0	0	0	0,01	0,93	0,01	0,05	0,042
2,25	14143	0	0,03	0,04	0	0,91	0	0	0	0,02	0	0,95	0,05	0	0,014
2,25	33542	0	0	0	0,07	0,91	0	0	0,02	0	0,09	0,91	0	0	0,048
2,25	36753	0	0,11	0	0	0,59	0,16	0,01	0,12	0,01	0,33	0,27	0,24	0,16	0,03
2,25	7893	0,04	0,01	0,04	0,01	0	0	0	0,89	0,01	0,01	0,94	0	0,05	0,046
2,25	26291	0	0,28	0	0	0,68	0	0	0,04	0	0,03	0,05	0,24	0,68	0,048
2,25	19684	0,04	0,03	0,02	0	0	0	0,02	0,88	0,01	0,06	0,02	0,04	0,88	0,048
2,25	4808	0	0	0	0,23	0,75	0	0	0	0,02	0,88	0,03	0,09	0	0,046
2,25	34254	0,01	0,1	0	0,05	0,83	0	0,01	0	0	0,1	0,83	0,02	0,05	0,046
2,25	8911	0,05	0	0	0	0,88	0,07	0	0	0	0,95	0,05	0	0	0,048
2,25	16957	0,01	0,06	0	0	0,92	0	0	0	0,01	0,92	0,06	0,01	0,01	0,046
2,25	11688	0,03	0,55	0	0	0	0	0	0,42	0	0	0,85	0	0,15	0,044
2,25	13987	0,03	0,01	0	0,03	0,18	0	0	0,73	0,02	0,73	0,22	0,05	0	0,04
2,25	14893	0	0	0	0,13	0,86	0,01	0	0	0	0,13	0	0,86	0,01	0,044
2,25	9627	0	0,25	0	0	0,74	0,01	0	0	0	0,11	0,79	0,09	0,01	0,048
2,25	40922	0	0	0	0,1	0,9	0	0	0	0	0,05	0	0,9	0,05	0,044
2,25	39361	0	0,01	0	0,13	0,82	0,02	0	0	0,02	0,14	0,11	0,74	0,01	0,048
2,25	7007	0	0	0,04	0	0,85	0	0	0,06	0,05	0,91	0,05	0,04	0	0,026
2,25	24048	0	0,08	0	0,06	0,86	0	0	0	0	0	0,08	0,06	0,86	0,04
2,25	16057	0,02	0,49	0	0	0,07	0	0	0,42	0	0	0,03	0,09	0,88	0,042
2,25	26740	0,02	0	0	0,04	0	0	0,03	0,91	0	0	0,15	0,82	0,03	0,048
2,25	12909	0,01	0	0	0,11	0,87	0	0,01	0	0	0,1	0	0,87	0,03	0,046
2,5	16601	0,06	0	0	0	0,84	0,01	0	0,09	0	0	0,36	0,49	0,15	0,046
2,5	39814	0,06	0	0	0	0,84	0,02	0	0	0,08	0	0,84	0,16	0	0,048
2,5	20671	0,02	0	0	0	0,01	0,77	0,15	0,05	0	0,78	0,21	0,01	0	0,048
2,5	13028	0	0	0	0,09	0	0,55	0	0,36	0	0	0,55	0,09	0,36	0,046

2,5	9387	0,04	0	0	0,01	0,95	0	0	0	0	0	0,95	0,04	0,01	0,032
2,5	21005	0	0,06	0	0,06	0,88	0	0	0	0	0,08	0,06	0,86	0	0,044
2,5	25210	0,04	0,08	0	0	0	0	0	0,87	0,01	0	0,94	0,06	0	0,042
2,5	16193	0	0	0,04	0	0,68	0	0	0,28	0	0,57	0,04	0,39	0	0,044
2,5	8111	0,05	0	0,01	0	0	0,27	0	0,65	0,02	0,65	0,02	0	0,33	0,038
2,5	13738	0,06	0	0	0	0,8	0,12	0,02	0	0	0,8	0,02	0,16	0,02	0,042
2,5	12519	0	0,96	0,04	0	0	0	0	0	0	0,04	0	0	0,96	0,036
2,5	13500	0,02	0	0	0	0,89	0	0,05	0,04	0	0	0,05	0,12	0,83	0,044
2,5	35333	0,06	0	0	0	0,02	0	0	0,92	0	0,01	0	0,07	0,92	0,048
2,5	14735	0,02	0,14	0,03	0	0,45	0	0	0,36	0	0,22	0,03	0,01	0,74	0,042
2,5	7300	0,04	0	0,01	0	0,95	0	0	0	0	0,95	0,04	0,01	0	0,04
2,5	25050	0	0	0	0,06	0,02	0,29	0,09	0,54	0	0,31	0,09	0,6	0	0,044
2,5	13763	0	0,11	0	0	0,17	0,13	0	0,59	0	0,62	0	0	0,38	0,048
2,5	10158	0	0	0	0,09	0,91	0	0	0	0	0	0,08	0,92	0	0,04
2,5	20465	0	0	0	0,08	0,07	0,01	0	0,81	0,03	0,03	0,86	0,03	0,08	0,048
2,5	8940	0,02	0,24	0	0	0,7	0,04	0	0	0	0,04	0,02	0,24	0,7	0,048
2,5	18482	0,02	0	0,04	0,05	0,82	0,07	0	0	0	0	0	0,82	0,18	0,046
2,5	34389	0	0	0	0,07	0,89	0,01	0	0,03	0	0,04	0	0,07	0,89	0,044
2,5	20079	0,04	0,11	0,03	0	0,81	0,01	0	0	0	0,11	0,03	0,05	0,81	0,048
2,5	12769	0,03	0	0	0	0,95	0	0,02	0	0	0,02	0,35	0,63	0	0,048
2,5	18985	0	0	0	0	0,48	0,37	0,15	0	0	0,48	0	0,37	0,15	0,048
2,5	4456	0,05	0	0	0	0,92	0,01	0	0,02	0	0	0,92	0	0,08	0,042
2,5	12732	0,01	0	0	0	0,78	0	0,05	0,16	0	0,16	0,05	0	0,79	0,042
2,5	13458	0,04	0	0	0,04	0	0	0	0,91	0,01	0,91	0	0,04	0,05	0,048
2,5	20225	0	0,09	0	0	0,91	0	0	0	0	0,8	0,11	0,09	0	0,042
2,5	38460	0,06	0	0	0	0,93	0	0,01	0	0	0,06	0	0,93	0,01	0,048
2,5	15136	0,05	0	0,01	0	0,93	0	0	0	0,01	0,31	0,01	0,01	0,67	0,038
2,5	16589	0	0,01	0	0,02	0,93	0	0,03	0	0,01	0,05	0,01	0	0,94	0,038
2,5	5220	0,05	0,28	0	0	0,11	0	0	0,56	0	0	0,67	0	0,33	0,046
2,5	8219	0	0	0,01	0,09	0,87	0	0	0,01	0,02	0,1	0,01	0,53	0,36	0,046
2,5	22490	0	0	0	0,07	0,93	0	0	0	0	0,02	0,93	0	0,05	0,048
2,5	35811	0	0	0	0,07	0,78	0,14	0,01	0	0	0,04	0,21	0,01	0,74	0,046
2,5	10079	0,03	0,01	0	0,02	0,94	0	0	0	0	0,03	0	0,03	0,94	0,048

2,5	35201	0,05	0	0	0,01	0,94	0	0	0	0	0,13	0	0,24	0,63	0,048
2,5	19036	0	0,18	0	0	0,81	0,01	0	0	0	0,3	0	0,18	0,52	0,042
2,5	10402	0,05	0	0	0	0,91	0	0,01	0,03	0	0,15	0,05	0	0,8	0,042
2,5	13670	0	0,02	0	0,16	0,82	0	0	0	0	0,82	0	0,18	0	0,044
2,5	15000	0,01	0,7	0	0	0,08	0	0	0,21	0	0	0,21	0,09	0,7	0,038
2,5	11960	0,06	0	0,01	0	0,93	0	0	0	0	0	0,01	0	0,99	0,044
2,5	5628	0,02	0	0	0,14	0,83	0	0,01	0	0	0,84	0	0,16	0	0,046
2,5	9344	0,01	0,99	0	0	0	0	0	0	0	0	0,01	0,04	0,95	0,04
2,5	21882	0,03	0	0	0	0,86	0,02	0,05	0,04	0	0,09	0,03	0,88	0	0,048
2,5	11824	0,02	0	0,04	0	0,94	0	0	0	0	0,04	0	0,96	0	0,03
2,5	19028	0,02	0,01	0	0,02	0,73	0,18	0,03	0	0,01	0,03	0,14	0,72	0,11	0,046
2,5	38777	0	0,02	0,04	0	0,2	0,31	0	0,43	0	0,26	0	0,43	0,31	0,036
2,5	8078	0,05	0	0,02	0	0,93	0	0	0	0	0	0,95	0,05	0	0,044
2,5	26570	0,06	0	0	0	0	0	0	0,89	0,05	0	0,51	0,44	0,05	0,046
2,5	32608	0,05	0,01	0	0	0,93	0	0,01	0	0	0	0,05	0,93	0,02	0,046
2,5	30629	0	0	0	0,11	0	0,86	0	0,03	0	0,03	0	0,56	0,41	0,046
2,5	6840	0,05	0,06	0	0	0,88	0	0	0,01	0	0,09	0	0	0,91	0,044
2,5	33224	0	0	0	0,06	0	0	0	0,94	0	0	1	0	0	0,046
2,5	31164	0	0	0	0,07	0,71	0,1	0	0,11	0,01	0,08	0,23	0	0,69	0,048
2,5	28508	0,06	0,01	0	0	0,93	0	0	0	0	0,01	0,06	0,93	0	0,038
2,5	26402	0	0,01	0	0,19	0,8	0	0	0	0	0,8	0,2	0	0	0,04
2,5	8628	0,06	0	0	0	0,94	0	0	0	0	0	0	0,06	0,94	0,048
2,5	11255	0	0,11	0	0	0	0	0	0,89	0	0	0	1	0	0,04
2,5	20862	0	0,03	0	0,1	0,87	0	0	0	0	0,02	0	0,94	0,04	0,048
2,5	15897	0	0	0,02	0,11	0,87	0	0	0	0	0	0,1	0,79	0,11	0,048
2,5	21637	0	0,03	0	0,11	0,68	0,16	0	0	0,02	0,13	0,15	0,72	0	0,048
2,5	8566	0	0	0	0,24	0	0,02	0	0,74	0	0,76	0,24	0	0	0,048
2,5	7430	0,01	0	0,04	0	0	0	0	0,95	0	0	0,04	0	0,96	0,01
2,5	24805	0	0,06	0	0,05	0,88	0	0	0	0,01	0,94	0	0,05	0,01	0,048
2,5	16216	0,04	0,04	0	0	0,86	0,01	0	0	0,05	0,05	0,01	0,94	0	0,048
2,5	25449	0	0	0	0,07	0,92	0	0,01	0	0	0	0,92	0,08	0	0,038
2,5	14844	0,05	0	0	0	0,46	0,28	0	0,21	0	0,19	0,07	0,27	0,47	0,046
2,5	8442	0	0,15	0	0	0,85	0	0	0	0	0	0,15	0,85	0	0,048

2,5	7536	0	0,09	0	0	0,52	0,39	0	0	0	0,61	0,39	0	0	0,048
2,5	14988	0,05	0	0,02	0	0,93	0	0	0	0	0	0,01	0,06	0,93	0,048
2,5	12090	0,02	0,01	0	0,05	0	0,48	0,04	0,4	0	0,31	0,5	0,09	0,1	0,042
2,5	7056	0,01	0	0,01	0,08	0	0,25	0	0,65	0	0,01	0,73	0,25	0,01	0,048
2,5	8282	0,01	0	0,04	0	0,01	0	0	0,93	0,01	0,03	0,96	0,01	0	0,024
2,5	16327	0	0	0	0,11	0,88	0,01	0	0	0	0	0	0,33	0,67	0,036
2,5	9542	0,03	0,96	0	0	0	0	0,01	0	0	0	0	0,01	0,99	0,044
2,5	12527	0	0	0	0,19	0,79	0	0	0	0,02	0,79	0,02	0,19	0	0,046
2,5	35196	0,05	0	0	0	0,95	0	0	0	0	0	0,95	0	0,05	0,046
2,5	4089	0,03	0	0,03	0	0	0,89	0,05	0	0	0,89	0,02	0,06	0,03	0,044
2,5	15024	0	0	0	0,06	0,94	0	0	0	0	0	0,94	0	0,06	0,046
2,5	24413	0	0,18	0	0	0,78	0	0	0,01	0,03	0,18	0,74	0,04	0,04	0,044
2,5	7985	0,06	0	0	0	0,92	0	0	0,02	0	0,92	0,02	0	0,06	0,046
2,5	15699	0	0	0	0,05	0	0,01	0,01	0,93	0	0,01	0,93	0,01	0,05	0,048
2,5	10671	0,05	0	0	0	0,93	0	0	0	0,02	0,02	0	0,98	0	0,046
2,5	12110	0	0,01	0,04	0,09	0,77	0,06	0,02	0	0,01	0,79	0,05	0,07	0,09	0,048
2,5	8897	0,06	0	0	0	0,91	0,03	0	0	0	0,04	0	0,96	0	0,042
2,5	27086	0	0,1	0,01	0,02	0,87	0	0	0	0	0,02	0	0,98	0	0,048
2,5	5894	0,04	0	0,01	0,02	0,21	0	0	0,72	0	0,23	0	0,76	0,01	0,046
2,5	7089	0,05	0	0	0	0,11	0	0	0,8	0,04	0,07	0	0,09	0,84	0,048
2,5	7327	0	0	0	0,06	0	0	0,02	0,92	0	0,06	0	0,02	0,92	0,046
2,5	49153	0,02	0,05	0	0	0,93	0	0	0	0	0,93	0,05	0,02	0	0,046
2,5	30853	0	0	0	0,07	0,93	0	0	0	0	0,01	0,92	0,07	0	0,042
2,5	20302	0	0	0	0,06	0,38	0	0,01	0,55	0	0,01	0,02	0,34	0,63	0,046
2,5	23428	0	0,94	0	0,01	0	0,05	0	0	0	0	0	0,05	0,95	0,048
2,5	28006	0	0	0	0,22	0,72	0,02	0,01	0	0,03	0,87	0,13	0	0	0,042
2,5	12215	0,04	0	0,04	0	0,7	0	0	0	0,22	0	0,73	0,23	0,04	0,038
2,5	30975	0	0,13	0	0	0,61	0,26	0	0	0	0,03	0	0,83	0,14	0,044
2,5	22583	0,06	0	0	0	0,29	0,08	0	0,57	0	0	0,02	0,37	0,61	0,048
2,5	10098	0,06	0	0	0	0,92	0	0,01	0	0,01	0	0,01	0,93	0,06	0,048
2,75	10985	0,06	0	0	0	0,91	0,03	0	0	0	0,89	0,03	0,02	0,06	0,048
2,75	58352	0,05	0	0,01	0	0	0	0	0,94	0	0	0,05	0,94	0,01	0,042
2,75	18321	0,04	0	0,01	0,03	0,72	0	0	0,2	0	0,72	0,23	0,01	0,04	0,044

2,75	31990	0	0	0	0,06	0,93	0	0	0,01	0	0,07	0	0	0,93	0,048
2,75	40515	0	0,27	0,02	0	0,71	0	0	0	0	0,27	0	0,02	0,71	0,042
2,75	48211	0,06	0,01	0	0	0	0	0	0,9	0,03	0,03	0,87	0,03	0,07	0,048
2,75	19484	0	0,06	0	0,06	0,78	0,06	0	0,04	0	0,82	0,06	0,05	0,07	0,048
2,75	13472	0,06	0	0	0	0,94	0	0	0	0	0	0,07	0,93	0	0,048
2,75	9125	0,06	0,01	0	0	0,9	0	0	0,02	0,01	0	0,93	0,01	0,06	0,046
2,75	24353	0,05	0	0,01	0	0,03	0,23	0	0,11	0,57	0,15	0,71	0,13	0,01	0,04
2,75	18139	0	0	0	0,06	0,94	0	0	0	0	0	0	0,06	0,94	0,048
2,75	5791	0,05	0	0	0	0,94	0,01	0	0	0	0,94	0,02	0,04	0	0,044
2,75	15832	0	0	0,01	0,07	0	0	0	0,9	0,02	0	1	0	0	0,048
2,75	38299	0	0,19	0	0,04	0,67	0	0	0,1	0	0	0,23	0,1	0,67	0,046
2,75	9027	0,05	0	0,02	0,01	0,91	0	0	0,01	0	0,01	0,02	0,04	0,93	0,048
2,75	8822	0	0,09	0	0,05	0	0,85	0,01	0	0	0,37	0	0,63	0	0,048
2,75	2970	0,01	0	0,04	0	0,62	0	0,01	0,32	0	0,29	0,38	0,01	0,32	0,024
2,75	28473	0,01	0,01	0,02	0,11	0,79	0	0,01	0,05	0	0,79	0	0,21	0	0,046
2,75	41210	0,06	0	0	0	0,72	0	0	0,2	0,02	0,72	0	0,02	0,26	0,046
2,75	48755	0,03	0,96	0	0	0,01	0	0	0	0	0	0,01	0	0,99	0,044
2,75	5091	0,05	0,05	0	0	0,9	0	0	0	0	0,05	0	0	0,95	0,034
2,75	13221	0	0	0	0,08	0,16	0,55	0	0,21	0	0	0,37	0,08	0,55	0,048
2,75	6356	0,02	0,01	0,04	0	0,31	0,6	0,02	0	0	0,02	0,36	0,62	0	0,032
2,75	6206	0	0,02	0,02	0,03	0	0,01	0,06	0,86	0	0	0,02	0,87	0,11	0,036
2,75	23482	0	0,28	0	0	0,71	0,01	0	0	0	0,28	0	0,01	0,71	0,04
2,75	42686	0,01	0	0	0,05	0,04	0	0	0,9	0	0	0,03	0	0,97	0,044
2,75	42575	0	0,99	0	0	0	0	0,01	0	0	0,01	0,19	0,04	0,76	0,048
2,75	8957	0	0	0,04	0	0,68	0,22	0,03	0,03	0	0,03	0,04	0,93	0	0,04
2,75	16041	0,04	0,06	0	0	0,9	0	0	0	0	0,01	0,73	0,17	0,09	0,048
2,75	9994	0,01	0	0	0,05	0,94	0	0	0	0	0,05	0,93	0,01	0,01	0,048
2,75	21826	0,04	0,07	0	0	0,89	0	0	0	0	0,14	0,82	0	0,04	0,048
2,75	18441	0	0,03	0	0,03	0,76	0,15	0,03	0	0	0,03	0,9	0	0,07	0,048
2,75	17276	0,05	0	0	0	0,51	0,44	0	0	0	0	0	0,58	0,42	0,046
2,75	9717	0,02	0	0	0,06	0,87	0,04	0	0	0,01	0,08	0,11	0,05	0,76	0,046
2,75	11547	0,02	0	0,01	0	0,89	0	0,05	0	0,03	0,03	0,95	0,02	0	0,04
2,75	13998	0,03	0	0,02	0	0,63	0	0,04	0,24	0,04	0	0,34	0,63	0,03	0,038

2,75	30535	0	0,2	0	0	0,79	0	0	0	0,01	0,2	0,79	0	0,01	0,042
2,75	13268	0	0	0	0,06	0	0,03	0,05	0,86	0	0,05	0,86	0,09	0	0,048
2,75	14804	0,02	0,18	0	0	0,78	0,02	0	0	0	0,19	0	0,02	0,79	0,042
2,75	17544	0,06	0	0	0	0,05	0,88	0,01	0	0	0,11	0,01	0,88	0	0,048
2,75	6623	0,06	0,02	0,01	0	0	0	0	0,91	0	0	0	0,01	0,99	0,046
2,75	27610	0	0	0	0,06	0,94	0	0	0	0	0,06	0	0	0,94	0,046
2,75	63301	0	0,14	0	0	0,86	0	0	0	0	0	0	0,85	0,15	0,048
2,75	48113	0	0,27	0	0,06	0	0	0	0,67	0	0	0,06	0	0,94	0,048
2,75	26140	0	0,12	0	0	0,17	0,71	0	0	0	0,29	0,02	0,69	0	0,042
2,75	16089	0,03	0,12	0	0	0,85	0	0	0	0	0	0,85	0,15	0	0,032
2,75	6438	0	0	0,04	0	0,46	0	0	0,45	0,05	0,47	0,05	0,48	0	0,028
2,75	5213	0	0	0	0,21	0,78	0,01	0	0	0	0,78	0,21	0,01	0	0,046
2,75	16189	0,03	0	0	0,07	0,89	0	0,01	0	0	0,94	0	0,06	0	0,046
2,75	41478	0	0,27	0	0	0,73	0	0	0	0	0,27	0	0,01	0,72	0,044
2,75	21871	0,03	0	0	0,01	0,81	0	0,05	0,1	0	0,79	0,06	0,15	0	0,046
2,75	19255	0,04	0,04	0,02	0	0,88	0	0	0,02	0	0,02	0,02	0,04	0,92	0,042
2,75	6176	0	0	0	0,29	0,68	0,03	0	0	0	0,97	0	0	0,03	0,04
2,75	30939	0	0	0	0,26	0,74	0	0	0	0	1	0	0	0	0,044
2,75	8569	0,02	0	0,04	0	0	0	0	0,93	0,01	0	0,05	0,94	0,01	0,022
2,75	12390	0,04	0	0	0,02	0	0	0	0,94	0	0	0,96	0,02	0,02	0,042
2,75	19694	0	0,07	0	0,04	0	0,86	0,03	0	0	0,03	0,85	0,02	0,1	0,042
2,75	3887	0	0,84	0,04	0,04	0,07	0	0	0,01	0	0	0,18	0,01	0,81	0,048
2,75	6642	0,02	0	0	0	0,3	0,01	0,1	0,5	0,07	0,65	0,15	0,08	0,12	0,048
2,75	5375	0,05	0	0	0,01	0,89	0,04	0	0	0,01	0,23	0,68	0,06	0,03	0,042
2,75	3314	0,02	0	0,02	0	0,92	0	0,04	0	0	0,01	0,02	0,02	0,95	0,048
2,75	32740	0	0,04	0,01	0,07	0,85	0	0	0	0,03	0	0,88	0,11	0,01	0,044
2,75	6751	0,06	0	0	0	0,31	0,63	0	0	0	0,31	0,01	0,62	0,06	0,048
2,75	10948	0,06	0	0	0	0	0	0	0,94	0	0	0	0	1	0,042
2,75	32073	0	0	0,04	0,09	0,01	0,01	0	0,81	0,04	0,02	0,04	0,92	0,02	0,042
2,75	27556	0,01	0,06	0	0	0,83	0,01	0,02	0,07	0	0,83	0,12	0,02	0,03	0,046
2,75	45962	0	0,22	0	0	0,77	0	0,01	0	0	0	0,77	0,15	0,08	0,034
2,75	56054	0	0,04	0	0	0	0,74	0,07	0,15	0	0	0,04	0,8	0,16	0,046
2,75	20725	0	0,07	0	0,02	0,91	0	0	0	0	0,94	0,02	0	0,04	0,046

2,75	8018	0	0	0	0,18	0,74	0	0	0,08	0	0,82	0	0,04	0,14	0,048
2,75	5416	0,01	0	0	0	0,77	0,04	0,18	0	0	0,95	0	0	0,05	0,048
2,75	12364	0	0	0	0,18	0,81	0	0,01	0	0	0,78	0,01	0,18	0,03	0,032
2,75	29528	0	0,24	0	0	0,27	0	0	0	0,49	0,01	0,72	0,01	0,26	0,048
2,75	8026	0,02	0,17	0,02	0	0,73	0,06	0	0	0	0,08	0,9	0,02	0	0,046
2,75	17826	0,03	0	0	0,11	0,18	0,6	0	0,03	0,05	0,86	0,02	0,03	0,09	0,04
2,75	19942	0	0	0	0,16	0,7	0,14	0	0	0	0,3	0	0,7	0	0,042
2,75	20137	0	0,09	0	0,04	0,84	0	0	0,03	0	0	0,03	0,97	0	0,046
2,75	8889	0,04	0	0	0,04	0	0,88	0	0,04	0	0,04	0,03	0,87	0,06	0,048
2,75	17194	0,03	0,97	0	0	0	0	0	0	0	0	0	0	1	0,032
2,75	5101	0,02	0,03	0	0,04	0,72	0	0,03	0,16	0	0,75	0,2	0,04	0,01	0,048
2,75	41729	0	0,12	0,01	0	0,86	0	0	0,01	0	0,02	0,01	0,97	0	0,044
2,75	25515	0	0	0	0,09	0,83	0	0,03	0,05	0	0	0	0,83	0,17	0,046
2,75	11985	0,01	0,01	0,04	0,04	0,9	0	0	0	0	0,01	0,9	0,04	0,05	0,042
2,75	7038	0,02	0	0	0,06	0,91	0	0	0	0,01	0	0,03	0,97	0	0,048
2,75	9942	0	0,27	0,01	0	0,58	0	0	0,14	0	0,26	0,01	0,01	0,72	0,046
2,75	11778	0,01	0,08	0	0,03	0,88	0	0	0	0	0,03	0	0,96	0,01	0,044
2,75	24051	0	0,14	0	0	0,86	0	0	0	0	0,14	0	0,86	0	0,048
2,75	11208	0,02	0	0	0	0,95	0	0,03	0	0	0,05	0	0	0,95	0,036
2,75	20123	0	0,01	0	0,23	0,74	0	0	0,02	0	0,75	0,25	0	0	0,046
2,75	31901	0	0,02	0	0,07	0,91	0	0	0	0	0	0	0,07	0,93	0,048
2,75	22086	0	0	0,02	0,06	0,12	0	0	0,8	0	0,03	0,91	0	0,06	0,04
2,75	37324	0,03	0	0	0	0,95	0	0,02	0	0	0	0,03	0,02	0,95	0,048
2,75	15631	0,03	0,15	0	0	0	0	0	0,82	0	0	0,85	0,08	0,07	0,048
2,75	11926	0,05	0	0	0	0,95	0	0	0	0	0,95	0	0,05	0	0,042
2,75	47881	0,05	0	0,01	0	0,9	0,03	0,01	0	0	0,94	0	0,05	0,01	0,046
2,75	35856	0	0	0	0,12	0,87	0	0	0	0,01	0,08	0	0,91	0,01	0,046
2,75	14156	0,06	0	0	0	0	0	0,01	0,93	0	0,01	0,06	0,93	0	0,028
2,75	27281	0,03	0	0	0,09	0,88	0	0	0	0	0,72	0,28	0	0	0,042
2,75	10443	0	0,12	0	0	0,86	0,01	0	0,01	0	0,86	0	0,13	0,01	0,048
2,75	7298	0,06	0	0	0	0,24	0,12	0	0,58	0	0,24	0	0,64	0,12	0,048
3	7775	0,03	0	0	0,08	0,88	0	0,01	0	0	0,79	0,05	0,08	0,08	0,042
3	32038	0,04	0,45	0	0	0,49	0	0,02	0	0	0,02	0	0	0,98	0,046

3	11867	0	0	0	0,12	0,87	0	0	0,01	0	0	0	0,88	0,12	0,046
3	8687	0,06	0,01	0	0	0,52	0,14	0	0,27	0	0	0,52	0,15	0,33	0,048
3	34095	0	0	0	0	0,84	0,09	0,07	0	0	0	0,84	0,09	0,07	0,042
3	20624	0,02	0,05	0	0,02	0,04	0,17	0	0,7	0	0,39	0,26	0,02	0,33	0,042
3	7183	0	0,43	0	0	0,55	0	0,02	0	0	0,05	0,43	0	0,52	0,048
3	22853	0,04	0	0,01	0,01	0,94	0	0	0	0	0,02	0,94	0,04	0	0,042
3	9413	0	1	0	0	0	0	0	0	0	0	0	0,05	0,95	0,036
3	14393	0,03	0	0	0	0,94	0	0,03	0	0	0	0,94	0,06	0	0,046
3	22519	0,05	0	0	0	0,91	0	0,01	0,03	0	0,03	0,01	0	0,96	0,044
3	12130	0	0	0,04	0	0	0,35	0	0,52	0,09	0,52	0,02	0,09	0,37	0,016
3	32592	0	0	0	0,06	0,87	0,02	0,01	0,04	0	0	0,97	0,03	0	0,046
3	19228	0,06	0	0	0	0,94	0	0	0	0	0,03	0,19	0	0,78	0,04
3	20944	0	0,96	0	0	0	0	0,04	0	0	0	0	0,01	0,99	0,048
3	35204	0	0,04	0	0,05	0,01	0	0	0,85	0,05	0,05	0,85	0,06	0,04	0,048
3	25691	0,04	0	0	0,02	0	0	0	0,94	0	0,04	0,94	0	0,02	0,042
3	12476	0,06	0	0,01	0,02	0,91	0	0	0	0	0,9	0,02	0,01	0,07	0,044
3	17087	0	0	0	0,19	0,81	0	0	0	0	0,7	0,1	0,2	0	0,044
3	15787	0	0	0	0,08	0,67	0	0	0,24	0,01	0,24	0,01	0,08	0,67	0,048
3	17742	0,01	0	0	0,04	0	0	0,01	0,94	0	0	0,98	0,01	0,01	0,044
3	11063	0	0,25	0	0,02	0,73	0	0	0	0	0	0,02	0,25	0,73	0,046
3	17800	0	0	0	0,07	0,92	0,01	0	0	0	0	0,93	0,07	0	0,044
3	11195	0,04	0,09	0,03	0	0	0	0	0,84	0	0,04	0,03	0,09	0,84	0,044
3	15757	0	0,04	0	0,06	0	0	0	0,9	0	0,04	0,96	0	0	0,048
3	27596	0,06	0	0	0	0,93	0	0	0,01	0	0,93	0,07	0	0	0,044
3	7798	0	0	0	0	0,74	0	0,26	0	0	0,75	0,25	0	0	0,044
3	22151	0	0	0	0,06	0,94	0	0	0	0	0	0	0,06	0,94	0,044
3	9245	0,04	0	0	0,04	0,91	0	0	0	0,01	0,02	0,07	0,91	0	0,046
3	10836	0	0,1	0	0	0,3	0,04	0,02	0,27	0,27	0,27	0,04	0,29	0,4	0,04
3	21567	0,05	0	0,01	0	0,88	0,06	0	0	0	0,05	0,07	0	0,88	0,042
3	37215	0	0,1	0	0	0,84	0	0	0,05	0,01	0,67	0,01	0,31	0,01	0,046
3	15962	0,04	0,06	0	0,02	0,87	0	0	0,01	0	0,08	0,02	0,03	0,87	0,048
3	21698	0,01	0,1	0	0	0,73	0,02	0	0,14	0	0	0,12	0,87	0,01	0,044
3	26211	0,01	0,06	0	0	0,93	0	0	0	0	0,99	0	0,01	0	0,048

3	29510	0,05	0	0	0	0,9	0,05	0	0	0	0,05	0,9	0	0,05	0,046
3	50209	0	0	0,04	0	0,95	0	0	0	0,01	0	0,01	0,99	0	0,026
3	18798	0	0,02	0,03	0,06	0,48	0,36	0	0,05	0	0,01	0,02	0,38	0,59	0,048
3	26817	0	0,29	0	0	0,71	0	0	0	0	0	0	0,29	0,71	0,044
3	26836	0,04	0	0	0	0,02	0	0,06	0,87	0,01	0,83	0	0,05	0,12	0,042
3	18360	0	0,31	0	0	0,58	0	0	0,11	0	0,31	0	0,02	0,67	0,046
3	38364	0	0	0,01	0,06	0,83	0,06	0	0	0,04	0,09	0,04	0,04	0,83	0,048
3	27971	0	0,86	0	0	0,12	0	0	0,02	0	0,02	0	0,08	0,9	0,046
3	27511	0	0	0	0,09	0,89	0	0,01	0,01	0	0,01	0	0,89	0,1	0,046
3	4256	0,01	0	0,02	0	0	0,01	0,07	0,89	0	0,01	0,07	0,03	0,89	0,048
3	18061	0,01	0,17	0	0,03	0,65	0	0	0,14	0	0,01	0,14	0,01	0,84	0,044
3	40949	0,05	0,02	0	0	0,81	0,12	0	0	0	0	0,05	0,02	0,93	0,046
3	40640	0,06	0	0,01	0	0,78	0	0	0,15	0	0	0	0,01	0,99	0,048
3	24721	0	0,02	0	0,09	0	0,89	0	0	0	0,09	0,27	0,58	0,06	0,048
3	6497	0,06	0	0	0	0	0,01	0	0,92	0,01	0,92	0	0,01	0,07	0,042
3	10883	0,06	0	0	0	0,86	0	0	0	0,08	0	0	0,08	0,92	0,046
3	22216	0,02	0,11	0	0,01	0,86	0	0	0	0	0	0,87	0,13	0	0,044
3	49779	0	0	0,02	0,09	0,89	0	0	0	0	0	0	1	0	0,044
3	6666	0,01	0,98	0	0	0	0	0,01	0	0	0	0	0,02	0,98	0,044
3	14287	0	0,09	0,02	0,01	0,74	0	0	0	0,14	0,26	0	0,6	0,14	0,04
3	5836	0,02	0	0,04	0	0,94	0	0	0	0	0,8	0,18	0,02	0	0,048
3	23169	0	0	0	0,2	0,56	0,22	0,01	0,01	0	0,77	0,11	0,01	0,11	0,042
3	21960	0	0	0	0,14	0,81	0	0	0,05	0	0,64	0,2	0,14	0,02	0,046
3	16761	0,02	0	0	0,19	0	0,78	0	0	0,01	0,79	0	0,21	0	0,046
3	18695	0,02	0,17	0	0	0,81	0	0	0	0	0	0,85	0,15	0	0,048
3	8076	0,05	0,01	0	0	0	0	0	0,94	0	0	0,18	0,01	0,81	0,04
3	23734	0,01	0	0,04	0	0,07	0	0	0,88	0	0,01	0,92	0,07	0	0,03
3	33811	0,06	0	0	0	0,88	0,06	0	0	0	0,84	0,1	0	0,06	0,048
3	11589	0,01	0	0	0,14	0,6	0,23	0,02	0	0	0,78	0,02	0,18	0,02	0,048
3	3898	0,05	0	0,01	0,01	0,57	0	0	0,36	0	0,36	0,01	0,57	0,06	0,048
3	26295	0	0,26	0	0	0	0	0,01	0,73	0	0	0	0,26	0,74	0,046
3	7393	0,04	0	0,01	0,03	0,92	0	0	0	0	0,07	0,02	0	0,91	0,046
3	15310	0	0,05	0	0,06	0	0	0	0,89	0	0,04	0,01	0,06	0,89	0,048

3	12623	0,01	0	0	0,04	0,9	0,04	0	0	0,01	0	0,05	0,03	0,92	0,038
3	21718	0,07	0	0	0	0,93	0	0	0	0	0,93	0	0,07	0	0,048
3	23498	0	0	0	0,1	0,02	0,02	0,01	0,85	0	0,03	0,1	0,85	0,02	0,046
3	28305	0,06	0	0	0	0,59	0	0	0,28	0,07	0,02	0,65	0	0,33	0,046
3	36863	0	0,07	0	0	0,93	0	0	0	0	0,93	0	0,07	0	0,044
3	42531	0,01	0,19	0,01	0	0	0	0,04	0,75	0	0,02	0,95	0	0,03	0,048
3	34897	0	0,1	0	0	0,75	0,15	0	0	0	0	0,18	0,82	0	0,04
3	16807	0	0,03	0	0,09	0,86	0	0,02	0	0	0,86	0,09	0,03	0,02	0,042
3	5943	0,06	0	0	0	0,93	0	0	0,01	0	0,01	0,01	0,98	0	0,044
3	13487	0	0	0	0,06	0	0	0	0,94	0	0	0	0	1	0,046
3	23873	0,04	0,12	0	0	0	0,84	0	0	0	0,18	0,03	0,01	0,78	0,048
3	22166	0	0,16	0,03	0	0,79	0	0	0,01	0,01	0,03	0,79	0,16	0,02	0,044
3	5840	0	0,2	0,01	0	0,06	0,01	0	0,72	0	0	0,84	0,16	0	0,046
3	40926	0,04	0	0	0	0	0,65	0,04	0,23	0,04	0,04	0,29	0,67	0	0,048
3	12777	0	0,3	0	0	0,7	0	0	0	0	0,3	0	0	0,7	0,048
3	22406	0	0	0	0,1	0,9	0	0	0	0	0,1	0,9	0	0	0,048
3	31096	0,01	0	0,04	0,03	0,91	0,01	0	0	0	0,94	0	0,05	0,01	0,046
3	19470	0,05	0	0	0	0,95	0	0	0	0	0	0,01	0,99	0	0,038
3	27858	0,06	0	0	0	0,94	0	0	0	0	0	0	0,94	0,06	0,048
3	44498	0	0,12	0	0	0,83	0,04	0	0	0,01	0,12	0,04	0,79	0,05	0,044
3	14782	0,04	0,02	0	0	0,83	0,07	0	0	0,04	0,02	0,15	0,83	0	0,042
3	4488	0	0	0	0,2	0,8	0	0	0	0	0,72	0,13	0,14	0,01	0,046
3	7551	0,05	0,01	0	0	0,94	0	0	0	0	0,93	0	0,07	0	0,048
3	23364	0,07	0	0	0	0,85	0,08	0	0	0	0,85	0,15	0	0	0,046
3	41907	0,01	0	0	0,06	0,89	0,04	0	0	0	0,06	0	0	0,94	0,048
3	22242	0	0	0	0,08	0,92	0	0	0	0	0,08	0,92	0	0	0,042
3	9415	0,05	0	0	0,01	0,94	0	0	0	0	0,94	0,05	0	0,01	0,038
3	29668	0	0	0	0,39	0	0,61	0	0	0	1	0	0	0	0,044
3	11442	0,06	0,03	0	0	0,89	0,02	0	0	0	0,03	0,91	0	0,06	0,044
3	15376	0,04	0	0,04	0	0	0	0	0,92	0	0	0	0,07	0,93	0,036
3	8652	0,01	0	0	0,2	0,77	0	0	0	0,02	0,98	0	0,01	0,01	0,04
3	25761	0,06	0	0	0	0	0,14	0	0,8	0	0,07	0	0,8	0,13	0,044
3,25	50889	0	0	0	0,11	0,82	0,05	0,02	0	0	0	0,05	0,95	0	0,048

3,25	8470	0,03	0,05	0	0	0,91	0	0,01	0	0	0,05	0	0,91	0,04	0,048
3,25	4910	0,02	0	0	0	0,86	0,01	0,05	0,01	0,05	0,06	0,85	0,09	0	0,034
3,25	24810	0,04	0	0	0	0	0,88	0,06	0	0,02	0,88	0,01	0,05	0,06	0,048
3,25	38069	0,03	0	0,04	0	0,55	0,04	0	0,3	0,04	0,04	0,06	0,02	0,88	0,038
3,25	10721	0,04	0	0,03	0	0,93	0	0	0	0	0,93	0,03	0	0,04	0,04
3,25	11781	0,03	0	0	0,08	0,79	0,1	0	0	0	0,92	0,08	0	0	0,044
3,25	14972	0	0	0	0,06	0	0,06	0	0,88	0	0	0,05	0,01	0,94	0,04
3,25	38277	0	0	0	0,09	0	0,02	0	0,86	0,03	0,04	0,85	0,11	0	0,044
3,25	25679	0	0,05	0	0,08	0	0,87	0	0	0	0,87	0	0,08	0,05	0,046
3,25	15631	0,06	0	0	0	0	0	0	0,94	0	0	0,06	0	0,94	0,048
3,25	28603	0	0,02	0	0,08	0,89	0,01	0	0	0	0,02	0,9	0,08	0	0,048
3,25	25209	0	0	0	0,34	0,66	0	0	0	0	1	0	0	0	0,038
3,25	20363	0,04	0	0	0	0	0	0,02	0,93	0,01	0,02	0,93	0,04	0,01	0,038
3,25	10619	0,05	0	0,01	0	0,91	0,03	0	0	0	0	0,03	0,92	0,05	0,046
3,25	20234	0,01	0,04	0	0,04	0,89	0	0	0,02	0	0	0,04	0,95	0,01	0,036
3,25	35988	0	0	0	0,11	0	0	0	0,89	0	0	0,11	0,89	0	0,048
3,25	7895	0,04	0	0	0	0,76	0	0,02	0,16	0,02	0	0,09	0,03	0,88	0,046
3,25	6684	0,02	0,03	0	0,11	0,81	0	0	0	0,03	0,93	0	0,02	0,05	0,038
3,25	17665	0,04	0	0,04	0	0,92	0	0	0	0	0	0,04	0,96	0	0,042
3,25	21918	0	0,96	0	0,02	0	0,02	0	0	0	0	0,19	0,02	0,79	0,038
3,25	14390	0,04	0	0,04	0	0,02	0,9	0	0	0	0,04	0	0,94	0,02	0,046
3,25	18486	0	0	0	0,22	0,76	0	0,01	0,01	0	0,76	0,22	0	0,02	0,046
3,25	23645	0	0	0	0,12	0	0,1	0	0,78	0	0,12	0	0,84	0,04	0,04
3,25	22896	0	0,99	0,01	0	0	0	0	0	0	0	0	0,06	0,94	0,046
3,25	7350	0,04	0	0,03	0	0,92	0	0,01	0	0	0,01	0,06	0,01	0,92	0,046
3,25	68514	0,05	0	0	0	0,9	0	0	0	0,05	0,05	0,89	0,01	0,05	0,044
3,25	39179	0,05	0,03	0	0	0,46	0,01	0	0,45	0	0,03	0,49	0,46	0,02	0,044
3,25	8513	0	0,48	0,01	0	0	0	0,04	0,47	0	0	0,52	0	0,48	0,048
3,25	33936	0	0	0,01	0,25	0	0,74	0	0	0	0,99	0	0,01	0	0,04
3,25	17597	0,04	0	0,01	0	0,89	0	0	0,03	0,03	0,89	0,06	0,01	0,04	0,044
3,25	9720	0,05	0,01	0	0	0,93	0	0	0,01	0	0,28	0	0	0,72	0,046
3,25	15987	0,04	0	0,02	0	0,94	0	0	0	0	0,93	0,04	0,02	0,01	0,048
3,25	10840	0,03	0	0	0,04	0,72	0	0,01	0,2	0	0,11	0,35	0,4	0,14	0,044

3,25	38622	0,05	0	0	0	0,8	0	0,01	0,12	0,02	0,02	0,8	0,01	0,17	0,048
3,25	16072	0	0,19	0	0	0,75	0	0	0,06	0	0,19	0,06	0,29	0,46	0,046
3,25	22847	0,05	0	0	0	0,02	0	0	0,91	0,02	0,03	0,01	0,92	0,04	0,048
3,25	10579	0	0	0	0,24	0,74	0	0	0	0,02	0,74	0,24	0,02	0	0,046
3,25	5205	0,04	0	0,02	0,01	0,93	0	0	0	0	0,94	0,02	0	0,04	0,042
3,25	24311	0	0,01	0	0,04	0,91	0,02	0,02	0	0	0,06	0,93	0	0,01	0,044
3,25	16986	0	0,16	0	0,02	0,77	0,05	0	0	0	0,02	0,77	0,16	0,05	0,04
3,25	25637	0	0,01	0,01	0,14	0,83	0,01	0	0	0	0,86	0,01	0,01	0,12	0,048
3,25	46222	0	0	0	0,15	0,85	0	0	0	0	0,58	0	0,42	0	0,04
3,25	28174	0,04	0	0,03	0	0,01	0,92	0	0	0	0,04	0,25	0,71	0	0,046
3,25	23638	0	0,08	0	0	0	0	0	0,92	0	0,86	0	0,14	0	0,048
3,25	32345	0,06	0,02	0	0	0,29	0	0	0,62	0,01	0,01	0,35	0,02	0,62	0,048
3,25	26130	0,06	0	0	0	0,34	0	0	0,6	0	0,26	0	0,68	0,06	0,044
3,25	15552	0	0	0,04	0	0	0	0	0,96	0	0	0,99	0,01	0	0,006
3,25	31137	0	0,08	0,01	0	0,9	0	0	0,01	0	0,9	0,01	0,09	0	0,044
3,25	25071	0	0,13	0	0	0,83	0	0,01	0	0,03	0,47	0,01	0,38	0,14	0,048
3,25	6801	0,06	0	0	0,02	0,28	0,64	0	0	0	0,35	0	0,64	0,01	0,048
3,25	45168	0,01	0	0,01	0,01	0,85	0,06	0,06	0	0	0,01	0,04	0,83	0,12	0,046
3,25	11803	0,06	0	0	0	0,75	0,18	0	0	0,01	0,76	0,06	0,18	0	0,044
3,25	28179	0,06	0	0	0	0,48	0,19	0	0,27	0	0,23	0,02	0,48	0,27	0,044
3,25	32692	0	0,04	0	0,07	0,72	0,17	0	0	0	0	0	0,92	0,08	0,046
3,25	30246	0	0	0	0,02	0	0,45	0,1	0,43	0	0	0,02	0,95	0,03	0,04
3,25	15985	0,05	0	0,01	0	0,02	0	0	0,92	0	0,01	0	0,02	0,97	0,044
3,25	29380	0,01	0	0	0,07	0	0,55	0,01	0	0,36	0,08	0,01	0,91	0	0,048
3,25	9061	0,04	0	0	0	0,94	0,02	0	0	0	0,94	0,04	0,01	0,01	0,04
3,25	7511	0,01	0	0,04	0	0,9	0	0,04	0,01	0	0	0,96	0	0,04	0,048
3,25	34334	0	0	0	0,12	0,88	0	0	0	0	0,27	0	0,73	0	0,042
3,25	30065	0,03	0,03	0	0	0,45	0,44	0	0,05	0	0,05	0,44	0,5	0,01	0,048
3,25	16797	0	0	0	0,06	0	0	0	0,94	0	0	0,06	0	0,94	0,048
3,25	22214	0	0,08	0	0,05	0	0	0	0,87	0	0	0,93	0,02	0,05	0,048
3,25	9811	0,06	0	0,02	0	0,62	0,29	0	0	0,01	0,62	0	0,32	0,06	0,048
3,25	9206	0,06	0	0,04	0	0,55	0,31	0	0,04	0	0	0,12	0,01	0,87	0,046
3,25	15771	0,01	0	0,01	0,06	0,3	0,62	0	0	0	0,08	0	0,45	0,47	0,04

3,25	13357	0	0	0,04	0	0,96	0	0	0	0	0	1	0	0	0,006
3,25	5679	0,05	0,75	0	0	0,2	0	0	0	0	0	0	0	1	0,046
3,25	4179	0	0,25	0	0,01	0,74	0	0	0	0	0	0,47	0,25	0,28	0,048
3,25	17863	0,04	0,96	0	0	0	0	0	0	0	0	0	0	1	0,046
3,25	7986	0,01	0	0,04	0,01	0	0	0	0,93	0,01	0,08	0,89	0	0,03	0,024
3,25	20292	0,03	0	0	0,04	0,91	0	0,02	0	0	0	0,03	0,95	0,02	0,048
3,25	17827	0	0	0	0,09	0,91	0	0	0	0	0,09	0,91	0	0	0,048
3,25	31767	0,05	0	0	0	0,93	0,02	0	0	0	0,93	0,04	0	0,03	0,048
3,25	9167	0	0,14	0	0,04	0,82	0	0	0	0	0,05	0	0,12	0,83	0,038
3,25	20609	0	0	0,04	0	0,93	0	0,03	0	0	0,93	0,04	0	0,03	0,032
3,25	26576	0	0,87	0	0,02	0	0,07	0	0,04	0	0,04	0,09	0	0,87	0,042
3,25	16507	0	0,36	0,02	0,03	0,18	0	0	0,41	0	0	0,41	0,03	0,56	0,048
3,25	7798	0,02	0,01	0,01	0,06	0,9	0	0	0	0	0,08	0,01	0,9	0,01	0,044
3,25	12034	0,06	0	0	0	0,93	0	0	0,01	0	0	0	0,06	0,94	0,048
3,25	27352	0,05	0	0	0	0	0,07	0	0,88	0	0	0,95	0	0,05	0,046
3,25	35389	0,05	0,01	0	0	0,91	0	0	0	0,03	0	0,97	0,03	0	0,048
3,25	28190	0,04	0	0	0,01	0,84	0	0	0,11	0	0,02	0,88	0	0,1	0,046
3,25	19909	0,02	0	0,02	0,03	0,91	0	0	0	0,02	0,32	0,63	0	0,05	0,04
3,25	11681	0	0,14	0	0,03	0	0	0	0,83	0	0,03	0	0,14	0,83	0,044
3,25	21883	0,01	0,98	0	0	0,01	0	0	0	0	0,01	0	0,04	0,95	0,034
3,25	11898	0	0,01	0	0,11	0	0,27	0	0,61	0	0,26	0,01	0,11	0,62	0,048
3,25	9329	0,06	0,04	0	0	0,9	0	0	0	0	0,09	0	0,03	0,88	0,048
3,25	12566	0,04	0,9	0	0	0	0	0	0,06	0	0	0,03	0	0,97	0,048
3,25	19198	0,05	0	0	0	0,01	0	0,01	0,92	0,01	0,05	0	0,01	0,94	0,046
3,25	30504	0,04	0,15	0	0	0,37	0	0	0,44	0	0	0,96	0,04	0	0,048
3,25	38866	0	0,03	0,01	0,04	0,91	0	0,01	0	0	0,03	0,96	0	0,01	0,042
3,25	1875	0,02	0	0,04	0	0,9	0	0,04	0	0	0,04	0,04	0,9	0,02	0,048
3,25	17470	0	0,1	0,04	0	0,81	0	0	0	0,05	0,07	0,07	0,81	0,05	0,046
3,25	27607	0	0,22	0	0	0,08	0	0	0,69	0,01	0,01	0	0,36	0,63	0,044
3,25	11935	0,02	0	0	0,02	0	0,44	0,05	0,47	0	0,5	0	0,02	0,48	0,044
3,25	25918	0,06	0	0	0	0,67	0	0	0,27	0	0,06	0,67	0,27	0	0,04
3,25	7715	0,05	0,02	0,03	0	0,9	0	0	0	0	0,01	0,01	0,91	0,07	0,04
3,25	30981	0	0,36	0	0,01	0,63	0	0	0	0	0,01	0,01	0,24	0,74	0,042

3,5	9488	0,06	0	0	0	0,88	0	0	0,06	0	0,06	0,47	0,41	0,06	0,048
3,5	26887	0	0	0	0,05	0,91	0,01	0	0	0,03	0,06	0,03	0	0,91	0,042
3,5	25748	0,04	0,09	0	0	0,78	0	0	0,03	0,06	0,09	0,88	0	0,03	0,048
3,5	8387	0,01	0	0,01	0,14	0,81	0	0	0,02	0,01	0,64	0,04	0,23	0,09	0,046
3,5	27194	0,05	0	0	0	0,74	0,01	0	0,2	0	0	0	0,96	0,04	0,038
3,5	13883	0,06	0	0,01	0	0,88	0,05	0	0	0	0,01	0,88	0,05	0,06	0,042
3,5	7618	0,05	0	0,03	0	0,9	0,02	0	0	0	0	0,02	0,93	0,05	0,048
3,5	60444	0	0	0	0,06	0,93	0	0	0	0,01	0,06	0,93	0,01	0	0,042
3,5	13382	0,02	0	0	0,02	0,62	0	0,02	0,32	0	0,32	0,04	0,02	0,62	0,048
3,5	10689	0,06	0	0	0	0	0,9	0	0,01	0,03	0,99	0	0	0,01	0,048
3,5	18836	0	0,13	0	0	0,87	0	0	0	0	0	0	1	0	0,048
3,5	10298	0	0	0	0,01	0	0,54	0,23	0,05	0,17	0,72	0,23	0,04	0,01	0,048
3,5	18215	0,02	0,03	0,01	0	0,94	0	0	0	0	0,96	0,01	0,03	0	0,046
3,5	44754	0,06	0	0	0	0	0,87	0,02	0,04	0,01	0,86	0,04	0,08	0,02	0,048
3,5	16376	0	0,18	0	0	0,05	0,06	0,01	0,07	0,63	0,21	0,15	0,06	0,58	0,046
3,5	5465	0,03	0,09	0,01	0	0,87	0	0	0	0	0,04	0,96	0	0	0,044
3,5	9694	0,05	0,02	0	0	0,04	0	0	0,82	0,07	0,07	0,04	0,82	0,07	0,046
3,5	5498	0,07	0	0	0	0	0	0	0,93	0	0	0,96	0	0,04	0,048
3,5	29119	0	0,01	0	0,07	0,92	0	0	0	0	0	0,99	0,01	0	0,044
3,5	8600	0,05	0	0	0,06	0,88	0	0	0,01	0	0,81	0	0,07	0,12	0,046
3,5	15791	0,03	0	0,01	0,04	0,91	0	0,01	0	0	0,04	0	0,91	0,05	0,048
3,5	6095	0,01	0	0	0,09	0,87	0,02	0	0,01	0	0,09	0,13	0,76	0,02	0,044
3,5	13809	0,05	0	0,01	0	0,49	0,34	0,03	0,08	0	0,03	0,83	0,14	0	0,04
3,5	11672	0,05	0,04	0,04	0	0,85	0	0,01	0,01	0	0	0,45	0,02	0,53	0,048
3,5	11299	0,04	0	0	0,03	0,92	0	0,01	0	0	0,21	0	0	0,79	0,046
3,5	28987	0,01	0,97	0	0	0,01	0,01	0	0	0	0,01	0,01	0,04	0,94	0,044
3,5	37383	0	0,26	0	0	0,73	0	0	0	0,01	0,24	0,04	0,02	0,7	0,048
3,5	43945	0	0,11	0	0,01	0,86	0	0,02	0	0	0,13	0,87	0	0	0,048
3,5	9056	0	0,29	0,01	0	0,7	0	0	0	0	0,29	0,01	0	0,7	0,048
3,5	11695	0,07	0,01	0,02	0	0,7	0,11	0	0,07	0,02	0,09	0,02	0,01	0,88	0,048
3,5	21288	0,02	0	0,04	0,01	0,82	0,1	0	0	0,01	0,06	0,02	0,86	0,06	0,048
3,5	25383	0,06	0	0	0	0,51	0,42	0	0,01	0	0,41	0,54	0,05	0	0,046
3,5	8191	0,06	0	0,01	0,01	0,9	0	0	0,02	0	0	0,92	0,07	0,01	0,048

3,5	16046	0	0,23	0	0	0,77	0	0	0	0	0,22	0,68	0	0,1	0,046
3,5	12168	0,02	0,08	0	0	0,9	0	0	0	0	0,08	0,02	0,9	0	0,048
3,5	36243	0	0	0	0,1	0,05	0	0	0,85	0	0,04	0,85	0,11	0	0,048
3,5	16830	0,01	0,14	0,01	0,03	0,76	0,01	0	0,04	0	0	0,01	0,08	0,91	0,032
3,5	23803	0,06	0	0,01	0	0,89	0,04	0	0	0	0,89	0,01	0,1	0	0,048
3,5	16486	0,04	0	0	0	0,95	0	0,01	0	0	0,03	0,02	0,95	0	0,04
3,5	21805	0,07	0	0	0	0,91	0,02	0	0	0	0,03	0,9	0	0,07	0,044
3,5	44306	0,05	0,07	0	0,01	0,84	0,01	0	0,02	0	0	0,08	0,08	0,84	0,048
3,5	22897	0,05	0	0	0	0,7	0,1	0	0,15	0	0,7	0	0,1	0,2	0,04
3,5	7646	0	0,32	0	0	0,68	0	0	0	0	0	0	0,25	0,75	0,044
3,5	51031	0	0,19	0	0,01	0,79	0	0,01	0	0	0	0,5	0,3	0,2	0,048
3,5	92914	0	0	0	0,15	0,3	0,54	0	0,01	0	0,44	0,01	0,55	0	0,048
3,5	11646	0	0	0	0,07	0,93	0	0	0	0	0,07	0,93	0	0	0,042
3,5	10087	0,05	0,01	0,04	0	0,65	0,01	0	0,24	0	0,05	0,7	0,25	0	0,046
3,5	21243	0,05	0	0	0	0,93	0,02	0	0	0	0,02	0,93	0,05	0	0,04
3,5	20864	0,06	0,01	0,02	0	0,87	0	0	0,02	0,02	0,01	0,91	0,06	0,02	0,048
3,5	15928	0	0,07	0	0	0,93	0	0	0	0	1	0	0	0	0,048
3,5	11795	0	0	0	0,09	0,84	0	0	0	0,07	0,39	0	0	0,61	0,04
3,5	17568	0,04	0	0	0,02	0,94	0	0	0	0	0	0,94	0	0,06	0,046
3,5	24468	0,01	0,94	0	0	0	0,01	0,02	0,01	0,01	0,02	0,03	0	0,95	0,046
3,5	33524	0,01	0,24	0	0	0,54	0,03	0	0,05	0,13	0,24	0,05	0,02	0,69	0,048
3,5	24674	0,06	0	0	0	0,21	0	0	0,01	0,72	0,22	0,72	0,06	0	0,046
3,5	29450	0	0	0	0,07	0,84	0	0	0,09	0	0	0,91	0,09	0	0,046
3,5	25366	0,03	0	0	0,01	0	0,03	0	0,93	0	0,01	0	0,02	0,97	0,038
3,5	42745	0	0	0	0,08	0,91	0	0	0	0,01	0,05	0,91	0,03	0,01	0,048
3,5	21725	0	0,99	0	0	0,01	0	0	0	0	0,01	0,17	0,03	0,79	0,044
3,5	12098	0,01	0	0,01	0,17	0,43	0	0	0,38	0	0,81	0,17	0,01	0,01	0,044
3,5	16985	0,04	0	0,01	0	0	0,2	0	0,68	0,07	0,96	0	0	0,04	0,046
3,5	18100	0	0	0	0,18	0,82	0	0	0	0	0,71	0,18	0	0,11	0,046
3,5	29611	0	0,05	0	0,06	0,87	0,02	0	0	0	0,06	0,07	0	0,87	0,046
3,5	35523	0,01	0,96	0	0,03	0	0	0	0	0	0,01	0,03	0	0,96	0,046
3,5	8551	0,02	0	0	0	0	0,27	0,08	0,63	0	0,58	0,02	0	0,4	0,038
3,5	12005	0	0	0	0,05	0,95	0	0	0	0	0	0,95	0	0,05	0,042

3,5	12311	0	0	0,04	0	0,87	0	0	0,09	0	0,85	0,04	0,11	0	0,032
3,5	16633	0,04	0	0	0,04	0,85	0,07	0	0	0	1	0	0	0	0,048
3,5	57825	0,05	0	0	0	0,82	0,13	0	0	0	0,87	0	0	0,13	0,04
3,5	67533	0	0,15	0	0,05	0	0	0,01	0,79	0	0	0,2	0	0,8	0,046
3,5	25291	0,03	0	0,04	0,01	0,1	0,44	0,01	0,37	0	0,37	0,02	0,15	0,46	0,044
3,5	54273	0,01	0	0,04	0	0	0	0,05	0,9	0	0	0,99	0	0,01	0,046
3,5	8821	0	0,05	0,04	0	0,88	0	0,03	0	0	0,03	0,06	0,88	0,03	0,048
3,5	49016	0,04	0,01	0,03	0,01	0	0,71	0	0,2	0	0,01	0,2	0,04	0,75	0,048
3,5	6319	0,05	0	0	0,01	0,94	0	0	0	0	0,08	0,05	0,87	0	0,044
3,5	25286	0	0,28	0	0	0,71	0,01	0	0	0	0,28	0,08	0,01	0,63	0,044
3,5	30528	0,03	0,12	0	0,01	0,73	0	0,01	0,1	0	0,01	0,01	0,18	0,8	0,038
3,5	32715	0	0,15	0,02	0	0,59	0	0	0,2	0,04	0,17	0,66	0,17	0	0,044
3,5	22070	0	0	0	0,13	0,8	0	0	0	0,07	0	0,07	0,93	0	0,048
3,5	28026	0	0	0	0,1	0	0,06	0	0,83	0,01	0	0,01	0,83	0,16	0,042
3,5	15371	0	0,14	0	0,02	0,83	0	0,01	0	0	0,14	0,83	0	0,03	0,048
3,5	19942	0	0,12	0	0	0,88	0	0	0	0	0,07	0	0,93	0	0,046
3,5	17112	0,03	0	0,01	0	0,92	0	0	0,02	0,02	0,96	0,02	0	0,02	0,04
3,5	8699	0,06	0	0,03	0	0,9	0	0	0,01	0	0	0,94	0	0,06	0,046
3,5	54088	0	0	0	0,07	0,9	0,02	0	0	0,01	0,07	0,01	0	0,92	0,048
3,5	18016	0	0	0	0,27	0,72	0	0	0,01	0	0,95	0	0,05	0	0,042
3,5	10335	0,06	0	0,03	0	0,78	0,08	0	0	0,05	0	0,08	0,06	0,86	0,048
3,5	22493	0	0	0	0,11	0,5	0,38	0	0	0,01	0,01	0,38	0,61	0	0,044
3,5	44068	0	0,21	0,01	0	0,76	0	0,02	0	0	0,02	0,01	0,37	0,6	0,048
3,5	21965	0,01	0,35	0	0	0,56	0,01	0,01	0,06	0	0,01	0,36	0,06	0,57	0,046
3,5	23635	0,02	0,08	0,02	0	0,5	0	0	0,03	0,35	0,21	0,02	0,14	0,63	0,042
3,5	6595	0,04	0	0,02	0,01	0	0,01	0	0,92	0	0,02	0,01	0,01	0,96	0,044
3,5	59344	0,05	0	0,02	0	0,83	0	0	0,1	0	0,02	0	0,05	0,93	0,048
3,5	12432	0,02	0	0,01	0,03	0,94	0	0	0	0	0,01	0	0	0,99	0,046
3,5	28719	0,02	0,35	0	0,02	0,61	0	0	0	0	0	0,37	0,02	0,61	0,04
3,5	7634	0,06	0	0,01	0	0	0,93	0	0	0	0	0,02	0,92	0,06	0,048
3,5	22943	0,06	0	0	0,01	0,93	0	0	0	0	0,36	0	0,06	0,58	0,048
3,5	16293	0,07	0	0	0	0,85	0	0	0,04	0,04	0	0,89	0,04	0,07	0,048
3,5	12979	0,05	0,01	0	0,01	0,93	0	0	0	0	0,18	0,11	0,02	0,69	0,046

3,5	17934	0,05	0	0	0	0,87	0,01	0	0,07	0	0,05	0,07	0,87	0,01	0,046
3,75	12112	0,02	0	0,04	0	0,48	0	0	0,46	0	0	0,48	0,46	0,06	0,028
3,75	27908	0,07	0	0	0	0,42	0,5	0,01	0	0	0,56	0,02	0,38	0,04	0,048
3,75	9829	0,05	0	0	0,02	0	0	0	0,93	0	0	0,02	0,98	0	0,046
3,75	14077	0	0	0	0,12	0,68	0,2	0	0	0	0,11	0	0,82	0,07	0,046
3,75	9649	0	0,16	0	0	0,76	0,08	0	0	0	0,08	0	0,92	0	0,046
3,75	24377	0,04	0	0	0,01	0,88	0,06	0,01	0	0	0	0,02	0,94	0,04	0,042
3,75	10894	0,06	0	0	0	0	0,01	0	0,93	0	0	0,93	0,06	0,01	0,046
3,75	8934	0,06	0	0	0	0	0,05	0	0,87	0,02	0,02	0,57	0	0,41	0,036
3,75	22280	0	0	0,01	0,11	0,86	0,02	0	0	0	0,02	0	0,98	0	0,048
3,75	47406	0	0,07	0	0,06	0,85	0,02	0	0	0	0,07	0	0,91	0,02	0,032
3,75	4739	0,03	0	0,02	0,05	0,9	0	0	0	0	0,91	0,05	0,04	0	0,038
3,75	12187	0,06	0	0,01	0	0,93	0	0	0	0	0,93	0,01	0,01	0,05	0,048
3,75	29566	0,02	0,13	0,01	0	0,09	0,73	0	0,01	0,01	0,17	0,01	0	0,82	0,046
3,75	23808	0,01	0	0	0,14	0	0,72	0	0,13	0	0,55	0,14	0,17	0,14	0,046
3,75	8558	0,05	0,02	0	0	0	0	0	0,92	0,01	0	0,93	0,02	0,05	0,038
3,75	22678	0,01	0,22	0	0	0,37	0,23	0	0,17	0	0	0,23	0,28	0,49	0,046
3,75	23653	0,06	0,01	0	0	0,87	0	0	0,06	0	0,25	0,68	0	0,07	0,048
3,75	6838	0	0,01	0,04	0,01	0,89	0	0,03	0,02	0	0	0,06	0,93	0,01	0,044
3,75	20319	0	0	0	0,08	0,92	0	0	0	0	0	0	1	0	0,048
3,75	41388	0,03	0	0,04	0	0,93	0	0	0	0	0,93	0,03	0,01	0,03	0,046
3,75	54410	0	0,23	0,01	0,02	0,72	0	0,02	0	0	0,23	0,02	0,01	0,74	0,048
3,75	27544	0,02	0	0	0,04	0,01	0,02	0,01	0,9	0	0,02	0,92	0,02	0,04	0,048
3,75	13239	0,04	0,1	0,02	0	0,68	0	0	0,15	0,01	0,1	0,7	0	0,2	0,048
3,75	11526	0,01	0	0	0,3	0	0,02	0	0,67	0	0,88	0,12	0	0	0,046
3,75	23267	0	0	0,02	0,11	0	0,81	0	0,06	0	0,17	0,02	0,81	0	0,048
3,75	13787	0,01	0,07	0	0,03	0,87	0,01	0	0	0,01	0,06	0,86	0,08	0	0,036
3,75	26123	0	0,09	0	0	0,9	0	0	0,01	0	0,9	0	0,09	0,01	0,046
3,75	14284	0,06	0	0,01	0	0,9	0	0	0,03	0	0	0,91	0,03	0,06	0,044
3,75	10898	0	0,02	0	0,18	0,78	0,02	0	0	0	0,78	0,14	0,06	0,02	0,04
3,75	8373	0	0,03	0	0	0,74	0	0,11	0	0,12	0,44	0,01	0,13	0,42	0,048
3,75	12508	0	0,02	0	0,03	0,95	0	0	0	0	0	0,01	0	0,99	0,046
3,75	9074	0,01	0	0,01	0,14	0,79	0,04	0	0,01	0	0,79	0	0,2	0,01	0,048

3,75	33426	0,05	0	0	0	0,11	0	0,03	0,74	0,07	0,11	0,07	0,81	0,01	0,046
3,75	13155	0,04	0	0	0	0	0,87	0	0,09	0	0,06	0,01	0,06	0,87	0,038
3,75	57103	0	0	0	0,16	0,81	0	0	0	0,03	0,81	0	0	0,19	0,036
3,75	100000	0,04	0,02	0,92	0	0	0	0,02	0	0	0,92	0,03	0,02	0,03	0,9
3,75	24403	0	0	0,03	0	0	0	0,11	0,84	0,02	0,65	0,07	0,04	0,24	0,044
3,75	5534	0	0,12	0,01	0	0,85	0,01	0	0	0,01	0	0,02	0,98	0	0,04
3,75	5889	0,02	0	0,04	0,01	0	0	0	0,93	0	0	0,02	0,97	0,01	0,046
3,75	22827	0	0,2	0,01	0	0,74	0	0,04	0	0,01	0	0,92	0,03	0,05	0,046
3,75	13251	0	0,21	0,02	0	0,62	0,13	0	0	0,02	0,23	0,15	0,06	0,56	0,044
3,75	15573	0	0	0,04	0	0,43	0,49	0	0,04	0	0,87	0,07	0	0,06	0,042
3,75	22546	0,04	0,02	0,01	0	0,93	0	0	0	0	0	0,07	0,93	0	0,04
3,75	53522	0,05	0	0	0	0,92	0	0,01	0,01	0,01	0,01	0,96	0,02	0,01	0,048
3,75	16967	0	0,96	0,04	0	0	0	0	0	0	0,01	0	0	0,99	0,024
3,75	31526	0,04	0,19	0	0	0,76	0	0	0	0,01	0,01	0,04	0,1	0,85	0,046
3,75	23921	0	0	0,02	0,16	0,82	0	0	0	0	0,69	0,31	0	0	0,046
3,75	5276	0,04	0	0	0,07	0,06	0	0	0,83	0	0,28	0,72	0	0	0,044
3,75	42300	0,06	0	0	0	0,83	0,03	0	0,08	0	0	0,1	0,73	0,17	0,046
3,75	18961	0	0	0	0,09	0,49	0,06	0,01	0,32	0,03	0	0,09	0,71	0,2	0,048
3,75	23850	0,05	0	0	0	0,94	0	0	0	0,01	0,93	0,02	0	0,05	0,046
3,75	7309	0,02	0,97	0	0	0	0	0,01	0	0	0,01	0	0	0,99	0,046
3,75	5252	0,04	0,01	0	0,03	0,88	0,04	0	0	0	0,88	0,05	0,04	0,03	0,04
3,75	8463	0,01	0,05	0	0	0	0	0,05	0,72	0,17	0,85	0,05	0	0,1	0,048
3,75	29695	0	0	0,02	0,06	0,82	0,1	0	0	0	0	0,11	0,05	0,84	0,044
3,75	21462	0	0	0	0	0	0	0,06	0,91	0,03	0,03	0	0	0,97	0,048
3,75	9876	0	0	0	0	0,87	0	0,07	0,01	0,05	0,12	0,87	0	0,01	0,046
3,75	8590	0,02	0,16	0	0	0,8	0	0	0	0,02	0	0	0,2	0,8	0,048
3,75	15939	0,05	0,02	0,01	0	0,92	0	0	0	0	0,01	0,02	0,92	0,05	0,046
3,75	28162	0	0,14	0	0	0,86	0	0	0	0	0,14	0	0,86	0	0,048
3,75	43210	0	0	0,01	0,15	0,83	0,01	0	0	0	0,83	0,04	0,01	0,12	0,034
3,75	35344	0	0,07	0	0,04	0,87	0	0,02	0	0	0,13	0	0,87	0	0,036
3,75	13445	0	0,01	0,04	0	0,01	0	0,04	0,9	0	0,94	0,05	0,01	0	0,048
3,75	30635	0	0,03	0	0,09	0,87	0	0,01	0	0	0,03	0,01	0,87	0,09	0,048
3,75	11652	0,01	0,04	0,01	0	0	0	0,04	0,9	0	0,05	0,01	0	0,94	0,046

3,75	8435	0,02	0,07	0	0,01	0,88	0	0,02	0	0	0,01	0,08	0,02	0,89	0,036
3,75	6052	0,04	0	0,03	0	0,83	0	0,02	0,07	0,01	0,02	0,86	0,07	0,05	0,048
3,75	34704	0	0,06	0	0	0,94	0	0	0	0	1	0	0	0	0,048
3,75	13180	0,05	0	0	0	0,95	0	0	0	0	0	0	0,95	0,05	0,048
3,75	22154	0,06	0	0	0	0,86	0	0	0,08	0	0	0,96	0,04	0	0,036
3,75	17737	0,02	0	0,04	0	0	0	0	0,94	0	0	0,04	0,01	0,95	0,028
3,75	13712	0,02	0,14	0,02	0,01	0,81	0	0	0	0	0,14	0,02	0,01	0,83	0,038
3,75	34032	0	0,46	0	0,02	0,04	0,02	0	0,46	0	0,04	0,01	0,04	0,91	0,048
3,75	12640	0	0,02	0	0,18	0,76	0,04	0	0	0	0,77	0	0,23	0	0,048
3,75	11043	0	0,04	0	0,09	0,13	0,06	0	0,68	0	0,23	0	0,77	0	0,04
3,75	25956	0,04	0	0	0	0,94	0	0,01	0	0,01	0	0,95	0,04	0,01	0,046
3,75	13352	0,03	0	0,03	0	0,94	0	0	0	0	0,94	0,05	0	0,01	0,046
3,75	7492	0,07	0	0	0	0,91	0	0	0	0,02	0	0	0,98	0,02	0,04
3,75	10287	0,04	0	0,02	0	0,75	0,19	0	0	0	0,75	0	0,04	0,21	0,046
3,75	17239	0	0	0	0,07	0,93	0	0	0	0	0	0,92	0,07	0,01	0,048
3,75	34713	0	0	0	0,05	0,94	0	0,01	0	0	0,01	0,94	0	0,05	0,038
3,75	15531	0	0,6	0	0,03	0,37	0	0	0	0	0	0,37	0,03	0,6	0,038
3,75	23498	0,02	0	0,04	0	0,4	0	0	0,54	0	0	0,06	0,59	0,35	0,026
3,75	100000	0,47	0	0,5	0,03	0	0	0	0	0	0	0,49	0,33	0,18	0,742
3,75	4515	0,06	0	0	0	0	0	0	0,69	0,25	0,01	0	0,94	0,05	0,042
3,75	7953	0,05	0	0,02	0	0,91	0,02	0	0	0	0,02	0,18	0,8	0	0,042
3,75	18752	0	0,16	0	0	0,84	0	0	0	0	0	0,16	0,84	0	0,048
3,75	12232	0,05	0	0	0	0,95	0	0	0	0	0	0,05	0,95	0	0,04
3,75	10203	0,01	0	0,04	0	0,91	0	0	0,04	0	0,53	0,04	0,42	0,01	0,048
3,75	9390	0,02	0	0,02	0	0	0	0,03	0,85	0,08	0,03	0,87	0,08	0,02	0,048
3,75	7685	0,01	0	0	0,12	0,87	0	0	0	0	0,32	0	0,68	0	0,046
3,75	8970	0,04	0	0,02	0	0,92	0	0,02	0	0	0	0,04	0	0,96	0,046
3,75	10282	0	0	0,01	0,05	0,89	0	0,04	0,01	0	0,05	0	0,89	0,06	0,048
3,75	19108	0	0	0	0,06	0,94	0	0	0	0	0	1	0	0	0,048
3,75	29846	0,06	0	0	0	0	0,06	0	0,86	0,02	0,06	0,08	0,86	0	0,048
3,75	11870	0	0,2	0,01	0	0,79	0	0	0	0	0,04	0,79	0,17	0	0,044
3,75	76770	0,02	0	0	0,1	0	0	0	0,88	0	0	0	0,88	0,12	0,048
3,75	29942	0,05	0	0	0,02	0	0,82	0	0	0,11	0,07	0	0,93	0	0,046

3,75	46015	0	0,07	0	0,05	0	0	0,01	0,87	0	0	0,09	0,01	0,9	0,03
3,75	22029	0	0,08	0	0,03	0,06	0	0	0,83	0	0,54	0,05	0,34	0,07	0,046
4	21718	0,01	0,01	0	0,27	0,7	0	0,01	0	0	0,97	0	0,02	0,01	0,046
4	25715	0	0	0,04	0,05	0,83	0	0	0,08	0	0,01	0,05	0,08	0,86	0,048
4	26967	0,05	0	0	0,01	0,94	0	0	0	0	0,96	0,01	0	0,03	0,042
4	59899	0,03	0,2	0	0	0,66	0	0	0,11	0	0,22	0,12	0	0,66	0,048
4	31998	0,02	0,17	0,04	0	0,72	0	0	0	0,05	0,21	0,05	0	0,74	0,048
4	13032	0,01	0,07	0	0,03	0,89	0	0	0	0	0,94	0,03	0,03	0	0,048
4	13738	0,06	0	0	0	0,91	0	0	0	0,03	0	0,91	0,09	0	0,048
4	22495	0,06	0,04	0	0	0,9	0	0	0	0	0	0,96	0,04	0	0,044
4	64396	0	0	0,01	0,1	0,4	0	0	0,49	0	0	0,02	0,87	0,11	0,048
4	27958	0	0	0	0,11	0,07	0	0	0,82	0	0	0,07	0,92	0,01	0,044
4	8131	0,04	0	0,01	0,03	0,92	0	0	0	0	0,54	0,02	0,06	0,38	0,048
4	7977	0,04	0,96	0	0	0	0	0	0	0	0	0,01	0	0,99	0,046
4	13886	0	0	0,03	0,1	0,28	0,59	0	0	0	0	0,38	0,62	0	0,048
4	5288	0,03	0	0	0,02	0,08	0,47	0	0,4	0	0,03	0,47	0,08	0,42	0,04
4	14172	0	0,08	0,04	0,01	0,85	0	0,01	0,01	0	0,26	0	0,46	0,28	0,042
4	14749	0,06	0	0	0	0,07	0,76	0	0,11	0	0,15	0,72	0,13	0	0,046
4	19001	0,07	0	0	0	0	0,74	0	0	0,19	0,19	0	0,06	0,75	0,048
4	12095	0,05	0,06	0,02	0	0	0	0	0,87	0	0	0,05	0,04	0,91	0,046
4	13926	0,07	0	0	0	0,02	0,14	0	0,77	0	0,77	0,14	0	0,09	0,048
4	6193	0,04	0,05	0	0	0,9	0	0,01	0	0	0,05	0,9	0,04	0,01	0,046
4	18564	0,06	0	0	0	0,89	0,01	0	0,04	0	0,06	0,93	0,01	0	0,044
4	51593	0,01	0,08	0,02	0	0,88	0	0	0,01	0	0,12	0,04	0,76	0,08	0,04
4	26630	0,01	0,1	0	0,01	0,88	0	0	0	0	0	0,04	0,96	0	0,04
4	9203	0	0	0	0,12	0,01	0	0	0,84	0,03	0,01	0,12	0,84	0,03	0,048
4	20342	0,01	0	0	0	0,27	0,53	0,15	0	0,04	0,53	0,14	0,14	0,19	0,048
4	7472	0	0,11	0,03	0	0,65	0,11	0	0,1	0	0	0,03	0,96	0,01	0,048
4	21732	0	0	0	0,25	0,47	0,28	0	0	0	0,75	0	0,25	0	0,046
4	43891	0	0	0,02	0,03	0	0,02	0,03	0,9	0	0	0,03	0,04	0,93	0,044
4	7036	0	0	0	0,22	0,78	0	0	0	0	0,78	0,22	0	0	0,046
4	17453	0,06	0	0	0,01	0,91	0,02	0	0	0	0,01	0	0,94	0,05	0,046
4	8220	0,01	0,01	0	0	0,5	0	0,08	0,4	0	0	0,08	0,58	0,34	0,042

4	9600	0	0	0,04	0	0	0,07	0	0,8	0,09	0,8	0	0,09	0,11	0,046
4	9349	0,02	0,08	0	0	0,89	0,01	0	0	0	0,01	0,27	0,72	0	0,034
4	25491	0	0,17	0,04	0,04	0,75	0	0	0	0	0	0,04	0	0,96	0,042
4	8685	0	0	0	0,09	0,91	0	0	0	0	0	0,91	0,09	0	0,046
4	27360	0,05	0,02	0	0	0,93	0	0	0	0	0,04	0	0,01	0,95	0,044
4	18834	0,01	0,95	0	0	0	0	0	0,04	0	0,07	0,04	0	0,89	0,046
4	27470	0	1	0	0	0	0	0	0	0	0	0,98	0	0,02	0,048
4	12542	0,01	0	0,04	0	0,94	0	0	0,01	0	0,04	0,01	0,95	0	0,03
4	17573	0,02	0	0,02	0,06	0	0,36	0	0,53	0,01	0	0,06	0,58	0,36	0,044
4	46451	0	0	0	0,14	0	0,86	0	0	0	0,01	0,13	0,86	0	0,044
4	21245	0,04	0	0,02	0,01	0	0	0	0,9	0,03	0,73	0,19	0	0,08	0,048
4	7421	0,03	0	0	0	0,94	0	0,02	0,01	0	0,01	0,02	0	0,97	0,04
4	7691	0,02	0,01	0,04	0	0,92	0	0	0	0,01	0,06	0,02	0,01	0,91	0,022
4	6300	0,02	0,55	0,04	0	0,05	0	0	0,34	0	0	0,43	0,01	0,56	0,032
4	27197	0	0	0,01	0	0,15	0	0,1	0,73	0,01	0	0,1	0,89	0,01	0,048
4	6676	0,06	0	0	0,02	0,86	0	0	0	0,06	0,84	0,08	0,08	0	0,048
4	16191	0,05	0,04	0	0	0,9	0	0,01	0	0	0	0,9	0,04	0,06	0,048
4	3351	0,04	0,02	0,01	0	0	0,15	0,01	0,77	0	0	0,16	0,83	0,01	0,046
4	17625	0,05	0,95	0	0	0	0	0	0	0	0	0	0	1	0,048
4	17725	0,06	0,01	0	0	0	0	0	0,92	0,01	0	0,01	0	0,99	0,044
4	8336	0	0,17	0	0,01	0,77	0	0,04	0,01	0	0	0,21	0,07	0,72	0,048
4	19657	0,05	0	0,01	0	0,93	0	0,01	0	0	0	0	0,05	0,95	0,046
4	16491	0	0	0	0,05	0,95	0	0	0	0	0	0,96	0,04	0	0,044
4	7692	0,07	0	0	0	0	0	0	0,93	0	0,07	0	0,93	0	0,034
4	5060	0	0	0	0,06	0,8	0	0	0,14	0	0	0,06	0,14	0,8	0,048
4	28919	0,01	0	0	0,07	0,01	0	0	0,89	0,02	0,05	0,87	0,07	0,01	0,048
4	32520	0,02	0	0	0,07	0	0	0	0,91	0	0	0	0,98	0,02	0,046
4	41855	0,05	0	0	0	0,76	0,17	0,02	0	0	0,01	0,18	0,81	0	0,042
4	7514	0	0	0	0,1	0,85	0	0	0,02	0,03	0,06	0,88	0,04	0,02	0,048
4	18639	0	0,06	0	0	0,94	0	0	0	0	0,94	0,06	0	0	0,046
4	8752	0,02	0	0,04	0,02	0,92	0	0	0	0	0	0	0,92	0,08	0,04
4	14072	0	0	0	0	0	0	0,15	0,85	0	0	0,15	0,85	0	0,048
4	36988	0,04	0,05	0,03	0	0,71	0	0	0	0,17	0	0,06	0,23	0,71	0,048

4	6473	0,03	0	0	0	0,42	0,5	0,05	0	0	0,08	0,36	0,2	0,36	0,048
4	26638	0,01	0,21	0	0	0,78	0	0	0	0	0,21	0,22	0,01	0,56	0,048
4	5149	0,07	0	0,01	0	0,81	0,06	0,01	0,04	0	0,81	0,05	0,14	0	0,048
4	8011	0,01	0	0	0,2	0,79	0	0	0	0	0,79	0,21	0	0	0,048
4	23377	0	0	0,04	0	0,73	0	0,01	0,22	0	0,01	0,03	0,73	0,23	0,026
4	15805	0	0	0	0,18	0	0,78	0,04	0	0	0,79	0,03	0,18	0	0,048
4	46658	0	0,95	0,01	0	0	0	0	0	0,04	0,02	0,01	0,04	0,93	0,048
4	16032	0,04	0,15	0	0,01	0,09	0	0	0,71	0	0	0	0,01	0,99	0,048
4	50832	0,01	0,22	0	0,01	0,73	0,02	0	0	0,01	0,25	0,02	0	0,73	0,048
4	41163	0	0,91	0,04	0	0,05	0	0	0	0	0	0,02	0,04	0,94	0,048
4	46402	0	0,07	0	0	0,75	0	0,03	0,15	0	0,15	0,85	0	0	0,046
4	16451	0	0,02	0	0,07	0,84	0,07	0	0	0	0	0,03	0,08	0,89	0,042
4	4966	0,01	0,72	0	0	0	0	0	0,24	0,03	0,03	0,02	0,01	0,94	0,044
4	16298	0,06	0	0	0	0,01	0	0	0,93	0	0,06	0,24	0,69	0,01	0,048
4	17693	0,02	0	0,03	0,02	0	0,25	0	0,62	0,06	0,62	0,03	0,21	0,14	0,042
4	6787	0,04	0,01	0	0,01	0,94	0	0	0	0	0,84	0,15	0	0,01	0,04
4	7143	0	0,02	0	0,11	0,86	0,01	0	0	0	0,13	0,02	0,79	0,06	0,048
4	31100	0	0	0	0,2	0	0,29	0	0,51	0	0,8	0	0	0,2	0,044
4	7093	0	0,3	0,02	0	0,67	0,01	0	0	0	0	0	0,32	0,68	0,044
4	63150	0,03	0,01	0	0	0,95	0	0	0,01	0	0,01	0	0,98	0,01	0,03
4	45571	0,04	0	0	0,04	0,88	0,04	0	0	0	0,86	0	0,1	0,04	0,038
4	9351	0,03	0	0	0,08	0,09	0,8	0	0	0	0,03	0,01	0,87	0,09	0,046
4	8668	0,01	0	0,01	0,04	0,63	0	0,01	0,3	0	0,03	0,01	0,03	0,93	0,048
4	21459	0	0	0,04	0,02	0	0,41	0	0,53	0	0,41	0,18	0,41	0	0,028
4	14112	0,05	0	0	0	0,02	0,2	0,01	0,7	0,02	0	0,27	0,19	0,54	0,044
4	18777	0,03	0,06	0	0,01	0,9	0	0	0	0	0	0,04	0,9	0,06	0,048
4	35950	0	0,07	0	0,03	0,89	0	0	0,01	0	0,89	0,07	0,03	0,01	0,046
4	32324	0,05	0	0	0,01	0	0	0	0,94	0	0	0,94	0	0,06	0,042
4	32533	0	0	0	0,08	0	0,92	0	0	0	0	0,08	0	0,92	0,046
4	24165	0,04	0	0	0	0,96	0	0	0	0	0,04	0,96	0	0	0,042
4	5946	0,02	0	0,04	0,01	0,89	0	0,01	0,03	0	0,01	0,04	0,04	0,91	0,036
4	19838	0,03	0,01	0	0	0,91	0	0,05	0	0	0,03	0,01	0,9	0,06	0,048
4	9230	0	0,75	0	0	0	0,05	0	0,2	0	0,05	0,2	0	0,75	0,04

4	18179	0	0,05	0	0,06	0,88	0	0,01	0	0	0,06	0,88	0,06	0	0,046
4	43037	0,04	0,04	0	0	0,92	0	0	0	0	0,41	0,04	0,54	0,01	0,048
4	14063	0	0	0,04	0,01	0,53	0,06	0	0,36	0	0	0,04	0,06	0,9	0,014
4,25	26577	0,06	0	0	0	0,77	0,16	0	0	0,01	0,81	0,18	0	0,01	0,048
4,25	12623	0	0,11	0,01	0	0,2	0,13	0	0,43	0,12	0,56	0,14	0,2	0,1	0,046
4,25	31678	0,05	0	0,03	0,02	0,9	0	0	0	0	0	0,02	0,97	0,01	0,046
4,25	25914	0,01	0,01	0	0,13	0	0,02	0	0,82	0,01	0,62	0,01	0,23	0,14	0,046
4,25	21483	0,04	0,96	0	0	0	0	0	0	0	0	0	0	1	0,04
4,25	15381	0	0	0,04	0	0,92	0	0	0,04	0	0	0,55	0,01	0,44	0,004
4,25	11480	0	0,05	0	0	0,86	0	0,09	0	0	0,86	0,09	0,05	0	0,046
4,25	9701	0	0,06	0,03	0	0,91	0	0	0	0	0,91	0,03	0,06	0	0,048
4,25	91714	0	0,26	0,04	0	0,67	0	0,02	0	0,01	0	0,06	0,09	0,85	0,046
4,25	21561	0	0,12	0,03	0	0,85	0	0	0	0	0,39	0,03	0,12	0,46	0,044
4,25	15113	0,03	0	0	0,14	0,68	0,08	0	0,07	0	0,81	0,09	0,02	0,08	0,046
4,25	17552	0	0,19	0	0,02	0,78	0	0,01	0	0	0,21	0	0,01	0,78	0,04
4,25	20467	0,05	0	0	0,01	0,94	0	0	0	0	0,94	0	0,06	0	0,048
4,25	7275	0,06	0	0	0	0,93	0	0	0	0,01	0,93	0,06	0,01	0	0,046
4,25	16492	0,05	0	0	0	0,93	0,02	0	0	0	0,67	0	0,28	0,05	0,046
4,25	22972	0,03	0,04	0	0	0,78	0,14	0,01	0	0	0,13	0,82	0,05	0	0,038
4,25	28930	0,04	0,05	0,01	0	0,87	0,03	0	0	0	0,01	0,09	0,87	0,03	0,046
4,25	4758	0,04	0	0	0,04	0,91	0,01	0	0	0	0,34	0,04	0,62	0	0,046
4,25	10244	0,05	0,79	0	0	0,16	0	0	0	0	0	0,16	0	0,84	0,044
4,25	15370	0,02	0,14	0,01	0,02	0	0	0	0,81	0	0,03	0,81	0	0,16	0,046
4,25	64456	0	0	0	0,13	0,86	0,01	0	0	0	0,04	0	0,96	0	0,042
4,25	27088	0	0,1	0	0	0	0	0	0,9	0	0,01	0,12	0,87	0	0,046
4,25	14492	0,05	0	0	0	0,03	0,84	0	0	0,08	0,04	0	0,88	0,08	0,046
4,25	42046	0,06	0	0	0	0	0,26	0,03	0,65	0	0,91	0	0,03	0,06	0,044
4,25	20958	0,02	0,01	0	0,05	0,9	0	0	0	0,02	0,39	0	0,56	0,05	0,042
4,25	16170	0,06	0	0	0	0,02	0	0	0,92	0	0	0,83	0,01	0,16	0,044
4,25	17046	0	0	0,04	0,09	0,65	0,01	0	0,19	0,02	0,29	0,03	0	0,68	0,04
4,25	27410	0,05	0	0	0	0,51	0	0	0	0,44	0,05	0	0,51	0,44	0,036
4,25	7633	0	0	0,03	0	0,82	0	0,15	0	0	0,66	0	0,19	0,15	0,046
4,25	23892	0,06	0	0	0,01	0	0	0	0,93	0	0,01	0,93	0,06	0	0,046

4,25	6632	0	0	0	0,2	0,79	0	0	0,01	0	0,8	0	0,15	0,05	0,04
4,25	42595	0,06	0,07	0	0	0,85	0	0	0,02	0	0	0,22	0	0,78	0,048
4,25	47017	0,04	0,02	0,03	0	0,16	0,04	0,02	0,69	0	0,04	0,03	0,03	0,9	0,048
4,25	13398	0	0	0	0,18	0,18	0,64	0	0	0	0,6	0,22	0,18	0	0,044
4,25	14415	0	0,43	0	0,04	0,14	0,01	0	0,37	0,01	0,01	0,37	0,01	0,61	0,046
4,25	34167	0	0,12	0	0	0,88	0	0	0	0	0,12	0	0,88	0	0,046
4,25	20345	0	0	0	0,1	0,88	0,02	0	0	0	0	0,11	0,89	0	0,044
4,25	6525	0	0,08	0	0	0,9	0	0,02	0	0	0,02	0	0,98	0	0,046
4,25	11855	0	0	0	0,08	0,92	0	0	0	0	0,09	0,83	0,08	0	0,044
4,25	36763	0,06	0	0,04	0	0,89	0	0,01	0	0	0	0,04	0	0,96	0,044
4,25	15519	0,05	0	0,01	0	0,52	0	0	0,41	0,01	0,12	0,02	0,58	0,28	0,048
4,25	36763	0,01	0	0	0,1	0,52	0,35	0,02	0	0	0,32	0,36	0,29	0,03	0,046
4,25	29152	0,06	0	0	0	0	0,81	0	0,07	0,06	0,82	0	0,18	0	0,042
4,25	20693	0,04	0,01	0,01	0	0,26	0	0	0,67	0,01	0,31	0	0,68	0,01	0,04
4,25	15062	0,06	0,01	0	0	0,52	0,19	0,02	0	0,2	0,01	0,19	0,6	0,2	0,046
4,25	44946	0,06	0	0	0	0,14	0,8	0	0	0	0,1	0,03	0,87	0	0,048
4,25	15699	0,02	0,03	0,02	0	0,82	0,04	0	0	0,07	0,81	0,01	0,16	0,02	0,042
4,25	22143	0	0,15	0	0	0,85	0	0	0	0	0,13	0	0,86	0,01	0,048
4,25	9716	0,07	0	0	0	0,93	0	0	0	0	0,04	0	0,93	0,03	0,046
4,25	13133	0	0	0	0,26	0	0	0	0,74	0	0,74	0,24	0,02	0	0,04
4,25	7768	0	0	0	0,1	0,89	0	0	0	0,01	0,01	0	0,99	0	0,036
4,25	6895	0,03	0,01	0,04	0	0,92	0	0	0	0	0,08	0,04	0,85	0,03	0,048
4,25	31175	0,06	0	0	0	0,93	0	0	0,01	0	0	0,07	0	0,93	0,046
4,25	16017	0,07	0,01	0	0	0,92	0	0	0	0	0,07	0,01	0	0,92	0,048
4,25	30180	0	0	0,01	0	0	0,01	0,12	0,86	0	0,13	0	0,85	0,02	0,042
4,25	36105	0	0,01	0	0,12	0,87	0	0	0	0	0,13	0	0,87	0	0,044
4,25	29451	0,06	0	0	0	0	0	0	0,94	0	0	0,92	0,04	0,04	0,048
4,25	8638	0,04	0,02	0	0	0,89	0,03	0	0	0,02	0,02	0,95	0,03	0	0,048
4,25	30582	0	0,95	0	0	0,03	0,02	0	0	0	0,05	0	0	0,95	0,046
4,25	29247	0,02	0,01	0,02	0	0,87	0	0,03	0,05	0	0,91	0,07	0,01	0,01	0,038
4,25	13600	0,02	0,02	0,01	0,06	0,47	0,12	0	0,3	0	0	0,91	0,06	0,03	0,048
4,25	22027	0,03	0,02	0,02	0	0,87	0,03	0	0,01	0,02	0,64	0,33	0	0,03	0,044
4,25	15206	0	0,01	0,04	0	0,84	0	0	0,07	0,04	0,85	0,07	0,01	0,07	0,04

4,25	2909	0	0	0	0	0,95	0	0,05	0	0	0,05	0	0	0,95	0,046
4,25	14315	0	0,01	0,01	0,12	0,64	0,2	0	0,02	0	0,15	0,01	0,67	0,17	0,042
4,25	8371	0	0,09	0	0	0,02	0,66	0,04	0,19	0	0,1	0,02	0,67	0,21	0,042
4,25	44630	0,02	0	0,04	0,05	0,87	0,02	0	0	0	0	0,02	0	0,98	0,044
4,25	15911	0,03	0,25	0	0	0	0,72	0	0	0	0	0,03	0	0,97	0,044
4,25	17345	0	0	0	0,05	0	0	0	0,95	0	0	0,03	0	0,97	0,044
4,25	18425	0,03	0	0	0,02	0,02	0	0,01	0,92	0	0,02	0,03	0	0,95	0,048
4,25	18043	0,04	0,04	0	0	0	0	0	0,88	0,04	0	0,04	0,92	0,04	0,046
4,25	19919	0,05	0	0,01	0	0	0,01	0	0,93	0	0,93	0,01	0	0,06	0,04
4,25	11860	0,01	0,06	0	0	0,91	0	0	0,02	0	0,92	0	0	0,08	0,046
4,25	11102	0	0,01	0	0,07	0	0,04	0,03	0,7	0,15	0	0,81	0,19	0	0,046
4,25	27152	0,01	0	0,04	0	0,93	0	0	0,02	0	0,96	0,02	0,01	0,01	0,048
4,25	36396	0	0	0	0,07	0,85	0	0	0,07	0,01	0	0,84	0,14	0,02	0,044
4,25	14801	0,06	0	0	0,01	0,8	0,13	0	0	0	0,85	0,02	0,13	0	0,046
4,25	12631	0,01	0	0	0,09	0,85	0	0	0,05	0	0,09	0,06	0,85	0	0,046
4,25	22974	0,02	0	0	0	0,86	0,03	0,08	0	0,01	0,01	0,01	0,96	0,02	0,046
4,25	19082	0	0,11	0,03	0	0	0,02	0	0,79	0,05	0,1	0,75	0,08	0,07	0,044
4,25	44588	0	0	0	0,2	0,75	0,01	0	0,04	0	0,77	0,02	0,21	0	0,048
4,25	47720	0	0	0	0,11	0,87	0	0,02	0	0	0	0	1	0	0,046
4,25	19586	0	0	0	0,03	0,5	0,29	0,05	0	0,13	0,2	0,5	0	0,3	0,048
4,25	27537	0,03	0	0	0	0,95	0	0,02	0	0	0,01	0,95	0	0,04	0,04
4,25	29492	0	0	0	0,11	0,63	0	0	0	0,26	0	0,26	0,63	0,11	0,048
4,25	55822	0	0	0	0,05	0,94	0	0,01	0	0	0,01	0,62	0	0,37	0,044
4,25	19918	0,03	0	0	0,02	0,95	0	0	0	0	0	0,98	0,02	0	0,042
4,25	29858	0,06	0,08	0	0	0,12	0	0	0,74	0	0	0,02	0,08	0,9	0,042
4,25	13762	0	0	0	0,14	0	0	0	0,86	0	0,45	0,41	0	0,14	0,044
4,25	51821	0,02	0,13	0	0,02	0,73	0,1	0	0	0	0,13	0	0,04	0,83	0,046
4,25	24543	0,01	0,07	0	0	0,92	0	0	0	0	0,88	0,07	0,04	0,01	0,046
4,25	12382	0	0,07	0	0	0,92	0	0,01	0	0	0,99	0	0	0,01	0,046
4,25	14766	0	0	0	0,11	0,89	0	0	0	0	0,14	0,12	0,74	0	0,042
4,25	7742	0,04	0,07	0	0	0,03	0,16	0	0,68	0,02	0,03	0,86	0,04	0,07	0,048
4,25	16826	0	0,01	0	0,04	0,95	0	0	0	0	0,02	0,98	0	0	0,048
4,25	14314	0	0	0	0,06	0,85	0	0	0,09	0	0	0,15	0	0,85	0,048

4,25	19439	0,02	0,9	0	0	0	0	0	0,08	0	0	0	0,02	0,98	0,044
4,25	53995	0	0,31	0	0	0,02	0,02	0,02	0,61	0,02	0,02	0,02	0,13	0,83	0,044
4,25	56933	0,02	0,97	0	0	0	0	0,01	0	0	0,02	0,01	0	0,97	0,038
4,25	27197	0,06	0	0	0	0	0	0	0,93	0,01	0	0,06	0	0,94	0,046
4,5	35629	0,02	0,19	0	0	0,04	0,23	0	0,52	0	0	0,73	0,04	0,23	0,036
4,5	11419	0	0	0	0,08	0,04	0,17	0	0,71	0	0,17	0,12	0	0,71	0,048
4,5	28896	0,04	0	0,01	0,03	0,91	0	0	0	0,01	0	0,03	0,43	0,54	0,042
4,5	7084	0	0,13	0	0	0,87	0	0	0	0	0	0,13	0,87	0	0,044
4,5	10613	0,01	0,75	0	0	0,24	0	0	0	0	0	0,75	0,01	0,24	0,044
4,5	7904	0	0	0	0,12	0,82	0,04	0,01	0,01	0	0,13	0,01	0,86	0	0,048
4,5	55435	0	0	0	0,09	0,9	0	0	0	0,01	0	0	0,99	0,01	0,038
4,5	8938	0,04	0	0,01	0	0,94	0	0	0,01	0	0,82	0	0,16	0,02	0,042
4,5	20474	0,05	0	0	0	0	0	0	0,95	0	0,17	0	0,78	0,05	0,048
4,5	13056	0,06	0	0	0	0,89	0,01	0	0	0,04	0,89	0	0,01	0,1	0,048
4,5	27616	0	0	0	0,05	0,79	0,02	0,01	0,04	0,09	0,06	0,11	0	0,83	0,048
4,5	8083	0,05	0	0,01	0	0,93	0	0	0	0,01	0	0,08	0,92	0	0,046
4,5	35709	0,01	0,2	0,01	0,01	0,76	0	0	0,01	0	0,2	0	0,01	0,79	0,044
4,5	61711	0,03	0,03	0	0	0,88	0,02	0,02	0	0,02	0,9	0,04	0,06	0	0,048
4,5	49350	0	0,08	0	0,04	0,83	0	0	0,05	0	0,05	0	0,91	0,04	0,048
4,5	11969	0,06	0	0	0	0,92	0	0	0	0,02	0,01	0,01	0,06	0,92	0,048
4,5	22147	0	0,05	0	0,06	0,89	0	0	0	0	0	0,89	0,11	0	0,046
4,5	14695	0	0	0	0,29	0,7	0	0	0,01	0	0,99	0	0,01	0	0,048
4,5	9871	0	0,05	0	0,02	0	0,86	0,07	0	0	0,39	0,48	0,06	0,07	0,046
4,5	16908	0	0	0	0,23	0,77	0	0	0	0	0,77	0,23	0	0	0,048
4,5	29239	0,02	0	0	0,04	0,91	0,03	0	0	0	0,01	0,91	0,08	0	0,044
4,5	22329	0,01	0,33	0	0	0,66	0	0	0	0	0,01	0,61	0,15	0,23	0,048
4,5	10567	0,06	0	0	0	0,91	0	0	0,03	0	0,03	0	0,05	0,92	0,046
4,5	9577	0,04	0,02	0	0	0,37	0,51	0,02	0	0,04	0,37	0,07	0,56	0	0,042
4,5	45377	0	0	0	0,15	0,75	0,04	0,01	0,03	0,02	0,81	0,04	0	0,15	0,038
4,5	6568	0,05	0	0,03	0	0	0,91	0	0	0,01	0,81	0,03	0,06	0,1	0,048
4,5	30937	0,05	0	0,03	0	0,79	0,13	0	0	0	0,77	0,01	0,22	0	0,046
4,5	8863	0	0,07	0	0	0	0	0	0,81	0,12	0,88	0,12	0	0	0,046
4,5	87608	0	0,99	0	0	0,01	0	0	0	0	0	0,99	0	0,01	0,04

4,5	11637	0,04	0	0	0	0,94	0	0,02	0	0	0,02	0,98	0	0	0,042
4,5	38747	0,05	0	0	0	0,95	0	0	0	0	0,05	0	0,95	0	0,046
4,5	27635	0,03	0,28	0	0	0,69	0	0	0	0	0	0,19	0,09	0,72	0,048
4,5	17277	0,02	0	0,01	0	0,92	0	0,05	0	0	0,05	0,9	0,02	0,03	0,048
4,5	33835	0	0,11	0	0,02	0	0	0	0,87	0	0,11	0,87	0,02	0	0,048
4,5	11882	0,02	0,03	0	0,03	0	0,81	0	0,11	0	0	0,03	0,51	0,46	0,044
4,5	28910	0	0	0	0,06	0,94	0	0	0	0	0,06	0	0	0,94	0,048
4,5	16419	0	0	0	0,08	0,92	0	0	0	0	0,08	0,92	0	0	0,046
4,5	20003	0	0,02	0	0,1	0,86	0	0	0,01	0,01	0	0,01	0,99	0	0,048
4,5	19578	0,01	0	0	0,05	0,94	0	0	0	0	0,05	0	0,01	0,94	0,044
4,5	9496	0,03	0	0	0	0,49	0,38	0,04	0	0,06	0,06	0,38	0,08	0,48	0,042
4,5	24399	0,05	0	0	0	0,91	0,01	0	0,03	0	0,05	0,01	0,04	0,9	0,046
4,5	18076	0,06	0	0	0	0,86	0	0,01	0,07	0	0,99	0	0,01	0	0,048
4,5	13917	0	0,02	0	0,07	0,69	0	0	0,18	0,04	0,04	0,7	0,19	0,07	0,046
4,5	25465	0,05	0	0	0	0,28	0,54	0	0,13	0	0,18	0,54	0	0,28	0,046
4,5	12745	0,05	0	0,01	0	0,91	0,03	0	0	0	0,93	0,03	0	0,04	0,04
4,5	37225	0	0	0,01	0,1	0,79	0,09	0	0,01	0	0,1	0,69	0,1	0,11	0,048
4,5	50731	0	1	0	0	0	0	0	0	0	0,07	0	0	0,93	0,044
4,5	18357	0	0	0	0,28	0,71	0	0,01	0	0	0,88	0,12	0	0	0,044
4,5	5618	0,05	0	0	0	0	0	0,01	0,88	0,06	0	0,94	0,01	0,05	0,046
4,5	10640	0,06	0	0	0	0,91	0,03	0	0	0	0,73	0,12	0,15	0	0,048
4,5	21298	0,04	0	0	0	0	0,96	0	0	0	0,04	0,05	0	0,91	0,02
4,5	15171	0,03	0,08	0	0,02	0,85	0	0	0,02	0	0,09	0,02	0,05	0,84	0,048
4,5	34721	0	0	0,04	0,1	0,45	0	0	0	0,41	0,37	0,04	0,24	0,35	0,048
4,5	20100	0,02	0	0,04	0	0,94	0	0	0	0	0	0	0,98	0,02	0,032
4,5	13698	0,01	0	0,01	0	0	0,19	0,05	0,74	0	0,05	0,2	0,01	0,74	0,044
4,5	22590	0,03	0,86	0,03	0	0	0	0	0,08	0	0	0,06	0	0,94	0,044
4,5	14729	0	0,12	0,04	0	0	0,05	0	0,78	0,01	0	0,24	0,17	0,59	0,03
4,5	18025	0	0,01	0	0,09	0,81	0	0	0,09	0	0	0,1	0,81	0,09	0,048
4,5	40472	0	0,26	0	0	0,62	0	0	0,12	0	0,12	0,88	0	0	0,046
4,5	12478	0,06	0,02	0	0	0,91	0,01	0	0	0	0,02	0,24	0	0,74	0,048
4,5	31678	0	0,08	0	0,05	0,87	0	0	0	0	0,05	0,05	0,9	0	0,046
4,5	23585	0,04	0	0,01	0	0,94	0	0,01	0	0	0,01	0	0,01	0,98	0,048

4,5	14436	0,04	0,04	0,03	0	0,11	0	0	0,78	0	0,07	0,04	0,11	0,78	0,036
4,5	16755	0,04	0,08	0	0	0,88	0	0	0	0	0	0,96	0,04	0	0,044
4,5	23904	0	0,08	0,01	0,01	0,16	0	0,04	0,7	0	0,01	0,05	0,78	0,16	0,048
4,5	6474	0,05	0	0,02	0	0,89	0	0	0	0,04	0	0,17	0,78	0,05	0,046
4,5	11872	0,05	0	0,01	0,06	0,88	0	0	0	0	0,88	0,07	0,05	0	0,044
4,5	32355	0,02	0,11	0	0	0,7	0	0	0	0,17	0	0	0,7	0,3	0,044
4,5	30565	0,03	0,03	0,01	0	0,87	0,02	0,02	0	0,02	0	0,01	0,97	0,02	0,048
4,5	20822	0,05	0	0,03	0	0,79	0	0	0,13	0	0,05	0	0,16	0,79	0,042
4,5	22381	0,03	0,41	0	0	0	0	0	0,56	0	0	0,57	0	0,43	0,036
4,5	11891	0,06	0,01	0	0	0,9	0	0	0,03	0	0,01	0,06	0	0,93	0,046
4,5	10776	0,05	0	0,03	0	0	0	0	0,92	0	0	0,95	0	0,05	0,048
4,5	30257	0	0,95	0,04	0	0	0	0	0	0,01	0	0,26	0	0,74	0,026
4,5	40973	0,04	0	0	0,01	0,94	0	0,01	0	0	0,4	0,05	0,52	0,03	0,042
4,5	8169	0,02	0,76	0,02	0	0	0	0	0,12	0,08	0,02	0,03	0	0,95	0,048
4,5	10262	0,01	0	0	0,11	0,88	0	0	0	0	0	0,01	0,88	0,11	0,048
4,5	7779	0	0,02	0,02	0,08	0,32	0,02	0,01	0,51	0,02	0,02	0,02	0,82	0,14	0,046
4,5	13354	0	0	0,01	0,06	0,9	0	0	0	0,03	0	0	0	1	0,036
4,5	7296	0	0,89	0	0	0,07	0,01	0,01	0,02	0	0,04	0,06	0,01	0,89	0,042
4,5	9513	0,05	0	0	0	0,94	0,01	0	0	0	0,06	0	0,94	0	0,048
4,5	18374	0	0,02	0	0,06	0,92	0	0	0	0	0,11	0,02	0	0,87	0,048
4,5	6811	0,02	0	0	0	0	0,02	0,06	0,87	0,03	0,03	0,87	0,06	0,04	0,048
4,5	17676	0,04	0	0	0,03	0,91	0,02	0	0	0	0,95	0,01	0,04	0	0,04
4,5	15835	0,04	0	0,03	0	0,89	0	0	0,04	0	0,04	0,03	0,93	0	0,048
4,5	34238	0	0,1	0	0	0,83	0	0	0,07	0	0,52	0,46	0	0,02	0,046
4,5	7828	0,05	0	0	0	0,95	0	0	0	0	0,31	0,69	0	0	0,048
4,5	67777	0,05	0	0,02	0	0,91	0,02	0	0	0	0,05	0,04	0	0,91	0,042
4,5	10564	0,03	0	0	0	0,29	0,17	0,09	0	0,42	0,19	0	0,69	0,12	0,048
4,5	39667	0	0	0	0,12	0,63	0	0	0,24	0,01	0,12	0,24	0,64	0	0,046
4,5	6474	0,04	0	0	0	0,88	0	0,02	0,03	0,03	0,88	0,02	0,05	0,05	0,048
4,5	35624	0,03	0	0,01	0,01	0,03	0,69	0,02	0,1	0,11	0,75	0	0,1	0,15	0,046
4,5	25756	0	0,1	0,04	0,03	0,79	0	0,01	0,01	0,02	0,06	0,81	0,02	0,11	0,046
4,5	23515	0,05	0	0	0	0,75	0,05	0	0,15	0	0,15	0,05	0,05	0,75	0,044
4,5	30994	0	0,17	0	0	0,76	0,06	0,01	0	0	0	0,79	0,17	0,04	0,042

4,5	60172	0,01	0	0,04	0	0	0,94	0	0	0,01	0	0,05	0,95	0	0,028
4,5	11197	0	0,02	0,01	0,06	0,91	0	0	0	0	0,06	0,91	0,02	0,01	0,044
4,5	18848	0	0	0	0,1	0,83	0,03	0	0,02	0,02	0,1	0,88	0,02	0	0,046
4,5	11585	0	0	0	0,06	0,94	0	0	0	0	0,07	0,93	0	0	0,042
4,5	31895	0	0,05	0,04	0	0,75	0	0	0,16	0	0,05	0	0,16	0,79	0,012
4,75	29691	0,05	0	0	0	0,95	0	0	0	0	0,04	0,96	0	0	0,038
4,75	14101	0	0,63	0,03	0,04	0,3	0	0	0	0	0	0,05	0	0,95	0,046
4,75	24447	0,02	0,14	0	0	0,26	0,13	0	0,45	0	0,13	0,45	0,02	0,4	0,046
4,75	10295	0,05	0	0	0	0,93	0,01	0	0,01	0	0	0	0,94	0,06	0,04
4,75	45224	0	0	0	0,13	0,76	0	0,01	0,09	0,01	0,1	0,13	0,77	0	0,048
4,75	13103	0,01	0,28	0	0	0	0	0	0,71	0	0	0,06	0,23	0,71	0,048
4,75	32619	0	0,51	0	0,01	0,46	0,01	0	0	0,01	0	0,56	0,08	0,36	0,048
4,75	9352	0,04	0	0,03	0,01	0,11	0	0	0,8	0,01	0,11	0,81	0,07	0,01	0,042
4,75	14155	0	0	0	0,34	0,45	0,17	0,01	0	0,03	0,92	0	0,01	0,07	0,044
4,75	31946	0,04	0	0,04	0	0,88	0,01	0,01	0	0,02	0,02	0,93	0,04	0,01	0,048
4,75	31028	0,05	0	0	0	0,95	0	0	0	0	0	0,57	0,05	0,38	0,042
4,75	12085	0,01	0	0	0,11	0,87	0	0	0,01	0	0,01	0	0,99	0	0,048
4,75	14575	0	0,1	0	0	0,78	0,12	0	0	0	0,63	0	0,27	0,1	0,048
4,75	34696	0	0,14	0	0	0,01	0	0,04	0,81	0	0	0,89	0,1	0,01	0,046
4,75	10279	0,04	0	0,01	0	0,89	0	0	0,06	0	0,89	0,03	0,08	0	0,048
4,75	9225	0	0	0	0,06	0	0	0	0,94	0	0,01	0,99	0	0	0,046
4,75	18014	0,03	0	0,03	0,03	0,91	0	0	0	0	0,03	0,06	0	0,91	0,048
4,75	7188	0,04	0,08	0	0	0	0	0	0,88	0	0	0,88	0,03	0,09	0,038
4,75	15114	0,04	0	0,02	0	0	0,94	0	0	0	0,03	0	0,95	0,02	0,046
4,75	16399	0	0	0	0,07	0,93	0	0	0	0	0,07	0,93	0	0	0,046
4,75	23373	0,05	0	0	0	0,9	0,05	0	0	0	0,9	0,04	0,06	0	0,044
4,75	15041	0	0	0	0,15	0,29	0	0	0,56	0	0,56	0,05	0,29	0,1	0,044
4,75	13401	0,06	0	0	0	0,82	0,12	0	0	0	0,88	0,08	0	0,04	0,048
4,75	15661	0	0,09	0,04	0	0	0	0	0,7	0,17	0,03	0,18	0,79	0	0,046
4,75	14952	0,05	0	0	0,01	0,94	0	0	0	0	0,99	0	0	0,01	0,048
4,75	32169	0	0	0	0,07	0,7	0,22	0	0,01	0	0,02	0,01	0,05	0,92	0,048
4,75	13501	0	0	0,04	0	0,93	0	0	0,03	0	0	0,07	0,93	0	0,02
4,75	15897	0,04	0	0	0,07	0,89	0	0	0	0	0,85	0,01	0,07	0,07	0,048

4,75	11125	0	0,03	0	0,05	0,9	0,02	0	0	0	0	0,97	0,03	0	0,042
4,75	9701	0,04	0	0	0,04	0,88	0	0,01	0	0,03	0,81	0,03	0	0,16	0,046
4,75	8968	0,04	0,02	0	0	0,93	0	0,01	0	0	0,97	0,02	0,01	0	0,036
4,75	11835	0,06	0,02	0,01	0	0,87	0	0,01	0	0,03	0,03	0,07	0,03	0,87	0,046
4,75	17127	0,01	0,04	0,04	0,01	0,53	0,01	0	0,36	0	0,23	0,04	0,04	0,69	0,044
4,75	16344	0,01	0,92	0	0	0,07	0	0	0	0	0,05	0	0,01	0,94	0,034
4,75	35193	0,03	0,02	0	0	0	0,93	0,02	0	0	0,02	0	0,96	0,02	0,036
4,75	18300	0	0,07	0	0	0,92	0	0	0	0,01	0,92	0,01	0,06	0,01	0,048
4,75	65026	0	0	0,04	0	0	0	0,02	0,94	0	0	0	0,9	0,1	0,038
4,75	13111	0,05	0	0	0	0,76	0,02	0	0	0,17	0,17	0,05	0,63	0,15	0,038
4,75	17085	0,05	0	0,01	0	0,94	0	0	0	0	0	0,01	0,99	0	0,042
4,75	18353	0,01	0,1	0	0	0,84	0,01	0,04	0	0	0,17	0,01	0,04	0,78	0,048
4,75	19101	0,05	0,02	0	0	0,93	0	0	0	0	0,05	0,02	0	0,93	0,044
4,75	8033	0,01	0	0,02	0,03	0,9	0,02	0,01	0	0,01	0,01	0,96	0	0,03	0,046
4,75	61385	0	0	0	0,11	0,89	0	0	0	0	0,17	0	0,77	0,06	0,044
4,75	7489	0,01	0	0	0,05	0,93	0,01	0	0	0	0,01	0,07	0	0,92	0,048
4,75	36115	0,01	0,01	0	0,06	0,92	0	0	0	0	0,01	0	0,06	0,93	0,042
4,75	16637	0	0,14	0	0	0,48	0,28	0	0,08	0,02	0,07	0,08	0,85	0	0,04
4,75	7481	0,03	0,1	0	0	0,15	0,67	0,01	0	0,04	0,04	0,17	0,03	0,76	0,038
4,75	6672	0,05	0	0,01	0	0,94	0	0	0	0	0	0,01	0,05	0,94	0,048
4,75	25291	0,01	0	0,04	0	0	0,15	0	0,53	0,27	0,68	0,04	0,28	0	0,042
4,75	14762	0,06	0	0	0	0,87	0	0	0,06	0,01	0,01	0,06	0	0,93	0,046
4,75	13454	0,04	0	0,04	0	0	0,06	0	0,86	0	0	0,86	0,12	0,02	0,04
4,75	5939	0,03	0,14	0	0	0,83	0	0	0	0	0	0,86	0,14	0	0,04
4,75	7654	0	0,04	0	0,05	0,89	0,01	0,01	0	0	0,03	0,9	0,05	0,02	0,048
4,75	33734	0,04	0,04	0	0	0	0,92	0	0	0	0	0,96	0	0,04	0,048
4,75	8085	0,05	0	0	0,03	0,5	0,07	0	0,24	0,11	0,11	0,17	0,48	0,24	0,044
4,75	14822	0	0,35	0	0,04	0,61	0	0	0	0	0	0,61	0,03	0,36	0,046
4,75	27083	0	0	0,03	0	0	0,39	0,12	0,46	0	0,46	0	0,4	0,14	0,048
4,75	24129	0	0	0	0,09	0,89	0,02	0	0	0	0,09	0	0	0,91	0,048
4,75	46579	0,05	0,15	0	0	0,74	0,06	0	0	0	0,03	0,12	0,05	0,8	0,044
4,75	24855	0	0	0,04	0	0,96	0	0	0	0	0,96	0	0,04	0	0,044
4,75	19955	0	0,02	0	0,07	0,91	0	0	0	0	0	1	0	0	0,048

4,75	32538	0,02	0	0,03	0,04	0,88	0	0,03	0	0	0,88	0,05	0	0,07	0,034
4,75	50621	0	0,18	0	0,03	0,19	0	0	0,6	0	0,15	0	0,22	0,63	0,042
4,75	8991	0	0,06	0	0,06	0,83	0	0	0,05	0	0,2	0,8	0	0	0,044
4,75	64890	0,06	0	0	0	0,94	0	0	0	0	0,05	0,01	0	0,94	0,048
4,75	26051	0,05	0	0,03	0	0,86	0	0	0	0,06	0	0,89	0,05	0,06	0,046
4,75	12990	0,06	0	0	0	0,9	0	0	0,03	0,01	0,01	0,99	0	0	0,046
4,75	13402	0,04	0,08	0	0	0,63	0	0,01	0,24	0	0,08	0,01	0,24	0,67	0,048
4,75	7963	0,04	0	0	0,03	0,25	0,03	0	0,65	0	0,03	0,94	0,03	0	0,048
4,75	23699	0	0	0,01	0,02	0,31	0	0,13	0,53	0	0,32	0,07	0,53	0,08	0,048
4,75	19328	0	0	0,04	0	0,95	0	0,01	0	0	0	0,05	0,95	0	0,024
4,75	37679	0,04	0	0,02	0	0,87	0,07	0	0	0	0,87	0,02	0	0,11	0,042
4,75	39598	0,05	0,01	0	0	0,07	0	0,01	0,86	0	0	0,86	0,09	0,05	0,048
4,75	31292	0	0	0	0,18	0,82	0	0	0	0	0,79	0,03	0	0,18	0,038
4,75	20689	0	0	0	0,16	0,34	0,37	0	0,13	0	0,63	0,01	0,26	0,1	0,048
4,75	25869	0,03	0	0	0,03	0,59	0,01	0	0,32	0,02	0,04	0	0,03	0,93	0,038
4,75	4906	0,06	0	0	0	0,03	0,2	0	0,71	0	0	0,39	0,52	0,09	0,048
4,75	21065	0,03	0	0	0,01	0,68	0,28	0	0	0	0,68	0,28	0,04	0	0,046
4,75	28820	0	0,14	0	0	0,86	0	0	0	0	0	0	0,74	0,26	0,042
4,75	25902	0	0	0	0	0,06	0,83	0,11	0	0	0,06	0,93	0,01	0	0,04
4,75	23160	0	0,06	0,01	0	0,93	0	0	0	0	0,93	0	0,06	0,01	0,044
4,75	33529	0,06	0	0	0	0,94	0	0	0	0	0,94	0,06	0	0	0,044
4,75	36198	0,06	0	0	0	0,93	0	0	0	0,01	0,37	0,02	0	0,61	0,046
4,75	21422	0,05	0,03	0	0	0,9	0,01	0,01	0	0	0	0,27	0,04	0,69	0,046
4,75	25057	0,01	0	0	0,04	0	0	0,01	0,94	0	0	0,94	0	0,06	0,048
4,75	7152	0,02	0	0	0	0,86	0	0,04	0,07	0,01	0,05	0	0	0,95	0,046
4,75	24776	0,01	0,07	0	0	0	0	0,01	0,81	0,1	0,3	0,08	0,62	0	0,046
4,75	24779	0	0	0	0,34	0,65	0	0,01	0	0	0,98	0,01	0,01	0	0,048
4,75	13451	0	0	0,04	0	0,88	0	0,04	0	0,04	0	0	0,96	0,04	0,042
4,75	36408	0	0,01	0,04	0	0,87	0,07	0	0	0,01	0,85	0,02	0,12	0,01	0,046
4,75	25714	0	0,07	0	0,05	0,86	0	0	0,01	0,01	0	0,93	0,05	0,02	0,034
4,75	36463	0,03	0	0,03	0	0,94	0	0	0	0	0,03	0	0,49	0,48	0,03
4,75	26033	0	0,01	0	0,31	0,68	0	0	0	0	0,99	0	0,01	0	0,046
4,75	15074	0	0,08	0	0	0,91	0	0	0	0,01	0,95	0	0	0,05	0,048

4,75	37547	0,06	0	0	0	0,04	0	0	0,9	0	0,03	0	0,92	0,05	0,042
4,75	48144	0	0,04	0	0,06	0,69	0,01	0	0,2	0	0,05	0	0,2	0,75	0,046
4,75	12197	0,03	0	0	0,07	0,89	0,01	0	0	0	0	0	0,96	0,04	0,038
4,75	8227	0,05	0	0	0,01	0,89	0	0	0,05	0	0	0,89	0,05	0,06	0,046
4,75	22686	0	0	0	0,09	0,91	0	0	0	0	0,06	0,94	0	0	0,046
4,75	18620	0,03	0,02	0	0,07	0,83	0,03	0	0,01	0,01	0,83	0,09	0,01	0,07	0,042
5	18899	0	0,07	0	0	0,89	0,01	0	0,02	0,01	0,92	0	0,08	0	0,038
5	15525	0,02	0	0	0,17	0,81	0	0	0	0	0,8	0,19	0,01	0	0,046
5	16379	0,01	0,01	0,03	0,01	0,04	0,02	0,03	0,85	0	0,01	0,04	0,04	0,91	0,04
5	33407	0,01	0,08	0	0	0	0,9	0,01	0	0	0,8	0,06	0,02	0,12	0,044
5	24285	0,04	0	0	0,02	0,56	0,38	0	0	0	0,01	0,01	0,42	0,56	0,048
5	7162	0,01	0	0,01	0	0,93	0	0,04	0,01	0	0	0,06	0	0,94	0,046
5	36150	0,04	0,01	0,02	0,09	0	0,84	0	0	0	0,76	0,14	0,08	0,02	0,046
5	20762	0,02	0,12	0	0	0,86	0	0	0	0	0,12	0,22	0,66	0	0,044
5	9875	0	1	0	0	0	0	0	0	0	0	1	0	0	0,034
5	14153	0	0	0	0,02	0	0,76	0,05	0	0,17	0,05	0	0,03	0,92	0,044
5	35476	0	0	0	0,06	0,88	0	0,02	0,03	0,01	0,02	0,87	0,09	0,02	0,04
5	14431	0,01	0	0	0,02	0,01	0	0,08	0,88	0	0,05	0,02	0,84	0,09	0,038
5	66179	0	0,08	0	0	0,91	0	0	0	0,01	0,91	0,08	0	0,01	0,04
5	15606	0,01	0,12	0,01	0	0,85	0	0	0,01	0	0	0	0,87	0,13	0,036
5	38422	0,07	0	0	0,01	0,83	0	0	0,09	0	0,1	0	0,83	0,07	0,046
5	12618	0,04	0	0,01	0	0	0	0,01	0,94	0	0	0,01	0	0,99	0,044
5	35231	0,03	0	0	0,02	0	0,25	0,05	0,65	0	0	0,28	0,72	0	0,046
5	13201	0,07	0	0	0	0,88	0	0	0,05	0	0,73	0,02	0,1	0,15	0,044
5	7346	0	0,07	0	0,02	0,71	0,18	0,02	0	0	0,16	0	0,62	0,22	0,036
5	31568	0,01	0,03	0,02	0	0,94	0	0	0	0	0,95	0	0,03	0,02	0,04
5	34557	0,01	0	0,02	0	0,79	0	0,16	0,01	0,01	0,96	0,02	0,02	0	0,046
5	58718	0,06	0	0	0	0,71	0,06	0	0,17	0	0,05	0,12	0,72	0,11	0,046
5	5933	0,01	0,12	0	0,04	0,83	0	0	0	0	0,12	0,01	0,08	0,79	0,042
5	17215	0,02	0,25	0	0	0	0	0,01	0,72	0	0	0,97	0,03	0	0,046
5	6700	0,03	0	0	0,07	0,26	0,46	0	0,18	0	0,64	0,07	0,04	0,25	0,044
5	11963	0	0,12	0	0	0,48	0	0	0,4	0	0,6	0	0	0,4	0,046
5	8533	0,02	0,2	0	0,03	0,71	0	0	0,03	0,01	0	0,74	0	0,26	0,048

5	4276	0	0	0	0,05	0,9	0,04	0	0,01	0	0,01	0,06	0,05	0,88	0,044
5	27613	0,03	0	0,04	0,02	0,85	0,05	0	0,01	0	0,02	0,07	0,08	0,83	0,048
5	22809	0,02	0,12	0,01	0,04	0,81	0	0	0	0	0,01	0,01	0,04	0,94	0,048
5	10722	0	0,17	0	0,02	0,78	0,02	0	0	0,01	0,02	0,78	0,18	0,02	0,038
5	35095	0,06	0	0	0	0,92	0,01	0	0,01	0	0	0,06	0,92	0,02	0,04
5	22399	0,05	0	0	0	0,95	0	0	0	0	0	0,95	0,05	0	0,048
5	48736	0	0,13	0	0	0,01	0	0	0,86	0	0	0,01	0,86	0,13	0,046
5	10451	0,03	0	0	0	0,9	0	0,04	0,02	0,01	0	0,04	0,91	0,05	0,048
5	14713	0,04	0	0	0	0,79	0	0	0,07	0,1	0,61	0,07	0,32	0	0,03
5	18642	0,03	0	0	0,15	0	0,8	0	0,02	0	0,95	0,05	0	0	0,042
5	15596	0,01	0,08	0	0,04	0,02	0,01	0	0,84	0	0	0,48	0,18	0,34	0,048
5	36445	0,06	0	0	0	0	0,94	0	0	0	1	0	0	0	0,042
5	59175	0,07	0	0,01	0	0,44	0,47	0	0,01	0	0,37	0,47	0,16	0	0,048
5	27421	0,04	0,01	0	0	0,95	0	0	0	0	0,96	0,04	0	0	0,044
5	17614	0	0,04	0	0,07	0,89	0	0	0	0	0,03	0	0,36	0,61	0,048
5	37303	0	0	0	0,04	0,86	0,08	0,02	0	0	0,04	0,02	0	0,94	0,042
5	6652	0,05	0	0	0	0,94	0	0,01	0	0	0,01	0,65	0,05	0,29	0,048
5	6552	0,07	0	0,03	0	0	0	0	0,9	0	0	0	0	1	0,048
5	12656	0	0	0	0,07	0,93	0	0	0	0	0	0,93	0,07	0	0,042
5	23543	0	0	0	0,06	0,94	0	0	0	0	0,06	0,94	0	0	0,032
5	16294	0,05	0	0	0,01	0	0	0	0,94	0	0	0	0	1	0,044
5	20750	0,05	0	0	0	0,94	0,01	0	0	0	0	0,99	0	0,01	0,04
5	15216	0,05	0	0	0,03	0,92	0	0	0	0	0	0,05	0,93	0,02	0,046
5	30765	0,02	0,04	0	0	0,93	0	0	0	0,01	0,81	0,09	0,05	0,05	0,042
5	12803	0,01	0,01	0,04	0,01	0,03	0,01	0,03	0,86	0	0,05	0,01	0,92	0,02	0,048
5	9497	0,05	0	0	0	0,95	0	0	0	0	0	0,65	0,3	0,05	0,038
5	35604	0,05	0	0,01	0	0,69	0,23	0	0,02	0	0,7	0	0,06	0,24	0,042
5	11084	0	0,07	0	0,04	0,89	0	0	0	0	0,16	0	0,82	0,02	0,046
5	25271	0	0	0	0	0,82	0	0,11	0,07	0	0	0	0,89	0,11	0,042
5	8093	0,05	0	0,01	0	0,1	0	0	0,84	0	0,01	0	0,92	0,07	0,048
5	8170	0	0	0,04	0	0,84	0,04	0	0,08	0	0	0,16	0,24	0,6	0,016
5	12263	0	0,07	0	0,04	0,88	0,01	0	0	0	0,88	0,04	0,08	0	0,034
5	12169	0,01	0	0,01	0,05	0,68	0,03	0,03	0,16	0,03	0,18	0,72	0,01	0,09	0,048

5	27241	0	0,01	0,02	0,05	0,02	0,01	0	0,89	0	0,03	0,02	0,01	0,94	0,048
5	17074	0,02	0	0,02	0,05	0,02	0	0	0,88	0,01	0,01	0,41	0,01	0,57	0,044
5	22344	0,04	0,1	0	0	0,28	0	0	0,58	0	0,08	0,86	0	0,06	0,044
5	35606	0	0,15	0	0	0	0,37	0,03	0,45	0	0,18	0,45	0	0,37	0,046
5	27803	0,04	0,04	0	0	0,88	0	0	0,02	0,02	0	0,88	0,12	0	0,038
5	22928	0,06	0,14	0	0	0,8	0	0	0	0	0	0,22	0	0,78	0,048
5	16429	0,04	0,16	0	0	0,04	0,09	0	0,67	0	0	0,06	0,29	0,65	0,044
5	14205	0	0	0	0,12	0,88	0	0	0	0	0,12	0	0,88	0	0,04
5	36424	0	0	0	0,01	0	0,73	0,1	0,16	0	0,16	0,74	0,09	0,01	0,048
5	12070	0,05	0	0	0	0,63	0,3	0,02	0	0	0,01	0,64	0,07	0,28	0,042
5	22640	0,04	0,01	0	0	0	0	0,03	0,92	0	0,2	0,75	0,01	0,04	0,046
5	28813	0,06	0	0	0	0,92	0,02	0	0	0	0,99	0	0	0,01	0,048
5	19992	0	0	0	0,1	0,87	0,01	0	0	0,02	0,1	0,62	0,28	0	0,04
5	11114	0	0	0	0,12	0,74	0	0	0,14	0	0	0,12	0,86	0,02	0,044
5	36027	0,06	0	0	0	0	0	0	0,93	0,01	0	0,87	0,1	0,03	0,038
5	13193	0	0	0,01	0,16	0,62	0,02	0	0,19	0	0,21	0	0,79	0	0,048
5	41345	0	0	0	0,12	0,87	0	0	0	0,01	0,23	0,01	0,64	0,12	0,048
5	21957	0,05	0	0,02	0,02	0,9	0	0	0,01	0	0	0,08	0,02	0,9	0,048
5	22979	0	0,02	0	0	0,07	0,8	0,11	0	0	0,12	0,78	0,08	0,02	0,048
5	75649	0,05	0,27	0	0	0,67	0	0	0,01	0	0,01	0	0,05	0,94	0,046
5	6357	0,05	0,03	0	0	0,74	0	0	0,18	0	0	0,74	0,18	0,08	0,044
5	18279	0	0	0	0,05	0,13	0,01	0,01	0,8	0	0	0,86	0,01	0,13	0,048
5	13530	0	0,94	0	0	0	0,02	0	0,04	0	0,06	0,04	0	0,9	0,048
5	25665	0	0,18	0	0,01	0,73	0,06	0	0,02	0	0,15	0,77	0,02	0,06	0,048
5	16240	0,06	0	0	0,01	0	0,46	0	0,47	0	0,49	0,01	0,42	0,08	0,046
5	11296	0	0	0	0,07	0,93	0	0	0	0	0,03	0,93	0,04	0	0,044
5	20505	0,04	0	0	0	0,67	0	0	0,29	0	0,31	0,69	0	0	0,048
5	71350	0	0	0	0,08	0,04	0,75	0	0,12	0,01	0,08	0	0,04	0,88	0,042
5	44948	0	0,08	0	0,02	0,9	0	0	0	0	0,04	0	0,96	0	0,038
5	9111	0,05	0	0	0,02	0,93	0	0	0	0	0,97	0,01	0,02	0	0,048
5	21453	0,03	0	0	0,05	0	0,89	0	0	0,03	0	0	0,92	0,08	0,044
5	62044	0	0,04	0	0,08	0,88	0	0	0	0	0,52	0,48	0	0	0,04
5	12867	0	0	0,01	0,05	0,93	0	0,01	0	0	0,19	0,01	0,01	0,79	0,042

5	30174	0	0,2	0	0,03	0,76	0	0,01	0	0	0,11	0	0,02	0,87	0,048
5	8148	0	0	0	0,18	0	0,01	0,02	0,74	0,05	0,75	0	0,07	0,18	0,048
5	62005	0,01	0	0	0,03	0,96	0	0	0	0	0,02	0,02	0,01	0,95	0,048
5	36371	0	0,23	0	0	0,76	0	0	0	0,01	0,23	0,26	0	0,51	0,04
5	39724	0	0	0	0,12	0,88	0	0	0	0	0,02	0	0,88	0,1	0,046
5	22539	0,06	0	0	0	0	0,01	0	0,93	0	0	0,01	0,3	0,69	0,048
5	21776	0	0,08	0,01	0	0	0,85	0	0	0,06	0,91	0	0,08	0,01	0,044
	20216,92	0,02219	0,09022	0,00825	0,03128	0,57264	0,08272	0,00743	0,17361	0,011671	0,23375	0,230965	0,254524	0,280765	0,044621
